# Genome-wide specificity of plant genome editing by both CRISPR–Cas9 and TALEN

**DOI:** 10.1038/s41598-022-13034-2

**Published:** 2022-06-04

**Authors:** Nadia Bessoltane, Florence Charlot, Anouchka Guyon-Debast, Delphine Charif, Kostlend Mara, Cécile Collonnier, Pierre-François Perroud, Mark Tepfer, Fabien Nogué

**Affiliations:** 1 Université Paris-Saclay, INRAE, AgroParisTech, Institut Jean-Pierre Bourgin (IJPB), 78000 Versailles, France; 2 CSIRO Health and Biosecurity, Australian Centre for Disease Preparedness, Geelong, VIC 3220 Australia; 3 Community Plant Variety Office (CPVO), 49101 Angers, France

**Keywords:** Plant biotechnology, Plant sciences, Molecular engineering in plants

## Abstract

CRISPR and TALENs are efficient systems for gene editing in many organisms including plants. In many cases the CRISPR–Cas or TALEN modules are expressed in the plant cell only transiently. Theoretically, transient expression of the editing modules should limit unexpected effects compared to stable transformation. However, very few studies have measured the off-target and unpredicted effects of editing strategies on the plant genome, and none of them have compared these two major editing systems. We conducted, in *Physcomitrium patens*, a comprehensive genome-wide investigation of off-target mutations using either a CRISPR–Cas9 or a TALEN strategy. We observed a similar number of differences for the two editing strategies compared to control non-transfected plants, with an average of 8.25 SNVs and 19.5 InDels for the CRISPR-edited plants, and an average of 17.5 SNVs and 32 InDels for the TALEN-edited plants. Interestingly, a comparable number of SNVs and InDels could be detected in the PEG-treated control plants. This shows that except for the on-target modifications, the gene editing tools used in this study did not show a significant off-target activity nor unpredicted effects on the genome, and did not lead to transgene integration. The PEG treatment, a well-established biotechnological method, in itself, was the main source of mutations found in the edited plants.

## Introduction

In recent years, the use of Site Directed Nucleases (SDNs) has paved the way to precise genome editing in plants^1^. SDNs include Meganucleases (MNs), zinc-finger nucleases (ZFNs), transcription-activator like (TAL) effector nucleases (TALENs) and bacterial type I and II CRISPR (Clustered Regulatory Interspaced Short Palindromic Repeats)-Cas systems. If CRISPR–Cas is by far the most popular and versatile tool for genome editing in plants, used for both gene functional analysis and crop breeding^2^, the TALEN strategy has also been used recently to produce new plant varieties^3^. In eukaryote cells, CRISPR–Cas unwanted off-target mutation has been rapidly identified as a potential impediment to its use (for early review see^4^) The optimization of the molecular tools as well as different transfection and selection technical improvements have mitigated the problem, but depending both on the organism and the cell type used, very different outcome can still be observed. For example, CRISPR–Cas9-mediated edition into human peripheral hematopoietic stem and progenitor cells yielded only specific edition events without any increase of non-specific novel non-targeted mutation compared to mock transfection^5^. On the other hand, a similar approach in zebrafish eggs generated multiple off-target indels as well as large structural genome re-organization^6^. In plants, both TALEN usage^7^ and the CRISPR–Cas^8,9^, albeit very efficient to induce on-target mutations, are suspected of causing, in certain conditions, undesired off-target mutations. In order to assess these potential side effects of genome editing with SDNs, a detailed evaluation of the occurrence of off-target mutations is important.

Off-target sites can be predicted in silico by searching for genomic sites showing a similar but not identical sequence compared to the target site. However, SDN specificity is complex and can be influence by the genomic or epigenomic context; consequently, the use of homology-dependent methods should be complemented with homology-independent, unbiased approaches. For this purpose, in vitro or in vivo methods based on high-throughput sequencing (HTS) have been developed, essentially dedicated to the CRISPR–Cas9 strategy. BLESS consists in the direct in situ labeling of breaks in fixed cells, and next-generation sequencing of enriched fragments; it will only detect DSBs that are present at the time of labeling^10^. Digenome-seq^11^, CIRCLE-seq^12^, and SITE-Seq^13^ are based on in vitro genomic DNA digestion and next-generation sequencing. If these techniques are easy to set up, they are nevertheless limited by the possible bias of in vitro digestion. GUIDE-seq is based on the integration of dsODNs into DSBs by NHEJ, amplification and next-generation sequencing^14^. This method is limited by the fact that dsODNs integrate only in ~ 30–50% of DSBs. HTGTS induces “bait” DSB that can capture DNA ends from other DSBs and form translocated sequences that are amplified and sequenced. It relies on concurrence of DSBs^15^. Two techniques can detect off-target events in vivo. VIVO is based on the CIRCLE-seq strategy and as such as the same possible bias as CIRCLE-seq^16^. DISCOVER-seq is a specialized version of ChIP-seq using an MRE11 antibody, it is very sensitive but comparison with other techniques suggests that it does not capture the entirety of the off-target landscape for some gRNAs^17^. Most of the methods of this non exhaustive list show high sensitivity in detecting off-target effects. However, there are limitations to each of them for a fully comprehensive analysis of off-targets in large genomes and tissues composed of multiple cell types, and whole genome sequencing (WGS) appears to be the method of choice for unbiased detection of off-target mutations in a given genome^2,18^.

To date, most studies in plants performed off-target analysis using homology-dependent approaches^19^. To our knowledge no unbiased WGS off-target analysis has been reported on plants edited by ZFNs or meganucleases. Only seven studies described unbiased off-target analyses using WGS, six concerning CRISPR–Cas-edited Arabidopsis, rice, cotton and grapevine^20–25^, and one concerning a TALEN-edited Arabidopsis^26^. The general conclusions of these studies were that off-target activity of CRISPR–Cas or TALENs is generally low, when detectable. However, it must be noted that these analyses concern only very few plants relative to the number of plants where SDN strategies have been used. In addition, the robustness of some of these studies can be limited by the lack of necessary controls and the difficulty to fully assess the levels of preexisting mutations, spontaneous mutations, and mutations caused by tissue culture or by the transfection/transformation method used to deliver the nuclease.

One possibility to overcome these limitations is to perform the WGS analysis on samples from a clonally derived system, thus yielding an objective assessment of the specificity of SDNs at the whole-genome level. For this purpose, we used in this study the model plant *Physcomitrium patens* (*Physcomitrella patens*), for which a reference genome of high quality is available^27,28^, and where SDN gene editing tools are now commonly used^29–35^. We performed a comprehensive investigation of genome-wide off-target mutations from two widely used SDN strategies, CRISPR–Cas9 and TALEN. We used WGS to show that off-target mutations are rare and not distinguishable in frequency when compared to naturally occurring mutations. Our results suggest that off-target mutations caused by Cas9 and TALENs are negligible when compared to spontaneous mutations or mutations caused by tissue culture and transformation in edited plants.

## Results

### Efficiency of the CRISPR–Cas9 and TALEN systems for targeted editing *in P. patens*

**Figure 1:**
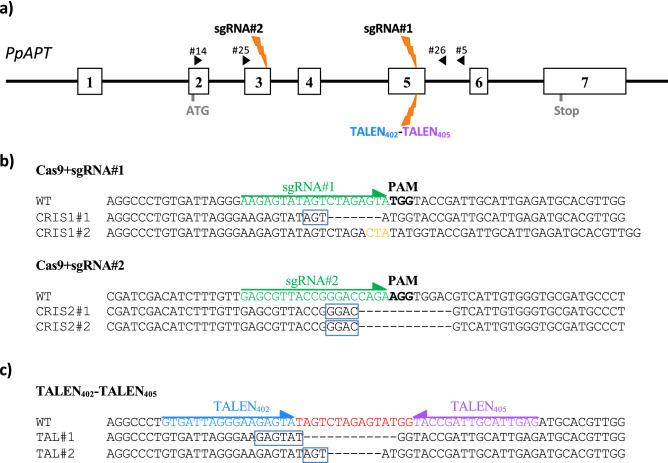
CRISPR–Cas and TALEN targeted editing on the APT gene. (**a**) Structure of the APT gene and sgRNAs and TALEN positions. Boxes in white represent the exons and black lines represent the introns. Black arrows represent the primers used for PCR and sequencing. (**b**) Target sequences for sgRNA#1 and sgRNA#2 on the APT gene and sequence of CRISPR–Cas mutants. PAM (protospacer adjacent motif) sequence is in bold, green letters plus arrow indicate the sequences targeted by sgRNA1 and sgRNA2. (**c**) Target sequences for TALEN402-TALEN405 on the APT gene. Blue and purple letters plus arrows indicate the sequences targeted by TALEN402 and TALEN405 respectively, in red target sequence for the FOK1 nuclease dimer. DNA insertions are shown in orange and deletions with dashes. In the blue frames, the microhomologies potentially involved in Alt-EJ-mediated repair of the induced DSBs.

We previously demonstrated that the CRISPR–Cas9 strategy is very efficient in *P. patens*^30^. We confirmed this here using sgRNA#1 and sgRNA#2, which target sites in exon 5 and exon 3 of the adenine phosphoribosyl transferase (*APT*) gene, respectively (Fig. 1). *P. patens* protoplasts from a wild-type plant derived from a single spore, WT_A_ (Fig. S1), were co-transfected with two plasmids, one expressing the Cas9 gene and another expressing sgRNA#1 or sgRNA#2^31^. Mutations in the *APT* gene leading to a loss of APRT activity confer resistance to the toxic adenine analogue 2-fluoroadenine, 2-FA^36^. Plants where the *APT* gene was edited were thus selected on 2-FA. The mutation rates obtained using sgRNA#1 and sgRNA#2 were, respectively, 2.41% and 3.39% (Table 1). It should be noted that mutation rates are probably underestimated here as only frameshift mutations or deletions of amino acids essential to APT activity lead to resistance to 2FA in our system. We have analyzed the nature of the CRISPR–Cas induced mutations in *P. patens* in a previous study^31^, showing that a large proportion of the on-target mutations due to sgRNA#1 or sgRNA#2 correspond to alternative end-joining (Alt-EJ)-mediated repair of the induced double-strand break (DSB).

**Table 1:** Mutation rates of the *APT* gene using the CRISPR–Cas and TALEN systems.

Editing strategy	Regenerant clones	2-FA^R^ clones	Relative mutation efficiency (%)^a^
CRISPR–Cas (sgRNA#1)	71,100	1718	2.41 ± 0.17 ^b^
CRISPR–Cas (sgRNA#2)	61,600	2077	3.39 ± 0.25
TALEN (TALEn402 + 405)	68,300	56	0.08 ± 0.01

^a^Relative mutation efficiency expresses the frequency of 2-FA resistant clones among the population of regenerants.

^b^Average and standard deviations were determined from three independent experiments.

Sanger sequencing analysis of the *APT* gene in the mutants showed that this is the case of three (CRIS1#1, CRIS2#1, CRIS2#2) of the four plants selected here for further study (Fig. 1b). The mutation in plant CRIS1#2 (Figs. S1, S2), corresponded to an indel potentially resulting from classical non-homologous end-joining (C-NHEJ) repair. Next, in order to evaluate the potential of the TALEN system to induce targeted mutagenesis in *P. patens*, a pair of TALENs was designed that targeted the same region as that targeted by sgRNA#1 on exon 5 of the *APT* gene (Fig. 1). *P. patens* protoplasts from a wild-type plant derived from a single spore, WT_B_ (Fig. S1), were co-transfected with the two plasmids, pAct-TALEN_402_ and pAct-TALEN_405_, and *APT* mutants selected as previously. The mutation rate obtained using TALEN_402_ and TALEN_405_, 0.08% (Table 1), is 30 time lower compared to the mutation efficiency observed using the CRISPR–Cas strategy with sgRNA#1, which targets the same locus. To characterize the mutations induced by the TALEN strategy, we amplified by PCR and sequenced the *APT* gene in 14 independent 2-FA-resistant plants (Fig. S2). As expected, all the mutations were located in the region flanked by TALEN_402_ and TALEN_405_, and as observed for CRISPR–Cas induced mutations, a majority of them (12 out of 14) corresponded to perfect Alt-EJ-mediated repair of the induced DSB, the other two being probably the result of Alt-EJ-mediated repair accompanied by a one base pair substitution (Fig. S2). We confirmed here that CRISPR–Cas is very efficient in *P. patens,* and demonstrated for the first time that the TALEN strategy can be used for gene editing in this model plant. Finally, we confirmed previous results^31,37^ showing the major role of Alt-EJ in the repair of CRISPR–Cas-induced DSBs in *P. patens* and extended it to TALEN-induced DSBs.

### Whole genome sequence analysis of the CRISPR- and TALEN-edited *P. patens* plants

**Figure 2:**
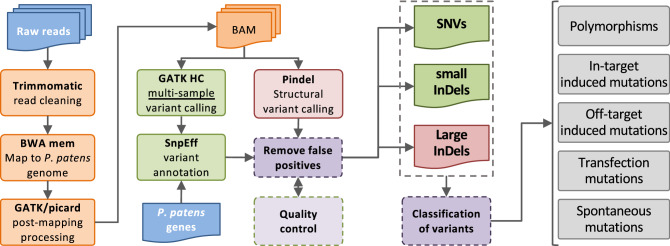
Workflow of whole-genome SNV and InDel analysis. SNV and small InDels (< 50 bp) detection was done using HaplotypeCaller algorithm from GATK tools. Large InDels detection was done using Pindel tool.

**Figure 3:**
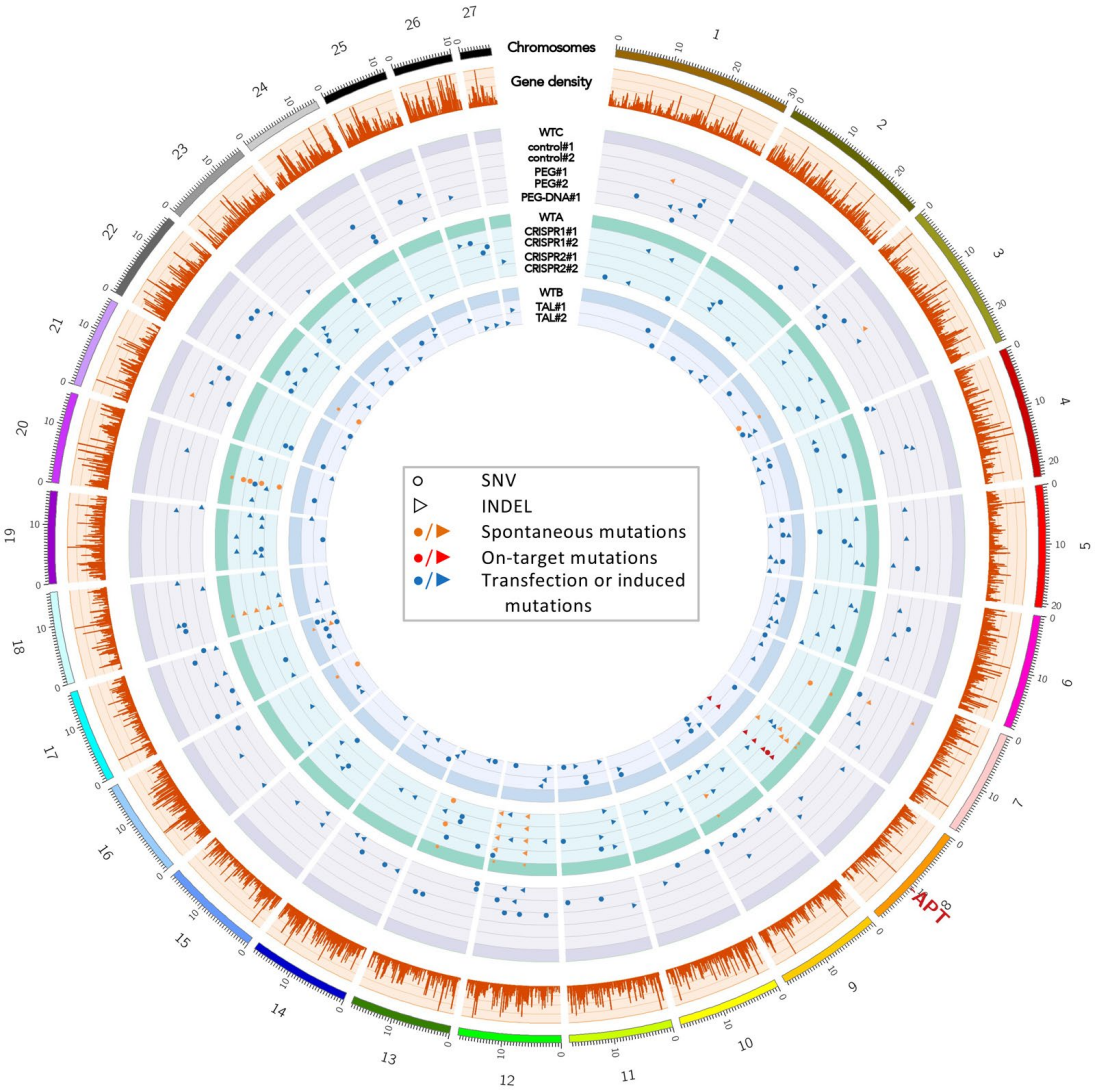
Distribution of spontaneous and transfection mediated mutations. Circos diagram illustrating the distribution along the genome of the different types of mutations detected in the treated plants (SupData 2). The genes affected by these mutations are also illustrated in this Circos. The size of the labels indicates the ratio of variant alleles (AR) in each sample. A large size corresponds to an AR close to 1, and a small size corresponds to an AR at the subclonal level in case of chimeric spontaneous mutations.

To evaluate the potential off-target activity of CRISPR–Cas and TALENs on the entire *P. patens* genome, we carried out WGS on four CRISPR-edited and two TALEN-edited plants, and on the WT_A_ and WT_B_ plants from which the mutants derived, respectively (Fig. S1). In addition, we set up control experiments to estimate the level of mutations that would not be the consequence of the CRISPR–Cas9 or TALEN nuclease activities themselves. For this purpose, we produced protoplasts from WT_C_, a wild-type plant derived from a single spore. From this same batch of protoplasts (Fig. S1) we isolated two independent plants that did not undergo any treatment (Control1#1 and Control1#2), two plants that were PEG treated but with no DNA (PEG#1 and PEG#2), and two plants that were PEG treated in presence of plasmid DNA that contains neither SDN nor sgRNA expression cassettes (PEG-DNA#1 and PEG-DNA#2). All these controls and the WT_C_ plant from which they derived were also sequenced. WT_A_, WT_B_ and WT_C_ plants correspond to three offsprings of the Switzerland (CH) Gransden pedigree^38^ that were grown independently since 1999. Sequencing depth for the different samples was in the range of 14X to 39X (Table S1, Fig. S3). An average of 99.54% of WGS reads were mapped to *P. patens* reference genome (Table S1), however, as expected^27^, a number of reads could not be assigned, entirely or in part, to the *P. patens* genome. From these, reads aligning to bacterial chromosomes or plasmids could be detected in samples TAL#1 and TAL#2. Careful analysis of these reads confirmed that they did not map on the *P. patens* genome. Additionally, we could not detect any contig (assembled reads) containing reads from both bacterial and moss origin. Considering these observations, we propose that these reads correspond to contaminations as already described^27^. This demonstrates that none of the treatments consisting in transfection with plasmid DNA led to the insertion of plasmid-derived DNA sequences into the plant genome. For WGS analysis of the reads that map on the *P. patens* genome for the 15 different plants (Fig. S1) a stringent variant analysis pipeline was developed (Fig. 2). This pipeline use GATK HaplotypeCaller to identify single-nucleotide variants (SNVs) and small insertions and deletions (InDels), and Pindel to identify large InDels (> 50 bp). Analysis of the *APT* locus (Fig. 3, red labels) confirmed the mutations in the *APT* gene of the CRISPR- and TALEN-treated plants, previously established by the Sanger sequencing analysis.

### SNVs and InDels due to spontaneous mutations occur during in vitro culture and propagation in *P. patens*

A total of 5184 SNVs and 4182 InDels were identified that could correspond to polymorphisms between the reference genome and the wild-type plants, between the different wild-type plants, and between the different wild-type plants and their respective treated plants (Fig. S4 and SupData 1). A majority of the variants (4487 SNVs + 2994 InDels) were common to the three different wild-type plants and respective treated plants used in this study (Fig. S5) and clustering based on these variants fits with the sampling of the experiments (Fig. S6). The number of variants is proportional to the size of the respective chromosomes (Table S2, Spearman correlation test *p* value = 8.5 × 10^−6^), however, they are not evenly distributed on the chromosomes, and as expected, are enriched in intergenic regions (Fig. S7). The presence of SNVs specific to the Switzerland (CH) Gransden pedigree in the three different WT lines used in our laboratory (Table S3) confirms that they originate from the Lausanne 1990 pedigree^38^. However, these three “wild types” have evolved independently, and variants (586 SNVs and 967 InDels) specific to each could be detected (Fig. S5). The presence of common and specific variants between the three wild-type plants is in accordance with a recent study^38^ showing that independent mutations can be present in different Gransden laboratory pedigrees, due to spontaneous mutations that occur and can accumulate during in vitro culture. In this respect, accumulation and fixation of spontaneous mutations is an ongoing process that can thus be also observed in the treated plants compared to their respective wild-types (Fig. 3, orange labels). Presence or absence of a subset of such variants found between a given wild type and its respective treated plants were analyzed by Sanger sequencing and confirmed the data obtained by the WGS analysis (Tables S4–S6). Assignment of these SNVs and InDels to spontaneous mutations is based on the fact that they could be found in more than one independently treated plant or, at a low level, in their respective wild-type plants (Table S7).

Of the 9366 variants detected, 318 could not be attributed either to the polymorphism between the reference genome and the wild plants or between the wild plants or to spontaneous mutations. In theory, these variants could be due to the SDNs activity or to the transfection procedure.

### PEG treatment of protoplasts induces SNV and InDel mutations in *P. patens*

In order to estimate the number of potential somaclonal variants due to either the tissue culture process, to pre-existing/inherent variations, or to the transfection process, we compared the number of SNVs and InDels found in the plants of the control experiment (Table 2, Fig. 3, blue labels).

**Table 2:** Whole genome sequence analysis and variants detected in each plant.

Experiment	Plant #	Nb of SNVs/WT	Nb of InDels/WT
Controls	WT_C_	–	–
	Control#1	0	0
	Control#2	0	3
	PEG#1	20	19
	PEG#2	10	19
	PEG-DNA#1	16	24
	PEG-DNA#2	Nd^a^	Nd
CRISPR–Cas	WT_A_	–	–
	CRIS1#1	9	16
	CRIS1#2	11	20
	CRIS2#1	6	29
	CRIS2#2	7	13
TALEN	WT_B_	–	–
	TAL#1	21	31
	TAL#2	11	33

^a^Not determined (nd). DNA sample from the PEG-DNA#2 plant was suspected to be contaminated. The sequence obtained from this sample could not be used for the analysis of variants.

No SNVs could be found between WT_C_ and control plants #1 and #2. Only three InDels were detected in one control plant (plant#2) compared to WT_C_. This means that somaclonal mutations that would be fixed in a clonal plant that originate from a WT protoplast are rare. On the contrary, a significant number of variations (30 SNVs and 38 InDels) were found in PEG-treated compared to WT_C_ (Fig. S8 Kruskal–Wallis test), which were distributed over all the chromosomes (Fig. 3, blue circles and triangles). Some of these mutations are present in predicted genes and 2 of them are in coding regions but does not change the predicted protein sequences (Table S8). These results demonstrate that the PEG treatment in itself is mutagenic and induces SNVs and InDels in the treated protoplast that are fixed in the resulting regenerating plant. Addition of DNA during the PEG treatment did not further increase significantly the number of variants in the treated cells (Table 2).

### CRISPR- and TALEN-edited plants do not show an increase in unpredicted mutations compared to PEG-treated plants

**Figure 4:**
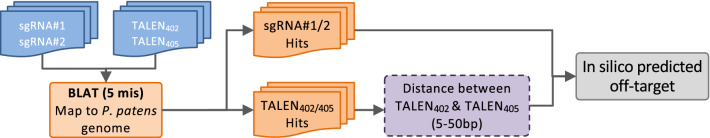
Homology based detection of potential off-targets. BLAT tool was used to predict potential off-target sequences. Results were crossed with detected mutations from WGS variant calling.

Predicted off-targets events, due to their identified genomic position, can be easily assessed when using a SDN strategy. In order to detect potential off-target activity of the CRISPR–Cas and TALEN strategies, we first investigated the status of the predicted off-targets in the edited plants. For this purpose, we used a global alignment approach with the BLAT tool to align target sequences for sgRNA#1, sgRNA#2 and TALEN_402_ and TALEN_405_ to the *P. patens* genome, allowing 5 mismatches for each sequence (Fig. 4). A total of 9 and 4 possible off-targets were found for sgRNA#1 and sgRNA#2, respectively (Table S9). Sequence analysis of the potential off-targets in plants CRIS1#1, CRIS1#2, CRIS2#1 and CRIS2#2 showed that none of them were modified in the edited plants. This confirms the high specificity of the CAS9 nuclease as previously observed in *P. patens*^31^. Concerning the TALEN strategy, we did not find any locus in the *P. patens* genome showing homology to the different possible pairs of TALENs allowing a distance between pairs of 5–50 bp, needed for the assembly of the two Fok1 domains and nuclease activity.

In order to estimate the number of mutations that could be the result of non-homology-mediated off-target activities of the CRISPR or TALEN strategies, we compared the number of SNVs and InDels found in the CRISPR- or TALEN-edited plants to those found in the plants of the control experiments (Table 2, Fig. 3). A low number of SNVs and InDels were found for both strategies of editing, with an average of 8.25 SNVs and 19.5 InDels for the CRISPR-edited plants, and an average of 17.5 SNVs and 32 InDels for the TALEN-edited plants. If the differences, in term of numbers of variants between the edited plants and the controls are significant, these numbers are not statistically different from the numbers found in the PEG-treated plants (Fig. S8 Kruskal–Wallis test). This shows that the SDNs used in this study do not display a significant unpredicted off-target activity and that except for the on-target modifications, the PEG treatment in itself is the main source of additional SNVs and InDels found in the transfected plants compared to the wild-type.

## Discussion

Despite the obvious potential of SDNs in plant breeding, there is an ongoing debate about their precise targeting and to what extent the occurrence of off-target effects matter^7–9^. Unbiased studies on the potential off-target activity of these nucleases in different plant species is essential to approach this debate in a rational way. *P. patens* has a relatively small genome (1 N~0.48 GB) compared to other plants such as wheat (2 N~5.5 GB) or maize (2 N~2.3 GB) but in the range of other crops such as rice (2 N~400 Mb) or grape (2 N~475 Mb)^39^. In addition, the haploid phase of the life cycle of *P. patens* is dominant, and regenerating a plant from a single cell is very easy. Therefore, the problem of heterozygosity and of population heterogeneity is easily solved in regenerated *P. patens* plants when using WGS. These characteristics make *P. patens* a suitable model organism to evaluate the specificity of genome editing tools.

In this study, we used *P. patens* to estimate possible off-target effects from the two most used SDN strategies, CRISPR–Cas9 and TALEN. For this purpose, we performed for the first time in this species a large-scale WGS analysis of three wild-type, six controls and six CRISPR- or TALEN-edited plants, to detect potential off-target mutations that would result from unwanted nuclease activity of these two SDNs.

WGS analysis did not detected insertion of vector-derived DNA sequences into the plant genome. The vast majority of the identified variants were found in the three different wild types compared to the reference genome. In addition, multiple SNVs and InDels distinguished the different wild types. These variations are due to spontaneous mutations that occur and can accumulate during in vitro culture, as shown previously^38^. Some of the SNVs found are representative of the genealogy of the lines used in the laboratory, the first “strain” used in our laboratory originating from the Switzerland (CH) Gransden pedigree, which had already diverged from the line that was used to establish the reference genome. Overall, the wild-type-to-reference genome variants demonstrate clearly that the three independent wild-type lines used in our laboratory have already diverged from the original line.

A small number of spontaneous mutations could be identified in some of the treated plants compared to their respective wild type. This number is very low and is similar to the observed mutation rates (changes per year and site, 7 × 10^−7^ to 4 × 10^−6^) for *P. patens*^38^. Interestingly, the production of protoplasts does not seem to be mutagenic in *P. patens* (Control1 vs. WT_C_). This is in contrast with what was observed in potato plants regenerated from protoplasts, which are subject to genomic instability^40^. One possible explanation of this result can be that regeneration of a plant from a *P. patens* protoplast does not require passage by a dedifferentiated callus state, unlike regeneration processes in most seed plants^41^. If protoplast preparation is not mutagenic in *P. patens*, the PEG treatment, on the other hand, does induce SNP and InDel mutations (PEG vs Control1 and WT_C_). This is in agreement with the already described mutagenic effect of a PEG treatment in yeast, which could in fact be due to the concomitant osmotic shock^42^.

Analysis of variations found in the CRISPR- and TALEN-treated plants confirmed the on-target modifications, and did not show significant mutations at predicted or unpredicted off-target sites for both strategies. These results are consistent with what was observed for CRISPR–Cas9 in Arabidopsis, rice, cotton and grapevine^20–25^ and for TALENs in Arabidopsis^26^.

Comparison for off-target activity of CRISPR–Cas9 and TALENs has been done in human stem cells^43^, and the authors found that off-target mutations attributable to the nucleases were very rare. Here, we compared the CRISPR–Cas9 and TALEN systems for the first time in plants, and show that in our experimental conditions they are highly specific in *P. patens*; and if changes due to off-target activity would be present they would be fewer than variations caused by spontaneous mutations and by PEG treatment. Due to the efforts needed for this type of analysis we restricted our study to two independent editing targets. Analysis of off-target activity of gene editing tools should be extended to a greater number of loci in *P. patens* and to more plants, including crops, in order to evaluate whether our observations can be broadened.

## Methods

### Plant material and transformation

All local, national or international guidelines and legislation were adhered to in the production of this study. Spores of *P. patens* wild type accession (Gransden strain), were obtained from the International Moss Stock Center (http://www.moss-stock-center.org/). Florence Charlot carried out *P. patens* cultivation and experimental material collection. *P. patens* wild-type Gransden strain was vegetatively propagated on PpNH_4_ medium (PpNO_3_ medium supplemented with 2.7 mM NH_4_-tartrate) in growth chambers set at 60% humidity with 16 h of light (quantum irradiance of 70 μmol m^−2^ s^−1^) at 23 °C and 8 h of dark at 23 °C. Moss protoplast isolation and polyethylene glycol (PEG)-mediated transfection were performed as previously described^44^. Protoplasts were transfected with a total of 20 µg of circular DNA. Transfected protoplasts were resuspended in an alginate solution consisting of 2% (w/v) Na-alginate (Sigma, St. Louis, USA) and 0.4 M mannitol, spread on cellophane disks and grown on PpNH_4_ medium supplemented with 0.33 M Mannitol and 6 mM CaCl_2_ for 1 week. Plants on cellophane disks were then transfered on PpNH_4_ supplemented with 10 μM 2-fluoroadenine (2-FA) (Fluorochem, Hadfield, United Kingdom) to select clones that were mutated at the *APT* locus^31^. The mutation rates (expressed in percentages) were estimated by dividing the number of 2-FA-resistant plants by the number of regenerating plants observed just before the transfer on 2-FA medium. For the control experiments (Fig. S1), protoplasts were treated or not with PEG in presence or not of 20 µg of mock DNA, plasmid pBNRF^45^ that carry a 35S::neoR cassette, cloned in a pMCS5 backbone (MoBiTec). Regenerating protoplasts were treated as described above but in the absence of the selective agent 2-FA.

### Molecular cloning

The pAct-Cas9 plasmid used in this study contains a Cas9 expression cassette driven by the rice actin 1 promoter and a codon-optimized version of Cas9. Plasmid pAct-Cas9 and the plasmids encoding the sgRNA expression cassettes sgRNA#1 or sgRNA#2 (driven by a snRNA U6 promoter from *P. patens*) targeting the *P. patens APT* gene (Phytozome gene#Pp3c8_16590) (Fig. 1), were reported in our previous study (Collonnier et al.^31^). The plasmids pAct-TALEN_402_ and pAct-TALEN_405_ contain a TALEN expression cassette driven by the rice actin 1 promoter and TALEN_402_ or TALEN_405_ respectively (Figs. S9, S10). The plasmids pAct-TALEN_402_ and pAct-TALEN_405_ were designed by the TalGene platform (TEFOR, https://tefor.net/). Target sequences for sgRNA#1, sgRNA#2, TALEN_402_ and TALEN_405_ on the *APT* gene are shown in Fig. 1.

### PCR and sequence analysis of the edited plants

For PCR analysis, genomic DNA was extracted from 50 mg of fresh tissue as previously described^30^. The quality of the DNA samples was controlled using primers targeting the *RAD51-1* gene from *P. patens*: PpRAD51-1#6 and PpRAD51-1#7^45^. Molecular analysis was based on Sanger sequencing (Genoscreen, Lille, France) of PCR fragments using primers PpAPT#25/PpAPT#26 surrounding the targeted locus (Fig. 1). PCR primers used in this study are listed in Tables S10 and S11.

### Genomic DNA isolation and library construction for whole genome sequencing

Genomic DNA was extracted from 1.5 g of 7-day fresh protonema culture on PpNH4 medium. DNA samples were extracted using the NucleoSpin™ PlantII Kit (Macherey–Nagel) as described by the manufacturer. For all 13 samples (WT_A_, WT_B_, WT_C_, Control#1, Control#2, PEG#1, PEG#2, PEG-DNA#1, PEG-DNA#2, CRIS1#1, CRIS1#2, CRIS2#1, CRIS2#2, TAL#1, TAL#2), 5 µg DNA were used to construct a sequencing library according to Illumina Kapa Hyper Prep PCR Free Kit (Fragmentation to 350 Bp and sizing with ratio 0.7×). Final libraries were sequenced using an Illumina Hiseq 4000 or NovaSeq (Paired-end 150 bp) with an average 15–20× sequencing depth (INRAE EPGV platform, CEA-IG/CNG Evry, France) (Table S1).

### Whole genome sequencing analysis

The WGS reads were cleaned up using the Trimmomatic tool (0.32)^46^, and then mapped to *P. patens* reference sequence v3.3^28^ with BWA mem (0.7.15) software^47^. Afterward alignment files were processed according to the genome analysis toolkit (GATK) Best Practices recommendations^48^. SNVs (single nucleotide variations) and small InDels (insertion, deletion variations, size < 50 bp) were called using GATK (3.5) HaplotypeCaller algorithm^49^ across all samples simultaneously, and annotated using SnpEff tools (3.6c)^50^. To reduce false positives, calls quality and coverage filtering was applied (QUAL > 20, total depth > 3, reads supporting variant allele > 1). Only positions covered at all samples were considered (4487 Positions/473 Mb genome size). Since *P. patens* is haploid, variants with allele fraction (VAF) lower than 0.8 are considered as false positive calls or chimera variants. Pindel software (v0.2)^51^ was used to detect large InDels (size > 50 bp) which were filtered in the same way as SNVs and small InDels.

Selected variants were classified either as polymorphisms or as potential mutations (Fig. S4). The variants classified as polymorphisms to the reference genome are the variants common to all the plants. Polymorphisms between the three wild types are the variants common to all the plants from a same experiment. The potential mutations detected in controls (control#1 and control#2) are considered as spontaneous mutations. Those detected in transfected plants (PEG#1/2 and PEG-DNA#1/2) are considered either as spontaneous mutations or as transfection mutations. Apart from on-target mutations, the mutations detected in CRISPR–Cas and TALEN mutants can be spontaneous mutations, transfection mutations, or off-target mutations. IGV^52^ was used to check manually all potential mutations.

### Genome-wide prediction of potential off-target cleavage sites

The sgRNA#1, sgRNA#2, TALEN402 and TALEN405 sequences were aligned to the *P. patens* genome using BLAT (the BLAST-like alignment tool)^53^ allowing 5 mismatches for each sequence fragments. For TALEN sequence hits, we considered all possible pairs between TALEN402 and TALEN405 and imposed a distance of 5–50 bp for each pair. Using these parameters, we did not find any loci in the *P. patens* genome showing homology to the TALENs. Resulting hits for sgRNA#1 and sgRNA#2 were crossed with potential mutations detected by WGS analysis.

### Ethics approval and consent to participate

No ethical approval was required for this study.

## Supplementary Information

Supplementary Information 1.

Supplementary Information 2.

Supplementary Information 3.

Supplementary Information 4.

## Data Availability

Raw sequence data generated in this study have been deposited in the ArrayExpress database at EMBL-EBI (https://www.ebi.ac.uk/arrayexpress) under the accession number E-MTAB-11497. A short description of the twelve genomic samples is presented in the Table S1.
