# Supplementary material for: Genome-wide specificity of plant genome editing by both CRISPR–Cas9 and TALEN
Source: Sci Rep. 2022 Jun 4;12:9330. doi: 10.1038/s41598-022-13034-2 (PMC9167288; doi:10.1038/s41598-022-13034-2)
Supplement: Supplementary file 1 — Supplementary Information 1. [file 41598_2022_13034_MOESM1_ESM.pdf]

CHROM	POS	REF	ALT	ID	CHANGE	CHANGE_TY	QUAL	WTB_AD	TAL#1_AD	TAL#2_AD	WTA_AD	CRIS1#1_AD	CRIS1#2_AD	CRIS2#1_AD	CRIS2#2_AD	WTC_AD	Control#1_A	Control#2_A	PEG#1_AD	PEG#2_AD	PEG-DNA#1_	WTB_DP	TAL#1_DP	TAL#2_DP	WTA_DP	CRIS1#1_DP	CRIS1#2_DP	CRIS2#1_DP	CRIS2#2_DP	WTC_DP	Control#1_D	Control#2_D
Chr01	33341	G	GA	Chr01_33341	G_GA	INDEL	14073.59	23	16	13	26	22	20	35	14	8	36	12	13	12	15	23	16	13	26	22	20	35	14	8	36	12
Chr01	33341	G	GA	Chr01_33341	G_GA	INDEL	14073.59	23	16	13	26	22	20	35	14	8	36	12	13	12	15	23	16	13	26	22	20	35	14	8	36	12
Chr01	33341	G	GA	Chr01_33341	G_GA	INDEL	14073.59	23	16	13	26	22	20	35	14	8	36	12	13	12	15	23	16	13	26	22	20	35	14	8	36	12
Chr01	33341	G	GA	Chr01_33341	G_GA	INDEL	14073.59	23	16	13	26	22	20	35	14	8	36	12	13	12	15	23	16	13	26	22	20	35	14	8	36	12
Chr01	33341	G	GA	Chr01_33341	G_GA	INDEL	14073.59	23	16	13	26	22	20	35	14	8	36	12	13	12	15	23	16	13	26	22	20	35	14	8	36	12
Chr01	33341	G	GA	Chr01_33341	G_GA	INDEL	14073.59	23	16	13	26	22	20	35	14	8	36	12	13	12	15	23	16	13	26	22	20	35	14	8	36	12
Chr01	33341	G	GA	Chr01_33341	G_GA	INDEL	14073.59	23	16	13	26	22	20	35	14	8	36	12	13	12	15	23	16	13	26	22	20	35	14	8	36	12
Chr01	33341	G	GA	Chr01_33341	G_GA	INDEL	14073.59	23	16	13	26	22	20	35	14	8	36	12	13	12	15	23	16	13	26	22	20	35	14	8	36	12
Chr01	33341	G	GA	Chr01_33341	G_GA	INDEL	14073.59	23	16	13	26	22	20	35	14	8	36	12	13	12	15	23	16	13	26	22	20	35	14	8	36	12
Chr01	33341	G	GA	Chr01_33341	G_GA	INDEL	14073.59	23	16	13	26	22	20	35	14	8	36	12	13	12	15	23	16	13	26	22	20	35	14	8	36	12
Chr01	45106	T	TC	Chr01_45106	T_TC	INDEL	16731.59	23	21	18	29	13	32	39	20	18	23	15	13	11	10	23	21	18	29	13	32	39	20	18	23	15
Chr01	45106	T	TC	Chr01_45106	T_TC	INDEL	16731.59	23	21	18	29	13	32	39	20	18	23	15	13	11	10	23	21	18	29	13	32	39	20	18	23	15
Chr01	62068	C	CTATA	Chr01_62068	C_CTATA	INDEL	2838.93	0	0	1	2	0	1	1	0	16	17	8	10	6	11	11	11	11	19	13	22	22	8	16	17	8
Chr01	62068	C	CTATA	Chr01_62068	C_CTATA	INDEL	2838.93	0	0	1	2	0	1	1	0	16	17	8	10	6	11	11	11	11	19	13	22	22	8	16	17	8
Chr01	62068	C	CTATA	Chr01_62068	C_CTATA	INDEL	2838.93	0	0	1	2	0	1	1	0	16	17	8	10	6	11	11	11	11	19	13	22	22	8	16	17	8
Chr01	102149	AAT	A	Chr01_10214	AAT_A	INDEL	3841.24	0	0	1	25	14	26	25	15	1	0	1	0	1	0	24	7	18	25	16	27	25	16	12	11	7
Chr01	102149	AAT	A	Chr01_10214	AAT_A	INDEL	3841.24	0	0	1	25	14	26	25	15	1	0	1	0	1	0	24	7	18	25	16	27	25	16	12	11	7
Chr01	102149	AAT	A	Chr01_10214	AAT_A	INDEL	3841.24	0	0	1	25	14	26	25	15	1	0	1	0	1	0	24	7	18	25	16	27	25	16	12	11	7
Chr01	102149	AAT	A	Chr01_10214	AAT_A	INDEL	3841.24	0	0	1	25	14	26	25	15	1	0	1	0	1	0	24	7	18	25	16	27	25	16	12	11	7
Chr01	102149	AAT	A	Chr01_10214	AAT_A	INDEL	3841.24	0	0	1	25	14	26	25	15	1	0	1	0	1	0	24	7	18	25	16	27	25	16	12	11	7
Chr01	103366	C	T	Chr01_10336	C_T	SNP	4048.98	0	0	0	0	0	0	0	0	18	22	22	15	10	12	20	13	10	24	18	33	26	23	18	22	22
Chr01	103366	C	T	Chr01_10336	C_T	SNP	4048.98	0	0	0	0	0	0	0	0	18	22	22	15	10	12	20	13	10	24	18	33	26	23	18	22	22
Chr01	103366	C	T	Chr01_10336	C_T	SNP	4048.98	0	0	0	0	0	0	0	0	18	22	22	15	10	12	20	13	10	24	18	33	26	23	18	22	22
Chr01	103366	C	T	Chr01_10336	C_T	SNP	4048.98	0	0	0	0	0	0	0	0	18	22	22	15	10	12	20	13	10	24	18	33	26	23	18	22	22
Chr01	103366	C	T	Chr01_10336	C_T	SNP	4048.98	0	0	0	0	0	0	0	0	18	22	22	15	10	12	20	13	10	24	18	33	26	23	18	22	22
Chr01	155068	G	GTA	Chr01_15506	G_GTA	INDEL	8005.59	26	7	4	21	10	17	28	14	9	9	6	13	8	10	26	7	4	21	10	17	29	14	9	10	6
Chr01	155068	G	GTA	Chr01_15506	G_GTA	INDEL	8005.59	26	7	4	21	10	17	28	14	9	9	6	13	8	10	26	7	4	21	10	17	29	14	9	10	6
Chr01	155068	G	GTA	Chr01_15506	G_GTA	INDEL	8005.59	26	7	4	21	10	17	28	14	9	9	6	13	8	10	26	7	4	21	10	17	29	14	9	10	6
Chr01	155068	G	GTA	Chr01_15506	G_GTA	INDEL	8005.59	26	7	4	21	10	17	28	14	9	9	6	13	8	10	26	7	4	21	10	17	29	14	9	10	6
Chr01	155068	G	GTA	Chr01_15506	G_GTA	INDEL	8005.59	26	7	4	21	10	17	28	14	9	9	6	13	8	10	26	7	4	21	10	17	29	14	9	10	6
Chr01	155068	G	GTA	Chr01_15506	G_GTA	INDEL	8005.59	26	7	4	21	10	17	28	14	9	9	6	13	8	10	26	7	4	21	10	17	29	14	9	10	6
Chr01	155068	G	GTA	Chr01_15506	G_GTA	INDEL	8005.59	26	7	4	21	10	17	28	14	9	9	6	13	8	10	26	7	4	21	10	17	29	14	9	10	6
Chr01	155068	G	GTA	Chr01_15506	G_GTA	INDEL	8005.59	26	7	4	21	10	17	28	14	9	9	6	13	8	10	26	7	4	21	10	17	29	14	9	10	6
Chr01	155068	G	GTA	Chr01_15506	G_GTA	INDEL	8005.59	26	7	4	21	10	17	28	14	9	9	6	13	8	10	26	7	4	21	10	17	29	14	9	10	6
Chr01	155068	G	GTA	Chr01_15506	G_GTA	INDEL	8005.59	26	7	4	21	10	17	28	14	9	9	6	13	8	10	26	7	4	21	10	17	29	14	9	10	6
Chr01	155068	G	GTA	Chr01_15506	G_GTA	INDEL	8005.59	26	7	4	21	10	17	28	14	9	9	6	13	8	10	26	7	4	21	10	17	29	14	9	10	6
Chr01	155068	G	GTA	Chr01_15506	G_GTA	INDEL	8005.59	26	7	4	21	10	17	28	14	9	9	6	13	8	10	26	7	4	21	10	17	29	14	9	10	6
Chr01	155068	G	GTA	Chr01_15506	G_GTA	INDEL	8005.59	26	7	4	21	10	17	28	14	9	9	6	13	8	10	26	7	4	21	10	17	29	14	9	10	6
Chr01	155068	G	GTA	Chr01_15506	G_GTA	INDEL	8005.59	26	7	4	21	10	17	28	14	9	9	6	13	8	10	26	7	4	21	10	17	29	14	9	10	6
Chr01	155068	G	GTA	Chr01_15506	G_GTA	INDEL	8005.59	26	7	4	21	10	17	28	14	9	9	6	13	8	10	26	7	4	21	10	17	29	14	9	10	6
Chr01	155068	G	GTA	Chr01_15506	G_GTA	INDEL	8005.59	26	7	4	21	10	17	28	14	9	9	6	13	8	10	26	7	4	21	10	17	29	14	9	10	6
Chr01	155068	G	GTA	Chr01_15506	G_GTA	INDEL	8005.59	26	7	4	21	10	17	28	14	9	9	6	13	8	10	26	7	4	21	10	17	29	14	9	10	6
Chr01	155068	G	GTA	Chr01_15506	G_GTA	INDEL	8005.59	26	7	4	21	10	17	28	14	9	9	6	13	8	10	26	7	4	21	10	17	29	14	9	10	6
Chr01	178402	T	A	Chr01_17840	T_A	SNP	15742.63	26	14	16	19	13	30	25	15	25	28	9	11	11	11	26	14	16	19	13	30	25	15	25	28	9
Chr01	178402	T	A	Chr01_17840	T_A	SNP	15742.63	26	14	16	19	13	30	25	15	25	28	9	11	11	11	26	14	16	19	13	30	25	15	25	28	9
Chr01	484863	G	T	Chr01_48486	G_T	SNP	20107.63	33	20	14	26	18	35	19	19	24	30	8	19	9	10	33	20	14	26	19	35	19	19	24	30	8
Chr01	496781	C	A	Chr01_49678	C_A	SNP	20922.63	35	14	21	25	18	25	25	12	21	27	9	23	15	10	35	14	21	25	18	25	25	12	21	27	9
Chr01	496781	C	A	Chr01_49678	C_A	SNP	20922.63	35	14	21	25	18	25	25	12	21	27	9	23	15	10	35	14	21	25	18	25	25	12	21	27	9
Chr01	496781	C	A	Chr01_49678	C_A	SNP	20922.63	35	14	21	25	18	25	25	12	21	27	9	23	15	10	35	14	21	25	18	25	25	12	21	27	9
Chr01	496783	C	A	Chr01_49678	C_A	SNP	20922.63	36	14	18	26	18	25	24	11	21	27	9	23	15	10	36	14	18	26	18	25	24	11	21	27	9
Chr01	496783	C	A	Chr01_49678	C_A	SNP	20922.63	36	14	18	26	18	25	24	11	21	27	9	23	15	10	36	14	18	26	18	25	24	11	21	27	9
Chr01	496783	C	A	Chr01_49678	C_A	SNP	20922.63	36	14	18	26	18	25	24	11	21	27	9	23	15	10	36	14	18	26	18	25	24	11	21	27	9
Chr01	497936	T	TA	Chr01_49793	T_TA	INDEL	14758.59	24	9	10	27	22	24	26	27	18	26	12	6	16	13	24	9	10	27	22	24	26	27	18	26	12
Chr01	497936	T	TA	Chr01_49793	T_TA	INDEL	14758.59	24	9	10	27	22	24	26	27	18	26	12	6	16	13	24	9	10	27	22	24	26	27	18	26	12
Chr01	497936	T	TA	Chr01_49793	T_TA	INDEL	14758.59	24	9	10	27	22	24	26	27	18	26	12	6	16	13	24	9	10	27	22	24	26	27	18	26	12
Chr01	533803	T	A	Chr01_53380	T_A	SNP	15078.63	24	12	10	19	19	22	26	16	13	15	10	20	5	4	24	12	10	19	19	22	26	16	13	15	10
Chr01	533803	T	A	Chr01_53380	T_A	SNP	15078.63	24	12	10	19	19	22	26	16	13	15	10	20	5	4	24	12	10	19	19	22	26	16	13	15	10
Chr01	533867	A	AT	Chr01_53386	A_AT	INDEL	8685.59	12	8	11	11	9	13	24	14	17	13	10	12	10	5	12	8	11	11	9	13	24	14	17	13	10
Chr01	533867	A	AT	Chr01_53386	A_AT	INDEL	8685.59	12	8	11	11	9	13	24	14	17	13	10	12	10	5	12	8	11	11	9	13	24	14	17	13	10
[illegible]	[illegible]	[illegible]	[illegible]	[illegible]	[illegible]	[illegible]	[illegible]	[illegible]	[illegible]	[illegible]	[illegible]	[illegible]	[illegible]	[illegible]	[illegible]	[illegible]	[illegible]	[illegible]	[illegible]	[illegible]	[illegible]	[illegible]	[illegible]	[illegible]	[illegible]	[illegible]	[illegible]	[illegible]	[illegible]	[illegible]

Chr01	3135857	T	TTA	Chr01_31358T_TTA	INDEL	1248.35	22	13	10	1	1	1	0	0	2	[illegible]
Chr01	3135857	T	TTA	Chr01_31358T_TTA	INDEL	1248.35	22	13	10	1	1	1	0	0	2	[illegible]
Chr01	3200062	CT	C	Chr01_32000CT_C	INDEL	12029.59	24	10	11	18	12	20	28	22	12	[illegible]
Chr01	3263451	G	GT	Chr01_32634G_GT	INDEL	15279.59	28	14	19	16	18	26	26	19	19	[illegible]
Chr01	3652399	C	A	Chr01_36523C_A	SNP	13302.29	35	18	12	0	1	0	0	0	21	[illegible]
Chr01	3664055	A	AAT	Chr01_36640A_AAT	INDEL	2706.93	1	1	0	0	0	0	0	0	14	[illegible]
Chr01	3759997	G	T	Chr01_37599G_T	SNP	22988.63	34	22	18	21	28	28	39	29	17	[illegible]
Chr01	3759997	G	T	Chr01_37599G_T	SNP	22988.63	34	22	18	21	28	28	39	29	17	[illegible]
Chr01	3759997	G	T	Chr01_37599G_T	SNP	22988.63	34	22	18	21	28	28	39	29	17	[illegible]
Chr01	3759998	A	T	Chr01_37599A_T	SNP	23078.63	34	22	18	22	27	28	39	28	17	[illegible]
Chr01	3759998	A	T	Chr01_37599A_T	SNP	23078.63	34	22	18	22	27	28	39	28	17	[illegible]
Chr01	3759998	A	T	Chr01_37599A_T	SNP	23078.63	34	22	18	22	27	28	39	28	17	[illegible]
Chr01	3760005	A	T	Chr01_37600A_T	SNP	22353.63	31	17	19	21	28	27	38	27	17	[illegible]
Chr01	3760005	A	T	Chr01_37600A_T	SNP	22353.63	31	17	19	21	28	27	38	27	17	[illegible]
Chr01	3760005	A	T	Chr01_37600A_T	SNP	22353.63	31	17	19	21	28	27	38	27	17	[illegible]
Chr01	3760006	C	T	Chr01_37600C_T	SNP	22128.63	31	18	19	21	28	26	38	27	17	[illegible]
Chr01	3760006	C	T	Chr01_37600C_T	SNP	22128.63	31	18	19	21	28	26	38	27	17	[illegible]
Chr01	3760006	C	T	Chr01_37600C_T	SNP	22128.63	31	18	19	21	28	26	38	27	17	[illegible]
Chr01	3875645	C	A	Chr01_38756C_A	SNP	14091.63	20	8	14	20	18	19	23	18	18	[illegible]
Chr01	3970009	A	AT	Chr01_39700A_AT	INDEL	15218.59	34	14	19	26	20	29	20	15	24	[illegible]
Chr01	3970199	C	A	Chr01_39701C_A	SNP	21076.63	32	15	16	32	18	20	30	27	21	[illegible]
Chr01	3970296	C	A	Chr01_39702C_A	SNP	21604.63	37	19	16	30	18	27	36	14	32	[illegible]
Chr01	3977692	C	CAT	Chr01_39776C_CAT	INDEL	1463.93	2	0	0	0	0	0	0	0	10	[illegible]
Chr01	3995585	C	A	Chr01_39955C_A	SNP	19897.63	29	15	16	26	19	27	18	24	32	[illegible]
[illegible]																

Chr01	8397278	A	AT	Chr01_83972A_AT	INDEL	11305.59	17	7	6	17	18	14	17	19	19	14	13	15	8	6	18	7	6	17	18	14	17	19	19	14	13	15	8	6	0.944
Chr01	8404903	A	AC	Chr01_84049A_AC	INDEL	14385.59	29	13	7	21	20	13	27	17	16	16	10	12	7	9	29	13	7	21	20	13	27	17	16	16	10	12	7	9	
Chr01	8404905	C	A	Chr01_84049C_A	SNP	14394.63	28	13	7	21	17	13	27	17	15	15	10	12	6	9	28	13	7	21	17	13	27	17	15	15	10	12	6	9	
Chr01	8512689	A	AT	Chr01_85126A_AT	INDEL	13030.59	22	14	12	26	25	31	21	16	17	27	19	21	7	8	22	14	12	26	25	31	21	16	17	27	19	21	7	8	
Chr01	8514184	A	AAT	Chr01_85141A_AAT	INDEL	1625.93	0	0	1	0	1	0	1	0	5	14	10	13	6	5	13	8	11	13	11	16	16	11	5	14	11	13	6	5	
Chr01	8527368	G	GA	Chr01_85273G_GA	INDEL	15470.59	21	11	13	22	9	18	25	23	21	23	25	12	8	13	21	11	13	22	9	18	25	23	21	23	25	12	8	13	
Chr01	8813922	C	T	Chr01_88139C_T	SNP	17861.63	26	16	12	18	20	24	22	16	15	25	18	20	12	15	26	16	12	18	20	24	22	16	15	25	18	20	12	15	
Chr01	8918866	G	T	Chr01_89188G_T	SNP	19366.63	23	15	18	24	24	32	20	30	20	31	11	13	10	8	23	15	18	24	24	32	20	30	20	31	11	13	10	8	
Chr01	9002901	C	CA	Chr01_90029C_CA	INDEL	23556.59	50	25	23	39	35	47	50	50	28	38	23	25	20	14	50	25	23	39	35	47	50	50	28	38	23	25	20	14	
Chr01	9002901	C	CA	Chr01_90029C_CA	INDEL	23556.59	50	25	23	39	35	47	50	50	28	38	23	25	20	14	50	25	23	39	35	47	50	50	28	38	23	25	20	14	
Chr01	9002901	C	CA	Chr01_90029C_CA	INDEL	23556.59	50	25	23	39	35	47	50	50	28	38	23	25	20	14	50	25	23	39	35	47	50	50	28	38	23	25	20	14	
Chr01	9002901	C	CA	Chr01_90029C_CA	INDEL	23556.59	50	25	23	39	35	47	50	50	28	38	23	25	20	14	50	25	23	39	35	47	50	50	28	38	23	25	20	14	
Chr01	9134330	C	T	Chr01_91343C_T	SNP	16021.63	30	13	11	17	19	19	20	35	16	17	18	20	14	14	30	13	11	17	19	19	20	35	16	17	18	20	14	14	
Chr01	9296642	G	C	Chr01_92966G_C	SNP	20543.63	27	13	14	28	21	22	30	28	27	23	16	13	10	9	27	13	14	28	21	22	30	28	27	23	16	13	10	9	
Chr01	9296643	C	A	Chr01_92966C_A	SNP	20705.63	27	13	14	27	21	23	29	28	27	24	16	13	10	9	27	13	14	27	21	23	29	28	27	24	16	13	10	9	
Chr01	9296660	CG	C	Chr01_92966CG_C	INDEL	20738.59	27	13	12	27	19	25	28	27	28	22	20	13	15	8	27	13	12	27	19	25	28	27	28	22	20	13	15	8	
Chr01	9322620	G	GTA	Chr01_93226G_GTA	INDEL	5667.24	23	11	9	0	3	1	2	0	15	16	6	9	8	14	23	11	9	13	14	29	20	11	15	17	6	9	8	14	
Chr01	9322620	G	GTA	Chr01_93226G_GTA	INDEL	5667.24	23	11	9	0	3	1	2	0	15	16	6	9	8	14	23	11	9	13	14	29	20	11	15	17	6	9	8	14	
Chr01	9322620	G	GTA	Chr01_93226G_GTA	INDEL	5667.24	23	11	9	0	3	1	2	0	15	16	6	9	8	14	23	11	9	13	14	29	20	11	15	17	6	9	8	14	
Chr01	9322620	G	GTA	Chr01_93226G_GTA	INDEL	5667.24	23	11	9	0	3	1	2	0	15	16	6	9	8	14	23	11	9	13	14	29	20	11	15	17	6	9	8	14	
Chr01	9322620	G	GTA	Chr01_93226G_GTA	INDEL	5667.24	23	11	9	0	3	1	2	0	15	16	6	9	8	14	23	11	9	13	14	29	20	11	15	17	6	9	8	14	
Chr01	9492611	G	GTA	Chr01_94926G_GTA	INDEL	873.35	13	9	11	1	0	0	1	0	0	0	0	0	0	1	13	9	11	20	19	17	34	15	8	15	10	9	7	5	
Chr01	9492611	G	GTA	Chr01_94926G_GTA	INDEL	873.35	13	9	11	1	0	0	1	0	0	0	0	0	0	1	13	9	11	20	19	17	34	15	8	15	10	9	7	5	
Chr01	9492611	G	GTA	Chr01_94926G_GTA	INDEL	873.35	13	9	11	1	0	0	1	0	0	0	0	0	0	1	13	9	11	20	19	17	34	15	8	15	10	9	7	5	
Chr01	9492611	G	GTA	Chr01_94926G_GTA	INDEL	873.35	13	9	11	1	0	0	1	0	0	0	0	0	0	1	13	9	11	20	19	17	34	15	8	15	10	9	7	5	
Chr01	9492611	G	GTA	Chr01_94926G_GTA	INDEL	873.35	13	9	11	1	0	0	1	0	0	0	0	0	0	1	13	9	11	20	19	17	34	15	8	15	10	9	7	5	
Chr01	9492611	G	GTA	Chr01_94926G_GTA	INDEL	873.35	13	9	11	1	0	0	1	0	0	0	0	0	0	1	13	9	11	20	19	17	34	15	8	15	10	9	7	5	
Chr01	9542360	C	A	Chr01_95423C_A	SNP	2611.39	24	19	21	0	0	0	0	0	0	0	0	0	0	0	24	19	21	19	13	29	34	24	23	18	20	13	11	11	
Chr01	9706874	C	CTA	Chr01_97068C_CTA	INDEL	4756.24	14	9	15	1	0	1	2	0	11	15	9	5	5	10	14	9	15	12	10	24	24	10	11	15	9	5	5	12	
Chr01	9706874	C	CTA	Chr01_97068C_CTA	INDEL	4756.24	14	9	15	1	0	1	2	0	11	15	9	5	5	10	14	9	15	12	10	24	24	10	11	15	9	5	5	12	
Chr01	9706874	C	CTA	Chr01_97068C_CTA	INDEL	4756.24	14	9	15	1	0	1	2	0	11	15	9	5	5	10	14	9	15	12	10	24	24	10	11	15	9	5	5	12	
Chr01	9706874	C	CTA	Chr01_97068C_CTA	INDEL	4756.24	14	9	15	1	0	1	2	0	11	15	9	5	5	10	14	9	15	12	10	24	24	10	11	15	9	5	5	12	
Chr01	9706874	C	CTA	Chr01_97068C_CTA	INDEL	4756.24	14	9	15	1	0	1	2	0	11	15	9	5	5	10	14	9	15	12	10	24	24	10	11	15	9	5	5	12	
Chr01	9706874	C	CTA	Chr01_97068C_CTA	INDEL	4756.24	14	9	15	1	0	1	2	0	11	15	9	5	5	10	14	9	15	12	10	24	24	10	11	15	9	5	5	12	
Chr01	9706874	C	CTA	Chr01_97068C_CTA	INDEL	4756.24	14	9	15	1	0	1	2	0	11	15	9	5	5	10	14	9	15	12	10	24	24	10	11	15	9	5	5	12	
Chr01	9707855	A	T	Chr01_97078A_T	SNP	17208.63	22	10	15	21	14	23	29	27	13	18	14	20	9	8	22	10	15	21	14	23	29	27	13	18	14	20	9	8	
Chr01	9707855	A	T	Chr01_97078A_T	SNP	17208.63	22	10	15	21	14	23	29	27	13	18	14	20	9	8	22	10	15	21	14	23	29	27	13	18	14	20	9	8	
Chr01	9707855	A	T	Chr01_97078A_T	SNP	17208.63	22	10	15	21	14	23	29	27	13	18	14	20	9	8	22	10	15	21	14	23	29	27	13	18	14	20	9	8	
Chr01	9707855	A	T	Chr01_97078A_T	SNP	17208.63	22	10	15	21	14	23	29	27	13	18	14	20	9	8	22	10	15	21	14	23	29	27	13	18	14	20	9	8	
Chr01	9707855	A	T	Chr01_97078A_T	SNP	17208.63	22	10	15	21	14	23	29	27	13	18	14	20	9	8	22	10	15	21	14	23	29	27	13	18	14	20	9	8	
Chr01	9707855	A	T	Chr01_97078A_T	SNP	17208.63	22	10	15	21	14	23	29	27	13	18	14	20	9	8	22	10	15	21	14	23	29	27	13	18	14	20	9	8	
Chr01	9707855	A	T	Chr01_97078A_T	SNP	17208.63	22	10	15	21	14	23	29	27	13	18	14	20	9	8	22	10	15	21	14	23	29	27	13	18	14	20	9	8	
Chr01	9864780	C	G	Chr01_98647C_G	SNP	16793.63	19	14	11	26	27	21	24	20	18	16	12	15	10	11	19	14	11	26	27	21	24	20	18	16	12	15	10	11	
Chr01	9864801	C	G	Chr01_98648C_G	SNP	17094.63	18	13	11	23	26	21	23	24	17	17	12	18	12	10	18	13	11	23	26	21	23	24	17	17	12	18	12	10	
Chr01	9864833	C	G	Chr01_98648C_G	SNP	17392.63	19	16	15	21	23	21	25	24	18	13	17	17	14	10	19	16	15	21	23	21	25	24	18	13	17	17	14	10	
Chr01	9864851	C	G	Chr01_98648C_G	SNP	16499.63	16	12	16	19	18	22	22	24	16	11	16	18	12	9	16	12	16	19	18	22	22	24	16	11	16	18	12	9	
Chr01	9915643	GTA	G	Chr01_99156GTA_G	INDEL	1743.35	26	15	14	1	0	0	0	0	0	1	0	0	0	0	26	15	14	20	16	22	28	26	24	30	11	10	13	9	
Chr01	10021716	T	A	Chr01_10021T_A	SNP	9081.63	12	4	7	6	9	13	7	11	19	11	9	8	4	4	12	4	7	6	9	13	7	11	19	11	9	8	4	4	
Chr01	10096516	G	A	Chr01_10096G_A	SNP	12648.29	32	14	18	0	0	0	0	0	14	19	22	22	8	7	32	14	18	26	14	25	40	35	14	19	22	22	8	7	
Chr01	10096516	G	A	Chr01_10096G_A	SNP	12648.29	32	14	18	0	0	0	0	0	14	19	22	22	8	7	32	14	18	26	14	25	40	35	14	19	22	22	8	7	
Chr01	10096516	G	A	Chr01_10096G_A	SNP	12648.29	32	14	18	0	0	0	0	0	14	19	22	22	8	7	32	14	18	26	14	25	40	35	14	19	22	22	8	7	
Chr01	10096516	G	A	Chr01_10096G_A	SNP	12648.29	32	14	18	0	0	0	0	0	14	19	22	22	8	7	32	14	18	26	14	25	40	35	14	19	22	22	8	7	
Chr01	10096516	G	A	Chr01_10096G_A	SNP	12648.29	32	14	18	0	0	0	0	0	14	19	22	22	8	7	32	14	18	26	14	25	40	35	14	19	22	22	8	7	
Chr01	10096516	G	A	Chr01_10096G_A	SNP	12648.29	32	14	18	0	0	0	0	0	14	19	22	22	8	7	32	14	18	26	14	25	40	35	14	19	22	22	8	7	
Chr01	10096516	G	A	Chr01_10096G_A	SNP	12648.29	32	14	18	0	0	0	0	0	14	19	22	22	8	7	32	14	18	26	14	25	40	35	14	19	22	22	8	7	
Chr01	10096516	G	A	Chr01_10096G_A	SNP	12648.29	32	14	18	0	0	0	0	0	14	19	22	22	8	7	32	14	18	26	14	25	40	35	14	19	22	22	8	7	
Chr01	10096516	G	A	Chr01_10096G_A	SNP	12648.29	32	14	18	0	0	0	0	0	14	19	22	22	8	7	32	14	18	26	14	25	40	35	14	19	22	22	8	7	
Chr01	10096516	G	A	Chr01_10096G_A	SNP	12648.29	32	14	18	0	0	0	0	0	14	19	22	22	8	7	32	14	18	26	14	25	40	35	14	19	22	22	8	7	
Chr01	10198867	G	A	Chr01_10198G_A	SNP	18607.63	28	16	11	27	18	34	37	30	35	31	14	9	4	7	29	16	11	27	18	35	40	30	35	31	14	9	4	7	0.966
Chr01	10198867	G	A	Chr01_10198G_A	SNP	18607.63	28	16	11	27	18	34	37	30	35	31	14	9	4	7	29	16	11	27	18	35	40	30	35	31	14	9	4	7	0.966
Chr01	10296485	G	T	Chr01_10296G_T	SNP	17099.63	16	19	15	26	16	29	17	15	20	20	17	12	8	10	16	19	15	26	16	29	17	15	20	20	17	12	8	10	
Chr01	10296486	T	G	Chr01_10296T_G	SNP	17099.63	16	19	15	26	17	29	17	15	20	20	17	12	8	10	16	19	15	26	17	29	17	15	20	20	17	12	8	10	
Chr01	10296516	GA	G	Chr01_10296GA_G	INDEL	14279.59	20	19	13	25	12	31	23	14	18	20	17	15	9	7	20	19	13	25	12	31	23	14	18	20	17	15	9	7	

Chr01	12509569	C	CT	Chr01_12509	C_CT	INDEL	17538.59	39	10	19	29	19	23	33	21	27	31
Chr01	12509569	C	CT	Chr01_12509	C_CT	INDEL	17538.59	39	10	19	29	19	23	33	21	27	31
Chr01	12570548	T	TTA	Chr01_12570	T_TTA	INDEL	8602.24	19	15	9	0	0	1	0	0	28	24
Chr01	12570548	T	TTA	Chr01_12570	T_TTA	INDEL	8602.24	19	15	9	0	0	1	0	0	28	24
Chr01	12570548	T	TTA	Chr01_12570	T_TTA	INDEL	8602.24	19	15	9	0	0	1	0	0	28	24
Chr01	12570548	T	TTA	Chr01_12570	T_TTA	INDEL	8602.24	19	15	9	0	0	1	0	0	28	24
Chr01	12570548	T	TTA	Chr01_12570	T_TTA	INDEL	8602.24	19	15	9	0	0	1	0	0	28	24
Chr01	12696122	A	AT	Chr01_12696	A_AT	INDEL	12985.59	10	14	17	21	15	27	24	20	10	15
Chr01	12696122	A	AT	Chr01_12696	A_AT	INDEL	12985.59	10	14	17	21	15	27	24	20	10	15
Chr01	12696122	A	AT	Chr01_12696	A_AT	INDEL	12985.59	10	14	17	21	15	27	24	20	10	15
Chr01	12696122	A	AT	Chr01_12696	A_AT	INDEL	12985.59	10	14	17	21	15	27	24	20	10	15
Chr01	12745606	TC	T	Chr01_12745	TC_T	INDEL	15177.59	18	15	18	21	14	21	37	15	16	22
Chr01	12887672	T	G	Chr01_12887	T_G	SNP	19793.63	36	8	16	26	15	28	34	19	16	27
Chr01	12974275	A	G	Chr01_12974	A_G	SNP	20382.63	28	8	16	22	26	27	27	20	19	31
Chr01	12974276	A	C	Chr01_12974	A_C	SNP	20382.63	29	8	16	22	25	27	26	20	19	31
Chr01	12974332	A	T	Chr01_12974	A_T	SNP	18768.63	35	10	15	26	20	32	24	18	17	29
Chr01	12981358	G	T	Chr01_12981	G_T	SNP	23527.63	41	25	11	40	14	36	34	27	19	18
Chr01	12981369	G	T	Chr01_12981	G_T	SNP	23647.63	41	24	12	40	15	36	36	25	19	21
Chr01	12983916	T	TTA	Chr01_12983	T_TTA	INDEL	2430.24	2	0	0	20	7	21	14	14	0	0
Chr01	12989419	G	GTA	Chr01_12989	G_GTA	INDEL	176.37	1	0	8	0	0	0	0	1	2	0
Chr01	12993504	A	AAT	Chr01_12993	A_AAT	INDEL	1742.35	26	15	12	0	0	0	1	0	0	0
Chr01	13092252	T	TC	Chr01_13092	T_TC	INDEL	14721.59	22	12	4	21	19	27	20	16	21	20
Chr01	13109064	C	CA	Chr01_13109	C_CA	INDEL	11670.59	18	9	12	23	15	23	20	10	16	24
Chr01	13109064	C	CA	Chr01_13109	C_CA	INDEL	11670.59	18	9	12	23	15	23	20	10	16	24
Chr01	13109100	G	T	Chr01_13109	G_T	SNP	10588.63	9	5	7	15	13	17	11	8	13	21
Chr01	13109100	G	T	Chr01_13109	G_T	SNP	10588.63	9	5	7	15	13	17	11	8	13	21
Chr01	13109115	G	T	Chr01_13109	G_T	SNP	10599.63	13	6	7	16	10	15	10	9	13	21
Chr01	13109115	G	T	Chr01_13109	G_T	SNP	10599.63	13	6	7	16	10	15	10	9	13	21
Chr01	13109142	G	T	Chr01_13109	G_T	SNP	11287.63	18	6	8	13	13	14	11	14	13	17
Chr01	13109142	G	T	Chr01_13109	G_T	SNP	11287.63	18	6	8	13	13	14	11	14	13	17
Chr01	13109157	G	T	Chr01_13109	G_T	SNP	11645.63	19	5	10	15	14	11	14	16	10	19
Chr01	13109157	G	T	Chr01_13109	G_T	SNP	11645.63	19	5	10	15	14	11	14	16	10	19
Chr01	13109191	G	T	Chr01_13109	G_T	SNP	10696.63	21	8	11	14	15	10	14	16	11	16
Chr01	13109191	G	T	Chr01_13109	G_T	SNP	10696.63	21	8	11	14	15	10	14	16	11	16
Chr01	13110434	AT	A	Chr01_13110	AT_A	INDEL	19526.59	27	10	13	20	26	20	19	19	33	21
Chr01	13110434	AT	A	Chr01_13110	AT_A	INDEL	19526.59	27	10	13	20	26	20	19	19	33	21
Chr01	13110442	T	TA	Chr01_13110	T_TA	INDEL	20186.59	29	10	14	19	26	21	20	19	33	23
Chr01	13110442	T	TA	Chr01_13110	T_TA	INDEL	20186.59	29	10	14	19	26	21	20	19	33	23
Chr01	13124589	G	GT	Chr01_13124	G_GT	INDEL	15774.59	33	7	13	29	19	19	29	16	22	23
Chr01	13124589	G	GT	Chr01_13124	G_GT	INDEL	15774.59	33	7	13	29	19	19	29	16	22	23
Chr01	13124589	G	GT	Chr01_13124	G_GT	INDEL	15774.59	33	7	13	29	19	19	29	16	22	23
Chr01	13124589	G	GT	Chr01_13124	G_GT	INDEL	15774.59	33	7	13	29	19	19	29	16	22	23
Chr01	13124589	G	GT	Chr01_13124	G_GT	INDEL	15774.59	33	7	13	29	19	19	29	16	22	23
Chr01	13124589	G	GT	Chr01_13124	G_GT	INDEL	15774.59	33	7	13	29	19	19	29	16	22	23
Chr01	13124589	G	GT	Chr01_13124	G_GT	INDEL	15774.59	33	7	13	29	19	19	29	16	22	23
Chr01	13124589	G	GT	Chr01_13124	G_GT	INDEL	15774.59	33	7	13	29	19	19	29	16	22	23
Chr01	13124589	G	GT	Chr01_13124	G_GT	INDEL	15774.59	33	7	13	29	19	19	29	16	22	23
Chr01	13366443	T	C	Chr01_13366	T_C	SNP	13458.63	22	9	11	19	19	17	24	15	16	14
Chr01	13366443	T	C	Chr01_13366	T_C	SNP	13458.63	22	9	11	19	19	17	24	15	16	14
Chr01	13366443	T	C	Chr01_13366	T_C	SNP	13458.63	22	9	11	19	19	17	24	15	16	14
Chr01	13366443	T	C	Chr01_13366	T_C	SNP	13458.63	22	9	11	19	19	17	24	15	16	14
Chr01	13366443	T	C	Chr01_13366	T_C	SNP	13458.63	22	9	11	19	19	17	24	15	16	14
Chr01	13366443	T	C	Chr01_13366	T_C	SNP	13458.63	22	9	11	19	19	17	24	15	16	14
Chr01	13366443	T	C	Chr01_13366	T_C	SNP	13458.63	22	9	11	19	19	17	24	15	16	14
Chr01	13384866	C	A	Chr01_13384	C_A	SNP	8014.63	16	4	5	4	8	12	15	17	14	14
Chr01	13385122	T	A	Chr01_13385	T_A	SNP	30494.63	39	32	22	29	24	45	52	31	30	57
Chr01	13385123	T	A	Chr01_13385	T_A	SNP	30809.63	40	32	22	29	24	45	52	32	30	58
Chr01	13385139	G	C	Chr01_13385	G_C	SNP	31936.63	47	34	26	30	27	48	56	32	30	55
Chr01	13385141	G	A	Chr01_13385	G_A	SNP	31756.63	46	35	27	30	27	47	56	32	32	55
Chr01	13427511	A	AT	Chr01_13427	A_AT	INDEL	16365.59	26	13	16	32	23	18	36	33	21	28
Chr01	13447273	C	T	Chr01_13447	C_T	SNP	3398.98	0	0	0	0	0	0	0	0	15	22
Chr01	13447273	C	T	Chr01_13447	C_T	SNP	3398.98	0	0	0	0	0	0	0	0	15	22
Chr01	13447273	C	T	Chr01_13447	C_T	SNP	3398.98	0	0	0	0	0	0	0	0	15	22
Chr01	13520894	GAT	G	Chr01_13520	GAT_G	INDEL	12777.59	34	10	9	27	21	23	29	21	16	14
Chr01	13520894	GAT	G	Chr01_13520	GAT_G	INDEL	12777.59	34	10	9	27	21	23	29	21	16	14
Chr01	13520894	GAT	G	Chr01_13520	GAT_G	INDEL	12777.59	34	10	9	27	21	23	29	21	16	14
Chr01	13520894	GAT	G	Chr01_13520	GAT_G	INDEL	12777.59	34	10	9	27	21	23	29	21	16	14
Chr01	13520894	GAT	G	Chr01_13520	GAT_G	INDEL	12777.59	34	10	9	27	21	23	29	21	16	14
Chr01	13520894	GAT	G	Chr01_13520	GAT_G	INDEL	12777.59	34	10	9	27	21	23	29	21	16	14
Chr01	13520894	GAT	G	Chr01_13520	GAT_G	INDEL	12777.59	34	10	9	27	21	23	29	21	16	14
Chr01	13931675	C	T	Chr01_13931	C_T	SNP	16318.63	29	8	15	16	24	21	22	20	11	16
Chr01	13931675	C	T	Chr01_13931	C_T	SNP	16318.63	29	8	15	16	24	21	22	20	11	16
Chr01	13931675	C	T	Chr01_13931	C_T	SNP	16318.63	29	8	15	16	24	21	22	20	11	16
Chr01	13931675	C	T	Chr01_13931	C_T	SNP	16318.63	29	8	15	16	24	21	22	20	11	16
Chr01	13931675	C	T	Chr01_13931	C_T	SNP	16318.63	29	8	15	16	24	21	22	20	11	16
Chr01	13931678	C	G	Chr01_13931	C_G	SNP	16836.63	29	8	14	16	24	22	21	20	11	16
Chr01	13931678	C	G	Chr01_13931	C_G	SNP	16836.63	29	8	14	16	24	22	21	20	11	16
Chr01	13931678	C	G	Chr01_13931	C_G	SNP	16836.63	29	8	14	16	24	22	21	20	11	16
Chr01	13931678	C	G	Chr01_13931	C_G	SNP	16836.63	29	8	14	16	24	22	21	20	11	16
Chr01	13931678	C	G	Chr01_13931	C_G	SNP	16836.63	29	8	14	16	24	22	21	20	11	16
Chr01	13931680	A	AGAT	Chr01_13931	A_AGAT	INDEL	17471.59	29	9	14	16	24	22	22	22	11	16
Chr01	13931680	A	AGAT	Chr01_13931	A_AGAT	INDEL	17471.59	29	9	14	16	24	22	22	22	11	16
Chr01	13931680	A	AGAT	Chr01_13931	A_AGAT	INDEL	17471.59	29	9	14	16	24	22	22	22	11	16
Chr01	13931680	A	AGAT	Chr01_13931	A_AGAT	INDEL	17471.59	29	9	14	16	24	22	22	22	11	16
Chr01	13931680	A	AGAT	Chr01_13931	A_AGAT	INDEL	17471.59	29	9	14	16	24	22	22	22	11	16
Chr01	13931682	A	AC	Chr01_13931	A_AC	INDEL	17651.59	29	9	17	16	23	23	22	23	13	15
Chr01	13931682	A	AC	Chr01_13931	A_AC	INDEL	17651.59	29	9	17	16	23	23	22	23	13	15
Chr01	13931682	A	AC	Chr01_13931	A_AC	INDEL	17651.59	29	9	17	16	23	23	22	23	13	15
Chr01	13931682	A	AC	Chr01_13931	A_AC	INDEL	17651.59	29	9	17	16	23	23	22	23	13	15
Chr01	13931682	A	AC	Chr01_13931	A_AC	INDEL	17651.59	29	9	17	16	23	23	22	23	13	15
Chr01	13931685	CCA	C	Chr01_13931	CCA_C	INDEL	17561.59	28	9	16	14	23	23	23	25	15	15
Chr01	13931685	CCA	C	Chr01_13931	CCA_C	INDEL	17561.59	28	9	16	14	23	23	23	25	15	15
Chr01	13931685	CCA	C	Chr01_13931	CCA_C	INDEL	17561.59	28	9	16	14	23	23	23	25	15	15
Chr01	13931685	CCA	C	Chr01_13931	CCA_C	INDEL	17561.59	28	9	16	14	23	23	23	25	15	15
Chr01	13931685	CCA	C	Chr01_13931	CCA_C	INDEL	17561.59	28	9	16	14	23	23	23	25	15	15
Chr01	13931691	G	GC	Chr01_13931	G_GC	INDEL	18011.59	28	11	15	14	22	22	24	26	15	15
Chr01	13931691	G	GC	Chr01_13931	G_GC	INDEL	18011.59	28	11	15	14	22	22	24	26	15	15
Chr01	13931691	G	GC	Chr01_13931	G_GC	INDEL	18011.59	28	11	15	14	22	22	24	26	15	15
Chr01	13931691	G	GC	Chr01_13931	G_GC	INDEL	18011.59	28	11	15	14	22	22	24	26	15	15
Chr01	13931691	G	GC	Chr01_13931	G_GC	INDEL	18011.59	28	11	15	14	22	22	24	26	15	15
Chr01	13931695	C	A	Chr01_13931	C_A	SNP	18110.63	28	12	13	13	20	22	24	26	17	16
Chr01	13931695	C	A	Chr01_13931	C_A	SNP	18110.63	28	12	13	13	20	22	24	26	17	16
Chr01	13931695	C	A	Chr01_13931	C_A	SNP	18110.63	28	12	13	13	20	22	24	26	17	16
Chr01	13931695	C	A	Chr01_13931	C_A	SNP	18110.63	28	12	13	13	20	22	24	26	17	16
Chr01	13931695	C	A	Chr01_13931	C_A	SNP	18110.63	28	12	13	13	20	22	24	26	17	16
Chr01	13931696	C	A	Chr01_13931	C_A	SNP	18020.63	28	12	12	12	20	22	25	27	16	16
Chr01	13931696	C	A	Chr01_13931	C_A	SNP	18020.63	28	12	12	12	20	22	25	27	16	16
Chr01	13931696	C	A	Chr01_13931	C_A	SNP	18020.63	28	12	12	12	20	22	25	27	16	16
Chr01	13931696	C	A	Chr01_13931	C_A	SNP	18020.63	28	12	12	12	20	22	25	27	16	16
Chr01	13931696	C	A	Chr01_13931	C_A	SNP	18020.63	28	12	12	12	20	22	25	27	16	16
Chr01	13931700	TA	T	Chr01_13931	TA_T	INDEL	17966.59	26	11	11	13	22	21	27	28	16	16
Chr01	13931700	TA	T	Chr01_13931	TA_T	INDEL	17966.59	26	11	11	13	22	21	27	28	16	16
Chr01	13931700	TA	T	Chr01_13931	TA_T	INDEL	17966.59	26	11	11	13	22	21	27	28	16	16
Chr01	13931700	TA	T	Chr01_13931	TA_T	INDEL	17966.59	26	11	11	13	22	21	27	28	16	16
Chr01	13931700	TA	T	Chr01_13931	TA_T	INDEL	17966.59	26	11	11	13	22	21	27	28	16	16
Chr01	13931702	A	T	Chr01_13931	A_T	SNP	17660.63	27	11	11	13	22	21	29	27	17	16
Chr01	13931702	A	T	Chr01_13931	A_T	SNP	17660.63	27	11	11	13	22	21	29	27	17	16
Chr01	13931702	A	T	Chr01_13931	A_T	SNP	17660.63	27	11	11	13	22	21	29	27	17	16
Chr01	13931702	A	T	Chr01_13931	A_T	SNP	17660.63	27	11	11	13	22	21	29	27	17	16
Chr01	13931702	A	T	Chr01_13931	A_T	SNP	17660.63	27	11	11	13	22	21	29	27	17	16
Chr01	13931708	G	GATCAA	Chr01_13931	G_GATCAA	INDEL	19181.59	25	10	10	14	22	22	33	28	18	19
Chr01	13931708	G	GATCAA	Chr01_13931	G_GATCAA	INDEL	19181.59	25	10	10	14	22	22	33	28	18	19
Chr01	13931708	G	GATCAA	Chr01_13931	G_GATCAA	INDEL	19181.59	25	10	10	14	22	22	33	28	18	19
Chr01	13931708	G	GATCAA	Chr01_13931	G_GATCAA	INDEL	19181.59	25	10	10	14	22	22	33	28	18	19
Chr01	13931708	G	GATCAA	Chr01_13931	G_GATCAA	INDEL	19181.59	25	10	10	14	22	22	33	28	18	19
Chr01	13931709	G	GGAA	Chr01_13931	G_GGAA	INDEL	19226.59	22	11	9	15	20	22	33	28	18	19
Chr01	13931709	G	GGAA	Chr01_13931	G_GGAA	INDEL	19226.59	22	11	9	15	20	22	33	28	18	19
Chr01	13931709	G	GGAA	Chr01_13931	G_GGAA	INDEL	19226.59	22	11	9	15	20	22	33	28	18	19
Chr01	13931709	G	GGAA	Chr01_13931	G_GGAA	INDEL	19226.59	22	11	9	15	20	22	33	28	18	19
Chr01	13931709	G	GGAA	Chr01_13931	G_GGAA	INDEL	19226.59	22	11	9	15	20	22	33	28	18	19
Chr01	13931714	AGCCTCAATT	A	Chr01_13931	AGCCTCAATT	INDEL	18641.59	24	13	9	16	20	20	32	30	15	19
Chr01	13931714	AGCCTCAATT	A	Chr01_13931	AGCCTCAATT	INDEL	18641.59	24	13	9	16	20	20	32	30	15	19
Chr01	13931714	AGCCTCAATT	A	Chr01_13931	AGCCTCAATT	INDEL	18641.59	24	13	9	16	20	20	32	30	15	19
Chr01	13931714	AGCCTCAATT	A	Chr01_13931	AGCCTCAATT	INDEL	18641.59	24	13	9	16	20	20	32	30	15	19
Chr01	13931714	AGCCTCAATT	A	Chr01_13931	AGCCTCAATT	INDEL	18641.59	24	13	9	16	20	20	32	30	15	19
Chr01	13931733	A	AC	Chr01_13931	A_AC	INDEL	19046.59	24	12	9	18	19	21	31	31	17	19
Chr01	13931733	A	AC	Chr01_13931	A_AC	INDEL	19046.59	24	12	9	18	19	21	31	31	17	19
Chr01	13931733	A	AC	Chr01_13931	A_AC	INDEL	19046.59	24	12	9	18	19	21	31	31	17	19
Chr01	13931733	A	AC	Chr01_13931	A_AC	INDEL	19046.59	24	12	9	18	19	21	31	31	17	19
Chr01	13931733	A	AC	Chr01_13931	A_AC	INDEL	19046.59	24	12	9	18	19	21	31	31	17	19
Chr01	13931735	AAT	A	Chr01_13931	AAT_A	INDEL	19181.59	24	12	9	15	19	22	31	32	18	19
Chr01	13931735	AAT	A	Chr01_13931	AAT_A	INDEL	19181.59	24	12	9	15	19	22	31	32	18	19
Chr01	13931735	AAT	A	Chr01_13931	AAT_A	INDEL	19181.59	24	12	9	15	19	22	31	32	18	19
Chr01	13931735	AAT	A	Chr01_13931	AAT_A	INDEL	19181.59	24	12	9	15	19	22	31	32	18	19
Chr01	13931735	AAT	A	Chr01_13931	AAT_A	INDEL	19181.59	24	12	9	15	19	22	31	32	18	19
Chr01	13931739	G	T	Chr01_13931	G_T	SNP	18920.63	25	11	7	15	19	22	30	32	18	19
Chr01	13931739	G	T	Chr01_13931	G_T	SNP	18920.63	25	11	7	15	19	22	30	32	18	19
Chr01	13931739	G	T	Chr01_13931	G_T	SNP	18920.63	25	11	7	15	19	22	30	32	18	19
Chr01	13931739	G	T	Chr01_13931	G_T	SNP	18920.63	25	11	7	15	19	22	30	32	18	19
Chr01	13931739	G	T	Chr01_13931	G_T	SNP	18920.63	25	11	7	15	19	22	30	32	18	19
Chr01	13931742	A	AAGG	Chr01_13931	A_AAGG	INDEL	19091.59	25	11	6	15	17	22	30	33	19	19
Chr01	13931742	A	AAGG	Chr01_13931	A_AAGG	INDEL	19091.59	25	11	6	15	17	22	30	33	19	19
Chr01	13931742	A	AAGG	Chr01_13931	A_AAGG	INDEL	19091.59	25	11	6	15	17	22	30	33	19	19
Chr01	13931742	A	AAGG	Chr01_13931	A_AAGG	INDEL	19091.59	25	11	6	15	17	22	30	33	19	19
Chr01	13931742	A	AAGG	Chr01_13931	A_AAGG	INDEL	19091.59	25	11	6	15	17	22	30	33	19	19
Chr01	13931746	C	CCAAGTCTG	Chr01_13931	C_CCAAGTCT	INDEL	19406.59	24	11	6	15	17	23	33	30	19	21
Chr01	13931746	C	CCAAGTCTG	Chr01_13931	C_CCAAGTCT	INDEL	19406.59	24	11	6	15	17	23	33	30	19	21
Chr01	13931746	C	CCAAGTCTG	Chr01_13931	C_CCAAGTCT	INDEL	19406.59	24	11	6	15	17	23	33	30	19	21
Chr01	13931746	C	CCAAGTCTG	Chr01_13931	C_CCAAGTCT	INDEL	19406.59	24	11	6	15	17	23	33	30	19	21
Chr01	13931746	C	CCAAGTCTG	Chr01_13931	C_CCAAGTCT	INDEL	19406.59	24	11	6	15	17	23	33	30	19	21
Chr01	13931751	TAA	T	Chr01_13931	TAA_T	INDEL	18256.59	26	8	7	15	17	23	33	30	19	25
Chr01	13931751	TAA	T	Chr01_13931	TAA_T	INDEL	18256.59	26	8	7	15	17	23	33	30	19	25
Chr01	13931751	TAA	T	Chr01_13931	TAA_T	INDEL	18256.59	26	8	7	15	17	23	33	30	19	25
Chr01	13931751	TAA	T	Chr01_13931	TAA_T	INDEL	18256.59	26	8	7	15	17	23	33	30	19	25
Chr01	13931751	TAA	T	Chr01_13931	TAA_T	INDEL	18256.59	26	8	7	15	17	23	33	30	19	25
Chr01	13931753	A	ATTTG	Chr01_13931	A_ATTTG	INDEL	18211.59	26	8	7	15	17	23	33	30	19	25
Chr01	13931753	A	ATTTG	Chr01_13931	A_ATTTG	INDEL	18211.59	26	8	7	15	17	23	33	30	19	25
Chr01	13931753	A	ATTTG	Chr01_13931	A_ATTTG	INDEL	18211.59	26	8	7	15	17	23	33	30	19	25
Chr01	13931753	A	ATTTG	Chr01_13931	A_ATTTG	INDEL	18211.59	26	8	7	15	17	23	33	30	19	25
Chr01	13931753	A	ATTTG	Chr01_13931	A_ATTTG	INDEL	18211.59	26	8	7	15	17	23	33	30	19	25
Chr01	14092559	G	GAT	Chr01_14092	G_GAT	INDEL	8725.59	0	1	0	10	11	20	12	11	1	0
Chr01	14092559	G	GATAT	Chr01_14092	G_GATAT	INDEL	8725.59	18	10	9	0	1	1	0	0	9	4
Chr01	14247357	C	T	Chr01_14247	C_T	SNP	14834.63	23	11	16	25	8	13	18	24	24	16
Chr01	14247357	C	T	Chr01_14247	C_T	SNP	14834.63	23	11	16	25	8	13	18	24	24	16
Chr01	14324737	A	C	Chr01_14324	A_C	SNP	18277.63	32	10	20	28	22	17	28	17	25	18
Chr01	14324738	TG	T	Chr01_14324	TG_T	INDEL	18268.59	32	10	20	28	22	17	28	17	25	18
Chr01	14415469	C	A	Chr01_14415	C_A	SNP	16392.63	27	12	13	9	21	28	34	24	21	20
Chr01	14528377	GATTTAGGA	G	Chr01_14528	GATTTAGGA	INDEL	9606.59	13	11	10	13	10	16	14	7	6	11
Chr01	14670037	T	C	Chr01_14670	T_C	SNP	17580.63	21	14	16	24	22	30	19	24	23	26
Chr01	14679400	T	A	Chr01_14679	T_A	SNP	19215.63	31	14	17	24	18	36	33	28	18	25
Chr01	14680738	C	A	Chr01_14680	C_A	SNP	20381.63	48	19	19	20	29	25	34	22	20	25
Chr01	14680768	C	T	Chr01_14680	C_T	SNP	19792.63	45	19	19	23	29	23	36	18	19	27
Chr01	14680782	A	C	Chr01_14680	A_C	SNP	20171.63	38	19	17	23	30	24	35	16	22	24
Chr01	14742987	T	C	Chr01_14742	T_C	SNP	17339.63	29	12	14	27	12	22	29	19	28	24
Chr01	14812646	C	T	Chr01_14812	C_T	SNP	20120.63	26	11	14	31	25	22	29	25	26	25
Chr01	14812646	C	T	Chr01_14812	C_T	SNP	20120.63	26	11	14	31	25	22	29	25	26	25
Chr01	14812646	C	T	Chr01_14812	C_T	SNP	20120.63	26	11	14	31	25	22	29	25	26	25
Chr01	14812646	C	T	Chr01_14812	C_T	SNP	20120.63	26	11	14	31	25	22	29	25	26	25
Chr01	14812646	C	T	Chr01_14812	C_T	SNP	20120.63	26	11	14	31	25	22	29	25	26	25
Chr01	14812646	C	T	Chr01_14812	C_T	SNP	20120.63	26	11	14	31	25	22	29	25	26	25
Chr01	14812646	C	T	Chr01_14812	C_T	SNP	20120.63	26	11	14	31	25	22	29	25	26	25
Chr01	14812646	C	T	Chr01_14812	C_T	SNP	20120.63	26	11	14	31	25	22	29	25	26	25
Chr01	14812646	C	T	Chr01_14812	C_T	SNP	20120.63	26	11	14	31	25	22	29	25	26	25
Chr01	14812647	T	A	Chr01_14812	T_A	SNP	20120.63	26	11	14	32	24	22	29	24	25	25
Chr01	14812647	T	A	Chr01_14812	T_A	SNP	20120.63	26	11	14	32	24	22	29	24	25	25
Chr01	14812647	T	A	Chr01_14812	T_A	SNP	20120.63	26	11	14	32	24	22	29	24	25	25
Chr01	14812647	T	A	Chr01_14812	T_A	SNP	20120.63	26	11	14	32	24	22	29	24	25	25
Chr01	14812647	T	A	Chr01_14812	T_A	SNP	20120.63	26	11	14	32	24	22	29	24	25	25
Chr01	14812647	T	A	Chr01_14812	T_A	SNP	20120.63	26	11	14	32	24	22	29	24	25	25
Chr01	14812647	T	A	Chr01_14812	T_A	SNP	20120.63	26	11	14	32	24	22	29	24	25	25
Chr01	14812647	T	A	Chr01_14812	T_A	SNP	20120.63	26	11	14	32	24	22	29	24	25	25
Chr01	14812647	T	A	Chr01_14812	T_A	SNP	20120.63	26	11	14	32	24	22	29	24	25	25

Chr01	14833076	A	G	Chr01_14833	A_G	SNP	18705.63
Chr01	14833076	A	G	Chr01_14833	A_G	SNP	18705.63
Chr01	14833076	A	G	Chr01_14833	A_G	SNP	18705.63
Chr01	15044949	A	AAG	Chr01_15044	A_AAG	INDEL	10377.59
Chr01	15044949	A	AAG	Chr01_15044	A_AAG	INDEL	10377.59
Chr01	15044949	A	AAG	Chr01_15044	A_AAG	INDEL	10377.59
Chr01	15044954	TCC	T	Chr01_15044	TCC_T	INDEL	10649.59
Chr01	15044954	TCC	T	Chr01_15044	TCC_T	INDEL	10649.59
Chr01	15044954	TCC	T	Chr01_15044	TCC_T	INDEL	10649.59
Chr01	15044957	A	G	Chr01_15044	A_G	SNP	10209.63
Chr01	15044957	A	G	Chr01_15044	A_G	SNP	10209.63
Chr01	15044957	A	G	Chr01_15044	A_G	SNP	10209.63
Chr01	15044959	C	CT	Chr01_15044	C_CT	INDEL	10759.59
Chr01	15044959	C	CT	Chr01_15044	C_CT	INDEL	10759.59
Chr01	15044959	C	CT	Chr01_15044	C_CT	INDEL	10759.59
Chr01	15044960	A	ATTATG	Chr01_15044	A_ATTATG	INDEL	10714.59
Chr01	15044960	A	ATTATG	Chr01_15044	A_ATTATG	INDEL	10714.59
Chr01	15044960	A	ATTATG	Chr01_15044	A_ATTATG	INDEL	10714.59
Chr01	15141374	T	TCTTAATAAA	Chr01_15141	T_TCTTAATA	INDEL	19826.59
Chr01	15193225	C	CAT	Chr01_15193	C_CAT	INDEL	7755.24
Chr01	15193225	C	CAT	Chr01_15193	C_CAT	INDEL	7755.24
Chr01	15193225	C	CAT	Chr01_15193	C_CAT	INDEL	7755.24
Chr01	15193225	C	CAT	Chr01_15193	C_CAT	INDEL	7755.24
Chr01	15193225	C	CAT	Chr01_15193	C_CAT	INDEL	7755.24
Chr01	15193225	C	CAT	Chr01_15193	C_CAT	INDEL	7755.24
Chr01	15193225	C	CAT	Chr01_15193	C_CAT	INDEL	7755.24
Chr01	15193225	C	CAT	Chr01_15193	C_CAT	INDEL	7755.24
Chr01	15193225	C	CAT	Chr01_15193	C_CAT	INDEL	7755.24
Chr01	15193225	C	CAT	Chr01_15193	C_CAT	INDEL	7755.24
Chr01	15193225	C	CAT	Chr01_15193	C_CAT	INDEL	7755.24
Chr01	15527811	C	A	Chr01_15527	C_A	SNP	9955.63
Chr01	15527811	C	A	Chr01_15527	C_A	SNP	9955.63
Chr01	15527811	C	A	Chr01_15527	C_A	SNP	9955.63
Chr01	15527811	C	A	Chr01_15527	C_A	SNP	9955.63
Chr01	15527811	C	A	Chr01_15527	C_A	SNP	9955.63
Chr01	15527811	C	A	Chr01_15527	C_A	SNP	9955.63
Chr01	15527811	C	A	Chr01_15527	C_A	SNP	9955.63
Chr01	15527811	C	A	Chr01_15527	C_A	SNP	9955.63
Chr01	15527811	C	A	Chr01_15527	C_A	SNP	9955.63
Chr01	15527811	C	A	Chr01_15527	C_A	SNP	9955.63
Chr01	15570734	C	CT	Chr01_15570	C_CT	INDEL	17297.59
Chr01	15591812	G	A	Chr01_15591	G_A	SNP	5311.29
Chr01	15606993	T	A	Chr01_15606	T_A	SNP	11210.63
Chr01	15766111	C	T	Chr01_15766	C_T	SNP	18598.63
Chr01	15766111	C	T	Chr01_15766	C_T	SNP	18598.63
Chr01	15766111	C	T	Chr01_15766	C_T	SNP	18598.63
Chr01	15766111	C	T	Chr01_15766	C_T	SNP	18598.63
Chr01	15805448	T	A	Chr01_15805	T_A	SNP	16257.63
Chr01	15805449	C	T	Chr01_15805	C_T	SNP	16257.63
Chr01	15864963	A	AAT	Chr01_15864	A_AAT	INDEL	4931.24
Chr01	15864963	A	AAT	Chr01_15864	A_AAT	INDEL	4931.24
Chr01	15864963	A	AAT	Chr01_15864	A_AAT	INDEL	4931.24
Chr01	15864963	A	AAT	Chr01_15864	A_AAT	INDEL	4931.24
Chr01	15864963	A	AAT	Chr01_15864	A_AAT	INDEL	4931.24
Chr01	15864963	A	AAT	Chr01_15864	A_AAT	INDEL	4931.24
Chr01	15864963	A	AAT	Chr01_15864	A_AAT	INDEL	4931.24
Chr01	16008899	C	A	Chr01_16008	C_A	SNP	13434.63
Chr01	16008899	C	A	Chr01_16008	C_A	SNP	13434.63
Chr01	16008899	C	A	Chr01_16008	C_A	SNP	13434.63
Chr01	16008899	C	A	Chr01_16008	C_A	SNP	13434.63
Chr01	16008899	C	A	Chr01_16008	C_A	SNP	13434.63
Chr01	16008899	C	A	Chr01_16008	C_A	SNP	13434.63
Chr01	16008899	C	A	Chr01_16008	C_A	SNP	13434.63
Chr01	16008899	C	A	Chr01_16008	C_A	SNP	13434.63
Chr01	16008918	G	C	Chr01_16008	G_C	SNP	14223.63
Chr01	16008918	G	C	Chr01_16008	G_C	SNP	14223.63
Chr01	16008918	G	C	Chr01_16008	G_C	SNP	14223.63
Chr01	16008918	G	C	Chr01_16008	G_C	SNP	14223.63
Chr01	16008918	G	C	Chr01_16008	G_C	SNP	14223.63
Chr01	16008918	G	C	Chr01_16008	G_C	SNP	14223.63
Chr01	16008918	G	C	Chr01_16008	G_C	SNP	14223.63
Chr01	16008918	G	C	Chr01_16008	G_C	SNP	14223.63
Chr01	16015927	T	G	Chr01_16015	T_G	SNP	19398.63
Chr01	16015927	T	G	Chr01_16015	T_G	SNP	19398.63
Chr01	16015927	T	G	Chr01_16015	T_G	SNP	19398.63
Chr01	16015927	T	G	Chr01_16015	T_G	SNP	19398.63
Chr01	16015927	T	G	Chr01_16015	T_G	SNP	19398.63
Chr01	16015927	T	G	Chr01_16015	T_G	SNP	19398.63
Chr01	16015927	T	G	Chr01_16015	T_G	SNP	19398.63
Chr01	16071886	C	CCTTG	Chr01_16071	C_CCTTG	INDEL	18500.59
Chr01	16071886	C	CCTTG	Chr01_16071	C_CCTTG	INDEL	18500.59
Chr01	16071886	C	CCTTG	Chr01_16071	C_CCTTG	INDEL	18500.59
Chr01	16071886	C	CCTTG	Chr01_16071	C_CCTTG	INDEL	18500.59
Chr01	16071886	C	CCTTG	Chr01_16071	C_CCTTG	INDEL	18500.59
Chr01	16071886	C	CCTTG	Chr01_16071	C_CCTTG	INDEL	18500.59
Chr01	16071886	C	CCTTG	Chr01_16071	C_CCTTG	INDEL	18500.59
Chr01	16071886	C	CCTTG	Chr01_16071	C_CCTTG	INDEL	18500.59
Chr01	16071887	GAGA	G	Chr01_16071	GAGA_G	INDEL	18500.59
Chr01	16071887	GAGA	G	Chr01_16071	GAGA_G	INDEL	18500.59
Chr01	16071887	GAGA	G	Chr01_16071	GAGA_G	INDEL	18500.59
Chr01	16071887	GAGA	G	Chr01_16071	GAGA_G	INDEL	18500.59
Chr01	16071887	GAGA	G	Chr01_16071	GAGA_G	INDEL	18500.59
Chr01	16071887	GAGA	G	Chr01_16071	GAGA_G	INDEL	18500.59
Chr01	16071887	GAGA	G	Chr01_16071	GAGA_G	INDEL	18500.59
Chr01	16071887	GAGA	G	Chr01_16071	GAGA_G	INDEL	18500.59
Chr01	16074417	TA	T	Chr01_16074	TA_T	INDEL	19769.59
Chr01	16074417	TA	T	Chr01_16074	TA_T	INDEL	19769.59
Chr01	16074417	TA	T	Chr01_16074	TA_T	INDEL	19769.59
Chr01	16074417	TA	T	Chr01_16074	TA_T	INDEL	19769.59
Chr01	16074417	TA	T	Chr01_16074	TA_T	INDEL	19769.59
Chr01	16074417	TA	T	Chr01_16074	TA_T	INDEL	19769.59
Chr01	16074417	TA	T	Chr01_16074	TA_T	INDEL	19769.59
Chr01	16074417	TA	T	Chr01_16074	TA_T	INDEL	19769.59
Chr01	16074425	A	T	Chr01_16074	A_T	SNP	19731.63
Chr01	16074425	A	T	Chr01_16074	A_T	SNP	19731.63
Chr01	16074425	A	T	Chr01_16074	A_T	SNP	19731.63
Chr01	16074425	A	T	Chr01_16074	A_T	SNP	19731.63
Chr01	16074425	A	T	Chr01_16074	A_T	SNP	19731.63
Chr01	16074425	A	T	Chr01_16074	A_T	SNP	19731.63
Chr01	16074425	A	T	Chr01_16074	A_T	SNP	19731.63
Chr01	16074425	A	T	Chr01_16074	A_T	SNP	19731.63
Chr01	16074492	AG	A	Chr01_16074	AG_A	INDEL	18237.59
Chr01	16074492	AG	A	Chr01_16074	AG_A	INDEL	18237.59
Chr01	16074492	AG	A	Chr01_16074	AG_A	INDEL	18237.59
Chr01	16074492	AG	A	Chr01_16074	AG_A	INDEL	18237.59
Chr01	16074492	AG	A	Chr01_16074	AG_A	INDEL	18237.59
Chr01	16074492	AG	A	Chr01_16074	AG_A	INDEL	18237.59
Chr01	16074492	AG	A	Chr01_16074	AG_A	INDEL	18237.59
Chr01	16074492	AG	A	Chr01_16074	AG_A	INDEL	18237.59
Chr01	16074597	G	T	Chr01_16074	G_T	SNP	20111.63
Chr01	16074597	G	T	Chr01_16074	G_T	SNP	20111.63
Chr01	16074597	G	T	Chr01_16074	G_T	SNP	20111.63
Chr01	16074597	G	T	Chr01_16074	G_T	SNP	20111.63
Chr01	16074597	G	T	Chr01_16074	G_T	SNP	20111.63
Chr01	16074597	G	T	Chr01_16074	G_T	SNP	20111.63
Chr01	16074597	G	T	Chr01_16074	G_T	SNP	20111.63
Chr01	16074597	G	T	Chr01_16074	G_T	SNP	20111.63
Chr01	16074598	T	G	Chr01_16074	T_G	SNP	20111.63
Chr01	16074598	T	G	Chr01_16074	T_G	SNP	20111.63
Chr01	16074598	T	G	Chr01_16074	T_G	SNP	20111.63
Chr01	16074598	T	G	Chr01_16074	T_G	SNP	20111.63
Chr01	16074598	T	G	Chr01_16074	T_G	SNP	20111.63
Chr01	16074598	T	G	Chr01_16074	T_G	SNP	20111.63
Chr01	16074598	T	G	Chr01_16074	T_G	SNP	20111.63
Chr01	16074598	T	G	Chr01_16074	T_G	SNP	20111.63
Chr01	16075429	TTG	T	Chr01_16075	TTG_T	INDEL	20756.59
Chr01	16075429	TTG	T	Chr01_16075	TTG_T	INDEL	20756.59
Chr01	16075429	TTG	T	Chr01_16075	TTG_T	INDEL	20756.59
Chr01	16075429	TTG	T	Chr01_16075	TTG_T	INDEL	20756.59
Chr01	16075429	TTG	T	Chr01_16075	TTG_T	INDEL	20756.59
Chr01	16075429	TTG	T	Chr01_16075	TTG_T	INDEL	20756.59
Chr01	16075429	TTG	T	Chr01_16075	TTG_T	INDEL	20756.59
Chr01	16075429	TTG	T	Chr01_16075	TTG_T	INDEL	20756.59
Chr01	16075429	TTG	T	Chr01_16075	TTG_T	INDEL	20756.59
Chr01	16075434	CA	C	Chr01_16075	CA_C	INDEL	20666.59
Chr01	16075434	CA	C	Chr01_16075	CA_C	INDEL	20666.59
Chr01	16075434	CA	C	Chr01_16075	CA_C	INDEL	20666.59
Chr01	16075434	CA	C	Chr01_16075	CA_C	INDEL	20666.59
Chr01	16075434	CA	C	Chr01_16075	CA_C	INDEL	20666.59
Chr01	16075434	CA	C	Chr01_16075	CA_C	INDEL	20666.59
Chr01	16075434	CA	C	Chr01_16075	CA_C	INDEL	20666.59
Chr01	16075434	CA	C	Chr01_16075	CA_C	INDEL	20666.59
Chr01	16075434	CA	C	Chr01_16075	CA_C	INDEL	20666.59
Chr01	16075437	TATAATATA	T	Chr01_16075	TATAATATA	INDEL	20396.59
Chr01	16075437	TATAATATA	T	Chr01_16075	TATAATATA	INDEL	20396.59
Chr01	16075437	TATAATATA	T	Chr01_16075	TATAATATA	INDEL	20396.59
Chr01	16075437	TATAATATA	T	Chr01_16075	TATAATATA	INDEL	20396.59
Chr01	16075437	TATAATATA	T	Chr01_16075	TATAATATA	INDEL	20396.59
Chr01	16075437	TATAATATA	T	Chr01_16075	TATAATATA	INDEL	20396.59
Chr01	16075437	TATAATATA	T	Chr01_16075	TATAATATA	INDEL	20396.59
Chr01	16075437	TATAATATA	T	Chr01_16075	TATAATATA	INDEL	20396.59
Chr01	16075437	TATAATATA	T	Chr01_16075	TATAATATA	INDEL	20396.59
Chr01	16075456	CTAA	C	Chr01_16075	CTAA_C	INDEL	20745.59
Chr01	16075456	CTAA	C	Chr01_16075	CTAA_C	INDEL	20745.59
Chr01	16075456	CTAA	C	Chr01_16075	CTAA_C	INDEL	20745.59
Chr01	16075456	CTAA	C	Chr01_16075	CTAA_C	INDEL	20745.59
Chr01	16075456	CTAA	C	Chr01_16075	CTAA_C	INDEL	20745.59
Chr01	16075456	CTAA	C	Chr01_16075	CTAA_C	INDEL	20745.59
Chr01	16075456	CTAA	C	Chr01_16075	CTAA_C	INDEL	20745.59
Chr01	16075456	CTAA	C	Chr01_16075	CTAA_C	INDEL	20745.59
Chr01	16075456	CTAA	C	Chr01_16075	CTAA_C	INDEL	20745.59
Chr01	16075461	CT	C	Chr01_16075	CT_C	INDEL	20689.59
Chr01	16075461	CT	C	Chr01_16075	CT_C	INDEL	20689.59
Chr01	16075461	CT	C	Chr01_16075	CT_C	INDEL	20689.59
Chr01	16075461	CT	C	Chr01_16075	CT_C	INDEL	20689.59
Chr01	16075461	CT	C	Chr01_16075	CT_C	INDEL	20689.59
Chr01	16075461	CT	C	Chr01_16075	CT_C	INDEL	20689.59
Chr01	16075461	CT	C	Chr01_16075	CT_C	INDEL	20689.59
Chr01	16075461	CT	C	Chr01_16075	CT_C	INDEL	20689.59
Chr01	16075461	CT	C	Chr01_16075	CT_C	INDEL	20689.59
Chr01	16075463	T	C	Chr01_16075	T_C	SNP	20563.63
Chr01	16075463	T	C	Chr01_16075	T_C	SNP	20563.63
Chr01	16075463	T	C	Chr01_16075	T_C	SNP	20563.63
Chr01	16075463	T	C	Chr01_16075	T_C	SNP	20563.63
Chr01	16075463	T	C	Chr01_16075	T_C	SNP	20563.63
Chr01	16075463	T	C	Chr01_16075	T_C	SNP	20563.63
Chr01	16075463	T	C	Chr01_16075	T_C	SNP	20563.63
Chr01	16075463	T	C	Chr01_16075	T_C	SNP	20563.63
Chr01	16075463	T	C	Chr01_16075	T_C	SNP	20563.63
Chr01	16298705	G	A	Chr01_16298	G_A	SNP	21714.63
Chr01	16298705	G	A	Chr01_16298	G_A	SNP	21714.63
Chr01	16298705	G	A	Chr01_16298	G_A	SNP	21714.63
Chr01	16298705	G	A	Chr01_16298	G_A	SNP	21714.63
Chr01	16298705	G	A	Chr01_16298	G_A	SNP	21714.63
Chr01	16331294	CATATAT	C	Chr01_16331	CATATAT_C	INDEL	168.37
Chr01	16331294	CATATAT	C	Chr01_16331	CATATAT_C	INDEL	168.37
Chr01	16331294	CATATAT	C	Chr01_16331	CATATAT_C	INDEL	168.37
Chr01	16331294	CATATAT	C	Chr01_16331	CATATAT_C	INDEL	168.37
Chr01	16331294	CATATAT	C	Chr01_16331	CATATAT_C	INDEL	168.37
Chr01	16331294	CATATAT	C	Chr01_16331	CATATAT_C	INDEL	168.37
Chr01	16331294	CATATAT	C	Chr01_16331	CATATAT_C	INDEL	168.37
Chr01	16588025	A	G	Chr01_16588	A_G	SNP	3755.98

Chr01	16856926	T	TTA	Chr01_16856T_TTA	INDEL	18437.59	23	16	10	28	24	20	33	14	22	26	18	16	11	9	[illegible]
Chr01	16856926	T	TTA	Chr01_16856T_TTA	INDEL	18437.59	23	16	10	28	24	20	33	14	22	26	18	16	11	9	[illegible]
Chr01	16856926	T	TTA	Chr01_16856T_TTA	INDEL	18437.59	23	16	10	28	24	20	33	14	22	26	18	16	11	9	[illegible]
Chr01	16856926	T	TTA	Chr01_16856T_TTA	INDEL	18437.59	23	16	10	28	24	20	33	14	22	26	18	16	11	9	[illegible]
Chr01	16856926	T	TTA	Chr01_16856T_TTA	INDEL	18437.59	23	16	10	28	24	20	33	14	22	26	18	16	11	9	[illegible]
Chr01	16904078	AAT	A	Chr01_16904AAT_A	INDEL	8921.59	17	8	6	11	27	15	32	17	15	12	11	12	9	5	[illegible]
Chr01	16904078	AAT	A	Chr01_16904AAT_A	INDEL	8921.59	17	8	6	11	27	15	32	17	15	12	11	12	9	5	[illegible]
Chr01	16904078	AAT	A	Chr01_16904AAT_A	INDEL	8921.59	17	8	6	11	27	15	32	17	15	12	11	12	9	5	[illegible]
Chr01	16904078	AAT	A	Chr01_16904AAT_A	INDEL	8921.59	17	8	6	11	27	15	32	17	15	12	11	12	9	5	[illegible]
Chr01	16904078	AAT	A	Chr01_16904AAT_A	INDEL	8921.59	17	8	6	11	27	15	32	17	15	12	11	12	9	5	[illegible]
Chr01	16904078	AAT	A	Chr01_16904AAT_A	INDEL	8921.59	17	8	6	11	27	15	32	17	15	12	11	12	9	5	[illegible]
Chr01	17010466	C	T	Chr01_17010C_T	SNP	19076.63	27	21	17	21	30	21	20	21	12	21	19	14	11	7	[illegible]
Chr01	17010488	G	T	Chr01_17010G_T	SNP	19704.63	30	22	19	22	26	23	21	19	13	21	19	10	9	9	[illegible]
Chr01	17010489	A	G	Chr01_17010A_G	SNP	19524.63	30	22	20	22	27	23	21	20	13	21	19	11	9	9	[illegible]
Chr01	17171827	A	G	Chr01_17171A_G	SNP	20945.63	27	16	16	24	17	19	31	26	22	32	22	18	12	13	[illegible]
Chr01	17171835	G	A	Chr01_17171G_A	SNP	20933.63	29	19	16	24	17	19	31	23	20	32	22	16	12	14	[illegible]
Chr01	17299149	C	G	Chr01_17299C_G	SNP	9027.63	12	6	5	4	8	17	17	15	12	9	11	10	5	6	[illegible]
Chr01	17299149	C	G	Chr01_17299C_G	SNP	9027.63	12	6	5	4	8	17	17	15	12	9	11	10	5	6	[illegible]
Chr01	17299149	C	G	Chr01_17299C_G	SNP	9027.63	12	6	5	4	8	17	17	15	12	9	11	10	5	6	[illegible]
Chr01	17299149	C	G	Chr01_17299C_G	SNP	9027.63	12	6	5	4	8	17	17	15	12	9	11	10	5	6	[illegible]
Chr01	17299149	C	G	Chr01_17299C_G	SNP	9027.63	12	6	5	4	8	17	17	15	12	9	11	10	5	6	[illegible]
Chr01	17299149	C	G	Chr01_17299C_G	SNP	9027.63	12	6	5	4	8	17	17	15	12	9	11	10	5	6	[illegible]
Chr01	17299149	C	G	Chr01_17299C_G	SNP	9027.63	12	6	5	4	8	17	17	15	12	9	11	10	5	6	[illegible]
Chr01	17299149	C	G	Chr01_17299C_G	SNP	9027.63	12	6	5	4	8	17	17	15	12	9	11	10	5	6	[illegible]
Chr01	17299149	C	G	Chr01_17299C_G	SNP	9027.63	12	6	5	4	8	17	17	15	12	9	11	10	5	6	[illegible]
Chr01	17299149	C	G	Chr01_17299C_G	SNP	9027.63	12	6	5	4	8	17	17	15	12	9	11	10	5	6	[illegible]
Chr01	17299149	C	G	Chr01_17299C_G	SNP	9027.63	12	6	5	4	8	17	17	15	12	9	11	10	5	6	[illegible]
Chr01	17299149	C	G	Chr01_17299C_G	SNP	9027.63	12	6	5	4	8	17	17	15	12	9	11	10	5	6	[illegible]
Chr01	17299197	A	T	Chr01_17299A_T	SNP	14044.63	16	7	8	12	17	20	28	19	23	15	18	15	10	8	[illegible]
Chr01	17299197	A	T	Chr01_17299A_T	SNP	14044.63	16	7	8	12	17	20	28	19	23	15	18	15	10	8	[illegible]
Chr01	17299197	A	T	Chr01_17299A_T	SNP	14044.63	16	7	8	12	17	20	28	19	23	15	18	15	10	8	[illegible]
Chr01	17299197	A	T	Chr01_17299A_T	SNP	14044.63	16	7	8	12	17	20	28	19	23	15	18	15	10	8	[illegible]
Chr01	17299197	A	T	Chr01_17299A_T	SNP	14044.63	16	7	8	12	17	20	28	19	23	15	18	15	10	8	[illegible]
Chr01	17299197	A	T	Chr01_17299A_T	SNP	14044.63	16	7	8	12	17	20	28	19	23	15	18	15	10	8	[illegible]
Chr01	17299197	A	T	Chr01_17299A_T	SNP	14044.63	16	7	8	12	17	20	28	19	23	15	18	15	10	8	[illegible]
Chr01	17299197	A	T	Chr01_17299A_T	SNP	14044.63	16	7	8	12	17	20	28	19	23	15	18	15	10	8	[illegible]
Chr01	17299197	A	T	Chr01_17299A_T	SNP	14044.63	16	7	8	12	17	20	28	19	23	15	18	15	10	8	[illegible]
Chr01	17299197	A	T	Chr01_17299A_T	SNP	14044.63	16	7	8	12	17	20	28	19	23	15	18	15	10	8	[illegible]
Chr01	17299197	A	T	Chr01_17299A_T	SNP	14044.63	16	7	8	12	17	20	28	19	23	15	18	15	10	8	[illegible]
Chr01	17299197	A	T	Chr01_17299A_T	SNP	14044.63	16	7	8	12	17	20	28	19	23	15	18	15	10	8	[illegible]
Chr01	17408802	C	T	Chr01_17408C_T	SNP	2692.98	0	0	0	0	0	0	0	0	13	16	6	5	8	6	[illegible]
Chr01	17481224	C	CAT	Chr01_17481C_CAT	INDEL	11250.59	26	15	9	12	18	16	38	13	13	21	22	10	6	8	[illegible]
Chr01	17481224	C	CAT	Chr01_17481C_CAT	INDEL	11250.59	26	15	9	12	18	16	38	13	13	21	22	10	6	8	[illegible]
Chr01	17481224	C	CAT	Chr01_17481C_CAT	INDEL	11250.59	26	15	9	12	18	16	38	13	13	21	22	10	6	8	[illegible]
Chr01	17481224	C	CAT	Chr01_17481C_CAT	INDEL	11250.59	26	15	9	12	18	16	38	13	13	21	22	10	6	8	[illegible]
Chr01	17481224	C	CAT	Chr01_17481C_CAT	INDEL	11250.59	26	15	9	12	18	16	38	13	13	21	22	10	6	8	[illegible]
Chr01	17481224	C	CAT	Chr01_17481C_CAT	INDEL	11250.59	26	15	9	12	18	16	38	13	13	21	22	10	6	8	[illegible]
[illegible]																					

Chr01	20690969	A	T	Chr01_20690A_T	SNP	21219.63	28	15	17	20	18	27	18	20	29	21
Chr01	20690974	A	T	Chr01_20690A_T	SNP	21451.63	27	15	17	21	19	26	18	22	28	24
Chr01	20786866	G	GT	Chr01_20786G_GT	INDEL	16981.59	25	17	18	31	22	25	27	19	25	18
Chr01	20786866	G	GT	Chr01_20786G_GT	INDEL	16981.59	25	17	18	31	22	25	27	19	25	18
Chr01	20786866	G	GT	Chr01_20786G_GT	INDEL	16981.59	25	17	18	31	22	25	27	19	25	18
Chr01	20786866	G	GT	Chr01_20786G_GT	INDEL	16981.59	25	17	18	31	22	25	27	19	25	18
Chr01	20786866	G	GT	Chr01_20786G_GT	INDEL	16981.59	25	17	18	31	22	25	27	19	25	18
Chr01	20786866	G	GT	Chr01_20786G_GT	INDEL	16981.59	25	17	18	31	22	25	27	19	25	18
Chr01	20786866	G	GT	Chr01_20786G_GT	INDEL	16981.59	25	17	18	31	22	25	27	19	25	18
Chr01	20877786	T	C	Chr01_20877T_C	SNP	19546.63	40	20	21	22	22	27	30	22	16	22
Chr01	21199148	G	A	Chr01_21199G_A	SNP	14252.63	27	14	10	14	12	20	23	23	12	17
Chr01	21199148	G	A	Chr01_21199G_A	SNP	14252.63	27	14	10	14	12	20	23	23	12	17
Chr01	21199148	G	A	Chr01_21199G_A	SNP	14252.63	27	14	10	14	12	20	23	23	12	17
Chr01	21332486	C	A	Chr01_21332C_A	SNP	15914.63	16	8	8	21	12	24	17	21	11	18
Chr01	21332488	C	A	Chr01_21332C_A	SNP	15914.63	16	9	8	21	12	24	17	22	12	17
Chr01	21565844	A	G	Chr01_21565A_G	SNP	5080.29	0	0	0	18	16	19	19	23	0	0
Chr01	21565844	A	G	Chr01_21565A_G	SNP	5080.29	0	0	0	18	16	19	19	23	0	0
Chr01	21565844	A	G	Chr01_21565A_G	SNP	5080.29	0	0	0	18	16	19	19	23	0	0
Chr01	21565844	A	G	Chr01_21565A_G	SNP	5080.29	0	0	0	18	16	19	19	23	0	0
Chr01	21565844	A	G	Chr01_21565A_G	SNP	5080.29	0	0	0	18	16	19	19	23	0	0
Chr01	21672598	T	G	Chr01_21672T_G	SNP	21406.63	30	14	17	30	23	24	23	21	27	25
Chr01	21672598	T	G	Chr01_21672T_G	SNP	21406.63	30	14	17	30	23	24	23	21	27	25
Chr01	21672600	G	T	Chr01_21672G_T	SNP	21406.63	30	14	17	29	23	23	23	21	26	25
Chr01	21672600	G	T	Chr01_21672G_T	SNP	21406.63	30	14	17	29	23	23	23	21	26	25

[illegible]

Chr01	23790160	C	G	Chr01_23790C_G	SNP	16554.63	18	10	19	[illegible]
Chr01	23790160	C	G	Chr01_23790C_G	SNP	16554.63	18	10	19	[illegible]
Chr01	23790160	C	G	Chr01_23790C_G	SNP	16554.63	18	10	19	[illegible]
Chr01	23790160	C	G	Chr01_23790C_G	SNP	16554.63	18	10	19	[illegible]
Chr01	23790160	C	G	Chr01_23790C_G	SNP	16554.63	18	10	19	[illegible]
Chr01	23790160	C	G	Chr01_23790C_G	SNP	16554.63	18	10	19	[illegible]
Chr01	23790160	C	G	Chr01_23790C_G	SNP	16554.63	18	10	19	[illegible]
Chr01	23790160	C	G	Chr01_23790C_G	SNP	16554.63	18	10	19	[illegible]
Chr01	23790160	C	G	Chr01_23790C_G	SNP	16554.63	18	10	19	[illegible]
Chr01	23791045	G	GT	Chr01_23791G_GT	INDEL	15984.59	28	16	6	[illegible]
Chr01	23791045	G	GT	Chr01_23791G_GT	INDEL	15984.59	28	16	6	[illegible]
Chr01	23791045	G	GT	Chr01_23791G_GT	INDEL	15984.59	28	16	6	[illegible]
Chr01	23791045	G	GT	Chr01_23791G_GT	INDEL	15984.59	28	16	6	[illegible]
Chr01	23791045	G	GT	Chr01_23791G_GT	INDEL	15984.59	28	16	6	[illegible]
Chr01	23791045	G	GT	Chr01_23791G_GT	INDEL	15984.59	28	16	6	[illegible]
Chr01	23791045	G	GT	Chr01_23791G_GT	INDEL	15984.59	28	16	6	[illegible]
Chr01	23791045	G	GT	Chr01_23791G_GT	INDEL	15984.59	28	16	6	[illegible]
Chr01	23791045	G	GT	Chr01_23791G_GT	INDEL	15984.59	28	16	6	[illegible]
Chr01	23791045	G	GT	Chr01_23791G_GT	INDEL	15984.59	28	16	6	[illegible]
Chr01	23791045	G	GT	Chr01_23791G_GT	INDEL	15984.59	28	16	6	[illegible]
Chr01	23791045	G	GT	Chr01_23791G_GT	INDEL	15984.59	28	16	6	[illegible]
Chr01	23854311	T	A	Chr01_23854T_A	SNP	16388.63	26	11	13	[illegible]
Chr01	23854311	T	A	Chr01_23854T_A	SNP	16388.63	26	11	13	[illegible]
Chr01	23854311	T	A	Chr01_23854T_A	SNP	16388.63	26	11	13	[illegible]
Chr01	23854311	T	A	Chr01_23854T_A	SNP	16388.63	26	11	13	[illegible]
Chr01	23854311	T	A	Chr01_23854T_A	SNP	16388.63	26	11	13	[illegible]
Chr01	23854311	T	A	Chr01_23854T_A	SNP	16388.63	26	11	13	[illegible]
Chr01	23854311	T	A	Chr01_23854T_A	SNP	16388.63	26	11	13	[illegible]
Chr01	23854311	T	A	Chr01_23854T_A	SNP	16388.63	26	11	13	[illegible]
Chr01	23854311	T	A	Chr01_23854T_A	SNP	16388.63	26	11	13	[illegible]
Chr01	23854311	T	A	Chr01_23854T_A	SNP	16388.63	26	11	13	[illegible]
Chr01	23854311	T	A	Chr01_23854T_A	SNP	16388.63	26	11	13	[illegible]
Chr01	23854311	T	A	Chr01_23854T_A	SNP	16388.63	26	11	13	[illegible]
Chr01	23854311	T	A	Chr01_23854T_A	SNP	16388.63	26	11	13	[illegible]
Chr01	23854358	CG	C	Chr01_23854CG_C	INDEL	16120.59	32	11	13	[illegible]
Chr01	23854358	CG	C	Chr01_23854CG_C	INDEL	16120.59	32	11	13	[illegible]
Chr01	23854358	CG	C	Chr01_23854CG_C	INDEL	16120.59	32	11	13	[illegible]
Chr01	23854358	CG	C	Chr01_23854CG_C	INDEL	16120.59	32	11	13	[illegible]
Chr01	23854358	CG	C	Chr01_23854CG_C	INDEL	16120.59	32	11	13	[illegible]
Chr01	23854358	CG	C	Chr01_23854CG_C	INDEL	16120.59	32	11	13	[illegible]
Chr01	23854358	CG	C	Chr01_23854CG_C	INDEL	16120.59	32	11	13	[illegible]
Chr01	23854358	CG	C	Chr01_23854CG_C	INDEL	16120.59	32	11	13	[illegible]
Chr01	23854358	CG	C	Chr01_23854CG_C	INDEL	16120.59	32	11	13	[illegible]
Chr01	23854358	CG	C	Chr01_23854CG_C	INDEL	16120.59	32	11	13	[illegible]
Chr01	23854358	CG	C	Chr01_23854CG_C	INDEL	16120.59	32	11	13	[illegible]
Chr01	23854358	CG	C	Chr01_23854CG_C	INDEL	16120.59	32	11	13	[illegible]
Chr01	23854358	CG	C	Chr01_23854CG_C	INDEL	16120.59	32	11	13	[illegible]
Chr01	23854360	T	A	Chr01_23854T_A	SNP	16129.63	32	12	13	[illegible]
Chr01	23854360	T	A	Chr01_23854T_A	SNP	16129.63	32	12	13	[illegible]
Chr01	23854360	T	A	Chr01_23854T_A	SNP	16129.63	32	12	13	[illegible]
Chr01	23854360	T	A	Chr01_23854T_A	SNP	16129.63	32	12	13	[illegible]
Chr01	23854360	T	A	Chr01_23854T_A	SNP	16129.63	32	12	13	[illegible]
Chr01	23854360	T	A	Chr01_23854T_A	SNP	16129.63	32	12	13	[illegible]
Chr01	23854360	T	A	Chr01_23854T_A	SNP	16129.63	32	12	13	[illegible]
Chr01	23854360	T	A	Chr01_23854T_A	SNP	16129.63	32	12	13	[illegible]
Chr01	23854360	T	A	Chr01_23854T_A	SNP	16129.63	32	12	13	[illegible]
Chr01	23854360	T	A	Chr01_23854T_A	SNP	16129.63	32	12	13	[illegible]
Chr01	23854360	T	A	Chr01_23854T_A	SNP	16129.63	32	12	13	[illegible]
Chr01	23854360	T	A	Chr01_23854T_A	SNP	16129.63	32	12	13	[illegible]
Chr01	23854360	T	A	Chr01_23854T_A	SNP	16129.63	32	12	13	[illegible]
Chr01	23890019	G	GT	Chr01_23890G_GT	INDEL	14001.59	18	10	16	[illegible]
Chr01	23890024	C	A	Chr01_23890C_A	SNP	14026.63	18	10	15	[illegible]
Chr01	23890089	A	T	Chr01_23890A_T	SNP	14808.63	25	10	14	[illegible]
Chr01	23957308	A	G	Chr01_23957A_G	SNP	2926.29	0	0	0	[illegible]
Chr01	23957309	G	GTTGTATA	Chr01_23957G_GTTGTAT	INDEL	2916.24	0	0	0	[illegible]
Chr01	24047784	G	T	Chr01_24047G_T	SNP	19278.63	32	12	14	[illegible]
Chr01	24086708	C	CT	Chr01_24086C_CT	INDEL	14012.59	19	18	14	[illegible]
Chr01	24161627	G	C	Chr01_24161G_C	SNP	11133.63	22	4	8	[illegible]
Chr01	24161627	G	C	Chr01_24161G_C	SNP	11133.63	22	4	8	[illegible]
Chr01	24161627	G	C	Chr01_24161G_C	SNP	11133.63	22	4	8	[illegible]
Chr01	24161627	G	C	Chr01_24161G_C	SNP	11133.63	22	4	8	[illegible]
Chr01	24161627	G	C	Chr01_24161G_C	SNP	11133.63	22	4	8	[illegible]
Chr01	24161627	G	C	Chr01_24161G_C	SNP	11133.63	22	4	8	[illegible]
Chr01	24161627	G	C	Chr01_24161G_C	SNP	11133.63	22	4	8	[illegible]
Chr01	24161627	G	C	Chr01_24161G_C	SNP	11133.63	22	4	8	[illegible]
Chr01	24161651	C	A	Chr01_24161C_A	SNP	12302.63	25	5	7	[illegible]
Chr01	24161651	C	A	Chr01_24161C_A	SNP	12302.63	25	5	7	[illegible]
Chr01	24161651	C	A	Chr01_24161C_A	SNP	12302.63	25	5	7	[illegible]
Chr01	24161651	C	A	Chr01_24161C_A	SNP	12302.63	25	5	7	[illegible]
Chr01	24161651	C	A	Chr01_24161C_A	SNP	12302.63	25	5	7	[illegible]
Chr01	24161651	C	A	Chr01_24161C_A	SNP	12302.63	25	5	7	[illegible]
Chr01	24161651	C	A	Chr01_24161C_A	SNP	12302.63	25	5	7	[illegible]
Chr01	24161651	C	A	Chr01_24161C_A	SNP	12302.63	25	5	7	[illegible]
Chr01	24161663	C	A	Chr01_24161C_A	SNP	12205.63	23	5	6	[illegible]
Chr01	24161663	C	A	Chr01_24161C_A	SNP	12205.63	23	5	6	[illegible]
Chr01	24161663	C	A	Chr01_24161C_A	SNP	12205.63	23	5	6	[illegible]
Chr01	24161663	C	A	Chr01_24161C_A	SNP	12205.63	23	5	6	[illegible]
Chr01	24161663	C	A	Chr01_24161C_A	SNP	12205.63	23	5	6	[illegible]
Chr01	24161663	C	A	Chr01_24161C_A	SNP	12205.63	23	5	6	[illegible]
Chr01	24161663	C	A	Chr01_24161C_A	SNP	12205.63	23	5	6	[illegible]
Chr01	24161663	C	A	Chr01_24161C_A	SNP	12205.63	23	5	6	[illegible]
Chr01	24161664	A	G	Chr01_24161A_G	SNP	11935.63	23	5	6	[illegible]
Chr01	24161664	A	G	Chr01_24161A_G	SNP	11935.63	23	5	6	[illegible]
Chr01	24161664	A	G	Chr01_24161A_G	SNP	11935.63	23	5	6	[illegible]
Chr01	24161664	A	G	Chr01_24161A_G	SNP	11935.63	23	5	6	[illegible]
Chr01	24161664	A	G	Chr01_24161A_G	SNP	11935.63	23	5	6	[illegible]
Chr01	24161664	A	G	Chr01_24161A_G	SNP	11935.63	23	5	6	[illegible]
Chr01	24161664	A	G	Chr01_24161A_G	SNP	11935.63	23	5	6	[illegible]
Chr01	24161664	A	G	Chr01_24161A_G	SNP	11935.63	23	5	6	[illegible]
Chr01	24242566	A	T	Chr01_24242A_T	SNP	15764.63	24	14	24	[illegible]
Chr01	24242566	A	T	Chr01_24242A_T	SNP	15764.63	24	14	24	[illegible]
Chr01	24242566	A	T	Chr01_24242A_T	SNP	15764.63	24	14	24	[illegible]
Chr01	24253532	AAT	A	Chr01_24253AAT_A	INDEL	385.37	0	1	0	[illegible]
Chr01	24314361	C	A	Chr01_24314C_A	SNP	19585.63	22	13	19	[illegible]
Chr01	24482552	G	T	Chr01_24482G_T	SNP	11237.63	20	13	13	[illegible]
Chr01	24482552	G	T	Chr01_24482G_T	SNP	11237.63	20	13	13	[illegible]
Chr01	24482552	G	T	Chr01_24482G_T	SNP	11237.63	20	13	13	[illegible]
Chr01	24482552	G	T	Chr01_24482G_T	SNP	11237.63	20	13	13	[illegible]
Chr01	24482552	G	T	Chr01_24482G_T	SNP	11237.63	20	13	13	[illegible]
Chr01	24482552	G	T	Chr01_24482G_T	SNP	11237.63	20	13	13	[illegible]
Chr01	24482552	G	T	Chr01_24482G_T	SNP	11237.63	20	13	13	[illegible]
Chr01	24482552	G	T	Chr01_24482G_T	SNP	11237.63	20	13	13	[illegible]
Chr01	24482552	G	T	Chr01_24482G_T	SNP	11237.63	20	13	13	[illegible]
Chr01	24482552	G	T	Chr01_24482G_T	SNP	11237.63	20	13	13	[illegible]
Chr01	24482552	G	T	Chr01_24482G_T	SNP	11237.63	20	13	13	[illegible]
Chr01	24482552	G	T	Chr01_24482G_T	SNP	11237.63	20	13	13	[illegible]
Chr01	24482552	G	T	Chr01_24482G_T	SNP	11237.63	20	13	13	[illegible]
Chr01	24557916	GA	G	Chr01_24557GA_G	INDEL	9396.59	18	4	9	[illegible]
Chr01	24557916	GA	G	Chr01_24557GA_G	INDEL	9396.59	18	4	9	[illegible]
Chr01	24557916	GA	G	Chr01_24557GA_G	INDEL	9396.59	18	4	9	[illegible]
Chr01	24557916	GA	G	Chr01_24557GA_G	INDEL	9396.59	18	4	9	[illegible]
Chr01	24557916	GA	G	Chr01_24557GA_G	INDEL	9396.59	18	4	9	[illegible]
Chr01	24557916	GA	G	Chr01_24557GA_G	INDEL	9396.59	18	4	9	[illegible]
Chr01	24557916	GA	G	Chr01_24557GA_G	INDEL	9396.59	18	4	9	[illegible]
Chr01	24557916	GA	G	Chr01_24557GA_G	INDEL	9396.59	18	4	9	[illegible]
Chr01	24655115	A	AAT	Chr01_24655A_AAT	INDEL	2326.24	1	0	1	[illegible]
Chr01	24655115	A	AAT	Chr01_24655A_AAT	INDEL	2326.24	1	0	1	[illegible]
Chr01	24655115	A	AAT	Chr01_24655A_AAT	INDEL	2326.24	1	0	1	[illegible]
Chr01	24750628	A	C	Chr01_24750A_C	SNP	19218.63	30	12	9	[illegible]
Chr01	24750628	A	C	Chr01_24750A_C	SNP	19218.63	30	12	9	[illegible]
Chr01	24750628	A	C	Chr01_24750A_C	SNP	19218.63	30	12	9	[illegible]
Chr01	24750629	C	A	Chr01_24750C_A	SNP	19218.63	30	12	9	[illegible]
Chr01	24750629	C	A	Chr01_24750C_A	SNP	19218.63	30	12	9	[illegible]
Chr01	24750629	C	A	Chr01_24750C_A	SNP	19218.63	30	12	9	[illegible]
Chr01	24750676	C	CG	Chr01_24750C_CG	INDEL	21117.59	33	9	16	[illegible]
Chr01	24750676	C	CG	Chr01_24750C_CG	INDEL	21117.59	33	9	16	[illegible]
Chr01	24750676	C	CG	Chr01_24750C_CG	INDEL	21117.59	33	9	16	[illegible]
Chr01	24750680	GA	G	Chr01_24750GA_G	INDEL	21193.59	32	9	18	[illegible]
Chr01	24750680	GA	G	Chr01_24750GA_G	INDEL	21193.59	32	9	18	[illegible]
Chr01	24750680	GA	G	Chr01_24750GA_G	INDEL	21193.59	32	9	18	[illegible]
Chr01	24750711	G	T	Chr01_24750G_T	SNP	19064.63	30	7	19	[illegible]
Chr01	24750711	G	T	Chr01_24750G_T	SNP	19064.63	30	7	19	[illegible]
Chr01	24750711	G	T	Chr01_24750G_T	SNP	19064.63	30	7	19	[illegible]
Chr01	24805420	A	AT	Chr01_24805A_AT	INDEL	14455.59	31	14	19	[illegible]
Chr01	24807440	C	A	Chr01_24807C_A	SNP	15581.63	23	14	16	[illegible]
Chr01	24890652	AAATCCTAA(	A	Chr01_24890AAATCCTAA(	INDEL	15426.59	21	13	6	[illegible]
Chr01	24992907	T	A	Chr01_24992T_A	SNP	18533.63	25	17	15	[illegible]
Chr01	24992908	T	A	Chr01_24992T_A	SNP	18533.63	25	17	15	[illegible]
Chr01	24992909	T	A	Chr01_24992T_A	SNP	18533.63	25	17	15	[illegible]
Chr01	25098540	TTATA	T	Chr01_25098TTATA_T	INDEL	15381.59	27	14	6	[illegible]
Chr01	25098540	TTATA	T	Chr01_25098TTATA_T	INDEL	15381.59	27	14	6	[illegible]
Chr01	25098540	TTATA	T	Chr01_25098TTATA_T	INDEL	15381.59	27	14	6	[illegible]
Chr01	25098540	TTATA	T	Chr01_25098TTATA_T	INDEL	15381.59	27	14	6	[illegible]
Chr01	25098540	TTATA	T	Chr01_25098TTATA_T	INDEL	15381.59	27	14	6	[illegible]
Chr01	25098540	TTATA	T	Chr01_25098TTATA_T	INDEL	15381.59	27	14	6	[illegible]
Chr01	25098540	TTATA	T	Chr01_25098TTATA_T	INDEL	15381.59	27	14	6	[illegible]
Chr01	25098540	TTATA	T	Chr01_25098TTATA_T	INDEL	15381.59	27	14	6	[illegible]
Chr01	25098540	TTATA	T	Chr01_25098TTATA_T	INDEL	15381.59	27	14	6	[illegible]
Chr01	25123643	A	AC	Chr01_25123A_AC	INDEL	12120.59	21	7	4	[illegible]
Chr01	25123643	A	AC	Chr01_25123A_AC	INDEL	12120.59	21	7	4	[illegible]
Chr01	25123643	A	AC	Chr01_25123A_AC	INDEL	12120.59	21	7	4	[illegible]
Chr01	25123643	A	AC	Chr01_25123A_AC	INDEL	12120.59	21	7	4	[illegible]
Chr01	25123643	A	AC	Chr01_25123A_AC	INDEL	12120.59	21	7	4	[illegible]
Chr01	25123643	A	AC	Chr01_25123A_AC	INDEL	12120.59	21	7	4	[illegible]
Chr01	25363236	T	C	Chr01_25363T_C	SNP	19857.63	29	23	15	[illegible]
Chr01	25561532	A	AT	Chr01_25561A_AT	INDEL	17461.59	32	17	17	[illegible]
Chr01	25698904	A	G	Chr01_25698A_G	SNP	13173.63	30	5	12	[illegible]
Chr01	25698904	A	G	Chr01_25698A_G	SNP	13173.63	30	5	12	[illegible]
Chr01	25698930	T	A	Chr01_25698T_A	SNP	14701.63	30	9	13	[illegible]
Chr01	25698930	T	A	Chr01_25698T_A	SNP	14701.63	30	9	13	[illegible]
Chr01	25948463	C	CA	Chr01_25948C_CA	INDEL	16310.59	22	15	16	[illegible]
Chr01	25948463	C	CA	Chr01_25948C_CA	INDEL	16310.59	22	15	16	[illegible]
Chr01	25948463	C	CA	Chr01_25948C_CA	INDEL	16310.59	22	15	16	[illegible]
Chr01	26247533	A	T	Chr01_26247A_T	SNP	7894.63	10	5	8	[illegible]
Chr01	26254863	T	A	Chr01_26254T_A	SNP	19044.63	34	13	17	[illegible]
Chr01	26254947	A	T	Chr01_26254A_T	SNP	11310.63	20	6	9	[illegible]
Chr01	26254949	T	TA	Chr01_26254T_TA	INDEL	11301.59	19	6	7	[illegible]
Chr01	26255251	A	AT	Chr01_26255A_AT	INDEL	16141.59	23	17	12	[illegible]
Chr01	26255251	A	AT	Chr01_26255A_AT	INDEL	16141.59	23	17	12	[illegible]
Chr01	26255251	A	AT	Chr01_26255A_AT	INDEL	16141.59	23	17	12	[illegible]
Chr01	26375729	C	A	Chr01_26375C_A	SNP	17470.63	25	11	11	[illegible]
Chr01	26572712	C	A	Chr01_26572C_A	SNP	20956.63	28	12	9	[illegible]
Chr01	26572724	C	A	Chr01_26572C_A	SNP	20685.63	27	12	7	[illegible]
Chr01	26572752	C	A	Chr01_26572C_A	SNP	19871.63	27	13	11	[illegible]
Chr01	26573814	G	A	Chr01_26573G_A	SNP	17971.63	24	13	20	[illegible]
Chr01	26573874	A	C	Chr01_26573A_C	SNP	16173.63	21	10	23	[illegible]
Chr01	26573875	C	A	Chr01_26573C_A	SNP	16173.63	22	10	23	[illegible]
Chr01	26573912	G	GA	Chr01_26573G_GA	INDEL	11600.59	29	13	19	[illegible]
Chr01	26580098	T	TTA	Chr01_26580T_TTA	INDEL	3077.93	0	0	0	[illegible]
Chr01	26580098	T	TTA	Chr01_26580T_TTA	INDEL	3077.93	0	0	0	[illegible]
Chr01	26580098	T	TTA	Chr01_26580T_TTA	INDEL	3077.93	0	0	0	[illegible]
Chr01	26703756	TGGA	T	Chr01_26703TGGA_T	INDEL	19413.59	27	12	22	[illegible]
Chr01	26703760	A	T	Chr01_26703A_T	SNP	19648.63	27	12	22	[illegible]
Chr01	26703762	G	GTCTTC	Chr01_26703G_GTCTTC	INDEL	20286.59	26	12	22	[illegible]
Chr01	26715070	G	A	Chr01_26715G_A	SNP	3147.98	0	0	0	[illegible]
Chr01	26715070	G	A	Chr01_26715G_A	SNP	3147.98	0	0	0	[illegible]
Chr01	26849618	AG	A	Chr01_26849AG_A	INDEL	18017.59	30	10	19	[illegible]
Chr01	27411094	C	T	Chr01_27411C_T	SNP	8234.63	12	4	6	[illegible]
Chr01	27411095	G	T	Chr01_27411G_T	SNP	8234.63	12	4	6	[illegible]
Chr01	27431951	C	CA	Chr01_27431C_CA	INDEL	15729.59	27	13	9	[illegible]
Chr01	27431951	C	CA	Chr01_27431C_CA	INDEL	15729.59	27	13	9	[illegible]
Chr01	27431951	C	CA	Chr01_27431C_CA	INDEL	15729.59	27	13	9	[illegible]

Chr01	27431951	C	CA	Chr01_27431	C_CA	INDEL	15729.59	27	13	9	[illegible]
Chr01	27431951	C	CA	Chr01_27431	C_CA	INDEL	15729.59	27	13	9	[illegible]
Chr01	27431951	C	CA	Chr01_27431	C_CA	INDEL	15729.59	27	13	9	[illegible]
Chr01	27482739	C	T	Chr01_27482	C_T	SNP	5034.29	0	0	0	[illegible]
Chr01	27524701	C	A	Chr01_27524	C_A	SNP	15639.63	23	17	9	[illegible]
Chr01	27524772	C	G	Chr01_27524	C_G	SNP	15547.63	23	19	14	[illegible]
Chr01	27538320	A	C	Chr01_27538	A_C	SNP	9865.63	17	9	9	[illegible]
Chr01	27548763	A	T	Chr01_27548	A_T	SNP	17849.63	28	9	12	[illegible]
Chr01	27548763	A	T	Chr01_27548	A_T	SNP	17849.63	28	9	12	[illegible]
Chr01	27548763	A	T	Chr01_27548	A_T	SNP	17849.63	28	9	12	[illegible]
Chr01	27548820	T	C	Chr01_27548	T_C	SNP	22016.63	28	11	21	[illegible]
Chr01	27548820	T	C	Chr01_27548	T_C	SNP	22016.63	28	11	21	[illegible]
Chr01	27548820	T	C	Chr01_27548	T_C	SNP	22016.63	28	11	21	[illegible]
Chr01	27548826	A	G	Chr01_27548	A_G	SNP	22149.63	28	11	22	[illegible]
Chr01	27548826	A	G	Chr01_27548	A_G	SNP	22149.63	28	11	22	[illegible]
Chr01	27548826	A	G	Chr01_27548	A_G	SNP	22149.63	28	11	22	[illegible]
Chr01	27630143	G	T	Chr01_27630	G_T	SNP	21515.63	27	15	21	[illegible]
Chr01	27636445	G	T	Chr01_27636	G_T	SNP	14431.63	16	18	18	[illegible]
Chr01	27713331	A	T	Chr01_27713	A_T	SNP	16500.63	25	10	19	[illegible]
Chr01	27713431	G	T	Chr01_27713	G_T	SNP	16891.63	25	13	18	[illegible]
Chr01	27819870	T	TA	Chr01_27819	T_TA	INDEL	2484.5	0	0	0	[illegible]
Chr01	27819870	T	TA	Chr01_27819	T_TA	INDEL	2484.5	0	0	0	[illegible]
Chr01	27819870	T	TA	Chr01_27819	T_TA	INDEL	2484.5	0	0	0	[illegible]
Chr01	28010653	G	T	Chr01_28010	G_T	SNP	2455.39	29	14	16	[illegible]
Chr01	28046682	AT	A	Chr01_28046	AT_A	INDEL	5245.24	0	0	0	[illegible]
Chr01	28046682	AT	A	Chr01_28046	AT_A	INDEL	5245.24	0	0	0	[illegible]
Chr01	28046682	AT	A	Chr01_28046	AT_A	INDEL	5245.24	0	0	0	[illegible]
Chr01	28046682	AT	A	Chr01_28046	AT_A	INDEL	5245.24	0	0	0	[illegible]
Chr01	28112701	G	A	Chr01_28112	G_A	SNP	6585.29	0	0	0	[illegible]
Chr01	28112701	G	A	Chr01_28112	G_A	SNP	6585.29	0	0	0	[illegible]
Chr01	28112701	G	A	Chr01_28112	G_A	SNP	6585.29	0	0	0	[illegible]
Chr01	28165822	G	A	Chr01_28165	G_A	SNP	20703.63	35	18	8	[illegible]
Chr01	28165912	TC	T	Chr01_28165	TC_T	INDEL	19788.59	30	13	10	[illegible]
Chr01	28165919	A	AT	Chr01_28165	A_AT	INDEL	20246.59	30	14	9	[illegible]
Chr01	28178082	A	T	Chr01_28178	A_T	SNP	10203.63	15	6	5	[illegible]
Chr01	28261793	C	CAAATCCTA	Chr01_28261	C_CAAATCCT	INDEL	20513.59	30	11	13	[illegible]
Chr01	28263654	G	C	Chr01_28263	G_C	SNP	12430.63	17	10	11	[illegible]
Chr01	28263672	G	C	Chr01_28263	G_C	SNP	11090.63	14	9	9	[illegible]
Chr01	28263691	G	C	Chr01_28263	G_C	SNP	9847.63	11	8	6	[illegible]
Chr01	28263694	G	C	Chr01_28263	G_C	SNP	9705.63	11	7	6	[illegible]
Chr01	28263795	G	C	Chr01_28263	G_C	SNP	13096.63	18	6	11	[illegible]
Chr01	28264082	G	A	Chr01_28264	G_A	SNP	15177.63	23	23	15	[illegible]
Chr01	28264091	G	A	Chr01_28264	G_A	SNP	16070.63	23	24	16	[illegible]
Chr01	28306644	A	C	Chr01_28306	A_C	SNP	16217.63	25	16	16	[illegible]
Chr01	28306644	A	C	Chr01_28306	A_C	SNP	16217.63	25	16	16	[illegible]
Chr01	28306644	A	C	Chr01_28306	A_C	SNP	16217.63	25	16	16	[illegible]
Chr01	28306644	A	C	Chr01_28306	A_C	SNP	16217.63	25	16	16	[illegible]
Chr01	28306644	A	C	Chr01_28306	A_C	SNP	16217.63	25	16	16	[illegible]
Chr01	28306644	A	C	Chr01_28306	A_C	SNP	16217.63	25	16	16	[illegible]
Chr01	28306647	A	C	Chr01_28306	A_C	SNP	16200.63	24	17	16	[illegible]
Chr01	28306647	A	C	Chr01_28306	A_C	SNP	16200.63	24	17	16	[illegible]
Chr01	28306647	A	C	Chr01_28306	A_C	SNP	16200.63	24	17	16	[illegible]
Chr01	28306647	A	C	Chr01_28306	A_C	SNP	16200.63	24	17	16	[illegible]
Chr01	28306647	A	C	Chr01_28306	A_C	SNP	16200.63	24	17	16	[illegible]
Chr01	28306647	A	C	Chr01_28306	A_C	SNP	16200.63	24	17	16	[illegible]
Chr01	28329710	A	G	Chr01_28329	A_G	SNP	13212.63	23	14	14	[illegible]
Chr01	28395993	A	AAT	Chr01_28395	A_AAT	INDEL	14058.59	19	12	17	[illegible]
Chr01	28395993	A	AAT	Chr01_28395	A_AAT	INDEL	14058.59	19	12	17	[illegible]
Chr01	28395993	A	AAT	Chr01_28395	A_AAT	INDEL	14058.59	19	12	17	[illegible]
Chr01	28396200	A	AAT	Chr01_28396	A_AAT	INDEL	2648.93	0	0	0	[illegible]
Chr01	28396200	A	AAT	Chr01_28396	A_AAT	INDEL	2648.93	0	0	0	[illegible]
Chr01	28396200	A	AAT	Chr01_28396	A_AAT	INDEL	2648.93	0	0	0	[illegible]
Chr01	28578073	G	A	Chr01_28578	G_A	SNP	14412.63	33	8	11	[illegible]
Chr01	28578164	G	T	Chr01_28578	G_T	SNP	9830.63	19	7	10	[illegible]
Chr01	28706380	C	T	Chr01_28706	C_T	SNP	11574.29	21	17	13	[illegible]
Chr01	28706561	C	CA	Chr01_28706	C_CA	INDEL	15502.59	24	17	14	[illegible]
Chr01	28726267	AG	A	Chr01_28726	AG_A	INDEL	18260.59	39	19	21	[illegible]
Chr01	28726334	AT	A	Chr01_28726	AT_A	INDEL	17249.59	43	20	18	[illegible]
Chr01	28776723	GTA	G	Chr01_28776	GTA_G	INDEL	6728.24	20	16	9	[illegible]
Chr01	28776723	GTA	G	Chr01_28776	GTA_G	INDEL	6728.24	20	16	9	[illegible]
Chr01	28776723	GTA	G	Chr01_28776	GTA_G	INDEL	6728.24	20	16	9	[illegible]
Chr01	28776723	GTA	G	Chr01_28776	GTA_G	INDEL	6728.24	20	16	9	[illegible]
Chr01	28776723	GTA	G	Chr01_28776	GTA_G	INDEL	6728.24	20	16	9	[illegible]
Chr01	29229011	A	AAT	Chr01_29229	A_AAT	INDEL	8648.59	22	14	11	[illegible]
Chr01	29392285	G	C	Chr01_29392	G_C	SNP	18681.63	17	11	8	[illegible]
Chr01	29406072	A	C	Chr01_29406	A_C	SNP	19427.63	30	15	15	[illegible]
Chr01	29442529	A	AG	Chr01_29442	A_AG	INDEL	13203.59	35	13	18	[illegible]
Chr01	29447042	C	CT	Chr01_29447	C_CT	INDEL	16201.59	34	26	12	[illegible]
Chr01	29488639	A	G	Chr01_29488	A_G	SNP	14752.63	26	15	9	[illegible]
Chr01	29695760	C	A	Chr01_29695	C_A	SNP	21095.63	26	18	13	[illegible]
Chr01	29695760	C	A	Chr01_29695	C_A	SNP	21095.63	26	18	13	[illegible]
Chr01	29695760	C	A	Chr01_29695	C_A	SNP	21095.63	26	18	13	[illegible]
Chr01	29695760	C	A	Chr01_29695	C_A	SNP	21095.63	26	18	13	[illegible]
Chr01	29695760	C	A	Chr01_29695	C_A	SNP	21095.63	26	18	13	[illegible]
Chr01	29695760	C	A	Chr01_29695	C_A	SNP	21095.63	26	18	13	[illegible]
Chr01	29695761	T	TCGAAACCG	Chr01_29695	T_TCGAAAC	INDEL	21131.59	25	19	13	[illegible]
Chr01	29695761	T	TCGAAACCG	Chr01_29695	T_TCGAAAC	INDEL	21131.59	25	19	13	[illegible]
Chr01	29695761	T	TCGAAACCG	Chr01_29695	T_TCGAAAC	INDEL	21131.59	25	19	13	[illegible]
Chr01	29695761	T	TCGAAACCG	Chr01_29695	T_TCGAAAC	INDEL	21131.59	25	19	13	[illegible]
Chr01	29695761	T	TCGAAACCG	Chr01_29695	T_TCGAAAC	INDEL	21131.59	25	19	13	[illegible]
Chr01	29695761	T	TCGAAACCG	Chr01_29695	T_TCGAAAC	INDEL	21131.59	25	19	13	[illegible]
Chr01	29695765	CCAA	C	Chr01_29695	CCAA_C	INDEL	19451.59	31	23	12	[illegible]
Chr01	29695765	CCAA	C	Chr01_29695	CCAA_C	INDEL	19451.59	31	23	12	[illegible]
Chr01	29695765	CCAA	C	Chr01_29695	CCAA_C	INDEL	19451.59	31	23	12	[illegible]
Chr01	29695765	CCAA	C	Chr01_29695	CCAA_C	INDEL	19451.59	31	23	12	[illegible]
Chr01	29695765	CCAA	C	Chr01_29695	CCAA_C	INDEL	19451.59	31	23	12	[illegible]
Chr01	29695765	CCAA	C	Chr01_29695	CCAA_C	INDEL	19451.59	31	23	12	[illegible]
Chr01	29695769	A	T	Chr01_29695	A_T	SNP	19029.63	31	23	12	[illegible]
Chr01	29695769	A	T	Chr01_29695	A_T	SNP	19029.63	31	23	12	[illegible]
Chr01	29695769	A	T	Chr01_29695	A_T	SNP	19029.63	31	23	12	[illegible]
Chr01	29695769	A	T	Chr01_29695	A_T	SNP	19029.63	31	23	12	[illegible]
Chr01	29695769	A	T	Chr01_29695	A_T	SNP	19029.63	31	23	12	[illegible]
Chr01	29695769	A	T	Chr01_29695	A_T	SNP	19029.63	31	23	12	[illegible]
Chr01	29695773	CATTGCGAA	C	Chr01_29695	CATTGCGAA_	INDEL	18960.59	32	25	12	[illegible]
Chr01	29695773	CATTGCGAA	C	Chr01_29695	CATTGCGAA_	INDEL	18960.59	32	25	12	[illegible]
Chr01	29695773	CATTGCGAA	C	Chr01_29695	CATTGCGAA_	INDEL	18960.59	32	25	12	[illegible]
Chr01	29695773	CATTGCGAA	C	Chr01_29695	CATTGCGAA_	INDEL	18960.59	32	25	12	[illegible]
Chr01	29695773	CATTGCGAA	C	Chr01_29695	CATTGCGAA_	INDEL	18960.59	32	25	12	[illegible]
Chr01	29695773	CATTGCGAA	C	Chr01_29695	CATTGCGAA_	INDEL	18960.59	32	25	12	[illegible]
Chr01	29884338	C	A	Chr01_29884	C_A	SNP	16728.63	34	8	9	[illegible]
Chr01	29884408	A	G	Chr01_29884	A_G	SNP	17281.63	27	6	9	[illegible]
Chr01	29884415	G	A	Chr01_29884	G_A	SNP	17083.63	25	7	10	[illegible]
Chr01	29914259	T	G	Chr01_29914	T_G	SNP	17708.63	27	15	13	[illegible]
Chr01	29991690	G	T	Chr01_29991	G_T	SNP	11016.63	16	16	6	[illegible]
Chr01	29991690	G	T	Chr01_29991	G_T	SNP	11016.63	16	16	6	[illegible]
Chr01	29991690	G	T	Chr01_29991	G_T	SNP	11016.63	16	16	6	[illegible]
Chr01	29991690	G	T	Chr01_29991	G_T	SNP	11016.63	16	16	6	[illegible]
Chr01	29991690	G	T	Chr01_29991	G_T	SNP	11016.63	16	16	6	[illegible]
Chr01	29991690	G	T	Chr01_29991	G_T	SNP	11016.63	16	16	6	[illegible]
Chr01	29991690	G	T	Chr01_29991	G_T	SNP	11016.63	16	16	6	[illegible]
Chr01	29991690	G	T	Chr01_29991	G_T	SNP	11016.63	16	16	6	[illegible]
Chr01	29991690	G	T	Chr01_29991	G_T	SNP	11016.63	16	16	6	[illegible]
Chr01	29991690	G	T	Chr01_29991	G_T	SNP	11016.63	16	16	6	[illegible]
Chr01	29991690	G	T	Chr01_29991	G_T	SNP	11016.63	16	16	6	[illegible]
Chr01	30111959	C	A	Chr01_30111	C_A	SNP	20818.63	30	17	24	[illegible]
Chr01	30111959	C	A	Chr01_30111	C_A	SNP	20818.63	30	17	24	[illegible]
Chr01	30111959	C	A	Chr01_30111	C_A	SNP	20818.63	30	17	24	[illegible]
Chr01	30111959	C	A	Chr01_30111	C_A	SNP	20818.63	30	17	24	[illegible]
Chr01	30111959	C	A	Chr01_30111	C_A	SNP	20818.63	30	17	24	[illegible]
Chr01	30111959	C	A	Chr01_30111	C_A	SNP	20818.63	30	17	24	[illegible]
Chr01	30112083	C	A	Chr01_30112	C_A	SNP	18846.63	27	11	25	[illegible]
Chr01	30112083	C	A	Chr01_30112	C_A	SNP	18846.63	27	11	25	[illegible]
Chr01	30112083	C	A	Chr01_30112	C_A	SNP	18846.63	27	11	25	[illegible]
Chr01	30112083	C	A	Chr01_30112	C_A	SNP	18846.63	27	11	25	[illegible]
Chr01	30112083	C	A	Chr01_30112	C_A	SNP	18846.63	27	11	25	[illegible]
Chr01	30112083	C	A	Chr01_30112	C_A	SNP	18846.63	27	11	25	[illegible]
Chr01	30112217	G	A	Chr01_30112	G_A	SNP	9597.63	18	7	11	[illegible]
Chr01	30112217	G	A	Chr01_30112	G_A	SNP	9597.63	18	7	11	[illegible]
Chr01	30112217	G	A	Chr01_30112	G_A	SNP	9597.63	18	7	11	[illegible]
Chr01	30112217	G	A	Chr01_30112	G_A	SNP	9597.63	18	7	11	[illegible]
Chr01	30112217	G	A	Chr01_30112	G_A	SNP	9597.63	18	7	11	[illegible]
Chr01	30112217	G	A	Chr01_30112	G_A	SNP	9597.63	18	7	11	[illegible]
Chr01	30112225	CA	C	Chr01_30112	CA_C	INDEL	8044.59	19	7	12	[illegible]
Chr01	30112225	CA	C	Chr01_30112	CA_C	INDEL	8044.59	19	7	12	[illegible]
Chr01	30112225	CA	C	Chr01_30112	CA_C	INDEL	8044.59	19	7	12	[illegible]
Chr01	30112225	CA	C	Chr01_30112	CA_C	INDEL	8044.59	19	7	12	[illegible]
Chr01	30112225	CA	C	Chr01_30112	CA_C	INDEL	8044.59	19	7	12	[illegible]
Chr01	30112225	CA	C	Chr01_30112	CA_C	INDEL	8044.59	19	7	12	[illegible]
Chr01	30151929	G	A	Chr01_30151	G_A	SNP	11362.63	31	8	11	[illegible]
Chr01	30152026	G	A	Chr01_30152	G_A	SNP	16168.63	36	16	12	[illegible]
Chr01	30200465	A	G	Chr01_30200	A_G	SNP	17763.63	33	18	11	[illegible]
Chr01	30200465	A	G	Chr01_30200	A_G	SNP	17763.63	33	18	11	[illegible]
Chr01	30200465	A	G	Chr01_30200	A_G	SNP	17763.63	33	18	11	[illegible]
Chr01	30200465	A	G	Chr01_30200	A_G	SNP	17763.63	33	18	11	[illegible]
Chr01	30200465	A	G	Chr01_30200	A_G	SNP	17763.63	33	18	11	[illegible]
Chr01	30200469	T	A	Chr01_30200	T_A	SNP	18026.63	33	18	12	[illegible]
Chr01	30200469	T	A	Chr01_30200	T_A	SNP	18026.63	33	18	12	[illegible]
Chr01	30200469	T	A	Chr01_30200	T_A	SNP	18026.63	33	18	12	[illegible]
Chr01	30200469	T	A	Chr01_30200	T_A	SNP	18026.63	33	18	12	[illegible]
Chr01	30200469	T	A	Chr01_30200	T_A	SNP	18026.63	33	18	12	[illegible]
Chr01	30200488	T	A	Chr01_30200	T_A	SNP	17788.63	29	17	15	[illegible]
Chr01	30200488	T	A	Chr01_30200	T_A	SNP	17788.63	29	17	15	[illegible]
Chr01	30200488	T	A	Chr01_30200	T_A	SNP	17788.63	29	17	15	[illegible]
Chr01	30200488	T	A	Chr01_30200	T_A	SNP	17788.63	29	17	15	[illegible]
Chr01	30200488	T	A	Chr01_30200	T_A	SNP	17788.63	29	17	15	[illegible]
Chr02	121084	A	AAT	Chr02_12108	A_AAT	INDEL	118.37	0	1	0	[illegible]
Chr02	121084	A	AAT	Chr02_12108	A_AAT	INDEL	118.37	0	1	0	[illegible]
Chr02	121084	A	AAT	Chr02_12108	A_AAT	INDEL	118.37	0	1	0	[illegible]
Chr02	121084	A	AAT	Chr02_12108	A_AAT	INDEL	118.37	0	1	0	[illegible]
Chr02	121084	A	AAT	Chr02_12108	A_AAT	INDEL	118.37	0	1	0	[illegible]
Chr02	121084	A	AAT	Chr02_12108	A_AAT	INDEL	118.37	0	1	0	[illegible]
Chr02	121084	A	AAT	Chr02_12108	A_AAT	INDEL	118.37	0	1	0	[illegible]
Chr02	121084	A	AAT	Chr02_12108	A_AAT	INDEL	118.37	0	1	0	[illegible]
Chr02	202624	C	CA	Chr02_20262	C_CA	INDEL	10165.59	19	18	8	[illegible]
Chr02	202624	C	CA	Chr02_20262	C_CA	INDEL	10165.59	19	18	8	[illegible]
Chr02	202624	C	CA	Chr02_20262	C_CA	INDEL	10165.59	19	18	8	[illegible]
Chr02	202624	C	CA	Chr02_20262	C_CA	INDEL	10165.59	19	18	8	[illegible]
Chr02	202624	C	CA	Chr02_20262	C_CA	INDEL	10165.59	19	18	8	[illegible]
Chr02	241932	G	T	Chr02_24193	G_T	SNP	1801.39	23	12	9	[illegible]
Chr02	241932	G	T	Chr02_24193	G_T	SNP	1801.39	23	12	9	[illegible]
Chr02	241932	G	T	Chr02_24193	G_T	SNP	1801.39	23	12	9	[illegible]
Chr02	241932	G	T	Chr02_24193	G_T	SNP	1801.39	23	12	9	[illegible]
Chr02	241932	G	T	Chr02_24193	G_T	SNP	1801.39	23	12	9	[illegible]
Chr02	313101	G	T	Chr02_31310	G_T	SNP	18847.63	27	12	12	[illegible]
Chr02	313148	G	T	Chr02_31314	G_T	SNP	17873.63	32	11	8	[illegible]
Chr02	535253	T	C	Chr02_53525	T_C	SNP	11576.63	31	5	8	[illegible]
Chr02	829399	G	A	Chr02_82939	G_A	SNP	9049.29	25	19	13	[illegible]
Chr02	829399	G	A	Chr02_82939	G_A	SNP	9049.29	25	19	13	[illegible]
Chr02	829399	G	A	Chr02_82939	G_A	SNP	9049.29	25	19	13	[illegible]
Chr02	863991	G	GTA	Chr02_86399	G_GTA	INDEL	3428.24	1	0	0	[illegible]
Chr02	863991	G	GTATA	Chr02_86399	G_GTATA	INDEL	3428.24	1	0	0	[illegible]
Chr02	892101	T	C	Chr02_89210	T_C	SNP	17238.63	24	19	13	[illegible]
Chr02	998003	CGGATATTTI	C	Chr02_99800	CGGATATTTI	INDEL	14692.59	23	12	9	[illegible]
Chr02	998020	ATGAC	A	Chr02_99802	ATGAC_A	INDEL	15052.59	22	12	11	[illegible]
Chr02	998026	TGCCTTTTCG	T	Chr02_99802	TGCCTTTTCG	INDEL	14861.59	22	13	11	[illegible]
Chr02	998042	T	A	Chr02_99804	T_A	SNP	15275.63	23	11	11	[illegible]
Chr02	998043	AGT	A	Chr02_99804	AGT_A	INDEL	15536.59	22	11	11	[illegible]
Chr02	998046	G	A	Chr02_99804	G_A	SNP	15050.63	22	11	11	[illegible]
Chr02	998050	T	TA	Chr02_99805	T_TA	INDEL	15491.59	22	12	10	[illegible]
Chr02	1002207	C	CT	Chr02_10022	C_CT	INDEL	14791.59	25	18	11	[illegible]
Chr02	1061121	C	CT	Chr02_10611	C_CT	INDEL	17667.59	23	18	19	[illegible]
Chr02	1061121	C	CT	Chr02_10611	C_CT	INDEL	17667.59	23	18	19	[illegible]

Chr02	1061121	C	CT	Chr02_10611	C_CT	INDEL	17667.59	23	18	[illegible]
Chr02	1061121	C	CT	Chr02_10611	C_CT	INDEL	17667.59	23	18	[illegible]
Chr02	1061121	C	CT	Chr02_10611	C_CT	INDEL	17667.59	23	18	[illegible]
Chr02	1061121	C	CT	Chr02_10611	C_CT	INDEL	17667.59	23	18	[illegible]
Chr02	1098294	TTTTCTCATA	T	Chr02_10982	TTTTCTCATA	INDEL	11507.59	14	8	[illegible]
Chr02	1098294	TTTTCTCATA	T	Chr02_10982	TTTTCTCATA	INDEL	11507.59	14	8	[illegible]
Chr02	1098294	TTTTCTCATA	T	Chr02_10982	TTTTCTCATA	INDEL	11507.59	14	8	[illegible]
Chr02	1098294	TTTTCTCATA	T	Chr02_10982	TTTTCTCATA	INDEL	11507.59	14	8	[illegible]
Chr02	1098294	TTTTCTCATA	T	Chr02_10982	TTTTCTCATA	INDEL	11507.59	14	8	[illegible]
Chr02	1098308	GTT	G	Chr02_10983	GTT_G	INDEL	11499.59	14	8	[illegible]
Chr02	1098308	GTT	G	Chr02_10983	GTT_G	INDEL	11499.59	14	8	[illegible]
Chr02	1098308	GTT	G	Chr02_10983	GTT_G	INDEL	11499.59	14	8	[illegible]
Chr02	1098308	GTT	G	Chr02_10983	GTT_G	INDEL	11499.59	14	8	[illegible]
Chr02	1098308	GTT	G	Chr02_10983	GTT_G	INDEL	11499.59	14	8	[illegible]
Chr02	1098311	GCCGTTGTC	G	Chr02_10983	GCCGTTGTC	INDEL	10747.59	14	8	[illegible]
Chr02	1098311	GCCGTTGTC	G	Chr02_10983	GCCGTTGTC	INDEL	10747.59	14	8	[illegible]
Chr02	1098311	GCCGTTGTC	G	Chr02_10983	GCCGTTGTC	INDEL	10747.59	14	8	[illegible]
Chr02	1098311	GCCGTTGTC	G	Chr02_10983	GCCGTTGTC	INDEL	10747.59	14	8	[illegible]
Chr02	1098311	GCCGTTGTC	G	Chr02_10983	GCCGTTGTC	INDEL	10747.59	14	8	[illegible]
Chr02	1100446	A	AACATGTCA	Chr02_11004	A_AACATGT	INDEL	110450.59	124	75	[illegible]
Chr02	1100446	A	AACATGTCA	Chr02_11004	A_AACATGT	INDEL	110450.59	124	75	[illegible]
Chr02	1100446	A	AACATGTCA	Chr02_11004	A_AACATGT	INDEL	110450.59	124	75	[illegible]
Chr02	1100446	A	AACATGTCA	Chr02_11004	A_AACATGT	INDEL	110450.59	124	75	[illegible]
Chr02	1100446	A	AACATGTCA	Chr02_11004	A_AACATGT	INDEL	110450.59	124	75	[illegible]
Chr02	1100446	A	AACATGTCA	Chr02_11004	A_AACATGT	INDEL	110450.59	124	75	[illegible]
Chr02	1100656	A	AGATCAACA	Chr02_11006	A_AGATCAA	INDEL	63143.59	81	58	[illegible]
Chr02	1100656	A	AGATCAACA	Chr02_11006	A_AGATCAA	INDEL	63143.59	81	58	[illegible]
Chr02	1100656	A	AGATCAACA	Chr02_11006	A_AGATCAA	INDEL	63143.59	81	58	[illegible]
Chr02	1100656	A	AGATCAACA	Chr02_11006	A_AGATCAA	INDEL	63143.59	81	58	[illegible]
Chr02	1100656	A	AGATCAACA	Chr02_11006	A_AGATCAA	INDEL	63143.59	81	58	[illegible]
Chr02	1100656	A	AGATCAACA	Chr02_11006	A_AGATCAA	INDEL	63143.59	81	58	[illegible]
Chr02	1100656	A	AGATCAACA	Chr02_11006	A_AGATCAA	INDEL	63143.59	81	58	[illegible]
Chr02	1309115	C	CAT	Chr02_13091	C_CAT	INDEL	3824.24	0	1	[illegible]
Chr02	1309115	C	CAT	Chr02_13091	C_CAT	INDEL	3824.24	0	1	[illegible]
Chr02	1309115	C	CAT	Chr02_13091	C_CAT	INDEL	3824.24	0	1	[illegible]
Chr02	1309115	C	CAT	Chr02_13091	C_CAT	INDEL	3824.24	0	1	[illegible]
Chr02	1309115	C	CAT	Chr02_13091	C_CAT	INDEL	3824.24	0	1	[illegible]
Chr02	1375137	AC	A	Chr02_13751	AC_A	INDEL	19860.59	16	17	[illegible]
Chr02	1375145	GC	G	Chr02_13751	GC_G	INDEL	20213.59	18	17	[illegible]
Chr02	1408823	TA	T	Chr02_14088	TA_T	INDEL	15951.59	23	16	[illegible]
Chr02	1408823	TA	T	Chr02_14088	TA_T	INDEL	15951.59	23	16	[illegible]
Chr02	1565180	C	A	Chr02_15651	C_A	SNP	20909.63	41	9	[illegible]
Chr02	1643282	G	T	Chr02_16432	G_T	SNP	12321.63	19	12	[illegible]
Chr02	1643282	G	T	Chr02_16432	G_T	SNP	12321.63	19	12	[illegible]
Chr02	1643282	G	T	Chr02_16432	G_T	SNP	12321.63	19	12	[illegible]
Chr02	1643282	G	T	Chr02_16432	G_T	SNP	12321.63	19	12	[illegible]
Chr02	1643282	G	T	Chr02_16432	G_T	SNP	12321.63	19	12	[illegible]
Chr02	1643282	G	T	Chr02_16432	G_T	SNP	12321.63	19	12	[illegible]
Chr02	1643294	A	C	Chr02_16432	A_C	SNP	12773.63	22	12	[illegible]
Chr02	1643294	A	C	Chr02_16432	A_C	SNP	12773.63	22	12	[illegible]
Chr02	1643294	A	C	Chr02_16432	A_C	SNP	12773.63	22	12	[illegible]
Chr02	1643294	A	C	Chr02_16432	A_C	SNP	12773.63	22	12	[illegible]
Chr02	1643294	A	C	Chr02_16432	A_C	SNP	12773.63	22	12	[illegible]
Chr02	1643294	A	C	Chr02_16432	A_C	SNP	12773.63	22	12	[illegible]
Chr02	1842841	T	TG	Chr02_18428	T_TG	INDEL	16807.59	29	21	[illegible]
Chr02	1842841	T	TG	Chr02_18428	T_TG	INDEL	16807.59	29	21	[illegible]
Chr02	1842841	T	TG	Chr02_18428	T_TG	INDEL	16807.59	29	21	[illegible]
Chr02	1842841	T	TG	Chr02_18428	T_TG	INDEL	16807.59	29	21	[illegible]
Chr02	1842841	T	TG	Chr02_18428	T_TG	INDEL	16807.59	29	21	[illegible]
Chr02	1842841	T	TG	Chr02_18428	T_TG	INDEL	16807.59	29	21	[illegible]
Chr02	1842841	T	TG	Chr02_18428	T_TG	INDEL	16807.59	29	21	[illegible]
Chr02	1981575	G	T	Chr02_19815	G_T	SNP	14312.63	14	15	[illegible]
Chr02	1981575	G	T	Chr02_19815	G_T	SNP	14312.63	14	15	[illegible]
Chr02	1981575	G	T	Chr02_19815	G_T	SNP	14312.63	14	15	[illegible]
Chr02	1981575	G	T	Chr02_19815	G_T	SNP	14312.63	14	15	[illegible]
Chr02	2012744	G	GAT	Chr02_20127	G_GAT	INDEL	986.35	18	10	[illegible]
Chr02	2096810	T	TTA	Chr02_20968	T_TTA	INDEL	2313.93	1	2	[illegible]
Chr02	2096810	T	TTA	Chr02_20968	T_TTA	INDEL	2313.93	1	2	[illegible]
Chr02	2236481	G	GAT	Chr02_22364	G_GAT	INDEL	4002.24	0	0	[illegible]
Chr02	2236481	G	GAT	Chr02_22364	G_GAT	INDEL	4002.24	0	0	[illegible]
Chr02	2236481	G	GAT	Chr02_22364	G_GAT	INDEL	4002.24	0	0	[illegible]
Chr02	2236481	G	GAT	Chr02_22364	G_GAT	INDEL	4002.24	0	0	[illegible]
Chr02	2236481	G	GAT	Chr02_22364	G_GAT	INDEL	4002.24	0	0	[illegible]
Chr02	2236481	G	GAT	Chr02_22364	G_GAT	INDEL	4002.24	0	0	[illegible]
Chr02	2236481	G	GAT	Chr02_22364	G_GAT	INDEL	4002.24	0	0	[illegible]
Chr02	2525693	A	T	Chr02_25256	A_T	SNP	17272.63	21	13	[illegible]
Chr02	2555139	C	A	Chr02_25551	C_A	SNP	15320.63	27	9	[illegible]
Chr02	2555139	C	A	Chr02_25551	C_A	SNP	15320.63	27	9	[illegible]
Chr02	2555139	C	A	Chr02_25551	C_A	SNP	15320.63	27	9	[illegible]
Chr02	2555139	C	A	Chr02_25551	C_A	SNP	15320.63	27	9	[illegible]
Chr02	2555139	C	A	Chr02_25551	C_A	SNP	15320.63	27	9	[illegible]
Chr02	2555139	C	A	Chr02_25551	C_A	SNP	15320.63	27	9	[illegible]
Chr02	2555139	C	A	Chr02_25551	C_A	SNP	15320.63	27	9	[illegible]
Chr02	2555142	TCCC	T	Chr02_25551	TCCC_T	INDEL	13736.59	27	9	[illegible]
Chr02	2555142	TCCC	T	Chr02_25551	TCCC_T	INDEL	13736.59	27	9	[illegible]
Chr02	2555142	TCCC	T	Chr02_25551	TCCC_T	INDEL	13736.59	27	9	[illegible]
Chr02	2555142	TCCC	T	Chr02_25551	TCCC_T	INDEL	13736.59	27	9	[illegible]
Chr02	2555142	TCCC	T	Chr02_25551	TCCC_T	INDEL	13736.59	27	9	[illegible]
Chr02	2555142	TCCC	T	Chr02_25551	TCCC_T	INDEL	13736.59	27	9	[illegible]
Chr02	2555142	TCCC	T	Chr02_25551	TCCC_T	INDEL	13736.59	27	9	[illegible]
Chr02	2555147	TCCACCC	T	Chr02_25551	TCCACCC_T	INDEL	13826.59	27	7	[illegible]
Chr02	2555147	TCCACCC	T	Chr02_25551	TCCACCC_T	INDEL	13826.59	27	7	[illegible]
Chr02	2555147	TCCACCC	T	Chr02_25551	TCCACCC_T	INDEL	13826.59	27	7	[illegible]
Chr02	2555147	TCCACCC	T	Chr02_25551	TCCACCC_T	INDEL	13826.59	27	7	[illegible]
Chr02	2555147	TCCACCC	T	Chr02_25551	TCCACCC_T	INDEL	13826.59	27	7	[illegible]
Chr02	2555147	TCCACCC	T	Chr02_25551	TCCACCC_T	INDEL	13826.59	27	7	[illegible]
Chr02	2555147	TCCACCC	T	Chr02_25551	TCCACCC_T	INDEL	13826.59	27	7	[illegible]
Chr02	2555155	T	TTA	Chr02_25551	T_TTA	INDEL	14240.59	28	8	[illegible]
Chr02	2555155	T	TTA	Chr02_25551	T_TTA	INDEL	14240.59	28	8	[illegible]
Chr02	2555155	T	TTA	Chr02_25551	T_TTA	INDEL	14240.59	28	8	[illegible]
Chr02	2555155	T	TTA	Chr02_25551	T_TTA	INDEL	14240.59	28	8	[illegible]
Chr02	2555155	T	TTA	Chr02_25551	T_TTA	INDEL	14240.59	28	8	[illegible]
Chr02	2555155	T	TTA	Chr02_25551	T_TTA	INDEL	14240.59	28	8	[illegible]
Chr02	2555155	T	TTA	Chr02_25551	T_TTA	INDEL	14240.59	28	8	[illegible]
Chr02	2555157	TCTCCCC	T	Chr02_25551	TCTCCCC_T	INDEL	14150.59	30	7	[illegible]
Chr02	2555157	TCTCCCC	T	Chr02_25551	TCTCCCC_T	INDEL	14150.59	30	7	[illegible]
Chr02	2555157	TCTCCCC	T	Chr02_25551	TCTCCCC_T	INDEL	14150.59	30	7	[illegible]
Chr02	2555157	TCTCCCC	T	Chr02_25551	TCTCCCC_T	INDEL	14150.59	30	7	[illegible]
Chr02	2555157	TCTCCCC	T	Chr02_25551	TCTCCCC_T	INDEL	14150.59	30	7	[illegible]
Chr02	2555157	TCTCCCC	T	Chr02_25551	TCTCCCC_T	INDEL	14150.59	30	7	[illegible]
Chr02	2555157	TCTCCCC	T	Chr02_25551	TCTCCCC_T	INDEL	14150.59	30	7	[illegible]
Chr02	2654236	G	T	Chr02_26542	G_T	SNP	20006.63	31	13	[illegible]
Chr02	2682684	C	T	Chr02_26826	C_T	SNP	9472.63	13	11	[illegible]
Chr02	2682684	C	T	Chr02_26826	C_T	SNP	9472.63	13	11	[illegible]
Chr02	2682684	C	T	Chr02_26826	C_T	SNP	9472.63	13	11	[illegible]
Chr02	2682684	C	T	Chr02_26826	C_T	SNP	9472.63	13	11	[illegible]
Chr02	2682684	C	T	Chr02_26826	C_T	SNP	9472.63	13	11	[illegible]
Chr02	2682729	AC	A	Chr02_26827	AC_A	INDEL	9076.59	13	10	[illegible]
Chr02	2682729	AC	A	Chr02_26827	AC_A	INDEL	9076.59	13	10	[illegible]
Chr02	2682729	AC	A	Chr02_26827	AC_A	INDEL	9076.59	13	10	[illegible]
Chr02	2682729	AC	A	Chr02_26827	AC_A	INDEL	9076.59	13	10	[illegible]
Chr02	2682729	AC	A	Chr02_26827	AC_A	INDEL	9076.59	13	10	[illegible]
Chr02	2682867	C	T	Chr02_26828	C_T	SNP	11582.63	14	5	[illegible]
Chr02	2682867	C	T	Chr02_26828	C_T	SNP	11582.63	14	5	[illegible]
Chr02	2682867	C	T	Chr02_26828	C_T	SNP	11582.63	14	5	[illegible]
Chr02	2682867	C	T	Chr02_26828	C_T	SNP	11582.63	14	5	[illegible]
Chr02	2682867	C	T	Chr02_26828	C_T	SNP	11582.63	14	5	[illegible]
Chr02	2682877	C	G	Chr02_26828	C_G	SNP	11687.63	14	5	[illegible]
Chr02	2682877	C	G	Chr02_26828	C_G	SNP	11687.63	14	5	[illegible]
Chr02	2682877	C	G	Chr02_26828	C_G	SNP	11687.63	14	5	[illegible]
Chr02	2682877	C	G	Chr02_26828	C_G	SNP	11687.63	14	5	[illegible]
Chr02	2682877	C	G	Chr02_26828	C_G	SNP	11687.63	14	5	[illegible]
Chr02	2682888	C	A	Chr02_26828	C_A	SNP	10672.63	14	6	[illegible]
Chr02	2682888	C	A	Chr02_26828	C_A	SNP	10672.63	14	6	[illegible]
Chr02	2682888	C	A	Chr02_26828	C_A	SNP	10672.63	14	6	[illegible]
Chr02	2682888	C	A	Chr02_26828	C_A	SNP	10672.63	14	6	[illegible]
Chr02	2682888	C	A	Chr02_26828	C_A	SNP	10672.63	14	6	[illegible]
Chr02	2816380	AT	A	Chr02_28163	AT_A	INDEL	12991.59	21	9	[illegible]
Chr02	2877156	C	T	Chr02_28771	C_T	SNP	17473.63	30	16	[illegible]
Chr02	2877156	C	T	Chr02_28771	C_T	SNP	17473.63	30	16	[illegible]
Chr02	2877156	C	T	Chr02_28771	C_T	SNP	17473.63	30	16	[illegible]
Chr02	2877156	C	T	Chr02_28771	C_T	SNP	17473.63	30	16	[illegible]
Chr02	2877156	C	T	Chr02_28771	C_T	SNP	17473.63	30	16	[illegible]
Chr02	2877156	C	T	Chr02_28771	C_T	SNP	17473.63	30	16	[illegible]
Chr02	2877156	C	T	Chr02_28771	C_T	SNP	17473.63	30	16	[illegible]
Chr02	2877156	C	T	Chr02_28771	C_T	SNP	17473.63	30	16	[illegible]
Chr02	2877156	C	T	Chr02_28771	C_T	SNP	17473.63	30	16	[illegible]
Chr02	3063395	G	A	Chr02_30633	G_A	SNP	4684.98	0	0	[illegible]
Chr02	3063395	G	A	Chr02_30633	G_A	SNP	4684.98	0	0	[illegible]
Chr02	3063395	G	A	Chr02_30633	G_A	SNP	4684.98	0	0	[illegible]
Chr02	3068029	C	CG	Chr02_30680	C_CG	INDEL	15129.59	21	11	[illegible]
Chr02	3068029	C	CG	Chr02_30680	C_CG	INDEL	15129.59	21	11	[illegible]
Chr02	3139538	T	TTA	Chr02_31395	T_TTA	INDEL	8676.59	19	7	[illegible]
Chr02	3139538	T	TTA	Chr02_31395	T_TTA	INDEL	8676.59	19	7	[illegible]
Chr02	3139538	T	TTA	Chr02_31395	T_TTA	INDEL	8676.59	19	7	[illegible]
Chr02	3346917	G	GA	Chr02_33469	G_GA	INDEL	13175.59	20	6	[illegible]
Chr02	3380229	TC	T	Chr02_33802	TC_T	INDEL	10153.59	18	9	[illegible]
Chr02	3380301	GT	G	Chr02_33803	GT_G	INDEL	11143.59	18	11	[illegible]
Chr02	3435479	C	A	Chr02_34354	C_A	SNP	21452.63	33	20	[illegible]
Chr02	3435479	C	A	Chr02_34354	C_A	SNP	21452.63	33	20	[illegible]
Chr02	3435479	C	A	Chr02_34354	C_A	SNP	21452.63	33	20	[illegible]
Chr02	3435479	C	A	Chr02_34354	C_A	SNP	21452.63	33	20	[illegible]
Chr02	3435479	C	A	Chr02_34354	C_A	SNP	21452.63	33	20	[illegible]
Chr02	3435479	C	A	Chr02_34354	C_A	SNP	21452.63	33	20	[illegible]
Chr02	3435479	C	A	Chr02_34354	C_A	SNP	21452.63	33	20	[illegible]
Chr02	3435479	C	A	Chr02_34354	C_A	SNP	21452.63	33	20	[illegible]
Chr02	3435514	G	T	Chr02_34355	G_T	SNP	24600.63	44	17	[illegible]
Chr02	3435514	G	T	Chr02_34355	G_T	SNP	24600.63	44	17	[illegible]
Chr02	3435514	G	T	Chr02_34355	G_T	SNP	24600.63	44	17	[illegible]
Chr02	3435514	G	T	Chr02_34355	G_T	SNP	24600.63	44	17	[illegible]
Chr02	3435514	G	T	Chr02_34355	G_T	SNP	24600.63	44	17	[illegible]
Chr02	3435514	G	T	Chr02_34355	G_T	SNP	24600.63	44	17	[illegible]
Chr02	3435514	G	T	Chr02_34355	G_T	SNP	24600.63	44	17	[illegible]
Chr02	3435514	G	T	Chr02_34355	G_T	SNP	24600.63	44	17	[illegible]
Chr02	3435516	G	T	Chr02_34355	G_T	SNP	24600.63	45	17	[illegible]
Chr02	3435516	G	T	Chr02_34355	G_T	SNP	24600.63	45	17	[illegible]
Chr02	3435516	G	T	Chr02_34355	G_T	SNP	24600.63	45	17	[illegible]
Chr02	3435516	G	T	Chr02_34355	G_T	SNP	24600.63	45	17	[illegible]
Chr02	3435516	G	T	Chr02_34355	G_T	SNP	24600.63	45	17	[illegible]
Chr02	3435516	G	T	Chr02_34355	G_T	SNP	24600.63	45	17	[illegible]
Chr02	3435516	G	T	Chr02_34355	G_T	SNP	24600.63	45	17	[illegible]
Chr02	3435516	G	T	Chr02_34355	G_T	SNP	24600.63	45	17	[illegible]
Chr02	3644810	AAAATATTT	A	Chr02_36448	AAAATATTT	INDEL	15175.59	20	13	[illegible]
Chr02	3644810	AAAATATTT	A	Chr02_36448	AAAATATTT	INDEL	15175.59	20	13	[illegible]
Chr02	3644810	AAAATATTT	A	Chr02_36448	AAAATATTT	INDEL	15175.59	20	13	[illegible]
Chr02	3644810	AAAATATTT	A	Chr02_36448	AAAATATTT	INDEL	15175.59	20	13	[illegible]
Chr02	3644810	AAAATATTT	A	Chr02_36448	AAAATATTT	INDEL	15175.59	20	13	[illegible]
Chr02	3644810	AAAATATTT	A	Chr02_36448	AAAATATTT	INDEL	15175.59	20	13	[illegible]
Chr02	3644810	AAAATATTT	A	Chr02_36448	AAAATATTT	INDEL	15175.59	20	13	[illegible]
Chr02	3644810	AAAATATTT	A	Chr02_36448	AAAATATTT	INDEL	15175.59	20	13	[illegible]
Chr02	3644810	AAAATATTT	A	Chr02_36448	AAAATATTT	INDEL	15175.59	20	13	[illegible]
Chr02	3644810	AAAATATTT	A	Chr02_36448	AAAATATTT	INDEL	15175.59	20	13	[illegible]
Chr02	3644810	AAAATATTT	A	Chr02_36448	AAAATATTT	INDEL	15175.59	20	13	[illegible]
Chr02	3739618	C	T	Chr02_37396	C_T	SNP	19884.63	23	18	[illegible]
Chr02	3739618	C	T	Chr02_37396	C_T	SNP	19884.63	23	18	[illegible]
Chr02	3739618	C	T	Chr02_37396	C_T	SNP	19884.63	23	18	[illegible]
Chr02	3751789	G	T	Chr02_37517	G_T	SNP	17572.63	28	15	[illegible]
Chr02	3769967	CAAGATAAA	C	Chr02_37699	CAAGATAAA	INDEL	16582.59	25	8	[illegible]
Chr02	3769967	CAAGATAAA	C	Chr02_37699	CAAGATAAA	INDEL	16582.59	25	8	[illegible]
Chr02	3769967	CAAGATAAA	C	Chr02_37699	CAAGATAAA	INDEL	16582.59	25	8	[illegible]
Chr02	3769967	CAAGATAAA	C	Chr02_37699	CAAGATAAA	INDEL	16582.59	25	8	[illegible]
Chr02	3769967	CAAGATAAA	C	Chr02_37699	CAAGATAAA	INDEL	16582.59	25	8	[illegible]

Chr02	3769967	CAAGATAAA	C	Chr02_37699	CAAGATAAA	INDEL	16582.59	25	8	12	20	24	24	23	25	15
Chr02	3769967	CAAGATAAA	C	Chr02_37699	CAAGATAAA	INDEL	16582.59	25	8	12	20	24	24	23	25	15
Chr02	3769967	CAAGATAAA	C	Chr02_37699	CAAGATAAA	INDEL	16582.59	25	8	12	20	24	24	23	25	15
Chr02	3769967	CAAGATAAA	C	Chr02_37699	CAAGATAAA	INDEL	16582.59	25	8	12	20	24	24	23	25	15
Chr02	3769967	CAAGATAAA	C	Chr02_37699	CAAGATAAA	INDEL	16582.59	25	8	12	20	24	24	23	25	15
Chr02	3769967	CAAGATAAA	C	Chr02_37699	CAAGATAAA	INDEL	16582.59	25	8	12	20	24	24	23	25	15
Chr02	3769967	CAAGATAAA	C	Chr02_37699	CAAGATAAA	INDEL	16582.59	25	8	12	20	24	24	23	25	15
Chr02	3769967	CAAGATAAA	C	Chr02_37699	CAAGATAAA	INDEL	16582.59	25	8	12	20	24	24	23	25	15
Chr02	3810728	G	GT	Chr02_38107	G_GT	INDEL	19778.59	27	16	12	31	28	21	19	24	31
Chr02	3830451	G	GAT	Chr02_38304	G_GAT	INDEL	9225.59	24	15	8	18	12	24	18	11	17
Chr02	3830451	G	GAT	Chr02_38304	G_GAT	INDEL	9225.59	24	15	8	18	12	24	18	11	17
Chr02	3830451	G	GAT	Chr02_38304	G_GAT	INDEL	9225.59	24	15	8	18	12	24	18	11	17
Chr02	3830451	G	GAT	Chr02_38304	G_GAT	INDEL	9225.59	24	15	8	18	12	24	18	11	17
Chr02	3830451	G	GAT	Chr02_38304	G_GAT	INDEL	9225.59	24	15	8	18	12	24	18	11	17
Chr02	3830451	G	GAT	Chr02_38304	G_GAT	INDEL	9225.59	24	15	8	18	12	24	18	11	17
Chr02	3857219	T	C	Chr02_38572	T_C	SNP	15941.63	23	10	14	19	14	18	22	18	16
Chr02	3918257	T	TTATA	Chr02_39182	T_TTATA	INDEL	1816.35	16	12	10	0	0	0	0	0	0
Chr02	3923461	TTA	T	Chr02_39234	TTA_T	INDEL	670.35	16	8	8	0	0	0	1	1	0
Chr02	3923461	TTA	T	Chr02_39234	TTA_T	INDEL	670.35	16	8	8	0	0	0	1	1	0
Chr02	3923461	TTA	T	Chr02_39234	TTA_T	INDEL	670.35	16	8	8	0	0	0	1	1	0
Chr02	3923461	TTA	T	Chr02_39234	TTA_T	INDEL	670.35	16	8	8	0	0	0	1	1	0
Chr02	3923461	TTA	T	Chr02_39234	TTA_T	INDEL	670.35	16	8	8	0	0	0	1	1	0
Chr02	3923461	TTA	T	Chr02_39234	TTA_T	INDEL	670.35	16	8	8	0	0	0	1	1	0
[illegible]																

Chr02	7880853	C	G	Chr02_78808 C_G	SNP	20246.63	28	12	17	25	25	27	25	22	29	20
Chr02	7880911	G	GA	Chr02_78809 G_GA	INDEL	17586.59	33	12	14	24	26	24	25	20	21	21
Chr02	8042142	T	A	Chr02_80421 T_A	SNP	20058.63	26	12	10	21	27	28	30	20	30	27
Chr02	8042142	T	A	Chr02_80421 T_A	SNP	20058.63	26	12	10	21	27	28	30	20	30	27
Chr02	8042142	T	A	Chr02_80421 T_A	SNP	20058.63	26	12	10	21	27	28	30	20	30	27
Chr02	8042142	T	A	Chr02_80421 T_A	SNP	20058.63	26	12	10	21	27	28	30	20	30	27
Chr02	8143540	C	CAT	Chr02_81435 C_CAT	INDEL	4817.24	21	6	12	0	0	0	0	0	16	15
Chr02	8143540	C	CAT	Chr02_81435 C_CAT	INDEL	4817.24	21	6	12	0	0	0	0	0	16	15
Chr02	8143540	C	CAT	Chr02_81435 C_CAT	INDEL	4817.24	21	6	12	0	0	0	0	0	16	15
Chr02	8143540	C	CAT	Chr02_81435 C_CAT	INDEL	4817.24	21	6	12	0	0	0	0	0	16	15
Chr02	8143540	C	CAT	Chr02_81435 C_CAT	INDEL	4817.24	21	6	12	0	0	0	0	0	16	15
Chr02	8143540	C	CAT	Chr02_81435 C_CAT	INDEL	4817.24	21	6	12	0	0	0	0	0	16	15
Chr02	8215396	T	TTA	Chr02_82153 T_TTA	INDEL	238.37	1	0	0	0	0	0	11	0	0	1
Chr02	8215396	T	TTA	Chr02_82153 T_TTA	INDEL	238.37	1	0	0	0	0	0	11	0	0	1
Chr02	8215396	T	TTA	Chr02_82153 T_TTA	INDEL	238.37	1	0	0	0	0	0	11	0	0	1
Chr02	8215396	T	TTA	Chr02_82153 T_TTA	INDEL	238.37	1	0	0	0	0	0	11	0	0	1
Chr02	8215396	T	TTA	Chr02_82153 T_TTA	INDEL	238.37	1	0	0	0	0	0	11	0	0	1
Chr02	8215396	T	TTA	Chr02_82153 T_TTA	INDEL	238.37	1	0	0	0	0	0	11	0	0	1
Chr02	8215396	T	TTA	Chr02_82153 T_TTA	INDEL	238.37	1	0	0	0	0	0	11	0	0	1
Chr02	8215396	T	TTA	Chr02_82153 T_TTA	INDEL	238.37	1	0	0	0	0	0	11	0	0	1
Chr02	8215396	T	TTA	Chr02_82153 T_TTA	INDEL	238.37	1	0	0	0	0	0	11	0	0	1
Chr02	8215396	T	TTA	Chr02_82153 T_TTA	INDEL	238.37	1	0	0	0	0	0	11	0	0	1
Chr02	8613209	T	C	Chr02_86132 T_C	SNP	14292.63	26	13	15	28	14	24	33	13	18	24
Chr02	8623431	C	T	Chr02_86234 C_T	SNP	5921.29	0	0	0	16	23	15	42	23	0	0
[illegible]																

Chr02	10717287	AAT	A	Chr02_10717	AAT_A	INDEL	2659.93	0	0	0	0	0	0	0	0	17	16	9	23	6	5	18	12	14	28	16	20	29	16	19	17	9	23	6	5	0	0	0	0	0	0	0	0	0.895	0.941	1	1	1	1	0	7	7	0	7	0	0	upstream_ge	MODIFIER	Pp3c2_1583(	Pp3c2_1583(	transcript	PAC:3293540	protein_codi	NA	c.-3059_-305	NA	NA	NA	NA	1179
Chr02	10717287	AAT	A	Chr02_10717	AAT_A	INDEL	2659.93	0	0	0	0	0	0	0	0	17	16	9	23	6	5	18	12	14	28	16	20	29	16	19	17	9	23	6	5	0	0	0	0	0	0	0	0	0.895	0.941	1	1	1	1	0	7	7	0	7	0	0	upstream_ge	MODIFIER	Pp3c2_1583(	Pp3c2_1583(	transcript	PAC:3293540	protein_codi	NA	c.-1689_-168	NA	NA	NA	NA	1179
Chr02	10717287	AAT	A	Chr02_10717	AAT_A	INDEL	2659.93	0	0	0	0	0	0	0	0	17	16	9	23	6	5	18	12	14	28	16	20	29	16	19	17	9	23	6	5	0	0	0	0	0	0	0	0	0.895	0.941	1	1	1	1	0	7	7	0	7	0	0	upstream_ge	MODIFIER	Pp3c2_1583(	Pp3c2_1583(	transcript	PAC:3293540	protein_codi	NA	c.-3545_-354	NA	NA	NA	NA	1179
Chr02	10717287	AAT	A	Chr02_10717	AAT_A	INDEL	2659.93	0	0	0	0	0	0	0	0	17	16	9	23	6	5	18	12	14	28	16	20	29	16	19	17	9	23	6	5	0	0	0	0	0	0	0	0	0.895	0.941	1	1	1	1	0	7	7	0	7	0	0	downstream_	MODIFIER	Pp3c2_1582(	Pp3c2_1582(	transcript	PAC:3293611	protein_codi	NA	c.*1647_*16	NA	NA	NA	NA	1054
Chr02	10717287	AAT	A	Chr02_10717	AAT_A	INDEL	2659.93	0	0	0	0	0	0	0	0	17	16	9	23	6	5	18	12	14	28	16	20	29	16	19	17	9	23	6	5	0	0	0	0	0	0	0	0	0.895	0.941	1	1	1	1	0	7	7	0	7	0	0	downstream_	MODIFIER	Pp3c2_1582(	Pp3c2_1582(	transcript	PAC:3293611	protein_codi	NA	c.*1647_*16	NA	NA	NA	NA	1129
Chr02	10717287	AAT	A	Chr02_10717	AAT_A	INDEL	2659.93	0	0	0	0	0	0	0	0	17	16	9	23	6	5	18	12	14	28	16	20	29	16	19	17	9	23	6	5	0	0	0	0	0	0	0	0	0.895	0.941	1	1	1	1	0	7	7	0	7	0	0	intergenic_re	MODIFIER	Pp3c2_1582(	Pp3c2_1582(	intergenic_re	Pp3c2_1582(	NA	NA	n.10717288_	NA	NA	NA	NA	NA
Chr02	10852931	GATAATACT	G	Chr02_10852	GATAATACT	INDEL	16180.59	28	19	15	23	15	23	22	21	17	20	8	6	11	6	28	19	15	23	15	23	22	21	17	20	8	6	11	6	1	1	1	1	1	1	1	1	1	1	1	1	1	1	0	15	15	0	7	5	3	downstream_	MODIFIER	Pp3c2_1608(	Pp3c2_1608(	transcript	PAC:3293321	protein_codi	NA	c.*3564_*35	NA	NA	NA	NA	2513
Chr02	10852931	GATAATACT	G	Chr02_10852	GATAATACT	INDEL	16180.59	28	19	15	23	15	23	22	21	17	20	8	6	11	6	28	19	15	23	15	23	22	21	17	20	8	6	11	6	1	1	1	1	1	1	1	1	1	1	1	1	1	1	0	15	15	0	7	5	3	downstream_	MODIFIER	Pp3c2_1608(	Pp3c2_1608(	transcript	PAC:3293321	protein_codi	NA	c.*3564_*35	NA	NA	NA	NA	2513
Chr02	10852931	GATAATACT	G	Chr02_10852	GATAATACT	INDEL	16180.59	28	19	15	23	15	23	22	21	17	20	8	6	11	6	28	19	15	23	15	23	22	21	17	20	8	6	11	6	1	1	1	1	1	1	1	1	1	1	1	1	1	1	0	15	15	0	7	5	3	downstream_	MODIFIER	Pp3c2_1608(	Pp3c2_1608(	transcript	PAC:3293322	protein_codi	NA	c.*3564_*35	NA	NA	NA	NA	2513
Chr02	10852931	GATAATACT	G	Chr02_10852	GATAATACT	INDEL	16180.59	28	19	15	23	15	23	22	21	17	20	8	6	11	6	28	19	15	23	15	23	22	21	17	20	8	6	11	6	1	1	1	1	1	1	1	1	1	1	1	1	1	1	0	15	15	0	7	5	3	downstream_	MODIFIER	Pp3c2_1608(	Pp3c2_1608(	transcript	PAC:3293322	protein_codi	NA	c.*3564_*35	NA	NA	NA	NA	2513
Chr02	10852931	GATAATACT	G	Chr02_10852	GATAATACT	INDEL	16180.59	28	19	15	23	15	23	22	21	17	20	8	6	11	6	28	19	15	23	15	23	22	21	17	20	8	6	11	6	1	1	1	1	1	1	1	1	1	1	1	1	1	1	0	15	15	0	7	5	3	downstream_	MODIFIER	Pp3c2_1608(	Pp3c2_1608(	transcript	PAC:3293322	protein_codi	NA	c.*3564_*35	NA	NA	NA	NA	2513
Chr02	10852931	GATAATACT	G	Chr02_10852	GATAATACT	INDEL	16180.59	28	19	15	23	15	23	22	21	17	20	8	6	11	6	28	19	15	23	15	23	22	21	17	20	8	6	11	6	1	1	1	1	1	1	1	1	1	1	1	1	1	1	0	15	15	0	7	5	3	downstream_	MODIFIER	Pp3c2_1608(	Pp3c2_1608(	transcript	PAC:3293321	protein_codi	NA	c.*3564_*35	NA	NA	NA	NA	2513
Chr02	10852931	GATAATACT	G	Chr02_10852	GATAATACT	INDEL	16180.59	28	19	15	23	15	23	22	21	17	20	8	6	11	6	28	19	15	23	15	23	22	21	17	20	8	6	11	6	1	1	1	1	1	1	1	1	1	1	1	1	1	1	0	15	15	0	7	5	3	downstream_	MODIFIER	Pp3c2_1608(	Pp3c2_1608(	transcript	PAC:3293321	protein_codi	NA	c.*3564_*35	NA	NA	NA	NA	2513
Chr02	10852931	GATAATACT	G	Chr02_10852	GATAATACT	INDEL	16180.59	28	19	15	23	15	23	22	21	17	20	8	6	11	6	28	19	15	23	15	23	22	21	17	20	8	6	11	6	1	1	1	1	1	1	1	1	1	1	1	1	1	1	0	15	15	0	7	5	3	downstream_	MODIFIER	Pp3c2_1608(	Pp3c2_1608(	transcript	PAC:3293321	protein_codi	NA	c.*3564_*35	NA	NA	NA	NA	2513
Chr02	10852931	GATAATACT	G	Chr02_10852	GATAATACT	INDEL	16180.59	28	19	15	23	15	23	22	21	17	20	8	6	11	6	28	19	15	23	15	23	22	21	17	20	8	6	11	6	1	1	1	1	1	1	1	1	1	1	1	1	1	1	0	15	15	0	7	5	3	downstream_	MODIFIER	Pp3c2_1608(	Pp3c2_1608(	transcript	PAC:3293322	protein_codi	NA	c.*3564_*35	NA	NA	NA	NA	2513
Chr02	10852931	GATAATACT	G	Chr02_10852	GATAATACT	INDEL	16180.59	28	19	15	23	15	23	22	21	17	20	8	6	11	6	28	19	15	23	15	23	22	21	17	20	8	6	11	6	1	1	1	1	1	1	1	1	1	1	1	1	1	1	0	15	15	0	7	5	3	downstream_	MODIFIER	Pp3c2_1608(	Pp3c2_1608(	transcript	PAC:3293322	protein_codi	NA	c.*3564_*35	NA	NA	NA	NA	2513
Chr02	10852931	GATAATACT	G	Chr02_10852	GATAATACT	INDEL	16180.59	28	19	15	23	15	23	22	21	17	20	8	6	11	6	28	19	15	23	15	23	22	21	17	20	8	6	11	6	1	1	1	1	1	1	1	1	1	1	1	1	1	1	0	15	15	0	7	5	3	downstream_	MODIFIER	Pp3c2_1608(	Pp3c2_1608(	transcript	PAC:3293322	protein_codi	NA	c.*3564_*35	NA	NA	NA	NA	2513
Chr02	10852931	GATAATACT	G	Chr02_10852	GATAATACT	INDEL	16180.59	28	19	15	23	15	23	22	21	17	20	8	6	11	6	28	19	15	23	15	23	22	21	17	20	8	6	11	6	1	1	1	1	1	1	1	1	1	1	1	1	1	1	0	15	15	0	7	5	3	downstream_	MODIFIER	Pp3c2_1608(	Pp3c2_1608(	transcript	PAC:3293322	protein_codi	NA	c.*3564_*35	NA	NA	NA	NA	2513
Chr02	10852931	GATAATACT	G	Chr02_10852	GATAATACT	INDEL	16180.59	28	19	15	23	15	23	22	21	17	20	8	6	11	6	28	19	15	23	15	23	22	21	17	20	8	6	11	6	1	1	1	1	1	1	1	1	1	1	1	1	1	1	0	15	15	0	7	5	3	downstream_	MODIFIER	Pp3c2_1608(	Pp3c2_1608(	transcript	PAC:3293322	protein_codi	NA	c.*3564_*35	NA	NA	NA	NA	2513
Chr02	10852931	GATAATACT	G	Chr02_10852	GATAATACT	INDEL	16180.59	28	19	15	23	15	23	22	21	17	20	8	6	11	6	28	19	15	23	15	23	22	21	17	20	8	6	11	6	1	1	1	1	1	1	1	1	1	1	1	1	1	1	0	15	15	0	7	5	3	intergenic_re	MODIFIER	Pp3c2_1608(	Pp3c2_1608(	intergenic_re	Pp3c2_1608(	NA	NA	n.10852932_	NA	NA	NA	NA	NA
Chr02	10868701	T	C	Chr02_10868	T_C	SNP	4644.98	0	0	0	0	0	0	0	0	26	28	11	14	16	10	21	17	13	19	20	23	32	30	26	28	11	14	17	10	0	0	0	0	0	0	0	0	1	1	1	1	0.941	1	0	7	7	0	7	0	0	intergenic_re	MODIFIER	Pp3c2_1608(	Pp3c2_1608(	intergenic_re	Pp3c2_1608(	NA	NA	n.10868701T	NA	NA	NA	NA	NA
Chr02	10908053	C	A	Chr02_10908	C_A	SNP	19193.63	28	12	11	19	12	34	32	27	19	21	18	14	5	5	28	12	11	19	12	34	32	27	19	21	18	14	5	5	1	1	1	1	1	1	1	1	1	1	1	1	1	1	0	15	15	0	7	5	3	downstream_	MODIFIER	Pp3c2_1615(	Pp3c2_1615(	transcript	PAC:3293698	protein_codi	NA	c.*535C>A	NA	NA	NA	NA	535
Chr02	10908053	C	A	Chr02_10908	C_A	SNP	19193.63	28	12	11	19	12	34	32	27	19	21	18	14	5	5	28	12	11	19	12	34	32	27	19	21	18	14	5	5	1	1	1	1	1	1	1	1	1	1	1	1	1	1	0	15	15	0	7	5	3	intergenic_re	MODIFIER	Pp3c2_1615(	Pp3c2_1615(	intergenic_re	Pp3c2_1615(	NA	NA	n.10908053C	NA	NA	NA	NA	NA
Chr02	10908068	C	A	Chr02_10908	C_A	SNP	20135.63	28	13	10	17	14	36	28	27	22	28	15	16	5	6	28	13	10	17	14	36	28	27	22	28	15	16	5	6	1	1	1	1	1	1	1	1	1	1	1	1	1	1	0	15	15	0	7	5	3	downstream_	MODIFIER	Pp3c2_1615(	Pp3c2_1615(	transcript	PAC:3293698	protein_codi	NA	c.*550C>A	NA	NA	NA	NA	550
Chr02	10908068	C	A	Chr02_10908	C_A	SNP	20135.63	28	13	10	17	14	36	28	27	22	28	15	16	5	6	28	13	10	17	14	36	28	27	22	28	15	16	5	6	1	1	1	1	1	1	1	1	1	1	1	1	1	1	0	15	15	0	7	5	3	intergenic_re	MODIFIER	Pp3c2_1615(	Pp3c2_1615(	intergenic_re	Pp3c2_1615(	NA	NA	n.10908068C	NA	NA	NA	NA	NA
Chr02	11119654	G	A	Chr02_11119	G_A	SNP	9550.63	23	10	12	14	9	16	19	16	8	9	8	6	4	5	23	10	12	14	9	16	19	16	8	9	8	6	4	5	1	1	1	1	1	1	1	1	1	1	1	1	1	1	0	15	15	0	7	5	3	upstream_ge	MODIFIER	Pp3c2_1660(	Pp3c2_1660(	transcript	PAC:3293386	protein_codi	NA	c.-1202C>T	NA	NA	NA	NA	1074
Chr02	11119654	G	A	Chr02_11119	G_A	SNP	9550.63	23	10	12	14	9	16	19	16	8	9	8	6	4	5	23	10	12	14	9	16	19	16	8	9	8	6	4	5	1	1	1	1	1	1	1	1	1	1	1	1	1	1	0	15	15	0	7	5	3	upstream_ge	MODIFIER	Pp3c2_1660(	Pp3c2_1660(	transcript	PAC:3293386	protein_codi	NA	c.-1202C>T	NA	NA	NA	NA	889
Chr02	11119654	G	A	Chr02_11119	G_A	SNP	9550.63	23	10	12	14	9	16	19	16	8	9	8	6	4	5	23	10	12	14	9	16	19	16	8	9	8	6	4	5	1	1	1	1	1	1	1	1	1	1	1	1	1	1	0	15	15	0	7	5	3	downstream_	MODIFIER	Pp3c2_1661(	Pp3c2_1661(	transcript	PAC:3293433	protein_codi	NA	c.*2053C>T	NA	NA	NA	NA	1686
Chr02	11119654	G	A	Chr02_11119	G_A	SNP	9550.63	23	10	12	14	9	16	19	16	8	9	8	6	4	5	23	10	12	14	9	16	19	16	8	9	8	6	4	5	1	1	1	1	1	1	1	1	1	1	1	1	1	1	0	15	15	0	7	5	3	downstream_	MODIFIER	Pp3c2_1661(	Pp3c2_1661(	transcript	PAC:3293433	protein_codi	NA	c.*2053C>T	NA	NA	NA	NA	1679
Chr02	11119654	G	A	Chr02_11119	G_A	SNP	9550.63	23	10	12	14	9	16	19	16	8	9	8	6	4	5	23	10	12	14	9	16	19	16	8	9	8	6	4	5	1	1	1	1	1	1	1	1	1	1	1	1	1	1	0	15	15	0	7	5	3	downstream_	MODIFIER	Pp3c2_1661(	Pp3c2_1661(	transcript	PAC:3293433	protein_codi	NA	c.*2053C>T	NA	NA	NA	NA	1686
Chr02	11119654	G	A	Chr02_11119	G_A	SNP	9550.63	23	10	12	14	9	16	19	16	8	9	8	6	4	5	23	10	12	14	9	16	19	16	8	9	8	6	4	5	1	1	1	1	1	1	1	1	1	1	1	1	1	1	0	15	15	0	7	5	3	downstream_	MODIFIER	Pp3c2_1661(	Pp3c2_1661(	transcript	PAC:3293433	protein_codi	NA	c.*2053C>T	NA	NA	NA	NA	1686
Chr02	11119654	G	A	Chr02_11119	G_A	SNP	9550.63	23	10	12	14	9	16	19	16	8	9	8	6	4	5	23	10	12	14	9	16	19	16	8	9	8	6	4	5	1	1	1	1	1	1	1	1	1	1	1	1	1	1	0	15	15	0	7	5	3	intergenic_re	MODIFIER	Pp3c2_1660(	Pp3c2_1660(	intergenic_re	Pp3c2_1660(	NA	NA	n.11119654G	NA	NA	NA	NA	NA
[illegible]																																																																						

Chr02	11327936	C	CATT	Chr02_11327C_CATT	INDEL	15184.59	21	5	8	23	12	24	22	16	15	18	7	9	6
Chr02	11327936	C	CATT	Chr02_11327C_CATT	INDEL	15184.59	21	5	8	23	12	24	22	16	15	18	7	9	6
Chr02	11327936	C	CATT	Chr02_11327C_CATT	INDEL	15184.59	21	5	8	23	12	24	22	16	15	18	7	9	6
Chr02	11327936	C	CATT	Chr02_11327C_CATT	INDEL	15184.59	21	5	8	23	12	24	22	16	15	18	7	9	6
Chr02	11327936	C	CATT	Chr02_11327C_CATT	INDEL	15184.59	21	5	8	23	12	24	22	16	15	18	7	9	6
Chr02	11327936	C	CATT	Chr02_11327C_CATT	INDEL	15184.59	21	5	8	23	12	24	22	16	15	18	7	9	6
Chr02	11327936	C	CATT	Chr02_11327C_CATT	INDEL	15184.59	21	5	8	23	12	24	22	16	15	18	7	9	6
Chr02	11327936	C	CATT	Chr02_11327C_CATT	INDEL	15184.59	21	5	8	23	12	24	22	16	15	18	7	9	6
Chr02	11327936	C	CATT	Chr02_11327C_CATT	INDEL	15184.59	21	5	8	23	12	24	22	16	15	18	7	9	6
Chr02	11327936	C	CATT	Chr02_11327C_CATT	INDEL	15184.59	21	5	8	23	12	24	22	16	15	18	7	9	6
Chr02	11327936	C	CATT	Chr02_11327C_CATT	INDEL	15184.59	21	5	8	23	12	24	22	16	15	18	7	9	6
Chr02	11327936	C	CATT	Chr02_11327C_CATT	INDEL	15184.59	21	5	8	23	12	24	22	16	15	18	7	9	6
Chr02	11327936	C	CATT	Chr02_11327C_CATT	INDEL	15184.59	21	5	8	23	12	24	22	16	15	18	7	9	6
Chr02	11327936	C	CATT	Chr02_11327C_CATT	INDEL	15184.59	21	5	8	23	12	24	22	16	15	18	7	9	6
Chr02	11392986	T	TA	Chr02_11392T_TA	INDEL	12815.59	28	9	21	11	10	25	11	12	16	24	14	14	14
Chr02	11392986	T	TA	Chr02_11392T_TA	INDEL	12815.59	28	9	21	11	10	25	11	12	16	24	14	14	14
Chr02	11392986	T	TA	Chr02_11392T_TA	INDEL	12815.59	28	9	21	11	10	25	11	12	16	24	14	14	14
Chr02	11413657	G	A	Chr02_11413G_A	SNP	11270.63	9	10	5	17	16	23	9	16	12	17	4	6	8
Chr02	11413662	C	A	Chr02_11413C_A	SNP	11540.63	9	10	5	18	15	23	9	18	12	18	4	6	8
Chr02	11413665	C	A	Chr02_11413C_A	SNP	11765.63	9	10	5	18	15	22	10	18	12	17	4	7	8
Chr02	11413693	G	T	Chr02_11413G_T	SNP	11648.63	10	14	6	18	11	23	9	18	14	17	4	7	8
Chr02	11443782	A	AT	Chr02_11443A_AT	INDEL	17905.59	31	18	9	20	15	18	35	19	19	24	16	12	15
Chr02	11443782	A	AT	Chr02_11443A_AT	INDEL	17905.59	31	18	9	20	15	18	35	19	19	24	16	12	15
Chr02	11443782	A	AT	Chr02_11443A_AT	INDEL	17905.59	31	18	9	20	15	18	35	19	19	24	16	12	15
Chr02	11443782	A	AT	Chr02_11443A_AT	INDEL	17905.59	31	18	9	20	15	18	35	19	19	24	16	12	15
Chr02	11443782	A	AT	Chr02_11443A_AT	INDEL	17905.59	31	18	9	20	15	18	35	19	19	24	16	12	15
Chr02	11443787	A	AT	Chr02_11443A_AT	INDEL	18459.59	30	18	9	22	15	18	35	18	17	25	17	13	15
Chr02	11443787	A	AT	Chr02_11443A_AT	INDEL	18459.59	30	18	9	22	15	18	35	18	17	25	17	13	15
Chr02	11443787	A	AT	Chr02_11443A_AT	INDEL	18459.59	30	18	9	22	15	18	35	18	17	25	17	13	15
Chr02	11443787	A	AT	Chr02_11443A_AT	INDEL	18459.59	30	18	9	22	15	18	35	18	17	25	17	13	15
Chr02	11443787	A	AT	Chr02_11443A_AT	INDEL	18459.59	30	18	9	22	15	18	35	18	17	25	17	13	15

Chr02	12074431	CGG	C	Chr02_12074CGG_C	INDEL	10226.59	17	11	8	10	11	10
Chr02	12074431	CGG	C	Chr02_12074CGG_C	INDEL	10226.59	17	11	8	10	11	10
Chr02	12074431	CGG	C	Chr02_12074CGG_C	INDEL	10226.59	17	11	8	10	11	10
Chr02	12074431	CGG	C	Chr02_12074CGG_C	INDEL	10226.59	17	11	8	10	11	10
Chr02	12074431	CGG	C	Chr02_12074CGG_C	INDEL	10226.59	17	11	8	10	11	10
Chr02	12074431	CGG	C	Chr02_12074CGG_C	INDEL	10226.59	17	11	8	10	11	10
Chr02	12074435	G	A	Chr02_12074G_A	SNP	10152.63	17	10	8	10	11	11
Chr02	12074435	G	A	Chr02_12074G_A	SNP	10152.63	17	10	8	10	11	11
Chr02	12074435	G	A	Chr02_12074G_A	SNP	10152.63	17	10	8	10	11	11
Chr02	12074435	G	A	Chr02_12074G_A	SNP	10152.63	17	10	8	10	11	11
Chr02	12074435	G	A	Chr02_12074G_A	SNP	10152.63	17	10	8	10	11	11
Chr02	12074435	G	A	Chr02_12074G_A	SNP	10152.63	17	10	8	10	11	11
Chr02	12074435	G	A	Chr02_12074G_A	SNP	10152.63	17	10	8	10	11	11
Chr02	12074437	G	A	Chr02_12074G_A	SNP	10062.63	17	10	9	10	12	12
Chr02	12074437	G	A	Chr02_12074G_A	SNP	10062.63	17	10	9	10	12	12
Chr02	12074437	G	A	Chr02_12074G_A	SNP	10062.63	17	10	9	10	12	12
Chr02	12074437	G	A	Chr02_12074G_A	SNP	10062.63	17	10	9	10	12	12
Chr02	12074437	G	A	Chr02_12074G_A	SNP	10062.63	17	10	9	10	12	12
Chr02	12074437	G	A	Chr02_12074G_A	SNP	10062.63	17	10	9	10	12	12
Chr02	12074437	G	A	Chr02_12074G_A	SNP	10062.63	17	10	9	10	12	12
Chr02	12241034	G	C	Chr02_12241G_C	SNP	16894.63	22	12	15	20	26	28
Chr02	12396748	A	AT	Chr02_12396A_AT	INDEL	12691.59	22	9	19	30	9	27
Chr02	12409502	G	A	Chr02_12409G_A	SNP	15276.63	22	8	7	15	19	33
Chr02	12409592	CT	C	Chr02_12409CT_C	INDEL	14945.59	20	5	16	24	16	34
[illegible]	[illegible]	[illegible]	[illegible]	[illegible]	[illegible]	[illegible]	[illegible]	[illegible]	[illegible]	[illegible]	[illegible]	[illegible]

Chr02	18111040	G	C	Chr02_18111	G_C	SNP	8805.63	14	5	9	10	9	8	21	10	13	10
Chr02	18529772	G	GT	Chr02_18529	G_GT	INDEL	16462.59	27	19	21	26	15	29	29	22	25	16
Chr02	18642769	T	A	Chr02_18642	T_A	SNP	20951.63	25	11	19	29	24	19	29	32	37	17
Chr02	18642772	T	G	Chr02_18642	T_G	SNP	20824.63	26	11	17	29	23	16	29	32	37	17
Chr02	18646256	G	GA	Chr02_18646	G_GA	INDEL	17888.59	36	14	27	24	19	19	29	29	26	26
Chr02	18646353	G	T	Chr02_18646	G_T	SNP	22858.63	37	11	20	28	27	24	39	30	31	30
Chr02	18646358	G	T	Chr02_18646	G_T	SNP	23004.63	38	10	20	25	27	22	39	30	31	29
Chr02	18646367	G	A	Chr02_18646	G_A	SNP	22759.63	40	11	18	26	27	22	39	29	29	30
Chr02	18728010	T	TTA	Chr02_18728	T_TTA	INDEL	8595.59	20	12	9	22	12	10	17	17	8	16
Chr02	19317768	G	T	Chr02_19317	G_T	SNP	17206.63	16	9	10	31	23	20	35	16	16	20
Chr02	19317791	G	T	Chr02_19317	G_T	SNP	17950.63	16	8	10	26	19	21	33	19	16	18
Chr02	19317801	G	T	Chr02_19317	G_T	SNP	18786.63	22	8	12	27	21	23	37	20	20	18
Chr02	19513452	A	AAT	Chr02_19513	A_AAT	INDEL	332.37	0	0	0	0	4	0	0	14	0	1
Chr02	19513452	A	AAT	Chr02_19513	A_AAT	INDEL	332.37	0	0	0	0	4	0	0	14	0	1
Chr02	19513452	A	AAT	Chr02_19513	A_AAT	INDEL	332.37	0	0	0	0	4	0	0	14	0	1
Chr02	19513452	A	AAT	Chr02_19513	A_AAT	INDEL	332.37	0	0	0	0	4	0	0	14	0	1
Chr02	19513452	A	AAT	Chr02_19513	A_AAT	INDEL	332.37	0	0	0	0	4	0	0	14	0	1
Chr02	19513452	A	AAT	Chr02_19513	A_AAT	INDEL	332.37	0	0	0	0	4	0	0	14	0	1
Chr02	19513452	A	AAT	Chr02_19513	A_AAT	INDEL	332.37	0	0	0	0	4	0	0	14	0	1
Chr02	19513452	A	AAT	Chr02_19513	A_AAT	INDEL	332.37	0	0	0	0	4	0	0	14	0	1
Chr02	19513452	A	AAT	Chr02_19513	A_AAT	INDEL	332.37	0	0	0	0	4	0	0	14	0	1
Chr02	19571992	CGCTGGA	C	Chr02_19571	CGCTGGA_C	INDEL	11383.59	18	9	10	12	10	15	19	16	13	9
Chr02	19571992	CGCTGGA	C	Chr02_19571	CGCTGGA_C	INDEL	11383.59	18	9	10	12	10	15	19	16	13	9
Chr02	19571992	CGCTGGA	C	Chr02_19571	CGCTGGA_C	INDEL	11383.59	18	9	10	12	10	15	19	16	13	9
Chr02	19571992	CGCTGGA	C	Chr02_19571	CGCTGGA_C	INDEL	11383.59	18	9	10	12	10	15	19	16	13	9
Chr02	19571992	CGCTGGA	C	Chr02_19571	CGCTGGA_C	INDEL	11383.59	18	9	10	12	10	15	19	16	13	9
Chr02	19571992	CGCTGGA	C	Chr02_19571	CGCTGGA_C	INDEL	11383.59	18	9	10	12	10	15	19	16	13	9
Chr02	19571992	CGCTGGA	C	Chr02_19571	CGCTGGA_C	INDEL	11383.59	18	9	10	12	10	15	19	16	13	9
Chr02	19571992	CGCTGGA	C	Chr02_19571	CGCTGGA_C	INDEL	11383.59	18	9	10	12	10	15	19	16	13	9
Chr02	19571992	CGCTGGA	C	Chr02_19571	CGCTGGA_C	INDEL	11383.59	18	9	10	12	10	15	19	16	13	9
Chr02	19571992	CGCTGGA	C	Chr02_19571	CGCTGGA_C	INDEL	11383.59	18	9	10	12	10	15	19	16	13	9
Chr02	19571992	CGCTGGA	C	Chr02_19571	CGCTGGA_C	INDEL	11383.59	18	9	10	12	10	15	19	16	13	9
Chr02	19572000	A	ATAATTT	Chr02_19572	A_ATAATTT	INDEL	13262.59	18	9	9	12	10	16	19	17	11	9
Chr02	19572000	A	ATAATTT	Chr02_19572	A_ATAATTT	INDEL	13262.59	18	9	9	12	10	16	19	17	11	9
Chr02	19572000	A	ATAATTT	Chr02_19572	A_ATAATTT	INDEL	13262.59	18	9	9	12	10	16	19	17	11	9
Chr02	19572000	A	ATAATTT	Chr02_19572	A_ATAATTT	INDEL	13262.59	18	9	9	12	10	16	19	17	11	9
Chr02	19572000	A	ATAATTT	Chr02_19572	A_ATAATTT	INDEL	13262.59	18	9	9	12	10	16	19	17	11	9
Chr02	19572000	A	ATAATTT	Chr02_19572	A_ATAATTT	INDEL	13262.59	18	9	9	12	10	16	19	17	11	9
Chr02	19572000	A	ATAATTT	Chr02_19572	A_ATAATTT	INDEL	13262.59	18	9	9	12	10	16	19	17	11	9
Chr02	19572000	A	ATAATTT	Chr02_19572	A_ATAATTT	INDEL	13262.59	18	9	9	12	10	16	19	17	11	9
Chr02	19572000	A	ATAATTT	Chr02_19572	A_ATAATTT	INDEL	13262.59	18	9	9	12	10	16	19	17	11	9
Chr02	19572000	A	ATAATTT	Chr02_19572	A_ATAATTT	INDEL	13262.59	18	9	9	12	10	16	19	17	11	9
Chr02	19572000	A	ATAATTT	Chr02_19572	A_ATAATTT	INDEL	13262.59	18	9	9	12	10	16	19	17	11	9
Chr02	19572001	G	GT	Chr02_19572	G_GT	INDEL	13262.59	20	10	10	13	11	16	24	17	13	10
Chr02	19572001	G	GT	Chr02_19572	G_GT	INDEL	13262.59	20	10	10	13	11	16	24	17	13	10
Chr02	19572001	G	GT	Chr02_19572	G_GT	INDEL	13262.59	20	10	10	13	11	16	24	17	13	10
Chr02	19572001	G	GT	Chr02_19572	G_GT	INDEL	13262.59	20	10	10	13	11	16	24	17	13	10
Chr02	19572001	G	GT	Chr02_19572	G_GT	INDEL	13262.59	20	10	10	13	11	16	24	17	13	10
Chr02	19572001	G	GT	Chr02_19572	G_GT	INDEL	13262.59	20	10	10	13	11	16	24	17	13	10
Chr02	19572001	G	GT	Chr02_19572	G_GT	INDEL	13262.59	20	10	10	13	11	16	24	17	13	10
Chr02	19572001	G	GT	Chr02_19572	G_GT	INDEL	13262.59	20	10	10	13	11	16	24	17	13	10
Chr02	19572001	G	GT	Chr02_19572	G_GT	INDEL	13262.59	20	10	10	13	11	16	24	17	13	10
Chr02	19572001	G	GT	Chr02_19572	G_GT	INDEL	13262.59	20	10	10	13	11	16	24	17	13	10
Chr02	19572001	G	GT	Chr02_19572	G_GT	INDEL	13262.59	20	10	10	13	11	16	24	17	13	10
Chr02	19620846	C	T	Chr02_19620	C_T	SNP	5722.29	0	0	0	19	16	31	28	27	0	0
Chr02	19620846	C	T	Chr02_19620	C_T	SNP	5722.29	0	0	0	19	16	31	28	27	0	0
Chr02	19620846	C	T	Chr02_19620	C_T	SNP	5722.29	0	0	0	19	16	31	28	27	0	0
Chr02	19620846	C	T	Chr02_19620	C_T	SNP	5722.29	0	0	0	19	16	31	28	27	0	0
Chr02	19620846	C	T	Chr02_19620	C_T	SNP	5722.29	0	0	0	19	16	31	28	27	0	0
Chr02	20047824	A	G	Chr02_20047	A_G	SNP	5397.29	0	0	0	21	24	21	28	21	0	0
Chr02	20047824	A	G	Chr02_20047	A_G	SNP	5397.29	0	0	0	21	24	21	28	21	0	0
Chr02	20047824	A	G	Chr02_20047	A_G	SNP	5397.29	0	0	0	21	24	21	28	21	0	0
Chr02	20047824	A	G	Chr02_20047	A_G	SNP	5397.29	0	0	0	21	24	21	28	21	0	0
Chr02	20067115	T	C	Chr02_20067	T_C	SNP	9245.29	27	17	11	0	0	1	0	0	6	13
Chr02	20067115	T	C	Chr02_20067	T_C	SNP	9245.29	27	17	11	0	0	1	0	0	6	13
Chr02	20067115	T	C	Chr02_20067	T_C	SNP	9245.29	27	17	11	0	0	1	0	0	6	13
Chr02	20067115	T	C	Chr02_20067	T_C	SNP	9245.29	27	17	11	0	0	1	0	0	6	13
Chr02	20244584	G	GTA	Chr02_20244	G_GTA	INDEL	253.37	1	8	0	0	0	0	0	0	0	0
Chr02	20244584	G	GTA	Chr02_20244	G_GTA	INDEL	253.37	1	8	0	0	0	0	0	0	0	0
Chr02	20244584	G	GTA	Chr02_20244	G_GTA	INDEL	253.37	1	8	0	0	0	0	0	0	0	0
Chr02	20260550	C	T	Chr02_20260	C_T	SNP	18197.63	34	12	16	25	19	27	31	22	25	19
Chr02	20279231	C	CA	Chr02_20279	C_CA	INDEL	14487.59	21	15	16	27	10	25	36	17	22	17
Chr02	20279231	C	CA	Chr02_20279	C_CA	INDEL	14487.59	21	15	16	27	10	25	36	17	22	17
Chr02	20279231	C	CA	Chr02_20279	C_CA	INDEL	14487.59	21	15	16	27	10	25	36	17	22	17
Chr02	20279231	C	CA	Chr02_20279	C_CA	INDEL	14487.59	21	15	16	27	10	25	36	17	22	17
Chr02	20279231	C	CA	Chr02_20279	C_CA	INDEL	14487.59	21	15	16	27	10	25	36	17	22	17
Chr02	20279231	C	CA	Chr02_20279	C_CA	INDEL	14487.59	21	15	16	27	10	25	36	17	22	17
Chr02	20279231	C	CA	Chr02_20279	C_CA	INDEL	14487.59	21	15	16	27	10	25	36	17	22	17
Chr02	20358809	C	T	Chr02_20358	C_T	SNP	4737.98	0	0	0	0	0	0	0	0	22	21
Chr02	20481875	T	TTA	Chr02_20481	T_TTA	INDEL	502.82	3	0	1	0	0	0	0	1	4	8
Chr02	20481875	T	TTA	Chr02_20481	T_TTA	INDEL	502.82	3	0	1	0	0	0	0	1	4	8
Chr02	20481875	T	TTA	Chr02_20481	T_TTA	INDEL	502.82	3	0	1	0	0	0	0	1	4	8
Chr02	20481875	T	TTA	Chr02_20481	T_TTA	INDEL	502.82	3	0	1	0	0	0	0	1	4	8
Chr02	20481875	T	TTA	Chr02_20481	T_TTA	INDEL	502.82	3	0	1	0	0	0	0	1	4	8
Chr02	20486277	G	GA	Chr02_20486	G_GA	INDEL	17046.59	27	23	18	24	20	19	32	18	21	18
Chr02	20486277	G	GA	Chr02_20486	G_GA	INDEL	17046.59	27	23	18	24	20	19	32	18	21	18
Chr02	20486277	G	GA	Chr02_20486	G_GA	INDEL	17046.59	27	23	18	24	20	19	32	18	21	18
Chr02	20486277	G	GA	Chr02_20486	G_GA	INDEL	17046.59	27	23	18	24	20	19	32	18	21	18
Chr02	20486277	G	GA	Chr02_20486	G_GA	INDEL	17046.59	27	23	18	24	20	19	32	18	21	18
Chr02	20524094	G	A	Chr02_20524	G_A	SNP	18682.63	32	16	13	23	16	22	27	24	23	26
Chr02	20524100	G	T	Chr02_20524	G_T	SNP	18952.63	33	16	12	22	17	20	27	22	19	24
Chr02	20524121	C	A	Chr02_20524	C_A	SNP	17008.63	30	15	12	17	18	19	22	21	19	24
Chr02	20728295	T	C	Chr02_20728	T_C	SNP	12080.63	23	9	7	15	12	16	13	8	13	24
Chr02	20728295	T	C	Chr02_20728	T_C	SNP	12080.63	23	9	7	15	12	16	13	8	13	24
Chr02	20728295	T	C	Chr02_20728	T_C	SNP	12080.63	23	9	7	15	12	16	13	8	13	24
Chr02	20728313	C	CA	Chr02_20728	C_CA	INDEL	14511.59	29	10	7	21	14	20	16	10	16	24
Chr02	20728313	C	CA	Chr02_20728	C_CA	INDEL	14511.59	29	10	7	21	14	20	16	10	16	24
Chr02	20728313	C	CA	Chr02_20728	C_CA	INDEL	14511.59	29	10	7	21	14	20	16	10	16	24
Chr02	20728331	C	CA	Chr02_20728	C_CA	INDEL	16677.59	31	11	8	24	18	26	20	12	15	26
Chr02	20728331	C	CA	Chr02_20728	C_CA	INDEL	16677.59	31	11	8	24	18	26	20	12	15	26
Chr02	20728331	C	CA	Chr02_20728	C_CA	INDEL	16677.59	31	11	8	24	18	26	20	12	15	26
Chr02	21002454	T	C	Chr02_21002	T_C	SNP	25322.63	26	18	17	25	28	40	45	28	24	29
Chr02	21002454	T	C	Chr02_21002	T_C	SNP	25322.63	26	18	17	25	28	40	45	28	24	29
Chr02	21002454	T	C	Chr02_21002	T_C	SNP	25322.63	26	18	17	25	28	40	45	28	24	29
Chr02	21002454	T	C	Chr02_21002	T_C	SNP	25322.63	26	18	17	25	28	40	45	28	24	29
Chr02	21002454	T	C	Chr02_21002	T_C	SNP	25322.63	26	18	17	25	28	40	45	28	24	29
Chr02	21002454	T	C	Chr02_21002	T_C	SNP	25322.63	26	18	17	25	28	40	45	28	24	29
Chr02	21002454	T	C	Chr02_21002	T_C	SNP	25322.63	26	18	17	25	28	40	45	28	24	29
Chr02	21002454	T	C	Chr02_21002	T_C	SNP	25322.63	26	18	17	25	28	40	45	28	24	29
Chr02	21002454	T	C	Chr02_21002	T_C	SNP	25322.63	26	18	17	25	28	40	45	28	24	29
Chr02	21002457	T	C	Chr02_21002	T_C	SNP	25327.63	25	18	17	24	30	42	45	27	24	27
Chr02	21002457	T	C	Chr02_21002	T_C	SNP	25327.63	25	18	17	24	30	42	45	27	24	27
Chr02	21002457	T	C	Chr02_21002	T_C	SNP	25327.63	25	18	17	24	30	42	45	27	24	27
Chr02	21002457	T	C	Chr02_21002	T_C	SNP	25327.63	25	18	17	24	30	42	45	27	24	27
Chr02	21002457	T	C	Chr02_21002	T_C	SNP	25327.63	25	18	17	24	30	42	45	27	24	27
Chr02	21002457	T	C	Chr02_21002	T_C	SNP	25327.63	25	18	17	24	30	42	45	27	24	27
Chr02	21002457	T	C	Chr02_21002	T_C	SNP	25327.63	25	18	17	24	30	42	45	27	24	27
Chr02	21002457	T	C	Chr02_21002	T_C	SNP	25327.63	25	18	17	24	30	42	45	27	24	27
Chr02	21002457	T	C	Chr02_21002	T_C	SNP	25327.63	25	18	17	24	30	42	45	27	24	27
Chr02	21002460	T	TA	Chr02_21002	T_TA	INDEL	25470.59	24	18	17	23	30	41	45	27	24	27
Chr02	21002460	T	TA	Chr02_21002	T_TA	INDEL	25470.59	24	18	17	23	30	41	45	27	24	27
Chr02	21002460	T	TA	Chr02_21002	T_TA	INDEL	25470.59	24	18	17	23	30	41	45	27	24	27
Chr02	21002460	T	TA	Chr02_21002	T_TA	INDEL	25470.59	24	18	17	23	30	41	45	27	24	27
Chr02	21002460	T	TA	Chr02_21002	T_TA	INDEL	25470.59	24	18	17	23	30	41	45	27	24	27
Chr02	21002460	T	TA	Chr02_21002	T_TA	INDEL	25470.59	24	18	17	23	30	41	45	27	24	27
Chr02	21002460	T	TA	Chr02_21002	T_TA	INDEL	25470.59	24	18	17	23	30	41	45	27	24	27
Chr02	21002460	T	TA	Chr02_21002	T_TA	INDEL	25470.59	24	18	17	23	30	41	45	27	24	27
Chr02	21002460	T	TA	Chr02_21002	T_TA	INDEL	25470.59	24	18	17	23	30	41	45	27	24	27
Chr02	21002463	C	A	Chr02_21002	C_A	SNP	25490.63	24	18	18	22	27	37	44	29	26	27
Chr02	21002463	C	A	Chr02_21002	C_A	SNP	25490.63	24	18	18	22	27	37	44	29	26	27
Chr02	21002463	C	A	Chr02_21002	C_A	SNP	25490.63	24	18	18	22	27	37	44	29	26	27
Chr02	21002463	C	A	Chr02_21002	C_A	SNP	25490.63	24	18	18	22	27	37	44	29	26	27
Chr02	21002463	C	A	Chr02_21002	C_A	SNP	25490.63	24	18	18	22	27	37	44	29	26	27
Chr02	21002463	C	A	Chr02_21002	C_A	SNP	25490.63	24	18	18	22	27	37	44	29	26	27
Chr02	21002463	C	A	Chr02_21002	C_A	SNP	25490.63	24	18	18	22	27	37	44	29	26	27
Chr02	21002463	C	A	Chr02_21002	C_A	SNP	25490.63	24	18	18	22	27	37	44	29	26	27
Chr02	21002463	C	A	Chr02_21002	C_A	SNP	25490.63	24	18	18	22	27	37	44	29	26	27
Chr02	21002470	A	C	Chr02_21002	A_C	SNP	25727.63	25	19	20	21	27	35	43	31	25	29
Chr02	21002470	A	C	Chr02_21002	A_C	SNP	25727.63	25	19	20	21	27	35	43	31	25	29
Chr02	21002470	A	C	Chr02_21002	A_C	SNP	25727.63	25	19	20	21	27	35	43	31	25	29
Chr02	21002470	A	C	Chr02_21002	A_C	SNP	25727.63	25	19	20	21	27	35	43	31	25	29
Chr02	21002470	A	C	Chr02_21002	A_C	SNP	25727.63	25	19	20	21	27	35	43	31	25	29
Chr02	21002470	A	C	Chr02_21002	A_C	SNP	25727.63	25	19	20	21	27	35	43	31	25	29
Chr02	21002470	A	C	Chr02_21002	A_C	SNP	25727.63	25	19	20	21	27	35	43	31	25	29
Chr02	21002470	A	C	Chr02_21002	A_C	SNP	25727.63	25	19	20	21	27	35	43	31	25	29
Chr02	21002470	A	C	Chr02_21002	A_C	SNP	25727.63	25	19	20	21	27	35	43	31	25	29
Chr02	21002477	A	AC	Chr02_21002	A_AC	INDEL	25618.59	27	18	21	21	23	38	40	32	23	27
Chr02	21002477	A	AC	Chr02_21002	A_AC	INDEL	25618.59	27	18	21	21	23	38	40	32	23	27
Chr02	21002477	A	AC	Chr02_21002	A_AC	INDEL	25618.59	27	18	21	21	23	38	40	32	23	27
Chr02	21002477	A	AC	Chr02_21002	A_AC	INDEL	25618.59	27	18	21	21	23	38	40	32	23	27
Chr02	21002477	A	AC	Chr02_21002	A_AC	INDEL	25618.59	27	18	21	21	23	38	40	32	23	27
Chr02	21002477	A	AC	Chr02_21002	A_AC	INDEL	25618.59	27	18	21	21	23	38	40	32	23	27
Chr02	21002477	A	AC	Chr02_21002	A_AC	INDEL	25618.59	27	18	21	21	23	38	40	32	23	27
Chr02	21002477	A	AC	Chr02_21002	A_AC	INDEL	25618.59	27	18	21	21	23	38	40	32	23	27
Chr02	21002477	A	AC	Chr02_21002	A_AC	INDEL	25618.59	27	18	21	21	23	38	40	32	23	27
Chr02	21005198	AGCGGG	A	Chr02_21005	AGCGGG_A	INDEL	11634.59	20	9	8	9	11	15	20	12	12	19
Chr02	21005198	AGCGGG	A	Chr02_21005	AGCGGG_A	INDEL	11634.59	20	9	8	9	11	15	20	12	12	19
Chr02	21005198	AGCGGG	A	Chr02_21005	AGCGGG_A	INDEL	11634.59	20	9	8	9	11	15	20	12	12	19
Chr02	21005198	AGCGGG	A	Chr02_21005	AGCGGG_A	INDEL	11634.59	20	9	8	9	11	15	20	12	12	19
Chr02	21005198	AGCGGG	A	Chr02_21005	AGCGGG_A	INDEL	11634.59	20	9	8	9	11	15	20	12	12	19
Chr02	21005198	AGCGGG	A	Chr02_21005	AGCGGG_A	INDEL	11634.59	20	9	8	9	11	15	20	12	12	19
Chr02	21005198	AGCGGG	A	Chr02_21005	AGCGGG_A	INDEL	11634.59	20	9	8	9	11	15	20	12	12	19
Chr02	21005198	AGCGGG	A	Chr02_21005	AGCGGG_A	INDEL	11634.59	20	9	8	9	11	15	20	12	12	19
Chr02	21005198	AGCGGG	A	Chr02_21005	AGCGGG_A	INDEL	11634.59	20	9	8	9	11	15	20	12	12	19
Chr02	21005203	G	GTACAAA	Chr02_21005	G_GTACAAA	INDEL	11634.59	20	9	8	9	11	15	20	12	12	19
Chr02	21005203	G	GTACAAA	Chr02_21005	G_GTACAAA	INDEL	11634.59	20	9	8	9	11	15	20	12	12	19
Chr02	21005203	G	GTACAAA	Chr02_21005	G_GTACAAA	INDEL	11634.59	20	9	8	9	11	15	20	12	12	19
Chr02	21005203	G	GTACAAA	Chr02_21005	G_GTACAAA	INDEL	11634.59	20	9	8	9	11	15	20	12	12	19
Chr02	21005203	G	GTACAAA	Chr02_21005	G_GTACAAA	INDEL	11634.59	20	9	8	9	11	15	20	12	12	19
Chr02	21005203	G	GTACAAA	Chr02_21005	G_GTACAAA	INDEL	11634.59	20	9	8	9	11	15	20	12	12	19
Chr02	21005203	G	GTACAAA	Chr02_21005	G_GTACAAA	INDEL	11634.59	20	9	8	9	11	15	20	12	12	19
Chr02	21005203	G	GTACAAA	Chr02_21005	G_GTACAAA	INDEL	11634.59	20	9	8	9	11	15	20	12	12	19
Chr02	21005203	G	GTACAAA	Chr02_21005	G_GTACAAA	INDEL	11634.59	20	9	8	9	11	15	20	12	12	19
Chr02	21005275	A	G	Chr02_21005	A_G	SNP	11494.63	18	9	12	14	10	10	17	13	13	16
Chr02	21005275	A	G	Chr02_21005	A_G	SNP	11494.63	18	9	12	14	10	10	17	13	13	16
Chr02	21005275	A	G	Chr02_21005	A_G	SNP	11494.63	18	9	12	14	10	10	17	13	13	16
Chr02	21005275	A	G	Chr02_21005	A_G	SNP	11494.63	18	9	12	14	10	10	17	13	13	16
Chr02	21005275	A	G	Chr02_21005	A_G	SNP	11494.63	18	9	12	14	10	10	17	13	13	16
Chr02	21005275	A	G	Chr02_21005	A_G	SNP	11494.63	18	9	12	14	10	10	17	13	13	16
Chr02	21005275	A	G	Chr02_21005	A_G	SNP	11494.63	18	9	12	14	10	10	17	13	13	16
Chr02	21005275	A	G	Chr02_21005	A_G	SNP	11494.63	18	9	12	14	10	10	17	13	13	16
Chr02	21005275	A	G	Chr02_21005	A_G	SNP	11494.63	18	9	12	14	10	10	17	13	13	16
Chr02	21005311	G	GA	Chr02_21005	G_GA	INDEL	12569.59	26	13	17	14	13	14	20	18	18	23
Chr02	21005311	G	GA	Chr02_21005	G_GA	INDEL	12569.59	26	13	17	14	13	14	20	18	18	23
Chr02	21005311	G	GA	Chr02_21005	G_GA	INDEL	12569.59	26	13	17	14	13	14	20	18	18	23
Chr02	21005311	G	GA	Chr02_21005	G_GA	INDEL	12569.59	26	13	17	14	13	14	20	18	18	23
Chr02	21005311	G	GA	Chr02_21005	G_GA	INDEL	12569.59	26	13	17	14	13	14	20	18	18	23
Chr02	21005311	G	GA	Chr02_21005	G_GA	INDEL	12569.59	26	13	17	14	13	14	20	18	18	23
Chr02	21005311	G	GA	Chr02_21005	G_GA	INDEL	12569.59	26	13	17	14	13	14	20	18	18	23
Chr02	21005311	G	GA	Chr02_21005	G_GA	INDEL	12569.59	26	13	17	14	13	14	20	18	18	23
Chr02	21005311	G	GA	Chr02_21005	G_GA	INDEL	12569.59	26	13	17	14	13	14	20	18	18	23
Chr02	21040592	G	T	Chr02_21040	G_T	SNP	6046.63	11	6	5	6	7	10	10	13	5	18
Chr02	21040592	G	T	Chr02_21040	G_T	SNP	6046.63	11	6	5	6	7	10	10	13	5	18
Chr02	21040592	G	T	Chr02_21040	G_T	SNP	6046.63	11	6	5	6	7	10	10	13	5	18
Chr02	21040595	A	ATTT	Chr02_21040	A_ATTT	INDEL	6903.59	10	5	4	5	6	10	9	13	5	14
Chr02	21040595	A	ATTT	Chr02_21040	A_ATTT	INDEL	6903.59	10	5	4	5	6	10	9	13	5	14
Chr02	21040595	A	ATTT	Chr02_21040	A_ATTT	INDEL	6903.59	10	5	4	5	6	10	9	13	5	14
Chr02	21087922	G	T	Chr02_21087	G_T	SNP	11042.63	16	6	7	19	7	16	10	16	10	7
Chr02	21092116	TA	T	Chr02_21092	TA_T	INDEL	16627.59	33	18	19	33	12	31	32	26	6	26
Chr02	21092116	TA	T	Chr02_21092	TA_T	INDEL	16627.59	33	18	19	33	12	31	32	26	6	26
Chr02	21092116	TA	T	Chr02_21092	TA_T	INDEL	16627.59	33	18	19	33	12	31	32	26	6	26
Chr02	21092116	TA	T	Chr02_21092	TA_T	INDEL	16627.59	33	18	19	33	12	31	32	26	6	26

Chr02	21112151	A	T	Chr02_21112	A_T	SNP	12844.63	30	17	14	[illegible]
Chr02	21112151	A	T	Chr02_21112	A_T	SNP	12844.63	30	17	14	[illegible]
Chr02	21112151	A	T	Chr02_21112	A_T	SNP	12844.63	30	17	14	[illegible]
Chr02	21115016	A	C	Chr02_21115	A_C	SNP	12974.63	16	11	17	[illegible]
Chr02	21115016	A	C	Chr02_21115	A_C	SNP	12974.63	16	11	17	[illegible]
Chr02	21115097	A	AC	Chr02_21115	A_AC	INDEL	14328.59	23	18	15	[illegible]
Chr02	21115097	A	AC	Chr02_21115	A_AC	INDEL	14328.59	23	18	15	[illegible]
Chr02	21117849	G	T	Chr02_21117	G_T	SNP	11223.63	16	4	10	[illegible]
Chr02	21117849	G	T	Chr02_21117	G_T	SNP	11223.63	16	4	10	[illegible]
Chr02	21117849	G	T	Chr02_21117	G_T	SNP	11223.63	16	4	10	[illegible]
Chr02	21151496	G	GAT	Chr02_21151	G_GAT	INDEL	2259.93	1	0	0	[illegible]
Chr02	21151496	G	GAT	Chr02_21151	G_GAT	INDEL	2259.93	1	0	0	[illegible]
Chr02	21151496	G	GAT	Chr02_21151	G_GAT	INDEL	2259.93	1	0	0	[illegible]
Chr02	21151496	G	GAT	Chr02_21151	G_GAT	INDEL	2259.93	1	0	0	[illegible]
Chr02	21151496	G	GAT	Chr02_21151	G_GAT	INDEL	2259.93	1	0	0	[illegible]
Chr02	21151496	G	GAT	Chr02_21151	G_GAT	INDEL	2259.93	1	0	0	[illegible]
Chr02	21151496	G	GAT	Chr02_21151	G_GAT	INDEL	2259.93	1	0	0	[illegible]
Chr02	21151496	G	GAT	Chr02_21151	G_GAT	INDEL	2259.93	1	0	0	[illegible]
Chr02	21151496	G	GAT	Chr02_21151	G_GAT	INDEL	2259.93	1	0	0	[illegible]
Chr02	21328895	T	TCC	Chr02_21328	T_TCC	INDEL	8147.59	7	5	9	[illegible]
Chr02	21328897	A	ATAG	Chr02_21328	A_ATAG	INDEL	8507.59	8	6	9	[illegible]
Chr02	21328899	A	ACGTCACCA(	Chr02_21328	A_ACGTCAC(	INDEL	9101.59	8	5	9	[illegible]
Chr02	21381387	A	G	Chr02_21381	A_G	SNP	34319.63	51	34	26	[illegible]
Chr02	21432563	A	AT	Chr02_21432	A_AT	INDEL	79555.59	116	60	84	[illegible]
Chr02	21432563	A	AT	Chr02_21432	A_AT	INDEL	79555.59	116	60	84	[illegible]
Chr02	21432563	A	AT	Chr02_21432	A_AT	INDEL	79555.59	116	60	84	[illegible]
Chr02	21432563	A	AT	Chr02_21432	A_AT	INDEL	79555.59	116	60	84	[illegible]
Chr02	21432563	A	AT	Chr02_21432	A_AT	INDEL	79555.59	116	60	84	[illegible]
Chr02	21432563	A	AT	Chr02_21432	A_AT	INDEL	79555.59	116	60	84	[illegible]
Chr02	21432563	A	AT	Chr02_21432	A_AT	INDEL	79555.59	116	60	84	[illegible]
Chr02	21558747	T	C	Chr02_21558	T_C	SNP	15174.63	19	12	16	[illegible]
Chr02	21616227	A	AT	Chr02_21616	A_AT	INDEL	13846.59	31	8	16	[illegible]
Chr02	21765072	AAATTCTAA/	A	Chr02_21765	AAATTCTAA/	INDEL	14045.59	13	6	8	[illegible]
Chr02	21765072	AAATTCTAA/	A	Chr02_21765	AAATTCTAA/	INDEL	14045.59	13	6	8	[illegible]
Chr02	21765072	AAATTCTAA/	A	Chr02_21765	AAATTCTAA/	INDEL	14045.59	13	6	8	[illegible]
Chr02	21765072	AAATTCTAA/	A	Chr02_21765	AAATTCTAA/	INDEL	14045.59	13	6	8	[illegible]
Chr02	21765072	AAATTCTAA/	A	Chr02_21765	AAATTCTAA/	INDEL	14045.59	13	6	8	[illegible]
Chr02	21964672	T	C	Chr02_21964	T_C	SNP	8956.63	15	12	8	[illegible]
Chr02	22019980	A	T	Chr02_22019	A_T	SNP	13403.63	18	11	14	[illegible]
Chr02	22019980	A	T	Chr02_22019	A_T	SNP	13403.63	18	11	14	[illegible]
Chr02	22020033	A	T	Chr02_22020	A_T	SNP	16171.63	21	16	17	[illegible]
Chr02	22020033	A	T	Chr02_22020	A_T	SNP	16171.63	21	16	17	[illegible]
Chr02	22020035	G	A	Chr02_22020	G_A	SNP	16171.63	21	16	17	[illegible]
Chr02	22020035	G	A	Chr02_22020	G_A	SNP	16171.63	21	16	17	[illegible]
Chr02	22339782	T	TA	Chr02_22339	T_TA	INDEL	14590.59	32	12	18	[illegible]
Chr02	22397406	G	A	Chr02_22397	G_A	SNP	9489.29	21	12	10	[illegible]
Chr02	22397409	T	C	Chr02_22397	T_C	SNP	9554.29	21	12	10	[illegible]
Chr02	22569472	T	TTA	Chr02_22569	T_TTA	INDEL	10188.59	14	8	14	[illegible]
Chr02	22569472	T	TTA	Chr02_22569	T_TTA	INDEL	10188.59	14	8	14	[illegible]
Chr02	22569472	T	TTA	Chr02_22569	T_TTA	INDEL	10188.59	14	8	14	[illegible]
Chr02	22569472	T	TTA	Chr02_22569	T_TTA	INDEL	10188.59	14	8	14	[illegible]
Chr02	22569472	T	TTA	Chr02_22569	T_TTA	INDEL	10188.59	14	8	14	[illegible]
Chr02	22569472	T	TTA	Chr02_22569	T_TTA	INDEL	10188.59	14	8	14	[illegible]
Chr02	22569472	T	TTA	Chr02_22569	T_TTA	INDEL	10188.59	14	8	14	[illegible]
Chr02	22569472	T	TTA	Chr02_22569	T_TTA	INDEL	10188.59	14	8	14	[illegible]
Chr02	22649770	G	GAT	Chr02_22649	G_GAT	INDEL	1809.93	1	1	0	[illegible]
Chr02	22679021	G	GA	Chr02_22679	G_GA	INDEL	7962.59	8	10	9	[illegible]
Chr02	22685246	A	AAT	Chr02_22685	A_AAT	INDEL	2730.24	1	0	0	[illegible]
Chr02	22685246	A	AAT	Chr02_22685	A_AAT	INDEL	2730.24	1	0	0	[illegible]
Chr02	22685246	A	AAT	Chr02_22685	A_AAT	INDEL	2730.24	1	0	0	[illegible]
Chr02	22685246	A	AAT	Chr02_22685	A_AAT	INDEL	2730.24	1	0	0	[illegible]
Chr02	22685246	A	AAT	Chr02_22685	A_AAT	INDEL	2730.24	1	0	0	[illegible]
Chr02	22685246	A	AAT	Chr02_22685	A_AAT	INDEL	2730.24	1	0	0	[illegible]
Chr02	22685246	A	AAT	Chr02_22685	A_AAT	INDEL	2730.24	1	0	0	[illegible]
Chr02	22685246	A	AAT	Chr02_22685	A_AAT	INDEL	2730.24	1	0	0	[illegible]
Chr02	22685246	A	AAT	Chr02_22685	A_AAT	INDEL	2730.24	1	0	0	[illegible]
Chr02	22737455	A	AT	Chr02_22737	A_AT	INDEL	10846.59	14	7	8	[illegible]
Chr02	22737700	C	A	Chr02_22737	C_A	SNP	15040.63	23	11	12	[illegible]
Chr02	22737703	T	A	Chr02_22737	T_A	SNP	15132.63	23	12	12	[illegible]
Chr02	22737951	C	A	Chr02_22737	C_A	SNP	9882.63	8	12	9	[illegible]
Chr02	22761034	G	A	Chr02_22761	G_A	SNP	12738.63	19	5	6	[illegible]
Chr02	22765036	C	A	Chr02_22765	C_A	SNP	21667.63	27	26	19	[illegible]
Chr02	22768913	C	A	Chr02_22768	C_A	SNP	17513.63	29	16	20	[illegible]
Chr02	22768913	C	A	Chr02_22768	C_A	SNP	17513.63	29	16	20	[illegible]
Chr02	22768913	C	A	Chr02_22768	C_A	SNP	17513.63	29	16	20	[illegible]
Chr02	22827348	G	C	Chr02_22827	G_C	SNP	18272.63	27	14	10	[illegible]
Chr02	23048301	CAT	C	Chr02_23048	CAT_C	INDEL	1259.35	23	11	10	[illegible]
Chr02	23048301	CAT	C	Chr02_23048	CAT_C	INDEL	1259.35	23	11	10	[illegible]
Chr02	23048301	CAT	C	Chr02_23048	CAT_C	INDEL	1259.35	23	11	10	[illegible]
Chr02	23065090	A	C	Chr02_23065	A_C	SNP	16616.63	22	11	9	[illegible]
Chr02	23071298	C	T	Chr02_23071	C_T	SNP	1841.39	22	10	14	[illegible]
Chr02	23226447	T	TG	Chr02_23226	T_TG	INDEL	11768.59	28	11	9	[illegible]
Chr02	23277430	G	A	Chr02_23277	G_A	SNP	17402.63	31	16	13	[illegible]
Chr02	23277436	T	TA	Chr02_23277	T_TA	INDEL	17354.59	30	13	12	[illegible]
Chr02	23277448	G	A	Chr02_23277	G_A	SNP	16214.63	29	11	12	[illegible]
Chr02	23284639	T	A	Chr02_23284	T_A	SNP	21239.63	27	18	18	[illegible]
Chr02	23284648	T	A	Chr02_23284	T_A	SNP	21353.63	25	16	18	[illegible]
Chr02	23372325	C	T	Chr02_23372	C_T	SNP	14096.63	24	11	14	[illegible]
Chr02	23674248	A	AAGAG	Chr02_23674	A_AAGAG	INDEL	18812.59	34	17	11	[illegible]
Chr02	23674248	A	AAGAG	Chr02_23674	A_AAGAG	INDEL	18812.59	34	17	11	[illegible]
Chr02	23674248	A	AAGAG	Chr02_23674	A_AAGAG	INDEL	18812.59	34	17	11	[illegible]
Chr02	23820260	T	TTA	Chr02_23820	T_TTA	INDEL	312.37	0	0	0	[illegible]
Chr02	23982474	G	T	Chr02_23982	G_T	SNP	12931.63	26	7	17	[illegible]
Chr02	23984633	CT	C	Chr02_23984	CT_C	INDEL	9176.59	9	11	4	[illegible]
Chr02	23984638	T	TA	Chr02_23984	T_TA	INDEL	9851.59	10	11	5	[illegible]
Chr02	23984641	TC	T	Chr02_23984	TC_T	INDEL	9649.59	11	9	5	[illegible]
Chr02	23984647	G	GA	Chr02_23984	G_GA	INDEL	9155.59	10	8	5	[illegible]
Chr02	24015889	A	G	Chr02_24015	A_G	SNP	26111.63	35	19	31	[illegible]
Chr02	24015889	A	G	Chr02_24015	A_G	SNP	26111.63	35	19	31	[illegible]
Chr02	24015889	A	G	Chr02_24015	A_G	SNP	26111.63	35	19	31	[illegible]
Chr02	24015889	A	G	Chr02_24015	A_G	SNP	26111.63	35	19	31	[illegible]
Chr02	24091125	C	A	Chr02_24091	C_A	SNP	11914.63	21	11	8	[illegible]
Chr02	24091132	C	T	Chr02_24091	C_T	SNP	11278.63	18	10	8	[illegible]
Chr02	24109721	T	TTA	Chr02_24109	T_TTA	INDEL	4724.24	14	8	6	[illegible]
Chr02	24110143	C	T	Chr02_24110	C_T	SNP	5176.29	0	0	0	[illegible]
Chr02	24497991	C	G	Chr02_24497	C_G	SNP	12511.29	31	14	15	[illegible]
Chr02	24516676	A	G	Chr02_24516	A_G	SNP	7680.63	21	10	16	[illegible]
Chr02	24516676	A	G	Chr02_24516	A_G	SNP	7680.63	21	10	16	[illegible]
Chr02	24516676	A	G	Chr02_24516	A_G	SNP	7680.63	21	10	16	[illegible]
Chr02	24533170	T	C	Chr02_24533	T_C	SNP	30970.63	63	25	27	[illegible]
Chr02	24533170	T	C	Chr02_24533	T_C	SNP	30970.63	63	25	27	[illegible]
Chr02	24533170	T	C	Chr02_24533	T_C	SNP	30970.63	63	25	27	[illegible]
Chr02	24533170	T	C	Chr02_24533	T_C	SNP	30970.63	63	25	27	[illegible]
Chr02	24533201	C	A	Chr02_24533	C_A	SNP	28767.63	61	24	31	[illegible]
Chr02	24533201	C	A	Chr02_24533	C_A	SNP	28767.63	61	24	31	[illegible]
Chr02	24533201	C	A	Chr02_24533	C_A	SNP	28767.63	61	24	31	[illegible]
Chr02	24533201	C	A	Chr02_24533	C_A	SNP	28767.63	61	24	31	[illegible]
Chr02	24537551	C	T	Chr02_24537	C_T	SNP	11698.63	22	8	21	[illegible]
Chr02	24537551	C	T	Chr02_24537	C_T	SNP	11698.63	22	8	21	[illegible]
Chr02	24537551	C	T	Chr02_24537	C_T	SNP	11698.63	22	8	21	[illegible]
Chr02	24537582	C	T	Chr02_24537	C_T	SNP	15644.63	28	7	24	[illegible]
Chr02	24537582	C	T	Chr02_24537	C_T	SNP	15644.63	28	7	24	[illegible]
Chr02	24537582	C	T	Chr02_24537	C_T	SNP	15644.63	28	7	24	[illegible]
Chr02	24537584	G	T	Chr02_24537	G_T	SNP	15686.63	28	7	26	[illegible]
Chr02	24537584	G	T	Chr02_24537	G_T	SNP	15686.63	28	7	26	[illegible]
Chr02	24537584	G	T	Chr02_24537	G_T	SNP	15686.63	28	7	26	[illegible]
Chr02	24537591	G	A	Chr02_24537	G_A	SNP	15761.63	27	7	26	[illegible]
Chr02	24537591	G	A	Chr02_24537	G_A	SNP	15761.63	27	7	26	[illegible]
Chr02	24537591	G	A	Chr02_24537	G_A	SNP	15761.63	27	7	26	[illegible]
Chr02	24610259	G	GT	Chr02_24610	G_GT	INDEL	14528.59	34	16	11	[illegible]
Chr02	24610259	G	GT	Chr02_24610	G_GT	INDEL	14528.59	34	16	11	[illegible]
Chr02	24610259	G	GT	Chr02_24610	G_GT	INDEL	14528.59	34	16	11	[illegible]
Chr02	24641996	A	ATATATATAT	Chr02_24641	A_ATATATAT	INDEL	10413.59	18	5	9	[illegible]
Chr02	24641996	A	ATATATATAT	Chr02_24641	A_ATATATAT	INDEL	10413.59	18	5	9	[illegible]
Chr02	24641996	A	ATATATATAT	Chr02_24641	A_ATATATAT	INDEL	10413.59	18	5	9	[illegible]
Chr02	24641996	A	ATATATATAT	Chr02_24641	A_ATATATAT	INDEL	10413.59	18	5	9	[illegible]
Chr02	24641996	A	ATATATATAT	Chr02_24641	A_ATATATAT	INDEL	10413.59	18	5	9	[illegible]
Chr02	25070855	A	T	Chr02_25070	A_T	SNP	19420.63	23	15	14	[illegible]
Chr02	25181190	A	AAT	Chr02_25181	A_AAT	INDEL	11660.59	25	9	16	[illegible]
Chr02	25181190	A	AAT	Chr02_25181	A_AAT	INDEL	11660.59	25	9	16	[illegible]
Chr02	25181190	A	AAT	Chr02_25181	A_AAT	INDEL	11660.59	25	9	16	[illegible]
Chr02	25239925	GTA	G	Chr02_25239	GTA_G	INDEL	5904.59	22	7	8	[illegible]
Chr02	25239925	GTA	G	Chr02_25239	GTA_G	INDEL	5904.59	22	7	8	[illegible]
Chr02	25239925	GTA	G	Chr02_25239	GTA_G	INDEL	5904.59	22	7	8	[illegible]
Chr02	25239925	GTA	G	Chr02_25239	GTA_G	INDEL	5904.59	22	7	8	[illegible]
Chr02	25239925	GTA	G	Chr02_25239	GTA_G	INDEL	5904.59	22	7	8	[illegible]
Chr02	25239925	GTA	G	Chr02_25239	GTA_G	INDEL	5904.59	22	7	8	[illegible]
Chr02	25239925	GTA	G	Chr02_25239	GTA_G	INDEL	5904.59	22	7	8	[illegible]
Chr02	25239925	GTA	G	Chr02_25239	GTA_G	INDEL	5904.59	22	7	8	[illegible]
Chr02	25239925	GTA	G	Chr02_25239	GTA_G	INDEL	5904.59	22	7	8	[illegible]
Chr02	25239925	GTA	G	Chr02_25239	GTA_G	INDEL	5904.59	22	7	8	[illegible]
Chr02	25239925	GTA	G	Chr02_25239	GTA_G	INDEL	5904.59	22	7	8	[illegible]
Chr02	25239925	GTA	G	Chr02_25239	GTA_G	INDEL	5904.59	22	7	8	[illegible]
Chr02	25239925	GTA	G	Chr02_25239	GTA_G	INDEL	5904.59	22	7	8	[illegible]
Chr02	25239925	GTA	G	Chr02_25239	GTA_G	INDEL	5904.59	22	7	8	[illegible]
Chr02	25239925	GTA	G	Chr02_25239	GTA_G	INDEL	5904.59	22	7	8	[illegible]
Chr02	25239925	GTA	G	Chr02_25239	GTA_G	INDEL	5904.59	22	7	8	[illegible]
Chr02	25239925	GTA	G	Chr02_25239	GTA_G	INDEL	5904.59	22	7	8	[illegible]
Chr02	25239925	GTA	G	Chr02_25239	GTA_G	INDEL	5904.59	22	7	8	[illegible]
Chr02	25239925	GTA	G	Chr02_25239	GTA_G	INDEL	5904.59	22	7	8	[illegible]
Chr02	25352803	G	GA	Chr02_25352	G_GA	INDEL	14739.59	29	12	12	[illegible]
Chr02	25517496	G	GA	Chr02_25517	G_GA	INDEL	16947.59	23	13	16	[illegible]
Chr02	25622021	G	A	Chr02_25622	G_A	SNP	17426.63	26	9	14	[illegible]
Chr02	25622022	G	A	Chr02_25622	G_A	SNP	17426.63	27	8	15	[illegible]
Chr02	25950238	A	AAT	Chr02_25950	A_AAT	INDEL	8041.59	25	9	10	[illegible]
Chr02	25951567	C	T	Chr02_25951	C_T	SNP	2199.39	23	12	16	[illegible]
Chr03	283085	C	T	Chr03_28308	C_T	SNP	11470.63	15	9	6	[illegible]
Chr03	283091	G	A	Chr03_28309	G_A	SNP	11866.63	18	11	7	[illegible]
Chr03	283117	G	C	Chr03_28311	G_C	SNP	13343.63	21	14	8	[illegible]
Chr03	414994	T	A	Chr03_41499	T_A	SNP	18398.63	33	21	15	[illegible]
Chr03	414994	T	A	Chr03_41499	T_A	SNP	18398.63	33	21	15	[illegible]
Chr03	414994	T	A	Chr03_41499	T_A	SNP	18398.63	33	21	15	[illegible]
Chr03	415059	C	CAATTTAACT	Chr03_41505	C_CAATTTAA	INDEL	11349.59	20	10	9	[illegible]
Chr03	415059	C	CAATTTAACT	Chr03_41505	C_CAATTTAA	INDEL	11349.59	20	10	9	[illegible]
Chr03	415059	C	CAATTTAACT	Chr03_41505	C_CAATTTAA	INDEL	11349.59	20	10	9	[illegible]
Chr03	573911	A	AT	Chr03_57391	A_AT	INDEL	12411.59	26	13	15	[illegible]
Chr03	600754	T	TTA	Chr03_60075	T_TTA	INDEL	277.37	1	1	0	[illegible]
Chr03	600754	T	TTA	Chr03_60075	T_TTA	INDEL	277.37	1	1	0	[illegible]
Chr03	697537	C	CAT	Chr03_69753	C_CAT	INDEL	4599.5	2	0	0	[illegible]
Chr03	697537	C	CAT	Chr03_69753	C_CAT	INDEL	4599.5	2	0	0	[illegible]
Chr03	697537	C	CAT	Chr03_69753	C_CAT	INDEL	4599.5	2	0	0	[illegible]
Chr03	697537	C	CAT	Chr03_69753	C_CAT	INDEL	4599.5	2	0	0	[illegible]
Chr03	785862	A	AAT	Chr03_78586	A_AAT	INDEL	2702.24	0	0	1	[illegible]
Chr03	785862	A	AAT	Chr03_78586	A_AAT	INDEL	2702.24	0	0	1	[illegible]
Chr03	785862	A	AAT	Chr03_78586	A_AAT	INDEL	2702.24	0	0	1	[illegible]
Chr03	785862	A	AAT	Chr03_78586	A_AAT	INDEL	2702.24	0	0	1	[illegible]
Chr03	785862	A	AAT	Chr03_78586	A_AAT	INDEL	2702.24	0	0	1	[illegible]
Chr03	785862	A	AAT	Chr03_78586	A_AAT	INDEL	2702.24	0	0	1	[illegible]
Chr03	1168184	A	ATGT	Chr03_11681	A_ATGT	INDEL	21041.59	31	17	19	[illegible]
Chr03	1215316	G	A	Chr03_12153	G_A	SNP	17705.63	24	13	17	[illegible]
Chr03	1215316	G	A	Chr03_12153	G_A	SNP	17705.63	24	13	17	[illegible]
Chr03	1267990	TTA	T	Chr03_12679	TTA_T	INDEL	1249.35	18	8	15	[illegible]
Chr03	1267990	TTA	T	Chr03_12679	TTA_T	INDEL	1249.35	18	8	15	[illegible]
Chr03	1267990	TTA	T	Chr03_12679	TTA_T	INDEL	1249.35	18	8	15	[illegible]
Chr03	1267990	TTA	T	Chr03_12679	TTA_T	INDEL	1249.35	18	8	15	[illegible]
Chr03	1267990	TTA	T	Chr03_12679	TTA_T	INDEL	1249.35	18	8	15	[illegible]
Chr03	1267990	TTA	T	Chr03_12679	TTA_T	INDEL	1249.35	18	8	15	[illegible]
Chr03	1267990	TTA	T	Chr03_12679	TTA_T	INDEL	1249.35	18	8	15	[illegible]
Chr03	1267990	TTA	T	Chr03_12679	TTA_T	INDEL	1249.35	18	8	15	[illegible]
Chr03	1267990	TTA	T	Chr03_12679	TTA_T	INDEL	1249.35	18	8	15	[illegible]
Chr03	1569690	G	T	Chr03_15696	G_T	SNP	14623.63	18	16	7	[illegible]
Chr03	1569690	G	T	Chr03_15696	G_T	SNP	14623.63	18	16	7	[illegible]

Chr03	1569690	G	T	Chr03_15696	G_T	SNP	14623.63	18	16	[illegible]
Chr03	1569690	G	T	Chr03_15696	G_T	SNP	14623.63	18	16	[illegible]
Chr03	1569690	G	T	Chr03_15696	G_T	SNP	14623.63	18	16	[illegible]
Chr03	1569690	G	T	Chr03_15696	G_T	SNP	14623.63	18	16	[illegible]
Chr03	1569690	G	T	Chr03_15696	G_T	SNP	14623.63	18	16	[illegible]
Chr03	1820572	A	G	Chr03_18205	A_G	SNP	22017.63	43	13	[illegible]
Chr03	1820572	A	G	Chr03_18205	A_G	SNP	22017.63	43	13	[illegible]
Chr03	1820572	A	G	Chr03_18205	A_G	SNP	22017.63	43	13	[illegible]
Chr03	1820572	A	G	Chr03_18205	A_G	SNP	22017.63	43	13	[illegible]
Chr03	1954697	C	T	Chr03_19546	C_T	SNP	13824.63	25	12	[illegible]
Chr03	1954700	C	T	Chr03_19547	C_T	SNP	13833.63	27	13	[illegible]
Chr03	2003655	G	GT	Chr03_20036	G_GT	INDEL	6837.59	19	11	[illegible]
Chr03	2003655	G	GT	Chr03_20036	G_GT	INDEL	6837.59	19	11	[illegible]
Chr03	2003655	G	GT	Chr03_20036	G_GT	INDEL	6837.59	19	11	[illegible]
Chr03	2003655	G	GT	Chr03_20036	G_GT	INDEL	6837.59	19	11	[illegible]
Chr03	2003655	G	GT	Chr03_20036	G_GT	INDEL	6837.59	19	11	[illegible]
Chr03	2003655	G	GT	Chr03_20036	G_GT	INDEL	6837.59	19	11	[illegible]
Chr03	2027274	CA	C	Chr03_20272	CA_C	INDEL	15072.59	27	22	[illegible]
Chr03	2027274	CA	C	Chr03_20272	CA_C	INDEL	15072.59	27	22	[illegible]
Chr03	2027274	CA	C	Chr03_20272	CA_C	INDEL	15072.59	27	22	[illegible]
Chr03	2027274	CA	C	Chr03_20272	CA_C	INDEL	15072.59	27	22	[illegible]
Chr03	2027274	CA	C	Chr03_20272	CA_C	INDEL	15072.59	27	22	[illegible]
Chr03	2027274	CA	C	Chr03_20272	CA_C	INDEL	15072.59	27	22	[illegible]
Chr03	2027274	CA	C	Chr03_20272	CA_C	INDEL	15072.59	27	22	[illegible]
Chr03	2580959	C	CAT	Chr03_25809	C_CAT	INDEL	3077.11	0	0	[illegible]
Chr03	2580959	C	CAT	Chr03_25809	C_CAT	INDEL	3077.11	0	0	[illegible]
Chr03	2580959	C	CAT	Chr03_25809	C_CAT	INDEL	3077.11	0	0	[illegible]
Chr03	2580959	C	CAT	Chr03_25809	C_CAT	INDEL	3077.11	0	0	[illegible]
Chr03	2580959	C	CAT	Chr03_25809	C_CAT	INDEL	3077.11	0	0	[illegible]
Chr03	2580959	C	CATATATATA	Chr03_25809	C_CATATATA	INDEL	3077.11	8	4	[illegible]
Chr03	2580959	C	CATATATATA	Chr03_25809	C_CATATATA	INDEL	3077.11	8	4	[illegible]
Chr03	2580959	C	CATATATATA	Chr03_25809	C_CATATATA	INDEL	3077.11	8	4	[illegible]
Chr03	2580959	C	CATATATATA	Chr03_25809	C_CATATATA	INDEL	3077.11	8	4	[illegible]
Chr03	2580959	C	CATATATATA	Chr03_25809	C_CATATATA	INDEL	3077.11	8	4	[illegible]
Chr03	2614518	GC	G	Chr03_26145	GC_G	INDEL	15671.59	21	8	[illegible]
Chr03	2642984	AAT	A	Chr03_26429	AAT_A	INDEL	1717.93	0	0	[illegible]
Chr03	2856594	G	T	Chr03_28565	G_T	SNP	16329.63	28	14	[illegible]
Chr03	2877635	C	G	Chr03_28776	C_G	SNP	2068.39	23	13	[illegible]
Chr03	2907758	G	GA	Chr03_29077	G_GA	INDEL	16113.59	28	11	[illegible]
Chr03	2964597	T	C	Chr03_29645	T_C	SNP	15887.63	16	13	[illegible]
Chr03	2964605	C	T	Chr03_29646	C_T	SNP	15497.63	16	13	[illegible]
Chr03	2967317	T	C	Chr03_29673	T_C	SNP	14254.63	22	10	[illegible]
Chr03	2967405	G	A	Chr03_29674	G_A	SNP	16613.63	21	9	[illegible]
Chr03	2967406	T	C	Chr03_29674	T_C	SNP	16613.63	21	8	[illegible]
Chr03	2967678	G	A	Chr03_29676	G_A	SNP	15754.63	33	16	[illegible]
Chr03	2967690	A	C	Chr03_29676	A_C	SNP	17038.63	37	18	[illegible]
Chr03	2981141	A	AAT	Chr03_29811	A_AAT	INDEL	939.35	17	9	[illegible]
Chr03	2981141	A	AAT	Chr03_29811	A_AAT	INDEL	939.35	17	9	[illegible]
Chr03	2981141	A	AAT	Chr03_29811	A_AAT	INDEL	939.35	17	9	[illegible]
Chr03	3230193	A	G	Chr03_32301	A_G	SNP	5491.98	0	0	[illegible]
Chr03	3230193	A	G	Chr03_32301	A_G	SNP	5491.98	0	0	[illegible]
Chr03	3290085	GATCTTTAAT	G	Chr03_32900	GATCTTTAAT	INDEL	9551.59	15	10	[illegible]
Chr03	3406186	G	A	Chr03_34061	G_A	SNP	17353.63	30	13	[illegible]
Chr03	3406186	G	A	Chr03_34061	G_A	SNP	17353.63	30	13	[illegible]
Chr03	3406186	G	A	Chr03_34061	G_A	SNP	17353.63	30	13	[illegible]
Chr03	3424472	T	TA	Chr03_34244	T_TA	INDEL	14790.59	33	13	[illegible]
Chr03	3456384	C	CT	Chr03_34563	C_CT	INDEL	14349.59	28	17	[illegible]
Chr03	3456384	C	CT	Chr03_34563	C_CT	INDEL	14349.59	28	17	[illegible]
Chr03	3456384	C	CT	Chr03_34563	C_CT	INDEL	14349.59	28	17	[illegible]
Chr03	3456384	C	CT	Chr03_34563	C_CT	INDEL	14349.59	28	17	[illegible]
Chr03	3456384	C	CT	Chr03_34563	C_CT	INDEL	14349.59	28	17	[illegible]
Chr03	3456384	C	CT	Chr03_34563	C_CT	INDEL	14349.59	28	17	[illegible]
Chr03	3722715	C	CA	Chr03_37227	C_CA	INDEL	15156.59	25	8	[illegible]
Chr03	3722715	C	CA	Chr03_37227	C_CA	INDEL	15156.59	25	8	[illegible]
Chr03	3722715	C	CA	Chr03_37227	C_CA	INDEL	15156.59	25	8	[illegible]
Chr03	3722715	C	CA	Chr03_37227	C_CA	INDEL	15156.59	25	8	[illegible]
Chr03	3722715	C	CA	Chr03_37227	C_CA	INDEL	15156.59	25	8	[illegible]
Chr03	3837665	CAT	C	Chr03_38376	CAT_C	INDEL	10038.59	21	14	[illegible]
Chr03	3982472	T	A	Chr03_39824	T_A	SNP	20960.63	25	18	[illegible]
Chr03	3982472	T	A	Chr03_39824	T_A	SNP	20960.63	25	18	[illegible]
Chr03	3982472	T	A	Chr03_39824	T_A	SNP	20960.63	25	18	[illegible]
Chr03	3982472	T	A	Chr03_39824	T_A	SNP	20960.63	25	18	[illegible]
Chr03	3982472	T	A	Chr03_39824	T_A	SNP	20960.63	25	18	[illegible]
Chr03	3982472	T	A	Chr03_39824	T_A	SNP	20960.63	25	18	[illegible]
Chr03	4125674	C	CA	Chr03_41256	C_CA	INDEL	14074.59	31	15	[illegible]
Chr03	4135717	C	A	Chr03_41357	C_A	SNP	18798.63	24	14	[illegible]
Chr03	4149057	G	T	Chr03_41490	G_T	SNP	19691.63	26	14	[illegible]
Chr03	4149094	G	T	Chr03_41490	G_T	SNP	16894.63	18	12	[illegible]
Chr03	4169821	T	TA	Chr03_41698	T_TA	INDEL	20015.59	23	15	[illegible]
Chr03	4169821	T	TA	Chr03_41698	T_TA	INDEL	20015.59	23	15	[illegible]
Chr03	4169821	T	TA	Chr03_41698	T_TA	INDEL	20015.59	23	15	[illegible]
Chr03	4169827	A	AT	Chr03_41698	A_AT	INDEL	19916.59	22	13	[illegible]
Chr03	4169827	A	AT	Chr03_41698	A_AT	INDEL	19916.59	22	13	[illegible]
Chr03	4169827	A	AT	Chr03_41698	A_AT	INDEL	19916.59	22	13	[illegible]
Chr03	4169870	C	T	Chr03_41698	C_T	SNP	12264.63	13	8	[illegible]
Chr03	4169870	C	T	Chr03_41698	C_T	SNP	12264.63	13	8	[illegible]
Chr03	4169870	C	T	Chr03_41698	C_T	SNP	12264.63	13	8	[illegible]
Chr03	4197373	TA	T	Chr03_41973	TA_T	INDEL	14647.59	37	12	[illegible]
Chr03	4198042	G	GA	Chr03_41980	G_GA	INDEL	15837.59	30	23	[illegible]
Chr03	4198094	G	T	Chr03_41980	G_T	SNP	19320.63	33	19	[illegible]
Chr03	4210105	G	T	Chr03_42101	G_T	SNP	11634.63	15	12	[illegible]
Chr03	4215755	AT	A	Chr03_42157	AT_A	INDEL	16230.59	28	10	[illegible]
Chr03	4215797	A	T	Chr03_42157	A_T	SNP	12632.63	15	5	[illegible]
Chr03	4215799	A	T	Chr03_42157	A_T	SNP	13082.63	15	5	[illegible]
Chr03	4215803	A	T	Chr03_42158	A_T	SNP	13162.63	16	5	[illegible]
Chr03	4215812	A	T	Chr03_42158	A_T	SNP	12679.63	14	5	[illegible]
Chr03	4215877	G	T	Chr03_42158	G_T	SNP	12041.63	13	4	[illegible]
Chr03	4222985	C	G	Chr03_42229	C_G	SNP	12784.63	21	5	[illegible]
Chr03	4222985	C	G	Chr03_42229	C_G	SNP	12784.63	21	5	[illegible]
Chr03	4222985	C	G	Chr03_42229	C_G	SNP	12784.63	21	5	[illegible]
Chr03	4262750	A	G	Chr03_42627	A_G	SNP	16796.63	24	12	[illegible]
Chr03	4307595	G	A	Chr03_43075	G_A	SNP	12459.63	16	9	[illegible]
Chr03	4324241	CTA	C	Chr03_43242	CTA_C	INDEL	10373.59	24	14	[illegible]
Chr03	4324241	CTA	C	Chr03_43242	CTA_C	INDEL	10373.59	24	14	[illegible]
Chr03	4394969	A	AT	Chr03_43949	A_AT	INDEL	17851.59	32	17	[illegible]
Chr03	4394969	A	AT	Chr03_43949	A_AT	INDEL	17851.59	32	17	[illegible]
Chr03	4442858	A	T	Chr03_44428	A_T	SNP	15450.63	17	12	[illegible]
Chr03	4442879	T	A	Chr03_44428	T_A	SNP	17050.63	20	14	[illegible]
Chr03	4480871	G	GA	Chr03_44808	G_GA	INDEL	17491.59	20	17	[illegible]
Chr03	4480879	T	A	Chr03_44808	T_A	SNP	18106.63	22	17	[illegible]
Chr03	4519239	A	AT	Chr03_45192	A_AT	INDEL	14938.59	18	16	[illegible]
Chr03	4519239	A	AT	Chr03_45192	A_AT	INDEL	14938.59	18	16	[illegible]
Chr03	4519239	A	AT	Chr03_45192	A_AT	INDEL	14938.59	18	16	[illegible]
Chr03	4697245	G	A	Chr03_46972	G_A	SNP	5112.29	0	0	[illegible]
Chr03	4719499	T	TTA	Chr03_47194	T_TTA	INDEL	654.37	0	0	[illegible]
Chr03	4719499	T	TTA	Chr03_47194	T_TTA	INDEL	654.37	0	0	[illegible]
Chr03	4719499	T	TTA	Chr03_47194	T_TTA	INDEL	654.37	0	0	[illegible]
Chr03	4719499	T	TTA	Chr03_47194	T_TTA	INDEL	654.37	0	0	[illegible]
Chr03	4750087	CATATAT	C	Chr03_47500	CATATAT_C	INDEL	3434.93	0	0	[illegible]
Chr03	4750087	CATATAT	C	Chr03_47500	CATATAT_C	INDEL	3434.93	0	0	[illegible]
Chr03	4750087	CATATAT	C	Chr03_47500	CATATAT_C	INDEL	3434.93	0	0	[illegible]
Chr03	4750087	CATATAT	C	Chr03_47500	CATATAT_C	INDEL	3434.93	0	0	[illegible]
Chr03	4750087	CATATAT	C	Chr03_47500	CATATAT_C	INDEL	3434.93	0	0	[illegible]
Chr03	4750087	CATATAT	C	Chr03_47500	CATATAT_C	INDEL	3434.93	0	0	[illegible]
Chr03	4750087	CATATAT	C	Chr03_47500	CATATAT_C	INDEL	3434.93	0	0	[illegible]
Chr03	4750087	CATATAT	C	Chr03_47500	CATATAT_C	INDEL	3434.93	0	0	[illegible]
Chr03	4750087	CATATAT	C	Chr03_47500	CATATAT_C	INDEL	3434.93	0	0	[illegible]
Chr03	4750087	CATATAT	CATATATAT	Chr03_47500	CATATAT_CA	INDEL	3434.93	0	0	[illegible]
Chr03	4750087	CATATAT	CATATATAT	Chr03_47500	CATATAT_CA	INDEL	3434.93	0	0	[illegible]
Chr03	4750087	CATATAT	CATATATAT	Chr03_47500	CATATAT_CA	INDEL	3434.93	0	0	[illegible]
Chr03	4750087	CATATAT	CATATATAT	Chr03_47500	CATATAT_CA	INDEL	3434.93	0	0	[illegible]
Chr03	4750087	CATATAT	CATATATAT	Chr03_47500	CATATAT_CA	INDEL	3434.93	0	0	[illegible]
Chr03	4750087	CATATAT	CATATATAT	Chr03_47500	CATATAT_CA	INDEL	3434.93	0	0	[illegible]
Chr03	4750087	CATATAT	CATATATAT	Chr03_47500	CATATAT_CA	INDEL	3434.93	0	0	[illegible]
Chr03	4750087	CATATAT	CATATATAT	Chr03_47500	CATATAT_CA	INDEL	3434.93	0	0	[illegible]
Chr03	4750087	CATATAT	CATATATAT	Chr03_47500	CATATAT_CA	INDEL	3434.93	0	0	[illegible]
Chr03	4750087	CATATAT	CATATATATA	Chr03_47500	CATATAT_CA	INDEL	3434.93	1	0	[illegible]
Chr03	4750087	CATATAT	CATATATATA	Chr03_47500	CATATAT_CA	INDEL	3434.93	1	0	[illegible]
Chr03	4750087	CATATAT	CATATATATA	Chr03_47500	CATATAT_CA	INDEL	3434.93	1	0	[illegible]
Chr03	4750087	CATATAT	CATATATATA	Chr03_47500	CATATAT_CA	INDEL	3434.93	1	0	[illegible]
Chr03	4750087	CATATAT	CATATATATA	Chr03_47500	CATATAT_CA	INDEL	3434.93	1	0	[illegible]
Chr03	4750087	CATATAT	CATATATATA	Chr03_47500	CATATAT_CA	INDEL	3434.93	1	0	[illegible]
Chr03	4750087	CATATAT	CATATATATA	Chr03_47500	CATATAT_CA	INDEL	3434.93	1	0	[illegible]
Chr03	4750087	CATATAT	CATATATATA	Chr03_47500	CATATAT_CA	INDEL	3434.93	1	0	[illegible]
Chr03	4750087	CATATAT	CATATATATA	Chr03_47500	CATATAT_CA	INDEL	3434.93	1	0	[illegible]
Chr03	4784838	G	A	Chr03_47848	G_A	SNP	12539.63	10	13	[illegible]
Chr03	4784850	G	A	Chr03_47848	G_A	SNP	12588.63	9	14	[illegible]
Chr03	4880159	G	T	Chr03_48801	G_T	SNP	18467.63	29	12	[illegible]
Chr03	4880199	G	T	Chr03_48801	G_T	SNP	20926.63	33	12	[illegible]
Chr03	4880213	G	A	Chr03_48802	G_A	SNP	21878.63	31	15	[illegible]
Chr03	4883779	G	A	Chr03_48837	G_A	SNP	5409.29	0	0	[illegible]
Chr03	4883785	C	T	Chr03_48837	C_T	SNP	5430.29	0	0	[illegible]
Chr03	5026958	G	T	Chr03_50269	G_T	SNP	3820.29	0	0	[illegible]
Chr03	5130964	G	C	Chr03_51309	G_C	SNP	20825.63	31	17	[illegible]
Chr03	5131315	C	A	Chr03_51313	C_A	SNP	18423.63	24	12	[illegible]
Chr03	5131429	C	A	Chr03_51314	C_A	SNP	12700.63	14	6	[illegible]
Chr03	5131442	C	A	Chr03_51314	C_A	SNP	10941.63	10	6	[illegible]
Chr03	5131455	C	A	Chr03_51314	C_A	SNP	12376.63	13	6	[illegible]
Chr03	5131462	A	AC	Chr03_51314	A_AC	INDEL	12352.59	13	5	[illegible]
Chr03	5131472	A	AC	Chr03_51314	A_AC	INDEL	10199.59	16	4	[illegible]
Chr03	5131512	TG	T	Chr03_51315	TG_T	INDEL	11267.59	22	4	[illegible]
Chr03	5189232	G	A	Chr03_51892	G_A	SNP	15956.63	19	12	[illegible]
Chr03	5222707	G	GA	Chr03_52227	G_GA	INDEL	18156.59	24	16	[illegible]
Chr03	5222718	C	CA	Chr03_52227	C_CA	INDEL	18336.59	24	14	[illegible]
Chr03	5223351	T	A	Chr03_52233	T_A	SNP	11231.63	9	8	[illegible]
Chr03	5225532	C	A	Chr03_52255	C_A	SNP	18365.63	24	18	[illegible]
Chr03	5232442	C	A	Chr03_52324	C_A	SNP	19715.63	33	19	[illegible]
Chr03	5232447	C	A	Chr03_52324	C_A	SNP	19842.63	34	19	[illegible]
Chr03	5232448	G	A	Chr03_52324	G_A	SNP	19666.63	33	18	[illegible]
Chr03	5235086	G	GT	Chr03_52350	G_GT	INDEL	9157.59	7	10	[illegible]
Chr03	5235121	T	A	Chr03_52351	T_A	SNP	11151.63	12	7	[illegible]
Chr03	5235129	C	CA	Chr03_52351	C_CA	INDEL	12047.59	13	5	[illegible]
Chr03	5256618	G	T	Chr03_52566	G_T	SNP	19029.63	29	12	[illegible]
Chr03	5337370	G	A	Chr03_53373	G_A	SNP	6197.29	0	0	[illegible]
Chr03	5417819	A	G	Chr03_54178	A_G	SNP	11734.63	12	14	[illegible]
Chr03	5417869	T	C	Chr03_54178	T_C	SNP	13160.63	17	16	[illegible]
Chr03	5417967	G	C	Chr03_54179	G_C	SNP	21280.63	31	15	[illegible]
Chr03	5417989	T	C	Chr03_54179	T_C	SNP	21572.63	30	14	[illegible]
Chr03	5418004	T	C	Chr03_54180	T_C	SNP	21422.63	32	15	[illegible]
Chr03	5511699	T	C	Chr03_55116	T_C	SNP	17588.63	18	7	[illegible]
Chr03	5575487	T	TTA	Chr03_55754	T_TTA	INDEL	2827.24	1	0	[illegible]
Chr03	5575487	T	TTA	Chr03_55754	T_TTA	INDEL	2827.24	1	0	[illegible]
Chr03	5575487	T	TTA	Chr03_55754	T_TTA	INDEL	2827.24	1	0	[illegible]
Chr03	5575487	T	TTA	Chr03_55754	T_TTA	INDEL	2827.24	1	0	[illegible]
Chr03	5575487	T	TTA	Chr03_55754	T_TTA	INDEL	2827.24	1	0	[illegible]
Chr03	5575487	T	TTA	Chr03_55754	T_TTA	INDEL	2827.24	1	0	[illegible]
Chr03	5575487	T	TTA	Chr03_55754	T_TTA	INDEL	2827.24	1	0	[illegible]
Chr03	5575487	T	TTA	Chr03_55754	T_TTA	INDEL	2827.24	1	0	[illegible]
Chr03	5575487	T	TTA	Chr03_55754	T_TTA	INDEL	2827.24	1	0	[illegible]
Chr03	6000925	C	CAT	Chr03_60009	C_CAT	INDEL	817.37	0	0	[illegible]
Chr03	6011037	C	T	Chr03_60110	C_T	SNP	2972.39	38	22	[illegible]
Chr03	6011037	C	T	Chr03_60110	C_T	SNP	2972.39	38	22	[illegible]
Chr03	6419141	A	AC	Chr03_64191	A_AC	INDEL	13440.59	17	21	[illegible]
Chr03	6565791	GAAGGCC	G	Chr03_65657	GAAGGCC_G	INDEL	12498.59	14	15	[illegible]
Chr03	6565799	C	T	Chr03_65657	C_T	SNP	12687.63	14	15	[illegible]
Chr03	6565801	A	T	Chr03_65658	A_T	SNP	13970.63	14	15	[illegible]
Chr03	6565802	C	CTTCCTT	Chr03_65658	C_CTTCCTT	INDEL	14051.59	15	15	[illegible]
Chr03	6565805	TGATCAAA	T	Chr03_65658	TGATCAAA_T	INDEL	13950.59	13	20	[illegible]
Chr03	6565812	A	ATTTTTTT	Chr03_65658	A_ATTTTTTT	INDEL	13005.59	13	20	[illegible]
Chr03	6565982	A	ATTTTCGT	Chr03_65659	A_ATTTTCGT	INDEL	14733.59	22	10	[illegible]
Chr03	6565983	AGGGGGG	A	Chr03_65659	AGGGGGG_	INDEL	14831.59	19	9	[illegible]
Chr03	6565990	G	C	Chr03_65659	G_C	SNP	13574.63	19	9	[illegible]

Chr03	6565993	A	C	Chr03_65659	A_C	SNP	13484.63	17	9	11
Chr03	6642740	T	C	Chr03_66427	T_C	SNP	11021.63	20	8	10
Chr03	6642740	T	C	Chr03_66427	T_C	SNP	11021.63	20	8	10
Chr03	6642740	T	C	Chr03_66427	T_C	SNP	11021.63	20	8	10
Chr03	6642740	T	C	Chr03_66427	T_C	SNP	11021.63	20	8	10
Chr03	6846897	C	T	Chr03_68468	C_T	SNP	9666.63	20	4	8
Chr03	6905157	G	C	Chr03_69051	G_C	SNP	17748.63	23	8	12
Chr03	6905157	G	C	Chr03_69051	G_C	SNP	17748.63	23	8	12
Chr03	6905157	G	C	Chr03_69051	G_C	SNP	17748.63	23	8	12
Chr03	6905157	G	C	Chr03_69051	G_C	SNP	17748.63	23	8	12
Chr03	6905157	G	C	Chr03_69051	G_C	SNP	17748.63	23	8	12
Chr03	6905157	G	C	Chr03_69051	G_C	SNP	17748.63	23	8	12
Chr03	6905157	G	C	Chr03_69051	G_C	SNP	17748.63	23	8	12
Chr03	6905157	G	C	Chr03_69051	G_C	SNP	17748.63	23	8	12
Chr03	6905157	G	C	Chr03_69051	G_C	SNP	17748.63	23	8	12
Chr03	6922040	A	AAT	Chr03_69220	A_AAT	INDEL	3069.24	0	0	1
Chr03	7462716	T	C	Chr03_74627	T_C	SNP	17262.63	19	12	16
Chr03	7462717	G	A	Chr03_74627	G_A	SNP	17442.63	19	12	16
Chr03	7462722	T	A	Chr03_74627	T_A	SNP	17692.63	19	12	15
Chr03	7462733	G	A	Chr03_74627	G_A	SNP	17030.63	16	12	14
Chr03	7713664	C	A	Chr03_77136	C_A	SNP	15127.63	21	13	10
Chr03	7713679	C	A	Chr03_77136	C_A	SNP	13676.63	22	12	7
Chr03	7828318	G	GTA	Chr03_78283	G_GTA	INDEL	3209.24	1	0	1
Chr03	7828318	G	GTA	Chr03_78283	G_GTA	INDEL	3209.24	1	0	1

[illegible]

Chr03	12095344	TTG	T	Chr03_12095TTG_T	INDEL	8426.59	11	5	4	10	18	11	13	9	6	7	4	5	5
Chr03	12095344	TTG	T	Chr03_12095TTG_T	INDEL	8426.59	11	5	4	10	18	11	13	9	6	7	4	5	5
Chr03	12095344	TTG	T	Chr03_12095TTG_T	INDEL	8426.59	11	5	4	10	18	11	13	9	6	7	4	5	5
Chr03	12095344	TTG	T	Chr03_12095TTG_T	INDEL	8426.59	11	5	4	10	18	11	13	9	6	7	4	5	5
Chr03	12095344	TTG	T	Chr03_12095TTG_T	INDEL	8426.59	11	5	4	10	18	11	13	9	6	7	4	5	5
Chr03	12095347	G	GTAA	Chr03_12095G_GTAA	INDEL	8201.59	11	5	4	10	18	10	13	9	6	7	4	5	5
Chr03	12095347	G	GTAA	Chr03_12095G_GTAA	INDEL	8201.59	11	5	4	10	18	10	13	9	6	7	4	5	5
Chr03	12095347	G	GTAA	Chr03_12095G_GTAA	INDEL	8201.59	11	5	4	10	18	10	13	9	6	7	4	5	5
Chr03	12095347	G	GTAA	Chr03_12095G_GTAA	INDEL	8201.59	11	5	4	10	18	10	13	9	6	7	4	5	5
Chr03	12095347	G	GTAA	Chr03_12095G_GTAA	INDEL	8201.59	11	5	4	10	18	10	13	9	6	7	4	5	5
Chr03	12095347	G	GTAA	Chr03_12095G_GTAA	INDEL	8201.59	11	5	4	10	18	10	13	9	6	7	4	5	5
Chr03	12095352	G	C	Chr03_12095G_C	SNP	7625.63	10	5	4	9	16	6	12	9	6	6	4	5	5
Chr03	12095352	G	C	Chr03_12095G_C	SNP	7625.63	10	5	4	9	16	6	12	9	6	6	4	5	5
Chr03	12095352	G	C	Chr03_12095G_C	SNP	7625.63	10	5	4	9	16	6	12	9	6	6	4	5	5
Chr03	12095352	G	C	Chr03_12095G_C	SNP	7625.63	10	5	4	9	16	6	12	9	6	6	4	5	5
Chr03	12095352	G	C	Chr03_12095G_C	SNP	7625.63	10	5	4	9	16	6	12	9	6	6	4	5	5
Chr03	12095352	G	C	Chr03_12095G_C	SNP	7625.63	10	5	4	9	16	6	12	9	6	6	4	5	5
Chr03	12095353	ATT	A	Chr03_12095ATT_A	INDEL	7481.59	10	5	4	9	16	6	12	9	6	6	4	5	5
Chr03	12095353	ATT	A	Chr03_12095ATT_A	INDEL	7481.59	10	5	4	9	16	6	12	9	6	6	4	5	5
Chr03	12095353	ATT	A	Chr03_12095ATT_A	INDEL	7481.59	10	5	4	9	16	6	12	9	6	6	4	5	5
Chr03	12095353	ATT	A	Chr03_12095ATT_A	INDEL	7481.59	10	5	4	9	16	6	12	9	6	6	4	5	5
Chr03	12095353	ATT	A	Chr03_12095ATT_A	INDEL	7481.59	10	5	4	9	16	6	12	9	6	6	4	5	5
Chr03	12095353	ATT	A	Chr03_12095ATT_A	INDEL	7481.59	10	5	4	9	16	6	12	9	6	6	4	5	5
Chr03	12102291	T	C	Chr03_12102T_C	SNP	18814.63	19	6	23	24	22	20	37	20	25	20	18	15	10

[illegible]

Chr03	13695632	GA	G	Chr03_13695GA_G	INDEL	10805.59	9	7	12	20	9	14	18	9	8	13	10	8	9	7
Chr03	13695632	GA	G	Chr03_13695GA_G	INDEL	10805.59	9	7	12	20	9	14	18	9	8	13	10	8	9	7
Chr03	13695677	G	T	Chr03_13695G_T	SNP	13758.63	12	8	16	24	15	14	21	16	13	15	13	12	11	6
Chr03	13695677	G	T	Chr03_13695G_T	SNP	13758.63	12	8	16	24	15	14	21	16	13	15	13	12	11	6
Chr03	13695677	G	T	Chr03_13695G_T	SNP	13758.63	12	8	16	24	15	14	21	16	13	15	13	12	11	6
Chr03	13695677	G	T	Chr03_13695G_T	SNP	13758.63	12	8	16	24	15	14	21	16	13	15	13	12	11	6
Chr03	13695677	G	T	Chr03_13695G_T	SNP	13758.63	12	8	16	24	15	14	21	16	13	15	13	12	11	6
Chr03	13918915	C	CTA	Chr03_13918C_CTA	INDEL	217.37	0	1	0	1	11	0	0	2	1	1	0	0	0	0
Chr03	13918915	C	CTA	Chr03_13918C_CTA	INDEL	217.37	0	1	0	1	11	0	0	2	1	1	0	0	0	0
Chr03	13918915	C	CTA	Chr03_13918C_CTA	INDEL	217.37	0	1	0	1	11	0	0	2	1	1	0	0	0	0
Chr03	13918915	C	CTA	Chr03_13918C_CTA	INDEL	217.37	0	1	0	1	11	0	0	2	1	1	0	0	0	0
Chr03	13918915	C	CTA	Chr03_13918C_CTA	INDEL	217.37	0	1	0	1	11	0	0	2	1	1	0	0	0	0
Chr03	13918915	C	CTA	Chr03_13918C_CTA	INDEL	217.37	0	1	0	1	11	0	0	2	1	1	0	0	0	0
Chr03	13918915	C	CTA	Chr03_13918C_CTA	INDEL	217.37	0	1	0	1	11	0	0	2	1	1	0	0	0	0
Chr03	14429157	C	CAT	Chr03_14429C_CAT	INDEL	3122.24	0	0	0	15	12	21	21	11	1	0	0	0	0	0
Chr03	14430942	TTA	T	Chr03_14430TTA_T	INDEL	6039.24	19	14	8	0	0	0	1	0	19	26	7	18	7	6
Chr03	14468334	GAT	G	Chr03_14468GAT_G	INDEL	6419.24	25	10	16	0	0	0	0	0	12	23	8	8	6	4
Chr03	14468334	GAT	G	Chr03_14468GAT_G	INDEL	6419.24	25	10	16	0	0	0	0	0	12	23	8	8	6	4
Chr03	14468334	GAT	G	Chr03_14468GAT_G	INDEL	6419.24	25	10	16	0	0	0	0	0	12	23	8	8	6	4
Chr03	14468334	GAT	G	Chr03_14468GAT_G	INDEL	6419.24	25	10	16	0	0	0	0	0	12	23	8	8	6	4
Chr03	14578166	T	A	Chr03_14578T_A	SNP	2590.39	33	22	11	0	0	0	0	0	0	0	0	0	0	0
Chr03	14766576	C	CT	Chr03_14766C_CT	INDEL	17458.59	26	11	16	31	20	31	20	21	20	23	21	22	6	10
Chr03	14766594	C	A	Chr03_14766C_A	SNP	18041.63	29	13	14	25	19	31	22	24	20	21	17	26	6	10
Chr03	14766639	T	A	Chr03_14766T_A	SNP	19723.63	33	16	14	26	16	28	21	25	21	18	18	21	10	10

[illegible]

Chr03	17735824 G	GTA	Chr03_17735G_GTA	INDEL	2912.24	0	0	0	16	12	21	26	17	0	1	0	1	0	1	[illegible]	
Chr03	17735824 G	GTA	Chr03_17735G_GTA	INDEL	2912.24	0	0	0	16	12	21	26	17	0	1	0	1	0	1	[illegible]	
Chr03	17735824 G	GTA	Chr03_17735G_GTA	INDEL	2912.24	0	0	0	16	12	21	26	17	0	1	0	1	0	1	[illegible]	
Chr03	17735824 G	GTA	Chr03_17735G_GTA	INDEL	2912.24	0	0	0	16	12	21	26	17	0	1	0	1	0	1	[illegible]	
Chr03	17735824 G	GTA	Chr03_17735G_GTA	INDEL	2912.24	0	0	0	16	12	21	26	17	0	1	0	1	0	1	[illegible]	
Chr03	17735824 G	GTA	Chr03_17735G_GTA	INDEL	2912.24	0	0	0	16	12	21	26	17	0	1	0	1	0	1	[illegible]	
Chr03	17735824 G	GTA	Chr03_17735G_GTA	INDEL	2912.24	0	0	0	16	12	21	26	17	0	1	0	1	0	1	[illegible]	
Chr03	17735824 G	GTA	Chr03_17735G_GTA	INDEL	2912.24	0	0	0	16	12	21	26	17	0	1	0	1	0	1	[illegible]	
Chr03	17735824 G	GTA	Chr03_17735G_GTA	INDEL	2912.24	0	0	0	16	12	21	26	17	0	1	0	1	0	1	[illegible]	
Chr03	17735824 G	GTA	Chr03_17735G_GTA	INDEL	2912.24	0	0	0	16	12	21	26	17	0	1	0	1	0	1	[illegible]	
Chr03	17735824 G	GTA	Chr03_17735G_GTA	INDEL	2912.24	0	0	0	16	12	21	26	17	0	1	0	1	0	1	[illegible]	
Chr03	17735824 G	GTA	Chr03_17735G_GTA	INDEL	2912.24	0	0	0	16	12	21	26	17	0	1	0	1	0	1	[illegible]	
Chr03	17735824 G	GTA	Chr03_17735G_GTA	INDEL	2912.24	0	0	0	16	12	21	26	17	0	1	0	1	0	1	[illegible]	
Chr03	17735824 G	GTA	Chr03_17735G_GTA	INDEL	2912.24	0	0	0	16	12	21	26	17	0	1	0	1	0	1	[illegible]	
Chr03	17735824 G	GTA	Chr03_17735G_GTA	INDEL	2912.24	0	0	0	16	12	21	26	17	0	1	0	1	0	1	[illegible]	
Chr03	17735824 G	GTA	Chr03_17735G_GTA	INDEL	2912.24	0	0	0	16	12	21	26	17	0	1	0	1	0	1	[illegible]	
Chr03	17799834 G	GTGTAC	Chr03_17799G_GTGTAC	INDEL	18144.59	20	21	5	18	24	30	19	18	20	20	17	10	17	8	[illegible]	
Chr03	17799834 G	GTGTAC	Chr03_17799G_GTGTAC	INDEL	18144.59	20	21	5	18	24	30	19	18	20	20	17	10	17	8	[illegible]	
Chr03	17799834 G	GTGTAC	Chr03_17799G_GTGTAC	INDEL	18144.59	20	21	5	18	24	30	19	18	20	20	17	10	17	8	[illegible]	
[illegible]																					

Chr03	21457693	A	T	Chr03_21457	A_T	SNP	19191.59	23	14	15	24	13	22	34	27	26	12	14	18	8	13
Chr03	21457693	A	T	Chr03_21457	A_T	SNP	19191.59	23	14	15	24	13	22	34	27	26	12	14	18	8	13
Chr03	21457865	G	GT	Chr03_21457	G_GT	INDEL	10977.59	23	16	11	35	9	20	20	23	22	20	14	16	10	5
Chr03	21457865	G	GT	Chr03_21457	G_GT	INDEL	10977.59	23	16	11	35	9	20	20	23	22	20	14	16	10	5
Chr03	21457865	G	GT	Chr03_21457	G_GT	INDEL	10977.59	23	16	11	35	9	20	20	23	22	20	14	16	10	5
Chr03	21485161	C	G	Chr03_21485	C_G	SNP	19096.63	25	12	14	24	32	23	38	27	18	17	14	15	21	7
Chr03	21485161	C	G	Chr03_21485	C_G	SNP	19096.63	25	12	14	24	32	23	38	27	18	17	14	15	21	7
Chr03	21485161	C	G	Chr03_21485	C_G	SNP	19096.63	25	12	14	24	32	23	38	27	18	17	14	15	21	7
Chr03	21630037	G	A	Chr03_21630	G_A	SNP	5249.29	0	0	0	27	19	18	28	14	0	0	0	0	0	0
Chr03	21678824	A	AT	Chr03_21678	A_AT	INDEL	18860.59	27	13	12	20	17	24	24	19	21	18	23	19	10	7
Chr03	21678838	C	T	Chr03_21678	C_T	SNP	18916.63	26	11	11	19	17	20	23	19	19	17	22	17	9	6
Chr03	21678857	TG	T	Chr03_21678	TG_T	INDEL	18882.59	24	9	10	24	15	19	24	18	23	19	21	20	8	5
Chr03	21678860	G	GT	Chr03_21678	G_GT	INDEL	18495.59	24	9	9	23	15	19	24	18	22	19	21	20	8	6
Chr03	21682676	T	TA	Chr03_21682	T_TA	INDEL	14937.59	30	11	16	27	22	36	29	24	17	17	17	16	17	9
Chr03	21809620	T	C	Chr03_21809	T_C	SNP	10552.63	8	9	13	16	11	9	15	13	15	13	10	12	7	6
Chr03	21820928	A	AAT	Chr03_21820	A_AAT	INDEL	689.35	15	8	8	0	1	2	2	1	1	0	0	0	0	0
Chr03	21820928	A	AAT	Chr03_21820	A_AAT	INDEL	689.35	15	8	8	0	1	2	2	1	1	0	0	0	0	0
Chr03	21820928	A	AAT	Chr03_21820	A_AAT	INDEL	689.35	15	8	8	0	1	2	2	1	1	0	0	0	0	0
Chr03	21820928	A	AAT	Chr03_21820	A_AAT	INDEL	689.35	15	8	8	0	1	2	2	1	1	0	0	0	0	0
Chr03	21820928	A	AAT	Chr03_21820	A_AAT	INDEL	689.35	15	8	8	0	1	2	2	1	1	0	0	0	0	0
Chr03	21868297	A	AAT	Chr03_21868	A_AAT	INDEL	969.35	12	14	8	0	0	0	0	2	0	0	0	0	1	0
Chr03	21868297	A	AAT	Chr03_21868	A_AAT	INDEL	969.35	12	14	8	0	0	0	0	2	0	0	0	0	1	0
Chr03	21868297	A	AAT	Chr03_21868	A_AAT	INDEL	969.35	12	14	8	0	0	0	0	2	0	0	0	0	1	0
Chr03	21868297	A	AAT	Chr03_21868	A_AAT	INDEL	969.35	12	14	8	0	0	0	0	2	0	0	0	0	1	0

[illegible]

Chr03	23886466	C	G	Chr03_23886	C_G	SNP	18965.63	16	23	18	21	13	27	24	29	21	22
Chr03	23886466	C	G	Chr03_23886	C_G	SNP	18965.63	16	23	18	21	13	27	24	29	21	22
Chr03	23886466	C	G	Chr03_23886	C_G	SNP	18965.63	16	23	18	21	13	27	24	29	21	22
Chr03	23886466	C	G	Chr03_23886	C_G	SNP	18965.63	16	23	18	21	13	27	24	29	21	22
Chr03	23886466	C	G	Chr03_23886	C_G	SNP	18965.63	16	23	18	21	13	27	24	29	21	22
Chr03	23886466	C	G	Chr03_23886	C_G	SNP	18965.63	16	23	18	21	13	27	24	29	21	22
Chr03	23886466	C	G	Chr03_23886	C_G	SNP	18965.63	16	23	18	21	13	27	24	29	21	22
Chr03	23886466	C	G	Chr03_23886	C_G	SNP	18965.63	16	23	18	21	13	27	24	29	21	22
Chr03	23886466	C	G	Chr03_23886	C_G	SNP	18965.63	16	23	18	21	13	27	24	29	21	22
Chr03	23886466	C	G	Chr03_23886	C_G	SNP	18965.63	16	23	18	21	13	27	24	29	21	22
Chr03	23886466	C	G	Chr03_23886	C_G	SNP	18965.63	16	23	18	21	13	27	24	29	21	22
Chr03	23886471	T	C	Chr03_23886	T_C	SNP	17795.63	16	21	18	21	13	24	24	25	20	23
Chr03	23886471	T	C	Chr03_23886	T_C	SNP	17795.63	16	21	18	21	13	24	24	25	20	23
Chr03	23886471	T	C	Chr03_23886	T_C	SNP	17795.63	16	21	18	21	13	24	24	25	20	23
Chr03	23886471	T	C	Chr03_23886	T_C	SNP	17795.63	16	21	18	21	13	24	24	25	20	23
Chr03	23886471	T	C	Chr03_23886	T_C	SNP	17795.63	16	21	18	21	13	24	24	25	20	23
Chr03	23886471	T	C	Chr03_23886	T_C	SNP	17795.63	16	21	18	21	13	24	24	25	20	23
Chr03	23886471	T	C	Chr03_23886	T_C	SNP	17795.63	16	21	18	21	13	24	24	25	20	23
Chr03	23886471	T	C	Chr03_23886	T_C	SNP	17795.63	16	21	18	21	13	24	24	25	20	23
Chr03	23886471	T	C	Chr03_23886	T_C	SNP	17795.63	16	21	18	21	13	24	24	25	20	23
Chr03	23886471	T	C	Chr03_23886	T_C	SNP	17795.63	16	21	18	21	13	24	24	25	20	23
Chr03	23886471	T	C	Chr03_23886	T_C	SNP	17795.63	16	21	18	21	13	24	24	25	20	23
Chr03	23886471	T	C	Chr03_23886	T_C	SNP	17795.63	16	21	18	21	13	24	24	25	20	23
Chr03	23886473	GCCTCAAC	G	Chr03_23886	GCCTCAAC_[illegible]	INDEL	17426.59	16	20	18	21	13	24	23	24	20	23

[illegible]

Chr04	583111	G	A	Chr04_58311 G_A	SNP	16175.63	36	[illegible]
Chr04	600012	A	T	Chr04_60001 A_T	SNP	18848.63	27	[illegible]
Chr04	774899	G	T	Chr04_77489 G_T	SNP	17993.63	20	[illegible]
Chr04	774899	G	T	Chr04_77489 G_T	SNP	17993.63	20	[illegible]
Chr04	774899	G	T	Chr04_77489 G_T	SNP	17993.63	20	[illegible]
Chr04	774899	G	T	Chr04_77489 G_T	SNP	17993.63	20	[illegible]
Chr04	774900	A	T	Chr04_77490 A_T	SNP	18224.63	20	[illegible]
Chr04	774900	A	T	Chr04_77490 A_T	SNP	18224.63	20	[illegible]
Chr04	774900	A	T	Chr04_77490 A_T	SNP	18224.63	20	[illegible]
Chr04	774900	A	T	Chr04_77490 A_T	SNP	18224.63	20	[illegible]
Chr04	774903	G	C	Chr04_77490 G_C	SNP	18223.63	19	[illegible]
Chr04	774903	G	C	Chr04_77490 G_C	SNP	18223.63	19	[illegible]
Chr04	774903	G	C	Chr04_77490 G_C	SNP	18223.63	19	[illegible]
Chr04	774903	G	C	Chr04_77490 G_C	SNP	18223.63	19	[illegible]
Chr04	787220	A	AT	Chr04_78722 A_AT	INDEL	14048.59	22	[illegible]
Chr04	787220	A	AT	Chr04_78722 A_AT	INDEL	14048.59	22	[illegible]
Chr04	787220	A	AT	Chr04_78722 A_AT	INDEL	14048.59	22	[illegible]
Chr04	787220	A	AT	Chr04_78722 A_AT	INDEL	14048.59	22	[illegible]
Chr04	787220	A	AT	Chr04_78722 A_AT	INDEL	14048.59	22	[illegible]
Chr04	787220	A	AT	Chr04_78722 A_AT	INDEL	14048.59	22	[illegible]
Chr04	897810	A	AAT	Chr04_89781 A_AAT	INDEL	130.37	2	[illegible]
Chr04	897810	A	AAT	Chr04_89781 A_AAT	INDEL	130.37	2	[illegible]
Chr04	897810	A	AAT	Chr04_89781 A_AAT	INDEL	130.37	2	[illegible]
Chr04	897810	A	AAT	Chr04_89781 A_AAT	INDEL	130.37	2	[illegible]
Chr04	897810	A	AAT	Chr04_89781 A_AAT	INDEL	130.37	2	[illegible]
Chr04	897810	A	AAT	Chr04_89781 A_AAT	INDEL	130.37	2	[illegible]
Chr04	897810	A	AAT	Chr04_89781 A_AAT	INDEL	130.37	2	[illegible]
Chr04	897810	A	AAT	Chr04_89781 A_AAT	INDEL	130.37	2	[illegible]
Chr04	897810	A	AAT	Chr04_89781 A_AAT	INDEL	130.37	2	[illegible]
Chr04	897810	A	AAT	Chr04_89781 A_AAT	INDEL	130.37	2	[illegible]
Chr04	897810	A	AAT	Chr04_89781 A_AAT	INDEL	130.37	2	[illegible]
Chr04	897810	A	AAT	Chr04_89781 A_AAT	INDEL	130.37	2	[illegible]
Chr04	897810	A	AAT	Chr04_89781 A_AAT	INDEL	130.37	2	[illegible]
Chr04	897810	A	AAT	Chr04_89781 A_AAT	INDEL	130.37	2	[illegible]
Chr04	897810	A	AAT	Chr04_89781 A_AAT	INDEL	130.37	2	[illegible]
Chr04	986574	A	G	Chr04_98657 A_G	SNP	15423.63	12	[illegible]
Chr04	986671	G	T	Chr04_98667 G_T	SNP	18427.63	18	[illegible]
Chr04	1137679	A	AAT	Chr04_11376 A_AAT	INDEL	297.37	0	[illegible]
Chr04	1189095	T	TTA	Chr04_11890 T_TTA	INDEL	16630.59	31	[illegible]
Chr04	1213879	T	TAAAAATTA/	Chr04_12138 T_TAAAAATT	INDEL	15460.59	24	[illegible]
Chr04	1213879	T	TAAAAATTA/	Chr04_12138 T_TAAAAATT	INDEL	15460.59	24	[illegible]
Chr04	1369713	C	CTA	Chr04_13697 C_CTA	INDEL	4242.24	0	[illegible]
Chr04	1369713	C	CTA	Chr04_13697 C_CTA	INDEL	4242.24	0	[illegible]
Chr04	1369713	C	CTA	Chr04_13697 C_CTA	INDEL	4242.24	0	[illegible]
Chr04	1369713	C	CTA	Chr04_13697 C_CTA	INDEL	4242.24	0	[illegible]
Chr04	1369713	C	CTA	Chr04_13697 C_CTA	INDEL	4242.24	0	[illegible]
Chr04	1369713	C	CTA	Chr04_13697 C_CTA	INDEL	4242.24	0	[illegible]
Chr04	1369713	C	CTA	Chr04_13697 C_CTA	INDEL	4242.24	0	[illegible]
Chr04	1369713	C	CTA	Chr04_13697 C_CTA	INDEL	4242.24	0	[illegible]
Chr04	1369713	C	CTA	Chr04_13697 C_CTA	INDEL	4242.24	0	[illegible]
Chr04	1498977	T	A	Chr04_14989 T_A	SNP	15016.63	16	[illegible]
Chr04	1498977	T	A	Chr04_14989 T_A	SNP	15016.63	16	[illegible]
Chr04	1498977	T	A	Chr04_14989 T_A	SNP	15016.63	16	[illegible]
Chr04	1498981	T	C	Chr04_14989 T_C	SNP	15417.63	16	[illegible]
Chr04	1498981	T	C	Chr04_14989 T_C	SNP	15417.63	16	[illegible]
Chr04	1498981	T	C	Chr04_14989 T_C	SNP	15417.63	16	[illegible]
Chr04	1498982	T	C	Chr04_14989 T_C	SNP	15303.63	16	[illegible]
Chr04	1498982	T	C	Chr04_14989 T_C	SNP	15303.63	16	[illegible]
Chr04	1498982	T	C	Chr04_14989 T_C	SNP	15303.63	16	[illegible]
Chr04	1617120	G	A	Chr04_16171 G_A	SNP	18516.63	28	[illegible]
Chr04	1617181	G	T	Chr04_16171 G_T	SNP	21469.63	25	[illegible]
Chr04	1617191	G	T	Chr04_16171 G_T	SNP	21359.63	26	[illegible]
Chr04	1657554	T	TA	Chr04_16575 T_TA	INDEL	7513.59	15	[illegible]
Chr04	1672902	A	AAT	Chr04_16729 A_AAT	INDEL	3788.24	13	[illegible]
Chr04	1672902	A	AAT	Chr04_16729 A_AAT	INDEL	3788.24	13	[illegible]
Chr04	1672902	A	AAT	Chr04_16729 A_AAT	INDEL	3788.24	13	[illegible]
Chr04	1672902	A	AAT	Chr04_16729 A_AAT	INDEL	3788.24	13	[illegible]
Chr04	1672902	A	AAT	Chr04_16729 A_AAT	INDEL	3788.24	13	[illegible]
Chr04	1827233	C	CTATA	Chr04_18272 C_CTATA	INDEL	2199.93	4	[illegible]
Chr04	1827233	C	CTATA	Chr04_18272 C_CTATA	INDEL	2199.93	4	[illegible]
Chr04	1827233	C	CTATA	Chr04_18272 C_CTATA	INDEL	2199.93	4	[illegible]
Chr04	1847749	CA	C	Chr04_18477 CA_C	INDEL	14652.59	32	[illegible]
Chr04	1931174	G	A	Chr04_19311 G_A	SNP	7404.63	23	[illegible]
Chr04	2154629	G	T	Chr04_21546 G_T	SNP	18396.63	39	[illegible]
Chr04	2154689	C	T	Chr04_21546 C_T	SNP	19968.63	38	[illegible]
Chr04	2154722	C	CA	Chr04_21547 C_CA	INDEL	16431.59	36	[illegible]
Chr04	2154752	G	GA	Chr04_21547 G_GA	INDEL	15328.59	31	[illegible]
Chr04	2333825	C	T	Chr04_23338 C_T	SNP	18736.63	34	[illegible]
Chr04	2333854	C	CA	Chr04_23338 C_CA	INDEL	17471.59	31	[illegible]
Chr04	2519197	ACTT	A	Chr04_25191 ACTT_A	INDEL	19226.59	29	[illegible]
Chr04	2519200	T	TGAAG	Chr04_25192 T_TGAAG	INDEL	19226.59	29	[illegible]
Chr04	2716003	C	CT	Chr04_27160 C_CT	INDEL	13890.59	17	[illegible]
Chr04	2958194	G	A	Chr04_29581 G_A	SNP	10255.63	22	[illegible]
Chr04	2958209	G	A	Chr04_29582 G_A	SNP	13998.63	25	[illegible]
Chr04	2958218	A	G	Chr04_29582 A_G	SNP	14497.63	28	[illegible]
Chr04	3021562	GTA	G	Chr04_30215 GTA_G	INDEL	8546.24	31	[illegible]
Chr04	3021562	GTA	G	Chr04_30215 GTA_G	INDEL	8546.24	31	[illegible]
Chr04	3037685	GT	G	Chr04_30376 GT_G	INDEL	11535.59	24	[illegible]
Chr04	3296017	G	A	Chr04_32960 G_A	SNP	2573.39	28	[illegible]
Chr04	3337238	G	A	Chr04_33372 G_A	SNP	2884.98	0	[illegible]
Chr04	3347037	G	T	Chr04_33470 G_T	SNP	19687.63	28	[illegible]
Chr04	3347037	G	T	Chr04_33470 G_T	SNP	19687.63	28	[illegible]
Chr04	3347037	G	T	Chr04_33470 G_T	SNP	19687.63	28	[illegible]
Chr04	3347037	G	T	Chr04_33470 G_T	SNP	19687.63	28	[illegible]
Chr04	3450824	CTA	C	Chr04_34508 CTA_C	INDEL	8684.59	26	[illegible]
Chr04	3450824	CTA	C	Chr04_34508 CTA_C	INDEL	8684.59	26	[illegible]
Chr04	3450824	CTA	C	Chr04_34508 CTA_C	INDEL	8684.59	26	[illegible]
Chr04	3450824	CTA	C	Chr04_34508 CTA_C	INDEL	8684.59	26	[illegible]
Chr04	3510034	G	T	Chr04_35100 G_T	SNP	15034.63	13	[illegible]
Chr04	3510034	G	T	Chr04_35100 G_T	SNP	15034.63	13	[illegible]
Chr04	3510034	G	T	Chr04_35100 G_T	SNP	15034.63	13	[illegible]
Chr04	3510034	G	T	Chr04_35100 G_T	SNP	15034.63	13	[illegible]
Chr04	3510034	G	T	Chr04_35100 G_T	SNP	15034.63	13	[illegible]
Chr04	3510126	G	T	Chr04_35101 G_T	SNP	16045.63	15	[illegible]
Chr04	3510126	G	T	Chr04_35101 G_T	SNP	16045.63	15	[illegible]
Chr04	3510126	G	T	Chr04_35101 G_T	SNP	16045.63	15	[illegible]
Chr04	3510126	G	T	Chr04_35101 G_T	SNP	16045.63	15	[illegible]
Chr04	3510126	G	T	Chr04_35101 G_T	SNP	16045.63	15	[illegible]
Chr04	3510530	AAT	A	Chr04_35105 AAT_A	INDEL	7884.59	22	[illegible]
Chr04	3510530	AAT	A	Chr04_35105 AAT_A	INDEL	7884.59	22	[illegible]
Chr04	3510530	AAT	A	Chr04_35105 AAT_A	INDEL	7884.59	22	[illegible]
Chr04	3510530	AAT	A	Chr04_35105 AAT_A	INDEL	7884.59	22	[illegible]
Chr04	3510530	AAT	A	Chr04_35105 AAT_A	INDEL	7884.59	22	[illegible]
Chr04	3957094	CTATATA	C	Chr04_39570 CTATATA_C	INDEL	21818.59	37	[illegible]
Chr04	3957094	CTATATA	C	Chr04_39570 CTATATA_C	INDEL	21818.59	37	[illegible]
Chr04	3957094	CTATATA	C	Chr04_39570 CTATATA_C	INDEL	21818.59	37	[illegible]
Chr04	3957094	CTATATA	C	Chr04_39570 CTATATA_C	INDEL	21818.59	37	[illegible]
Chr04	3957094	CTATATA	C	Chr04_39570 CTATATA_C	INDEL	21818.59	37	[illegible]
Chr04	4100184	G	GT	Chr04_41001 G_GT	INDEL	14990.59	24	[illegible]
Chr04	4227121	G	A	Chr04_42271 G_A	SNP	17533.63	21	[illegible]
Chr04	4227121	G	A	Chr04_42271 G_A	SNP	17533.63	21	[illegible]
Chr04	4327218	G	A	Chr04_43272 G_A	SNP	11922.63	17	[illegible]
Chr04	4542954	G	A	Chr04_45429 G_A	SNP	17781.63	27	[illegible]
Chr04	4542990	G	A	Chr04_45429 G_A	SNP	17171.63	24	[illegible]
Chr04	4543005	A	G	Chr04_45430 A_G	SNP	17600.63	22	[illegible]
Chr04	4560635	A	G	Chr04_45606 A_G	SNP	10319.63	6	[illegible]
Chr04	4678420	G	A	Chr04_46784 G_A	SNP	18448.63	24	[illegible]
Chr04	4700301	C	CAT	Chr04_47003 C_CAT	INDEL	3008.24	0	[illegible]
Chr04	4700301	C	CAT	Chr04_47003 C_CAT	INDEL	3008.24	0	[illegible]
Chr04	4700301	C	CAT	Chr04_47003 C_CAT	INDEL	3008.24	0	[illegible]
Chr04	4864726	C	G	Chr04_48647 C_G	SNP	14206.63	20	[illegible]
Chr04	4865570	A	T	Chr04_48655 A_T	SNP	18956.63	25	[illegible]
Chr04	4865571	T	A	Chr04_48655 T_A	SNP	18956.63	25	[illegible]
Chr04	4865647	A	T	Chr04_48656 A_T	SNP	17441.63	29	[illegible]
Chr04	4865710	A	AT	Chr04_48657 A_AT	INDEL	11652.59	31	[illegible]
Chr04	5143795	G	T	Chr04_51437 G_T	SNP	7456.29	0	[illegible]
Chr04	5166702	A	AT	Chr04_51667 A_AT	INDEL	12706.59	25	[illegible]
Chr04	5166945	A	T	Chr04_51669 A_T	SNP	14939.63	33	[illegible]
Chr04	5285040	G	GT	Chr04_52850 G_GT	INDEL	19599.59	37	[illegible]
Chr04	5285040	G	GT	Chr04_52850 G_GT	INDEL	19599.59	37	[illegible]
Chr04	5285040	G	GT	Chr04_52850 G_GT	INDEL	19599.59	37	[illegible]
Chr04	5290425	A	AAT	Chr04_52904 A_AAT	INDEL	1712.93	0	[illegible]
Chr04	5451302	C	CA	Chr04_54513 C_CA	INDEL	12424.59	33	[illegible]
Chr04	5451302	C	CA	Chr04_54513 C_CA	INDEL	12424.59	33	[illegible]
Chr04	5451302	C	CA	Chr04_54513 C_CA	INDEL	12424.59	33	[illegible]
Chr04	5451302	C	CA	Chr04_54513 C_CA	INDEL	12424.59	33	[illegible]
Chr04	5451302	C	CA	Chr04_54513 C_CA	INDEL	12424.59	33	[illegible]
Chr04	5451302	C	CA	Chr04_54513 C_CA	INDEL	12424.59	33	[illegible]
Chr04	5451302	C	CA	Chr04_54513 C_CA	INDEL	12424.59	33	[illegible]
Chr04	5451332	T	G	Chr04_54513 T_G	SNP	21126.63	35	[illegible]
Chr04	5451332	T	G	Chr04_54513 T_G	SNP	21126.63	35	[illegible]
Chr04	5451332	T	G	Chr04_54513 T_G	SNP	21126.63	35	[illegible]
Chr04	5451332	T	G	Chr04_54513 T_G	SNP	21126.63	35	[illegible]
Chr04	5451332	T	G	Chr04_54513 T_G	SNP	21126.63	35	[illegible]
Chr04	5451332	T	G	Chr04_54513 T_G	SNP	21126.63	35	[illegible]
Chr04	5451332	T	G	Chr04_54513 T_G	SNP	21126.63	35	[illegible]
Chr04	5451333	G	A	Chr04_54513 G_A	SNP	21126.63	35	[illegible]
Chr04	5451333	G	A	Chr04_54513 G_A	SNP	21126.63	35	[illegible]
Chr04	5451333	G	A	Chr04_54513 G_A	SNP	21126.63	35	[illegible]
Chr04	5451333	G	A	Chr04_54513 G_A	SNP	21126.63	35	[illegible]
Chr04	5451333	G	A	Chr04_54513 G_A	SNP	21126.63	35	[illegible]
Chr04	5451333	G	A	Chr04_54513 G_A	SNP	21126.63	35	[illegible]
Chr04	5451333	G	A	Chr04_54513 G_A	SNP	21126.63	35	[illegible]
Chr04	5451362	A	T	Chr04_54513 A_T	SNP	19607.63	34	[illegible]
Chr04	5451362	A	T	Chr04_54513 A_T	SNP	19607.63	34	[illegible]
Chr04	5451362	A	T	Chr04_54513 A_T	SNP	19607.63	34	[illegible]
Chr04	5451362	A	T	Chr04_54513 A_T	SNP	19607.63	34	[illegible]
Chr04	5451362	A	T	Chr04_54513 A_T	SNP	19607.63	34	[illegible]
Chr04	5451362	A	T	Chr04_54513 A_T	SNP	19607.63	34	[illegible]
Chr04	5451362	A	T	Chr04_54513 A_T	SNP	19607.63	34	[illegible]
Chr04	5465346	G	T	Chr04_54653 G_T	SNP	11879.63	22	[illegible]
Chr04	5465346	G	T	Chr04_54653 G_T	SNP	11879.63	22	[illegible]
Chr04	5465346	G	T	Chr04_54653 G_T	SNP	11879.63	22	[illegible]
Chr04	5465346	G	T	Chr04_54653 G_T	SNP	11879.63	22	[illegible]
Chr04	5465346	G	T	Chr04_54653 G_T	SNP	11879.63	22	[illegible]
Chr04	5465346	G	T	Chr04_54653 G_T	SNP	11879.63	22	[illegible]
Chr04	5465360	G	T	Chr04_54653 G_T	SNP	12758.63	20	[illegible]
Chr04	5465360	G	T	Chr04_54653 G_T	SNP	12758.63	20	[illegible]
Chr04	5465360	G	T	Chr04_54653 G_T	SNP	12758.63	20	[illegible]
Chr04	5465360	G	T	Chr04_54653 G_T	SNP	12758.63	20	[illegible]
Chr04	5465360	G	T	Chr04_54653 G_T	SNP	12758.63	20	[illegible]
Chr04	5465360	G	T	Chr04_54653 G_T	SNP	12758.63	20	[illegible]
Chr04	5465400	G	A	Chr04_54654 G_A	SNP	13667.63	23	[illegible]
Chr04	5465400	G	A	Chr04_54654 G_A	SNP	13667.63	23	[illegible]
Chr04	5465400	G	A	Chr04_54654 G_A	SNP	13667.63	23	[illegible]
Chr04	5465400	G	A	Chr04_54654 G_A	SNP	13667.63	23	[illegible]
Chr04	5465400	G	A	Chr04_54654 G_A	SNP	13667.63	23	[illegible]
Chr04	5465400	G	A	Chr04_54654 G_A	SNP	13667.63	23	[illegible]
Chr04	5471022	A	AAT	Chr04_54710 A_AAT	INDEL	9448.59	21	[illegible]
Chr04	5471022	A	AAT	Chr04_54710 A_AAT	INDEL	9448.59	21	[illegible]
Chr04	5471022	A	AAT	Chr04_54710 A_AAT	INDEL	9448.59	21	[illegible]
Chr04	5471022	A	AAT	Chr04_54710 A_AAT	INDEL	9448.59	21	[illegible]
Chr04	5471022	A	AAT	Chr04_54710 A_AAT	INDEL	9448.59	21	[illegible]
Chr04	5471022	A	AAT	Chr04_54710 A_AAT	INDEL	9448.59	21	[illegible]
Chr04	5471022	A	AAT	Chr04_54710 A_AAT	INDEL	9448.59	21	[illegible]
Chr04	5471022	A	AAT	Chr04_54710 A_AAT	INDEL	9448.59	21	[illegible]
Chr04	5524838	T	TTA	Chr04_55248 T_TTA	INDEL	1359.93	2	[illegible]
Chr04	5524838	T	TTA	Chr04_55248 T_TTA	INDEL	1359.93	2	[illegible]
Chr04	5524838	T	TTA	Chr04_55248 T_TTA	INDEL	1359.93	2	[illegible]
Chr04	5524838	T	TTA	Chr04_55248 T_TTA	INDEL	1359.93	2	[illegible]
Chr04	5740443	G	A	Chr04_57404 G_A	SNP	11280.29	25	[illegible]
Chr04	5740443	G	A	Chr04_57404 G_A	SNP	11280.29	25	[illegible]

Chr04	5740443	G	A	Chr04_57404G_A	SNP	11280.29	25	12	14	0	0	0	0	0	29	21
Chr04	5778402	A	AAT	Chr04_57784A_AAT	INDEL	6724.24	19	9	16	0	0	1	1	1	16	18
Chr04	5793405	T	C	Chr04_57934T_C	SNP	16809.63	24	9	12	21	12	17	36	26	23	31
Chr04	5846211	A	T	Chr04_58462A_T	SNP	24128.63	44	16	23	20	12	35	36	30	30	35
Chr04	5846212	T	A	Chr04_58462T_A	SNP	24128.63	45	16	23	20	12	36	37	30	29	35
Chr04	5849285	G	GA	Chr04_58492G_GA	INDEL	11928.59	22	9	8	17	18	26	36	23	15	26
Chr04	5849285	G	GA	Chr04_58492G_GA	INDEL	11928.59	22	9	8	17	18	26	36	23	15	26
Chr04	5849285	G	GA	Chr04_58492G_GA	INDEL	11928.59	22	9	8	17	18	26	36	23	15	26
Chr04	5849330	G	A	Chr04_58493G_A	SNP	14649.63	18	10	12	14	13	24	39	23	12	26
Chr04	5849330	G	A	Chr04_58493G_A	SNP	14649.63	18	10	12	14	13	24	39	23	12	26
Chr04	5849330	G	A	Chr04_58493G_A	SNP	14649.63	18	10	12	14	13	24	39	23	12	26
Chr04	5858120	G	GATAT	Chr04_58581G_GATAT	INDEL	12633.59	21	10	10	15	8	18	16	16	10	15
Chr04	5858120	G	GATAT	Chr04_58581G_GATAT	INDEL	12633.59	21	10	10	15	8	18	16	16	10	15
Chr04	5858120	G	GATAT	Chr04_58581G_GATAT	INDEL	12633.59	21	10	10	15	8	18	16	16	10	15
Chr04	5871730	A	AC	Chr04_58717A_AC	INDEL	20710.59	24	13	18	25	14	33	21	27	14	18
Chr04	5871730	A	AC	Chr04_58717A_AC	INDEL	20710.59	24	13	18	25	14	33	21	27	14	18
Chr04	5871730	A	AC	Chr04_58717A_AC	INDEL	20710.59	24	13	18	25	14	33	21	27	14	18
Chr04	5871730	A	AC	Chr04_58717A_AC	INDEL	20710.59	24	13	18	25	14	33	21	27	14	18
Chr04	5871730	A	AC	Chr04_58717A_AC	INDEL	20710.59	24	13	18	25	14	33	21	27	14	18
Chr04	5871730	A	AC	Chr04_58717A_AC	INDEL	20710.59	24	13	18	25	14	33	21	27	14	18
Chr04	5871730	A	AC	Chr04_58717A_AC	INDEL	20710.59	24	13	18	25	14	33	21	27	14	18
Chr04	5871730	A	AC	Chr04_58717A_AC	INDEL	20710.59	24	13	18	25	14	33	21	27	14	18
Chr04	5871730	A	AC	Chr04_58717A_AC	INDEL	20710.59	24	13	18	25	14	33	21	27	14	18

Chr04	9315885	T	G	Chr04_93158T_G	SNP	9488.63	18	6	10	8	4	13	13	10	11	14	4	6	6	5	[illegible]
Chr04	9315885	T	G	Chr04_93158T_G	SNP	9488.63	18	6	10	8	4	13	13	10	11	14	4	6	6	5	[illegible]
Chr04	9315885	T	G	Chr04_93158T_G	SNP	9488.63	18	6	10	8	4	13	13	10	11	14	4	6	6	5	[illegible]
Chr04	9315885	T	G	Chr04_93158T_G	SNP	9488.63	18	6	10	8	4	13	13	10	11	14	4	6	6	5	[illegible]
Chr04	9315885	T	G	Chr04_93158T_G	SNP	9488.63	18	6	10	8	4	13	13	10	11	14	4	6	6	5	[illegible]
Chr04	9315885	T	G	Chr04_93158T_G	SNP	9488.63	18	6	10	8	4	13	13	10	11	14	4	6	6	5	[illegible]
Chr04	9315885	T	G	Chr04_93158T_G	SNP	9488.63	18	6	10	8	4	13	13	10	11	14	4	6	6	5	[illegible]
Chr04	9315885	T	G	Chr04_93158T_G	SNP	9488.63	18	6	10	8	4	13	13	10	11	14	4	6	6	5	[illegible]
Chr04	9315885	T	G	Chr04_93158T_G	SNP	9488.63	18	6	10	8	4	13	13	10	11	14	4	6	6	5	[illegible]
Chr04	9315885	T	G	Chr04_93158T_G	SNP	9488.63	18	6	10	8	4	13	13	10	11	14	4	6	6	5	[illegible]
Chr04	9315885	T	G	Chr04_93158T_G	SNP	9488.63	18	6	10	8	4	13	13	10	11	14	4	6	6	5	[illegible]
Chr04	9315885	T	G	Chr04_93158T_G	SNP	9488.63	18	6	10	8	4	13	13	10	11	14	4	6	6	5	[illegible]
Chr04	9315886	A	AGGAGGGG	Chr04_93158A_AGGAGG	INDEL	9570.59	17	6	10	7	4	13	13	10	11	14	4	5	6	4	[illegible]
Chr04	9315886	A	AGGAGGGG	Chr04_93158A_AGGAGG	INDEL	9570.59	17	6	10	7	4	13	13	10	11	14	4	5	6	4	[illegible]
Chr04	9315886	A	AGGAGGGG	Chr04_93158A_AGGAGG	INDEL	9570.59	17	6	10	7	4	13	13	10	11	14	4	5	6	4	[illegible]
Chr04	9315886	A	AGGAGGGG	Chr04_93158A_AGGAGG	INDEL	9570.59	17	6	10	7	4	13	13	10	11	14	4	5	6	4	[illegible]
Chr04	9315886	A	AGGAGGGG	Chr04_93158A_AGGAGG	INDEL	9570.59	17	6	10	7	4	13	13	10	11	14	4	5	6	4	[illegible]
Chr04	9315886	A	AGGAGGGG	Chr04_93158A_AGGAGG	INDEL	9570.59	17	6	10	7	4	13	13	10	11	14	4	5	6	4	[illegible]
Chr04	9315886	A	AGGAGGGG	Chr04_93158A_AGGAGG	INDEL	9570.59	17	6	10	7	4	13	13	10	11	14	4	5	6	4	[illegible]
Chr04	9315886	A	AGGAGGGG	Chr04_93158A_AGGAGG	INDEL	9570.59	17	6	10	7	4	13	13	10	11	14	4	5	6	4	[illegible]
Chr04	9315886	A	AGGAGGGG	Chr04_93158A_AGGAGG	INDEL	9570.59	17	6	10	7	4	13	13	10	11	14	4	5	6	4	[illegible]
Chr04	9315886	A	AGGAGGGG	Chr04_93158A_AGGAGG	INDEL	9570.59	17	6	10	7	4	13	13	10	11	14	4	5	6	4	[illegible]
Chr04	9315886	A	AGGAGGGG	Chr04_93158A_AGGAGG	INDEL	9570.59	17	6	10	7	4	13	13	10	11	14	4	5	6	4	[illegible]
Chr04	9315886	A	AGGAGGGG	Chr04_93158A_AGGAGG	INDEL	9570.59	17	6	10	7	4	13	13	10	11	14	4	5	6	4	[illegible]
[illegible]	[illegible]	[illegible]	[illegible]	[illegible]	[illegible]	[illegible]	[illegible]	[illegible]	[illegible]	[illegible]	[illegible]	[illegible]	[illegible]	[illegible]	[illegible]	[illegible]	[illegible]	[illegible]	[illegible]	[illegible]	[illegible]

Chr04	11168413	A	AT	Chr04_11168A_AT	INDEL	17093.59	29	17	19	27	19	29	40	19	29	15	16	18	11	7
Chr04	11168413	A	AT	Chr04_11168A_AT	INDEL	17093.59	29	17	19	27	19	29	40	19	29	15	16	18	11	7
Chr04	11168413	A	AT	Chr04_11168A_AT	INDEL	17093.59	29	17	19	27	19	29	40	19	29	15	16	18	11	7
Chr04	11168413	A	AT	Chr04_11168A_AT	INDEL	17093.59	29	17	19	27	19	29	40	19	29	15	16	18	11	7
Chr04	11168413	A	AT	Chr04_11168A_AT	INDEL	17093.59	29	17	19	27	19	29	40	19	29	15	16	18	11	7
Chr04	11201906	G	A	Chr04_11201G_A	SNP	16219.63	28	18	15	16	11	24	31	21	21	23	12	11	11	7
Chr04	11223098	TTA	T	Chr04_11223TTA_T	INDEL	10341.59	19	7	5	16	19	16	23	23	12	11	7	15	9	7
Chr04	11223098	TTA	T	Chr04_11223TTA_T	INDEL	10341.59	19	7	5	16	19	16	23	23	12	11	7	15	9	7
Chr04	11223098	TTA	T	Chr04_11223TTA_T	INDEL	10341.59	19	7	5	16	19	16	23	23	12	11	7	15	9	7
Chr04	11289159	TTA	T	Chr04_11289TTA_T	INDEL	6670.59	17	12	6	13	18	13	18	14	8	11	6	7	9	5
Chr04	11289159	TTA	T	Chr04_11289TTA_T	INDEL	6670.59	17	12	6	13	18	13	18	14	8	11	6	7	9	5
Chr04	11289159	TTA	T	Chr04_11289TTA_T	INDEL	6670.59	17	12	6	13	18	13	18	14	8	11	6	7	9	5
Chr04	11303237	A	G	Chr04_11303A_G	SNP	19303.63	21	9	18	24	22	32	25	22	23	27	16	19	10	10
Chr04	11303267	TG	T	Chr04_11303TG_T	INDEL	20025.59	21	12	18	20	18	27	22	26	17	26	17	20	10	9
Chr04	11303288	AG	A	Chr04_11303AG_A	INDEL	20857.59	20	12	20	18	20	31	25	26	25	30	18	22	13	8
Chr04	11360788	G	C	Chr04_11360G_C	SNP	17509.63	31	16	9	24	13	28	30	25	14	18	8	21	7	9
Chr04	11360788	G	C	Chr04_11360G_C	SNP	17509.63	31	16	9	24	13	28	30	25	14	18	8	21	7	9
Chr04	11360788	G	C	Chr04_11360G_C	SNP	17509.63	31	16	9	24	13	28	30	25	14	18	8	21	7	9
Chr04	11360788	G	C	Chr04_11360G_C	SNP	17509.63	31	16	9	24	13	28	30	25	14	18	8	21	7	9
Chr04	11360788	G	C	Chr04_11360G_C	SNP	17509.63	31	16	9	24	13	28	30	25	14	18	8	21	7	9
Chr04	11360788	G	C	Chr04_11360G_C	SNP	17509.63	31	16	9	24	13	28	30	25	14	18	8	21	7	9
Chr04	11360829	G	C	Chr04_11360G_C	SNP	18103.63	26	15	8	22	11	32	38	19	13	16	9	20	6	9
Chr04	11360829	G	C	Chr04_11360G_C	SNP	18103.63	26	15	8	22	11	32	38	19	13	16	9	20	6	9
Chr04	11360829	G	C	Chr04_11360G_C	SNP	18103.63	26	15	8	22	11	32	38	19	13	16	9	20	6	9
Chr04	11360829	G	C	Chr04_11360G_C	SNP	18103.63	26	15	8	22	11	32	38	19	13	16	9	20	6	9
Chr04	11360829	G	C	Chr04_11360G_C	SNP	18103.63	26	15	8	22	11	32	38	19	13	16	9	20	6	9
Chr04	11360829	G	C	Chr04_11360G_C	SNP	18103.63	26	15	8	22	11	32	38	19	13	16	9	20	6	9
Chr04	11421277	A	T	Chr04_11421A_T	SNP	15575.63	15	10	10	35	14	30	14	20	24	19	9	7	10	8
Chr04	11421277	A	T	Chr04_11421A_T	SNP	15575.63	15	10	10	35	14	30	14	20	24	19	9	7	10	8
Chr04	11455249	T	TTA	Chr04_11455T_TTA	INDEL	284.37	0	2	0	0	0	1	0	1	0	0	0	0	0	10
Chr04	11455249	T	TTA	Chr04_11455T_TTA	INDEL	284.37	0	2	0	0	0	1	0	1	0	0	0	0	0	10

Chr04	14949021	G	A	Chr04_14949G_A	SNP	19093.63	32	20	12	20	20	14	[illegible]
Chr04	14949021	G	A	Chr04_14949G_A	SNP	19093.63	32	20	12	20	20	14	[illegible]
Chr04	15115968	G	T	Chr04_15115G_T	SNP	16587.63	23	14	19	21	16	26	[illegible]
Chr04	15119198	T	TGGCC	Chr04_15119T_TGGCC	INDEL	20102.59	24	10	11	28	21	25	[illegible]
Chr04	15119201	T	C	Chr04_15119T_C	SNP	19955.63	25	11	10	28	22	25	[illegible]
Chr04	15119204	CCAGCA	C	Chr04_15119CCAGCA_C	INDEL	20300.59	26	11	8	29	22	25	[illegible]
Chr04	15119210	C	CTT	Chr04_15119C_CTT	INDEL	19895.59	26	11	7	30	22	25	[illegible]
Chr04	15149798	CG	C	Chr04_15149CG_C	INDEL	12891.59	17	7	10	21	11	11	[illegible]
Chr04	15149822	G	C	Chr04_15149G_C	SNP	13842.63	17	8	10	25	11	11	[illegible]
Chr04	15149832	G	C	Chr04_15149G_C	SNP	14060.63	18	7	11	24	13	11	[illegible]
Chr04	15149841	G	A	Chr04_15149G_A	SNP	13809.63	21	9	11	22	13	10	[illegible]
Chr04	15149855	G	C	Chr04_15149G_C	SNP	14626.63	24	10	12	29	13	11	[illegible]
Chr04	15150919	C	T	Chr04_15150C_T	SNP	16087.63	16	15	15	25	15	17	[illegible]
Chr04	15150919	C	T	Chr04_15150C_T	SNP	16087.63	16	15	15	25	15	17	[illegible]
Chr04	15150919	C	T	Chr04_15150C_T	SNP	16087.63	16	15	15	25	15	17	[illegible]
Chr04	15163125	G	GAT	Chr04_15163G_GAT	INDEL	3132.24	0	0	0	18	13	13	[illegible]
Chr04	15163125	G	GAT	Chr04_15163G_GAT	INDEL	3132.24	0	0	0	18	13	13	[illegible]
Chr04	15163125	G	GAT	Chr04_15163G_GAT	INDEL	3132.24	0	0	0	18	13	13	[illegible]
Chr04	15223522	A	G	Chr04_15223A_G	SNP	22648.63	51	22	34	39	28	37	[illegible]
Chr04	15544700	A	C	Chr04_15544A_C	SNP	16201.63	27	15	10	12	17	21	[illegible]
Chr04	15544700	A	C	Chr04_15544A_C	SNP	16201.63	27	15	10	12	17	21	[illegible]
Chr04	15544701	C	A	Chr04_15544C_A	SNP	16201.63	28	15	10	12	15	21	[illegible]
Chr04	15544701	C	A	Chr04_15544C_A	SNP	16201.63	28	15	10	12	15	21	[illegible]
Chr04	15544728	C	CA	Chr04_15544C_CA	INDEL	14577.59	26	15	7	14	16	19	[illegible]
Chr04	15544728	C	CA	Chr04_15544C_CA	INDEL	14577.59	26	15	7	14	16	19	[illegible]
Chr04	15730951	AT	A	Chr04_15730AT_A	INDEL	14065.59	30	11	11	20	16	26	[illegible]
Chr04	15793349	G	T	Chr04_15793G_T	SNP	18467.63	17	14	15	24	26	19	[illegible]
Chr04	15793349	G	T	Chr04_15793G_T	SNP	18467.63	17	14	15	24	26	19	[illegible]
Chr04	15793349	G	T	Chr04_15793G_T	SNP	18467.63	17	14	15	24	26	19	[illegible]
Chr04	15793349	G	T	Chr04_15793G_T	SNP	18467.63	17	14	15	24	26	19	[illegible]
Chr04	15793349	G	T	Chr04_15793G_T	SNP	18467.63	17	14	15	24	26	19	[illegible]
Chr04	15793350	G	T	Chr04_15793G_T	SNP	18467.63	17	14	15	24	26	20	[illegible]
Chr04	15793350	G	T	Chr04_15793G_T	SNP	18467.63	17	14	15	24	26	20	[illegible]
Chr04	15793350	G	T	Chr04_15793G_T	SNP	18467.63	17	14	15	24	26	20	[illegible]
Chr04	15793350	G	T	Chr04_15793G_T	SNP	18467.63	17	14	15	24	26	20	[illegible]
Chr04	15793350	G	T	Chr04_15793G_T	SNP	18467.63	17	14	15	24	26	20	[illegible]
Chr04	16095914	C	T	Chr04_16095C_T	SNP	10852.63	16	11	6	28	15	14	[illegible]
Chr04	16095914	C	T	Chr04_16095C_T	SNP	10852.63	16	11	6	28	15	14	[illegible]
Chr04	16095914	C	T	Chr04_16095C_T	SNP	10852.63	16	11	6	28	15	14	[illegible]
Chr04	16095914	C	T	Chr04_16095C_T	SNP	10852.63	16	11	6	28	15	14	[illegible]
Chr04	16095914	C	T	Chr04_16095C_T	SNP	10852.63	16	11	6	28	15	14	[illegible]
Chr04	16095914	C	T	Chr04_16095C_T	SNP	10852.63	16	11	6	28	15	14	[illegible]
Chr04	16095914	C	T	Chr04_16095C_T	SNP	10852.63	16	11	6	28	15	14	[illegible]
Chr04	16253840	G	A	Chr04_16253G_A	SNP	5983.29	0	0	0	22	18	23	[illegible]
Chr04	16650741	AAT	A	Chr04_16650AAT_A	INDEL	8717.59	22	15	14	19	7	17	[illegible]
Chr04	16650741	AAT	A	Chr04_16650AAT_A	INDEL	8717.59	22	15	14	19	7	17	[illegible]
Chr04	16650741	AAT	A	Chr04_16650AAT_A	INDEL	8717.59	22	15	14	19	7	17	[illegible]
Chr04	16650741	AAT	A	Chr04_16650AAT_A	INDEL	8717.59	22	15	14	19	7	17	[illegible]
Chr04	16650741	AAT	A	Chr04_16650AAT_A	INDEL	8717.59	22	15	14	19	7	17	[illegible]
Chr04	16650741	AAT	A	Chr04_16650AAT_A	INDEL	8717.59	22	15	14	19	7	17	[illegible]
Chr04	16650741	AAT	A	Chr04_16650AAT_A	INDEL	8717.59	22	15	14	19	7	17	[illegible]
Chr04	16650741	AAT	A	Chr04_16650AAT_A	INDEL	8717.59	22	15	14	19	7	17	[illegible]
Chr04	16650741	AAT	A	Chr04_16650AAT_A	INDEL	8717.59	22	15	14	19	7	17	[illegible]
Chr04	16650741	AAT	A	Chr04_16650AAT_A	INDEL	8717.59	22	15	14	19	7	17	[illegible]
Chr04	16763217	A	AATAT	Chr04_16763A_AATAT	INDEL	13208.59	27	8	11	18	15	17	[illegible]
Chr04	16763217	A	AATAT	Chr04_16763A_AATAT	INDEL	13208.59	27	8	11	18	15	17	[illegible]
Chr04	16763217	A	AATAT	Chr04_16763A_AATAT	INDEL	13208.59	27	8	11	18	15	17	[illegible]
Chr04	16848116	G	GAT	Chr04_16848G_GAT	INDEL	1605.93	1	0	1	1	0	1	[illegible]
Chr04	16848116	G	GAT	Chr04_16848G_GAT	INDEL	1605.93	1	0	1	1	0	1	[illegible]
Chr04	16848116	G	GAT	Chr04_16848G_GAT	INDEL	1605.93	1	0	1	1	0	1	[illegible]
Chr04	16848116	G	GAT	Chr04_16848G_GAT	INDEL	1605.93	1	0	1	1	0	1	[illegible]
Chr04	16848116	G	GAT	Chr04_16848G_GAT	INDEL	1605.93	1	0	1	1	0	1	[illegible]
Chr04	16848116	G	GAT	Chr04_16848G_GAT	INDEL	1605.93	1	0	1	1	0	1	[illegible]
Chr04	17063609	T	TA	Chr04_17063T_TA	INDEL	14650.59	23	11	11	22	20	23	[illegible]
Chr04	17096211	T	C	Chr04_17096T_C	SNP	17373.63	25	11	15	27	21	19	[illegible]
Chr04	17097338	G	A	Chr04_17097G_A	SNP	3708.98	0	0	0	0	0	0	[illegible]
Chr04	17122013	A	AAT	Chr04_17122A_AAT	INDEL	2366.24	0	0	0	11	9	19	[illegible]
Chr04	17125394	T	A	Chr04_17125T_A	SNP	13818.63	20	6	16	19	21	19	[illegible]
Chr04	17166323	GAT	G	Chr04_17166GAT_G	INDEL	2466.93	0	0	0	1	0	0	[illegible]
Chr04	17166323	GAT	G	Chr04_17166GAT_G	INDEL	2466.93	0	0	0	1	0	0	[illegible]
Chr04	17166323	GAT	G	Chr04_17166GAT_G	INDEL	2466.93	0	0	0	1	0	0	[illegible]
Chr04	17166323	GAT	G	Chr04_17166GAT_G	INDEL	2466.93	0	0	0	1	0	0	[illegible]
Chr04	17166323	GAT	G	Chr04_17166GAT_G	INDEL	2466.93	0	0	0	1	0	0	[illegible]
Chr04	17166323	GAT	G	Chr04_17166GAT_G	INDEL	2466.93	0	0	0	1	0	0	[illegible]
Chr04	17301677	C	CA	Chr04_17301C_CA	INDEL	15566.59	18	9	10	24	12	25	[illegible]
Chr04	17375413	T	TA	Chr04_17375T_TA	INDEL	10686.59	32	8	13	23	10	27	[illegible]
Chr04	17375413	T	TA	Chr04_17375T_TA	INDEL	10686.59	32	8	13	23	10	27	[illegible]
Chr04	17375413	T	TA	Chr04_17375T_TA	INDEL	10686.59	32	8	13	23	10	27	[illegible]
Chr04	17809625	C	T	Chr04_17809C_T	SNP	5322.29	0	0	0	22	15	22	[illegible]
Chr04	17928152	CAT	C	Chr04_17928CAT_C	INDEL	11086.59	27	11	13	23	16	25	[illegible]
Chr04	17928152	CAT	C	Chr04_17928CAT_C	INDEL	11086.59	27	11	13	23	16	25	[illegible]
Chr04	17928152	CAT	C	Chr04_17928CAT_C	INDEL	11086.59	27	11	13	23	16	25	[illegible]
Chr04	18012094	G	T	Chr04_18012G_T	SNP	12292.63	19	10	16	10	8	25	[illegible]
Chr04	18041324	C	T	Chr04_18041C_T	SNP	17971.63	26	12	18	32	15	25	[illegible]
Chr04	18378160	C	A	Chr04_18378C_A	SNP	4027.98	0	0	0	0	0	0	[illegible]
Chr04	18456827	A	G	Chr04_18456A_G	SNP	17854.63	27	14	15	27	16	22	[illegible]
Chr04	18583717	G	A	Chr04_18583G_A	SNP	23275.63	43	22	19	26	20	35	[illegible]
Chr04	18583823	T	TA	Chr04_18583T_TA	INDEL	22777.59	36	18	13	25	24	23	[illegible]
Chr04	18583847	C	A	Chr04_18583C_A	SNP	23698.63	34	18	14	27	23	25	[illegible]
Chr04	18712806	G	C	Chr04_18712G_C	SNP	19542.63	22	14	11	20	16	29	[illegible]
Chr04	18712816	GA	G	Chr04_18712GA_G	INDEL	19587.59	24	12	12	18	16	33	[illegible]
Chr04	18750324	A	T	Chr04_18750A_T	SNP	4419.29	0	0	0	29	19	15	[illegible]
Chr04	18750324	A	T	Chr04_18750A_T	SNP	4419.29	0	0	0	29	19	15	[illegible]
Chr04	18750324	A	T	Chr04_18750A_T	SNP	4419.29	0	0	0	29	19	15	[illegible]
Chr04	19253594	T	C	Chr04_19253T_C	SNP	11065.63	16	8	12	20	6	20	[illegible]
Chr04	19255097	A	T	Chr04_19255A_T	SNP	11070.63	15	4	13	17	5	10	[illegible]
Chr04	19361531	T	A	Chr04_19361T_A	SNP	18020.63	29	13	14	19	31	17	[illegible]
Chr04	19466098	C	CTA	Chr04_19466C_CTA	INDEL	7044.59	24	8	7	26	10	11	[illegible]
Chr04	19466098	C	CTA	Chr04_19466C_CTA	INDEL	7044.59	24	8	7	26	10	11	[illegible]
Chr04	19507718	T	A	Chr04_19507T_A	SNP	19767.63	27	15	22	24	29	29	[illegible]
Chr04	19507764	A	T	Chr04_19507A_T	SNP	19597.63	25	12	19	22	26	28	[illegible]
Chr04	19507766	T	A	Chr04_19507T_A	SNP	19597.63	25	12	19	23	27	28	[illegible]
Chr04	19514902	C	G	Chr04_19514C_G	SNP	18905.63	23	19	19	36	13	21	[illegible]
Chr04	19514903	AC	A	Chr04_19514AC_A	INDEL	18896.59	22	19	19	35	13	21	[illegible]
Chr04	19540255	C	CT	Chr04_19540C_CT	INDEL	16569.59	27	14	16	32	18	23	[illegible]
Chr04	19540255	C	CT	Chr04_19540C_CT	INDEL	16569.59	27	14	16	32	18	23	[illegible]
Chr04	19540284	C	A	Chr04_19540C_A	SNP	21182.63	31	12	14	35	14	24	[illegible]
Chr04	19540284	C	A	Chr04_19540C_A	SNP	21182.63	31	12	14	35	14	24	[illegible]
Chr04	19554063	T	G	Chr04_19554T_G	SNP	14571.63	22	13	18	14	10	25	[illegible]
Chr04	19554063	T	G	Chr04_19554T_G	SNP	14571.63	22	13	18	14	10	25	[illegible]
Chr04	19554063	T	G	Chr04_19554T_G	SNP	14571.63	22	13	18	14	10	25	[illegible]
Chr04	19554228	A	T	Chr04_19554A_T	SNP	12222.63	26	15	12	12	10	20	[illegible]
Chr04	19554228	A	T	Chr04_19554A_T	SNP	12222.63	26	15	12	12	10	20	[illegible]
Chr04	19554228	A	T	Chr04_19554A_T	SNP	12222.63	26	15	12	12	10	20	[illegible]
Chr04	19602870	A	T	Chr04_19602A_T	SNP	10931.63	15	13	8	12	18	11	[illegible]
Chr04	19602898	A	G	Chr04_19602A_G	SNP	15200.63	18	14	8	17	20	12	[illegible]
Chr04	19602901	C	A	Chr04_19602C_A	SNP	15385.63	19	14	8	17	20	13	[illegible]
Chr04	19602995	T	G	Chr04_19602T_G	SNP	18899.63	21	16	17	23	21	24	[illegible]
Chr04	19690143	C	CT	Chr04_19690C_CT	INDEL	9781.59	15	11	9	18	10	16	[illegible]
Chr04	19690143	C	CT	Chr04_19690C_CT	INDEL	9781.59	15	11	9	18	10	16	[illegible]
Chr04	19896378	A	G	Chr04_19896A_G	SNP	13775.63	19	12	8	17	12	15	[illegible]
Chr04	19931608	G	A	Chr04_19931G_A	SNP	14162.63	16	16	12	12	15	13	[illegible]
Chr04	19980468	TATATATATA	T	Chr04_19980TATATATATA	INDEL	279934.59	521	409	405	499	438	639	[illegible]
Chr04	19980468	TATATATATA	T	Chr04_19980TATATATATA	INDEL	279934.59	521	409	405	499	438	639	[illegible]
Chr04	19980468	TATATATATA	T	Chr04_19980TATATATATA	INDEL	279934.59	521	409	405	499	438	639	[illegible]
Chr04	19980468	TATATATATA	T	Chr04_19980TATATATATA	INDEL	279934.59	521	409	405	499	438	639	[illegible]
Chr04	19980468	TATATATATA	T	Chr04_19980TATATATATA	INDEL	279934.59	521	409	405	499	438	639	[illegible]
Chr04	19980491	TC	T	Chr04_19980TC_T	INDEL	284781.59	662	489	513	604	554	775	[illegible]
Chr04	19980491	TC	T	Chr04_19980TC_T	INDEL	284781.59	662	489	513	604	554	775	[illegible]
Chr04	19980491	TC	T	Chr04_19980TC_T	INDEL	284781.59	662	489	513	604	554	775	[illegible]
Chr04	19980491	TC	T	Chr04_19980TC_T	INDEL	284781.59	662	489	513	604	554	775	[illegible]
Chr04	19980491	TC	T	Chr04_19980TC_T	INDEL	284781.59	662	489	513	604	554	775	[illegible]
Chr04	19980779	C	T	Chr04_19980C_T	SNP	452713.63	606	343	455	529	512	727	[illegible]
Chr04	19980779	C	T	Chr04_19980C_T	SNP	452713.63	606	343	455	529	512	727	[illegible]
Chr04	19980779	C	T	Chr04_19980C_T	SNP	452713.63	606	343	455	529	512	727	[illegible]
Chr04	19980779	C	T	Chr04_19980C_T	SNP	452713.63	606	343	455	529	512	727	[illegible]
Chr04	19980779	C	T	Chr04_19980C_T	SNP	452713.63	606	343	455	529	512	727	[illegible]
Chr04	20003664	C	T	Chr04_20003C_T	SNP	11347.63	14	13	9	26	11	9	[illegible]
Chr04	20003664	C	T	Chr04_20003C_T	SNP	11347.63	14	13	9	26	11	9	[illegible]
Chr04	20003664	C	T	Chr04_20003C_T	SNP	11347.63	14	13	9	26	11	9	[illegible]
Chr04	20078257	G	GT	Chr04_20078G_GT	INDEL	11568.59	29	6	10	14	30	15	[illegible]
Chr04	20078257	G	GT	Chr04_20078G_GT	INDEL	11568.59	29	6	10	14	30	15	[illegible]
Chr04	20078257	G	GT	Chr04_20078G_GT	INDEL	11568.59	29	6	10	14	30	15	[illegible]
Chr04	20078257	G	GT	Chr04_20078G_GT	INDEL	11568.59	29	6	10	14	30	15	[illegible]
Chr04	20078257	G	GT	Chr04_20078G_GT	INDEL	11568.59	29	6	10	14	30	15	[illegible]
Chr04	20082861	T	A	Chr04_20082T_A	SNP	20194.63	27	13	19	32	26	31	[illegible]
Chr04	20082908	C	A	Chr04_20082C_A	SNP	20198.63	28	13	18	28	23	27	[illegible]
Chr04	20084804	A	AT	Chr04_20084A_AT	INDEL	18941.59	33	19	25	23	25	24	[illegible]
Chr04	20151434	C	CA	Chr04_20151C_CA	INDEL	16507.59	28	14	25	24	14	29	[illegible]
Chr04	20151434	C	CA	Chr04_20151C_CA	INDEL	16507.59	28	14	25	24	14	29	[illegible]
Chr04	20151434	C	CA	Chr04_20151C_CA	INDEL	16507.59	28	14	25	24	14	29	[illegible]
Chr04	20225338	GTCTTATATG	G	Chr04_20225GTCTTATATG	INDEL	22871.59	48	17	16	28	21	30	[illegible]
Chr04	20225338	GTCTTATATG	G	Chr04_20225GTCTTATATG	INDEL	22871.59	48	17	16	28	21	30	[illegible]
Chr04	20225338	GTCTTATATG	G	Chr04_20225GTCTTATATG	INDEL	22871.59	48	17	16	28	21	30	[illegible]
Chr04	20225338	GTCTTATATG	G	Chr04_20225GTCTTATATG	INDEL	22871.59	48	17	16	28	21	30	[illegible]
Chr04	20229388	TTATTGGCTC	T	Chr04_20229TTATTGGCTC	INDEL	24690.59	25	10	24	29	21	33	[illegible]
Chr04	20229388	TTATTGGCTC	T	Chr04_20229TTATTGGCTC	INDEL	24690.59	25	10	24	29	21	33	[illegible]
Chr04	20229388	TTATTGGCTC	T	Chr04_20229TTATTGGCTC	INDEL	24690.59	25	10	24	29	21	33	[illegible]
Chr04	20229388	TTATTGGCTC	T	Chr04_20229TTATTGGCTC	INDEL	24690.59	25	10	24	29	21	33	[illegible]
Chr04	20257767	A	T	Chr04_20257A_T	SNP	17874.63	33	8	18	31	15	21	[illegible]
Chr04	20257772	A	T	Chr04_20257A_T	SNP	17671.63	33	8	18	29	18	22	[illegible]
Chr04	20259226	G	GA	Chr04_20259G_GA	INDEL	13481.59	30	12	10	23	20	20	[illegible]
Chr04	20293629	G	C	Chr04_20293G_C	SNP	20863.63	25	18	15	26	13	24	[illegible]
Chr04	20293642	G	T	Chr04_20293G_T	SNP	21254.63	22	17	15	26	15	27	[illegible]
Chr04	20293655	G	A	Chr04_20293G_A	SNP	20992.63	23	21	16	27	16	25	[illegible]
Chr04	20293660	G	T	Chr04_20293G_T	SNP	20700.63	21	21	17	28	17	24	[illegible]
Chr04	20389908	A	AAT	Chr04_20389A_AAT	INDEL	3884.24	0	1	0	24	13	32	[illegible]
Chr04	20389908	A	AAT	Chr04_20389A_AAT	INDEL	3884.24	0	1	0	24	13	32	[illegible]
Chr04	20430959	A	AAT	Chr04_20430A_AAT	INDEL	1446.35	21	12	14	0	0	0	[illegible]
Chr04	20430959	A	AAT	Chr04_20430A_AAT	INDEL	1446.35	21	12	14	0	0	0	[illegible]
Chr04	20430959	A	AAT	Chr04_20430A_AAT	INDEL	1446.35	21	12	14	0	0	0	[illegible]
Chr04	20433860	G	A	Chr04_20433G_A	SNP	14677.63	16	16	13	20	18	23	[illegible]
Chr04	20433860	G	A	Chr04_20433G_A	SNP	14677.63	16	16	13	20	18	23	[illegible]
Chr04	20433860	G	A	Chr04_20433G_A	SNP	14677.63	16	16	13	20	18	23	[illegible]
Chr04	20433860	G	A	Chr04_20433G_A	SNP	14677.63	16	16	13	20	18	23	[illegible]
Chr04	20433860	G	A	Chr04_20433G_A	SNP	14677.63	16	16	13	20	18	23	[illegible]
Chr04	20433860	G	A	Chr04_20433G_A	SNP	14677.63	16	16	13	20	18	23	[illegible]
Chr04	20497113	C	A	Chr04_20497C_A	SNP	17267.63	22	11	13	15	17	43	[illegible]
Chr04	20497113	C	A	Chr04_20497C_A	SNP	17267.63	22	11	13	15	17	43	[illegible]
Chr04	20497113	C	A	Chr04_20497C_A	SNP	17267.63	22	11	13	15	17	43	[illegible]
Chr04	20497114	C	A	Chr04_20497C_A	SNP	17267.63	22	11	13	14	17	43	[illegible]
Chr04	20497114	C	A	Chr04_20497C_A	SNP	17267.63	22	11	13	14	17	43	[illegible]
Chr04	20497114	C	A	Chr04_20497C_A	SNP	17267.63	22	11	13	14	17	43	[illegible]
Chr04	20497149	T	A	Chr04_20497T_A	SNP	17987.63	32	13	16	20	21	44	[illegible]
Chr04	20497149	T	A	Chr04_20497T_A	SNP	17987.63	32	13	16	20	21	44	[illegible]
Chr04	20497149	T	A	Chr04_20497T_A	SNP	17987.63	32	13	16	20	21	44	[illegible]
Chr04	20600605	T	A	Chr04_20600T_A	SNP	11456.63	22	7	5	15	14	12	[illegible]
Chr04	20707314	C	A	Chr04_20707C_A	SNP	17748.63	35	19	14	25	15	28	[illegible]
Chr04	20708605	G	T	Chr04_20708G_T	SNP	19363.63	19	12	21	29	25	28	[illegible]
Chr04	20746205	T	TTA	Chr04_20746T_TTA	INDEL	78.37	1	0	0	0	5	1	[illegible]
Chr04	20894265	T	C	Chr04_20894T_C	SNP	21799.63	39	17	14	30	18	37	[illegible]
Chr04	20894265	T	C	Chr04_20894T_C	SNP	21799.63	39	17	14	30	18	37	[illegible]
Chr04	20894265	T	C	Chr04_20894T_C	SNP	21799.63	39	17	14	30	18	37	[illegible]
Chr04	20894265	T	C	Chr04_20894T_C	SNP	21799.63	39	17	14	30	18	37	[illegible]
Chr04	20894265	T	C	Chr04_20894T_C	SNP	21799.63	39	17	14	30	18	37	[illegible]
Chr04	20894265	T	C	Chr04_20894T_C	SNP	21799.63	39	17	14	30	18	37	[illegible]
Chr04	20894265	T	C	Chr04_20894T_C	SNP	21799.63	39	17	14	30	18	37	[illegible]
Chr04	20894265	T	C	Chr04_20894T_C	SNP	21799.63	39	17	14	30	18	37	[illegible]
Chr04	20894265	T	C	Chr04_20894T_C	SNP	21799.63	39	17	14	30	18	37	[illegible]
Chr04	20894265	T	C	Chr04_20894T_C	SNP	21799.63	39	17	14	30	18	37	[illegible]
Chr04	20894265	T	C	Chr04_20894T_C	SNP	21799.63	39	17	14	30	18	37	[illegible]
Chr04	20894265	T	C	Chr04_20894T_C	SNP	21799.63	39	17	14	30	18	37	[illegible]

Chr04	20894265	T	C	Chr04_20894T_C	SNP	21799.63	39	17	14	30	18	37	38	33	23	36	17	15	18	21
Chr04	20894265	T	C	Chr04_20894T_C	SNP	21799.63	39	17	14	30	18	37	38	33	23	36	17	15	18	21
Chr04	20894265	T	C	Chr04_20894T_C	SNP	21799.63	39	17	14	30	18	37	38	33	23	36	17	15	18	21
Chr04	20938703	A	G	Chr04_20938A_G	SNP	2038.39	24	15	13	0	0	0	0	0	0	0	0	0	0	0
Chr04	21135675	GC	G	Chr04_21135GC_G	INDEL	13031.59	21	16	11	14	20	20	28	16	9	16	14	9	16	11
Chr04	21231463	T	TCTAATACTC	Chr04_21231T_TCTAATAC	INDEL	14565.59	19	9	12	27	15	20	9	18	15	18	7	13	6	11
Chr04	21239483	C	A	Chr04_21239C_A	SNP	12256.63	17	14	6	9	16	14	14	19	13	12	19	10	10	5
Chr04	21241201	TG	T	Chr04_21241TG_T	INDEL	20798.59	31	22	26	23	23	37	34	29	30	18	20	18	20	10
Chr04	21383665	G	A	Chr04_21383G_A	SNP	12283.63	16	20	11	17	17	26	32	14	20	16	13	9	6	7
Chr04	21383665	G	A	Chr04_21383G_A	SNP	12283.63	16	20	11	17	17	26	32	14	20	16	13	9	6	7
Chr04	21383665	G	A	Chr04_21383G_A	SNP	12283.63	16	20	11	17	17	26	32	14	20	16	13	9	6	7
Chr04	21383665	G	A	Chr04_21383G_A	SNP	12283.63	16	20	11	17	17	26	32	14	20	16	13	9	6	7
Chr04	21391234	C	CA	Chr04_21391C_CA	INDEL	12498.59	16	11	10	13	10	17	23	16	10	12	15	17	14	5
Chr04	21391234	C	CA	Chr04_21391C_CA	INDEL	12498.59	16	11	10	13	10	17	23	16	10	12	15	17	14	5
Chr04	21391234	C	CA	Chr04_21391C_CA	INDEL	12498.59	16	11	10	13	10	17	23	16	10	12	15	17	14	5
Chr04	21391236	T	TA	Chr04_21391T_TA	INDEL	12659.59	16	11	10	12	10	17	24	16	10	12	15	17	14	6
Chr04	21391236	T	TA	Chr04_21391T_TA	INDEL	12659.59	16	11	10	12	10	17	24	16	10	12	15	17	14	6
Chr04	21391236	T	TA	Chr04_21391T_TA	INDEL	12659.59	16	11	10	12	10	17	24	16	10	12	15	17	14	6
Chr04	21391245	C	CA	Chr04_21391C_CA	INDEL	11928.59	15	11	9	12	10	17	21	15	10	11	15	15	13	6
Chr04	21391245	C	CA	Chr04_21391C_CA	INDEL	11928.59	15	11	9	12	10	17	21	15	10	11	15	15	13	6
Chr04	21391245	C	CA	Chr04_21391C_CA	INDEL	11928.59	15	11	9	12	10	17	21	15	10	11	15	15	13	6
Chr04	21522269	C	T	Chr04_21522C_T	SNP	15330.63	21	4	13	18	19	16	20	25	16	25	18	13	8	8
Chr04	21522270	C	A	Chr04_21522C_A	SNP	15330.63	21	4	13	18	19	16	20	25	17	25	18	13	8	8
Chr04	21522419	G	T	Chr04_21522G_T	SNP	9122.63	14	9	8	9	11	9	20	8	7	12	5	14	6	8

[illegible]

Chr05	1283330	C	G	Chr05_12833C_G	SNP	3285.98	0	0	0	1	0	0	0	0	21	18	10	13	5	9	24	17	17	23	15	21	21	17	21	18	10	13	5	9	0	0	0
Chr05	1283330	C	G	Chr05_12833C_G	SNP	3285.98	0	0	0	1	0	0	0	0	21	18	10	13	5	9	24	17	17	23	15	21	21	17	21	18	10	13	5	9	0	0	0
Chr05	1438838	T	A	Chr05_14388T_A	SNP	12397.63	20	15	11	11	15	18	20	19	6	8	9	12	7	4	20	15	11	11	15	18	20	19	6	8	9	12	7	4	1	1	1
Chr05	1438838	T	A	Chr05_14388T_A	SNP	12397.63	20	15	11	11	15	18	20	19	6	8	9	12	7	4	20	15	11	11	15	18	20	19	6	8	9	12	7	4	1	1	1
Chr05	1438838	T	A	Chr05_14388T_A	SNP	12397.63	20	15	11	11	15	18	20	19	6	8	9	12	7	4	20	15	11	11	15	18	20	19	6	8	9	12	7	4	1	1	1
Chr05	1438838	T	A	Chr05_14388T_A	SNP	12397.63	20	15	11	11	15	18	20	19	6	8	9	12	7	4	20	15	11	11	15	18	20	19	6	8	9	12	7	4	1	1	1
Chr05	1438838	T	A	Chr05_14388T_A	SNP	12397.63	20	15	11	11	15	18	20	19	6	8	9	12	7	4	20	15	11	11	15	18	20	19	6	8	9	12	7	4	1	1	1
Chr05	1438839	T	A	Chr05_14388T_A	SNP	12442.63	20	15	11	11	15	17	20	19	6	8	9	12	7	4	20	15	11	11	15	17	20	19	6	8	9	12	7	4	1	1	1
Chr05	1438839	T	A	Chr05_14388T_A	SNP	12442.63	20	15	11	11	15	17	20	19	6	8	9	12	7	4	20	15	11	11	15	17	20	19	6	8	9	12	7	4	1	1	1
Chr05	1438839	T	A	Chr05_14388T_A	SNP	12442.63	20	15	11	11	15	17	20	19	6	8	9	12	7	4	20	15	11	11	15	17	20	19	6	8	9	12	7	4	1	1	1
Chr05	1438839	T	A	Chr05_14388T_A	SNP	12442.63	20	15	11	11	15	17	20	19	6	8	9	12	7	4	20	15	11	11	15	17	20	19	6	8	9	12	7	4	1	1	1
Chr05	1438839	T	A	Chr05_14388T_A	SNP	12442.63	20	15	11	11	15	17	20	19	6	8	9	12	7	4	20	15	11	11	15	17	20	19	6	8	9	12	7	4	1	1	1
Chr05	1438853	T	A	Chr05_14388T_A	SNP	12863.63	22	14	13	13	14	18	22	18	10	8	11	13	7	4	22	14	13	13	14	18	22	18	10	8	11	13	7	4	1	1	1
Chr05	1438853	T	A	Chr05_14388T_A	SNP	12863.63	22	14	13	13	14	18	22	18	10	8	11	13	7	4	22	14	13	13	14	18	22	18	10	8	11	13	7	4	1	1	1
Chr05	1438853	T	A	Chr05_14388T_A	SNP	12863.63	22	14	13	13	14	18	22	18	10	8	11	13	7	4	22	14	13	13	14	18	22	18	10	8	11	13	7	4	1	1	1
Chr05	1438853	T	A	Chr05_14388T_A	SNP	12863.63	22	14	13	13	14	18	22	18	10	8	11	13	7	4	22	14	13	13	14	18	22	18	10	8	11	13	7	4	1	1	1
Chr05	1438853	T	A	Chr05_14388T_A	SNP	12863.63	22	14	13	13	14	18	22	18	10	8	11	13	7	4	22	14	13	13	14	18	22	18	10	8	11	13	7	4	1	1	1
Chr05	1439012	T	A	Chr05_14390T_A	SNP	11469.63	18	13	13	9	14	18	20	21	14	10	9	12	6	4	18	14	13	9	14	18	20	21	14	10	9	13	6	4	1	0.929	1
Chr05	1439012	T	A	Chr05_14390T_A	SNP	11469.63	18	13	13	9	14	18	20	21	14	10	9	12	6	4	18	14	13	9	14	18	20	21	14	10	9	13	6	4	1	0.929	1
Chr05	1439012	T	A	Chr05_14390T_A	SNP	11469.63	18	13	13	9	14	18	20	21	14	10	9	12	6	4	18	14	13	9	14	18	20	21	14	10	9	13	6	4	1	0.929	1
Chr05	1439012	T	A	Chr05_14390T_A	SNP	11469.63	18	13	13	9	14	18	20	21	14	10	9	12	6	4	18	14	13	9	14	18	20	21	14	10	9	13	6	4	1	0.929	1
Chr05	1439012	T	A	Chr05_14390T_A	SNP	11469.63	18	13	13	9	14	18	20	21	14	10	9	12	6	4	18	14	13	9	14	18	20	21	14	10	9	13	6	4	1	0.929	1
Chr05	1439012	T	A	Chr05_14390T_A	SNP	11469.63	18	13	13	9	14	18	20	21	14	10	9	12	6	4	18	14	13	9	14	18	20	21	14	10	9	13	6	4	1	0.929	1
Chr05	1439106	A	G	Chr05_14391A_G	SNP	10762.63	16	12	7	8	8	12	21	14	24	12	11	8	6	5	16	12	7	8	8	12	21	14	24	12	11	8	6	5	1	1	1
Chr05	1439106	A	G	Chr05_14391A_G	SNP	10762.63	16	12	7	8	8	12	21	14	24	12	11	8	6	5	16	12	7	8	8	12	21	14	24	12	11	8	6	5	1	1	1
Chr05	1439106	A	G	Chr05_14391A_G	SNP	10762.63	16	12	7	8	8	12	21	14	24	12	11	8	6	5	16	12	7	8	8	12	21	14	24	12	11	8	6	5	1	1	1
Chr05	1439106	A	G	Chr05_14391A_G	SNP	10762.63	16	12	7	8	8	12	21	14	24	12	11	8	6	5	16	12	7	8	8	12	21	14	24	12	11	8	6	5	1	1	1
Chr05	1439106	A	G	Chr05_14391A_G	SNP	10762.63	16	12	7	8	8	12	21	14	24	12	11	8	6	5	16	12	7	8	8	12	21	14	24	12	11	8	6	5	1	1	1
Chr05	1439106	A	G	Chr05_14391A_G	SNP	10762.63	16	12	7	8	8	12	21	14	24	12	11	8	6	5	16	12	7	8	8	12	21	14	24	12	11	8	6	5	1	1	1
Chr05	1439156	A	T	Chr05_14391A_T	SNP	11923.63	18	10	9	13	9	19	16	13	17	17	10	8	7	7	18	10	9	13	9	19	16	13	17	17	10	8	7	7	1	1	1
Chr05	1439156	A	T	Chr05_14391A_T	SNP	11923.63	18	10	9	13	9	19	16	13	17	17	10	8	7	7	18	10	9	13	9	19	16	13	17	17	10	8	7	7	1	1	1
Chr05	1439156	A	T	Chr05_14391A_T	SNP	11923.63	18	10	9	13	9	19	16	13	17	17	10	8	7	7	18	10	9	13	9	19	16	13	17	17	10	8	7	7	1	1	1
Chr05	1439156	A	T	Chr05_14391A_T	SNP	11923.63	18	10	9	13	9	19	16	13	17	17	10	8	7	7	18	10	9	13	9	19	16	13	17	17	10	8	7	7	1	1	1
Chr05	1439156	A	T	Chr05_14391A_T	SNP	11923.63	18	10	9	13	9	19	16	13	17	17	10	8	7	7	18	10	9	13	9	19	16	13	17	17	10	8	7	7	1	1	1
Chr05	1439156	A	T	Chr05_14391A_T	SNP	11923.63	18	10	9	13	9	19	16	13	17	17	10	8	7	7	18	10	9	13	9	19	16	13	17	17	10	8	7	7	1	1	1
Chr05	1439243	C	T	Chr05_14392C_T	SNP	12640.63	19	5	10	15	13	20	23	18	21	11	5	7	4	7	19	5	10	15	13	20	23	18	21	11	5	7	4	7	1	1	1

[illegible]

Chr05	5213641	G	T	Chr05_52136G_T	SNP	17337.63	31
Chr05	5215391	TC	T	Chr05_52153TC_T	INDEL	17920.59	19
Chr05	5281111	C	T	Chr05_52811C_T	SNP	14737.63	25
Chr05	5292450	G	A	Chr05_52924G_A	SNP	9221.29	25
Chr05	5490277	T	C	Chr05_54902T_C	SNP	2632.39	34
Chr05	5648440	C	CA	Chr05_56484C_CA	INDEL	10041.59	15
Chr05	5769473	G	GATAT	Chr05_57694G_GATAT	INDEL	3586.24	0
Chr05	5843532	TG	T	Chr05_58435TG_T	INDEL	10647.59	27
Chr05	5924497	G	GA	Chr05_59244G_GA	INDEL	8991.59	14
Chr05	5924497	G	GA	Chr05_59244G_GA	INDEL	8991.59	14
Chr05	5924497	G	GA	Chr05_59244G_GA	INDEL	8991.59	14
Chr05	5924497	G	GA	Chr05_59244G_GA	INDEL	8991.59	14
Chr05	5924497	G	GA	Chr05_59244G_GA	INDEL	8991.59	14
Chr05	6165507	T	TTA	Chr05_61655T_TTA	INDEL	1377.35	26
Chr05	6165507	T	TTA	Chr05_61655T_TTA	INDEL	1377.35	26
Chr05	6165507	T	TTA	Chr05_61655T_TTA	INDEL	1377.35	26
Chr05	6165507	T	TTA	Chr05_61655T_TTA	INDEL	1377.35	26
Chr05	6165507	T	TTA	Chr05_61655T_TTA	INDEL	1377.35	26
Chr05	6165507	T	TTA	Chr05_61655T_TTA	INDEL	1377.35	26
Chr05	6206159	G	C	Chr05_62061G_C	SNP	14935.63	22
Chr05	6225919	TA	T	Chr05_62259TA_T	INDEL	14612.59	37
Chr05	6226069	CA	C	Chr05_62260CA_C	INDEL	10545.59	18
Chr05	6226149	TA	T	Chr05_62261TA_T	INDEL	8701.59	22
Chr05	6226167	TC	T	Chr05_62261TC_T	INDEL	10623.59	23
Chr05	6242176	C	A	Chr05_62421C_A	SNP	20096.63	30
Chr05	6242177	T	A	Chr05_62421T_A	SNP	20096.63	30
Chr05	6457436	C	CAT	Chr05_64574C_CAT	INDEL	6479.24	26
Chr05	6507648	G	C	Chr05_65076G_C	SNP	22629.63	31
Chr05	6507649	C	A	Chr05_65076C_A	SNP	22629.63	31
Chr05	6507713	GC	G	Chr05_65077GC_G	INDEL	21809.59	28
Chr05	6507723	G	GT	Chr05_65077G_GT	INDEL	23093.59	28
Chr05	6507810	G	C	Chr05_65078G_C	SNP	22278.63	29
Chr05	6580883	G	A	Chr05_65808G_A	SNP	4838.29	0
Chr05	6580883	G	A	Chr05_65808G_A	SNP	4838.29	0
Chr05	6580883	G	A	Chr05_65808G_A	SNP	4838.29	0
Chr05	6580883	G	A	Chr05_65808G_A	SNP	4838.29	0
Chr05	6580883	G	A	Chr05_65808G_A	SNP	4838.29	0
Chr05	6580883	G	A	Chr05_65808G_A	SNP	4838.29	0
Chr05	6580883	G	A	Chr05_65808G_A	SNP	4838.29	0
Chr05	6580883	G	A	Chr05_65808G_A	SNP	4838.29	0
Chr05	6580883	G	A	Chr05_65808G_A	SNP	4838.29	0
Chr05	6603949	A	T	Chr05_66039A_T	SNP	22341.63	29
Chr05	6603958	C	A	Chr05_66039C_A	SNP	22501.63	26
Chr05	6604006	A	AT	Chr05_66040A_AT	INDEL	21922.59	27
Chr05	6604011	G	T	Chr05_66040G_T	SNP	22221.63	27
Chr05	6604041	C	T	Chr05_66040C_T	SNP	19801.63	30
Chr05	6605913	G	T	Chr05_66059G_T	SNP	18051.63	21
Chr05	6605935	G	C	Chr05_66059G_C	SNP	17613.63	19
Chr05	6605962	GT	G	Chr05_66059GT_G	INDEL	13241.59	17
Chr05	6606043	C	T	Chr05_66060C_T	SNP	17460.63	22
Chr05	6606049	G	T	Chr05_66060G_T	SNP	17762.63	22
Chr05	6606050	A	T	Chr05_66060A_T	SNP	17675.63	22
Chr05	6873842	T	TTA	Chr05_68738T_TTA	INDEL	10358.59	21
Chr05	6873842	T	TTA	Chr05_68738T_TTA	INDEL	10358.59	21
Chr05	6873842	T	TTA	Chr05_68738T_TTA	INDEL	10358.59	21
Chr05	6987623	C	CAT	Chr05_69876C_CAT	INDEL	3438.01	0
Chr05	6987623	C	CAT	Chr05_69876C_CAT	INDEL	3438.01	0
Chr05	6987623	C	CAT	Chr05_69876C_CAT	INDEL	3438.01	0
Chr05	6987623	C	CAT	Chr05_69876C_CAT	INDEL	3438.01	0
Chr05	6987623	C	CAT	Chr05_69876C_CAT	INDEL	3438.01	0
Chr05	6987623	C	CAT	Chr05_69876C_CAT	INDEL	3438.01	0
Chr05	7046357	A	AT	Chr05_70463A_AT	INDEL	25454.59	46
Chr05	7097350	AG	A	Chr05_70973AG_A	INDEL	14537.59	29
Chr05	7097350	AG	A	Chr05_70973AG_A	INDEL	14537.59	29
Chr05	7097350	AG	A	Chr05_70973AG_A	INDEL	14537.59	29
Chr05	7097502	G	T	Chr05_70975G_T	SNP	11741.63	17
Chr05	7097502	G	T	Chr05_70975G_T	SNP	11741.63	17
Chr05	7097502	G	T	Chr05_70975G_T	SNP	11741.63	17
Chr05	7115825	T	TTATA	Chr05_71158T_TTATA	INDEL	392.37	0
Chr05	7240480	G	A	Chr05_72404G_A	SNP	18570.63	36
Chr05	7240480	G	A	Chr05_72404G_A	SNP	18570.63	36
Chr05	7240480	G	A	Chr05_72404G_A	SNP	18570.63	36
Chr05	7240480	G	A	Chr05_72404G_A	SNP	18570.63	36
Chr05	7247430	T	C	Chr05_72474T_C	SNP	15513.63	29
Chr05	7247430	T	C	Chr05_72474T_C	SNP	15513.63	29
Chr05	7247430	T	C	Chr05_72474T_C	SNP	15513.63	29
Chr05	7247524	TA	T	Chr05_72475TA_T	INDEL	13025.59	34
Chr05	7247524	TA	T	Chr05_72475TA_T	INDEL	13025.59	34
Chr05	7247524	TA	T	Chr05_72475TA_T	INDEL	13025.59	34
Chr05	7251205	T	TTA	Chr05_72512T_TTA	INDEL	2945.24	0
Chr05	7251205	T	TTA	Chr05_72512T_TTA	INDEL	2945.24	0
Chr05	7251205	T	TTA	Chr05_72512T_TTA	INDEL	2945.24	0
Chr05	7405121	T	TTA	Chr05_74051T_TTA	INDEL	1206.35	29
Chr05	7441696	TTAACCAAAC	T	Chr05_74416TTAACCAAAC	INDEL	13432.59	18
Chr05	7441696	TTAACCAAAC	T	Chr05_74416TTAACCAAAC	INDEL	13432.59	18
Chr05	7441696	TTAACCAAAC	T	Chr05_74416TTAACCAAAC	INDEL	13432.59	18
Chr05	7468026	A	AAT	Chr05_74680A_AAT	INDEL	1882.24	1
Chr05	7481547	A	AAT	Chr05_74815A_AAT	INDEL	1716.93	0
Chr05	7481547	A	AAT	Chr05_74815A_AAT	INDEL	1716.93	0
Chr05	7481547	A	AAT	Chr05_74815A_AAT	INDEL	1716.93	0
Chr05	7481547	A	AAT	Chr05_74815A_AAT	INDEL	1716.93	0
Chr05	7481547	A	AAT	Chr05_74815A_AAT	INDEL	1716.93	0
Chr05	7489895	A	T	Chr05_74898A_T	SNP	19151.63	25
Chr05	7497998	G	GT	Chr05_74979G_GT	INDEL	16937.59	27
Chr05	7502277	C	A	Chr05_75022C_A	SNP	21262.63	30
Chr05	7502277	C	A	Chr05_75022C_A	SNP	21262.63	30
Chr05	7502277	C	A	Chr05_75022C_A	SNP	21262.63	30
Chr05	7502277	C	A	Chr05_75022C_A	SNP	21262.63	30
Chr05	7502319	T	TA	Chr05_75023T_TA	INDEL	23497.59	32
Chr05	7502319	T	TA	Chr05_75023T_TA	INDEL	23497.59	32
Chr05	7502319	T	TA	Chr05_75023T_TA	INDEL	23497.59	32
Chr05	7502319	T	TA	Chr05_75023T_TA	INDEL	23497.59	32
Chr05	7502323	CT	C	Chr05_75023CT_C	INDEL	23636.59	31
Chr05	7502323	CT	C	Chr05_75023CT_C	INDEL	23636.59	31
Chr05	7502323	CT	C	Chr05_75023CT_C	INDEL	23636.59	31
Chr05	7502323	CT	C	Chr05_75023CT_C	INDEL	23636.59	31
Chr05	7502371	A	AT	Chr05_75023A_AT	INDEL	18044.59	23
Chr05	7502371	A	AT	Chr05_75023A_AT	INDEL	18044.59	23
Chr05	7502371	A	AT	Chr05_75023A_AT	INDEL	18044.59	23
Chr05	7502371	A	AT	Chr05_75023A_AT	INDEL	18044.59	23
Chr05	7509449	A	T	Chr05_75094A_T	SNP	12712.29	21
Chr05	7509449	A	T	Chr05_75094A_T	SNP	12712.29	21
Chr05	7509449	A	T	Chr05_75094A_T	SNP	12712.29	21
Chr05	7509449	A	T	Chr05_75094A_T	SNP	12712.29	21
Chr05	7509754	T	G	Chr05_75097T_G	SNP	14035.29	34
Chr05	7509754	T	G	Chr05_75097T_G	SNP	14035.29	34
Chr05	7509754	T	G	Chr05_75097T_G	SNP	14035.29	34
Chr05	7509754	T	G	Chr05_75097T_G	SNP	14035.29	34
Chr05	7642043	C	CAT	Chr05_76420C_CAT	INDEL	3235.24	0
Chr05	7642043	C	CAT	Chr05_76420C_CAT	INDEL	3235.24	0
Chr05	7642043	C	CAT	Chr05_76420C_CAT	INDEL	3235.24	0
Chr05	7642043	C	CAT	Chr05_76420C_CAT	INDEL	3235.24	0
Chr05	7642043	C	CAT	Chr05_76420C_CAT	INDEL	3235.24	0
Chr05	7642043	C	CAT	Chr05_76420C_CAT	INDEL	3235.24	0
Chr05	7642043	C	CAT	Chr05_76420C_CAT	INDEL	3235.24	0
Chr05	7642043	C	CAT	Chr05_76420C_CAT	INDEL	3235.24	0
Chr05	7642043	C	CAT	Chr05_76420C_CAT	INDEL	3235.24	0
Chr05	7642043	C	CAT	Chr05_76420C_CAT	INDEL	3235.24	0
Chr05	7642043	C	CAT	Chr05_76420C_CAT	INDEL	3235.24	0
Chr05	7642043	C	CAT	Chr05_76420C_CAT	INDEL	3235.24	0
Chr05	7642043	C	CAT	Chr05_76420C_CAT	INDEL	3235.24	0
Chr05	7672763	A	T	Chr05_76727A_T	SNP	20971.63	30
Chr05	7672763	A	T	Chr05_76727A_T	SNP	20971.63	30
Chr05	7672763	A	T	Chr05_76727A_T	SNP	20971.63	30
Chr05	7672763	A	T	Chr05_76727A_T	SNP	20971.63	30
Chr05	7672763	A	T	Chr05_76727A_T	SNP	20971.63	30
Chr05	7672763	A	T	Chr05_76727A_T	SNP	20971.63	30
Chr05	7672763	A	T	Chr05_76727A_T	SNP	20971.63	30
Chr05	7672763	A	T	Chr05_76727A_T	SNP	20971.63	30
Chr05	7672763	A	T	Chr05_76727A_T	SNP	20971.63	30
Chr05	7672763	A	T	Chr05_76727A_T	SNP	20971.63	30
Chr05	7672763	A	T	Chr05_76727A_T	SNP	20971.63	30
Chr05	7672763	A	T	Chr05_76727A_T	SNP	20971.63	30
Chr05	7740045	T	C	Chr05_77400T_C	SNP	17245.63	30
Chr05	7853152	C	A	Chr05_78531C_A	SNP	11697.63	13
Chr05	7895858	G	T	Chr05_78958G_T	SNP	21641.63	23
Chr05	7895858	G	T	Chr05_78958G_T	SNP	21641.63	23
Chr05	7895858	G	T	Chr05_78958G_T	SNP	21641.63	23
Chr05	8044207	A	T	Chr05_80442A_T	SNP	11232.63	14
Chr05	8044219	C	T	Chr05_80442C_T	SNP	12663.63	19
Chr05	8044222	C	T	Chr05_80442C_T	SNP	12710.63	16
Chr05	8044225	C	T	Chr05_80442C_T	SNP	12530.63	15
Chr05	8044230	C	T	Chr05_80442C_T	SNP	12350.63	13
Chr05	8044232	C	T	Chr05_80442C_T	SNP	12395.63	13
Chr05	8044233	C	T	Chr05_80442C_T	SNP	12522.63	14
Chr05	8044234	C	T	Chr05_80442C_T	SNP	12452.63	14
Chr05	8044240	T	TA	Chr05_80442T_TA	INDEL	11816.59	15
Chr05	8051551	A	G	Chr05_80515A_G	SNP	10967.63	15
Chr05	8262291	G	GTA	Chr05_82622G_GTA	INDEL	1406.35	23
Chr05	8300883	T	TA	Chr05_83008T_TA	INDEL	8146.59	17
Chr05	8300883	T	TA	Chr05_83008T_TA	INDEL	8146.59	17
Chr05	8300883	T	TA	Chr05_83008T_TA	INDEL	8146.59	17
Chr05	8300883	T	TA	Chr05_83008T_TA	INDEL	8146.59	17
Chr05	8300883	T	TA	Chr05_83008T_TA	INDEL	8146.59	17
Chr05	8372505	G	T	Chr05_83725G_T	SNP	9934.63	21
Chr05	8372506	A	G	Chr05_83725A_G	SNP	9889.63	22
Chr05	8511836	A	G	Chr05_85118A_G	SNP	21095.63	24
Chr05	8572446	C	T	Chr05_85724C_T	SNP	16827.63	21
Chr05	8719545	A	AAT	Chr05_87195A_AAT	INDEL	2949.24	1
Chr05	8798241	T	TTA	Chr05_87982T_TTA	INDEL	88.37	0
Chr05	8798241	T	TTA	Chr05_87982T_TTA	INDEL	88.37	0
Chr05	8798241	T	TTA	Chr05_87982T_TTA	INDEL	88.37	0
Chr05	8798241	T	TTA	Chr05_87982T_TTA	INDEL	88.37	0
Chr05	8798241	T	TTA	Chr05_87982T_TTA	INDEL	88.37	0
Chr05	8798241	T	TTA	Chr05_87982T_TTA	INDEL	88.37	0
Chr05	8798241	T	TTA	Chr05_87982T_TTA	INDEL	88.37	0
Chr05	8798241	T	TTA	Chr05_87982T_TTA	INDEL	88.37	0
Chr05	8798241	T	TTA	Chr05_87982T_TTA	INDEL	88.37	0
Chr05	8934949	A	G	Chr05_89349A_G	SNP	18032.63	26
Chr05	9034484	G	A	Chr05_90344G_A	SNP	5220.29	0
Chr05	9034484	G	A	Chr05_90344G_A	SNP	5220.29	0
Chr05	9034484	G	A	Chr05_90344G_A	SNP	5220.29	0
Chr05	9034484	G	A	Chr05_90344G_A	SNP	5220.29	0
Chr05	9034484	G	A	Chr05_90344G_A	SNP	5220.29	0
Chr05	9034484	G	A	Chr05_90344G_A	SNP	5220.29	0
Chr05	9101994	G	T	Chr05_91019G_T	SNP	23028.63	37
Chr05	9101994	G	T	Chr05_91019G_T	SNP	23028.63	37
Chr05	9101996	T	TA	Chr05_91019T_TA	INDEL	23118.59	36
Chr05	9101996	T	TA	Chr05_91019T_TA	INDEL	23118.59	36
Chr05	9110123	C	A	Chr05_91101C_A	SNP	19392.63	28
Chr05	9110242	GT	G	Chr05_91102GT_G	INDEL	15218.59	20
Chr05	9151839	A	AAT	Chr05_91518A_AAT	INDEL	1864.93	1
Chr05	9151839	A	AAT	Chr05_91518A_AAT	INDEL	1864.93	1
Chr05	9151839	A	AAT	Chr05_91518A_AAT	INDEL	1864.93	1
Chr05	9160005	G	GA	Chr05_91600G_GA	INDEL	19848.59	28
Chr05	9160033	T	TG	Chr05_91600T_TG	INDEL	18839.59	30
Chr05	9167786	A	T	Chr05_91677A_T	SNP	17404.63	22
Chr05	9167786	A	T	Chr05_91677A_T	SNP	17404.63	22
Chr05	9167786	A	T	Chr05_91677A_T	SNP	17404.63	22
Chr05	9167786	A	T	Chr05_91677A_T	SNP	17404.63	22
Chr05	9246174	C	T	Chr05_92461C_T	SNP	7126.29	0
Chr05	9246174	C	T	Chr05_92461C_T	SNP	7126.29	0

Chr05	9246174	C	T	Chr05_92461	C_T	SNP	7126.29	0	0	0	19	[illegible]
Chr05	9246174	C	T	Chr05_92461	C_T	SNP	7126.29	0	0	0	19	[illegible]
Chr05	9246174	C	T	Chr05_92461	C_T	SNP	7126.29	0	0	0	19	[illegible]
Chr05	9246174	C	T	Chr05_92461	C_T	SNP	7126.29	0	0	0	19	[illegible]
Chr05	9288719	C	T	Chr05_92887	C_T	SNP	4855.98	0	0	0	0	[illegible]
Chr05	9305626	T	A	Chr05_93056	T_A	SNP	18490.63	22	7	21	22	[illegible]
Chr05	9305627	T	TGA	Chr05_93056	T_TGA	INDEL	18481.59	22	8	21	22	[illegible]
Chr05	9305628	TCC	T	Chr05_93056	TCC_T	INDEL	18481.59	21	8	20	21	[illegible]
Chr05	9326356	C	A	Chr05_93263	C_A	SNP	15159.63	30	17	6	21	[illegible]
Chr05	9326378	C	CA	Chr05_93263	C_CA	INDEL	15598.59	27	16	7	18	[illegible]
Chr05	9326404	A	T	Chr05_93264	A_T	SNP	14942.63	30	17	10	17	[illegible]
Chr05	9327878	C	A	Chr05_93278	C_A	SNP	20360.63	27	22	9	23	[illegible]
Chr05	9327928	TC	T	Chr05_93279	TC_T	INDEL	19428.59	21	15	10	13	[illegible]
Chr05	9327931	C	A	Chr05_93279	C_A	SNP	19437.63	21	15	9	13	[illegible]
Chr05	9327936	C	A	Chr05_93279	C_A	SNP	18830.63	20	13	8	12	[illegible]
Chr05	9327958	C	A	Chr05_93279	C_A	SNP	15635.63	16	8	8	10	[illegible]
Chr05	9327967	C	A	Chr05_93279	C_A	SNP	14600.63	14	7	8	7	[illegible]
Chr05	9327968	C	A	Chr05_93279	C_A	SNP	14375.63	14	7	8	7	[illegible]
Chr05	9327973	C	A	Chr05_93279	C_A	SNP	13430.63	13	6	7	7	[illegible]
Chr05	9327975	C	A	Chr05_93279	C_A	SNP	13430.63	13	6	7	7	[illegible]
Chr05	9327977	TC	T	Chr05_93279	TC_T	INDEL	12971.59	13	6	7	7	[illegible]
Chr05	9327980	G	GA	Chr05_93279	G_GA	INDEL	12746.59	12	5	7	7	[illegible]
Chr05	9327982	C	A	Chr05_93279	C_A	SNP	12395.63	12	5	6	7	[illegible]
Chr05	9327985	C	A	Chr05_93279	C_A	SNP	11540.63	11	4	6	7	[illegible]
Chr05	9327988	C	T	Chr05_93279	C_T	SNP	11135.63	10	5	5	7	[illegible]
Chr05	9327990	C	A	Chr05_93279	C_A	SNP	10820.63	10	5	5	7	[illegible]
Chr05	9327998	C	T	Chr05_93279	C_T	SNP	9605.63	10	5	4	7	[illegible]
Chr05	9327999	A	T	Chr05_93279	A_T	SNP	9515.63	10	5	4	7	[illegible]
Chr05	9328000	C	T	Chr05_93280	C_T	SNP	9380.63	10	5	4	7	[illegible]
Chr05	9328006	G	T	Chr05_93280	G_T	SNP	8750.63	10	4	4	7	[illegible]
Chr05	9328332	A	T	Chr05_93283	A_T	SNP	19695.63	38	8	13	32	[illegible]
Chr05	9573935	G	GA	Chr05_95739	G_GA	INDEL	13527.59	19	8	13	13	[illegible]
Chr05	9685598	T	C	Chr05_96855	T_C	SNP	16315.63	33	15	12	28	[illegible]
Chr05	9736002	G	A	Chr05_97360	G_A	SNP	19508.63	31	12	9	37	[illegible]
Chr05	9802699	A	T	Chr05_98026	A_T	SNP	15537.63	19	16	11	20	[illegible]
Chr05	9955105	G	GATAT	Chr05_99551	G_GATAT	INDEL	9813.59	13	8	13	9	[illegible]
Chr05	9955105	G	GATAT	Chr05_99551	G_GATAT	INDEL	9813.59	13	8	13	9	[illegible]
Chr05	9955105	G	GATAT	Chr05_99551	G_GATAT	INDEL	9813.59	13	8	13	9	[illegible]
Chr05	9955105	G	GATAT	Chr05_99551	G_GATAT	INDEL	9813.59	13	8	13	9	[illegible]
Chr05	9955105	G	GATAT	Chr05_99551	G_GATAT	INDEL	9813.59	13	8	13	9	[illegible]
Chr05	9955107	G	GTC	Chr05_99551	G_GTC	INDEL	10398.59	14	8	10	9	[illegible]
Chr05	9955107	G	GTC	Chr05_99551	G_GTC	INDEL	10398.59	14	8	10	9	[illegible]
Chr05	9955107	G	GTC	Chr05_99551	G_GTC	INDEL	10398.59	14	8	10	9	[illegible]
Chr05	9955107	G	GTC	Chr05_99551	G_GTC	INDEL	10398.59	14	8	10	9	[illegible]
Chr05	9955107	G	GTC	Chr05_99551	G_GTC	INDEL	10398.59	14	8	10	9	[illegible]
Chr05	9955108	CTGG	C	Chr05_99551	CTGG_C	INDEL	10021.59	12	8	8	10	[illegible]
Chr05	9955108	CTGG	C	Chr05_99551	CTGG_C	INDEL	10021.59	12	8	8	10	[illegible]
Chr05	9955108	CTGG	C	Chr05_99551	CTGG_C	INDEL	10021.59	12	8	8	10	[illegible]
Chr05	9955108	CTGG	C	Chr05_99551	CTGG_C	INDEL	10021.59	12	8	8	10	[illegible]
Chr05	9955108	CTGG	C	Chr05_99551	CTGG_C	INDEL	10021.59	12	8	8	10	[illegible]
Chr05	9971203	T	TTA	Chr05_99712	T_TTA	INDEL	1634.93	0	0	0	1	[illegible]
Chr05	10117226	G	GA	Chr05_10117	G_GA	INDEL	7969.59	10	10	4	13	[illegible]
Chr05	10296784	AT	A	Chr05_10296	AT_A	INDEL	15785.59	27	12	13	30	[illegible]
Chr05	10297728	TA	T	Chr05_10297	TA_T	INDEL	10634.59	15	4	7	12	[illegible]
Chr05	10379816	T	A	Chr05_10379	T_A	SNP	12601.63	24	7	9	13	[illegible]
Chr05	10379816	T	A	Chr05_10379	T_A	SNP	12601.63	24	7	9	13	[illegible]
Chr05	10379816	T	A	Chr05_10379	T_A	SNP	12601.63	24	7	9	13	[illegible]
Chr05	10379816	T	A	Chr05_10379	T_A	SNP	12601.63	24	7	9	13	[illegible]
Chr05	10379816	T	A	Chr05_10379	T_A	SNP	12601.63	24	7	9	13	[illegible]
Chr05	10386491	A	AT	Chr05_10386	A_AT	INDEL	10342.59	17	12	16	13	[illegible]
Chr05	10386491	A	AT	Chr05_10386	A_AT	INDEL	10342.59	17	12	16	13	[illegible]
Chr05	10387698	T	A	Chr05_10387	T_A	SNP	793.39	13	4	13	0	[illegible]
Chr05	10387698	T	A	Chr05_10387	T_A	SNP	793.39	13	4	13	0	[illegible]
Chr05	10387698	T	A	Chr05_10387	T_A	SNP	793.39	13	4	13	0	[illegible]
Chr05	10387698	T	A	Chr05_10387	T_A	SNP	793.39	13	4	13	0	[illegible]
Chr05	10387698	T	A	Chr05_10387	T_A	SNP	793.39	13	4	13	0	[illegible]
Chr05	10387698	T	A	Chr05_10387	T_A	SNP	793.39	13	4	13	0	[illegible]
Chr05	10486713	A	AAT	Chr05_10486	A_AAT	INDEL	3262.93	0	0	0	0	[illegible]
Chr05	10486713	A	AAT	Chr05_10486	A_AAT	INDEL	3262.93	0	0	0	0	[illegible]
Chr05	10488359	C	G	Chr05_10488	C_G	SNP	21390.63	32	18	28	27	[illegible]
Chr05	10488359	C	G	Chr05_10488	C_G	SNP	21390.63	32	18	28	27	[illegible]
Chr05	10488359	C	G	Chr05_10488	C_G	SNP	21390.63	32	18	28	27	[illegible]
Chr05	10488400	C	G	Chr05_10488	C_G	SNP	21858.63	28	18	22	38	[illegible]
Chr05	10488400	C	G	Chr05_10488	C_G	SNP	21858.63	28	18	22	38	[illegible]
Chr05	10488400	C	G	Chr05_10488	C_G	SNP	21858.63	28	18	22	38	[illegible]
Chr05	10488428	G	A	Chr05_10488	G_A	SNP	21021.63	28	17	20	37	[illegible]
Chr05	10488428	G	A	Chr05_10488	G_A	SNP	21021.63	28	17	20	37	[illegible]
Chr05	10488428	G	A	Chr05_10488	G_A	SNP	21021.63	28	17	20	37	[illegible]
Chr05	10559267	C	T	Chr05_10559	C_T	SNP	17620.63	25	13	18	24	[illegible]
Chr05	10559267	C	T	Chr05_10559	C_T	SNP	17620.63	25	13	18	24	[illegible]
Chr05	10559267	C	T	Chr05_10559	C_T	SNP	17620.63	25	13	18	24	[illegible]
Chr05	10559267	C	T	Chr05_10559	C_T	SNP	17620.63	25	13	18	24	[illegible]
Chr05	10687691	CA	C	Chr05_10687	CA_C	INDEL	15706.59	22	16	11	17	[illegible]
Chr05	10687693	A	T	Chr05_10687	A_T	SNP	15760.63	22	16	11	17	[illegible]
Chr05	10687696	TAA	T	Chr05_10687	TAA_T	INDEL	15982.59	22	16	11	17	[illegible]
Chr05	10687698	A	AGGC	Chr05_10687	A_AGGC	INDEL	15982.59	22	16	11	15	[illegible]
Chr05	10695877	A	AT	Chr05_10695	A_AT	INDEL	13709.59	23	9	15	19	[illegible]
Chr05	10702160	G	GA	Chr05_10702	G_GA	INDEL	14838.59	28	12	18	20	[illegible]
Chr05	10702160	G	GA	Chr05_10702	G_GA	INDEL	14838.59	28	12	18	20	[illegible]
Chr05	10702160	G	GA	Chr05_10702	G_GA	INDEL	14838.59	28	12	18	20	[illegible]
Chr05	10702160	G	GA	Chr05_10702	G_GA	INDEL	14838.59	28	12	18	20	[illegible]
Chr05	10702160	G	GA	Chr05_10702	G_GA	INDEL	14838.59	28	12	18	20	[illegible]
Chr05	10702160	G	GA	Chr05_10702	G_GA	INDEL	14838.59	28	12	18	20	[illegible]
Chr05	10702160	G	GA	Chr05_10702	G_GA	INDEL	14838.59	28	12	18	20	[illegible]
Chr05	10963799	A	AAT	Chr05_10963	A_AAT	INDEL	9546.59	16	12	13	22	[illegible]
Chr05	10963799	A	AAT	Chr05_10963	A_AAT	INDEL	9546.59	16	12	13	22	[illegible]
Chr05	10963799	A	AAT	Chr05_10963	A_AAT	INDEL	9546.59	16	12	13	22	[illegible]
Chr05	10963799	A	AAT	Chr05_10963	A_AAT	INDEL	9546.59	16	12	13	22	[illegible]
Chr05	10963799	A	AAT	Chr05_10963	A_AAT	INDEL	9546.59	16	12	13	22	[illegible]
Chr05	11003733	CT	C	Chr05_11003	CT_C	INDEL	18253.59	28	21	17	26	[illegible]
Chr05	11003733	CT	C	Chr05_11003	CT_C	INDEL	18253.59	28	21	17	26	[illegible]
Chr05	11003733	CT	C	Chr05_11003	CT_C	INDEL	18253.59	28	21	17	26	[illegible]
Chr05	11003733	CT	C	Chr05_11003	CT_C	INDEL	18253.59	28	21	17	26	[illegible]
Chr05	11003733	CT	C	Chr05_11003	CT_C	INDEL	18253.59	28	21	17	26	[illegible]
Chr05	11003733	CT	C	Chr05_11003	CT_C	INDEL	18253.59	28	21	17	26	[illegible]
Chr05	11050939	C	A	Chr05_11050	C_A	SNP	11360.63	22	5	15	22	[illegible]
Chr05	11071567	G	T	Chr05_11071	G_T	SNP	19155.63	37	8	20	32	[illegible]
Chr05	11075085	GC	G	Chr05_11075	GC_G	INDEL	15763.59	27	23	17	18	[illegible]
Chr05	11075197	A	AT	Chr05_11075	A_AT	INDEL	22143.59	32	17	19	32	[illegible]
Chr05	11075200	C	T	Chr05_11075	C_T	SNP	22347.63	32	17	21	33	[illegible]
Chr05	11075269	G	T	Chr05_11075	G_T	SNP	19422.63	32	17	15	21	[illegible]
Chr05	11344021	A	AAT	Chr05_11344	A_AAT	INDEL	1960.93	0	0	1	0	[illegible]
Chr05	11344021	A	AAT	Chr05_11344	A_AAT	INDEL	1960.93	0	0	1	0	[illegible]
Chr05	11344021	A	AAT	Chr05_11344	A_AAT	INDEL	1960.93	0	0	1	0	[illegible]
Chr05	11344021	A	AAT	Chr05_11344	A_AAT	INDEL	1960.93	0	0	1	0	[illegible]
Chr05	11638812	A	AT	Chr05_11638	A_AT	INDEL	14835.59	33	10	16	20	[illegible]
Chr05	11638812	A	AT	Chr05_11638	A_AT	INDEL	14835.59	33	10	16	20	[illegible]
Chr05	11638812	A	AT	Chr05_11638	A_AT	INDEL	14835.59	33	10	16	20	[illegible]
Chr05	11638812	A	AT	Chr05_11638	A_AT	INDEL	14835.59	33	10	16	20	[illegible]
Chr05	11638812	A	AT	Chr05_11638	A_AT	INDEL	14835.59	33	10	16	20	[illegible]
Chr05	11646200	C	T	Chr05_11646	C_T	SNP	21021.63	38	29	8	22	[illegible]
Chr05	11646200	C	T	Chr05_11646	C_T	SNP	21021.63	38	29	8	22	[illegible]
Chr05	11646200	C	T	Chr05_11646	C_T	SNP	21021.63	38	29	8	22	[illegible]
Chr05	11646200	C	T	Chr05_11646	C_T	SNP	21021.63	38	29	8	22	[illegible]
Chr05	11646200	C	T	Chr05_11646	C_T	SNP	21021.63	38	29	8	22	[illegible]
Chr05	11669771	AATAT	AAT	Chr05_11669	AATAT_AAT	INDEL	4935.24	20	7	5	1	[illegible]
Chr05	11669771	AATAT	AAT	Chr05_11669	AATAT_AAT	INDEL	4935.24	20	7	5	1	[illegible]
Chr05	11669771	AATAT	AAT	Chr05_11669	AATAT_AAT	INDEL	4935.24	20	7	5	1	[illegible]
Chr05	11669771	AATAT	AAT	Chr05_11669	AATAT_AAT	INDEL	4935.24	20	7	5	1	[illegible]
Chr05	11669771	AATAT	AAT	Chr05_11669	AATAT_AAT	INDEL	4935.24	20	7	5	1	[illegible]
Chr05	11781409	T	G	Chr05_11781	T_G	SNP	13498.63	30	9	16	19	[illegible]
Chr05	11781409	T	G	Chr05_11781	T_G	SNP	13498.63	30	9	16	19	[illegible]
Chr05	11781409	T	G	Chr05_11781	T_G	SNP	13498.63	30	9	16	19	[illegible]
Chr05	11781409	T	G	Chr05_11781	T_G	SNP	13498.63	30	9	16	19	[illegible]
Chr05	11781416	T	C	Chr05_11781	T_C	SNP	13369.63	29	7	17	19	[illegible]
Chr05	11781416	T	C	Chr05_11781	T_C	SNP	13369.63	29	7	17	19	[illegible]
Chr05	11781416	T	C	Chr05_11781	T_C	SNP	13369.63	29	7	17	19	[illegible]
Chr05	11781416	T	C	Chr05_11781	T_C	SNP	13369.63	29	7	17	19	[illegible]
Chr05	11781480	T	G	Chr05_11781	T_G	SNP	13756.63	25	12	19	20	[illegible]
Chr05	11781480	T	G	Chr05_11781	T_G	SNP	13756.63	25	12	19	20	[illegible]
Chr05	11781480	T	G	Chr05_11781	T_G	SNP	13756.63	25	12	19	20	[illegible]
Chr05	11781480	T	G	Chr05_11781	T_G	SNP	13756.63	25	12	19	20	[illegible]
Chr05	11950461	C	A	Chr05_11950	C_A	SNP	8031.63	8	6	4	10	[illegible]
Chr05	12055366	C	T	Chr05_12055	C_T	SNP	13469.63	23	9	14	25	[illegible]
Chr05	12082572	C	A	Chr05_12082	C_A	SNP	14397.63	13	7	7	20	[illegible]
Chr05	12082572	C	A	Chr05_12082	C_A	SNP	14397.63	13	7	7	20	[illegible]
Chr05	12082572	C	A	Chr05_12082	C_A	SNP	14397.63	13	7	7	20	[illegible]
Chr05	12082572	C	A	Chr05_12082	C_A	SNP	14397.63	13	7	7	20	[illegible]
Chr05	12082575	T	G	Chr05_12082	T_G	SNP	14466.63	13	7	8	20	[illegible]
Chr05	12082575	T	G	Chr05_12082	T_G	SNP	14466.63	13	7	8	20	[illegible]
Chr05	12082575	T	G	Chr05_12082	T_G	SNP	14466.63	13	7	8	20	[illegible]
Chr05	12082575	T	G	Chr05_12082	T_G	SNP	14466.63	13	7	8	20	[illegible]
Chr05	12082577	T	A	Chr05_12082	T_A	SNP	14325.63	13	7	8	20	[illegible]
Chr05	12082577	T	A	Chr05_12082	T_A	SNP	14325.63	13	7	8	20	[illegible]
Chr05	12082577	T	A	Chr05_12082	T_A	SNP	14325.63	13	7	8	20	[illegible]
Chr05	12082577	T	A	Chr05_12082	T_A	SNP	14325.63	13	7	8	20	[illegible]
Chr05	12082578	A	T	Chr05_12082	A_T	SNP	14325.63	13	7	8	20	[illegible]
Chr05	12082578	A	T	Chr05_12082	A_T	SNP	14325.63	13	7	8	20	[illegible]
Chr05	12082578	A	T	Chr05_12082	A_T	SNP	14325.63	13	7	8	20	[illegible]
Chr05	12082578	A	T	Chr05_12082	A_T	SNP	14325.63	13	7	8	20	[illegible]
Chr05	12082582	T	TAGAG	Chr05_12082	T_TAGAG	INDEL	14002.59	13	7	7	21	[illegible]
Chr05	12082582	T	TAGAG	Chr05_12082	T_TAGAG	INDEL	14002.59	13	7	7	21	[illegible]
Chr05	12082582	T	TAGAG	Chr05_12082	T_TAGAG	INDEL	14002.59	13	7	7	21	[illegible]
Chr05	12082582	T	TAGAG	Chr05_12082	T_TAGAG	INDEL	14002.59	13	7	7	21	[illegible]
Chr05	12082584	ACCGCTCC	A	Chr05_12082	ACCGCTCC_A	INDEL	12303.37	13	7	7	19	[illegible]
Chr05	12082584	ACCGCTCC	A	Chr05_12082	ACCGCTCC_A	INDEL	12303.37	13	7	7	19	[illegible]
Chr05	12082584	ACCGCTCC	A	Chr05_12082	ACCGCTCC_A	INDEL	12303.37	13	7	7	19	[illegible]
Chr05	12082584	ACCGCTCC	A	Chr05_12082	ACCGCTCC_A	INDEL	12303.37	13	7	7	19	[illegible]
Chr05	12082593	C	A	Chr05_12082	C_A	SNP	11277.41	12	7	7	20	[illegible]
Chr05	12082593	C	A	Chr05_12082	C_A	SNP	11277.41	12	7	7	20	[illegible]
Chr05	12082593	C	A	Chr05_12082	C_A	SNP	11277.41	12	7	7	20	[illegible]
Chr05	12082593	C	A	Chr05_12082	C_A	SNP	11277.41	12	7	7	20	[illegible]
Chr05	12082594	G	GT	Chr05_12082	G_GT	INDEL	11313.37	12	6	7	20	[illegible]
Chr05	12082594	G	GT	Chr05_12082	G_GT	INDEL	11313.37	12	6	7	20	[illegible]
Chr05	12082594	G	GT	Chr05_12082	G_GT	INDEL	11313.37	12	6	7	20	[illegible]
Chr05	12082594	G	GT	Chr05_12082	G_GT	INDEL	11313.37	12	6	7	20	[illegible]
Chr05	12082596	G	GCA	Chr05_12082	G_GCA	INDEL	11403.37	12	6	7	20	[illegible]
Chr05	12082596	G	GCA	Chr05_12082	G_GCA	INDEL	11403.37	12	6	7	20	[illegible]
Chr05	12082596	G	GCA	Chr05_12082	G_GCA	INDEL	11403.37	12	6	7	20	[illegible]
Chr05	12082596	G	GCA	Chr05_12082	G_GCA	INDEL	11403.37	12	6	7	20	[illegible]
Chr05	12082598	AAC	A	Chr05_12082	AAC_A	INDEL	10998.37	12	7	7	20	[illegible]
Chr05	12082598	AAC	A	Chr05_12082	AAC_A	INDEL	10998.37	12	7	7	20	[illegible]
Chr05	12082598	AAC	A	Chr05_12082	AAC_A	INDEL	10998.37	12	7	7	20	[illegible]
Chr05	12082598	AAC	A	Chr05_12082	AAC_A	INDEL	10998.37	12	7	7	20	[illegible]
Chr05	12082602	C	T	Chr05_12082	C_T	SNP	10422.41	12	7	7	20	[illegible]
Chr05	12082602	C	T	Chr05_12082	C_T	SNP	10422.41	12	7	7	20	[illegible]
Chr05	12082602	C	T	Chr05_12082	C_T	SNP	10422.41	12	7	7	20	[illegible]
Chr05	12082602	C	T	Chr05_12082	C_T	SNP	10422.41	12	7	7	20	[illegible]
Chr05	12082604	T	TTGATGCTA	Chr05_12082	T_TTGATGCT	INDEL	11088.37	12	7	6	20	[illegible]
Chr05	12082604	T	TTGATGCTA	Chr05_12082	T_TTGATGCT	INDEL	11088.37	12	7	6	20	[illegible]
Chr05	12082604	T	TTGATGCTA	Chr05_12082	T_TTGATGCT	INDEL	11088.37	12	7	6	20	[illegible]
Chr05	12082604	T	TTGATGCTA	Chr05_12082	T_TTGATGCT	INDEL	11088.37	12	7	6	20	[illegible]
Chr05	12082607	C	A	Chr05_12082	C_A	SNP	10030.41	14	8	7	22	[illegible]
Chr05	12082607	C	A	Chr05_12082	C_A	SNP	10030.41	14	8	7	22	[illegible]
Chr05	12082607	C	A	Chr05_12082	C_A	SNP	10030.41	14	8	7	22	[illegible]
Chr05	12082607	C	A	Chr05_12082	C_A	SNP	10030.41	14	8	7	22	[illegible]
Chr05	12082609	A	T	Chr05_12082	A_T	SNP	9760.41	14	7	7	22	[illegible]
Chr05	12082609	A	T	Chr05_12082	A_T	SNP	9760.41	14	7	7	22	[illegible]
Chr05	12082609	A	T	Chr05_12082	A_T	SNP	9760.41	14	7	7	22	[illegible]
Chr05	12082609	A	T	Chr05_12082	A_T	SNP	9760.41	14	7	7	22	[illegible]
Chr05	12082611	AAGGG	A	Chr05_12082	AAGGG_A	INDEL	9706.37	14	7	7	21	[illegible]
Chr05	12082611	AAGGG	A	Chr05_12082	AAGGG_A	INDEL	9706.37	14	7	7	21	[illegible]

Chr05	12082611	AAGGG	A	Chr05_12082AAGGG_A	INDEL	9706.37	[illegible]
Chr05	12082611	AAGGG	A	Chr05_12082AAGGG_A	INDEL	9706.37	[illegible]
Chr05	12082619	G	C	Chr05_12082G_C	SNP	9220.41	[illegible]
Chr05	12082619	G	C	Chr05_12082G_C	SNP	9220.41	[illegible]
Chr05	12082619	G	C	Chr05_12082G_C	SNP	9220.41	[illegible]
Chr05	12082619	G	C	Chr05_12082G_C	SNP	9220.41	[illegible]
Chr05	12082620	TA	T	Chr05_12082TA_T	INDEL	9166.37	[illegible]
Chr05	12082620	TA	T	Chr05_12082TA_T	INDEL	9166.37	[illegible]
Chr05	12082620	TA	T	Chr05_12082TA_T	INDEL	9166.37	[illegible]
Chr05	12082620	TA	T	Chr05_12082TA_T	INDEL	9166.37	[illegible]
Chr05	12082624	G	GTA	Chr05_12082G_GTA	INDEL	8931.37	[illegible]
Chr05	12082624	G	GTA	Chr05_12082G_GTA	INDEL	8931.37	[illegible]
Chr05	12082624	G	GTA	Chr05_12082G_GTA	INDEL	8931.37	[illegible]
Chr05	12082624	G	GTA	Chr05_12082G_GTA	INDEL	8931.37	[illegible]
Chr05	12087058	A	AT	Chr05_12087A_AT	INDEL	12025.59	[illegible]
Chr05	12087058	A	AT	Chr05_12087A_AT	INDEL	12025.59	[illegible]
Chr05	12087058	A	AT	Chr05_12087A_AT	INDEL	12025.59	[illegible]
Chr05	12087058	A	AT	Chr05_12087A_AT	INDEL	12025.59	[illegible]
Chr05	12087058	A	AT	Chr05_12087A_AT	INDEL	12025.59	[illegible]
Chr05	12087071	C	CG	Chr05_12087C_CG	INDEL	12441.59	[illegible]
Chr05	12087071	C	CG	Chr05_12087C_CG	INDEL	12441.59	[illegible]
Chr05	12087071	C	CG	Chr05_12087C_CG	INDEL	12441.59	[illegible]
Chr05	12087071	C	CG	Chr05_12087C_CG	INDEL	12441.59	[illegible]
Chr05	12087071	C	CG	Chr05_12087C_CG	INDEL	12441.59	[illegible]
Chr05	12087080	T	A	Chr05_12087T_A	SNP	12272.63	[illegible]
Chr05	12087080	T	A	Chr05_12087T_A	SNP	12272.63	[illegible]
Chr05	12087080	T	A	Chr05_12087T_A	SNP	12272.63	[illegible]
Chr05	12087080	T	A	Chr05_12087T_A	SNP	12272.63	[illegible]
Chr05	12087080	T	A	Chr05_12087T_A	SNP	12272.63	[illegible]
Chr05	12203493	GC	G	Chr05_12203GC_G	INDEL	18319.59	[illegible]
Chr05	12331969	A	AAT	Chr05_12331A_AAT	INDEL	1453.93	[illegible]
Chr05	12331969	A	AAT	Chr05_12331A_AAT	INDEL	1453.93	[illegible]
Chr05	12331969	A	AAT	Chr05_12331A_AAT	INDEL	1453.93	[illegible]
Chr05	12331969	A	AAT	Chr05_12331A_AAT	INDEL	1453.93	[illegible]
Chr05	12331969	A	AAT	Chr05_12331A_AAT	INDEL	1453.93	[illegible]
Chr05	12360457	A	AT	Chr05_12360A_AT	INDEL	14107.59	[illegible]
Chr05	12393622	C	A	Chr05_12393C_A	SNP	5684.29	[illegible]
Chr05	12454322	C	T	Chr05_12454C_T	SNP	15133.63	[illegible]
Chr05	12533979	TTAAAACTTA	T	Chr05_12533TTAAAACTTA	INDEL	35498.59	[illegible]
Chr05	12595332	C	A	Chr05_12595C_A	SNP	16715.63	[illegible]
Chr05	12598568	A	AAT	Chr05_12598A_AAT	INDEL	10927.59	[illegible]
Chr05	12598568	A	AAT	Chr05_12598A_AAT	INDEL	10927.59	[illegible]
Chr05	12598568	A	AAT	Chr05_12598A_AAT	INDEL	10927.59	[illegible]
Chr05	12630985	G	GTAAGTAAA	Chr05_12630G_GTAAGTA	INDEL	43149.59	[illegible]
Chr05	12631054	ATTTGCTATA	A	Chr05_12631ATTTGCTATA	INDEL	26551.59	[illegible]
Chr05	12827289	C	A	Chr05_12827C_A	SNP	22613.63	[illegible]
Chr05	12827292	G	GC	Chr05_12827G_GC	INDEL	23248.59	[illegible]
Chr05	12827294	A	AT	Chr05_12827A_AT	INDEL	23286.59	[illegible]
Chr05	12827296	G	T	Chr05_12827G_T	SNP	22855.63	[illegible]
Chr05	12844614	G	A	Chr05_12844G_A	SNP	3241.98	[illegible]
Chr05	12983394	G	A	Chr05_12983G_A	SNP	4502.98	[illegible]
Chr05	13130536	A	T	Chr05_13130A_T	SNP	21841.63	[illegible]
Chr05	13286622	C	T	Chr05_13286C_T	SNP	5604.29	[illegible]
Chr05	13321689	T	C	Chr05_13321T_C	SNP	16926.63	[illegible]
Chr05	13321689	T	C	Chr05_13321T_C	SNP	16926.63	[illegible]
Chr05	13321689	T	C	Chr05_13321T_C	SNP	16926.63	[illegible]
Chr05	13435761	G	A	Chr05_13435G_A	SNP	2394.39	[illegible]
Chr05	13480211	T	G	Chr05_13480T_G	SNP	20792.63	[illegible]
Chr05	13600334	T	TA	Chr05_13600T_TA	INDEL	8238.59	[illegible]
Chr05	13600334	T	TA	Chr05_13600T_TA	INDEL	8238.59	[illegible]
Chr05	13600334	T	TA	Chr05_13600T_TA	INDEL	8238.59	[illegible]
Chr05	13600334	T	TA	Chr05_13600T_TA	INDEL	8238.59	[illegible]
Chr05	13600334	T	TA	Chr05_13600T_TA	INDEL	8238.59	[illegible]
Chr05	13600334	T	TA	Chr05_13600T_TA	INDEL	8238.59	[illegible]
Chr05	13600334	T	TA	Chr05_13600T_TA	INDEL	8238.59	[illegible]
Chr05	13738860	G	A	Chr05_13738G_A	SNP	14104.63	[illegible]
Chr05	13776911	A	AC	Chr05_13776A_AC	INDEL	17711.59	[illegible]
Chr05	13776911	A	AC	Chr05_13776A_AC	INDEL	17711.59	[illegible]
Chr05	13776911	A	AC	Chr05_13776A_AC	INDEL	17711.59	[illegible]
Chr05	13776911	A	AC	Chr05_13776A_AC	INDEL	17711.59	[illegible]
Chr05	13776911	A	AC	Chr05_13776A_AC	INDEL	17711.59	[illegible]
Chr05	13776911	A	AC	Chr05_13776A_AC	INDEL	17711.59	[illegible]
Chr05	13776911	A	AC	Chr05_13776A_AC	INDEL	17711.59	[illegible]
Chr05	13820523	C	T	Chr05_13820C_T	SNP	22142.63	[illegible]
Chr05	13820523	C	T	Chr05_13820C_T	SNP	22142.63	[illegible]
Chr05	13820523	C	T	Chr05_13820C_T	SNP	22142.63	[illegible]
Chr05	13820523	C	T	Chr05_13820C_T	SNP	22142.63	[illegible]
Chr05	13878004	T	G	Chr05_13878T_G	SNP	18535.63	[illegible]
Chr05	14091713	C	A	Chr05_14091C_A	SNP	22100.63	[illegible]
Chr05	14091713	C	A	Chr05_14091C_A	SNP	22100.63	[illegible]
Chr05	14091713	C	A	Chr05_14091C_A	SNP	22100.63	[illegible]
Chr05	14091713	C	A	Chr05_14091C_A	SNP	22100.63	[illegible]
Chr05	14091713	C	A	Chr05_14091C_A	SNP	22100.63	[illegible]
Chr05	14091713	C	A	Chr05_14091C_A	SNP	22100.63	[illegible]
Chr05	14091713	C	A	Chr05_14091C_A	SNP	22100.63	[illegible]
Chr05	14091714	T	G	Chr05_14091T_G	SNP	22100.63	[illegible]
Chr05	14091714	T	G	Chr05_14091T_G	SNP	22100.63	[illegible]
Chr05	14091714	T	G	Chr05_14091T_G	SNP	22100.63	[illegible]
Chr05	14091714	T	G	Chr05_14091T_G	SNP	22100.63	[illegible]
Chr05	14091714	T	G	Chr05_14091T_G	SNP	22100.63	[illegible]
Chr05	14091714	T	G	Chr05_14091T_G	SNP	22100.63	[illegible]
Chr05	14091714	T	G	Chr05_14091T_G	SNP	22100.63	[illegible]
Chr05	14284433	A	AT	Chr05_14284A_AT	INDEL	21696.59	[illegible]
Chr05	14284447	C	T	Chr05_14284C_T	SNP	21667.63	[illegible]
Chr05	14459472	A	AAT	Chr05_14459A_AAT	INDEL	2848.24	[illegible]
Chr05	14460096	A	AG	Chr05_14460A_AG	INDEL	17893.59	[illegible]
Chr05	14460099	T	A	Chr05_14460T_A	SNP	17834.63	[illegible]
Chr05	14547163	AG	A	Chr05_14547AG_A	INDEL	21727.59	[illegible]
Chr05	14547171	TG	T	Chr05_14547TG_T	INDEL	21932.59	[illegible]
Chr05	14620589	AT	A	Chr05_14620AT_A	INDEL	18941.59	[illegible]
Chr05	14620589	AT	A	Chr05_14620AT_A	INDEL	18941.59	[illegible]
Chr05	14620589	AT	A	Chr05_14620AT_A	INDEL	18941.59	[illegible]
Chr05	14681924	C	A	Chr05_14681C_A	SNP	20405.63	[illegible]
Chr05	14681937	C	A	Chr05_14681C_A	SNP	20679.63	[illegible]
Chr05	14681958	G	GT	Chr05_14681G_GT	INDEL	20906.59	[illegible]
Chr05	14681981	C	A	Chr05_14681C_A	SNP	19917.63	[illegible]
Chr05	14721203	A	AT	Chr05_14721A_AT	INDEL	17909.59	[illegible]
Chr05	14740159	TG	T	Chr05_14740TG_T	INDEL	18552.59	[illegible]
Chr05	14740159	TG	T	Chr05_14740TG_T	INDEL	18552.59	[illegible]
Chr05	14740159	TG	T	Chr05_14740TG_T	INDEL	18552.59	[illegible]
Chr05	15018309	A	G	Chr05_15018A_G	SNP	16315.63	[illegible]
Chr05	15018618	A	C	Chr05_15018A_C	SNP	15355.63	[illegible]
Chr05	15018619	A	T	Chr05_15018A_T	SNP	15805.63	[illegible]
Chr05	15018620	T	TCC	Chr05_15018T_TCC	INDEL	15841.59	[illegible]
Chr05	15018621	G	T	Chr05_15018G_T	SNP	15844.63	[illegible]
Chr05	15018623	G	C	Chr05_15018G_C	SNP	15712.63	[illegible]
Chr05	15018625	TGGAAAG	T	Chr05_15018TGGAAAG_T	INDEL	15658.59	[illegible]
Chr05	15020210	C	A	Chr05_15020C_A	SNP	17599.63	[illegible]
Chr05	15020243	AC	A	Chr05_15020AC_A	INDEL	16090.59	[illegible]
Chr05	15020276	T	A	Chr05_15020T_A	SNP	13292.63	[illegible]
Chr05	15048587	A	AAT	Chr05_15048A_AAT	INDEL	280.37	[illegible]
Chr05	15048587	A	AAT	Chr05_15048A_AAT	INDEL	280.37	[illegible]
Chr05	15048587	A	AAT	Chr05_15048A_AAT	INDEL	280.37	[illegible]
Chr05	15048587	A	AAT	Chr05_15048A_AAT	INDEL	280.37	[illegible]
Chr05	15048587	A	AAT	Chr05_15048A_AAT	INDEL	280.37	[illegible]
Chr05	15048587	A	AAT	Chr05_15048A_AAT	INDEL	280.37	[illegible]
Chr05	15077859	A	AT	Chr05_15077A_AT	INDEL	17342.59	[illegible]
Chr05	15241968	A	AAT	Chr05_15241A_AAT	INDEL	175.37	[illegible]
Chr05	15241968	A	AAT	Chr05_15241A_AAT	INDEL	175.37	[illegible]
Chr05	15241968	A	AAT	Chr05_15241A_AAT	INDEL	175.37	[illegible]
Chr05	15241968	A	AAT	Chr05_15241A_AAT	INDEL	175.37	[illegible]
Chr05	15241968	A	AAT	Chr05_15241A_AAT	INDEL	175.37	[illegible]
Chr05	15241968	A	AAT	Chr05_15241A_AAT	INDEL	175.37	[illegible]
Chr05	15241968	A	AAT	Chr05_15241A_AAT	INDEL	175.37	[illegible]
Chr05	15241968	A	AAT	Chr05_15241A_AAT	INDEL	175.37	[illegible]
Chr05	15241968	A	AAT	Chr05_15241A_AAT	INDEL	175.37	[illegible]
Chr05	15241968	A	AAT	Chr05_15241A_AAT	INDEL	175.37	[illegible]
Chr05	15241968	A	AAT	Chr05_15241A_AAT	INDEL	175.37	[illegible]
Chr05	15241968	A	AAT	Chr05_15241A_AAT	INDEL	175.37	[illegible]
Chr05	15241968	A	AAT	Chr05_15241A_AAT	INDEL	175.37	[illegible]
Chr05	15289088	C	CTA	Chr05_15289C_CTA	INDEL	3350.24	[illegible]
Chr05	15345547	G	A	Chr05_15345G_A	SNP	4265.98	[illegible]
Chr05	15426413	A	T	Chr05_15426A_T	SNP	22326.63	[illegible]
Chr05	15426413	A	T	Chr05_15426A_T	SNP	22326.63	[illegible]
Chr05	15426413	A	T	Chr05_15426A_T	SNP	22326.63	[illegible]
Chr05	15426413	A	T	Chr05_15426A_T	SNP	22326.63	[illegible]
Chr05	15426414	G	A	Chr05_15426G_A	SNP	22326.63	[illegible]
Chr05	15426414	G	A	Chr05_15426G_A	SNP	22326.63	[illegible]
Chr05	15426414	G	A	Chr05_15426G_A	SNP	22326.63	[illegible]
Chr05	15426414	G	A	Chr05_15426G_A	SNP	22326.63	[illegible]
Chr05	15511095	A	T	Chr05_15511A_T	SNP	18601.63	[illegible]
Chr05	15522144	A	T	Chr05_15522A_T	SNP	3876.98	[illegible]
Chr05	15681081	A	G	Chr05_15681A_G	SNP	16685.63	[illegible]
Chr05	15721885	A	AT	Chr05_15721A_AT	INDEL	10222.59	[illegible]
Chr05	15721918	CA	C	Chr05_15721CA_C	INDEL	7790.59	[illegible]
Chr05	15721963	GC	G	Chr05_15721GC_G	INDEL	12409.59	[illegible]
Chr05	15721965	TTA	T	Chr05_15721TTA_T	INDEL	12409.59	[illegible]
Chr05	15722029	C	A	Chr05_15722C_A	SNP	18512.63	[illegible]
Chr05	15722050	G	GA	Chr05_15722G_GA	INDEL	19375.59	[illegible]
Chr05	15748980	G	GA	Chr05_15748G_GA	INDEL	16453.59	[illegible]
Chr05	15767590	A	T	Chr05_15767A_T	SNP	20410.63	[illegible]
Chr05	15767590	A	T	Chr05_15767A_T	SNP	20410.63	[illegible]
Chr05	15767590	A	T	Chr05_15767A_T	SNP	20410.63	[illegible]
Chr05	15767590	A	T	Chr05_15767A_T	SNP	20410.63	[illegible]
Chr05	15767590	A	T	Chr05_15767A_T	SNP	20410.63	[illegible]
Chr05	15767642	G	T	Chr05_15767G_T	SNP	20224.63	[illegible]
Chr05	15767642	G	T	Chr05_15767G_T	SNP	20224.63	[illegible]
Chr05	15767642	G	T	Chr05_15767G_T	SNP	20224.63	[illegible]
Chr05	15767642	G	T	Chr05_15767G_T	SNP	20224.63	[illegible]
Chr05	15767642	G	T	Chr05_15767G_T	SNP	20224.63	[illegible]
Chr05	15771417	A	AAT	Chr05_15771A_AAT	INDEL	1507.24	[illegible]
Chr05	15822137	T	C	Chr05_15822T_C	SNP	11124.29	[illegible]
Chr05	15822962	G	A	Chr05_15822G_A	SNP	8220.29	[illegible]
Chr05	15826091	G	T	Chr05_15826G_T	SNP	10000.63	[illegible]
Chr05	15939852	C	CA	Chr05_15939C_CA	INDEL	14757.59	[illegible]
Chr05	15939852	C	CA	Chr05_15939C_CA	INDEL	14757.59	[illegible]
Chr05	15939852	C	CA	Chr05_15939C_CA	INDEL	14757.59	[illegible]
Chr05	15939852	C	CA	Chr05_15939C_CA	INDEL	14757.59	[illegible]
Chr05	15939852	C	CA	Chr05_15939C_CA	INDEL	14757.59	[illegible]
Chr05	15939852	C	CA	Chr05_15939C_CA	INDEL	14757.59	[illegible]
Chr05	16011624	G	T	Chr05_16011G_T	SNP	14809.63	[illegible]
Chr05	16112979	T	TC	Chr05_16112T_TC	INDEL	22016.59	[illegible]
Chr05	16112979	T	TC	Chr05_16112T_TC	INDEL	22016.59	[illegible]
Chr05	16112979	T	TC	Chr05_16112T_TC	INDEL	22016.59	[illegible]
Chr05	16112979	T	TC	Chr05_16112T_TC	INDEL	22016.59	[illegible]
Chr05	16112979	T	TC	Chr05_16112T_TC	INDEL	22016.59	[illegible]
Chr05	16112979	T	TC	Chr05_16112T_TC	INDEL	22016.59	[illegible]
Chr05	16112983	TA	T	Chr05_16112TA_T	INDEL	21807.59	[illegible]
Chr05	16112983	TA	T	Chr05_16112TA_T	INDEL	21807.59	[illegible]
Chr05	16112983	TA	T	Chr05_16112TA_T	INDEL	21807.59	[illegible]
Chr05	16112983	TA	T	Chr05_16112TA_T	INDEL	21807.59	[illegible]
Chr05	16112983	TA	T	Chr05_16112TA_T	INDEL	21807.59	[illegible]
Chr05	16112983	TA	T	Chr05_16112TA_T	INDEL	21807.59	[illegible]
Chr05	16279630	AAT	A	Chr05_16279AAT_A	INDEL	10697.59	[illegible]
Chr05	16279630	AAT	A	Chr05_16279AAT_A	INDEL	10697.59	[illegible]
Chr05	16279630	AAT	A	Chr05_16279AAT_A	INDEL	10697.59	[illegible]
Chr05	16279630	AAT	A	Chr05_16279AAT_A	INDEL	10697.59	[illegible]
Chr05	16288236	G	T	Chr05_16288G_T	SNP	6182.29	[illegible]
Chr05	16288236	G	T	Chr05_16288G_T	SNP	6182.29	[illegible]
Chr05	16288236	G	T	Chr05_16288G_T	SNP	6182.29	[illegible]
Chr05	16288236	G	T	Chr05_16288G_T	SNP	6182.29	[illegible]
Chr05	16351925	AAT	A	Chr05_16351AAT_A	INDEL	12992.59	[illegible]

Chr05	16351925	AAT	A	Chr05_16351	AAT_A	INDEL	12992.59	33	19	13	34	16	27	24	13	16	13	14	5	9
Chr05	16394836	A	T	Chr05_16394	A_T	SNP	20593.63	39	19	17	23	19	26	34	29	32	29	20	17	9
Chr05	16551910	G	GTA	Chr05_16551	G_GTA	INDEL	142.37	0	6	1	1	2	0	1	0	0	1	0	0	0
Chr05	16551910	G	GTA	Chr05_16551	G_GTA	INDEL	142.37	0	6	1	1	2	0	1	0	0	1	0	0	0
Chr05	16551910	G	GTA	Chr05_16551	G_GTA	INDEL	142.37	0	6	1	1	2	0	1	0	0	1	0	0	0
Chr05	16775940	TTA	T	Chr05_16775	TTA_T	INDEL	2685.24	1	0	0	22	12	13	14	20	0	0	0	0	0
Chr05	16775940	TTA	T	Chr05_16775	TTA_T	INDEL	2685.24	1	0	0	22	12	13	14	20	0	0	0	0	0
Chr05	16775940	TTA	T	Chr05_16775	TTA_T	INDEL	2685.24	1	0	0	22	12	13	14	20	0	0	0	0	0
Chr05	16775940	TTA	T	Chr05_16775	TTA_T	INDEL	2685.24	1	0	0	22	12	13	14	20	0	0	0	0	0
Chr05	16775940	TTA	T	Chr05_16775	TTA_T	INDEL	2685.24	1	0	0	22	12	13	14	20	0	0	0	0	0
Chr05	16775940	TTA	T	Chr05_16775	TTA_T	INDEL	2685.24	1	0	0	22	12	13	14	20	0	0	0	0	0
Chr05	16775940	TTA	T	Chr05_16775	TTA_T	INDEL	2685.24	1	0	0	22	12	13	14	20	0	0	0	0	0
Chr05	16775940	TTA	T	Chr05_16775	TTA_T	INDEL	2685.24	1	0	0	22	12	13	14	20	0	0	0	0	0
Chr05	16775940	TTA	T	Chr05_16775	TTA_T	INDEL	2685.24	1	0	0	22	12	13	14	20	0	0	0	0	0
Chr05	16850013	C	A	Chr05_16850	C_A	SNP	12890.63	20	6	8	16	13	10	14	15	10	15	16	10	4
Chr05	16850031	C	A	Chr05_16850	C_A	SNP	13598.63	19	8	10	19	10	9	16	19	9	18	17	12	4
Chr05	16850059	C	A	Chr05_16850	C_A	SNP	14140.63	19	8	11	15	16	12	21	18	14	18	15	13	6
Chr05	16850061	C	A	Chr05_16850	C_A	SNP	14410.63	19	8	11	15	16	13	21	19	13	17	15	14	6
Chr05	16850070	ATGCTG	A	Chr05_16850	ATGCTG_A	INDEL	13562.59	15	10	10	14	13	15	21	17	11	14	15	13	5
Chr05	16850075	G	GAAA	Chr05_16850	G_GAAA	INDEL	13022.59	14	10	10	14	13	15	21	17	11	14	13	13	5
Chr05	16850079	A	AT	Chr05_16850	A_AT	INDEL	12741.59	15	11	11	14	12	12	20	17	11	14	11	13	6
Chr05	16989912	C	T	Chr05_16989	C_T	SNP	19234.63	26	17	22	29	19	32	29	19	21	22	23	16	13
Chr05	16989912	C	T	Chr05_16989	C_T	SNP	19234.63	26	17	22	29	19	32	29	19	21	22	23	16	13

[illegible]

Chr06	307856	A	T	Chr06_30785A_T	SNP	17302.63	25	18	14	24	16	21	28	21	18	12	9	10	11	13
Chr06	307856	A	T	Chr06_30785A_T	SNP	17302.63	25	18	14	24	16	21	28	21	18	12	9	10	11	13
Chr06	307856	A	T	Chr06_30785A_T	SNP	17302.63	25	18	14	24	16	21	28	21	18	12	9	10	11	13
Chr06	307857	T	A	Chr06_30785T_A	SNP	16851.63	24	17	14	24	16	20	29	21	17	12	8	12	10	13
Chr06	307857	T	A	Chr06_30785T_A	SNP	16851.63	24	17	14	24	16	20	29	21	17	12	8	12	10	13
Chr06	307857	T	A	Chr06_30785T_A	SNP	16851.63	24	17	14	24	16	20	29	21	17	12	8	12	10	13
Chr06	307857	T	A	Chr06_30785T_A	SNP	16851.63	24	17	14	24	16	20	29	21	17	12	8	12	10	13
Chr06	307857	T	A	Chr06_30785T_A	SNP	16851.63	24	17	14	24	16	20	29	21	17	12	8	12	10	13
Chr06	307857	T	A	Chr06_30785T_A	SNP	16851.63	24	17	14	24	16	20	29	21	17	12	8	12	10	13
Chr06	307857	T	A	Chr06_30785T_A	SNP	16851.63	24	17	14	24	16	20	29	21	17	12	8	12	10	13
Chr06	307871	A	AT	Chr06_30787A_AT	INDEL	17106.59	24	16	15	24	14	21	27	18	16	12	8	12	9	10
Chr06	307871	A	AT	Chr06_30787A_AT	INDEL	17106.59	24	16	15	24	14	21	27	18	16	12	8	12	9	10
Chr06	307871	A	AT	Chr06_30787A_AT	INDEL	17106.59	24	16	15	24	14	21	27	18	16	12	8	12	9	10
Chr06	307871	A	AT	Chr06_30787A_AT	INDEL	17106.59	24	16	15	24	14	21	27	18	16	12	8	12	9	10
Chr06	307871	A	AT	Chr06_30787A_AT	INDEL	17106.59	24	16	15	24	14	21	27	18	16	12	8	12	9	10
Chr06	307871	A	AT	Chr06_30787A_AT	INDEL	17106.59	24	16	15	24	14	21	27	18	16	12	8	12	9	10
Chr06	307871	A	AT	Chr06_30787A_AT	INDEL	17106.59	24	16	15	24	14	21	27	18	16	12	8	12	9	10
Chr06	307890	A	AT	Chr06_30789A_AT	INDEL	16458.59	27	14	16	19	13	18	34	17	15	10	10	13	8	10
Chr06	307890	A	AT	Chr06_30789A_AT	INDEL	16458.59	27	14	16	19	13	18	34	17	15	10	10	13	8	10
Chr06	307890	A	AT	Chr06_30789A_AT	INDEL	16458.59	27	14	16	19	13	18	34	17	15	10	10	13	8	10
Chr06	307890	A	AT	Chr06_30789A_AT	INDEL	16458.59	27	14	16	19	13	18	34	17	15	10	10	13	8	10
Chr06	307890	A	AT	Chr06_30789A_AT	INDEL	16458.59	27	14	16	19	13	18	34	17	15	10	10	13	8	10
Chr06	307890	A	AT	Chr06_30789A_AT	INDEL	16458.59	27	14	16	19	13	18	34	17	15	10	10	13	8	10
Chr06	307890	A	AT	Chr06_30789A_AT	INDEL	16458.59	27	14	16	19	13	18	34	17	15	10	10	13	8	10

[illegible]

Chr06	3385132	G	C	Chr06_33851G_C	SNP	13804.63	23	12	8	20	14	16	17	25	10	12	13	5	6	4	23	12	8	20	14	16	17	25	10	12	13	5	6	4	1	1	[illegible]
Chr06	3385132	G	C	Chr06_33851G_C	SNP	13804.63	23	12	8	20	14	16	17	25	10	12	13	5	6	4	23	12	8	20	14	16	17	25	10	12	13	5	6	4	1	1	[illegible]
Chr06	3385132	G	C	Chr06_33851G_C	SNP	13804.63	23	12	8	20	14	16	17	25	10	12	13	5	6	4	23	12	8	20	14	16	17	25	10	12	13	5	6	4	1	1	[illegible]
Chr06	3385132	G	C	Chr06_33851G_C	SNP	13804.63	23	12	8	20	14	16	17	25	10	12	13	5	6	4	23	12	8	20	14	16	17	25	10	12	13	5	6	4	1	1	[illegible]
Chr06	3422742	A	C	Chr06_34227A_C	SNP	20607.63	30	17	17	25	26	27	34	26	39	25	15	21	8	12	30	17	17	25	26	27	34	26	39	25	15	21	8	12	1	1	[illegible]
Chr06	3422742	A	C	Chr06_34227A_C	SNP	20607.63	30	17	17	25	26	27	34	26	39	25	15	21	8	12	30	17	17	25	26	27	34	26	39	25	15	21	8	12	1	1	[illegible]
Chr06	3422742	A	C	Chr06_34227A_C	SNP	20607.63	30	17	17	25	26	27	34	26	39	25	15	21	8	12	30	17	17	25	26	27	34	26	39	25	15	21	8	12	1	1	[illegible]
Chr06	3422742	A	C	Chr06_34227A_C	SNP	20607.63	30	17	17	25	26	27	34	26	39	25	15	21	8	12	30	17	17	25	26	27	34	26	39	25	15	21	8	12	1	1	[illegible]
Chr06	3466583	C	A	Chr06_34665C_A	SNP	20317.63	23	14	17	25	14	28	41	33	18	23	28	23	12	17	23	14	17	25	14	28	41	33	18	23	28	23	12	17	1	1	[illegible]
Chr06	3468460	T	TC	Chr06_34684T_TC	INDEL	19472.59	33	14	14	30	27	27	45	16	26	18	24	26	7	12	33	14	14	30	27	27	45	16	26	18	24	26	7	12	1	1	[illegible]
Chr06	3522630	G	GTA	Chr06_35226G_GTA	INDEL	3572.24	0	1	0	15	19	18	24	16	0	0	1	0	0	0	31	8	12	16	19	18	24	16	18	10	6	13	12	8	0	0	[illegible]
Chr06	3522630	G	GTA	Chr06_35226G_GTA	INDEL	3572.24	0	1	0	15	19	18	24	16	0	0	1	0	0	0	31	8	12	16	19	18	24	16	18	10	6	13	12	8	0	0	[illegible]
Chr06	3522630	G	GTA	Chr06_35226G_GTA	INDEL	3572.24	0	1	0	15	19	18	24	16	0	0	1	0	0	0	31	8	12	16	19	18	24	16	18	10	6	13	12	8	0	0	[illegible]
Chr06	3522630	G	GTA	Chr06_35226G_GTA	INDEL	3572.24	0	1	0	15	19	18	24	16	0	0	1	0	0	0	31	8	12	16	19	18	24	16	18	10	6	13	12	8	0	0	[illegible]
Chr06	3528609	T	TG	Chr06_35286T_TG	INDEL	19358.59	28	18	13	32	22	28	22	33	37	22	16	22	7	17	28	18	13	32	22	28	22	33	37	22	16	22	7	17	1	1	[illegible]
Chr06	3528717	C	A	Chr06_35287C_A	SNP	8362.63	19	4	6	10	9	9	10	17	17	11	5	4	6	5	19	4	6	10	9	9	10	17	17	11	5	4	6	5	1	1	[illegible]
Chr06	3537733	T	A	Chr06_35377T_A	SNP	19244.63	25	20	14	28	15	24	36	21	16	17	9	16	14	18	25	20	14	28	15	24	36	21	16	17	10	16	14	18	1	1	[illegible]
Chr06	3537733	T	A	Chr06_35377T_A	SNP	19244.63	25	20	14	28	15	24	36	21	16	17	9	16	14	18	25	20	14	28	15	24	36	21	16	17	10	16	14	18	1	1	[illegible]
Chr06	3537733	T	A	Chr06_35377T_A	SNP	19244.63	25	20	14	28	15	24	36	21	16	17	9	16	14	18	25	20	14	28	15	24	36	21	16	17	10	16	14	18	1	1	[illegible]
Chr06	3547067	C	A	Chr06_35470C_A	SNP	18221.63	27	9	14	25	19	28	20	19	23	18	14	15	12	12	27	9	14	25	19	28	20	19	23	18	14	15	12	12	1	1	[illegible]
Chr06	3547067	C	A	Chr06_35470C_A	SNP	18221.63	27	9	14	25	19	28	20	19	23	18	14	15	12	12	27	9	14	25	19	28	20	19	23	18	14	15	12	12	1	1	[illegible]
Chr06	3547068	A	G	Chr06_35470A_G	SNP	18221.63	27	9	14	25	19	28	20	19	23	18	16	15	12	12	27	9	14	25	19	28	20	19	23	18	16	15	12	12	1	1	[illegible]
Chr06	3547068	A	G	Chr06_35470A_G	SNP	18221.63	27	9	14	25	19	28	20	19	23	18	16	15	12	12	27	9	14	25	19	28	20	19	23	18	16	15	12	12	1	1	[illegible]
Chr06	3553542	C	T	Chr06_35535C_T	SNP	19348.63	33	16	12	21	19	26	34	28	25	28	20	18	14	9	33	16	12	21	19	26	34	28	25	28	20	18	14	9	1	1	[illegible]
Chr06	3553542	C	T	Chr06_35535C_T	SNP	19348.63	33	16	12	21	19	26	34	28	25	28	20	18	14	9	33	16	12	21	19	26	34	28	25	28	20	18	14	9	1	1	[illegible]
Chr06	3553542	C	T	Chr06_35535C_T	SNP	19348.63	33	16	12	21	19	26	34	28	25	28	20	18	14	9	33	16	12	21	19	26	34	28	25	28	20	18	14	9	1	1	[illegible]
Chr06	3569965	A	AAT	Chr06_35699A_AAT	INDEL	1896.24	0	1	0	10	10	7	22	8	0	0	0	0	0	0	20	15	11	10	11	7	22	8	14	13	7	13	5	9	0	0	[illegible]
Chr06	3569965	A	AAT	Chr06_35699A_AAT	INDEL	1896.24	0	1	0	10	10	7	22	8	0	0	0	0	0	0	20	15	11	10	11	7	22	8	14	13	7	13	5	9	0	0	[illegible]
Chr06	3569965	A	AAT	Chr06_35699A_AAT	INDEL	1896.24	0	1	0	10	10	7	22	8	0	0	0	0	0	0	20	15	11	10	11	7	22	8	14	13	7	13	5	9	0	0	[illegible]
Chr06	3569965	A	AAT	Chr06_35699A_AAT	INDEL	1896.24	0	1	0	10	10	7	22	8	0	0	0	0	0	0	20	15	11	10	11	7	22	8	14	13	7	13	5	9	0	0	[illegible]
Chr06	3570175	AAT	AATAT	Chr06_35701AAT_AATAT	INDEL	1627.93	2	0	0	0	1	0	0	0	0	0	0	0	4	0	14	10	7	12	7	13	11	9	9	9	9	6	4	5	0.143	0	[illegible]
Chr06	3570175	AAT	AATAT	Chr06_35701AAT_AATAT	INDEL	1627.93	2	0	0	0	1	0	0	0	0	0	0	0	4	0	14	10	7	12	7	13	11	9	9	9	9	6	4	5	0.143	0	[illegible]
Chr06	3570175	AAT	AATAT	Chr06_35701AAT_AATAT	INDEL	1627.93	2	0	0	0	1	0	0	0	0	0	0	0	4	0	14	10	7	12	7	13	11	9	9	9	9	6	4	5	0.143	0	[illegible]
Chr06	3570175	AAT	AATAT	Chr06_35701AAT_AATAT	INDEL	1627.93	2	0	0	0	1	0	0	0	0	0	0	0	4	0	14	10	7	12	7	13	11	9	9	9	9	6	4	5	0.143	0	[illegible]
Chr06	3588882	A	AT	Chr06_35888A_AT	INDEL	13975.59	30	9	11	27	16	25	22	20	19	13	16	14	6	8	30	9	11	27	16	25	22	20	19	13	16	14	6	8	1	1	[illegible]
Chr06	3588882	A	AT	Chr06_35888A_AT	INDEL	13975.59	30	9	11	27	16	25	22	20	19	13	16	14	6	8	30	9	11	27	16	25	22	20	19	13	16	14	6	8	1	1	[illegible]
Chr06	3588882	A	AT	Chr06_35888A_AT	INDEL	13975.59	30	9	11	27	16	25	22	20	19	13	16	14	6	8	30	9	11	27	16	25	22	20	19	13	16	14	6	8	1	1	[illegible]
Chr06	3588882	A	AT	Chr06_35888A_AT	INDEL	13975.59	30	9	11	27	16	25	22	20	19	13	16	14	6	8	30	9	11	27	16	25	22	20	19	13	16	14	6	8	1	1	[illegible]
Chr06	3594916	T	C	Chr06_35949T_C	SNP	11162.29	26	14	14	0	0	0	0	0	19	18	13	22	7	7	26	14	14	28	20	15	21	16	19	19	13	22	8	7	1	1	[illegible]
Chr06	3610085	A	AAT	Chr06_36100A_AAT	INDEL	2488.24	0	0	0	9	13	17	16	16	0	0	0	0	0	0	27	10	8	9	14	17	16	16	12	13	5	6	7	8	0	0	[illegible]
Chr06	3610085	A	AAT	Chr06_36100A_AAT	INDEL	2488.24	0	0	0	9	13	17	16	16	0	0	0	0	0	0	27	10	8	9	14	17	16	16	12	13	5	6	7	8	0	0	[illegible]
Chr06	3610085	A	AAT	Chr06_36100A_AAT	INDEL	2488.24	0	0	0	9	13	17	16	16	0	0	0	0	0	0	27	10	8	9	14	17	16	16	12	13	5	6	7	8	0	0	[illegible]
Chr06	3610085	A	AAT	Chr06_36100A_AAT	INDEL	2488.24	0	0	0	9	13	17	16	16	0	0	0	0	0	0	27	10	8	9	14	17	16	16	12	13	5	6	7	8	0	0	[illegible]
Chr06	3610085	A	AAT	Chr06_36100A_AAT	INDEL	2488.24	0	0	0	9	13	17	16	16	0	0	0	0	0	0	27	10	8	9	14	17	16	16	12	13	5	6	7	8	0	0	[illegible]
Chr06	3610085	A	AAT	Chr06_36100A_AAT	INDEL	2488.24	0	0	0	9	13	17	16	16	0	0	0	0	0	0	27	10	8	9	14	17	16	16	12	13	5	6	7	8	0	0	[illegible]
Chr06	3610085	A	AAT	Chr06_36100A_AAT	INDEL	2488.24	0	0	0	9	13	17	16	16	0	0	0	0	0	0	27	10	8	9	14	17	16	16	12	13	5	6	7	8	0	0	[illegible]
Chr06	3707522	C	T	Chr06_37075C_T	SNP	21010.63	23	19	23	31	24	19	33	37	21	35	22	17	14	7	23	19	23	31	24	19	33	37	21	35	22	17	14	7	1	1	[illegible]
Chr06	3707522	C	T	Chr06_37075C_T	SNP	21010.63	23	19	23	31	24	19	33	37	21	35	22	17	14	7	23	19	23	31	24	19	33	37	21	35	22	17	14	7	1	1	[illegible]
Chr06	3707522	C	T	Chr06_37075C_T	SNP	21010.63	23	19	23	31	24	19	33	37	21	35	22	17	14	7	23	19	23	31	24	19	33	37	21	35	22	17	14	7	1	1	[illegible]
Chr06	3707522	C	T	Chr06_37075C_T	SNP	21010.63	23	19	23	31	24	19	33	37	21	35	22	17	14	7	23	19	23	31	24	19	33	37	21	35	22	17	14	7	1	1	[illegible]
[illegible]																																					

Chr06	5644105	C	CAT	Chr06_56441C_CAT	INDEL	5588.24	16	9	13	0	0	0
Chr06	5644105	C	CAT	Chr06_56441C_CAT	INDEL	5588.24	16	9	13	0	0	0
Chr06	5644105	C	CAT	Chr06_56441C_CAT	INDEL	5588.24	16	9	13	0	0	0
Chr06	5644105	C	CAT	Chr06_56441C_CAT	INDEL	5588.24	16	9	13	0	0	0
Chr06	5644105	C	CAT	Chr06_56441C_CAT	INDEL	5588.24	16	9	13	0	0	0
Chr06	5644105	C	CAT	Chr06_56441C_CAT	INDEL	5588.24	16	9	13	0	0	0
Chr06	5644105	C	CAT	Chr06_56441C_CAT	INDEL	5588.24	16	9	13	0	0	0
Chr06	5644105	C	CAT	Chr06_56441C_CAT	INDEL	5588.24	16	9	13	0	0	0
Chr06	5644105	C	CAT	Chr06_56441C_CAT	INDEL	5588.24	16	9	13	0	0	0
Chr06	5644105	C	CAT	Chr06_56441C_CAT	INDEL	5588.24	16	9	13	0	0	0
Chr06	5644105	C	CAT	Chr06_56441C_CAT	INDEL	5588.24	16	9	13	0	0	0
Chr06	5739121	T	TTA	Chr06_57391T_TTA	INDEL	267.37	0	0	1	0	10	0
Chr06	5739121	T	TTA	Chr06_57391T_TTA	INDEL	267.37	0	0	1	0	10	0
Chr06	5739121	T	TTA	Chr06_57391T_TTA	INDEL	267.37	0	0	1	0	10	0
Chr06	5739121	T	TTA	Chr06_57391T_TTA	INDEL	267.37	0	0	1	0	10	0
Chr06	5739121	T	TTA	Chr06_57391T_TTA	INDEL	267.37	0	0	1	0	10	0
Chr06	5739121	T	TTA	Chr06_57391T_TTA	INDEL	267.37	0	0	1	0	10	0
Chr06	5966300	A	T	Chr06_59663A_T	SNP	17977.63	33	20	15	31	21	25
Chr06	5966300	A	T	Chr06_59663A_T	SNP	17977.63	33	20	15	31	21	25
Chr06	5966300	A	T	Chr06_59663A_T	SNP	17977.63	33	20	15	31	21	25
Chr06	5966300	A	T	Chr06_59663A_T	SNP	17977.63	33	20	15	31	21	25
Chr06	5966300	A	T	Chr06_59663A_T	SNP	17977.63	33	20	15	31	21	25
Chr06	5966300	A	T	Chr06_59663A_T	SNP	17977.63	33	20	15	31	21	25
Chr06	5966300	A	T	Chr06_59663A_T	SNP	17977.63	33	20	15	31	21	25
[illegible]												

Chr06	8288862	G	T	Chr06_82888	G_T	SNP	15437.63	18	7	11	21	13	26	23	18	20	23	8	9	7	5
Chr06	8288888	T	A	Chr06_82888	T_A	SNP	15977.63	20	8	11	22	14	27	27	19	20	23	11	7	7	9
Chr06	8363764	CTA	C	Chr06_83637	CTA_C	INDEL	3763.24	0	0	0	22	15	18	24	24	0	0	0	0	0	0
Chr06	8439736	A	T	Chr06_84397	A_T	SNP	12915.63	22	13	5	17	10	17	14	14	15	15	8	12	7	10
Chr06	8439842	C	A	Chr06_84398	C_A	SNP	15757.63	23	14	12	16	23	22	24	19	13	26	16	13	10	12
Chr06	8439910	TC	T	Chr06_84399	TC_T	INDEL	16592.59	33	13	14	25	20	37	24	31	15	20	13	10	12	15
Chr06	8463927	A	AAATTCTAA[illegible]	Chr06_84639	A_AAATTCTA[illegible]	INDEL	19295.59	38	15	18	31	21	20	31	16	19	17	5	13	8	9
Chr06	8479740	G	T	Chr06_84797	G_T	SNP	11415.63	16	6	8	13	14	16	21	10	8	12	7	15	5	13
Chr06	8479744	G	T	Chr06_84797	G_T	SNP	11861.63	17	6	8	13	16	16	21	11	8	14	7	15	5	13
Chr06	8479753	C	T	Chr06_84797	C_T	SNP	12199.63	16	7	10	15	19	16	21	16	8	13	7	15	5	13
Chr06	8666246	G	T	Chr06_86662	G_T	SNP	20804.63	27	22	12	18	24	25	38	33	27	30	4	10	23	10
Chr06	8666280	G	T	Chr06_86662	G_T	SNP	19650.63	29	19	13	18	21	27	30	28	26	28	5	9	18	13
Chr06	8696376	A	AAT	Chr06_86963	A_AAT	INDEL	1831.35	18	19	16	0	0	0	1	0	0	0	0	0	0	0
Chr06	8699023	G	T	Chr06_86990	G_T	SNP	23865.63	32	26	13	44	21	33	37	22	20	18	7	16	15	15
Chr06	8699028	G	T	Chr06_86990	G_T	SNP	24059.63	30	27	14	46	23	33	35	23	21	19	8	16	15	14
Chr06	8865209	C	CAT	Chr06_88652	C_CAT	INDEL	319.37	0	0	11	0	0	0	2	0	1	0	0	0	2	0
Chr06	8865209	C	CAT	Chr06_88652	C_CAT	INDEL	319.37	0	0	11	0	0	0	2	0	1	0	0	0	2	0
Chr06	8865209	C	CAT	Chr06_88652	C_CAT	INDEL	319.37	0	0	11	0	0	0	2	0	1	0	0	0	2	0
Chr06	8865209	C	CAT	Chr06_88652	C_CAT	INDEL	319.37	0	0	11	0	0	0	2	0	1	0	0	0	2	0
Chr06	8865209	C	CAT	Chr06_88652	C_CAT	INDEL	319.37	0	0	11	0	0	0	2	0	1	0	0	0	2	0
Chr06	8865209	C	CAT	Chr06_88652	C_CAT	INDEL	319.37	0	0	11	0	0	0	2	0	1	0	0	0	2	0
Chr06	8865209	C	CAT	Chr06_88652	C_CAT	INDEL	319.37	0	0	11	0	0	0	2	0	1	0	0	0	2	0
Chr06	8865209	C	CAT	Chr06_88652	C_CAT	INDEL	319.37	0	0	11	0	0	0	2	0	1	0	0	0	2	0
Chr06	8865209	C	CAT	Chr06_88652	C_CAT	INDEL	319.37	0	0	11	0	0	0	2	0	1	0	0	0	2	0

Chr06	11886632	A	AAT	Chr06_11886A_AAT	INDEL	2797.24	1	0	0	12	17	21	20	11	0	1	0	0	0	1
Chr06	11886632	A	AAT	Chr06_11886A_AAT	INDEL	2797.24	1	0	0	12	17	21	20	11	0	1	0	0	0	1
Chr06	11886632	A	AAT	Chr06_11886A_AAT	INDEL	2797.24	1	0	0	12	17	21	20	11	0	1	0	0	0	1
Chr06	11886632	A	AAT	Chr06_11886A_AAT	INDEL	2797.24	1	0	0	12	17	21	20	11	0	1	0	0	0	1
Chr06	11886632	A	AAT	Chr06_11886A_AAT	INDEL	2797.24	1	0	0	12	17	21	20	11	0	1	0	0	0	1
Chr06	11886632	A	AAT	Chr06_11886A_AAT	INDEL	2797.24	1	0	0	12	17	21	20	11	0	1	0	0	0	1
Chr06	11886632	A	AAT	Chr06_11886A_AAT	INDEL	2797.24	1	0	0	12	17	21	20	11	0	1	0	0	0	1
Chr06	11886632	A	AAT	Chr06_11886A_AAT	INDEL	2797.24	1	0	0	12	17	21	20	11	0	1	0	0	0	1
Chr06	11886632	A	AAT	Chr06_11886A_AAT	INDEL	2797.24	1	0	0	12	17	21	20	11	0	1	0	0	0	1
Chr06	11886632	A	AAT	Chr06_11886A_AAT	INDEL	2797.24	1	0	0	12	17	21	20	11	0	1	0	0	0	1
Chr06	11886632	A	AAT	Chr06_11886A_AAT	INDEL	2797.24	1	0	0	12	17	21	20	11	0	1	0	0	0	1
Chr06	12005450	C	T	Chr06_12005C_T	SNP	17337.63	23	20	16	30	21	17	30	21	27	24	16	15	10	14
Chr06	12200177	C	A	Chr06_12200C_A	SNP	10390.29	18	15	17	0	0	0	0	0	17	24	11	7	12	9
Chr06	12335161	AC	A	Chr06_12335AC_A	INDEL	11976.59	18	14	14	14	12	17	24	15	20	21	8	7	7	8
Chr06	12335161	AC	A	Chr06_12335AC_A	INDEL	11976.59	18	14	14	14	12	17	24	15	20	21	8	7	7	8
Chr06	12335161	AC	A	Chr06_12335AC_A	INDEL	11976.59	18	14	14	14	12	17	24	15	20	21	8	7	7	8
Chr06	12335161	AC	A	Chr06_12335AC_A	INDEL	11976.59	18	14	14	14	12	17	24	15	20	21	8	7	7	8
Chr06	12335161	AC	A	Chr06_12335AC_A	INDEL	11976.59	18	14	14	14	12	17	24	15	20	21	8	7	7	8
Chr06	12335161	AC	A	Chr06_12335AC_A	INDEL	11976.59	18	14	14	14	12	17	24	15	20	21	8	7	7	8
Chr06	12558056	G	T	Chr06_12558G_T	SNP	5569.29	0	0	0	30	20	24	19	28	0	0	0	0	0	0
Chr06	12582805	A	AT	Chr06_12582A_AT	INDEL	15210.59	33	13	21	21	26	30	29	23	20	36	24	18	16	15
Chr06	12707228	A	AT	Chr06_12707A_AT	INDEL	16058.59	26	16	13	34	15	14	34	27	18	22	14	15	11	6
Chr06	12707228	A	AT	Chr06_12707A_AT	INDEL	16058.59	26	16	13	34	15	14	34	27	18	22	14	15	11	6
Chr06	12707228	A	AT	Chr06_12707A_AT	INDEL	16058.59	26	16	13	34	15	14	34	27	18	22	14	15	11	6

[illegible]

Chr06	15640121	T	TC	Chr06_15640	T_TC	INDEL	12968.59	24	13 13 20 20 22 32 20 10 21 8 9 6 8 [illegible]
Chr06	15688872	G	A	Chr06_15688	G_A	SNP	1914.39	23	18 6 0 0 0 0 0 0 0 0 0 0 0 [illegible]
Chr06	16332135	A	AT	Chr06_16332	A_AT	INDEL	11887.59	29	10 14 17 16 23 28 22 23 21 11 17 8 9 [illegible]
Chr06	16535016	G	A	Chr06_16535	G_A	SNP	5620.29	0	0 0 21 16 21 30 21 0 0 0 0 0 0 [illegible]
Chr06	16640762	C	A	Chr06_16640	C_A	SNP	18021.63	26	14 19 23 16 17 29 21 25 19 17 20 11 10 [illegible]
Chr06	16686573	A	C	Chr06_16686	A_C	SNP	15097.63	19	14 14 18 21 28 30 23 14 17 8 11 7 8 [illegible]
Chr06	16686573	A	C	Chr06_16686	A_C	SNP	15097.63	19	14 14 18 21 28 30 23 14 17 8 11 7 8 [illegible]
Chr06	16686573	A	C	Chr06_16686	A_C	SNP	15097.63	19	14 14 18 21 28 30 23 14 17 8 11 7 8 [illegible]
Chr06	16686573	A	C	Chr06_16686	A_C	SNP	15097.63	19	14 14 18 21 28 30 23 14 17 8 11 7 8 [illegible]
Chr06	16686573	A	C	Chr06_16686	A_C	SNP	15097.63	19	14 14 18 21 28 30 23 14 17 8 11 7 8 [illegible]
Chr06	16686573	A	C	Chr06_16686	A_C	SNP	15097.63	19	14 14 18 21 28 30 23 14 17 8 11 7 8 [illegible]
Chr06	16686573	A	C	Chr06_16686	A_C	SNP	15097.63	19	14 14 18 21 28 30 23 14 17 8 11 7 8 [illegible]
Chr06	16686573	A	C	Chr06_16686	A_C	SNP	15097.63	19	14 14 18 21 28 30 23 14 17 8 11 7 8 [illegible]
Chr06	16723052	G	A	Chr06_16723	G_A	SNP	5045.29	0	0 0 17 19 22 25 22 0 0 0 0 0 0 [illegible]
Chr06	17070152	TC	T	Chr06_17070	TC_T	INDEL	14682.59	20	14 10 26 17 19 21 14 22 22 28 20 15 5 [illegible]
Chr06	17213758	A	AAT	Chr06_17213	A_AAT	INDEL	279.37	2	13 1 1 1 2 0 1 2 1 0 1 0 0 [illegible]
Chr06	17213758	A	AAT	Chr06_17213	A_AAT	INDEL	279.37	2	13 1 1 1 2 0 1 2 1 0 1 0 0 [illegible]
Chr06	17213758	A	AAT	Chr06_17213	A_AAT	INDEL	279.37	2	13 1 1 1 2 0 1 2 1 0 1 0 0 [illegible]
Chr06	17213758	A	AAT	Chr06_17213	A_AAT	INDEL	279.37	2	13 1 1 1 2 0 1 2 1 0 1 0 0 [illegible]
Chr06	17502580	A	AAT	Chr06_17502	A_AAT	INDEL	200.37	0	0 8 0 0 1 1 0 0 0 0 0 0 0 [illegible]
Chr06	17502580	A	AAT	Chr06_17502	A_AAT	INDEL	200.37	0	0 8 0 0 1 1 0 0 0 0 0 0 0 [illegible]
Chr06	17502580	A	AAT	Chr06_17502	A_AAT	INDEL	200.37	0	0 8 0 0 1 1 0 0 0 0 0 0 0 [illegible]
Chr06	17502580	A	AAT	Chr06_17502	A_AAT	INDEL	200.37	0	0 8 0 0 1 1 0 0 0 0 0 0 0 [illegible]
Chr06	17502580	A	AAT	Chr06_17502	A_AAT	INDEL	200.37	0	0 8 0 0 1 1 0 0 0 0 0 0 0 [illegible]
Chr06	17502580	A	AAT	Chr06_17502	A_AAT	INDEL	200.37	0	[illegible]
Chr06	17502580	A	AAT	Chr06_17502	A_AAT	INDEL	200.37	0	[illegible]
Chr06	17606430	G	T	Chr06_17606	G_T	SNP	7378.63	11	[illegible]
Chr06	17606430	G	T	Chr06_17606	G_T	SNP	7378.63	11	[illegible]
Chr06	17606430	G	T	Chr06_17606	G_T	SNP	7378.63	11	[illegible]
Chr06	17655233	C	T	Chr06_17655	C_T	SNP	20467.63	27	[illegible]
Chr06	17655233	C	T	Chr06_17655	C_T	SNP	20467.63	27	[illegible]
Chr06	17655329	TG	T	Chr06_17655	TG_T	INDEL	18413.59	31	[illegible]
Chr06	17655329	TG	T	Chr06_17655	TG_T	INDEL	18413.59	31	[illegible]
Chr06	17655361	T	TA	Chr06_17655	T_TA	INDEL	16385.59	31	[illegible]
Chr06	17655361	T	TA	Chr06_17655	T_TA	INDEL	16385.59	31	[illegible]
Chr06	17666037	TA	T	Chr06_17666	TA_T	INDEL	17830.59	35	[illegible]
Chr06	17666142	C	CTT	Chr06_17666	C_CTT	INDEL	20579.59	35	[illegible]
Chr06	17666144	TGG	T	Chr06_17666	TGG_T	INDEL	20579.59	33	[illegible]
Chr06	17666147	G	A	Chr06_17666	G_A	SNP	20045.63	33	[illegible]
Chr06	17666394	AAT	A	Chr06_17666	AAT_A	INDEL	12389.59	31	[illegible]
Chr06	17868797	G	GA	Chr06_17868	G_GA	INDEL	14089.59	25	[illegible]
Chr06	17871841	G	T	Chr06_17871	G_T	SNP	20290.63	32	[illegible]
Chr06	17909880	T	C	Chr06_17909	T_C	SNP	11057.63	12	[illegible]
Chr06	17927810	G	T	Chr06_17927	G_T	SNP	7964.63	10	[illegible]
Chr06	17951220	G	T	Chr06_17951	G_T	SNP	19282.63	19	[illegible]
Chr06	17951230	G	T	Chr06_17951	G_T	SNP	19387.63	21	[illegible]
Chr06	18005175	TG	T	Chr06_18005	TG_T	INDEL	16913.59	26	[illegible]
Chr06	18099265	T	TTA	Chr06_18099	T_TTA	INDEL	2161.93	0	[illegible]
Chr06	18099265	T	TTA	Chr06_18099	T_TTA	INDEL	2161.93	0	[illegible]
Chr06	18099265	T	TTA	Chr06_18099	T_TTA	INDEL	2161.93	0	[illegible]
Chr06	18099265	T	TTA	Chr06_18099	T_TTA	INDEL	2161.93	0	[illegible]
Chr06	18099265	T	TTA	Chr06_18099	T_TTA	INDEL	2161.93	0	[illegible]
Chr06	18099265	T	TTA	Chr06_18099	T_TTA	INDEL	2161.93	0	[illegible]
Chr06	18099265	T	TTA	Chr06_18099	T_TTA	INDEL	2161.93	0	[illegible]
Chr06	18179420	T	C	Chr06_18179	T_C	SNP	16196.63	24	[illegible]
Chr06	18179420	T	C	Chr06_18179	T_C	SNP	16196.63	24	[illegible]
Chr06	18179420	T	C	Chr06_18179	T_C	SNP	16196.63	24	[illegible]
Chr06	18179420	T	C	Chr06_18179	T_C	SNP	16196.63	24	[illegible]
Chr06	18179420	T	C	Chr06_18179	T_C	SNP	16196.63	24	[illegible]
Chr06	18179420	T	C	Chr06_18179	T_C	SNP	16196.63	24	[illegible]
Chr06	18199606	G	A	Chr06_18199	G_A	SNP	33768.63	54	[illegible]
Chr06	18211868	G	GA	Chr06_18211	G_GA	INDEL	49553.59	82	[illegible]
Chr06	18222202	A	G	Chr06_18222	A_G	SNP	24820.63	40	[illegible]
Chr06	18222202	A	G	Chr06_18222	A_G	SNP	24820.63	40	[illegible]
Chr06	18222202	A	G	Chr06_18222	A_G	SNP	24820.63	40	[illegible]
Chr06	18225821	C	T	Chr06_18225	C_T	SNP	17033.63	29	[illegible]
Chr06	18225821	C	T	Chr06_18225	C_T	SNP	17033.63	29	[illegible]
Chr06	18411058	G	GT	Chr06_18411	G_GT	INDEL	15173.59	23	[illegible]
Chr06	18411084	T	G	Chr06_18411	T_G	SNP	19470.63	20	[illegible]
Chr06	18411087	T	A	Chr06_18411	T_A	SNP	19373.63	20	[illegible]
Chr06	18434915	A	G	Chr06_18434	A_G	SNP	18002.63	17	[illegible]
Chr06	18466582	T	A	Chr06_18466	T_A	SNP	19963.63	33	[illegible]
Chr06	18688449	T	TTATATA	Chr06_18688	T_TTATATA	INDEL	14349.59	19	[illegible]
Chr06	18688449	T	TTATATA	Chr06_18688	T_TTATATA	INDEL	14349.59	19	[illegible]
Chr06	18688449	T	TTATATA	Chr06_18688	T_TTATATA	INDEL	14349.59	19	[illegible]
Chr06	18688449	T	TTATATA	Chr06_18688	T_TTATATA	INDEL	14349.59	19	[illegible]
Chr06	18688449	T	TTATATA	Chr06_18688	T_TTATATA	INDEL	14349.59	19	[illegible]
Chr06	18688449	T	TTATATA	Chr06_18688	T_TTATATA	INDEL	14349.59	19	[illegible]
Chr06	18688449	T	TTATATA	Chr06_18688	T_TTATATA	INDEL	14349.59	19	[illegible]
Chr06	18710749	C	CAT	Chr06_18710	C_CAT	INDEL	295.37	0	[illegible]
Chr06	18710749	C	CAT	Chr06_18710	C_CAT	INDEL	295.37	0	[illegible]
Chr06	18710749	C	CAT	Chr06_18710	C_CAT	INDEL	295.37	0	[illegible]
Chr06	18710749	C	CAT	Chr06_18710	C_CAT	INDEL	295.37	0	[illegible]
Chr06	18710749	C	CAT	Chr06_18710	C_CAT	INDEL	295.37	0	[illegible]
Chr06	18710749	C	CAT	Chr06_18710	C_CAT	INDEL	295.37	0	[illegible]
Chr06	18710749	C	CAT	Chr06_18710	C_CAT	INDEL	295.37	0	[illegible]
Chr06	18710749	C	CAT	Chr06_18710	C_CAT	INDEL	295.37	0	[illegible]
Chr06	18710749	C	CAT	Chr06_18710	C_CAT	INDEL	295.37	0	[illegible]
Chr06	18710749	C	CAT	Chr06_18710	C_CAT	INDEL	295.37	0	[illegible]
Chr06	18710749	C	CAT	Chr06_18710	C_CAT	INDEL	295.37	0	[illegible]
Chr06	18710749	C	CAT	Chr06_18710	C_CAT	INDEL	295.37	0	[illegible]
Chr06	18710749	C	CAT	Chr06_18710	C_CAT	INDEL	295.37	0	[illegible]
Chr06	18710749	C	CAT	Chr06_18710	C_CAT	INDEL	295.37	0	[illegible]
Chr06	18710749	C	CAT	Chr06_18710	C_CAT	INDEL	295.37	0	[illegible]
Chr06	18712435	G	GAT	Chr06_18712	G_GAT	INDEL	1838.24	1	[illegible]
Chr06	18712435	G	GAT	Chr06_18712	G_GAT	INDEL	1838.24	1	[illegible]
Chr06	18712435	G	GAT	Chr06_18712	G_GAT	INDEL	1838.24	1	[illegible]
Chr06	18712435	G	GAT	Chr06_18712	G_GAT	INDEL	1838.24	1	[illegible]
Chr06	18712435	G	GAT	Chr06_18712	G_GAT	INDEL	1838.24	1	[illegible]
Chr06	18712435	G	GAT	Chr06_18712	G_GAT	INDEL	1838.24	1	[illegible]
Chr06	18712435	G	GAT	Chr06_18712	G_GAT	INDEL	1838.24	1	[illegible]
Chr06	18712435	G	GAT	Chr06_18712	G_GAT	INDEL	1838.24	1	[illegible]
Chr06	18712435	G	GAT	Chr06_18712	G_GAT	INDEL	1838.24	1	[illegible]
Chr06	18712435	G	GAT	Chr06_18712	G_GAT	INDEL	1838.24	1	[illegible]
Chr06	18712435	G	GAT	Chr06_18712	G_GAT	INDEL	1838.24	1	[illegible]
Chr06	18887533	A	AAT	Chr06_18887	A_AAT	INDEL	1998.5	0	[illegible]
Chr06	18887533	A	AAT	Chr06_18887	A_AAT	INDEL	1998.5	0	[illegible]
Chr06	18887533	A	AAT	Chr06_18887	A_AAT	INDEL	1998.5	0	[illegible]
Chr06	18887533	A	AAT	Chr06_18887	A_AAT	INDEL	1998.5	0	[illegible]
Chr06	18920918	A	AAT	Chr06_18920	A_AAT	INDEL	2486.12	18	[illegible]
Chr06	18920918	A	AAT	Chr06_18920	A_AAT	INDEL	2486.12	18	[illegible]
Chr06	18920918	A	AAT	Chr06_18920	A_AAT	INDEL	2486.12	18	[illegible]
Chr06	18920918	A	AAT	Chr06_18920	A_AAT	INDEL	2486.12	18	[illegible]
Chr06	18920918	A	AAT	Chr06_18920	A_AAT	INDEL	2486.12	18	[illegible]
Chr06	18920918	A	AAT	Chr06_18920	A_AAT	INDEL	2486.12	18	[illegible]
Chr06	19071302	A	AG	Chr06_19071	A_AG	INDEL	16967.59	28	[illegible]
Chr06	19071302	A	AG	Chr06_19071	A_AG	INDEL	16967.59	28	[illegible]
Chr06	19071302	A	AG	Chr06_19071	A_AG	INDEL	16967.59	28	[illegible]
Chr06	19071302	A	AG	Chr06_19071	A_AG	INDEL	16967.59	28	[illegible]
Chr06	19092606	AT	A	Chr06_19092	AT_A	INDEL	15040.59	29	[illegible]
Chr06	19092606	AT	A	Chr06_19092	AT_A	INDEL	15040.59	29	[illegible]
Chr06	19092606	AT	A	Chr06_19092	AT_A	INDEL	15040.59	29	[illegible]
Chr06	19092606	AT	A	Chr06_19092	AT_A	INDEL	15040.59	29	[illegible]
Chr06	19092606	AT	A	Chr06_19092	AT_A	INDEL	15040.59	29	[illegible]
Chr06	19092606	AT	A	Chr06_19092	AT_A	INDEL	15040.59	29	[illegible]
Chr06	19092606	AT	A	Chr06_19092	AT_A	INDEL	15040.59	29	[illegible]
Chr06	19156902	G	A	Chr06_19156	G_A	SNP	5220.29	0	[illegible]
Chr06	19388990	G	T	Chr06_19388	G_T	SNP	11964.63	20	[illegible]
Chr06	19388990	G	T	Chr06_19388	G_T	SNP	11964.63	20	[illegible]
Chr06	19388990	G	T	Chr06_19388	G_T	SNP	11964.63	20	[illegible]
Chr06	19388990	G	T	Chr06_19388	G_T	SNP	11964.63	20	[illegible]
Chr06	19388990	G	T	Chr06_19388	G_T	SNP	11964.63	20	[illegible]
Chr06	19388994	G	T	Chr06_19388	G_T	SNP	12246.63	19	[illegible]
Chr06	19388994	G	T	Chr06_19388	G_T	SNP	12246.63	19	[illegible]
Chr06	19388994	G	T	Chr06_19388	G_T	SNP	12246.63	19	[illegible]
Chr06	19388994	G	T	Chr06_19388	G_T	SNP	12246.63	19	[illegible]
Chr06	19388994	G	T	Chr06_19388	G_T	SNP	12246.63	19	[illegible]
Chr06	19388996	G	T	Chr06_19388	G_T	SNP	12080.63	19	[illegible]
Chr06	19388996	G	T	Chr06_19388	G_T	SNP	12080.63	19	[illegible]
Chr06	19388996	G	T	Chr06_19388	G_T	SNP	12080.63	19	[illegible]
Chr07	304692	A	ATAAT	Chr07_30469	A_ATAAT	INDEL	21566.59	23	[illegible]
Chr07	304692	A	ATAAT	Chr07_30469	A_ATAAT	INDEL	21566.59	23	[illegible]
Chr07	304693	C	A	Chr07_30469	C_A	SNP	21611.63	24	[illegible]
Chr07	304693	C	A	Chr07_30469	C_A	SNP	21611.63	24	[illegible]
Chr07	304696	TCCAG	T	Chr07_30469	TCCAG_T	INDEL	21112.59	25	[illegible]
Chr07	304696	TCCAG	T	Chr07_30469	TCCAG_T	INDEL	21112.59	25	[illegible]
Chr07	486857	A	C	Chr07_48685	A_C	SNP	13385.63	20	[illegible]
Chr07	486857	A	C	Chr07_48685	A_C	SNP	13385.63	20	[illegible]
Chr07	486857	A	C	Chr07_48685	A_C	SNP	13385.63	20	[illegible]
Chr07	486857	A	C	Chr07_48685	A_C	SNP	13385.63	20	[illegible]
Chr07	486857	A	C	Chr07_48685	A_C	SNP	13385.63	20	[illegible]
Chr07	576695	C	A	Chr07_57669	C_A	SNP	21877.63	29	[illegible]
Chr07	576695	C	A	Chr07_57669	C_A	SNP	21877.63	29	[illegible]
Chr07	1163612	G	GAT	Chr07_11636	G_GAT	INDEL	809.35	15	[illegible]
Chr07	1163612	G	GAT	Chr07_11636	G_GAT	INDEL	809.35	15	[illegible]
Chr07	1163612	G	GAT	Chr07_11636	G_GAT	INDEL	809.35	15	[illegible]
Chr07	1163612	G	GAT	Chr07_11636	G_GAT	INDEL	809.35	15	[illegible]
Chr07	1163612	G	GAT	Chr07_11636	G_GAT	INDEL	809.35	15	[illegible]
Chr07	1163612	G	GAT	Chr07_11636	G_GAT	INDEL	809.35	15	[illegible]
Chr07	1163612	G	GAT	Chr07_11636	G_GAT	INDEL	809.35	15	[illegible]
Chr07	1163612	G	GAT	Chr07_11636	G_GAT	INDEL	809.35	15	[illegible]
Chr07	1165231	T	A	Chr07_11652	T_A	SNP	19251.63	36	[illegible]
Chr07	1165327	C	A	Chr07_11653	C_A	SNP	18315.63	29	[illegible]
Chr07	1207639	C	T	Chr07_12076	C_T	SNP	32559.63	44	[illegible]
Chr07	1207639	C	T	Chr07_12076	C_T	SNP	32559.63	44	[illegible]
Chr07	1221417	A	AAT	Chr07_12214	A_AAT	INDEL	9872.59	22	[illegible]
Chr07	1221417	A	AAT	Chr07_12214	A_AAT	INDEL	9872.59	22	[illegible]
Chr07	1221417	A	AAT	Chr07_12214	A_AAT	INDEL	9872.59	22	[illegible]
Chr07	1221417	A	AAT	Chr07_12214	A_AAT	INDEL	9872.59	22	[illegible]
Chr07	1221417	A	AAT	Chr07_12214	A_AAT	INDEL	9872.59	22	[illegible]
Chr07	1302102	A	T	Chr07_13021	A_T	SNP	18162.63	36	[illegible]
Chr07	1302188	GC	G	Chr07_13021	GC_G	INDEL	13999.59	27	[illegible]
Chr07	1304132	A	G	Chr07_13041	A_G	SNP	13262.63	16	[illegible]
Chr07	1354375	T	TA	Chr07_13543	T_TA	INDEL	12638.59	22	[illegible]
Chr07	1701037	C	T	Chr07_17010	C_T	SNP	1975.29	0	[illegible]
Chr07	1701444	C	T	Chr07_17014	C_T	SNP	2137.29	0	[illegible]
Chr07	1732321	G	GAT	Chr07_17323	G_GAT	INDEL	2858.24	0	[illegible]
Chr07	1732321	G	GAT	Chr07_17323	G_GAT	INDEL	2858.24	0	[illegible]
Chr07	1732321	G	GAT	Chr07_17323	G_GAT	INDEL	2858.24	0	[illegible]
Chr07	1732321	G	GAT	Chr07_17323	G_GAT	INDEL	2858.24	0	[illegible]
Chr07	1732321	G	GAT	Chr07_17323	G_GAT	INDEL	2858.24	0	[illegible]
Chr07	1732321	G	GAT	Chr07_17323	G_GAT	INDEL	2858.24	0	[illegible]
Chr07	1732321	G	GAT	Chr07_17323	G_GAT	INDEL	2858.24	0	[illegible]
Chr07	1732321	G	GAT	Chr07_17323	G_GAT	INDEL	2858.24	0	[illegible]
Chr07	1732321	G	GAT	Chr07_17323	G_GAT	INDEL	2858.24	0	[illegible]
Chr07	1748764	T	TTA	Chr07_17487	T_TTA	INDEL	6217.24	25	[illegible]
Chr07	1748764	T	TTA	Chr07_17487	T_TTA	INDEL	6217.24	25	[illegible]
Chr07	1748764	T	TTA	Chr07_17487	T_TTA	INDEL	6217.24	25	[illegible]
Chr07	1748764	T	TTA	Chr07_17487	T_TTA	INDEL	6217.24	25	[illegible]
Chr07	1748764	T	TTA	Chr07_17487	T_TTA	INDEL	6217.24	25	[illegible]
Chr07	1748764	T	TTA	Chr07_17487	T_TTA	INDEL	6217.24	25	[illegible]
Chr07	1748764	T	TTA	Chr07_17487	T_TTA	INDEL	6217.24	25	[illegible]
Chr07	1748764	T	TTA	Chr07_17487	T_TTA	INDEL	6217.24	25	[illegible]
Chr07	1748764	T	TTA	Chr07_17487	T_TTA	INDEL	6217.24	25	[illegible]
Chr07	1748764	T	TTA	Chr07_17487	T_TTA	INDEL	6217.24	25	[illegible]
Chr07	1748764	T	TTA	Chr07_17487	T_TTA	INDEL	6217.24	25	[illegible]
Chr07	1790822	AAT	A	Chr07_17908	AAT_A	INDEL	10028.59	34	[illegible]
Chr07	1790822	AAT	A	Chr07_17908	AAT_A	INDEL	10028.59	34	[illegible]
Chr07	1790822	AAT	A	Chr07_17908	AAT_A	INDEL	10028.59	34	[illegible]
Chr07	1790822	AAT	A	Chr07_17908	AAT_A	INDEL	10028.59	34	[illegible]
Chr07	1790822	AAT	A	Chr07_17908	AAT_A	INDEL	10028.59	34	[illegible]
Chr07	1790822	AAT	A	Chr07_17908	AAT_A	INDEL	10028.59	34	[illegible]

Chr07	1790822	AAT	A	Chr07_17908	AAT_A	INDEL	10028.59	34	12	13	[illegible]
Chr07	1790822	AAT	A	Chr07_17908	AAT_A	INDEL	10028.59	34	12	13	[illegible]
Chr07	1790822	AAT	A	Chr07_17908	AAT_A	INDEL	10028.59	34	12	13	[illegible]
Chr07	1790822	AAT	A	Chr07_17908	AAT_A	INDEL	10028.59	34	12	13	[illegible]
Chr07	1798700	T	TA	Chr07_17987	T_TA	INDEL	25005.59	32	12	24	[illegible]
Chr07	1798700	T	TA	Chr07_17987	T_TA	INDEL	25005.59	32	12	24	[illegible]
Chr07	1798700	T	TA	Chr07_17987	T_TA	INDEL	25005.59	32	12	24	[illegible]
Chr07	1798700	T	TA	Chr07_17987	T_TA	INDEL	25005.59	32	12	24	[illegible]
Chr07	1798700	T	TA	Chr07_17987	T_TA	INDEL	25005.59	32	12	24	[illegible]
Chr07	1798700	T	TA	Chr07_17987	T_TA	INDEL	25005.59	32	12	24	[illegible]
Chr07	1798700	T	TA	Chr07_17987	T_TA	INDEL	25005.59	32	12	24	[illegible]
Chr07	1798700	T	TA	Chr07_17987	T_TA	INDEL	25005.59	32	12	24	[illegible]
Chr07	1798702	T	A	Chr07_17987	T_A	SNP	25014.63	30	12	24	[illegible]
Chr07	1798702	T	A	Chr07_17987	T_A	SNP	25014.63	30	12	24	[illegible]
Chr07	1798702	T	A	Chr07_17987	T_A	SNP	25014.63	30	12	24	[illegible]
Chr07	1798702	T	A	Chr07_17987	T_A	SNP	25014.63	30	12	24	[illegible]
Chr07	1798702	T	A	Chr07_17987	T_A	SNP	25014.63	30	12	24	[illegible]
Chr07	1798702	T	A	Chr07_17987	T_A	SNP	25014.63	30	12	24	[illegible]
Chr07	1798702	T	A	Chr07_17987	T_A	SNP	25014.63	30	12	24	[illegible]
Chr07	1798702	T	A	Chr07_17987	T_A	SNP	25014.63	30	12	24	[illegible]
Chr07	1822692	AT	A	Chr07_18226	AT_A	INDEL	15279.59	28	13	17	[illegible]
Chr07	1822809	C	A	Chr07_18228	C_A	SNP	17357.63	29	18	24	[illegible]
Chr07	1822847	A	AT	Chr07_18228	A_AT	INDEL	18303.59	25	15	20	[illegible]
Chr07	1822855	A	T	Chr07_18228	A_T	SNP	19379.63	27	16	18	[illegible]
Chr07	1822859	AT	A	Chr07_18228	AT_A	INDEL	19002.59	26	16	18	[illegible]
Chr07	1843215	T	A	Chr07_18432	T_A	SNP	10356.63	19	15	5	[illegible]
Chr07	1843215	T	A	Chr07_18432	T_A	SNP	10356.63	19	15	5	[illegible]
Chr07	1843215	T	A	Chr07_18432	T_A	SNP	10356.63	19	15	5	[illegible]
Chr07	1843215	T	A	Chr07_18432	T_A	SNP	10356.63	19	15	5	[illegible]
Chr07	1843215	T	A	Chr07_18432	T_A	SNP	10356.63	19	15	5	[illegible]
Chr07	1904363	G	GGTTTAGGG	Chr07_19043	G_GGTTTAG	INDEL	17920.59	21	11	13	[illegible]
Chr07	1916765	G	GTA	Chr07_19167	G_GTA	INDEL	3249.24	1	0	0	[illegible]
Chr07	1916765	G	GTA	Chr07_19167	G_GTA	INDEL	3249.24	1	0	0	[illegible]
Chr07	1916765	G	GTA	Chr07_19167	G_GTA	INDEL	3249.24	1	0	0	[illegible]
Chr07	1916765	G	GTA	Chr07_19167	G_GTA	INDEL	3249.24	1	0	0	[illegible]
Chr07	1929022	T	TA	Chr07_19290	T_TA	INDEL	11293.59	17	12	13	[illegible]
Chr07	1929066	T	A	Chr07_19290	T_A	SNP	15030.63	18	10	9	[illegible]
Chr07	1929068	T	A	Chr07_19290	T_A	SNP	15030.63	18	10	9	[illegible]
Chr07	2052122	G	GTATATATA	Chr07_20521	G_GTATATA	INDEL	2904.24	0	0	0	[illegible]
Chr07	2052122	G	GTATATATA	Chr07_20521	G_GTATATA	INDEL	2904.24	0	0	0	[illegible]
Chr07	2052122	G	GTATATATA	Chr07_20521	G_GTATATA	INDEL	2904.24	0	0	0	[illegible]
Chr07	2052122	G	GTATATATA	Chr07_20521	G_GTATATA	INDEL	2904.24	0	0	0	[illegible]
Chr07	2052122	G	GTATATATA	Chr07_20521	G_GTATATA	INDEL	2904.24	0	0	0	[illegible]
Chr07	2075280	A	AT	Chr07_20752	A_AT	INDEL	20889.59	24	22	18	[illegible]
Chr07	2075283	G	A	Chr07_20752	G_A	SNP	21439.63	25	22	19	[illegible]
Chr07	2075289	G	A	Chr07_20752	G_A	SNP	21858.63	26	24	21	[illegible]
Chr07	2075290	T	A	Chr07_20752	T_A	SNP	21678.63	27	24	21	[illegible]
Chr07	2083474	G	T	Chr07_20834	G_T	SNP	14772.63	31	11	15	[illegible]
Chr07	2083474	G	T	Chr07_20834	G_T	SNP	14772.63	31	11	15	[illegible]
Chr07	2083474	G	T	Chr07_20834	G_T	SNP	14772.63	31	11	15	[illegible]
Chr07	2273658	G	GT	Chr07_22736	G_GT	INDEL	14477.59	27	12	13	[illegible]
Chr07	2273658	G	GT	Chr07_22736	G_GT	INDEL	14477.59	27	12	13	[illegible]
Chr07	2273658	G	GT	Chr07_22736	G_GT	INDEL	14477.59	27	12	13	[illegible]
Chr07	2273658	G	GT	Chr07_22736	G_GT	INDEL	14477.59	27	12	13	[illegible]
Chr07	2273658	G	GT	Chr07_22736	G_GT	INDEL	14477.59	27	12	13	[illegible]
Chr07	2273658	G	GT	Chr07_22736	G_GT	INDEL	14477.59	27	12	13	[illegible]
Chr07	2363277	A	T	Chr07_23632	A_T	SNP	19777.63	27	21	13	[illegible]
Chr07	2363277	A	T	Chr07_23632	A_T	SNP	19777.63	27	21	13	[illegible]
Chr07	2363277	A	T	Chr07_23632	A_T	SNP	19777.63	27	21	13	[illegible]
Chr07	2363277	A	T	Chr07_23632	A_T	SNP	19777.63	27	21	13	[illegible]
Chr07	2364545	A	AT	Chr07_23645	A_AT	INDEL	20246.59	30	17	13	[illegible]
Chr07	2364545	A	AT	Chr07_23645	A_AT	INDEL	20246.59	30	17	13	[illegible]
Chr07	2364545	A	AT	Chr07_23645	A_AT	INDEL	20246.59	30	17	13	[illegible]
Chr07	2364545	A	AT	Chr07_23645	A_AT	INDEL	20246.59	30	17	13	[illegible]
Chr07	2364548	C	A	Chr07_23645	C_A	SNP	20795.63	32	17	13	[illegible]
Chr07	2364548	C	A	Chr07_23645	C_A	SNP	20795.63	32	17	13	[illegible]
Chr07	2364548	C	A	Chr07_23645	C_A	SNP	20795.63	32	17	13	[illegible]
Chr07	2364548	C	A	Chr07_23645	C_A	SNP	20795.63	32	17	13	[illegible]
Chr07	2364567	T	TA	Chr07_23645	T_TA	INDEL	20936.59	35	17	16	[illegible]
Chr07	2364567	T	TA	Chr07_23645	T_TA	INDEL	20936.59	35	17	16	[illegible]
Chr07	2364567	T	TA	Chr07_23645	T_TA	INDEL	20936.59	35	17	16	[illegible]
Chr07	2364567	T	TA	Chr07_23645	T_TA	INDEL	20936.59	35	17	16	[illegible]
Chr07	2364570	A	AT	Chr07_23645	A_AT	INDEL	20926.59	35	15	16	[illegible]
Chr07	2364570	A	AT	Chr07_23645	A_AT	INDEL	20926.59	35	15	16	[illegible]
Chr07	2364570	A	AT	Chr07_23645	A_AT	INDEL	20926.59	35	15	16	[illegible]
Chr07	2364570	A	AT	Chr07_23645	A_AT	INDEL	20926.59	35	15	16	[illegible]
Chr07	2364575	AT	A	Chr07_23645	AT_A	INDEL	20527.59	35	16	18	[illegible]
Chr07	2364575	AT	A	Chr07_23645	AT_A	INDEL	20527.59	35	16	18	[illegible]
Chr07	2364575	AT	A	Chr07_23645	AT_A	INDEL	20527.59	35	16	18	[illegible]
Chr07	2364575	AT	A	Chr07_23645	AT_A	INDEL	20527.59	35	16	18	[illegible]
Chr07	2417085	CTA	C	Chr07_24170	CTA_C	INDEL	8287.59	19	10	12	[illegible]
Chr07	2417085	CTA	C	Chr07_24170	CTA_C	INDEL	8287.59	19	10	12	[illegible]
Chr07	2417085	CTA	C	Chr07_24170	CTA_C	INDEL	8287.59	19	10	12	[illegible]
Chr07	2417085	CTA	C	Chr07_24170	CTA_C	INDEL	8287.59	19	10	12	[illegible]
Chr07	2417085	CTA	C	Chr07_24170	CTA_C	INDEL	8287.59	19	10	12	[illegible]
Chr07	2417085	CTA	C	Chr07_24170	CTA_C	INDEL	8287.59	19	10	12	[illegible]
Chr07	2417085	CTA	C	Chr07_24170	CTA_C	INDEL	8287.59	19	10	12	[illegible]
Chr07	2417085	CTA	C	Chr07_24170	CTA_C	INDEL	8287.59	19	10	12	[illegible]
Chr07	2417085	CTA	C	Chr07_24170	CTA_C	INDEL	8287.59	19	10	12	[illegible]
Chr07	2471919	C	CAT	Chr07_24719	C_CAT	INDEL	1256.35	21	12	14	[illegible]
Chr07	2471919	C	CAT	Chr07_24719	C_CAT	INDEL	1256.35	21	12	14	[illegible]
Chr07	2471919	C	CAT	Chr07_24719	C_CAT	INDEL	1256.35	21	12	14	[illegible]
Chr07	2471919	C	CAT	Chr07_24719	C_CAT	INDEL	1256.35	21	12	14	[illegible]
Chr07	2471919	C	CAT	Chr07_24719	C_CAT	INDEL	1256.35	21	12	14	[illegible]
Chr07	2564281	G	C	Chr07_25642	G_C	SNP	11377.63	14	8	10	[illegible]
Chr07	2564292	T	A	Chr07_25642	T_A	SNP	10440.63	11	8	9	[illegible]
Chr07	2564310	T	A	Chr07_25643	T_A	SNP	8785.63	9	5	5	[illegible]
Chr07	2564443	C	A	Chr07_25644	C_A	SNP	18064.63	31	19	11	[illegible]
Chr07	2568467	G	T	Chr07_25684	G_T	SNP	19442.63	26	15	17	[illegible]
Chr07	2675818	G	T	Chr07_26758	G_T	SNP	18201.63	33	18	11	[illegible]
Chr07	2725096	AAT	A	Chr07_27250	AAT_A	INDEL	3449.24	0	1	0	[illegible]
Chr07	2725096	AAT	A	Chr07_27250	AAT_A	INDEL	3449.24	0	1	0	[illegible]
Chr07	2725096	AAT	A	Chr07_27250	AAT_A	INDEL	3449.24	0	1	0	[illegible]
Chr07	2752690	G	T	Chr07_27526	G_T	SNP	19791.63	25	9	15	[illegible]
Chr07	2752690	G	T	Chr07_27526	G_T	SNP	19791.63	25	9	15	[illegible]
Chr07	2752690	G	T	Chr07_27526	G_T	SNP	19791.63	25	9	15	[illegible]
Chr07	2752690	G	T	Chr07_27526	G_T	SNP	19791.63	25	9	15	[illegible]
Chr07	2752691	A	T	Chr07_27526	A_T	SNP	19791.63	25	9	14	[illegible]
Chr07	2752691	A	T	Chr07_27526	A_T	SNP	19791.63	25	9	14	[illegible]
Chr07	2752691	A	T	Chr07_27526	A_T	SNP	19791.63	25	9	14	[illegible]
Chr07	2752691	A	T	Chr07_27526	A_T	SNP	19791.63	25	9	14	[illegible]
Chr07	2766361	A	G	Chr07_27663	A_G	SNP	14021.63	12	10	10	[illegible]
Chr07	2766446	A	G	Chr07_27664	A_G	SNP	16922.63	27	13	16	[illegible]
Chr07	2766487	T	A	Chr07_27664	T_A	SNP	17799.63	24	13	15	[illegible]
Chr07	2811279	G	A	Chr07_28112	G_A	SNP	14507.63	24	10	13	[illegible]
Chr07	2811279	G	A	Chr07_28112	G_A	SNP	14507.63	24	10	13	[illegible]
Chr07	2811279	G	A	Chr07_28112	G_A	SNP	14507.63	24	10	13	[illegible]
Chr07	2811279	G	A	Chr07_28112	G_A	SNP	14507.63	24	10	13	[illegible]
Chr07	2811279	G	A	Chr07_28112	G_A	SNP	14507.63	24	10	13	[illegible]
Chr07	2891196	A	AAT	Chr07_28911	A_AAT	INDEL	7502.59	20	11	8	[illegible]
Chr07	2891196	A	AAT	Chr07_28911	A_AAT	INDEL	7502.59	20	11	8	[illegible]
Chr07	2891196	A	AAT	Chr07_28911	A_AAT	INDEL	7502.59	20	11	8	[illegible]
Chr07	2891196	A	AAT	Chr07_28911	A_AAT	INDEL	7502.59	20	11	8	[illegible]
Chr07	2891196	A	AAT	Chr07_28911	A_AAT	INDEL	7502.59	20	11	8	[illegible]
Chr07	2891196	A	AAT	Chr07_28911	A_AAT	INDEL	7502.59	20	11	8	[illegible]
Chr07	2917923	A	AAT	Chr07_29179	A_AAT	INDEL	4382.24	14	11	11	[illegible]
Chr07	2928503	A	AT	Chr07_29285	A_AT	INDEL	15013.59	22	15	11	[illegible]
Chr07	2928503	A	AT	Chr07_29285	A_AT	INDEL	15013.59	22	15	11	[illegible]
Chr07	3045419	C	CT	Chr07_30454	C_CT	INDEL	19045.59	29	17	14	[illegible]
Chr07	3045435	G	GA	Chr07_30454	G_GA	INDEL	19488.59	33	15	13	[illegible]
Chr07	3045513	G	T	Chr07_30455	G_T	SNP	20239.63	39	18	14	[illegible]
Chr07	3048011	C	A	Chr07_30480	C_A	SNP	21035.63	30	14	19	[illegible]
Chr07	3048026	C	A	Chr07_30480	C_A	SNP	22876.63	35	15	23	[illegible]
Chr07	3170068	T	TTA	Chr07_31700	T_TTA	INDEL	13604.59	27	21	14	[illegible]
Chr07	3170068	T	TTA	Chr07_31700	T_TTA	INDEL	13604.59	27	21	14	[illegible]
Chr07	3190132	T	G	Chr07_31901	T_G	SNP	212610.63	265	426	273	[illegible]
Chr07	3190132	T	G	Chr07_31901	T_G	SNP	212610.63	265	426	273	[illegible]
Chr07	3190132	T	G	Chr07_31901	T_G	SNP	212610.63	265	426	273	[illegible]
Chr07	3190132	T	G	Chr07_31901	T_G	SNP	212610.63	265	426	273	[illegible]
Chr07	3190132	T	G	Chr07_31901	T_G	SNP	212610.63	265	426	273	[illegible]
Chr07	3190132	T	G	Chr07_31901	T_G	SNP	212610.63	265	426	273	[illegible]
Chr07	3190132	T	G	Chr07_31901	T_G	SNP	212610.63	265	426	273	[illegible]
Chr07	3190132	T	G	Chr07_31901	T_G	SNP	212610.63	265	426	273	[illegible]
Chr07	3239673	A	T	Chr07_32396	A_T	SNP	15755.63	19	16	12	[illegible]
Chr07	3239673	A	T	Chr07_32396	A_T	SNP	15755.63	19	16	12	[illegible]
Chr07	3262944	G	A	Chr07_32629	G_A	SNP	13262.63	11	18	16	[illegible]
Chr07	3262944	G	A	Chr07_32629	G_A	SNP	13262.63	11	18	16	[illegible]
Chr07	3262944	G	A	Chr07_32629	G_A	SNP	13262.63	11	18	16	[illegible]
Chr07	3262944	G	A	Chr07_32629	G_A	SNP	13262.63	11	18	16	[illegible]
Chr07	3262944	G	A	Chr07_32629	G_A	SNP	13262.63	11	18	16	[illegible]
Chr07	3262944	G	A	Chr07_32629	G_A	SNP	13262.63	11	18	16	[illegible]
Chr07	3312701	G	GA	Chr07_33127	G_GA	INDEL	17234.59	31	20	14	[illegible]
Chr07	3312701	G	GA	Chr07_33127	G_GA	INDEL	17234.59	31	20	14	[illegible]
Chr07	3312701	G	GA	Chr07_33127	G_GA	INDEL	17234.59	31	20	14	[illegible]
Chr07	3312701	G	GA	Chr07_33127	G_GA	INDEL	17234.59	31	20	14	[illegible]
Chr07	3312701	G	GA	Chr07_33127	G_GA	INDEL	17234.59	31	20	14	[illegible]
Chr07	3351468	C	A	Chr07_33514	C_A	SNP	14140.63	16	19	13	[illegible]
Chr07	3351468	C	A	Chr07_33514	C_A	SNP	14140.63	16	19	13	[illegible]
Chr07	3351468	C	A	Chr07_33514	C_A	SNP	14140.63	16	19	13	[illegible]
Chr07	3351468	C	A	Chr07_33514	C_A	SNP	14140.63	16	19	13	[illegible]
Chr07	3472369	T	TTA	Chr07_34723	T_TTA	INDEL	317.37	0	0	11	[illegible]
Chr07	3472369	T	TTA	Chr07_34723	T_TTA	INDEL	317.37	0	0	11	[illegible]
Chr07	3472369	T	TTA	Chr07_34723	T_TTA	INDEL	317.37	0	0	11	[illegible]
Chr07	3472369	T	TTA	Chr07_34723	T_TTA	INDEL	317.37	0	0	11	[illegible]
Chr07	3472369	T	TTA	Chr07_34723	T_TTA	INDEL	317.37	0	0	11	[illegible]
Chr07	3472369	T	TTA	Chr07_34723	T_TTA	INDEL	317.37	0	0	11	[illegible]
Chr07	3573325	AAT	A	Chr07_35733	AAT_A	INDEL	9972.59	21	8	6	[illegible]
Chr07	3573325	AAT	A	Chr07_35733	AAT_A	INDEL	9972.59	21	8	6	[illegible]
Chr07	3573325	AAT	A	Chr07_35733	AAT_A	INDEL	9972.59	21	8	6	[illegible]
Chr07	3573325	AAT	A	Chr07_35733	AAT_A	INDEL	9972.59	21	8	6	[illegible]
Chr07	3573325	AAT	A	Chr07_35733	AAT_A	INDEL	9972.59	21	8	6	[illegible]
Chr07	3573325	AAT	A	Chr07_35733	AAT_A	INDEL	9972.59	21	8	6	[illegible]
Chr07	3573325	AAT	A	Chr07_35733	AAT_A	INDEL	9972.59	21	8	6	[illegible]
Chr07	3573325	AAT	A	Chr07_35733	AAT_A	INDEL	9972.59	21	8	6	[illegible]
Chr07	3573325	AAT	A	Chr07_35733	AAT_A	INDEL	9972.59	21	8	6	[illegible]
Chr07	3573325	AAT	A	Chr07_35733	AAT_A	INDEL	9972.59	21	8	6	[illegible]
Chr07	3573325	AAT	A	Chr07_35733	AAT_A	INDEL	9972.59	21	8	6	[illegible]
Chr07	3573325	AAT	A	Chr07_35733	AAT_A	INDEL	9972.59	21	8	6	[illegible]
Chr07	3573325	AAT	A	Chr07_35733	AAT_A	INDEL	9972.59	21	8	6	[illegible]
Chr07	3573325	AAT	A	Chr07_35733	AAT_A	INDEL	9972.59	21	8	6	[illegible]
Chr07	3573325	AAT	A	Chr07_35733	AAT_A	INDEL	9972.59	21	8	6	[illegible]
Chr07	3601460	A	T	Chr07_36014	A_T	SNP	18258.63	23	11	15	[illegible]
Chr07	3637114	TA	T	Chr07_36371	TA_T	INDEL	11933.59	14	13	12	[illegible]
Chr07	3637114	TA	T	Chr07_36371	TA_T	INDEL	11933.59	14	13	12	[illegible]
Chr07	3637114	TA	T	Chr07_36371	TA_T	INDEL	11933.59	14	13	12	[illegible]
Chr07	3637122	A	T	Chr07_36371	A_T	SNP	12337.63	15	13	15	[illegible]
Chr07	3637122	A	T	Chr07_36371	A_T	SNP	12337.63	15	13	15	[illegible]
Chr07	3637122	A	T	Chr07_36371	A_T	SNP	12337.63	15	13	15	[illegible]
Chr07	3638089	G	T	Chr07_36380	G_T	SNP	25122.63	44	13	18	[illegible]
Chr07	3638109	G	A	Chr07_36381	G_A	SNP	24351.63	43	12	18	[illegible]
Chr07	3639728	C	A	Chr07_36397	C_A	SNP	21367.63	27	22	11	[illegible]
Chr07	3752388	G	A	Chr07_37523	G_A	SNP	19553.63	35	21	9	[illegible]
Chr07	3757082	A	AAT	Chr07_37570	A_AAT	INDEL	11902.59	22	11	6	[illegible]
Chr07	3841961	G	A	Chr07_38419	G_A	SNP	3804.98	0	0	0	[illegible]
Chr07	3841961	G	A	Chr07_38419	G_A	SNP	3804.98	0	0	0	[illegible]
Chr07	3841961	G	A	Chr07_38419	G_A	SNP	3804.98	0	0	0	[illegible]
Chr07	3884352	C	CAT	Chr07_38843	C_CAT	INDEL	917.35	20	9	4	[illegible]
Chr07	4012387	C	CA	Chr07_40123	C_CA	INDEL	19929.59	40	22	18	[illegible]
Chr07	4012387	C	CA	Chr07_40123	C_CA	INDEL	19929.59	40	22	18	[illegible]
Chr07	4012387	C	CA	Chr07_40123	C_CA	INDEL	19929.59	40	22	18	[illegible]
Chr07	4012387	C	CA	Chr07_40123	C_CA	INDEL	19929.59	40	22	18	[illegible]
Chr07	4012387	C	CA	Chr07_40123	C_CA	INDEL	19929.59	40	22	18	[illegible]

Chr07	4012387	C	CA	Chr07_40123	C_CA	INDEL	19929.59	40	22	18	33	24	21	33	28	21	37	20	15	15	10 [illegible]
Chr07	4012387	C	CA	Chr07_40123	C_CA	INDEL	19929.59	40	22	18	33	24	21	33	28	21	37	20	15	15	10 [illegible]
Chr07	4012387	C	CA	Chr07_40123	C_CA	INDEL	19929.59	40	22	18	33	24	21	33	28	21	37	20	15	15	10 [illegible]
Chr07	4172282	GTATAAATC	G	Chr07_41722	GTATAAATC	INDEL	16127.59	19	14	16	23	16	22	23	18	15	26	13	10	4	9 [illegible]
Chr07	4174238	C	A	Chr07_41742	C_A	SNP	18756.63	24	7	9	19	23	22	28	31	18	22	15	20	8	10 [illegible]
Chr07	4174238	C	A	Chr07_41742	C_A	SNP	18756.63	24	7	9	19	23	22	28	31	18	22	15	20	8	10 [illegible]
Chr07	4174238	C	A	Chr07_41742	C_A	SNP	18756.63	24	7	9	19	23	22	28	31	18	22	15	20	8	10 [illegible]
Chr07	4217886	C	A	Chr07_42178	C_A	SNP	16728.63	23	13	13	29	18	24	28	31	7	18	17	14	10	10 [illegible]
Chr07	4309820	G	A	Chr07_43098	G_A	SNP	18363.63	20	17	12	25	14	36	17	22	26	25	15	16	9	15 [illegible]
Chr07	4312186	T	TTATA	Chr07_43121	T_TTATA	INDEL	137.37	0	0	1	0	0	0	1	0	0	0	0	0	0	4 [illegible]
Chr07	4410261	A	AAT	Chr07_44102	A_AAT	INDEL	2524.24	1	0	0	15	9	14	16	20	0	0	0	0	0	0 [illegible]
Chr07	4410261	A	AAT	Chr07_44102	A_AAT	INDEL	2524.24	1	0	0	15	9	14	16	20	0	0	0	0	0	0 [illegible]
Chr07	4410261	A	AAT	Chr07_44102	A_AAT	INDEL	2524.24	1	0	0	15	9	14	16	20	0	0	0	0	0	0 [illegible]
Chr07	4743302	G	T	Chr07_47433	G_T	SNP	13862.63	20	5	10	16	13	13	11	11	20	18	18	7	10	7 [illegible]
Chr07	4743302	G	T	Chr07_47433	G_T	SNP	13862.63	20	5	10	16	13	13	11	11	20	18	18	7	10	7 [illegible]
Chr07	4743302	G	T	Chr07_47433	G_T	SNP	13862.63	20	5	10	16	13	13	11	11	20	18	18	7	10	7 [illegible]
Chr07	4743302	G	T	Chr07_47433	G_T	SNP	13862.63	20	5	10	16	13	13	11	11	20	18	18	7	10	7 [illegible]
Chr07	4743302	G	T	Chr07_47433	G_T	SNP	13862.63	20	5	10	16	13	13	11	11	20	18	18	7	10	7 [illegible]
Chr07	4743312	G	T	Chr07_47433	G_T	SNP	15263.63	24	5	11	18	14	13	12	12	20	17	18	10	11	7 [illegible]
Chr07	4743312	G	T	Chr07_47433	G_T	SNP	15263.63	24	5	11	18	14	13	12	12	20	17	18	10	11	7 [illegible]
Chr07	4743312	G	T	Chr07_47433	G_T	SNP	15263.63	24	5	11	18	14	13	12	12	20	17	18	10	11	7 [illegible]
Chr07	4743312	G	T	Chr07_47433	G_T	SNP	15263.63	24	5	11	18	14	13	12	12	20	17	18	10	11	7 [illegible]
Chr07	4743312	G	T	Chr07_47433	G_T	SNP	15263.63	24	5	11	18	14	13	12	12	20	17	18	10	11	7 [illegible]
Chr07	4743337	G	T	Chr07_47433	G_T	SNP	17599.63	30	6	12	18	13	18	15	10	23	18	19	14	12	9 [illegible]
[illegible]																					

Chr07	7992881	T	C	Chr07_79928T_C	SNP	11461.63	20	8	8	7	22	11	23	12	14	13	11	14	7	6	20	8	8	7	22	11	23	12	14	13	11	14	7	6	1	1	1	1	1	1	1	1	1	1	1	1	1	1	0	15	15	0	7	5	3	upstream_ge	MODIFIER	Pp3c7_1187	Pp3c7_1187	transcript	PAC:3292365	protein_codi	NA	c.-4974T>C	NA	NA	NA	NA	3553
Chr07	7992881	T	C	Chr07_79928T_C	SNP	11461.63	20	8	8	7	22	11	23	12	14	13	11	14	7	6	20	8	8	7	22	11	23	12	14	13	11	14	7	6	1	1	1	1	1	1	1	1	1	1	1	1	1	1	0	15	15	0	7	5	3	upstream_ge	MODIFIER	Pp3c7_1187	Pp3c7_1187	transcript	PAC:3292364	protein_codi	NA	c.-4974T>C	NA	NA	NA	NA	3553
Chr07	7992881	T	C	Chr07_79928T_C	SNP	11461.63	20	8	8	7	22	11	23	12	14	13	11	14	7	6	20	8	8	7	22	11	23	12	14	13	11	14	7	6	1	1	1	1	1	1	1	1	1	1	1	1	1	1	0	15	15	0	7	5	3	upstream_ge	MODIFIER	Pp3c7_1187	Pp3c7_1187	transcript	PAC:3292365	protein_codi	NA	c.-4974T>C	NA	NA	NA	NA	3553
Chr07	7992881	T	C	Chr07_79928T_C	SNP	11461.63	20	8	8	7	22	11	23	12	14	13	11	14	7	6	20	8	8	7	22	11	23	12	14	13	11	14	7	6	1	1	1	1	1	1	1	1	1	1	1	1	1	1	0	15	15	0	7	5	3	upstream_ge	MODIFIER	Pp3c7_1187	Pp3c7_1187	transcript	PAC:3292365	protein_codi	NA	c.-4974T>C	NA	NA	NA	NA	4617
Chr07	7992881	T	C	Chr07_79928T_C	SNP	11461.63	20	8	8	7	22	11	23	12	14	13	11	14	7	6	20	8	8	7	22	11	23	12	14	13	11	14	7	6	1	1	1	1	1	1	1	1	1	1	1	1	1	1	0	15	15	0	7	5	3	intron_variar	MODIFIER	Pp3c7_1186	Pp3c7_1186	transcript	PAC:3292496	protein_codi	04-sept	c.649-135T>	NA	NA	NA	NA	NA
Chr07	7992881	T	C	Chr07_79928T_C	SNP	11461.63	20	8	8	7	22	11	23	12	14	13	11	14	7	6	20	8	8	7	22	11	23	12	14	13	11	14	7	6	1	1	1	1	1	1	1	1	1	1	1	1	1	1	0	15	15	0	7	5	3	intron_variar	MODIFIER	Pp3c7_1186	Pp3c7_1186	transcript	PAC:3292496	protein_codi	04-sept	c.649-135T>	NA	NA	NA	NA	NA
Chr07	7992881	T	C	Chr07_79928T_C	SNP	11461.63	20	8	8	7	22	11	23	12	14	13	11	14	7	6	20	8	8	7	22	11	23	12	14	13	11	14	7	6	1	1	1	1	1	1	1	1	1	1	1	1	1	1	0	15	15	0	7	5	3	intron_variar	MODIFIER	Pp3c7_1186	Pp3c7_1186	transcript	PAC:3292496	protein_codi	03-août	c.382-135T>	NA	NA	NA	NA	NA
Chr07	7992905	C	A	Chr07_79929C_A	SNP	12064.63	18	4	7	11	16	13	25	12	11	13	12	14	8	6	18	4	7	11	16	13	25	12	11	13	12	14	8	6	1	1	1	1	1	1	1	1	1	1	1	1	1	1	0	15	15	0	7	5	3	upstream_ge	MODIFIER	Pp3c7_1187	Pp3c7_1187	transcript	PAC:3292364	protein_codi	NA	c.-4950C>A	NA	NA	NA	NA	3731
Chr07	7992905	C	A	Chr07_79929C_A	SNP	12064.63	18	4	7	11	16	13	25	12	11	13	12	14	8	6	18	4	7	11	16	13	25	12	11	13	12	14	8	6	1	1	1	1	1	1	1	1	1	1	1	1	1	1	0	15	15	0	7	5	3	upstream_ge	MODIFIER	Pp3c7_1187	Pp3c7_1187	transcript	PAC:3292364	protein_codi	NA	c.-4950C>A	NA	NA	NA	NA	3529
Chr07	7992905	C	A	Chr07_79929C_A	SNP	12064.63	18	4	7	11	16	13	25	12	11	13	12	14	8	6	18	4	7	11	16	13	25	12	11	13	12	14	8	6	1	1	1	1	1	1	1	1	1	1	1	1	1	1	0	15	15	0	7	5	3	upstream_ge	MODIFIER	Pp3c7_1187	Pp3c7_1187	transcript	PAC:3292365	protein_codi	NA	c.-4950C>A	NA	NA	NA	NA	3529
Chr07	7992905	C	A	Chr07_79929C_A	SNP	12064.63	18	4	7	11	16	13	25	12	11	13	12	14	8	6	18	4	7	11	16	13	25	12	11	13	12	14	8	6	1	1	1	1	1	1	1	1	1	1	1	1	1	1	0	15	15	0	7	5	3	upstream_ge	MODIFIER	Pp3c7_1187	Pp3c7_1187	transcript	PAC:3292365	protein_codi	NA	c.-4950C>A	NA	NA	NA	NA	3529
Chr07	7992905	C	A	Chr07_79929C_A	SNP	12064.63	18	4	7	11	16	13	25	12	11	13	12	14	8	6	18	4	7	11	16	13	25	12	11	13	12	14	8	6	1	1	1	1	1	1	1	1	1	1	1	1	1	1	0	15	15	0	7	5	3	upstream_ge	MODIFIER	Pp3c7_1187	Pp3c7_1187	transcript	PAC:3292364	protein_codi	NA	c.-4950C>A	NA	NA	NA	NA	3529
Chr07	7992905	C	A	Chr07_79929C_A	SNP	12064.63	18	4	7	11	16	13	25	12	11	13	12	14	8	6	18	4	7	11	16	13	25	12	11	13	12	14	8	6	1	1	1	1	1	1	1	1	1	1	1	1	1	1	0	15	15	0	7	5	3	upstream_ge	MODIFIER	Pp3c7_1187	Pp3c7_1187	transcript	PAC:3292365	protein_codi	NA	c.-4950C>A	NA	NA	NA	NA	3529
Chr07	7992905	C	A	Chr07_79929C_A	SNP	12064.63	18	4	7	11	16	13	25	12	11	13	12	14	8	6	18	4	7	11	16	13	25	12	11	13	12	14	8	6	1	1	1	1	1	1	1	1	1	1	1	1	1	1	0	15	15	0	7	5	3	upstream_ge	MODIFIER	Pp3c7_1187	Pp3c7_1187	transcript	PAC:3292365	protein_codi	NA	c.-4950C>A	NA	NA	NA	NA	4593
Chr07	7992905	C	A	Chr07_79929C_A	SNP	12064.63	18	4	7	11	16	13	25	12	11	13	12	14	8	6	18	4	7	11	16	13	25	12	11	13	12	14	8	6	1	1	1	1	1	1	1	1	1	1	1	1	1	1	0	15	15	0	7	5	3	intron_variar	MODIFIER	Pp3c7_1186	Pp3c7_1186	transcript	PAC:3292496	protein_codi	04-sept	c.649-111C>	NA	NA	NA	NA	NA
Chr07	7992905	C	A	Chr07_79929C_A	SNP	12064.63	18	4	7	11	16	13	25	12	11	13	12	14	8	6	18	4	7	11	16	13	25	12	11	13	12	14	8	6	1	1	1	1	1	1	1	1	1	1	1	1	1	1	0	15	15	0	7	5	3	intron_variar	MODIFIER	Pp3c7_1186	Pp3c7_1186	transcript	PAC:3292496	protein_codi	04-sept	c.649-111C>	NA	NA	NA	NA	NA
Chr07	7992905	C	A	Chr07_79929C_A	SNP	12064.63	18	4	7	11	16	13	25	12	11	13	12	14	8	6	18	4	7	11	16	13	25	12	11	13	12	14	8	6	1	1	1	1	1	1	1	1	1	1	1	1	1	1	0	15	15	0	7	5	3	intron_variar	MODIFIER	Pp3c7_1186	Pp3c7_1186	transcript	PAC:3292496	protein_codi	03-août	c.382-111C>	NA	NA	NA	NA	NA
Chr07	7992906	C	G	Chr07_79929C_G	SNP	12064.63	18	4	7	12	16	13	25	12	11	13	12	13	8	6	18	4	7	12	16	13	25	12	11	13	12	13	8	6	1	1	1	1	1	1	1	1	1	1	1	1	1	1	0	15	15	0	7	5	3	upstream_ge	MODIFIER	Pp3c7_1187	Pp3c7_1187	transcript	PAC:3292364	protein_codi	NA	c.-4949C>G	NA	NA	NA	NA	3730
Chr07	7992906	C	G	Chr07_79929C_G	SNP	12064.63	18	4	7	12	16	13	25	12	11	13	12	13	8	6	18	4	7	12	16	13	25	12	11	13	12	13	8	6	1	1	1	1	1	1	1	1	1	1	1	1	1	1	0	15	15	0	7	5	3	upstream_ge	MODIFIER	Pp3c7_1187	Pp3c7_1187	transcript	PAC:3292364	protein_codi	NA	c.-4949C>G	NA	NA	NA	NA	3528
Chr07	7992906	C	G	Chr07_79929C_G	SNP	12064.63	18	4	7	12	16	13	25	12	11	13	12	13	8	6	18	4	7	12	16	13	25	12	11	13	12	13	8	6	1	1	1	1	1	1	1	1	1	1	1	1	1	1	0	15	15	0	7	5	3	upstream_ge	MODIFIER	Pp3c7_1187	Pp3c7_1187	transcript	PAC:3292365	protein_codi	NA	c.-4949C>G	NA	NA	NA	NA	3528
Chr07	7992906	C	G	Chr07_79929C_G	SNP	12064.63	18	4	7	12	16	13	25	12	11	13	12	13	8	6	18	4	7	12	16	13	25	12	11	13	12	13	8	6	1	1	1	1	1	1	1	1	1	1	1	1	1	1	0	15	15	0	7	5	3	upstream_ge	MODIFIER	Pp3c7_1187	Pp3c7_1187	transcript	PAC:3292365	protein_codi	NA	c.-4949C>G	NA	NA	NA	NA	3528
Chr07	7992906	C	G	Chr07_79929C_G	SNP	12064.63	18	4	7	12	16	13	25	12	11	13	12	13	8	6	18	4	7	12	16	13	25	12	11	13	12	13	8	6	1	1	1	1	1	1	1	1	1	1	1	1	1	1	0	15	15	0	7	5	3	upstream_ge	MODIFIER	Pp3c7_1187	Pp3c7_1187	transcript	PAC:3292364	protein_codi	NA	c.-4949C>G	NA	NA	NA	NA	3528
Chr07	7992906	C	G	Chr07_79929C_G	SNP	12064.63	18	4	7	12	16	13	25	12	11	13	12	13	8	6	18	4	7	12	16	13	25	12	11	13	12	13	8	6	1	1	1	1	1	1	1	1	1	1	1	1	1	1	0	15	15	0	7	5	3	upstream_ge	MODIFIER	Pp3c7_1187	Pp3c7_1187	transcript	PAC:3292365	protein_codi	NA	c.-4949C>G	NA	NA	NA	NA	3528
Chr07	7992906	C	G	Chr07_79929C_G	SNP	12064.63	18	4	7	12	16	13	25	12	11	13	12	13	8	6	18	4	7	12	16	13	25	12	11	13	12	13	8	6	1	1	1	1	1	1	1	1	1	1	1	1	1	1	0	15	15	0	7	5	3	upstream_ge	MODIFIER	Pp3c7_1187	Pp3c7_1187	transcript	PAC:3292365	protein_codi	NA	c.-4949C>G	NA	NA	NA	NA	4592

[illegible]

Chr07	9875240	A	G	Chr07_98752 A_G	SNP	16400.63	26	10	13	24	24	23	23	22	10	12	22	6	6	5
Chr07	9875243	AAC	A	Chr07_98752 AAC_A	INDEL	16391.59	26	10	12	25	24	23	25	23	11	11	22	7	6	4
Chr07	9875243	AAC	A	Chr07_98752 AAC_A	INDEL	16391.59	26	10	12	25	24	23	25	23	11	11	22	7	6	4
Chr07	9875243	AAC	A	Chr07_98752 AAC_A	INDEL	16391.59	26	10	12	25	24	23	25	23	11	11	22	7	6	4
Chr07	9875246	T	TGG	Chr07_98752 T_TGG	INDEL	16391.59	27	10	12	25	24	23	26	23	11	11	22	7	6	4
Chr07	9875246	T	TGG	Chr07_98752 T_TGG	INDEL	16391.59	27	10	12	25	24	23	26	23	11	11	22	7	6	4
Chr07	9875246	T	TGG	Chr07_98752 T_TGG	INDEL	16391.59	27	10	12	25	24	23	26	23	11	11	22	7	6	4
Chr07	9875250	G	A	Chr07_98752 G_A	SNP	16625.63	24	9	13	25	24	23	25	23	10	11	21	7	6	5
Chr07	9875250	G	A	Chr07_98752 G_A	SNP	16625.63	24	9	13	25	24	23	25	23	10	11	21	7	6	5
Chr07	9875250	G	A	Chr07_98752 G_A	SNP	16625.63	24	9	13	25	24	23	25	23	10	11	21	7	6	5
Chr07	9875251	A	G	Chr07_98752 A_G	SNP	16670.63	25	9	13	24	24	23	25	23	10	11	21	7	6	5
Chr07	9875251	A	G	Chr07_98752 A_G	SNP	16670.63	25	9	13	24	24	23	25	23	10	11	21	7	6	5
Chr07	9875251	A	G	Chr07_98752 A_G	SNP	16670.63	25	9	13	24	24	23	25	23	10	11	21	7	6	5
Chr07	9875254	ACTTTT	A	Chr07_98752 ACTTTT_A	INDEL	16375.59	26	8	13	24	24	23	24	23	10	11	21	8	6	7
Chr07	9875254	ACTTTT	A	Chr07_98752 ACTTTT_A	INDEL	16375.59	26	8	13	24	24	23	24	23	10	11	21	8	6	7
Chr07	9875254	ACTTTT	A	Chr07_98752 ACTTTT_A	INDEL	16375.59	26	8	13	24	24	23	24	23	10	11	21	8	6	7
Chr07	9875260	GCA	G	Chr07_98752 GCA_G	INDEL	16015.59	26	8	13	24	24	23	24	23	9	11	20	8	6	7
Chr07	9875260	GCA	G	Chr07_98752 GCA_G	INDEL	16015.59	26	8	13	24	24	23	24	23	9	11	20	8	6	7
Chr07	9875260	GCA	G	Chr07_98752 GCA_G	INDEL	16015.59	26	8	13	24	24	23	24	23	9	11	20	8	6	7
Chr07	9877871	C	CAT	Chr07_98778 C_CAT	INDEL	254.37	0	0	0	0	0	0	0	0	0	0	0	0	0	9
Chr07	9877871	C	CAT	Chr07_98778 C_CAT	INDEL	254.37	0	0	0	0	0	0	0	0	0	0	0	0	0	9
Chr07	9877871	C	CAT	Chr07_98778 C_CAT	INDEL	254.37	0	0	0	0	0	0	0	0	0	0	0	0	0	9
Chr07	10085900	AT	A	Chr07_10085 AT_A	INDEL	16113.59	22	13	10	32	18	33	19	26	29	18	16	19	10	7
Chr07	10102507	G	T	Chr07_10102 G_T	SNP	3596.98	0	0	0	0	0	0	0	0	17	23	11	12	13	5
[illegible]																				

Chr07	11680021	GTA	G	Chr07_11680GTA_G	INDEL	9393.59	25	6	8	14	14	19	18	16	9	9	12	9	8	6	[illegible]
Chr07	11680021	GTA	G	Chr07_11680GTA_G	INDEL	9393.59	25	6	8	14	14	19	18	16	9	9	12	9	8	6	[illegible]
Chr07	11711102	G	GAT	Chr07_11711G_GAT	INDEL	1257.35	23	7	14	0	0	0	2	0	0	1	0	0	0	1	[illegible]
Chr07	11711102	G	GAT	Chr07_11711G_GAT	INDEL	1257.35	23	7	14	0	0	0	2	0	0	1	0	0	0	1	[illegible]
Chr07	11711102	G	GAT	Chr07_11711G_GAT	INDEL	1257.35	23	7	14	0	0	0	2	0	0	1	0	0	0	1	[illegible]
Chr07	11711102	G	GAT	Chr07_11711G_GAT	INDEL	1257.35	23	7	14	0	0	0	2	0	0	1	0	0	0	1	[illegible]
Chr07	11711102	G	GAT	Chr07_11711G_GAT	INDEL	1257.35	23	7	14	0	0	0	2	0	0	1	0	0	0	1	[illegible]
Chr07	11711102	G	GAT	Chr07_11711G_GAT	INDEL	1257.35	23	7	14	0	0	0	2	0	0	1	0	0	0	1	[illegible]
Chr07	11711102	G	GAT	Chr07_11711G_GAT	INDEL	1257.35	23	7	14	0	0	0	2	0	0	1	0	0	0	1	[illegible]
Chr07	11711102	G	GAT	Chr07_11711G_GAT	INDEL	1257.35	23	7	14	0	0	0	2	0	0	1	0	0	0	1	[illegible]
Chr07	11711102	G	GAT	Chr07_11711G_GAT	INDEL	1257.35	23	7	14	0	0	0	2	0	0	1	0	0	0	1	[illegible]
Chr07	11711102	G	GAT	Chr07_11711G_GAT	INDEL	1257.35	23	7	14	0	0	0	2	0	0	1	0	0	0	1	[illegible]
Chr07	11711102	G	GAT	Chr07_11711G_GAT	INDEL	1257.35	23	7	14	0	0	0	2	0	0	1	0	0	0	1	[illegible]
Chr07	12078683	C	T	Chr07_12078C_T	SNP	2598.39	35	14	15	0	0	0	0	0	0	0	0	0	0	0	[illegible]
Chr07	12168352	G	GTA	Chr07_12168G_GTA	INDEL	2957.24	0	0	0	11	16	13	25	16	0	0	1	0	0	0	[illegible]
Chr07	12365728	G	T	Chr07_12365G_T	SNP	19936.63	37	17	16	29	26	32	24	22	26	18	26	30	8	5	[illegible]
Chr07	12399905	C	CT	Chr07_12399C_CT	INDEL	10403.59	16	7	5	19	11	22	17	17	17	11	12	12	5	7	[illegible]
Chr07	12399960	G	T	Chr07_12399G_T	SNP	16901.63	24	7	8	24	10	27	19	15	22	24	19	18	7	8	[illegible]
Chr07	12399963	C	T	Chr07_12399C_T	SNP	17047.63	22	7	7	23	9	26	18	16	22	25	19	18	7	8	[illegible]
Chr07	12399985	T	A	Chr07_12399T_A	SNP	16420.63	22	8	5	25	13	23	17	12	21	28	18	18	7	8	[illegible]
Chr07	12400125	C	A	Chr07_12400C_A	SNP	19386.63	26	18	13	26	21	28	17	25	31	32	17	12	10	10	[illegible]
Chr07	12974565	T	G	Chr07_12974T_G	SNP	18112.63	19	18	8	22	11	18	32	28	21	20	11	19	12	12	[illegible]
Chr07	12974568	G	A	Chr07_12974G_A	SNP	18067.63	19	18	8	22	11	19	31	27	21	20	12	18	12	14	[illegible]
Chr07	13008637	C	A	Chr07_13008C_A	SNP	12266.63	23	6	7	21	15	25	14	12	10	21	12	13	8	6	[illegible]
[illegible]																					

Chr07	17493947	C	A	Chr07_17493C_A	SNP	18378.63	22	15	20	20	20	25	30	20	17	23	7	12	18
Chr07	17493947	C	A	Chr07_17493C_A	SNP	18378.63	22	15	20	20	20	25	30	20	17	23	7	12	18
Chr07	17493947	C	A	Chr07_17493C_A	SNP	18378.63	22	15	20	20	20	25	30	20	17	23	7	12	18
Chr07	17493947	C	A	Chr07_17493C_A	SNP	18378.63	22	15	20	20	20	25	30	20	17	23	7	12	18
Chr07	17493947	C	A	Chr07_17493C_A	SNP	18378.63	22	15	20	20	20	25	30	20	17	23	7	12	18
Chr07	17493947	C	A	Chr07_17493C_A	SNP	18378.63	22	15	20	20	20	25	30	20	17	23	7	12	18
Chr07	17493947	C	A	Chr07_17493C_A	SNP	18378.63	22	15	20	20	20	25	30	20	17	23	7	12	18
Chr07	17493947	C	A	Chr07_17493C_A	SNP	18378.63	22	15	20	20	20	25	30	20	17	23	7	12	18
Chr07	17493949	C	A	Chr07_17493C_A	SNP	18378.63	23	15	19	20	20	25	30	20	18	23	7	14	15
Chr07	17493949	C	A	Chr07_17493C_A	SNP	18378.63	23	15	19	20	20	25	30	20	18	23	7	14	15
Chr07	17493949	C	A	Chr07_17493C_A	SNP	18378.63	23	15	19	20	20	25	30	20	18	23	7	14	15
Chr07	17493949	C	A	Chr07_17493C_A	SNP	18378.63	23	15	19	20	20	25	30	20	18	23	7	14	15
Chr07	17493949	C	A	Chr07_17493C_A	SNP	18378.63	23	15	19	20	20	25	30	20	18	23	7	14	15
Chr07	17493949	C	A	Chr07_17493C_A	SNP	18378.63	23	15	19	20	20	25	30	20	18	23	7	14	15
Chr07	17493949	C	A	Chr07_17493C_A	SNP	18378.63	23	15	19	20	20	25	30	20	18	23	7	14	15
Chr07	17493949	C	A	Chr07_17493C_A	SNP	18378.63	23	15	19	20	20	25	30	20	18	23	7	14	15
Chr07	17493949	C	A	Chr07_17493C_A	SNP	18378.63	23	15	19	20	20	25	30	20	18	23	7	14	15
Chr07	17493961	T	A	Chr07_17493T_A	SNP	17435.63	25	14	21	23	20	26	31	21	18	25	6	14	16
Chr07	17493961	T	A	Chr07_17493T_A	SNP	17435.63	25	14	21	23	20	26	31	21	18	25	6	14	16
Chr07	17493961	T	A	Chr07_17493T_A	SNP	17435.63	25	14	21	23	20	26	31	21	18	25	6	14	16
Chr07	17493961	T	A	Chr07_17493T_A	SNP	17435.63	25	14	21	23	20	26	31	21	18	25	6	14	16

[illegible]

Chr08	3076831	T	TA	Chr08_30768	T_TA	INDEL	10919.59	26	10	8	29	12	13	21	18	16	17	11	9	10
Chr08	3089955	T	G	Chr08_30899	T_G	SNP	22245.63	32	11	19	33	21	21	41	32	28	28	19	23	7
Chr08	3089955	T	G	Chr08_30899	T_G	SNP	22245.63	32	11	19	33	21	21	41	32	28	28	19	23	7
Chr08	3089955	T	G	Chr08_30899	T_G	SNP	22245.63	32	11	19	33	21	21	41	32	28	28	19	23	7
Chr08	3090046	G	GT	Chr08_30900	G_GT	INDEL	18475.59	32	14	13	33	20	18	47	40	21	23	13	18	10
Chr08	3090046	G	GT	Chr08_30900	G_GT	INDEL	18475.59	32	14	13	33	20	18	47	40	21	23	13	18	10
Chr08	3090046	G	GT	Chr08_30900	G_GT	INDEL	18475.59	32	14	13	33	20	18	47	40	21	23	13	18	10
Chr08	3091356	T	TA	Chr08_30913	T_TA	INDEL	11359.59	34	14	13	20	15	20	25	18	13	28	7	12	4
Chr08	3091356	T	TA	Chr08_30913	T_TA	INDEL	11359.59	34	14	13	20	15	20	25	18	13	28	7	12	4
Chr08	3091356	T	TA	Chr08_30913	T_TA	INDEL	11359.59	34	14	13	20	15	20	25	18	13	28	7	12	4
Chr08	3313412	CTTATTAAAA	C	Chr08_33134	CTTATTAAAA	INDEL	15819.59	21	13	9	26	17	15	14	22	13	18	17	6	7
Chr08	3313412	CTTATTAAAA	C	Chr08_33134	CTTATTAAAA	INDEL	15819.59	21	13	9	26	17	15	14	22	13	18	17	6	7
Chr08	3313412	CTTATTAAAA	C	Chr08_33134	CTTATTAAAA	INDEL	15819.59	21	13	9	26	17	15	14	22	13	18	17	6	7
Chr08	3313455	C	CTATATATAT	Chr08_33134	C_CTATATAT	INDEL	15289.59	16	11	7	24	16	18	10	19	15	17	15	9	10
Chr08	3313455	C	CTATATATAT	Chr08_33134	C_CTATATAT	INDEL	15289.59	16	11	7	24	16	18	10	19	15	17	15	9	10
Chr08	3313455	C	CTATATATAT	Chr08_33134	C_CTATATAT	INDEL	15289.59	16	11	7	24	16	18	10	19	15	17	15	9	10
Chr08	3313473	C	CTA	Chr08_33134	C_CTA	INDEL	10848.59	11	6	5	24	12	16	12	13	10	13	10	8	7
Chr08	3313473	C	CTA	Chr08_33134	C_CTA	INDEL	10848.59	11	6	5	24	12	16	12	13	10	13	10	8	7
Chr08	3313473	C	CTA	Chr08_33134	C_CTA	INDEL	10848.59	11	6	5	24	12	16	12	13	10	13	10	8	7
Chr08	3313502	A	T	Chr08_33135	A_T	SNP	12315.63	12	6	6	28	9	21	14	17	11	12	12	8	8
Chr08	3313502	A	T	Chr08_33135	A_T	SNP	12315.63	12	6	6	28	9	21	14	17	11	12	12	8	8
Chr08	3313502	A	T	Chr08_33135	A_T	SNP	12315.63	12	6	6	28	9	21	14	17	11	12	12	8	8
Chr08	3526281	AAT	A	Chr08_35262	AAT_A	INDEL	8159.59	14	6	12	9	10	21	24	20	14	17	11	8	5
Chr08	3563350	C	CCGCCT	Chr08_35633	C_CCGCCT	INDEL	19145.59	16	19	8	31	23	21	33	16	16	20	21	17	8
[illegible]																				

Chr08	8477675	C	CA	Chr08_84776C_CA	INDEL	16409.59	30	13	17	24	23	26	29	27	25	30	19	13	11	17
Chr08	8477693	T	TC	Chr08_84776T_TC	INDEL	23064.59	33	15	20	18	28	29	31	28	20	30	22	15	12	18
Chr08	8477700	G	C	Chr08_84777G_C	SNP	23472.63	33	16	22	18	30	30	33	30	21	29	24	13	13	15
Chr08	8530812	G	A	Chr08_85308G_A	SNP	10131.63	13	8	7	11	12	14	17	8	9	16	7	10	9	5
Chr08	8530814	G	C	Chr08_85308G_C	SNP	10266.63	13	8	7	11	12	14	16	8	9	16	6	10	9	5
Chr08	8530815	AAAGC	A	Chr08_85308AAAGC_A	INDEL	10360.59	13	8	7	11	12	15	16	8	9	16	7	10	9	5
Chr08	8530822	GAGGTGGA/	G	Chr08_85308GAGGTGGA/	INDEL	10361.59	12	8	8	11	12	15	16	6	9	19	8	10	9	5
Chr08	8530842	T	TACGA	Chr08_85308T_TACGA	INDEL	9956.59	12	7	8	11	12	14	13	5	9	17	7	8	10	5
Chr08	8557745	AG	A	Chr08_85577AG_A	INDEL	13467.59	20	15	17	22	20	19	23	12	9	17	20	18	10	15
Chr08	8557745	AG	A	Chr08_85577AG_A	INDEL	13467.59	20	15	17	22	20	19	23	12	9	17	20	18	10	15
Chr08	8557745	AG	A	Chr08_85577AG_A	INDEL	13467.59	20	15	17	22	20	19	23	12	9	17	20	18	10	15
Chr08	8557745	AG	A	Chr08_85577AG_A	INDEL	13467.59	20	15	17	22	20	19	23	12	9	17	20	18	10	15
Chr08	8557745	AG	A	Chr08_85577AG_A	INDEL	13467.59	20	15	17	22	20	19	23	12	9	17	20	18	10	15
Chr08	8557745	AG	A	Chr08_85577AG_A	INDEL	13467.59	20	15	17	22	20	19	23	12	9	17	20	18	10	15
Chr08	8557745	AG	A	Chr08_85577AG_A	INDEL	13467.59	20	15	17	22	20	19	23	12	9	17	20	18	10	15
Chr08	8557745	AG	A	Chr08_85577AG_A	INDEL	13467.59	20	15	17	22	20	19	23	12	9	17	20	18	10	15
Chr08	8557745	AG	A	Chr08_85577AG_A	INDEL	13467.59	20	15	17	22	20	19	23	12	9	17	20	18	10	15
Chr08	8557745	AG	A	Chr08_85577AG_A	INDEL	13467.59	20	15	17	22	20	19	23	12	9	17	20	18	10	15
Chr08	8593977	T	C	Chr08_85939T_C	SNP	14549.63	17	14	8	20	19	20	18	13	16	18	10	10	9	9
Chr08	8593978	C	A	Chr08_85939C_A	SNP	14549.63	16	15	8	20	19	20	20	14	16	18	9	10	9	9
Chr08	8594016	G	T	Chr08_85940G_T	SNP	9893.63	12	8	7	13	12	17	13	10	13	10	7	9	7	6
Chr08	8594017	A	AT	Chr08_85940A_AT	INDEL	9884.59	12	8	5	13	12	17	13	10	13	10	7	9	7	5
Chr08	8594113	C	CT	Chr08_85941C_CT	INDEL	10316.59	11	8	8	14	8	14	10	14	10	8	7	13	7	8
Chr08	8594119	CA	C	Chr08_85941CA_C	INDEL	11112.59	11	8	7	14	9	14	10	14	10	10	8	14	7	8

[illegible]

Chr08	13553179	T	A	Chr08_13553 T_A	SNP	11090.63	12	9	9	[illegible]
Chr08	13553216	G	A	Chr08_13553 G_A	SNP	11630.63	17	11	8	[illegible]
Chr08	13553217	ACGT	A	Chr08_13553 ACGT_A	INDEL	12566.59	17	11	8	[illegible]
Chr08	13553220	T	TAAAAAAA	Chr08_13553 T_TAAAAAA	INDEL	12161.59	16	11	8	[illegible]
Chr08	13553222	TC	T	Chr08_13553 TC_T	INDEL	12791.59	17	10	9	[illegible]
Chr08	13553240	C	A	Chr08_13553 C_A	SNP	14139.63	24	11	8	[illegible]
Chr08	13553242	T	A	Chr08_13553 T_A	SNP	14139.63	24	10	8	[illegible]
Chr08	13553358	T	G	Chr08_13553 T_G	SNP	17580.63	23	10	11	[illegible]
Chr08	13592773	A	AAT	Chr08_13592 A_AAT	INDEL	880.35	12	7	14	[illegible]
Chr08	13592773	A	AAT	Chr08_13592 A_AAT	INDEL	880.35	12	7	14	[illegible]
Chr08	13592773	A	AAT	Chr08_13592 A_AAT	INDEL	880.35	12	7	14	[illegible]
Chr08	13592773	A	AAT	Chr08_13592 A_AAT	INDEL	880.35	12	7	14	[illegible]
Chr08	13592773	A	AAT	Chr08_13592 A_AAT	INDEL	880.35	12	7	14	[illegible]
Chr08	13592773	A	AAT	Chr08_13592 A_AAT	INDEL	880.35	12	7	14	[illegible]
Chr08	13592773	A	AAT	Chr08_13592 A_AAT	INDEL	880.35	12	7	14	[illegible]
Chr08	13592773	A	AAT	Chr08_13592 A_AAT	INDEL	880.35	12	7	14	[illegible]
Chr08	13592773	A	AAT	Chr08_13592 A_AAT	INDEL	880.35	12	7	14	[illegible]
Chr08	13725855	A	AAT	Chr08_13725 A_AAT	INDEL	604.37	0	0	1	[illegible]
Chr08	13728718	A	AAACTAAAT	Chr08_13728 A_AAACTAA	INDEL	17768.59	23	18	4	[illegible]
Chr08	13806281	C	A	Chr08_13806 C_A	SNP	20632.63	36	12	16	[illegible]
Chr08	13807066	A	AT	Chr08_13807 A_AT	INDEL	9074.59	24	8	10	[illegible]
Chr08	13927564	T	TA	Chr08_13927 T_TA	INDEL	20564.59	35	15	14	[illegible]
Chr08	13927566	G	GA	Chr08_13927 G_GA	INDEL	20674.59	34	15	14	[illegible]
Chr08	13973975	A	AT	Chr08_13973 A_AT	INDEL	17860.59	35	15	11	[illegible]
Chr08	13973975	A	AT	Chr08_13973 A_AT	INDEL	17860.59	35	15	11	[illegible]
Chr08	13973975	A	AT	Chr08_13973 A_AT	INDEL	17860.59	35	15	11	[illegible]
Chr08	13973975	A	AT	Chr08_13973 A_AT	INDEL	17860.59	35	15	11	[illegible]
Chr08	13973975	A	AT	Chr08_13973 A_AT	INDEL	17860.59	35	15	11	[illegible]
Chr08	13973996	G	T	Chr08_13973 G_T	SNP	18397.63	36	15	13	[illegible]
Chr08	13973996	G	T	Chr08_13973 G_T	SNP	18397.63	36	15	13	[illegible]
Chr08	13973996	G	T	Chr08_13973 G_T	SNP	18397.63	36	15	13	[illegible]
Chr08	13973996	G	T	Chr08_13973 G_T	SNP	18397.63	36	15	13	[illegible]
Chr08	13973996	G	T	Chr08_13973 G_T	SNP	18397.63	36	15	13	[illegible]
Chr08	14140344	T	TC	Chr08_14140 T_TC	INDEL	22256.59	29	17	18	[illegible]
Chr08	14140350	C	A	Chr08_14140 C_A	SNP	22439.63	31	18	19	[illegible]
Chr08	14140351	A	T	Chr08_14140 A_T	SNP	22289.63	31	18	19	[illegible]
Chr08	14248381	G	GT	Chr08_14248 G_GT	INDEL	16119.59	40	12	16	[illegible]
Chr08	14260865	A	ATTTGATTA	Chr08_14260 A_ATTTGATT	INDEL	17741.59	19	9	6	[illegible]
Chr08	14351306	G	GT	Chr08_14351 G_GT	INDEL	15678.59	18	18	15	[illegible]
Chr08	14351306	G	GT	Chr08_14351 G_GT	INDEL	15678.59	18	18	15	[illegible]
Chr08	14351306	G	GT	Chr08_14351 G_GT	INDEL	15678.59	18	18	15	[illegible]
Chr08	14351306	G	GT	Chr08_14351 G_GT	INDEL	15678.59	18	18	15	[illegible]
Chr08	14404226	C	A	Chr08_14404 C_A	SNP	20928.63	32	13	9	[illegible]
Chr08	14411638	C	A	Chr08_14411 C_A	SNP	13634.63	18	10	7	[illegible]
Chr08	14414847	C	CA	Chr08_14414 C_CA	INDEL	21192.59	37	8	12	[illegible]
Chr08	14414848	C	CA	Chr08_14414 C_CA	INDEL	21192.59	37	8	11	[illegible]
Chr08	14657518	T	C	Chr08_14657 T_C	SNP	20474.63	28	11	23	[illegible]
Chr08	14657518	T	C	Chr08_14657 T_C	SNP	20474.63	28	11	23	[illegible]
Chr08	14657518	T	C	Chr08_14657 T_C	SNP	20474.63	28	11	23	[illegible]
Chr08	14841051	T	TTCATTCAAG	Chr08_14841 T_TTCATTCA	INDEL	40450.59	47	31	19	[illegible]
Chr08	14841051	T	TTCATTCAAG	Chr08_14841 T_TTCATTCA	INDEL	40450.59	47	31	19	[illegible]
Chr08	14848303	T	C	Chr08_14848 T_C	SNP	13328.63	16	13	10	[illegible]
Chr08	14848303	T	C	Chr08_14848 T_C	SNP	13328.63	16	13	10	[illegible]
Chr08	14849823	G	A	Chr08_14849 G_A	SNP	12405.63	13	11	13	[illegible]
Chr08	14849859	C	T	Chr08_14849 C_T	SNP	13455.63	14	17	10	[illegible]
Chr08	14850004	A	T	Chr08_14850 A_T	SNP	12754.63	22	15	17	[illegible]
Chr08	14850802	C	T	Chr08_14850 C_T	SNP	8226.63	14	9	12	[illegible]
Chr08	14923403	T	TTATA	Chr08_14923 T_TTATA	INDEL	3590.24	0	0	0	[illegible]
Chr08	14923403	T	TTATA	Chr08_14923 T_TTATA	INDEL	3590.24	0	0	0	[illegible]
Chr08	14923403	T	TTATA	Chr08_14923 T_TTATA	INDEL	3590.24	0	0	0	[illegible]
Chr08	14923403	T	TTATA	Chr08_14923 T_TTATA	INDEL	3590.24	0	0	0	[illegible]
Chr08	14923403	T	TTATA	Chr08_14923 T_TTATA	INDEL	3590.24	0	0	0	[illegible]
Chr08	14923403	T	TTATA	Chr08_14923 T_TTATA	INDEL	3590.24	0	0	0	[illegible]
Chr08	14923403	T	TTATA	Chr08_14923 T_TTATA	INDEL	3590.24	0	0	0	[illegible]
Chr08	14929460	T	TACAAATCCC	Chr08_14929 T_TACAAATC	INDEL	13318.59	9	10	11	[illegible]
Chr08	14929460	T	TACAAATCCC	Chr08_14929 T_TACAAATC	INDEL	13318.59	9	10	11	[illegible]
Chr08	14929460	T	TACAAATCCC	Chr08_14929 T_TACAAATC	INDEL	13318.59	9	10	11	[illegible]
Chr08	14929460	T	TACAAATCCC	Chr08_14929 T_TACAAATC	INDEL	13318.59	9	10	11	[illegible]
Chr08	14929460	T	TACAAATCCC	Chr08_14929 T_TACAAATC	INDEL	13318.59	9	10	11	[illegible]
Chr08	14929460	T	TACAAATCCC	Chr08_14929 T_TACAAATC	INDEL	13318.59	9	10	11	[illegible]
Chr08	14929460	T	TACAAATCCC	Chr08_14929 T_TACAAATC	INDEL	13318.59	9	10	11	[illegible]
Chr08	14929460	T	TACAAATCCC	Chr08_14929 T_TACAAATC	INDEL	13318.59	9	10	11	[illegible]
Chr08	14929476	GCAA	G	Chr08_14929 GCAA_G	INDEL	12116.59	11	10	11	[illegible]
Chr08	14929476	GCAA	G	Chr08_14929 GCAA_G	INDEL	12116.59	11	10	11	[illegible]
Chr08	14929476	GCAA	G	Chr08_14929 GCAA_G	INDEL	12116.59	11	10	11	[illegible]
Chr08	14929476	GCAA	G	Chr08_14929 GCAA_G	INDEL	12116.59	11	10	11	[illegible]
Chr08	14929476	GCAA	G	Chr08_14929 GCAA_G	INDEL	12116.59	11	10	11	[illegible]
Chr08	14929476	GCAA	G	Chr08_14929 GCAA_G	INDEL	12116.59	11	10	11	[illegible]
Chr08	14929476	GCAA	G	Chr08_14929 GCAA_G	INDEL	12116.59	11	10	11	[illegible]
Chr08	14929476	GCAA	G	Chr08_14929 GCAA_G	INDEL	12116.59	11	10	11	[illegible]
Chr08	15235411	C	T	Chr08_15235 C_T	SNP	15211.63	24	8	4	[illegible]
Chr08	15235411	C	T	Chr08_15235 C_T	SNP	15211.63	24	8	4	[illegible]
Chr08	15235411	C	T	Chr08_15235 C_T	SNP	15211.63	24	8	4	[illegible]
Chr08	15235411	C	T	Chr08_15235 C_T	SNP	15211.63	24	8	4	[illegible]
Chr08	15235411	C	T	Chr08_15235 C_T	SNP	15211.63	24	8	4	[illegible]
Chr08	15235411	C	T	Chr08_15235 C_T	SNP	15211.63	24	8	4	[illegible]
Chr08	15235411	C	T	Chr08_15235 C_T	SNP	15211.63	24	8	4	[illegible]
Chr08	15235431	C	A	Chr08_15235 C_A	SNP	15680.63	22	10	4	[illegible]
Chr08	15235431	C	A	Chr08_15235 C_A	SNP	15680.63	22	10	4	[illegible]
Chr08	15235431	C	A	Chr08_15235 C_A	SNP	15680.63	22	10	4	[illegible]
Chr08	15235431	C	A	Chr08_15235 C_A	SNP	15680.63	22	10	4	[illegible]
Chr08	15235431	C	A	Chr08_15235 C_A	SNP	15680.63	22	10	4	[illegible]
Chr08	15235431	C	A	Chr08_15235 C_A	SNP	15680.63	22	10	4	[illegible]
Chr08	15235431	C	A	Chr08_15235 C_A	SNP	15680.63	22	10	4	[illegible]
Chr08	15264105	CCTT	C	Chr08_15264 CCTT_C	INDEL	18399.59	13	11	11	[illegible]
Chr08	15264105	CCTT	C	Chr08_15264 CCTT_C	INDEL	18399.59	13	11	11	[illegible]
Chr08	15264108	T	TAAGA	Chr08_15264 T_TAAGA	INDEL	18399.59	13	11	11	[illegible]
Chr08	15264108	T	TAAGA	Chr08_15264 T_TAAGA	INDEL	18399.59	13	11	11	[illegible]
Chr08	15264110	C	G	Chr08_15264 C_G	SNP	18408.63	18	11	10	[illegible]
Chr08	15264110	C	G	Chr08_15264 C_G	SNP	18408.63	18	11	10	[illegible]
Chr08	15337449	C	CAT	Chr08_15337 C_CAT	INDEL	878.35	15	7	9	[illegible]
Chr08	15337449	C	CAT	Chr08_15337 C_CAT	INDEL	878.35	15	7	9	[illegible]
Chr08	15337449	C	CAT	Chr08_15337 C_CAT	INDEL	878.35	15	7	9	[illegible]
Chr08	15337449	C	CAT	Chr08_15337 C_CAT	INDEL	878.35	15	7	9	[illegible]
Chr08	15468692	TAAGACTCA	T	Chr08_15468 TAAGACTCA	INDEL	223.37	0	6	0	[illegible]
Chr08	15468692	TAAGACTCA	T	Chr08_15468 TAAGACTCA	INDEL	223.37	0	6	0	[illegible]
Chr08	15468692	TAAGACTCA	T	Chr08_15468 TAAGACTCA	INDEL	223.37	0	6	0	[illegible]
Chr08	15468692	TAAGACTCA	T	Chr08_15468 TAAGACTCA	INDEL	223.37	0	6	0	[illegible]
Chr08	15468692	TAAGACTCA	T	Chr08_15468 TAAGACTCA	INDEL	223.37	0	6	0	[illegible]
Chr08	15498935	T	TTA	Chr08_15498 T_TTA	INDEL	2198.93	1	0	0	[illegible]
Chr08	15498935	T	TTA	Chr08_15498 T_TTA	INDEL	2198.93	1	0	0	[illegible]
Chr08	15498935	T	TTA	Chr08_15498 T_TTA	INDEL	2198.93	1	0	0	[illegible]
Chr08	15498935	T	TTA	Chr08_15498 T_TTA	INDEL	2198.93	1	0	0	[illegible]
Chr08	15498935	T	TTA	Chr08_15498 T_TTA	INDEL	2198.93	1	0	0	[illegible]
Chr08	15518200	GTA	G	Chr08_15518 GTA_G	INDEL	2823.24	0	0	0	[illegible]
Chr08	15518200	GTA	G	Chr08_15518 GTA_G	INDEL	2823.24	0	0	0	[illegible]
Chr08	15518200	GTA	G	Chr08_15518 GTA_G	INDEL	2823.24	0	0	0	[illegible]
Chr08	15720521	G	GT	Chr08_15720 G_GT	INDEL	1651.93	1	0	1	[illegible]
Chr08	15734814	C	T	Chr08_15734 C_T	SNP	22909.63	29	28	16	[illegible]
Chr08	15734837	G	A	Chr08_15734 G_A	SNP	22561.63	28	24	14	[illegible]
Chr08	15734850	A	G	Chr08_15734 A_G	SNP	21449.63	22	23	14	[illegible]
Chr08	15734859	A	G	Chr08_15734 A_G	SNP	20510.63	25	20	16	[illegible]
Chr08	15750704	G	A	Chr08_15750 G_A	SNP	7848.63	13	19	14	[illegible]
Chr08	15752987	G	A	Chr08_15752 G_A	SNP	12750.63	19	11	4	[illegible]
Chr08	15754841	A	T	Chr08_15754 A_T	SNP	10211.63	7	4	8	[illegible]
Chr08	15754847	C	G	Chr08_15754 C_G	SNP	10548.63	13	4	9	[illegible]
Chr08	15755825	A	C	Chr08_15755 A_C	SNP	15156.63	22	16	18	[illegible]
Chr08	15755875	A	G	Chr08_15755 A_G	SNP	15472.63	24	20	20	[illegible]
Chr08	15813895	C	T	Chr08_15813 C_T	SNP	14257.63	20	13	14	[illegible]
Chr08	15813910	A	G	Chr08_15813 A_G	SNP	13747.63	20	11	15	[illegible]
Chr08	15816790	G	A	Chr08_15816 G_A	SNP	14861.63	26	9	16	[illegible]
Chr08	16019776	TG	T	Chr08_16019 TG_T	INDEL	19761.59	32	25	10	[illegible]
Chr08	16019780	TG	T	Chr08_16019 TG_T	INDEL	19876.59	32	26	10	[illegible]
Chr08	16123484	G	T	Chr08_16123 G_T	SNP	18295.63	17	15	8	[illegible]
Chr08	16123615	A	C	Chr08_16123 A_C	SNP	16973.63	26	15	9	[illegible]
Chr08	16213844	GAT	GATAT	Chr08_16213 GAT_GATAT	INDEL	597.35	2	1	0	[illegible]
Chr08	16213844	GAT	GATAT	Chr08_16213 GAT_GATAT	INDEL	597.35	2	1	0	[illegible]
Chr08	16213844	GAT	GATAT	Chr08_16213 GAT_GATAT	INDEL	597.35	2	1	0	[illegible]
Chr08	16213844	GAT	GATAT	Chr08_16213 GAT_GATAT	INDEL	597.35	2	1	0	[illegible]
Chr08	16213844	GAT	GATAT	Chr08_16213 GAT_GATAT	INDEL	597.35	2	1	0	[illegible]
Chr08	16213844	GAT	GATAT	Chr08_16213 GAT_GATAT	INDEL	597.35	2	1	0	[illegible]
Chr08	16213844	GAT	GATAT	Chr08_16213 GAT_GATAT	INDEL	597.35	2	1	0	[illegible]
Chr08	16213844	GAT	GATAT	Chr08_16213 GAT_GATAT	INDEL	597.35	2	1	0	[illegible]
Chr08	16260313	C	A	Chr08_16260 C_A	SNP	16307.63	36	11	6	[illegible]
Chr08	16273339	G	T	Chr08_16273 G_T	SNP	15008.63	25	11	13	[illegible]
Chr08	16300290	C	A	Chr08_16300 C_A	SNP	13639.63	17	9	8	[illegible]
Chr08	16300381	C	A	Chr08_16300 C_A	SNP	9708.63	14	8	5	[illegible]
Chr08	16300403	C	T	Chr08_16300 C_T	SNP	11777.63	17	9	6	[illegible]
Chr08	16300519	A	T	Chr08_16300 A_T	SNP	14515.63	24	10	7	[illegible]
Chr08	16300553	T	G	Chr08_16300 T_G	SNP	11656.63	18	9	5	[illegible]
Chr08	16587475	A	C	Chr08_16587 A_C	SNP	5166.29	0	0	0	[illegible]
Chr08	16587475	A	C	Chr08_16587 A_C	SNP	5166.29	0	0	0	[illegible]
Chr08	16587475	A	C	Chr08_16587 A_C	SNP	5166.29	0	0	0	[illegible]
Chr08	16625767	C	T	Chr08_16625 C_T	SNP	13167.63	13	18	11	[illegible]
Chr08	16727027	TTATA	T	Chr08_16727 TTATA_T	INDEL	2621.93	0	0	0	[illegible]
Chr08	16756072	A	AT	Chr08_16756 A_AT	INDEL	15496.59	29	13	12	[illegible]
Chr08	16756084	C	CT	Chr08_16756 C_CT	INDEL	15933.59	31	15	13	[illegible]
Chr08	16834300	TGCTGG	T	Chr08_16834 TGCTGG_T	INDEL	18188.59	11	14	11	[illegible]
Chr08	16834307	A	AAAATAT	Chr08_16834 A_AAAATAT	INDEL	18820.59	12	14	11	[illegible]
Chr08	16834309	G	T	Chr08_16834 G_T	SNP	18852.63	13	13	10	[illegible]
Chr08	16894345	T	TTA	Chr08_16894 T_TTA	INDEL	2113.93	0	0	0	[illegible]
Chr08	16894345	T	TTA	Chr08_16894 T_TTA	INDEL	2113.93	0	0	0	[illegible]
Chr08	16894345	T	TTA	Chr08_16894 T_TTA	INDEL	2113.93	0	0	0	[illegible]
Chr08	17101493	A	AT	Chr08_17101 A_AT	INDEL	14228.59	21	12	13	[illegible]
Chr08	17103244	T	TA	Chr08_17103 T_TA	INDEL	13365.59	23	14	16	[illegible]
Chr08	17119621	G	GA	Chr08_17119 G_GA	INDEL	15309.59	30	16	13	[illegible]
Chr08	17131128	G	T	Chr08_17131 G_T	SNP	18879.63	37	12	14	[illegible]
Chr08	17131128	G	T	Chr08_17131 G_T	SNP	18879.63	37	12	14	[illegible]
Chr08	17192149	C	A	Chr08_17192 C_A	SNP	20304.63	25	16	17	[illegible]
Chr08	17192149	C	A	Chr08_17192 C_A	SNP	20304.63	25	16	17	[illegible]
Chr08	17192149	C	A	Chr08_17192 C_A	SNP	20304.63	25	16	17	[illegible]
Chr08	17192151	C	A	Chr08_17192 C_A	SNP	20394.63	25	16	17	[illegible]
Chr08	17192151	C	A	Chr08_17192 C_A	SNP	20394.63	25	16	17	[illegible]
Chr08	17192151	C	A	Chr08_17192 C_A	SNP	20394.63	25	16	17	[illegible]
Chr08	17192159	C	A	Chr08_17192 C_A	SNP	19631.63	25	14	15	[illegible]
Chr08	17192159	C	A	Chr08_17192 C_A	SNP	19631.63	25	14	15	[illegible]
Chr08	17192159	C	A	Chr08_17192 C_A	SNP	19631.63	25	14	15	[illegible]
Chr08	17192194	C	T	Chr08_17192 C_T	SNP	17519.63	21	12	12	[illegible]
Chr08	17192194	C	T	Chr08_17192 C_T	SNP	17519.63	21	12	12	[illegible]
Chr08	17192194	C	T	Chr08_17192 C_T	SNP	17519.63	21	12	12	[illegible]
Chr08	17192202	C	A	Chr08_17192 C_A	SNP	18154.63	20	12	11	[illegible]
Chr08	17192202	C	A	Chr08_17192 C_A	SNP	18154.63	20	12	11	[illegible]
Chr08	17192202	C	A	Chr08_17192 C_A	SNP	18154.63	20	12	11	[illegible]
Chr08	17192206	C	T	Chr08_17192 C_T	SNP	18425.63	22	12	12	[illegible]
Chr08	17192206	C	T	Chr08_17192 C_T	SNP	18425.63	22	12	12	[illegible]
Chr08	17192206	C	T	Chr08_17192 C_T	SNP	18425.63	22	12	12	[illegible]
Chr08	17192210	G	C	Chr08_17192 G_C	SNP	18920.63	22	13	12	[illegible]
Chr08	17192210	G	C	Chr08_17192 G_C	SNP	18920.63	22	13	12	[illegible]
Chr08	17192210	G	C	Chr08_17192 G_C	SNP	18920.63	22	13	12	[illegible]
Chr08	17192211	C	A	Chr08_17192 C_A	SNP	19190.63	23	12	11	[illegible]
Chr08	17192211	C	A	Chr08_17192 C_A	SNP	19190.63	23	12	11	[illegible]
Chr08	17192211	C	A	Chr08_17192 C_A	SNP	19190.63	23	12	11	[illegible]
Chr08	17192215	C	A	Chr08_17192 C_A	SNP	18965.63	21	12	10	[illegible]
Chr08	17192215	C	A	Chr08_17192 C_A	SNP	18965.63	21	12	10	[illegible]
Chr08	17192215	C	A	Chr08_17192 C_A	SNP	18965.63	21	12	10	[illegible]
Chr08	17192219	C	A	Chr08_17192 C_A	SNP	19325.63	21	11	11	[illegible]
Chr08	17192219	C	A	Chr08_17192 C_A	SNP	19325.63	21	11	11	[illegible]
Chr08	17192219	C	A	Chr08_17192 C_A	SNP	19325.63	21	11	11	[illegible]
Chr08	17192220	C	A	Chr08_17192 C_A	SNP	19415.63	20	11	10	[illegible]
Chr08	17192220	C	A	Chr08_17192 C_A	SNP	19415.63	20	11	10	[illegible]
Chr08	17192220	C	A	Chr08_17192 C_A	SNP	19415.63	20	11	10	[illegible]
Chr08	17192222	C	A	Chr08_17192 C_A	SNP	19505.63	20	11	10	[illegible]

Chr08	17192222	C	A	Chr08_17192C_A	SNP	19505.63	20	11	10	28
Chr08	17192222	C	A	Chr08_17192C_A	SNP	19505.63	20	11	10	28
Chr08	17192225	C	A	Chr08_17192C_A	SNP	19550.63	19	11	10	28
Chr08	17192225	C	A	Chr08_17192C_A	SNP	19550.63	19	11	10	28
Chr08	17192225	C	A	Chr08_17192C_A	SNP	19550.63	19	11	10	28
Chr08	17192227	C	A	Chr08_17192C_A	SNP	19550.63	19	12	9	29
Chr08	17192227	C	A	Chr08_17192C_A	SNP	19550.63	19	12	9	29
Chr08	17192227	C	A	Chr08_17192C_A	SNP	19550.63	19	12	9	29
Chr08	17192229	C	A	Chr08_17192C_A	SNP	20045.63	18	12	9	30
Chr08	17192229	C	A	Chr08_17192C_A	SNP	20045.63	18	12	9	30
Chr08	17192229	C	A	Chr08_17192C_A	SNP	20045.63	18	12	9	30
Chr08	17192233	C	A	Chr08_17192C_A	SNP	19865.63	18	11	8	31
Chr08	17192233	C	A	Chr08_17192C_A	SNP	19865.63	18	11	8	31
Chr08	17192233	C	A	Chr08_17192C_A	SNP	19865.63	18	11	8	31
Chr08	17192237	C	A	Chr08_17192C_A	SNP	19505.63	17	10	7	27
Chr08	17192237	C	A	Chr08_17192C_A	SNP	19505.63	17	10	7	27
Chr08	17192237	C	A	Chr08_17192C_A	SNP	19505.63	17	10	7	27
Chr08	17192243	C	A	Chr08_17192C_A	SNP	19370.63	18	11	9	27
Chr08	17192243	C	A	Chr08_17192C_A	SNP	19370.63	18	11	9	27
Chr08	17192243	C	A	Chr08_17192C_A	SNP	19370.63	18	11	9	27
Chr08	17192246	C	A	Chr08_17192C_A	SNP	19730.63	20	11	9	28
Chr08	17192246	C	A	Chr08_17192C_A	SNP	19730.63	20	11	9	28
Chr08	17192246	C	A	Chr08_17192C_A	SNP	19730.63	20	11	9	28
Chr08	17192247	GGA	G	Chr08_17192GGA_G	INDEL	20253.59	20	11	9	28

[illegible]

Chr09	2578614	G	GTA	Chr09_25786G_GTA	INDEL	7192.24	19	14	8	0	0	0	0	0	22	15	8	15	7	8	[illegible]
Chr09	2710516	C	CATAT	Chr09_27105C_CATAT	INDEL	9828.59	18	7	7	0	0	0	1	2	10	8	9	8	4	4	[illegible]
Chr09	2710516	C	CATAT	Chr09_27105C_CATAT	INDEL	9828.59	18	7	7	0	0	0	1	2	10	8	9	8	4	4	[illegible]
Chr09	2710516	C	CATAT	Chr09_27105C_CATAT	INDEL	9828.59	18	7	7	0	0	0	1	2	10	8	9	8	4	4	[illegible]
Chr09	2710516	C	CATAT	Chr09_27105C_CATAT	INDEL	9828.59	18	7	7	0	0	0	1	2	10	8	9	8	4	4	[illegible]
Chr09	2710516	C	CATAT	Chr09_27105C_CATAT	INDEL	9828.59	18	7	7	0	0	0	1	2	10	8	9	8	4	4	[illegible]
Chr09	2710516	C	CATAT	Chr09_27105C_CATAT	INDEL	9828.59	18	7	7	0	0	0	1	2	10	8	9	8	4	4	[illegible]
Chr09	2710516	C	CATAT	Chr09_27105C_CATAT	INDEL	9828.59	18	7	7	0	0	0	1	2	10	8	9	8	4	4	[illegible]
Chr09	2710516	C	CATAT	Chr09_27105C_CATAT	INDEL	9828.59	18	7	7	0	0	0	1	2	10	8	9	8	4	4	[illegible]
Chr09	2710516	C	CATAT	Chr09_27105C_CATAT	INDEL	9828.59	18	7	7	0	0	0	1	2	10	8	9	8	4	4	[illegible]
Chr09	2710516	C	CATAT	Chr09_27105C_CATAT	INDEL	9828.59	18	7	7	0	0	0	1	2	10	8	9	8	4	4	[illegible]
Chr09	2710516	C	CATAT	Chr09_27105C_CATAT	INDEL	9828.59	18	7	7	0	0	0	1	2	10	8	9	8	4	4	[illegible]
Chr09	2728402	TA	T	Chr09_27284TA_T	INDEL	11911.59	10	18	16	21	17	28	9	16	21	18	15	8	8	14	[illegible]
Chr09	2835729	A	T	Chr09_28357A_T	SNP	16928.63	23	25	17	31	24	13	28	22	22	21	7	11	7	5	[illegible]
Chr09	2835729	A	T	Chr09_28357A_T	SNP	16928.63	23	25	17	31	24	13	28	22	22	21	7	11	7	5	[illegible]
Chr09	2835729	A	T	Chr09_28357A_T	SNP	16928.63	23	25	17	31	24	13	28	22	22	21	7	11	7	5	[illegible]
Chr09	2835729	A	T	Chr09_28357A_T	SNP	16928.63	23	25	17	31	24	13	28	22	22	21	7	11	7	5	[illegible]
Chr09	3147818	AC	A	Chr09_31478AC_A	INDEL	19234.59	30	12	19	16	19	49	30	18	28	26	17	19	10	9	[illegible]
Chr09	3181264	C	T	Chr09_31812C_T	SNP	20091.63	23	12	23	27	21	25	23	24	29	13	16	21	11	10	[illegible]
Chr09	3181291	TC	T	Chr09_31812TC_T	INDEL	19395.59	18	9	20	28	20	23	22	18	25	14	15	17	12	6	[illegible]
Chr09	3181295	T	TA	Chr09_31812T_TA	INDEL	19501.59	18	10	22	27	20	23	23	18	26	13	15	17	11	6	[illegible]
Chr09	3181407	AC	A	Chr09_31814AC_A	INDEL	15250.59	27	10	16	21	18	22	21	15	23	31	19	17	4	8	[illegible]
Chr09	3217385	C	A	Chr09_32173C_A	SNP	20346.63	31	20	13	30	19	33	32	31	20	21	11	22	16	17	[illegible]
Chr09	3217435	CA	C	Chr09_32174CA_C	INDEL	20985.59	29	17	11	24	20	28	37	23	24	21	14	22	16	14	[illegible]
[illegible]																					

Chr09	8973256	A	AAT	Chr09_89732A_AAT	INDEL	2211.24	1	0	0	14	7	17	24	10	1	0	0	0	0	0
Chr09	8973256	A	AAT	Chr09_89732A_AAT	INDEL	2211.24	1	0	0	14	7	17	24	10	1	0	0	0	0	0
Chr09	8973256	A	AAT	Chr09_89732A_AAT	INDEL	2211.24	1	0	0	14	7	17	24	10	1	0	0	0	0	0
Chr09	8973256	A	AAT	Chr09_89732A_AAT	INDEL	2211.24	1	0	0	14	7	17	24	10	1	0	0	0	0	0
Chr09	8973256	A	AAT	Chr09_89732A_AAT	INDEL	2211.24	1	0	0	14	7	17	24	10	1	0	0	0	0	0
Chr09	8973256	A	AAT	Chr09_89732A_AAT	INDEL	2211.24	1	0	0	14	7	17	24	10	1	0	0	0	0	0
Chr09	8973256	A	AAT	Chr09_89732A_AAT	INDEL	2211.24	1	0	0	14	7	17	24	10	1	0	0	0	0	0
Chr09	8973256	A	AAT	Chr09_89732A_AAT	INDEL	2211.24	1	0	0	14	7	17	24	10	1	0	0	0	0	0
Chr09	8973256	A	AAT	Chr09_89732A_AAT	INDEL	2211.24	1	0	0	14	7	17	24	10	1	0	0	0	0	0
Chr09	9021617	G	GTA	Chr09_90216G_GTA	INDEL	231.37	1	0	0	0	1	0	1	0	0	0	1	0	0	10
Chr09	9021617	G	GTA	Chr09_90216G_GTA	INDEL	231.37	1	0	0	0	1	0	1	0	0	0	1	0	0	10
Chr09	9021617	G	GTA	Chr09_90216G_GTA	INDEL	231.37	1	0	0	0	1	0	1	0	0	0	1	0	0	10
Chr09	9021617	G	GTA	Chr09_90216G_GTA	INDEL	231.37	1	0	0	0	1	0	1	0	0	0	1	0	0	10
Chr09	9021617	G	GTA	Chr09_90216G_GTA	INDEL	231.37	1	0	0	0	1	0	1	0	0	0	1	0	0	10
Chr09	9077708	C	A	Chr09_90777C_A	SNP	18286.63	17	9	13	24	23	27	12	23	27	21	16	17	14	4
Chr09	9098347	T	TTA	Chr09_90983T_TTA	INDEL	3802.24	0	0	0	27	20	24	22	24	0	0	0	0	0	0
Chr09	9098347	T	TTA	Chr09_90983T_TTA	INDEL	3802.24	0	0	0	27	20	24	22	24	0	0	0	0	0	0
Chr09	9098347	T	TTA	Chr09_90983T_TTA	INDEL	3802.24	0	0	0	27	20	24	22	24	0	0	0	0	0	0
Chr09	9098347	T	TTA	Chr09_90983T_TTA	INDEL	3802.24	0	0	0	27	20	24	22	24	0	0	0	0	0	0
Chr09	9098347	T	TTA	Chr09_90983T_TTA	INDEL	3802.24	0	0	0	27	20	24	22	24	0	0	0	0	0	0
Chr09	9098347	T	TTA	Chr09_90983T_TTA	INDEL	3802.24	0	0	0	27	20	24	22	24	0	0	0	0	0	0
Chr09	9098347	T	TTA	Chr09_90983T_TTA	INDEL	3802.24	0	0	0	27	20	24	22	24	0	0	0	0	0	0
Chr09	9098347	T	TTA	Chr09_90983T_TTA	INDEL	3802.24	0	0	0	27	20	24	22	24	0	0	0	0	0	0
[illegible]																				

Chr09	14624986	C	T	Chr09_14624	C_T	SNP	12792.63	19	[illegible]
Chr09	14625008	G	A	Chr09_14625	G_A	SNP	11076.63	16	[illegible]
Chr09	14666735	C	T	Chr09_14666	C_T	SNP	13056.63	27	[illegible]
Chr09	14666735	C	T	Chr09_14666	C_T	SNP	13056.63	27	[illegible]
Chr09	14666735	C	T	Chr09_14666	C_T	SNP	13056.63	27	[illegible]
Chr09	14679841	C	T	Chr09_14679	C_T	SNP	2736.39	34	[illegible]
Chr09	14754604	T	A	Chr09_14754	T_A	SNP	17401.63	26	[illegible]
Chr09	14754604	T	A	Chr09_14754	T_A	SNP	17401.63	26	[illegible]
Chr09	14845217	CTA	C	Chr09_14845	CTA_C	INDEL	9652.59	23	[illegible]
Chr09	14845217	CTA	C	Chr09_14845	CTA_C	INDEL	9652.59	23	[illegible]
Chr09	14845217	CTA	C	Chr09_14845	CTA_C	INDEL	9652.59	23	[illegible]
Chr09	14845217	CTA	C	Chr09_14845	CTA_C	INDEL	9652.59	23	[illegible]
Chr09	14845217	CTA	C	Chr09_14845	CTA_C	INDEL	9652.59	23	[illegible]
Chr09	14845217	CTA	C	Chr09_14845	CTA_C	INDEL	9652.59	23	[illegible]
Chr09	14845217	CTA	C	Chr09_14845	CTA_C	INDEL	9652.59	23	[illegible]
Chr09	14845217	CTA	C	Chr09_14845	CTA_C	INDEL	9652.59	23	[illegible]
Chr09	14845217	CTA	C	Chr09_14845	CTA_C	INDEL	9652.59	23	[illegible]
Chr09	15086878	T	C	Chr09_15086	T_C	SNP	12623.29	28	[illegible]
Chr09	15086878	T	C	Chr09_15086	T_C	SNP	12623.29	28	[illegible]
Chr09	15136423	T	A	Chr09_15136	T_A	SNP	3579.29	0	[illegible]
Chr09	15136424	T	C	Chr09_15136	T_C	SNP	3579.29	0	[illegible]
Chr09	15295187	A	AAT	Chr09_15295	A_AAT	INDEL	285.37	0	[illegible]
Chr09	15390015	CT	C	Chr09_15390	CT_C	INDEL	13791.59	30	[illegible]
Chr09	15390017	C	A	Chr09_15390	C_A	SNP	13872.63	30	[illegible]
Chr09	15390026	C	CA	Chr09_15390	C_CA	INDEL	14160.59	28	[illegible]
Chr09	15390076	G	T	Chr09_15390	G_T	SNP	15776.63	31	[illegible]
Chr09	15393507	T	TA	Chr09_15393	T_TA	INDEL	12610.59	21	[illegible]
Chr09	15409677	A	T	Chr09_15409	A_T	SNP	11534.63	20	[illegible]
Chr09	15567151	A	AT	Chr09_15567	A_AT	INDEL	4062.24	0	[illegible]
Chr09	15633400	T	C	Chr09_15633	T_C	SNP	12949.63	13	[illegible]
Chr09	15652303	G	C	Chr09_15652	G_C	SNP	5736.29	0	[illegible]
Chr09	15660026	C	CT	Chr09_15660	C_CT	INDEL	15598.59	32	[illegible]
Chr09	15665608	C	T	Chr09_15665	C_T	SNP	19756.63	23	[illegible]
Chr09	15706206	G	A	Chr09_15706	G_A	SNP	19588.63	26	[illegible]
Chr09	15776516	C	A	Chr09_15776	C_A	SNP	20740.63	24	[illegible]
Chr09	15776516	C	A	Chr09_15776	C_A	SNP	20740.63	24	[illegible]
Chr09	15831418	C	CA	Chr09_15831	C_CA	INDEL	11680.59	17	[illegible]
Chr09	15831418	C	CA	Chr09_15831	C_CA	INDEL	11680.59	17	[illegible]
Chr09	15845226	C	A	Chr09_15845	C_A	SNP	13008.63	22	[illegible]
Chr09	15866618	C	A	Chr09_15866	C_A	SNP	13751.63	18	[illegible]
Chr09	15866786	T	C	Chr09_15866	T_C	SNP	10360.63	20	[illegible]
Chr09	15866807	C	G	Chr09_15866	C_G	SNP	9489.63	15	[illegible]
Chr09	15914680	T	A	Chr09_15914	T_A	SNP	12344.63	18	[illegible]
Chr09	15914754	G	A	Chr09_15914	G_A	SNP	10277.63	19	[illegible]
Chr09	15985999	T	A	Chr09_15985	T_A	SNP	21733.63	23	[illegible]
Chr09	15986038	G	A	Chr09_15986	G_A	SNP	19994.63	17	[illegible]
Chr09	16010042	TCGGATTTT(	T	Chr09_16010	TCGGATTTT(	INDEL	7901.83	18	[illegible]
Chr09	16123410	A	ATTGT	Chr09_16123	A_ATTGT	INDEL	4810.02	9	[illegible]
Chr09	16138745	C	G	Chr09_16138	C_G	SNP	12784.63	27	[illegible]
Chr09	16141208	G	T	Chr09_16141	G_T	SNP	18474.63	25	[illegible]
Chr09	16141218	G	T	Chr09_16141	G_T	SNP	18033.63	25	[illegible]
Chr09	16141265	C	CA	Chr09_16141	C_CA	INDEL	13478.59	23	[illegible]
Chr09	16179557	G	GTA	Chr09_16179	G_GTA	INDEL	208.37	0	[illegible]
Chr09	16179557	G	GTA	Chr09_16179	G_GTA	INDEL	208.37	0	[illegible]
Chr09	16179557	G	GTA	Chr09_16179	G_GTA	INDEL	208.37	0	[illegible]
Chr09	16179557	G	GTA	Chr09_16179	G_GTA	INDEL	208.37	0	[illegible]
Chr09	16179557	G	GTA	Chr09_16179	G_GTA	INDEL	208.37	0	[illegible]
Chr09	16310185	G	A	Chr09_16310	G_A	SNP	7828.63	9	[illegible]
Chr09	16313109	C	A	Chr09_16313	C_A	SNP	21205.63	41	[illegible]
Chr09	16340057	A	T	Chr09_16340	A_T	SNP	19707.63	28	[illegible]
Chr09	16340093	A	AT	Chr09_16340	A_AT	INDEL	17718.59	28	[illegible]
Chr09	16429785	GGCTGTTTG.	G	Chr09_16429	GGCTGTTTG.	INDEL	16456.59	25	[illegible]
Chr09	16605708	A	T	Chr09_16605	A_T	SNP	18622.63	37	[illegible]
Chr09	16605708	A	T	Chr09_16605	A_T	SNP	18622.63	37	[illegible]
Chr09	16605708	A	T	Chr09_16605	A_T	SNP	18622.63	37	[illegible]
Chr09	16605708	A	T	Chr09_16605	A_T	SNP	18622.63	37	[illegible]
Chr09	16605708	A	T	Chr09_16605	A_T	SNP	18622.63	37	[illegible]
Chr09	16605708	A	T	Chr09_16605	A_T	SNP	18622.63	37	[illegible]
Chr09	16605708	A	T	Chr09_16605	A_T	SNP	18622.63	37	[illegible]
Chr09	16605708	A	T	Chr09_16605	A_T	SNP	18622.63	37	[illegible]
Chr09	16605710	G	T	Chr09_16605	G_T	SNP	18622.63	37	[illegible]
Chr09	16605710	G	T	Chr09_16605	G_T	SNP	18622.63	37	[illegible]
Chr09	16605710	G	T	Chr09_16605	G_T	SNP	18622.63	37	[illegible]
Chr09	16605710	G	T	Chr09_16605	G_T	SNP	18622.63	37	[illegible]
Chr09	16605710	G	T	Chr09_16605	G_T	SNP	18622.63	37	[illegible]
Chr09	16605710	G	T	Chr09_16605	G_T	SNP	18622.63	37	[illegible]
Chr09	16605710	G	T	Chr09_16605	G_T	SNP	18622.63	37	[illegible]
Chr09	16605710	G	T	Chr09_16605	G_T	SNP	18622.63	37	[illegible]
Chr09	16648645	G	GT	Chr09_16648	G_GT	INDEL	17743.59	30	[illegible]
Chr09	16648645	G	GT	Chr09_16648	G_GT	INDEL	17743.59	30	[illegible]
Chr09	16648645	G	GT	Chr09_16648	G_GT	INDEL	17743.59	30	[illegible]
Chr09	16648645	G	GT	Chr09_16648	G_GT	INDEL	17743.59	30	[illegible]
Chr09	16648645	G	GT	Chr09_16648	G_GT	INDEL	17743.59	30	[illegible]
Chr09	16648645	G	GT	Chr09_16648	G_GT	INDEL	17743.59	30	[illegible]
Chr09	16671372	AAT	A	Chr09_16671	AAT_A	INDEL	6548.59	9	[illegible]
Chr09	16671372	AAT	A	Chr09_16671	AAT_A	INDEL	6548.59	9	[illegible]
Chr09	16671372	AAT	A	Chr09_16671	AAT_A	INDEL	6548.59	9	[illegible]
Chr09	16671372	AAT	A	Chr09_16671	AAT_A	INDEL	6548.59	9	[illegible]
Chr09	16671372	AAT	A	Chr09_16671	AAT_A	INDEL	6548.59	9	[illegible]
Chr09	16671372	AAT	A	Chr09_16671	AAT_A	INDEL	6548.59	9	[illegible]
Chr09	16671372	AAT	A	Chr09_16671	AAT_A	INDEL	6548.59	9	[illegible]
Chr09	16671372	AAT	A	Chr09_16671	AAT_A	INDEL	6548.59	9	[illegible]
Chr09	16671372	AAT	A	Chr09_16671	AAT_A	INDEL	6548.59	9	[illegible]
Chr09	16671372	AAT	A	Chr09_16671	AAT_A	INDEL	6548.59	9	[illegible]
Chr09	16776672	C	CAT	Chr09_16776	C_CAT	INDEL	928.35	20	[illegible]
Chr09	16776672	C	CAT	Chr09_16776	C_CAT	INDEL	928.35	20	[illegible]
Chr09	16776672	C	CAT	Chr09_16776	C_CAT	INDEL	928.35	20	[illegible]
Chr09	16776672	C	CAT	Chr09_16776	C_CAT	INDEL	928.35	20	[illegible]
Chr09	16776672	C	CAT	Chr09_16776	C_CAT	INDEL	928.35	20	[illegible]
Chr09	16776672	C	CAT	Chr09_16776	C_CAT	INDEL	928.35	20	[illegible]
Chr09	16776672	C	CAT	Chr09_16776	C_CAT	INDEL	928.35	20	[illegible]
Chr09	16776672	C	CAT	Chr09_16776	C_CAT	INDEL	928.35	20	[illegible]
Chr09	16776672	C	CAT	Chr09_16776	C_CAT	INDEL	928.35	20	[illegible]
Chr09	16776672	C	CAT	Chr09_16776	C_CAT	INDEL	928.35	20	[illegible]
Chr09	16776672	C	CAT	Chr09_16776	C_CAT	INDEL	928.35	20	[illegible]
Chr09	16776672	C	CAT	Chr09_16776	C_CAT	INDEL	928.35	20	[illegible]
Chr09	16776672	C	CAT	Chr09_16776	C_CAT	INDEL	928.35	20	[illegible]
Chr09	16776672	C	CAT	Chr09_16776	C_CAT	INDEL	928.35	20	[illegible]
Chr09	16776672	C	CAT	Chr09_16776	C_CAT	INDEL	928.35	20	[illegible]
Chr09	16805183	A	AAT	Chr09_16805	A_AAT	INDEL	2118.24	3	[illegible]
Chr09	16907140	GA	G	Chr09_16907	GA_G	INDEL	16999.59	30	[illegible]
Chr09	16907140	GA	G	Chr09_16907	GA_G	INDEL	16999.59	30	[illegible]
Chr09	16907140	GA	G	Chr09_16907	GA_G	INDEL	16999.59	30	[illegible]
Chr09	16907140	GA	G	Chr09_16907	GA_G	INDEL	16999.59	30	[illegible]
Chr09	16907140	GA	G	Chr09_16907	GA_G	INDEL	16999.59	30	[illegible]
Chr09	16907140	GA	G	Chr09_16907	GA_G	INDEL	16999.59	30	[illegible]
Chr09	16907230	TG	T	Chr09_16907	TG_T	INDEL	18416.59	23	[illegible]
Chr09	16907230	TG	T	Chr09_16907	TG_T	INDEL	18416.59	23	[illegible]
Chr09	16907230	TG	T	Chr09_16907	TG_T	INDEL	18416.59	23	[illegible]
Chr09	16907230	TG	T	Chr09_16907	TG_T	INDEL	18416.59	23	[illegible]
Chr09	16907230	TG	T	Chr09_16907	TG_T	INDEL	18416.59	23	[illegible]
Chr09	16907230	TG	T	Chr09_16907	TG_T	INDEL	18416.59	23	[illegible]
Chr09	16944995	G	A	Chr09_16944	G_A	SNP	19539.63	32	[illegible]
Chr09	17163374	GAT	G	Chr09_17163	GAT_G	INDEL	1829.35	33	[illegible]
Chr09	17163374	GAT	G	Chr09_17163	GAT_G	INDEL	1829.35	33	[illegible]
Chr09	17163374	GAT	G	Chr09_17163	GAT_G	INDEL	1829.35	33	[illegible]
Chr09	17163374	GAT	G	Chr09_17163	GAT_G	INDEL	1829.35	33	[illegible]
Chr09	17163374	GAT	G	Chr09_17163	GAT_G	INDEL	1829.35	33	[illegible]
Chr09	17163374	GAT	G	Chr09_17163	GAT_G	INDEL	1829.35	33	[illegible]
Chr09	17163374	GAT	G	Chr09_17163	GAT_G	INDEL	1829.35	33	[illegible]
Chr09	17163374	GAT	G	Chr09_17163	GAT_G	INDEL	1829.35	33	[illegible]
Chr09	17163374	GAT	G	Chr09_17163	GAT_G	INDEL	1829.35	33	[illegible]
Chr09	17163374	GAT	G	Chr09_17163	GAT_G	INDEL	1829.35	33	[illegible]
Chr09	17204428	C	T	Chr09_17204	C_T	SNP	5083.98	0	[illegible]
Chr09	17204428	C	T	Chr09_17204	C_T	SNP	5083.98	0	[illegible]
Chr09	17204428	C	T	Chr09_17204	C_T	SNP	5083.98	0	[illegible]
Chr09	17204428	C	T	Chr09_17204	C_T	SNP	5083.98	0	[illegible]
Chr09	17272197	A	G	Chr09_17272	A_G	SNP	19029.63	20	[illegible]
Chr09	17280319	A	AATAT	Chr09_17280	A_AATAT	INDEL	8660.59	0	[illegible]
Chr09	17315835	C	CAT	Chr09_17315	C_CAT	INDEL	5269.24	13	[illegible]
Chr09	17315835	C	CAT	Chr09_17315	C_CAT	INDEL	5269.24	13	[illegible]
Chr09	17315835	C	CAT	Chr09_17315	C_CAT	INDEL	5269.24	13	[illegible]
Chr09	17315835	C	CAT	Chr09_17315	C_CAT	INDEL	5269.24	13	[illegible]
Chr09	17315835	C	CAT	Chr09_17315	C_CAT	INDEL	5269.24	13	[illegible]
Chr09	17315835	C	CAT	Chr09_17315	C_CAT	INDEL	5269.24	13	[illegible]
Chr09	17315835	C	CAT	Chr09_17315	C_CAT	INDEL	5269.24	13	[illegible]
Chr09	17315835	C	CAT	Chr09_17315	C_CAT	INDEL	5269.24	13	[illegible]
Chr09	17357025	T	C	Chr09_17357	T_C	SNP	17082.63	21	[illegible]
Chr09	17357025	T	C	Chr09_17357	T_C	SNP	17082.63	21	[illegible]
Chr09	17357025	T	C	Chr09_17357	T_C	SNP	17082.63	21	[illegible]
Chr09	17357025	T	C	Chr09_17357	T_C	SNP	17082.63	21	[illegible]
Chr09	17357025	T	C	Chr09_17357	T_C	SNP	17082.63	21	[illegible]
Chr09	17357025	T	C	Chr09_17357	T_C	SNP	17082.63	21	[illegible]
Chr09	17357025	T	C	Chr09_17357	T_C	SNP	17082.63	21	[illegible]
Chr09	17357025	T	C	Chr09_17357	T_C	SNP	17082.63	21	[illegible]
Chr09	17471763	C	CT	Chr09_17471	C_CT	INDEL	16234.59	36	[illegible]
Chr09	17474512	A	AAGAT	Chr09_17474	A_AAGAT	INDEL	21174.59	32	[illegible]
Chr09	17515477	C	T	Chr09_17515	C_T	SNP	16568.63	26	[illegible]
Chr09	17664809	C	CTA	Chr09_17664	C_CTA	INDEL	1614.93	1	[illegible]
Chr09	17684184	C	CTA	Chr09_17684	C_CTA	INDEL	8416.59	19	[illegible]
Chr10	114253	GTACATTGC(	G	Chr10_11425	GTACATTGC(	INDEL	9183.59	10	[illegible]
Chr10	235778	T	A	Chr10_23577	T_A	SNP	13099.63	24	[illegible]
Chr10	235779	T	A	Chr10_23577	T_A	SNP	13099.63	24	[illegible]
Chr10	371828	G	GAT	Chr10_37182	G_GAT	INDEL	4060.24	0	[illegible]
Chr10	436407	G	T	Chr10_43640	G_T	SNP	10407.29	14	[illegible]
Chr10	671072	A	C	Chr10_67107	A_C	SNP	9913.63	14	[illegible]
Chr10	684393	T	C	Chr10_68439	T_C	SNP	18755.63	27	[illegible]
Chr10	700881	T	A	Chr10_70088	T_A	SNP	6865.63	8	[illegible]
Chr10	700883	G	C	Chr10_70088	G_C	SNP	6865.63	8	[illegible]
Chr10	726440	G	C	Chr10_72644	G_C	SNP	18086.63	37	[illegible]
Chr10	726490	C	A	Chr10_72649	C_A	SNP	18696.63	34	[illegible]
Chr10	726510	A	T	Chr10_72651	A_T	SNP	18842.63	33	[illegible]
Chr10	767232	TG	T	Chr10_76723	TG_T	INDEL	11508.59	17	[illegible]
Chr10	767264	T	A	Chr10_76726	T_A	SNP	12403.63	20	[illegible]
Chr10	785544	G	T	Chr10_78554	G_T	SNP	16699.63	29	[illegible]
Chr10	785574	G	C	Chr10_78557	G_C	SNP	16056.63	25	[illegible]
Chr10	832830	CT	C	Chr10_83283	CT_C	INDEL	16387.59	32	[illegible]
Chr10	849900	G	A	Chr10_84990	G_A	SNP	3890.98	0	[illegible]
Chr10	947511	T	C	Chr10_94751	T_C	SNP	15070.63	22	[illegible]
Chr10	947518	G	A	Chr10_94751	G_A	SNP	15885.63	24	[illegible]
Chr10	947524	A	T	Chr10_94752	A_T	SNP	16149.63	26	[illegible]
Chr10	947528	T	C	Chr10_94752	T_C	SNP	16121.63	26	[illegible]
Chr10	999375	C	T	Chr10_99937	C_T	SNP	2085.39	30	[illegible]
Chr10	1097688	C	CA	Chr10_10976	C_CA	INDEL	11999.59	24	[illegible]
Chr10	1102321	G	T	Chr10_11023	G_T	SNP	13747.63	23	[illegible]
Chr10	1102424	C	A	Chr10_11024	C_A	SNP	17221.63	25	[illegible]
Chr10	1102438	C	G	Chr10_11024	C_G	SNP	17453.63	23	[illegible]
Chr10	1165037	C	T	Chr10_11650	C_T	SNP	7037.29	0	[illegible]
Chr10	1185698	G	A	Chr10_11856	G_A	SNP	4855.98	0	[illegible]
Chr10	1190707	A	AAT	Chr10_11907	A_AAT	INDEL	565.37	0	[illegible]
Chr10	1260834	C	CAT	Chr10_12608	C_CAT	INDEL	8730.59	18	[illegible]
Chr10	1260834	C	CAT	Chr10_12608	C_CAT	INDEL	8730.59	18	[illegible]
Chr10	1260834	C	CAT	Chr10_12608	C_CAT	INDEL	8730.59	18	[illegible]
Chr10	1312339	A	G	Chr10_13123	A_G	SNP	9408.63	18	[illegible]
Chr10	1312339	A	G	Chr10_13123	A_G	SNP	9408.63	18	[illegible]
Chr10	1312339	A	G	Chr10_13123	A_G	SNP	9408.63	18	[illegible]
Chr10	1567147	AC	A	Chr10_15671	AC_A	INDEL	18115.59	29	[illegible]
Chr10	1567203	T	A	Chr10_15672	T_A	SNP	20199.63	32	[illegible]
Chr10	1568671	A	T	Chr10_15686	A_T	SNP	16285.63	20	[illegible]
Chr10	1568704	G	A	Chr10_15687	G_A	SNP	15365.63	20	[illegible]
Chr10	1568706	A	T	Chr10_15687	A_T	SNP	15185.63	20	[illegible]
Chr10	1568720	TTAA	T	Chr10_15687	TTAA_T	INDEL	15536.59	21	[illegible]

Chr10	1568726	G	A	Chr10_15687G_A	SNP	15365.63	21	10	5	27	8	13	22	25	14	21	5	13	10	5	[illegible]
Chr10	1568730	T	TAGC	Chr10_15687T_TAGC	INDEL	15353.59	22	10	5	29	7	13	21	25	13	21	5	11	9	6	[illegible]
Chr10	1633618	C	A	Chr10_16336C_A	SNP	19686.63	23	20	18	24	22	31	19	20	17	26	14	12	14	7	[illegible]
Chr10	1633619	T	A	Chr10_16336T_A	SNP	19686.63	21	20	20	24	22	30	19	20	17	25	14	12	14	6	[illegible]
Chr10	1646941	C	CT	Chr10_16469C_CT	INDEL	9732.59	18	12	10	16	8	20	24	14	7	15	7	8	4	8	[illegible]
Chr10	1646941	C	CT	Chr10_16469C_CT	INDEL	9732.59	18	12	10	16	8	20	24	14	7	15	7	8	4	8	[illegible]
Chr10	1646941	C	CT	Chr10_16469C_CT	INDEL	9732.59	18	12	10	16	8	20	24	14	7	15	7	8	4	8	[illegible]
Chr10	1671411	A	AAT	Chr10_16714A_AAT	INDEL	2626.24	1	0	1	8	20	11	18	13	0	1	0	0	0	0	[illegible]
Chr10	1671411	A	AAT	Chr10_16714A_AAT	INDEL	2626.24	1	0	1	8	20	11	18	13	0	1	0	0	0	0	[illegible]
Chr10	1671411	A	AAT	Chr10_16714A_AAT	INDEL	2626.24	1	0	1	8	20	11	18	13	0	1	0	0	0	0	[illegible]
Chr10	1671411	A	AAT	Chr10_16714A_AAT	INDEL	2626.24	1	0	1	8	20	11	18	13	0	1	0	0	0	0	[illegible]
Chr10	1671411	A	AAT	Chr10_16714A_AAT	INDEL	2626.24	1	0	1	8	20	11	18	13	0	1	0	0	0	0	[illegible]
Chr10	1671411	A	AAT	Chr10_16714A_AAT	INDEL	2626.24	1	0	1	8	20	11	18	13	0	1	0	0	0	0	[illegible]
Chr10	1671411	A	AAT	Chr10_16714A_AAT	INDEL	2626.24	1	0	1	8	20	11	18	13	0	1	0	0	0	0	[illegible]
Chr10	1671411	A	AAT	Chr10_16714A_AAT	INDEL	2626.24	1	0	1	8	20	11	18	13	0	1	0	0	0	0	[illegible]
Chr10	1801786	C	CA	Chr10_18017C_CA	INDEL	15856.59	23	15	12	22	16	39	21	30	28	23	23	21	14	19	[illegible]
Chr10	1977000	C	A	Chr10_19770C_A	SNP	11928.63	16	9	10	16	13	17	12	18	8	11	10	9	17	8	[illegible]
Chr10	1977000	C	A	Chr10_19770C_A	SNP	11928.63	16	9	10	16	13	17	12	18	8	11	10	9	17	8	[illegible]
Chr10	1977067	C	T	Chr10_19770C_T	SNP	13796.63	13	9	13	24	17	22	10	20	12	13	8	10	15	7	[illegible]
Chr10	1977067	C	T	Chr10_19770C_T	SNP	13796.63	13	9	13	24	17	22	10	20	12	13	8	10	15	7	[illegible]
Chr10	2161151	T	TTA	Chr10_21611T_TTA	INDEL	1310.35	26	12	7	0	0	0	0	0	0	1	0	0	0	1	[illegible]
Chr10	2161151	T	TTA	Chr10_21611T_TTA	INDEL	1310.35	26	12	7	0	0	0	0	0	0	1	0	0	0	1	[illegible]
Chr10	2161151	T	TTA	Chr10_21611T_TTA	INDEL	1310.35	26	12	7	0	0	0	0	0	0	1	0	0	0	1	[illegible]
Chr10	2161151	T	TTA	Chr10_21611T_TTA	INDEL	1310.35	26	12	7	0	0	0	0	0	0	1	0	0	0	1	[illegible]
[illegible]																					

Chr10	6427848	G	T	Chr10_64278G_T	SNP	21610.63	44	21	12	30	25	31	40	27	17	28	15	13	12	10	[illegible]
Chr10	6427848	G	T	Chr10_64278G_T	SNP	21610.63	44	21	12	30	25	31	40	27	17	28	15	13	12	10	[illegible]
Chr10	6427848	G	T	Chr10_64278G_T	SNP	21610.63	44	21	12	30	25	31	40	27	17	28	15	13	12	10	[illegible]
Chr10	6427848	G	T	Chr10_64278G_T	SNP	21610.63	44	21	12	30	25	31	40	27	17	28	15	13	12	10	[illegible]
Chr10	6427848	G	T	Chr10_64278G_T	SNP	21610.63	44	21	12	30	25	31	40	27	17	28	15	13	12	10	[illegible]
Chr10	6427855	G	A	Chr10_64278G_A	SNP	21870.63	44	17	10	29	23	31	40	28	19	29	15	14	13	10	[illegible]
Chr10	6427855	G	A	Chr10_64278G_A	SNP	21870.63	44	17	10	29	23	31	40	28	19	29	15	14	13	10	[illegible]
Chr10	6427855	G	A	Chr10_64278G_A	SNP	21870.63	44	17	10	29	23	31	40	28	19	29	15	14	13	10	[illegible]
Chr10	6427855	G	A	Chr10_64278G_A	SNP	21870.63	44	17	10	29	23	31	40	28	19	29	15	14	13	10	[illegible]
Chr10	6427855	G	A	Chr10_64278G_A	SNP	21870.63	44	17	10	29	23	31	40	28	19	29	15	14	13	10	[illegible]
Chr10	6427920	G	C	Chr10_64279G_C	SNP	19978.63	40	20	15	22	19	35	27	23	27	29	13	18	11	13	[illegible]
Chr10	6427920	G	C	Chr10_64279G_C	SNP	19978.63	40	20	15	22	19	35	27	23	27	29	13	18	11	13	[illegible]
Chr10	6427920	G	C	Chr10_64279G_C	SNP	19978.63	40	20	15	22	19	35	27	23	27	29	13	18	11	13	[illegible]
Chr10	6427920	G	C	Chr10_64279G_C	SNP	19978.63	40	20	15	22	19	35	27	23	27	29	13	18	11	13	[illegible]
Chr10	6427920	G	C	Chr10_64279G_C	SNP	19978.63	40	20	15	22	19	35	27	23	27	29	13	18	11	13	[illegible]
Chr10	6696789	AT	A	Chr10_66967AT_A	INDEL	14159.59	23	16	18	41	29	30	16	19	24	17	20	12	8	11	[illegible]
Chr10	6696819	T	A	Chr10_66968T_A	SNP	19361.63	22	18	17	39	32	30	19	20	18	20	16	13	7	12	[illegible]
Chr10	6786519	T	TA	Chr10_67865T_TA	INDEL	15129.59	20	14	15	21	19	20	24	19	13	13	7	12	4	7	[illegible]
Chr10	6786540	A	AT	Chr10_67865A_AT	INDEL	15630.59	22	13	15	26	18	19	29	21	14	15	8	9	4	6	[illegible]
Chr10	6797345	C	G	Chr10_67973C_G	SNP	22059.63	30	13	24	31	33	35	36	21	27	26	18	26	7	9	[illegible]
Chr10	6797353	G	T	Chr10_67973G_T	SNP	22473.63	32	14	25	27	33	35	38	22	26	25	17	22	9	10	[illegible]
Chr10	6797355	T	C	Chr10_67973T_C	SNP	22441.63	32	13	25	27	33	33	38	22	25	25	17	20	10	10	[illegible]
Chr10	6797357	A	C	Chr10_67973A_C	SNP	21946.63	32	13	25	27	34	33	38	22	27	24	17	20	10	9	[illegible]
Chr10	6845301	C	CTA	Chr10_68453C_CTA	INDEL	2244.24	0	0	1	14	10	14	16	10	0	0	2	0	0	2	[illegible]
Chr10	6845301	C	CTA	Chr10_68453C_CTA	INDEL	2244.24	0	0	1	14	10	14	16	10	0	0	2	0	0	2	[illegible]
Chr10	6922126	TA	T	Chr10_69221TA_T	INDEL	15727.59	31	14	15	30	15	27	26	17	15	19	20	32	10	14	[illegible]
Chr10	6922126	TA	T	Chr10_69221TA_T	INDEL	15727.59	31	14	15	30	15	27	26	17	15	19	20	32	10	14	[illegible]
Chr10	6922126	TA	T	Chr10_69221TA_T	INDEL	15727.59	31	14	15	30	15	27	26	17	15	19	20	32	10	14	[illegible]
Chr10	6922155	TA	T	Chr10_69221TA_T	INDEL	14579.59	32	10	14	27	15	32	27	18	15	17	16	27	8	13	[illegible]
Chr10	6922155	TA	T	Chr10_69221TA_T	INDEL	14579.59	32	10	14	27	15	32	27	18	15	17	16	27	8	13	[illegible]
Chr10	6922155	TA	T	Chr10_69221TA_T	INDEL	14579.59	32	10	14	27	15	32	27	18	15	17	16	27	8	13	[illegible]
Chr10	7203126	AG	A	Chr10_72031AG_A	INDEL	18647.59	25	6	17	26	16	38	33	19	18	28	12	26	11	10	[illegible]
Chr10	7423066	C	T	Chr10_74230C_T	SNP	6256.29	0	0	0	23	21	28	31	20	0	0	0	0	0	0	[illegible]
Chr10	7519365	C	CTA	Chr10_75193C_CTA	INDEL	3126.24	0	0	0	17	6	23	26	11	0	0	0	0	0	0	[illegible]
Chr10	7519365	C	CTA	Chr10_75193C_CTA	INDEL	3126.24	0	0	0	17	6	23	26	11	0	0	0	0	0	0	[illegible]
Chr10	7519365	C	CTA	Chr10_75193C_CTA	INDEL	3126.24	0	0	0	17	6	23	26	11	0	0	0	0	0	0	[illegible]
Chr10	7519365	C	CTA	Chr10_75193C_CTA	INDEL	3126.24	0	0	0	17	6	23	26	11	0	0	0	0	0	0	[illegible]
Chr10	7519365	C	CTA	Chr10_75193C_CTA	INDEL	3126.24	0	0	0	17	6	23	26	11	0	0	0	0	0	0	[illegible]
Chr10	7519365	C	CTA	Chr10_75193C_CTA	INDEL	3126.24	0	0	0	17	6	23	26	11	0	0	0	0	0	0	[illegible]
Chr10	7519365	C	CTA	Chr10_75193C_CTA	INDEL	3126.24	0	0	0	17	6	23	26	11	0	0	0	0	0	0	[illegible]
Chr10	7519365	C	CTA	Chr10_75193C_CTA	INDEL	3126.24	0	0	0	17	6	23	26	11	0	0	0	0	0	0	[illegible]
Chr10	7519365	C	CTA	Chr10_75193C_CTA	INDEL	3126.24	0	0	0	17	6	23	26	11	0	0	0	0	0	0	[illegible]
Chr10	7519365	C	CTA	Chr10_75193C_CTA	INDEL	3126.24	0	0	0	17	6	23	26	11	0	0	0	0	0	0	[illegible]
Chr10	7519365	C	CTA	Chr10_75193C_CTA	INDEL	3126.24	0	0	0	17	6	23	26	11	0	0	0	0	0	0	[illegible]
Chr10	7519365	C	CTA	Chr10_75193C_CTA	INDEL	3126.24	0	0	0	17	6	23	26	11	0	0	0	0	0	0	[illegible]
Chr10	7519365	C	CTA	Chr10_75193C_CTA	INDEL	3126.24	0	0	0	17	6	23	26	11	0	0	0	0	0	0	[illegible]
Chr10	7635827	GC	G	Chr10_76358GC_G	INDEL	9591.59	13	15	8	21	7	15	19	11	11	9	12	9	6	9	[illegible]
Chr10	7635827	GC	G	Chr10_76358GC_G	INDEL	9591.59	13	15	8	21	7	15	19	11	11	9	12	9	6	9	[illegible]
Chr10	7635827	GC	G	Chr10_76358GC_G	INDEL	9591.59	13	15	8	21	7	15	19	11	11	9	12	9	6	9	[illegible]
Chr10	7635827	GC	G	Chr10_76358GC_G	INDEL	9591.59	13	15	8	21	7	15	19	11	11	9	12	9	6	9	[illegible]
Chr10	7635827	GC	G	Chr10_76358GC_G	INDEL	9591.59	13	15	8	21	7	15	19	11	11	9	12	9	6	9	[illegible]
Chr10	7635827	GC	G	Chr10_76358GC_G	INDEL	9591.59	13	15	8	21	7	15	19	11	11	9	12	9	6	9	[illegible]
Chr10	7635827	GC	G	Chr10_76358GC_G	INDEL	9591.59	13	15	8	21	7	15	19	11	11	9	12	9	6	9	[illegible]
Chr10	7635827	GC	G	Chr10_76358GC_G	INDEL	9591.59	13	15	8	21	7	15	19	11	11	9	12	9	6	9	[illegible]
Chr10	7635827	GC	G	Chr10_76358GC_G	INDEL	9591.59	13	15	8	21	7	15	19	11	11	9	12	9	6	9	[illegible]
Chr10	7667788	G	T	Chr10_76677G_T	SNP	12638.63	19	14	14	17	9	18	24	18	13	17	12	14	9	7	[illegible]
Chr10	7667788	G	T	Chr10_76677G_T	SNP	12638.63	19	14	14	17	9	18	24	18	13	17	12	14	9	7	[illegible]
Chr10	7667788	G	T	Chr10_76677G_T	SNP	12638.63	19	14	14	17	9	18	24	18	13	17	12	14	9	7	[illegible]
Chr10	7667788	G	T	Chr10_76677G_T	SNP	12638.63	19	14	14	17	9	18	24	18	13	17	12	14	9	7	[illegible]
Chr10	7667788	G	T	Chr10_76677G_T	SNP	12638.63	19	14	14	17	9	18	24	18	13	17	12	14	9	7	[illegible]
Chr10	7667788	G	T	Chr10_76677G_T	SNP	12638.63	19	14	14	17	9	18	24	18	13	17	12	14	9	7	[illegible]
Chr10	7667788	G	T	Chr10_76677G_T	SNP	12638.63	19	14	14	17	9	18	24	18	13	17	12	14	9	7	[illegible]
Chr10	7667788	G	T	Chr10_76677G_T	SNP	12638.63	19	14	14	17	9	18	24	18	13	17	12	14	9	7	[illegible]
Chr10	7667788	G	T	Chr10_76677G_T	SNP	12638.63	19	14	14	17	9	18	24	18	13	17	12	14	9	7	[illegible]
Chr10	7667788	G	T	Chr10_76677G_T	SNP	12638.63	19	14	14	17	9	18	24	18	13	17	12	14	9	7	[illegible]
Chr10	7667788	G	T	Chr10_76677G_T	SNP	12638.63	19	14	14	17	9	18	24	18	13	17	12	14	9	7	[illegible]
Chr10	7737070	AACACATGG	A	Chr10_77370AACACATGG	INDEL	15346.59	26	14	11	19	21	13	25	11	18	14	22	11	10	9	[illegible]
Chr10	7737070	AACACATGG	A	Chr10_77370AACACATGG	INDEL	15346.59	26	14	11	19	21	13	25	11	18	14	22	11	10	9	[illegible]
Chr10	7765914	C	A	Chr10_77659C_A	SNP	20512.63	30	15	15	36	21	37	30	29	30	27	15	20	11	12	[illegible]
Chr10	7766046	C	CT	Chr10_77660C_CT	INDEL	18835.59	35	20	15	30	18	34	36	25	26	36	9	13	10	20	[illegible]
Chr10	7766896	C	CAAATCTTA	Chr10_77668C_CAAATCTT	INDEL	23005.59	32	15	13	28	20	23	36	19	26	33	20	13	11	12	[illegible]
Chr10	7886687	C	CT	Chr10_78866C_CT	INDEL	16355.59	23	19	24	21	20	21	27	19	19	26	14	23	9	7	[illegible]
Chr10	7886710	A	AT	Chr10_78867A_AT	INDEL	15753.59	28	17	26	21	18	20	31	23	21	27	14	22	11	8	[illegible]
Chr10	7886887	G	T	Chr10_78868G_T	SNP	16666.63	25	12	19	23	20	15	19	21	27	22	21	13	5	11	[illegible]
Chr10	8046110	C	T	Chr10_80461C_T	SNP	20388.63	25	18	18	29	26	31	16	20	25	28	11	14	11	14	[illegible]
Chr10	8046132	C	A	Chr10_80461C_A	SNP	20550.63	25	19	16	25	24	32	17	21	26	26	12	14	11	14	[illegible]
Chr10	8059772	G	GTA	Chr10_80597G_GTA	INDEL	9842.59	19	7	7	13	18	14	26	17	18	17	16	13	8	6	[illegible]
Chr10	8059772	G	GTA	Chr10_80597G_GTA	INDEL	9842.59	19	7	7	13	18	14	26	17	18	17	16	13	8	6	[illegible]
Chr10	8059772	G	GTA	Chr10_80597G_GTA	INDEL	9842.59	19	7	7	13	18	14	26	17	18	17	16	13	8	6	[illegible]
Chr10	8059772	G	GTA	Chr10_80597G_GTA	INDEL	9842.59	19	7	7	13	18	14	26	17	18	17	16	13	8	6	[illegible]
Chr10	8059772	G	GTA	Chr10_80597G_GTA	INDEL	9842.59	19	7	7	13	18	14	26	17	18	17	16	13	8	6	[illegible]
Chr10	8137019	GT	G	Chr10_81370GT_G	INDEL	15886.59	24	13	17	23	33	23	18	18	26	28	19	21	8	11	[illegible]
Chr10	8354669	AATAT	A	Chr10_83546AATAT_A	INDEL	4373.24	8	7	11	0	0	0	0	0	0	0	0	0	0	1	[illegible]
Chr10	8354669	AATAT	A	Chr10_83546AATAT_A	INDEL	4373.24	8	7	11	0	0	0	0	0	0	0	0	0	0	1	[illegible]
Chr10	8354669	AATAT	A	Chr10_83546AATAT_A	INDEL	4373.24	8	7	11	0	0	0	0	0	0	0	0	0	0	1	[illegible]
Chr10	8354669	AATAT	A	Chr10_83546AATAT_A	INDEL	4373.24	8	7	11	0	0	0	0	0	0	0	0	0	0	1	[illegible]
Chr10	8488609	G	A	Chr10_84886G_A	SNP	4381.98	0	0	0	0	0	0	0	0	15	31	19	17	9	10	[illegible]
Chr10	8792911	T	TC	Chr10_87929T_TC	INDEL	17363.59	32	10	9	28	23	28	27	19	26	18	13	14	19	7	[illegible]
Chr10	8978760	C	T	Chr10_89787C_T	SNP	4485.98	0	0	0	0	0	0	0	0	28	19	21	26	11	9	[illegible]
Chr10	8978760	C	T	Chr10_89787C_T	SNP	4485.98	0	0	0	0	0	0	0	0	28	19	21	26	11	9	[illegible]
Chr10	9087862	A	G	Chr10_90878A_G	SNP	18908.63	35	13	16	30	15	24	26	18	26	26	10	23	12	10	[illegible]
Chr10	9087864	G	C	Chr10_90878G_C	SNP	18908.63	36	13	16	30	15	25	27	18	26	26	10	23	13	10	[illegible]
Chr10	9122327	A	AT	Chr10_91223A_AT	INDEL	15228.59	29	16	11	28	24	18	20	28	18	20	13	10	6	11	[illegible]
Chr10	9122327	A	AT	Chr10_91223A_AT	INDEL	15228.59	29	16	11	28	24	18	20	28	18	20	13	10	6	11	[illegible]
Chr10	9122327	A	AT	Chr10_91223A_AT	INDEL	15228.59	29	16	11	28	24	18	20	28	18	20	13	10	6	11	[illegible]
Chr10	9122327	A	AT	Chr10_91223A_AT	INDEL	15228.59	29	16	11	28	24	18	20	28	18	20	13	10	6	11	[illegible]
Chr10	9122327	A	AT	Chr10_91223A_AT	INDEL	15228.59	29	16	11	28	24	18	20	28	18	20	13	10	6	11	[illegible]
Chr10	9122327	A	AT	Chr10_91223A_AT	INDEL	15228.59	29	16	11	28	24	18	20	28	18	20	13	10	6	11	[illegible]
Chr10	9122327	A	AT	Chr10_91223A_AT	INDEL	15228.59	29	16	11	28	24	18	20	28	18	20	13	10	6	11	[illegible]
Chr10	9122327	A	AT	Chr10_91223A_AT	INDEL	15228.59	29	16	11	28	24	18	20	28	18	20	13	10	6	11	[illegible]
Chr10	9122327	A	AT	Chr10_91223A_AT	INDEL	15228.59	29	16	11	28	24	18	20	28	18	20	13	10	6	11	[illegible]
Chr10	9122327	A	AT	Chr10_91223A_AT	INDEL	15228.59	29	16	11	28	24	18	20	28	18	20	13	10	6	11	[illegible]
Chr10	9122327	A	AT	Chr10_91223A_AT	INDEL	15228.59	29	16	11	28	24	18	20	28	18	20	13	10	6	11	[illegible]
Chr10	9122327	A	AT	Chr10_91223A_AT	INDEL	15228.59	29	16	11	28	24	18	20	28	18	20	13	10	6	11	[illegible]
Chr10	9122327	A	AT	Chr10_91223A_AT	INDEL	15228.59	29	16	11	28	24	18	20	28	18	20	13	10	6	11	[illegible]
Chr10	9122327	A	AT	Chr10_91223A_AT	INDEL	15228.59	29	16	11	28	24	18	20	28	18	20	13	10	6	11	[illegible]
Chr10	9122327	A	AT	Chr10_91223A_AT	INDEL	15228.59	29	16	11	28	24	18	20	28	18	20	13	10	6	11	[illegible]
Chr10	9122327	A	AT	Chr10_91223A_AT	INDEL	15228.59	29	16	11	28	24	18	20	28	18	20	13	10	6	11	[illegible]
Chr10	9122327	A	AT	Chr10_91223A_AT	INDEL	15228.59	29	16	11	28	24	18	20	28	18	20	13	10	6	11	[illegible]
Chr10	9122327	A	AT	Chr10_91223A_AT	INDEL	15228.59	29	16	11	28	24	18	20	28	18	20	13	10	6	11	[illegible]
Chr10	9122327	A	AT	Chr10_91223A_AT	INDEL	15228.59	29	16	11	28	24	18	20	28	18	20	13	10	6	11	[illegible]
Chr10	9172640	CGATTAAAG	C	Chr10_91726CGATTAAAG	INDEL	11934.59	19	8	9	21	9	8	21	12	18	22	8	9	5	11	[illegible]
Chr10	9172640	CGATTAAAG	C	Chr10_91726CGATTAAAG	INDEL	11934.59	19	8	9	21	9	8	21	12	18	22	8	9	5	11	[illegible]
Chr10	9172640	CGATTAAAG	C	Chr10_91726CGATTAAAG	INDEL	11934.59	19	8	9	21	9	8	21	12	18	22	8	9	5	11	[illegible]
Chr10	9172640	CGATTAAAG	C	Chr10_91726CGATTAAAG	INDEL	11934.59	19	8	9	21	9	8	21	12	18	22	8	9	5	11	[illegible]
Chr10	9172640	CGATTAAAG	C	Chr10_91726CGATTAAAG	INDEL	11934.59	19	8	9	21	9	8	21	12	18	22	8	9	5	11	[illegible]
Chr10	9172640	CGATTAAAG	C	Chr10_91726CGATTAAAG	INDEL	11934.59	19	8	9	21	9	8	21	12	18	22	8	9	5	11	[illegible]
Chr10	9218969	C	T	Chr10_92189C_T	SNP	16945.63	29	11	15	20	15	36	26	26	21	21	18	26	9	13	[illegible]
Chr10	9218969	C	T	Chr10_92189C_T	SNP	16945.63	29	11	15	20	15	36	26	26	21	21	18	26	9	13	[illegible]
Chr10	9218969	C	T	Chr10_92189C_T	SNP	16945.63	29	11	15	20	15	36	26	26	21	21	18	26	9	13	[illegible]
Chr10	9218969	C	T	Chr10_92189C_T	SNP	16945.63	29	11	15	20	15	36	26	26	21	21	18	26	9	13	[illegible]
Chr10	9218969	C	T	Chr10_92189C_T	SNP	16945.63	29	11	15	20	15	36	26	26	21	21	18	26	9	13	[illegible]
Chr10	9218969	C	T	Chr10_92189C_T	SNP	16945.63	29	11	15	20	15	36	26	26	21	21	18	26	9	13	[illegible]
Chr10	9218969	C	T	Chr10_92189C_T	SNP	16945.63	29	11	15	20	15	36	26	26	21	21	18	26	9	13	[illegible]
Chr10	9218969	C	T	Chr10_92189C_T	SNP	16945.63	29	11	15	20	15	36	26	26	21	21	18	26	9	13	[illegible]
Chr10	9218969	C	T	Chr10_92189C_T	SNP	16945.63	29	11	15	20	15	36	26	26	21	21	18	26	9	13	[illegible]
Chr10	9218969	C	T	Chr10_92189C_T	SNP	16945.63	29	11	15	20	15	36	26	26	21	21	18	26	9	13	[illegible]
Chr10	9343226	C	A	Chr10_93432C_A	SNP	13159.63	15	9	9	29	21	11	15	18	13	10	10	5	11	10	[illegible]
Chr10	9343226	C	A	Chr10_93432C_A	SNP	13159.63	15	9	9	29	21	11	15	18	13	10	10	5	11	10	[illegible]
Chr10	9374902	C	A	Chr10_93749C_A	SNP	17794.63	37	13	23	21	17	32	33	15	20	22	25	17	8	9	[illegible]
Chr10	9374902	C	A	Chr10_93749C_A	SNP	17794.63	37	13	23	21	17	32	33	15	20	22	25	17	8	9	[illegible]
Chr10	9407060	G	A	Chr10_94070G_A	SNP	4328.98	0	0	0	0	0	0	0	0	25	18	15	21	13	6	[illegible]
Chr10	9439403	C	T	Chr10_94394C_T	SNP	5576.29	0	0	0	20	25	17	31	12	0	0	0	0	0	0	[illegible]
Chr10	9490736	T	TA	Chr10_94907T_TA	INDEL	16613.59	22	15	12	29	16	33	25	31	18	19	18	17	17	10	[illegible]
Chr10	9521989	C	A	Chr10_95219C_A	SNP	16932.63	28	15	15	21	20	17	24	18	18	26	13	12	10	7	[illegible]
Chr10	9524482	TC	T	Chr10_95244TC_T	INDEL	6776.24	22	10	12	0	0	0	0	0	13	11	5	10	4	9	[illegible]
Chr10	9525432	A	G	Chr10_95254A_G	SNP	10603.29	25	6	21	0	0	0	0	1	17	19	11	21	8	18	[illegible]
Chr10	9535643	C	G	Chr10_95356C_G	SNP	15080.63	27	15	13	17	10	26	22	12	23	17	9	35	4	10	[illegible]
Chr10	9612860	TCTTTCC	T	Chr10_96128TCTTTCC_T	INDEL	18227.59	16	16	10	17	21	21	25	25	14	27	8	21	6	8	[illegible]
Chr10	9612860	TCTTTCC	T	Chr10_96128TCTTTCC_T	INDEL	18227.59	16	16	10	17	21	21	25	25	14	27	8	21	6	8	[illegible]
Chr10	9612860	TCTTTCC	T	Chr10_96128TCTTTCC_T	INDEL	18227.59	16	16	10	17	21	21	25	25	14	27	8	21	6	8	[illegible]
Chr10	9612860	TCTTTCC	T	Chr10_96128TCTTTCC_T	INDEL	18227.59	16	16	10	17	21	21	25	25	14	27	8	21	6	8	[illegible]
Chr10	9612860	TCTTTCC	T	Chr10_96128TCTTTCC_T	INDEL	18227.59	16	16	10	17	21	21	25	25	14	27	8	21	6	8	[illegible]
Chr10	9612860	TCTTTCC	T	Chr10_96128TCTTTCC_T	INDEL	18227.59	16	16	10	17	21	21	25	25	14	27	8	21	6	8	[illegible]
Chr10	9612860	TCTTTCC	T	Chr10_96128TCTTTCC_T	INDEL	18227.59	16	16	10	17	21	21	25	25	14	27	8	21	6	8	[illegible]
Chr10	9612860	TCTTTCC	T	Chr10_96128TCTTTCC_T	INDEL	18227.59	16	16	10	17	21	21	25	25	14	27	8	21	6	8	[illegible]
Chr10	9612867	A	AGAATGT	Chr10_96128A_AGAATGT	INDEL	18227.59	16	16	10	18	21	21	27	25	14	28	8	21	6	8	[illegible]
Chr10	9612867	A	AGAATGT	Chr10_96128A_AGAATGT	INDEL	18227.59	16	16	10	18	21	21	27	25	14	28	8	21	6	8	[illegible]
Chr10	9612867	A	AGAATGT	Chr10_96128A_AGAATGT	INDEL	18227.59	16	16	10	18	21	21	27	25	14	28	8	21	6	8	[illegible]
Chr10	9612867	A	AGAATGT	Chr10_96128A_AGAATGT	INDEL	18227.59	16	16	10	18	21	21	27	25	14	28	8	21	6	8	[illegible]
Chr10	9612867	A	AGAATGT	Chr10_96128A_AGAATGT	INDEL	18227.59	16	16	10	18	21	21	27	25	14	28	8	21	6	8	[illegible]
Chr10	9612867	A	AGAATGT	Chr10_96128A_AGAATGT	INDEL	18227.59	16	16	10	18	21	21	27	25	14	28	8	21	6	8	[illegible]
Chr10	9612867	A	AGAATGT	Chr10_96128A_AGAATGT	INDEL	18227.59	16	16	10	18	21	21	27	25	14	28	8	21	6	8	[illegible]
Chr10	9612867	A	AGAATGT	Chr10_96128A_AGAATGT	INDEL	18227.59	16	16	10	18	21	21	27	25	14	28	8	21	6	8	[illegible]
Chr10	9646246	A	G	Chr10_96462A_G	SNP	10078.63	17	14	10	10	6	7	12	9	9	6	7	8	5	4	[illegible]
Chr10	9646246	A	G	Chr10_96462A_G	SNP	10078.63	17	14	10	10	6	7	12	9	9	6	7	8	5	4	[illegible]
Chr10	9646246	A	G	Chr10_96462A_G	SNP	10078.63	17	14	10	10	6	7	12	9	9	6	7	8	5	4	[illegible]
Chr10	9646246	A	G	Chr10_96462A_G	SNP	10078.63	17	14	10	10	6	7	12	9	9	6	7	8	5	4	[illegible]
Chr10	9646254	C	T	Chr10_96462C_T	SNP	10530.63	18	14	10	10	7	8	14	11	9	7	7	8	5	8	[illegible]
Chr10	9646254	C	T	Chr10_96462C_T	SNP	10530.63	18	14	10	10	7	8	14	11	9	7	7	8	5	8	[illegible]
Chr10	9646254	C	T	Chr10_96462C_T	SNP	10530.63	18	14	10	10	7	8	14	11	9	7	7	8	5	8	[illegible]
Chr10	9646254	C	T	Chr10_96462C_T	SNP	10530.63	18	14	10	10	7	8	14	11	9	7	7	8	5	8	[illegible]
Chr10	9743303	A	T	Chr10_97433A_T	SNP	10704.29	29	10	15	0	0	0	0	0	15	25	20	19	8	8	[illegible]
Chr10	9940044	T	C	Chr10_99400T_C	SNP	17838.63	23	12	15	14	11	26	31	20	13	26	17	16	14	17	[illegible]
Chr10	10242389	G	GT	Chr10_10242G_GT	INDEL	15714.59	22	9	15	28	16	15	26	30	15	18	18	18	11	6	[illegible]
Chr10	10305495	A	AT	Chr10_10305A_AT	INDEL	14833.59	23	20	21	27	18	30	21	14	18	27	17	27	11	7	[illegible]
Chr10	10305495	A	AT	Chr10_10305A_AT	INDEL	14833.59	23	20	21	27	18	30	21	14	18	27	17	27	11	7	[illegible]
Chr10	10305495	A	AT	Chr10_10305A_AT	INDEL	14833.59	23	20	21	27	18	30	21	14	18	27	17	27	11	7	[illegible]
Chr10	10427489	CATATAT	C	Chr10_10427CATATAT_C	INDEL	3153.24	0	0	0	12	11	19	16	11	0	0	0	0	0	0	[illegible]
Chr10	10427489	CATATAT	C	Chr10_10427CATATAT_C	INDEL	3153.24	0	0	0	12	11	19	16	11	0	0	0	0	0	0	[illegible]
Chr10	10427489	CATATAT	C	Chr10_10427CATATAT_C	INDEL	3153.24	0	0	0	12	11	19	16	11	0	0	0	0	0	0	[illegible]
Chr10	10427489	CATATAT	C	Chr10_10427CATATAT_C	INDEL	3153.24	0	0	0	12	11	19	16	11	0	0	0	0	0	0	[illegible]
Chr10	10427489	CATATAT	C	Chr10_10427CATATAT_C	INDEL	3153.24	0	0	0	12	11	19	16	11	0	0	0	0	0	0	[illegible]
Chr10	10427489	CATATAT	C	Chr10_10427CATATAT_C	INDEL	3153.24	0	0	0	12	11	19	16	11	0	0	0	0	0	0	[illegible]
Chr10	10427489	CATATAT	C	Chr10_10427CATATAT_C	INDEL	3153.24	0	0	0	12	11	19	16	11	0	0	0	0	0	0	[illegible]
Chr10	10517873	C	CAT	Chr10_10517C_CAT	INDEL	1701.35	30	11	12	0	1	0	0	0	0	0	0	0	0	0	[illegible]
Chr10	10517873	C	CAT	Chr10_10517C_CAT	INDEL	1701.35	30	11	12	0	1	0	0	0	0	0	0	0	0	0	[illegible]
Chr10	10517873	C	CAT	Chr10_10517C_CAT	INDEL	1701.35	30	11	12	0	1	0	0	0	0	0	0	0	0	0	[illegible]
Chr10	10517873	C	CAT	Chr10_10517C_CAT	INDEL	1701.35	30	11	12	0	1	0	0	0	0	0	0	0	0	0	[illegible]
Chr10	10517873	C	CAT	Chr10_10517C_CAT	INDEL	1701.35	30	11	12	0	1	0	0	0	0	0	0	0	0	0	[illegible]
Chr10	10517873	C	CAT	Chr10_10517C_CAT	INDEL	1701.35	30	11	12	0	1	0	0	0	0	0	0	0	0	0	[illegible]
Chr10	10517873	C	CAT	Chr10_10517C_CAT	INDEL	1701.35	30	11	12	0	1	0	0	0	0	0	0	0	0	0	[illegible]
Chr10	10596148	C	A	Chr10_10596C_A	SNP	22958.63	28	15	22	42	17	28	15	25	34	32	31	21	8	14	[illegible]
Chr10	10596150	A	C	Chr10_10596A_C	SNP	22958.63	28	15	22	42	17	28	15	25	32	33	30	20	8	15	[illegible]
Chr10	10759340	T	C	Chr10_10759T_C	SNP	21670.63	36	22	20	29	23	31	47	25	26	28	16	19	5	12	[illegible]
Chr10	10759340	T	C	Chr10_10759T_C	SNP	21670.63	36	22	20	29	23	31	47	25	26	28	16	19	5	12	[illegible]
Chr10	10764760	A	AAT	Chr10_10764A_AAT	INDEL	216.37	0	0	0	1	0	1	10	0	0	0	0	0	0	1	[illegible]
Chr10	10764760	A	AAT	Chr10_10764A_AAT	INDEL	216.37	0	0	0	1	0	1	10	0	0	0	0	0	0	1	[illegible]
Chr10	10764760	A	AAT	Chr10_10764A_AAT	INDEL	216.37	0	0	0	1	0	1	10	0	0	0	0	0	0	1	[illegible]
Chr10	10764760	A	AAT	Chr10_10764A_AAT	INDEL	216.37	0	0	0	1	0	1	10	0	0	0	0	0	0	1	[illegible]
Chr10	10764760	A	AAT	Chr10_10764A_AAT	INDEL	216.37	0	0	0	1	0	1	10	0	0	0	0	0	0	1	[illegible]
Chr10	10764760	A	AAT	Chr10_10764A_AAT	INDEL	216.37	0	0	0	1	0	1	10	0	0	0	0	0	0	1	[illegible]
Chr10	10919158	G	A	Chr10_10919G_A	SNP	6517.29	0	0	0	28	22	33	26	20	0	0	0	0	0	0	[illegible]
Chr10	11072770	C	A	Chr10_11072C_A	SNP	8340.63	13	4	4	19	12	16	14	13	8	16	4	7	4	4	[illegible]
Chr10	11072770	C	A	Chr10_11072C_A	SNP	8340.63	13	4	4	19	12	16	14	13	8	16	4	7	4	4	[illegible]
Chr10	11072770	C	A	Chr10_11072C_A	SNP	8340.63	13	4	4	19	12	16	14	13	8	16	4	7	4	4	[illegible]
Chr10	11072770	C	A	Chr10_11072C_A	SNP	8340.63	13	4	4	19	12	16	14	13	8	16	4	7	4	4	[illegible]
Chr10	11072770	C	A	Chr10_11072C_A	SNP	8340.63	13	4	4	19	12	16	14	13	8	16	4	7	4	4	[illegible]
Chr10	11072770	C	A	Chr10_11072C_A	SNP	8340.63	13	4	4	19	12	16	14	13	8	16	4	7	4	4	[illegible]
Chr10	11072770	C	A	Chr10_11072C_A	SNP	8340.63	13	4	4	19	12	16	14	13	8	16	4	7	4	4	[illegible]
Chr10	11072770	C	A	Chr10_11072C_A	SNP	8340.63	13	4	4	19	12	16	14	13	8	16	4	7	4	4	[illegible]
Chr10	11072770	C	A	Chr10_11072C_A	SNP	8340.63	13	4	4	19	12	16	14	13	8	16	4	7	4	4	[illegible]

Chr10	11072770	C	A	Chr10_11072C_A	SNP	8340.63	13	4	4	19	12	16	14	13	8	16
Chr10	11093749	A	AAT	Chr10_11093A_AAT	INDEL	1913.93	0	1	0	0	1	0	0	0	12	10
Chr10	11093749	A	AAT	Chr10_11093A_AAT	INDEL	1913.93	0	1	0	0	1	0	0	0	12	10
Chr10	11093749	A	AAT	Chr10_11093A_AAT	INDEL	1913.93	0	1	0	0	1	0	0	0	12	10
Chr10	11093749	A	AAT	Chr10_11093A_AAT	INDEL	1913.93	0	1	0	0	1	0	0	0	12	10
Chr10	11093749	A	AAT	Chr10_11093A_AAT	INDEL	1913.93	0	1	0	0	1	0	0	0	12	10
Chr10	11093749	A	AAT	Chr10_11093A_AAT	INDEL	1913.93	0	1	0	0	1	0	0	0	12	10
Chr10	11093749	A	AAT	Chr10_11093A_AAT	INDEL	1913.93	0	1	0	0	1	0	0	0	12	10
Chr10	11093749	A	AAT	Chr10_11093A_AAT	INDEL	1913.93	0	1	0	0	1	0	0	0	12	10
Chr10	11251533	G	T	Chr10_11251G_T	SNP	17508.63	29	18	12	25	13	20	27	25	25	15
Chr10	11251533	G	T	Chr10_11251G_T	SNP	17508.63	29	18	12	25	13	20	27	25	25	15
Chr10	11251533	G	T	Chr10_11251G_T	SNP	17508.63	29	18	12	25	13	20	27	25	25	15
Chr10	11251533	G	T	Chr10_11251G_T	SNP	17508.63	29	18	12	25	13	20	27	25	25	15
Chr10	11251533	G	T	Chr10_11251G_T	SNP	17508.63	29	18	12	25	13	20	27	25	25	15
Chr10	11251533	G	T	Chr10_11251G_T	SNP	17508.63	29	18	12	25	13	20	27	25	25	15
Chr10	11251533	G	T	Chr10_11251G_T	SNP	17508.63	29	18	12	25	13	20	27	25	25	15
Chr10	11251533	G	T	Chr10_11251G_T	SNP	17508.63	29	18	12	25	13	20	27	25	25	15
Chr10	11251533	G	T	Chr10_11251G_T	SNP	17508.63	29	18	12	25	13	20	27	25	25	15
Chr10	11251533	G	T	Chr10_11251G_T	SNP	17508.63	29	18	12	25	13	20	27	25	25	15
Chr10	11251533	G	T	Chr10_11251G_T	SNP	17508.63	29	18	12	25	13	20	27	25	25	15
Chr10	11251533	G	T	Chr10_11251G_T	SNP	17508.63	29	18	12	25	13	20	27	25	25	15
Chr10	11372429	T	TTA	Chr10_11372T_TTA	INDEL	8317.24	25	8	14	1	0	0	0	0	24	23
Chr10	11372429	T	TTA	Chr10_11372T_TTA	INDEL	8317.24	25	8	14	1	0	0	0	0	24	23
Chr10	11553724	G	A	Chr10_11553G_A	SNP	12421.29	30	11	26	0	0	0	0	1	18	22

Chr10	16563066	A	AAT	Chr10_16563A_AAT	INDEL	2538.24	0	0	2	20	11	15	18	12	0	2	0	0	0	0
Chr10	16563066	A	AAT	Chr10_16563A_AAT	INDEL	2538.24	0	0	2	20	11	15	18	12	0	2	0	0	0	0
Chr10	16702562	GAT	G	Chr10_16702GAT_G	INDEL	8999.59	12	9	12	11	19	16	18	26	8	16	12	7	6	9
Chr10	16745831	G	T	Chr10_16745G_T	SNP	19542.63	36	6	23	31	24	30	29	31	18	24	15	15	17	7
Chr10	16745869	T	G	Chr10_16745T_G	SNP	20362.63	29	4	20	30	25	30	24	28	20	22	12	15	20	6
Chr10	16839843	T	A	Chr10_16839T_A	SNP	20501.63	28	15	12	24	27	29	36	23	26	25	10	24	17	11
Chr10	16842011	CT	C	Chr10_16842CT_C	INDEL	11989.59	30	13	12	26	22	23	26	21	13	27	11	11	8	9
Chr10	16842011	CT	C	Chr10_16842CT_C	INDEL	11989.59	30	13	12	26	22	23	26	21	13	27	11	11	8	9
Chr10	16842011	CT	C	Chr10_16842CT_C	INDEL	11989.59	30	13	12	26	22	23	26	21	13	27	11	11	8	9
Chr10	16876958	C	T	Chr10_16876C_T	SNP	16808.63	20	12	22	25	26	18	40	22	23	14	17	10	6	8
Chr10	16876958	C	T	Chr10_16876C_T	SNP	16808.63	20	12	22	25	26	18	40	22	23	14	17	10	6	8
Chr10	16876958	C	T	Chr10_16876C_T	SNP	16808.63	20	12	22	25	26	18	40	22	23	14	17	10	6	8
Chr10	16877007	G	T	Chr10_16877G_T	SNP	17117.63	20	10	21	19	24	26	37	24	22	17	15	9	7	10
Chr10	16877007	G	T	Chr10_16877G_T	SNP	17117.63	20	10	21	19	24	26	37	24	22	17	15	9	7	10
Chr10	16877007	G	T	Chr10_16877G_T	SNP	17117.63	20	10	21	19	24	26	37	24	22	17	15	9	7	10
Chr10	17087008	G	GT	Chr10_17087G_GT	INDEL	36958.59	62	28	31	61	47	45	76	41	61	41	32	45	32	27
Chr10	17087008	G	GT	Chr10_17087G_GT	INDEL	36958.59	62	28	31	61	47	45	76	41	61	41	32	45	32	27
Chr10	17087008	G	GT	Chr10_17087G_GT	INDEL	36958.59	62	28	31	61	47	45	76	41	61	41	32	45	32	27
Chr10	17087008	G	GT	Chr10_17087G_GT	INDEL	36958.59	62	28	31	61	47	45	76	41	61	41	32	45	32	27
Chr10	17087008	G	GT	Chr10_17087G_GT	INDEL	36958.59	62	28	31	61	47	45	76	41	61	41	32	45	32	27
Chr10	17087008	G	GT	Chr10_17087G_GT	INDEL	36958.59	62	28	31	61	47	45	76	41	61	41	32	45	32	27
Chr10	17120069	CA	C	Chr10_17120CA_C	INDEL	13630.59	15	13	17	14	18	21	30	22	21	13	14	18	7	8
Chr10	17120172	T	A	Chr10_17120T_A	SNP	15062.63	16	9	17	14	14	21	32	25	23	13	10	16	13	15
Chr10	17120270	T	A	Chr10_17120T_A	SNP	11728.63	15	11	13	9	12	10	24	16	22	18	13	11	13	7

[illegible]

Chr11	2416623	G	T	Chr11_24166G_T	SNP	17657.63	24	19	8	[illegible]
Chr11	2416623	G	T	Chr11_24166G_T	SNP	17657.63	24	19	8	[illegible]
Chr11	2416623	G	T	Chr11_24166G_T	SNP	17657.63	24	19	8	[illegible]
Chr11	2416623	G	T	Chr11_24166G_T	SNP	17657.63	24	19	8	[illegible]
Chr11	2416623	G	T	Chr11_24166G_T	SNP	17657.63	24	19	8	[illegible]
Chr11	2416623	G	T	Chr11_24166G_T	SNP	17657.63	24	19	8	[illegible]
Chr11	2443720	C	T	Chr11_24437C_T	SNP	13580.63	17	4	7	[illegible]
Chr11	2443721	A	G	Chr11_24437A_G	SNP	13580.63	17	4	7	[illegible]
Chr11	2443746	T	TA	Chr11_24437T_TA	INDEL	14041.59	19	5	8	[illegible]
Chr11	2699659	AT	A	Chr11_26996AT_A	INDEL	4999.24	16	13	12	[illegible]
Chr11	2749908	C	T	Chr11_27499C_T	SNP	11316.63	19	10	6	[illegible]
Chr11	2749908	C	T	Chr11_27499C_T	SNP	11316.63	19	10	6	[illegible]
Chr11	2749946	T	C	Chr11_27499T_C	SNP	9841.63	17	9	6	[illegible]
Chr11	2749946	T	C	Chr11_27499T_C	SNP	9841.63	17	9	6	[illegible]
Chr11	2912741	C	CTA	Chr11_29127C_CTA	INDEL	340.37	0	0	0	[illegible]
Chr11	2912741	C	CTA	Chr11_29127C_CTA	INDEL	340.37	0	0	0	[illegible]
Chr11	2912741	C	CTA	Chr11_29127C_CTA	INDEL	340.37	0	0	0	[illegible]
Chr11	2912741	C	CTA	Chr11_29127C_CTA	INDEL	340.37	0	0	0	[illegible]
Chr11	2912741	C	CTA	Chr11_29127C_CTA	INDEL	340.37	0	0	0	[illegible]
Chr11	2912741	C	CTA	Chr11_29127C_CTA	INDEL	340.37	0	0	0	[illegible]
Chr11	2912741	C	CTA	Chr11_29127C_CTA	INDEL	340.37	0	0	0	[illegible]
Chr11	2912741	C	CTA	Chr11_29127C_CTA	INDEL	340.37	0	0	0	[illegible]
Chr11	2912741	C	CTA	Chr11_29127C_CTA	INDEL	340.37	0	0	0	[illegible]
Chr11	2912741	C	CTA	Chr11_29127C_CTA	INDEL	340.37	0	0	0	[illegible]
Chr11	2912741	C	CTA	Chr11_29127C_CTA	INDEL	340.37	0	0	0	[illegible]
Chr11	2912741	C	CTA	Chr11_29127C_CTA	INDEL	340.37	0	0	0	[illegible]
Chr11	2912741	C	CTA	Chr11_29127C_CTA	INDEL	340.37	0	0	0	[illegible]
Chr11	2912741	C	CTA	Chr11_29127C_CTA	INDEL	340.37	0	0	0	[illegible]
Chr11	2912741	C	CTA	Chr11_29127C_CTA	INDEL	340.37	0	0	0	[illegible]
Chr11	2912741	C	CTA	Chr11_29127C_CTA	INDEL	340.37	0	0	0	[illegible]
Chr11	2912741	C	CTA	Chr11_29127C_CTA	INDEL	340.37	0	0	0	[illegible]
Chr11	2925179	G	T	Chr11_29251G_T	SNP	21513.63	38	18	23	[illegible]
Chr11	2925179	G	T	Chr11_29251G_T	SNP	21513.63	38	18	23	[illegible]
Chr11	2925179	G	T	Chr11_29251G_T	SNP	21513.63	38	18	23	[illegible]
Chr11	2925179	G	T	Chr11_29251G_T	SNP	21513.63	38	18	23	[illegible]
Chr11	2925179	G	T	Chr11_29251G_T	SNP	21513.63	38	18	23	[illegible]
Chr11	2925179	G	T	Chr11_29251G_T	SNP	21513.63	38	18	23	[illegible]
Chr11	2925179	G	T	Chr11_29251G_T	SNP	21513.63	38	18	23	[illegible]
Chr11	2925179	G	T	Chr11_29251G_T	SNP	21513.63	38	18	23	[illegible]
Chr11	2925179	G	T	Chr11_29251G_T	SNP	21513.63	38	18	23	[illegible]
Chr11	2925179	G	T	Chr11_29251G_T	SNP	21513.63	38	18	23	[illegible]
Chr11	2925179	G	T	Chr11_29251G_T	SNP	21513.63	38	18	23	[illegible]
Chr11	2925179	G	T	Chr11_29251G_T	SNP	21513.63	38	18	23	[illegible]
Chr11	2925179	G	T	Chr11_29251G_T	SNP	21513.63	38	18	23	[illegible]
Chr11	2925179	G	T	Chr11_29251G_T	SNP	21513.63	38	18	23	[illegible]
Chr11	2925179	G	T	Chr11_29251G_T	SNP	21513.63	38	18	23	[illegible]
Chr11	2925179	G	T	Chr11_29251G_T	SNP	21513.63	38	18	23	[illegible]
Chr11	2925179	G	T	Chr11_29251G_T	SNP	21513.63	38	18	23	[illegible]
Chr11	2925301	CG	C	Chr11_29253CG_C	INDEL	17043.59	21	14	17	[illegible]
Chr11	2925301	CG	C	Chr11_29253CG_C	INDEL	17043.59	21	14	17	[illegible]
Chr11	2925301	CG	C	Chr11_29253CG_C	INDEL	17043.59	21	14	17	[illegible]
Chr11	2925301	CG	C	Chr11_29253CG_C	INDEL	17043.59	21	14	17	[illegible]
Chr11	2925301	CG	C	Chr11_29253CG_C	INDEL	17043.59	21	14	17	[illegible]
Chr11	2925301	CG	C	Chr11_29253CG_C	INDEL	17043.59	21	14	17	[illegible]
Chr11	2925301	CG	C	Chr11_29253CG_C	INDEL	17043.59	21	14	17	[illegible]
Chr11	2925301	CG	C	Chr11_29253CG_C	INDEL	17043.59	21	14	17	[illegible]
Chr11	2925301	CG	C	Chr11_29253CG_C	INDEL	17043.59	21	14	17	[illegible]
Chr11	2925301	CG	C	Chr11_29253CG_C	INDEL	17043.59	21	14	17	[illegible]
Chr11	2925301	CG	C	Chr11_29253CG_C	INDEL	17043.59	21	14	17	[illegible]
Chr11	2925301	CG	C	Chr11_29253CG_C	INDEL	17043.59	21	14	17	[illegible]
Chr11	2925301	CG	C	Chr11_29253CG_C	INDEL	17043.59	21	14	17	[illegible]
Chr11	2925301	CG	C	Chr11_29253CG_C	INDEL	17043.59	21	14	17	[illegible]
Chr11	2925301	CG	C	Chr11_29253CG_C	INDEL	17043.59	21	14	17	[illegible]
Chr11	2925301	CG	C	Chr11_29253CG_C	INDEL	17043.59	21	14	17	[illegible]
Chr11	2925301	CG	C	Chr11_29253CG_C	INDEL	17043.59	21	14	17	[illegible]
Chr11	2925313	G	A	Chr11_29253G_A	SNP	17268.63	22	15	15	[illegible]
Chr11	2925313	G	A	Chr11_29253G_A	SNP	17268.63	22	15	15	[illegible]
Chr11	2925313	G	A	Chr11_29253G_A	SNP	17268.63	22	15	15	[illegible]
Chr11	2925313	G	A	Chr11_29253G_A	SNP	17268.63	22	15	15	[illegible]
Chr11	2925313	G	A	Chr11_29253G_A	SNP	17268.63	22	15	15	[illegible]
Chr11	2925313	G	A	Chr11_29253G_A	SNP	17268.63	22	15	15	[illegible]
Chr11	2925313	G	A	Chr11_29253G_A	SNP	17268.63	22	15	15	[illegible]
Chr11	2925313	G	A	Chr11_29253G_A	SNP	17268.63	22	15	15	[illegible]
Chr11	2925313	G	A	Chr11_29253G_A	SNP	17268.63	22	15	15	[illegible]
Chr11	2925313	G	A	Chr11_29253G_A	SNP	17268.63	22	15	15	[illegible]
Chr11	2925313	G	A	Chr11_29253G_A	SNP	17268.63	22	15	15	[illegible]
Chr11	2925313	G	A	Chr11_29253G_A	SNP	17268.63	22	15	15	[illegible]
Chr11	2925313	G	A	Chr11_29253G_A	SNP	17268.63	22	15	15	[illegible]
Chr11	2925313	G	A	Chr11_29253G_A	SNP	17268.63	22	15	15	[illegible]
Chr11	2925313	G	A	Chr11_29253G_A	SNP	17268.63	22	15	15	[illegible]
Chr11	2925313	G	A	Chr11_29253G_A	SNP	17268.63	22	15	15	[illegible]
Chr11	2925313	G	A	Chr11_29253G_A	SNP	17268.63	22	15	15	[illegible]
Chr11	2925325	G	T	Chr11_29253G_T	SNP	17036.63	22	14	15	[illegible]
Chr11	2925325	G	T	Chr11_29253G_T	SNP	17036.63	22	14	15	[illegible]
Chr11	2925325	G	T	Chr11_29253G_T	SNP	17036.63	22	14	15	[illegible]
Chr11	2925325	G	T	Chr11_29253G_T	SNP	17036.63	22	14	15	[illegible]
Chr11	2925325	G	T	Chr11_29253G_T	SNP	17036.63	22	14	15	[illegible]
Chr11	2925325	G	T	Chr11_29253G_T	SNP	17036.63	22	14	15	[illegible]
Chr11	2925325	G	T	Chr11_29253G_T	SNP	17036.63	22	14	15	[illegible]
Chr11	2925325	G	T	Chr11_29253G_T	SNP	17036.63	22	14	15	[illegible]
Chr11	2925325	G	T	Chr11_29253G_T	SNP	17036.63	22	14	15	[illegible]
Chr11	2925325	G	T	Chr11_29253G_T	SNP	17036.63	22	14	15	[illegible]
Chr11	2925325	G	T	Chr11_29253G_T	SNP	17036.63	22	14	15	[illegible]
Chr11	2925325	G	T	Chr11_29253G_T	SNP	17036.63	22	14	15	[illegible]
Chr11	2925325	G	T	Chr11_29253G_T	SNP	17036.63	22	14	15	[illegible]
Chr11	2925325	G	T	Chr11_29253G_T	SNP	17036.63	22	14	15	[illegible]
Chr11	2925325	G	T	Chr11_29253G_T	SNP	17036.63	22	14	15	[illegible]
Chr11	2925325	G	T	Chr11_29253G_T	SNP	17036.63	22	14	15	[illegible]
Chr11	2925325	G	T	Chr11_29253G_T	SNP	17036.63	22	14	15	[illegible]
Chr11	2925372	G	A	Chr11_29253G_A	SNP	16209.63	22	10	19	[illegible]
Chr11	2925372	G	A	Chr11_29253G_A	SNP	16209.63	22	10	19	[illegible]
Chr11	2925372	G	A	Chr11_29253G_A	SNP	16209.63	22	10	19	[illegible]
Chr11	2925372	G	A	Chr11_29253G_A	SNP	16209.63	22	10	19	[illegible]
Chr11	2925372	G	A	Chr11_29253G_A	SNP	16209.63	22	10	19	[illegible]
Chr11	2925372	G	A	Chr11_29253G_A	SNP	16209.63	22	10	19	[illegible]
Chr11	2925372	G	A	Chr11_29253G_A	SNP	16209.63	22	10	19	[illegible]
Chr11	2925372	G	A	Chr11_29253G_A	SNP	16209.63	22	10	19	[illegible]
Chr11	2925372	G	A	Chr11_29253G_A	SNP	16209.63	22	10	19	[illegible]
Chr11	2925372	G	A	Chr11_29253G_A	SNP	16209.63	22	10	19	[illegible]
Chr11	2925372	G	A	Chr11_29253G_A	SNP	16209.63	22	10	19	[illegible]
Chr11	2925372	G	A	Chr11_29253G_A	SNP	16209.63	22	10	19	[illegible]
Chr11	2925372	G	A	Chr11_29253G_A	SNP	16209.63	22	10	19	[illegible]
Chr11	2925372	G	A	Chr11_29253G_A	SNP	16209.63	22	10	19	[illegible]
Chr11	2925372	G	A	Chr11_29253G_A	SNP	16209.63	22	10	19	[illegible]
Chr11	2925372	G	A	Chr11_29253G_A	SNP	16209.63	22	10	19	[illegible]
Chr11	2925372	G	A	Chr11_29253G_A	SNP	16209.63	22	10	19	[illegible]
Chr11	3046298	T	A	Chr11_30462T_A	SNP	10942.63	17	14	9	[illegible]
Chr11	3697435	A	T	Chr11_36974A_T	SNP	21456.63	32	15	17	[illegible]
Chr11	3697436	A	T	Chr11_36974A_T	SNP	21501.63	32	15	17	[illegible]
Chr11	3697440	TC	T	Chr11_36974TC_T	INDEL	21539.59	31	16	16	[illegible]
Chr11	3697442	T	TAG	Chr11_36974T_TAG	INDEL	21325.59	32	16	16	[illegible]
Chr11	3697475	A	T	Chr11_36974A_T	SNP	20728.63	27	10	13	[illegible]
Chr11	3697478	G	T	Chr11_36974G_T	SNP	21076.63	28	11	13	[illegible]
Chr11	3697490	A	AATTATT	Chr11_36974A_AATTATT	INDEL	23096.59	29	15	15	[illegible]
Chr11	3697491	C	T	Chr11_36974C_T	SNP	14710.63	21	11	9	[illegible]
Chr11	3697495	C	T	Chr11_36974C_T	SNP	14980.63	21	11	9	[illegible]
Chr11	3697496	ACACCG	A	Chr11_36974ACACCG_A	INDEL	15241.59	22	12	9	[illegible]
Chr11	3697502	G	T	Chr11_36975G_T	SNP	15430.63	22	12	9	[illegible]
Chr11	3697504	TTCGTCCCGC	T	Chr11_36975TTCGTCCCGC	INDEL	15826.59	23	12	9	[illegible]
Chr11	3697521	A	AC	Chr11_36975A_AC	INDEL	16141.59	23	12	9	[illegible]
Chr11	3697528	A	ATCTATTTTT.	Chr11_36975A_ATCTATTT	INDEL	19246.59	25	12	11	[illegible]
Chr11	3697532	G	T	Chr11_36975G_T	SNP	19799.63	32	14	14	[illegible]
Chr11	3697533	G	T	Chr11_36975G_T	SNP	19799.63	32	14	14	[illegible]
Chr11	3920246	C	G	Chr11_39202C_G	SNP	11805.63	16	12	9	[illegible]
Chr11	3920246	C	G	Chr11_39202C_G	SNP	11805.63	16	12	9	[illegible]
Chr11	3920246	C	G	Chr11_39202C_G	SNP	11805.63	16	12	9	[illegible]
Chr11	3920246	C	G	Chr11_39202C_G	SNP	11805.63	16	12	9	[illegible]
Chr11	3920246	C	G	Chr11_39202C_G	SNP	11805.63	16	12	9	[illegible]
Chr11	3920246	C	G	Chr11_39202C_G	SNP	11805.63	16	12	9	[illegible]
Chr11	3920246	C	G	Chr11_39202C_G	SNP	11805.63	16	12	9	[illegible]
Chr11	3920246	C	G	Chr11_39202C_G	SNP	11805.63	16	12	9	[illegible]
Chr11	3987948	A	C	Chr11_39879A_C	SNP	20308.63	30	13	9	[illegible]
Chr11	4112956	G	T	Chr11_41129G_T	SNP	20880.63	26	20	17	[illegible]
Chr11	4112957	G	T	Chr11_41129G_T	SNP	21005.63	26	20	18	[illegible]
Chr11	4112963	A	C	Chr11_41129A_C	SNP	20832.63	27	20	17	[illegible]
Chr11	4112994	A	T	Chr11_41129A_T	SNP	17453.63	25	16	14	[illegible]
Chr11	4226118	T	TTA	Chr11_42261T_TTA	INDEL	2695.24	1	0	0	[illegible]
Chr11	4226118	T	TTA	Chr11_42261T_TTA	INDEL	2695.24	1	0	0	[illegible]
Chr11	4226118	T	TTA	Chr11_42261T_TTA	INDEL	2695.24	1	0	0	[illegible]
Chr11	4226118	T	TTA	Chr11_42261T_TTA	INDEL	2695.24	1	0	0	[illegible]
Chr11	4226118	T	TTA	Chr11_42261T_TTA	INDEL	2695.24	1	0	0	[illegible]
Chr11	4226118	T	TTA	Chr11_42261T_TTA	INDEL	2695.24	1	0	0	[illegible]
Chr11	4226118	T	TTA	Chr11_42261T_TTA	INDEL	2695.24	1	0	0	[illegible]
Chr11	4358815	T	C	Chr11_43588T_C	SNP	16245.63	29	21	14	[illegible]
Chr11	4533679	G	T	Chr11_45336G_T	SNP	5519.29	0	0	0	[illegible]
Chr11	4829975	T	A	Chr11_48299T_A	SNP	30042.63	63	23	30	[illegible]
Chr11	4829975	T	A	Chr11_48299T_A	SNP	30042.63	63	23	30	[illegible]
Chr11	4829975	T	A	Chr11_48299T_A	SNP	30042.63	63	23	30	[illegible]
Chr11	4829975	T	A	Chr11_48299T_A	SNP	30042.63	63	23	30	[illegible]
Chr11	4829975	T	A	Chr11_48299T_A	SNP	30042.63	63	23	30	[illegible]
Chr11	4829975	T	A	Chr11_48299T_A	SNP	30042.63	63	23	30	[illegible]
Chr11	4829975	T	A	Chr11_48299T_A	SNP	30042.63	63	23	30	[illegible]
Chr11	4829975	T	A	Chr11_48299T_A	SNP	30042.63	63	23	30	[illegible]
Chr11	4829989	G	A	Chr11_48299G_A	SNP	26495.63	48	21	27	[illegible]
Chr11	4829989	G	A	Chr11_48299G_A	SNP	26495.63	48	21	27	[illegible]
Chr11	4829989	G	A	Chr11_48299G_A	SNP	26495.63	48	21	27	[illegible]
Chr11	4829989	G	A	Chr11_48299G_A	SNP	26495.63	48	21	27	[illegible]
Chr11	4829989	G	A	Chr11_48299G_A	SNP	26495.63	48	21	27	[illegible]
Chr11	4829989	G	A	Chr11_48299G_A	SNP	26495.63	48	21	27	[illegible]
Chr11	4829989	G	A	Chr11_48299G_A	SNP	26495.63	48	21	27	[illegible]
Chr11	4829989	G	A	Chr11_48299G_A	SNP	26495.63	48	21	27	[illegible]
Chr11	5448198	C	A	Chr11_54481C_A	SNP	20140.63	34	24	19	[illegible]
Chr11	5448198	C	A	Chr11_54481C_A	SNP	20140.63	34	24	19	[illegible]
Chr11	5448198	C	A	Chr11_54481C_A	SNP	20140.63	34	24	19	[illegible]
Chr11	5448258	G	T	Chr11_54482G_T	SNP	20081.63	28	25	21	[illegible]
Chr11	5448258	G	T	Chr11_54482G_T	SNP	20081.63	28	25	21	[illegible]
Chr11	5448258	G	T	Chr11_54482G_T	SNP	20081.63	28	25	21	[illegible]
Chr11	5470881	G	T	Chr11_54708G_T	SNP	8944.63	13	5	13	[illegible]
Chr11	5470989	G	T	Chr11_54709G_T	SNP	14182.63	15	13	16	[illegible]
Chr11	5596512	C	CA	Chr11_55965C_CA	INDEL	14678.59	25	6	22	[illegible]
Chr11	5619151	T	A	Chr11_56191T_A	SNP	9920.63	12	5	4	[illegible]
Chr11	5619151	T	A	Chr11_56191T_A	SNP	9920.63	12	5	4	[illegible]
Chr11	5619151	T	A	Chr11_56191T_A	SNP	9920.63	12	5	4	[illegible]
Chr11	5619152	T	TAC	Chr11_56191T_TAC	INDEL	10091.59	12	5	4	[illegible]
Chr11	5619152	T	TAC	Chr11_56191T_TAC	INDEL	10091.59	12	5	4	[illegible]
Chr11	5619152	T	TAC	Chr11_56191T_TAC	INDEL	10091.59	12	5	4	[illegible]
Chr11	5619156	TA	T	Chr11_56191TA_T	INDEL	10676.59	12	6	6	[illegible]
Chr11	5619156	TA	T	Chr11_56191TA_T	INDEL	10676.59	12	6	6	[illegible]
Chr11	5619156	TA	T	Chr11_56191TA_T	INDEL	10676.59	12	6	6	[illegible]
Chr11	5619160	T	TAA	Chr11_56191T_TAA	INDEL	11486.59	12	6	6	[illegible]
Chr11	5619160	T	TAA	Chr11_56191T_TAA	INDEL	11486.59	12	6	6	[illegible]
Chr11	5619160	T	TAA	Chr11_56191T_TAA	INDEL	11486.59	12	6	6	[illegible]
Chr11	5619162	TG	T	Chr11_56191TG_T	INDEL	12566.59	12	6	6	[illegible]
Chr11	5619162	TG	T	Chr11_56191TG_T	INDEL	12566.59	12	6	6	[illegible]
Chr11	5619162	TG	T	Chr11_56191TG_T	INDEL	12566.59	12	6	6	[illegible]
Chr11	5619164	T	TAAAAAA	Chr11_56191T_TAAAAAA	INDEL	12431.59	12	6	6	[illegible]
Chr11	5619164	T	TAAAAAA	Chr11_56191T_TAAAAAA	INDEL	12431.59	12	6	6	[illegible]
Chr11	5619164	T	TAAAAAA	Chr11_56191T_TAAAAAA	INDEL	12431.59	12	6	6	[illegible]
Chr11	5619171	TACC	T	Chr11_56191TACC_T	INDEL	13376.59	14	6	9	[illegible]
Chr11	5619171	TACC	T	Chr11_56191TACC_T	INDEL	13376.59	14	6	9	[illegible]
Chr11	5619171	TACC	T	Chr11_56191TACC_T	INDEL	13376.59	14	6	9	[illegible]
Chr11	5619176	A	G	Chr11_56191A_G	SNP	13610.63	15	6	9	[illegible]

Chr11	5619176	A	G	Chr11_56191	A_G	SNP	13610.63	15	6	9	18	11	17
Chr11	5619176	A	G	Chr11_56191	A_G	SNP	13610.63	15	6	9	18	11	17
Chr11	5619180	TG	T	Chr11_56191	TG_T	INDEL	14051.59	15	8	9	18	12	21
Chr11	5619180	TG	T	Chr11_56191	TG_T	INDEL	14051.59	15	8	9	18	12	21
Chr11	5619180	TG	T	Chr11_56191	TG_T	INDEL	14051.59	15	8	9	18	12	21
Chr11	5619182	AGC	A	Chr11_56191	AGC_A	INDEL	14276.59	15	8	9	18	12	21
Chr11	5619182	AGC	A	Chr11_56191	AGC_A	INDEL	14276.59	15	8	9	18	12	21
Chr11	5619182	AGC	A	Chr11_56191	AGC_A	INDEL	14276.59	15	8	9	18	12	21
Chr11	5619189	G	GACATAGAA	Chr11_56191	G_GACATAG	INDEL	15806.59	15	8	9	18	12	22
Chr11	5619189	G	GACATAGAA	Chr11_56191	G_GACATAG	INDEL	15806.59	15	8	9	18	12	22
Chr11	5619189	G	GACATAGAA	Chr11_56191	G_GACATAG	INDEL	15806.59	15	8	9	18	12	22
Chr11	5619194	T	A	Chr11_56191	T_A	SNP	16130.63	19	10	10	19	12	22
Chr11	5619194	T	A	Chr11_56191	T_A	SNP	16130.63	19	10	10	19	12	22
Chr11	5619194	T	A	Chr11_56191	T_A	SNP	16130.63	19	10	10	19	12	22
Chr11	5619196	C	G	Chr11_56191	C_G	SNP	16355.63	19	10	10	19	12	22
Chr11	5619196	C	G	Chr11_56191	C_G	SNP	16355.63	19	10	10	19	12	22
Chr11	5619196	C	G	Chr11_56191	C_G	SNP	16355.63	19	10	10	19	12	22
Chr11	5619197	T	A	Chr11_56191	T_A	SNP	16445.63	19	10	10	19	12	22
Chr11	5619197	T	A	Chr11_56191	T_A	SNP	16445.63	19	10	10	19	12	22
Chr11	5619197	T	A	Chr11_56191	T_A	SNP	16445.63	19	10	10	19	12	22
Chr11	5619199	ATTTTTTTTC	A	Chr11_56191	ATTTTTTTTC_	INDEL	16736.59	19	10	11	20	12	22
Chr11	5619199	ATTTTTTTTC	A	Chr11_56191	ATTTTTTTTC_	INDEL	16736.59	19	10	11	20	12	22
Chr11	5619199	ATTTTTTTTC	A	Chr11_56191	ATTTTTTTTC_	INDEL	16736.59	19	10	11	20	12	22
Chr11	5619211	T	G	Chr11_56192	T_G	SNP	16727.63	19	9	11	20	12	22
[illegible]													

Chr11	11893700	GC	G	Chr11_11893	GC_G	INDEL	19921.59	34	16	19	27	15	19	34	26	15	20	18	11	8	5	[illegible]
Chr11	11893700	GC	G	Chr11_11893	GC_G	INDEL	19921.59	34	16	19	27	15	19	34	26	15	20	18	11	8	5	[illegible]
Chr11	11893700	GC	G	Chr11_11893	GC_G	INDEL	19921.59	34	16	19	27	15	19	34	26	15	20	18	11	8	5	[illegible]
Chr11	11893700	GC	G	Chr11_11893	GC_G	INDEL	19921.59	34	16	19	27	15	19	34	26	15	20	18	11	8	5	[illegible]
Chr11	11893705	A	G	Chr11_11893	A_G	SNP	20264.63	32	15	19	28	15	19	35	26	17	20	18	11	9	5	[illegible]
Chr11	11893705	A	G	Chr11_11893	A_G	SNP	20264.63	32	15	19	28	15	19	35	26	17	20	18	11	9	5	[illegible]
Chr11	11893705	A	G	Chr11_11893	A_G	SNP	20264.63	32	15	19	28	15	19	35	26	17	20	18	11	9	5	[illegible]
Chr11	11893705	A	G	Chr11_11893	A_G	SNP	20264.63	32	15	19	28	15	19	35	26	17	20	18	11	9	5	[illegible]
Chr11	11893705	A	G	Chr11_11893	A_G	SNP	20264.63	32	15	19	28	15	19	35	26	17	20	18	11	9	5	[illegible]
Chr11	11893706	AAG	A	Chr11_11893	AAG_A	INDEL	20216.59	32	16	19	28	15	20	34	26	17	20	18	11	9	5	[illegible]
Chr11	11893706	AAG	A	Chr11_11893	AAG_A	INDEL	20216.59	32	16	19	28	15	20	34	26	17	20	18	11	9	5	[illegible]
Chr11	11893706	AAG	A	Chr11_11893	AAG_A	INDEL	20216.59	32	16	19	28	15	20	34	26	17	20	18	11	9	5	[illegible]
Chr11	11893706	AAG	A	Chr11_11893	AAG_A	INDEL	20216.59	32	16	19	28	15	20	34	26	17	20	18	11	9	5	[illegible]
Chr11	11893706	AAG	A	Chr11_11893	AAG_A	INDEL	20216.59	32	16	19	28	15	20	34	26	17	20	18	11	9	5	[illegible]
Chr11	11979066	C	CA	Chr11_11979	C_CA	INDEL	14950.59	31	12	20	28	20	21	25	23	36	20	15	22	9	10	[illegible]
Chr11	12091585	AAT	A	Chr11_12091	AAT_A	INDEL	8645.59	14	11	13	18	16	18	19	10	12	16	7	11	7	6	[illegible]
Chr11	12091585	AAT	A	Chr11_12091	AAT_A	INDEL	8645.59	14	11	13	18	16	18	19	10	12	16	7	11	7	6	[illegible]
Chr11	12091585	AAT	A	Chr11_12091	AAT_A	INDEL	8645.59	14	11	13	18	16	18	19	10	12	16	7	11	7	6	[illegible]
Chr11	12091585	AAT	A	Chr11_12091	AAT_A	INDEL	8645.59	14	11	13	18	16	18	19	10	12	16	7	11	7	6	[illegible]
Chr11	12091585	AAT	A	Chr11_12091	AAT_A	INDEL	8645.59	14	11	13	18	16	18	19	10	12	16	7	11	7	6	[illegible]
Chr11	12091585	AAT	A	Chr11_12091	AAT_A	INDEL	8645.59	14	11	13	18	16	18	19	10	12	16	7	11	7	6	[illegible]
Chr11	12091585	AAT	A	Chr11_12091	AAT_A	INDEL	8645.59	14	11	13	18	16	18	19	10	12	16	7	11	7	6	[illegible]
Chr11	12091585	AAT	A	Chr11_12091	AAT_A	INDEL	8645.59	14	11	13	18	16	18	19	10	12	16	7	11	7	6	[illegible]
Chr11	12138275	G	A	Chr11_12138	G_A	SNP	22542.63	35	17	14	25	26	33	26	20	26	23	20	21	13	16	[illegible]
Chr11	12138276	T	A	Chr11_12138	T_A	SNP	22542.63	33	17	14	25	26	33	26	20	27	23	20	21	13	16	[illegible]
Chr11	12140860	TA	T	Chr11_12140	TA_T	INDEL	20446.59	28	8	23	31	12	29	37	16	31	24	28	15	10	11	[illegible]
Chr11	12140875	G	T	Chr11_12140	G_T	SNP	20342.63	28	10	20	32	11	28	34	17	29	21	33	12	6	10	[illegible]
Chr11	12140919	G	A	Chr11_12140	G_A	SNP	21145.63	30	10	16	28	16	24	36	22	30	19	26	15	8	13	[illegible]
Chr11	12140920	G	A	Chr11_12140	G_A	SNP	21245.63	30	11	16	29	16	24	35	22	30	18	26	15	8	13	[illegible]
Chr11	12140925	A	C	Chr11_12140	A_C	SNP	20903.63	31	13	13	28	17	22	35	21	29	17	25	17	9	13	[illegible]
Chr11	12157701	T	TTA	Chr11_12157	T_TTA	INDEL	2459.24	0	0	1	14	13	26	15	15	0	0	0	1	0	0	[illegible]
Chr11	12157701	T	TTA	Chr11_12157	T_TTA	INDEL	2459.24	0	0	1	14	13	26	15	15	0	0	0	1	0	0	[illegible]
Chr11	12157701	T	TTA	Chr11_12157	T_TTA	INDEL	2459.24	0	0	1	14	13	26	15	15	0	0	0	1	0	0	[illegible]
Chr11	12157701	T	TTA	Chr11_12157	T_TTA	INDEL	2459.24	0	0	1	14	13	26	15	15	0	0	0	1	0	0	[illegible]
Chr11	12157701	T	TTA	Chr11_12157	T_TTA	INDEL	2459.24	0	0	1	14	13	26	15	15	0	0	0	1	0	0	[illegible]
Chr11	12157701	T	TTA	Chr11_12157	T_TTA	INDEL	2459.24	0	0	1	14	13	26	15	15	0	0	0	1	0	0	[illegible]
Chr11	12169046	TG	T	Chr11_12169	TG_T	INDEL	19321.59	22	14	21	37	22	29	35	22	30	21	19	14	16	19	[illegible]
Chr11	12169046	TG	T	Chr11_12169	TG_T	INDEL	19321.59	22	14	21	37	22	29	35	22	30	21	19	14	16	19	[illegible]
Chr11	12169046	TG	T	Chr11_12169	TG_T	INDEL	19321.59	22	14	21	37	22	29	35	22	30	21	19	14	16	19	[illegible]
Chr11	12169046	TG	T	Chr11_12169	TG_T	INDEL	19321.59	22	14	21	37	22	29	35	22	30	21	19	14	16	19	[illegible]
Chr11	12172235	GGACGTGCG	G	Chr11_12172	GGACGTGCG	INDEL	148982.59	255	116	129	190	177	214	276	216	164	190	90	114	80	87	[illegible]
Chr11	12172235	GGACGTGCG	G	Chr11_12172	GGACGTGCG	INDEL	148982.59	255	116	129	190	177	214	276	216	164	190	90	114	80	87	[illegible]
Chr11	12172235	GGACGTGCG	G	Chr11_12172	GGACGTGCG	INDEL	148982.59	255	116	129	190	177	214	276	216	164	190	90	114	80	87	[illegible]
Chr11	12172235	GGACGTGCG	G	Chr11_12172	GGACGTGCG	INDEL	148982.59	255	116	129	190	177	214	276	216	164	190	90	114	80	87	[illegible]
Chr11	12238183	CAT	C	Chr11_12238	CAT_C	INDEL	702.35	17	7	8	0	0	1	0	0	0	0	0	1	0	0	[illegible]
Chr11	12724452	G	GT	Chr11_12724	G_GT	INDEL	16718.59	29	25	19	24	32	32	20	24	32	22	20	19	18	13	[illegible]
Chr11	12917419	C	CTATATA	Chr11_12917	C_CTATATA	INDEL	13233.59	23	6	6	0	0	0	1	1	14	12	5	5	5	8	[illegible]
Chr11	12917419	C	CTATATA	Chr11_12917	C_CTATATA	INDEL	13233.59	23	6	6	0	0	0	1	1	14	12	5	5	5	8	[illegible]
Chr11	12917419	C	CTATATA	Chr11_12917	C_CTATATA	INDEL	13233.59	23	6	6	0	0	0	1	1	14	12	5	5	5	8	[illegible]
Chr11	12917419	C	CTATATATA	Chr11_12917	C_CTATATAT	INDEL	13233.59	0	0	0	14	9	12	18	19	0	1	0	0	1	0	[illegible]
Chr11	12917419	C	CTATATATA	Chr11_12917	C_CTATATAT	INDEL	13233.59	0	0	0	14	9	12	18	19	0	1	0	0	1	0	[illegible]
Chr11	12917419	C	CTATATATA	Chr11_12917	C_CTATATAT	INDEL	13233.59	0	0	0	14	9	12	18	19	0	1	0	0	1	0	[illegible]
Chr11	12928268	G	GT	Chr11_12928	G_GT	INDEL	16314.59	26	15	14	25	18	34	31	26	25	26	20	15	13	5	[illegible]
Chr11	12989163	G	GGCTTTAGG	Chr11_12989	G_GGCTTTA	INDEL	21623.59	22	8	10	22	13	22	25	18	14	13	14	5	6	13	[illegible]
Chr11	13038058	G	C	Chr11_13038	G_C	SNP	16955.63	21	13	21	29	17	29	18	23	19	26	18	19	6	4	[illegible]
Chr11	13061002	G	A	Chr11_13061	G_A	SNP	15866.63	32	13	17	24	32	17	42	19	19	25	23	21	4	12	[illegible]
Chr11	13152764	T	C	Chr11_13152	T_C	SNP	3732.29	0	0	0	16	12	20	22	19	0	0	0	0	0	0	[illegible]
Chr11	13253495	G	C	Chr11_13253	G_C	SNP	21884.63	25	22	26	26	22	24	34	29	24	25	13	25	20	14	[illegible]
Chr11	13253495	G	C	Chr11_13253	G_C	SNP	21884.63	25	22	26	26	22	24	34	29	24	25	13	25	20	14	[illegible]
Chr11	13253495	G	C	Chr11_13253	G_C	SNP	21884.63	25	22	26	26	22	24	34	29	24	25	13	25	20	14	[illegible]
Chr11	13420881	AAT	A	Chr11_13420	AAT_A	INDEL	1210.35	20	12	10	1	0	1	1	0	0	0	0	0	0	0	[illegible]
Chr11	13420881	AAT	A	Chr11_13420	AAT_A	INDEL	1210.35	20	12	10	1	0	1	1	0	0	0	0	0	0	0	[illegible]
Chr11	13447175	C	A	Chr11_13447	C_A	SNP	4582.29	0	0	0	9	14	26	35	18	0	0	0	0	0	0	[illegible]
Chr11	13613556	A	C	Chr11_13613	A_C	SNP	10954.63	14	12	6	10	13	15	17	11	12	7	6	23	6	10	[illegible]
Chr11	13613556	A	C	Chr11_13613	A_C	SNP	10954.63	14	12	6	10	13	15	17	11	12	7	6	23	6	10	[illegible]
Chr11	13613556	A	C	Chr11_13613	A_C	SNP	10954.63	14	12	6	10	13	15	17	11	12	7	6	23	6	10	[illegible]
Chr11	13613556	A	C	Chr11_13613	A_C	SNP	10954.63	14	12	6	10	13	15	17	11	12	7	6	23	6	10	[illegible]
Chr11	13613556	A	C	Chr11_13613	A_C	SNP	10954.63	14	12	6	10	13	15	17	11	12	7	6	23	6	10	[illegible]
Chr11	13613556	A	C	Chr11_13613	A_C	SNP	10954.63	14	12	6	10	13	15	17	11	12	7	6	23	6	10	[illegible]
Chr11	13613556	A	C	Chr11_13613	A_C	SNP	10954.63	14	12	6	10	13	15	17	11	12	7	6	23	6	10	[illegible]
Chr11	13613559	A	C	Chr11_13613	A_C	SNP	9992.63	15	9	5	9	12	13	15	7	8	5	4	19	4	9	[illegible]
Chr11	13613559	A	C	Chr11_13613	A_C	SNP	9992.63	15	9	5	9	12	13	15	7	8	5	4	19	4	9	[illegible]
Chr11	13613559	A	C	Chr11_13613	A_C	SNP	9992.63	15	9	5	9	12	13	15	7	8	5	4	19	4	9	[illegible]
Chr11	13613559	A	C	Chr11_13613	A_C	SNP	9992.63	15	9	5	9	12	13	15	7	8	5	4	19	4	9	[illegible]
Chr11	13613559	A	C	Chr11_13613	A_C	SNP	9992.63	15	9	5	9	12	13	15	7	8	5	4	19	4	9	[illegible]
Chr11	13613559	A	C	Chr11_13613	A_C	SNP	9992.63	15	9	5	9	12	13	15	7	8	5	4	19	4	9	[illegible]
Chr11	13613559	A	C	Chr11_13613	A_C	SNP	9992.63	15	9	5	9	12	13	15	7	8	5	4	19	4	9	[illegible]
Chr11	14024084	T	TC	Chr11_14024	T_TC	INDEL	13623.59	24	15	11	23	21	23	11	27	14	18	14	13	9	10	[illegible]
Chr11	14024084	T	TC	Chr11_14024	T_TC	INDEL	13623.59	24	15	11	23	21	23	11	27	14	18	14	13	9	10	[illegible]
Chr11	14024084	T	TC	Chr11_14024	T_TC	INDEL	13623.59	24	15	11	23	21	23	11	27	14	18	14	13	9	10	[illegible]
Chr11	14024084	T	TC	Chr11_14024	T_TC	INDEL	13623.59	24	15	11	23	21	23	11	27	14	18	14	13	9	10	[illegible]
Chr11	14024084	T	TC	Chr11_14024	T_TC	INDEL	13623.59	24	15	11	23	21	23	11	27	14	18	14	13	9	10	[illegible]
Chr11	14024084	T	TC	Chr11_14024	T_TC	INDEL	13623.59	24	15	11	23	21	23	11	27	14	18	14	13	9	10	[illegible]
Chr11	14024084	T	TC	Chr11_14024	T_TC	INDEL	13623.59	24	15	11	23	21	23	11	27	14	18	14	13	9	10	[illegible]
Chr11	14045856	A	AAT	Chr11_14045	A_AAT	INDEL	1134.35	15	13	12	0	0	0	2	2	0	1	0	1	1	0	[illegible]
Chr11	14045856	A	AAT	Chr11_14045	A_AAT	INDEL	1134.35	15	13	12	0	0	0	2	2	0	1	0	1	1	0	[illegible]
Chr11	14045856	A	AAT	Chr11_14045	A_AAT	INDEL	1134.35	15	13	12	0	0	0	2	2	0	1	0	1	1	0	[illegible]
Chr11	14045856	A	AAT	Chr11_14045	A_AAT	INDEL	1134.35	15	13	12	0	0	0	2	2	0	1	0	1	1	0	[illegible]
Chr11	14045856	A	AAT	Chr11_14045	A_AAT	INDEL	1134.35	15	13	12	0	0	0	2	2	0	1	0	1	1	0	[illegible]
Chr11	14136631	G	GA	Chr11_14136	G_GA	INDEL	14102.59	29	18	12	17	17	31	25	21	12	22	8	14	5	10	[illegible]
Chr11	14136631	G	GA	Chr11_14136	G_GA	INDEL	14102.59	29	18	12	17	17	31	25	21	12	22	8	14	5	10	[illegible]
Chr11	14136631	G	GA	Chr11_14136	G_GA	INDEL	14102.59	29	18	12	17	17	31	25	21	12	22	8	14	5	10	[illegible]
Chr11	14137679	C	CTA	Chr11_14137	C_CTA	INDEL	2939.24	0	0	2	14	11	17	26	10	0	1	0	0	0	0	[illegible]
Chr11	14137679	C	CTA	Chr11_14137	C_CTA	INDEL	2939.24	0	0	2	14	11	17	26	10	0	1	0	0	0	0	[illegible]
Chr11	14137679	C	CTA	Chr11_14137	C_CTA	INDEL	2939.24	0	0	2	14	11	17	26	10	0	1	0	0	0	0	[illegible]
Chr11	14308803	G	GA	Chr11_14308	G_GA	INDEL	16193.59	24	11	19	21	26	25	27	28	29	15	10	14	12	9	[illegible]
Chr11	14308803	G	GA	Chr11_14308	G_GA	INDEL	16193.59	24	11	19	21	26	25	27	28	29	15	10	14	12	9	[illegible]
Chr11	14308803	G	GA	Chr11_14308	G_GA	INDEL	16193.59	24	11	19	21	26	25	27	28	29	15	10	14	12	9	[illegible]
Chr11	14308803	G	GA	Chr11_14308	G_GA	INDEL	16193.59	24	11	19	21	26	25	27	28	29	15	10	14	12	9	[illegible]
Chr11	14308803	G	GA	Chr11_14308	G_GA	INDEL	16193.59	24	11	19	21	26	25	27	28	29	15	10	14	12	9	[illegible]
Chr11	14432293	CA	C	Chr11_14432	CA_C	INDEL	16438.59	29	15	12	28	21	20	13	30	27	16	18	8	8	15	[illegible]
Chr11	14530345	T	TGAAC	Chr11_14530	T_TGAAC	INDEL	19420.59	23	6	18	27	9	18	21	26	20	27	9	16	10	9	[illegible]
Chr11	14530345	T	TGAAC	Chr11_14530	T_TGAAC	INDEL	19420.59	23	6	18	27	9	18	21	26	20	27	9	16	10	9	[illegible]
Chr11	14530345	T	TGAAC	Chr11_14530	T_TGAAC	INDEL	19420.59	23	6	18	27	9	18	21	26	20	27	9	16	10	9	[illegible]
Chr11	14530345	T	TGAAC	Chr11_14530	T_TGAAC	INDEL	19420.59	23	6	18	27	9	18	21	26	20	27	9	16	10	9	[illegible]
Chr11	14530345	T	TGAAC	Chr11_14530	T_TGAAC	INDEL	19420.59	23	6	18	27	9	18	21	26	20	27	9	16	10	9	[illegible]
Chr11	14530345	T	TGAAC	Chr11_14530	T_TGAAC	INDEL	19420.59	23	6	18	27	9	18	21	26	20	27	9	16	10	9	[illegible]
Chr11	14530347	TTTCC	T	Chr11_14530	TTTCC_T	INDEL	19420.59	24	7	19	27	9	16	22	27	22	28	10	17	9	9	[illegible]
Chr11	14530347	TTTCC	T	Chr11_14530	TTTCC_T	INDEL	19420.59	24	7	19	27	9	16	22	27	22	28	10	17	9	9	[illegible]
Chr11	14530347	TTTCC	T	Chr11_14530	TTTCC_T	INDEL	19420.59	24	7	19	27	9	16	22	27	22	28	10	17	9	9	[illegible]
Chr11	14530347	TTTCC	T	Chr11_14530	TTTCC_T	INDEL	19420.59	24	7	19	27	9	16	22	27	22	28	10	17	9	9	[illegible]
Chr11	14530347	TTTCC	T	Chr11_14530	TTTCC_T	INDEL	19420.59	24	7	19	27	9	16	22	27	22	28	10	17	9	9	[illegible]
Chr11	14530347	TTTCC	T	Chr11_14530	TTTCC_T	INDEL	19420.59	24	7	19	27	9	16	22	27	22	28	10	17	9	9	[illegible]
Chr11	14646211	G	C	Chr11_14646	G_C	SNP	16300.63	26	10	14	25	15	21	31	25	14	24	17	12	6	10	[illegible]
Chr11	14646211	G	C	Chr11_14646	G_C	SNP	16300.63	26	10	14	25	15	21	31	25	14	24	17	12	6	10	[illegible]
Chr11	14646211	G	C	Chr11_14646	G_C	SNP	16300.63	26	10	14	25	15	21	31	25	14	24	17	12	6	10	[illegible]
Chr11	14894092	AAT	A	Chr11_14894	AAT_A	INDEL	5447.11	0	0	2	11	15	17	20	13	0	0	0	0	0	0	[illegible]
Chr11	14894092	AAT	A	Chr11_14894	AAT_A	INDEL	5447.11	0	0	2	11	15	17	20	13	0	0	0	0	0	0	[illegible]
Chr11	14894092	AAT	A	Chr11_14894	AAT_A	INDEL	5447.11	0	0	2	11	15	17	20	13	0	0	0	0	0	0	[illegible]
Chr11	14970443	C	A	Chr11_14970	C_A	SNP	7901.63	9	5	8	8	13	11	12	10	6	9	4	10	4	6	[illegible]
Chr11	14970443	C	A	Chr11_14970	C_A	SNP	7901.63	9	5	8	8	13	11	12	10	6	9	4	10	4	6	[illegible]
Chr11	14970443	C	A	Chr11_14970	C_A	SNP	7901.63	9	5	8	8	13	11	12	10	6	9	4	10	4	6	[illegible]
Chr11	14970443	C	A	Chr11_14970	C_A	SNP	7901.63	9	5	8	8	13	11	12	10	6	9	4	10	4	6	[illegible]
Chr11	14970443	C	A	Chr11_14970	C_A	SNP	7901.63	9	5	8	8	13	11	12	10	6	9	4	10	4	6	[illegible]
Chr11	14970443	C	A	Chr11_14970	C_A	SNP	7901.63	9	5	8	8	13	11	12	10	6	9	4	10	4	6	[illegible]
Chr11	14970445	C	A	Chr11_14970	C_A	SNP	7945.63	9	5	7	9	13	11	11	10	6	9	4	10	4	5	[illegible]
Chr11	14970445	C	A	Chr11_14970	C_A	SNP	7945.63	9	5	7	9	13	11	11	10	6	9	4	10	4	5	[illegible]
Chr11	14970445	C	A	Chr11_14970	C_A	SNP	7945.63	9	5	7	9	13	11	11	10	6	9	4	10	4	5	[illegible]
Chr11	14970445	C	A	Chr11_14970	C_A	SNP	7945.63	9	5	7	9	13	11	11	10	6	9	4	10	4	5	[illegible]
Chr11	14970445	C	A	Chr11_14970	C_A	SNP	7945.63	9	5	7	9	13	11	11	10	6	9	4	10	4	5	[illegible]
Chr11	14970445	C	A	Chr11_14970	C_A	SNP	7945.63	9	5	7	9	13	11	11	10	6	9	4	10	4	5	[illegible]
Chr11	14970447	C	A	Chr11_14970	C_A	SNP	7764.63	9	5	7	10	13	11	11	9	7	9	4	10	4	5	[illegible]
Chr11	14970447	C	A	Chr11_14970	C_A	SNP	7764.63	9	5	7	10	13	11	11	9	7	9	4	10	4	5	[illegible]
Chr11	14970447	C	A	Chr11_14970	C_A	SNP	7764.63	9	5	7	10	13	11	11	9	7	9	4	10	4	5	[illegible]
Chr11	14970447	C	A	Chr11_14970	C_A	SNP	7764.63	9	5	7	10	13	11	11	9	7	9	4	10	4	5	[illegible]
Chr11	14970447	C	A	Chr11_14970	C_A	SNP	7764.63	9	5	7	10	13	11	11	9	7	9	4	10	4	5	[illegible]
Chr11	14970447	C	A	Chr11_14970	C_A	SNP	7764.63	9	5	7	10	13	11	11	9	7	9	4	10	4	5	[illegible]
Chr11	14973943	C	CT	Chr11_14973	C_CT	INDEL	17570.59	26	16	13	28	16	20	35	26	32	25	9	18	7	6	[illegible]
Chr11	14973943	C	CT	Chr11_14973	C_CT	INDEL	17570.59	26	16	13	28	16	20	35	26	32	25	9	18	7	6	[illegible]
Chr11	14973943	C	CT	Chr11_14973	C_CT	INDEL	17570.59	26	16	13	28	16	20	35	26	32	25	9	18	7	6	[illegible]
Chr11	14973943	C	CT	Chr11_14973	C_CT	INDEL	17570.59	26	16	13	28	16	20	35	26	32	25	9	18	7	6	[illegible]
Chr11	14973943	C	CT	Chr11_14973	C_CT	INDEL	17570.59	26	16	13	28	16	20	35	26	32	25	9	18	7	6	[illegible]
Chr11	14994965	G	T	Chr11_14994	G_T	SNP	18395.63	31	10	18	33	29	29	26	27	33	29	11	17	16	10	[illegible]
Chr11	14994965	G	T	Chr11_14994	G_T	SNP	18395.63	31	10	18	33	29	29	26	27	33	29	11	17	16	10	[illegible]
Chr11	14994965	G	T	Chr11_14994	G_T	SNP	18395.63	31	10	18	33	29	29	26	27	33	29	11	17	16	10	[illegible]
Chr11	14994965	G	T	Chr11_14994	G_T	SNP	18395.63	31	10	18	33	29	29	26	27	33	29	11	17	16	10	[illegible]
Chr11	15254357	A	AT	Chr11_15254	A_AT	INDEL	17617.59	31	10	22	24	25	34	24	34	20	39	10	19	12	11	[illegible]
Chr11	15865163	G	GAT	Chr11_15865	G_GAT	INDEL	2725.24	0	1	0	19	11	14	22	16	0	0	0	0	0	1	[illegible]
Chr11	15865163	G	GAT	Chr11_15865	G_GAT	INDEL	2725.24	0	1	0	19	11	14	22	16	0	0	0	0	0	1	[illegible]
Chr11	15865163	G	GAT	Chr11_15865	G_GAT	INDEL	2725.24	0	1	0	19	11	14	22	16	0	0	0	0	0	1	[illegible]
Chr11	15865163	G	GAT	Chr11_15865	G_GAT	INDEL	2725.24	0	1	0	19	11	14	22	16	0	0	0	0	0	1	[illegible]
Chr11	15865163	G	GAT	Chr11_15865	G_GAT	INDEL	2725.24	0	1	0	19	11	14	22	16	0	0	0	0	0	1	[illegible]
Chr11	15895516	A	AT	Chr11_15895	A_AT	INDEL	16415.59	20	13	20	32	20	14	15	21	24	17	21	12	10	13	[illegible]
Chr11	15898182	C	A	Chr11_15898	C_A	SNP	20278.63	21	11	23	39	26	33	28	18	19	30	16	20	11	16	[illegible]
Chr11	15898420	C	A	Chr11_15898	C_A	SNP	18986.63	32	16	20	22	21	32	22	21	23	28	19	26	8	14	[illegible]
Chr11	15904093	A	C	Chr11_15904	A_C	SNP	19584.63	22	16	13	23	23	30	22	22	20	19	14	32	11	12	[illegible]
Chr11	15904172	TG	T	Chr11_15904	TG_T	INDEL	17082.59	22	15	12	27	24	25	19	22	26	17	19	17	14	7	[illegible]
Chr11	15926130	A	G	Chr11_15926	A_G	SNP	18211.63	28	10	13	29	17	24	24	27	18	25	19	12	15	9	[illegible]
Chr11	16186593	A	C	Chr11_16186	A_C	SNP	2001.39	26	12	9	0	0	0	0	0	0	0	0	0	0	0	[illegible]
Chr11	16186593	A	C	Chr11_16186	A_C	SNP	2001.39	26	12	9	0	0	0	0	0	0	0	0	0	0	0	[illegible]
Chr11	16186593	A	C	Chr11_16186	A_C	SNP	2001.39	26	12	9	0	0	0	0	0	0	0	0	0	0	0	[illegible]
Chr11	16186593	A	C	Chr11_16186	A_C	SNP	2001.39	26	12	9	0	0	0	0	0	0	0	0	0	0	0	[illegible]
Chr11	16203774	G	T	Chr11_16203	G_T	SNP	16235.63	25	12	13	22	19	26	21	18	23	8	16	13	10	8	[illegible]
Chr11	16203801	G	A	Chr11_16203	G_A	SNP	18951.63	26	13	15	24	18	31	25	16	27	9	16	14	11	12	[illegible]
Chr11	16203812	AG	A	Chr11_16203	AG_A	INDEL	19563.59	27	13	14	29	20	33	23	17	27	9	18	13	10	12	[illegible]
Chr11	16203815	AG	A	Chr11_16203	AG_A	INDEL	18959.59	27	13	14	29	18	32	23	16	27	9	18	13	10	12	[illegible]
Chr11	16211871	C	G	Chr11_16211	C_G	SNP	19537.63	26	11	14	26	12	30	22	21	21	27	20	16	4	11	[illegible]
Chr11	16211872	G	T	Chr11_16211	G_T	SNP	19537.63	26	11	14	26	12	30	23	21	22	27	19	16	4	12	[illegible]
Chr11	16215794	TA	T	Chr11_16215	TA_T	INDEL	15602.59	33	11	16	29	18	28	27	28	17	36	14	20	14	12	[illegible]
Chr11	16354356	G	GCCTTCTAAC	Chr11_16354	G_GCCTTCTA	INDEL	23994.59	25	15	15	33	25	30	30	27	19	28	17	10	8	13	[illegible]
Chr11	16354356	G	GCCTTCTAAC	Chr11_16354	G_GCCTTCTA	INDEL	23994.59	25	15	15	33	25	30	30	27	19	28	17	10	8	13	[illegible]
Chr11	16354356	G	GCCTTCTAAC	Chr11_16354	G_GCCTTCTA	INDEL	23994.59	25	15	15	33	25	30	30	27	19	28	17	10	8	13	[illegible]
Chr11	16354356	G	GCCTTCTAAC	Chr11_16354	G_GCCTTCTA	INDEL	23994.59	25	15	15	33	25	30	30	27	19	28	17	10	8	13	[illegible]
Chr11	16354451	AATTGGCAT	A	Chr11_16354	AATTGGCAT	INDEL	18827.59	27	8	23	25	22	26	26	17	19	20	10	4	6	12	[illegible]
Chr11	16354451	AATTGGCAT	A	Chr11_16354	AATTGGCAT	INDEL	18827.59	27	8	23	25	22	26	26	17	19	20	10	4	6	12	[illegible]
Chr11	16354451	AATTGGCAT	A	Chr11_16354	AATTGGCAT	INDEL	18827.59	27	8	23	25	22	26	26	17	19	20	10	4	6	12	[illegible]
Chr11	16354451	AATTGGCAT	A	Chr11_16354	AATTGGCAT	INDEL	18827.59	27	8	23	25	22	26	26	17	19	20	10	4	6	12	[illegible]
Chr11	16354546	G	GACTTAATT	Chr11_16354	G_GACTTAA	INDEL	24589.59	33	5	20	26	23	33	30	20	32	19	14	4	7	9	[illegible]
Chr11	16354546	G	GACTTAATT	Chr11_16354	G_GACTTAA	INDEL	24589.59	33	5	20	26	23	33	30	20	32	19	14	4	7	9	[illegible]
Chr11	16354546	G	GACTTAATT	Chr11_16354	G_GACTTAA	INDEL	24589.59	33	5	20	26	23	33	30	20	32	19	14	4	7	9	[illegible]
Chr11	16354546	G	GACTTAATT	Chr11_16354	G_GACTTAA	INDEL	24589.59	33	5	20	26	23	33	30	20	32	19	14	4	7	9	[illegible]
Chr11	16407743	CTA	C	Chr11_16407	CTA_C	INDEL	13969.59	26	11	18	28	10	21	33	25	23	21	15	10	6	9	[illegible]
Chr11	16407743	CTA	C	Chr11_16407	CTA_C	INDEL	13969.59	26	11	18	28	10	21	33	25	23	21	15	10	6	9	[illegible]
Chr11	16407743	CTA	C	Chr11_16407	CTA_C	INDEL	13969.59	26	11	18	28	10	21	33	25	23	21	15	10	6	9	[illegible]
Chr11	16407743	CTA	C	Chr11_16407	CTA_C	INDEL	13969.59	26	11	18	28	10	21	33	25	23	21	15	10	6	9	[illegible]
Chr11	16551324	C	G	Chr11_16551	C_G	SNP	5725.29	0	0	0	23	17	29	29	16	0	0	0	0	0	0	[illegible]
Chr11	16841941	G	GT	Chr11_16841	G_GT	INDEL	9577.59	23	13	8	12	11	19	18	13	10	5	10	5	7	4	[illegible]
Chr11	16841941	G	GT	Chr11_16841	G_GT	INDEL	9577.59	23	13	8	12	11	19	18	13	10	5	10	5	7	4	[illegible]
Chr11	16841941	G	GT	Chr11_16841	G_GT	INDEL	9577.59	23	13	8	12	11	19	18	13	10	5	10	5	7	4	[illegible]
Chr11	16841941	G	GT	Chr11_16841	G_GT	INDEL	9577.59	23	13	8	12	11	19	18	13	10	5	10	5	7	4	[illegible]
Chr11	16841941	G	GT	Chr11_16841	G_GT	INDEL	9577.59	23	13	8	12	11	19	18	13	10	5	10	5	7	4	[illegible]
Chr11	16841941	G	GT	Chr11_16841	G_GT	INDEL	9577.59	23	13	8	12	11	19	18	13	10	5	10	5	7	4	[illegible]
Chr11	16841941	G	GT	Chr11_16841	G_GT	INDEL	9577.59	23	13	8	12	11	19	18	13	10	5	10	5	7	4	[illegible]
Chr11	16841941	G	GT	Chr11_16841	G_GT	INDEL	9577.59	23	13	8	12	11	19	18	13	10	5	10	5	7	4	[illegible]
Chr11	16841941	G	GT	Chr11_16841	G_GT	INDEL	9577.59	23	13	8	12	11	19	18	13	10	5	10	5	7	4	[illegible]
Chr11	16841941	G	GT	Chr11_16841	G_GT	INDEL	9577.59	23	13	8	12	11	19	18	13	10	5	10	5	7	4	[illegible]
Chr11	16841941	G	GT	Chr11_16841	G_GT	INDEL	9577.59	23	13	8	12	11	19	18	13	10	5	10	5	7	4	[illegible]
Chr11	16841941	G	GT	Chr11_16841	G_GT	INDEL	9577.59	23	13	8	12	11	19	18	13	10	5	10	5	7	4	[illegible]
Chr11	16841941	G	GT	Chr11_16841	G_GT	INDEL	9577.59	23	13	8	12	11	19	18	13	10	5	10	5	7	4	[illegible]
Chr11	16894022	CATAT	C	Chr11_16894	CATAT_C	INDEL	14960.59	19	9	11	20	21	18	28	25	19	17	6	13	10	6	[illegible]
Chr11	16894022	CATAT	C	Chr11_16894	CATAT_C	INDEL	14960.59	19	9	11	20	21	18	28	25	19	17	6	13	10	6	[illegible]
Chr11	16894022	CATAT	C	Chr11_16894	CATAT_C	INDEL	14960.59	19	9	11	20	21	18	28	25	19	17	6	13	10	6	[illegible]

Chr11	16894022	CATAT	C	Chr11_16894CATAT_C	INDEL	14960.59	19	9	11	20	21	18	28	25	19	17	6	13	10	6
Chr11	17047152	AGTGC	A	Chr11_17047AGTGC_A	INDEL	18799.59	26	12	15	30	27	25	33	23	21	18	14	13	13	4
Chr11	17047152	AGTGC	A	Chr11_17047AGTGC_A	INDEL	18799.59	26	12	15	30	27	25	33	23	21	18	14	13	13	4
Chr11	17048058	A	AT	Chr11_17048A_AT	INDEL	21656.59	30	17	14	32	22	35	39	24	18	26	21	12	22	13
Chr11	17048058	A	AT	Chr11_17048A_AT	INDEL	21656.59	30	17	14	32	22	35	39	24	18	26	21	12	22	13
Chr11	17048076	A	AT	Chr11_17048A_AT	INDEL	22531.59	30	18	10	31	24	37	41	26	19	27	22	12	25	14
Chr11	17048076	A	AT	Chr11_17048A_AT	INDEL	22531.59	30	18	10	31	24	37	41	26	19	27	22	12	25	14
Chr11	17065797	G	A	Chr11_17065G_A	SNP	15426.63	30	12	16	16	11	24	24	19	31	24	19	16	9	11
Chr11	17309623	CA	C	Chr11_17309CA_C	INDEL	17460.59	31	18	16	24	26	26	38	27	18	28	20	12	14	5
Chr11	17309623	CA	C	Chr11_17309CA_C	INDEL	17460.59	31	18	16	24	26	26	38	27	18	28	20	12	14	5
Chr11	17309623	CA	C	Chr11_17309CA_C	INDEL	17460.59	31	18	16	24	26	26	38	27	18	28	20	12	14	5
Chr11	17309623	CA	C	Chr11_17309CA_C	INDEL	17460.59	31	18	16	24	26	26	38	27	18	28	20	12	14	5
Chr11	17494105	C	G	Chr11_17494C_G	SNP	7359.63	8	5	10	10	6	5	6	9	15	9	4	13	4	7
Chr11	17494105	C	G	Chr11_17494C_G	SNP	7359.63	8	5	10	10	6	5	6	9	15	9	4	13	4	7
Chr11	17494105	C	G	Chr11_17494C_G	SNP	7359.63	8	5	10	10	6	5	6	9	15	9	4	13	4	7
Chr12	35	G	T	Chr12_35_G_G_T	SNP	13278.63	19	6	9	19	11	10	15	17	18	20	10	15	5	10
Chr12	35	G	T	Chr12_35_G_G_T	SNP	13278.63	19	6	9	19	11	10	15	17	18	20	10	15	5	10
Chr12	35	G	T	Chr12_35_G_G_T	SNP	13278.63	19	6	9	19	11	10	15	17	18	20	10	15	5	10
Chr12	35	G	T	Chr12_35_G_G_T	SNP	13278.63	19	6	9	19	11	10	15	17	18	20	10	15	5	10
Chr12	35	G	T	Chr12_35_G_G_T	SNP	13278.63	19	6	9	19	11	10	15	17	18	20	10	15	5	10
Chr12	35	G	T	Chr12_35_G_G_T	SNP	13278.63	19	6	9	19	11	10	15	17	18	20	10	15	5	10
Chr12	35	G	T	Chr12_35_G_G_T	SNP	13278.63	19	6	9	19	11	10	15	17	18	20	10	15	5	10
Chr12	35	G	T	Chr12_35_G_G_T	SNP	13278.63	19	6	9	19	11	10	15	17	18	20	10	15	5	10
Chr12	35	G	T	Chr12_35_G_G_T	SNP	13278.63	19	6	9	19	11	10	15	17	18	20	10	15	5	10

[illegible]

Chr12	2652689	TA	T	Chr12_26526TA_T	INDEL	16076.59	26	14	9	16	11	20	22	20	12	18	6	20	12	9	26	14	9	16	11	20	22	20	12	18	6	20	12	9	1	1	1	1	1	1	1	1	1	1	1	1	1	1	1	1	1	0	15	15	0	7	5	3	3_prime_UTI	MODIFIER	Pp3c12_336(	Pp3c12_336(	transcript	PAC:3297471	protein_codi	22/22	c.*324delA	NA	NA	NA	NA	324
Chr12	2652689	TA	T	Chr12_26526TA_T	INDEL	16076.59	26	14	9	16	11	20	22	20	12	18	6	20	12	9	26	14	9	16	11	20	22	20	12	18	6	20	12	9	1	1	1	1	1	1	1	1	1	1	1	1	1	1	1	1	1	0	15	15	0	7	5	3	downstream_	MODIFIER	Pp3c12_339(	Pp3c12_339(	transcript	PAC:3297495	protein_codi	NA	c.*942delT	NA	NA	NA	NA	452
Chr12	2652689	TA	T	Chr12_26526TA_T	INDEL	16076.59	26	14	9	16	11	20	22	20	12	18	6	20	12	9	26	14	9	16	11	20	22	20	12	18	6	20	12	9	1	1	1	1	1	1	1	1	1	1	1	1	1	1	1	1	1	0	15	15	0	7	5	3	downstream_	MODIFIER	Pp3c12_336(	Pp3c12_336(	transcript	PAC:3297471	protein_codi	NA	c.*324delA	NA	NA	NA	NA	61
Chr12	2652689	TA	T	Chr12_26526TA_T	INDEL	16076.59	26	14	9	16	11	20	22	20	12	18	6	20	12	9	26	14	9	16	11	20	22	20	12	18	6	20	12	9	1	1	1	1	1	1	1	1	1	1	1	1	1	1	1	1	1	0	15	15	0	7	5	3	downstream_	MODIFIER	Pp3c12_339(	Pp3c12_339(	transcript	PAC:3297495	protein_codi	NA	c.*3812delT	NA	NA	NA	NA	3482
Chr12	2652691	CATT	C	Chr12_26526CATT_C	INDEL	15986.59	26	14	9	16	11	20	21	20	12	18	6	20	12	9	26	14	9	16	11	20	21	20	12	18	6	20	12	9	1	1	1	1	1	1	1	1	1	1	1	1	1	1	1	1	1	0	15	15	0	7	5	3	3_prime_UTI	MODIFIER	Pp3c12_336(	Pp3c12_336(	transcript	PAC:3297471	protein_codi	22/22	c.*326_*328	NA	NA	NA	NA	326
Chr12	2652691	CATT	C	Chr12_26526CATT_C	INDEL	15986.59	26	14	9	16	11	20	21	20	12	18	6	20	12	9	26	14	9	16	11	20	21	20	12	18	6	20	12	9	1	1	1	1	1	1	1	1	1	1	1	1	1	1	1	1	1	0	15	15	0	7	5	3	downstream_	MODIFIER	Pp3c12_339(	Pp3c12_339(	transcript	PAC:3297495	protein_codi	NA	c.*938_*940	NA	NA	NA	NA	450
Chr12	2652691	CATT	C	Chr12_26526CATT_C	INDEL	15986.59	26	14	9	16	11	20	21	20	12	18	6	20	12	9	26	14	9	16	11	20	21	20	12	18	6	20	12	9	1	1	1	1	1	1	1	1	1	1	1	1	1	1	1	1	1	0	15	15	0	7	5	3	downstream_	MODIFIER	Pp3c12_336(	Pp3c12_336(	transcript	PAC:3297471	protein_codi	NA	c.*326_*328	NA	NA	NA	NA	63
Chr12	2652691	CATT	C	Chr12_26526CATT_C	INDEL	15986.59	26	14	9	16	11	20	21	20	12	18	6	20	12	9	26	14	9	16	11	20	21	20	12	18	6	20	12	9	1	1	1	1	1	1	1	1	1	1	1	1	1	1	1	1	1	0	15	15	0	7	5	3	downstream_	MODIFIER	Pp3c12_339(	Pp3c12_339(	transcript	PAC:3297495	protein_codi	NA	c.*3808_*38	NA	NA	NA	NA	3480
Chr12	2652696	GGAGC	G	Chr12_26526GGAGC_G	INDEL	16166.59	25	13	9	16	10	20	21	20	13	18	6	20	11	10	25	13	9	16	10	20	21	20	13	18	6	20	11	10	1	1	1	1	1	1	1	1	1	1	1	1	1	1	1	1	1	0	15	15	0	7	5	3	3_prime_UTI	MODIFIER	Pp3c12_336(	Pp3c12_336(	transcript	PAC:3297471	protein_codi	22/22	c.*331_*334	NA	NA	NA	NA	331
Chr12	2652696	GGAGC	G	Chr12_26526GGAGC_G	INDEL	16166.59	25	13	9	16	10	20	21	20	13	18	6	20	11	10	25	13	9	16	10	20	21	20	13	18	6	20	11	10	1	1	1	1	1	1	1	1	1	1	1	1	1	1	1	1	1	0	15	15	0	7	5	3	downstream_	MODIFIER	Pp3c12_339(	Pp3c12_339(	transcript	PAC:3297495	protein_codi	NA	c.*932_*935	NA	NA	NA	NA	445
Chr12	2652696	GGAGC	G	Chr12_26526GGAGC_G	INDEL	16166.59	25	13	9	16	10	20	21	20	13	18	6	20	11	10	25	13	9	16	10	20	21	20	13	18	6	20	11	10	1	1	1	1	1	1	1	1	1	1	1	1	1	1	1	1	1	0	15	15	0	7	5	3	downstream_	MODIFIER	Pp3c12_336(	Pp3c12_336(	transcript	PAC:3297471	protein_codi	NA	c.*331_*334	NA	NA	NA	NA	68
Chr12	2652696	GGAGC	G	Chr12_26526GGAGC_G	INDEL	16166.59	25	13	9	16	10	20	21	20	13	18	6	20	11	10	25	13	9	16	10	20	21	20	13	18	6	20	11	10	1	1	1	1	1	1	1	1	1	1	1	1	1	1	1	1	1	0	15	15	0	7	5	3	downstream_	MODIFIER	Pp3c12_339(	Pp3c12_339(	transcript	PAC:3297495	protein_codi	NA	c.*3802_*38	NA	NA	NA	NA	3475
Chr12	2652702	A	T	Chr12_26527A_T	SNP	16310.63	25	13	9	17	10	21	21	20	13	18	7	20	11	10	25	13	9	17	10	21	21	20	13	18	7	20	11	10	1	1	1	1	1	1	1	1	1	1	1	1	1	1	1	1	1	0	15	15	0	7	5	3	3_prime_UTI	MODIFIER	Pp3c12_336(	Pp3c12_336(	transcript	PAC:3297471	protein_codi	22/22	c.*336A>T	NA	NA	NA	NA	336
Chr12	2652702	A	T	Chr12_26527A_T	SNP	16310.63	25	13	9	17	10	21	21	20	13	18	7	20	11	10	25	13	9	17	10	21	21	20	13	18	7	20	11	10	1	1	1	1	1	1	1	1	1	1	1	1	1	1	1	1	1	0	15	15	0	7	5	3	downstream_	MODIFIER	Pp3c12_339(	Pp3c12_339(	transcript	PAC:3297495	protein_codi	NA	c.*930T>A	NA	NA	NA	NA	440
Chr12	2652702	A	T	Chr12_26527A_T	SNP	16310.63	25	13	9	17	10	21	21	20	13	18	7	20	11	10	25	13	9	17	10	21	21	20	13	18	7	20	11	10	1	1	1	1	1	1	1	1	1	1	1	1	1	1	1	1	1	0	15	15	0	7	5	3	downstream_	MODIFIER	Pp3c12_336(	Pp3c12_336(	transcript	PAC:3297471	protein_codi	NA	c.*336A>T	NA	NA	NA	NA	73
Chr12	2652702	A	T	Chr12_26527A_T	SNP	16310.63	25	13	9	17	10	21	21	20	13	18	7	20	11	10	25	13	9	17	10	21	21	20	13	18	7	20	11	10	1	1	1	1	1	1	1	1	1	1	1	1	1	1	1	1	1	0	15	15	0	7	5	3	downstream_	MODIFIER	Pp3c12_339(	Pp3c12_339(	transcript	PAC:3297495	protein_codi	NA	c.*3800T>A	NA	NA	NA	NA	3470
Chr12	2652705	T	G	Chr12_26527T_G	SNP	16490.63	25	13	9	19	9	22	21	20	14	18	9	20	10	10	25	13	9	19	9	22	21	20	14	18	9	20	10	10	1	1	1	1	1	1	1	1	1	1	1	1	1	1	1	1	1	0	15	15	0	7	5	3	3_prime_UTI	MODIFIER	Pp3c12_336(	Pp3c12_336(	transcript	PAC:3297471	protein_codi	22/22	c.*339T>G	NA	NA	NA	NA	339
Chr12	2652705	T	G	Chr12_26527T_G	SNP	16490.63	25	13	9	19	9	22	21	20	14	18	9	20	10	10	25	13	9	19	9	22	21	20	14	18	9	20	10	10	1	1	1	1	1	1	1	1	1	1	1	1	1	1	1	1	1	0	15	15	0	7	5	3	downstream_	MODIFIER	Pp3c12_339(	Pp3c12_339(	transcript	PAC:3297495	protein_codi	NA	c.*927A>C	NA	NA	NA	NA	437
Chr12	2652705	T	G	Chr12_26527T_G	SNP	16490.63	25	13	9	19	9	22	21	20	14	18	9	20	10	10	25	13	9	19	9	22	21	20	14	18	9	20	10	10	1	1	1	1	1	1	1	1	1	1	1	1	1	1	1	1	1	0	15	15	0	7	5	3	downstream_	MODIFIER	Pp3c12_336(	Pp3c12_336(	transcript	PAC:3297471	protein_codi	NA	c.*339T>G	NA	NA	NA	NA	76
Chr12	2652705	T	G	Chr12_26527T_G	SNP	16490.63	25	13	9	19	9	22	21	20	14	18	9	20	10	10	25	13	9	19	9	22	21	20	14	18	9	20	10	10	1	1	1	1	1	1	1	1	1	1	1	1	1	1	1	1	1	0	15	15	0	7	5	3	downstream_	MODIFIER	Pp3c12_339(	Pp3c12_339(	transcript	PAC:3297495	protein_codi	NA	c.*3797A>C	NA	NA	NA	NA	3467
Chr12	2652708	C	G	Chr12_26527C_G	SNP	16967.63	24	13	10	18	9	22	22	19	15	18	10	21	10	10	24	13	10	18	9	22	22	19	15	18	10	21	10	10	1	1	1	1	1	1	1	1	1	1	1	1	1	1	1	1	1	0	15	15	0	7	5	3	3_prime_UTI	MODIFIER	Pp3c12_336(	Pp3c12_336(	transcript	PAC:3297471	protein_codi	22/22	c.*342C>G	NA	NA	NA	NA	342
Chr12	2652708	C	G	Chr12_26527C_G	SNP	16967.63	24	13	10	18	9	22	22	19	15	18	10	21	10	10	24	13	10	18	9	22	22	19	15	18	10	21	10	10	1	1	1	1	1	1	1	1	1	1	1	1	1	1	1	1	1	0	15	15	0	7	5	3	downstream_	MODIFIER	Pp3c12_339(	Pp3c12_339(	transcript	PAC:3297495	protein_codi	NA	c.*924G>C	NA	NA	NA	NA	434
Chr12	2652708	C	G	Chr12_26527C_G	SNP	16967.63	24	13	10	18	9	22	22	19	15	18	10	21	10	10	24	13	10	18	9	22	22	19	15	18	10	21	10	10	1	1	1	1	1	1	1	1	1	1	1	1	1	1	1	1	1	0	15	15	0	7	5	3	downstream_	MODIFIER	Pp3c12_336(	Pp3c12_336(	transcript	PAC:3297471	protein_codi	NA	c.*342C>G	NA	NA	NA	NA	79
Chr12	2652708	C	G	Chr12_26527C_G	SNP	16967.63	24	13	10	18	9	22	22	19	15	18	10	21	10	10	24	13	10	18	9	22	22	19	15	18	10	21	10	10	1	1	1	1	1	1	1	1	1	1	1	1	1	1	1	1	1	0	15	15	0	7	5	3	downstream_	MODIFIER	Pp3c12_339(	Pp3c12_339(	transcript	PAC:3297495	protein_codi	NA	c.*3794G>C	NA	NA	NA	NA	3464
[illegible]																																																																									

Chr12	6309867	A	AATTTTTT	Chr12_63098 A_AATTTTTT	INDEL	15761.59	28	10	5	20	15	14	27	25	15	16	7	9	10	7	[illegible]
Chr12	6309867	A	AATTTTTT	Chr12_63098 A_AATTTTTT	INDEL	15761.59	28	10	5	20	15	14	27	25	15	16	7	9	10	7	[illegible]
Chr12	6309867	A	AATTTTTT	Chr12_63098 A_AATTTTTT	INDEL	15761.59	28	10	5	20	15	14	27	25	15	16	7	9	10	7	[illegible]
Chr12	6309867	A	AATTTTTT	Chr12_63098 A_AATTTTTT	INDEL	15761.59	28	10	5	20	15	14	27	25	15	16	7	9	10	7	[illegible]
Chr12	6309867	A	AATTTTTT	Chr12_63098 A_AATTTTTT	INDEL	15761.59	28	10	5	20	15	14	27	25	15	16	7	9	10	7	[illegible]
Chr12	6309867	A	AATTTTTT	Chr12_63098 A_AATTTTTT	INDEL	15761.59	28	10	5	20	15	14	27	25	15	16	7	9	10	7	[illegible]
Chr12	6338349	G	T	Chr12_63383 G_T	SNP	18986.63	28	9	19	25	25	22	34	22	25	33	16	12	14	13	[illegible]
Chr12	6338349	G	T	Chr12_63383 G_T	SNP	18986.63	28	9	19	25	25	22	34	22	25	33	16	12	14	13	[illegible]
Chr12	6338349	G	T	Chr12_63383 G_T	SNP	18986.63	28	9	19	25	25	22	34	22	25	33	16	12	14	13	[illegible]
Chr12	6338349	G	T	Chr12_63383 G_T	SNP	18986.63	28	9	19	25	25	22	34	22	25	33	16	12	14	13	[illegible]
Chr12	6648493	T	A	Chr12_66484 T_A	SNP	19333.63	29	14	17	23	23	26	21	27	26	24	21	15	6	5	[illegible]
Chr12	6923721	A	G	Chr12_69237 A_G	SNP	23143.63	31	18	14	23	32	26	33	17	40	28	17	15	16	11	[illegible]
Chr12	6923721	A	G	Chr12_69237 A_G	SNP	23143.63	31	18	14	23	32	26	33	17	40	28	17	15	16	11	[illegible]
Chr12	6923721	A	G	Chr12_69237 A_G	SNP	23143.63	31	18	14	23	32	26	33	17	40	28	17	15	16	11	[illegible]
Chr12	6923721	A	G	Chr12_69237 A_G	SNP	23143.63	31	18	14	23	32	26	33	17	40	28	17	15	16	11	[illegible]
Chr12	6923721	A	G	Chr12_69237 A_G	SNP	23143.63	31	18	14	23	32	26	33	17	40	28	17	15	16	11	[illegible]
Chr12	6923721	A	G	Chr12_69237 A_G	SNP	23143.63	31	18	14	23	32	26	33	17	40	28	17	15	16	11	[illegible]
Chr12	6923724	G	GTT	Chr12_69237 G_GTT	INDEL	23471.59	31	18	15	23	31	26	32	18	39	29	17	15	16	11	[illegible]
Chr12	6923724	G	GTT	Chr12_69237 G_GTT	INDEL	23471.59	31	18	15	23	31	26	32	18	39	29	17	15	16	11	[illegible]
Chr12	6923724	G	GTT	Chr12_69237 G_GTT	INDEL	23471.59	31	18	15	23	31	26	32	18	39	29	17	15	16	11	[illegible]
Chr12	6923724	G	GTT	Chr12_69237 G_GTT	INDEL	23471.59	31	18	15	23	31	26	32	18	39	29	17	15	16	11	[illegible]
Chr12	6923724	G	GTT	Chr12_69237 G_GTT	INDEL	23471.59	31	18	15	23	31	26	32	18	39	29	17	15	16	11	[illegible]
Chr12	6923724	G	GTT	Chr12_69237 G_GTT	INDEL	23471.59	31	18	15	23	31	26	32	18	39	29	17	15	16	11	[illegible]
[illegible]																					

Chr12	11924286	C	CT	Chr12_11924C_CT	INDEL	18169.59	35	[illegible]
Chr12	11924286	C	CT	Chr12_11924C_CT	INDEL	18169.59	35	[illegible]
Chr12	11924286	C	CT	Chr12_11924C_CT	INDEL	18169.59	35	[illegible]
Chr12	11973323	G	GAT	Chr12_11973G_GAT	INDEL	2380.24	0	[illegible]
Chr12	11973323	G	GAT	Chr12_11973G_GAT	INDEL	2380.24	0	[illegible]
Chr12	11973323	G	GAT	Chr12_11973G_GAT	INDEL	2380.24	0	[illegible]
Chr12	11973323	G	GAT	Chr12_11973G_GAT	INDEL	2380.24	0	[illegible]
Chr12	11973323	G	GAT	Chr12_11973G_GAT	INDEL	2380.24	0	[illegible]
Chr12	11973323	G	GAT	Chr12_11973G_GAT	INDEL	2380.24	0	[illegible]
Chr12	11973323	G	GAT	Chr12_11973G_GAT	INDEL	2380.24	0	[illegible]
Chr12	11973323	G	GAT	Chr12_11973G_GAT	INDEL	2380.24	0	[illegible]
Chr12	11973323	G	GAT	Chr12_11973G_GAT	INDEL	2380.24	0	[illegible]
Chr12	12005957	G	GTA	Chr12_12005G_GTA	INDEL	5035.24	15	[illegible]
Chr12	12109257	A	AAAGG	Chr12_12109A_AAAGG	INDEL	20325.59	24	[illegible]
Chr12	12109257	A	AAAGG	Chr12_12109A_AAAGG	INDEL	20325.59	24	[illegible]
Chr12	12109257	A	AAAGG	Chr12_12109A_AAAGG	INDEL	20325.59	24	[illegible]
Chr12	12109257	A	AAAGG	Chr12_12109A_AAAGG	INDEL	20325.59	24	[illegible]
Chr12	12109257	A	AAAGG	Chr12_12109A_AAAGG	INDEL	20325.59	24	[illegible]
Chr12	12109261	GCTGGAA	G	Chr12_12109GCTGGAA_G	INDEL	19616.59	23	[illegible]
Chr12	12109261	GCTGGAA	G	Chr12_12109GCTGGAA_G	INDEL	19616.59	23	[illegible]
Chr12	12109261	GCTGGAA	G	Chr12_12109GCTGGAA_G	INDEL	19616.59	23	[illegible]
Chr12	12109261	GCTGGAA	G	Chr12_12109GCTGGAA_G	INDEL	19616.59	23	[illegible]
Chr12	12109261	GCTGGAA	G	Chr12_12109GCTGGAA_G	INDEL	19616.59	23	[illegible]
Chr12	12109269	G	C	Chr12_12109G_C	SNP	18860.63	23	[illegible]
Chr12	12109269	G	C	Chr12_12109G_C	SNP	18860.63	23	[illegible]
Chr12	12109269	G	C	Chr12_12109G_C	SNP	18860.63	23	[illegible]
Chr12	12109269	G	C	Chr12_12109G_C	SNP	18860.63	23	[illegible]
Chr12	12109269	G	C	Chr12_12109G_C	SNP	18860.63	23	[illegible]
Chr12	12250114	G	GA	Chr12_12250G_GA	INDEL	12494.59	23	[illegible]
Chr12	12402886	G	A	Chr12_12402G_A	SNP	5298.29	0	[illegible]
Chr12	12439623	G	GT	Chr12_12439G_GT	INDEL	13163.59	13	[illegible]
Chr12	12504793	CTA	C	Chr12_12504CTA_C	INDEL	133.37	1	[illegible]
Chr12	12575271	T	C	Chr12_12575T_C	SNP	16691.63	30	[illegible]
Chr12	12798808	A	AAT	Chr12_12798A_AAT	INDEL	1905.24	1	[illegible]
Chr12	12913295	C	T	Chr12_12913C_T	SNP	3975.98	0	[illegible]
Chr12	12930740	C	T	Chr12_12930C_T	SNP	6608.29	0	[illegible]
Chr12	13016094	A	T	Chr12_13016A_T	SNP	19901.63	34	[illegible]
Chr12	13016112	C	T	Chr12_13016C_T	SNP	21000.63	43	[illegible]
Chr12	13016113	A	G	Chr12_13016A_G	SNP	20955.63	42	[illegible]
Chr12	13019519	C	CA	Chr12_13019C_CA	INDEL	14391.59	29	[illegible]
Chr12	13049624	G	GAT	Chr12_13049G_GAT	INDEL	3717.24	1	[illegible]
Chr12	13339641	A	AT	Chr12_13339A_AT	INDEL	17184.59	21	[illegible]
Chr12	13339653	G	T	Chr12_13339G_T	SNP	17140.63	19	[illegible]
Chr12	13422048	G	A	Chr12_13422G_A	SNP	17840.63	36	[illegible]
Chr12	13562992	G	GAT	Chr12_13562G_GAT	INDEL	366.37	0	[illegible]
Chr12	13562992	G	GAT	Chr12_13562G_GAT	INDEL	366.37	0	[illegible]
Chr12	13562992	G	GAT	Chr12_13562G_GAT	INDEL	366.37	0	[illegible]
Chr12	13611953	A	T	Chr12_13611A_T	SNP	19252.63	21	[illegible]
Chr12	13612022	C	A	Chr12_13612C_A	SNP	19850.63	24	[illegible]
Chr12	13738305	G	GT	Chr12_13738G_GT	INDEL	14973.59	26	[illegible]
Chr12	13921681	C	A	Chr12_13921C_A	SNP	22534.63	29	[illegible]
Chr12	13921682	C	A	Chr12_13921C_A	SNP	22534.63	29	[illegible]
Chr12	13921693	G	A	Chr12_13921G_A	SNP	21891.63	26	[illegible]
Chr12	13921697	AG	A	Chr12_13921AG_A	INDEL	21948.59	26	[illegible]
Chr12	13921712	G	T	Chr12_13921G_T	SNP	19868.63	30	[illegible]
Chr12	13937082	C	CA	Chr12_13937C_CA	INDEL	15673.59	34	[illegible]
Chr12	13982962	C	CT	Chr12_13982C_CT	INDEL	17601.59	24	[illegible]
Chr12	13982962	C	CT	Chr12_13982C_CT	INDEL	17601.59	24	[illegible]
Chr12	13995435	G	T	Chr12_13995G_T	SNP	17788.63	17	[illegible]
Chr12	13995436	G	T	Chr12_13995G_T	SNP	17923.63	17	[illegible]
Chr12	13995441	TGGG	T	Chr12_13995TGGG_T	INDEL	17884.59	19	[illegible]
Chr12	13995444	G	GTT	Chr12_13995G_GTT	INDEL	17212.59	19	[illegible]
Chr12	14044750	T	TTA	Chr12_14044T_TTA	INDEL	1307.93	0	[illegible]
Chr12	14044750	T	TTA	Chr12_14044T_TTA	INDEL	1307.93	0	[illegible]
Chr12	14044750	T	TTA	Chr12_14044T_TTA	INDEL	1307.93	0	[illegible]
Chr12	14177050	C	T	Chr12_14177C_T	SNP	15104.63	16	[illegible]
Chr12	14272319	G	GA	Chr12_14272G_GA	INDEL	12658.59	20	[illegible]
Chr12	14272319	G	GA	Chr12_14272G_GA	INDEL	12658.59	20	[illegible]
Chr12	14272319	G	GA	Chr12_14272G_GA	INDEL	12658.59	20	[illegible]
Chr12	14272319	G	GA	Chr12_14272G_GA	INDEL	12658.59	20	[illegible]
Chr12	14272319	G	GA	Chr12_14272G_GA	INDEL	12658.59	20	[illegible]
Chr12	14272319	G	GA	Chr12_14272G_GA	INDEL	12658.59	20	[illegible]
Chr12	14272319	G	GA	Chr12_14272G_GA	INDEL	12658.59	20	[illegible]
Chr12	14272319	G	GA	Chr12_14272G_GA	INDEL	12658.59	20	[illegible]
Chr12	14272319	G	GA	Chr12_14272G_GA	INDEL	12658.59	20	[illegible]
Chr12	14272319	G	GA	Chr12_14272G_GA	INDEL	12658.59	20	[illegible]
Chr12	14272319	G	GA	Chr12_14272G_GA	INDEL	12658.59	20	[illegible]
Chr12	14310671	AT	A	Chr12_14310AT_A	INDEL	11996.59	13	[illegible]
Chr12	14315726	GC	G	Chr12_14315GC_G	INDEL	20512.59	28	[illegible]
Chr12	14345820	G	GA	Chr12_14345G_GA	INDEL	17244.59	19	[illegible]
Chr12	14363579	G	A	Chr12_14363G_A	SNP	16092.63	18	[illegible]
Chr12	14377467	G	T	Chr12_14377G_T	SNP	17378.63	23	[illegible]
Chr12	14377582	C	T	Chr12_14377C_T	SNP	17979.63	31	[illegible]
Chr12	14377687	C	T	Chr12_14377C_T	SNP	18843.63	29	[illegible]
Chr12	14388398	G	C	Chr12_14388G_C	SNP	6494.29	0	[illegible]
Chr12	14535132	T	C	Chr12_14535T_C	SNP	1885.39	24	[illegible]
Chr12	14535132	T	C	Chr12_14535T_C	SNP	1885.39	24	[illegible]
Chr12	14535132	T	C	Chr12_14535T_C	SNP	1885.39	24	[illegible]
Chr12	14535132	T	C	Chr12_14535T_C	SNP	1885.39	24	[illegible]
Chr12	14535132	T	C	Chr12_14535T_C	SNP	1885.39	24	[illegible]
Chr12	14535132	T	C	Chr12_14535T_C	SNP	1885.39	24	[illegible]
Chr12	14535132	T	C	Chr12_14535T_C	SNP	1885.39	24	[illegible]
Chr12	14535132	T	C	Chr12_14535T_C	SNP	1885.39	24	[illegible]
Chr12	14535132	T	C	Chr12_14535T_C	SNP	1885.39	24	[illegible]
Chr12	14535132	T	C	Chr12_14535T_C	SNP	1885.39	24	[illegible]
Chr12	14537433	T	TTA	Chr12_14537T_TTA	INDEL	68.37	2	[illegible]
Chr12	14537433	T	TTA	Chr12_14537T_TTA	INDEL	68.37	2	[illegible]
Chr12	14537433	T	TTA	Chr12_14537T_TTA	INDEL	68.37	2	[illegible]
Chr12	14537433	T	TTA	Chr12_14537T_TTA	INDEL	68.37	2	[illegible]
Chr12	14537433	T	TTA	Chr12_14537T_TTA	INDEL	68.37	2	[illegible]
Chr12	14537433	T	TTA	Chr12_14537T_TTA	INDEL	68.37	2	[illegible]
Chr12	14537433	T	TTA	Chr12_14537T_TTA	INDEL	68.37	2	[illegible]
Chr12	14537433	T	TTA	Chr12_14537T_TTA	INDEL	68.37	2	[illegible]
Chr12	14537433	T	TTA	Chr12_14537T_TTA	INDEL	68.37	2	[illegible]
Chr12	14537433	T	TTA	Chr12_14537T_TTA	INDEL	68.37	2	[illegible]
Chr12	14541457	G	T	Chr12_14541G_T	SNP	18124.63	30	[illegible]
Chr12	14541457	G	T	Chr12_14541G_T	SNP	18124.63	30	[illegible]
Chr12	14541457	G	T	Chr12_14541G_T	SNP	18124.63	30	[illegible]
Chr12	14541457	G	T	Chr12_14541G_T	SNP	18124.63	30	[illegible]
Chr12	14541457	G	T	Chr12_14541G_T	SNP	18124.63	30	[illegible]
Chr12	14541457	G	T	Chr12_14541G_T	SNP	18124.63	30	[illegible]
Chr12	14541457	G	T	Chr12_14541G_T	SNP	18124.63	30	[illegible]
Chr12	14541457	G	T	Chr12_14541G_T	SNP	18124.63	30	[illegible]
Chr12	14541457	G	T	Chr12_14541G_T	SNP	18124.63	30	[illegible]
Chr12	14541457	G	T	Chr12_14541G_T	SNP	18124.63	30	[illegible]
Chr12	14541457	G	T	Chr12_14541G_T	SNP	18124.63	30	[illegible]
Chr12	14574487	A	C	Chr12_14574A_C	SNP	2176.29	0	[illegible]
Chr12	14574487	A	C	Chr12_14574A_C	SNP	2176.29	0	[illegible]
Chr12	14574487	A	C	Chr12_14574A_C	SNP	2176.29	0	[illegible]
Chr12	14636390	T	TTA	Chr12_14636T_TTA	INDEL	5497.24	14	[illegible]
Chr12	14817323	A	AT	Chr12_14817A_AT	INDEL	15944.59	22	[illegible]
Chr12	14893766	A	AT	Chr12_14893A_AT	INDEL	13971.59	20	[illegible]
Chr12	14893766	A	AT	Chr12_14893A_AT	INDEL	13971.59	20	[illegible]
Chr12	14893766	A	AT	Chr12_14893A_AT	INDEL	13971.59	20	[illegible]
Chr12	14893766	A	AT	Chr12_14893A_AT	INDEL	13971.59	20	[illegible]
Chr12	14893766	A	AT	Chr12_14893A_AT	INDEL	13971.59	20	[illegible]
Chr12	14893766	A	AT	Chr12_14893A_AT	INDEL	13971.59	20	[illegible]
Chr12	14893766	A	AT	Chr12_14893A_AT	INDEL	13971.59	20	[illegible]
Chr12	14893766	A	AT	Chr12_14893A_AT	INDEL	13971.59	20	[illegible]
Chr12	14893766	A	AT	Chr12_14893A_AT	INDEL	13971.59	20	[illegible]
Chr12	14893766	A	AT	Chr12_14893A_AT	INDEL	13971.59	20	[illegible]
Chr12	14893766	A	AT	Chr12_14893A_AT	INDEL	13971.59	20	[illegible]
Chr12	14893766	A	AT	Chr12_14893A_AT	INDEL	13971.59	20	[illegible]
Chr12	14893766	A	AT	Chr12_14893A_AT	INDEL	13971.59	20	[illegible]
Chr12	14893766	A	AT	Chr12_14893A_AT	INDEL	13971.59	20	[illegible]
Chr12	14893766	A	AT	Chr12_14893A_AT	INDEL	13971.59	20	[illegible]
Chr12	14893766	A	AT	Chr12_14893A_AT	INDEL	13971.59	20	[illegible]
Chr12	14921013	GTA	G	Chr12_14921GTA_G	INDEL	6512.59	15	[illegible]
Chr12	14932823	C	T	Chr12_14932C_T	SNP	7339.29	0	[illegible]
Chr12	14932823	C	T	Chr12_14932C_T	SNP	7339.29	0	[illegible]
Chr12	14932823	C	T	Chr12_14932C_T	SNP	7339.29	0	[illegible]
Chr12	14932823	C	T	Chr12_14932C_T	SNP	7339.29	0	[illegible]
Chr12	14932823	C	T	Chr12_14932C_T	SNP	7339.29	0	[illegible]
Chr12	14932823	C	T	Chr12_14932C_T	SNP	7339.29	0	[illegible]
Chr12	14932823	C	T	Chr12_14932C_T	SNP	7339.29	0	[illegible]
Chr12	14955562	G	T	Chr12_14955G_T	SNP	15036.63	20	[illegible]
Chr12	14955562	G	T	Chr12_14955G_T	SNP	15036.63	20	[illegible]
Chr12	14955563	A	ACC	Chr12_14955A_ACC	INDEL	15027.59	20	[illegible]
Chr12	14955563	A	ACC	Chr12_14955A_ACC	INDEL	15027.59	20	[illegible]
Chr12	14955566	G	A	Chr12_14955G_A	SNP	14982.63	20	[illegible]
Chr12	14955566	G	A	Chr12_14955G_A	SNP	14982.63	20	[illegible]
Chr12	15046531	T	C	Chr12_15046T_C	SNP	14578.63	19	[illegible]
Chr12	15183412	AG	A	Chr12_15183AG_A	INDEL	21060.59	21	[illegible]
Chr12	15183412	AG	A	Chr12_15183AG_A	INDEL	21060.59	21	[illegible]
Chr12	15183412	AG	A	Chr12_15183AG_A	INDEL	21060.59	21	[illegible]
Chr12	15183412	AG	A	Chr12_15183AG_A	INDEL	21060.59	21	[illegible]
Chr12	15210544	G	A	Chr12_15210G_A	SNP	13468.63	24	[illegible]
Chr12	15210544	G	A	Chr12_15210G_A	SNP	13468.63	24	[illegible]
Chr12	15210544	G	A	Chr12_15210G_A	SNP	13468.63	24	[illegible]
Chr12	15210544	G	A	Chr12_15210G_A	SNP	13468.63	24	[illegible]
Chr12	15210544	G	A	Chr12_15210G_A	SNP	13468.63	24	[illegible]
Chr12	15210561	C	T	Chr12_15210C_T	SNP	12581.63	21	[illegible]
Chr12	15210561	C	T	Chr12_15210C_T	SNP	12581.63	21	[illegible]
Chr12	15210561	C	T	Chr12_15210C_T	SNP	12581.63	21	[illegible]
Chr12	15210561	C	T	Chr12_15210C_T	SNP	12581.63	21	[illegible]
Chr12	15210561	C	T	Chr12_15210C_T	SNP	12581.63	21	[illegible]
Chr12	15289646	G	GTA	Chr12_15289G_GTA	INDEL	1060.35	16	[illegible]
Chr12	15289646	G	GTA	Chr12_15289G_GTA	INDEL	1060.35	16	[illegible]
Chr12	15289646	G	GTA	Chr12_15289G_GTA	INDEL	1060.35	16	[illegible]
Chr12	15289646	G	GTA	Chr12_15289G_GTA	INDEL	1060.35	16	[illegible]
Chr12	15289646	G	GTA	Chr12_15289G_GTA	INDEL	1060.35	16	[illegible]
Chr12	15289646	G	GTA	Chr12_15289G_GTA	INDEL	1060.35	16	[illegible]
Chr12	15289646	G	GTA	Chr12_15289G_GTA	INDEL	1060.35	16	[illegible]
Chr12	15406376	A	AAT	Chr12_15406A_AAT	INDEL	1958.24	1	[illegible]
Chr12	15406376	A	AAT	Chr12_15406A_AAT	INDEL	1958.24	1	[illegible]
Chr12	15406376	A	AAT	Chr12_15406A_AAT	INDEL	1958.24	1	[illegible]
Chr12	15406376	A	AAT	Chr12_15406A_AAT	INDEL	1958.24	1	[illegible]
Chr12	15406376	A	AAT	Chr12_15406A_AAT	INDEL	1958.24	1	[illegible]
Chr12	15406376	A	AAT	Chr12_15406A_AAT	INDEL	1958.24	1	[illegible]
Chr12	15406376	A	AAT	Chr12_15406A_AAT	INDEL	1958.24	1	[illegible]
Chr12	15406376	A	AAT	Chr12_15406A_AAT	INDEL	1958.24	1	[illegible]
Chr12	15406376	A	AAT	Chr12_15406A_AAT	INDEL	1958.24	1	[illegible]
Chr12	15406376	A	AAT	Chr12_15406A_AAT	INDEL	1958.24	1	[illegible]
Chr12	15406376	A	AAT	Chr12_15406A_AAT	INDEL	1958.24	1	[illegible]
Chr12	15406376	A	AAT	Chr12_15406A_AAT	INDEL	1958.24	1	[illegible]
Chr12	15406376	A	AAT	Chr12_15406A_AAT	INDEL	1958.24	1	[illegible]
Chr12	15406376	A	AAT	Chr12_15406A_AAT	INDEL	1958.24	1	[illegible]
Chr12	15406376	A	AAT	Chr12_15406A_AAT	INDEL	1958.24	1	[illegible]
Chr12	15670040	AAT	A	Chr12_15670AAT_A	INDEL	5950.24	17	[illegible]
Chr12	15670040	AAT	A	Chr12_15670AAT_A	INDEL	5950.24	17	[illegible]
Chr12	15670040	AAT	A	Chr12_15670AAT_A	INDEL	5950.24	17	[illegible]
Chr12	15714451	T	TTA	Chr12_15714T_TTA	INDEL	9479.59	26	[illegible]
Chr12	15714451	T	TTA	Chr12_15714T_TTA	INDEL	9479.59	26	[illegible]
Chr12	15714451	T	TTA	Chr12_15714T_TTA	INDEL	9479.59	26	[illegible]
Chr12	15802863	C	T	Chr12_15802C_T	SNP	2189.39	27	[illegible]
Chr12	15821902	C	A	Chr12_15821C_A	SNP	22501.63	37	[illegible]
Chr12	15821976	A	AT	Chr12_15821A_AT	INDEL	16550.59	23	[illegible]
Chr12	15824328	T	TAAG	Chr12_15824T_TAAG	INDEL	22149.59	31	[illegible]
Chr12	15824329	C	A	Chr12_15824C_A	SNP	22248.63	32	[illegible]
Chr12	15824332	TCCA	T	Chr12_15824TCCA_T	INDEL	21760.59	30	[illegible]
Chr12	15969136	A	T	Chr12_15969A_T	SNP	12477.63	17	[illegible]
Chr12	15969136	A	T	Chr12_15969A_T	SNP	12477.63	17	[illegible]

Chr12	15973318	C	A	Chr12_15973	C_A	SNP	19894.63	28	13	11	[illegible]
Chr12	15973372	C	T	Chr12_15973	C_T	SNP	19154.63	23	10	7	[illegible]
Chr12	15973511	T	G	Chr12_15973	T_G	SNP	18934.63	29	12	15	[illegible]
Chr12	16044262	G	A	Chr12_16044	G_A	SNP	12131.63	16	8	15	[illegible]
Chr12	16090346	C	T	Chr12_16090	C_T	SNP	4944.29	0	0	0	[illegible]
Chr12	16266255	CAT	C	Chr12_16266	CAT_C	INDEL	1338.5	1	0	0	[illegible]
Chr12	16306510	T	TTA	Chr12_16306	T_TTA	INDEL	9163.59	14	14	7	[illegible]
Chr12	16306510	T	TTA	Chr12_16306	T_TTA	INDEL	9163.59	14	14	7	[illegible]
Chr12	16306510	T	TTA	Chr12_16306	T_TTA	INDEL	9163.59	14	14	7	[illegible]
Chr12	16353563	TTA	T	Chr12_16353	TTA_T	INDEL	12031.59	31	18	12	[illegible]
Chr12	16353563	TTA	T	Chr12_16353	TTA_T	INDEL	12031.59	31	18	12	[illegible]
Chr12	16353563	TTA	T	Chr12_16353	TTA_T	INDEL	12031.59	31	18	12	[illegible]
Chr12	16446393	G	GTA	Chr12_16446	G_GTA	INDEL	9219.24	21	15	11	[illegible]
Chr12	16446393	G	GTA	Chr12_16446	G_GTA	INDEL	9219.24	21	15	11	[illegible]
Chr12	16446393	G	GTA	Chr12_16446	G_GTA	INDEL	9219.24	21	15	11	[illegible]
Chr12	16490813	C	G	Chr12_16490	C_G	SNP	19096.63	16	14	17	[illegible]
Chr12	16490823	G	T	Chr12_16490	G_T	SNP	19086.63	15	15	17	[illegible]
Chr12	16490844	G	GT	Chr12_16490	G_GT	INDEL	15130.59	11	14	13	[illegible]
Chr12	16753860	T	TA	Chr12_16753	T_TA	INDEL	20176.59	25	16	14	[illegible]
Chr12	16753860	T	TA	Chr12_16753	T_TA	INDEL	20176.59	25	16	14	[illegible]
Chr12	16779730	T	TA	Chr12_16779	T_TA	INDEL	13286.59	32	11	18	[illegible]
Chr12	16783805	G	GA	Chr12_16783	G_GA	INDEL	11958.59	26	12	9	[illegible]
Chr12	16808049	G	T	Chr12_16808	G_T	SNP	13127.63	24	5	11	[illegible]
Chr12	16808049	G	T	Chr12_16808	G_T	SNP	13127.63	24	5	11	[illegible]
Chr12	16808049	G	T	Chr12_16808	G_T	SNP	13127.63	24	5	11	[illegible]
Chr12	16808049	G	T	Chr12_16808	G_T	SNP	13127.63	24	5	11	[illegible]
Chr12	16808049	G	T	Chr12_16808	G_T	SNP	13127.63	24	5	11	[illegible]
Chr12	16864190	TA	T	Chr12_16864	TA_T	INDEL	14038.59	19	10	19	[illegible]
Chr12	16864308	CA	C	Chr12_16864	CA_C	INDEL	13748.59	15	11	10	[illegible]
Chr12	16864315	G	T	Chr12_16864	G_T	SNP	14612.63	15	11	10	[illegible]
Chr12	16864346	C	T	Chr12_16864	C_T	SNP	14690.63	19	13	16	[illegible]
Chr12	16864348	A	G	Chr12_16864	A_G	SNP	14960.63	18	13	16	[illegible]
Chr12	16864351	C	A	Chr12_16864	C_A	SNP	15230.63	19	11	16	[illegible]
Chr12	16864354	C	A	Chr12_16864	C_A	SNP	15050.63	19	11	16	[illegible]
Chr12	16864367	T	A	Chr12_16864	T_A	SNP	14870.63	20	10	18	[illegible]
Chr12	16864372	C	A	Chr12_16864	C_A	SNP	14690.63	19	9	17	[illegible]
Chr12	16864374	A	T	Chr12_16864	A_T	SNP	14600.63	19	8	17	[illegible]
Chr12	16864378	T	G	Chr12_16864	T_G	SNP	14735.63	21	8	17	[illegible]
Chr12	16864380	A	G	Chr12_16864	A_G	SNP	14195.63	19	8	17	[illegible]
Chr12	16864388	A	T	Chr12_16864	A_T	SNP	14105.63	19	7	17	[illegible]
Chr12	16864389	T	A	Chr12_16864	T_A	SNP	14105.63	22	7	17	[illegible]
Chr12	16864399	T	A	Chr12_16864	T_A	SNP	14510.63	22	11	16	[illegible]
Chr12	16864403	TG	T	Chr12_16864	TG_T	INDEL	14498.59	23	10	15	[illegible]
Chr12	16864427	G	A	Chr12_16864	G_A	SNP	16220.63	26	11	20	[illegible]
Chr12	16864476	G	A	Chr12_16864	G_A	SNP	18924.63	29	12	22	[illegible]
Chr12	16864506	G	A	Chr12_16864	G_A	SNP	19812.63	28	12	20	[illegible]
Chr12	16864558	G	A	Chr12_16864	G_A	SNP	19480.63	29	16	22	[illegible]
Chr12	16940418	C	A	Chr12_16940	C_A	SNP	4904.29	0	0	0	[illegible]
Chr12	16940418	C	A	Chr12_16940	C_A	SNP	4904.29	0	0	0	[illegible]
Chr12	16940418	C	A	Chr12_16940	C_A	SNP	4904.29	0	0	0	[illegible]
Chr12	16940418	C	A	Chr12_16940	C_A	SNP	4904.29	0	0	0	[illegible]
Chr12	16943042	C	CA	Chr12_16943	C_CA	INDEL	16744.59	28	14	5	[illegible]
Chr12	16943042	C	CA	Chr12_16943	C_CA	INDEL	16744.59	28	14	5	[illegible]
Chr12	16943042	C	CA	Chr12_16943	C_CA	INDEL	16744.59	28	14	5	[illegible]
Chr12	17000414	C	CAG	Chr12_17000	C_CAG	INDEL	16616.59	27	10	10	[illegible]
Chr12	17000417	T	G	Chr12_17000	T_G	SNP	16895.63	29	10	10	[illegible]
Chr12	17000420	C	T	Chr12_17000	C_T	SNP	16940.63	29	11	10	[illegible]
Chr12	17000422	C	A	Chr12_17000	C_A	SNP	16977.63	29	11	10	[illegible]
Chr12	17000424	C	A	Chr12_17000	C_A	SNP	16977.63	30	11	10	[illegible]
Chr12	17039719	C	G	Chr12_17039	C_G	SNP	15464.63	16	12	13	[illegible]
Chr12	17039719	C	G	Chr12_17039	C_G	SNP	15464.63	16	12	13	[illegible]
Chr12	17039719	C	G	Chr12_17039	C_G	SNP	15464.63	16	12	13	[illegible]
Chr12	17039719	C	G	Chr12_17039	C_G	SNP	15464.63	16	12	13	[illegible]
Chr12	17039719	C	G	Chr12_17039	C_G	SNP	15464.63	16	12	13	[illegible]
Chr12	17102597	A	ATT	Chr12_17102	A_ATT	INDEL	21090.59	35	17	12	[illegible]
Chr12	17102602	C	A	Chr12_17102	C_A	SNP	21391.63	35	17	11	[illegible]
Chr12	17102605	G	A	Chr12_17102	G_A	SNP	21232.63	34	16	11	[illegible]
Chr12	17102606	C	A	Chr12_17102	C_A	SNP	21097.63	35	16	11	[illegible]
Chr12	17222553	C	A	Chr12_17222	C_A	SNP	19782.63	21	11	12	[illegible]
Chr12	17430806	G	A	Chr12_17430	G_A	SNP	19241.63	23	11	17	[illegible]
Chr13	26794	A	T	Chr13_26794	A_T	SNP	13707.63	30	7	12	[illegible]
Chr13	26799	A	AGT	Chr13_26799	A_AGT	INDEL	14068.59	29	7	9	[illegible]
Chr13	26802	AATTCAT	A	Chr13_26802	AATTCAT_A	INDEL	12543.37	29	8	10	[illegible]
Chr13	26812	T	TATC	Chr13_26812	T_TATC	INDEL	12453.39	28	9	11	[illegible]
Chr13	26816	TCTG	T	Chr13_26816	TCTG_T	INDEL	12183.37	26	10	11	[illegible]
Chr13	26821	AGAT	A	Chr13_26821	AGAT_A	INDEL	12228.38	26	10	11	[illegible]
Chr13	26831	AG	A	Chr13_26831	AG_A	INDEL	12452.63	28	10	11	[illegible]
Chr13	26833	T	TAA	Chr13_26833	T_TAA	INDEL	12317.63	28	10	11	[illegible]
Chr13	26836	T	A	Chr13_26836	T_A	SNP	12439.41	30	9	13	[illegible]
Chr13	26839	A	ATT	Chr13_26839	A_ATT	INDEL	12610.37	29	9	13	[illegible]
Chr13	26842	C	CAAAGAAAG	Chr13_26842	C_CAAAGAA	INDEL	14702.61	29	9	13	[illegible]
Chr13	26844	C	T	Chr13_26844	C_T	SNP	14711.63	30	8	16	[illegible]
Chr13	26845	C	A	Chr13_26845	C_A	SNP	12911.66	29	8	16	[illegible]
Chr13	26848	T	TAGAAGAA	Chr13_26848	T_TAGAAGA	INDEL	14252.59	27	7	16	[illegible]
Chr13	26851	C	CA	Chr13_26851	C_CA	INDEL	13757.59	23	8	15	[illegible]
Chr13	26854	GC	G	Chr13_26854	GC_G	INDEL	13802.59	24	7	16	[illegible]
Chr13	26856	C	T	Chr13_26856	C_T	SNP	13451.63	25	8	16	[illegible]
Chr13	26859	TCAGCTAAA	T	Chr13_26859	TCAGCTAAA	INDEL	13411.6	25	8	16	[illegible]
Chr13	201171	A	AT	Chr13_20117	A_AT	INDEL	22728.59	26	15	17	[illegible]
Chr13	201172	C	CA	Chr13_20117	C_CA	INDEL	22835.59	26	15	17	[illegible]
Chr13	201174	T	A	Chr13_20117	T_A	SNP	22761.63	26	15	17	[illegible]
Chr13	561778	C	A	Chr13_56177	C_A	SNP	16442.63	27	12	7	[illegible]
Chr13	561784	AC	A	Chr13_56178	AC_A	INDEL	16267.59	23	11	7	[illegible]
Chr13	561790	G	T	Chr13_56179	G_T	SNP	16175.63	22	12	8	[illegible]
Chr13	561827	CG	C	Chr13_56182	CG_C	INDEL	16076.59	22	14	11	[illegible]
Chr13	561831	AC	A	Chr13_56183	AC_A	INDEL	15851.59	22	15	11	[illegible]
Chr13	561834	CA	C	Chr13_56183	CA_C	INDEL	15941.59	22	15	11	[illegible]
Chr13	561845	G	T	Chr13_56184	G_T	SNP	16445.63	20	15	11	[illegible]
Chr13	561846	C	A	Chr13_56184	C_A	SNP	16445.63	19	15	11	[illegible]
Chr13	561854	G	T	Chr13_56185	G_T	SNP	16265.63	17	14	10	[illegible]
Chr13	561855	AC	A	Chr13_56185	AC_A	INDEL	16571.59	18	14	11	[illegible]
Chr13	561859	T	TAG	Chr13_56185	T_TAG	INDEL	16526.59	19	14	11	[illegible]
Chr13	561860	TTTC	T	Chr13_56186	TTTC_T	INDEL	16526.59	20	14	11	[illegible]
Chr13	561869	G	GT	Chr13_56186	G_GT	INDEL	16346.59	19	14	11	[illegible]
Chr13	561871	G	T	Chr13_56187	G_T	SNP	16310.63	19	15	11	[illegible]
Chr13	561873	A	C	Chr13_56187	A_C	SNP	16220.63	18	14	11	[illegible]
Chr13	561874	TCGAGGAA	T	Chr13_56187	TCGAGGAA_	INDEL	16031.59	17	14	11	[illegible]
Chr13	561885	C	T	Chr13_56188	C_T	SNP	15815.63	17	13	11	[illegible]
Chr13	561888	G	GAT	Chr13_56188	G_GAT	INDEL	16031.59	16	13	12	[illegible]
Chr13	561889	G	GTTAC	Chr13_56188	G_GTTAC	INDEL	16076.59	16	13	12	[illegible]
Chr13	561892	AAT	A	Chr13_56189	AAT_A	INDEL	16366.59	17	11	12	[illegible]
Chr13	561895	C	CAA	Chr13_56189	C_CAA	INDEL	16075.59	17	11	12	[illegible]
Chr13	561912	T	TA	Chr13_56191	T_TA	INDEL	14416.59	14	9	14	[illegible]
Chr13	621537	A	AAT	Chr13_62153	A_AAT	INDEL	3563.24	0	0	0	[illegible]
Chr13	621537	A	AAT	Chr13_62153	A_AAT	INDEL	3563.24	0	0	0	[illegible]
Chr13	621537	A	AAT	Chr13_62153	A_AAT	INDEL	3563.24	0	0	0	[illegible]
Chr13	621537	A	AAT	Chr13_62153	A_AAT	INDEL	3563.24	0	0	0	[illegible]
Chr13	653537	CAT	C	Chr13_65353	CAT_C	INDEL	11454.59	14	12	12	[illegible]
Chr13	653537	CAT	C	Chr13_65353	CAT_C	INDEL	11454.59	14	12	12	[illegible]
Chr13	653537	CAT	C	Chr13_65353	CAT_C	INDEL	11454.59	14	12	12	[illegible]
Chr13	658497	AAT	A	Chr13_65849	AAT_A	INDEL	11650.59	12	12	14	[illegible]
Chr13	714310	G	T	Chr13_71431	G_T	SNP	22978.63	34	16	13	[illegible]
Chr13	714310	G	T	Chr13_71431	G_T	SNP	22978.63	34	16	13	[illegible]
Chr13	714310	G	T	Chr13_71431	G_T	SNP	22978.63	34	16	13	[illegible]
Chr13	714311	G	T	Chr13_71431	G_T	SNP	23089.63	35	16	13	[illegible]
Chr13	714311	G	T	Chr13_71431	G_T	SNP	23089.63	35	16	13	[illegible]
Chr13	714311	G	T	Chr13_71431	G_T	SNP	23089.63	35	16	13	[illegible]
Chr13	714313	A	T	Chr13_71431	A_T	SNP	23016.63	36	16	12	[illegible]
Chr13	714313	A	T	Chr13_71431	A_T	SNP	23016.63	36	16	12	[illegible]
Chr13	714313	A	T	Chr13_71431	A_T	SNP	23016.63	36	16	12	[illegible]
Chr13	729402	G	C	Chr13_72940	G_C	SNP	16229.63	29	17	15	[illegible]
Chr13	729402	G	C	Chr13_72940	G_C	SNP	16229.63	29	17	15	[illegible]
Chr13	729402	G	C	Chr13_72940	G_C	SNP	16229.63	29	17	15	[illegible]
Chr13	729402	G	C	Chr13_72940	G_C	SNP	16229.63	29	17	15	[illegible]
Chr13	729402	G	C	Chr13_72940	G_C	SNP	16229.63	29	17	15	[illegible]
Chr13	729402	G	C	Chr13_72940	G_C	SNP	16229.63	29	17	15	[illegible]
Chr13	729402	G	C	Chr13_72940	G_C	SNP	16229.63	29	17	15	[illegible]
Chr13	729402	G	C	Chr13_72940	G_C	SNP	16229.63	29	17	15	[illegible]
Chr13	729402	G	C	Chr13_72940	G_C	SNP	16229.63	29	17	15	[illegible]
Chr13	729402	G	C	Chr13_72940	G_C	SNP	16229.63	29	17	15	[illegible]
Chr13	744815	G	GAT	Chr13_74481	G_GAT	INDEL	180.37	1	0	0	[illegible]
Chr13	744815	G	GAT	Chr13_74481	G_GAT	INDEL	180.37	1	0	0	[illegible]
Chr13	744815	G	GAT	Chr13_74481	G_GAT	INDEL	180.37	1	0	0	[illegible]
Chr13	744815	G	GAT	Chr13_74481	G_GAT	INDEL	180.37	1	0	0	[illegible]
Chr13	744815	G	GAT	Chr13_74481	G_GAT	INDEL	180.37	1	0	0	[illegible]
Chr13	822370	AGT	A	Chr13_82237	AGT_A	INDEL	4300	15	5	4	[illegible]
Chr13	822374	TTA	T	Chr13_82237	TTA_T	INDEL	3953.65	15	5	4	[illegible]
Chr13	876859	T	A	Chr13_87685	T_A	SNP	11349.63	9	7	8	[illegible]
Chr13	876859	T	A	Chr13_87685	T_A	SNP	11349.63	9	7	8	[illegible]
Chr13	876859	T	A	Chr13_87685	T_A	SNP	11349.63	9	7	8	[illegible]
Chr13	876859	T	A	Chr13_87685	T_A	SNP	11349.63	9	7	8	[illegible]
Chr13	876859	T	A	Chr13_87685	T_A	SNP	11349.63	9	7	8	[illegible]
Chr13	876859	T	A	Chr13_87685	T_A	SNP	11349.63	9	7	8	[illegible]
Chr13	876859	T	A	Chr13_87685	T_A	SNP	11349.63	9	7	8	[illegible]
Chr13	876859	T	A	Chr13_87685	T_A	SNP	11349.63	9	7	8	[illegible]
Chr13	877805	C	A	Chr13_87780	C_A	SNP	17839.63	17	17	11	[illegible]
Chr13	877805	C	A	Chr13_87780	C_A	SNP	17839.63	17	17	11	[illegible]
Chr13	877805	C	A	Chr13_87780	C_A	SNP	17839.63	17	17	11	[illegible]
Chr13	877805	C	A	Chr13_87780	C_A	SNP	17839.63	17	17	11	[illegible]
Chr13	877805	C	A	Chr13_87780	C_A	SNP	17839.63	17	17	11	[illegible]
Chr13	878748	G	GC	Chr13_87874	G_GC	INDEL	19985.59	19	14	14	[illegible]
Chr13	878748	G	GC	Chr13_87874	G_GC	INDEL	19985.59	19	14	14	[illegible]
Chr13	878748	G	GC	Chr13_87874	G_GC	INDEL	19985.59	19	14	14	[illegible]
Chr13	878748	G	GC	Chr13_87874	G_GC	INDEL	19985.59	19	14	14	[illegible]
Chr13	878748	G	GC	Chr13_87874	G_GC	INDEL	19985.59	19	14	14	[illegible]
Chr13	878769	G	T	Chr13_87876	G_T	SNP	20658.63	24	15	15	[illegible]
Chr13	878769	G	T	Chr13_87876	G_T	SNP	20658.63	24	15	15	[illegible]
Chr13	878769	G	T	Chr13_87876	G_T	SNP	20658.63	24	15	15	[illegible]
Chr13	878769	G	T	Chr13_87876	G_T	SNP	20658.63	24	15	15	[illegible]
Chr13	878769	G	T	Chr13_87876	G_T	SNP	20658.63	24	15	15	[illegible]
Chr13	916629	A	T	Chr13_91662	A_T	SNP	8199.63	19	8	5	[illegible]
Chr13	1001933	C	CA	Chr13_10019	C_CA	INDEL	16793.59	28	7	21	[illegible]
Chr13	1001952	A	C	Chr13_10019	A_C	SNP	20998.63	28	7	23	[illegible]
Chr13	1001953	C	A	Chr13_10019	C_A	SNP	20998.63	28	7	22	[illegible]
Chr13	1001985	A	G	Chr13_10019	A_G	SNP	19732.63	31	7	22	[illegible]
Chr13	1005847	C	T	Chr13_10058	C_T	SNP	15993.63	18	23	12	[illegible]
Chr13	1005867	A	T	Chr13_10058	A_T	SNP	16731.63	17	23	12	[illegible]
Chr13	1005869	A	C	Chr13_10058	A_C	SNP	16725.63	19	23	12	[illegible]
Chr13	1005871	A	G	Chr13_10058	A_G	SNP	16635.63	19	23	13	[illegible]
Chr13	1006007	A	AT	Chr13_10060	A_AT	INDEL	16389.59	36	24	12	[illegible]
Chr13	1006045	G	GT	Chr13_10060	G_GT	INDEL	15190.59	30	19	15	[illegible]
Chr13	1475499	G	GAT	Chr13_14754	G_GAT	INDEL	2015.93	1	1	1	[illegible]
Chr13	1475499	G	GAT	Chr13_14754	G_GAT	INDEL	2015.93	1	1	1	[illegible]
Chr13	1475499	G	GAT	Chr13_14754	G_GAT	INDEL	2015.93	1	1	1	[illegible]
Chr13	1475499	G	GAT	Chr13_14754	G_GAT	INDEL	2015.93	1	1	1	[illegible]
Chr13	1475499	G	GAT	Chr13_14754	G_GAT	INDEL	2015.93	1	1	1	[illegible]
Chr13	1475499	G	GAT	Chr13_14754	G_GAT	INDEL	2015.93	1	1	1	[illegible]
Chr13	1475499	G	GAT	Chr13_14754	G_GAT	INDEL	2015.93	1	1	1	[illegible]
Chr13	1516971	A	AT	Chr13_15169	A_AT	INDEL	18890.59	32	15	17	[illegible]
Chr13	1516972	A	G	Chr13_15169	A_G	SNP	19068.63	33	15	17	[illegible]
Chr13	1516974	G	C	Chr13_15169	G_C	SNP	18921.63	33	15	17	[illegible]
Chr13	1530991	C	T	Chr13_15309	C_T	SNP	3832.98	0	0	0	[illegible]
Chr13	1623513	A	AAT	Chr13_16235	A_AAT	INDEL	2100.24	0	0	0	[illegible]
Chr13	1709385	C	CT	Chr13_17093	C_CT	INDEL	17473.59	28	12	20	[illegible]
Chr13	1709385	C	CT	Chr13_17093	C_CT	INDEL	17473.59	28	12	20	[illegible]
Chr13	1760374	T	G	Chr13_17603	T_G	SNP	9621.63	20	5	5	[illegible]
Chr13	1760375	T	C	Chr13_17603	T_C	SNP	9621.63	19	5	5	[illegible]
Chr13	1771270	G	A	Chr13_17712	G_A	SNP	10848.63	25	16	15	[illegible]
Chr13	1774272	T	TA	Chr13_17742	T_TA	INDEL	18221.59	28	19	13	[illegible]
Chr13	1774282	C	CA	Chr13_17742	C_CA	INDEL	18476.59	29	19	13	[illegible]
Chr13	1911584	G	GT	Chr13_19115	G_GT	INDEL	16666.59	24	6	11	[illegible]
Chr13	1911584	G	GT	Chr13_19115	G_GT	INDEL	16666.59	24	6	11	[illegible]

Chr13	1911584	G	GT	Chr13_19115	G_GT	INDEL	16666.59	24	6	11
Chr13	1911584	G	GT	Chr13_19115	G_GT	INDEL	16666.59	24	6	11
Chr13	1911584	G	GT	Chr13_19115	G_GT	INDEL	16666.59	24	6	11
Chr13	1911584	G	GT	Chr13_19115	G_GT	INDEL	16666.59	24	6	11
Chr13	1911584	G	GT	Chr13_19115	G_GT	INDEL	16666.59	24	6	11
Chr13	1911584	G	GT	Chr13_19115	G_GT	INDEL	16666.59	24	6	11
Chr13	1911584	G	GT	Chr13_19115	G_GT	INDEL	16666.59	24	6	11
Chr13	1911584	G	GT	Chr13_19115	G_GT	INDEL	16666.59	24	6	11
Chr13	1911584	G	GT	Chr13_19115	G_GT	INDEL	16666.59	24	6	11
Chr13	1911584	G	GT	Chr13_19115	G_GT	INDEL	16666.59	24	6	11
Chr13	1911584	G	GT	Chr13_19115	G_GT	INDEL	16666.59	24	6	11
Chr13	1959227	A	T	Chr13_19592	A_T	SNP	19648.63	35	28	15
Chr13	2064722	C	T	Chr13_20647	C_T	SNP	4782.29	0	0	0
Chr13	2077259	T	C	Chr13_20772	T_C	SNP	5956.29	0	0	0
Chr13	2082755	G	A	Chr13_20827	G_A	SNP	16340.63	29	12	13
Chr13	2194799	AG	A	Chr13_21947	AG_A	INDEL	16672.59	13	20	21
Chr13	2243904	G	T	Chr13_22439	G_T	SNP	18885.63	30	7	20
Chr13	2266043	C	CAT	Chr13_22660	C_CAT	INDEL	3928.24	12	9	15
Chr13	2286446	T	C	Chr13_22864	T_C	SNP	18705.63	30	20	22
Chr13	2286446	T	C	Chr13_22864	T_C	SNP	18705.63	30	20	22
Chr13	2286446	T	C	Chr13_22864	T_C	SNP	18705.63	30	20	22
Chr13	2293933	T	TGTGC	Chr13_22939	T_TGTGC	INDEL	19750.59	34	8	17
Chr13	2293934	A	C	Chr13_22939	A_C	SNP	19797.63	34	8	17
Chr13	2293939	TATCA	T	Chr13_22939	TATCA_T	INDEL	19164.59	37	9	16
Chr13	2363031	CA	C	Chr13_23630	CA_C	INDEL	11092.59	25	14	16
Chr13	2363031	CA	C	Chr13_23630	CA_C	INDEL	11092.59	25	14	16
Chr13	2363031	CA	C	Chr13_23630	CA_C	INDEL	11092.59	25	14	16
Chr13	2363031	CA	C	Chr13_23630	CA_C	INDEL	11092.59	25	14	16
Chr13	2363031	CA	C	Chr13_23630	CA_C	INDEL	11092.59	25	14	16
Chr13	2363031	CA	C	Chr13_23630	CA_C	INDEL	11092.59	25	14	16
Chr13	2369378	T	TA	Chr13_23693	T_TA	INDEL	14369.59	13	7	13
Chr13	2369378	T	TA	Chr13_23693	T_TA	INDEL	14369.59	13	7	13
Chr13	2369378	T	TA	Chr13_23693	T_TA	INDEL	14369.59	13	7	13
Chr13	2369378	T	TA	Chr13_23693	T_TA	INDEL	14369.59	13	7	13
Chr13	2369378	T	TA	Chr13_23693	T_TA	INDEL	14369.59	13	7	13
Chr13	2369378	T	TA	Chr13_23693	T_TA	INDEL	14369.59	13	7	13
Chr13	2369378	T	TA	Chr13_23693	T_TA	INDEL	14369.59	13	7	13
Chr13	2369394	G	A	Chr13_23693	G_A	SNP	13958.63	11	7	11
Chr13	2369394	G	A	Chr13_23693	G_A	SNP	13958.63	11	7	11
Chr13	2369394	G	A	Chr13_23693	G_A	SNP	13958.63	11	7	11
Chr13	2369394	G	A	Chr13_23693	G_A	SNP	13958.63	11	7	11
Chr13	2369394	G	A	Chr13_23693	G_A	SNP	13958.63	11	7	11
Chr13	2369394	G	A	Chr13_23693	G_A	SNP	13958.63	11	7	11
Chr13	2369394	G	A	Chr13_23693	G_A	SNP	13958.63	11	7	11
Chr13	2376070	TTA	T	Chr13_23760	TTA_T	INDEL	3632.24	0	0	0
Chr13	2376070	TTA	T	Chr13_23760	TTA_T	INDEL	3632.24	0	0	0
Chr13	2376070	TTA	T	Chr13_23760	TTA_T	INDEL	3632.24	0	0	0
Chr13	2376070	TTA	T	Chr13_23760	TTA_T	INDEL	3632.24	0	0	0
Chr13	2527472	T	TTA	Chr13_25274	T_TTA	INDEL	2052.5	17	4	10
Chr13	2527472	T	TTA	Chr13_25274	T_TTA	INDEL	2052.5	17	4	10
Chr13	2527472	T	TTA	Chr13_25274	T_TTA	INDEL	2052.5	17	4	10
Chr13	2527472	T	TTA	Chr13_25274	T_TTA	INDEL	2052.5	17	4	10
Chr13	2550792	G	T	Chr13_25507	G_T	SNP	9931.63	14	10	5
Chr13	2550813	G	T	Chr13_25508	G_T	SNP	11352.63	16	11	5
Chr13	2578449	A	AT	Chr13_25784	A_AT	INDEL	15851.59	25	21	15
Chr13	2592568	C	T	Chr13_25925	C_T	SNP	19682.63	22	9	19
Chr13	2754636	C	A	Chr13_27546	C_A	SNP	18900.63	28	19	15
Chr13	2754648	G	T	Chr13_27546	G_T	SNP	19052.63	25	20	15
Chr13	2754654	A	T	Chr13_27546	A_T	SNP	19230.63	21	18	16
Chr13	2754657	C	T	Chr13_27546	C_T	SNP	18693.63	21	18	16
Chr13	2755010	A	AG	Chr13_27550	A_AG	INDEL	18756.59	24	25	17
Chr13	2755201	G	T	Chr13_27552	G_T	SNP	20793.63	26	12	5
Chr13	2906363	G	C	Chr13_29063	G_C	SNP	10706.63	15	8	9
Chr13	2906363	G	C	Chr13_29063	G_C	SNP	10706.63	15	8	9
Chr13	2906363	G	C	Chr13_29063	G_C	SNP	10706.63	15	8	9
Chr13	2906363	G	C	Chr13_29063	G_C	SNP	10706.63	15	8	9
Chr13	2906363	G	C	Chr13_29063	G_C	SNP	10706.63	15	8	9
Chr13	2906363	G	C	Chr13_29063	G_C	SNP	10706.63	15	8	9
Chr13	2906363	G	C	Chr13_29063	G_C	SNP	10706.63	15	8	9
Chr13	2906363	G	C	Chr13_29063	G_C	SNP	10706.63	15	8	9
Chr13	2906396	G	C	Chr13_29063	G_C	SNP	9651.63	11	6	7
Chr13	2906396	G	C	Chr13_29063	G_C	SNP	9651.63	11	6	7
Chr13	2906396	G	C	Chr13_29063	G_C	SNP	9651.63	11	6	7
Chr13	2906396	G	C	Chr13_29063	G_C	SNP	9651.63	11	6	7
Chr13	2906396	G	C	Chr13_29063	G_C	SNP	9651.63	11	6	7
Chr13	2906396	G	C	Chr13_29063	G_C	SNP	9651.63	11	6	7
Chr13	2906396	G	C	Chr13_29063	G_C	SNP	9651.63	11	6	7
Chr13	2906396	G	C	Chr13_29063	G_C	SNP	9651.63	11	6	7
Chr13	2915966	G	T	Chr13_29159	G_T	SNP	14349.63	28	16	10
Chr13	3012394	C	A	Chr13_30123	C_A	SNP	16525.63	32	11	10
Chr13	3012394	C	A	Chr13_30123	C_A	SNP	16525.63	32	11	10
Chr13	3012394	C	A	Chr13_30123	C_A	SNP	16525.63	32	11	10
Chr13	3012457	GA	G	Chr13_30124	GA_G	INDEL	12476.59	27	10	6
Chr13	3012457	GA	G	Chr13_30124	GA_G	INDEL	12476.59	27	10	6
Chr13	3012457	GA	G	Chr13_30124	GA_G	INDEL	12476.59	27	10	6
Chr13	3012462	C	CA	Chr13_30124	C_CA	INDEL	12476.59	27	10	6
Chr13	3012462	C	CA	Chr13_30124	C_CA	INDEL	12476.59	27	10	6
Chr13	3012462	C	CA	Chr13_30124	C_CA	INDEL	12476.59	27	10	6
Chr13	3060015	AAT	A	Chr13_30600	AAT_A	INDEL	2330.93	0	0	0
Chr13	3259936	G	A	Chr13_32599	G_A	SNP	13700.63	21	13	10
Chr13	3259936	G	A	Chr13_32599	G_A	SNP	13700.63	21	13	10
Chr13	3259936	G	A	Chr13_32599	G_A	SNP	13700.63	21	13	10
Chr13	3259936	G	A	Chr13_32599	G_A	SNP	13700.63	21	13	10
Chr13	3259936	G	A	Chr13_32599	G_A	SNP	13700.63	21	13	10
Chr13	3273647	A	T	Chr13_32736	A_T	SNP	9308.63	11	6	8
Chr13	3273744	A	G	Chr13_32737	A_G	SNP	10301.63	17	10	10
Chr13	3298324	G	T	Chr13_32983	G_T	SNP	18576.63	29	14	12
Chr13	3298328	A	G	Chr13_32983	A_G	SNP	18587.63	27	15	12
Chr13	3328859	C	A	Chr13_33288	C_A	SNP	19253.63	36	16	10
Chr13	3328974	C	A	Chr13_33289	C_A	SNP	19658.63	27	17	20
Chr13	3515902	C	CA	Chr13_35159	C_CA	INDEL	18318.59	29	15	18
Chr13	3515907	AG	A	Chr13_35159	AG_A	INDEL	18579.59	28	14	17
Chr13	3622417	G	T	Chr13_36224	G_T	SNP	18561.63	24	6	14
Chr13	3771752	G	GAT	Chr13_37717	G_GAT	INDEL	3697.24	2	1	0
Chr13	3771752	G	GAT	Chr13_37717	G_GAT	INDEL	3697.24	2	1	0
Chr13	3771752	G	GAT	Chr13_37717	G_GAT	INDEL	3697.24	2	1	0
Chr13	3771752	G	GAT	Chr13_37717	G_GAT	INDEL	3697.24	2	1	0
Chr13	3771752	G	GAT	Chr13_37717	G_GAT	INDEL	3697.24	2	1	0
Chr13	3984434	G	A	Chr13_39844	G_A	SNP	4194.29	0	0	0
Chr13	4062316	AATATATAT	AATATATAT	Chr13_40623	AATATATAT_	INDEL	2890.91	0	0	1
Chr13	4062316	AATATATAT	AATATATAT	Chr13_40623	AATATATAT_	INDEL	2890.91	0	0	1
Chr13	4062316	AATATATAT	AATATATAT	Chr13_40623	AATATATAT_	INDEL	2890.91	0	0	1
Chr13	4062316	AATATATAT	AATATATAT	Chr13_40623	AATATATAT_	INDEL	2890.91	0	0	1
Chr13	4062316	AATATATAT	AATATATAT	Chr13_40623	AATATATAT_	INDEL	2890.91	0	0	1
Chr13	4064924	G	GT	Chr13_40649	G_GT	INDEL	15167.59	28	20	15
Chr13	4064924	G	GT	Chr13_40649	G_GT	INDEL	15167.59	28	20	15
Chr13	4064924	G	GT	Chr13_40649	G_GT	INDEL	15167.59	28	20	15
Chr13	4064924	G	GT	Chr13_40649	G_GT	INDEL	15167.59	28	20	15
Chr13	4064924	G	GT	Chr13_40649	G_GT	INDEL	15167.59	28	20	15
Chr13	4163297	T	TA	Chr13_41632	T_TA	INDEL	13885.59	23	22	11
Chr13	4183883	T	A	Chr13_41838	T_A	SNP	14519.63	30	12	13
Chr13	4183883	T	A	Chr13_41838	T_A	SNP	14519.63	30	12	13
Chr13	4224715	T	TA	Chr13_42247	T_TA	INDEL	15134.59	36	14	14
Chr13	4271101	A	AAT	Chr13_42711	A_AAT	INDEL	1138.35	18	13	9
Chr13	4271101	A	AAT	Chr13_42711	A_AAT	INDEL	1138.35	18	13	9
Chr13	4271101	A	AAT	Chr13_42711	A_AAT	INDEL	1138.35	18	13	9
Chr13	4271101	A	AAT	Chr13_42711	A_AAT	INDEL	1138.35	18	13	9
Chr13	4271101	A	AAT	Chr13_42711	A_AAT	INDEL	1138.35	18	13	9
Chr13	4271101	A	AAT	Chr13_42711	A_AAT	INDEL	1138.35	18	13	9
Chr13	4271101	A	AAT	Chr13_42711	A_AAT	INDEL	1138.35	18	13	9
Chr13	4271101	A	AAT	Chr13_42711	A_AAT	INDEL	1138.35	18	13	9
Chr13	4271101	A	AAT	Chr13_42711	A_AAT	INDEL	1138.35	18	13	9
Chr13	4271101	A	AAT	Chr13_42711	A_AAT	INDEL	1138.35	18	13	9
Chr13	4271101	A	AAT	Chr13_42711	A_AAT	INDEL	1138.35	18	13	9
Chr13	4271101	A	AAT	Chr13_42711	A_AAT	INDEL	1138.35	18	13	9
Chr13	4445259	T	C	Chr13_44452	T_C	SNP	15843.63	24	14	12
Chr13	4445259	T	C	Chr13_44452	T_C	SNP	15843.63	24	14	12
Chr13	4483822	C	T	Chr13_44838	C_T	SNP	19496.63	40	22	16
Chr13	4599828	T	A	Chr13_45998	T_A	SNP	9335.63	6	9	8
Chr13	4599837	G	T	Chr13_45998	G_T	SNP	10460.63	7	9	10
Chr13	4599853	G	T	Chr13_45998	G_T	SNP	13132.63	12	12	15
Chr13	4599857	T	G	Chr13_45998	T_G	SNP	13124.63	13	12	15
Chr13	4800038	C	T	Chr13_48000	C_T	SNP	20019.63	27	20	20
Chr13	5131021	A	ATTTAGG	Chr13_51310	A_ATTTAGG	INDEL	16613.59	17	8	18
Chr13	5131027	G	GAGATTGTT	Chr13_51310	G_GAGATTG	INDEL	17935.59	17	8	19
Chr13	5131031	T	TTTGATTTAG	Chr13_51310	T_TTTGATTT	INDEL	17766.59	18	8	17
Chr13	5388755	T	A	Chr13_53887	T_A	SNP	16972.63	25	15	9
Chr13	5398798	G	A	Chr13_53987	G_A	SNP	18716.63	30	16	20
Chr13	5476837	T	TGA	Chr13_54768	T_TGA	INDEL	20779.59	27	17	13
Chr13	5476837	T	TGA	Chr13_54768	T_TGA	INDEL	20779.59	27	17	13
Chr13	5476838	TCC	T	Chr13_54768	TCC_T	INDEL	20779.59	28	16	13
Chr13	5476838	TCC	T	Chr13_54768	TCC_T	INDEL	20779.59	28	16	13
Chr13	5636893	C	CTA	Chr13_56368	C_CTA	INDEL	3699.24	4	1	1
Chr13	5636893	C	CTA	Chr13_56368	C_CTA	INDEL	3699.24	4	1	1
Chr13	5636893	C	CTA	Chr13_56368	C_CTA	INDEL	3699.24	4	1	1
Chr13	5636893	C	CTA	Chr13_56368	C_CTA	INDEL	3699.24	4	1	1
Chr13	5636893	C	CTA	Chr13_56368	C_CTA	INDEL	3699.24	4	1	1
Chr13	5636893	C	CTA	Chr13_56368	C_CTA	INDEL	3699.24	4	1	1
Chr13	5766194	AATATATAT	A	Chr13_57661	AATATATAT	INDEL	5339.93	14	6	7
Chr13	5766233	ATATATATA	A	Chr13_57662	ATATATATA	INDEL	2453.93	0	0	0
Chr13	5767981	AAT	AATAT	Chr13_57679	AAT_AATAT	INDEL	2680.5	17	6	14
Chr13	5907244	A	T	Chr13_59072	A_T	SNP	10867.63	18	6	11
Chr13	6124868	A	T	Chr13_61248	A_T	SNP	13309.63	21	6	9
Chr13	6124868	A	T	Chr13_61248	A_T	SNP	13309.63	21	6	9
Chr13	6181672	G	T	Chr13_61816	G_T	SNP	16013.63	27	15	9
Chr13	6498683	A	AAT	Chr13_64986	A_AAT	INDEL	3497.93	0	0	0
Chr13	6498683	A	AAT	Chr13_64986	A_AAT	INDEL	3497.93	0	0	0
Chr13	6498683	A	AAT	Chr13_64986	A_AAT	INDEL	3497.93	0	0	0
Chr13	6546060	T	A	Chr13_65460	T_A	SNP	13889.63	23	20	10
Chr13	6549838	G	T	Chr13_65498	G_T	SNP	19203.63	34	29	36
Chr13	6558423	A	G	Chr13_65584	A_G	SNP	18118.63	19	11	16
Chr13	6558446	G	GA	Chr13_65584	G_GA	INDEL	18429.59	20	12	15
Chr13	6562413	C	G	Chr13_65624	C_G	SNP	14779.63	28	16	8
Chr13	6562416	C	T	Chr13_65624	C_T	SNP	14779.63	28	16	8
Chr13	6563288	G	T	Chr13_65632	G_T	SNP	16189.63	27	13	21
Chr13	6563318	C	T	Chr13_65633	C_T	SNP	16226.63	29	14	21
Chr13	6564004	G	A	Chr13_65640	G_A	SNP	7868.63	9	4	8
Chr13	6566614	G	A	Chr13_65666	G_A	SNP	12109.63	13	8	11
Chr13	6566615	A	T	Chr13_65666	A_T	SNP	12109.63	13	7	11
Chr13	6566644	A	G	Chr13_65666	A_G	SNP	7947.63	12	5	6
Chr13	6566873	T	G	Chr13_65668	T_G	SNP	8833.63	14	7	5
Chr13	6568546	A	T	Chr13_65685	A_T	SNP	6737.63	13	4	4
Chr13	6568687	A	G	Chr13_65686	A_G	SNP	11323.63	13	9	11
Chr13	6568697	T	G	Chr13_65686	T_G	SNP	11579.63	14	9	11
Chr13	6592679	T	A	Chr13_65926	T_A	SNP	20952.63	31	15	18
Chr13	6593938	C	G	Chr13_65939	C_G	SNP	13834.63	15	10	9
Chr13	6593947	A	G	Chr13_65939	A_G	SNP	13320.63	14	9	9
Chr13	6594587	T	C	Chr13_65945	T_C	SNP	12115.63	11	11	12
Chr13	6674441	A	T	Chr13_66744	A_T	SNP	18282.63	42	16	10
Chr13	6674468	C	A	Chr13_66744	C_A	SNP	17049.63	38	15	10
Chr13	6733381	C	CA	Chr13_67333	C_CA	INDEL	15693.59	22	11	15
Chr13	6782214	C	T	Chr13_67822	C_T	SNP	10364.29	24	20	14
Chr13	6825574	C	T	Chr13_68255	C_T	SNP	2287.98	0	0	0
Chr13	6825574	C	T	Chr13_68255	C_T	SNP	2287.98	0	0	0
Chr13	6825574	C	T	Chr13_68255	C_T	SNP	2287.98	0	0	0
Chr13	6825574	C	T	Chr13_68255	C_T	SNP	2287.98	0	0	0
Chr13	6888514	G	C	Chr13_68885	G_C	SNP	20344.63	30	16	15
Chr13	6888514	G	C	Chr13_68885	G_C	SNP	20344.63	30	16	15
Chr13	6888514	G	C	Chr13_68885	G_C	SNP	20344.63	30	16	15
Chr13	6948176	G	GC	Chr13_69481	G_GC	INDEL	14802.59	23	13	11
Chr13	6964430	G	T	Chr13_69644	G_T	SNP	5497.29	0	0	0
Chr13	7026409	T	TA	Chr13_70264	T_TA	INDEL	14770.59	24	12	9

Chr13	7117733	C	T	Chr13_71177C_T	SNP	5270.98	0	0	0	1	0	0
Chr13	7348218	C	T	Chr13_73482C_T	SNP	11669.63	17	9	8	17	6	18
Chr13	7351119	G	A	Chr13_73511G_A	SNP	12472.63	23	10	6	21	16	23
Chr13	7414336	T	C	Chr13_74143T_C	SNP	13583.63	19	11	14	15	19	13
Chr13	7414336	T	C	Chr13_74143T_C	SNP	13583.63	19	11	14	15	19	13
Chr13	7414336	T	C	Chr13_74143T_C	SNP	13583.63	19	11	14	15	19	13
Chr13	7414336	T	C	Chr13_74143T_C	SNP	13583.63	19	11	14	15	19	13
Chr13	7414336	T	C	Chr13_74143T_C	SNP	13583.63	19	11	14	15	19	13
Chr13	7630415	T	TA	Chr13_76304T_TA	INDEL	15603.59	23	11	19	23	17	24
Chr13	7652752	C	T	Chr13_76527C_T	SNP	5561.29	0	0	0	19	20	20
Chr13	7901804	A	T	Chr13_79018A_T	SNP	4896.98	0	0	0	0	0	0
Chr13	7915703	A	G	Chr13_79157A_G	SNP	15856.63	23	13	12	33	12	25
Chr13	8429113	T	C	Chr13_84291T_C	SNP	3701.98	0	0	0	0	0	0
Chr13	8429113	T	C	Chr13_84291T_C	SNP	3701.98	0	0	0	0	0	0
Chr13	8429113	T	C	Chr13_84291T_C	SNP	3701.98	0	0	0	0	0	0
Chr13	8429113	T	C	Chr13_84291T_C	SNP	3701.98	0	0	0	0	0	0
Chr13	8429113	T	C	Chr13_84291T_C	SNP	3701.98	0	0	0	0	0	0
Chr13	8429113	T	C	Chr13_84291T_C	SNP	3701.98	0	0	0	0	0	0
Chr13	8429113	T	C	Chr13_84291T_C	SNP	3701.98	0	0	0	0	0	0
Chr13	8429113	T	C	Chr13_84291T_C	SNP	3701.98	0	0	0	0	0	0
Chr13	8429113	T	C	Chr13_84291T_C	SNP	3701.98	0	0	0	0	0	0
Chr13	8429113	T	C	Chr13_84291T_C	SNP	3701.98	0	0	0	0	0	0
Chr13	8472767	T	C	Chr13_84727T_C	SNP	18449.63	22	18	16	21	21	30

Chr13	10766703	C	T	Chr13_10766C_T	SNP	6317.29	0	0	0	19	20	27	38	30	0	0	0	0	0	0	40	17	15	19	20	27	38
Chr13	10814986	G	GTA	Chr13_10814G_GTA	INDEL	2092.93	1	0	0	1	0	1	3	1	18	14	4	12	4	8	22	8	14	16	17	22	36
Chr13	10814986	G	GTA	Chr13_10814G_GTA	INDEL	2092.93	1	0	0	1	0	1	3	1	18	14	4	12	4	8	22	8	14	16	17	22	36
Chr13	10814986	G	GTA	Chr13_10814G_GTA	INDEL	2092.93	1	0	0	1	0	1	3	1	18	14	4	12	4	8	22	8	14	16	17	22	36
Chr13	10814986	G	GTA	Chr13_10814G_GTA	INDEL	2092.93	1	0	0	1	0	1	3	1	18	14	4	12	4	8	22	8	14	16	17	22	36
Chr13	10814986	G	GTA	Chr13_10814G_GTA	INDEL	2092.93	1	0	0	1	0	1	3	1	18	14	4	12	4	8	22	8	14	16	17	22	36
Chr13	10876831	A	AAT	Chr13_10876A_AAT	INDEL	1264.35	23	11	6	0	0	1	0	0	0	0	0	2	0	0	23	11	6	21	16	21	41
Chr13	10876831	A	AAT	Chr13_10876A_AAT	INDEL	1264.35	23	11	6	0	0	1	0	0	0	0	0	2	0	0	23	11	6	21	16	21	41
Chr13	10876831	A	AAT	Chr13_10876A_AAT	INDEL	1264.35	23	11	6	0	0	1	0	0	0	0	0	2	0	0	23	11	6	21	16	21	41
Chr13	10876831	A	AAT	Chr13_10876A_AAT	INDEL	1264.35	23	11	6	0	0	1	0	0	0	0	0	2	0	0	23	11	6	21	16	21	41
Chr13	10916344	AAT	A	Chr13_10916AAT_A	INDEL	9357.59	21	7	13	14	14	20	34	24	12	9	13	7	8	8	22	7	13	14	14	20	35
Chr13	10916344	AAT	A	Chr13_10916AAT_A	INDEL	9357.59	21	7	13	14	14	20	34	24	12	9	13	7	8	8	22	7	13	14	14	20	35
Chr13	10916344	AAT	A	Chr13_10916AAT_A	INDEL	9357.59	21	7	13	14	14	20	34	24	12	9	13	7	8	8	22	7	13	14	14	20	35
Chr13	10916344	AAT	A	Chr13_10916AAT_A	INDEL	9357.59	21	7	13	14	14	20	34	24	12	9	13	7	8	8	22	7	13	14	14	20	35
Chr13	10916344	AAT	A	Chr13_10916AAT_A	INDEL	9357.59	21	7	13	14	14	20	34	24	12	9	13	7	8	8	22	7	13	14	14	20	35
Chr13	10990102	GTA	G	Chr13_10990GTA_G	INDEL	1595.5	1	0	0	1	0	1	0	1	16	8	10	7	4	4	26	10	10	21	13	14	26
Chr13	10990102	GTA	G	Chr13_10990GTA_G	INDEL	1595.5	1	0	0	1	0	1	0	1	16	8	10	7	4	4	26	10	10	21	13	14	26
Chr13	10990102	GTA	G	Chr13_10990GTA_G	INDEL	1595.5	1	0	0	1	0	1	0	1	16	8	10	7	4	4	26	10	10	21	13	14	26
Chr13	10990504	C	A	Chr13_10990C_A	SNP	20247.63	42	12	11	19	17	26	28	31	19	33	19	7	14	15	42	12	11	19	17	26	28
Chr13	10990504	C	A	Chr13_10990C_A	SNP	20247.63	42	12	11	19	17	26	28	31	19	33	19	7	14	15	42	12	11	19	17	26	28
Chr13	10990504	C	A	Chr13_10990C_A	SNP	20247.63	42	12	11	19	17	26	28	31	19	33	19	7	14	15	42	12	11	19	17	26	28
Chr13	11023996	A	T	Chr13_11023A_T	SNP	15465.63	25	11	17	15	22	30	28	27	24	27	7	6	7	12	25	11	17	15	22	30	28
Chr13	11030984	A	G	Chr13_11030A_G	SNP	16200.63	20	13	12	24	24	25	24	18	16	22	9	17	9	11	20	13	12	24	24	25	24
Chr13	11039034	AT	A	Chr13_11039AT_A	INDEL	20267.59	33	14	16	24	22	29	17	25	20	25	14	11	8	12	33	14	16	24	22	29	17
Chr13	11039039	T	TA	Chr13_11039T_TA	INDEL	20544.59	35	14	16	24	22	32	18	25	20	25	14	11	8	12	35	14	16	24	22	32	18
Chr13	11039087	A	T	Chr13_11039A_T	SNP	18847.63	32	16	16	21	15	29	13	27	25	24	18	18	6	10	32	16	16	21	15	29	13
Chr13	11041082	A	AT	Chr13_11041A_AT	INDEL	23508.59	36	23	11	37	21	29	28	24	24	18	15	18	12	11	36	23	11	37	21	29	28
Chr13	11041096	AG	A	Chr13_11041AG_A	INDEL	23322.59	38	23	13	39	18	30	25	24	24	18	19	16	11	13	38	23	13	39	18	30	25
Chr13	11168227	C	G	Chr13_11168C_G	SNP	19616.63	30	14	15	23	30	27	34	29	10	21	16	11	8	9	30	14	15	23	30	27	34
Chr13	11168227	C	G	Chr13_11168C_G	SNP	19616.63	30	14	15	23	30	27	34	29	10	21	16	11	8	9	30	14	15	23	30	27	34
Chr13	11168227	C	G	Chr13_11168C_G	SNP	19616.63	30	14	15	23	30	27	34	29	10	21	16	11	8	9	30	14	15	23	30	27	34
Chr13	11168227	C	G	Chr13_11168C_G	SNP	19616.63	30	14	15	23	30	27	34	29	10	21	16	11	8	9	30	14	15	23	30	27	34
Chr13	11168227	C	G	Chr13_11168C_G	SNP	19616.63	30	14	15	23	30	27	34	29	10	21	16	11	8	9	30	14	15	23	30	27	34
Chr13	11168227	C	G	Chr13_11168C_G	SNP	19616.63	30	14	15	23	30	27	34	29	10	21	16	11	8	9	30	14	15	23	30	27	34
Chr13	11168228	T	TG	Chr13_11168T_TG	INDEL	19607.59	30	13	15	23	31	28	34	29	11	20	17	11	8	9	30	13	15	23	31	28	34
Chr13	11168228	T	TG	Chr13_11168T_TG	INDEL	19607.59	30	13	15	23	31	28	34	29	11	20	17	11	8	9	30	13	15	23	31	28	34
Chr13	11168228	T	TG	Chr13_11168T_TG	INDEL	19607.59	30	13	15	23	31	28	34	29	11	20	17	11	8	9	30	13	15	23	31	28	34
Chr13	11168228	T	TG	Chr13_11168T_TG	INDEL	19607.59	30	13	15	23	31	28	34	29	11	20	17	11	8	9	30	13	15	23	31	28	34
Chr13	11168228	T	TG	Chr13_11168T_TG	INDEL	19607.59	30	13	15	23	31	28	34	29	11	20	17	11	8	9	30	13	15	23	31	28	34
Chr13	11218950	G	C	Chr13_11218G_C	SNP	10930.63	14	12	6	19	9	14	13	16	20	10	9	9	12	8	14	12	6	20	10	14	13
Chr13	11218950	G	C	Chr13_11218G_C	SNP	10930.63	14	12	6	19	9	14	13	16	20	10	9	9	12	8	14	12	6	20	10	14	13
Chr13	11218950	G	C	Chr13_11218G_C	SNP	10930.63	14	12	6	19	9	14	13	16	20	10	9	9	12	8	14	12	6	20	10	14	13
Chr13	11218950	G	C	Chr13_11218G_C	SNP	10930.63	14	12	6	19	9	14	13	16	20	10	9	9	12	8	14	12	6	20	10	14	13
Chr13	11218950	G	C	Chr13_11218G_C	SNP	10930.63	14	12	6	19	9	14	13	16	20	10	9	9	12	8	14	12	6	20	10	14	13
Chr13	11218950	G	C	Chr13_11218G_C	SNP	10930.63	14	12	6	19	9	14	13	16	20	10	9	9	12	8	14	12	6	20	10	14	13
Chr13	11218950	G	C	Chr13_11218G_C	SNP	10930.63	14	12	6	19	9	14	13	16	20	10	9	9	12	8	14	12	6	20	10	14	13
Chr13	11218954	G	GAGGTGTCG	Chr13_11218G_GAGGTG	INDEL	10942.59	14	12	6	19	9	14	13	16	20	10	9	9	12	8	14	12	6	20	10	14	13
Chr13	11218954	G	GAGGTGTCG	Chr13_11218G_GAGGTG	INDEL	10942.59	14	12	6	19	9	14	13	16	20	10	9	9	12	8	14	12	6	20	10	14	13
Chr13	11218954	G	GAGGTGTCG	Chr13_11218G_GAGGTG	INDEL	10942.59	14	12	6	19	9	14	13	16	20	10	9	9	12	8	14	12	6	20	10	14	13
Chr13	11218954	G	GAGGTGTCG	Chr13_11218G_GAGGTG	INDEL	10942.59	14	12	6	19	9	14	13	16	20	10	9	9	12	8	14	12	6	20	10	14	13

Chr13	12814097	C	G	Chr13_12814C_G	SNP	19519.63	23	22	18	22	16	23	30	17	32	24	24	12	12	7	23	22	18	22	16	23	30	17	32	24	24	12	12	7
Chr13	12814097	C	G	Chr13_12814C_G	SNP	19519.63	23	22	18	22	16	23	30	17	32	24	24	12	12	7	23	22	18	22	16	23	30	17	32	24	24	12	12	7
Chr13	12814097	C	G	Chr13_12814C_G	SNP	19519.63	23	22	18	22	16	23	30	17	32	24	24	12	12	7	23	22	18	22	16	23	30	17	32	24	24	12	12	7
Chr13	12814097	C	G	Chr13_12814C_G	SNP	19519.63	23	22	18	22	16	23	30	17	32	24	24	12	12	7	23	22	18	22	16	23	30	17	32	24	24	12	12	7
Chr13	12814097	C	G	Chr13_12814C_G	SNP	19519.63	23	22	18	22	16	23	30	17	32	24	24	12	12	7	23	22	18	22	16	23	30	17	32	24	24	12	12	7
Chr13	12814097	C	G	Chr13_12814C_G	SNP	19519.63	23	22	18	22	16	23	30	17	32	24	24	12	12	7	23	22	18	22	16	23	30	17	32	24	24	12	12	7
Chr13	12814097	C	G	Chr13_12814C_G	SNP	19519.63	23	22	18	22	16	23	30	17	32	24	24	12	12	7	23	22	18	22	16	23	30	17	32	24	24	12	12	7
Chr13	12814097	C	G	Chr13_12814C_G	SNP	19519.63	23	22	18	22	16	23	30	17	32	24	24	12	12	7	23	22	18	22	16	23	30	17	32	24	24	12	12	7
Chr13	12814097	C	G	Chr13_12814C_G	SNP	19519.63	23	22	18	22	16	23	30	17	32	24	24	12	12	7	23	22	18	22	16	23	30	17	32	24	24	12	12	7
Chr13	12814098	C	A	Chr13_12814C_A	SNP	19339.63	22	22	18	22	16	22	30	17	32	24	25	12	11	7	22	22	18	22	16	22	30	17	32	24	25	12	11	7
Chr13	12814098	C	A	Chr13_12814C_A	SNP	19339.63	22	22	18	22	16	22	30	17	32	24	25	12	11	7	22	22	18	22	16	22	30	17	32	24	25	12	11	7
Chr13	12814098	C	A	Chr13_12814C_A	SNP	19339.63	22	22	18	22	16	22	30	17	32	24	25	12	11	7	22	22	18	22	16	22	30	17	32	24	25	12	11	7
Chr13	12814098	C	A	Chr13_12814C_A	SNP	19339.63	22	22	18	22	16	22	30	17	32	24	25	12	11	7	22	22	18	22	16	22	30	17	32	24	25	12	11	7
Chr13	12814098	C	A	Chr13_12814C_A	SNP	19339.63	22	22	18	22	16	22	30	17	32	24	25	12	11	7	22	22	18	22	16	22	30	17	32	24	25	12	11	7
Chr13	12814098	C	A	Chr13_12814C_A	SNP	19339.63	22	22	18	22	16	22	30	17	32	24	25	12	11	7	22	22	18	22	16	22	30	17	32	24	25	12	11	7
Chr13	12814098	C	A	Chr13_12814C_A	SNP	19339.63	22	22	18	22	16	22	30	17	32	24	25	12	11	7	22	22	18	22	16	22	30	17	32	24	25	12	11	7
Chr13	12814098	C	A	Chr13_12814C_A	SNP	19339.63	22	22	18	22	16	22	30	17	32	24	25	12	11	7	22	22	18	22	16	22	30	17	32	24	25	12	11	7
Chr13	12814098	C	A	Chr13_12814C_A	SNP	19339.63	22	22	18	22	16	22	30	17	32	24	25	12	11	7	22	22	18	22	16	22	30	17	32	24	25	12	11	7
Chr13	12814098	C	A	Chr13_12814C_A	SNP	19339.63	22	22	18	22	16	22	30	17	32	24	25	12	11	7	22	22	18	22	16	22	30	17	32	24	25	12	11	7
Chr13	12814098	C	A	Chr13_12814C_A	SNP	19339.63	22	22	18	22	16	22	30	17	32	24	25	12	11	7	22	22	18	22	16	22	30	17	32	24	25	12	11	7
Chr13	13089514	G	GT	Chr13_13089G_GT	INDEL	14321.59	29	11	10	22	17	26	19	19	8	16	10	14	9	6	29	11	10	22	17	26	19	19	8	16	10	14	9	6
Chr13	13188354	T	TA	Chr13_13188T_TA	INDEL	20959.59	24	21	12	31	30	23	24	31	17	28	10	16	12	16	24	21	12	31	30	23	24	31	17	28	10	16	12	16
Chr13	13188365	A	T	Chr13_13188A_T	SNP	21690.63	25	21	15	32	28	21	27	34	19	28	10	16	13	15	25	21	15	32	28	21	27	34	19	29	10	16	13	15
Chr13	13245094	C	CA	Chr13_13245C_CA	INDEL	8716.59	15	12	5	16	9	9	28	8	10	10	9	8	6	6	15	12	5	16	9	9	28	8	10	10	9	8	6	6
Chr13	13358044	A	AAT	Chr13_13358A_AAT	INDEL	3055.24	0	0	0	12	10	20	28	14	0	0	0	1	0	0	18	13	15	12	10	21	29	14	15	16	26	14	6	8
Chr13	13502095	T	A	Chr13_13502T_A	SNP	19088.63	23	10	23	20	20	37	24	22	18	31	15	19	11	9	23	10	23	20	20	37	24	22	18	31	15	20	11	9
Chr13	13660080	G	A	Chr13_13660G_A	SNP	14631.63	15	6	11	25	20	11	27	20	22	13	10	15	10	8	15	6	11	25	20	11	27	20	22	13	10	15	10	8
Chr13	13660119	G	T	Chr13_13660G_T	SNP	17179.63	20	9	13	26	18	12	26	29	18	20	12	19	9	11	20	9	13	26	18	12	26	29	18	20	12	19	9	11
Chr13	13692441	C	CAA	Chr13_13692C_CAA	INDEL	19563.59	25	6	10	22	28	29	23	22	32	23	13	19	12	11	25	6	10	22	28	29	23	22	32	23	13	19	12	11
Chr13	13692442	CT	C	Chr13_13692CT_C	INDEL	19886.59	25	5	11	22	30	29	22	22	33	24	15	19	12	12	25	5	11	22	30	29	22	22	33	24	15	19	12	12
Chr13	13692444	T	G	Chr13_13692T_G	SNP	19878.63	25	5	11	22	30	30	22	22	33	25	15	20	12	13	25	5	11	22	30	30	22	22	33	25	15	20	12	13
Chr13	13692447	C	A	Chr13_13692C_A	SNP	20033.63	25	5	12	23	30	30	26	21	33	25	15	21	12	13	25	5	12	23	30	30	26	21	33	25	15	21	12	14
Chr13	13715421	G	C	Chr13_13715G_C	SNP	2669.39	33	18	13	0	0	0	0	0	0	0	0	0	0	0	33	18	13	20	18	30	21	38	25	26	22	23	12	27
Chr13	13732599	C	T	Chr13_13732C_T	SNP	6231.29	0	0	0	20	14	26	27	30	0	0	0	0	0	0	25	12	12	20	14	26	27	30	32	26	20	22	9	11
Chr13	13757110	G	A	Chr13_13757G_A	SNP	1414.39	15	10	10	0	0	0	0	0	0	1	0	0	0	0	15	10	10	21	18	22	37	23	25	16	12	31	9	10
Chr13	13758044	C	T	Chr13_13758C_T	SNP	9611.63	20	17	5	16	12	14	24	15	9	7	7	5	6	5	20	17	5	16	12	14	24	15	9	7	7	5	6	5
Chr13	13758091	G	A	Chr13_13758G_A	SNP	10630.63	22	19	7	18	11	14	23	12	12	8	11	7	7	6	22	19	7	18	11	14	23	12	12	8	11	7	7	6

Chr13	16494094	T	TTA	Chr13_16494T_TTA	INDEL	2666.93	0	0	0	1	1	0	1	0	13	19	9	14	7	8	25	10	9	17	12	21	21	15	14	19	9	14	7	8
Chr13	16495271	A	T	Chr13_16495A_T	SNP	4480.29	0	0	0	21	20	25	20	18	0	0	0	0	0	0	33	9	13	23	20	25	20	18	19	25	9	19	8	13
Chr13	16495271	A	T	Chr13_16495A_T	SNP	4480.29	0	0	0	21	20	25	20	18	0	0	0	0	0	0	33	9	13	23	20	25	20	18	19	25	9	19	8	13
Chr13	16495271	A	T	Chr13_16495A_T	SNP	4480.29	0	0	0	21	20	25	20	18	0	0	0	0	0	0	33	9	13	23	20	25	20	18	19	25	9	19	8	13
Chr13	16495271	A	T	Chr13_16495A_T	SNP	4480.29	0	0	0	21	20	25	20	18	0	0	0	0	0	0	33	9	13	23	20	25	20	18	19	25	9	19	8	13
Chr13	16495271	A	T	Chr13_16495A_T	SNP	4480.29	0	0	0	21	20	25	20	18	0	0	0	0	0	0	33	9	13	23	20	25	20	18	19	25	9	19	8	13
Chr13	16695650	G	A	Chr13_16695G_A	SNP	15825.63	9	20	11	29	13	19	29	16	15	26	11	14	4	4	9	20	11	29	13	19	29	16	15	26	11	14	4	4
Chr13	16695650	G	A	Chr13_16695G_A	SNP	15825.63	9	20	11	29	13	19	29	16	15	26	11	14	4	4	9	20	11	29	13	19	29	16	15	26	11	14	4	4
Chr13	16716046	GC	G	Chr13_16716GC_G	INDEL	4675.24	0	0	0	23	16	14	31	16	0	0	0	0	0	0	24	10	12	23	16	14	31	16	17	19	11	18	16	9
Chr13	16744346	A	G	Chr13_16744A_G	SNP	18663.63	22	15	25	31	23	28	18	25	21	23	16	9	12	11	22	15	25	31	23	28	18	25	21	23	16	9	12	11
Chr13	16921465	AAT	A	Chr13_16921AAT_A	INDEL	346.37	0	10	0	0	0	0	1	0	0	0	0	0	1	0	17	10	10	23	15	25	16	14	13	6	10	6	8	7
Chr13	16921465	AAT	A	Chr13_16921AAT_A	INDEL	346.37	0	10	0	0	0	0	1	0	0	0	0	0	1	0	17	10	10	23	15	25	16	14	13	6	10	6	8	7
Chr13	16921465	AAT	A	Chr13_16921AAT_A	INDEL	346.37	0	10	0	0	0	0	1	0	0	0	0	0	1	0	17	10	10	23	15	25	16	14	13	6	10	6	8	7
Chr13	16921465	AAT	A	Chr13_16921AAT_A	INDEL	346.37	0	10	0	0	0	0	1	0	0	0	0	0	1	0	17	10	10	23	15	25	16	14	13	6	10	6	8	7
Chr13	16921465	AAT	A	Chr13_16921AAT_A	INDEL	346.37	0	10	0	0	0	0	1	0	0	0	0	0	1	0	17	10	10	23	15	25	16	14	13	6	10	6	8	7
Chr14	138393	C	T	Chr14_13839C_T	SNP	7033.29	0	0	0	30	17	36	35	20	0	0	0	0	0	0	26	13	20	30	17	36	35	20	23	27	18	20	20	11
Chr14	157890	C	CA	Chr14_15789C_CA	INDEL	12797.59	12	19	11	29	15	16	42	23	22	20	9	14	6	12	12	19	11	29	15	16	42	23	22	20	9	14	6	12
Chr14	218818	T	A	Chr14_21881T_A	SNP	14104.63	17	14	13	20	19	20	19	22	22	12	6	25	11	7	17	14	13	20	19	20	19	22	22	12	6	25	11	7
Chr14	218818	T	A	Chr14_21881T_A	SNP	14104.63	17	14	13	20	19	20	19	22	22	12	6	25	11	7	17	14	13	20	19	20	19	22	22	12	6	25	11	7
Chr14	218818	T	A	Chr14_21881T_A	SNP	14104.63	17	14	13	20	19	20	19	22	22	12	6	25	11	7	17	14	13	20	19	20	19	22	22	12	6	25	11	7
Chr14	218818	T	A	Chr14_21881T_A	SNP	14104.63	17	14	13	20	19	20	19	22	22	12	6	25	11	7	17	14	13	20	19	20	19	22	22	12	6	25	11	7
Chr14	218818	T	A	Chr14_21881T_A	SNP	14104.63	17	14	13	20	19	20	19	22	22	12	6	25	11	7	17	14	13	20	19	20	19	22	22	12	6	25	11	7
Chr14	218818	T	A	Chr14_21881T_A	SNP	14104.63	17	14	13	20	19	20	19	22	22	12	6	25	11	7	17	14	13	20	19	20	19	22	22	12	6	25	11	7
Chr14	219594	C	CAT	Chr14_21959C_CAT	INDEL	4566.24	15	10	8	1	0	1	2	0	14	11	12	11	5	6	15	10	8	14	16	13	23	21	14	12	12	11	5	6
Chr14	219594	C	CAT	Chr14_21959C_CAT	INDEL	4566.24	15	10	8	1	0	1	2	0	14	11	12	11	5	6	15	10	8	14	16	13	23	21	14	12	12	11	5	6
Chr14	219594	C	CAT	Chr14_21959C_CAT	INDEL	4566.24	15	10	8	1	0	1	2	0	14	11	12	11	5	6	15	10	8	14	16	13	23	21	14	12	12	11	5	6
Chr14	219594	C	CAT	Chr14_21959C_CAT	INDEL	4566.24	15	10	8	1	0	1	2	0	14	11	12	11	5	6	15	10	8	14	16	13	23	21	14	12	12	11	5	6
Chr14	219594	C	CAT	Chr14_21959C_CAT	INDEL	4566.24	15	10	8	1	0	1	2	0	14	11	12	11	5	6	15	10	8	14	16	13	23	21	14	12	12	11	5	6
Chr14	219594	C	CAT	Chr14_21959C_CAT	INDEL	4566.24	15	10	8	1	0	1	2	0	14	11	12	11	5	6	15	10	8	14	16	13	23	21	14	12	12	11	5	6
Chr14	448304	A	AAATCAATT(	Chr14_44830A_AAATCAA1	INDEL	19309.59	18	13	17	23	17	21	32	17	17	22	7	11	12	6	18	13	17	23	17	21	32	17	17	22	7	11	12	6
Chr14	658063	AT	A	Chr14_65806AT_A	INDEL	16987.59	14	18	12	22	28	21	33	23	21	23	18	13	7	9	14	18	12	22	28	21	33	23	21	23	18	13	7	9
Chr14	658063	AT	A	Chr14_65806AT_A	INDEL	16987.59	14	18	12	22	28	21	33	23	21	23	18	13	7	9	14	18	12	22	28	21	33	23	21	23	18	13	7	9
Chr14	658063	AT	A	Chr14_65806AT_A	INDEL	16987.59	14	18	12	22	28	21	33	23	21	23	18	13	7	9	14	18	12	22	28	21	33	23	21	23	18	13	7	9
Chr14	658063	AT	A	Chr14_65806AT_A	INDEL	16987.59	14	18	12	22	28	21	33	23	21	23	18	13	7	9	14	18	12	22	28	21	33	23	21	23	18	13	7	9
Chr14	748607	G	C	Chr14_74860G_C	SNP	20315.63	29	16	26	24	19	32	24	28	34	21	14	14	15	15	29	16	26	24	19	32	24	28	34	21	14	14	15	15
Chr14	752387	G	GA	Chr14_75238G_GA	INDEL	16365.59	34	10	15	20	24	26	19	30	19	19	11	13	13	6	34	10	15	20	24	26	19	30	19	19	11	13	13	6
Chr14	824852	A	AAT	Chr14_82485A_AAT	INDEL	99.37	0	0	0	1	0	0	0	0	0	0	0	0	5	0	23	10	14	14	17	23	12	25	14	22	10	7	5	4
Chr14	1084788	G	GA	Chr14_10847G_GA	INDEL	14047.59	13	13	15	18	19	21	28	23	19	16	18	22	5	12	13	13	15	18	19	21	28	23	19	16	18	22	5	12
Chr14	1084788	G	GA	Chr14_10847G_GA	INDEL	14047.59	13	13	15	18	19	21	28	23	19	16	18	22	5	12	13	13	15	18	19	21	28	23	19	16	18	22	5	12
Chr14	1084788	G	GA	Chr14_10847G_GA	INDEL	14047.59	13	13	15	18	19	21	28	23	19	16	18	22	5	12	13	13	15	18	19	21	28	23	19	16	18	22	5	12
Chr14	1084788	G	GA	Chr14_10847G_GA	INDEL	14047.59	13	13	15	18	19	21	28	23	19	16	18	22	5	12	13	13	15	18	19	21	28	23	19	16	18	22	5	12
Chr14	1368669	C	T	Chr14_13686C_T	SNP	18609.63	28	19	17	24	15	16	23	23	25	19	9	13	12	8	28	19	17	24	15	16	23	23	25	19	9	13	12	8
Chr14	1368671	C	T	Chr14_13686C_T	SNP	18703.63	27	19	17	26	15	16	24	23	25	19	9	14	12	9	27	19	17	26	15	16	24	23	25	19	9	14	12	9
Chr14	1368674	C	A	Chr14_13686C_A	SNP	18437.63	27	20	17	26	15	16	23	22	25	19	8	14	12	9	27	20	17	26	15	16	23	22	25	19	8	14	12	9
Chr14	1368708	T	TG	Chr14_13687T_TG	INDEL	13667.59	27	14	17	27	18	18	20	19	24	19	11	14	11	8	27	14	17	27	18	18	20	19	24	19	11	14	11	8
Chr14	1368774	C	CA	Chr14_13687C_CA	INDEL	16803.59	35	17	17	25	22	22	23	22	16	22	13	14	9	11	35	17	17	25	22	22	23	22	16	22	13	14	9	11
Chr14	1409161	A	C	Chr14_14091A_C	SNP	18051.63	24	12	14	24	20	26	23	26	18	14	15	15	12	11	24	12	14	24	20	26	23	26	18	14	15	15	12	11
Chr14	1409161	A	C	Chr14_14091A_C	SNP	18051.63	24	12	14	24	20	26	23	26	18	14	15	15	12	11	24	12	14	24	20	26	23	26	18	14	15	15	12	11
Chr14	1409161	A	C	Chr14_14091A_C	SNP	18051.63	24	12	14	24	20	26	23	26	18	14	15	15	12	11	24	12	14	24	20	26	23	26	18	14	15	15	12	11
Chr14	1409162	G	T	Chr14_14091G_T	SNP	18051.63	24	12	14	24	18	26	23	26	18	14	15	15	12	11	24	12	14	24	18	26	23	26	18	14	15	15	12	11
Chr14	1409162	G	T	Chr14_14091G_T	SNP	18051.63	24	12	14	24	18	26	23	26	18	14	15	15	12	11	24	12	14	24	18	26	23	26	18	14	15	15	12	11
Chr14	1409162	G	T	Chr14_14091G_T	SNP	18051.63	24	12	14	24	18	26	23	26	18	14	15	15	12	11	24	12	14	24	18	26	23	26	18	14	15	15	12	11
Chr14	1451412	G	GA	Chr14_14514G_GA	INDEL	10340.59	11	13	7	13	15	19	8	16	8	12	12	16	9	4	11	13	7	13	15	19	8	16	8	12	12	16	9	4
Chr14	1526017	C	T	Chr14_15260C_T	SNP	1624.39	18	16	7	0	0	0	0	0	0	0	0	0	0	0	18	16	7	24	28	30	31	18	14	28	17	10	8	4
Chr14	1553338	G	T	Chr14_15533G_T	SNP	37290.63	60	32	25	45	42	67	65	39	34	57	30	34	16	25	60	32	25	45	42	67	65	39	34	57	30	34	16	25
Chr14	1639431	T	C	Chr14_16394T_C	SNP	16858.63	21	11	8	20	15	27	38	14	23	13	21	13	4	9	21	11	8	20	15	27	38	14	23	13	21	13	4	9
Chr14	1639431	T	C	Chr14_16394T_C	SNP	16858.63	21	11	8	20	15	27	38	14	23	13	21	13	4	9	21	11	8	20	15	27	38	14	23	13	21	13	4	9
Chr14	1639431	T	C	Chr14_16394T_C	SNP	16858.63	21	11	8	20	15	27	38	14	23	13	21	13	4	9	21	11	8	20	15	27	38	14	23	13	21	13	4	9
Chr14	1639431	T	C	Chr14_16394T_C	SNP	16858.63	21	11	8	20	15	27	38	14	23	13	21	13	4	9	21	11	8	20	15	27	38	14	23	13	21	13	4	9
Chr14	1639431	T	C	Chr14_16394T_C	SNP	16858.63	21	11	8	20	15	27	38	14	23	13	21	13	4	9	21	11	8	20	15	27	38	14	23	13	21	13	4	9
Chr14	1639431	T	C	Chr14_16394T_C	SNP	16858.63	21	11	8	20	15	27	38	14	23	13	21	13	4	9	21	11	8	20	15	27	38	14	23	13	21	13	4	9
Chr14	1639450	T	TA	Chr14_16394T_TA	INDEL	18099.59	22	11	10	20	13	26	39	12	23	14	21	11	4	10	22	11	10	20	13	26	39	12	23	14	21	11	4	10
Chr14	1639450	T	TA	Chr14_16394T_TA	INDEL	18099.59	22	11	10	20	13	26	39	12	23	14	21	11	4	10	22	11	10	20	13	26	39	12	23	14	21	11	4	10

Chr14	4099415	C	CAT	Chr14_40994C_CAT	INDEL	1325.35	24	10	10	0	2	2	1	1	2	0
Chr14	4099415	C	CAT	Chr14_40994C_CAT	INDEL	1325.35	24	10	10	0	2	2	1	1	2	0
Chr14	4229863	T	TTA	Chr14_42298T_TTA	INDEL	2808.5	12	7	12	0	1	0	0	0	0	1
Chr14	4229863	T	TTA	Chr14_42298T_TTA	INDEL	2808.5	12	7	12	0	1	0	0	0	0	1
Chr14	4229863	T	TTA	Chr14_42298T_TTA	INDEL	2808.5	12	7	12	0	1	0	0	0	0	1
Chr14	4229863	T	TTA	Chr14_42298T_TTA	INDEL	2808.5	12	7	12	0	1	0	0	0	0	1
Chr14	4229863	T	TTA	Chr14_42298T_TTA	INDEL	2808.5	12	7	12	0	1	0	0	0	0	1
Chr14	4229863	T	TTA	Chr14_42298T_TTA	INDEL	2808.5	12	7	12	0	1	0	0	0	0	1
Chr14	4229863	T	TTA	Chr14_42298T_TTA	INDEL	2808.5	12	7	12	0	1	0	0	0	0	1
Chr14	4229863	T	TTA	Chr14_42298T_TTA	INDEL	2808.5	12	7	12	0	1	0	0	0	0	1
Chr14	4229863	T	TTA	Chr14_42298T_TTA	INDEL	2808.5	12	7	12	0	1	0	0	0	0	1
Chr14	4229863	T	TTA	Chr14_42298T_TTA	INDEL	2808.5	12	7	12	0	1	0	0	0	0	1
Chr14	4249364	A	AAT	Chr14_42493A_AAT	INDEL	12478.59	23	13	12	15	13	14	15	17	13	19
Chr14	4249364	A	AAT	Chr14_42493A_AAT	INDEL	12478.59	23	13	12	15	13	14	15	17	13	19
Chr14	4249364	A	AAT	Chr14_42493A_AAT	INDEL	12478.59	23	13	12	15	13	14	15	17	13	19
Chr14	4249364	A	AAT	Chr14_42493A_AAT	INDEL	12478.59	23	13	12	15	13	14	15	17	13	19
Chr14	4249364	A	AAT	Chr14_42493A_AAT	INDEL	12478.59	23	13	12	15	13	14	15	17	13	19
Chr14	4249364	A	AAT	Chr14_42493A_AAT	INDEL	12478.59	23	13	12	15	13	14	15	17	13	19
Chr14	4249364	A	AAT	Chr14_42493A_AAT	INDEL	12478.59	23	13	12	15	13	14	15	17	13	19
Chr14	4249364	A	AAT	Chr14_42493A_AAT	INDEL	12478.59	23	13	12	15	13	14	15	17	13	19
Chr14	4361073	C	A	Chr14_43610C_A	SNP	14566.63	21	13	8	20	14	22	23	24	11	19
Chr14	4361081	C	A	Chr14_43610C_A	SNP	14577.63	21	13	8	20	14	22	23	24	11	19
Chr14	4387561	T	C	Chr14_43875T_C	SNP	17271.63	29	16	16	24	21	20	30	14	25	23
Chr14	4552637	T	TTA	Chr14_45526T_TTA	INDEL	9873.59	19	13	18	17	13	20	19	26	15	16
[illegible]																

Chr14	7323520	AAT	A	Chr14_73235	AAT_A	INDEL	331.37	1	12	[illegible]
Chr14	7323520	AAT	A	Chr14_73235	AAT_A	INDEL	331.37	1	12	[illegible]
Chr14	7323520	AAT	A	Chr14_73235	AAT_A	INDEL	331.37	1	12	[illegible]
Chr14	7323520	AAT	A	Chr14_73235	AAT_A	INDEL	331.37	1	12	[illegible]
Chr14	7323520	AAT	A	Chr14_73235	AAT_A	INDEL	331.37	1	12	[illegible]
Chr14	7323520	AAT	A	Chr14_73235	AAT_A	INDEL	331.37	1	12	[illegible]
Chr14	7323520	AAT	A	Chr14_73235	AAT_A	INDEL	331.37	1	12	[illegible]
Chr14	7323520	AAT	A	Chr14_73235	AAT_A	INDEL	331.37	1	12	[illegible]
Chr14	7444867	G	GA	Chr14_74448	G_GA	INDEL	16680.59	27	18	[illegible]
Chr14	7444867	G	GA	Chr14_74448	G_GA	INDEL	16680.59	27	18	[illegible]
Chr14	7444867	G	GA	Chr14_74448	G_GA	INDEL	16680.59	27	18	[illegible]
Chr14	7444867	G	GA	Chr14_74448	G_GA	INDEL	16680.59	27	18	[illegible]
Chr14	7444867	G	GA	Chr14_74448	G_GA	INDEL	16680.59	27	18	[illegible]
Chr14	7444867	G	GA	Chr14_74448	G_GA	INDEL	16680.59	27	18	[illegible]
Chr14	7444867	G	GA	Chr14_74448	G_GA	INDEL	16680.59	27	18	[illegible]
Chr14	7444867	G	GA	Chr14_74448	G_GA	INDEL	16680.59	27	18	[illegible]
Chr14	7444867	G	GA	Chr14_74448	G_GA	INDEL	16680.59	27	18	[illegible]
Chr14	7444867	G	GA	Chr14_74448	G_GA	INDEL	16680.59	27	18	[illegible]
Chr14	7444867	G	GA	Chr14_74448	G_GA	INDEL	16680.59	27	18	[illegible]
Chr14	7444867	G	GA	Chr14_74448	G_GA	INDEL	16680.59	27	18	[illegible]
Chr14	7444867	G	GA	Chr14_74448	G_GA	INDEL	16680.59	27	18	[illegible]
Chr14	7444867	G	GA	Chr14_74448	G_GA	INDEL	16680.59	27	18	[illegible]
Chr14	7507335	G	GAT	Chr14_75073	G_GAT	INDEL	1031.35	16	12	[illegible]
Chr14	7560668	G	T	Chr14_75606	G_T	SNP	13231.63	20	7	[illegible]
Chr14	7560668	G	T	Chr14_75606	G_T	SNP	13231.63	20	7	[illegible]
Chr14	7560668	G	T	Chr14_75606	G_T	SNP	13231.63	20	7	[illegible]
Chr14	7645119	G	GAT	Chr14_76451	G_GAT	INDEL	5467.91	3	0	[illegible]
Chr14	7649747	A	AAT	Chr14_76497	A_AAT	INDEL	1255.35	25	12	[illegible]
Chr14	7649747	A	AAT	Chr14_76497	A_AAT	INDEL	1255.35	25	12	[illegible]
Chr14	7695681	C	A	Chr14_76956	C_A	SNP	13508.63	18	6	[illegible]
Chr14	7695681	C	A	Chr14_76956	C_A	SNP	13508.63	18	6	[illegible]
Chr14	7695681	C	A	Chr14_76956	C_A	SNP	13508.63	18	6	[illegible]
Chr14	7695681	C	A	Chr14_76956	C_A	SNP	13508.63	18	6	[illegible]
Chr14	7695681	C	A	Chr14_76956	C_A	SNP	13508.63	18	6	[illegible]
Chr14	7695681	C	A	Chr14_76956	C_A	SNP	13508.63	18	6	[illegible]
Chr14	7695681	C	A	Chr14_76956	C_A	SNP	13508.63	18	6	[illegible]
Chr14	7695681	C	A	Chr14_76956	C_A	SNP	13508.63	18	6	[illegible]
Chr14	7695681	C	A	Chr14_76956	C_A	SNP	13508.63	18	6	[illegible]
Chr14	7708474	T	TG	Chr14_77084	T_TG	INDEL	17237.59	32	17	[illegible]
Chr14	7708474	T	TG	Chr14_77084	T_TG	INDEL	17237.59	32	17	[illegible]
Chr14	7708474	T	TG	Chr14_77084	T_TG	INDEL	17237.59	32	17	[illegible]
Chr14	7708474	T	TG	Chr14_77084	T_TG	INDEL	17237.59	32	17	[illegible]
Chr14	7708474	T	TG	Chr14_77084	T_TG	INDEL	17237.59	32	17	[illegible]
Chr14	7708474	T	TG	Chr14_77084	T_TG	INDEL	17237.59	32	17	[illegible]
Chr14	7708474	T	TG	Chr14_77084	T_TG	INDEL	17237.59	32	17	[illegible]
Chr14	7708474	T	TG	Chr14_77084	T_TG	INDEL	17237.59	32	17	[illegible]
Chr14	7708474	T	TG	Chr14_77084	T_TG	INDEL	17237.59	32	17	[illegible]
Chr14	7708474	T	TG	Chr14_77084	T_TG	INDEL	17237.59	32	17	[illegible]
Chr14	7810195	G	A	Chr14_78101	G_A	SNP	4126.98	0	0	[illegible]
Chr14	7930453	C	A	Chr14_79304	C_A	SNP	12841.63	10	16	[illegible]
Chr14	7930453	C	A	Chr14_79304	C_A	SNP	12841.63	10	16	[illegible]
Chr14	7930488	C	A	Chr14_79304	C_A	SNP	11912.63	10	10	[illegible]
Chr14	7930488	C	A	Chr14_79304	C_A	SNP	11912.63	10	10	[illegible]
Chr14	7930524	T	A	Chr14_79305	T_A	SNP	11086.63	11	11	[illegible]
Chr14	7930524	T	A	Chr14_79305	T_A	SNP	11086.63	11	11	[illegible]
Chr14	7930524	T	A	Chr14_79305	T_A	SNP	11086.63	11	11	[illegible]
Chr14	7930524	T	A	Chr14_79305	T_A	SNP	11086.63	11	11	[illegible]
Chr14	8018753	AG	A	Chr14_80187	AG_A	INDEL	15037.59	17	8	[illegible]
Chr14	8018779	G	C	Chr14_80187	G_C	SNP	15770.63	19	9	[illegible]
Chr14	8029585	C	A	Chr14_80295	C_A	SNP	19658.63	25	12	[illegible]
Chr14	8073601	TG	T	Chr14_80736	TG_T	INDEL	11882.59	18	8	[illegible]
Chr14	8141465	T	C	Chr14_81414	T_C	SNP	17294.63	37	10	[illegible]
Chr14	8141465	T	C	Chr14_81414	T_C	SNP	17294.63	37	10	[illegible]
Chr14	8141465	T	C	Chr14_81414	T_C	SNP	17294.63	37	10	[illegible]
Chr14	8141465	T	C	Chr14_81414	T_C	SNP	17294.63	37	10	[illegible]
Chr14	8141465	T	C	Chr14_81414	T_C	SNP	17294.63	37	10	[illegible]
Chr14	8141465	T	C	Chr14_81414	T_C	SNP	17294.63	37	10	[illegible]
Chr14	8141465	T	C	Chr14_81414	T_C	SNP	17294.63	37	10	[illegible]
Chr14	8141465	T	C	Chr14_81414	T_C	SNP	17294.63	37	10	[illegible]
Chr14	8141465	T	C	Chr14_81414	T_C	SNP	17294.63	37	10	[illegible]
Chr14	8141465	T	C	Chr14_81414	T_C	SNP	17294.63	37	10	[illegible]
Chr14	8141465	T	C	Chr14_81414	T_C	SNP	17294.63	37	10	[illegible]
Chr14	8141465	T	C	Chr14_81414	T_C	SNP	17294.63	37	10	[illegible]
Chr14	8141465	T	C	Chr14_81414	T_C	SNP	17294.63	37	10	[illegible]
Chr14	8141465	T	C	Chr14_81414	T_C	SNP	17294.63	37	10	[illegible]
Chr14	8141465	T	C	Chr14_81414	T_C	SNP	17294.63	37	10	[illegible]
Chr14	8141465	T	C	Chr14_81414	T_C	SNP	17294.63	37	10	[illegible]
Chr14	8141465	T	C	Chr14_81414	T_C	SNP	17294.63	37	10	[illegible]
Chr14	8141465	T	C	Chr14_81414	T_C	SNP	17294.63	37	10	[illegible]
Chr14	8144325	G	GA	Chr14_81443	G_GA	INDEL	12461.59	20	10	[illegible]
Chr14	8188029	G	GTA	Chr14_81880	G_GTA	INDEL	3317.24	0	0	[illegible]
Chr14	8188029	G	GTA	Chr14_81880	G_GTA	INDEL	3317.24	0	0	[illegible]
Chr14	8189800	C	T	Chr14_81898	C_T	SNP	18269.63	21	12	[illegible]
Chr14	8189800	C	T	Chr14_81898	C_T	SNP	18269.63	21	12	[illegible]
Chr14	8226032	G	C	Chr14_82260	G_C	SNP	15409.63	20	12	[illegible]
Chr14	8226036	G	C	Chr14_82260	G_C	SNP	15483.63	20	13	[illegible]
Chr14	8226388	G	T	Chr14_82263	G_T	SNP	13550.63	19	14	[illegible]
Chr14	8324653	T	TTA	Chr14_83246	T_TTA	INDEL	3697.24	0	0	[illegible]
Chr14	8346654	A	AAT	Chr14_83466	A_AAT	INDEL	3551.24	0	0	[illegible]
Chr14	8416956	C	CTA	Chr14_84169	C_CTA	INDEL	2292.93	1	0	[illegible]
Chr14	8416956	C	CTA	Chr14_84169	C_CTA	INDEL	2292.93	1	0	[illegible]
Chr14	8416956	C	CTA	Chr14_84169	C_CTA	INDEL	2292.93	1	0	[illegible]
Chr14	8416956	C	CTA	Chr14_84169	C_CTA	INDEL	2292.93	1	0	[illegible]
Chr14	8416956	C	CTA	Chr14_84169	C_CTA	INDEL	2292.93	1	0	[illegible]
Chr14	8416956	C	CTA	Chr14_84169	C_CTA	INDEL	2292.93	1	0	[illegible]
Chr14	8416956	C	CTA	Chr14_84169	C_CTA	INDEL	2292.93	1	0	[illegible]
Chr14	8426178	C	T	Chr14_84261	C_T	SNP	7078.29	0	0	[illegible]
Chr14	8426178	C	T	Chr14_84261	C_T	SNP	7078.29	0	0	[illegible]
Chr14	8659654	C	A	Chr14_86596	C_A	SNP	18413.63	33	16	[illegible]
Chr14	8659689	C	A	Chr14_86596	C_A	SNP	18450.63	27	14	[illegible]
Chr14	8786187	C	CA	Chr14_87861	C_CA	INDEL	16163.59	24	19	[illegible]
Chr14	8790474	T	TTA	Chr14_87904	T_TTA	INDEL	11205.59	25	14	[illegible]
Chr14	8790474	T	TTA	Chr14_87904	T_TTA	INDEL	11205.59	25	14	[illegible]
Chr14	8790474	T	TTA	Chr14_87904	T_TTA	INDEL	11205.59	25	14	[illegible]
Chr14	8856908	T	G	Chr14_88569	T_G	SNP	17526.63	28	12	[illegible]
Chr14	8864412	AT	A	Chr14_88644	AT_A	INDEL	14566.59	18	18	[illegible]
Chr14	8868739	T	TAGAG	Chr14_88687	T_TAGAG	INDEL	6678.24	11	8	[illegible]
Chr14	8868739	T	TAGAG	Chr14_88687	T_TAGAG	INDEL	6678.24	11	8	[illegible]
Chr14	8868739	T	TAGAG	Chr14_88687	T_TAGAG	INDEL	6678.24	11	8	[illegible]
Chr14	8868739	T	TAGAG	Chr14_88687	T_TAGAG	INDEL	6678.24	11	8	[illegible]
Chr14	8868739	T	TAGAG	Chr14_88687	T_TAGAG	INDEL	6678.24	11	8	[illegible]
Chr14	8928035	A	AAT	Chr14_89280	A_AAT	INDEL	6004.24	16	10	[illegible]
Chr14	8928035	A	AAT	Chr14_89280	A_AAT	INDEL	6004.24	16	10	[illegible]
Chr14	8928035	A	AAT	Chr14_89280	A_AAT	INDEL	6004.24	16	10	[illegible]
Chr14	8928035	A	AAT	Chr14_89280	A_AAT	INDEL	6004.24	16	10	[illegible]
Chr14	9112648	G	GA	Chr14_91126	G_GA	INDEL	14225.59	27	10	[illegible]
Chr14	9279653	T	TA	Chr14_92796	T_TA	INDEL	16907.59	29	18	[illegible]
Chr14	9567124	C	A	Chr14_95671	C_A	SNP	14381.63	16	17	[illegible]
Chr14	9567124	C	A	Chr14_95671	C_A	SNP	14381.63	16	17	[illegible]
Chr14	9749239	A	AAT	Chr14_97492	A_AAT	INDEL	447.37	0	0	[illegible]
Chr14	9749239	A	AAT	Chr14_97492	A_AAT	INDEL	447.37	0	0	[illegible]
Chr14	9749239	A	AAT	Chr14_97492	A_AAT	INDEL	447.37	0	0	[illegible]
Chr14	9836256	C	A	Chr14_98362	C_A	SNP	14999.63	26	17	[illegible]
Chr14	9836256	C	A	Chr14_98362	C_A	SNP	14999.63	26	17	[illegible]
Chr14	9836256	C	A	Chr14_98362	C_A	SNP	14999.63	26	17	[illegible]
Chr14	9836256	C	A	Chr14_98362	C_A	SNP	14999.63	26	17	[illegible]
Chr14	9836256	C	A	Chr14_98362	C_A	SNP	14999.63	26	17	[illegible]
Chr14	9836256	C	A	Chr14_98362	C_A	SNP	14999.63	26	17	[illegible]
Chr14	9836963	A	AG	Chr14_98369	A_AG	INDEL	17523.59	30	24	[illegible]
Chr14	9836963	A	AG	Chr14_98369	A_AG	INDEL	17523.59	30	24	[illegible]
Chr14	9836963	A	AG	Chr14_98369	A_AG	INDEL	17523.59	30	24	[illegible]
Chr14	9836963	A	AG	Chr14_98369	A_AG	INDEL	17523.59	30	24	[illegible]
Chr14	9836963	A	AG	Chr14_98369	A_AG	INDEL	17523.59	30	24	[illegible]
Chr14	9836963	A	AG	Chr14_98369	A_AG	INDEL	17523.59	30	24	[illegible]
Chr14	9907721	C	A	Chr14_99077	C_A	SNP	18293.63	21	17	[illegible]
Chr14	9907721	C	A	Chr14_99077	C_A	SNP	18293.63	21	17	[illegible]
Chr14	9907721	C	A	Chr14_99077	C_A	SNP	18293.63	21	17	[illegible]
Chr14	9907721	C	A	Chr14_99077	C_A	SNP	18293.63	21	17	[illegible]
Chr14	9907721	C	A	Chr14_99077	C_A	SNP	18293.63	21	17	[illegible]
Chr14	9907721	C	A	Chr14_99077	C_A	SNP	18293.63	21	17	[illegible]
Chr14	9907721	C	A	Chr14_99077	C_A	SNP	18293.63	21	17	[illegible]
Chr14	9907721	C	A	Chr14_99077	C_A	SNP	18293.63	21	17	[illegible]
Chr14	10179658	G	T	Chr14_10179	G_T	SNP	5248.29	0	0	[illegible]
Chr14	10218327	TTA	T	Chr14_10218	TTA_T	INDEL	8196.59	20	10	[illegible]
Chr14	10218327	TTA	T	Chr14_10218	TTA_T	INDEL	8196.59	20	10	[illegible]
Chr14	10218327	TTA	T	Chr14_10218	TTA_T	INDEL	8196.59	20	10	[illegible]
Chr14	10218327	TTA	T	Chr14_10218	TTA_T	INDEL	8196.59	20	10	[illegible]
Chr14	10241971	A	AAT	Chr14_10241	A_AAT	INDEL	70.37	0	0	[illegible]
Chr14	10241971	A	AAT	Chr14_10241	A_AAT	INDEL	70.37	0	0	[illegible]
Chr14	10241971	A	AAT	Chr14_10241	A_AAT	INDEL	70.37	0	0	[illegible]
Chr14	10241971	A	AAT	Chr14_10241	A_AAT	INDEL	70.37	0	0	[illegible]
Chr14	10302171	G	GA	Chr14_10302	G_GA	INDEL	15445.59	29	21	[illegible]
Chr14	10302259	T	A	Chr14_10302	T_A	SNP	17584.63	24	24	[illegible]
Chr14	10306793	G	A	Chr14_10306	G_A	SNP	13162.29	24	22	[illegible]
Chr14	10332965	A	AT	Chr14_10332	A_AT	INDEL	11821.59	19	15	[illegible]
Chr14	10332965	A	AT	Chr14_10332	A_AT	INDEL	11821.59	19	15	[illegible]
Chr14	10332965	A	AT	Chr14_10332	A_AT	INDEL	11821.59	19	15	[illegible]
Chr14	10332965	A	AT	Chr14_10332	A_AT	INDEL	11821.59	19	15	[illegible]
Chr14	10332965	A	AT	Chr14_10332	A_AT	INDEL	11821.59	19	15	[illegible]
Chr14	10332965	A	AT	Chr14_10332	A_AT	INDEL	11821.59	19	15	[illegible]
Chr14	10332965	A	AT	Chr14_10332	A_AT	INDEL	11821.59	19	15	[illegible]
Chr14	10332965	A	AT	Chr14_10332	A_AT	INDEL	11821.59	19	15	[illegible]
Chr14	10332965	A	AT	Chr14_10332	A_AT	INDEL	11821.59	19	15	[illegible]
Chr14	10333014	C	A	Chr14_10333	C_A	SNP	9303.59	16	10	[illegible]
Chr14	10333014	C	A	Chr14_10333	C_A	SNP	9303.59	16	10	[illegible]
Chr14	10333014	C	A	Chr14_10333	C_A	SNP	9303.59	16	10	[illegible]
Chr14	10333014	C	A	Chr14_10333	C_A	SNP	9303.59	16	10	[illegible]
Chr14	10333014	C	A	Chr14_10333	C_A	SNP	9303.59	16	10	[illegible]
Chr14	10333014	C	A	Chr14_10333	C_A	SNP	9303.59	16	10	[illegible]
Chr14	10333014	C	A	Chr14_10333	C_A	SNP	9303.59	16	10	[illegible]
Chr14	10333014	C	A	Chr14_10333	C_A	SNP	9303.59	16	10	[illegible]
Chr14	10333014	C	A	Chr14_10333	C_A	SNP	9303.59	16	10	[illegible]
Chr14	10333047	C	CT	Chr14_10333	C_CT	INDEL	10882.59	20	8	[illegible]
Chr14	10333047	C	CT	Chr14_10333	C_CT	INDEL	10882.59	20	8	[illegible]
Chr14	10333047	C	CT	Chr14_10333	C_CT	INDEL	10882.59	20	8	[illegible]
Chr14	10333047	C	CT	Chr14_10333	C_CT	INDEL	10882.59	20	8	[illegible]
Chr14	10333047	C	CT	Chr14_10333	C_CT	INDEL	10882.59	20	8	[illegible]
Chr14	10333047	C	CT	Chr14_10333	C_CT	INDEL	10882.59	20	8	[illegible]
Chr14	10333047	C	CT	Chr14_10333	C_CT	INDEL	10882.59	20	8	[illegible]
Chr14	10333047	C	CT	Chr14_10333	C_CT	INDEL	10882.59	20	8	[illegible]
Chr14	10333047	C	CT	Chr14_10333	C_CT	INDEL	10882.59	20	8	[illegible]
Chr14	10419041	G	GT	Chr14_10419	G_GT	INDEL	15351.59	31	14	[illegible]
Chr14	10419041	G	GT	Chr14_10419	G_GT	INDEL	15351.59	31	14	[illegible]
Chr14	10419041	G	GT	Chr14_10419	G_GT	INDEL	15351.59	31	14	[illegible]
Chr14	10419041	G	GT	Chr14_10419	G_GT	INDEL	15351.59	31	14	[illegible]
Chr14	10419041	G	GT	Chr14_10419	G_GT	INDEL	15351.59	31	14	[illegible]
Chr14	10575055	T	C	Chr14_10575	T_C	SNP	4104.29	0	0	[illegible]
Chr14	10652625	G	GA	Chr14_10652	G_GA	INDEL	20880.59	26	8	[illegible]
Chr14	10652625	G	GA	Chr14_10652	G_GA	INDEL	20880.59	26	8	[illegible]
Chr14	10652625	G	GA	Chr14_10652	G_GA	INDEL	20880.59	26	8	[illegible]
Chr14	10652625	G	GA	Chr14_10652	G_GA	INDEL	20880.59	26	8	[illegible]
Chr14	10652625	G	GA	Chr14_10652	G_GA	INDEL	20880.59	26	8	[illegible]
Chr14	10652628	T	TA	Chr14_10652	T_TA	INDEL	21292.59	24	9	[illegible]
Chr14	10652628	T	TA	Chr14_10652	T_TA	INDEL	21292.59	24	9	[illegible]
Chr14	10652628	T	TA	Chr14_10652	T_TA	INDEL	21292.59	24	9	[illegible]
Chr14	10652628	T	TA	Chr14_10652	T_TA	INDEL	21292.59	24	9	[illegible]
Chr14	10652628	T	TA	Chr14_10652	T_TA	INDEL	21292.59	24	9	[illegible]
Chr14	10652639	T	TC	Chr14_10652	T_TC	INDEL	20702.59	25	9	[illegible]
Chr14	10652639	T	TC	Chr14_10652	T_TC	INDEL	20702.59	25	9	[illegible]
Chr14	10652639	T	TC	Chr14_10652	T_TC	INDEL	20702.59	25	9	[illegible]
Chr14	10652639	T	TC	Chr14_10652	T_TC	INDEL	20702.59	25	9	[illegible]
Chr14	10652639	T	TC	Chr14_10652	T_TC	INDEL	20702.59	25	9	[illegible]

Chr14	10665277	AT	A	Chr14_10665	AT_A	INDEL	10785.59	31	11	17	22	[illegible]
Chr14	10718077	G	GAT	Chr14_10718	G_GAT	INDEL	998.35	17	10	10	1	[illegible]
Chr14	10720203	A	AAT	Chr14_10720	A_AAT	INDEL	1792.93	1	0	0	0	[illegible]
Chr14	10720203	A	AAT	Chr14_10720	A_AAT	INDEL	1792.93	1	0	0	0	[illegible]
Chr14	10720203	A	AAT	Chr14_10720	A_AAT	INDEL	1792.93	1	0	0	0	[illegible]
Chr14	10720203	A	AAT	Chr14_10720	A_AAT	INDEL	1792.93	1	0	0	0	[illegible]
Chr14	10756129	A	AAT	Chr14_10756	A_AAT	INDEL	7989.59	15	6	11	13	[illegible]
Chr14	10784346	T	TA	Chr14_10784	T_TA	INDEL	18487.59	38	15	15	34	[illegible]
Chr14	10791066	A	AAT	Chr14_10791	A_AAT	INDEL	2246.93	0	0	0	0	[illegible]
Chr14	10975554	T	TC	Chr14_10975	T_TC	INDEL	11405.59	29	9	6	13	[illegible]
Chr14	10992179	G	A	Chr14_10992	G_A	SNP	12414.29	27	13	18	0	[illegible]
Chr14	11146962	C	CAT	Chr14_11146	C_CAT	INDEL	2281.93	0	0	0	0	[illegible]
Chr14	11146962	C	CAT	Chr14_11146	C_CAT	INDEL	2281.93	0	0	0	0	[illegible]
Chr14	11146962	C	CAT	Chr14_11146	C_CAT	INDEL	2281.93	0	0	0	0	[illegible]
Chr14	11202801	C	T	Chr14_11202	C_T	SNP	6015.29	0	0	0	26	[illegible]
Chr14	11256776	C	T	Chr14_11256	C_T	SNP	13192.29	28	13	16	0	[illegible]
Chr14	11276245	A	AT	Chr14_11276	A_AT	INDEL	12901.59	17	19	12	23	[illegible]
Chr14	11276245	A	AT	Chr14_11276	A_AT	INDEL	12901.59	17	19	12	23	[illegible]
Chr14	11276245	A	AT	Chr14_11276	A_AT	INDEL	12901.59	17	19	12	23	[illegible]
Chr14	11276245	A	AT	Chr14_11276	A_AT	INDEL	12901.59	17	19	12	23	[illegible]
Chr14	11276245	A	AT	Chr14_11276	A_AT	INDEL	12901.59	17	19	12	23	[illegible]
Chr14	11276245	A	AT	Chr14_11276	A_AT	INDEL	12901.59	17	19	12	23	[illegible]
Chr14	11276245	A	AT	Chr14_11276	A_AT	INDEL	12901.59	17	19	12	23	[illegible]
Chr14	11276245	A	AT	Chr14_11276	A_AT	INDEL	12901.59	17	19	12	23	[illegible]
Chr14	11276245	A	AT	Chr14_11276	A_AT	INDEL	12901.59	17	19	12	23	[illegible]
Chr14	11360033	C	CAT	Chr14_11360	C_CAT	INDEL	151.37	0	0	0	1	[illegible]
Chr14	11360033	C	CAT	Chr14_11360	C_CAT	INDEL	151.37	0	0	0	1	[illegible]
Chr14	11360033	C	CAT	Chr14_11360	C_CAT	INDEL	151.37	0	0	0	1	[illegible]
Chr14	11630650	AAT	A	Chr14_11630	AAT_A	INDEL	2013.24	0	2	2	13	[illegible]
Chr14	11819875	C	CA	Chr14_11819	C_CA	INDEL	13534.59	21	11	18	23	[illegible]
Chr14	11846922	T	A	Chr14_11846	T_A	SNP	16390.63	32	15	10	17	[illegible]
Chr14	11846922	T	A	Chr14_11846	T_A	SNP	16390.63	32	15	10	17	[illegible]
Chr14	11849402	T	TGAAATGAA	Chr14_11849	T_TGAAATG	INDEL	21667.59	22	21	10	24	[illegible]
Chr14	11849402	T	TGAAATGAA	Chr14_11849	T_TGAAATG	INDEL	21667.59	22	21	10	24	[illegible]
Chr14	12049054	C	A	Chr14_12049	C_A	SNP	18030.63	22	22	14	30	[illegible]
Chr14	12049054	C	A	Chr14_12049	C_A	SNP	18030.63	22	22	14	30	[illegible]
Chr14	12060464	TG	T	Chr14_12060	TG_T	INDEL	17123.59	23	14	16	32	[illegible]
Chr14	12060548	T	TA	Chr14_12060	T_TA	INDEL	19330.59	25	12	17	24	[illegible]
Chr14	12060570	T	A	Chr14_12060	T_A	SNP	20815.63	30	10	21	31	[illegible]
Chr14	12060704	G	T	Chr14_12060	G_T	SNP	19214.63	37	19	15	38	[illegible]
Chr14	12114588	G	A	Chr14_12114	G_A	SNP	18325.63	25	15	11	21	[illegible]
Chr14	12114588	G	A	Chr14_12114	G_A	SNP	18325.63	25	15	11	21	[illegible]
Chr14	12114618	T	C	Chr14_12114	T_C	SNP	18430.63	23	16	6	18	[illegible]
Chr14	12114618	T	C	Chr14_12114	T_C	SNP	18430.63	23	16	6	18	[illegible]
Chr14	12142605	T	A	Chr14_12142	T_A	SNP	15775.63	18	10	10	23	[illegible]
Chr14	12142672	G	T	Chr14_12142	G_T	SNP	17176.63	26	12	11	19	[illegible]
Chr14	12142711	G	T	Chr14_12142	G_T	SNP	17246.63	22	13	14	16	[illegible]
Chr14	12195276	G	T	Chr14_12195	G_T	SNP	14043.63	24	19	16	19	[illegible]
Chr14	12195288	G	T	Chr14_12195	G_T	SNP	15703.63	23	20	15	19	[illegible]
Chr14	12195291	C	A	Chr14_12195	C_A	SNP	15753.63	24	20	15	19	[illegible]
Chr14	12195302	C	A	Chr14_12195	C_A	SNP	13683.63	23	17	15	18	[illegible]
Chr14	12249752	A	G	Chr14_12249	A_G	SNP	37923.63	52	37	26	65	[illegible]
Chr14	12249752	A	G	Chr14_12249	A_G	SNP	37923.63	52	37	26	65	[illegible]
Chr14	12249752	A	G	Chr14_12249	A_G	SNP	37923.63	52	37	26	65	[illegible]
Chr14	12249752	A	G	Chr14_12249	A_G	SNP	37923.63	52	37	26	65	[illegible]
Chr14	12249759	G	A	Chr14_12249	G_A	SNP	38039.63	55	36	24	65	[illegible]
Chr14	12249759	G	A	Chr14_12249	G_A	SNP	38039.63	55	36	24	65	[illegible]
Chr14	12249759	G	A	Chr14_12249	G_A	SNP	38039.63	55	36	24	65	[illegible]
Chr14	12249759	G	A	Chr14_12249	G_A	SNP	38039.63	55	36	24	65	[illegible]
Chr14	12250319	C	T	Chr14_12250	C_T	SNP	19378.63	28	15	20	32	[illegible]
Chr14	12250319	C	T	Chr14_12250	C_T	SNP	19378.63	28	15	20	32	[illegible]
Chr14	12250319	C	T	Chr14_12250	C_T	SNP	19378.63	28	15	20	32	[illegible]
Chr14	12250319	C	T	Chr14_12250	C_T	SNP	19378.63	28	15	20	32	[illegible]
Chr14	12250399	C	T	Chr14_12250	C_T	SNP	14098.63	23	11	16	20	[illegible]
Chr14	12250399	C	T	Chr14_12250	C_T	SNP	14098.63	23	11	16	20	[illegible]
Chr14	12250399	C	T	Chr14_12250	C_T	SNP	14098.63	23	11	16	20	[illegible]
Chr14	12250399	C	T	Chr14_12250	C_T	SNP	14098.63	23	11	16	20	[illegible]
Chr14	12250442	T	C	Chr14_12250	T_C	SNP	13041.63	24	6	12	22	[illegible]
Chr14	12250442	T	C	Chr14_12250	T_C	SNP	13041.63	24	6	12	22	[illegible]
Chr14	12250442	T	C	Chr14_12250	T_C	SNP	13041.63	24	6	12	22	[illegible]
Chr14	12250442	T	C	Chr14_12250	T_C	SNP	13041.63	24	6	12	22	[illegible]
Chr14	12250539	A	C	Chr14_12250	A_C	SNP	11229.63	27	5	8	29	[illegible]
Chr14	12250539	A	C	Chr14_12250	A_C	SNP	11229.63	27	5	8	29	[illegible]
Chr14	12250539	A	C	Chr14_12250	A_C	SNP	11229.63	27	5	8	29	[illegible]
Chr14	12250539	A	C	Chr14_12250	A_C	SNP	11229.63	27	5	8	29	[illegible]
Chr14	12250662	C	A	Chr14_12250	C_A	SNP	14596.63	13	12	16	19	[illegible]
Chr14	12250662	C	A	Chr14_12250	C_A	SNP	14596.63	13	12	16	19	[illegible]
Chr14	12250662	C	A	Chr14_12250	C_A	SNP	14596.63	13	12	16	19	[illegible]
Chr14	12250662	C	A	Chr14_12250	C_A	SNP	14596.63	13	12	16	19	[illegible]
Chr14	12250668	C	T	Chr14_12250	C_T	SNP	15073.63	13	14	17	17	[illegible]
Chr14	12250668	C	T	Chr14_12250	C_T	SNP	15073.63	13	14	17	17	[illegible]
Chr14	12250668	C	T	Chr14_12250	C_T	SNP	15073.63	13	14	17	17	[illegible]
Chr14	12250668	C	T	Chr14_12250	C_T	SNP	15073.63	13	14	17	17	[illegible]
Chr14	12267839	T	C	Chr14_12267	T_C	SNP	17266.63	21	11	24	26	[illegible]
Chr14	12267839	T	C	Chr14_12267	T_C	SNP	17266.63	21	11	24	26	[illegible]
Chr14	12267839	T	C	Chr14_12267	T_C	SNP	17266.63	21	11	24	26	[illegible]
Chr14	12267855	T	C	Chr14_12267	T_C	SNP	16729.63	19	11	24	21	[illegible]
Chr14	12267855	T	C	Chr14_12267	T_C	SNP	16729.63	19	11	24	21	[illegible]
Chr14	12267855	T	C	Chr14_12267	T_C	SNP	16729.63	19	11	24	21	[illegible]
Chr14	12268255	G	A	Chr14_12268	G_A	SNP	16380.63	33	11	10	18	[illegible]
Chr14	12268255	G	A	Chr14_12268	G_A	SNP	16380.63	33	11	10	18	[illegible]
Chr14	12268255	G	A	Chr14_12268	G_A	SNP	16380.63	33	11	10	18	[illegible]
Chr14	12268261	G	C	Chr14_12268	G_C	SNP	16052.63	31	10	10	17	[illegible]
Chr14	12268261	G	C	Chr14_12268	G_C	SNP	16052.63	31	10	10	17	[illegible]
Chr14	12268261	G	C	Chr14_12268	G_C	SNP	16052.63	31	10	10	17	[illegible]
Chr14	12303276	A	G	Chr14_12303	A_G	SNP	12583.63	19	9	17	12	[illegible]
Chr14	12303295	A	C	Chr14_12303	A_C	SNP	12770.63	17	10	13	13	[illegible]
Chr14	12401620	C	G	Chr14_12401	C_G	SNP	13598.63	20	13	11	16	[illegible]
Chr14	12550525	A	AT	Chr14_12550	A_AT	INDEL	13474.59	32	10	10	18	[illegible]
Chr14	12550525	A	AT	Chr14_12550	A_AT	INDEL	13474.59	32	10	10	18	[illegible]
Chr14	12550525	A	AT	Chr14_12550	A_AT	INDEL	13474.59	32	10	10	18	[illegible]
Chr14	12550525	A	AT	Chr14_12550	A_AT	INDEL	13474.59	32	10	10	18	[illegible]
Chr14	12636166	G	GA	Chr14_12636	G_GA	INDEL	16248.59	36	11	6	30	[illegible]
Chr14	12636189	G	GA	Chr14_12636	G_GA	INDEL	19641.59	36	11	8	35	[illegible]
Chr14	12636200	TC	T	Chr14_12636	TC_T	INDEL	20034.59	32	12	8	30	[illegible]
Chr14	12636204	T	TTTTAAA	Chr14_12636	T_TTTTAAA	INDEL	20576.59	31	12	10	29	[illegible]
Chr14	12636206	ATC	A	Chr14_12636	ATC_A	INDEL	20621.59	31	15	10	27	[illegible]
Chr14	12636212	G	A	Chr14_12636	G_A	SNP	20225.63	33	16	11	24	[illegible]
Chr14	12636214	GC	G	Chr14_12636	GC_G	INDEL	20351.59	33	15	11	24	[illegible]
Chr14	12636218	T	TAA	Chr14_12636	T_TAA	INDEL	20801.59	35	15	11	23	[illegible]
Chr14	12636219	G	GTT	Chr14_12636	G_GTT	INDEL	20936.59	35	15	12	24	[illegible]
Chr14	12636220	G	T	Chr14_12636	G_T	SNP	20958.63	36	15	12	22	[illegible]
Chr14	12636222	GGACGC	G	Chr14_12636	GGACGC_G	INDEL	20139.59	36	15	12	22	[illegible]
Chr14	12636247	A	AT	Chr14_12636	A_AT	INDEL	13882.59	31	10	14	22	[illegible]
Chr14	12797646	TGTTCACGA	T	Chr14_12797	TGTTCACGA_	INDEL	448.37	0	11	0	0	[illegible]
Chr14	12797646	TGTTCACGA	T	Chr14_12797	TGTTCACGA_	INDEL	448.37	0	11	0	0	[illegible]
Chr14	12797646	TGTTCACGA	T	Chr14_12797	TGTTCACGA_	INDEL	448.37	0	11	0	0	[illegible]
Chr14	12797646	TGTTCACGA	T	Chr14_12797	TGTTCACGA_	INDEL	448.37	0	11	0	0	[illegible]
Chr14	12797646	TGTTCACGA	T	Chr14_12797	TGTTCACGA_	INDEL	448.37	0	11	0	0	[illegible]
Chr14	12797646	TGTTCACGA	T	Chr14_12797	TGTTCACGA_	INDEL	448.37	0	11	0	0	[illegible]
Chr14	12797646	TGTTCACGA	T	Chr14_12797	TGTTCACGA_	INDEL	448.37	0	11	0	0	[illegible]
Chr14	12797646	TGTTCACGA	T	Chr14_12797	TGTTCACGA_	INDEL	448.37	0	11	0	0	[illegible]
Chr14	12836767	G	A	Chr14_12836	G_A	SNP	4423.98	0	0	0	1	[illegible]
Chr14	12862496	T	TA	Chr14_12862	T_TA	INDEL	14793.59	29	12	21	28	[illegible]
Chr14	12862496	T	TA	Chr14_12862	T_TA	INDEL	14793.59	29	12	21	28	[illegible]
Chr14	13073468	G	T	Chr14_13073	G_T	SNP	18546.63	31	18	18	30	[illegible]
Chr14	13261026	T	TTA	Chr14_13261	T_TTA	INDEL	200.37	1	1	0	0	[illegible]
Chr14	13278040	G	T	Chr14_13278	G_T	SNP	21775.63	28	20	13	22	[illegible]
Chr14	13278050	G	A	Chr14_13278	G_A	SNP	22126.63	28	20	12	22	[illegible]
Chr14	13278063	AT	A	Chr14_13278	AT_A	INDEL	22865.59	27	19	15	25	[illegible]
Chr14	13278068	CT	C	Chr14_13278	CT_C	INDEL	22287.59	27	20	15	26	[illegible]
Chr14	13341052	G	A	Chr14_13341	G_A	SNP	19227.63	25	15	15	16	[illegible]
Chr14	13347571	G	A	Chr14_13347	G_A	SNP	19493.63	25	12	22	29	[illegible]
Chr14	13347704	C	CA	Chr14_13347	C_CA	INDEL	16782.59	32	9	16	19	[illegible]
Chr14	13348893	T	G	Chr14_13348	T_G	SNP	21791.63	18	13	18	27	[illegible]
Chr14	13348894	G	A	Chr14_13348	G_A	SNP	21791.63	18	13	18	27	[illegible]
Chr14	13351383	C	CA	Chr14_13351	C_CA	INDEL	14466.59	22	12	12	19	[illegible]
Chr14	13351390	C	CA	Chr14_13351	C_CA	INDEL	14647.59	22	12	13	20	[illegible]
Chr14	13513858	GTATATA	G	Chr14_13513	GTATATA_G	INDEL	313.37	0	0	0	0	[illegible]
Chr14	13513858	GTATATA	G	Chr14_13513	GTATATA_G	INDEL	313.37	0	0	0	0	[illegible]
Chr14	13513858	GTATATA	G	Chr14_13513	GTATATA_G	INDEL	313.37	0	0	0	0	[illegible]
Chr14	13513858	GTATATA	G	Chr14_13513	GTATATA_G	INDEL	313.37	0	0	0	0	[illegible]
Chr14	13649060	C	CA	Chr14_13649	C_CA	INDEL	16817.59	31	22	12	26	[illegible]
Chr14	13697603	AC	A	Chr14_13697	AC_A	INDEL	17807.59	25	14	11	28	[illegible]
Chr14	13697603	AC	A	Chr14_13697	AC_A	INDEL	17807.59	25	14	11	28	[illegible]
Chr14	13697603	AC	A	Chr14_13697	AC_A	INDEL	17807.59	25	14	11	28	[illegible]
Chr14	13697606	C	G	Chr14_13697	C_G	SNP	17816.63	26	15	11	28	[illegible]
Chr14	13697606	C	G	Chr14_13697	C_G	SNP	17816.63	26	15	11	28	[illegible]
Chr14	13697606	C	G	Chr14_13697	C_G	SNP	17816.63	26	15	11	28	[illegible]
Chr14	13749227	A	G	Chr14_13749	A_G	SNP	5813.29	0	0	0	31	[illegible]
Chr14	13749227	A	G	Chr14_13749	A_G	SNP	5813.29	0	0	0	31	[illegible]
Chr14	13749227	A	G	Chr14_13749	A_G	SNP	5813.29	0	0	0	31	[illegible]
Chr14	13749227	A	G	Chr14_13749	A_G	SNP	5813.29	0	0	0	31	[illegible]
Chr14	13749227	A	G	Chr14_13749	A_G	SNP	5813.29	0	0	0	31	[illegible]
Chr14	13759023	TA	T	Chr14_13759	TA_T	INDEL	12373.59	22	11	10	16	[illegible]
Chr14	13792733	C	A	Chr14_13792	C_A	SNP	20571.63	27	9	9	32	[illegible]
Chr14	13792733	C	A	Chr14_13792	C_A	SNP	20571.63	27	9	9	32	[illegible]
Chr14	13796872	T	TA	Chr14_13796	T_TA	INDEL	15315.59	29	23	19	22	[illegible]
Chr14	13796872	T	TA	Chr14_13796	T_TA	INDEL	15315.59	29	23	19	22	[illegible]
Chr14	13796872	T	TA	Chr14_13796	T_TA	INDEL	15315.59	29	23	19	22	[illegible]
Chr14	13796872	T	TA	Chr14_13796	T_TA	INDEL	15315.59	29	23	19	22	[illegible]
Chr14	14177138	A	AT	Chr14_14177	A_AT	INDEL	18563.59	28	10	17	19	[illegible]
Chr14	14177143	CG	C	Chr14_14177	CG_C	INDEL	18835.59	26	10	18	21	[illegible]
Chr14	14290907	TC	T	Chr14_14290	TC_T	INDEL	18662.59	37	23	22	34	[illegible]
Chr14	14290907	TC	T	Chr14_14290	TC_T	INDEL	18662.59	37	23	22	34	[illegible]
Chr14	14290907	TC	T	Chr14_14290	TC_T	INDEL	18662.59	37	23	22	34	[illegible]
Chr14	14290907	TC	T	Chr14_14290	TC_T	INDEL	18662.59	37	23	22	34	[illegible]
Chr14	14290907	TC	T	Chr14_14290	TC_T	INDEL	18662.59	37	23	22	34	[illegible]
Chr14	14290907	TC	T	Chr14_14290	TC_T	INDEL	18662.59	37	23	22	34	[illegible]
Chr14	14315789	A	AAT	Chr14_14315	A_AAT	INDEL	9017.59	23	8	12	11	[illegible]
Chr14	14315789	A	AAT	Chr14_14315	A_AAT	INDEL	9017.59	23	8	12	11	[illegible]
Chr14	14315789	A	AAT	Chr14_14315	A_AAT	INDEL	9017.59	23	8	12	11	[illegible]
Chr14	14315789	A	AATAT	Chr14_14315	A_AATAT	INDEL	9017.59	0	0	0	0	[illegible]
Chr14	14315789	A	AATAT	Chr14_14315	A_AATAT	INDEL	9017.59	0	0	0	0	[illegible]
Chr14	14315789	A	AATAT	Chr14_14315	A_AATAT	INDEL	9017.59	0	0	0	0	[illegible]
Chr14	14403462	TTA	T	Chr14_14403	TTA_T	INDEL	6683.24	12	9	10	0	[illegible]
Chr14	14403462	TTA	T	Chr14_14403	TTA_T	INDEL	6683.24	12	9	10	0	[illegible]
Chr14	14403462	TTA	T	Chr14_14403	TTA_T	INDEL	6683.24	12	9	10	0	[illegible]
Chr14	14539900	A	AAT	Chr14_14539	A_AAT	INDEL	2580.24	0	1	1	13	[illegible]
Chr14	14539900	A	AAT	Chr14_14539	A_AAT	INDEL	2580.24	0	1	1	13	[illegible]
Chr14	14539900	A	AAT	Chr14_14539	A_AAT	INDEL	2580.24	0	1	1	13	[illegible]
Chr14	14539900	A	AAT	Chr14_14539	A_AAT	INDEL	2580.24	0	1	1	13	[illegible]
Chr14	14539900	A	AAT	Chr14_14539	A_AAT	INDEL	2580.24	0	1	1	13	[illegible]
Chr14	14539900	A	AAT	Chr14_14539	A_AAT	INDEL	2580.24	0	1	1	13	[illegible]
Chr14	14539900	A	AAT	Chr14_14539	A_AAT	INDEL	2580.24	0	1	1	13	[illegible]
Chr14	14614820	TA	T	Chr14_14614	TA_T	INDEL	15175.59	25	12	20	21	[illegible]
Chr14	14614874	C	A	Chr14_14614	C_A	SNP	20915.63	24	13	19	21	[illegible]
Chr14	14614899	C	T	Chr14_14614	C_T	SNP	20735.63	23	15	19	25	[illegible]
Chr14	14634677	A	AAT	Chr14_14634	A_AAT	INDEL	2099.93	0	0	0	0	[illegible]
Chr14	14662408	GA	G	Chr14_14662	GA_G	INDEL	17063.59	23	17	17	30	[illegible]
Chr14	14662408	GA	G	Chr14_14662	GA_G	INDEL	17063.59	23	17	17	30	[illegible]
Chr14	14662408	GA	G	Chr14_14662	GA_G	INDEL	17063.59	23	17	17	30	[illegible]
Chr14	14662408	GA	G	Chr14_14662	GA_G	INDEL	17063.59	23	17	17	30	[illegible]
Chr14	14662408	GA	G	Chr14_14662	GA_G	INDEL	17063.59	23	17	17	30	[illegible]
Chr14	14662408	GA	G	Chr14_14662	GA_G	INDEL	17063.59	23	17	17	30	[illegible]
Chr14	14662454	A	T	Chr14_14662	A_T	SNP	11510.63	14	13	10	18	[illegible]
Chr14	14662454	A	T	Chr14_14662	A_T	SNP	11510.63	14	13	10	18	[illegible]
Chr14	14662454	A	T	Chr14_14662	A_T	SNP	11510.63	14	13	10	18	[illegible]
Chr14	14662454	A	T	Chr14_14662	A_T	SNP	11510.63	14	13	10	18	[illegible]
Chr14	14662454	A	T	Chr14_14662	A_T	SNP	11510.63	14	13	10	18	[illegible]
Chr14	14662454	A	T	Chr14_14662	A_T	SNP	11510.63	14	13	10	18	[illegible]
Chr14	14662463	G	T	Chr14_14662	G_T	SNP	11113.63	14	10	6	15	[illegible]

Chr14	14662463	G	T	Chr14_14662G_T	SNP	11113.63	14	10	6	15	12	8	15	14	12	12	10	9	8	5
Chr14	14662463	G	T	Chr14_14662G_T	SNP	11113.63	14	10	6	15	12	8	15	14	12	12	10	9	8	5
Chr14	14662463	G	T	Chr14_14662G_T	SNP	11113.63	14	10	6	15	12	8	15	14	12	12	10	9	8	5
Chr14	14662463	G	T	Chr14_14662G_T	SNP	11113.63	14	10	6	15	12	8	15	14	12	12	10	9	8	5
Chr14	14662463	G	T	Chr14_14662G_T	SNP	11113.63	14	10	6	15	12	8	15	14	12	12	10	9	8	5
Chr14	14662519	G	A	Chr14_14662G_A	SNP	12705.63	16	7	8	19	10	15	21	12	12	15	6	10	4	10
Chr14	14662519	G	A	Chr14_14662G_A	SNP	12705.63	16	7	8	19	10	15	21	12	12	15	6	10	4	10
Chr14	14662519	G	A	Chr14_14662G_A	SNP	12705.63	16	7	8	19	10	15	21	12	12	15	6	10	4	10
Chr14	14662519	G	A	Chr14_14662G_A	SNP	12705.63	16	7	8	19	10	15	21	12	12	15	6	10	4	10
Chr14	14662519	G	A	Chr14_14662G_A	SNP	12705.63	16	7	8	19	10	15	21	12	12	15	6	10	4	10
Chr14	14662519	G	A	Chr14_14662G_A	SNP	12705.63	16	7	8	19	10	15	21	12	12	15	6	10	4	10
Chr14	14662522	G	T	Chr14_14662G_T	SNP	12432.63	17	7	8	18	10	14	23	11	13	14	6	10	4	10
Chr14	14662522	G	T	Chr14_14662G_T	SNP	12432.63	17	7	8	18	10	14	23	11	13	14	6	10	4	10
Chr14	14662522	G	T	Chr14_14662G_T	SNP	12432.63	17	7	8	18	10	14	23	11	13	14	6	10	4	10
Chr14	14662522	G	T	Chr14_14662G_T	SNP	12432.63	17	7	8	18	10	14	23	11	13	14	6	10	4	10
Chr14	14662522	G	T	Chr14_14662G_T	SNP	12432.63	17	7	8	18	10	14	23	11	13	14	6	10	4	10
Chr14	14662522	G	T	Chr14_14662G_T	SNP	12432.63	17	7	8	18	10	14	23	11	13	14	6	10	4	10
Chr14	14763394	G	T	Chr14_14763G_T	SNP	7689.29	20	11	10	0	2	0	0	0	18	7	10	13	8	5
Chr14	14803817	TC	T	Chr14_14803TC_T	INDEL	10287.59	14	6	10	13	15	16	16	6	7	8	7	11	5	10
Chr14	14891135	C	CA	Chr14_14891C_CA	INDEL	14096.59	18	9	16	18	14	21	16	12	15	15	4	12	4	11
Chr14	14891135	C	CA	Chr14_14891C_CA	INDEL	14096.59	18	9	16	18	14	21	16	12	15	15	4	12	4	11
Chr14	14891135	C	CA	Chr14_14891C_CA	INDEL	14096.59	18	9	16	18	14	21	16	12	15	15	4	12	4	11
Chr14	14891135	C	CA	Chr14_14891C_CA	INDEL	14096.59	18	9	16	18	14	21	16	12	15	15	4	12	4	11
Chr14	14891135	C	CA	Chr14_14891C_CA	INDEL	14096.59	18	9	16	18	14	21	16	12	15	15	4	12	4	11

[illegible]

Chr15	949929	G	GTA	Chr15_94992G_GTA	INDEL	3380.24	0	0	0	18	15	20	23	15	1	2	0	0	0	0
Chr15	949929	G	GTA	Chr15_94992G_GTA	INDEL	3380.24	0	0	0	18	15	20	23	15	1	2	0	0	0	0
Chr15	949929	G	GTA	Chr15_94992G_GTA	INDEL	3380.24	0	0	0	18	15	20	23	15	1	2	0	0	0	0
Chr15	1091103	T	A	Chr15_10911T_A	SNP	5628.29	0	0	0	22	16	25	21	22	0	0	0	0	0	0
Chr15	1309528	G	GAAT	Chr15_13095G_GAAT	INDEL	18164.59	28	20	18	24	11	17	21	23	19	26	18	15	7	11
Chr15	1309528	G	GAAT	Chr15_13095G_GAAT	INDEL	18164.59	28	20	18	24	11	17	21	23	19	26	18	15	7	11
Chr15	1309528	G	GAAT	Chr15_13095G_GAAT	INDEL	18164.59	28	20	18	24	11	17	21	23	19	26	18	15	7	11
Chr15	1309528	G	GAAT	Chr15_13095G_GAAT	INDEL	18164.59	28	20	18	24	11	17	21	23	19	26	18	15	7	11
Chr15	1309528	G	GAAT	Chr15_13095G_GAAT	INDEL	18164.59	28	20	18	24	11	17	21	23	19	26	18	15	7	11
Chr15	1309528	G	GAAT	Chr15_13095G_GAAT	INDEL	18164.59	28	20	18	24	11	17	21	23	19	26	18	15	7	11
Chr15	1309528	G	GAAT	Chr15_13095G_GAAT	INDEL	18164.59	28	20	18	24	11	17	21	23	19	26	18	15	7	11
Chr15	1309528	G	GAAT	Chr15_13095G_GAAT	INDEL	18164.59	28	20	18	24	11	17	21	23	19	26	18	15	7	11
Chr15	1309529	CTG	C	Chr15_13095CTG_C	INDEL	18434.59	30	21	19	25	11	18	19	24	21	26	18	14	6	11
Chr15	1309529	CTG	C	Chr15_13095CTG_C	INDEL	18434.59	30	21	19	25	11	18	19	24	21	26	18	14	6	11
Chr15	1309529	CTG	C	Chr15_13095CTG_C	INDEL	18434.59	30	21	19	25	11	18	19	24	21	26	18	14	6	11
Chr15	1309529	CTG	C	Chr15_13095CTG_C	INDEL	18434.59	30	21	19	25	11	18	19	24	21	26	18	14	6	11
Chr15	1309529	CTG	C	Chr15_13095CTG_C	INDEL	18434.59	30	21	19	25	11	18	19	24	21	26	18	14	6	11
Chr15	1309529	CTG	C	Chr15_13095CTG_C	INDEL	18434.59	30	21	19	25	11	18	19	24	21	26	18	14	6	11
Chr15	1309529	CTG	C	Chr15_13095CTG_C	INDEL	18434.59	30	21	19	25	11	18	19	24	21	26	18	14	6	11
Chr15	1309529	CTG	C	Chr15_13095CTG_C	INDEL	18434.59	30	21	19	25	11	18	19	24	21	26	18	14	6	11
Chr15	1309536	C	A	Chr15_13095C_A	SNP	18470.63	31	22	19	25	13	17	20	25	22	26	18	13	4	11
Chr15	1309536	C	A	Chr15_13095C_A	SNP	18470.63	31	22	19	25	13	17	20	25	22	26	18	13	4	11
Chr15	1309536	C	A	Chr15_13095C_A	SNP	18470.63	31	22	19	25	13	17	20	25	22	26	18	13	4	11
Chr15	1309536	C	A	Chr15_13095C_A	SNP	18470.63	31	22	19	25	13	17	20	25	22	26	18	13	4	11

[illegible]

Chr15	4857294	AAT	A	Chr15_48572	AAT_A	INDEL	10195.59	24	7	11	9	14	18	24	23	14	15	10	8	6	11
Chr15	4857294	AAT	A	Chr15_48572	AAT_A	INDEL	10195.59	24	7	11	9	14	18	24	23	14	15	10	8	6	11
Chr15	4857294	AAT	A	Chr15_48572	AAT_A	INDEL	10195.59	24	7	11	9	14	18	24	23	14	15	10	8	6	11
Chr15	4857294	AAT	A	Chr15_48572	AAT_A	INDEL	10195.59	24	7	11	9	14	18	24	23	14	15	10	8	6	11
Chr15	4965960	TTGTATGGT	T	Chr15_49659	TTGTATGGT	INDEL	11502.59	10	10	10	21	21	5	21	14	14	14	12	4	11	11
Chr15	4969364	G	GA	Chr15_49693	G_GA	INDEL	18015.59	25	17	14	29	23	34	33	23	37	21	17	17	10	14
Chr15	5026398	G	GA	Chr15_50263	G_GA	INDEL	15995.59	23	14	24	23	23	12	35	17	17	18	17	9	7	12
Chr15	5026398	G	GA	Chr15_50263	G_GA	INDEL	15995.59	23	14	24	23	23	12	35	17	17	18	17	9	7	12
Chr15	5026398	G	GA	Chr15_50263	G_GA	INDEL	15995.59	23	14	24	23	23	12	35	17	17	18	17	9	7	12
Chr15	5026398	G	GA	Chr15_50263	G_GA	INDEL	15995.59	23	14	24	23	23	12	35	17	17	18	17	9	7	12
Chr15	5026398	G	GA	Chr15_50263	G_GA	INDEL	15995.59	23	14	24	23	23	12	35	17	17	18	17	9	7	12
Chr15	5026398	G	GA	Chr15_50263	G_GA	INDEL	15995.59	23	14	24	23	23	12	35	17	17	18	17	9	7	12
Chr15	5108016	C	T	Chr15_51080	C_T	SNP	15661.63	18	16	14	17	24	28	25	34	21	17	14	15	13	7
Chr15	5109573	C	T	Chr15_51095	C_T	SNP	17164.63	23	10	14	18	21	26	15	16	16	29	16	23	12	5
Chr15	5260834	A	AAATTATATT	Chr15_52608	A_AAATTATA	INDEL	7440.59	9	10	4	5	9	8	12	9	9	6	4	7	6	6
Chr15	5324491	T	A	Chr15_53244	T_A	SNP	15198.63	28	18	15	23	12	21	28	25	26	19	13	16	17	6
Chr15	5324560	T	C	Chr15_53245	T_C	SNP	12003.63	30	14	10	19	14	15	24	17	20	10	5	13	7	11
Chr15	5324985	G	C	Chr15_53249	G_C	SNP	15013.63	24	15	12	15	21	23	29	13	26	21	14	13	11	10
Chr15	5324997	C	T	Chr15_53249	C_T	SNP	15732.63	27	16	11	17	19	24	29	15	26	22	14	13	10	11
Chr15	5470175	G	A	Chr15_54701	G_A	SNP	6230.29	0	0	0	24	23	30	31	19	0	0	0	0	0	0
Chr15	5676314	G	A	Chr15_56763	G_A	SNP	8575.63	14	5	9	16	15	14	18	12	7	13	6	5	5	4
Chr15	5787224	T	G	Chr15_57872	T_G	SNP	7209.63	4	5	10	8	13	18	18	11	5	11	7	12	8	11
Chr15	5852020	G	T	Chr15_58520	G_T	SNP	18167.63	19	17	13	25	22	28	10	27	20	33	20	15	23	11
Chr15	5859489	T	G	Chr15_58594	T_G	SNP	17454.63	19	15	16	26	30	33	14	24	21	27	17	19	14	14
[illegible]																					

Chr15	8432451	G	T	Chr15_84324G_T	SNP	24001.63	30	18	17	29	34	36	39	20	35	30
Chr15	8432833	T	A	Chr15_84328T_A	SNP	1903.39	19	10	19	0	0	0	0	0	0	0
Chr15	8432833	T	A	Chr15_84328T_A	SNP	1903.39	19	10	19	0	0	0	0	0	0	0
Chr15	8432833	T	A	Chr15_84328T_A	SNP	1903.39	19	10	19	0	0	0	0	0	0	0
Chr15	8432833	T	A	Chr15_84328T_A	SNP	1903.39	19	10	19	0	0	0	0	0	0	0
Chr15	8432833	T	A	Chr15_84328T_A	SNP	1903.39	19	10	19	0	0	0	0	0	0	0
Chr15	8443682	C	A	Chr15_84436C_A	SNP	12231.63	21	9	13	14	13	20	14	23	16	10
Chr15	8443684	A	T	Chr15_84436A_T	SNP	12231.63	20	9	13	14	13	21	14	24	16	10
Chr15	8445803	A	T	Chr15_84458A_T	SNP	15523.63	20	9	15	18	9	22	15	16	13	22
Chr15	8511896	C	G	Chr15_85118C_G	SNP	18616.63	26	16	6	24	14	24	24	23	19	23
Chr15	8511928	T	C	Chr15_85119T_C	SNP	19411.63	28	17	13	23	14	24	22	24	22	20
Chr15	8511943	G	GT	Chr15_85119G_GT	INDEL	19775.59	30	19	13	25	13	26	23	27	22	25
Chr15	8560562	A	AAT	Chr15_85605A_AAT	INDEL	8214.59	17	10	8	13	12	18	15	16	14	10
Chr15	8578154	C	A	Chr15_85781C_A	SNP	11152.63	23	5	11	18	16	13	20	12	10	11
Chr15	8584093	G	GA	Chr15_85840G_GA	INDEL	15283.59	23	21	25	27	23	27	36	21	29	20
Chr15	8695650	CT	C	Chr15_86956CT_C	INDEL	18349.59	37	14	15	22	15	21	33	22	29	26
Chr15	8786582	A	T	Chr15_87865A_T	SNP	19356.63	37	13	23	27	17	29	22	10	25	20
Chr15	8807078	G	T	Chr15_88070G_T	SNP	19391.63	21	15	11	31	24	21	25	26	15	29
Chr15	8812499	C	A	Chr15_88124C_A	SNP	17338.63	25	10	15	36	17	21	20	13	19	22
Chr15	8821565	C	CAT	Chr15_88215C_CAT	INDEL	3236.24	1	0	0	12	11	19	26	12	2	2
Chr15	8902307	T	A	Chr15_89023T_A	SNP	21565.63	25	15	15	20	24	29	21	22	28	18
Chr15	8902315	C	A	Chr15_89023C_A	SNP	21746.63	27	14	17	22	23	27	24	20	27	20
Chr15	9036234	A	T	Chr15_90362A_T	SNP	14205.63	13	12	15	17	17	25	23	13	18	20
Chr15	9036293	G	T	Chr15_90362G_T	SNP	15613.63	22	11	24	17	20	36	23	14	18	19

Chr15	12058384	G	C	Chr15_12058	G_C	SNP	8868.63	9	6	4	14	5	15	17	14	11	7
Chr15	12058384	G	C	Chr15_12058	G_C	SNP	8868.63	9	6	4	14	5	15	17	14	11	7
Chr15	12524713	A	AAT	Chr15_12524	A_AAT	INDEL	2072.93	0	0	0	0	0	0	0	0	8	18
Chr15	12524713	A	AAT	Chr15_12524	A_AAT	INDEL	2072.93	0	0	0	0	0	0	0	0	8	18
Chr15	12524713	A	AAT	Chr15_12524	A_AAT	INDEL	2072.93	0	0	0	0	0	0	0	0	8	18
Chr15	12524713	A	AAT	Chr15_12524	A_AAT	INDEL	2072.93	0	0	0	0	0	0	0	0	8	18
Chr15	12524713	A	AAT	Chr15_12524	A_AAT	INDEL	2072.93	0	0	0	0	0	0	0	0	8	18
Chr15	12524713	A	AAT	Chr15_12524	A_AAT	INDEL	2072.93	0	0	0	0	0	0	0	0	8	18
Chr15	12524713	A	AAT	Chr15_12524	A_AAT	INDEL	2072.93	0	0	0	0	0	0	0	0	8	18
Chr15	12701245	G	C	Chr15_12701	G_C	SNP	20111.63	28	13	15	20	14	25	36	27	26	32
Chr15	12701245	G	C	Chr15_12701	G_C	SNP	20111.63	28	13	15	20	14	25	36	27	26	32
Chr15	12701245	G	C	Chr15_12701	G_C	SNP	20111.63	28	13	15	20	14	25	36	27	26	32
Chr15	12701245	G	C	Chr15_12701	G_C	SNP	20111.63	28	13	15	20	14	25	36	27	26	32
Chr15	12701245	G	C	Chr15_12701	G_C	SNP	20111.63	28	13	15	20	14	25	36	27	26	32
Chr15	12701245	G	C	Chr15_12701	G_C	SNP	20111.63	28	13	15	20	14	25	36	27	26	32
Chr15	12701245	G	C	Chr15_12701	G_C	SNP	20111.63	28	13	15	20	14	25	36	27	26	32
Chr15	12818844	A	AT	Chr15_12818	A_AT	INDEL	39023.59	59	23	32	38	37	50	61	40	38	51
Chr15	12818844	A	AT	Chr15_12818	A_AT	INDEL	39023.59	59	23	32	38	37	50	61	40	38	51
Chr15	12818844	A	AT	Chr15_12818	A_AT	INDEL	39023.59	59	23	32	38	37	50	61	40	38	51
Chr15	12818851	A	T	Chr15_12818	A_T	SNP	40043.63	61	22	34	42	39	52	60	42	44	52
Chr15	12818851	A	T	Chr15_12818	A_T	SNP	40043.63	61	22	34	42	39	52	60	42	44	52
Chr15	12818851	A	T	Chr15_12818	A_T	SNP	40043.63	61	22	34	42	39	52	60	42	44	52
Chr15	12818976	G	C	Chr15_12818	G_C	SNP	40029.63	67	34	27	35	33	61	61	39	54	63
Chr15	12818976	G	C	Chr15_12818	G_C	SNP	40029.63	67	34	27	35	33	61	61	39	54	63

[illegible]

Chr15	14680820	C	A	Chr15_14680C_A	SNP	13340.63	23	5	11	18	10	15	22	16	11	8	5	10	5	6	23	5	11	18	10	15	22	16	11	8	5	10	5	6	1	1	1	1	1	1	1	1	1	1	1	1	1	1	0	15	15	0	7	5	3	downstream_	MODIFIER	Pp3c15_2272	Pp3c15_2272	transcript	PAC:3292774	protein_codi	NA		c.*1983C>A	NA	NA	NA	NA	1810
Chr15	14680820	C	A	Chr15_14680C_A	SNP	13340.63	23	5	11	18	10	15	22	16	11	8	5	10	5	6	23	5	11	18	10	15	22	16	11	8	5	10	5	6	1	1	1	1	1	1	1	1	1	1	1	1	1	1	0	15	15	0	7	5	3	downstream_	MODIFIER	Pp3c15_2273	Pp3c15_2273	transcript	PAC:3292661	protein_codi	NA		c.*158G>T	NA	NA	NA	NA	1
Chr15	14680820	C	A	Chr15_14680C_A	SNP	13340.63	23	5	11	18	10	15	22	16	11	8	5	10	5	6	23	5	11	18	10	15	22	16	11	8	5	10	5	6	1	1	1	1	1	1	1	1	1	1	1	1	1	1	0	15	15	0	7	5	3	downstream_	MODIFIER	Pp3c15_2274	Pp3c15_2274	transcript	PAC:3292851	protein_codi	NA		c.*2218G>T	NA	NA	NA	NA	1735
Chr15	14680820	C	A	Chr15_14680C_A	SNP	13340.63	23	5	11	18	10	15	22	16	11	8	5	10	5	6	23	5	11	18	10	15	22	16	11	8	5	10	5	6	1	1	1	1	1	1	1	1	1	1	1	1	1	1	0	15	15	0	7	5	3	downstream_	MODIFIER	Pp3c15_2274	Pp3c15_2274	transcript	PAC:3292851	protein_codi	NA		c.*2218G>T	NA	NA	NA	NA	1735
Chr15	14680825	GTCTT	G	Chr15_14680GTCTT_G	INDEL	13376.59	21	5	11	20	9	16	23	17	12	7	5	10	5	7	21	5	11	20	9	16	23	17	12	7	5	10	5	7	1	1	1	1	1	1	1	1	1	1	1	1	1	1	0	15	15	0	7	5	3	3_prime_UTI	MODIFIER	Pp3c15_2273	Pp3c15_2273	transcript	PAC:3292661	protein_codi		02-févr	c.*149_*152	NA	NA	NA	NA	149
Chr15	14680825	GTCTT	G	Chr15_14680GTCTT_G	INDEL	13376.59	21	5	11	20	9	16	23	17	12	7	5	10	5	7	21	5	11	20	9	16	23	17	12	7	5	10	5	7	1	1	1	1	1	1	1	1	1	1	1	1	1	1	0	15	15	0	7	5	3	3_prime_UTI	MODIFIER	Pp3c15_2273	Pp3c15_2273	transcript	PAC:3292661	protein_codi		02-févr	c.*149_*152	NA	NA	NA	NA	149
Chr15	14680825	GTCTT	G	Chr15_14680GTCTT_G	INDEL	13376.59	21	5	11	20	9	16	23	17	12	7	5	10	5	7	21	5	11	20	9	16	23	17	12	7	5	10	5	7	1	1	1	1	1	1	1	1	1	1	1	1	1	1	0	15	15	0	7	5	3	upstream_ge	MODIFIER	Pp3c15_2271	Pp3c15_2271	transcript	PAC:3292658	protein_codi	NA		c.-4718_-471	NA	NA	NA	NA	3920
Chr15	14680825	GTCTT	G	Chr15_14680GTCTT_G	INDEL	13376.59	21	5	11	20	9	16	23	17	12	7	5	10	5	7	21	5	11	20	9	16	23	17	12	7	5	10	5	7	1	1	1	1	1	1	1	1	1	1	1	1	1	1	0	15	15	0	7	5	3	upstream_ge	MODIFIER	Pp3c15_2275	Pp3c15_2275	transcript	PAC:3292661	protein_codi	NA		c.-4599_-459	NA	NA	NA	NA	4237
Chr15	14680825	GTCTT	G	Chr15_14680GTCTT_G	INDEL	13376.59	21	5	11	20	9	16	23	17	12	7	5	10	5	7	21	5	11	20	9	16	23	17	12	7	5	10	5	7	1	1	1	1	1	1	1	1	1	1	1	1	1	1	0	15	15	0	7	5	3	upstream_ge	MODIFIER	Pp3c15_2271	Pp3c15_2271	transcript	PAC:3292658	protein_codi	NA		c.-4718_-471	NA	NA	NA	NA	3895
Chr15	14680825	GTCTT	G	Chr15_14680GTCTT_G	INDEL	13376.59	21	5	11	20	9	16	23	17	12	7	5	10	5	7	21	5	11	20	9	16	23	17	12	7	5	10	5	7	1	1	1	1	1	1	1	1	1	1	1	1	1	1	0	15	15	0	7	5	3	downstream_	MODIFIER	Pp3c15_2272	Pp3c15_2272	transcript	PAC:3292774	protein_codi	NA		c.*1989_*19	NA	NA	NA	NA	1796
Chr15	14680825	GTCTT	G	Chr15_14680GTCTT_G	INDEL	13376.59	21	5	11	20	9	16	23	17	12	7	5	10	5	7	21	5	11	20	9	16	23	17	12	7	5	10	5	7	1	1	1	1	1	1	1	1	1	1	1	1	1	1	0	15	15	0	7	5	3	downstream_	MODIFIER	Pp3c15_2274	Pp3c15_2274	transcript	PAC:3292851	protein_codi	NA		c.*2209_*22	NA	NA	NA	NA	1748
Chr15	14680825	GTCTT	G	Chr15_14680GTCTT_G	INDEL	13376.59	21	5	11	20	9	16	23	17	12	7	5	10	5	7	21	5	11	20	9	16	23	17	12	7	5	10	5	7	1	1	1	1	1	1	1	1	1	1	1	1	1	1	0	15	15	0	7	5	3	downstream_	MODIFIER	Pp3c15_2272	Pp3c15_2272	transcript	PAC:3292774	protein_codi	NA		c.*1989_*19	NA	NA	NA	NA	1816
Chr15	14680825	GTCTT	G	Chr15_14680GTCTT_G	INDEL	13376.59	21	5	11	20	9	16	23	17	12	7	5	10	5	7	21	5	11	20	9	16	23	17	12	7	5	10	5	7	1	1	1	1	1	1	1	1	1	1	1	1	1	1	0	15	15	0	7	5	3	downstream_	MODIFIER	Pp3c15_2274	Pp3c15_2274	transcript	PAC:3292851	protein_codi	NA		c.*2209_*22	NA	NA	NA	NA	1729
Chr15	14680825	GTCTT	G	Chr15_14680GTCTT_G	INDEL	13376.59	21	5	11	20	9	16	23	17	12	7	5	10	5	7	21	5	11	20	9	16	23	17	12	7	5	10	5	7	1	1	1	1	1	1	1	1	1	1	1	1	1	1	0	15	15	0	7	5	3	downstream_	MODIFIER	Pp3c15_2274	Pp3c15_2274	transcript	PAC:3292851	protein_codi	NA		c.*2209_*22	NA	NA	NA	NA	1729
Chr15	14680830	GTTATT	G	Chr15_14680GTTATT_G	INDEL	13286.59	21	5	11	20	9	16	23	17	12	7	5	10	5	7	21	5	11	20	9	16	23	17	12	7	5	10	5	7	1	1	1	1	1	1	1	1	1	1	1	1	1	1	0	15	15	0	7	5	3	3_prime_UTI	MODIFIER	Pp3c15_2273	Pp3c15_2273	transcript	PAC:3292661	protein_codi		02-févr	c.*143_*147	NA	NA	NA	NA	143
Chr15	14680830	GTTATT	G	Chr15_14680GTTATT_G	INDEL	13286.59	21	5	11	20	9	16	23	17	12	7	5	10	5	7	21	5	11	20	9	16	23	17	12	7	5	10	5	7	1	1	1	1	1	1	1	1	1	1	1	1	1	1	0	15	15	0	7	5	3	3_prime_UTI	MODIFIER	Pp3c15_2273	Pp3c15_2273	transcript	PAC:3292661	protein_codi		02-févr	c.*143_*147	NA	NA	NA	NA	143
Chr15	14680830	GTTATT	G	Chr15_14680GTTATT_G	INDEL	13286.59	21	5	11	20	9	16	23	17	12	7	5	10	5	7	21	5	11	20	9	16	23	17	12	7	5	10	5	7	1	1	1	1	1	1	1	1	1	1	1	1	1	1	0	15	15	0	7	5	3	upstream_ge	MODIFIER	Pp3c15_2271	Pp3c15_2271	transcript	PAC:3292658	protein_codi	NA		c.-4724_-472	NA	NA	NA	NA	3925
Chr15	14680830	GTTATT	G	Chr15_14680GTTATT_G	INDEL	13286.59	21	5	11	20	9	16	23	17	12	7	5	10	5	7	21	5	11	20	9	16	23	17	12	7	5	10	5	7	1	1	1	1	1	1	1	1	1	1	1	1	1	1	0	15	15	0	7	5	3	upstream_ge	MODIFIER	Pp3c15_2275	Pp3c15_2275	transcript	PAC:3292661	protein_codi	NA		c.-4594_-459	NA	NA	NA	NA	4232
Chr15	14680830	GTTATT	G	Chr15_14680GTTATT_G	INDEL	13286.59	21	5	11	20	9	16	23	17	12	7	5	10	5	7	21	5	11	20	9	16	23	17	12	7	5	10	5	7	1	1	1	1	1	1	1	1	1	1	1	1	1	1	0	15	15	0	7	5	3	upstream_ge	MODIFIER	Pp3c15_2271	Pp3c15_2271	transcript	PAC:3292658	protein_codi	NA		c.-4724_-472	NA	NA	NA	NA	3900
Chr15	14680830	GTTATT	G	Chr15_14680GTTATT_G	INDEL	13286.59	21	5	11	20	9	16	23	17	12	7	5	10	5	7	21	5	11	20	9	16	23	17	12	7	5	10	5	7	1	1	1	1	1	1	1	1	1	1	1	1	1	1	0	15	15	0	7	5	3	downstream_	MODIFIER	Pp3c15_2272	Pp3c15_2272	transcript	PAC:3292774	protein_codi	NA		c.*1994_*19	NA	NA	NA	NA	1801
Chr15	14680830	GTTATT	G	Chr15_14680GTTATT_G	INDEL	13286.59	21	5	11	20	9	16	23	17	12	7	5	10	5	7	21	5	11	20	9	16	23	17	12	7	5	10	5	7	1	1	1	1	1	1	1	1	1	1	1	1	1	1	0	15	15	0	7	5	3	downstream_	MODIFIER	Pp3c15_2274	Pp3c15_2274	transcript	PAC:3292851	protein_codi	NA		c.*2203_*22	NA	NA	NA	NA	1743
Chr15	14680830	GTTATT	G	Chr15_14680GTTATT_G	INDEL	13286.59	21	5	11	20	9	16	23	17	12	7	5	10	5	7	21	5	11	20	9	16	23	17	12	7	5	10	5	7	1	1	1	1	1	1	1	1	1	1	1	1	1	1	0	15	15	0	7	5	3	downstream_	MODIFIER	Pp3c15_2272	Pp3c15_2272	transcript	PAC:3292774	protein_codi	NA		c.*1994_*19	NA	NA	NA	NA	1821
Chr15	14680830	GTTATT	G	Chr15_14680GTTATT_G	INDEL	13286.59	21	5	11	20	9	16	23	17	12	7	5	10	5	7	21	5	11	20	9	16	23	17	12	7	5	10	5	7	1	1	1	1	1	1	1	1	1	1	1	1	1	1	0	15	15	0	7	5	3	downstream_	MODIFIER	Pp3c15_2274	Pp3c15_2274	transcript	PAC:3292851	protein_codi	NA		c.*2203_*22	NA	NA	NA	NA	1724
Chr15	14680830	GTTATT	G	Chr15_14680GTTATT_G	INDEL	13286.59	21	5	11	20	9	16	23	17	12	7	5	10	5	7	21	5	11	20	9	16	23	17	12	7	5	10	5	7	1	1	1	1	1	1	1	1	1	1	1	1	1	1	0	15	15	0	7	5	3	downstream_	MODIFIER	Pp3c15_2274	Pp3c15_2274	transcript	PAC:3292851	protein_codi	NA		c.*2203_*22	NA	NA	NA	NA	1724
[illegible]																																																																					

Chr16	1014785	A	G	Chr16_10147A_G	SNP	10771.29	34	12	12	1	0	0	0	0	27	16	8	13	15	7	34	12	12	20	22	25	26	30	27	16	8	13	15	7	1	1	1	0	0	0	0	0	1	1	1	1	1	1	0	10	10	0	7	0	3	intergenic_re	MODIFIER	Pp3c16_162	Pp3c16_162	intergenic_re	Pp3c16_162	NA	NA	n.1014785A>	NA	NA	NA	NA	NA
Chr16	1106992	C	G	Chr16_11069C_G	SNP	15036.63	22	12	16	15	17	19	31	19	12	12	7	13	16	9	22	12	16	15	17	19	31	19	12	12	7	13	16	9	1	1	1	1	1	1	1	1	1	1	1	1	1	1	0	15	15	0	7	5	3	3_prime_UT	MODIFIER	Pp3c16_174	Pp3c16_174	transcript	PAC:3298551	protein_codi	01-janv	c.*210C>G	NA	NA	NA	NA	210
Chr16	1106992	C	G	Chr16_11069C_G	SNP	15036.63	22	12	16	15	17	19	31	19	12	12	7	13	16	9	22	12	16	15	17	19	31	19	12	12	7	13	16	9	1	1	1	1	1	1	1	1	1	1	1	1	1	1	0	15	15	0	7	5	3	downstream_	MODIFIER	Pp3c16_174	Pp3c16_174	transcript	PAC:3298551	protein_codi	NA	c.*210C>G	NA	NA	NA	NA	12
Chr16	1111235	A	T	Chr16_11112A_T	SNP	10939.63	19	8	5	9	13	16	18	10	13	10	12	7	10	10	19	8	5	9	13	16	18	10	13	10	12	7	10	10	1	1	1	1	1	1	1	1	1	1	1	1	1	1	0	15	15	0	7	5	3	downstream_	MODIFIER	Pp3c16_174	Pp3c16_174	transcript	PAC:3298551	protein_codi	NA	c.*4453A>T	NA	NA	NA	NA	3617
Chr16	1111235	A	T	Chr16_11112A_T	SNP	10939.63	19	8	5	9	13	16	18	10	13	10	12	7	10	10	19	8	5	9	13	16	18	10	13	10	12	7	10	10	1	1	1	1	1	1	1	1	1	1	1	1	1	1	0	15	15	0	7	5	3	downstream_	MODIFIER	Pp3c16_174	Pp3c16_174	transcript	PAC:3298551	protein_codi	NA	c.*4453A>T	NA	NA	NA	NA	4255
Chr16	1111235	A	T	Chr16_11112A_T	SNP	10939.63	19	8	5	9	13	16	18	10	13	10	12	7	10	10	19	8	5	9	13	16	18	10	13	10	12	7	10	10	1	1	1	1	1	1	1	1	1	1	1	1	1	1	0	15	15	0	7	5	3	intergenic_re	MODIFIER	Pp3c16_174	Pp3c16_174	intergenic_re	Pp3c16_174	NA	NA	n.1111235A>	NA	NA	NA	NA	NA
Chr16	1111236	A	T	Chr16_11112A_T	SNP	10939.63	19	7	4	8	13	16	18	10	13	9	12	7	10	9	19	7	4	8	13	16	18	10	13	9	12	7	10	9	1	1	1	1	1	1	1	1	1	1	1	1	1	1	0	15	15	0	7	5	3	downstream_	MODIFIER	Pp3c16_174	Pp3c16_174	transcript	PAC:3298551	protein_codi	NA	c.*4454A>T	NA	NA	NA	NA	3618
Chr16	1111236	A	T	Chr16_11112A_T	SNP	10939.63	19	7	4	8	13	16	18	10	13	9	12	7	10	9	19	7	4	8	13	16	18	10	13	9	12	7	10	9	1	1	1	1	1	1	1	1	1	1	1	1	1	1	0	15	15	0	7	5	3	downstream_	MODIFIER	Pp3c16_174	Pp3c16_174	transcript	PAC:3298551	protein_codi	NA	c.*4454A>T	NA	NA	NA	NA	4256
Chr16	1111236	A	T	Chr16_11112A_T	SNP	10939.63	19	7	4	8	13	16	18	10	13	9	12	7	10	9	19	7	4	8	13	16	18	10	13	9	12	7	10	9	1	1	1	1	1	1	1	1	1	1	1	1	1	1	0	15	15	0	7	5	3	intergenic_re	MODIFIER	Pp3c16_174	Pp3c16_174	intergenic_re	Pp3c16_174	NA	NA	n.1111236A>	NA	NA	NA	NA	NA
Chr16	1251470	C	A	Chr16_12514C_A	SNP	18225.63	23	9	14	23	26	21	14	21	21	27	12	18	22	6	23	9	14	23	26	21	14	21	21	27	12	18	22	6	1	1	1	1	1	1	1	1	1	1	1	1	1	1	0	15	15	0	7	5	3	intergenic_re	MODIFIER	Pp3c16_190	Pp3c16_190	intergenic_re	Pp3c16_190	NA	NA	n.1251470C>	NA	NA	NA	NA	NA
Chr16	1305108	A	G	Chr16_13051A_G	SNP	12480.63	10	7	5	12	10	22	24	19	13	19	15	10	10	7	10	7	5	12	10	22	24	19	13	19	15	10	10	7	1	1	1	1	1	1	1	1	1	1	1	1	1	1	0	15	15	0	7	5	3	upstream_ge	MODIFIER	Pp3c16_202	Pp3c16_202	transcript	PAC:3298574	protein_codi	NA	c.-4075A>G	NA	NA	NA	NA	3880
Chr16	1305108	A	G	Chr16_13051A_G	SNP	12480.63	10	7	5	12	10	22	24	19	13	19	15	10	10	7	10	7	5	12	10	22	24	19	13	19	15	10	10	7	1	1	1	1	1	1	1	1	1	1	1	1	1	1	0	15	15	0	7	5	3	upstream_ge	MODIFIER	Pp3c16_202	Pp3c16_202	transcript	PAC:3298574	protein_codi	NA	c.-4476A>G	NA	NA	NA	NA	3635
Chr16	1305108	A	G	Chr16_13051A_G	SNP	12480.63	10	7	5	12	10	22	24	19	13	19	15	10	10	7	10	7	5	12	10	22	24	19	13	19	15	10	10	7	1	1	1	1	1	1	1	1	1	1	1	1	1	1	0	15	15	0	7	5	3	upstream_ge	MODIFIER	Pp3c16_202	Pp3c16_202	transcript	PAC:3298574	protein_codi	NA	c.-4476A>G	NA	NA	NA	NA	3712
Chr16	1305108	A	G	Chr16_13051A_G	SNP	12480.63	10	7	5	12	10	22	24	19	13	19	15	10	10	7	10	7	5	12	10	22	24	19	13	19	15	10	10	7	1	1	1	1	1	1	1	1	1	1	1	1	1	1	0	15	15	0	7	5	3	upstream_ge	MODIFIER	Pp3c16_202	Pp3c16_202	transcript	PAC:3298574	protein_codi	NA	c.-4476A>G	NA	NA	NA	NA	3712
Chr16	1305108	A	G	Chr16_13051A_G	SNP	12480.63	10	7	5	12	10	22	24	19	13	19	15	10	10	7	10	7	5	12	10	22	24	19	13	19	15	10	10	7	1	1	1	1	1	1	1	1	1	1	1	1	1	1	0	15	15	0	7	5	3	upstream_ge	MODIFIER	Pp3c16_202	Pp3c16_202	transcript	PAC:3298574	protein_codi	NA	c.-4476A>G	NA	NA	NA	NA	3748
Chr16	1305108	A	G	Chr16_13051A_G	SNP	12480.63	10	7	5	12	10	22	24	19	13	19	15	10	10	7	10	7	5	12	10	22	24	19	13	19	15	10	10	7	1	1	1	1	1	1	1	1	1	1	1	1	1	1	0	15	15	0	7	5	3	upstream_ge	MODIFIER	Pp3c16_202	Pp3c16_202	transcript	PAC:3298574	protein_codi	NA	c.-4476A>G	NA	NA	NA	NA	3807
Chr16	1305108	A	G	Chr16_13051A_G	SNP	12480.63	10	7	5	12	10	22	24	19	13	19	15	10	10	7	10	7	5	12	10	22	24	19	13	19	15	10	10	7	1	1	1	1	1	1	1	1	1	1	1	1	1	1	0	15	15	0	7	5	3	upstream_ge	MODIFIER	Pp3c16_202	Pp3c16_202	transcript	PAC:3298574	protein_codi	NA	c.-4476A>G	NA	NA	NA	NA	3843
Chr16	1305108	A	G	Chr16_13051A_G	SNP	12480.63	10	7	5	12	10	22	24	19	13	19	15	10	10	7	10	7	5	12	10	22	24	19	13	19	15	10	10	7	1	1	1	1	1	1	1	1	1	1	1	1	1	1	0	15	15	0	7	5	3	downstream_	MODIFIER	Pp3c16_1971	Pp3c16_1971	transcript	PAC:3298665	protein_codi	NA	c.*4441A>G	NA	NA	NA	NA	3564
Chr16	1305108	A	G	Chr16_13051A_G	SNP	12480.63	10	7	5	12	10	22	24	19	13	19	15	10	10	7	10	7	5	12	10	22	24	19	13	19	15	10	10	7	1	1	1	1	1	1	1	1	1	1	1	1	1	1	0	15	15	0	7	5	3	downstream_	MODIFIER	Pp3c16_197	Pp3c16_197	transcript	PAC:3298390	protein_codi	NA	c.*1256A>G	NA	NA	NA	NA	1249
Chr16	1305108	A	G	Chr16_13051A_G	SNP	12480.63	10	7	5	12	10	22	24	19	13	19	15	10	10	7	10	7	5	12	10	22	24	19	13	19	15	10	10	7	1	1	1	1	1	1	1	1	1	1	1	1	1	1	0	15	15	0	7	5	3	downstream_	MODIFIER	Pp3c16_200	Pp3c16_200	transcript	PAC:3298484	protein_codi	NA	c.*538T>C	NA	NA	NA	NA	328
Chr16	1305108	A	G	Chr16_13051A_G	SNP	12480.63	10	7	5	12	10	22	24	19	13	19	15	10	10	7	10	7	5	12	10	22	24	19	13	19	15	10	10	7	1	1	1	1	1	1	1	1	1	1	1	1	1	1	0	15	15	0	7	5	3	downstream_	MODIFIER	Pp3c16_1971	Pp3c16_1971	transcript	PAC:3298665	protein_codi	NA	c.*4441A>G	NA	NA	NA	NA	3564
Chr16	1305108	A	G	Chr16_13051A_G	SNP	12480.63	10	7	5	12	10	22	24	19	13	19	15	10	10	7	10	7	5	12	10	22	24	19	13	19	15	10	10	7	1	1	1	1	1	1	1	1	1	1	1	1	1	1	0	15	15	0	7	5	3	downstream_	MODIFIER	Pp3c16_199	Pp3c16_199	transcript	PAC:3298384	protein_codi	NA	c.*173A>G	NA	NA	NA	NA	173
Chr16	1305108	A	G	Chr16_13051A_G	SNP	12480.63	10	7	5	12	10	22	24	19	13	19	15	10	10	7	10	7	5	12	10	22	24	19	13	19	15	10	10	7	1	1	1	1	1	1	1	1	1	1	1	1	1	1	0	15	15	0	7	5	3	intron_varia	MODIFIER	Pp3c16_199	Pp3c16_199	transcript	PAC:3298384	protein_codi	03-juin	c.329-23A>G	NA	NA	NA	NA	NA
Chr16	1305133	G	GA	Chr16_13051G_GA	INDEL	15320.59	16	12	10	17	17	26	32	24	17	22	19	13	11	7	16	12	10	17	17	26	32	24	17	22	19	13	11	7	1	1	1	1	1	1	1	1	1	1	1	1	1	1	0	15	15	0	7	5	3	frameshift_v	HIGH	Pp3c16_199	Pp3c16_199	transcript	PAC:3298384	protein_codi	04-juil	c.332dupA	p.Asp111fs	333/1722	333/1722	111/573	NA

[illegible]

Chr16	4546270	T	TTA	Chr16_45462	T_TTA	INDEL	10629.59	19	11	11	22	11	17	[illegible]
Chr16	4546270	T	TTA	Chr16_45462	T_TTA	INDEL	10629.59	19	11	11	22	11	17	[illegible]
Chr16	4546270	T	TTA	Chr16_45462	T_TTA	INDEL	10629.59	19	11	11	22	11	17	[illegible]
Chr16	4546270	T	TTA	Chr16_45462	T_TTA	INDEL	10629.59	19	11	11	22	11	17	[illegible]
Chr16	4559769	C	CTA	Chr16_45597	C_CTA	INDEL	7414.24	29	12	17	2	0	0	[illegible]
Chr16	4559769	C	CTA	Chr16_45597	C_CTA	INDEL	7414.24	29	12	17	2	0	0	[illegible]
Chr16	4559769	C	CTA	Chr16_45597	C_CTA	INDEL	7414.24	29	12	17	2	0	0	[illegible]
Chr16	4559769	C	CTA	Chr16_45597	C_CTA	INDEL	7414.24	29	12	17	2	0	0	[illegible]
Chr16	4559769	C	CTA	Chr16_45597	C_CTA	INDEL	7414.24	29	12	17	2	0	0	[illegible]
Chr16	4559769	C	CTA	Chr16_45597	C_CTA	INDEL	7414.24	29	12	17	2	0	0	[illegible]
Chr16	4559769	C	CTA	Chr16_45597	C_CTA	INDEL	7414.24	29	12	17	2	0	0	[illegible]
Chr16	4682796	A	AT	Chr16_46827	A_AT	INDEL	15505.59	25	16	12	22	22	35	[illegible]
Chr16	4682796	A	AT	Chr16_46827	A_AT	INDEL	15505.59	25	16	12	22	22	35	[illegible]
Chr16	4689535	T	TA	Chr16_46895	T_TA	INDEL	15509.59	23	11	18	17	10	26	[illegible]
Chr16	4689535	T	TA	Chr16_46895	T_TA	INDEL	15509.59	23	11	18	17	10	26	[illegible]
Chr16	4689576	G	T	Chr16_46895	G_T	SNP	19671.63	28	14	16	24	8	31	[illegible]
Chr16	4689576	G	T	Chr16_46895	G_T	SNP	19671.63	28	14	16	24	8	31	[illegible]
Chr16	4689578	G	T	Chr16_46895	G_T	SNP	19671.63	28	15	17	23	9	31	[illegible]
Chr16	4689578	G	T	Chr16_46895	G_T	SNP	19671.63	28	15	17	23	9	31	[illegible]
Chr16	4771423	C	T	Chr16_47714	C_T	SNP	18473.63	19	14	18	23	25	25	[illegible]
Chr16	4771518	T	C	Chr16_47715	T_C	SNP	13291.63	15	7	15	13	19	22	[illegible]
Chr16	4773725	T	A	Chr16_47737	T_A	SNP	13186.63	17	13	9	14	14	20	[illegible]
[illegible]														

Chr16	8158169	C	CAT	Chr16_81581C_CAT	INDEL	9974.59	30	12	14	13	15	23	14	14	15	22
Chr16	8158169	C	CAT	Chr16_81581C_CAT	INDEL	9974.59	30	12	14	13	15	23	14	14	15	22
Chr16	8171278	C	CATATAT	Chr16_81712C_CATATAT	INDEL	15170.59	22	11	12	11	13	22	31	18	19	14
Chr16	8171278	C	CATATAT	Chr16_81712C_CATATAT	INDEL	15170.59	22	11	12	11	13	22	31	18	19	14
Chr16	8171278	C	CATATAT	Chr16_81712C_CATATAT	INDEL	15170.59	22	11	12	11	13	22	31	18	19	14
Chr16	8198707	AC	A	Chr16_81987AC_A	INDEL	14364.59	14	8	12	15	16	13	31	21	10	19
Chr16	8198707	AC	A	Chr16_81987AC_A	INDEL	14364.59	14	8	12	15	16	13	31	21	10	19
Chr16	8198707	AC	A	Chr16_81987AC_A	INDEL	14364.59	14	8	12	15	16	13	31	21	10	19
Chr16	8198707	AC	A	Chr16_81987AC_A	INDEL	14364.59	14	8	12	15	16	13	31	21	10	19
Chr16	8198707	AC	A	Chr16_81987AC_A	INDEL	14364.59	14	8	12	15	16	13	31	21	10	19
Chr16	8198707	AC	A	Chr16_81987AC_A	INDEL	14364.59	14	8	12	15	16	13	31	21	10	19
Chr16	8198707	AC	A	Chr16_81987AC_A	INDEL	14364.59	14	8	12	15	16	13	31	21	10	19
Chr16	8198711	GC	G	Chr16_81987GC_G	INDEL	14509.59	16	8	12	15	16	13	30	21	10	19
Chr16	8198711	GC	G	Chr16_81987GC_G	INDEL	14509.59	16	8	12	15	16	13	30	21	10	19
Chr16	8198711	GC	G	Chr16_81987GC_G	INDEL	14509.59	16	8	12	15	16	13	30	21	10	19
Chr16	8198711	GC	G	Chr16_81987GC_G	INDEL	14509.59	16	8	12	15	16	13	30	21	10	19
Chr16	8198711	GC	G	Chr16_81987GC_G	INDEL	14509.59	16	8	12	15	16	13	30	21	10	19
Chr16	8198711	GC	G	Chr16_81987GC_G	INDEL	14509.59	16	8	12	15	16	13	30	21	10	19
Chr16	8198711	GC	G	Chr16_81987GC_G	INDEL	14509.59	16	8	12	15	16	13	30	21	10	19
Chr16	8198720	C	G	Chr16_81987C_G	SNP	14753.63	15	7	12	15	14	14	31	20	9	18
Chr16	8198720	C	G	Chr16_81987C_G	SNP	14753.63	15	7	12	15	14	14	31	20	9	18
Chr16	8198720	C	G	Chr16_81987C_G	SNP	14753.63	15	7	12	15	14	14	31	20	9	18
Chr16	8198720	C	G	Chr16_81987C_G	SNP	14753.63	15	7	12	15	14	14	31	20	9	18
Chr16	8198720	C	G	Chr16_81987C_G	SNP	14753.63	15	7	12	15	14	14	31	20	9	18

[illegible]

Chr16	9233984	T	TATC	Chr16_92339T_TATC	INDEL	18544.59	29	15	11	25	11	11	21	26	22	15	10	10	8	15
Chr16	9233984	T	TATC	Chr16_92339T_TATC	INDEL	18544.59	29	15	11	25	11	11	21	26	22	15	10	10	8	15
Chr16	9233984	T	TATC	Chr16_92339T_TATC	INDEL	18544.59	29	15	11	25	11	11	21	26	22	15	10	10	8	15
Chr16	9233984	T	TATC	Chr16_92339T_TATC	INDEL	18544.59	29	15	11	25	11	11	21	26	22	15	10	10	8	15
Chr16	9233984	T	TATC	Chr16_92339T_TATC	INDEL	18544.59	29	15	11	25	11	11	21	26	22	15	10	10	8	15
Chr16	9574198	C	T	Chr16_95741C_T	SNP	13605.63	16	5	8	20	19	25	21	13	20	17	8	16	6	6
Chr16	9574248	T	TA	Chr16_95742T_TA	INDEL	11490.59	16	7	14	26	18	22	24	16	23	19	9	15	6	9
Chr16	9673089	G	T	Chr16_96730G_T	SNP	4586.98	0	0	0	0	0	0	0	0	26	21	22	19	8	13
Chr16	9694720	T	C	Chr16_96947T_C	SNP	18750.63	26	23	18	26	17	29	19	32	24	30	11	23	14	8
Chr16	9697166	CT	C	Chr16_96971CT_C	INDEL	19755.59	22	25	12	20	21	24	27	29	18	19	17	20	13	10
Chr16	9697178	T	TA	Chr16_96971T_TA	INDEL	20985.59	24	25	12	20	22	24	29	31	22	22	17	22	14	9
Chr16	9697236	G	T	Chr16_96972G_T	SNP	19681.63	25	23	14	22	18	24	30	27	23	18	17	24	12	10
Chr16	9697261	G	T	Chr16_96972G_T	SNP	19052.63	27	19	12	17	22	25	29	23	23	13	16	21	8	12
Chr16	9730251	A	AAT	Chr16_97302A_AAT	INDEL	6839.37	33	7	6	20	8	12	13	17	18	8	9	6	7	1
Chr16	9730251	A	AAT	Chr16_97302A_AAT	INDEL	6839.37	33	7	6	20	8	12	13	17	18	8	9	6	7	1
Chr16	9730251	A	AAT	Chr16_97302A_AAT	INDEL	6839.37	33	7	6	20	8	12	13	17	18	8	9	6	7	1
Chr16	9730251	A	AAT	Chr16_97302A_AAT	INDEL	6839.37	33	7	6	20	8	12	13	17	18	8	9	6	7	1
Chr16	9736287	C	CAT	Chr16_97362C_CAT	INDEL	9539.59	22	10	7	14	11	15	22	18	12	24	10	6	7	4
Chr16	9736287	C	CAT	Chr16_97362C_CAT	INDEL	9539.59	22	10	7	14	11	15	22	18	12	24	10	6	7	4
Chr16	9736287	C	CAT	Chr16_97362C_CAT	INDEL	9539.59	22	10	7	14	11	15	22	18	12	24	10	6	7	4
Chr16	9768262	G	T	Chr16_97682G_T	SNP	15520.63	32	11	12	19	14	22	31	12	16	19	14	16	9	7
Chr16	9768262	G	T	Chr16_97682G_T	SNP	15520.63	32	11	12	19	14	22	31	12	16	19	14	16	9	7
Chr16	9768330	A	AT	Chr16_97683A_AT	INDEL	12191.59	20	10	11	18	15	19	32	11	14	16	17	13	10	5
Chr16	9768330	A	AT	Chr16_97683A_AT	INDEL	12191.59	20	10	11	18	15	19	32	11	14	16	17	13	10	5

Chr16	13620515	CAT	C	Chr16_13620CAT_C	INDEL	1665.24	0	0	0	9	11	11	18	8	0	0	0	0	0	0	17	7	9	11	11	11	20	8	10	15	6	6	8	9	0
Chr16	13620515	CAT	C	Chr16_13620CAT_C	INDEL	1665.24	0	0	0	9	11	11	18	8	0	0	0	0	0	0	17	7	9	11	11	11	20	8	10	15	6	6	8	9	0
Chr16	13620515	CAT	C	Chr16_13620CAT_C	INDEL	1665.24	0	0	0	9	11	11	18	8	0	0	0	0	0	0	17	7	9	11	11	11	20	8	10	15	6	6	8	9	0
Chr16	13620515	CAT	C	Chr16_13620CAT_C	INDEL	1665.24	0	0	0	9	11	11	18	8	0	0	0	0	0	0	17	7	9	11	11	11	20	8	10	15	6	6	8	9	0
Chr16	13620515	CAT	C	Chr16_13620CAT_C	INDEL	1665.24	0	0	0	9	11	11	18	8	0	0	0	0	0	0	17	7	9	11	11	11	20	8	10	15	6	6	8	9	0
Chr16	13620515	CAT	C	Chr16_13620CAT_C	INDEL	1665.24	0	0	0	9	11	11	18	8	0	0	0	0	0	0	17	7	9	11	11	11	20	8	10	15	6	6	8	9	0
Chr16	13620515	CAT	C	Chr16_13620CAT_C	INDEL	1665.24	0	0	0	9	11	11	18	8	0	0	0	0	0	0	17	7	9	11	11	11	20	8	10	15	6	6	8	9	0
Chr16	13620515	CAT	C	Chr16_13620CAT_C	INDEL	1665.24	0	0	0	9	11	11	18	8	0	0	0	0	0	0	17	7	9	11	11	11	20	8	10	15	6	6	8	9	0
Chr16	13620515	CAT	C	Chr16_13620CAT_C	INDEL	1665.24	0	0	0	9	11	11	18	8	0	0	0	0	0	0	17	7	9	11	11	11	20	8	10	15	6	6	8	9	0
Chr16	13620515	CAT	C	Chr16_13620CAT_C	INDEL	1665.24	0	0	0	9	11	11	18	8	0	0	0	0	0	0	17	7	9	11	11	11	20	8	10	15	6	6	8	9	0
Chr16	13620515	CAT	C	Chr16_13620CAT_C	INDEL	1665.24	0	0	0	9	11	11	18	8	0	0	0	0	0	0	17	7	9	11	11	11	20	8	10	15	6	6	8	9	0
Chr16	13620515	CAT	C	Chr16_13620CAT_C	INDEL	1665.24	0	0	0	9	11	11	18	8	0	0	0	0	0	0	17	7	9	11	11	11	20	8	10	15	6	6	8	9	0
Chr16	13620515	CAT	C	Chr16_13620CAT_C	INDEL	1665.24	0	0	0	9	11	11	18	8	0	0	0	0	0	0	17	7	9	11	11	11	20	8	10	15	6	6	8	9	0
Chr16	13620515	CAT	C	Chr16_13620CAT_C	INDEL	1665.24	0	0	0	9	11	11	18	8	0	0	0	0	0	0	17	7	9	11	11	11	20	8	10	15	6	6	8	9	0
Chr16	13620515	CAT	C	Chr16_13620CAT_C	INDEL	1665.24	0	0	0	9	11	11	18	8	0	0	0	0	0	0	17	7	9	11	11	11	20	8	10	15	6	6	8	9	0
Chr16	13620515	CAT	C	Chr16_13620CAT_C	INDEL	1665.24	0	0	0	9	11	11	18	8	0	0	0	0	0	0	17	7	9	11	11	11	20	8	10	15	6	6	8	9	0
Chr16	13620515	CAT	C	Chr16_13620CAT_C	INDEL	1665.24	0	0	0	9	11	11	18	8	0	0	0	0	0	0	17	7	9	11	11	11	20	8	10	15	6	6	8	9	0
Chr16	13620515	CAT	C	Chr16_13620CAT_C	INDEL	1665.24	0	0	0	9	11	11	18	8	0	0	0	0	0	0	17	7	9	11	11	11	20	8	10	15	6	6	8	9	0
Chr16	13786628	C	T	Chr16_13786C_T	SNP	12934.63	22	12	12	22	13	15	25	14	15	14	11	12	7	8	22	12	12	22	13	15	25	14	15	14	11	12	7	8	1
Chr16	13788104	A	G	Chr16_13788A_G	SNP	13369.63	22	16	13	17	16	21	19	14	18	14	18	25	9	6	22	16	13	17	16	21	19	14	18	14	18	25	9	6	1
Chr16	14024058	G	T	Chr16_14024G_T	SNP	9542.63	17	10	9	12	12	15	18	10	9	11	8	5	4	7	17	10	9	12	12	15	18	10	9	11	8	5	4	7	1
Chr16	14024059	T	A	Chr16_14024T_A	SNP	9542.63	16	10	9	11	11	14	17	10	9	11	8	5	4	7	16	10	9	11	11	14	17	10	9	11	8	5	4	7	1
Chr16	14101464	A	AAT	Chr16_14101A_AAT	INDEL	2815.93	0	0	0	1	0	0	0	0	18	18	9	7	12	5	30	16	9	27	14	26	27	21	18	18	9	7	12	6	0
Chr16	14101464	A	AAT	Chr16_14101A_AAT	INDEL	2815.93	0	0	0	1	0	0	0	0	18	18	9	7	12	5	30	16	9	27	14	26	27	21	18	18	9	7	12	6	0
Chr16	14101464	A	AAT	Chr16_14101A_AAT	INDEL	2815.93	0	0	0	1	0	0	0	0	18	18	9	7	12	5	30	16	9	27	14	26	27	21	18	18	9	7	12	6	0
Chr16	14101464	A	AAT	Chr16_14101A_AAT	INDEL	2815.93	0	0	0	1	0	0	0	0	18	18	9	7	12	5	30	16	9	27	14	26	27	21	18	18	9	7	12	6	0
Chr16	14101464	A	AAT	Chr16_14101A_AAT	INDEL	2815.93	0	0	0	1	0	0	0	0	18	18	9	7	12	5	30	16	9	27	14	26	27	21	18	18	9	7	12	6	0
Chr16	14101464	A	AAT	Chr16_14101A_AAT	INDEL	2815.93	0	0	0	1	0	0	0	0	18	18	9	7	12	5	30	16	9	27	14	26	27	21	18	18	9	7	12	6	0
Chr16	14101464	A	AAT	Chr16_14101A_AAT	INDEL	2815.93	0	0	0	1	0	0	0	0	18	18	9	7	12	5	30	16	9	27	14	26	27	21	18	18	9	7	12	6	0
Chr16	14101464	A	AAT	Chr16_14101A_AAT	INDEL	2815.93	0	0	0	1	0	0	0	0	18	18	9	7	12	5	30	16	9	27	14	26	27	21	18	18	9	7	12	6	0
Chr16	14101464	A	AAT	Chr16_14101A_AAT	INDEL	2815.93	0	0	0	1	0	0	0	0	18	18	9	7	12	5	30	16	9	27	14	26	27	21	18	18	9	7	12	6	0
Chr16	14347558	A	T	Chr16_14347A_T	SNP	17289.63	27	19	8	23	16	22	28	14	22	18	7	24	18	8	27	19	8	23	16	22	28	14	22	18	7	24	18	8	1
Chr16	14520229	C	T	Chr16_14520C_T	SNP	18147.63	25	9	12	25	20	26	35	23	16	30	11	17	16	7	25	9	12	25	20	26	35	23	16	30	11	17	16	7	1
Chr16	14520390	C	T	Chr16_14520C_T	SNP	18987.63	28	15	16	23	20	21	28	21	13	21	16	25	14	8	28	15	16	23	20	21	28	21	13	21	16	25	14	8	1
Chr16	14612905	C	A	Chr16_14612C_A	SNP	14087.63	12	9	9	18	9	23	20	23	15	26	9	9	9	7	12	9	9	18	9	23	20	23	15	26	9	9	9	7	1
Chr16	14612905	C	A	Chr16_14612C_A	SNP	14087.63	12	9	9	18	9	23	20	23	15	26	9	9	9	7	12	9	9	18	9	23	20	23	15	26	9	9	9	7	1
Chr16	14612905	C	A	Chr16_14612C_A	SNP	14087.63	12	9	9	18	9	23	20	23	15	26	9	9	9	7	12	9	9	18	9	23	20	23	15	26	9	9	9	7	1
Chr16	14612905	C	A	Chr16_14612C_A	SNP	14087.63	12	9	9	18	9	23	20	23	15	26	9	9	9	7	12	9	9	18	9	23	20	23	15	26	9	9	9	7	1
Chr16	14612919	C	A	Chr16_14612C_A	SNP	13241.63	13	9	8	18	10	20	19	22	14	24	9	7	7	5	13	9	8	18	10	20	19	22	14	24	9	7	7	5	1
Chr16	14612919	C	A	Chr16_14612C_A	SNP	13241.63	13	9	8	18	10	20	19	22	14	24	9	7	7	5	13	9	8	18	10	20	19	22	14	24	9	7	7	5	1
Chr16	14612919	C	A	Chr16_14612C_A	SNP	13241.63	13	9	8	18	10	20	19	22	14	24	9	7	7	5	13	9	8	18	10	20	19	22	14	24	9	7	7	5	1
Chr16	14612919	C	A	Chr16_14612C_A	SNP	13241.63	13	9	8	18	10	20	19	22	14	24	9	7	7	5	13	9	8	18	10	20	19	22	14	24	9	7	7	5	1
Chr16	14629377	GT	G	Chr16_14629GT_G	INDEL	11793.59	33	8	9	27	12	34	30	20	15	25	11	22	8	13	33	8	9	27	12	34	30	20	16	25	11	22	8	13	1
Chr16	14629377	GT	G	Chr16_14629GT_G	INDEL	11793.59	33	8	9	27	12	34	30	20	15	25	11	22	8	13	33	8	9	27	12	34	30	20	16	25	11	22	8	13	1
Chr16	14782865	G	GT	Chr16_14782G_GT	INDEL	12576.59	18	12	13	22	18	18	26	22	16	11	19	10	5	5	18	12	13	22	18	18	26	22	16	11	19	10	5	5	1
Chr16	14782865	G	GT	Chr16_14782G_GT	INDEL	12576.59	18	12	13	22	18	18	26	22	16	11	19	10	5	5	18	12	13	22	18	18	26	22	16	11	19	10	5	5	1
Chr16	14782865	G	GT	Chr16_14782G_GT	INDEL	12576.59	18	12	13	22	18	18	26	22	16	11	19	10	5	5	18	12	13	22	18	18	26	22	16	11	19	10	5	5	1
Chr16	14782865	G	GT	Chr16_14782G_GT	INDEL	12576.59	18	12	13	22	18	18	26	22	16	11	19	10	5	5	18	12	13	22	18	18	26	22	16	11	19	10	5	5	1
Chr16	14847694	C	G	Chr16_14847C_G	SNP	13012.63	18	15	9	16	17	15	22	20	19	17	9	22	5	4	18	15	9	16	17	15	22	20	19	17	9	22	5	4	1
Chr16	14886047	C	T	Chr16_14886C_T	SNP	20109.63	19	23	16	29	17	25	26	22	21	24	28	13	11	13	19	23	16	29	17	25	26	22	21	24	28	13	11	13	1
[illegible]																																			

Chr17	1214802	A	AAT	Chr17_12148A_AAT	INDEL	3487.24	0	[illegible]
Chr17	1214802	A	AAT	Chr17_12148A_AAT	INDEL	3487.24	0	[illegible]
Chr17	1214802	A	AAT	Chr17_12148A_AAT	INDEL	3487.24	0	[illegible]
Chr17	1234706	G	GT	Chr17_12347G_GT	INDEL	15333.59	24	[illegible]
Chr17	1234789	C	T	Chr17_12347C_T	SNP	17352.63	35	[illegible]
Chr17	1252984	C	A	Chr17_12529C_A	SNP	20064.63	33	[illegible]
Chr17	1295722	C	CAT	Chr17_12957C_CAT	INDEL	701.37	2	[illegible]
Chr17	1295722	C	CAT	Chr17_12957C_CAT	INDEL	701.37	2	[illegible]
Chr17	1295722	C	CAT	Chr17_12957C_CAT	INDEL	701.37	2	[illegible]
Chr17	1333614	G	GTATA	Chr17_13336G_GTATA	INDEL	10918.59	0	[illegible]
Chr17	1333614	G	GTATA	Chr17_13336G_GTATA	INDEL	10918.59	0	[illegible]
Chr17	1333614	G	GTATA	Chr17_13336G_GTATA	INDEL	10918.59	0	[illegible]
Chr17	1333614	G	GTATA	Chr17_13336G_GTATA	INDEL	10918.59	0	[illegible]
Chr17	1333614	G	GTATA	Chr17_13336G_GTATA	INDEL	10918.59	0	[illegible]
Chr17	1333614	G	GTATA	Chr17_13336G_GTATA	INDEL	10918.59	0	[illegible]
Chr17	1333614	G	GTATA	Chr17_13336G_GTATA	INDEL	10918.59	0	[illegible]
Chr17	1333614	G	GTATA	Chr17_13336G_GTATA	INDEL	10918.59	0	[illegible]
Chr17	1333614	G	GTATA	Chr17_13336G_GTATA	INDEL	10918.59	0	[illegible]
Chr17	1342643	G	A	Chr17_13426G_A	SNP	16316.63	21	[illegible]
Chr17	1342643	G	A	Chr17_13426G_A	SNP	16316.63	21	[illegible]
Chr17	1342643	G	A	Chr17_13426G_A	SNP	16316.63	21	[illegible]
Chr17	1436880	C	CAT	Chr17_14368C_CAT	INDEL	173.37	0	[illegible]
Chr17	1436880	C	CAT	Chr17_14368C_CAT	INDEL	173.37	0	[illegible]
Chr17	1517345	A	AAT	Chr17_15173A_AAT	INDEL	1507.35	29	[illegible]
Chr17	1517345	A	AAT	Chr17_15173A_AAT	INDEL	1507.35	29	[illegible]
Chr17	1517345	A	AAT	Chr17_15173A_AAT	INDEL	1507.35	29	[illegible]
Chr17	1646937	A	T	Chr17_16469A_T	SNP	20416.63	28	[illegible]
Chr17	1745967	G	A	Chr17_17459G_A	SNP	2699.39	40	[illegible]
Chr17	1799511	G	GA	Chr17_17995G_GA	INDEL	17180.59	24	[illegible]
Chr17	2067999	G	T	Chr17_20679G_T	SNP	14894.63	19	[illegible]
Chr17	2068187	G	T	Chr17_20681G_T	SNP	18248.63	30	[illegible]
Chr17	2346365	G	A	Chr17_23463G_A	SNP	5962.29	0	[illegible]
Chr17	2392148	C	G	Chr17_23921C_G	SNP	11692.63	25	[illegible]
Chr17	2395977	A	T	Chr17_23959A_T	SNP	14897.63	21	[illegible]
Chr17	2397582	C	A	Chr17_23975C_A	SNP	10804.63	8	[illegible]
Chr17	2397592	TC	T	Chr17_23975TC_T	INDEL	8221.59	7	[illegible]
Chr17	2411480	A	T	Chr17_24114A_T	SNP	13590.63	27	[illegible]
Chr17	2591717	A	C	Chr17_25917A_C	SNP	16352.63	13	[illegible]
Chr17	2591719	A	T	Chr17_25917A_T	SNP	16352.63	13	[illegible]
Chr17	2677932	G	A	Chr17_26779G_A	SNP	12227.63	15	[illegible]
Chr17	2679373	G	C	Chr17_26793G_C	SNP	16121.63	17	[illegible]
Chr17	2679392	T	A	Chr17_26793T_A	SNP	17607.63	21	[illegible]
Chr17	2679395	T	A	Chr17_26793T_A	SNP	17672.63	19	[illegible]
Chr17	2679421	T	C	Chr17_26794T_C	SNP	16860.63	20	[illegible]
Chr17	2682432	T	G	Chr17_26824T_G	SNP	14201.63	26	[illegible]
Chr17	2682434	C	T	Chr17_26824C_T	SNP	14246.63	26	[illegible]
Chr17	2682446	C	T	Chr17_26824C_T	SNP	12936.63	18	[illegible]
Chr17	2685914	G	T	Chr17_26859G_T	SNP	15611.63	23	[illegible]
Chr17	2689020	T	A	Chr17_26890T_A	SNP	17743.63	20	[illegible]
Chr17	2691515	C	T	Chr17_26915C_T	SNP	17143.63	26	[illegible]
Chr17	2717544	A	G	Chr17_27175A_G	SNP	16295.63	27	[illegible]
Chr17	2717577	T	C	Chr17_27175T_C	SNP	14934.63	27	[illegible]
Chr17	2766718	G	A	Chr17_27667G_A	SNP	19360.63	31	[illegible]
Chr17	2769961	TTTCC	T	Chr17_27699TTTCC_T	INDEL	21913.59	42	[illegible]
Chr17	2769961	TTTCC	T	Chr17_27699TTTCC_T	INDEL	21913.59	42	[illegible]
Chr17	2769968	C	CAACTT	Chr17_27699C_CAACTT	INDEL	22585.59	42	[illegible]
Chr17	2769968	C	CAACTT	Chr17_27699C_CAACTT	INDEL	22585.59	42	[illegible]
Chr17	2904083	A	AAT	Chr17_29040A_AAT	INDEL	4414.24	0	[illegible]
Chr17	2904083	A	AAT	Chr17_29040A_AAT	INDEL	4414.24	0	[illegible]
Chr17	3138400	C	A	Chr17_31384C_A	SNP	14082.63	25	[illegible]
Chr17	3178610	AAT	A	Chr17_31786AAT_A	INDEL	7318.37	19	[illegible]
Chr17	3234124	G	T	Chr17_32341G_T	SNP	18656.63	25	[illegible]
Chr17	3234129	G	T	Chr17_32341G_T	SNP	18835.63	26	[illegible]
Chr17	3234136	G	T	Chr17_32341G_T	SNP	18793.63	27	[illegible]
Chr17	3481785	A	AAT	Chr17_34817A_AAT	INDEL	3820.59	10	[illegible]
Chr17	3578159	AAT	A	Chr17_35781AAT_A	INDEL	7775.59	13	[illegible]
Chr17	3578159	AAT	A	Chr17_35781AAT_A	INDEL	7775.59	13	[illegible]
Chr17	3578159	AAT	A	Chr17_35781AAT_A	INDEL	7775.59	13	[illegible]
Chr17	3578159	AAT	A	Chr17_35781AAT_A	INDEL	7775.59	13	[illegible]
Chr17	3578159	AAT	A	Chr17_35781AAT_A	INDEL	7775.59	13	[illegible]
Chr17	3578159	AAT	A	Chr17_35781AAT_A	INDEL	7775.59	13	[illegible]
Chr17	3578159	AAT	A	Chr17_35781AAT_A	INDEL	7775.59	13	[illegible]
Chr17	3594185	A	C	Chr17_35941A_C	SNP	19805.63	27	[illegible]
Chr17	3594196	A	C	Chr17_35941A_C	SNP	19994.63	27	[illegible]
Chr17	3604626	GA	G	Chr17_36046GA_G	INDEL	16059.59	29	[illegible]
Chr17	3604626	GA	G	Chr17_36046GA_G	INDEL	16059.59	29	[illegible]
Chr17	3604630	T	TCTAGCAGT	Chr17_36046T_TCTAGCAG	INDEL	17590.59	29	[illegible]
Chr17	3604630	T	TCTAGCAGT	Chr17_36046T_TCTAGCAG	INDEL	17590.59	29	[illegible]
Chr17	3604632	T	C	Chr17_36046T_C	SNP	17824.63	29	[illegible]
Chr17	3604632	T	C	Chr17_36046T_C	SNP	17824.63	29	[illegible]
Chr17	3604636	C	G	Chr17_36046C_G	SNP	16400.63	29	[illegible]
Chr17	3604636	C	G	Chr17_36046C_G	SNP	16400.63	29	[illegible]
Chr17	3604638	T	C	Chr17_36046T_C	SNP	16355.63	29	[illegible]
Chr17	3604638	T	C	Chr17_36046T_C	SNP	16355.63	29	[illegible]
Chr17	3604640	C	G	Chr17_36046C_G	SNP	16445.63	29	[illegible]
Chr17	3604640	C	G	Chr17_36046C_G	SNP	16445.63	29	[illegible]
Chr17	3604643	A	G	Chr17_36046A_G	SNP	16040.63	28	[illegible]
Chr17	3604643	A	G	Chr17_36046A_G	SNP	16040.63	28	[illegible]
Chr17	3604644	A	G	Chr17_36046A_G	SNP	16085.63	28	[illegible]
Chr17	3604644	A	G	Chr17_36046A_G	SNP	16085.63	28	[illegible]
Chr17	3604645	C	G	Chr17_36046C_G	SNP	15995.63	27	[illegible]
Chr17	3604645	C	G	Chr17_36046C_G	SNP	15995.63	27	[illegible]
Chr17	3604647	TATAA	T	Chr17_36046TATAA_T	INDEL	16526.59	27	[illegible]
Chr17	3604647	TATAA	T	Chr17_36046TATAA_T	INDEL	16526.59	27	[illegible]
Chr17	3604652	C	CAG	Chr17_36046C_CAG	INDEL	16166.59	26	[illegible]
Chr17	3604652	C	CAG	Chr17_36046C_CAG	INDEL	16166.59	26	[illegible]
Chr17	3604655	T	TACC	Chr17_36046T_TACC	INDEL	16391.59	26	[illegible]
Chr17	3604655	T	TACC	Chr17_36046T_TACC	INDEL	16391.59	26	[illegible]
Chr17	3604657	T	G	Chr17_36046T_G	SNP	16445.63	27	[illegible]
Chr17	3604657	T	G	Chr17_36046T_G	SNP	16445.63	27	[illegible]
Chr17	3604661	T	TG	Chr17_36046T_TG	INDEL	15851.59	26	[illegible]
Chr17	3604661	T	TG	Chr17_36046T_TG	INDEL	15851.59	26	[illegible]
Chr17	3604664	TTAATTTCCA	T	Chr17_36046TTAATTTCCA	INDEL	14771.59	26	[illegible]
Chr17	3604664	TTAATTTCCA	T	Chr17_36046TTAATTTCCA	INDEL	14771.59	26	[illegible]
Chr17	3688824	C	CAT	Chr17_36888C_CAT	INDEL	1649.93	0	[illegible]
Chr17	3688824	C	CAT	Chr17_36888C_CAT	INDEL	1649.93	0	[illegible]
Chr17	3688824	C	CAT	Chr17_36888C_CAT	INDEL	1649.93	0	[illegible]
Chr17	3688824	C	CAT	Chr17_36888C_CAT	INDEL	1649.93	0	[illegible]
Chr17	3688824	C	CAT	Chr17_36888C_CAT	INDEL	1649.93	0	[illegible]
Chr17	3688824	C	CAT	Chr17_36888C_CAT	INDEL	1649.93	0	[illegible]
Chr17	3688824	C	CAT	Chr17_36888C_CAT	INDEL	1649.93	0	[illegible]
Chr17	3727666	GTA	G	Chr17_37276GTA_G	INDEL	217.37	2	[illegible]
Chr17	3727666	GTA	G	Chr17_37276GTA_G	INDEL	217.37	2	[illegible]
Chr17	3727666	GTA	G	Chr17_37276GTA_G	INDEL	217.37	2	[illegible]
Chr17	4103714	G	GA	Chr17_41037G_GA	INDEL	19535.59	24	[illegible]
Chr17	4195893	G	T	Chr17_41958G_T	SNP	8866.63	17	[illegible]
Chr17	4390107	T	TTA	Chr17_43901T_TTA	INDEL	1476.93	1	[illegible]
Chr17	4390107	T	TTA	Chr17_43901T_TTA	INDEL	1476.93	1	[illegible]
Chr17	4390107	T	TTA	Chr17_43901T_TTA	INDEL	1476.93	1	[illegible]
Chr17	4390107	T	TTA	Chr17_43901T_TTA	INDEL	1476.93	1	[illegible]
Chr17	4390107	T	TTA	Chr17_43901T_TTA	INDEL	1476.93	1	[illegible]
Chr17	4390107	T	TTA	Chr17_43901T_TTA	INDEL	1476.93	1	[illegible]
Chr17	4390107	T	TTA	Chr17_43901T_TTA	INDEL	1476.93	1	[illegible]
Chr17	4390107	T	TTA	Chr17_43901T_TTA	INDEL	1476.93	1	[illegible]
Chr17	4390107	T	TTA	Chr17_43901T_TTA	INDEL	1476.93	1	[illegible]
Chr17	4390107	T	TTA	Chr17_43901T_TTA	INDEL	1476.93	1	[illegible]
Chr17	4390107	T	TTA	Chr17_43901T_TTA	INDEL	1476.93	1	[illegible]
Chr17	4390107	T	TTA	Chr17_43901T_TTA	INDEL	1476.93	1	[illegible]
Chr17	4390107	T	TTA	Chr17_43901T_TTA	INDEL	1476.93	1	[illegible]
Chr17	4473493	ATTTGAG	A	Chr17_44734ATTTGAG_A	INDEL	17752.59	28	[illegible]
Chr17	4473509	G	A	Chr17_44735G_A	SNP	17609.63	27	[illegible]
Chr17	4473515	A	AG	Chr17_44735A_AG	INDEL	16841.59	27	[illegible]
Chr17	4473516	A	ATTTG	Chr17_44735A_ATTTG	INDEL	16751.59	27	[illegible]
Chr17	4473519	T	TTA	Chr17_44735T_TTA	INDEL	15549.59	26	[illegible]
Chr17	4547503	C	CA	Chr17_45475C_CA	INDEL	15308.59	22	[illegible]
Chr17	4742183	C	CA	Chr17_47421C_CA	INDEL	12897.59	25	[illegible]
Chr17	4742183	C	CA	Chr17_47421C_CA	INDEL	12897.59	25	[illegible]
Chr17	4742183	C	CA	Chr17_47421C_CA	INDEL	12897.59	25	[illegible]
Chr17	4742223	A	T	Chr17_47422A_T	SNP	18401.63	24	[illegible]
Chr17	4742223	A	T	Chr17_47422A_T	SNP	18401.63	24	[illegible]
Chr17	4742223	A	T	Chr17_47422A_T	SNP	18401.63	24	[illegible]
Chr17	4779508	T	TC	Chr17_47795T_TC	INDEL	16709.59	22	[illegible]
Chr17	4870136	G	GAT	Chr17_48701G_GAT	INDEL	9354.59	19	[illegible]
Chr17	4870136	G	GAT	Chr17_48701G_GAT	INDEL	9354.59	19	[illegible]
Chr17	4870136	G	GAT	Chr17_48701G_GAT	INDEL	9354.59	19	[illegible]
Chr17	4870136	G	GAT	Chr17_48701G_GAT	INDEL	9354.59	19	[illegible]
Chr17	4924849	CTA	C	Chr17_49248CTA_C	INDEL	1379.35	22	[illegible]
Chr17	4988512	T	TTA	Chr17_49885T_TTA	INDEL	799.35	13	[illegible]
Chr17	4988512	T	TTA	Chr17_49885T_TTA	INDEL	799.35	13	[illegible]
Chr17	4988512	T	TTA	Chr17_49885T_TTA	INDEL	799.35	13	[illegible]
Chr17	4988512	T	TTA	Chr17_49885T_TTA	INDEL	799.35	13	[illegible]
Chr17	4988512	T	TTA	Chr17_49885T_TTA	INDEL	799.35	13	[illegible]
Chr17	4988512	T	TTA	Chr17_49885T_TTA	INDEL	799.35	13	[illegible]
Chr17	4988512	T	TTA	Chr17_49885T_TTA	INDEL	799.35	13	[illegible]
Chr17	4988512	T	TTA	Chr17_49885T_TTA	INDEL	799.35	13	[illegible]
Chr17	4988512	T	TTA	Chr17_49885T_TTA	INDEL	799.35	13	[illegible]
Chr17	4988512	T	TTA	Chr17_49885T_TTA	INDEL	799.35	13	[illegible]
Chr17	4988512	T	TTA	Chr17_49885T_TTA	INDEL	799.35	13	[illegible]
Chr17	4988512	T	TTA	Chr17_49885T_TTA	INDEL	799.35	13	[illegible]
Chr17	4988512	T	TTA	Chr17_49885T_TTA	INDEL	799.35	13	[illegible]
Chr17	4988512	T	TTA	Chr17_49885T_TTA	INDEL	799.35	13	[illegible]
Chr17	4988512	T	TTA	Chr17_49885T_TTA	INDEL	799.35	13	[illegible]
Chr17	4988512	T	TTA	Chr17_49885T_TTA	INDEL	799.35	13	[illegible]
Chr17	4988512	T	TTA	Chr17_49885T_TTA	INDEL	799.35	13	[illegible]
Chr17	4992971	C	G	Chr17_49929C_G	SNP	20620.63	26	[illegible]
Chr17	4992971	C	G	Chr17_49929C_G	SNP	20620.63	26	[illegible]
Chr17	4992971	C	G	Chr17_49929C_G	SNP	20620.63	26	[illegible]
Chr17	4992971	C	G	Chr17_49929C_G	SNP	20620.63	26	[illegible]
Chr17	4992971	C	G	Chr17_49929C_G	SNP	20620.63	26	[illegible]
Chr17	4992971	C	G	Chr17_49929C_G	SNP	20620.63	26	[illegible]
Chr17	4992971	C	G	Chr17_49929C_G	SNP	20620.63	26	[illegible]
Chr17	4992971	C	G	Chr17_49929C_G	SNP	20620.63	26	[illegible]
Chr17	4992971	C	G	Chr17_49929C_G	SNP	20620.63	26	[illegible]
Chr17	4992971	C	G	Chr17_49929C_G	SNP	20620.63	26	[illegible]
Chr17	4992973	G	T	Chr17_49929G_T	SNP	20620.63	27	[illegible]
Chr17	4992973	G	T	Chr17_49929G_T	SNP	20620.63	27	[illegible]
Chr17	4992973	G	T	Chr17_49929G_T	SNP	20620.63	27	[illegible]
Chr17	4992973	G	T	Chr17_49929G_T	SNP	20620.63	27	[illegible]
Chr17	4992973	G	T	Chr17_49929G_T	SNP	20620.63	27	[illegible]
Chr17	4992973	G	T	Chr17_49929G_T	SNP	20620.63	27	[illegible]
Chr17	4992973	G	T	Chr17_49929G_T	SNP	20620.63	27	[illegible]
Chr17	4992973	G	T	Chr17_49929G_T	SNP	20620.63	27	[illegible]
Chr17	4992973	G	T	Chr17_49929G_T	SNP	20620.63	27	[illegible]
Chr17	4992973	G	T	Chr17_49929G_T	SNP	20620.63	27	[illegible]
Chr17	5164037	A	G	Chr17_51640A_G	SNP	18571.63	31	[illegible]
Chr17	5207476	CAT	C	Chr17_52074CAT_C	INDEL	8171.59	19	[illegible]
Chr17	5207476	CAT	C	Chr17_52074CAT_C	INDEL	8171.59	19	[illegible]
Chr17	5207476	CAT	C	Chr17_52074CAT_C	INDEL	8171.59	19	[illegible]
Chr17	5207476	CAT	C	Chr17_52074CAT_C	INDEL	8171.59	19	[illegible]
Chr17	5207476	CAT	C	Chr17_52074CAT_C	INDEL	8171.59	19	[illegible]
Chr17	5207476	CAT	C	Chr17_52074CAT_C	INDEL	8171.59	19	[illegible]
Chr17	5207476	CAT	C	Chr17_52074CAT_C	INDEL	8171.59	19	[illegible]
Chr17	5284815	T	TA	Chr17_52848T_TA	INDEL	13496.59	28	[illegible]
Chr17	5470620	C	A	Chr17_54706C_A	SNP	5432.29	0	[illegible]
Chr17	5473842	C	T	Chr17_54738C_T	SNP	18281.63	22	[illegible]
Chr17	5585483	T	C	Chr17_55854T_C	SNP	16875.63	25	[illegible]
Chr17	5585483	T	C	Chr17_55854T_C	SNP	16875.63	25	[illegible]
Chr17	5603635	C	A	Chr17_56036C_A	SNP	21802.63	45	[illegible]
Chr17	5603635	C	A	Chr17_56036C_A	SNP	21802.63	45	[illegible]
Chr17	5603635	C	A	Chr17_56036C_A	SNP	21802.63	45	[illegible]
Chr17	5603635	C	A	Chr17_56036C_A	SNP	21802.63	45	[illegible]
Chr17	5884784	C	T	Chr17_58847C_T	SNP	11516.63	13	[illegible]

Chr17	5886442	G	T	Chr17_58864	G_T	SNP	17700.63	17	10	14	32	15	26	22	17	13	26
Chr17	5886445	T	TA	Chr17_58864	T_TA	INDEL	18360.59	17	10	15	32	16	27	22	17	11	27
Chr17	5886463	G	GC	Chr17_58864	G_GC	INDEL	18056.59	17	11	11	34	13	24	23	18	16	25
Chr17	5886467	C	CA	Chr17_58864	C_CA	INDEL	17921.59	17	12	11	31	13	22	20	18	17	25
Chr17	5886469	T	A	Chr17_58864	T_A	SNP	17795.63	17	10	11	29	13	22	20	19	16	25
Chr17	5886475	C	CA	Chr17_58864	C_CA	INDEL	17561.59	17	11	11	28	16	22	20	19	17	24
Chr17	5886476	T	TA	Chr17_58864	T_TA	INDEL	17561.59	18	11	12	30	17	22	20	20	17	23
Chr17	5886479	T	A	Chr17_58864	T_A	SNP	17255.63	17	11	12	31	17	21	19	21	16	23
Chr17	5886481	T	A	Chr17_58864	T_A	SNP	17210.63	18	11	12	32	16	21	20	21	15	24
Chr17	5886483	T	A	Chr17_58864	T_A	SNP	17345.63	18	13	12	31	16	22	18	21	15	24
Chr17	5886485	T	A	Chr17_58864	T_A	SNP	17390.63	18	12	11	32	15	23	19	21	15	22
Chr17	5886492	ACTTT	A	Chr17_58864	ACTTT_A	INDEL	17353.59	19	12	11	29	15	21	21	23	16	22
Chr17	5886498	T	C	Chr17_58864	T_C	SNP	16912.63	19	12	10	28	16	21	21	22	18	22
Chr17	5886569	A	AT	Chr17_58865	A_AT	INDEL	15121.59	23	13	16	26	17	24	22	25	31	31
Chr17	5994652	C	T	Chr17_59946	C_T	SNP	6845.29	0	0	0	27	21	36	31	35	0	0
Chr17	6053771	C	T	Chr17_60537	C_T	SNP	6186.29	0	0	0	25	33	23	22	19	0	0
Chr17	6078615	C	T	Chr17_60786	C_T	SNP	4130.29	0	0	0	18	11	17	20	15	0	0
Chr17	6143210	A	AAT	Chr17_61432	A_AAT	INDEL	6942.37	14	8	6	20	10	15	17	15	15	9
Chr17	6230448	A	T	Chr17_62304	A_T	SNP	10366.63	18	5	10	7	19	16	18	18	12	10
Chr17	6508713	A	AAT	Chr17_65087	A_AAT	INDEL	2432.24	1	0	0	11	7	15	24	13	1	0
Chr17	6508713	A	AAT	Chr17_65087	A_AAT	INDEL	2432.24	1	0	0	11	7	15	24	13	1	0
Chr17	6554855	T	TA	Chr17_65548	T_TA	INDEL	13888.59	24	21	14	18	17	26	42	21	13	30
Chr17	6564078	G	T	Chr17_65640	G_T	SNP	21151.63	43	11	18	27	23	31	31	29	25	21
Chr17	6654534	C	CA	Chr17_66545	C_CA	INDEL	17220.59	28	19	17	20	23	32	29	24	35	25
[illegible]																	

Chr17	9824027	T	A	Chr17_98240	T_A	SNP	17757.63	30	15	18
Chr17	9824027	T	A	Chr17_98240	T_A	SNP	17757.63	30	15	18
Chr17	9824032	C	A	Chr17_98240	C_A	SNP	17671.63	29	14	18
Chr17	9824032	C	A	Chr17_98240	C_A	SNP	17671.63	29	14	18
Chr17	9824052	G	GA	Chr17_98240	G_GA	INDEL	13697.59	27	13	16
Chr17	9824052	G	GA	Chr17_98240	G_GA	INDEL	13697.59	27	13	16
Chr17	9824105	G	GA	Chr17_98241	G_GA	INDEL	16286.59	32	8	18
Chr17	9824105	G	GA	Chr17_98241	G_GA	INDEL	16286.59	32	8	18
Chr17	9879451	A	AAT	Chr17_98794	A_AAT	INDEL	3437.24	1	0	0
Chr17	9879451	A	AAT	Chr17_98794	A_AAT	INDEL	3437.24	1	0	0
Chr17	9879451	A	AAT	Chr17_98794	A_AAT	INDEL	3437.24	1	0	0
Chr17	9879451	A	AAT	Chr17_98794	A_AAT	INDEL	3437.24	1	0	0
Chr17	9879451	A	AAT	Chr17_98794	A_AAT	INDEL	3437.24	1	0	0
Chr17	9879451	A	AAT	Chr17_98794	A_AAT	INDEL	3437.24	1	0	0
Chr17	9879451	A	AAT	Chr17_98794	A_AAT	INDEL	3437.24	1	0	0
Chr17	9907424	C	CT	Chr17_99074	C_CT	INDEL	16652.59	28	17	18
Chr17	9907471	C	T	Chr17_99074	C_T	SNP	19912.63	36	17	14
Chr17	9913097	A	T	Chr17_99130	A_T	SNP	13945.29	36	11	21
Chr17	9913097	A	T	Chr17_99130	A_T	SNP	13945.29	36	11	21
Chr17	9913097	A	T	Chr17_99130	A_T	SNP	13945.29	36	11	21
Chr17	9913097	A	T	Chr17_99130	A_T	SNP	13945.29	36	11	21
Chr17	9913097	A	T	Chr17_99130	A_T	SNP	13945.29	36	11	21
Chr17	10061204	A	T	Chr17_10061	A_T	SNP	9555.63	12	7	10
Chr17	10064463	T	TA	Chr17_10064	T_TA	INDEL	16711.59	25	18	15
Chr17	10178515	AAT	A	Chr17_10178	AAT_A	INDEL	1057.35	17	10	9
Chr17	10178515	AAT	A	Chr17_10178	AAT_A	INDEL	1057.35	17	10	9
Chr17	10178515	AAT	A	Chr17_10178	AAT_A	INDEL	1057.35	17	10	9
Chr17	10178515	AAT	A	Chr17_10178	AAT_A	INDEL	1057.35	17	10	9
Chr17	10266393	G	A	Chr17_10266	G_A	SNP	6240.29	0	0	0
Chr17	10291529	TG	T	Chr17_10291	TG_T	INDEL	10749.59	18	4	11
Chr17	10291538	T	A	Chr17_10291	T_A	SNP	10309.63	16	5	11
Chr17	10291564	T	A	Chr17_10291	T_A	SNP	9597.63	13	7	10
Chr17	10291575	T	A	Chr17_10291	T_A	SNP	10618.63	15	8	11
Chr17	10291591	G	A	Chr17_10291	G_A	SNP	10876.63	18	7	11
Chr17	10291602	T	TA	Chr17_10291	T_TA	INDEL	8449.59	16	5	8
Chr17	10291614	G	GA	Chr17_10291	G_GA	INDEL	10092.59	18	5	12
Chr17	10291634	G	T	Chr17_10291	G_T	SNP	14174.63	21	7	14
Chr17	10413815	G	T	Chr17_10413	G_T	SNP	17623.63	28	20	10
Chr17	10422037	A	T	Chr17_10422	A_T	SNP	14409.63	17	17	22
Chr17	10518242	T	TG	Chr17_10518	T_TG	INDEL	17111.59	13	20	15
Chr17	10518242	T	TG	Chr17_10518	T_TG	INDEL	17111.59	13	20	15
Chr17	10518242	T	TG	Chr17_10518	T_TG	INDEL	17111.59	13	20	15
Chr17	10518243	A	AG	Chr17_10518	A_AG	INDEL	17111.59	13	20	15
Chr17	10518243	A	AG	Chr17_10518	A_AG	INDEL	17111.59	13	20	15
Chr17	10518243	A	AG	Chr17_10518	A_AG	INDEL	17111.59	13	20	15
Chr17	10518247	C	G	Chr17_10518	C_G	SNP	17345.63	14	22	15
Chr17	10518247	C	G	Chr17_10518	C_G	SNP	17345.63	14	22	15
Chr17	10518247	C	G	Chr17_10518	C_G	SNP	17345.63	14	22	15
Chr17	10518248	TTA	T	Chr17_10518	TTA_T	INDEL	18731.59	13	21	15
Chr17	10518248	TTA	T	Chr17_10518	TTA_T	INDEL	18731.59	13	21	15
Chr17	10518248	TTA	T	Chr17_10518	TTA_T	INDEL	18731.59	13	21	15
Chr17	10518251	A	AGCCCTGGC	Chr17_10518	A_AGCCCTG	INDEL	18506.59	13	21	15
Chr17	10518251	A	AGCCCTGGC	Chr17_10518	A_AGCCCTG	INDEL	18506.59	13	21	15
Chr17	10518251	A	AGCCCTGGC	Chr17_10518	A_AGCCCTG	INDEL	18506.59	13	21	15
Chr17	10518254	A	AGGGG	Chr17_10518	A_AGGGG	INDEL	17876.59	13	21	16
Chr17	10518254	A	AGGGG	Chr17_10518	A_AGGGG	INDEL	17876.59	13	21	16
Chr17	10518254	A	AGGGG	Chr17_10518	A_AGGGG	INDEL	17876.59	13	21	16
Chr17	10518255	CATGTTTT	C	Chr17_10518	CATGTTTT_C	INDEL	18011.59	12	23	13
Chr17	10518255	CATGTTTT	C	Chr17_10518	CATGTTTT_C	INDEL	18011.59	12	23	13
Chr17	10518255	CATGTTTT	C	Chr17_10518	CATGTTTT_C	INDEL	18011.59	12	23	13
Chr17	10518266	A	T	Chr17_10518	A_T	SNP	17210.63	13	23	14
Chr17	10518266	A	T	Chr17_10518	A_T	SNP	17210.63	13	23	14
Chr17	10518266	A	T	Chr17_10518	A_T	SNP	17210.63	13	23	14
Chr17	10518268	T	C	Chr17_10518	T_C	SNP	17030.63	14	23	14
Chr17	10518268	T	C	Chr17_10518	T_C	SNP	17030.63	14	23	14
Chr17	10518268	T	C	Chr17_10518	T_C	SNP	17030.63	14	23	14
Chr17	10518270	ATGT	A	Chr17_10518	ATGT_A	INDEL	17201.59	14	22	14
Chr17	10518270	ATGT	A	Chr17_10518	ATGT_A	INDEL	17201.59	14	22	14
Chr17	10518270	ATGT	A	Chr17_10518	ATGT_A	INDEL	17201.59	14	22	14
Chr17	10518274	G	GCC	Chr17_10518	G_GCC	INDEL	17156.59	14	21	14
Chr17	10518274	G	GCC	Chr17_10518	G_GCC	INDEL	17156.59	14	21	14
Chr17	10518274	G	GCC	Chr17_10518	G_GCC	INDEL	17156.59	14	21	14
Chr17	10518276	T	G	Chr17_10518	T_G	SNP	17705.63	15	21	14
Chr17	10518276	T	G	Chr17_10518	T_G	SNP	17705.63	15	21	14
Chr17	10518276	T	G	Chr17_10518	T_G	SNP	17705.63	15	21	14
Chr17	10518281	A	T	Chr17_10518	A_T	SNP	17930.63	16	20	14
Chr17	10518281	A	T	Chr17_10518	A_T	SNP	17930.63	16	20	14
Chr17	10518281	A	T	Chr17_10518	A_T	SNP	17930.63	16	20	14
Chr17	10518282	G	GC	Chr17_10518	G_GC	INDEL	18011.59	17	20	15
Chr17	10518282	G	GC	Chr17_10518	G_GC	INDEL	18011.59	17	20	15
Chr17	10518282	G	GC	Chr17_10518	G_GC	INDEL	18011.59	17	20	15
Chr17	10518285	GTAT	G	Chr17_10518	GTAT_G	INDEL	17966.59	18	20	14
Chr17	10518285	GTAT	G	Chr17_10518	GTAT_G	INDEL	17966.59	18	20	14
Chr17	10518285	GTAT	G	Chr17_10518	GTAT_G	INDEL	17966.59	18	20	14
Chr17	10518290	GT	G	Chr17_10518	GT_G	INDEL	17696.59	18	20	14
Chr17	10518290	GT	G	Chr17_10518	GT_G	INDEL	17696.59	18	20	14
Chr17	10518290	GT	G	Chr17_10518	GT_G	INDEL	17696.59	18	20	14
Chr17	10518292	ATGTTTTT	A	Chr17_10518	ATGTTTTT_A	INDEL	18011.59	18	20	14
Chr17	10518292	ATGTTTTT	A	Chr17_10518	ATGTTTTT_A	INDEL	18011.59	18	20	14
Chr17	10518292	ATGTTTTT	A	Chr17_10518	ATGTTTTT_A	INDEL	18011.59	18	20	14
Chr17	10518300	C	CGAG	Chr17_10518	C_CGAG	INDEL	17876.59	18	19	14
Chr17	10518300	C	CGAG	Chr17_10518	C_CGAG	INDEL	17876.59	18	19	14
Chr17	10518300	C	CGAG	Chr17_10518	C_CGAG	INDEL	17876.59	18	19	14
Chr17	10518303	CTTGTTT	C	Chr17_10518	CTTGTTT_C	INDEL	17606.59	17	19	14
Chr17	10518303	CTTGTTT	C	Chr17_10518	CTTGTTT_C	INDEL	17606.59	17	19	14
Chr17	10518303	CTTGTTT	C	Chr17_10518	CTTGTTT_C	INDEL	17606.59	17	19	14
Chr17	10518311	A	AACGG	Chr17_10518	A_AACGG	INDEL	17696.59	17	19	13
Chr17	10518311	A	AACGG	Chr17_10518	A_AACGG	INDEL	17696.59	17	19	13
Chr17	10518311	A	AACGG	Chr17_10518	A_AACGG	INDEL	17696.59	17	19	13
Chr17	10518319	A	G	Chr17_10518	A_G	SNP	17525.63	17	19	15
Chr17	10518319	A	G	Chr17_10518	A_G	SNP	17525.63	17	19	15
Chr17	10518319	A	G	Chr17_10518	A_G	SNP	17525.63	17	19	15
Chr17	10518320	GAT	G	Chr17_10518	GAT_G	INDEL	18018.59	17	20	16
Chr17	10518320	GAT	G	Chr17_10518	GAT_G	INDEL	18018.59	17	20	16
Chr17	10518320	GAT	G	Chr17_10518	GAT_G	INDEL	18018.59	17	20	16
Chr17	10518323	G	GGTT	Chr17_10518	G_GGTT	INDEL	17514.59	17	18	16
Chr17	10518323	G	GGTT	Chr17_10518	G_GGTT	INDEL	17514.59	17	18	16
Chr17	10518323	G	GGTT	Chr17_10518	G_GGTT	INDEL	17514.59	17	18	16
Chr17	10518325	T	C	Chr17_10518	T_C	SNP	17523.63	17	18	14
Chr17	10518325	T	C	Chr17_10518	T_C	SNP	17523.63	17	18	14
Chr17	10518325	T	C	Chr17_10518	T_C	SNP	17523.63	17	18	14
Chr17	10530076	G	GT	Chr17_10530	G_GT	INDEL	13019.59	25	10	11
Chr17	10530076	G	GT	Chr17_10530	G_GT	INDEL	13019.59	25	10	11
Chr17	10530076	G	GT	Chr17_10530	G_GT	INDEL	13019.59	25	10	11
Chr17	10530076	G	GT	Chr17_10530	G_GT	INDEL	13019.59	25	10	11
Chr17	10530076	G	GT	Chr17_10530	G_GT	INDEL	13019.59	25	10	11
Chr17	10530076	G	GT	Chr17_10530	G_GT	INDEL	13019.59	25	10	11
Chr17	10530076	G	GT	Chr17_10530	G_GT	INDEL	13019.59	25	10	11
Chr17	10530076	G	GT	Chr17_10530	G_GT	INDEL	13019.59	25	10	11
Chr17	10530076	G	GT	Chr17_10530	G_GT	INDEL	13019.59	25	10	11
Chr17	10530076	G	GT	Chr17_10530	G_GT	INDEL	13019.59	25	10	11
Chr17	10530076	G	GT	Chr17_10530	G_GT	INDEL	13019.59	25	10	11
Chr17	10530076	G	GT	Chr17_10530	G_GT	INDEL	13019.59	25	10	11
Chr17	10550190	G	A	Chr17_10550	G_A	SNP	5173.29	0	0	0
Chr17	10845690	C	A	Chr17_10845	C_A	SNP	18486.63	18	16	11
Chr17	10845698	G	T	Chr17_10845	G_T	SNP	19200.63	21	16	11
Chr17	10845701	AC	A	Chr17_10845	AC_A	INDEL	19032.59	21	16	10
Chr17	10845732	T	TG	Chr17_10845	T_TG	INDEL	18526.59	22	13	10
Chr17	10845755	G	T	Chr17_10845	G_T	SNP	17405.63	23	6	12
Chr17	10846763	TG	T	Chr17_10846	TG_T	INDEL	19358.59	24	19	17
Chr17	10846779	C	A	Chr17_10846	C_A	SNP	19308.63	22	18	17
Chr17	10846849	A	T	Chr17_10846	A_T	SNP	15999.63	16	14	12
Chr17	10846853	A	T	Chr17_10846	A_T	SNP	16011.63	17	15	12
Chr17	10846879	C	A	Chr17_10846	C_A	SNP	13629.63	14	14	9
Chr17	10846924	C	T	Chr17_10846	C_T	SNP	13872.63	14	8	8
Chr17	10846968	C	T	Chr17_10846	C_T	SNP	16157.63	15	12	4
Chr17	10846971	TC	T	Chr17_10846	TC_T	INDEL	16212.59	15	12	4
Chr17	10911307	C	CAG	Chr17_10911	C_CAG	INDEL	14585.59	25	18	12
Chr17	10911307	C	CAG	Chr17_10911	C_CAG	INDEL	14585.59	25	18	12
Chr17	10911307	C	CAG	Chr17_10911	C_CAG	INDEL	14585.59	25	18	12
Chr17	10911310	T	A	Chr17_10911	T_A	SNP	14999.63	25	18	12
Chr17	10911310	T	A	Chr17_10911	T_A	SNP	14999.63	25	18	12
Chr17	10911310	T	A	Chr17_10911	T_A	SNP	14999.63	25	18	12
Chr17	10911313	CCCT	C	Chr17_10911	CCCT_C	INDEL	15004.59	24	18	13
Chr17	10911313	CCCT	C	Chr17_10911	CCCT_C	INDEL	15004.59	24	18	13
Chr17	10911313	CCCT	C	Chr17_10911	CCCT_C	INDEL	15004.59	24	18	13
Chr17	10911316	T	TAA	Chr17_10911	T_TAA	INDEL	14959.59	24	18	13
Chr17	10911316	T	TAA	Chr17_10911	T_TAA	INDEL	14959.59	24	18	13
Chr17	10911316	T	TAA	Chr17_10911	T_TAA	INDEL	14959.59	24	18	13
Chr17	10915163	G	A	Chr17_10915	G_A	SNP	16923.63	21	18	11
Chr17	10915163	G	A	Chr17_10915	G_A	SNP	16923.63	21	18	11
Chr17	10915163	G	A	Chr17_10915	G_A	SNP	16923.63	21	18	11
Chr17	10915163	G	A	Chr17_10915	G_A	SNP	16923.63	21	18	11
Chr17	10915168	G	A	Chr17_10915	G_A	SNP	17252.63	21	18	12
Chr17	10915168	G	A	Chr17_10915	G_A	SNP	17252.63	21	18	12
Chr17	10915168	G	A	Chr17_10915	G_A	SNP	17252.63	21	18	12
Chr17	10915168	G	A	Chr17_10915	G_A	SNP	17252.63	21	18	12
Chr17	10915170	A	G	Chr17_10915	A_G	SNP	17132.63	20	18	12
Chr17	10915170	A	G	Chr17_10915	A_G	SNP	17132.63	20	18	12
Chr17	10915170	A	G	Chr17_10915	A_G	SNP	17132.63	20	18	12
Chr17	10915170	A	G	Chr17_10915	A_G	SNP	17132.63	20	18	12
Chr17	11000736	C	T	Chr17_11000	C_T	SNP	20582.63	29	18	17
Chr17	11026812	T	A	Chr17_11026	T_A	SNP	6067.29	0	0	0
Chr17	11026812	T	A	Chr17_11026	T_A	SNP	6067.29	0	0	0
Chr17	11026812	T	A	Chr17_11026	T_A	SNP	6067.29	0	0	0
Chr17	11026812	T	A	Chr17_11026	T_A	SNP	6067.29	0	0	0
Chr17	11026812	T	A	Chr17_11026	T_A	SNP	6067.29	0	0	0
Chr17	11044224	T	G	Chr17_11044	T_G	SNP	22411.63	31	12	14
Chr17	11044224	T	G	Chr17_11044	T_G	SNP	22411.63	31	12	14
Chr17	11044224	T	G	Chr17_11044	T_G	SNP	22411.63	31	12	14
Chr17	11044224	T	G	Chr17_11044	T_G	SNP	22411.63	31	12	14
Chr17	11044224	T	G	Chr17_11044	T_G	SNP	22411.63	31	12	14
Chr17	11044224	T	G	Chr17_11044	T_G	SNP	22411.63	31	12	14
Chr17	11044224	T	G	Chr17_11044	T_G	SNP	22411.63	31	12	14
Chr17	11071805	TTATA	T	Chr17_11071	TTATA_T	INDEL	6802.37	0	0	0
Chr17	11221039	A	AAT	Chr17_11221	A_AAT	INDEL	2037.24	3	0	0
Chr17	11221039	A	AAT	Chr17_11221	A_AAT	INDEL	2037.24	3	0	0
Chr17	11317354	T	TA	Chr17_11317	T_TA	INDEL	16858.59	30	18	15
Chr17	11334577	T	TG	Chr17_11334	T_TG	INDEL	13083.59	19	15	14
Chr17	11334577	T	TG	Chr17_11334	T_TG	INDEL	13083.59	19	15	14
Chr17	11334577	T	TG	Chr17_11334	T_TG	INDEL	13083.59	19	15	14
Chr17	11334577	T	TG	Chr17_11334	T_TG	INDEL	13083.59	19	15	14
Chr17	11334577	T	TG	Chr17_11334	T_TG	INDEL	13083.59	19	15	14
Chr17	11334605	G	T	Chr17_11334	G_T	SNP	10578.63	17	11	11
Chr17	11334605	G	T	Chr17_11334	G_T	SNP	10578.63	17	11	11
Chr17	11334605	G	T	Chr17_11334	G_T	SNP	10578.63	17	11	11
Chr17	11334605	G	T	Chr17_11334	G_T	SNP	10578.63	17	11	11
Chr17	11334605	G	T	Chr17_11334	G_T	SNP	10578.63	17	11	11
Chr17	11334782	C	CA	Chr17_11334	C_CA	INDEL	11922.59	20	9	13
Chr17	11334782	C	CA	Chr17_11334	C_CA	INDEL	11922.59	20	9	13
Chr17	11334782	C	CA	Chr17_11334	C_CA	INDEL	11922.59	20	9	13
Chr17	11334782	C	CA	Chr17_11334	C_CA	INDEL	11922.59	20	9	13
Chr17	11334782	C	CA	Chr17_11334	C_CA	INDEL	11922.59	20	9	13
Chr17	11475638	A	AAT	Chr17_11475	A_AAT	INDEL	5657.24	25	8	9
Chr17	11475638	A	AAT	Chr17_11475	A_AAT	INDEL	5657.24	25	8	9
Chr17	11475638	A	AAT	Chr17_11475	A_AAT	INDEL	5657.24	25	8	9
Chr17	11475638	A	AAT	Chr17_11475	A_AAT	INDEL	5657.24	25	8	9
Chr17	11475638	A	AAT	Chr17_11475	A_AAT	INDEL	5657.24	25	8	9
Chr17	11475638	A	AAT	Chr17_11475	A_AAT	INDEL	5657.24	25	8	9
Chr17	11475638	A	AAT	Chr17_11475	A_AAT	INDEL	5657.24	25	8	9
Chr17	11475638	A	AAT	Chr17_11475	A_AAT	INDEL	5657.24	25	8	9
Chr17	11679193	C	A	Chr17_11679	C_A	SNP	16589.63	24	15	18

Chr17	11832761	G	GT	Chr17_11832G_GT	INDEL	16958.59	31	9	14	30	24	28
Chr17	11854539	C	CT	Chr17_11854C_CT	INDEL	12023.59	14	7	8	14	10	20
Chr17	11854539	C	CT	Chr17_11854C_CT	INDEL	12023.59	14	7	8	14	10	20
Chr17	11854539	C	CT	Chr17_11854C_CT	INDEL	12023.59	14	7	8	14	10	20
Chr17	11854539	C	CT	Chr17_11854C_CT	INDEL	12023.59	14	7	8	14	10	20
Chr17	11854539	C	CT	Chr17_11854C_CT	INDEL	12023.59	14	7	8	14	10	20
Chr17	11854539	C	CT	Chr17_11854C_CT	INDEL	12023.59	14	7	8	14	10	20
Chr17	11854539	C	CT	Chr17_11854C_CT	INDEL	12023.59	14	7	8	14	10	20
Chr17	11854539	C	CT	Chr17_11854C_CT	INDEL	12023.59	14	7	8	14	10	20
Chr17	11854539	C	CT	Chr17_11854C_CT	INDEL	12023.59	14	7	8	14	10	20
Chr17	11854539	C	CT	Chr17_11854C_CT	INDEL	12023.59	14	7	8	14	10	20
Chr17	11854539	C	CT	Chr17_11854C_CT	INDEL	12023.59	14	7	8	14	10	20
Chr17	11854539	C	CT	Chr17_11854C_CT	INDEL	12023.59	14	7	8	14	10	20
Chr17	11854539	C	CT	Chr17_11854C_CT	INDEL	12023.59	14	7	8	14	10	20
Chr17	11854539	C	CT	Chr17_11854C_CT	INDEL	12023.59	14	7	8	14	10	20
Chr17	11854539	C	CT	Chr17_11854C_CT	INDEL	12023.59	14	7	8	14	10	20
Chr17	11854539	C	CT	Chr17_11854C_CT	INDEL	12023.59	14	7	8	14	10	20
Chr17	11854539	C	CT	Chr17_11854C_CT	INDEL	12023.59	14	7	8	14	10	20

[illegible]

Chr17	14434878	GT	G	Chr17_14434GT_G	INDEL	10911.59	17	13
Chr17	14434878	GT	G	Chr17_14434GT_G	INDEL	10911.59	17	13
Chr17	14434880	A	G	Chr17_14434A_G	SNP	10785.63	17	13
Chr17	14434880	A	G	Chr17_14434A_G	SNP	10785.63	17	13
Chr17	14434880	A	G	Chr17_14434A_G	SNP	10785.63	17	13
Chr17	14434880	A	G	Chr17_14434A_G	SNP	10785.63	17	13
Chr17	14434880	A	G	Chr17_14434A_G	SNP	10785.63	17	13
Chr17	14434880	A	G	Chr17_14434A_G	SNP	10785.63	17	13
Chr17	14490392	T	TCA	Chr17_14490T_TCA	INDEL	11646.59	17	8
Chr17	14627379	AATAT	A	Chr17_14627AATAT_A	INDEL	17869.59	22	11
Chr17	14627379	AATAT	A	Chr17_14627AATAT_A	INDEL	17869.59	22	11
Chr17	14688840	C	T	Chr17_14688C_T	SNP	4686.98	0	0
Chr17	14691460	T	TG	Chr17_14691T_TG	INDEL	16346.59	31	15
Chr17	14691495	G	T	Chr17_14691G_T	SNP	18788.63	31	15
Chr17	14691503	G	T	Chr17_14691G_T	SNP	18231.63	30	16
Chr17	14928645	G	GAT	Chr17_14928G_GAT	INDEL	2633.24	1	0
Chr17	14928645	G	GAT	Chr17_14928G_GAT	INDEL	2633.24	1	0
Chr17	14928645	G	GAT	Chr17_14928G_GAT	INDEL	2633.24	1	0
Chr17	14928645	G	GAT	Chr17_14928G_GAT	INDEL	2633.24	1	0
Chr17	14987033	G	C	Chr17_14987G_C	SNP	4434.98	0	0
Chr17	15094522	A	T	Chr17_15094A_T	SNP	19462.63	32	21
Chr17	15094615	A	AT	Chr17_15094A_AT	INDEL	20925.59	36	19
Chr17	15094631	T	TA	Chr17_15094T_TA	INDEL	21639.59	38	20
Chr17	15382391	G	A	Chr17_15382G_A	SNP	3791.98	0	0
Chr17	15450263	G	A	Chr17_15450G_A	SNP	19499.63	36	17
Chr17	15450263	G	A	Chr17_15450G_A	SNP	19499.63	36	17
Chr17	15450263	G	A	Chr17_15450G_A	SNP	19499.63	36	17
Chr17	15468751	C	CTA	Chr17_15468C_CTA	INDEL	2370.24	0	1
Chr17	15525033	C	T	Chr17_15525C_T	SNP	18948.63	26	7
Chr17	15576940	TTA	T	Chr17_15576TTA_T	INDEL	6177.93	14	8
Chr17	15673099	A	AAT	Chr17_15673A_AAT	INDEL	309.37	0	2
Chr17	15673099	A	AAT	Chr17_15673A_AAT	INDEL	309.37	0	2
Chr17	15673099	A	AAT	Chr17_15673A_AAT	INDEL	309.37	0	2
Chr17	15673099	A	AAT	Chr17_15673A_AAT	INDEL	309.37	0	2
Chr17	15694260	GTA	G	Chr17_15694GTA_G	INDEL	8566.59	25	12
Chr17	15694260	GTA	G	Chr17_15694GTA_G	INDEL	8566.59	25	12
Chr17	15694260	GTA	G	Chr17_15694GTA_G	INDEL	8566.59	25	12
Chr17	15694260	GTA	G	Chr17_15694GTA_G	INDEL	8566.59	25	12
Chr17	15806151	G	T	Chr17_15806G_T	SNP	19723.63	37	12
Chr17	15829912	T	C	Chr17_15829T_C	SNP	17207.63	24	20
Chr17	15840068	T	C	Chr17_15840T_C	SNP	6076.29	0	0
Chr17	15844257	C	T	Chr17_15844C_T	SNP	3025.98	0	0
Chr17	15908917	C	G	Chr17_15908C_G	SNP	4791.29	0	0
Chr17	15969836	A	AT	Chr17_15969A_AT	INDEL	14229.59	33	11
Chr18	31779	G	T	Chr18_31779G_T	SNP	19824.63	22	14
Chr18	31781	G	A	Chr18_31781G_A	SNP	19824.63	22	14
Chr18	59362	TA	T	Chr18_59362TA_T	INDEL	18498.59	39	20
Chr18	59362	TA	T	Chr18_59362TA_T	INDEL	18498.59	39	20
Chr18	59362	TA	T	Chr18_59362TA_T	INDEL	18498.59	39	20
Chr18	59362	TA	T	Chr18_59362TA_T	INDEL	18498.59	39	20
Chr18	59362	TA	T	Chr18_59362TA_T	INDEL	18498.59	39	20
Chr18	59643	A	C	Chr18_59643A_C	SNP	17269.63	20	4
Chr18	59643	A	C	Chr18_59643A_C	SNP	17269.63	20	4
Chr18	59643	A	C	Chr18_59643A_C	SNP	17269.63	20	4
Chr18	59643	A	C	Chr18_59643A_C	SNP	17269.63	20	4
Chr18	59643	A	C	Chr18_59643A_C	SNP	17269.63	20	4
Chr18	59645	A	G	Chr18_59645A_G	SNP	17269.63	19	4
Chr18	59645	A	G	Chr18_59645A_G	SNP	17269.63	19	4
Chr18	59645	A	G	Chr18_59645A_G	SNP	17269.63	19	4
Chr18	59645	A	G	Chr18_59645A_G	SNP	17269.63	19	4
Chr18	59645	A	G	Chr18_59645A_G	SNP	17269.63	19	4
Chr18	242731	C	T	Chr18_24273C_T	SNP	10235.63	17	9
Chr18	242734	C	T	Chr18_24273C_T	SNP	10415.63	19	10
Chr18	242738	C	CATA	Chr18_24273C_CATA	INDEL	10811.59	18	10
Chr18	242743	A	AGT	Chr18_24274A_AGT	INDEL	11216.59	20	10
Chr18	242744	AACAAAAC	A	Chr18_24274AACAAAAC_	INDEL	11126.59	21	9
Chr18	242752	C	T	Chr18_24275C_T	SNP	10685.63	21	9
Chr18	295803	C	A	Chr18_29580C_A	SNP	10737.63	16	6
Chr18	643674	A	AAT	Chr18_64367A_AAT	INDEL	1268.35	16	17
Chr18	683667	G	GA	Chr18_68366G_GA	INDEL	17049.59	25	23
Chr18	699415	G	A	Chr18_69941G_A	SNP	19258.63	28	22
Chr18	741823	A	AG	Chr18_74182A_AG	INDEL	19873.59	25	9
Chr18	741824	T	C	Chr18_74182T_C	SNP	19882.63	25	9
Chr18	741878	C	A	Chr18_74187C_A	SNP	17047.63	23	17
Chr18	742035	C	A	Chr18_74203C_A	SNP	17533.63	23	16
Chr18	743831	C	A	Chr18_74383C_A	SNP	17384.63	28	13
Chr18	745647	G	T	Chr18_74564G_T	SNP	18119.63	24	13
Chr18	757717	G	T	Chr18_75771G_T	SNP	13836.63	17	22
Chr18	757749	G	C	Chr18_75774G_C	SNP	14313.63	19	16
Chr18	757759	G	C	Chr18_75775G_C	SNP	14885.63	23	17
Chr18	757822	TG	T	Chr18_75782TG_T	INDEL	13737.59	25	10
Chr18	757869	G	T	Chr18_75786G_T	SNP	10339.63	21	6
Chr18	762845	A	G	Chr18_76284A_G	SNP	13389.63	22	16
Chr18	781779	T	C	Chr18_78177T_C	SNP	30414.63	56	31
Chr18	781846	C	CA	Chr18_78184C_CA	INDEL	22474.59	50	21
Chr18	781851	C	A	Chr18_78185C_A	SNP	22527.63	49	18
Chr18	795676	T	C	Chr18_79567T_C	SNP	25838.63	32	28
Chr18	796411	T	C	Chr18_79641T_C	SNP	21974.63	42	22
Chr18	796426	G	A	Chr18_79642G_A	SNP	22619.63	43	18
Chr18	796444	G	A	Chr18_79644G_A	SNP	21339.63	46	21
Chr18	802788	G	A	Chr18_80278G_A	SNP	12885.63	21	18
Chr18	815447	C	T	Chr18_81544C_T	SNP	12046.63	30	10
Chr18	815603	G	A	Chr18_81560G_A	SNP	18901.63	36	10
Chr18	815637	G	A	Chr18_81563G_A	SNP	21712.63	26	16
Chr18	823390	TC	T	Chr18_82339TC_T	INDEL	16470.59	32	12
Chr18	888282	T	C	Chr18_88828T_C	SNP	17425.63	25	16
Chr18	992934	A	G	Chr18_99293A_G	SNP	11249.63	13	16
Chr18	1035674	A	G	Chr18_10356A_G	SNP	21197.63	36	16
Chr18	1035840	C	A	Chr18_10358C_A	SNP	19174.63	25	15
Chr18	1036207	T	A	Chr18_10362T_A	SNP	13048.63	23	12
Chr18	1088188	T	TTA	Chr18_10881T_TTA	INDEL	5162.24	22	7
Chr18	1088188	T	TTA	Chr18_10881T_TTA	INDEL	5162.24	22	7
Chr18	1088188	T	TTA	Chr18_10881T_TTA	INDEL	5162.24	22	7
Chr18	1088188	T	TTA	Chr18_10881T_TTA	INDEL	5162.24	22	7
Chr18	1088188	T	TTA	Chr18_10881T_TTA	INDEL	5162.24	22	7
Chr18	1196676	A	T	Chr18_11966A_T	SNP	20341.63	30	23
Chr18	1199573	TTA	T	Chr18_11995TTA_T	INDEL	10154.59	25	12
Chr18	1276456	G	T	Chr18_12764G_T	SNP	18068.63	28	10
Chr18	1423340	G	GTA	Chr18_14233G_GTA	INDEL	2444.24	0	0
Chr18	1423340	G	GTA	Chr18_14233G_GTA	INDEL	2444.24	0	0
Chr18	1423340	G	GTA	Chr18_14233G_GTA	INDEL	2444.24	0	0
Chr18	1426591	G	C	Chr18_14265G_C	SNP	14117.63	24	11
Chr18	1426591	G	C	Chr18_14265G_C	SNP	14117.63	24	11
Chr18	1426608	CG	C	Chr18_14266CG_C	INDEL	15861.59	24	11
Chr18	1426608	CG	C	Chr18_14266CG_C	INDEL	15861.59	24	11
Chr18	1426615	G	T	Chr18_14266G_T	SNP	15912.63	21	11
Chr18	1426615	G	T	Chr18_14266G_T	SNP	15912.63	21	11
Chr18	1426639	G	C	Chr18_14266G_C	SNP	15198.63	16	11
Chr18	1426639	G	C	Chr18_14266G_C	SNP	15198.63	16	11
Chr18	1426648	C	A	Chr18_14266C_A	SNP	15596.63	18	11
Chr18	1426648	C	A	Chr18_14266C_A	SNP	15596.63	18	11
Chr18	1429912	G	GTA	Chr18_14299G_GTA	INDEL	1946.93	0	0
Chr18	1429912	G	GTA	Chr18_14299G_GTA	INDEL	1946.93	0	0
Chr18	1429912	G	GTA	Chr18_14299G_GTA	INDEL	1946.93	0	0
Chr18	1434446	C	A	Chr18_14344C_A	SNP	19736.63	28	13
Chr18	1603269	C	A	Chr18_16032C_A	SNP	6055.29	0	0
Chr18	1603269	C	A	Chr18_16032C_A	SNP	6055.29	0	0
Chr18	1603269	C	A	Chr18_16032C_A	SNP	6055.29	0	0
Chr18	1654603	A	AT	Chr18_16546A_AT	INDEL	16665.59	26	14
Chr18	1655208	A	T	Chr18_16552A_T	SNP	11588.63	11	8
Chr18	1655250	A	G	Chr18_16552A_G	SNP	11470.63	10	8
Chr18	1655299	G	T	Chr18_16552G_T	SNP	13688.63	17	7
Chr18	1655316	C	T	Chr18_16553C_T	SNP	14613.63	21	8
Chr18	1655344	A	T	Chr18_16553A_T	SNP	15984.63	24	11
Chr18	2123502	T	G	Chr18_21235T_G	SNP	17137.63	30	13
Chr18	2214308	G	GA	Chr18_22143G_GA	INDEL	14494.59	28	12
Chr18	2214308	G	GA	Chr18_22143G_GA	INDEL	14494.59	28	12
Chr18	2433549	C	A	Chr18_24335C_A	SNP	17004.63	20	16
Chr18	2433549	C	A	Chr18_24335C_A	SNP	17004.63	20	16
Chr18	2433549	C	A	Chr18_24335C_A	SNP	17004.63	20	16
Chr18	2433604	T	A	Chr18_24336T_A	SNP	17032.63	22	14
Chr18	2433604	T	A	Chr18_24336T_A	SNP	17032.63	22	14
Chr18	2433604	T	A	Chr18_24336T_A	SNP	17032.63	22	14
Chr18	2438883	G	A	Chr18_24388G_A	SNP	15169.63	19	11
Chr18	2438883	G	A	Chr18_24388G_A	SNP	15169.63	19	11
Chr18	2438883	G	A	Chr18_24388G_A	SNP	15169.63	19	11
Chr18	2438883	G	A	Chr18_24388G_A	SNP	15169.63	19	11
Chr18	2438883	G	A	Chr18_24388G_A	SNP	15169.63	19	11
Chr18	2438883	G	A	Chr18_24388G_A	SNP	15169.63	19	11
Chr18	2438883	G	A	Chr18_24388G_A	SNP	15169.63	19	11
Chr18	2475955	TC	T	Chr18_24759TC_T	INDEL	18222.59	28	11
Chr18	2475955	TC	T	Chr18_24759TC_T	INDEL	18222.59	28	11
Chr18	2476044	G	GT	Chr18_24760G_GT	INDEL	16646.59	21	16
Chr18	2476044	G	GT	Chr18_24760G_GT	INDEL	16646.59	21	16
Chr18	2759706	T	C	Chr18_27597T_C	SNP	10602.63	6	13
Chr18	2759729	C	T	Chr18_27597C_T	SNP	8877.63	5	10
Chr18	2759752	C	T	Chr18_27597C_T	SNP	6866.63	7	10
Chr18	2883151	G	A	Chr18_28831G_A	SNP	5646.98	0	0
Chr18	2883151	G	A	Chr18_28831G_A	SNP	5646.98	0	0
Chr18	2883151	G	A	Chr18_28831G_A	SNP	5646.98	0	0
Chr18	2929721	A	AAT	Chr18_29297A_AAT	INDEL	1912.93	0	1
Chr18	2929721	A	AAT	Chr18_29297A_AAT	INDEL	1912.93	0	1
Chr18	2929721	A	AAT	Chr18_29297A_AAT	INDEL	1912.93	0	1
Chr18	2929721	A	AAT	Chr18_29297A_AAT	INDEL	1912.93	0	1
Chr18	2929721	A	AAT	Chr18_29297A_AAT	INDEL	1912.93	0	1
Chr18	2929721	A	AAT	Chr18_29297A_AAT	INDEL	1912.93	0	1
Chr18	2929721	A	AAT	Chr18_29297A_AAT	INDEL	1912.93	0	1
Chr18	2929721	A	AAT	Chr18_29297A_AAT	INDEL	1912.93	0	1
Chr18	2961210	T	C	Chr18_29612T_C	SNP	17357.63	34	17
Chr18	2989302	T	TA	Chr18_29893T_TA	INDEL	14938.59	20	12
Chr18	2989302	T	TA	Chr18_29893T_TA	INDEL	14938.59	20	12
Chr18	2989302	T	TA	Chr18_29893T_TA	INDEL	14938.59	20	12
Chr18	2989302	T	TA	Chr18_29893T_TA	INDEL	14938.59	20	12
Chr18	2989302	T	TA	Chr18_29893T_TA	INDEL	14938.59	20	12
Chr18	2989302	T	TA	Chr18_29893T_TA	INDEL	14938.59	20	12
Chr18	2989302	T	TA	Chr18_29893T_TA	INDEL	14938.59	20	12
Chr18	2989302	T	TA	Chr18_29893T_TA	INDEL	14938.59	20	12
Chr18	2989302	T	TA	Chr18_29893T_TA	INDEL	14938.59	20	12
Chr18	2989302	T	TA	Chr18_29893T_TA	INDEL	14938.59	20	12
Chr18	2989302	T	TA	Chr18_29893T_TA	INDEL	14938.59	20	12
Chr18	2989341	T	A	Chr18_29893T_A	SNP	15476.63	19	11
Chr18	2989341	T	A	Chr18_29893T_A	SNP	15476.63	19	11
Chr18	2989341	T	A	Chr18_29893T_A	SNP	15476.63	19	11
Chr18	2989341	T	A	Chr18_29893T_A	SNP	15476.63	19	11
Chr18	2989341	T	A	Chr18_29893T_A	SNP	15476.63	19	11
Chr18	2989341	T	A	Chr18_29893T_A	SNP	15476.63	19	11
Chr18	2989341	T	A	Chr18_29893T_A	SNP	15476.63	19	11
Chr18	2989341	T	A	Chr18_29893T_A	SNP	15476.63	19	11
Chr18	2989341	T	A	Chr18_29893T_A	SNP	15476.63	19	11
Chr18	2989341	T	A	Chr18_29893T_A	SNP	15476.63	19	11
Chr18	2989341	T	A	Chr18_29893T_A	SNP	15476.63	19	11
Chr18	3082176	C	CT	Chr18_30821C_CT	INDEL	13968.59	19	9
Chr18	3115956	CAT	C	Chr18_31159CAT_C	INDEL	9648.59	22	6
Chr18	3115956	CAT	C	Chr18_31159CAT_C	INDEL	9648.59	22	6
Chr18	3115956	CAT	C	Chr18_31159CAT_C	INDEL	9648.59	22	6
Chr18	3150969	T	C	Chr18_31509T_C	SNP	13670.63	17	12
Chr18	3150970	T	G	Chr18_31509T_G	SNP	13940.63	16	12
Chr18	3150972	T	C	Chr18_31509T_C	SNP	14017.63	16	12
Chr18	3150973	T	A	Chr18_31509T_A	SNP	13972.63	17	12
Chr18	3185386	C	G	Chr18_31853C_G	SNP	3717.98	0	0
Chr18	3233840	G	A	Chr18_32338G_A	SNP	23277.63	36	19
Chr18	3233840	G	A	Chr18_32338G_A	SNP	23277.63	36	19

Chr18	3233840	G	A	Chr18_32338G_A	SNP	23277.63	36	19	29	[illegible]
Chr18	3233851	G	T	Chr18_32338G_T	SNP	23653.63	39	17	31	[illegible]
Chr18	3233851	G	T	Chr18_32338G_T	SNP	23653.63	39	17	31	[illegible]
Chr18	3233851	G	T	Chr18_32338G_T	SNP	23653.63	39	17	31	[illegible]
Chr18	3273532	G	A	Chr18_32735G_A	SNP	2685.39	38	18	8	[illegible]
Chr18	3538283	C	CAT	Chr18_35382C_CAT	INDEL	180.37	1	0	9	[illegible]
Chr18	3652389	C	CAT	Chr18_36523C_CAT	INDEL	1321.35	19	17	7	[illegible]
Chr18	3652389	C	CAT	Chr18_36523C_CAT	INDEL	1321.35	19	17	7	[illegible]
Chr18	3652389	C	CAT	Chr18_36523C_CAT	INDEL	1321.35	19	17	7	[illegible]
Chr18	3716689	CTA	C	Chr18_37166CTA_C	INDEL	11800.59	16	13	13	[illegible]
Chr18	3716689	CTA	C	Chr18_37166CTA_C	INDEL	11800.59	16	13	13	[illegible]
Chr18	3716689	CTA	C	Chr18_37166CTA_C	INDEL	11800.59	16	13	13	[illegible]
Chr18	3716689	CTA	C	Chr18_37166CTA_C	INDEL	11800.59	16	13	13	[illegible]
Chr18	3716689	CTA	C	Chr18_37166CTA_C	INDEL	11800.59	16	13	13	[illegible]
Chr18	3717074	A	AT	Chr18_37170A_AT	INDEL	10591.59	23	7	10	[illegible]
Chr18	3717074	A	AT	Chr18_37170A_AT	INDEL	10591.59	23	7	10	[illegible]
Chr18	3717074	A	AT	Chr18_37170A_AT	INDEL	10591.59	23	7	10	[illegible]
Chr18	3717074	A	AT	Chr18_37170A_AT	INDEL	10591.59	23	7	10	[illegible]
Chr18	3717074	A	AT	Chr18_37170A_AT	INDEL	10591.59	23	7	10	[illegible]
Chr18	3737484	G	T	Chr18_37374G_T	SNP	22473.63	36	15	15	[illegible]
Chr18	3737484	G	T	Chr18_37374G_T	SNP	22473.63	36	15	15	[illegible]
Chr18	3737484	G	T	Chr18_37374G_T	SNP	22473.63	36	15	15	[illegible]
Chr18	3737484	G	T	Chr18_37374G_T	SNP	22473.63	36	15	15	[illegible]
Chr18	3737484	G	T	Chr18_37374G_T	SNP	22473.63	36	15	15	[illegible]
Chr18	3737484	G	T	Chr18_37374G_T	SNP	22473.63	36	15	15	[illegible]
Chr18	3737484	G	T	Chr18_37374G_T	SNP	22473.63	36	15	15	[illegible]
Chr18	3737484	G	T	Chr18_37374G_T	SNP	22473.63	36	15	15	[illegible]
Chr18	3737484	G	T	Chr18_37374G_T	SNP	22473.63	36	15	15	[illegible]
Chr18	3737484	G	T	Chr18_37374G_T	SNP	22473.63	36	15	15	[illegible]
Chr18	3737484	G	T	Chr18_37374G_T	SNP	22473.63	36	15	15	[illegible]
Chr18	3737484	G	T	Chr18_37374G_T	SNP	22473.63	36	15	15	[illegible]
Chr18	3737484	G	T	Chr18_37374G_T	SNP	22473.63	36	15	15	[illegible]
Chr18	3737484	G	T	Chr18_37374G_T	SNP	22473.63	36	15	15	[illegible]
Chr18	3737484	G	T	Chr18_37374G_T	SNP	22473.63	36	15	15	[illegible]
Chr18	3737484	G	T	Chr18_37374G_T	SNP	22473.63	36	15	15	[illegible]
Chr18	3737486	G	GA	Chr18_37374G_GA	INDEL	23230.59	38	14	14	[illegible]
Chr18	3737486	G	GA	Chr18_37374G_GA	INDEL	23230.59	38	14	14	[illegible]
Chr18	3737486	G	GA	Chr18_37374G_GA	INDEL	23230.59	38	14	14	[illegible]
Chr18	3737486	G	GA	Chr18_37374G_GA	INDEL	23230.59	38	14	14	[illegible]
Chr18	3737486	G	GA	Chr18_37374G_GA	INDEL	23230.59	38	14	14	[illegible]
Chr18	3737486	G	GA	Chr18_37374G_GA	INDEL	23230.59	38	14	14	[illegible]
Chr18	3737486	G	GA	Chr18_37374G_GA	INDEL	23230.59	38	14	14	[illegible]
Chr18	3737486	G	GA	Chr18_37374G_GA	INDEL	23230.59	38	14	14	[illegible]
Chr18	3737486	G	GA	Chr18_37374G_GA	INDEL	23230.59	38	14	14	[illegible]
Chr18	3737486	G	GA	Chr18_37374G_GA	INDEL	23230.59	38	14	14	[illegible]
Chr18	3737486	G	GA	Chr18_37374G_GA	INDEL	23230.59	38	14	14	[illegible]
Chr18	3737486	G	GA	Chr18_37374G_GA	INDEL	23230.59	38	14	14	[illegible]
Chr18	3737486	G	GA	Chr18_37374G_GA	INDEL	23230.59	38	14	14	[illegible]
Chr18	3737486	G	GA	Chr18_37374G_GA	INDEL	23230.59	38	14	14	[illegible]
Chr18	3737486	G	GA	Chr18_37374G_GA	INDEL	23230.59	38	14	14	[illegible]
Chr18	3737486	G	GA	Chr18_37374G_GA	INDEL	23230.59	38	14	14	[illegible]
Chr18	3737487	C	CT	Chr18_37374C_CT	INDEL	23249.59	38	14	14	[illegible]
Chr18	3737487	C	CT	Chr18_37374C_CT	INDEL	23249.59	38	14	14	[illegible]
Chr18	3737487	C	CT	Chr18_37374C_CT	INDEL	23249.59	38	14	14	[illegible]
Chr18	3737487	C	CT	Chr18_37374C_CT	INDEL	23249.59	38	14	14	[illegible]
Chr18	3737487	C	CT	Chr18_37374C_CT	INDEL	23249.59	38	14	14	[illegible]
Chr18	3737487	C	CT	Chr18_37374C_CT	INDEL	23249.59	38	14	14	[illegible]
Chr18	3737487	C	CT	Chr18_37374C_CT	INDEL	23249.59	38	14	14	[illegible]
Chr18	3737487	C	CT	Chr18_37374C_CT	INDEL	23249.59	38	14	14	[illegible]
Chr18	3737487	C	CT	Chr18_37374C_CT	INDEL	23249.59	38	14	14	[illegible]
Chr18	3737487	C	CT	Chr18_37374C_CT	INDEL	23249.59	38	14	14	[illegible]
Chr18	3737487	C	CT	Chr18_37374C_CT	INDEL	23249.59	38	14	14	[illegible]
Chr18	3737487	C	CT	Chr18_37374C_CT	INDEL	23249.59	38	14	14	[illegible]
Chr18	3737487	C	CT	Chr18_37374C_CT	INDEL	23249.59	38	14	14	[illegible]
Chr18	3737487	C	CT	Chr18_37374C_CT	INDEL	23249.59	38	14	14	[illegible]
Chr18	3737487	C	CT	Chr18_37374C_CT	INDEL	23249.59	38	14	14	[illegible]
Chr18	3737487	C	CT	Chr18_37374C_CT	INDEL	23249.59	38	14	14	[illegible]
Chr18	3737490	G	GCC	Chr18_37374G_GCC	INDEL	23494.59	35	13	14	[illegible]
Chr18	3737490	G	GCC	Chr18_37374G_GCC	INDEL	23494.59	35	13	14	[illegible]
Chr18	3737490	G	GCC	Chr18_37374G_GCC	INDEL	23494.59	35	13	14	[illegible]
Chr18	3737490	G	GCC	Chr18_37374G_GCC	INDEL	23494.59	35	13	14	[illegible]
Chr18	3737490	G	GCC	Chr18_37374G_GCC	INDEL	23494.59	35	13	14	[illegible]
Chr18	3737490	G	GCC	Chr18_37374G_GCC	INDEL	23494.59	35	13	14	[illegible]
Chr18	3737490	G	GCC	Chr18_37374G_GCC	INDEL	23494.59	35	13	14	[illegible]
Chr18	3737490	G	GCC	Chr18_37374G_GCC	INDEL	23494.59	35	13	14	[illegible]
Chr18	3737490	G	GCC	Chr18_37374G_GCC	INDEL	23494.59	35	13	14	[illegible]
Chr18	3737490	G	GCC	Chr18_37374G_GCC	INDEL	23494.59	35	13	14	[illegible]
Chr18	3737490	G	GCC	Chr18_37374G_GCC	INDEL	23494.59	35	13	14	[illegible]
Chr18	3737490	G	GCC	Chr18_37374G_GCC	INDEL	23494.59	35	13	14	[illegible]
Chr18	3737490	G	GCC	Chr18_37374G_GCC	INDEL	23494.59	35	13	14	[illegible]
Chr18	3737490	G	GCC	Chr18_37374G_GCC	INDEL	23494.59	35	13	14	[illegible]
Chr18	3737490	G	GCC	Chr18_37374G_GCC	INDEL	23494.59	35	13	14	[illegible]
Chr18	3737490	G	GCC	Chr18_37374G_GCC	INDEL	23494.59	35	13	14	[illegible]
Chr18	3737493	A	T	Chr18_37374A_T	SNP	23047.63	36	13	14	[illegible]
Chr18	3737493	A	T	Chr18_37374A_T	SNP	23047.63	36	13	14	[illegible]
Chr18	3737493	A	T	Chr18_37374A_T	SNP	23047.63	36	13	14	[illegible]
Chr18	3737493	A	T	Chr18_37374A_T	SNP	23047.63	36	13	14	[illegible]
Chr18	3737493	A	T	Chr18_37374A_T	SNP	23047.63	36	13	14	[illegible]
Chr18	3737493	A	T	Chr18_37374A_T	SNP	23047.63	36	13	14	[illegible]
Chr18	3737493	A	T	Chr18_37374A_T	SNP	23047.63	36	13	14	[illegible]
Chr18	3737493	A	T	Chr18_37374A_T	SNP	23047.63	36	13	14	[illegible]
Chr18	3737493	A	T	Chr18_37374A_T	SNP	23047.63	36	13	14	[illegible]
Chr18	3737493	A	T	Chr18_37374A_T	SNP	23047.63	36	13	14	[illegible]
Chr18	3737493	A	T	Chr18_37374A_T	SNP	23047.63	36	13	14	[illegible]
Chr18	3737493	A	T	Chr18_37374A_T	SNP	23047.63	36	13	14	[illegible]
Chr18	3737493	A	T	Chr18_37374A_T	SNP	23047.63	36	13	14	[illegible]
Chr18	3737493	A	T	Chr18_37374A_T	SNP	23047.63	36	13	14	[illegible]
Chr18	3737493	A	T	Chr18_37374A_T	SNP	23047.63	36	13	14	[illegible]
Chr18	3737493	A	T	Chr18_37374A_T	SNP	23047.63	36	13	14	[illegible]
Chr18	3737494	G	T	Chr18_37374G_T	SNP	22867.63	36	13	14	[illegible]
Chr18	3737494	G	T	Chr18_37374G_T	SNP	22867.63	36	13	14	[illegible]
Chr18	3737494	G	T	Chr18_37374G_T	SNP	22867.63	36	13	14	[illegible]
Chr18	3737494	G	T	Chr18_37374G_T	SNP	22867.63	36	13	14	[illegible]
Chr18	3737494	G	T	Chr18_37374G_T	SNP	22867.63	36	13	14	[illegible]
Chr18	3737494	G	T	Chr18_37374G_T	SNP	22867.63	36	13	14	[illegible]
Chr18	3737494	G	T	Chr18_37374G_T	SNP	22867.63	36	13	14	[illegible]
Chr18	3737494	G	T	Chr18_37374G_T	SNP	22867.63	36	13	14	[illegible]
Chr18	3737494	G	T	Chr18_37374G_T	SNP	22867.63	36	13	14	[illegible]
Chr18	3737494	G	T	Chr18_37374G_T	SNP	22867.63	36	13	14	[illegible]
Chr18	3737494	G	T	Chr18_37374G_T	SNP	22867.63	36	13	14	[illegible]
Chr18	3737494	G	T	Chr18_37374G_T	SNP	22867.63	36	13	14	[illegible]
Chr18	3737494	G	T	Chr18_37374G_T	SNP	22867.63	36	13	14	[illegible]
Chr18	3737494	G	T	Chr18_37374G_T	SNP	22867.63	36	13	14	[illegible]
Chr18	3737494	G	T	Chr18_37374G_T	SNP	22867.63	36	13	14	[illegible]
Chr18	3737494	G	T	Chr18_37374G_T	SNP	22867.63	36	13	14	[illegible]
Chr18	3997801	A	G	Chr18_39978A_G	SNP	20912.63	24	16	23	[illegible]
Chr18	3997802	G	T	Chr18_39978G_T	SNP	20912.63	24	16	23	[illegible]
Chr18	4180165	A	T	Chr18_41801A_T	SNP	17973.63	29	20	9	[illegible]
Chr18	4222874	A	AT	Chr18_42228A_AT	INDEL	15366.59	30	12	22	[illegible]
Chr18	4236118	C	A	Chr18_42361C_A	SNP	13915.63	22	9	8	[illegible]
Chr18	4236123	C	A	Chr18_42361C_A	SNP	13867.63	22	8	8	[illegible]
Chr18	4265044	A	G	Chr18_42650A_G	SNP	15937.63	25	8	16	[illegible]
Chr18	4272739	C	G	Chr18_42727C_G	SNP	5418.29	0	0	0	[illegible]
Chr18	4362022	C	T	Chr18_43620C_T	SNP	7195.29	0	0	0	[illegible]
Chr18	4362022	C	T	Chr18_43620C_T	SNP	7195.29	0	0	0	[illegible]
Chr18	4362022	C	T	Chr18_43620C_T	SNP	7195.29	0	0	0	[illegible]
Chr18	4362022	C	T	Chr18_43620C_T	SNP	7195.29	0	0	0	[illegible]
Chr18	4368045	CAT	C	Chr18_43680CAT_C	INDEL	7664.59	16	10	9	[illegible]
Chr18	4368045	CAT	C	Chr18_43680CAT_C	INDEL	7664.59	16	10	9	[illegible]
Chr18	4368045	CAT	C	Chr18_43680CAT_C	INDEL	7664.59	16	10	9	[illegible]
Chr18	4368045	CAT	C	Chr18_43680CAT_C	INDEL	7664.59	16	10	9	[illegible]
Chr18	4368045	CAT	C	Chr18_43680CAT_C	INDEL	7664.59	16	10	9	[illegible]
Chr18	4380270	T	TA	Chr18_43802T_TA	INDEL	15768.59	18	13	19	[illegible]
Chr18	4380381	A	AT	Chr18_43803A_AT	INDEL	21021.59	26	17	14	[illegible]
Chr18	4380385	C	T	Chr18_43803C_T	SNP	21159.63	27	17	14	[illegible]
Chr18	4420641	C	CTTCT	Chr18_44206C_CTTCT	INDEL	10747.59	14	6	9	[illegible]
Chr18	4420647	A	AAATTATTTT	Chr18_44206A_AAATTATT	INDEL	9371.59	12	4	9	[illegible]
Chr18	4420648	C	CTTTTAGGT	Chr18_44206C_CTTTTAGG	INDEL	9281.59	11	4	8	[illegible]
Chr18	4438247	AAT	A	Chr18_44382AAT_A	INDEL	3898.93	0	1	0	[illegible]
Chr18	4438247	AAT	A	Chr18_44382AAT_A	INDEL	3898.93	0	1	0	[illegible]
Chr18	4438247	AAT	A	Chr18_44382AAT_A	INDEL	3898.93	0	1	0	[illegible]
Chr18	4438247	AAT	A	Chr18_44382AAT_A	INDEL	3898.93	0	1	0	[illegible]
Chr18	4438247	AAT	A	Chr18_44382AAT_A	INDEL	3898.93	0	1	0	[illegible]
Chr18	4438247	AAT	A	Chr18_44382AAT_A	INDEL	3898.93	0	1	0	[illegible]
Chr18	4438247	AAT	A	Chr18_44382AAT_A	INDEL	3898.93	0	1	0	[illegible]
Chr18	4438247	AAT	A	Chr18_44382AAT_A	INDEL	3898.93	0	1	0	[illegible]
Chr18	4438247	AAT	A	Chr18_44382AAT_A	INDEL	3898.93	0	1	0	[illegible]
Chr18	4438247	AAT	A	Chr18_44382AAT_A	INDEL	3898.93	0	1	0	[illegible]
Chr18	4438247	AAT	A	Chr18_44382AAT_A	INDEL	3898.93	0	1	0	[illegible]
Chr18	4438247	AAT	A	Chr18_44382AAT_A	INDEL	3898.93	0	1	0	[illegible]
Chr18	4438247	AAT	A	Chr18_44382AAT_A	INDEL	3898.93	0	1	0	[illegible]
Chr18	4438247	AAT	A	Chr18_44382AAT_A	INDEL	3898.93	0	1	0	[illegible]
Chr18	4438247	AAT	A	Chr18_44382AAT_A	INDEL	3898.93	0	1	0	[illegible]
Chr18	4438247	AAT	A	Chr18_44382AAT_A	INDEL	3898.93	0	1	0	[illegible]
Chr18	4510979	C	CTA	Chr18_45109C_CTA	INDEL	11142.59	45	11	11	[illegible]
Chr18	4510979	C	CTA	Chr18_45109C_CTA	INDEL	11142.59	45	11	11	[illegible]
Chr18	4510979	C	CTA	Chr18_45109C_CTA	INDEL	11142.59	45	11	11	[illegible]
Chr18	4510979	C	CTA	Chr18_45109C_CTA	INDEL	11142.59	45	11	11	[illegible]
Chr18	4510979	C	CTA	Chr18_45109C_CTA	INDEL	11142.59	45	11	11	[illegible]
Chr18	4510979	C	CTA	Chr18_45109C_CTA	INDEL	11142.59	45	11	11	[illegible]
Chr18	4510979	C	CTA	Chr18_45109C_CTA	INDEL	11142.59	45	11	11	[illegible]
Chr18	4510979	C	CTA	Chr18_45109C_CTA	INDEL	11142.59	45	11	11	[illegible]
Chr18	4765170	G	GT	Chr18_47651G_GT	INDEL	15766.59	31	15	17	[illegible]
Chr18	4805311	A	AT	Chr18_48053A_AT	INDEL	15666.59	32	12	11	[illegible]
Chr18	4805311	A	AT	Chr18_48053A_AT	INDEL	15666.59	32	12	11	[illegible]
Chr18	4805311	A	AT	Chr18_48053A_AT	INDEL	15666.59	32	12	11	[illegible]
Chr18	4812799	TA	T	Chr18_48127TA_T	INDEL	15361.59	12	11	15	[illegible]
Chr18	4812819	TA	T	Chr18_48128TA_T	INDEL	16239.59	11	11	15	[illegible]
Chr18	4812825	T	C	Chr18_48128T_C	SNP	16339.63	12	10	16	[illegible]
Chr18	4812831	GA	G	Chr18_48128GA_G	INDEL	16913.59	15	10	17	[illegible]
Chr18	4812843	A	AC	Chr18_48128A_AC	INDEL	16929.59	18	11	18	[illegible]
Chr18	4822380	C	T	Chr18_48223C_T	SNP	22009.63	37	18	17	[illegible]
Chr18	4822419	C	A	Chr18_48224C_A	SNP	22404.63	32	22	15	[illegible]
Chr18	4822420	TC	T	Chr18_48224TC_T	INDEL	22394.59	32	22	15	[illegible]
Chr18	4822466	C	T	Chr18_48224C_T	SNP	21044.63	33	19	15	[illegible]
Chr18	4822513	G	T	Chr18_48225G_T	SNP	23368.63	34	18	16	[illegible]
Chr18	4822532	G	T	Chr18_48225G_T	SNP	23125.63	35	20	15	[illegible]
Chr18	4822600	AG	A	Chr18_48226AG_A	INDEL	20706.59	34	25	16	[illegible]
Chr18	4893443	T	TA	Chr18_48934T_TA	INDEL	14782.59	29	11	11	[illegible]
Chr18	5002913	T	TTA	Chr18_50029T_TTA	INDEL	2367.82	1	0	0	[illegible]
Chr18	5002913	T	TTA	Chr18_50029T_TTA	INDEL	2367.82	1	0	0	[illegible]
Chr18	5002913	T	TTA	Chr18_50029T_TTA	INDEL	2367.82	1	0	0	[illegible]
Chr18	5002913	T	TTA	Chr18_50029T_TTA	INDEL	2367.82	1	0	0	[illegible]
Chr18	5187050	C	A	Chr18_51870C_A	SNP	22537.63	39	13	12	[illegible]
Chr18	5187050	C	A	Chr18_51870C_A	SNP	22537.63	39	13	12	[illegible]
Chr18	5187050	C	A	Chr18_51870C_A	SNP	22537.63	39	13	12	[illegible]
Chr18	5187050	C	A	Chr18_51870C_A	SNP	22537.63	39	13	12	[illegible]
Chr18	5187050	C	A	Chr18_51870C_A	SNP	22537.63	39	13	12	[illegible]
Chr18	5187050	C	A	Chr18_51870C_A	SNP	22537.63	39	13	12	[illegible]
Chr18	5187050	C	A	Chr18_51870C_A	SNP	22537.63	39	13	12	[illegible]
Chr18	5187050	C	A	Chr18_51870C_A	SNP	22537.63	39	13	12	[illegible]
Chr18	5187050	C	A	Chr18_51870C_A	SNP	22537.63	39	13	12	[illegible]
Chr18	5210935	T	TA	Chr18_52109T_TA	INDEL	17268.59	38	20	17	[illegible]
Chr18	5234048	G	T	Chr18_52340G_T	SNP	7817.59	17	8	7	[illegible]
Chr18	5234048	G	T	Chr18_52340G_T	SNP	7817.59	17	8	7	[illegible]
Chr18	5234048	G	T	Chr18_52340G_T	SNP	7817.59	17	8	7	[illegible]
Chr18	5367216	G	A	Chr18_53672G_A	SNP	5343.29	0	0	0	[illegible]
Chr18	5367216	G	A	Chr18_53672G_A	SNP	5343.29	0	0	0	[illegible]
Chr18	5367216	G	A	Chr18_53672G_A	SNP	5343.29	0	0	0	[illegible]
Chr18	5367216	G	A	Chr18_53672G_A	SNP	5343.29	0	0	0	[illegible]
Chr18	5399904	T	TTA	Chr18_53999T_TTA	INDEL	171.37	0	0	0	[illegible]
Chr18	5445113	T	C	Chr18_54451T_C	SNP	5566.29	0	0	0	[illegible]

Chr18	5484213	C	A	Chr18_54842C_A	SNP	18908.63	20	22	10	25	18	32	24	31	23	20	20	12	10	13	20	22	10	25	18	32	24	31	23	20	20	12	10	13		1	1	1	[illegible]
Chr18	5518610	CT	C	Chr18_55186CT_C	INDEL	9196.59	12	8	9	23	10	29	14	15	18	14	9	5	7	6	12	8	9	23	10	29	14	15	18	14	9	5	7	6		1	1	1	[illegible]
Chr18	5664306	G	A	Chr18_56643G_A	SNP	6365.29	0	0	0	26	28	19	32	25	0	0	0	0	0	0	20	20	10	26	28	19	32	25	25	37	18	16	16	9		0	0	0	[illegible]
Chr18	5786984	C	CAT	Chr18_57869C_CAT	INDEL	4446.24	1	0	0	20	17	27	31	21	0	0	1	0	0	0	18	11	13	20	18	27	31	21	17	20	13	19	13	10		0	0	0	[illegible]
Chr18	5786984	C	CAT	Chr18_57869C_CAT	INDEL	4446.24	1	0	0	20	17	27	31	21	0	0	1	0	0	0	18	11	13	20	18	27	31	21	17	20	13	19	13	10		0	0	0	[illegible]
Chr18	5786984	C	CAT	Chr18_57869C_CAT	INDEL	4446.24	1	0	0	20	17	27	31	21	0	0	1	0	0	0	18	11	13	20	18	27	31	21	17	20	13	19	13	10		0	0	0	[illegible]
Chr18	5786984	C	CAT	Chr18_57869C_CAT	INDEL	4446.24	1	0	0	20	17	27	31	21	0	0	1	0	0	0	18	11	13	20	18	27	31	21	17	20	13	19	13	10		0	0	0	[illegible]
Chr18	5786984	C	CAT	Chr18_57869C_CAT	INDEL	4446.24	1	0	0	20	17	27	31	21	0	0	1	0	0	0	18	11	13	20	18	27	31	21	17	20	13	19	13	10		0	0	0	[illegible]
Chr18	5858655	G	T	Chr18_58586G_T	SNP	17549.63	26	20	12	18	25	23	19	19	18	28	12	20	11	14	27	20	12	18	25	23	19	19	18	28	12	20	11	14	0.963		1	1	[illegible]
Chr18	5859693	C	A	Chr18_58596C_A	SNP	15742.63	32	6	7	27	14	25	19	25	20	21	11	18	4	13	32	6	7	27	14	25	19	25	20	21	11	18	4	13		1	1	1	[illegible]
Chr18	5909027	A	AAT	Chr18_59090A_AAT	INDEL	1467.35	23	8	16	0	0	0	0	0	1	1	1	0	0	0	24	8	16	20	14	15	21	14	13	21	12	11	12	8	0.958		1	1	[illegible]
Chr18	5909027	A	AAT	Chr18_59090A_AAT	INDEL	1467.35	23	8	16	0	0	0	0	0	1	1	1	0	0	0	24	8	16	20	14	15	21	14	13	21	12	11	12	8	0.958		1	1	[illegible]
Chr18	5909412	C	T	Chr18_59094C_T	SNP	19830.63	38	16	15	24	13	31	35	23	26	22	28	21	13	10	39	16	15	24	13	31	35	23	26	22	28	21	13	10	0.974		1	1	[illegible]
Chr18	5909412	C	T	Chr18_59094C_T	SNP	19830.63	38	16	15	24	13	31	35	23	26	22	28	21	13	10	39	16	15	24	13	31	35	23	26	22	28	21	13	10	0.974		1	1	[illegible]
Chr18	5970395	A	AAT	Chr18_59703A_AAT	INDEL	1041.35	24	10	8	0	0	0	1	0	0	1	0	0	2	0	25	10	8	12	8	15	15	8	8	12	6	8	7	6	0.96		1	1	[illegible]
Chr18	6014679	A	AT	Chr18_60146A_AT	INDEL	18485.59	31	5	15	32	22	29	32	21	28	26	15	21	12	15	31	5	15	32	22	29	32	21	28	26	15	21	12	15		1	1	1	[illegible]
Chr18	6014679	A	AT	Chr18_60146A_AT	INDEL	18485.59	31	5	15	32	22	29	32	21	28	26	15	21	12	15	31	5	15	32	22	29	32	21	28	26	15	21	12	15		1	1	1	[illegible]
Chr18	6014679	A	AT	Chr18_60146A_AT	INDEL	18485.59	31	5	15	32	22	29	32	21	28	26	15	21	12	15	31	5	15	32	22	29	32	21	28	26	15	21	12	15		1	1	1	[illegible]
Chr18	6014679	A	AT	Chr18_60146A_AT	INDEL	18485.59	31	5	15	32	22	29	32	21	28	26	15	21	12	15	31	5	15	32	22	29	32	21	28	26	15	21	12	15		1	1	1	[illegible]
Chr18	6014679	A	AT	Chr18_60146A_AT	INDEL	18485.59	31	5	15	32	22	29	32	21	28	26	15	21	12	15	31	5	15	32	22	29	32	21	28	26	15	21	12	15		1	1	1	[illegible]
Chr18	6014695	A	AT	Chr18_60146A_AT	INDEL	18320.59	33	6	14	28	18	28	36	19	25	23	17	21	12	18	33	6	14	28	18	28	36	19	25	23	17	21	12	18		1	1	1	[illegible]
Chr18	6014695	A	AT	Chr18_60146A_AT	INDEL	18320.59	33	6	14	28	18	28	36	19	25	23	17	21	12	18	33	6	14	28	18	28	36	19	25	23	17	21	12	18		1	1	1	[illegible]
Chr18	6014695	A	AT	Chr18_60146A_AT	INDEL	18320.59	33	6	14	28	18	28	36	19	25	23	17	21	12	18	33	6	14	28	18	28	36	19	25	23	17	21	12	18		1	1	1	[illegible]
Chr18	6014695	A	AT	Chr18_60146A_AT	INDEL	18320.59	33	6	14	28	18	28	36	19	25	23	17	21	12	18	33	6	14	28	18	28	36	19	25	23	17	21	12	18		1	1	1	[illegible]
Chr18	6014695	A	AT	Chr18_60146A_AT	INDEL	18320.59	33	6	14	28	18	28	36	19	25	23	17	21	12	18	33	6	14	28	18	28	36	19	25	23	17	21	12	18		1	1	1	[illegible]
Chr18	6015577	C	A	Chr18_60155C_A	SNP	21812.63	25	15	21	39	17	29	32	19	25	25	15	11	20	17	25	15	21	39	17	29	32	19	25	25	15	11	20	17		1	1	1	[illegible]
Chr18	6015577	C	A	Chr18_60155C_A	SNP	21812.63	25	15	21	39	17	29	32	19	25	25	15	11	20	17	25	15	21	39	17	29	32	19	25	25	15	11	20	17		1	1	1	[illegible]
Chr18	6015577	C	A	Chr18_60155C_A	SNP	21812.63	25	15	21	39	17	29	32	19	25	25	15	11	20	17	25	15	21	39	17	29	32	19	25	25	15	11	20	17		1	1	1	[illegible]
Chr18	6015577	C	A	Chr18_60155C_A	SNP	21812.63	25	15	21	39	17	29	32	19	25	25	15	11	20	17	25	15	21	39	17	29	32	19	25	25	15	11	20	17		1	1	1	[illegible]
Chr18	6015577	C	A	Chr18_60155C_A	SNP	21812.63	25	15	21	39	17	29	32	19	25	25	15	11	20	17	25	15	21	39	17	29	32	19	25	25	15	11	20	17		1	1	1	[illegible]
Chr18	6015581	C	A	Chr18_60155C_A	SNP	22238.63	25	15	21	40	17	29	33	19	25	23	15	11	20	17	25	15	21	40	17	29	33	19	25	23	15	11	20	17		1	1	1	[illegible]
Chr18	6015581	C	A	Chr18_60155C_A	SNP	22238.63	25	15	21	40	17	29	33	19	25	23	15	11	20	17	25	15	21	40	17	29	33	19	25	23	15	11	20	17		1	1	1	[illegible]
Chr18	6015581	C	A	Chr18_60155C_A	SNP	22238.63	25	15	21	40	17	29	33	19	25	23	15	11	20	17	25	15	21	40	17	29	33	19	25	23	15	11	20	17		1	1	1	[illegible]
Chr18	6015581	C	A	Chr18_60155C_A	SNP	22238.63	25	15	21	40	17	29	33	19	25	23	15	11	20	17	25	15	21	40	17	29	33	19	25	23	15	11	20	17		1	1	1	[illegible]
Chr18	6015581	C	A	Chr18_60155C_A	SNP	22238.63	25	15	21	40	17	29	33	19	25	23	15	11	20	17	25	15	21	40	17	29	33	19	25	23	15	11	20	17		1	1	1	[illegible]
Chr18	6015583	C	A	Chr18_60155C_A	SNP	22006.63	25	15	22	40	17	28	33	19	25	23	15	11	20	19	25	15	22	40	17	28	33	19	25	23	15	11	20	19		1	1	1	[illegible]
Chr18	6015583	C	A	Chr18_60155C_A	SNP	22006.63	25	15	22	40	17	28	33	19	25	23	15	11	20	19	25	15	22	40	17	28	33	19	25	23	15	11	20	19		1	1	1	[illegible]
Chr18	6015583	C	A	Chr18_60155C_A	SNP	22006.63	25	15	22	40	17	28	33	19	25	23	15	11	20	19	25	15	22	40	17	28	33	19	25	23	15	11	20	19		1	1	1	[illegible]
Chr18	6015583	C	A	Chr18_60155C_A	SNP	22006.63	25	15	22	40	17	28	33	19	25	23	15	11	20	19	25	15	22	40	17	28	33	19	25	23	15	11	20	19		1	1	1	[illegible]
Chr18	6015583	C	A	Chr18_60155C_A	SNP	22006.63	25	15	22	40	17	28	33	19	25	23	15	11	20	19	25	15	22	40	17	28	33	19	25	23	15	11	20	19		1	1	1	[illegible]
Chr18	6015620	C	A	Chr18_60156C_A	SNP	19547.63	23	14	22	34	20	26	30	16	24	20	12	11	17	20	23	14	22	34	20	26	30	16	24	20	12	11	17	20		1	1	1	[illegible]
Chr18	6015620	C	A	Chr18_60156C_A	SNP	19547.63	23	14	22	34	20	26	30	16	24	20	12	11	17	20	23	14	22	34	20	26	30	16	24	20	12	11	17	20		1	1	1	[illegible]
Chr18	6015620	C	A	Chr18_60156C_A	SNP	19547.63	23	14	22	34	20	26	30	16	24	20	12	11	17	20	23	14	22	34	20	26	30	16	24	20	12	11	17	20		1	1	1	[illegible]
Chr18	6015620	C	A	Chr18_60156C_A	SNP	19547.63	23	14	22	34	20	26	30	16	24	20	12	11	17	20	23	14	22	34	20	26	30	16	24	20	12	11	17	20		1	1	1	[illegible]
Chr18	6015620	C	A	Chr18_60156C_A	SNP	19547.63	23	14	22	34	20	26	30	16	24	20	12	11	17	20	23	14	22	34	20	26	30	16	24	20	12	11	17	20		1	1	1	[illegible]
Chr18	6328962	G	T	Chr18_63289G_T	SNP	19661.63	40	16	18	30	18	19	29	36	14	26	15	19	15	8	40	16	18	30	18	19	29	36	14	26	15	19	15	8		1	1	1	[illegible]
Chr18	6328992	T	G	Chr18_63289T_G	SNP	19847.63	31	17	18	31	22	21	32	35	15	22	14	15	14	5	31	17	18	31	22	21	32	35	15	22	14	15	14	5		1	1	1	[illegible]
Chr18	6329079	G	T	Chr18_63290G_T	SNP	18617.63	23	16	14	28	25	23	31	31	15	28	13	15	11	10	23	16	14	28	25	23	31	31	15	28	13	15	11	10		1	1	1	[illegible]
Chr18	6330302	G	C	Chr18_63303G_C	SNP	17572.63	17	14	12	23	24	23	36	30	18	18	19	18	8	11	17	14	12	23	24	23	36	30	18	18	19	18	8	11		1	1	1	[illegible]
Chr18	6635459	C	A	Chr18_66354C_A	SNP	11546.63	11	11	5	10	15	14	12	15	15	16	9	8	9	8	11	11	5	10	15	14	12	15	15	16	9	8	9	8		1	1	1	[illegible]
Chr18	6637773	C	A	Chr18_66377C_A	SNP	18111.63	24	12	13	17	19	20	23	16	17	17	4	17	10	11	24	12	13	17	19	20	23	16	17	17	4	17	10	11		1	1	1	[illegible]
Chr18	6637775	G	A	Chr18_66377G_A	SNP	18111.63	24	12	14	18	19	21	23	17	17	17	5	19	10	11	24	12	14	18	19	21	23	17	17	17	5	19	10	11		1	1	1	[illegible]
Chr18	6640353	AT	A	Chr18_66403AT_A	INDEL	21281.59	20	17	17	27	15	27	33	30	22	29	22	17	6	9	20	17	17	27	15	27	33	30	22	29	22	17	6	9		1	1	1	[illegible]
Chr18	6640365	AG	A	Chr18_66403AG_A	INDEL	22554.59	21	17	18	27	18	27	34	28	26	31	22	15	8	10	21	17	18	27	18	27	34	28	26	31	22	15	8	10		1	1	1	[illegible]
Chr18	6640368	G	GA	Chr18_66403G_GA	INDEL	22431.59	21	17	18	27	18	28	34	27	25	31	25	15	7	10	21	17	18	27	18	28	34	27	25	31	25	15	7	10		1	1	1	[illegible]
Chr18	6662659	G	A	Chr18_66626G_A	SNP	5736.29	0	0	0	27	18	24	29	13	0	0	0	0	0	0	25	14	15	27	18	24	29	13	17	19	16	18	7	10		0	0	0	[illegible]
Chr18	6701616	C	A	Chr18_67016C_A	SNP	11811.63	12	7	6	15	10	18	20	8	16	21	13	14	11	9	12	7	6	15	10	18	20	8	16	21	13	14	11	9		1	1	1	[illegible]
Chr18	6701716	T	A	Chr18_67017T_A	SNP	9844.63	7	9	4	14	7	19	20	10	12	12	5	7	5	8	7	9	4	14	7	19	20	10	12	12	5	7	5	8		1	1	1	[illegible]
Chr18	6701718	T	C	Chr18_67017T_C	SNP	9844.63	7	9	4	14	7	18	19	10	12	12	5	7	5	8	7	9	4	14	7	18	19	10	12	12	5	7	5	8		1	1	1	[illegible]
Chr18	6977464	A	AT	Chr18_69774A_AT	INDEL	20001.59	29	20	13	25	14	26	27	24	20	20	11	23	11	16	29	20	13	25	14	26	27	24	20	20	11	23	11	16		1	1	1	[illegible]
Chr18	6977464	A	AT	Chr18_69774A_AT	INDEL	20001.59	29	20	13	25	14	26	27	24	20	20	11	23	11	16	29	20	13	25	14	26	27	24	20	20	11	23	11	16		1	1	1	[illegible]
Chr18	6977464	A	AT	Chr18_69774A_AT	INDEL	20001.59	29	20	13	25	14	26	27	24	20	20	11	23	11	16	29	20	13	25	14	26	27	24	20	20	11	23	11	16		1	1	1	[illegible]
Chr18	6977464	A	AT	Chr18_69774A_AT	INDEL	20001.59	29	20	13	25	14	26	27	24	20	20	11	23	11	16	29	20	13	25	14	26	27	24	20	20	11	23	11	16		1	1	1	[illegible]
Chr18	6977464	A	AT	Chr18_69774A_AT	INDEL	20001.59	29	20	13	25	14	26	27	24	20	20	11	23	11	16	29	20	13	25	14	26	27	24	20	20	11	23	11	16		1	1	1	[illegible]
Chr18	6977472	A	AT	Chr18_69774A_AT	INDEL	20303.59	28	22	14	25	15	28	27	23	21	19	12	22	12	17	28	22	14	25	15	28	27	23	21	19	12	22	12	17		1	1	1	[illegible]
Chr18	6977472	A	AT	Chr18_69774A_AT	INDEL	20303.59	28	22	14	25	15	28	27	23	21	19	12	22	12	17	28	22	14	25	15	28	27	23	21	19	12	22	12	17		1	1	1	[illegible]
Chr18	6977472	A	AT	Chr18_69774A_AT	INDEL	20303.59	28	22	14	25	15	28	27	23	21	19	12	22	12	17	28	22	14	25	15	28	27	23	21	19	12	22	12	17		1	1	1	[illegible]
Chr18	6977472	A	AT	Chr18_69774A_AT	INDEL	20303.59	28	22	14	25	15	28	27	23	21	19	12	22	12	17	28	22	14	25	15	28	27	23	21	19	12	22	12	17		1	1	1	[illegible]
Chr18	6977472	A	AT	Chr18_69774A_AT	INDEL	20303.59	28	22	14	25	15	28	27	23	21	19	12	22	12	17	28	22	14	25	15	28	27	23	21	19	12	22	12	17		1	1	1	[illegible]
Chr18	7054646	G	GT	Chr18_70546G_GT	INDEL	14113.59	19	11	12	27	11	16	21	19	23	14	16	13	9	12	19	11	12	27	11	16	21	19	23	14	16	13	9	12		1	1	1	[illegible]
Chr18	7054646	G	GT	Chr18_70546G_GT	INDEL	14113.59	19	11	12	27	11	16	21	19	23	14	16	13	9	12	19	11	12	27	11	16	21	19	23	14	16	13	9	12		1	1	1	[illegible]
Chr18	7286187	A	T	Chr18_72861A_T	SNP	13029.63	11	11	11	16	16	17	13	17	18	26	12	14	8	12	11	11	11	16	16	17	13	17	18	26	12	14	8	12		1	1	1	[illegible]
Chr18	7286187	A	T	Chr18_72861A_T	SNP	13029.63	11	11	11	16	16	17	13	17	18	26	12	14	8	12	11	11	11	16	16	17	13	17	18	26	12	14	8	12		1	1	1	[illegible]
Chr18	7286187	A	T	Chr18_72861A_T	SNP	13029.63	11	11	11	16	16	17	13	17	18	26	12	14	8	12	11	11	11	16	16	17	13	17	18	26	12	14	8	12		1	1	1	[illegible]
Chr18	7286187	A	T	Chr18_72861A_T	SNP	13029.63	11	11	11	16	16	17	13	17	18	26	12	14	8	12	11	11	11	16	16	17	13	17	18	26	12	14	8	12		1	1	1	[illegible]
Chr18	7286187	A	T	Chr18_72861A_T	SNP	13029.63	11	11	11	16	16	17	13	17	18	26	12	14	8	12	11	11	11	16	16	17	13	17	18	26	12	14	8	12		1	1	1	[illegible]
Chr18	7286200	A	C	Chr18_72862A_C	SNP	11941.63	11	10	10	14	15	13	14	15	17	24	9	13	7	11	11	10	10	14	15	13	14	15	17	24	9	13	7	11		1	1	1	[illegible]
Chr18	7286200	A	C	Chr18_72862A_C	SNP	11941.63	11	10	10	14	15	13	14	15	17	24	9	13	7	11	11	10	10	14	15	13	14	15	17	24	9	13	7	11		1	1	1	[illegible]
Chr18	7286200	A	C	Chr18_72862A_C	SNP	11941.63	11	10	10	14	15	13	14	15	17	24	9	13	7	11	11	10	10	14	15	13	14	15	17	24	9	13	7	11		1	1	1	[illegible]
Chr18	7286200	A	C	Chr18_72862A_C	SNP	11941.63	11	10	10	14	15	13	14	15	17	24	9	13	7	11	11	10	10	14	15	13	14	15	17	24	9	13	7	11		1	1	1	[illegible]
Chr18	7286200	A	C	Chr18_72862A_C	SNP	11941.63	11	10	10	14	15	13	14	15	17	24	9	13	7	11	11	10	10	14	15	13	14	15	17	24	9	13	7	11		1	1	1	[illegible]
Chr18	7286213	A	G	Chr18_72862A_G	SNP	10477.63	12	9	10	14	13	11	12	12	16	17	8	13	8	7	12	9	10	14	13	11	12	12	16	17	8	13	8	7		1	1	1	[illegible]
Chr18	7286213	A	G	Chr18_72862A_G	SNP	10477.63	12	9	10	14	13	11	12	12	16	17	8	13	8	7	12	9	10	14	13	11	12	12	16	17	8	13	8	7		1	1	1	[illegible]
Chr18	7286213	A	G	Chr18_72862A_G	SNP	10477.63	12	9	10	14	13	11	12	12	16	17	8	13	8	7	12	9	10	14	13	11	12	12	16	17	8	13	8	7		1	1	1	[illegible]
Chr18	7286213	A	G	Chr18_72862A_G	SNP	10477.63	12	9	10	14	13	11	12	12	16	17	8	13	8	7	12	9	10	14	13	11	12	12	16	17	8	13	8	7		1	1	1	[illegible]
Chr18	7286213	A	G	Chr18_72862A_G	SNP	10477.63	12	9	10	14	13	11	12	12	16	17	8	13	8	7	12	9	10	14	13	11	12	12	16	17	8	13	8	7		1	1	1	[illegible]
Chr18	7286342	T	A	Chr18_72863T_A	SNP	9993.63	14	9	7	15	15	13	15	12	8	15	5	4	6	5	15	9	7	15	15	13	15	12	8	15	5	4	6	5	0.933		1	1	[illegible]
Chr18	7286342	T	A	Chr18_72863T_A	SNP	9993.63	14	9	7	15	15	13	15	12	8	15	5	4	6	5	15	9	7	15	15	13	15	12	8	15	5	4	6	5	0.933		1	1	[illegible]
Chr18	7286342	T	A	Chr18_72863T_A	SNP	9993.63	14	9	7	15	15	13	15	12	8	15	5	4	6	5	15	9	7	15	15	13	15	12	8	15	5	4	6	5	0.933		1	1	[illegible]
Chr18	7286342	T	A	Chr18_72863T_A	SNP	9993.63	14	9	7	15	15	13	15	12	8	15	5	4	6	5	15	9	7	15	15	13	15	12	8	15	5	4	6	5	0.933		1	1	[illegible]
Chr18	7286342	T	A	Chr18_72863T_A	SNP	9993.63	14	9	7	15	15	13	15	12	8	15	5	4	6	5	15	9	7	15	15	13	15	12	8	15	5	4	6	5	0.933		1	1	[illegible]
Chr18	7286411	T	C	Chr18_72864T_C	SNP	13345.63	19	10	10	19	19	24	25	12	9	19	7	4	9	6	19	10	10	19	19	24	25	12	9	19	7	4	9	6		1	1	1	[illegible]
Chr18	7286411	T	C	Chr18_72864T_C	SNP	13345.63	19	10	10	19	19	24	25	12	9	19	7	4	9	6	19	10	10	19	19	24	25	12	9	19	7	4	9	6		1	1	1	[illegible]
Chr18	7286411	T	C	Chr18_72864T_C	SNP	13345.63	19	10	10	19	19	24	25	12	9	19	7	4	9	6	19	10	10	19	19	24	25	12	9	19	7	4	9	6		1	1	1	[illegible]
Chr18	7286411	T	C	Chr18_72864T_C	SNP	13345.63	19	10	10	19	19	24	25	12	9	19	7	4	9	6	19	10	10	19	19	24	25	12	9	19	7	4	9	6		1	1	1	[illegible]
Chr18	7286411	T	C	Chr18_72864T_C	SNP	13345.63	19	10	10	19	19	24	25	12	9	19	7	4	9	6	19	10	10	19	19	24	25	12	9	19	7	4	9	6		1	1	1	[illegible]

[illegible]

Chr18	9082495	G	C	Chr18_90824G_C	SNP	17808.63	23	16	9	18	16	15	30	14	24	18	8	19	11	12	23	16	9	18	16	15	30	14	24	18	8	19	11	12	1	1	1	1	1	1	1	1	1	1	1	1	1	1	1	1	1	0	15	15	0	7	5	3	intron_variar	MODIFIER	Pp3c18_1234	Pp3c18_1234	transcript	PAC:3298316	protein_codi	juin-17	c.908+29C>G	NA	NA	NA	NA	NA	
Chr18	9210319	G	T	Chr18_92103G_T	SNP	15532.63	24	9	12	27	21	15	29	16	18	13	14	5	13	11	24	9	12	27	21	15	29	16	18	13	14	5	13	11	1	1	1	1	1	1	1	1	1	1	1	1	1	1	1	1	1	0	15	15	0	7	5	3	downstream_	MODIFIER	Pp3c18_125	Pp3c18_125	transcript	PAC:3298234	protein_codi	NA	c.*4622G>T	NA	NA	NA	NA		4084
Chr18	9210319	G	T	Chr18_92103G_T	SNP	15532.63	24	9	12	27	21	15	29	16	18	13	14	5	13	11	24	9	12	27	21	15	29	16	18	13	14	5	13	11	1	1	1	1	1	1	1	1	1	1	1	1	1	1	1	1	1	0	15	15	0	7	5	3	downstream_	MODIFIER	Pp3c18_125	Pp3c18_125	transcript	PAC:3298234	protein_codi	NA	c.*4622G>T	NA	NA	NA	NA		4084
Chr18	9210319	G	T	Chr18_92103G_T	SNP	15532.63	24	9	12	27	21	15	29	16	18	13	14	5	13	11	24	9	12	27	21	15	29	16	18	13	14	5	13	11	1	1	1	1	1	1	1	1	1	1	1	1	1	1	1	1	1	0	15	15	0	7	5	3	downstream_	MODIFIER	Pp3c18_125	Pp3c18_125	transcript	PAC:3298234	protein_codi	NA	c.*4622G>T	NA	NA	NA	NA		4309
Chr18	9210319	G	T	Chr18_92103G_T	SNP	15532.63	24	9	12	27	21	15	29	16	18	13	14	5	13	11	24	9	12	27	21	15	29	16	18	13	14	5	13	11	1	1	1	1	1	1	1	1	1	1	1	1	1	1	1	1	1	0	15	15	0	7	5	3	intergenic_re	MODIFIER	Pp3c18_125	Pp3c18_125	intergenic_re	Pp3c18_125	NA	NA	n.9210319G>	NA	NA	NA	NA	NA	
Chr18	9210329	C	CT	Chr18_92103C_CT	INDEL	12645.59	24	8	13	24	19	15	28	14	17	15	12	5	11	10	24	8	13	24	19	15	28	14	17	15	12	5	11	10	1	1	1	1	1	1	1	1	1	1	1	1	1	1	1	1	1	0	15	15	0	7	5	3	downstream_	MODIFIER	Pp3c18_125	Pp3c18_125	transcript	PAC:3298234	protein_codi	NA	c.*4632_*46	NA	NA	NA	NA		4095
Chr18	9210329	C	CT	Chr18_92103C_CT	INDEL	12645.59	24	8	13	24	19	15	28	14	17	15	12	5	11	10	24	8	13	24	19	15	28	14	17	15	12	5	11	10	1	1	1	1	1	1	1	1	1	1	1	1	1	1	1	1	1	0	15	15	0	7	5	3	downstream_	MODIFIER	Pp3c18_125	Pp3c18_125	transcript	PAC:3298234	protein_codi	NA	c.*4632_*46	NA	NA	NA	NA		4095
Chr18	9210329	C	CT	Chr18_92103C_CT	INDEL	12645.59	24	8	13	24	19	15	28	14	17	15	12	5	11	10	24	8	13	24	19	15	28	14	17	15	12	5	11	10	1	1	1	1	1	1	1	1	1	1	1	1	1	1	1	1	1	0	15	15	0	7	5	3	downstream_	MODIFIER	Pp3c18_125	Pp3c18_125	transcript	PAC:3298234	protein_codi	NA	c.*4632_*46	NA	NA	NA	NA		4320
Chr18	9210329	C	CT	Chr18_92103C_CT	INDEL	12645.59	24	8	13	24	19	15	28	14	17	15	12	5	11	10	24	8	13	24	19	15	28	14	17	15	12	5	11	10	1	1	1	1	1	1	1	1	1	1	1	1	1	1	1	1	1	0	15	15	0	7	5	3	intergenic_re	MODIFIER	Pp3c18_125	Pp3c18_125	intergenic_re	Pp3c18_125	NA	NA	n.9210329_9	NA	NA	NA	NA	NA	
Chr18	9210341	C	CG	Chr18_92103C_CG	INDEL	15716.59	18	9	13	20	18	15	30	14	16	12	12	5	10	10	18	9	13	20	18	15	30	14	16	12	12	5	10	10	1	1	1	1	1	1	1	1	1	1	1	1	1	1	1	1	1	0	15	15	0	7	5	3	downstream_	MODIFIER	Pp3c18_125	Pp3c18_125	transcript	PAC:3298234	protein_codi	NA	c.*4644_*46	NA	NA	NA	NA		4107
Chr18	9210341	C	CG	Chr18_92103C_CG	INDEL	15716.59	18	9	13	20	18	15	30	14	16	12	12	5	10	10	18	9	13	20	18	15	30	14	16	12	12	5	10	10	1	1	1	1	1	1	1	1	1	1	1	1	1	1	1	1	1	0	15	15	0	7	5	3	downstream_	MODIFIER	Pp3c18_125	Pp3c18_125	transcript	PAC:3298234	protein_codi	NA	c.*4644_*46	NA	NA	NA	NA		4107
Chr18	9210341	C	CG	Chr18_92103C_CG	INDEL	15716.59	18	9	13	20	18	15	30	14	16	12	12	5	10	10	18	9	13	20	18	15	30	14	16	12	12	5	10	10	1	1	1	1	1	1	1	1	1	1	1	1	1	1	1	1	1	0	15	15	0	7	5	3	downstream_	MODIFIER	Pp3c18_125	Pp3c18_125	transcript	PAC:3298234	protein_codi	NA	c.*4644_*46	NA	NA	NA	NA		4332
Chr18	9210341	C	CG	Chr18_92103C_CG	INDEL	15716.59	18	9	13	20	18	15	30	14	16	12	12	5	10	10	18	9	13	20	18	15	30	14	16	12	12	5	10	10	1	1	1	1	1	1	1	1	1	1	1	1	1	1	1	1	1	0	15	15	0	7	5	3	intergenic_re	MODIFIER	Pp3c18_125	Pp3c18_125	intergenic_re	Pp3c18_125	NA	NA	n.9210341_9	NA	NA	NA	NA	NA	
Chr18	9210342	A	AG	Chr18_92103A_AG	INDEL	15851.59	17	9	13	20	17	15	31	14	17	12	12	5	10	9	17	9	13	20	17	15	31	14	17	12	12	5	10	9	1	1	1	1	1	1	1	1	1	1	1	1	1	1	1	1	1	0	15	15	0	7	5	3	downstream_	MODIFIER	Pp3c18_125	Pp3c18_125	transcript	PAC:3298234	protein_codi	NA	c.*4645_*46	NA	NA	NA	NA		4108
Chr18	9210342	A	AG	Chr18_92103A_AG	INDEL	15851.59	17	9	13	20	17	15	31	14	17	12	12	5	10	9	17	9	13	20	17	15	31	14	17	12	12	5	10	9	1	1	1	1	1	1	1	1	1	1	1	1	1	1	1	1	1	0	15	15	0	7	5	3	downstream_	MODIFIER	Pp3c18_125	Pp3c18_125	transcript	PAC:3298234	protein_codi	NA	c.*4645_*46	NA	NA	NA	NA		4108
Chr18	9210342	A	AG	Chr18_92103A_AG	INDEL	15851.59	17	9	13	20	17	15	31	14	17	12	12	5	10	9	17	9	13	20	17	15	31	14	17	12	12	5	10	9	1	1	1	1	1	1	1	1	1	1	1	1	1	1	1	1	1	0	15	15	0	7	5	3	downstream_	MODIFIER	Pp3c18_125	Pp3c18_125	transcript	PAC:3298234	protein_codi	NA	c.*4645_*46	NA	NA	NA	NA		4333
Chr18	9210342	A	AG	Chr18_92103A_AG	INDEL	15851.59	17	9	13	20	17	15	31	14	17	12	12	5	10	9	17	9	13	20	17	15	31	14	17	12	12	5	10	9	1	1	1	1	1	1	1	1	1	1	1	1	1	1	1	1	1	0	15	15	0	7	5	3	intergenic_re	MODIFIER	Pp3c18_125	Pp3c18_125	intergenic_re	Pp3c18_125	NA	NA	n.9210342_9	NA	NA	NA	NA	NA	
Chr18	9210349	G	A	Chr18_92103G_A	SNP	15850.63	18	11	11	20	19	12	26	15	18	15	13	6	11	9	18	11	11	20	19	12	26	15	18	15	13	6	11	9	1	1	1	1	1	1	1	1	1	1	1	1	1	1	1	1	1	0	15	15	0	7	5	3	downstream_	MODIFIER	Pp3c18_125	Pp3c18_125	transcript	PAC:3298234	protein_codi	NA	c.*4652G>A	NA	NA	NA	NA		4114
Chr18	9210349	G	A	Chr18_92103G_A	SNP	15850.63	18	11	11	20	19	12	26	15	18	15	13	6	11	9	18	11	11	20	19	12	26	15	18	15	13	6	11	9	1	1	1	1	1	1	1	1	1	1	1	1	1	1	1	1	1	0	15	15	0	7	5	3	downstream_	MODIFIER	Pp3c18_125	Pp3c18_125	transcript	PAC:3298234	protein_codi	NA	c.*4652G>A	NA	NA	NA	NA		4114
Chr18	9210349	G	A	Chr18_92103G_A	SNP	15850.63	18	11	11	20	19	12	26	15	18	15	13	6	11	9	18	11	11	20	19	12	26	15	18	15	13	6	11	9	1	1	1	1	1	1	1	1	1	1	1	1	1	1	1	1	1	0	15	15	0	7	5	3	downstream_	MODIFIER	Pp3c18_125	Pp3c18_125	transcript	PAC:3298234	protein_codi	NA	c.*4652G>A	NA	NA	NA	NA		4339
Chr18	9210349	G	A	Chr18_92103G_A	SNP	15850.63	18	11	11	20	19	12	26	15	18	15	13	6	11	9	18	11	11	20	19	12	26	15	18	15	13	6	11	9	1	1	1	1	1	1	1	1	1	1	1	1	1	1	1	1	1	0	15	15	0	7	5	3	intergenic_re	MODIFIER	Pp3c18_125	Pp3c18_125	intergenic_re	Pp3c18_125	NA	NA	n.9210349G>	NA	NA	NA	NA	NA	
Chr18	9210350	T	A	Chr18_92103T_A	SNP	15625.63	18	11	11	21	19	12	26	15	18	15	13	6	11	9	18	11	11	21	19	12	26	15	18	15	13	6	11	9	1	1	1	1	1	1	1	1	1	1	1	1	1	1	1	1	1	0	15	15	0	7	5	3	downstream_	MODIFIER	Pp3c18_125	Pp3c18_125	transcript	PAC:3298234	protein_codi	NA	c.*4653T>A	NA	NA	NA	NA		4115
Chr18	9210350	T	A	Chr18_92103T_A	SNP	15625.63	18	11	11	21	19	12	26	15	18	15	13	6	11	9	18	11	11	21	19	12	26	15	18	15	13	6	11	9	1	1	1	1	1	1	1	1	1	1	1	1	1	1	1	1	1	0	15	15	0	7	5	3	downstream_	MODIFIER	Pp3c18_125	Pp3c18_125	transcript	PAC:3298234	protein_codi	NA	c.*4653T>A	NA	NA	NA	NA		4115
Chr18	9210350	T	A	Chr18_92103T_A	SNP	15625.63	18	11	11	21	19	12	26	15	18	15	13	6	11	9	18	11	11	21	19	12	26	15	18	15	13	6	11	9	1	1	1	1	1	1	1	1	1	1	1	1	1	1	1	1	1	0	15	15	0	7	5	3	downstream_	MODIFIER	Pp3c18_125	Pp3c18_125	transcript	PAC:3298234	protein_codi	NA	c.*4653T>A	NA	NA	NA	NA		4340
[illegible]																																																																									

Chr18	10782916	A	AAT	Chr18_10782A_AAT	INDEL	1516.93	0	0	0	0	0	1	0	0	5	8	8	12	9	6
Chr18	10792489	G	T	Chr18_10792G_T	SNP	14765.63	23	17	13	14	18	30	23	20	14	17	12	10	5	6
Chr18	10929072	A	AT	Chr18_10929A_AT	INDEL	18292.59	35	18	19	38	20	25	29	23	29	23	11	17	13	15
Chr18	10931437	G	T	Chr18_10931G_T	SNP	16148.63	21	11	10	26	18	21	22	31	18	17	12	16	8	7
Chr18	10936816	T	A	Chr18_10936T_A	SNP	18975.63	23	13	25	24	16	36	29	24	28	17	13	12	12	4
Chr18	10936823	A	AT	Chr18_10936A_AT	INDEL	19601.59	22	13	26	26	18	38	30	26	30	19	13	13	12	5
Chr18	10968407	AC	A	Chr18_10968AC_A	INDEL	15700.59	32	21	15	28	17	24	26	21	30	20	13	19	10	11
Chr18	10968407	AC	A	Chr18_10968AC_A	INDEL	15700.59	32	21	15	28	17	24	26	21	30	20	13	19	10	11
Chr18	10968407	AC	A	Chr18_10968AC_A	INDEL	15700.59	32	21	15	28	17	24	26	21	30	20	13	19	10	11
Chr18	10968407	AC	A	Chr18_10968AC_A	INDEL	15700.59	32	21	15	28	17	24	26	21	30	20	13	19	10	11
Chr18	10989229	AAT	A	Chr18_10989AAT_A	INDEL	6862.59	15	4	9	22	6	20	20	9	7	9	16	15	5	4
Chr18	10997195	G	T	Chr18_10997G_T	SNP	20405.63	30	13	15	31	19	25	29	23	31	28	13	23	10	16
Chr18	10997196	A	G	Chr18_10997A_G	SNP	20585.63	30	13	15	30	19	25	28	23	31	29	14	23	9	16
Chr18	10997214	G	T	Chr18_10997G_T	SNP	20521.63	29	13	15	30	20	28	29	25	32	27	15	24	10	21
Chr18	11003796	A	AAT	Chr18_11003A_AAT	INDEL	2791.24	0	0	0	17	14	17	19	16	0	0	1	0	0	0
Chr18	11029128	G	A	Chr18_11029G_A	SNP	3070.98	0	0	0	0	0	0	0	0	15	16	7	23	6	6
Chr18	11036915	T	C	Chr18_11036T_C	SNP	19150.63	22	11	9	27	17	25	26	28	28	25	23	17	13	7
Chr18	11036915	T	C	Chr18_11036T_C	SNP	19150.63	22	11	9	27	17	25	26	28	28	25	23	17	13	7
Chr18	11036915	T	C	Chr18_11036T_C	SNP	19150.63	22	11	9	27	17	25	26	28	28	25	23	17	13	7
Chr18	11103183	TG	T	Chr18_11103TG_T	INDEL	18583.59	20	13	10	25	23	19	29	22	25	31	18	10	11	11
Chr18	11103187	A	ATCC	Chr18_11103A_ATCC	INDEL	19183.59	20	13	10	25	23	20	29	22	24	31	18	10	12	12
Chr18	11103189	G	A	Chr18_11103G_A	SNP	19057.63	21	13	9	24	22	19	30	22	23	30	17	11	12	12
Chr18	11170223	A	G	Chr18_11170A_G	SNP	18993.63	37	11	19	25	17	26	37	24	17	24	14	14	12	6
Chr18	11264051	G	GT	Chr18_11264G_GT	INDEL	16584.59	21	18	15	30	17	25	23	23	23	25	17	17	7	20
Chr18	11482331	AAT	A	Chr18_11482AAT_A	INDEL	11233.59	17	7	16	14	23	22	31	22	16	12	12	7	6	9
Chr18	11482331	AAT	A	Chr18_11482AAT_A	INDEL	11233.59	17	7	16	14	23	22	31	22	16	12	12	7	6	9
Chr18	11482331	AAT	A	Chr18_11482AAT_A	INDEL	11233.59	17	7	16	14	23	22	31	22	16	12	12	7	6	9
Chr18	11482331	AAT	A	Chr18_11482AAT_A	INDEL	11233.59	17	7	16	14	23	22	31	22	16	12	12	7	6	9
Chr18	11482331	AAT	A	Chr18_11482AAT_A	INDEL	11233.59	17	7	16	14	23	22	31	22	16	12	12	7	6	9
Chr18	11482331	AAT	A	Chr18_11482AAT_A	INDEL	11233.59	17	7	16	14	23	22	31	22	16	12	12	7	6	9
Chr18	11482331	AAT	A	Chr18_11482AAT_A	INDEL	11233.59	17	7	16	14	23	22	31	22	16	12	12	7	6	9
Chr18	11482331	AAT	A	Chr18_11482AAT_A	INDEL	11233.59	17	7	16	14	23	22	31	22	16	12	12	7	6	9
Chr18	11482331	AAT	A	Chr18_11482AAT_A	INDEL	11233.59	17	7	16	14	23	22	31	22	16	12	12	7	6	9
Chr18	11520725	A	AAT	Chr18_11520A_AAT	INDEL	2133.24	0	0	0	18	13	19	13	10	2	3	1	1	0	0
Chr18	11635283	T	TTA	Chr18_11635T_TTA	INDEL	2339.24	1	0	1	11	6	15	21	13	0	0	0	0	0	0
Chr18	11642262	AC	A	Chr18_11642AC_A	INDEL	8053.59	15	10	7	9	5	6	19	10	7	6	6	8	4	8
Chr18	11645528	C	CAT	Chr18_11645C_CAT	INDEL	7961.24	30	10	14	0	0	0	0	0	16	18	11	8	8	4
Chr18	11648305	T	TTA	Chr18_11648T_TTA	INDEL	851.35	14	7	6	0	0	0	1	0	0	0	0	0	0	0
Chr18	11797548	A	AAT	Chr18_11797A_AAT	INDEL	1763.93	0	0	0	0	3	0	2	0	18	17	5	6	5	6
Chr18	11849694	T	A	Chr18_11849T_A	SNP	19565.63	24	11	17	23	20	27	22	23	30	25	5	21	8	5
Chr18	11849706	T	A	Chr18_11849T_A	SNP	20349.63	22	12	18	22	20	29	19	24	33	23	6	21	7	7
Chr18	11849714	T	TA	Chr18_11849T_TA	INDEL	20891.59	23	12	18	23	21	29	21	26	34	22	6	20	6	7
Chr18	11849738	C	T	Chr18_11849C_T	SNP	21742.63	23	11	15	25	22	29	23	29	35	25	6	20	9	8
Chr18	11849741	G	GA	Chr18_11849G_GA	INDEL	21573.59	23	12	15	25	23	30	25	29	35	24	7	21	9	8
Chr18	11931238	G	T	Chr18_11931G_T	SNP	12022.63	17	7	9	17	13	13	12	12	16	14	8	8	5	4
Chr18	11931368	A	G	Chr18_11931A_G	SNP	17590.63	34	14	7	23	19	33	22	20	25	12	20	11	10	12
Chr18	11935647	A	AAT	Chr18_11935A_AAT	INDEL	3951.24	20	9	10	0	2	0	0	2	7	5	4	8	5	5
Chr18	12047750	G	A	Chr18_12047G_A	SNP	5362.98	0	0	0	0	0	0	0	0	30	26	7	33	12	13
Chr18	12066978	C	CA	Chr18_12066C_CA	INDEL	13488.59	25	10	13	28	15	19	33	26	23	20	19	11	4	13
Chr18	12066978	C	CA	Chr18_12066C_CA	INDEL	13488.59	25	10	13	28	15	19	33	26	23	20	19	11	4	13
Chr18	12098375	C	A	Chr18_12098C_A	SNP	11743.63	11	11	4	15	10	22	18	21	14	16	10	12	4	4
Chr18	12620189	A	AT	Chr18_12620A_AT	INDEL	16950.59	24	12	16	22	24	33	31	22	24	26	13	22	16	7
Chr18	12698974	G	GT	Chr18_12698G_GT	INDEL	13291.59	21	13	6	26	19	29	32	20	26	21	5	16	7	10
Chr18	12803649	C	CA	Chr18_12803C_CA	INDEL	15863.59	20	13	12	29	20	28	32	19	26	21	12	18	13	14
Chr18	12803649	C	CA	Chr18_12803C_CA	INDEL	15863.59	20	13	12	29	20	28	32	19	26	21	12	18	13	14
Chr18	12803649	C	CA	Chr18_12803C_CA	INDEL	15863.59	20	13	12	29	20	28	32	19	26	21	12	18	13	14
Chr18	12964277	G	GA	Chr18_12964G_GA	INDEL	16025.59	29	17	18	22	17	21	43	18	13	28	11	21	14	11
Chr18	12964277	G	GA	Chr18_12964G_GA	INDEL	16025.59	29	17	18	22	17	21	43	18	13	28	11	21	14	11
Chr18	12964277	G	GA	Chr18_12964G_GA	INDEL	16025.59	29	17	18	22	17	21	43	18	13	28	11	21	14	11
Chr18	12964277	G	GA	Chr18_12964G_GA	INDEL	16025.59	29	17	18	22	17	21	43	18	13	28	11	21	14	11
Chr18	12964277	G	GA	Chr18_12964G_GA	INDEL	16025.59	29	17	18	22	17	21	43	18	13	28	11	21	14	11
Chr18	12964277	G	GA	Chr18_12964G_GA	INDEL	16025.59	29	17	18	22	17	21	43	18	13	28	11	21	14	11
Chr18	12964277	G	GA	Chr18_12964G_GA	INDEL	16025.59	29	17	18	22	17	21	43	18	13	28	11	21	14	11
Chr18	12964277	G	GA	Chr18_12964G_GA	INDEL	16025.59	29	17	18	22	17	21	43	18	13	28	11	21	14	11
Chr18	12964277	G	GA	Chr18_12964G_GA	INDEL	16025.59	29	17	18	22	17	21	43	18	13	28	11	21	14	11
Chr18	12994201	AAT	A	Chr18_12994AAT_A	INDEL	4660.24	19	13	9	0	0	0	0	0	11	12	7	11	6	5
Chr18	12994201	AAT	A	Chr18_12994AAT_A	INDEL	4660.24	19	13	9	0	0	0	0	0	11	12	7	11	6	5
Chr18	12994201	AAT	A	Chr18_12994AAT_A	INDEL	4660.24	19	13	9	0	0	0	0	0	11	12	7	11	6	5
Chr18	12994201	AAT	A	Chr18_12994AAT_A	INDEL	4660.24	19	13	9	0	0	0	0	0	11	12	7	11	6	5
Chr18	12994201	AAT	A	Chr18_12994AAT_A	INDEL	4660.24	19	13	9	0	0	0	0	0	11	12	7	11	6	5
Chr18	12994201	AAT	A	Chr18_12994AAT_A	INDEL	4660.24	19	13	9	0	0	0	0	0	11	12	7	11	6	5
Chr18	12994201	AAT	A	Chr18_12994AAT_A	INDEL	4660.24	19	13	9	0	0	0	0	0	11	12	7	11	6	5
Chr18	12994201	AAT	A	Chr18_12994AAT_A	INDEL	4660.24	19	13	9	0	0	0	0	0	11	12	7	11	6	5
Chr18	12994201	AAT	A	Chr18_12994AAT_A	INDEL	4660.24	19	13	9	0	0	0	0	0	11	12	7	11	6	5
Chr18	13002985	TTATATA	T	Chr18_13002TTATATA_T	INDEL	526.37	0	0	0	0	0	0	0	0	0	0	0	0	0	14
Chr18	13002985	TTATATA	T	Chr18_13002TTATATA_T	INDEL	526.37	0	0	0	0	0	0	0	0	0	0	0	0	0	14
Chr18	13002985	TTATATA	T	Chr18_13002TTATATA_T	INDEL	526.37	0	0	0	0	0	0	0	0	0	0	0	0	0	14
Chr18	13002985	TTATATA	T	Chr18_13002TTATATA_T	INDEL	526.37	0	0	0	0	0	0	0	0	0	0	0	0	0	14
Chr18	13002985	TTATATA	T	Chr18_13002TTATATA_T	INDEL	526.37	0	0	0	0	0	0	0	0	0	0	0	0	0	14
Chr18	13002985	TTATATA	T	Chr18_13002TTATATA_T	INDEL	526.37	0	0	0	0	0	0	0	0	0	0	0	0	0	14
Chr18	13002985	TTATATA	T	Chr18_13002TTATATA_T	INDEL	526.37	0	0	0	0	0	0	0	0	0	0	0	0	0	14
Chr18	13002985	TTATATA	T	Chr18_13002TTATATA_T	INDEL	526.37	0	0	0	0	0	0	0	0	0	0	0	0	0	14
Chr18	13002985	TTATATA	T	Chr18_13002TTATATA_T	INDEL	526.37	0	0	0	0	0	0	0	0	0	0	0	0	0	14
Chr18	13005533	TTA	T	Chr18_13005TTA_T	INDEL	1953.24	0	0	0	8	9	15	24	8	0	1	0	0	0	1
Chr18	13005533	TTA	T	Chr18_13005TTA_T	INDEL	1953.24	0	0	0	8	9	15	24	8	0	1	0	0	0	1
Chr18	13005533	TTA	T	Chr18_13005TTA_T	INDEL	1953.24	0	0	0	8	9	15	24	8	0	1	0	0	0	1
Chr18	13005533	TTA	T	Chr18_13005TTA_T	INDEL	1953.24	0	0	0	8	9	15	24	8	0	1	0	0	0	1
Chr18	13005533	TTA	T	Chr18_13005TTA_T	INDEL	1953.24	0	0	0	8	9	15	24	8	0	1	0	0	0	1
Chr18	13005533	TTA	T	Chr18_13005TTA_T	INDEL	1953.24	0	0	0	8	9	15	24	8	0	1	0	0	0	1
Chr18	13005533	TTA	T	Chr18_13005TTA_T	INDEL	1953.24	0	0	0	8	9	15	24	8	0	1	0	0	0	1
Chr18	13005533	TTA	T	Chr18_13005TTA_T	INDEL	1953.24	0	0	0	8	9	15	24	8	0	1	0	0	0	1
Chr18	13005533	TTA	T	Chr18_13005TTA_T	INDEL	1953.24	0	0	0	8	9	15	24	8	0	1	0	0	0	1
Chr18	13036554	G	A	Chr18_13036G_A	SNP	18067.63	21	11	17	17	18	21	24	22	26	29	18	20	13	13
Chr18	13849612	C	A	Chr18_13849C_A	SNP	17759.63	29	7	16	17	17	23	44	28	17	21	16	18	13	7
Chr18	13876722	C	A	Chr18_13876C_A	SNP	10496.63	14	6	10	9	20	15	13	12	17	13	7	11	4	5
Chr18	13983044	T	TTA	Chr18_13983T_TTA	INDEL	6218.24	23	5	7	0	1	0	0	0	12	14	12	20	4	9
Chr18	13983044	T	TTA	Chr18_13983T_TTA	INDEL	6218.24	23	5	7	0	1	0	0	0	12	14	12	20	4	9
Chr18	13983044	T	TTA	Chr18_13983T_TTA	INDEL	6218.24	23	5	7	0	1	0	0	0	12	14	12	20	4	9
Chr18	14039143	C	A	Chr18_14039C_A	SNP	20001.63	30	10	23	28	17	27	29	24	22	18	8	20	11	18
Chr18	14039143	C	A	Chr18_14039C_A	SNP	20001.63	30	10	23	28	17	27	29	24	22	18	8	20	11	18
Chr18	14039143	C	A	Chr18_14039C_A	SNP	20001.63	30	10	23	28	17	27	29	24	22	18	8	20	11	18
Chr18	14039143	C	A	Chr18_14039C_A	SNP	20001.63	30	10	23	28	17	27	29	24	22	18	8	20	11	18
Chr18	14039143	C	A	Chr18_14039C_A	SNP	20001.63	30	10	23	28	17	27	29	24	22	18	8	20	11	18
Chr18	14039143	C	A	Chr18_14039C_A	SNP	20001.63	30	10	23	28	17	27	29	24	22	18	8	20	11	18
Chr18	14039143	C	A	Chr18_14039C_A	SNP	20001.63	30	10	23	28	17	27	29	24	22	18	8	20	11	18
Chr18	14039143	C	A	Chr18_14039C_A	SNP	20001.63	30	10	23	28	17	27	29	24	22	18	8	20	11	18
Chr18	14039143	C	A	Chr18_14039C_A	SNP	20001.63	30	10	23	28	17	27	29	24	22	18	8	20	11	18
Chr18	14039143	C	A	Chr18_14039C_A	SNP	20001.63	30	10	23	28	17	27	29	24	22	18	8	20	11	18
Chr18	14039143	C	A	Chr18_14039C_A	SNP	20001.63	30	10	23	28	17	27	29	24	22	18	8	20	11	18
Chr18	14039143	C	A	Chr18_14039C_A	SNP	20001.63	30	10	23	28	17	27	29	24	22	18	8	20	11	18
Chr18	14039143	C	A	Chr18_14039C_A	SNP	20001.63	30	10	23	28	17	27	29	24	22	18	8	20	11	18
Chr18	14039145	T	A	Chr18_14039T_A	SNP	20001.63	29	10	23	29	16	28	29	25	22	19	8	20	11	18
Chr18	14039145	T	A	Chr18_14039T_A	SNP	20001.63	29	10	23	29	16	28	29	25	22	19	8	20	11	18
Chr18	14039145	T	A	Chr18_14039T_A	SNP	20001.63	29	10	23	29	16	28	29	25	22	19	8	20	11	18
Chr18	14039145	T	A	Chr18_14039T_A	SNP	20001.63	29	10	23	29	16	28	29	25	22	19	8	20	11	18
Chr18	14039145	T	A	Chr18_14039T_A	SNP	20001.63	29	10	23	29	16	28	29	25	22	19	8	20	11	18
Chr18	14039145	T	A	Chr18_14039T_A	SNP	20001.63	29	10	23	29	16	28	29	25	22	19	8	20	11	18
Chr18	14039145	T	A	Chr18_14039T_A	SNP	20001.63	29	10	23	29	16	28	29	25	22	19	8	20	11	18
Chr18	14039145	T	A	Chr18_14039T_A	SNP	20001.63	29	10	23	29	16	28	29	25	22	19	8	20	11	18
Chr18	14039145	T	A	Chr18_14039T_A	SNP	20001.63	29	10	23	29	16	28	29	25	22	19	8	20	11	18
Chr18	14039145	T	A	Chr18_14039T_A	SNP	20001.63	29	10	23	29	16	28	29	25	22	19	8	20	11	18
Chr18	14039145	T	A	Chr18_14039T_A	SNP	20001.63	29	10	23	29	16	28	29	25	22	19	8	20	11	18
Chr18	14039145	T	A	Chr18_14039T_A	SNP	20001.63	29	10	23	29	16	28	29	25	22	19	8	20	11	18
Chr18	14039145	T	A	Chr18_14039T_A	SNP	20001.63	29	10	23	29	16	28	29	25	22	19	8	20	11	18
Chr18	14111914	A	AT	Chr18_14111A_AT	INDEL	14099.59	20	9	17	21	25	25	22	20	18	20	19	22	9	6
Chr18	14111914	A	AT	Chr18_14111A_AT	INDEL	14099.59	20	9	17	21	25	25	22	20	18	20	19	22	9	6
Chr18	14124773	G	A	Chr18_14124G_A	SNP	5797.29	0	0	0	17	23	24	31	22	0	0	0	0	0	0
Chr18	14155262	A	AAT	Chr18_14155A_AAT	INDEL	4787.24	24	16	10	0	0	0	1	1	13	14	12	6	14	4
Chr18	14155262	A	AAT	Chr18_14155A_AAT	INDEL	4787.24	24	16	10	0	0	0	1	1	13	14	12	6	14	4
Chr18	14155262	A	AAT	Chr18_14155A_AAT	INDEL	4787.24	24	16	10	0	0	0	1	1	13	14	12	6	14	4
Chr18	14218228	GTA	G	Chr18_14218GTA_G	INDEL	9827.59	15	14	11	18	11	18	20	19	15	14	7	12	7	6
Chr18	14218228	GTA	G	Chr18_14218GTA_G	INDEL	9827.59	15	14	11	18	11	18	20	19	15	14	7	12	7	6
Chr18	14218228	GTA	G	Chr18_14218GTA_G	INDEL	9827.59	15	14	11	18	11	18	20	19	15	14	7	12	7	6
Chr18	14417669	A	G	Chr18_14417A_G	SNP	14806.63	20	20	6	10	16	27	27	21	15	18	10	14	10	7
Chr18	14474910	TA	T	Chr18_14474TA_T	INDEL	9549.59	25	13	17	22	13	12	22	16	16	9	11	12	7	5
Chr18	14474910	TA	T	Chr18_14474TA_T	INDEL	9549.59	25	13	17	22	13	12	22	16	16	9	11	12	7	5
Chr18	14696783	G	T	Chr18_14696G_T	SNP	16534.63	23	17	15	14	19	26	36	24	29	33	16	11	7	15
Chr18	14714931	G	C	Chr18_14714G_C	SNP	17872.63	30	19	23	16	23	24	29	20	27	28	28	13	11	15
Chr18	14714931	G	C	Chr18_14714G_C	SNP	17872.63	30	19	23	16	23	24	29	20	27	28	28	13	11	15
Chr18	14714931	G	C	Chr18_14714G_C	SNP	17872.63	30	19	23	16	23	24	29	20	27	28	28	13	11	15
Chr18	14716474	A	T	Chr18_14716A_T	SNP	10428.63	19	11	9	15	12	11	13	18	21	21	23	23	10	8
Chr18	14716474	A	T	Chr18_14716A_T	SNP	10428.63	19	11	9	15	12	11	13	18	21	21	23	23	10	8
Chr18	14716474	A	T	Chr18_14716A_T	SNP	10428.63	19	11	9	15	12	11	13	18	21	21	23	23	10	8
Chr18	14795162	T	TTA	Chr18_14795T_TTA	INDEL	2123.93	0	0	0	0	0	0	1	0	18	20	10	4	5	5
Chr18	14795162	T	TTA	Chr18_14795T_TTA	INDEL	2123.93	0	0	0	0	0	0	1	0	18	20	10	4	5	5
Chr18	14795162	T	TTA	Chr18_14795T_TTA	INDEL	2123.93	0	0	0	0	0	0	1	0	18	20	10	4	5	5
Chr18	14845764	A	AAT	Chr18_14845A_AAT	INDEL	319.37	0	0	0	0	0	0	0	0	0	0	0	12	0	0
Chr18	14845764	A	AAT	Chr18_14845A_AAT	INDEL	319.37	0	0	0	0	0	0	0	0	0	0	0	12	0	0
Chr18	14845764	A	AAT	Chr18_14845A_AAT	INDEL	319.37	0	0	0	0	0	0	0	0	0	0	0	12	0	0
Chr18	14845764	A	AAT	Chr18_14845A_AAT	INDEL	319.37	0	0	0	0	0	0	0	0	0	0	0	12	0	0
Chr18	14845764	A	AAT	Chr18_14845A_AAT	INDEL	319.37	0	0	0	0	0	0	0	0	0	0	0	12	0	0
Chr18	14845764	A	AAT	Chr18_14845A_AAT	INDEL	319.37	0	0	0	0	0	0	0	0	0	0	0	12	0	0
Chr18	14845764	A	AAT	Chr18_14845A_AAT	INDEL	319.37	0	0	0	0	0	0	0	0	0	0	0	12	0	0
Chr18	14909630	A	T	Chr18_14909A_T	SNP	5219.29	0	0	0	26	12	22	28	23	0	0	0	0	0	0
Chr18	14909630	A	T	Chr18_14909A_T	SNP	5219.29	0	0	0	26	12	22	28	23	0	0	0	0	0	0
Chr18	14909630	A	T	Chr18_14909A_T	SNP	5219.29	0	0	0	26	12	22	28	23	0	0	0	0	0	0
Chr18	14994034	AAT	A	Chr18_14994AAT_A	INDEL	1660.24	0	0	0	12	8	12	14	12	0	0	0	1	0	1
Chr18	14994034	AAT	A	Chr18_14994AAT_A	INDEL	1660.24	0	0	0	12	8	12	14	12	0	0	0	1	0	1
Chr18	14994034	AAT	A	Chr18_14994AAT_A	INDEL	1660.24	0	0	0	12	8	12	14	12	0	0	0	1	0	1
Chr18	14994034	AAT	A	Chr18_14994AAT_A	INDEL	1660.24	0	0	0	12	8	12	14	12	0	0	0	1	0	1
Chr18	14994034	AAT	A	Chr18_14994AAT_A	INDEL	1660.24	0	0	0	12	8	12	14	12	0	0	0	1	0	1
Chr18	14994034	AAT	A	Chr18_14994AAT_A	INDEL	1660.24	0	0	0	12	8	12	14	12	0	0	0	1	0	1
Chr18	14994034	AAT	A	Chr18_14994AAT_A	INDEL	1660.24	0	0	0	12	8	12	14	12	0	0	0	1	0	1
Chr18	15084058	C	CTA	Chr18_15084C_CTA	INDEL	754.37	1	0	2	0	0	1	25	0	2	0	0	0	0	1
Chr18	15084058	C	CTA	Chr18_15084C_CTA	INDEL	754.37	1	0	2	0	0	1	25	0	2	0	0	0	0	1
Chr18	15084058	C	CTA	Chr18_15084C_CTA	INDEL	754.37	1	0	2	0	0	1	25	0	2	0	0	0	0	1
Chr18	15084058	C	CTA	Chr18_15084C_CTA	INDEL	754.37	1	0	2	0	0	1	25	0	2	0	0	0	0	1
Chr18	15084058	C	CTA	Chr18_15084C_CTA	INDEL	754.37	1	0	2	0	0	1	25	0	2	0	0	0	0	1
Chr18	15270363	C	T	Chr18_15270C_T	SNP	6276.24	0	0	0	21	19	30	28	33	0	0	0	0	0	0
Chr18	15312317	T	TA	Chr18_15312T_TA	INDEL	16080.59	29	17	15	16	21	29	40	24	29	14	16	21	7	11
Chr18	15312317	T	TA	Chr18_15312T_TA	INDEL	16080.59	29	17	15	16	21	29	40	24	29	14	16	21	7	11
Chr18	15312317	T	TA	Chr18_15312T_TA	INDEL	16080.59	29	17	15	16	21	29	40	24	29	14	16	21	7	11
Chr18	15312317	T	TA	Chr18_15312T_TA	INDEL	16080.59	29	17	15	16	21	29	40	24	29	14	16	21	7	11
Chr18	15312317	T	TA	Chr18_15312T_TA	INDEL	16080.59	29	17	15	16	21	29	40	24	29	14	16	21	7	11
Chr18	15343051	A	T	Chr18_15343A_T	SNP	19874.63	28	15	23	24	9	37	25	18	24	20	21	14	13	12
Chr18	15343138	G	A	Chr18_15343G_A	SNP	20242.63	34	16	18	27	13	42	18	24	33	33	13	14	15	13
Chr18	15369890	A	AT	Chr18_15369A_AT	INDEL	16001.59	30	18	11	17	14	25	24	29	25	34	11	17	8	11
Chr18	15385520	G	T	Chr18_15385G_T	SNP	12299.63	22	13	5	18	14	11	17	20	11	17	13	13	7	7
Chr18	15385520	G	T	Chr18_15385G_T	SNP	12299.63	22	13	5	18	14	11	17	20	11	17	13	13	7	7
Chr18	15385520	G	T	Chr18_15385G_T	SNP	12299.63	22	13	5	18	14	11	17	20	11	17	13	13	7	7
Chr18	15385520	G	T	Chr18_15385G_T	SNP	12299.63	22	13	5	18	14	11	17	20	11	17	13	13	7	7
Chr18	15385520	G	T	Chr18_15385G_T	SNP	12299.63	22	13	5	18	14	11	17	20	11	17	13	13	7	7
Chr18	15385520	G	T	Chr18_15385G_T	SNP	12299.63	22	13	5	18	14	11	17	20	11	17	13	13	7	7
Chr18	15385520	G	T	Chr18_15385G_T	SNP	12299.63	22	13	5	18	14	11	17	20	11	17	13	13	7	7
Chr18	15385520	G	T	Chr18_15385G_T	SNP	12299.63	22	13	5	18	14	11	17	20	11	17	13	13	7	7
Chr18	15517206	C	CT	Chr18_15517C_CT	INDEL	1637.93	0	0	0	1	0	0	0	0	12	14	5	11	6	9
Chr18	15517206	C	CT	Chr18_15517C_CT	INDEL	1637.93	0	0	0	1	0	0	0	0	12	14	5	11	6	9
Chr18	15517206	C	CT	Chr18_15517C_CT	INDEL	1637.93	0	0	0	1	0	0	0	0	12	14	5	11	6	9
Chr18	15517206	C	CT	Chr18_15517C_CT	INDEL	1637.93	0	0	0	1	0	0	0	0	12	14	5	11	6	9
Chr18	15517206	C	CT	Chr18_15517C_CT	INDEL	1637.93	0	0	0	1	0	0	0	0	12	14	5	11	6	9
Chr18	15521538	A	AAT	Chr18_15521A_AAT	INDEL	958.35	21	4	13	0	0	2	1	1	1	0	1	0	1	1
Chr18	15521538	A	AAT	Chr18_15521A_AAT	INDEL	958.35	21	4	13	0	0	2	1	1	1	0	1	0	1	1
Chr18	15521538	A	AAT	Chr18_15521A_AAT	INDEL	958.35	21	4	13	0	0	2	1	1	1	0	1	0	1	1
Chr18	15521538	A	AAT	Chr18_15521A_AAT	INDEL	958.35	21	4	13	0	0	2	1	1	1	0	1	0	1	1
Chr18	15521538	A	AAT	Chr18_15521A_AAT	INDEL	958.35	21	4	13	0	0	2	1	1	1	0	1	0	1	1
Chr18	15521538	A	AAT	Chr18_15521A_AAT	INDEL	958.35	21	4	13	0	0	2	1	1	1	0	1	0	1	1
Chr18	15553737	G	GT	Chr18_15553G_GT	INDEL	16084.59	23	9	13	20	15	36	46	31	17	18	12	30	8	7
Chr18	15553737	G	GT	Chr18_15553G_GT	INDEL	16084.59	23	9	13	20	15	36	46	31	17	18	12	30	8	7
Chr18	15553737	G	GT	Chr18_15553G_GT	INDEL	16084.59	23	9	13	20	15	36	46	31	17	18	12	30	8	7
Chr18	15554249	A	T	Chr18_15554A_T	SNP	19796.63	47	12	14	29	31	26	22	17	29	25	17	20	15	14
Chr18	15554249	A	T	Chr18_15554A_T	SNP	19796.63	47	12	14	29	31	26	22	17	29	25	17	20	15	14
Chr18	15656609	A	AT	Chr18_15656A_AT	INDEL	17334.59	24	18	23	25	18	24	31	35	25	22	19	22	15	16

Chr18	15704482	G	A	Chr18_15704G_A	SNP	6542.29	0	0	0	27	14	23	35	31	0	0	0	0	0	0	15	19	14	27	14	23	35	31	25	25	21	23	16	15	0	0	0	1	1	1	1	1	0	0	0	0	0	0	0	5	5	0	0	5	0	intergenic_re	MODIFIER	Pp3c18_225	Pp3c18_225	intergenic_re	Pp3c18_225	NA	NA	n.15704482G	NA	NA	NA	NA	NA
Chr18	15732967	T	A	Chr18_15732T_A	SNP	13438.63	22	12	8	24	11	17	28	15	23	15	7	8	12	10	22	12	8	24	11	17	28	15	23	15	7	8	12	10	1	1	1	1	1	1	1	1	1	1	1	1	1	1	0	15	15	0	7	5	3	intergenic_re	MODIFIER	Pp3c18_225	Pp3c18_225	intergenic_re	Pp3c18_225	NA	NA	n.15732967T	NA	NA	NA	NA	NA
Chr19	175721	A	G	Chr19_17572A_G	SNP	14190.63	18	8	11	24	15	19	13	14	26	21	9	13	9	7	18	8	11	24	15	19	13	14	26	21	9	13	9	7	1	1	1	1	1	1	1	1	1	1	1	1	1	1	0	15	15	0	7	5	3	upstream_ge	MODIFIER	Pp3c19_350	Pp3c19_350	transcript	PAC:3293890	protein_codi	NA	c.-4066T>C	NA	NA	NA	NA	4066
Chr19	175721	A	G	Chr19_17572A_G	SNP	14190.63	18	8	11	24	15	19	13	14	26	21	9	13	9	7	18	8	11	24	15	19	13	14	26	21	9	13	9	7	1	1	1	1	1	1	1	1	1	1	1	1	1	1	0	15	15	0	7	5	3	intergenic_re	MODIFIER	Pp3c19_350-	Pp3c19_350-	intergenic_re	Pp3c19_350-	NA	NA	n.175721A>G	NA	NA	NA	NA	NA
Chr19	371382	G	A	Chr19_37138G_A	SNP	4099.98	0	0	0	0	0	0	0	0	22	21	10	23	14	11	25	12	21	24	14	25	37	24	22	21	10	23	14	11	0	0	0	0	0	0	0	0	1	1	1	1	1	1	0	7	7	0	7	0	0	intergenic_re	MODIFIER	Pp3c19_930-	Pp3c19_930-	intergenic_re	Pp3c19_930-	NA	NA	n.371382G>A	NA	NA	NA	NA	NA
Chr19	421763	C	A	Chr19_42176C_A	SNP	14299.63	18	11	17	17	13	26	24	12	20	14	13	12	6	15	18	11	17	17	13	26	24	12	20	14	13	12	6	15	1	1	1	1	1	1	1	1	1	1	1	1	1	1	0	15	15	0	7	5	3	upstream_ge	MODIFIER	Pp3c19_990	Pp3c19_990	transcript	PAC:3293980	protein_codi	NA	c.-3419G>T	NA	NA	NA	NA	3349
Chr19	421763	C	A	Chr19_42176C_A	SNP	14299.63	18	11	17	17	13	26	24	12	20	14	13	12	6	15	18	11	17	17	13	26	24	12	20	14	13	12	6	15	1	1	1	1	1	1	1	1	1	1	1	1	1	1	0	15	15	0	7	5	3	upstream_ge	MODIFIER	Pp3c19_990	Pp3c19_990	transcript	PAC:3293981	protein_codi	NA	c.-3042G>T	NA	NA	NA	NA	2599
Chr19	421763	C	A	Chr19_42176C_A	SNP	14299.63	18	11	17	17	13	26	24	12	20	14	13	12	6	15	18	11	17	17	13	26	24	12	20	14	13	12	6	15	1	1	1	1	1	1	1	1	1	1	1	1	1	1	0	15	15	0	7	5	3	upstream_ge	MODIFIER	Pp3c19_990	Pp3c19_990	transcript	PAC:3293980	protein_codi	NA	c.-3042G>T	NA	NA	NA	NA	3042
Chr19	421763	C	A	Chr19_42176C_A	SNP	14299.63	18	11	17	17	13	26	24	12	20	14	13	12	6	15	18	11	17	17	13	26	24	12	20	14	13	12	6	15	1	1	1	1	1	1	1	1	1	1	1	1	1	1	0	15	15	0	7	5	3	intergenic_re	MODIFIER	Pp3c19_990-	Pp3c19_990-	intergenic_re	Pp3c19_990-	NA	NA	n.421763C>A	NA	NA	NA	NA	NA
Chr19	421775	C	A	Chr19_42177C_A	SNP	14655.63	20	12	17	18	16	28	24	11	21	15	14	12	7	14	20	12	17	18	16	28	24	11	21	15	14	12	7	14	1	1	1	1	1	1	1	1	1	1	1	1	1	1	0	15	15	0	7	5	3	upstream_ge	MODIFIER	Pp3c19_990	Pp3c19_990	transcript	PAC:3293980	protein_codi	NA	c.-3431G>T	NA	NA	NA	NA	3361
Chr19	421775	C	A	Chr19_42177C_A	SNP	14655.63	20	12	17	18	16	28	24	11	21	15	14	12	7	14	20	12	17	18	16	28	24	11	21	15	14	12	7	14	1	1	1	1	1	1	1	1	1	1	1	1	1	1	0	15	15	0	7	5	3	upstream_ge	MODIFIER	Pp3c19_990	Pp3c19_990	transcript	PAC:3293981	protein_codi	NA	c.-3054G>T	NA	NA	NA	NA	2611
Chr19	421775	C	A	Chr19_42177C_A	SNP	14655.63	20	12	17	18	16	28	24	11	21	15	14	12	7	14	20	12	17	18	16	28	24	11	21	15	14	12	7	14	1	1	1	1	1	1	1	1	1	1	1	1	1	1	0	15	15	0	7	5	3	upstream_ge	MODIFIER	Pp3c19_990	Pp3c19_990	transcript	PAC:3293980	protein_codi	NA	c.-3054G>T	NA	NA	NA	NA	3054
Chr19	421775	C	A	Chr19_42177C_A	SNP	14655.63	20	12	17	18	16	28	24	11	21	15	14	12	7	14	20	12	17	18	16	28	24	11	21	15	14	12	7	14	1	1	1	1	1	1	1	1	1	1	1	1	1	1	0	15	15	0	7	5	3	intergenic_re	MODIFIER	Pp3c19_990-	Pp3c19_990-	intergenic_re	Pp3c19_990-	NA	NA	n.421775C>A	NA	NA	NA	NA	NA
Chr19	446935	A	AAT	Chr19_44693A_AAT	INDEL	1179.35	16	14	9	0	0	0	0	2	0	0	0	0	0	1	17	14	9	11	18	15	23	19	19	13	6	7	7	7	0.941	1	1	0	0	0	0	0.105	0	0	0	0	0	0	0	3	3	1	0	0	3	upstream_ge	MODIFIER	Pp3c19_1060	Pp3c19_1060	transcript	PAC:3293875	protein_codi	NA	c.-3254_-325	NA	NA	NA	NA	3254
Chr19	446935	A	AAT	Chr19_44693A_AAT	INDEL	1179.35	16	14	9	0	0	0	0	2	0	0	0	0	0	1	17	14	9	11	18	15	23	19	19	13	6	7	7	7	0.941	1	1	0	0	0	0	0.105	0	0	0	0	0	0	0	3	3	1	0	0	3	upstream_ge	MODIFIER	Pp3c19_1061	Pp3c19_1061	transcript	PAC:3293983	protein_codi	NA	c.-3049_-304	NA	NA	NA	NA	3049
Chr19	446935	A	AAT	Chr19_44693A_AAT	INDEL	1179.35	16	14	9	0	0	0	0	2	0	0	0	0	0	1	17	14	9	11	18	15	23	19	19	13	6	7	7	7	0.941	1	1	0	0	0	0	0.105	0	0	0	0	0	0	0	3	3	1	0	0	3	upstream_ge	MODIFIER	Pp3c19_1062	Pp3c19_1062	transcript	PAC:3293850	protein_codi	NA	c.-3049_-304	NA	NA	NA	NA	3049
Chr19	446935	A	AAT	Chr19_44693A_AAT	INDEL	1179.35	16	14	9	0	0	0	0	2	0	0	0	0	0	1	17	14	9	11	18	15	23	19	19	13	6	7	7	7	0.941	1	1	0	0	0	0	0.105	0	0	0	0	0	0	0	3	3	1	0	0	3	downstream	MODIFIER	Pp3c19_1050	Pp3c19_1050	transcript	PAC:3293868	protein_codi	NA	c.*4391_*43	NA	NA	NA	NA	4392
Chr19	446935	A	AAT	Chr19_44693A_AAT	INDEL	1179.35	16	14	9	0	0	0	0	2	0	0	0	0	0	1	17	14	9	11	18	15	23	19	19	13	6	7	7	7	0.941	1	1	0	0	0	0	0.105	0	0	0	0	0	0	0	3	3	1	0	0	3	downstream	MODIFIER	Pp3c19_1050	Pp3c19_1050	transcript	PAC:3293868	protein_codi	NA	c.*4225_*42	NA	NA	NA	NA	4226
Chr19	446935	A	AAT	Chr19_44693A_AAT	INDEL	1179.35	16	14	9	0	0	0	0	2	0	0	0	0	0	1	17	14	9	11	18	15	23	19	19	13	6	7	7	7	0.941	1	1	0	0	0	0	0.105	0	0	0	0	0	0	0	3	3	1	0	0	3	intergenic_re	MODIFIER	Pp3c19_1061	Pp3c19_1061	intergenic_re	Pp3c19_1061	NA	NA	n.446935_44	NA	NA	NA	NA	NA
Chr19	517033	T	A	Chr19_51703T_A	SNP	15159.63	23	22	8	14	11	18	21	21	14	11	11	16	18	9	23	22	8	14	11	18	21	21	14	11	11	16	18	9	1	1	1	1	1	1	1	1	1	1	1	1	1	1	0	15	15	0	7	5	3	upstream_ge	MODIFIER	Pp3c19_1270	Pp3c19_1270	transcript	PAC:3293940	protein_codi	NA	c.-256A>T	NA	NA	NA	NA	256
Chr19	517033	T	A	Chr19_51703T_A	SNP	15159.63	23	22	8	14	11	18	21	21	14	11	11	16	18	9	23	22	8	14	11	18	21	21	14	11	11	16	18	9	1	1	1	1	1	1	1	1	1	1	1	1	1	1	0	15	15	0	7	5	3	intergenic_re	MODIFIER	Pp3c19_1270	Pp3c19_1270	intergenic_re	Pp3c19_1270	NA	NA	n.517033T>A	NA	NA	NA	NA	NA
Chr19	517038	C	A	Chr19_51703C_A	SNP	15161.63	22	22	7	14	12	16	21	22	15	11	12	17	20	8	22	22	7	14	12	16	21	22	15	11	12	17	20	8	1	1	1	1	1	1	1	1	1	1	1	1	1	1	0	15	15	0	7	5	3	upstream_ge	MODIFIER	Pp3c19_1270	Pp3c19_1270	transcript	PAC:3293940	protein_codi	NA	c.-261G>T	NA	NA	NA	NA	261
Chr19	517038	C	A	Chr19_51703C_A	SNP	15161.63	22	22	7	14	12	16	21	22	15	11	12	17	20	8	22	22	7	14	12	16	21	22	15	11	12	17	20	8	1	1	1	1	1	1	1	1	1	1	1	1	1	1	0	15	15	0	7	5	3	intergenic_re	MODIFIER	Pp3c19_1270	Pp3c19_1270	intergenic_re	Pp3c19_1270	NA	NA	n.517038C>A	NA	NA	NA	NA	NA
Chr19	517065	C	A	Chr19_51706C_A	SNP	13618.63	21	19	10	17	11	14	20	21	12	12	10	18	18	8	21	19	10	17	11	14	20	21	12	12	10	18	18	8	1	1	1	1	1	1	1	1	1	1	1	1	1	1	0	15	15	0	7	5	3	upstream_ge	MODIFIER	Pp3c19_1270	Pp3c19_1270	transcript	PAC:3293940	protein_codi	NA	c.-288G>T	NA	NA	NA	NA	288
[illegible]																																																																						

Chr19	2533947	G	T	Chr19_25339G_T	SNP	13770.63	20	6	19	15	12	14	26	15	16	16	15	10	4
Chr19	2533947	G	T	Chr19_25339G_T	SNP	13770.63	20	6	19	15	12	14	26	15	16	16	15	10	4
Chr19	2533947	G	T	Chr19_25339G_T	SNP	13770.63	20	6	19	15	12	14	26	15	16	16	15	10	4
Chr19	2533947	G	T	Chr19_25339G_T	SNP	13770.63	20	6	19	15	12	14	26	15	16	16	15	10	4
Chr19	2533947	G	T	Chr19_25339G_T	SNP	13770.63	20	6	19	15	12	14	26	15	16	16	15	10	4
Chr19	2533947	G	T	Chr19_25339G_T	SNP	13770.63	20	6	19	15	12	14	26	15	16	16	15	10	4
Chr19	2533952	G	C	Chr19_25339G_C	SNP	13323.63	19	6	17	15	12	14	25	15	16	18	15	11	4
Chr19	2533952	G	C	Chr19_25339G_C	SNP	13323.63	19	6	17	15	12	14	25	15	16	18	15	11	4
Chr19	2533952	G	C	Chr19_25339G_C	SNP	13323.63	19	6	17	15	12	14	25	15	16	18	15	11	4
Chr19	2533952	G	C	Chr19_25339G_C	SNP	13323.63	19	6	17	15	12	14	25	15	16	18	15	11	4
Chr19	2533952	G	C	Chr19_25339G_C	SNP	13323.63	19	6	17	15	12	14	25	15	16	18	15	11	4
Chr19	2533952	G	C	Chr19_25339G_C	SNP	13323.63	19	6	17	15	12	14	25	15	16	18	15	11	4
Chr19	2533952	G	C	Chr19_25339G_C	SNP	13323.63	19	6	17	15	12	14	25	15	16	18	15	11	4
Chr19	2533961	AC	A	Chr19_25339AC_A	INDEL	9825.59	18	6	17	16	10	14	26	13	16	22	14	11	4
Chr19	2533961	AC	A	Chr19_25339AC_A	INDEL	9825.59	18	6	17	16	10	14	26	13	16	22	14	11	4
Chr19	2533961	AC	A	Chr19_25339AC_A	INDEL	9825.59	18	6	17	16	10	14	26	13	16	22	14	11	4
Chr19	2533961	AC	A	Chr19_25339AC_A	INDEL	9825.59	18	6	17	16	10	14	26	13	16	22	14	11	4
Chr19	2533961	AC	A	Chr19_25339AC_A	INDEL	9825.59	18	6	17	16	10	14	26	13	16	22	14	11	4
Chr19	2533961	AC	A	Chr19_25339AC_A	INDEL	9825.59	18	6	17	16	10	14	26	13	16	22	14	11	4

[illegible]

Chr19	6891270	T	G	Chr19_68912T_G	SNP	12996.63	18	10	11	15	13	21	22	22	12	14	8	16	6	5
Chr19	6891270	T	G	Chr19_68912T_G	SNP	12996.63	18	10	11	15	13	21	22	22	12	14	8	16	6	5
Chr19	6891317	G	A	Chr19_68913G_A	SNP	9324.63	14	8	6	14	9	18	15	15	9	14	6	10	4	4
Chr19	6891317	G	A	Chr19_68913G_A	SNP	9324.63	14	8	6	14	9	18	15	15	9	14	6	10	4	4
Chr19	6892142	G	GA	Chr19_68921G_GA	INDEL	6874.59	12	4	9	9	12	12	17	8	10	10	13	5	10	6
Chr19	6892142	G	GA	Chr19_68921G_GA	INDEL	6874.59	12	4	9	9	12	12	17	8	10	10	13	5	10	6
Chr19	7124418	C	T	Chr19_71244C_T	SNP	20586.63	38	22	26	22	31	30	37	31	23	28	18	27	9	10
Chr19	7124907	A	G	Chr19_71249A_G	SNP	10406.63	10	6	6	9	7	13	12	7	28	13	16	19	6	8
Chr19	7354764	C	CA	Chr19_73547C_CA	INDEL	11573.59	27	7	14	14	9	16	18	15	7	23	7	10	10	8
Chr19	7389390	G	A	Chr19_73893G_A	SNP	9328.63	23	7	7	6	8	19	19	8	9	8	13	7	6	5
Chr19	7389410	G	A	Chr19_73894G_A	SNP	10881.63	26	7	7	6	7	22	21	11	9	13	14	9	6	6
Chr19	7399037	G	T	Chr19_73990G_T	SNP	19416.63	26	17	10	24	21	25	35	33	28	31	15	23	12	4
Chr19	7399037	G	T	Chr19_73990G_T	SNP	19416.63	26	17	10	24	21	25	35	33	28	31	15	23	12	4
Chr19	7399037	G	T	Chr19_73990G_T	SNP	19416.63	26	17	10	24	21	25	35	33	28	31	15	23	12	4
Chr19	7453175	TA	T	Chr19_74531TA_T	INDEL	13077.59	29	10	18	22	26	27	35	18	21	23	9	15	11	8
Chr19	7493304	A	AATATAT	Chr19_74933A_AATATAT	INDEL	13495.59	23	8	11	17	14	16	21	19	16	15	11	9	8	12
Chr19	7493304	A	AATATAT	Chr19_74933A_AATATAT	INDEL	13495.59	23	8	11	17	14	16	21	19	16	15	11	9	8	12
Chr19	7493304	A	AATATAT	Chr19_74933A_AATATAT	INDEL	13495.59	23	8	11	17	14	16	21	19	16	15	11	9	8	12
Chr19	7493304	A	AATATAT	Chr19_74933A_AATATAT	INDEL	13495.59	23	8	11	17	14	16	21	19	16	15	11	9	8	12
Chr19	7493304	A	AATATAT	Chr19_74933A_AATATAT	INDEL	13495.59	23	8	11	17	14	16	21	19	16	15	11	9	8	12
Chr19	7493304	A	AATATAT	Chr19_74933A_AATATAT	INDEL	13495.59	23	8	11	17	14	16	21	19	16	15	11	9	8	12
Chr19	7493304	A	AATATAT	Chr19_74933A_AATATAT	INDEL	13495.59	23	8	11	17	14	16	21	19	16	15	11	9	8	12
Chr19	7493304	A	AATATAT	Chr19_74933A_AATATAT	INDEL	13495.59	23	8	11	17	14	16	21	19	16	15	11	9	8	12
Chr19	7493304	A	AATATAT	Chr19_74933A_AATATAT	INDEL	13495.59	23	8	11	17	14	16	21	19	16	15	11	9	8	12

Chr19	10776488	A	T	Chr19_10776A_T	SNP	14231.63	23	12	11	13	17	15	21	18	10	17	13	5	5	18
Chr19	10776512	T	C	Chr19_10776T_C	SNP	14484.63	24	10	9	15	17	15	21	17	9	21	9	6	7	15
Chr19	10776512	T	C	Chr19_10776T_C	SNP	14484.63	24	10	9	15	17	15	21	17	9	21	9	6	7	15
Chr19	10776512	T	C	Chr19_10776T_C	SNP	14484.63	24	10	9	15	17	15	21	17	9	21	9	6	7	15
Chr19	10968303	TAA	T	Chr19_10968TAA_T	INDEL	14797.59	19	20	15	22	12	15	25	20	18	19	12	15	4	10
Chr19	10968303	TAA	T	Chr19_10968TAA_T	INDEL	14797.59	19	20	15	22	12	15	25	20	18	19	12	15	4	10
Chr19	10968303	TAA	T	Chr19_10968TAA_T	INDEL	14797.59	19	20	15	22	12	15	25	20	18	19	12	15	4	10
Chr19	11001006	T	TA	Chr19_11001T_TA	INDEL	15869.59	38	13	17	30	15	30	26	20	21	18	12	21	12	16
Chr19	11001006	T	TA	Chr19_11001T_TA	INDEL	15869.59	38	13	17	30	15	30	26	20	21	18	12	21	12	16
Chr19	11001006	T	TA	Chr19_11001T_TA	INDEL	15869.59	38	13	17	30	15	30	26	20	21	18	12	21	12	16
Chr19	11001080	C	CA	Chr19_11001C_CA	INDEL	15766.59	27	11	22	34	14	32	26	17	20	30	19	16	15	16
Chr19	11001080	C	CA	Chr19_11001C_CA	INDEL	15766.59	27	11	22	34	14	32	26	17	20	30	19	16	15	16
Chr19	11001080	C	CA	Chr19_11001C_CA	INDEL	15766.59	27	11	22	34	14	32	26	17	20	30	19	16	15	16
Chr19	11083477	C	A	Chr19_11083C_A	SNP	19079.63	34	13	20	24	17	26	32	31	18	24	18	20	11	9
Chr19	11289201	G	A	Chr19_11289G_A	SNP	16339.63	25	23	12	22	19	29	21	23	26	29	13	22	12	13
Chr19	11417901	T	TA	Chr19_11417T_TA	INDEL	19137.59	22	17	15	27	23	22	36	22	14	26	9	18	17	12
Chr19	11417901	T	TA	Chr19_11417T_TA	INDEL	19137.59	22	17	15	27	23	22	36	22	14	26	9	18	17	12
Chr19	11417901	T	TA	Chr19_11417T_TA	INDEL	19137.59	22	17	15	27	23	22	36	22	14	26	9	18	17	12
Chr19	11417901	T	TA	Chr19_11417T_TA	INDEL	19137.59	22	17	15	27	23	22	36	22	14	26	9	18	17	12
Chr19	11417906	AG	A	Chr19_11417AG_A	INDEL	19490.59	26	18	15	28	20	22	35	23	14	27	10	19	18	12
Chr19	11417906	AG	A	Chr19_11417AG_A	INDEL	19490.59	26	18	15	28	20	22	35	23	14	27	10	19	18	12
Chr19	11417906	AG	A	Chr19_11417AG_A	INDEL	19490.59	26	18	15	28	20	22	35	23	14	27	10	19	18	12
Chr19	11417906	AG	A	Chr19_11417AG_A	INDEL	19490.59	26	18	15	28	20	22	35	23	14	27	10	19	18	12
Chr19	11417956	A	T	Chr19_11417A_T	SNP	19812.63	36	15	18	27	25	22	34	26	19	27	7	13	14	17

Chr19	14049107	A	ACAACAT	Chr19_14049	A_ACAACAT	INDEL	22154.59	37	13	17	32	26	26	24	20	19	24	15	17	11	10	37	13
Chr19	14049108	G	C	Chr19_14049	G_C	SNP	22163.63	37	13	17	31	26	24	29	21	20	22	18	16	11	10	37	13
Chr19	14049728	G	T	Chr19_14049	G_T	SNP	15691.63	28	14	17	22	20	20	24	20	30	22	14	19	10	6	28	14
Chr19	14122461	G	T	Chr19_14122	G_T	SNP	18883.63	18	14	22	32	18	17	28	30	17	20	11	20	14	8	18	14
Chr19	14122461	G	T	Chr19_14122	G_T	SNP	18883.63	18	14	22	32	18	17	28	30	17	20	11	20	14	8	18	14
Chr19	14122461	G	T	Chr19_14122	G_T	SNP	18883.63	18	14	22	32	18	17	28	30	17	20	11	20	14	8	18	14
Chr19	14122461	G	T	Chr19_14122	G_T	SNP	18883.63	18	14	22	32	18	17	28	30	17	20	11	20	14	8	18	14
Chr19	14122461	G	T	Chr19_14122	G_T	SNP	18883.63	18	14	22	32	18	17	28	30	17	20	11	20	14	8	18	14
Chr19	14136649	C	T	Chr19_14136	C_T	SNP	20451.63	26	16	15	33	17	32	36	21	26	28	17	14	7	8	26	16
Chr19	14136649	C	T	Chr19_14136	C_T	SNP	20451.63	26	16	15	33	17	32	36	21	26	28	17	14	7	8	26	16
Chr19	14257001	AAT	A	Chr19_14257	AAT_A	INDEL	299.37	2	1	1	0	0	0	1	1	0	0	0	5	0	0	14	9
Chr19	14257001	AAT	A	Chr19_14257	AAT_A	INDEL	299.37	2	1	1	0	0	0	1	1	0	0	0	5	0	0	14	9
Chr19	14257001	AAT	A	Chr19_14257	AAT_A	INDEL	299.37	2	1	1	0	0	0	1	1	0	0	0	5	0	0	14	9
Chr19	14257740	CTA	C	Chr19_14257	CTA_C	INDEL	10845.59	13	8	8	16	10	24	31	26	13	17	7	12	6	6	13	8
Chr19	14257740	CTA	C	Chr19_14257	CTA_C	INDEL	10845.59	13	8	8	16	10	24	31	26	13	17	7	12	6	6	13	8
Chr19	14257740	CTA	C	Chr19_14257	CTA_C	INDEL	10845.59	13	8	8	16	10	24	31	26	13	17	7	12	6	6	13	8
Chr19	14362650	G	GA	Chr19_14362	G_GA	INDEL	14504.59	18	20	23	25	22	19	23	21	21	16	16	17	15	9	18	20
Chr19	14477128	A	C	Chr19_14477	A_C	SNP	20665.63	35	18	18	27	19	37	27	24	28	22	21	19	8	10	35	18
Chr19	14477129	G	T	Chr19_14477	G_T	SNP	20665.63	35	18	18	27	19	37	27	24	27	22	20	19	8	10	35	18
Chr19	14504128	T	A	Chr19_14504	T_A	SNP	17981.63	37	23	14	27	20	24	26	23	23	26	7	15	15	5	37	23
Chr19	14504184	G	T	Chr19_14504	G_T	SNP	17371.63	31	20	15	20	13	24	26	25	17	22	10	18	9	9	31	20
Chr19	14534858	C	A	Chr19_14534	C_A	SNP	10996.63	18	11	11	9	6	17	18	10	13	13	7	13	7	10	18	11
Chr19	14535016	G	A	Chr19_14535	G_A	SNP	14149.63	18	8	16	26	12	18	21	19	15	20	17	15	12	8	18	8
Chr19	14535066	G	A	Chr19_14535	G_A	SNP	16967.63	23	11	19	30	18	24	33	16	25	19	21	25	10	10	23	11
Chr19	14535216	G	C	Chr19_14535	G_C	SNP	18846.63	27	17	20	23	14	27	31	13	24	20	31	20	10	11	27	17
Chr19	14551292	G	C	Chr19_14551	G_C	SNP	18017.63	21	25	18	17	19	31	20	21	20	18	8	13	12	9	21	25
Chr19	14551316	T	A	Chr19_14551	T_A	SNP	17976.63	20	21	15	19	20	31	22	23	21	13	8	8	8	6	20	21
Chr19	14551337	C	A	Chr19_14551	C_A	SNP	17773.63	19	20	18	17	23	30	25	19	20	16	7	7	7	9	19	20
Chr19	14551423	C	G	Chr19_14551	C_G	SNP	17477.63	28	10	14	16	21	35	23	18	28	27	12	13	10	9	28	10
Chr19	14555638	C	T	Chr19_14555	C_T	SNP	12110.29	28	16	28	0	0	0	0	0	30	18	15	23	18	7	28	16
Chr19	14560715	T	A	Chr19_14560	T_A	SNP	11409.63	13	12	9	6	13	14	19	10	15	13	15	17	7	9	13	12
Chr19	14637523	T	A	Chr19_14637	T_A	SNP	19353.63	33	17	15	18	18	23	21	28	22	26	19	29	12	7	33	17
Chr19	14637531	C	A	Chr19_14637	C_A	SNP	19120.63	33	17	12	16	16	22	21	27	24	23	20	31	12	6	33	17
Chr19	14637610	A	AAT	Chr19_14637	A_AAT	INDEL	18400.59	39	16	12	21	19	19	20	20	22	16	19	24	8	7	39	16
Chr19	14637612	GT	G	Chr19_14637	GT_G	INDEL	18400.59	39	15	13	21	19	20	21	20	19	16	19	24	8	7	39	15
Chr19	14882489	AG	A	Chr19_14882	AG_A	INDEL	12627.59	18	7	10	12	16	17	18	17	17	15	13	5	9	4	18	7
Chr19	14882492	G	A	Chr19_14882	G_A	SNP	12636.63	19	7	10	12	18	17	18	17	16	15	14	5	9	4	19	7
Chr19	14934202	G	GA	Chr19_14934	G_GA	INDEL	19217.59	30	16	16	27	29	26	22	36	37	30	9	16	15	9	30	16
Chr19	15046108	C	T	Chr19_15046	C_T	SNP	18883.63	17	11	22	24	23	24	16	21	20	31	13	27	6	8	17	11
Chr19	15046126	C	G	Chr19_15046	C_G	SNP	19015.63	19	11	19	26	22	26	17	20	21	27	9	24	6	12	19	11
Chr19	15130600	T	TA	Chr19_15130	T_TA	INDEL	12519.59	29	13	11	16	14	25	24	25	20	21	16	13	10	13	29	13
Chr19	15130611	G	T	Chr19_15130	G_T	SNP	18848.63	34	15	14	17	15	29	28	26	22	26	18	12	10	14	34	15
Chr19	15154651	A	T	Chr19_15154	A_T	SNP	4098.98	0	0	0	0	0	0	0	0	12	23	21	15	10	7	38	15
Chr19	15184457	T	A	Chr19_15184	T_A	SNP	1061.39	14	11	5	0	0	0	0	0	0	0	0	0	0	0	14	11
Chr19	15184457	T	A	Chr19_15184	T_A	SNP	1061.39	14	11	5	0	0	0	0	0	0	0	0	0	0	0	14	11
Chr19	15184457	T	A	Chr19_15184	T_A	SNP	1061.39	14	11	5	0	0	0	0	0	0	0	0	0	0	0	14	11
Chr19	15184457	T	A	Chr19_15184	T_A	SNP	1061.39	14	11	5	0	0	0	0	0	0	0	0	0	0	0	14	11
Chr19	15217800	A	G	Chr19_15217	A_G	SNP	18577.63	17	16	18	27	18	28	30	20	26	19	8	21	19	15	17	16
Chr19	15217800	A	G	Chr19_15217	A_G	SNP	18577.63	17	16	18	27	18	28	30	20	26	19	8	21	19	15	17	16
Chr19	15217800	A	G	Chr19_15217	A_G	SNP	18577.63	17	16	18	27	18	28	30	20	26	19	8	21	19	15	17	16
Chr19	15217800	A	G	Chr19_15217	A_G	SNP	18577.63	17	16	18	27	18	28	30	20	26	19	8	21	19	15	17	16
Chr19	15354886	C	CT	Chr19_15354	C_CT	INDEL	11888.59	32	14	14	14	17	23	22	11	17	31	10	12	9	13	32	14
Chr19	15445240	G	A	Chr19_15445	G_A	SNP	2377.39	28	17	13	0	0	0	0	0	0	0	0	0	0	0	28	17
Chr19	15565649	G	GT	Chr19_15565	G_GT	INDEL	17935.59	29	13	17	22	14	30	32	23	28	22	25	11	10	13	29	13
Chr19	15565649	G	GT	Chr19_15565	G_GT	INDEL	17935.59	29	13	17	22	14	30	32	23	28	22	25	11	10	13	29	13
Chr19	15565649	G	GT	Chr19_15565	G_GT	INDEL	17935.59	29	13	17	22	14	30	32	23	28	22	25	11	10	13	29	13
Chr19	15565649	G	GT	Chr19_15565	G_GT	INDEL	17935.59	29	13	17	22	14	30	32	23	28	22	25	11	10	13	29	13
Chr19	15565649	G	GT	Chr19_15565	G_GT	INDEL	17935.59	29	13	17	22	14	30	32	23	28	22	25	11	10	13	29	13
Chr19	15565649	G	GT	Chr19_15565	G_GT	INDEL	17935.59	29	13	17	22	14	30	32	23	28	22	25	11	10	13	29	13
Chr19	15566223	CTATATA	C	Chr19_15566	CTATATA_C	INDEL	125.37	0	0	0	0	0	0	0	0	0	0	0	0	0	4	17	11
Chr19	15566223	CTATATA	C	Chr19_15566	CTATATA_C	INDEL	125.37	0	0	0	0	0	0	0	0	0	0	0	0	0	4	17	11
Chr19	15566223	CTATATA	C	Chr19_15566	CTATATA_C	INDEL	125.37	0	0	0	0	0	0	0	0	0	0	0	0	0	4	17	11
Chr19	15566223	CTATATA	C	Chr19_15566	CTATATA_C	INDEL	125.37	0	0	0	0	0	0	0	0	0	0	0	0	0	4	17	11
Chr19	15566223	CTATATA	C	Chr19_15566	CTATATA_C	INDEL	125.37	0	0	0	0	0	0	0	0	0	0	0	0	0	4	17	11
Chr19	15566223	CTATATA	C	Chr19_15566	CTATATA_C	INDEL	125.37	0	0	0	0	0	0	0	0	0	0	0	0	0	4	17	11
Chr20	220951	C	CTTTGTTCT	Chr20_22095	C_CTTTGTTC	INDEL	298416.59	1026	212	556	506	409	560	725	453	219	247	116	169	109	123	1027	212
Chr20	220951	C	CTTTGTTCT	Chr20_22095	C_CTTTGTTC	INDEL	298416.59	1026	212	556	506	409	560	725	453	219	247	116	169	109	123	1027	212
Chr20	220951	C	CTTTGTTCT	Chr20_22095	C_CTTTGTTC	INDEL	298416.59	1026	212	556	506	409	560	725	453	219	247	116	169	109	123	1027	212
Chr20	232421	T	TTCTAAACC	Chr20_23242	T_TTCTAAAC	INDEL	320911.59	120	66	124	69	72	135	137	97	41	32	24	41	19	24	120	66
Chr20	232421	T	TTCTAAACC	Chr20_23242	T_TTCTAAAC	INDEL	320911.59	120	66	124	69	72	135	137	97	41	32	24	41	19	24	120	66
Chr20	232503	A	C	Chr20_23250	A_C	SNP	1458478.59	4179	1179	2639	2255	1803	2565	3144	2138	1093	1348	635	926	525	724	4179	1179
Chr20	232503	A	C	Chr20_23250	A_C	SNP	1458478.59	4179	1179	2639	2255	1803	2565	3144	2138	1093	1348	635	926	525	724	4179	1179
Chr20	251226	T	C	Chr20_25122	T_C	SNP	19190.63	31	16	25	18	22	20	23	27	18	21	12	9	5	11	31	16

Chr20	1593555	T	TA	Chr20_15935	T_TA	INDEL	14261.59	[illegible]
Chr20	1593555	T	TA	Chr20_15935	T_TA	INDEL	14261.59	[illegible]
Chr20	1639512	GT	G	Chr20_16395	GT_G	INDEL	11889.59	[illegible]
Chr20	1639525	G	T	Chr20_16395	G_T	SNP	10945.63	[illegible]
Chr20	1731769	G	GTA	Chr20_17317	G_GTA	INDEL	2468.93	[illegible]
Chr20	1731769	G	GTA	Chr20_17317	G_GTA	INDEL	2468.93	[illegible]
Chr20	1852824	C	T	Chr20_18528	C_T	SNP	16456.63	[illegible]
Chr20	1875319	C	A	Chr20_18753	C_A	SNP	9405.29	[illegible]
Chr20	1900640	A	AATATAT	Chr20_19006	A_AATATAT	INDEL	12899.59	[illegible]
Chr20	1900640	A	AATATAT	Chr20_19006	A_AATATAT	INDEL	12899.59	[illegible]
Chr20	1900640	A	AATATAT	Chr20_19006	A_AATATAT	INDEL	12899.59	[illegible]
Chr20	1900640	A	AATATAT	Chr20_19006	A_AATATAT	INDEL	12899.59	[illegible]
Chr20	2024176	G	GA	Chr20_20241	G_GA	INDEL	15921.59	[illegible]
Chr20	2047285	G	T	Chr20_20472	G_T	SNP	22953.63	[illegible]
Chr20	2047304	G	T	Chr20_20473	G_T	SNP	22787.63	[illegible]
Chr20	2047378	G	T	Chr20_20473	G_T	SNP	20370.63	[illegible]
Chr20	2097072	T	C	Chr20_20970	T_C	SNP	15979.63	[illegible]
Chr20	2098746	G	A	Chr20_20987	G_A	SNP	16513.63	[illegible]
Chr20	2098746	G	A	Chr20_20987	G_A	SNP	16513.63	[illegible]
Chr20	2098800	A	C	Chr20_20988	A_C	SNP	12755.63	[illegible]
Chr20	2098800	A	C	Chr20_20988	A_C	SNP	12755.63	[illegible]
Chr20	2098831	C	T	Chr20_20988	C_T	SNP	11987.63	[illegible]
Chr20	2098831	C	T	Chr20_20988	C_T	SNP	11987.63	[illegible]
Chr20	2098839	G	A	Chr20_20988	G_A	SNP	11990.63	[illegible]
Chr20	2098839	G	A	Chr20_20988	G_A	SNP	11990.63	[illegible]
Chr20	2099682	T	A	Chr20_20996	T_A	SNP	18572.63	[illegible]
Chr20	2099682	T	A	Chr20_20996	T_A	SNP	18572.63	[illegible]
Chr20	2099684	G	A	Chr20_20996	G_A	SNP	18617.63	[illegible]
Chr20	2099684	G	A	Chr20_20996	G_A	SNP	18617.63	[illegible]
Chr20	2099687	C	A	Chr20_20996	C_A	SNP	18226.63	[illegible]
Chr20	2099687	C	A	Chr20_20996	C_A	SNP	18226.63	[illegible]
Chr20	2132406	A	ATAGAAGAG	Chr20_21324	A_ATAGAAG	INDEL	27032.59	[illegible]
Chr20	2283203	C	T	Chr20_22832	C_T	SNP	14365.63	[illegible]
Chr20	2283203	C	T	Chr20_22832	C_T	SNP	14365.63	[illegible]
Chr20	2283203	C	T	Chr20_22832	C_T	SNP	14365.63	[illegible]
Chr20	2371051	G	T	Chr20_23710	G_T	SNP	16303.63	[illegible]
Chr20	2445371	A	AAT	Chr20_24453	A_AAT	INDEL	9407.59	[illegible]
Chr20	2445371	A	AAT	Chr20_24453	A_AAT	INDEL	9407.59	[illegible]
Chr20	2445371	A	AAT	Chr20_24453	A_AAT	INDEL	9407.59	[illegible]
Chr20	2468245	T	TA	Chr20_24682	T_TA	INDEL	11270.59	[illegible]
Chr20	2511530	CT	C	Chr20_25115	CT_C	INDEL	19913.59	[illegible]
Chr20	2511539	C	T	Chr20_25115	C_T	SNP	20598.63	[illegible]
Chr20	2511594	C	CT	Chr20_25115	C_CT	INDEL	18875.59	[illegible]
Chr20	2511598	G	T	Chr20_25115	G_T	SNP	18923.63	[illegible]
Chr20	2511641	T	A	Chr20_25116	T_A	SNP	17254.63	[illegible]
Chr20	2511683	C	CT	Chr20_25116	C_CT	INDEL	14880.59	[illegible]
Chr20	2582561	G	C	Chr20_25825	G_C	SNP	5933.29	[illegible]
Chr20	2606452	A	AT	Chr20_26064	A_AT	INDEL	13538.59	[illegible]
Chr20	2871849	C	T	Chr20_28718	C_T	SNP	16757.63	[illegible]
Chr20	2913000	A	AAT	Chr20_29130	A_AAT	INDEL	1098.35	[illegible]
Chr20	2913000	A	AAT	Chr20_29130	A_AAT	INDEL	1098.35	[illegible]
Chr20	2913000	A	AAT	Chr20_29130	A_AAT	INDEL	1098.35	[illegible]
Chr20	2913000	A	AAT	Chr20_29130	A_AAT	INDEL	1098.35	[illegible]
Chr20	2913000	A	AAT	Chr20_29130	A_AAT	INDEL	1098.35	[illegible]
Chr20	2913000	A	AAT	Chr20_29130	A_AAT	INDEL	1098.35	[illegible]
Chr20	2913000	A	AAT	Chr20_29130	A_AAT	INDEL	1098.35	[illegible]
Chr20	2913000	A	AAT	Chr20_29130	A_AAT	INDEL	1098.35	[illegible]
Chr20	2913000	A	AAT	Chr20_29130	A_AAT	INDEL	1098.35	[illegible]
Chr20	2913000	A	AAT	Chr20_29130	A_AAT	INDEL	1098.35	[illegible]
Chr20	2913000	A	AAT	Chr20_29130	A_AAT	INDEL	1098.35	[illegible]
Chr20	2913000	A	AAT	Chr20_29130	A_AAT	INDEL	1098.35	[illegible]
Chr20	3242694	TTA	T	Chr20_32426	TTA_T	INDEL	3747.24	[illegible]
Chr20	3242694	TTA	T	Chr20_32426	TTA_T	INDEL	3747.24	[illegible]
Chr20	3242694	TTA	T	Chr20_32426	TTA_T	INDEL	3747.24	[illegible]
Chr20	3266300	CCT	C	Chr20_32663	CCT_C	INDEL	19071.59	[illegible]
Chr20	3266305	T	TCAG	Chr20_32663	T_TCAG	INDEL	19558.59	[illegible]
Chr20	3267011	G	A	Chr20_32670	G_A	SNP	1968.39	[illegible]
Chr20	3394354	C	CTA	Chr20_33943	C_CTA	INDEL	1081.35	[illegible]
Chr20	3429446	A	T	Chr20_34294	A_T	SNP	21363.63	[illegible]
Chr20	3566675	AAT	A	Chr20_35666	AAT_A	INDEL	6800.59	[illegible]
Chr20	3667447	A	AT	Chr20_36674	A_AT	INDEL	13507.59	[illegible]
Chr20	3667447	A	AT	Chr20_36674	A_AT	INDEL	13507.59	[illegible]
Chr20	3667447	A	AT	Chr20_36674	A_AT	INDEL	13507.59	[illegible]
Chr20	3667447	A	AT	Chr20_36674	A_AT	INDEL	13507.59	[illegible]
Chr20	3667447	A	AT	Chr20_36674	A_AT	INDEL	13507.59	[illegible]
Chr20	3678565	G	T	Chr20_36785	G_T	SNP	20615.63	[illegible]
Chr20	3678574	G	T	Chr20_36785	G_T	SNP	21005.63	[illegible]
Chr20	3678575	G	T	Chr20_36785	G_T	SNP	20960.63	[illegible]
Chr20	3678620	G	T	Chr20_36786	G_T	SNP	18868.63	[illegible]
Chr20	3681611	A	AAT	Chr20_36816	A_AAT	INDEL	10097.59	[illegible]
Chr20	3887669	G	T	Chr20_38876	G_T	SNP	20139.63	[illegible]
Chr20	3942365	T	TTA	Chr20_39423	T_TTA	INDEL	3939.24	[illegible]
Chr20	3942365	T	TTA	Chr20_39423	T_TTA	INDEL	3939.24	[illegible]
Chr20	3942365	T	TTA	Chr20_39423	T_TTA	INDEL	3939.24	[illegible]
Chr20	3955242	TA	T	Chr20_39552	TA_T	INDEL	14761.59	[illegible]
Chr20	4121539	C	A	Chr20_41215	C_A	SNP	13922.63	[illegible]
Chr20	4121539	C	A	Chr20_41215	C_A	SNP	13922.63	[illegible]
Chr20	4121539	C	A	Chr20_41215	C_A	SNP	13922.63	[illegible]
Chr20	4121539	C	A	Chr20_41215	C_A	SNP	13922.63	[illegible]
Chr20	4121539	C	A	Chr20_41215	C_A	SNP	13922.63	[illegible]
Chr20	4121569	G	T	Chr20_41215	G_T	SNP	15822.63	[illegible]
Chr20	4121569	G	T	Chr20_41215	G_T	SNP	15822.63	[illegible]
Chr20	4121569	G	T	Chr20_41215	G_T	SNP	15822.63	[illegible]
Chr20	4121569	G	T	Chr20_41215	G_T	SNP	15822.63	[illegible]
Chr20	4121569	G	T	Chr20_41215	G_T	SNP	15822.63	[illegible]
Chr20	4121579	C	CA	Chr20_41215	C_CA	INDEL	15249.59	[illegible]
Chr20	4121579	C	CA	Chr20_41215	C_CA	INDEL	15249.59	[illegible]
Chr20	4121579	C	CA	Chr20_41215	C_CA	INDEL	15249.59	[illegible]
Chr20	4121579	C	CA	Chr20_41215	C_CA	INDEL	15249.59	[illegible]
Chr20	4121579	C	CA	Chr20_41215	C_CA	INDEL	15249.59	[illegible]
Chr20	4263592	T	TA	Chr20_42635	T_TA	INDEL	16315.59	[illegible]
Chr20	4263689	G	A	Chr20_42636	G_A	SNP	19989.63	[illegible]
Chr20	4318921	G	GAT	Chr20_43189	G_GAT	INDEL	2972.24	[illegible]
Chr20	4318921	G	GAT	Chr20_43189	G_GAT	INDEL	2972.24	[illegible]
Chr20	4318921	G	GAT	Chr20_43189	G_GAT	INDEL	2972.24	[illegible]
Chr20	4323759	G	GTA	Chr20_43237	G_GTA	INDEL	3310.24	[illegible]
Chr20	4323759	G	GTA	Chr20_43237	G_GTA	INDEL	3310.24	[illegible]
Chr20	4323759	G	GTA	Chr20_43237	G_GTA	INDEL	3310.24	[illegible]
Chr20	4323759	G	GTA	Chr20_43237	G_GTA	INDEL	3310.24	[illegible]
Chr20	4323759	G	GTA	Chr20_43237	G_GTA	INDEL	3310.24	[illegible]
Chr20	4323759	G	GTA	Chr20_43237	G_GTA	INDEL	3310.24	[illegible]
Chr20	4392037	T	A	Chr20_43920	T_A	SNP	12912.29	[illegible]
Chr20	4421000	G	C	Chr20_44210	G_C	SNP	9366.63	[illegible]
Chr20	4421000	G	C	Chr20_44210	G_C	SNP	9366.63	[illegible]
Chr20	4421000	G	C	Chr20_44210	G_C	SNP	9366.63	[illegible]
Chr20	4421000	G	C	Chr20_44210	G_C	SNP	9366.63	[illegible]
Chr20	4421000	G	C	Chr20_44210	G_C	SNP	9366.63	[illegible]
Chr20	4421000	G	C	Chr20_44210	G_C	SNP	9366.63	[illegible]
Chr20	4421110	C	T	Chr20_44211	C_T	SNP	17710.63	[illegible]
Chr20	4421110	C	T	Chr20_44211	C_T	SNP	17710.63	[illegible]
Chr20	4421110	C	T	Chr20_44211	C_T	SNP	17710.63	[illegible]
Chr20	4421110	C	T	Chr20_44211	C_T	SNP	17710.63	[illegible]
Chr20	4421110	C	T	Chr20_44211	C_T	SNP	17710.63	[illegible]
Chr20	4421110	C	T	Chr20_44211	C_T	SNP	17710.63	[illegible]
Chr20	4485287	C	CA	Chr20_44852	C_CA	INDEL	13851.59	[illegible]
Chr20	4485287	C	CA	Chr20_44852	C_CA	INDEL	13851.59	[illegible]
Chr20	4485316	T	C	Chr20_44853	T_C	SNP	20474.63	[illegible]
Chr20	4485316	T	C	Chr20_44853	T_C	SNP	20474.63	[illegible]
Chr20	4485318	G	T	Chr20_44853	G_T	SNP	20474.63	[illegible]
Chr20	4485318	G	T	Chr20_44853	G_T	SNP	20474.63	[illegible]
Chr20	4507908	GAT	G	Chr20_45079	GAT_G	INDEL	1163.35	[illegible]
Chr20	4509912	AAT	A	Chr20_45099	AAT_A	INDEL	5430.59	[illegible]
Chr20	4598525	G	T	Chr20_45985	G_T	SNP	20367.63	[illegible]
Chr20	4598525	G	T	Chr20_45985	G_T	SNP	20367.63	[illegible]
Chr20	4599637	C	A	Chr20_45996	C_A	SNP	14355.63	[illegible]
Chr20	4740096	C	A	Chr20_47400	C_A	SNP	17728.63	[illegible]
Chr20	4740097	C	A	Chr20_47400	C_A	SNP	17728.63	[illegible]
Chr20	4740129	C	A	Chr20_47401	C_A	SNP	16385.63	[illegible]
Chr20	4861278	TC	T	Chr20_48612	TC_T	INDEL	15835.59	[illegible]
Chr20	4899965	A	AC	Chr20_48999	A_AC	INDEL	11060.59	[illegible]
Chr20	5150830	C	A	Chr20_51508	C_A	SNP	18411.63	[illegible]
Chr20	5150830	C	A	Chr20_51508	C_A	SNP	18411.63	[illegible]
Chr20	5161176	G	T	Chr20_51611	G_T	SNP	6159.29	[illegible]
Chr20	5167915	T	A	Chr20_51679	T_A	SNP	19670.63	[illegible]
Chr20	5167915	T	A	Chr20_51679	T_A	SNP	19670.63	[illegible]
Chr20	5167915	T	A	Chr20_51679	T_A	SNP	19670.63	[illegible]
Chr20	5167915	T	A	Chr20_51679	T_A	SNP	19670.63	[illegible]
Chr20	5167915	T	A	Chr20_51679	T_A	SNP	19670.63	[illegible]
Chr20	5167915	T	A	Chr20_51679	T_A	SNP	19670.63	[illegible]
Chr20	5167915	T	A	Chr20_51679	T_A	SNP	19670.63	[illegible]
Chr20	5175568	A	AAT	Chr20_51755	A_AAT	INDEL	3209.24	[illegible]
Chr20	5175568	A	AAT	Chr20_51755	A_AAT	INDEL	3209.24	[illegible]
Chr20	5175568	A	AAT	Chr20_51755	A_AAT	INDEL	3209.24	[illegible]
Chr20	5175568	A	AAT	Chr20_51755	A_AAT	INDEL	3209.24	[illegible]
Chr20	5175568	A	AAT	Chr20_51755	A_AAT	INDEL	3209.24	[illegible]
Chr20	5175568	A	AAT	Chr20_51755	A_AAT	INDEL	3209.24	[illegible]
Chr20	5175568	A	AAT	Chr20_51755	A_AAT	INDEL	3209.24	[illegible]
Chr20	5175568	A	AAT	Chr20_51755	A_AAT	INDEL	3209.24	[illegible]
Chr20	5175568	A	AAT	Chr20_51755	A_AAT	INDEL	3209.24	[illegible]
Chr20	5175568	A	AAT	Chr20_51755	A_AAT	INDEL	3209.24	[illegible]
Chr20	5175568	A	AAT	Chr20_51755	A_AAT	INDEL	3209.24	[illegible]
Chr20	5404852	C	CA	Chr20_54048	C_CA	INDEL	20387.59	[illegible]
Chr20	5587180	ATT	A	Chr20_55871	ATT_A	INDEL	12150.59	[illegible]
Chr20	5587180	ATT	A	Chr20_55871	ATT_A	INDEL	12150.59	[illegible]
Chr20	5587180	ATT	A	Chr20_55871	ATT_A	INDEL	12150.59	[illegible]
Chr20	5587180	ATT	A	Chr20_55871	ATT_A	INDEL	12150.59	[illegible]
Chr20	5587180	ATT	A	Chr20_55871	ATT_A	INDEL	12150.59	[illegible]
Chr20	5587180	ATT	A	Chr20_55871	ATT_A	INDEL	12150.59	[illegible]
Chr20	5587180	ATT	A	Chr20_55871	ATT_A	INDEL	12150.59	[illegible]
Chr20	5587180	ATT	A	Chr20_55871	ATT_A	INDEL	12150.59	[illegible]
Chr20	5587186	ACAG	A	Chr20_55871	ACAG_A	INDEL	12438.59	[illegible]
Chr20	5587186	ACAG	A	Chr20_55871	ACAG_A	INDEL	12438.59	[illegible]
Chr20	5587186	ACAG	A	Chr20_55871	ACAG_A	INDEL	12438.59	[illegible]
Chr20	5587186	ACAG	A	Chr20_55871	ACAG_A	INDEL	12438.59	[illegible]
Chr20	5587186	ACAG	A	Chr20_55871	ACAG_A	INDEL	12438.59	[illegible]
Chr20	5587186	ACAG	A	Chr20_55871	ACAG_A	INDEL	12438.59	[illegible]
Chr20	5587186	ACAG	A	Chr20_55871	ACAG_A	INDEL	12438.59	[illegible]
Chr20	5587186	ACAG	A	Chr20_55871	ACAG_A	INDEL	12438.59	[illegible]
Chr20	5587191	AG	A	Chr20_55871	AG_A	INDEL	12386.59	[illegible]
Chr20	5587191	AG	A	Chr20_55871	AG_A	INDEL	12386.59	[illegible]
Chr20	5587191	AG	A	Chr20_55871	AG_A	INDEL	12386.59	[illegible]
Chr20	5587191	AG	A	Chr20_55871	AG_A	INDEL	12386.59	[illegible]
Chr20	5587191	AG	A	Chr20_55871	AG_A	INDEL	12386.59	[illegible]
Chr20	5587191	AG	A	Chr20_55871	AG_A	INDEL	12386.59	[illegible]
Chr20	5587191	AG	A	Chr20_55871	AG_A	INDEL	12386.59	[illegible]
Chr20	5587191	AG	A	Chr20_55871	AG_A	INDEL	12386.59	[illegible]
Chr20	5587195	G	A	Chr20_55871	G_A	SNP	12575.63	[illegible]
Chr20	5587195	G	A	Chr20_55871	G_A	SNP	12575.63	[illegible]
Chr20	5587195	G	A	Chr20_55871	G_A	SNP	12575.63	[illegible]
Chr20	5587195	G	A	Chr20_55871	G_A	SNP	12575.63	[illegible]
Chr20	5587195	G	A	Chr20_55871	G_A	SNP	12575.63	[illegible]
Chr20	5587195	G	A	Chr20_55871	G_A	SNP	12575.63	[illegible]
Chr20	5587195	G	A	Chr20_55871	G_A	SNP	12575.63	[illegible]
Chr20	5587195	G	A	Chr20_55871	G_A	SNP	12575.63	[illegible]
Chr20	5587199	C	A	Chr20_55871	C_A	SNP	12890.63	[illegible]
Chr20	5587199	C	A	Chr20_55871	C_A	SNP	12890.63	[illegible]
Chr20	5587199	C	A	Chr20_55871	C_A	SNP	12890.63	[illegible]
Chr20	5587199	C	A	Chr20_55871	C_A	SNP	12890.63	[illegible]
Chr20	5587199	C	A	Chr20_55871	C_A	SNP	12890.63	[illegible]
Chr20	5587199	C	A	Chr20_55871	C_A	SNP	12890.63	[illegible]
Chr20	5587199	C	A	Chr20_55871	C_A	SNP	12890.63	[illegible]
Chr20	5587199	C	A	Chr20_55871	C_A	SNP	12890.63	[illegible]

Chr20	5587200	A	T	Chr20_55872	A_T	SNP	12845.63	17	10	15	17	10	19	22	8	13	12
Chr20	5587200	A	T	Chr20_55872	A_T	SNP	12845.63	17	10	15	17	10	19	22	8	13	12
Chr20	5587200	A	T	Chr20_55872	A_T	SNP	12845.63	17	10	15	17	10	19	22	8	13	12
Chr20	5587200	A	T	Chr20_55872	A_T	SNP	12845.63	17	10	15	17	10	19	22	8	13	12
Chr20	5587200	A	T	Chr20_55872	A_T	SNP	12845.63	17	10	15	17	10	19	22	8	13	12
Chr20	5587200	A	T	Chr20_55872	A_T	SNP	12845.63	17	10	15	17	10	19	22	8	13	12
Chr20	5587200	A	T	Chr20_55872	A_T	SNP	12845.63	17	10	15	17	10	19	22	8	13	12
Chr20	5587200	A	T	Chr20_55872	A_T	SNP	12845.63	17	10	15	17	10	19	22	8	13	12
Chr20	5587201	G	T	Chr20_55872	G_T	SNP	12755.63	17	9	15	17	10	19	21	8	13	12
Chr20	5587201	G	T	Chr20_55872	G_T	SNP	12755.63	17	9	15	17	10	19	21	8	13	12
Chr20	5587201	G	T	Chr20_55872	G_T	SNP	12755.63	17	9	15	17	10	19	21	8	13	12
Chr20	5587201	G	T	Chr20_55872	G_T	SNP	12755.63	17	9	15	17	10	19	21	8	13	12
Chr20	5587201	G	T	Chr20_55872	G_T	SNP	12755.63	17	9	15	17	10	19	21	8	13	12
Chr20	5587201	G	T	Chr20_55872	G_T	SNP	12755.63	17	9	15	17	10	19	21	8	13	12
Chr20	5587201	G	T	Chr20_55872	G_T	SNP	12755.63	17	9	15	17	10	19	21	8	13	12
Chr20	5587201	G	T	Chr20_55872	G_T	SNP	12755.63	17	9	15	17	10	19	21	8	13	12
Chr20	5587205	ACAGGAGG(	A	Chr20_55872	ACAGGAGG(	INDEL	12521.59	18	9	13	17	10	19	20	8	14	13
Chr20	5587205	ACAGGAGG(	A	Chr20_55872	ACAGGAGG(	INDEL	12521.59	18	9	13	17	10	19	20	8	14	13
Chr20	5587205	ACAGGAGG(	A	Chr20_55872	ACAGGAGG(	INDEL	12521.59	18	9	13	17	10	19	20	8	14	13
Chr20	5587205	ACAGGAGG(	A	Chr20_55872	ACAGGAGG(	INDEL	12521.59	18	9	13	17	10	19	20	8	14	13
Chr20	5587205	ACAGGAGG(	A	Chr20_55872	ACAGGAGG(	INDEL	12521.59	18	9	13	17	10	19	20	8	14	13
Chr20	5587205	ACAGGAGG(	A	Chr20_55872	ACAGGAGG(	INDEL	12521.59	18	9	13	17	10	19	20	8	14	13
Chr20	5587205	ACAGGAGG(	A	Chr20_55872	ACAGGAGG(	INDEL	12521.59	18	9	13	17	10	19	20	8	14	13
Chr20	5587205	ACAGGAGG(	A	Chr20_55872	ACAGGAGG(	INDEL	12521.59	18	9	13	17	10	19	20	8	14	13

Chr20	7424958	T	A	Chr20_74249T_A	SNP	1653.39	19	10	14	0	0	0	0	0	0	0	0	0	0	0	[illegible]
Chr20	7503003	G	A	Chr20_75030G_A	SNP	5916.29	0	0	0	24	19	32	29	23	0	0	0	0	0	0	[illegible]
Chr20	7503003	G	A	Chr20_75030G_A	SNP	5916.29	0	0	0	24	19	32	29	23	0	0	0	0	0	0	[illegible]
Chr20	7585428	G	GA	Chr20_75854G_GA	INDEL	14747.59	34	9	10	20	20	19	29	23	19	20	17	19	12	13	[illegible]
Chr20	7632926	G	T	Chr20_76329G_T	SNP	16217.63	18	8	11	17	16	21	15	23	22	17	13	16	8	13	[illegible]
Chr20	7781843	G	GTA	Chr20_77818G_GTA	INDEL	3214.24	0	0	0	23	7	22	19	18	0	0	0	1	1	0	[illegible]
Chr20	7781843	G	GTA	Chr20_77818G_GTA	INDEL	3214.24	0	0	0	23	7	22	19	18	0	0	0	1	1	0	[illegible]
Chr20	7944952	C	T	Chr20_79449C_T	SNP	2963.29	0	0	0	13	10	15	21	10	0	0	0	0	0	0	[illegible]
Chr20	8088848	T	C	Chr20_80888T_C	SNP	11169.63	24	7	9	17	11	12	14	15	8	17	11	8	6	5	[illegible]
Chr20	8088856	G	A	Chr20_80888G_A	SNP	11826.63	25	7	9	18	12	13	13	15	7	18	14	8	7	5	[illegible]
Chr20	8088862	A	T	Chr20_80888A_T	SNP	11627.63	24	6	9	18	12	13	14	14	7	17	14	10	9	5	[illegible]
Chr20	8088871	C	A	Chr20_80888C_A	SNP	11903.63	21	7	8	17	13	12	14	14	7	18	15	11	10	5	[illegible]
Chr20	8088873	T	TC	Chr20_80888T_TC	INDEL	12042.59	21	7	8	18	13	12	14	14	7	18	15	12	10	5	[illegible]
Chr20	8143946	C	T	Chr20_81439C_T	SNP	18971.63	23	13	21	26	16	30	36	25	25	21	17	24	15	10	[illegible]
Chr20	8219562	T	C	Chr20_82195T_C	SNP	16302.63	20	8	13	23	18	22	39	20	12	23	10	14	9	12	[illegible]
Chr20	8250475	C	CAT	Chr20_82504C_CAT	INDEL	6150.24	15	12	15	0	0	2	1	1	12	16	8	15	8	7	[illegible]
Chr20	8250475	C	CAT	Chr20_82504C_CAT	INDEL	6150.24	15	12	15	0	0	2	1	1	12	16	8	15	8	7	[illegible]
Chr20	8250475	C	CAT	Chr20_82504C_CAT	INDEL	6150.24	15	12	15	0	0	2	1	1	12	16	8	15	8	7	[illegible]
Chr20	8250475	C	CAT	Chr20_82504C_CAT	INDEL	6150.24	15	12	15	0	0	2	1	1	12	16	8	15	8	7	[illegible]
Chr20	8250475	C	CAT	Chr20_82504C_CAT	INDEL	6150.24	15	12	15	0	0	2	1	1	12	16	8	15	8	7	[illegible]
Chr20	8250475	C	CAT	Chr20_82504C_CAT	INDEL	6150.24	15	12	15	0	0	2	1	1	12	16	8	15	8	7	[illegible]
Chr20	8250475	C	CAT	Chr20_82504C_CAT	INDEL	6150.24	15	12	15	0	0	2	1	1	12	16	8	15	8	7	[illegible]
Chr20	8250475	C	CAT	Chr20_82504C_CAT	INDEL	6150.24	15	12	15	0	0	2	1	1	12	16	8	15	8	7	[illegible]
Chr20	8250475	C	CAT	Chr20_82504C_CAT	INDEL	6150.24	15	12	15	0	0	2	1	1	12	16	8	15	8	7	[illegible]
[illegible]																					

Chr20	12553846	C	CTA	Chr20_12553C_CTA	INDEL	8718.59	17	8	12	16	16	12	22	18	11	14	8	9	6	8	[illegible]
Chr20	12553846	C	CTA	Chr20_12553C_CTA	INDEL	8718.59	17	8	12	16	16	12	22	18	11	14	8	9	6	8	[illegible]
Chr20	12553846	C	CTA	Chr20_12553C_CTA	INDEL	8718.59	17	8	12	16	16	12	22	18	11	14	8	9	6	8	[illegible]
Chr20	12553846	C	CTA	Chr20_12553C_CTA	INDEL	8718.59	17	8	12	16	16	12	22	18	11	14	8	9	6	8	[illegible]
Chr20	12553846	C	CTA	Chr20_12553C_CTA	INDEL	8718.59	17	8	12	16	16	12	22	18	11	14	8	9	6	8	[illegible]
Chr20	12610096	GAT	G	Chr20_12610GAT_G	INDEL	817.93	0	0	0	1	0	0	1	0	7	7	7	9	4	6	[illegible]
Chr20	12610838	AAT	A	Chr20_12610AAT_A	INDEL	55.37	0	0	0	0	0	0	0	1	1	0	1	0	4	0	[illegible]
Chr20	12795798	G	T	Chr20_12795G_T	SNP	4910.98	0	0	0	0	0	0	0	0	24	26	20	21	8	11	[illegible]
Chr20	12864051	G	A	Chr20_12864G_A	SNP	19338.63	35	18	21	30	12	19	27	23	17	31	18	14	11	13	[illegible]
Chr20	12864051	G	A	Chr20_12864G_A	SNP	19338.63	35	18	21	30	12	19	27	23	17	31	18	14	11	13	[illegible]
Chr20	12864051	G	A	Chr20_12864G_A	SNP	19338.63	35	18	21	30	12	19	27	23	17	31	18	14	11	13	[illegible]
Chr20	12983730	G	GATTTATTTA	Chr20_12983G_GATTTATT	INDEL	24190.59	23	15	11	23	15	26	29	15	15	17	16	14	6	15	[illegible]
Chr20	13000034	G	GA	Chr20_13000G_GA	INDEL	16214.59	17	19	22	29	20	26	27	22	25	21	25	13	6	7	[illegible]
Chr20	13000075	G	T	Chr20_13000G_T	SNP	19468.63	23	19	22	20	20	27	28	25	23	23	23	16	7	8	[illegible]
Chr20	13000166	G	T	Chr20_13000G_T	SNP	17498.63	28	15	17	25	19	30	22	25	19	19	18	17	5	8	[illegible]
Chr20	13086249	C	T	Chr20_13086C_T	SNP	18392.63	30	13	11	21	17	18	31	21	26	17	13	14	8	16	[illegible]
Chr20	13086266	C	G	Chr20_13086C_G	SNP	19322.63	31	10	13	22	17	16	31	23	24	18	14	14	7	17	[illegible]
Chr20	13086268	C	T	Chr20_13086C_T	SNP	19170.63	31	10	13	21	17	15	31	26	23	18	14	14	7	17	[illegible]
Chr20	13086283	T	G	Chr20_13086T_G	SNP	18932.63	30	10	14	23	18	15	26	23	25	21	17	16	8	13	[illegible]
Chr20	13134603	A	G	Chr20_13134A_G	SNP	1427.39	22	12	11	0	0	0	0	0	0	0	0	0	0	0	[illegible]
Chr20	13134603	A	G	Chr20_13134A_G	SNP	1427.39	22	12	11	0	0	0	0	0	0	0	0	0	0	0	[illegible]
Chr20	13134603	A	G	Chr20_13134A_G	SNP	1427.39	22	12	11	0	0	0	0	0	0	0	0	0	0	0	[illegible]
Chr20	13210946	G	C	Chr20_13210G_C	SNP	20403.63	19	12	9	26	18	25	39	26	23	27	20	25	8	10	[illegible]
Chr20	13210946	G	C	Chr20_13210G_C	SNP	20403.63	19	12	9	26	18	25	39	26	23	27	20	25	8	10	[illegible]
[illegible]																					

Chr20	14647767	G	A	Chr20_14647G_A	SNP	9930.63	11	10	12	12	10	12	11	13	7	10	6	9	7	6	[illegible]
Chr20	14647767	G	A	Chr20_14647G_A	SNP	9930.63	11	10	12	12	10	12	11	13	7	10	6	9	7	6	[illegible]
Chr20	14647768	A	C	Chr20_14647A_C	SNP	9840.63	12	10	12	12	10	12	12	13	7	11	6	9	7	6	[illegible]
Chr20	14647768	A	C	Chr20_14647A_C	SNP	9840.63	12	10	12	12	10	12	12	13	7	11	6	9	7	6	[illegible]
Chr20	14647768	A	C	Chr20_14647A_C	SNP	9840.63	12	10	12	12	10	12	12	13	7	11	6	9	7	6	[illegible]
Chr20	14647768	A	C	Chr20_14647A_C	SNP	9840.63	12	10	12	12	10	12	12	13	7	11	6	9	7	6	[illegible]
Chr20	14648709	C	T	Chr20_14648C_T	SNP	11489.29	25	14	17	1	0	0	0	0	20	18	21	18	13	11	[illegible]
Chr20	14648709	C	T	Chr20_14648C_T	SNP	11489.29	25	14	17	1	0	0	0	0	20	18	21	18	13	11	[illegible]
Chr20	14648709	C	T	Chr20_14648C_T	SNP	11489.29	25	14	17	1	0	0	0	0	20	18	21	18	13	11	[illegible]
Chr20	14648709	C	T	Chr20_14648C_T	SNP	11489.29	25	14	17	1	0	0	0	0	20	18	21	18	13	11	[illegible]
Chr20	14666916	G	GAT	Chr20_14666G_GAT	INDEL	10007.59	32	8	16	22	10	16	16	16	8	12	6	10	13	12	[illegible]
Chr20	14671694	A	G	Chr20_14671A_G	SNP	21193.63	25	17	20	29	19	21	26	31	28	27	6	10	15	8	[illegible]
Chr20	14671694	A	G	Chr20_14671A_G	SNP	21193.63	25	17	20	29	19	21	26	31	28	27	6	10	15	8	[illegible]
Chr20	14671696	C	A	Chr20_14671C_A	SNP	21232.63	25	18	18	30	19	20	25	31	29	27	6	10	15	8	[illegible]
Chr20	14671696	C	A	Chr20_14671C_A	SNP	21232.63	25	18	18	30	19	20	25	31	29	27	6	10	15	8	[illegible]
Chr20	14671697	C	G	Chr20_14671C_G	SNP	21196.63	25	18	18	30	19	19	24	31	29	27	6	10	15	8	[illegible]
Chr20	14671697	C	G	Chr20_14671C_G	SNP	21196.63	25	18	18	30	19	19	24	31	29	27	6	10	15	8	[illegible]
Chr20	14747882	A	AAT	Chr20_14747A_AAT	INDEL	2189.24	0	2	0	14	8	15	18	13	1	0	0	0	0	0	[illegible]
Chr20	14747882	A	AAT	Chr20_14747A_AAT	INDEL	2189.24	0	2	0	14	8	15	18	13	1	0	0	0	0	0	[illegible]
Chr20	14752303	G	T	Chr20_14752G_T	SNP	11619.63	11	5	13	13	14	19	21	14	13	17	5	11	6	4	[illegible]
Chr20	14752345	A	AT	Chr20_14752A_AT	INDEL	7183.59	9	5	12	15	10	17	15	6	9	16	6	9	6	8	[illegible]
Chr20	14752428	G	T	Chr20_14752G_T	SNP	14956.63	26	14	10	20	17	25	14	17	9	20	10	14	6	6	[illegible]
Chr20	14752578	G	T	Chr20_14752G_T	SNP	18856.63	23	18	10	26	19	27	16	28	21	30	15	11	5	10	[illegible]
Chr20	14752583	G	T	Chr20_14752G_T	SNP	19070.63	23	17	9	28	20	30	16	30	21	30	15	11	4	11	[illegible]

[illegible]

Chr21	1575819	A	C	Chr21_15758A_C	SNP	41150.63	51	28	37	40	44	46	78	47	46	41	26	35	24	19	51	28	37	40	44	46	78	47	46	41	26	35	24	19	1	1	1	1	1	1	1	1	1	1	1	1	1	1	0	15	15	0	7	5	3	downstream	MODIFIER	Pp3c21_260	Pp3c21_260	transcript	PAC:3291596	protein_codi	NA	c.*4791T>G	NA	NA	NA	NA		4541
Chr21	1575819	A	C	Chr21_15758A_C	SNP	41150.63	51	28	37	40	44	46	78	47	46	41	26	35	24	19	51	28	37	40	44	46	78	47	46	41	26	35	24	19	1	1	1	1	1	1	1	1	1	1	1	1	1	1	0	15	15	0	7	5	3	downstream	MODIFIER	Pp3c21_259	Pp3c21_259	transcript	PAC:3291564	protein_codi	NA	c.*2958T>G	NA	NA	NA	NA		2958
Chr21	1575819	A	C	Chr21_15758A_C	SNP	41150.63	51	28	37	40	44	46	78	47	46	41	26	35	24	19	51	28	37	40	44	46	78	47	46	41	26	35	24	19	1	1	1	1	1	1	1	1	1	1	1	1	1	1	0	15	15	0	7	5	3	downstream	MODIFIER	Pp3c21_260	Pp3c21_260	transcript	PAC:3291597	protein_codi	NA	c.*4791T>G	NA	NA	NA	NA		4535
Chr21	1575819	A	C	Chr21_15758A_C	SNP	41150.63	51	28	37	40	44	46	78	47	46	41	26	35	24	19	51	28	37	40	44	46	78	47	46	41	26	35	24	19	1	1	1	1	1	1	1	1	1	1	1	1	1	1	0	15	15	0	7	5	3	downstream	MODIFIER	Pp3c21_260	Pp3c21_260	transcript	PAC:3291597	protein_codi	NA	c.*4791T>G	NA	NA	NA	NA		4535
Chr21	1575819	A	C	Chr21_15758A_C	SNP	41150.63	51	28	37	40	44	46	78	47	46	41	26	35	24	19	51	28	37	40	44	46	78	47	46	41	26	35	24	19	1	1	1	1	1	1	1	1	1	1	1	1	1	1	0	15	15	0	7	5	3	downstream	MODIFIER	Pp3c21_260	Pp3c21_260	transcript	PAC:3291597	protein_codi	NA	c.*4791T>G	NA	NA	NA	NA		4535
Chr21	1575819	A	C	Chr21_15758A_C	SNP	41150.63	51	28	37	40	44	46	78	47	46	41	26	35	24	19	51	28	37	40	44	46	78	47	46	41	26	35	24	19	1	1	1	1	1	1	1	1	1	1	1	1	1	1	0	15	15	0	7	5	3	downstream	MODIFIER	Pp3c21_260	Pp3c21_260	transcript	PAC:3291597	protein_codi	NA	c.*4791T>G	NA	NA	NA	NA		4535
Chr21	1575819	A	C	Chr21_15758A_C	SNP	41150.63	51	28	37	40	44	46	78	47	46	41	26	35	24	19	51	28	37	40	44	46	78	47	46	41	26	35	24	19	1	1	1	1	1	1	1	1	1	1	1	1	1	1	0	15	15	0	7	5	3	intergenic_re	MODIFIER	Pp3c21_2521	Pp3c21_2521	intergenic_re	Pp3c21_2521	NA	NA	n.1575819A>	NA	NA	NA	NA	NA	
Chr21	1575823	T	TTGG	Chr21_15758T_TTGG	INDEL	43166.59	48	29	35	38	45	47	78	48	44	43	26	36	21	18	48	29	35	38	45	47	78	48	44	43	26	36	21	18	1	1	1	1	1	1	1	1	1	1	1	1	1	1	0	15	15	0	7	5	3	upstream_ge	MODIFIER	Pp3c21_249	Pp3c21_249	transcript	PAC:3291597	protein_codi	NA	c.-2609_-260	NA	NA	NA	NA		2039
Chr21	1575823	T	TTGG	Chr21_15758T_TTGG	INDEL	43166.59	48	29	35	38	45	47	78	48	44	43	26	36	21	18	48	29	35	38	45	47	78	48	44	43	26	36	21	18	1	1	1	1	1	1	1	1	1	1	1	1	1	1	0	15	15	0	7	5	3	downstream	MODIFIER	Pp3c21_2521	Pp3c21_2521	transcript	PAC:3291689	protein_codi	NA	c.*1062_*10	NA	NA	NA	NA		1063
Chr21	1575823	T	TTGG	Chr21_15758T_TTGG	INDEL	43166.59	48	29	35	38	45	47	78	48	44	43	26	36	21	18	48	29	35	38	45	47	78	48	44	43	26	36	21	18	1	1	1	1	1	1	1	1	1	1	1	1	1	1	0	15	15	0	7	5	3	downstream	MODIFIER	Pp3c21_259	Pp3c21_259	transcript	PAC:3291564	protein_codi	NA	c.*2953_*29	NA	NA	NA	NA		2401
Chr21	1575823	T	TTGG	Chr21_15758T_TTGG	INDEL	43166.59	48	29	35	38	45	47	78	48	44	43	26	36	21	18	48	29	35	38	45	47	78	48	44	43	26	36	21	18	1	1	1	1	1	1	1	1	1	1	1	1	1	1	0	15	15	0	7	5	3	downstream	MODIFIER	Pp3c21_260	Pp3c21_260	transcript	PAC:3291596	protein_codi	NA	c.*4786_*47	NA	NA	NA	NA		4536
Chr21	1575823	T	TTGG	Chr21_15758T_TTGG	INDEL	43166.59	48	29	35	38	45	47	78	48	44	43	26	36	21	18	48	29	35	38	45	47	78	48	44	43	26	36	21	18	1	1	1	1	1	1	1	1	1	1	1	1	1	1	0	15	15	0	7	5	3	downstream	MODIFIER	Pp3c21_259	Pp3c21_259	transcript	PAC:3291564	protein_codi	NA	c.*2953_*29	NA	NA	NA	NA		2953
Chr21	1575823	T	TTGG	Chr21_15758T_TTGG	INDEL	43166.59	48	29	35	38	45	47	78	48	44	43	26	36	21	18	48	29	35	38	45	47	78	48	44	43	26	36	21	18	1	1	1	1	1	1	1	1	1	1	1	1	1	1	0	15	15	0	7	5	3	downstream	MODIFIER	Pp3c21_260	Pp3c21_260	transcript	PAC:3291597	protein_codi	NA	c.*4786_*47	NA	NA	NA	NA		4530
Chr21	1575823	T	TTGG	Chr21_15758T_TTGG	INDEL	43166.59	48	29	35	38	45	47	78	48	44	43	26	36	21	18	48	29	35	38	45	47	78	48	44	43	26	36	21	18	1	1	1	1	1	1	1	1	1	1	1	1	1	1	0	15	15	0	7	5	3	downstream	MODIFIER	Pp3c21_260	Pp3c21_260	transcript	PAC:3291597	protein_codi	NA	c.*4786_*47	NA	NA	NA	NA		4530
Chr21	1575823	T	TTGG	Chr21_15758T_TTGG	INDEL	43166.59	48	29	35	38	45	47	78	48	44	43	26	36	21	18	48	29	35	38	45	47	78	48	44	43	26	36	21	18	1	1	1	1	1	1	1	1	1	1	1	1	1	1	0	15	15	0	7	5	3	downstream	MODIFIER	Pp3c21_260	Pp3c21_260	transcript	PAC:3291597	protein_codi	NA	c.*4786_*47	NA	NA	NA	NA		4530
Chr21	1575823	T	TTGG	Chr21_15758T_TTGG	INDEL	43166.59	48	29	35	38	45	47	78	48	44	43	26	36	21	18	48	29	35	38	45	47	78	48	44	43	26	36	21	18	1	1	1	1	1	1	1	1	1	1	1	1	1	1	0	15	15	0	7	5	3	downstream	MODIFIER	Pp3c21_260	Pp3c21_260	transcript	PAC:3291597	protein_codi	NA	c.*4786_*47	NA	NA	NA	NA		4530
Chr21	1575823	T	TTGG	Chr21_15758T_TTGG	INDEL	43166.59	48	29	35	38	45	47	78	48	44	43	26	36	21	18	48	29	35	38	45	47	78	48	44	43	26	36	21	18	1	1	1	1	1	1	1	1	1	1	1	1	1	1	0	15	15	0	7	5	3	intergenic_re	MODIFIER	Pp3c21_2521	Pp3c21_2521	intergenic_re	Pp3c21_2521	NA	NA	n.1575823_1	NA	NA	NA	NA	NA	
Chr21	1575824	A	AGCACCGG	Chr21_15758A_AGCACCG	INDEL	42941.59	48	29	36	40	47	46	75	51	44	40	26	37	21	18	48	29	36	40	47	46	75	51	44	40	26	37	21	18	1	1	1	1	1	1	1	1	1	1	1	1	1	1	0	15	15	0	7	5	3	upstream_ge	MODIFIER	Pp3c21_249	Pp3c21_249	transcript	PAC:3291597	protein_codi	NA	c.-2610_-260	NA	NA	NA	NA		2040
Chr21	1575824	A	AGCACCGG	Chr21_15758A_AGCACCG	INDEL	42941.59	48	29	36	40	47	46	75	51	44	40	26	37	21	18	48	29	36	40	47	46	75	51	44	40	26	37	21	18	1	1	1	1	1	1	1	1	1	1	1	1	1	1	0	15	15	0	7	5	3	downstream	MODIFIER	Pp3c21_2521	Pp3c21_2521	transcript	PAC:3291689	protein_codi	NA	c.*1063_*10	NA	NA	NA	NA		1064
Chr21	1575824	A	AGCACCGG	Chr21_15758A_AGCACCG	INDEL	42941.59	48	29	36	40	47	46	75	51	44	40	26	37	21	18	48	29	36	40	47	46	75	51	44	40	26	37	21	18	1	1	1	1	1	1	1	1	1	1	1	1	1	1	0	15	15	0	7	5	3	downstream	MODIFIER	Pp3c21_259	Pp3c21_259	transcript	PAC:3291564	protein_codi	NA	c.*2952_*29	NA	NA	NA	NA		2400
Chr21	1575824	A	AGCACCGG	Chr21_15758A_AGCACCG	INDEL	42941.59	48	29	36	40	47	46	75	51	44	40	26	37	21	18	48	29	36	40	47	46	75	51	44	40	26	37	21	18	1	1	1	1	1	1	1	1	1	1	1	1	1	1	0	15	15	0	7	5	3	downstream	MODIFIER	Pp3c21_260	Pp3c21_260	transcript	PAC:3291596	protein_codi	NA	c.*4785_*47	NA	NA	NA	NA		4535
Chr21	1575824	A	AGCACCGG	Chr21_15758A_AGCACCG	INDEL	42941.59	48	29	36	40	47	46	75	51	44	40	26	37	21	18	48	29	36	40	47	46	75	51	44	40	26	37	21	18	1	1	1	1	1	1	1	1	1	1	1	1	1	1	0	15	15	0	7	5	3	downstream	MODIFIER	Pp3c21_259	Pp3c21_259	transcript	PAC:3291564	protein_codi	NA	c.*2952_*29	NA	NA	NA	NA		2952
Chr21	1575824	A	AGCACCGG	Chr21_15758A_AGCACCG	INDEL	42941.59	48	29	36	40	47	46	75	51	44	40	26	37	21	18	48	29	36	40	47	46	75	51	44	40	26	37	21	18	1	1	1	1	1	1	1	1	1	1	1	1	1	1	0	15	15	0	7	5	3	downstream	MODIFIER	Pp3c21_260	Pp3c21_260	transcript	PAC:3291597	protein_codi	NA	c.*4785_*47	NA	NA	NA	NA		4529
Chr21	1575824	A	AGCACCGG	Chr21_15758A_AGCACCG	INDEL	42941.59	48	29	36	40	47	46	75	51	44	40	26	37	21	18	48	29	36	40	47	46	75	51	44	40	26	37	21	18	1	1	1	1	1	1	1	1	1	1	1	1	1	1	0	15	15	0	7	5	3	downstream	MODIFIER	Pp3c21_260	Pp3c21_260	transcript	PAC:3291597	protein_codi	NA	c.*4785_*47	NA	NA	NA	NA		4529
Chr21	1575824	A	AGCACCGG	Chr21_15758A_AGCACCG	INDEL	42941.59	48	29	36	40	47	46	75	51	44	40	26	37	21	18	48	29	36	40	47	46	75	51	44	40	26	37	21	18	1	1	1	1	1	1	1	1	1	1	1	1	1	1	0	15	15	0	7	5	3	downstream	MODIFIER	Pp3c21_260	Pp3c21_260	transcript	PAC:3291597	protein_codi	NA	c.*4785_*47	NA	NA	NA	NA		4529
Chr21	1575824	A	AGCACCGG	Chr21_15758A_AGCACCG	INDEL	42941.59	48	29	36	40	47	46	75	51	44	40	26	37	21	18	48	29	36	40	47	46	75	51	44	40	26	37	21	18	1	1	1	1	1	1	1	1	1	1	1	1	1	1	0	15	15	0	7	5	3	downstream	MODIFIER	Pp3c21_260	Pp3c21_260	transcript	PAC:3291597	protein_codi	NA	c.*4785_*47	NA	NA	NA	NA		4529
Chr21	1575824	A	AGCACCGG	Chr21_15758A_AGCACCG	INDEL	42941.59	48	29	36	40	47	46	75	51	44	40	26	37	21	18	48	29	36	40	47	46	75	51	44	40	26	37	21	18	1	1	1	1	1	1	1	1	1	1	1	1	1	1	0	15	15	0	7	5	3	intergenic_re	MODIFIER	Pp3c21_2521	Pp3c21_2521	intergenic_re	Pp3c21_2521	NA	NA	n.1575824_1	NA	NA	NA	NA	NA	
Chr21	1575826	C	G	Chr21_15758C_G	SNP	41960.63	49	31	35	43	43	49	76	50	47	43	27	33	20	19	49	31	35	43	43	49	76	50	47	43	27	33	20	19	1	1	1	1	1	1	1	1	1	1	1	1	1	1	0	15	15	0	7	5	3	upstream_ge	MODIFIER	Pp3c21_249	Pp3c21_249	transcript	PAC:3291597	protein_codi	NA	c.-2611G>C	NA	NA	NA	NA		2041
Chr21	1575826	C	G	Chr21_15758C_G	SNP	41960.63	49	31	35	43	43	49	76	50	47	43	27	33	20	19	49	31	35	43	43	49	76	50	47	43	27	33	20	19	1	1	1	1	1	1	1	1	1	1	1	1	1	1	0	15	15	0	7	5	3	downstream	MODIFIER	Pp3c21_2521	Pp3c21_2521	transcript	PAC:3291689	protein_codi	NA	c.*1065C>G	NA	NA	NA	NA		1065
Chr21	1575826	C	G	Chr21_15758C_G	SNP	41960.63	49	31	35	43	43	49	76	50	47	43	27	33	20	19	49	31	35	43	43	49	76	50	47	43	27	33	20	19	1	1	1	1	1	1	1	1	1	1	1	1	1	1	0	15	15	0	7	5	3	downstream	MODIFIER	Pp3c21_259	Pp3c21_259	transcript	PAC:3291564	protein_codi	NA	c.*2951G>C	NA	NA	NA	NA		2399
Chr21	1575826	C	G	Chr21_15758C_G	SNP	41960.63	49	31	35	43	43	49	76	50	47	43	27	33	20	19	49	31	35	43	43	49	76	50	47	43	27	33	20	19	1	1	1	1	1	1	1	1	1	1	1	1	1	1	0	15	15	0	7	5	3	downstream	MODIFIER	Pp3c21_260	Pp3c21_260	transcript	PAC:3291596	protein_codi	NA	c.*4784G>C	NA	NA	NA	NA		4534
Chr21	1575826	C	G	Chr21_15758C_G	SNP	41960.63	49	31	35	43	43	49	76	50	47	43	27	33	20	19	49	31	35	43	43	49	76	50	47	43	27	33	20	19	1	1	1	1	1	1	1	1	1	1	1	1	1	1	0	15	15	0	7	5	3	downstream	MODIFIER	Pp3c21_259	Pp3c21_259	transcript	PAC:3291564	protein_codi	NA	c.*2951G>C	NA	NA	NA	NA		2951
Chr21	1575826	C	G	Chr21_15758C_G	SNP	41960.63	49	31	35	43	43	49	76	50	47	43	27	33	20	19	49	31	35	43	43	49	76	50	47	43	27	33	20	19	1	1	1	1	1	1	1	1	1	1	1	1	1	1	0	15	15	0	7	5	3	downstream	MODIFIER	Pp3c21_260	Pp3c21_260	transcript	PAC:3291597	protein_codi	NA	c.*4784G>C	NA	NA	NA	NA		4528
Chr21	1575826	C	G	Chr21_15758C_G	SNP	41960.63	49	31	35	43	43	49	76	50	47	43	27	33	20	19	49	31	35	43	43	49	76	50	47	43	27	33	20	19	1	1	1	1	1	1	1	1	1	1	1	1	1	1	0	15	15	0	7	5	3	downstream	MODIFIER	Pp3c21_260	Pp3c21_260	transcript	PAC:3291597	protein_codi	NA	c.*4784G>C	NA	NA	NA	NA		4528
Chr21	1575826	C	G	Chr21_15758C_G	SNP	41960.63	49	31	35	43	43	49	76	50	47	43	27	33	20	19	49	31	35	43	43	49	76	50	47	43	27	33	20	19	1	1	1	1	1	1	1	1	1	1	1	1	1	1	0	15	15	0	7	5	3	downstream	MODIFIER	Pp3c21_260	Pp3c21_260	transcript	PAC:3291597	protein_codi	NA	c.*4784G>C	NA	NA	NA	NA		4528
Chr21	1575826	C	G	Chr21_15758C_G	SNP	41960.63	49	31	35	43	43	49	76	50	47	43	27	33	20	19	49	31	35	43	43	49	76	50	47	43	27	33	20	19	1	1	1	1	1	1	1	1	1	1	1	1	1	1	0	15	15	0	7	5	3	downstream	MODIFIER	Pp3c21_260	Pp3c21_260	transcript	PAC:3291597	protein_codi	NA	c.*4784G>C	NA	NA	NA	NA		4528

[illegible]

Chr21	1580358	G	T	Chr21_15803G_T	SNP	15089.63	19	10	19	22	11	29	25	13	18	14	6	11	12	8
Chr21	1580358	G	T	Chr21_15803G_T	SNP	15089.63	19	10	19	22	11	29	25	13	18	14	6	11	12	8
Chr21	1580358	G	T	Chr21_15803G_T	SNP	15089.63	19	10	19	22	11	29	25	13	18	14	6	11	12	8
Chr21	1580359	G	A	Chr21_15803G_A	SNP	15089.63	19	10	19	22	11	29	25	13	18	14	6	13	12	8
Chr21	1580359	G	A	Chr21_15803G_A	SNP	15089.63	19	10	19	22	11	29	25	13	18	14	6	13	12	8
Chr21	1580359	G	A	Chr21_15803G_A	SNP	15089.63	19	10	19	22	11	29	25	13	18	14	6	13	12	8
Chr21	1580359	G	A	Chr21_15803G_A	SNP	15089.63	19	10	19	22	11	29	25	13	18	14	6	13	12	8
Chr21	1580359	G	A	Chr21_15803G_A	SNP	15089.63	19	10	19	22	11	29	25	13	18	14	6	13	12	8
Chr21	1580359	G	A	Chr21_15803G_A	SNP	15089.63	19	10	19	22	11	29	25	13	18	14	6	13	12	8
Chr21	1580359	G	A	Chr21_15803G_A	SNP	15089.63	19	10	19	22	11	29	25	13	18	14	6	13	12	8
Chr21	1580359	G	A	Chr21_15803G_A	SNP	15089.63	19	10	19	22	11	29	25	13	18	14	6	13	12	8
Chr21	1621127	C	A	Chr21_16211C_A	SNP	12045.63	12	11	12	17	16	17	19	15	20	14	7	15	6	14
Chr21	1855062	T	A	Chr21_18550T_A	SNP	12366.63	20	8	10	15	8	28	24	21	12	13	18	17	9	5
Chr21	1855062	T	A	Chr21_18550T_A	SNP	12366.63	20	8	10	15	8	28	24	21	12	13	18	17	9	5
Chr21	1855062	T	A	Chr21_18550T_A	SNP	12366.63	20	8	10	15	8	28	24	21	12	13	18	17	9	5
Chr21	1947169	G	A	Chr21_19471G_A	SNP	4375.29	0	0	0	26	14	14	26	17	0	0	0	0	0	0
Chr21	1958531	A	AT	Chr21_19585A_AT	INDEL	10556.59	19	11	10	16	14	26	22	12	11	14	8	5	4	9
Chr21	1988076	C	A	Chr21_19880C_A	SNP	17310.63	37	17	13	19	14	24	43	21	15	21	16	14	13	11
Chr21	1988076	C	A	Chr21_19880C_A	SNP	17310.63	37	17	13	19	14	24	43	21	15	21	16	14	13	11
Chr21	1988076	C	A	Chr21_19880C_A	SNP	17310.63	37	17	13	19	14	24	43	21	15	21	16	14	13	11
Chr21	1988076	C	A	Chr21_19880C_A	SNP	17310.63	37	17	13	19	14	24	43	21	15	21	16	14	13	11
Chr21	1988076	C	A	Chr21_19880C_A	SNP	17310.63	37	17	13	19	14	24	43	21	15	21	16	14	13	11
Chr21	1988076	C	A	Chr21_19880C_A	SNP	17310.63	37	17	13	19	14	24	43	21	15	21	16	14	13	11
Chr21	1988076	C	A	Chr21_19880C_A	SNP	17310.63	37	17	13	19	14	24	43	21	15	21	16	14	13	11

[illegible]

Chr21	4934309	G	T	Chr21_49343G_T	SNP	10550.63	25	6	5	12	7	14	16	11	8	9	5	10	8	7	25	6	5	12	7	14	16	11	8	9	5	10	8	7	1	1	1	1	1	1	1	1	1	1	1	1	1	1	0	15	15	0	7	5	3	downstream	MODIFIER	Pp3c21_766	Pp3c21_766	transcript	PAC:3291462	protein_codi	NA		c.*3156C>A	NA	NA	NA	NA		3000
Chr21	4934309	G	T	Chr21_49343G_T	SNP	10550.63	25	6	5	12	7	14	16	11	8	9	5	10	8	7	25	6	5	12	7	14	16	11	8	9	5	10	8	7	1	1	1	1	1	1	1	1	1	1	1	1	1	1	0	15	15	0	7	5	3	intergenic_re	MODIFIER	Pp3c21_757	Pp3c21_757	intergenic_re	Pp3c21_757	NA	NA		n.4934309G>	NA	NA	NA	NA	NA	
Chr21	4934310	C	T	Chr21_49343C_T	SNP	10955.63	25	6	5	12	7	14	16	11	8	9	5	10	8	7	25	6	5	12	7	14	16	11	8	9	5	10	8	7	1	1	1	1	1	1	1	1	1	1	1	1	1	1	0	15	15	0	7	5	3	upstream_ge	MODIFIER	Pp3c21_764	Pp3c21_764	transcript	PAC:329165	protein_codi	NA		c.-85C>T	NA	NA	NA	NA		85
Chr21	4934310	C	T	Chr21_49343C_T	SNP	10955.63	25	6	5	12	7	14	16	11	8	9	5	10	8	7	25	6	5	12	7	14	16	11	8	9	5	10	8	7	1	1	1	1	1	1	1	1	1	1	1	1	1	1	0	15	15	0	7	5	3	upstream_ge	MODIFIER	Pp3c21_764	Pp3c21_764	transcript	PAC:329165	protein_codi	NA		c.-175C>T	NA	NA	NA	NA		175
Chr21	4934310	C	T	Chr21_49343C_T	SNP	10955.63	25	6	5	12	7	14	16	11	8	9	5	10	8	7	25	6	5	12	7	14	16	11	8	9	5	10	8	7	1	1	1	1	1	1	1	1	1	1	1	1	1	1	0	15	15	0	7	5	3	downstream	MODIFIER	Pp3c21_766	Pp3c21_766	transcript	PAC:3291462	protein_codi	NA		c.*3155G>A	NA	NA	NA	NA		3051
Chr21	4934310	C	T	Chr21_49343C_T	SNP	10955.63	25	6	5	12	7	14	16	11	8	9	5	10	8	7	25	6	5	12	7	14	16	11	8	9	5	10	8	7	1	1	1	1	1	1	1	1	1	1	1	1	1	1	0	15	15	0	7	5	3	downstream	MODIFIER	Pp3c21_766	Pp3c21_766	transcript	PAC:3291462	protein_codi	NA		c.*3155G>A	NA	NA	NA	NA		2999
Chr21	4934310	C	T	Chr21_49343C_T	SNP	10955.63	25	6	5	12	7	14	16	11	8	9	5	10	8	7	25	6	5	12	7	14	16	11	8	9	5	10	8	7	1	1	1	1	1	1	1	1	1	1	1	1	1	1	0	15	15	0	7	5	3	intergenic_re	MODIFIER	Pp3c21_757	Pp3c21_757	intergenic_re	Pp3c21_757	NA	NA		n.4934310C>	NA	NA	NA	NA	NA	
Chr21	4934311	C	CAAA	Chr21_49343C_CAAA	INDEL	11351.59	24	6	6	12	7	14	16	11	8	10	5	10	8	7	24	6	6	12	7	14	16	11	8	10	5	10	8	7	1	1	1	1	1	1	1	1	1	1	1	1	1	1	0	15	15	0	7	5	3	upstream_ge	MODIFIER	Pp3c21_764	Pp3c21_764	transcript	PAC:329165	protein_codi	NA		c.-84_-83ins	NA	NA	NA	NA		83
Chr21	4934311	C	CAAA	Chr21_49343C_CAAA	INDEL	11351.59	24	6	6	12	7	14	16	11	8	10	5	10	8	7	24	6	6	12	7	14	16	11	8	10	5	10	8	7	1	1	1	1	1	1	1	1	1	1	1	1	1	1	0	15	15	0	7	5	3	upstream_ge	MODIFIER	Pp3c21_764	Pp3c21_764	transcript	PAC:329165	protein_codi	NA		c.-174_-173i	NA	NA	NA	NA		173
Chr21	4934311	C	CAAA	Chr21_49343C_CAAA	INDEL	11351.59	24	6	6	12	7	14	16	11	8	10	5	10	8	7	24	6	6	12	7	14	16	11	8	10	5	10	8	7	1	1	1	1	1	1	1	1	1	1	1	1	1	1	0	15	15	0	7	5	3	downstream	MODIFIER	Pp3c21_766	Pp3c21_766	transcript	PAC:3291462	protein_codi	NA		c.*3153_*31	NA	NA	NA	NA		3049
Chr21	4934311	C	CAAA	Chr21_49343C_CAAA	INDEL	11351.59	24	6	6	12	7	14	16	11	8	10	5	10	8	7	24	6	6	12	7	14	16	11	8	10	5	10	8	7	1	1	1	1	1	1	1	1	1	1	1	1	1	1	0	15	15	0	7	5	3	downstream	MODIFIER	Pp3c21_766	Pp3c21_766	transcript	PAC:3291462	protein_codi	NA		c.*3153_*31	NA	NA	NA	NA		2997
Chr21	4934311	C	CAAA	Chr21_49343C_CAAA	INDEL	11351.59	24	6	6	12	7	14	16	11	8	10	5	10	8	7	24	6	6	12	7	14	16	11	8	10	5	10	8	7	1	1	1	1	1	1	1	1	1	1	1	1	1	1	0	15	15	0	7	5	3	intergenic_re	MODIFIER	Pp3c21_757	Pp3c21_757	intergenic_re	Pp3c21_757	NA	NA		n.4934311_4	NA	NA	NA	NA	NA	
Chr21	4934312	C	CTAA	Chr21_49343C_CTAA	INDEL	11531.59	24	5	7	13	6	15	15	11	8	9	5	9	8	7	24	5	7	13	6	15	15	11	8	9	5	9	8	7	1	1	1	1	1	1	1	1	1	1	1	1	1	1	0	15	15	0	7	5	3	upstream_ge	MODIFIER	Pp3c21_764	Pp3c21_764	transcript	PAC:329165	protein_codi	NA		c.-83_-82ins	NA	NA	NA	NA		82
Chr21	4934312	C	CTAA	Chr21_49343C_CTAA	INDEL	11531.59	24	5	7	13	6	15	15	11	8	9	5	9	8	7	24	5	7	13	6	15	15	11	8	9	5	9	8	7	1	1	1	1	1	1	1	1	1	1	1	1	1	1	0	15	15	0	7	5	3	upstream_ge	MODIFIER	Pp3c21_764	Pp3c21_764	transcript	PAC:329165	protein_codi	NA		c.-173_-172i	NA	NA	NA	NA		172
Chr21	4934312	C	CTAA	Chr21_49343C_CTAA	INDEL	11531.59	24	5	7	13	6	15	15	11	8	9	5	9	8	7	24	5	7	13	6	15	15	11	8	9	5	9	8	7	1	1	1	1	1	1	1	1	1	1	1	1	1	1	0	15	15	0	7	5	3	downstream	MODIFIER	Pp3c21_766	Pp3c21_766	transcript	PAC:3291462	protein_codi	NA		c.*3152_*31	NA	NA	NA	NA		3048
Chr21	4934312	C	CTAA	Chr21_49343C_CTAA	INDEL	11531.59	24	5	7	13	6	15	15	11	8	9	5	9	8	7	24	5	7	13	6	15	15	11	8	9	5	9	8	7	1	1	1	1	1	1	1	1	1	1	1	1	1	1	0	15	15	0	7	5	3	downstream	MODIFIER	Pp3c21_766	Pp3c21_766	transcript	PAC:3291462	protein_codi	NA		c.*3152_*31	NA	NA	NA	NA		2996
Chr21	4934312	C	CTAA	Chr21_49343C_CTAA	INDEL	11531.59	24	5	7	13	6	15	15	11	8	9	5	9	8	7	24	5	7	13	6	15	15	11	8	9	5	9	8	7	1	1	1	1	1	1	1	1	1	1	1	1	1	1	0	15	15	0	7	5	3	intergenic_re	MODIFIER	Pp3c21_757	Pp3c21_757	intergenic_re	Pp3c21_757	NA	NA		n.4934312_4	NA	NA	NA	NA	NA	
Chr21	4934316	T	TATGTGAG	Chr21_49343T_TATGTGT	INDEL	12341.59	26	6	7	14	6	16	17	12	9	11	7	10	8	7	26	6	7	14	6	16	17	12	9	11	7	10	8	7	1	1	1	1	1	1	1	1	1	1	1	1	1	1	0	15	15	0	7	5	3	upstream_ge	MODIFIER	Pp3c21_764	Pp3c21_764	transcript	PAC:329165	protein_codi	NA		c.-79_-78ins	NA	NA	NA	NA		78
Chr21	4934316	T	TATGTGAG	Chr21_49343T_TATGTGT	INDEL	12341.59	26	6	7	14	6	16	17	12	9	11	7	10	8	7	26	6	7	14	6	16	17	12	9	11	7	10	8	7	1	1	1	1	1	1	1	1	1	1	1	1	1	1	0	15	15	0	7	5	3	upstream_ge	MODIFIER	Pp3c21_764	Pp3c21_764	transcript	PAC:329165	protein_codi	NA		c.-169_-168i	NA	NA	NA	NA		168
Chr21	4934316	T	TATGTGAG	Chr21_49343T_TATGTGT	INDEL	12341.59	26	6	7	14	6	16	17	12	9	11	7	10	8	7	26	6	7	14	6	16	17	12	9	11	7	10	8	7	1	1	1	1	1	1	1	1	1	1	1	1	1	1	0	15	15	0	7	5	3	downstream	MODIFIER	Pp3c21_766	Pp3c21_766	transcript	PAC:3291462	protein_codi	NA		c.*3148_*31	NA	NA	NA	NA		3044
Chr21	4934316	T	TATGTGAG	Chr21_49343T_TATGTGT	INDEL	12341.59	26	6	7	14	6	16	17	12	9	11	7	10	8	7	26	6	7	14	6	16	17	12	9	11	7	10	8	7	1	1	1	1	1	1	1	1	1	1	1	1	1	1	0	15	15	0	7	5	3	downstream	MODIFIER	Pp3c21_766	Pp3c21_766	transcript	PAC:3291462	protein_codi	NA		c.*3148_*31	NA	NA	NA	NA		2992
Chr21	4934316	T	TATGTGAG	Chr21_49343T_TATGTGT	INDEL	12341.59	26	6	7	14	6	16	17	12	9	11	7	10	8	7	26	6	7	14	6	16	17	12	9	11	7	10	8	7	1	1	1	1	1	1	1	1	1	1	1	1	1	1	0	15	15	0	7	5	3	intergenic_re	MODIFIER	Pp3c21_757	Pp3c21_757	intergenic_re	Pp3c21_757	NA	NA		n.4934316_4	NA	NA	NA	NA	NA	
Chr21	4934318	C	A	Chr21_49343C_A	SNP	12755.63	26	8	10	12	7	13	19	12	6	13	7	11	9	7	26	8	10	12	7	13	19	12	6	13	7	11	9	7	1	1	1	1	1	1	1	1	1	1	1	1	1	1	0	15	15	0	7	5	3	upstream_ge	MODIFIER	Pp3c21_764	Pp3c21_764	transcript	PAC:329165	protein_codi	NA		c.-77C>A	NA	NA	NA	NA		77
Chr21	4934318	C	A	Chr21_49343C_A	SNP	12755.63	26	8	10	12	7	13	19	12	6	13	7	11	9	7	26	8	10	12	7	13	19	12	6	13	7	11	9	7	1	1	1	1	1	1	1	1	1	1	1	1	1	1	0	15	15	0	7	5	3	upstream_ge	MODIFIER	Pp3c21_764	Pp3c21_764	transcript	PAC:329165	protein_codi	NA		c.-167C>A	NA	NA	NA	NA		167
Chr21	4934318	C	A	Chr21_49343C_A	SNP	12755.63	26	8	10	12	7	13	19	12	6	13	7	11	9	7	26	8	10	12	7	13	19	12	6	13	7	11	9	7	1	1	1	1	1	1	1	1	1	1	1	1	1	1	0	15	15	0	7	5	3	downstream	MODIFIER	Pp3c21_766	Pp3c21_766	transcript	PAC:3291462	protein_codi	NA		c.*3147G>T	NA	NA	NA	NA		3043
Chr21	4934318	C	A	Chr21_49343C_A	SNP	12755.63	26	8	10	12	7	13	19	12	6	13	7	11	9	7	26	8	10	12	7	13	19	12	6	13	7	11	9	7	1	1	1	1	1	1	1	1	1	1	1	1	1	1	0	15	15	0	7	5	3	downstream	MODIFIER	Pp3c21_766	Pp3c21_766	transcript	PAC:3291462	protein_codi	NA		c.*3147G>T	NA	NA	NA	NA		2991
Chr21	4934318	C	A	Chr21_49343C_A	SNP	12755.63	26	8	10	12	7	13	19	12	6	13	7	11	9	7	26	8	10	12	7	13	19	12	6	13	7	11	9	7	1	1	1	1	1	1	1	1	1	1	1	1	1	1	0	15	15	0	7	5	3	intergenic_re	MODIFIER	Pp3c21_757	Pp3c21_757	intergenic_re	Pp3c21_757	NA	NA		n.4934318C>	NA	NA	NA	NA	NA	
Chr21	4934321	A	T	Chr21_49343A_T	SNP	11540.63	25	8	9	11	7	13	21	13	6	14	9	12	7	7	25	8	9	11	7	13	21	13	6	14	9	12	7	7	1	1	1	1	1	1	1	1	1	1	1	1	1	1	0	15	15	0	7	5	3	upstream_ge	MODIFIER	Pp3c21_764	Pp3c21_764	transcript	PAC:329165	protein_codi	NA		c.-74A>T	NA	NA	NA	NA		74
Chr21	4934321	A	T	Chr21_49343A_T	SNP	11540.63	25	8	9	11	7	13	21	13	6	14	9	12	7	7	25	8	9	11	7	13	21	13	6	14	9	12	7	7	1	1	1	1	1	1	1	1	1	1	1	1	1	1	0	15	15	0	7	5	3	upstream_ge	MODIFIER	Pp3c21_764	Pp3c21_764	transcript	PAC:329165	protein_codi	NA		c.-164A>T	NA	NA	NA	NA		164
Chr21	4934321	A	T	Chr21_49343A_T	SNP	11540.63	25	8	9	11	7	13	21	13	6	14	9	12	7	7	25	8	9	11	7	13	21	13	6	14	9	12	7	7	1	1	1	1	1	1	1	1	1	1	1	1	1	1	0	15	15	0	7	5	3	downstream	MODIFIER	Pp3c21_766	Pp3c21_766	transcript	PAC:3291462	protein_codi	NA		c.*3144T>A	NA	NA	NA	NA		3040
Chr21	4934321	A	T	Chr21_49343A_T	SNP	11540.63	25	8	9	11	7	13	21	13	6	14	9	12	7	7	25	8	9	11	7	13	21	13	6	14	9	12	7	7	1	1	1	1	1	1	1	1	1	1	1	1	1	1	0	15	15	0	7	5	3	downstream	MODIFIER	Pp3c21_766	Pp3c21_766	transcript	PAC:3291462	protein_codi	NA		c.*3144T>A	NA	NA	NA	NA		2988
Chr21	4934321	A	T	Chr21_49343A_T	SNP	11540.63	25	8	9	11	7	13	21	13	6	14	9	12	7	7	25	8	9	11	7	13	21	13	6	14	9	12	7	7	1	1	1	1	1	1	1	1	1	1	1	1	1	1	0	15	15	0	7	5	3	intergenic_re	MODIFIER	Pp3c21_757	Pp3c21_757	intergenic_re	Pp3c21_757	NA	NA		n.4934321A>	NA	NA	NA	NA	NA	
Chr21	4934324	C	A	Chr21_49343C_A	SNP	11585.63	26	8	10	10	7	14	19	11	5	13	8	12	6	7	26	8	10	10	7	14	19	11	5	13	8	12	6	7	1	1	1	1	1	1	1	1	1	1	1	1	1	1	0	15	15	0	7	5	3	upstream_ge	MODIFIER	Pp3c21_764	Pp3c21_764	transcript	PAC:329165	protein_codi	NA		c.-71C>A	NA	NA	NA	NA		71
Chr21	4934324	C	A	Chr21_49343C_A	SNP	11585.63	26	8	10	10	7	14	19	11	5	13	8	12	6	7	26	8	10	10	7	14	19	11	5	13	8	12	6	7	1	1	1	1	1	1	1	1	1	1	1	1	1	1	0	15	15	0	7	5	3	upstream_ge	MODIFIER	Pp3c21_764	Pp3c21_764	transcript	PAC:329165	protein_codi	NA		c.-161C>A	NA	NA	NA	NA		161
Chr21	4934324	C	A	Chr21_49343C_A	SNP	11585.63	26	8	10	10	7	14	19	11	5	13	8	12	6	7	26	8	10	10	7	14	19	11	5	13	8	12	6	7	1	1	1	1	1	1	1	1	1	1	1	1	1	1	0	15	15	0	7	5	3	downstream	MODIFIER	Pp3c21_766	Pp3c21_766	transcript	PAC:3291462	protein_codi	NA		c.*3141G>T	NA	NA	NA	NA		3037
Chr21	4934324	C	A	Chr21_49343C_A	SNP	11585.63	26	8	10	10	7	14	19	11	5	13	8	12	6	7	26	8	10	10	7	14	19	11	5	13	8	12	6	7	1	1	1	1	1	1	1	1	1	1	1	1	1	1	0	15	15	0	7	5	3	downstream	MODIFIER	Pp3c21_766	Pp3c21_766	transcript	PAC:3291462	protein_codi	NA		c.*3141G>T	NA	NA	NA	NA		2985
Chr21	4934324	C	A	Chr21_49343C_A	SNP	11585.63	26	8	10	10	7	14	19	11	5	13	8	12	6	7	26	8	10	10	7	14	19	11	5	13	8	12	6	7	1	1	1	1	1	1	1	1	1	1	1	1	1	1	0	15	15	0	7	5	3	intergenic_re	MODIFIER	Pp3c21_757	Pp3c21_757	intergenic_re	Pp3c21_757	NA	NA		n.4934324C>	NA	NA	NA	NA	NA	
Chr21	4934325	C	CAACTT	Chr21_49343C_CAACTT	INDEL	11666.59	26	8	9	10	7	14	19	11	6	14	8	12	6	7	26	8	9	10	7	14	19	11	6	14	8	12	6	7	1	1	1	1	1	1	1	1	1	1	1	1	1	1	0	15	15	0	7	5	3	upstream_ge	MODIFIER	Pp3c21_764	Pp3c21_764	transcript	PAC:329165	protein_codi	NA		c.-70_-69ins	NA	NA	NA	NA		69
Chr21	4934325	C	CAACTT	Chr21_49343C_CAACTT	INDEL	11666.59	26	8	9	10	7	14	19	11	6	14	8	12	6	7	26	8	9	10	7	14	19	11	6	14	8	12	6	7	1	1	1	1	1	1	1	1	1	1	1	1	1	1	0	15	15	0	7	5	3	upstream_ge	MODIFIER	Pp3c21_764	Pp3c21_764	transcript	PAC:329165	protein_codi	NA		c.-160_-159i	NA	NA	NA	NA		159
Chr21	4934325	C	CAACTT	Chr21_49343C_CAACTT	INDEL	11666.59	26	8	9	10	7	14	19	11	6	14	8	12	6	7	26	8	9	10	7	14	19	11	6	14	8	12	6	7	1	1	1	1	1	1	1	1	1	1	1	1	1	1	0	15	15	0	7	5	3	downstream	MODIFIER	Pp3c21_766	Pp3c21_766	transcript	PAC:3291462	protein_codi	NA		c.*3139_*31	NA	NA	NA	NA		3035
Chr21	4934325	C	CAACTT	Chr21_49343C_CAACTT	INDEL	11666.59	26	8	9	10	7	14	19	11	6	14	8	12	6	7	26	8	9	10	7	14	19	11	6	14	8	12	6	7	1	1	1	1	1	1	1	1	1	1	1	1	1	1	0	15	15	0	7	5	3	downstream	MODIFIER	Pp3c21_766	Pp3c21_766	transcript	PAC:3291462	protein_codi	NA		c.*3139_*31	NA	NA	NA	NA		2983
Chr21	4934325	C	CAACTT	Chr21_49343C_CAACTT	INDEL	11666.59	26	8	9	10	7	14	19	11	6	14	8	12	6	7	26	8	9	10	7	14	19	11	6	14	8	12	6	7	1	1	1	1	1	1	1	1	1	1	1	1	1	1	0	15	15	0	7	5	3	intergenic_re	MODIFIER	Pp3c21_757	Pp3c21_757	intergenic_re	Pp3c21_757	NA	NA		n.4934325_4	NA	NA	NA	NA	NA	
Chr21	4934326	G	GT	Chr21_49343G_GT	INDEL	11621.59	25	9	9	10	6	15	19	10	6	15	8	11	6	5	25	9	9	10	6	15	19	10	6	15	8	11	6	5	1	1	1	1	1	1	1	1	1	1	1	1	1	1	0	15	15	0	7	5	3	upstream_ge	MODIFIER	Pp3c21_764	Pp3c21_764	transcript	PAC:329165	protein_codi	NA		c.-69_-68ins	NA	NA	NA	NA		68
Chr21	4934326	G	GT	Chr21_49343G_GT	INDEL	11621.59	25	9	9	10	6	15	19	10	6	15	8	11	6	5	25	9	9	10	6	15	19	10	6	15	8	11	6	5	1	1	1	1	1	1	1	1	1	1	1	1	1	1	0	15	15	0	7	5	3	upstream_ge	MODIFIER	Pp3c21_764	Pp3c21_764	transcript	PAC:329165	protein_codi	NA		c.-159_-158i	NA	NA	NA	NA		158
Chr21	4934326	G	GT	Chr21_49343G_GT	INDEL	11621.59	25	9	9	10	6	15	19	10	6	15	8	11	6	5	25	9	9	10	6	15	19	10	6	15	8	11	6	5	1	1	1	1	1	1	1	1	1	1	1	1	1	1	0	15	15	0	7	5	3	downstream	MODIFIER	Pp3c21_766	Pp3c21_766	transcript	PAC:3291462	protein_codi	NA		c.*3138_*31	NA	NA	NA	NA		3034
Chr21	4934326	G	GT	Chr21_49343G_GT	INDEL	11621.59	25	9	9	10	6	15	19	10	6	15	8	11	6	5	25	9	9	10	6	15	19	10	6	15	8	11	6	5	1	1	1	1	1	1	1	1	1	1	1	1	1	1	0	15	15	0	7	5	3	downstream	MODIFIER	Pp3c21_766	Pp3c21_766	transcript	PAC:3291462	protein_codi	NA		c.*3138_*31	NA	NA	NA	NA		2982
Chr21	4934326	G	GT	Chr21_49343G_GT	INDEL	11621.59	25	9	9	10	6	15	19	10	6	15	8	11	6	5	25	9	9	10	6	15	19	10	6	15	8	11	6	5	1	1	1	1	1	1	1	1	1	1	1	1	1	1	0	15	15	0	7	5	3	intergenic_re	MODIFIER	Pp3c21_757	Pp3c21_757	intergenic_re	Pp3c21_757	NA	NA		n.4934326_4	NA	NA	NA	NA	NA	
Chr21	4934328	C	G	Chr21_49343C_G	SNP	11180.63	25	9	10	11	6	16	19	10	7	15	8	12	6	5	25	9	10	11	6	16	19	10	7	15	8	12	6	5	1	1	1	1	1	1	1	1	1	1	1	1	1	1	0	15	15	0	7	5	3	upstream_ge	MODIFIER	Pp3c21_764	Pp3c21_764	transcript	PAC:329165	protein_codi	NA		c.-67C>G	NA	NA	NA	NA		67
Chr21	4934328	C	G	Chr21_49343C_G	SNP	11180.63	25	9	10	11	6	16	19	10	7	15	8	12	6	5	25	9	10	11	6	16	19	10	7	15	8	12	6	5	1	1	1	1	1	1	1	1	1	1	1	1	1	1	0	15	15	0	7	5	3	upstream_ge	MODIFIER	Pp3c21_764	Pp3c21_764	transcript	PAC:329165	protein_codi	NA		c.-157C>G	NA	NA	NA	NA		157
Chr21	4934328	C	G	Chr21_49343C_G	SNP	11180.63	25	9	10	11	6	16	19	10	7	15	8	12	6	5	25	9	10	11	6	16	19	10	7	15	8	12	6	5	1	1	1	1	1	1	1	1	1	1	1	1	1	1	0	15	15	0	7	5	3	downstream	MODIFIER	Pp3c21_766	Pp3c21_766	transcript	PAC:3291462	protein_codi	NA		c.*3137G>C	NA	NA	NA	NA		3033
Chr21	4934328	C	G	Chr21_49343C_G	SNP	11180.63	25	9	10	11	6	16	19	10	7	15	8	12	6	5	25	9	10	11	6	16	19	10	7	15	8	12	6	5	1	1	1	1	1	1	1	1	1	1	1	1	1	1	0	15	15	0	7	5	3	downstream	MODIFIER	Pp3c21_766	Pp3c21_766	transcript	PAC:3291462	protein_codi	NA		c.*3137G>C	NA	NA	NA	NA		2981
Chr21	4934328	C	G	Chr21_49343C_G	SNP	11180.63	25	9	10	11	6	16	19	10	7	15	8	12	6	5	25	9	10	11	6	16	19	10	7	15	8	12	6	5	1	1	1	1	1	1	1	1	1	1	1	1	1	1	0	15	15	0	7	5	3	intergenic_re	MODIFIER	Pp3c21_757	Pp3c21_757	intergenic_re	Pp3c21_757	NA	NA		n.4934328C>	NA	NA	NA	NA	NA	
Chr21	4934331	CGAGGACGC	C	Chr21_49343CGAGGACGC	INDEL	11148.59	25	9	10	11	7	16	20	11	8	14	8	12	6	6	25	9	10	11	7	16	20	11	8	14	8	12	6	6	1	1	1	1	1	1	1	1	1	1	1	1	1	1	0	15	15	0	7	5	3	upstream_ge	MODIFIER	Pp3c21_764	Pp3c21_764	transcript	PAC:329165	protein_codi	NA		c.-63_-56del	NA	NA	NA	NA		63
Chr21	4934331	CGAGGACGC	C	Chr21_49343CGAGGACGC	INDEL	11148.59	25	9	10	11	7	16	20	11	8	14	8	12	6	6	25	9	10	11	7	16	20	11	8	14	8	12	6	6	1	1	1	1	1	1	1	1	1	1	1	1	1	1	0	15	15	0	7	5	3	upstream_ge	MODIFIER	Pp3c21_764	Pp3c21_764	transcript	PAC:329165	protein_codi	NA		c.-153_-146c	NA	NA	NA	NA		153
Chr21	4934331	CGAGGACGC	C	Chr21_49343CGAGGACGC	INDEL	11148.59	25	9	10	11	7	16	20	11	8	14	8	12	6	6	25	9	10	11	7	16	20	11	8	14	8	12	6	6	1	1	1	1	1	1	1	1	1	1	1	1	1	1	0	15	15	0	7	5	3	downstream	MODIFIER	Pp3c21_766	Pp3c21_766	transcript	PAC:3291462	protein_codi	NA		c.*3126_*31	NA	NA	NA	NA		3029
Chr21	4934331	CGAGGACGC	C	Chr21_49343CGAGGACGC	INDEL	11148.59	25	9	10	11	7	16	20	11	8	14	8	12	6	6	25	9	10	11	7	16	20	11	8	14	8	12	6	6	1	1	1	1	1	1	1	1	1	1	1	1	1	1	0	15	15	0	7	5	3	downstream	MODIFIER	Pp3c21_766	Pp3c21_766	transcript	PAC:3291462	protein_codi	NA		c.*3126_*31	NA	NA	NA	NA		2977
Chr21	4934331	CGAGGACGC	C	Chr21_49343CGAGGACGC	INDEL	11148.59	25	9	10	11	7	16	20	11	8	14	8	12	6	6	25	9	10	11	7	16	20	11	8	14	8	12	6	6	1	1	1	1	1	1	1	1	1	1	1	1	1	1	0	15	15	0	7	5	3	intergenic_re	MODIFIER	Pp3c21_757	Pp3c21_757	intergenic_re	Pp3c21_757	NA	NA		n.4934332_4	NA	NA	NA	NA	NA	
Chr21	4939445	C	CA	Chr21_49394C_CA	INDEL	35514.59	58	28	50	59	49	87	82	54	35	42	17	19	19	15	58	28	50	59	49	87	82	54	35	42	17	19	19	15	1	1	1	1	1	1	1	1	1	1	1	1	1	1	0	15	15	0	7	5	3	upstream_ge	MODIFIER	Pp3c21_767	Pp3c21_767	transcript	PAC:3291681	protein_codi	NA		c.-472_-471i	NA	NA	NA	NA		471
Chr21	4939445	C	CA	Chr21_49394C_CA	INDEL	35514.59	58	28	50	59	49	87	82	54	35	42	17	19	19	15	58	28	50	59	49	87	82	54	35	42	17	19	19	15	1	1	1	1	1	1	1	1	1	1	1	1	1	1	0	15	15	0	7	5	3	upstream_ge	MODIFIER	Pp3c21_766	Pp3c21_766	transcript	PAC:3291462	protein_codi	NA		c.-788_-787i	NA	NA	NA	NA		492
Chr21	4939445	C	CA	Chr21_49394C_CA	INDEL	35514.59	58	28	50	59	49	87	82	54	35	42	17	19	19	15	58	28	50	59	49	87	82	54	35	42	17	19	19	15	1	1	1	1	1	1	1	1	1	1	1	1	1	1	0	15	15	0	7	5	3	upstream_ge	MODIFIER	Pp3c21_767	Pp3c21_767	transcript	PAC:3291681	protein_codi	NA		c.-538_-537i	NA	NA	NA	NA		490
Chr21	4939445	C	CA	Chr21_49394C_CA	INDEL	35514.59	58	28	50	59	49	87	82	54	35	42	17	19	19	15	58	28	50	59	49	87	82	54	35	42	17	19	19	15	1	1	1	1	1	1	1	1	1	1	1	1	1	1	0	15	15	0	7	5	3	downstream	MODIFIER	Pp3c21_764	Pp3c21_764	transcript	PAC:329165	protein_codi	NA		c.*3632_*36	NA	NA	NA	NA		3259
Chr21	4939445	C	CA	Chr21_49394C_CA	INDEL	35514.59	58	28	50	59	49	87	82	54	35	42	17	19	19	15	58	28	50	59	49	87	82	54	35	42	17	19	19	15	1	1	1	1	1	1	1	1	1	1	1	1	1	1	0	15	15	0	7	5	3	downstream	MODIFIER	Pp3c21_764	Pp3c21_764	transcript	PAC:329165	protein_codi	NA		c.*3632_*36	NA	NA	NA	NA		3268
Chr21	4939445	C	CA	Chr21_49394C_CA	INDEL	35514.59	58	28	50	59	49	87	82	54	35	42	17	19	19	15	58	28	50	59	49	87	82	54	35	42	17	19	19	15	1	1	1	1	1	1	1	1	1	1	1	1	1	1	0	15	15	0	7	5	3	intron_variar	MODIFIER	Pp3c21_766	Pp3c21_766	transcript	PAC:3291462	protein_codi		01-janv	c.-708-80_-7	NA	NA	NA	NA	NA	
Chr21	4939622	G	GA	Chr21_49396G_GA	INDEL	55007.59	84	76	68	80	57	95	110	80	92	80	64	98	40	62	84	76	69	82	57	95	110	80	92	80	64	98	40	62	1	1	0.986	0.976	1	1	1	1	1	1	1	1	1	1	0	15	15	0	7	5	3	5_prime_UTI	MODIFIER	Pp3c21_766	Pp3c21_766	transcript	PAC:3291462	protein_codi		01-févr	c.-752dupT	NA	NA	NA	NA		965
Chr21	4939622	G	GA	Chr21_49396G_GA	INDEL	55007.59	84	76	68	80	57	95	110	80	92	80	64	98	40	62	84	76	69	82	57	95	110	80	92	80	64	98	40	62	1	1	0.986	0.976	1	1	1	1	1	1	1	1	1	1	0	15	15	0	7	5	3	upstream_ge	MODIFIER	Pp3c21_767	Pp3c21_767	transcript	PAC:3291681	protein_codi	NA		c.-295_-294i	NA	NA	NA	NA		294
Chr21	4939622	G	GA	Chr21_49396G_GA	INDEL	55007.59	84	76	68	80	57	95	110	80	92	80	64	98	40	62	84	76	69	82	57	95	110	80	92	80	64	98	40	62	1	1	0.986	0.976	1	1	1	1	1	1	1	1	1	1	0	15	15	0	7	5	3	upstream_ge	MODIFIER	Pp3c21_766	Pp3c21_766	transcript	PAC:3291462	protein_codi	NA		c.-965dupT	NA	NA	NA	NA		669
Chr21	4939622	G	GA	Chr21_49396G_GA	INDEL	55007.59	84	76	68	80	57	95	110	80	92	80	64	98	40	62	84	76	69	82	57	95	110	80	92	80	64	98	40	62	1	1	0.986	0.976	1	1	1	1	1	1	1	1	1	1	0	15	15	0	7	5	3	upstream_ge	MODIFIER	Pp3c21_767	Pp3c21_767	transcript	PAC:3291681	protein_codi	NA		c.-361_-360i	NA	NA	NA	NA		313
Chr21	4939622	G	GA	Chr21_49396G_GA	INDEL	55007.59	84	76	68	80	57	95	110	80	92	80	64	98	40	62	84	76	69	82	57	95	110	80	92	80	64	98	40	62	1	1	0.986	0.976	1	1	1	1	1	1	1	1	1	1	0	15	15	0	7	5	3	downstream	MODIFIER	Pp3c21_764	Pp3c21_764	transcript	PAC:329165	protein_codi	NA		c.*3809_*38	NA	NA	NA	NA		3436
Chr21	4939622	G	GA	Chr21_49396G_GA	INDEL	55007.59	84	76	68	80	57	95	110	80	92	80	64	98	40	62	84	76	69	82	57	95	110	80	92	80	64	98	40	62	1	1	0.986	0.976	1	1	1	1	1	1	1	1	1	1	0	15	15	0	7	5	3	downstream	MODIFIER	Pp3c21_764	Pp3c21_764	transcript	PAC:329165	protein_codi	NA		c.*3809_*38	NA	NA	NA	NA		3445
Chr21	4965286	T	TC	Chr21_49652T_TC	INDEL	18540.59	22	11	21	20	25	19	29	20	23	27	28	8	9	13	22	11	21	20	25	19	29	20	23	27	28	8	9	13	1	1	1	1	1	1	1	1	1	1	1	1	1	1	0	15	15	0	7	5	3	downstream	MODIFIER	Pp3c21_772	Pp3c21_772	transcript	PAC:3291615	protein_codi	NA		c.*2108_*21	NA	NA	NA	NA		1388
Chr21	4965286	T	TC	Chr21_49652T_TC	INDEL	18540.59	22	11	21	20	25	19	29	20	23	27	28	8	9	13	22	11	21	20	25	19	29	20	23	27	28	8	9	13	1	1	1	1	1	1	1	1	1	1	1	1	1	1	0	15	15	0	7	5	3	downstream	MODIFIER	Pp3c21_772	Pp3c21_772	transcript	PAC:3291615	protein_codi	NA		c.*2108_*21	NA	NA	NA	NA		1388
Chr21	4965286	T	TC	Chr21_49652T_TC	INDEL	18540.59	22	11	21	20	25	19	29	20	23	27	28	8	9	13	22	11	21	20	25	19	29	20	23	27	28	8	9	13	1	1	1	1	1	1	1	1	1	1	1	1	1	1	0	15	15	0	7	5	3	downstream	MODIFIER	Pp3c21_772	Pp3c21_772	transcript	PAC:3291616	protein_codi	NA		c.*2108_*21	NA	NA	NA	NA		1388
Chr21	4965286	T	TC	Chr21_49652T_TC	INDEL	18540.59	22	11	21	20	25	19	29	20	23	27	28	8	9	13	22	11	21	20	25	19	29	20	23	27	28	8	9	13	1	1	1	1	1	1	1	1	1	1	1	1	1	1	0	15	15	0	7	5	3	downstream	MODIFIER	Pp3c21_772	Pp3c21_772	transcript	PAC:3291616	protein_codi	NA		c.*2108_*21	NA	NA	NA	NA		1412
Chr21	4965286	T	TC	Chr21_49652T_TC	INDEL	18540.59	22	11	21	20	25	19	29	20	23	27	28	8	9	13	22	11	21	20	25	19	29	20	23	27	28	8	9	13	1	1	1	1	1	1	1	1	1	1	1	1	1	1	0	15	15	0	7	5	3	intergenic_re	MODIFIER	Pp3c21_769	Pp3c21_769	intergenic_re	Pp3c21_769	NA	NA		n.4965286_4	NA	NA	NA	NA	NA	
Chr21	5031075	G	GA	Chr21_50310G_GA	INDEL	16097.59	30	22	18	20	20	22	32	24	27	38	20	13	9	8	31	22	18	20	20	22	32	24	27	38	20	13	9	8	0.968	1	1	1	1	1	1	1	1	1	1	1	1	1	0	15	15	0	7	5	3	upstream_ge	MODIFIER	Pp3c21_781	Pp3c21_781	transcript	PAC:3291503	protein_codi	NA		c.-3151_-315	NA	NA	NA	NA		3151
Chr21	5031075	G	GA	Chr21_50310G_GA	INDEL	16097.59	30	22	18	20	20	22	32	24	27	38	20	13	9	8	31	22	18	20	20	22	32	24	27	38	20	13	9	8	0.968	1	1	1	1	1	1	1	1	1	1	1	1	1	0	15	15	0	7	5	3	intergenic_re	MODIFIER	Pp3c21_781	Pp3c21_781	intergenic_re	Pp3c21_781	NA	NA		n.5031075_5	NA	NA	NA	NA	NA	
Chr21	5439131	T	TTA	Chr21_54391T_TTA	INDEL	1799.24	2	0	1	8	12	13	10	8	0	0	0	0	0	0	13	8	7	9	13	13	10	8	7	14	9	10	4	7	0.154	0	0	0.889	0.923	1	1	1	0	0	0	0	0	0	0	5	5	1	0	5	0	intron_variar	MODIFIER	Pp3c21_845	Pp3c21_845	transcript	PAC:3291532	protein_codi		janv-46	c.84-52_84-5	NA	NA	NA	NA	NA	
Chr21	5439131	T	TTA	Chr21_54391T_TTA	INDEL	1799.24	2	0	1	8	12	13	10	8	0	0	0	0	0	0	13	8	7	9	13	13	10	8	7	14	9	10	4	7	0.154	0	0	0.889	0.923	1	1	1	0	0	0	0	0	0	0	5	5	1	0	5	0	intron_variar	MODIFIER	Pp3c21_845	Pp3c21_845	transcript	PAC:3291532	protein_codi		janv-46	c.84-52_84-5	NA	NA	NA	NA	NA	
Chr21	5532097	A	G	Chr21_55320A_G	SNP	17704.63	28	14	20	28	15	19	27	20	22	22	11	7	10	10	28	14	20	28	15	19	27	20	22	22	11	7	10	10	1	1	1	1	1	1	1	1	1	1	1	1	1	1	0	15	15	0	7	5	3	intergenic_re	MODIFIER	Pp3c21_855	Pp3c21_855	intergenic_re	Pp3c21_855	NA	NA		n.5532097A>	NA	NA	NA	NA	NA	
Chr21	5532103	C	G	Chr21_55321C_G	SNP	18007.63	26	13	22	29	14	18	28	19	25	23	11	8	10	10	26	13	22	29	14	18	28	19	25	23	11	8	10	10	1	1	1	1	1	1	1	1	1	1	1	1	1	1	0	15	15	0	7	5	3	intergenic_re	MODIFIER	Pp3c21_855	Pp3c21_855	intergenic_re	Pp3c21_855	NA	NA		n.5532103C>	NA	NA	NA	NA	NA	
Chr21	5550534	G	C	Chr21_55505G_C	SNP	11872.29	25	10	26	0	0	1	0	0	22	15	29	17	11	5	25	10	26	26	23	26	26	20	22	15	29	17	11	5	1	1	1	0	0	0	0	0	1	1	1	1	1	1	0	10	10	0	7	0	3	intergenic_re	MODIFIER	Pp3c21_855	Pp3c21_855	intergenic_re	Pp3c21_855	NA	NA		n.5550534G>	NA	NA	NA	NA	NA	
Chr21	5651268	A	AT	Chr21_56512A_AT	INDEL	16813.59	31	8	8	29	13	18	27	28	22	15	20	11	6	11	31	8	8	29	13	18	27	28	22	15	20	11	6	11	1	1	1	1	1	1	1	1	1	1	1	1	1	1	0	15	15	0	7	5	3	intergenic_re	MODIFIER	Pp3c21_878	Pp3c21_878	intergenic_re	Pp3c21_878	NA	NA		n.5651268_5	NA	NA	NA	NA	NA	
Chr21	5667768	GC	G	Chr21_56677GC_G	INDEL	15661.59	26	17	17	26	16	24	26	21	20	24	19	13	7	5	26	17	17	26	16	24	26	21	20	24	19	13	7	5	1	1	1	1	1	1	1	1	1	1	1	1	1	1	0	15	15	0	7	5	3	downstream	MODIFIER	Pp3c21_880	Pp3c21_880	transcript	PAC:3291662	protein_codi	NA		c.*1984delC	NA	NA	NA	NA		1658
Chr21	5667768	GC	G	Chr21_56677GC_G	INDEL	15661.59	26	17	17	26	16	24	26	21	20	24	19	13	7	5	26	17	17	26	16	24	26	21	20	24	19	13	7	5	1	1	1	1	1	1	1	1	1	1	1	1	1	1	0	15	15	0	7	5	3	downstream	MODIFIER	Pp3c21_880	Pp3c21_880	transcript	PAC:3291662	protein_codi	NA		c.*1984delC	NA	NA	NA	NA		1513
Chr21	5667768	GC	G	Chr21_56677GC_G	INDEL	15661.59	26	17	17	26	16	24	26	21	20	24	19	13	7	5	26	17	17	26	16	24	26	21	20	24	19	13	7	5	1	1	1	1	1	1	1	1	1	1	1	1	1	1	0	15	15	0	7	5	3	intergenic_re	MODIFIER	Pp3c21_880	Pp3c21_880	intergenic_re	Pp3c21_880	NA	NA		n.5667769de	NA	NA	NA	NA	NA	
Chr21	5667857	A	AT	Chr21_56678A_AT	INDEL	16555.59	23	17	13	29	20	25	37	13	32	24	19	16	12	6	23	17	13	29	20	25	37	13	32	24	19	16	12	6	1	1	1	1	1	1	1	1	1	1	1	1	1	1	0	15	15	0	7	5	3	downstream	MODIFIER	Pp3c21_880	Pp3c21_880	transcript	PAC:3291662	protein_codi	NA		c.*2072_*20	NA	NA	NA	NA		1747
Chr21	5667857	A	AT	Chr21_56678A_AT	INDEL	16555.59	23	17	13	29	20	25	37	13	32	24	19	16	12	6	23	17	13	29	20	25	37	13	32	24	19	16	12	6	1	1	1	1	1	1	1	1	1	1	1	1	1	1	0	15	15	0	7	5	3	downstream	MODIFIER	Pp3c21_880	Pp3c21_880	transcript	PAC:3291662	protein_codi	NA		c.*2072_*20	NA	NA	NA	NA		1602
Chr21	5667857	A	AT	Chr21_56678A_AT	INDEL	16555.59	23	17	13	29	20	25	37	13	32	24	19	16	12	6	23	17	13	29	20	25	37	13	32	24	19	16	12	6	1	1	1	1	1	1	1	1	1	1	1	1	1	1	0	15	15	0	7	5	3	intergenic_re	MODIFIER	Pp3c21_880	Pp3c21_880	intergenic_re	Pp3c21_880	NA	NA		n.5667857_5	NA	NA	NA	NA	NA	
Chr21	5668017	G	C	Chr21_56680G_C	SNP	17649.63	26	20	14	29	9	32	21	25	23	31	17	14	19	5	26	20	14	29	9	32	21	25	23	31	17	14	19	5	1	1	1	1	1	1	1	1	1	1	1	1	1	1	0	15	15	0	7	5	3	downstream	MODIFIER	Pp3c21_880	Pp3c21_880	transcript	PAC:3291662	protein_codi	NA		c.*2232G>C	NA	NA	NA	NA		1906
Chr21	5668017	G	C	Chr21_56680G_C	SNP	17649.63	26	20	14	29	9	32	21	25	23	31	17	14	19	5	26	20	14	29	9	32	21	25	23	31	17	14	19	5	1	1	1	1	1	1	1	1	1	1	1	1	1	1	0	15	15	0	7	5	3	downstream	MODIFIER	Pp3c21_880	Pp3c21_880	transcript	PAC:3291662	protein_codi	NA		c.*2232G>C	NA	NA	NA	NA		1761
Chr21	5668017	G	C	Chr21_56680G_C	SNP	17649.63	26	20	14	29	9	32	21	25	23	31	17	14	19	5	26	20	14	29	9	32	21	25	23	31	17	14	19	5	1	1	1	1	1	1	1	1	1	1	1	1	1	1	0	15	15	0	7	5	3	intergenic_re	MODIFIER	Pp3c21_880	Pp3c21_880	intergenic_re	Pp3c21_880	NA	NA		n.5668017G>	NA	NA	NA	NA	NA	
Chr21	5702344	A	AT	Chr21_57023A_AT	INDEL	16664.59	34	12	19	32	17	25	24	26	26	29	12	15	14	10	34	12	19	32	17	25	24	26	26	29	12	15	14	10	1	1	1	1	1	1	1	1	1	1	1	1	1	1	0	15	15	0	7	5	3	intergenic_re	MODIFIER	Pp3c21_881	Pp3c21_881	intergenic_re	Pp3c21_881	NA	NA		n.5702344_5	NA	NA	NA	NA	NA	
Chr21	5748892	C	T	Chr21_57488C_T	SNP	5251.29	0	0	0	30	14	19	29	16	0	0	0	0	0	0	29	8	8	30	14	19	29	16	16	29	25	22	10	13	0	0	0	1	1	1	1	1	0	0	0	0	0	0	0	5	5	0	0	0	0	intergenic_re	MODIFIER	Pp3c21_881	Pp3c21_881	intergenic_re	Pp3c21_881	NA	NA		n.5748892C>	NA	NA	NA	NA	NA	
Chr21	5886117	C	CT	Chr21_58861C_CT	INDEL	17059.59	27	21	10	33	21	28	29	24	18	20	13	17	13	12	27	21	10	33	21	28	29	24	18	20	13	17	13	12	1	1	1	1	1	1	1	1	1	1	1	1	1	1	0	15	15	0	7	5	3	downstream	MODIFIER	Pp3c21_896	Pp3c21_896	transcript	PAC:3291471	protein_codi	NA		c.*3383_*33	NA	NA	NA	NA		2482
Chr21	5886117	C	CT	Chr21_58861C_CT	INDEL	17059.59	27	21	10	33	21	28	29	24	18	20	13	17	13	12	27	21	10	33	21	28	29	24	18	20	13	17	13	12	1	1	1	1	1	1	1	1	1	1	1	1	1	1	0	15	15	0	7	5	3	downstream	MODIFIER	Pp3c21_897	Pp3c21_897	transcript	PAC:3291605	protein_codi	NA		c.*1343_*13	NA	NA	NA	NA		1344
Chr21	5886117	C	CT	Chr21_58861C_CT	INDEL	17059.59	27	21	10	33	21	28	29	24	18	20	13	17	13	12	27	21	10	33	21	28	29	24	18	20	13	17	13	12	1	1	1	1	1	1	1	1	1	1	1	1	1	1	0	15	15	0	7	5	3	downstream	MODIFIER	Pp3c21_896	Pp3c21_896	transcript	PAC:3291471	protein_codi	NA		c.*3383_*33	NA	NA	NA	NA		2801
Chr21	5886117	C	CT	Chr21_58861C_CT	INDEL	17059.59	27	21	10	33	21	28	29	24	18	20	13	17	13	12	27	21	10	33	21	28	29	24	18	20	13	17	13	12	1	1	1	1	1	1	1	1	1	1	1	1	1	1	0	15	15	0	7	5	3	intergenic_re	MODIFIER	Pp3c21_897	Pp3c21_897	intergenic_re	Pp3c21_897	NA	NA		n.5886117_5	NA	NA	NA	NA	NA	
Chr21	5922753	TA	T	Chr21_59227TA_T	INDEL	13991.59	31	13	12	24	19	29	25	23	21	22	20	20	14	12	31	13	12	24	19	29	25	23	21	22	20	20	14	13	1	1	1	1	1	1	1	1	1	1	1	1	1	0.923	0	15	15	0	7	5	3	intergenic_re	MODIFIER	Pp3c21_898	Pp3c21_898	intergenic_re	Pp3c21_898	NA	NA		n.5922754de	NA	NA	NA	NA	NA	
Chr21	5925542	G	A	Chr21_59255G_A	SNP	18594.63	31	20	16	23	22	28	20	25	26	18	10	20	9	14	31	20	16	23	22	28	20	25	26	18	10	20	9	14	1	1	1	1	1	1	1	1	1	1	1	1	1	1	0	15	15	0	7	5	3	intergenic_re	MODIFIER	Pp3c21_898	Pp3c21_898	intergenic_re	Pp3c21_898	NA	NA		n.5925542G>	NA	NA	NA	NA	NA	
Chr21	5925571	G	A	Chr21_59255G_A	SNP	18717.63	34	19	16	24	24	27	17	30	22	20	12	21	12	14	34	19	16	24	24	27	17	30	22	20	12	21	12	14	1	1	1	1	1	1	1	1	1	1	1	1	1	1	0	15	15	0	7	5	3	intergenic_re	MODIFIER	Pp3c21_898	Pp3c21_898	intergenic_re	Pp3c21_898	NA	NA		n.5925571G>	NA	NA	NA	NA	NA	
Chr21	5925617	A	G	Chr21_59256A_G	SNP	19673.63	36	18	15	26	26	28	25	25	18	23	15	23	11	13	36	18	15	26	26	28	25	25	18	23	15	23	11	13	1	1	1	1	1	1	1	1	1	1	1	1	1	1	0	15	15	0	7	5	3	intergenic_re	MODIFIER	Pp3c21_898	Pp3c21_898	intergenic_re	Pp3c21_898	NA	NA		n.5925617A>	NA	NA	NA	NA	NA	
Chr21	5928690	T	TA	Chr21_59286T_TA	INDEL	13789.59	34	9	16	22	19	23	20	24	18	19	21	20	8	7	34	9	16	22	19	23	20	24	18	19	21	20	8	7	1	1	1	1	1	1	1	1	1	1	1	1	1	1	0	15	15	0	7	5	3	intergenic_re	MODIFIER	Pp3c21_898	Pp3c21_898	intergenic_re	Pp3c21_898	NA	NA		n.5928690_5	NA	NA	NA	NA	NA	
Chr21	5991041	G	T	Chr21_59910G_T	SNP	15708.63	15	9	11	28	17	32	13	13	20	16	21	17	7	9	15	9	11	28	17	32	13	13	20	16	21	17	7	9	1	1	1	1	1	1	1	1	1	1	1	1	1	1	0	15	15	0	7	5	3	intergenic_re	MODIFIER	Pp3c21_908	Pp3c21_908	intergenic_re	Pp3c21_908	NA	NA		n.5991041G>	NA	NA	NA	NA	NA	
Chr21	5991047	G	T	Chr21_59910G_T	SNP	16029.63	15	9	12	29	18	33	13	13	22	16	21	18	9	9	15	9	12	29	18	33	13	13	22	16	21	18	9	9	1	1	1	1	1	1	1	1	1	1	1	1	1	1	0	15	15	0	7	5	3	intergenic_re	MODIFIER	Pp3c21_908	Pp3c21_908	intergenic_re	Pp3c21_908	NA	NA		n.5991047G>	NA	NA	NA	NA	NA	
Chr21	6045354	C	CAT	Chr21_60453C_CAT	INDEL	74.37	0	0	0	0	1	0	1	0	6	0	0	0	0	0	18	16	14	12	8	20	25	15	19	13	7	4	6	11	0	0	0	0	0	0	0	0	0	0	0.857	0	0	0	0	1	1	0	1	0	0	intergenic_re	MODIFIER	Pp3c21_915	Pp3c21_915	intergenic_re	Pp3c21_915	NA	NA		n.6045354_6	NA	NA	NA	NA	NA	
Chr21	6403252	AAT	AATAT	Chr21_64032AAT_AATAT	INDEL	4048.5	1	0	0	14	18	18	30	16	0	0	0	0	0	0	14	8	14	14	18	19	32	17	15	11	14	7	8	6	0	0	0	1	1	0.947	0.938	0.941	0	0	0	0	0	0	0	5	5	0	0	5	0	upstream_ge	MODIFIER	Pp3c21_975	Pp3c21_975	transcript	PAC:3291675	protein_codi	NA		c.-3137_-313	NA	NA	NA	NA		2945
Chr21	6403252	AAT	AATAT	Chr21_64032AAT_AATAT	INDEL	4048.5	1	0	0	14	18	18	30	16	0	0	0	0	0	0	14	8	14	14	18	19	32	17	15	11	14	7	8	6	0	0	0	1	1	0.947	0.938	0.941	0	0	0	0	0	0	0	5	5	0	0	5	0	upstream_ge	MODIFIER	Pp3c21_977	Pp3c21_977	transcript	PAC:3291547	protein_codi	NA		c.-2752_-275	NA	NA	NA	NA		1953
Chr21	6403252	AAT	AATAT	Chr21_64032AAT_AATAT	INDEL	4048.5	1	0	0	14	18	18	30	16	0	0	0	0	0	0	14	8	14	14	18	19	32	17	15	11	14	7	8	6	0	0	0	1	1	0.947	0.938	0.941	0	0	0	0	0	0	0	5	5	0	0	5	0	upstream_ge	MODIFIER	Pp3c21_975	Pp3c21_975	transcript	PAC:3291675	protein_codi	NA		c.-3137_-313	NA	NA	NA	NA		2716
Chr21	6403252	AAT	AATAT	Chr21_64032AAT_AATAT	INDEL	4048.5	1	0	0	14	18	18	30	16	0	0	0	0	0	0	14	8	14	14	18	19	32	17	15	11	14	7	8	6	0	0	0	1	1	0.947	0.938	0.941	0	0	0	0	0	0	0	5	5	0	0	5	0	upstream_ge	MODIFIER	Pp3c21_977	Pp3c21_977	transcript	PAC:3291548	protein_codi	NA		c.-2752_-275	NA	NA	NA	NA		1964
Chr21	6403252	AAT	AATAT	Chr21_64032AAT_AATAT	INDEL	4048.5	1	0	0	14	18	18	30	16	0	0	0	0	0	0	14	8	14	14	18	19	32	17	15	11	14	7	8	6	0	0	0	1	1	0.947	0.938	0.941	0	0	0	0	0	0	0	5	5	0	0	5	0	upstream_ge	MODIFIER	Pp3c21_977	Pp3c21_977	transcript	PAC:3291548	protein_codi	NA		c.-2752_-275	NA	NA	NA	NA		1964
Chr21	6403252	AAT	AATAT	Chr21_64032AAT_AATAT	INDEL	4048.5	1	0	0	14	18	18	30	16	0	0	0	0	0	0	14	8	14	14	18	19	32	17	15	11	14	7	8	6	0	0	0	1	1	0.947	0.938	0.941	0	0	0	0	0	0	0	5	5	0	0	5	0	upstream_ge	MODIFIER	Pp3c21_977	Pp3c21_977	transcript	PAC:3291548	protein_codi	NA		c.-2752_-275	NA	NA	NA	NA		1964
Chr21	6403252	AAT	AATAT	Chr21_64032AAT_AATAT	INDEL	4048.5	1	0	0	14	18	18	30	16	0	0	0	0	0	0	14	8	14	14	18	19	32	17	15	11	14	7	8	6	0	0	0	1	1	0.947	0.938	0.941	0	0	0	0	0	0	0	5	5	0	0	5	0	upstream_ge	MODIFIER	Pp3c21_977	Pp3c21_977	transcript	PAC:3291547	protein_codi	NA		c.-2752_-275	NA	NA	NA	NA		1964
Chr21	6403252	AAT	AATAT	Chr21_64032AAT_AATAT	INDEL	4048.5	1	0	0	14	18	18	30	16	0	0	0	0	0	0	14	8	14	14	18	19	32	17	15	11	14	7	8	6	0	0	0	1	1	0.947	0.938	0.941	0	0	0	0	0	0	0	5	5	0	0	5	0	upstream_ge	MODIFIER	Pp3c21_977	Pp3c21_977	transcript	PAC:3291548	protein_codi	NA		c.-2752_-275	NA	NA	NA	NA		2391
Chr21	6403252	AAT	AATAT	Chr21_64032AAT_AATAT	INDEL	4048.5	1	0	0	14	18	18	30	16	0	0	0	0	0	0	14	8	14	14	18	19	32	17	15	11	14	7	8	6	0	0	0	1	1	0.947	0.938	0.941	0	0	0	0	0	0	0	5	5	0	0	5	0	downstream	MODIFIER	Pp3c21_976	Pp3c21_976	transcript	PAC:329147	protein_codi	NA		c.*1044_*10	NA	NA	NA	NA		485
Chr21	6403252	AAT	AATAT	Chr21_64032AAT_AATAT	INDEL	4048.5	1	0	0	14	18	18	30	16	0	0	0	0	0	0	14	8	14	14	18	19	32	17	15	11	14	7	8	6	0	0	0	1	1	0.947	0.938	0.941	0	0	0	0	0	0	0	5	5	0	0	5	0	downstream	MODIFIER	Pp3c21_976	Pp3c21_976	transcript	PAC:329147	protein_codi	NA		c.*1172_*11	NA	NA	NA	NA		485
Chr21	6403252	AAT	AATAT	Chr21_64032AAT_AATAT	INDEL	4048.5	1	0	0	14	18	18	30	16	0	0	0	0	0	0	14	8	14	14	18	19	32	17	15	11	14	7	8	6	0	0	0	1	1	0.947	0.938	0.941	0	0	0	0	0	0	0	5	5	0	0	5	0	downstream	MODIFIER	Pp3c21_976	Pp3c21_976	transcript	PAC:329147	protein_codi	NA		c.*1172_*11	NA	NA	NA	NA		457
Chr21	6403252	AAT	AATAT	Chr21_64032AAT_AATAT	INDEL	4048.5	1	0	0	14	18	18	30	16	0	0	0	0	0	0	14	8	14	14	18	19	32	17	15	11	14	7	8	6	0	0	0	1	1	0.947	0.938	0.941	0	0	0	0	0	0	0	5	5	0	0	5	0	downstream	MODIFIER	Pp3c21_976	Pp3c21_976	transcript	PAC:329147	protein_codi	NA		c.*1044_*10	NA	NA	NA	NA		442
Chr21	6403252	AAT	AATAT	Chr21_64032AAT_AATAT	INDEL	4048.5	1	0	0	14	18	18	30	16	0	0	0	0	0	0	14	8	14	14	18	19	32	17	15	11	14	7	8	6	0	0	0	1	1	0.947	0.938	0.941	0	0	0	0	0	0	0	5	5	0	0	5	0	downstream	MODIFIER	Pp3c21_976	Pp3c21_976	transcript	PAC:329147	protein_codi	NA		c.*1172_*11	NA	NA	NA	NA		442
Chr21	6403252	AAT	AATAT	Chr21_64032AAT_AATAT	INDEL	4048.5	1	0	0	14	18	18	30	16	0	0	0	0	0	0	14	8	14	14	18	19	32	17	15	11	14	7	8	6	0	0	0	1	1	0.947	0.938	0.941	0	0	0	0	0	0	0	5	5	0	0	5	0	intergenic_re	MODIFIER	Pp3c21_976	Pp3c21_976	intergenic_re	Pp3c21_976	NA	NA		n.6403254_6	NA	NA	NA	NA	NA	
Chr21	6488915	C	T	Chr21_64889C_T	SNP	9263.63	11	13	14	15	8	19	14	16	23	19	12	16	9	4	11	13	14	15	8	19	14	16	23	19	12	16	9	5	1	1	1	1	1	1	1	1	1	1	1	1	1	0.8	0	15	15	0	7	5	3	intergenic_re	MODIFIER	Pp3c21_100	Pp3c21_100	intergenic_re	Pp3c21_100	NA	NA		n.6488915C>	NA	NA	NA	NA	NA	
Chr21	6555078	T	TTA	Chr21_65550T_TTA	INDEL	11558.59	19	18	12	18	21	16	23	20	9	22	17	15	6	7	19	19	13	18	21	17	23	20	9	22	17	15	6	7	1	0.947	0.923	1	1	0.941	1	1	1	1	1	1	1	1	0	15	15	0	7	5	3	upstream_ge	MODIFIER	Pp3c21_101	Pp3c21_101	transcript	PAC:329148	protein_codi	NA		c.-3936_-393	NA	NA	NA	NA		3936
Chr21	6555078	T	TTA	Chr21_65550T_TTA	INDEL	11558.59	19	18	12	18	21	16	23	20	9	22	17	15	6	7	19	19	13	18	21	17	23	20	9	22	17	15	6	7	1	0.947	0.923	1	1	0.941	1	1	1	1	1	1	1	1	0	15	15	0	7	5	3	upstream_ge	MODIFIER	Pp3c21_101	Pp3c21_101	transcript	PAC:329148	protein_codi	NA		c.-3936_-393	NA	NA	NA	NA		3182
Chr21	6555078	T	TTA	Chr21_65550T_TTA	INDEL	11558.59	19	18	12	18	21	16	23	20	9	22	17	15	6	7	19	19	13	18	21	17	23	20	9	22	17	15	6	7	1	0.947	0.923	1	1	0.941	1	1	1	1	1	1	1	1	0	15	15	0	7	5	3	upstream_ge	MODIFIER	Pp3c21_101	Pp3c21_101	transcript	PAC:329148	protein_codi	NA		c.-2394_-239	NA	NA	NA	NA		1439
Chr21	6555078	T	TTA	Chr21_65550T_TTA	INDEL	11558.59	19	18	12	18	21	16	23	20	9	22	17	15	6	7	19	19	13	18	21	17	23	20	9	22	17	15	6	7	1	0.947	0.923	1	1	0.941	1	1	1	1	1	1	1	1	0	15	15	0	7	5	3	downstream	MODIFIER	Pp3c21_1011	Pp3c21_1011	transcript	PAC:3291582	protein_codi	NA		c.*1261_*12	NA	NA	NA	NA		1262
Chr21	6555078	T	TTA	Chr21_65550T_TTA	INDEL	11558.59	19	18	12	18	21	16	23	20	9	22	17	15	6	7	19	19	13	18	21	17	23	20	9	22	17	15	6	7	1	0.947	0.923	1	1	0.941	1	1	1	1	1	1	1	1	0	15	15	0	7	5	3	intergenic_re	MODIFIER	Pp3c21_1011	Pp3c21_1011	intergenic_re	Pp3c21_1011	NA	NA		n.6555078_6	NA	NA	NA	NA	NA	
Chr21	6566592	A	T	Chr21_65665A_T	SNP	15693.63	20	10	12	19	26	26	28	20	14	19	24	19	6	10	20	10	12	19	26	26	28	20	14	19	25	19	6	10	1	1	1	1	1	1	1	1	1	1	0.96	1	1	1	0	15	15	0	7	5	3	downstream	MODIFIER	Pp3c21_101	Pp3c21_101	transcript	PAC:3291452	protein_codi	NA		c.*1755A>T	NA	NA	NA	NA		1755
Chr21	6566592	A	T	Chr21_65665A_T	SNP	15693.63	20	10	12	19	26	26	28	20	14	19	24	19	6	10	20	10	12	19	26	26	28	20	14	19	25	19	6	10	1	1	1	1	1	1	1	1	1	1	0.96	1	1	1	0	15	15	0	7	5	3	intergenic_re	MODIFIER	Pp3c21_101	Pp3c21_101	intergenic_re	Pp3c21_101	NA	NA		n.6566592A>	NA	NA	NA	NA	NA	
Chr21	6578076	TCG	T	Chr21_65780TCG_T	INDEL	10901.59	15	5	8	12	9	11	11	10	13	14	11	8	8	4	15	5	8	12	9	11	11	10	13	14	11	8	8	4	1	1	1	1	1	1	1	1	1	1	1	1	1	1	0	15	15	0	7	5	3	intergenic_re	MODIFIER	Pp3c21_101	Pp3c21_101	intergenic_re	Pp3c21_101	NA	NA		n.6578077_6	NA	NA	NA	NA	NA	
Chr21	6662887	TG	T	Chr21_66628TG_T	INDEL	15780.59	19	8	10	22	16	23	25	20	23	23	18	14	5	5	19	8	10	22	16	23	25	20	23	23	18	14	5	5	1	1	1	1	1	1	1	1	1	1	1	1	1	1	0	15	15	0	7	5	3	upstream_ge	MODIFIER	Pp3c21_103	Pp3c21_103	transcript	PAC:3291667	protein_codi	NA		c.-1845delC	NA	NA	NA	NA		1845
Chr21	6662887	TG	T	Chr21_66628TG_T	INDEL	15780.59	19	8	10	22	16	23	25	20	23	23	18	14	5	5	19	8	10	22	16	23	25	20	23	23	18	14	5	5	1	1	1	1	1	1	1	1	1	1	1	1	1	1	0	15	15	0	7	5	3	upstream_ge	MODIFIER	Pp3c21_103	Pp3c21_103	transcript	PAC:3291667	protein_codi	NA		c.-1845delC	NA	NA	NA	NA		1845
Chr21	6662887	TG	T	Chr21_66628TG_T	INDEL	15780.59	19	8	10	22	16	23	25	20	23	23	18	14	5	5	19	8	10	22	16	23	25	20	23	23	18	14	5	5	1	1	1	1	1	1	1	1	1	1	1	1	1	1	0	15	15	0	7	5	3	intergenic_re	MODIFIER	Pp3c21_103	Pp3c21_103	intergenic_re	Pp3c21_103	NA	NA		n.6662888de	NA	NA	NA	NA	NA	
Chr21	6662893	C	A	Chr21_66628C_A	SNP	15936.63	20	8	10	22	16	23	26	20	23	20	17	15	5	5	20	8	10	22	16	23	26	20	23	20	17	15	5	5	1	1	1	1	1	1	1	1	1	1	1	1	1	1	0	15	15	0	7	5	3	upstream_ge	MODIFIER	Pp3c21_103	Pp3c21_103	transcript	PAC:3291667	protein_codi	NA		c.-1850G>T	NA	NA	NA	NA		1850
Chr21	6662893	C	A	Chr21_66628C_A	SNP	15936.63	20	8	10	22	16	23	26	20	23	20	17	15	5	5	20	8	10	22	16	23	26	20	23	20	17	15	5	5	1	1	1	1	1	1	1	1	1	1	1	1	1	1	0	15	15	0	7	5	3	upstream_ge	MODIFIER	Pp3c21_103	Pp3c21_103	transcript	PAC:3291667	protein_codi	NA		c.-1850G>T	NA	NA	NA	NA		1850
Chr21	6662893	C	A	Chr21_66628C_A	SNP	15936.63	20	8	10	22	16	23	26	20	23	20	17	15	5	5	20	8	10	22	16	23	26	20	23	20	17	15	5	5	1	1	1	1	1	1	1	1	1	1	1	1	1	1	0	15	15	0	7	5	3	intergenic_re	MODIFIER	Pp3c21_103	Pp3c21_103	intergenic_re	Pp3c21_103	NA	NA		n.6662893C>	NA	NA	NA	NA	NA	
Chr21	6762677	A	AT	Chr21_67626A_AT	INDEL	9925.59	14	11	6	20	18	12	20	14	12	13	18	13	7	5	14	11	6	20	18	12	20	14	13	16	18	13	7	5	1	1	1	1	1	1	1	1	0.923	0.812	1	1	1	1	0	15	15	0	7	5	3	upstream_ge	MODIFIER	Pp3c21_105	Pp3c21_105	transcript	PAC:3291614	protein_codi	NA		c.-2444_-244	NA	NA	NA	NA		508
Chr21	6762677	A	AT	Chr21_67626A_AT	INDEL	9925.59	14	11	6	20	18	12	20	14	12	13	18	13	7	5	14	11	6	20	18	12	20	14	13	16	18	13	7	5	1	1	1	1	1	1	1	1	0.923	0.812	1	1	1	1	0	15	15	0	7	5	3	upstream_ge	MODIFIER	Pp3c21_105	Pp3c21_105	transcript	PAC:3291615	protein_codi	NA		c.-2444_-244	NA	NA	NA	NA		508
Chr21	6762677	A	AT	Chr21_67626A_AT	INDEL	9925.59	14	11	6	20	18	12	20	14	12	13	18	13	7	5	14	11	6	20	18	12	20	14	13	16	18	13	7	5	1	1	1	1	1	1	1	1	0.923	0.812	1	1	1	1	0	15	15	0	7	5	3	upstream_ge	MODIFIER	Pp3c21_105	Pp3c21_105	transcript	PAC:3291614	protein_codi	NA		c.-2444_-244	NA	NA	NA	NA		1415
Chr21	6762677	A	AT	Chr21_67626A_AT	INDEL	9925.59	14	11	6	20	18	12	20	14	12	13	18	13	7	5	14	11	6	20	18	12	20	14	13	16	18	13	7	5	1	1	1	1	1	1	1	1	0.923	0.812	1	1	1	1	0	15	15	0	7	5	3	intergenic_re	MODIFIER	Pp3c21_1047	Pp3c21_1047	intergenic_re	Pp3c21_1047	NA	NA		n.6762677_6	NA	NA	NA	NA	NA	
Chr21	6806491	C	CT	Chr21_68064C_CT	INDEL	16471.59	24	16	19	24	17	26	32	25	21	16	21	12	19	6	24	16	19	24	17	26	32	25	21	16	21	12	19	6	1	1	1	1	1	1	1	1	1	1	1	1	1	1	0	15	15	0	7	5	3	intergenic_re	MODIFIER	Pp3c21_105	Pp3c21_105	intergenic_re	Pp3c21_105	NA	NA		n.6806491_6	NA	NA	NA	NA	NA	
Chr21	6806528	C	CT	Chr21_68065C_CT	INDEL	16074.59	24	19	17	20	16	31	30	18	21	10	12	16	14	12	24	19	17	20	16	31	30	18	21	10	12	16	14	12	1	1	1	1	1	1	1	1	1	1	1	1	1	1	0	15	15	0	7	5	3	intergenic_re	MODIFIER	Pp3c21_105	Pp3c21_105	intergenic_re	Pp3c21_105	NA	NA		n.6806528_6	NA	NA	NA	NA	NA	
Chr21	7050743	G	T	Chr21_70507G_T	SNP	18625.63	33	17	19	17	19	37	25	20	23	28	20	20	10	10	33	17	19	17	19	37	25	20	23	28	20	20	10	10	1	1	1	1	1	1	1	1	1	1	1	1	1	1	0	15	15	0	7	5	3	intergenic_re	MODIFIER	Pp3c21_111	Pp3c21_111	intergenic_re	Pp3c21_111	NA	NA		n.7050743G>	NA	NA	NA	NA	NA	
Chr21	7078420	G	A	Chr21_70784G_A	SNP	20843.63	39	22	18	30	22	33	23	30	19	23	16	21	22	16	39	22	18	30	22	33	23	30	19	23	16	21	22	16	1	1	1	1	1	1	1	1	1	1	1	1	1	1	0	15	15	0	7	5	3	intergenic_re	MODIFIER	Pp3c21_111	Pp3c21_111	intergenic_re	Pp3c21_111	NA	NA		n.7078420G>	NA	NA	NA	NA	NA	
Chr21	7239654	G	T	Chr21_72396G_T	SNP	8306.29	0	0	0	29	29	31	38	35	0	0	0	0	0	0	28	15	23	30	29	31	38	35	36	31	10	19	16	8	0	0	0	0.967	1	1	1	1	0	0	0	0	0	0	0	5	5	0	0	5	0	upstream_ge	MODIFIER	Pp3c21_114	Pp3c21_114	transcript	PAC:3291529	protein_codi	NA		c.-7663G>T	NA	NA	NA	NA		4365
Chr21	7239654	G	T	Chr21_72396G_T	SNP	8306.29	0	0	0	29	29	31	38	35	0	0	0	0	0	0	28	15	23	30	29	31	38	35	36	31	10	19	16	8	0	0	0	0.967	1	1	1	1	0	0	0	0	0	0	0	5	5	0	0	5	0	intergenic_re	MODIFIER	Pp3c21_113	Pp3c21_113	intergenic_re	Pp3c21_113	NA	NA		n.7239654G>	NA	NA	NA	NA	NA	
Chr21	7263054	A	AT	Chr21_72630A_AT	INDEL	13283.59	23	9	18	24	18	23	34	24	26	23	13	6	5	17	23	9	18	24	18	23	34	24	26	23	13	6	5	17	1	1	1	1	1	1	1	1	1	1	1	1	1	1	0	15	15	0	7	5	3	upstream_ge	MODIFIER	Pp3c21_114	Pp3c21_114	transcript	PAC:3291561	protein_codi	NA		c.-4197_-419	NA	NA	NA	NA		3534
Chr21	7263054	A	AT	Chr21_72630A_AT	INDEL	13283.59	23	9	18	24	18	23	34	24	26	23	13	6	5	17	23	9	18	24	18	23	34	24	26	23	13	6	5	17	1	1	1	1	1	1	1	1	1	1	1	1	1	1	0	15	15	0	7	5	3	upstream_ge	MODIFIER	Pp3c21_114	Pp3c21_114	transcript	PAC:3291561	protein_codi	NA		c.-3723_-372	NA	NA	NA	NA		3540
Chr21	7263054	A	AT	Chr21_72630A_AT	INDEL	13283.59	23	9	18	24	18	23	34	24	26	23	13	6	5	17	23	9	18	24	18	23	34	24	26	23	13	6	5	17	1	1	1	1	1	1	1	1	1	1	1	1	1	1	0	15	15	0	7	5	3	upstream_ge	MODIFIER	Pp3c21_114	Pp3c21_114	transcript	PAC:3291561	protein_codi	NA		c.-4197_-419	NA	NA	NA	NA		4157
Chr21	7263054	A	AT	Chr21_72630A_AT	INDEL	13283.59	23	9	18	24	18	23	34	24	26	23	13	6	5	17	23	9	18	24	18	23	34	24	26	23	13	6	5	17	1	1	1	1	1	1	1	1	1	1	1	1	1	1	0	15	15	0	7	5	3	upstream_ge	MODIFIER	Pp3c21_114	Pp3c21_114	transcript	PAC:3291561	protein_codi	NA		c.-3723_-372	NA	NA	NA	NA		3543
Chr21	7263054	A	AT	Chr21_72630A_AT	INDEL	13283.59	23	9	18	24	18	23	34	24	26	23	13	6	5	17	23	9	18	24	18	23	34	24	26	23	13	6	5	17	1	1	1	1	1	1	1	1	1	1	1	1	1	1	0	15	15	0	7	5	3	upstream_ge	MODIFIER	Pp3c21_114	Pp3c21_114	transcript	PAC:3291561	protein_codi	NA		c.-3723_-372	NA	NA	NA	NA		3543
Chr21	7263054	A	AT	Chr21_72630A_AT	INDEL	13283.59	23	9	18	24	18	23	34	24	26	23	13	6	5	17	23	9	18	24	18	23	34	24	26	23	13	6	5	17	1	1	1	1	1	1	1	1	1	1	1	1	1	1	0	15	15	0	7	5	3	upstream_ge	MODIFIER	Pp3c21_114	Pp3c21_114	transcript	PAC:3291561	protein_codi	NA		c.-3723_-372	NA	NA	NA	NA		3543
Chr21	7263054	A	AT	Chr21_72630A_AT	INDEL	13283.59	23	9	18	24	18	23	34	24	26	23	13	6	5	17	23	9	18	24	18	23	34	24	26	23	13	6	5	17	1	1	1	1	1	1	1	1	1	1	1	1	1	1	0	15	15	0	7	5	3	upstream_ge	MODIFIER	Pp3c21_114	Pp3c21_114	transcript	PAC:3291561	protein_codi	NA		c.-3723_-372	NA	NA	NA	NA		3543
Chr21	7263054	A	AT	Chr21_72630A_AT	INDEL	13283.59	23	9	18	24	18	23	34	24	26	23	13	6	5	17	23	9	18	24	18	23	34	24	26	23	13	6	5	17	1	1	1	1	1	1	1	1	1	1	1	1	1	1	0	15	15	0	7	5	3	upstream_ge	MODIFIER	Pp3c21_114	Pp3c21_114	transcript	PAC:3291561	protein_codi	NA		c.-3723_-372	NA	NA	NA	NA		3543
Chr21	7263054	A	AT	Chr21_72630A_AT	INDEL	13283.59	23	9	18	24	18	23	34	24	26	23	13	6	5	17	23	9	18	24	18	23	34	24	26	23	13	6	5	17	1	1	1	1	1	1	1	1	1	1	1	1	1	1	0	15	15	0	7	5	3	upstream_ge	MODIFIER	Pp3c21_114	Pp3c21_114	transcript	PAC:3291561	protein_codi	NA		c.-4197_-419	NA	NA	NA	NA		3534
Chr21	7263054	A	AT	Chr21_72630A_AT	INDEL	13283.59	23	9	18	24	18	23	34	24	26	23	13	6	5	17	23	9	18	24	18	23	34	24	26	23	13	6	5	17	1	1	1	1	1	1	1	1	1	1	1	1	1	1	0	15	15	0	7	5	3	intergenic_re	MODIFIER	Pp3c21_114	Pp3c21_114	intergenic_re	Pp3c21_114	NA	NA		n.7263054_7	NA	NA	NA	NA	NA	
Chr21	7329000	C	T	Chr21_73290C_T	SNP	9743.63	10	10	10	14	7	15	16	19	18	12	7	8	6	9	10	10	10	14	7	15	16	19	18	12	7	8	6	9	1	1	1	1	1	1	1	1	1	1	1	1	1	1	0	15	15	0	7	5	3	downstream	MODIFIER	Pp3c21_115	Pp3c21_115	transcript	PAC:3291507	protein_codi	NA		c.*3360C>T	NA	NA	NA	NA		3360
Chr21	7329000	C	T	Chr21_73290C_T	SNP	9743.63	10	10	10	14	7	15	16	19	18	12	7	8	6	9	10	10	10	14	7	15	16	19	18	12	7	8	6	9	1	1	1	1	1	1	1	1	1	1	1	1	1	1	0	15	15	0	7	5	3	intergenic_re	MODIFIER	Pp3c21_115	Pp3c21_115	intergenic_re	Pp3c21_115	NA	NA		n.7329000C>	NA	NA	NA	NA	NA	
Chr21	7330146	C	CT	Chr21_73301C_CT	INDEL	1769.24	0	0	0	16	6	7	10	8	0	0	0	0	0	0	9	4	5	16	6	7	10	8	7	9	7	6	4	6	0	0	0	1	1	1	1	1	0	0	0	0	0	0	0	5	5	0	0	5	0	downstream	MODIFIER	Pp3c21_115	Pp3c21_115	transcript	PAC:3291507	protein_codi	NA		c.*4506_*45	NA	NA	NA	NA		4507
Chr21	7330146	C	CT	Chr21_73301C_CT	INDEL	1769.24	0	0	0	16	6	7	10	8	0	0	0	0	0	0	9	4	5	16	6	7	10	8	7	9	7	6	4	6	0	0	0	1	1	1	1	1	0	0	0	0	0	0	0	5	5	0	0	5	0	intergenic_re	MODIFIER	Pp3c21_115	Pp3c21_115	intergenic_re	Pp3c21_115	NA	NA		n.7330146_7	NA	NA	NA	NA	NA	
Chr21	7330767	GA	G	Chr21_73307GA_G	INDEL	20020.59	28	9	17	19	24	32	18	26	20	31	16	15	8	16	28	9	17	19	24	32	18	26	20	31	16	15	8	16	1	1	1	1	1	1	1	1	1	1	1	1	1	1	0	15	15	0	7	5	3	intergenic_re	MODIFIER	Pp3c21_115	Pp3c21_115	intergenic_re	Pp3c21_115	NA	NA		n.7330768de	NA	NA	NA	NA	NA	
Chr21	7330772	A	AT	Chr21_73307A_AT	INDEL	20020.59	28	9	17	19	24	31	18	26	20	31	16	13	8	16	28	9	17	19	24	31	18	26	20	31	16	13	8	16	1	1	1	1	1	1	1	1	1	1	1	1	1	1	0	15	15	0	7	5	3	intergenic_re	MODIFIER	Pp3c21_115	Pp3c21_115	intergenic_re	Pp3c21_115	NA	NA		n.7330772_7	NA	NA	NA	NA	NA	
Chr21	7330819	AT	A	Chr21_73308AT_A	INDEL	11429.59	11	6	11	6	15	19	14	16	14	23	7	4	6	7	11	6	11	6	15	19	14	16	14	23	7	4	6	7	1	1	1	1	1	1	1	1	1	1	1	1	1	1	0	15	15	0	7	5	3	intergenic_re	MODIFIER	Pp3c21_115	Pp3c21_115	intergenic_re	Pp3c21_115	NA	NA		n.7330820de	NA	NA	NA	NA	NA	
Chr21	7337876	C	A	Chr21_73378C_A	SNP	20215.63	33	18	11	26	26	25	25	33	24	25	23	14	10	10	33	18	11	26	26	25	25	33	24	25	23	14	10	10	1	1	1	1	1	1	1	1	1	1	1	1	1	1	0	15	15	0	7	5	3	intergenic_re	MODIFIER	Pp3c21_115	Pp3c21_115	intergenic_re	Pp3c21_115	NA	NA		n.7337876C>	NA	NA	NA	NA	NA	
Chr21	7340213	A	T	Chr21_73402A_T	SNP	18631.63	32	12	21	20	20	28	19	15	23	9	24	11	7	18	32	12	21	20	20	28	19	15	23	9	24	11	7	18	1	1	1	1	1	1	1	1	1	1	1	1	1	1	0	15	15	0	7	5	3	intergenic_re	MODIFIER	Pp3c21_115	Pp3c21_115	intergenic_re	Pp3c21_115	NA	NA		n.7340213A>	NA	NA	NA	NA	NA	
Chr21	7340214	A	T	Chr21_73402A_T	SNP	18631.63	32	11	21	20	20	29	19	15	23	9	24	11	7	18	32	11	21	20	20	29	19	15	23	9	24	11	7	18	1	1	1	1	1	1	1	1	1	1	1	1	1	1	0	15	15	0	7	5	3	intergenic_re	MODIFIER	Pp3c21_115	Pp3c21_115	intergenic_re	Pp3c21_115	NA	NA		n.7340214A>	NA	NA	NA	NA	NA	
Chr21	7514479	A	T	Chr21_75144A_T	SNP	5705.29	0	0	0	25	18	22	21	18	0	0	0	0	0	0	26	16	13	25	18	22	21	18	16	14	13	10	13	8	0	0	0	1	1	1	1	1	0	0	0	0	0	0	0	5	5	0	0	5	0	downstream	MODIFIER	Pp3c21_117	Pp3c21_117	transcript	PAC:3291503	protein_codi	NA		c.*3347A>T	NA	NA	NA	NA		2933
Chr21	7514479	A	T	Chr21_75144A_T	SNP	5705.29	0	0	0	25	18	22	21	18	0	0	0	0	0	0	26	16	13	25	18	22	21	18	16	14	13	10	13	8	0	0	0	1	1	1	1	1	0	0	0	0	0	0	0	5	5	0	0	5	0	downstream	MODIFIER	Pp3c21_117	Pp3c21_117	transcript	PAC:3291503	protein_codi	NA		c.*3347A>T	NA	NA	NA	NA		2954
Chr21	7514479	A	T	Chr21_75144A_T	SNP	5705.29	0	0	0	25	18	22	21	18	0	0	0	0	0	0	26	16	13	25	18	22	21	18	16	14	13	10	13	8	0	0	0	1	1	1	1	1	0	0	0	0	0	0	0	5	5	0	0	5	0	intergenic_re	MODIFIER	Pp3c21_117	Pp3c21_117	intergenic_re	Pp3c21_117	NA	NA		n.7514479A>	NA	NA	NA	NA	NA	
Chr21	7516021	GA	G	Chr21_75160GA_G	INDEL	17320.59	29	15	18	28	21	21	32	13	22	20	14	14	10	14	29	15	18	28	21	21	32	13	22	20	14	14	10	14	1	1	1	1	1	1	1	1	1	1	1	1	1	1	0	15	15	0	7	5	3	downstream	MODIFIER	Pp3c21_117	Pp3c21_117	transcript	PAC:3291503	protein_codi	NA		c.*4890delA	NA	NA	NA	NA		4476
Chr21	7516021	GA	G	Chr21_75160GA_G	INDEL	17320.59	29	15	18	28	21	21	32	13	22	20	14	14	10	14	29	15	18	28	21	21	32	13	22	20	14	14	10	14	1	1	1	1	1	1	1	1	1	1	1	1	1	1	0	15	15	0	7	5	3	downstream	MODIFIER	Pp3c21_117	Pp3c21_117	transcript	PAC:3291503	protein_codi	NA		c.*4890delA	NA	NA	NA	NA		4497
Chr21	7516021	GA	G	Chr21_75160GA_G	INDEL	17320.59	29	15	18	28	21	21	32	13	22	20	14	14	10	14	29	15	18	28	21	21	32	13	22	20	14	14	10	14	1	1	1	1	1	1	1	1	1	1	1	1	1	1	0	15	15	0	7	5	3	intergenic_re	MODIFIER	Pp3c21_117	Pp3c21_117	intergenic_re	Pp3c21_117	NA	NA		n.7516022de	NA	NA	NA	NA	NA	
Chr21	7600431	A	AAT	Chr21_76004A_AAT	INDEL	1769.35	27	12	15	0	0	0	0	0	0	0	1	0	1	0	27	13	15	13	15	24	25	15	17	18	9	11	9	9	1	0.923	1	0	0	0	0	0	0	0	0	0	0	0	0	3	3	0	0	0	0	upstream_ge	MODIFIER	Pp3c21_118	Pp3c21_118	transcript	PAC:3291621	protein_codi	NA		c.-2426_-242	NA	NA	NA	NA		2218
Chr21	7600431	A	AAT	Chr21_76004A_AAT	INDEL	1769.35	27	12	15	0	0	0	0	0	0	0	1	0	1	0	27	13	15	13	15	24	25	15	17	18	9	11	9	9	1	0.923	1	0	0	0	0	0	0	0	0	0	0	0	0	3	3	0	0	0	0	upstream_ge	MODIFIER	Pp3c21_118	Pp3c21_118	transcript	PAC:3291486	protein_codi	NA		c.-4079_-407	NA	NA	NA	NA		3824
Chr21	7600431	A	AAT	Chr21_76004A_AAT	INDEL	1769.35	27	12	15	0	0	0	0	0	0	0	1	0	1	0	27	13	15	13	15	24	25	15	17	18	9	11	9	9	1	0.923	1	0	0	0	0	0	0	0	0	0	0	0	0	3	3	0	0	0	0	upstream_ge	MODIFIER	Pp3c21_118	Pp3c21_118	transcript	PAC:3291621	protein_codi	NA		c.-2668_-266	NA	NA	NA	NA		2278
Chr21	7600431	A	AAT	Chr21_76004A_AAT	INDEL	1769.35	27	12	15	0	0	0	0	0	0	0	1	0	1	0	27	13	15	13	15	24	25	15	17	18	9	11	9	9	1	0.923	1	0	0	0	0	0	0	0	0	0	0	0	0	3	3	0	0	0	0	upstream_ge	MODIFIER	Pp3c21_118	Pp3c21_118	transcript	PAC:3291622	protein_codi	NA		c.-2668_-266	NA	NA	NA	NA		2218
Chr21	7600431	A	AAT	Chr21_76004A_AAT	INDEL	1769.35	27	12	15	0	0	0	0	0	0	0	1	0	1	0	27	13	15	13	15	24	25	15	17	18	9	11	9	9	1	0.923	1	0	0	0	0	0	0	0	0	0	0	0	0	3	3	0	0	0	0	upstream_ge	MODIFIER	Pp3c21_118	Pp3c21_118	transcript	PAC:3291622	protein_codi	NA		c.-3381_-338	NA	NA	NA	NA		2218
Chr21	7600431	A	AAT	Chr21_76004A_AAT	INDEL	1769.35	27	12	15	0	0	0	0	0	0	0	1	0	1	0	27	13	15	13	15	24	25	15	17	18	9	11	9	9	1	0.923	1	0	0	0	0	0	0	0	0	0	0	0	0	3	3	0	0	0	0	intergenic_re	MODIFIER	Pp3c21_118	Pp3c21_118	intergenic_re	Pp3c21_118	NA	NA		n.7600431_7	NA	NA	NA	NA	NA	
Chr21	7653322	T	A	Chr21_76533T_A	SNP	18389.63	21	15	12	23	20	27	31	31	31	27	17	18	5	11	21	15	12	23	20	27	31	31	31	27	17	18	5	11	1	1	1	1	1	1	1	1	1	1	1	1	1	1	0	15	15	0	7	5	3	intergenic_re	MODIFIER	Pp3c21_119	Pp3c21_119	intergenic_re	Pp3c21_119	NA	NA		n.7653322T>	NA	NA	NA	NA	NA	
Chr21	7693744	A	T	Chr21_76937A_T	SNP	15497.63	33	11	18	15	11	31	20	16	9	13	13	9	10	4	33	11	18	15	11	31	20	16	9	13	13	9	10	4	1	1	1	1	1	1	1	1	1	1	1	1	1	1	0	15	15	0	7	5	3	intergenic_re	MODIFIER	Pp3c21_119	Pp3c21_119	intergenic_re	Pp3c21_119	NA	NA		n.7693744A>	NA	NA	NA	NA	NA	
Chr21	7693745	A	T	Chr21_76937A_T	SNP	15497.63	34	11	18	16	11	31	20	16	10	13	13	9	10	4	34	11	18	16	11	31	20	16	10	13	13	9	10	4	1	1	1	1	1	1	1	1	1	1	1	1	1	1	0	15	15	0	7	5	3	intergenic_re	MODIFIER	Pp3c21_119	Pp3c21_119	intergenic_re	Pp3c21_119	NA	NA		n.7693745A>	NA	NA	NA	NA	NA	
Chr21	7820271	A	T	Chr21_78202A_T	SNP	18364.63	30	18	10	14	16	32	29	27	26	13	16	24	9	6	30	18	10	14	16	32	29	27	26	13	16	24	9	6	1	1	1	1	1	1	1	1	1	1	1	1	1	1	0	15	15	0	7	5	3	intergenic_re	MODIFIER	Pp3c21_123	Pp3c21_123	intergenic_re	Pp3c21_123	NA	NA		n.7820271A>	NA	NA	NA	NA	NA	
Chr21	7975792	T	C	Chr21_79757T_C	SNP	15782.63	24	10	12	13	16	22	25	17	30	15	9	12	5	8	24	10	12	13	16	22	25	17	30	15	9	12	5	8	1	1	1	1	1	1	1	1	1	1	1	1	1	1	0	15	15	0	7	5	3	intergenic_re	MODIFIER	Pp3c21_124	Pp3c21_124	intergenic_re	Pp3c21_124	NA	NA		n.7975792T>	NA	NA	NA	NA	NA	
Chr21	7978907	T	C	Chr21_79789T_C	SNP	9456.29	19	20	14	0	0	0	0	0	18	15	11	16	7	14	19	20	14	21	12	16	34	18	18	15	11	16	7	14	1	1	1	0	0	0	0	0	1	1	1	1	1	1	0	10	10	0	7	0	3	intergenic_re	MODIFIER	Pp3c21_124	Pp3c21_124	intergenic_re	Pp3c21_124	NA	NA		n.7978907T>	NA	NA	NA	NA	NA	
Chr21	8028991	A	AAT	Chr21_80289A_AAT	INDEL	2326.35	29	17	18	0	0	0	0	0	0	0	0	0	0	0	29	17	18	13	26	34	35	23	24	24	11	12	9	8	1	1	1	0	0	0	0	0	0	0	0	0	0	0	0	3	3	0	0	0	0	intergenic_re	MODIFIER	Pp3c21_124	Pp3c21_124	intergenic_re	Pp3c21_124	NA	NA		n.8028991_8	NA	NA	NA	NA	NA	
Chr21	8060041	C	A	Chr21_80600C_A	SNP	15894.63	22	9	10	24	16	19	25	22	6	10	16	18	6	5	22	9	10	24	16	19	25	22	6	10	16	18	6	5	1	1	1	1	1	1	1	1	1	1	1	1	1	1	0	15	15	0	7	5	3	intergenic_re	MODIFIER	Pp3c21_126	Pp3c21_126	intergenic_re	Pp3c21_126	NA	NA		n.8060041C>	NA	NA	NA	NA	NA	
Chr21	8060042	C	A	Chr21_80600C_A	SNP	15939.63	22	9	10	24	16	19	23	22	6	10	16	18	6	5	22	9	10	24	16	19	23	22	6	10	16	18	6	5	1	1	1	1	1	1	1	1	1	1	1	1	1	1	0	15	15	0	7	5	3	intergenic_re	MODIFIER	Pp3c21_126	Pp3c21_126	intergenic_re	Pp3c21_126	NA	NA		n.8060042C>	NA	NA	NA	NA	NA	
Chr21	8060049	C	A	Chr21_80600C_A	SNP	15905.63	20	10	11	24	16	19	22	20	6	14	14	18	6	5	20	10	11	24	16	19	22	20	6	14	14	18	6	5	1	1	1	1	1	1	1	1	1	1	1	1	1	1	0	15	15	0	7	5	3	intergenic_re	MODIFIER	Pp3c21_126	Pp3c21_126	intergenic_re	Pp3c21_126	NA	NA		n.8060049C>	NA	NA	NA	NA	NA	
Chr21	8060056	C	T	Chr21_80600C_T	SNP	15636.63	20	10	11	25	19	20	20	20	7	14	16	16	6	4	20	10	11	25	19	20	20	20	7	14	16	16	6	4	1	1	1	1	1	1	1	1	1	1	1	1	1	1	0	15	15	0	7	5	3	intergenic_re	MODIFIER	Pp3c21_126	Pp3c21_126	intergenic_re	Pp3c21_126	NA	NA		n.8060056C>	NA	NA	NA	NA	NA	
Chr21	8062606	G	GA	Chr21_80626G_GA	INDEL	9945.59	14	11	6	15	15	12	18	13	15	18	11	5	8	7	14	11	6	15	15	12	18	13	15	18	11	5	8	7	1	1	1	1	1	1	1	1	1	1	1	1	1	1	0	15	15	0	7	5	3	intergenic_re	MODIFIER	Pp3c21_126	Pp3c21_126	intergenic_re	Pp3c21_126	NA	NA		n.8062606_8	NA	NA	NA	NA	NA	
Chr21	8062665	CT	C	Chr21_80626CT_C	INDEL	11610.59	14	9	7	12	15	12	17	12	14	17	9	10	8	8	14	9	7	12	15	12	17	12	14	17	9	10	8	8	1	1	1	1	1	1	1	1	1	1	1	1	1	1	0	15	15	0	7	5	3	intergenic_re	MODIFIER	Pp3c21_126	Pp3c21_126	intergenic_re	Pp3c21_126	NA	NA		n.8062666de	NA	NA	NA	NA	NA	
Chr21	8062667	T	A	Chr21_80626T_A	SNP	11619.63	14	9	7	12	15	12	17	12	14	17	9	10	8	8	14	9	7	12	15	12	17	12	14	17	9	10	8	8	1	1	1	1	1	1	1	1	1	1	1	1	1	1	0	15	15	0	7	5	3	intergenic_re	MODIFIER	Pp3c21_126	Pp3c21_126	intergenic_re	Pp3c21_126	NA	NA		n.8062667T>	NA	NA	NA	NA	NA	
Chr21	8062687	T	A	Chr21_80626T_A	SNP	9290.63	12	6	6	10	12	11	14	8	12	8	8	7	8	5	12	6	6	10	12	11	14	8	12	8	8	7	8	5	1	1	1	1	1	1	1	1	1	1	1	1	1	1	0	15	15	0	7	5	3	intergenic_re	MODIFIER	Pp3c21_126	Pp3c21_126	intergenic_re	Pp3c21_126	NA	NA		n.8062687T>	NA	NA	NA	NA	NA	
Chr21	8073103	C	T	Chr21_80731C_T	SNP	12402.63	19	12	11	16	13	13	17	12	12	17	8	13	6	7	19	12	11	16	13	13	17	12	12	17	8	13	6	7	1	1	1	1	1	1	1	1	1	1	1	1	1	1	0	15	15	0	7	5	3	intergenic_re	MODIFIER	Pp3c21_126	Pp3c21_126	intergenic_re	Pp3c21_126	NA	NA		n.8073103C>	NA	NA	NA	NA	NA	
Chr21	8074642	C	A	Chr21_80746C_A	SNP	16135.63	22	9	12	30	17	32	9	20	11	18	8	18	10	7	22	9	12	30	17	32	9	20	11	18	8	18	10	7	1	1	1	1	1	1	1	1	1	1	1	1	1	1	0	15	15	0	7	5	3	intergenic_re	MODIFIER	Pp3c21_126	Pp3c21_126	intergenic_re	Pp3c21_126	NA	NA		n.8074642C>	NA	NA	NA	NA	NA	
Chr21	8101586	C	T	Chr21_81015C_T	SNP	15566.63	34	13	15	26	5	16	28	19	26	16	17	20	8	10	34	13	15	26	5	16	28	19	26	16	17	20	8	10	1	1	1	1	1	1	1	1	1	1	1	1	1	1	0	15	15	0	7	5	3	intergenic_re	MODIFIER	Pp3c21_126	Pp3c21_126	intergenic_re	Pp3c21_126	NA	NA		n.8101586C>	NA	NA	NA	NA	NA	
Chr21	8109512	G	C	Chr21_81095G_C	SNP	20428.63	26	13	14	31	23	29	43	23	31	20	18	18	8	18	26	13	14	31	23	29	43	23	31	20	18	18	8	18	1	1	1	1	1	1	1	1	1	1	1	1	1	1	0	15	15	0	7	5	3	intergenic_re	MODIFIER	Pp3c21_126	Pp3c21_126	intergenic_re	Pp3c21_126	NA	NA		n.8109512G>	NA	NA	NA	NA	NA	
Chr21	8171846	T	A	Chr21_81718T_A	SNP	15084.63	37	11	9	22	12	24	28	21	13	15	19	15	4	8	37	11	9	22	12	24	28	21	13	15	19	15	4	8	1	1	1	1	1	1	1	1	1	1	1	1	1	1	0	15	15	0	7	5	3	intergenic_re	MODIFIER	Pp3c21_126	Pp3c21_126	intergenic_re	Pp3c21_126	NA	NA		n.8171846T>	NA	NA	NA	NA	NA	
Chr21	8171902	G	A	Chr21_81719G_A	SNP	10768.63	23	7	5	17	10	22	25	9	9	9	10	12	8	4	23	7	5	17	10	22	25	9	9	9	10	12	8	4	1	1	1	1	1	1	1	1	1	1	1	1	1	1	0	15	15	0	7	5	3	intergenic_re	MODIFIER	Pp3c21_126	Pp3c21_126	intergenic_re	Pp3c21_126	NA	NA		n.8171902G>	NA	NA	NA	NA	NA	
Chr21	8299202	G	A	Chr21_82992G_A	SNP	16938.63	27	14	13	21	19	25	16	22	22	17	16	19	9	16	27	14	13	21	19	25	16	22	22	17	16	19	9	16	1	1	1	1	1	1	1	1	1	1	1	1	1	1	0	15	15	0	7	5	3	intergenic_re	MODIFIER	Pp3c21_127	Pp3c21_127	intergenic_re	Pp3c21_127	NA	NA		n.8299202G>	NA	NA	NA	NA	NA	
Chr21	8511292	C	T	Chr21_85112C_T	SNP	5762.29	0	0	0	25	15	19	27	25	0	0	0	0	0	0	35	11	14	25	15	19	27	25	16	20	12	17	19	5	0	0	0	1	1	1	1	1	0	0	0	0	0	0	0	5	5	0	0	5	0	intergenic_re	MODIFIER	Pp3c21_132	Pp3c21_132	intergenic_re	Pp3c21_132	NA	NA		n.8511292C>	NA	NA	NA	NA	NA	
Chr21	8736305	A	AT	Chr21_87363A_AT	INDEL	14782.59	28	11	12	28	19	15	24	19	19	26	11	27	6	8	28	11	12	28	19	15	24	19	19	26	11	27	6	8	1	1	1	1	1	1	1	1	1	1	1	1	1	1	0	15	15	0	7	5	3	intergenic_re	MODIFIER	Pp3c21_135	Pp3c21_135	intergenic_re	Pp3c21_135	NA	NA		n.8736305_8	NA	NA	NA	NA	NA	
Chr21	8775075	C	A	Chr21_87750C_A	SNP	13199.29	31	18	17	0	0	2	0	0	17	20	16	21	9	9	31	18	17	27	24	28	27	27	17	20	16	21	9	9	1	1	1	0	0	0.071	0	0	1	1	1	1	1	1	0	10	10	1	7	0	3	intergenic_re	MODIFIER	Pp3c21_136	Pp3c21_136	intergenic_re	Pp3c21_136	NA	NA		n.8775075C>	NA	NA	NA	NA	NA	
Chr21	8781993	GC	G	Chr21_87819GC_G	INDEL	8557.59	9	5	8	13	8	15	12	9	13	13	8	8	4	4	9	5	8	13	8	15	12	9	13	13	8	8	4	4	1	1	1	1	1	1	1	1	1	1	1	1	1	1	0	15	15	0	7	5	3	intergenic_re	MODIFIER	Pp3c21_136	Pp3c21_136	intergenic_re	Pp3c21_136	NA	NA		n.8781994de	NA	NA	NA	NA	NA	

Chr21	8847414	C	G	Chr21_88474C_G	SNP	32378.63	53	31	25	52	43	39	61	47	41
Chr21	8847414	C	G	Chr21_88474C_G	SNP	32378.63	53	31	25	52	43	39	61	47	41
Chr21	8847414	C	G	Chr21_88474C_G	SNP	32378.63	53	31	25	52	43	39	61	47	41
Chr21	8938776	T	G	Chr21_89387T_G	SNP	20992.63	40	21	14	23	22	25	41	25	20
Chr21	8938776	T	G	Chr21_89387T_G	SNP	20992.63	40	21	14	23	22	25	41	25	20
Chr21	8938776	T	G	Chr21_89387T_G	SNP	20992.63	40	21	14	23	22	25	41	25	20
Chr21	9024978	G	GA	Chr21_90249G_GA	INDEL	24759.59	63	32	18	28	34	34	48	28	32
Chr21	9024978	G	GA	Chr21_90249G_GA	INDEL	24759.59	63	32	18	28	34	34	48	28	32
Chr21	9100281	G	GA	Chr21_91002G_GA	INDEL	14193.59	16	9	16	16	13	23	33	18	17
Chr21	9100281	G	GA	Chr21_91002G_GA	INDEL	14193.59	16	9	16	16	13	23	33	18	17
Chr21	9100281	G	GA	Chr21_91002G_GA	INDEL	14193.59	16	9	16	16	13	23	33	18	17
Chr21	9100281	G	GA	Chr21_91002G_GA	INDEL	14193.59	16	9	16	16	13	23	33	18	17
Chr21	9130421	G	GTA	Chr21_91304G_GTA	INDEL	3242.24	0	0	0	21	13	14	22	21	0
Chr21	9130421	G	GTA	Chr21_91304G_GTA	INDEL	3242.24	0	0	0	21	13	14	22	21	0
Chr21	9130421	G	GTA	Chr21_91304G_GTA	INDEL	3242.24	0	0	0	21	13	14	22	21	0
Chr21	9130421	G	GTA	Chr21_91304G_GTA	INDEL	3242.24	0	0	0	21	13	14	22	21	0
Chr21	9130421	G	GTA	Chr21_91304G_GTA	INDEL	3242.24	0	0	0	21	13	14	22	21	0
Chr21	9130421	G	GTA	Chr21_91304G_GTA	INDEL	3242.24	0	0	0	21	13	14	22	21	0
Chr21	9130421	G	GTA	Chr21_91304G_GTA	INDEL	3242.24	0	0	0	21	13	14	22	21	0
Chr21	9130421	G	GTA	Chr21_91304G_GTA	INDEL	3242.24	0	0	0	21	13	14	22	21	0
Chr21	9140995	TTA	T	Chr21_91409TTA_T	INDEL	9011.59	25	8	12	14	10	14	28	19	8
Chr21	9140995	TTA	T	Chr21_91409TTA_T	INDEL	9011.59	25	8	12	14	10	14	28	19	8
Chr21	9140995	TTA	T	Chr21_91409TTA_T	INDEL	9011.59	25	8	12	14	10	14	28	19	8
Chr21	9140995	TTA	T	Chr21_91409TTA_T	INDEL	9011.59	25	8	12	14	10	14	28	19	8

[illegible]

Chr21	12170074	A	AAT	Chr21_12170A_AAT	INDEL	1632.35	27	14	10	0	0	0	1	0	0	0
Chr21	12224142	G	GA	Chr21_12224G_GA	INDEL	9664.59	16	5	9	13	9	17	28	19	22	11
Chr21	12299470	C	A	Chr21_12299C_A	SNP	19834.63	31	16	17	25	22	21	13	29	20	24
Chr21	12299471	C	A	Chr21_12299C_A	SNP	19834.63	31	16	17	25	21	21	13	29	20	24
Chr21	12299561	G	T	Chr21_12299G_T	SNP	17967.63	22	18	14	28	9	27	14	23	25	22
Chr21	12305051	TG	T	Chr21_12305TG_T	INDEL	15465.59	20	7	15	24	22	17	15	21	12	19
Chr21	12305054	G	T	Chr21_12305G_T	SNP	15474.63	20	7	15	24	23	18	15	22	12	19
Chr21	12406380	AT	A	Chr21_12406AT_A	INDEL	2847.24	0	0	0	19	20	24	21	17	0	1
Chr21	12406380	AT	A	Chr21_12406AT_A	INDEL	2847.24	0	0	0	19	20	24	21	17	0	1
Chr21	12406380	AT	A	Chr21_12406AT_A	INDEL	2847.24	0	0	0	19	20	24	21	17	0	1
Chr21	12434823	C	A	Chr21_12434C_A	SNP	18324.63	27	21	19	26	23	25	22	21	13	24
Chr21	12434869	T	TA	Chr21_12434T_TA	INDEL	12668.59	19	14	14	19	15	17	17	13	12	16
Chr21	12434888	G	GA	Chr21_12434G_GA	INDEL	11197.59	15	12	8	19	13	15	14	14	7	15
Chr21	12434918	T	G	Chr21_12434T_G	SNP	11183.63	12	12	8	13	20	15	13	10	7	15
Chr21	12434919	G	A	Chr21_12434G_A	SNP	11183.63	12	12	8	13	20	16	14	10	7	15
Chr21	12448702	TA	T	Chr21_12448TA_T	INDEL	12436.59	25	5	7	24	23	23	17	17	16	12
Chr21	12448733	CT	C	Chr21_12448CT_C	INDEL	16921.59	22	5	9	23	20	25	15	15	14	11
Chr21	12448752	T	C	Chr21_12448T_C	SNP	17954.63	23	6	10	23	18	28	21	17	15	9
Chr21	12448753	C	A	Chr21_12448C_A	SNP	17985.63	23	6	10	23	19	28	21	17	16	9
Chr21	12448766	T	C	Chr21_12448T_C	SNP	18382.63	23	6	12	23	20	29	22	16	16	9
Chr21	12448824	AT	A	Chr21_12448AT_A	INDEL	11813.59	20	9	11	18	12	19	22	20	16	11
Chr21	12448872	A	G	Chr21_12448A_G	SNP	18818.63	25	11	15	19	20	24	27	21	25	14
Chr21	12448987	G	T	Chr21_12448G_T	SNP	23943.63	35	14	18	35	26	27	30	23	35	26
Chr21	12448997	G	T	Chr21_12448G_T	SNP	23971.63	34	16	17	39	27	26	30	22	34	26
Chr21	12450270	C	A	Chr21_12450C_A	SNP	17905.63	31	11	20	19	20	22	23	17	20	17
Chr21	12450294	C	A	Chr21_12450C_A	SNP	18526.63	32	10	20	21	20	22	22	23	20	19
Chr21	12564504	A	T	Chr21_12564A_T	SNP	16859.63	24	7	14	15	17	16	36	15	26	25
Chr21	12564512	A	T	Chr21_12564A_T	SNP	17681.63	22	8	15	16	19	18	34	14	25	25
Chr21	12564514	A	T	Chr21_12564A_T	SNP	17501.63	23	8	17	17	17	19	36	14	25	25
Chr21	12937181	G	GA	Chr21_12937G_GA	INDEL	15555.59	30	19	18	30	21	26	19	14	24	23
Chr21	13016781	CT	C	Chr21_13016CT_C	INDEL	11631.59	22	14	20	25	15	28	20	21	18	24
Chr21	13016781	CT	C	Chr21_13016CT_C	INDEL	11631.59	22	14	20	25	15	28	20	21	18	24
Chr21	13016781	CT	C	Chr21_13016CT_C	INDEL	11631.59	22	14	20	25	15	28	20	21	18	24
Chr21	13016781	CT	C	Chr21_13016CT_C	INDEL	11631.59	22	14	20	25	15	28	20	21	18	24
Chr21	13016781	CT	C	Chr21_13016CT_C	INDEL	11631.59	22	14	20	25	15	28	20	21	18	24
Chr21	13046443	G	C	Chr21_13046G_C	SNP	9178.63	18	9	14	6	4	12	14	11	19	11
Chr21	13046443	G	C	Chr21_13046G_C	SNP	9178.63	18	9	14	6	4	12	14	11	19	11
Chr21	13046443	G	C	Chr21_13046G_C	SNP	9178.63	18	9	14	6	4	12	14	11	19	11
Chr21	13087348	A	T	Chr21_13087A_T	SNP	19739.63	25	14	20	25	23	13	36	31	16	21
Chr21	13087348	A	T	Chr21_13087A_T	SNP	19739.63	25	14	20	25	23	13	36	31	16	21
Chr21	13087348	A	T	Chr21_13087A_T	SNP	19739.63	25	14	20	25	23	13	36	31	16	21
Chr21	13087348	A	T	Chr21_13087A_T	SNP	19739.63	25	14	20	25	23	13	36	31	16	21
Chr21	13087348	A	T	Chr21_13087A_T	SNP	19739.63	25	14	20	25	23	13	36	31	16	21
Chr21	13087385	T	TA	Chr21_13087T_TA	INDEL	17567.59	28	14	17	31	21	8	30	31	19	22
Chr21	13087385	T	TA	Chr21_13087T_TA	INDEL	17567.59	28	14	17	31	21	8	30	31	19	22
Chr21	13087385	T	TA	Chr21_13087T_TA	INDEL	17567.59	28	14	17	31	21	8	30	31	19	22
Chr21	13087385	T	TA	Chr21_13087T_TA	INDEL	17567.59	28	14	17	31	21	8	30	31	19	22
Chr21	13087385	T	TA	Chr21_13087T_TA	INDEL	17567.59	28	14	17	31	21	8	30	31	19	22
Chr21	13096184	GTA	G	Chr21_13096GTA_G	INDEL	3387.24	0	0	0	22	14	16	20	15	0	0
Chr21	13096184	GTA	G	Chr21_13096GTA_G	INDEL	3387.24	0	0	0	22	14	16	20	15	0	0
Chr21	13096184	GTA	G	Chr21_13096GTA_G	INDEL	3387.24	0	0	0	22	14	16	20	15	0	0
Chr21	13124792	T	TTA	Chr21_13124T_TTA	INDEL	6521.24	22	7	9	0	0	2	1	0	17	16
Chr21	13124792	T	TTA	Chr21_13124T_TTA	INDEL	6521.24	22	7	9	0	0	2	1	0	17	16
Chr21	13124792	T	TTA	Chr21_13124T_TTA	INDEL	6521.24	22	7	9	0	0	2	1	0	17	16
Chr21	13124792	T	TTA	Chr21_13124T_TTA	INDEL	6521.24	22	7	9	0	0	2	1	0	17	16
Chr21	13144095	A	G	Chr21_13144A_G	SNP	14046.63	16	9	9	14	12	22	37	21	13	16
Chr21	13212982	T	TA	Chr21_13212T_TA	INDEL	7003.59	15	10	8	10	12	26	16	20	8	18
Chr21	13212982	T	TA	Chr21_13212T_TA	INDEL	7003.59	15	10	8	10	12	26	16	20	8	18
Chr21	13212982	T	TA	Chr21_13212T_TA	INDEL	7003.59	15	10	8	10	12	26	16	20	8	18
Chr21	13212982	T	TA	Chr21_13212T_TA	INDEL	7003.59	15	10	8	10	12	26	16	20	8	18
Chr21	13212982	T	TA	Chr21_13212T_TA	INDEL	7003.59	15	10	8	10	12	26	16	20	8	18
Chr21	13239129	A	AAT	Chr21_13239A_AAT	INDEL	3193.24	0	0	0	17	6	22	26	16	0	0
Chr21	13239129	A	AAT	Chr21_13239A_AAT	INDEL	3193.24	0	0	0	17	6	22	26	16	0	0
Chr21	13343264	C	T	Chr21_13343C_T	SNP	5781.29	0	0	0	21	14	27	35	21	0	0
Chr21	13736862	C	CAT	Chr21_13736C_CAT	INDEL	4009.24	0	0	1	15	13	26	29	21	0	0
Chr21	13736862	C	CAT	Chr21_13736C_CAT	INDEL	4009.24	0	0	1	15	13	26	29	21	0	0
Chr21	13736862	C	CAT	Chr21_13736C_CAT	INDEL	4009.24	0	0	1	15	13	26	29	21	0	0
Chr21	13736862	C	CAT	Chr21_13736C_CAT	INDEL	4009.24	0	0	1	15	13	26	29	21	0	0
Chr21	13736862	C	CAT	Chr21_13736C_CAT	INDEL	4009.24	0	0	1	15	13	26	29	21	0	0
Chr21	13736862	C	CAT	Chr21_13736C_CAT	INDEL	4009.24	0	0	1	15	13	26	29	21	0	0
Chr21	13800530	CAT	C	Chr21_13800CAT_C	INDEL	2491.24	2	0	0	13	10	23	11	14	0	0
Chr21	13815072	TG	T	Chr21_13815TG_T	INDEL	11962.59	14	15	12	21	15	15	22	17	12	18
Chr21	13815072	TG	T	Chr21_13815TG_T	INDEL	11962.59	14	15	12	21	15	15	22	17	12	18
Chr21	13815072	TG	T	Chr21_13815TG_T	INDEL	11962.59	14	15	12	21	15	15	22	17	12	18
Chr21	13815072	TG	T	Chr21_13815TG_T	INDEL	11962.59	14	15	12	21	15	15	22	17	12	18
Chr21	13815072	TG	T	Chr21_13815TG_T	INDEL	11962.59	14	15	12	21	15	15	22	17	12	18
Chr21	13823244	G	GT	Chr21_13823G_GT	INDEL	17564.59	30	14	13	18	22	33	27	20	21	26
Chr21	13823244	G	GT	Chr21_13823G_GT	INDEL	17564.59	30	14	13	18	22	33	27	20	21	26
Chr21	13823244	G	GT	Chr21_13823G_GT	INDEL	17564.59	30	14	13	18	22	33	27	20	21	26
Chr21	13823244	G	GT	Chr21_13823G_GT	INDEL	17564.59	30	14	13	18	22	33	27	20	21	26
[illegible]	[illegible]	[illegible]	[illegible]	[illegible]	[illegible]	[illegible]	[illegible]	[illegible]	[illegible]	[illegible]	[illegible]	[illegible]	[illegible]	[illegible]	[illegible]	[illegible]

[illegible]

Chr22	1038555	C	CA	Chr22_10385C_CA	INDEL	11775.59	21	15	16	17	22	26	25	15	12	13	5	11	7	8
Chr22	1038555	C	CA	Chr22_10385C_CA	INDEL	11775.59	21	15	16	17	22	26	25	15	12	13	5	11	7	8
Chr22	1038555	C	CA	Chr22_10385C_CA	INDEL	11775.59	21	15	16	17	22	26	25	15	12	13	5	11	7	8
Chr22	1038555	C	CA	Chr22_10385C_CA	INDEL	11775.59	21	15	16	17	22	26	25	15	12	13	5	11	7	8
Chr22	1149530	C	CA	Chr22_11495C_CA	INDEL	18644.59	33	14	22	32	22	30	35	22	22	23	16	19	9	18
Chr22	1360707	CG	C	Chr22_13607CG_C	INDEL	16482.59	35	20	30	28	21	22	30	17	25	14	16	23	8	14
Chr22	1360707	CG	C	Chr22_13607CG_C	INDEL	16482.59	35	20	30	28	21	22	30	17	25	14	16	23	8	14
Chr22	1360707	CG	C	Chr22_13607CG_C	INDEL	16482.59	35	20	30	28	21	22	30	17	25	14	16	23	8	14
Chr22	1360707	CG	C	Chr22_13607CG_C	INDEL	16482.59	35	20	30	28	21	22	30	17	25	14	16	23	8	14
Chr22	1477355	TTA	T	Chr22_14773TTA_T	INDEL	9258.59	27	16	12	18	11	20	23	20	11	12	10	9	6	6
Chr22	1581157	GT	G	Chr22_15811GT_G	INDEL	15097.59	23	12	17	13	18	14	23	17	18	15	6	19	5	6
Chr22	1581157	GT	G	Chr22_15811GT_G	INDEL	15097.59	23	12	17	13	18	14	23	17	18	15	6	19	5	6
Chr22	1581166	T	TA	Chr22_15811T_TA	INDEL	16448.59	27	14	19	13	19	14	23	17	18	17	7	23	5	6
Chr22	1581166	T	TA	Chr22_15811T_TA	INDEL	16448.59	27	14	19	13	19	14	23	17	18	17	7	23	5	6
Chr22	1581210	C	A	Chr22_15812C_A	SNP	17075.63	23	11	14	17	18	17	23	23	21	21	6	14	8	4
Chr22	1581210	C	A	Chr22_15812C_A	SNP	17075.63	23	11	14	17	18	17	23	23	21	21	6	14	8	4
Chr22	1581212	CT	C	Chr22_15812CT_C	INDEL	16886.59	23	11	14	18	20	17	25	23	20	22	7	14	9	5
Chr22	1581212	CT	C	Chr22_15812CT_C	INDEL	16886.59	23	11	14	18	20	17	25	23	20	22	7	14	9	5
Chr22	1581214	AGAAAAC	A	Chr22_15812AGAAAAC_A	INDEL	16976.59	22	11	14	18	20	17	25	23	20	23	7	14	9	5
Chr22	1581214	AGAAAAC	A	Chr22_15812AGAAAAC_A	INDEL	16976.59	22	11	14	18	20	17	25	23	20	23	7	14	9	5
Chr22	1581223	A	ATTTATTGA1	Chr22_15812A_ATTTATTG	INDEL	18038.59	23	10	13	17	20	16	26	23	20	25	7	13	10	5
Chr22	1581223	A	ATTTATTGA1	Chr22_15812A_ATTTATTG	INDEL	18038.59	23	10	13	17	20	16	26	23	20	25	7	13	10	5
Chr22	1581223	A	ATTTATTGA1	Chr22_15812A_ATTTATTG	INDEL	18038.59	23	10	13	17	20	16	26	23	20	25	7	13	10	5
Chr22	1651876	T	G	Chr22_16518T_G	SNP	16881.63	22	12	8	24	15	21	32	11	13	21	22	10	8	12
Chr22	1651876	T	G	Chr22_16518T_G	SNP	16881.63	22	12	8	24	15	21	32	11	13	21	22	10	8	12
Chr22	1651876	T	G	Chr22_16518T_G	SNP	16881.63	22	12	8	24	15	21	32	11	13	21	22	10	8	12
Chr22	1651876	T	G	Chr22_16518T_G	SNP	16881.63	22	12	8	24	15	21	32	11	13	21	22	10	8	12
Chr22	1651876	T	G	Chr22_16518T_G	SNP	16881.63	22	12	8	24	15	21	32	11	13	21	22	10	8	12
Chr22	1651876	T	G	Chr22_16518T_G	SNP	16881.63	22	12	8	24	15	21	32	11	13	21	22	10	8	12
Chr22	1725602	C	CA	Chr22_17256C_CA	INDEL	20551.59	19	20	20	27	21	24	31	27	18	19	20	29	9	6
Chr22	1725623	A	AC	Chr22_17256A_AC	INDEL	21289.59	22	16	20	26	21	24	36	23	19	19	16	30	10	7
Chr22	1866457	G	T	Chr22_18664G_T	SNP	18340.63	29	14	16	32	19	23	31	21	15	25	10	13	9	6
Chr22	1881208	A	AAT	Chr22_18812A_AAT	INDEL	2624.93	0	0	0	0	0	0	0	0	20	11	16	11	8	9
Chr22	1900287	C	A	Chr22_19002C_A	SNP	15211.63	25	12	10	25	17	24	33	23	14	18	12	14	7	7
Chr22	1900380	G	T	Chr22_19003G_T	SNP	18786.63	37	11	13	31	18	27	31	31	13	14	14	15	9	11
Chr22	1900413	G	T	Chr22_19004G_T	SNP	19045.63	35	10	15	29	21	26	31	32	12	17	13	17	7	10
Chr22	1900459	C	CA	Chr22_19004C_CA	INDEL	14778.59	28	6	13	23	18	25	29	33	8	21	11	17	8	13
Chr22	1901253	C	T	Chr22_19012C_T	SNP	13211.63	18	11	7	20	17	12	10	12	17	17	7	21	9	14
Chr22	1901268	TC	T	Chr22_19012TC_T	INDEL	13900.59	15	9	7	21	13	10	12	12	16	14	8	20	9	14
Chr22	1901275	A	AT	Chr22_19012A_AT	INDEL	13736.59	13	9	8	23	14	12	12	12	15	13	8	17	9	15
Chr22	1901306	A	T	Chr22_19013A_T	SNP	12550.63	7	5	8	21	12	10	12	13	21	11	11	14	9	11
Chr22	1931541	AT	A	Chr22_19315AT_A	INDEL	17257.59	28	17	16	28	28	25	28	24	26	17	16	31	7	9
Chr22	1931664	T	C	Chr22_19316T_C	SNP	19580.63	11	24	12	27	18	26	21	24	30	14	20	12	9	11
Chr22	1931665	C	A	Chr22_19316C_A	SNP	19580.63	11	25	12	27	18	26	21	24	30	14	20	12	9	11
Chr22	1973319	A	G	Chr22_19733A_G	SNP	16326.63	28	15	14	24	21	20	24	20	18	17	16	30	8	9
Chr22	1975764	A	AAT	Chr22_19757A_AAT	INDEL	1807.93	0	1	1	0	0	2	1	0	14	13	8	8	11	7
Chr22	2225405	G	A	Chr22_22254G_A	SNP	11017.29	30	16	16	1	0	0	0	0	19	30	18	25	11	6
Chr22	2755804	A	AAC	Chr22_27558A_AAC	INDEL	17054.59	12	15	10	21	22	24	26	21	19	15	15	11	5	7
Chr22	2755804	A	AAC	Chr22_27558A_AAC	INDEL	17054.59	12	15	10	21	22	24	26	21	19	15	15	11	5	7
Chr22	2755804	A	AAC	Chr22_27558A_AAC	INDEL	17054.59	12	15	10	21	22	24	26	21	19	15	15	11	5	7
Chr22	2755804	A	AAC	Chr22_27558A_AAC	INDEL	17054.59	12	15	10	21	22	24	26	21	19	15	15	11	5	7
Chr22	2755804	A	AAC	Chr22_27558A_AAC	INDEL	17054.59	12	15	10	21	22	24	26	21	19	15	15	11	5	7
Chr22	2755804	A	AAC	Chr22_27558A_AAC	INDEL	17054.59	12	15	10	21	22	24	26	21	19	15	15	11	5	7
Chr22	2755806	A	AAGC	Chr22_27558A_AAGC	INDEL	17594.59	12	15	10	21	23	24	26	21	20	14	15	11	5	7
Chr22	2755806	A	AAGC	Chr22_27558A_AAGC	INDEL	17594.59	12	15	10	21	23	24	26	21	20	14	15	11	5	7
Chr22	2755806	A	AAGC	Chr22_27558A_AAGC	INDEL	17594.59	12	15	10	21	23	24	26	21	20	14	15	11	5	7
Chr22	2755806	A	AAGC	Chr22_27558A_AAGC	INDEL	17594.59	12	15	10	21	23	24	26	21	20	14	15	11	5	7
Chr22	2755806	A	AAGC	Chr22_27558A_AAGC	INDEL	17594.59	12	15	10	21	23	24	26	21	20	14	15	11	5	7
Chr22	2755806	A	AAGC	Chr22_27558A_AAGC	INDEL	17594.59	12	15	10	21	23	24	26	21	20	14	15	11	5	7
Chr22	2755808	T	TA	Chr22_27558T_TA	INDEL	18236.59	15	15	11	19	24	24	29	20	20	13	15	11	5	7
Chr22	2755808	T	TA	Chr22_27558T_TA	INDEL	18236.59	15	15	11	19	24	24	29	20	20	13	15	11	5	7
Chr22	2755808	T	TA	Chr22_27558T_TA	INDEL	18236.59	15	15	11	19	24	24	29	20	20	13	15	11	5	7
Chr22	2755808	T	TA	Chr22_27558T_TA	INDEL	18236.59	15	15	11	19	24	24	29	20	20	13	15	11	5	7
Chr22	2755808	T	TA	Chr22_27558T_TA	INDEL	18236.59	15	15	11	19	24	24	29	20	20	13	15	11	5	7
Chr22	2755808	T	TA	Chr22_27558T_TA	INDEL	18236.59	15	15	11	19	24	24	29	20	20	13	15	11	5	7
Chr22	2755809	G	GCT	Chr22_27558G_GCT	INDEL	18011.59	14	15	11	18	24	24	30	21	20	14	15	11	5	7
Chr22	2755809	G	GCT	Chr22_27558G_GCT	INDEL	18011.59	14	15	11	18	24	24	30	21	20	14	15	11	5	7
Chr22	2755809	G	GCT	Chr22_27558G_GCT	INDEL	18011.59	14	15	11	18	24	24	30	21	20	14	15	11	5	7
Chr22	2755809	G	GCT	Chr22_27558G_GCT	INDEL	18011.59	14	15	11	18	24	24	30	21	20	14	15	11	5	7
Chr22	2755809	G	GCT	Chr22_27558G_GCT	INDEL	18011.59	14	15	11	18	24	24	30	21	20	14	15	11	5	7
Chr22	2755809	G	GCT	Chr22_27558G_GCT	INDEL	18011.59	14	15	11	18	24	24	30	21	20	14	15	11	5	7
Chr22	2755811	T	TGG	Chr22_27558T_TGG	INDEL	18236.59	14	15	12	17	23	24	30	22	21	15	15	10	5	7
Chr22	2755811	T	TGG	Chr22_27558T_TGG	INDEL	18236.59	14	15	12	17	23	24	30	22	21	15	15	10	5	7
Chr22	2755811	T	TGG	Chr22_27558T_TGG	INDEL	18236.59	14	15	12	17	23	24	30	22	21	15	15	10	5	7
Chr22	2755811	T	TGG	Chr22_27558T_TGG	INDEL	18236.59	14	15	12	17	23	24	30	22	21	15	15	10	5	7
Chr22	2755811	T	TGG	Chr22_27558T_TGG	INDEL	18236.59	14	15	12	17	23	24	30	22	21	15	15	10	5	7
Chr22	2755811	T	TGG	Chr22_27558T_TGG	INDEL	18236.59	14	15	12	17	23	24	30	22	21	15	15	10	5	7
Chr22	2755815	A	C	Chr22_27558A_C	SNP	17390.63	15	16	11	16	22	24	28	23	21	14	14	12	5	7
Chr22	2755815	A	C	Chr22_27558A_C	SNP	17390.63	15	16	11	16	22	24	28	23	21	14	14	12	5	7
Chr22	2755815	A	C	Chr22_27558A_C	SNP	17390.63	15	16	11	16	22	24	28	23	21	14	14	12	5	7
Chr22	2755815	A	C	Chr22_27558A_C	SNP	17390.63	15	16	11	16	22	24	28	23	21	14	14	12	5	7
Chr22	2755815	A	C	Chr22_27558A_C	SNP	17390.63	15	16	11	16	22	24	28	23	21	14	14	12	5	7
Chr22	2755815	A	C	Chr22_27558A_C	SNP	17390.63	15	16	11	16	22	24	28	23	21	14	14	12	5	7
Chr22	2755817	GGGTTTTTA'	G	Chr22_27558GGGTTTTTA'	INDEL	17246.59	15	16	11	16	22	24	27	23	21	14	15	12	5	6
Chr22	2755817	GGGTTTTTA'	G	Chr22_27558GGGTTTTTA'	INDEL	17246.59	15	16	11	16	22	24	27	23	21	14	15	12	5	6
Chr22	2755817	GGGTTTTTA'	G	Chr22_27558GGGTTTTTA'	INDEL	17246.59	15	16	11	16	22	24	27	23	21	14	15	12	5	6
Chr22	2755817	GGGTTTTTA'	G	Chr22_27558GGGTTTTTA'	INDEL	17246.59	15	16	11	16	22	24	27	23	21	14	15	12	5	6
Chr22	2755817	GGGTTTTTA'	G	Chr22_27558GGGTTTTTA'	INDEL	17246.59	15	16	11	16	22	24	27	23	21	14	15	12	5	6
Chr22	2755817	GGGTTTTTA'	G	Chr22_27558GGGTTTTTA'	INDEL	17246.59	15	16	11	16	22	24	27	23	21	14	15	12	5	6
Chr22	2755841	T	TGCACAAA	Chr22_27558T_TGCACAA/	INDEL	17741.59	15	15	11	15	21	25	27	23	20	15	15	11	5	6
Chr22	2755841	T	TGCACAAA	Chr22_27558T_TGCACAA/	INDEL	17741.59	15	15	11	15	21	25	27	23	20	15	15	11	5	6
Chr22	2755841	T	TGCACAAA	Chr22_27558T_TGCACAA/	INDEL	17741.59	15	15	11	15	21	25	27	23	20	15	15	11	5	6
Chr22	2755841	T	TGCACAAA	Chr22_27558T_TGCACAA/	INDEL	17741.59	15	15	11	15	21	25	27	23	20	15	15	11	5	6
Chr22	2755841	T	TGCACAAA	Chr22_27558T_TGCACAA/	INDEL	17741.59	15	15	11	15	21	25	27	23	20	15	15	11	5	6
Chr22	2755841	T	TGCACAAA	Chr22_27558T_TGCACAA/	INDEL	17741.59	15	15	11	15	21	25	27	23	20	15	15	11	5	6
Chr22	2755843	T	TCAAACGA	Chr22_27558T_TCAAACG/	INDEL	18281.59	15	16	12	13	23	25	26	23	20	16	14	10	5	7
Chr22	2755843	T	TCAAACGA	Chr22_27558T_TCAAACG/	INDEL	18281.59	15	16	12	13	23	25	26	23	20	16	14	10	5	7
Chr22	2755843	T	TCAAACGA	Chr22_27558T_TCAAACG/	INDEL	18281.59	15	16	12	13	23	25	26	23	20	16	14	10	5	7
Chr22	2755843	T	TCAAACGA	Chr22_27558T_TCAAACG/	INDEL	18281.59	15	16	12	13	23	25	26	23	20	16	14	10	5	7
Chr22	2755843	T	TCAAACGA	Chr22_27558T_TCAAACG/	INDEL	18281.59	15	16	12	13	23	25	26	23	20	16	14	10	5	7
Chr22	2755843	T	TCAAACGA	Chr22_27558T_TCAAACG/	INDEL	18281.59	15	16	12	13	23	25	26	23	20	16	14	10	5	7
Chr22	2755845	T	C	Chr22_27558T_C	SNP	17390.63	16	16	11	11	22	23	25	24	18	16	14	11	4	8
Chr22	2755845	T	C	Chr22_27558T_C	SNP	17390.63	16	16	11	11	22	23	25	24	18	16	14	11	4	8
Chr22	2755845	T	C	Chr22_27558T_C	SNP	17390.63	16	16	11	11	22	23	25	24	18	16	14	11	4	8
Chr22	2755845	T	C	Chr22_27558T_C	SNP	17390.63	16	16	11	11	22	23	25	24	18	16	14	11	4	8
Chr22	2755845	T	C	Chr22_27558T_C	SNP	17390.63	16	16	11	11	22	23	25	24	18	16	14	11	4	8
Chr22	2755845	T	C	Chr22_27558T_C	SNP	17390.63	16	16	11	11	22	23	25	24	18	16	14	11	4	8
Chr22	2755848	C	CACTG	Chr22_27558C_CACTG	INDEL	16616.59	17	16	11	11	23	23	25	22	18	16	13	12	4	8
Chr22	2755848	C	CACTG	Chr22_27558C_CACTG	INDEL	16616.59	17	16	11	11	23	23	25	22	18	16	13	12	4	8
Chr22	2755848	C	CACTG	Chr22_27558C_CACTG	INDEL	16616.59	17	16	11	11	23	23	25	22	18	16	13	12	4	8
Chr22	2755848	C	CACTG	Chr22_27558C_CACTG	INDEL	16616.59	17	16	11	11	23	23	25	22	18	16	13	12	4	8
Chr22	2755848	C	CACTG	Chr22_27558C_CACTG	INDEL	16616.59	17	16	11	11	23	23	25	22	18	16	13	12	4	8
Chr22	2755848	C	CACTG	Chr22_27558C_CACTG	INDEL	16616.59	17	16	11	11	23	23	25	22	18	16	13	12	4	8
Chr22	2755850	T	TA	Chr22_27558T_TA	INDEL	16616.59	17	15	11	11	23	25	25	21	17	14	11	12	4	7
Chr22	2755850	T	TA	Chr22_27558T_TA	INDEL	16616.59	17	15	11	11	23	25	25	21	17	14	11	12	4	7
Chr22	2755850	T	TA	Chr22_27558T_TA	INDEL	16616.59	17	15	11	11	23	25	25	21	17	14	11	12	4	7
Chr22	2755850	T	TA	Chr22_27558T_TA	INDEL	16616.59	17	15	11	11	23	25	25	21	17	14	11	12	4	7
Chr22	2755850	T	TA	Chr22_27558T_TA	INDEL	16616.59	17	15	11	11	23	25	25	21	17	14	11	12	4	7
Chr22	2755850	T	TA	Chr22_27558T_TA	INDEL	16616.59	17	15	11	11	23	25	25	21	17	14	11	12	4	7
Chr22	2755855	C	CGTCTA	Chr22_27558C_CGTCTA	INDEL	15176.59	16	14	10	10	22	24	26	20	16	14	9	10	4	6
Chr22	2755855	C	CGTCTA	Chr22_27558C_CGTCTA	INDEL	15176.59	16	14	10	10	22	24	26	20	16	14	9	10	4	6
Chr22	2755855	C	CGTCTA	Chr22_27558C_CGTCTA	INDEL	15176.59	16	14	10	10	22	24	26	20	16	14	9	10	4	6
Chr22	2755855	C	CGTCTA	Chr22_27558C_CGTCTA	INDEL	15176.59	16	14	10	10	22	24	26	20	16	14	9	10	4	6
Chr22	2755855	C	CGTCTA	Chr22_27558C_CGTCTA	INDEL	15176.59	16	14	10	10	22	24	26	20	16	14	9	10	4	6
Chr22	2755855	C	CGTCTA	Chr22_27558C_CGTCTA	INDEL	15176.59	16	14	10	10	22	24	26	20	16	14	9	10	4	6
Chr22	2755863	CATTGTGCC1	C	Chr22_27558CATTGTGCC1	INDEL	14276.59	15	14	9	11	25	22	21	19	14	13	10	9	5	6
Chr22	2755863	CATTGTGCC1	C	Chr22_27558CATTGTGCC1	INDEL	14276.59	15	14	9	11	25	22	21	19	14	13	10	9	5	6
Chr22	2755863	CATTGTGCC1	C	Chr22_27558CATTGTGCC1	INDEL	14276.59	15	14	9	11	25	22	21	19	14	13	10	9	5	6
Chr22	2755863	CATTGTGCC1	C	Chr22_27558CATTGTGCC1	INDEL	14276.59	15	14	9	11	25	22	21	19	14	13	10	9	5	6
Chr22	2755863	CATTGTGCC1	C	Chr22_27558CATTGTGCC1	INDEL	14276.59	15	14	9	11	25	22	21	19	14	13	10	9	5	6
Chr22	2755863	CATTGTGCC1	C	Chr22_27558CATTGTGCC1	INDEL	14276.59	15	14	9	11	25	22	21	19	14	13	10	9	5	6
Chr22	2755878	G	A	Chr22_27558G_A	SNP	13970.63	16	13	9	13	24	21	21	19	14	12	10	10	5	6
Chr22	2755878	G	A	Chr22_27558G_A	SNP	13970.63	16	13	9	13	24	21	21	19	14	12	10	10	5	6
Chr22	2755878	G	A	Chr22_27558G_A	SNP	13970.63	16	13	9	13	24	21	21	19	14	12	10	10	5	6
Chr22	2755878	G	A	Chr22_27558G_A	SNP	13970.63	16	13	9	13	24	21	21	19	14	12	10	10	5	6
Chr22	2755878	G	A	Chr22_27558G_A	SNP	13970.63	16	13	9	13	24	21	21	19	14	12	10	10	5	6
Chr22	2755878	G	A	Chr22_27558G_A	SNP	13970.63	16	13	9	13	24	21	21	19	14	12	10	10	5	6
Chr22	2755879	CTT	C	Chr22_27558CTT_C	INDEL	14096.59	16	13	8	13	23	21	21	19	14	12	10	10	5	6
Chr22	2755879	CTT	C	Chr22_27558CTT_C	INDEL	14096.59	16	13	8	13	23	21	21	19	14	12	10	10	5	6
Chr22	2755879	CTT	C	Chr22_27558CTT_C	INDEL	14096.59	16	13	8	13	23	21	21	19	14	12	10	10	5	6
Chr22	2755879	CTT	C	Chr22_27558CTT_C	INDEL	14096.59	16	13	8	13	23	21	21	19	14	12	10	10	5	6
Chr22	2755879	CTT	C	Chr22_27558CTT_C	INDEL	14096.59	16	13	8	13	23	21	21	19	14	12	10	10	5	6
Chr22	2755879	CTT	C	Chr22_27558CTT_C	INDEL	14096.59	16	13	8	13	23	21	21	19	14	12	10	10	5	6
Chr22	2755882	GTTT	G	Chr22_27558GTTT_G	INDEL	14051.59	16	13	8	13	23	21	21	20	14	13	9	10	5	6
Chr22	2755882	GTTT	G	Chr22_27558GTTT_G	INDEL	14051.59	16	13	8	13	23	21	21	20	14	13	9	10	5	6
Chr22	2755882	GTTT	G	Chr22_27558GTTT_G	INDEL	14051.59	16	13	8	13	23	21	21	20	14	13	9	10	5	6
Chr22	2755882	GTTT	G	Chr22_27558GTTT_G	INDEL	14051.59	16	13	8	13	23	21	21	20	14	13	9	10	5	6
Chr22	2755882	GTTT	G	Chr22_27558GTTT_G	INDEL	14051.59	16	13	8	13	23	21	21	20	14	13	9	10	5	6
Chr22	2755882	GTTT	G	Chr22_27558GTTT_G	INDEL	14051.59	16	13	8	13	23	21	21	20	14	13	9	10	5	6
Chr22	2755888	G	A	Chr22_27558G_A	SNP	14015.63	15	15	8	14	23	21	21	19	14	13	9	10	4	6
Chr22	2755888	G	A	Chr22_27558G_A	SNP	14015.63	15	15	8	14	23	21	21	19	14	13	9	10	4	6
Chr22	2755888	G	A	Chr22_27558G_A	SNP	14015.63	15	15	8	14	23	21	21	19	14	13	9	10	4	6
Chr22	2755888	G	A	Chr22_27558G_A	SNP	14015.63	15	15	8	14	23	21	21	19	14	13	9	10	4	6
Chr22	2755888	G	A	Chr22_27558G_A	SNP	14015.63	15	15	8	14	23	21	21	19	14	13	9	10	4	6
Chr22	2755888	G	A	Chr22_27558G_A	SNP	14015.63	15	15	8	14	23	21	21	19	14	13	9	10	4	6
Chr22	2755889	ATT	A	Chr22_27558ATT_A	INDEL	14186.59	15	15	8	13	23	21	21	19	14	13	9	10	4	6
Chr22	2755889	ATT	A	Chr22_27558ATT_A	INDEL	14186.59	15	15	8	13	23	21	21	19	14	13	9	10	4	6
Chr22	2755889	ATT	A	Chr22_27558ATT_A	INDEL	14186.59	15	15	8	13	23	21	21	19	14	13	9	10	4	6
Chr22	2755889	ATT	A	Chr22_27558ATT_A	INDEL	14186.59	15	15	8	13	23	21	21	19	14	13	9	10	4	6
Chr22	2755889	ATT	A	Chr22_27558ATT_A	INDEL	14186.59	15	15	8	13	23	21	21	19	14	13	9	10	4	6
Chr22	2755889	ATT	A	Chr22_27558ATT_A	INDEL	14186.59	15	15	8	13	23	21	21	19	14	13	9	10	4	6
Chr22	2755892	G	A	Chr22_27558G_A	SNP	14215.63	16	14	8	12	23	21	22	19	14	13	9	10	4	6
Chr22	2755892	G	A	Chr22_27558G_A	SNP	14215.63	16	14	8	12	23	21	22	19	14	13	9	10	4	6
Chr22	2755892	G	A	Chr22_27558G_A	SNP	14215.63	16	14	8	12	23	21	22	19	14	13	9	10	4	6
Chr22	2755892	G	A	Chr22_27558G_A	SNP	14215.63	16	14	8	12	23	21	22	19	14	13	9	10	4	6
Chr22	2755892	G	A	Chr22_27558G_A	SNP	14215.63	16	14	8	12	23	21	22	19	14	13	9	10	4	6
Chr22	2755892	G	A	Chr22_27558G_A	SNP	14215.63	16	14	8	12	23	21	22	19	14	13	9	10	4	6
Chr22	2755893	T	TCCCC	Chr22_27558T_TCCCC	INDEL	14206.59	16	14	8	12	23	21	23	19	14	13	8	10	4	6
Chr22	2755893	T	TCCCC	Chr22_27558T_TCCCC	INDEL	14206.59	16	14	8	12	23	21	23	19	14	13	8	10	4	6
Chr22	2755893	T	TCCCC	Chr22_27558T_TCCCC	INDEL	14206.59	16	14	8	12	23	21	23	19	14	13	8	10	4	6
Chr22	2755893	T	TCCCC	Chr22_27558T_TCCCC	INDEL	14206.59	16	14	8	12	23	21	23	19	14	13	8	10	4	6
Chr22	2755893	T	TCCCC	Chr22_27558T_TCCCC	INDEL	14206.59	16	14	8	12	23	21	23	19	14	13	8	10	4	6
Chr22	2755893	T	TCCCC	Chr22_27558T_TCCCC	INDEL	14206.59	16	14	8	12	23	21	23	19	14	13	8	10	4	6
Chr22	2759137	AAT	A	Chr22_27591AAT_A	INDEL	2436.24	0	0	0	12	18	19	16	11	0	0	0	0	1	0
Chr22	2759137	AAT	A	Chr22_27591AAT_A	INDEL	2436.24	0	0	0	12	18	19	16	11	0	0	0	0	1	0
Chr22	2759137	AAT	A	Chr22_27591AAT_A	INDEL	2436.24	0	0	0	12	18	19	16	11	0	0	0	0	1	0
Chr22	2759137	AAT	A	Chr22_27591AAT_A	INDEL	2436.24	0	0	0	12	18	19	16	11	0	0	0	0	1	0
Chr22	2759137	AAT	A	Chr22_27591AAT_A	INDEL	2436.24	0	0	0	12	18	19	16	11	0	0	0	0	1	0
Chr22	2759137	AAT	A	Chr22_27591AAT_A	INDEL	2436.24	0	0	0	12	18	19	16	11	0	0	0	0	1	0
Chr22	2759137	AAT	A	Chr22_27591AAT_A	INDEL	2436.24	0	0	0	12	18	19	16	11	0	0	0	0	1	0
Chr22	2817674	C	A	Chr22_28176C_A	SNP	12330.63	24	9	13	12	6	14	17	16	21	11	7	10	4	7
Chr22	2817674	C	A	Chr22_28176C_A	SNP	12330.63	24	9	13	12	6	14	17	16	21	11	7	10	4	7
Chr22	2817674	C	A	Chr22_28176C_A	SNP	12330.63	24	9	13	12	6	14	17	16	21	11	7	10	4	7
Chr22	2817674	C	A	Chr22_28176C_A	SNP	12330.63	24	9	13	12	6	14	17	16	21	11	7	10	4	7
Chr22	2817674	C	A	Chr22_28176C_A	SNP	12330.63	24	9	13	12	6	14	17	16	21	11	7	10	4	7
Chr22	2817674	C	A	Chr22_28176C_A	SNP	12330.63	24	9	13	12	6	14	17	16	21	11	7	10	4	7
Chr22	2817674	C	A	Chr22_28176C_A	SNP	12330.63	24	9	13	12	6	14	17	16	21	11	7	10	4	7
Chr22	2817674	C	A	Chr22_28176C_A	SNP	12330.63	24	9	13	12	6	14	17	16	21	11	7	10	4	7
Chr22	2817674	C	A	Chr22_28176C_A	SNP	12330.63	24	9	13	12	6	14	17	16	21	11	7	10	4	7
Chr22	2817674	C	A	Chr22_28176C_A	SNP	12330.63	24	9	13	12	6	14	17	16	21	11	7	10	4	7
Chr22	2817675	TTTCC	T	Chr22_28176TTTCC_T	INDEL	12366.59	24	9	14	11	6	14	18	16	21	11	7	10	4	7
Chr22	2817675	TTTCC	T	Chr22_28176TTTCC_T	INDEL	12366.59	24	9	14	11	6	14	18	16	21	11	7	10	4	7
Chr22	2817675	TTTCC	T	Chr22_28176TTTCC_T	INDEL	12366.59	24	9	14	11	6	14	18	16	21	11	7	10	4	7
Chr22	2817675	TTTCC	T	Chr22_28176TTTCC_T	INDEL	12366.59	24	9	14	11	6	14	18	16	21	11	7	10	4	7
Chr22	2817675	TTTCC	T	Chr22_28176TTTCC_T	INDEL	12366.59	24	9	14	11	6	14	18	16	21	11	7	10	4	7
Chr22	2817675	TTTCC	T	Chr22_28176TTTCC_T	INDEL	12366.59	24	9	14	11	6	14	18	16	21	11	7	10	4	7
Chr22	2817675	TTTCC	T	Chr22_28176TTTCC_T	INDEL	12366.59	24	9	14	11	6	14	18	16	21	11	7	10	4	7
Chr22	2817675	TTTCC	T	Chr22_28176TTTCC_T	INDEL	12366.59	24	9	14	11	6	14	18	16	21	11	7	10	4	7
Chr22	2817675	TTTCC	T	Chr22_28176TTTCC_T	INDEL	12366.59	24	9	14	11	6	14	18	16	21	11	7	10	4	7
Chr22	2817675	TTTCC	T	Chr22_28176TTTCC_T	INDEL	12366.59	24	9	14	11	6	14	18	16	21	11	7	10	4	7
Chr22	2817680	AG	A	Chr22_28176AG_A	INDEL	12431.59	24	9	14	11	6	14	18	16	20	11	6	10	4	7
Chr22	2817680	AG	A	Chr22_28176AG_A	INDEL	12431.59	24	9	14	11	6	14	18	16	20	11	6	10	4	7

Chr22	2817680	AG	A	Chr22_28176 AG_A	INDEL	12431.59	24	9	14	11	6	14	18	16	20	11	6	10	4	7	[illegible]
Chr22	2817680	AG	A	Chr22_28176 AG_A	INDEL	12431.59	24	9	14	11	6	14	18	16	20	11	6	10	4	7	[illegible]
Chr22	2817680	AG	A	Chr22_28176 AG_A	INDEL	12431.59	24	9	14	11	6	14	18	16	20	11	6	10	4	7	[illegible]
Chr22	2817680	AG	A	Chr22_28176 AG_A	INDEL	12431.59	24	9	14	11	6	14	18	16	20	11	6	10	4	7	[illegible]
Chr22	2817680	AG	A	Chr22_28176 AG_A	INDEL	12431.59	24	9	14	11	6	14	18	16	20	11	6	10	4	7	[illegible]
Chr22	2817680	AG	A	Chr22_28176 AG_A	INDEL	12431.59	24	9	14	11	6	14	18	16	20	11	6	10	4	7	[illegible]
Chr22	2817680	AG	A	Chr22_28176 AG_A	INDEL	12431.59	24	9	14	11	6	14	18	16	20	11	6	10	4	7	[illegible]
Chr22	2817680	AG	A	Chr22_28176 AG_A	INDEL	12431.59	24	9	14	11	6	14	18	16	20	11	6	10	4	7	[illegible]
Chr22	2817684	C	A	Chr22_28176 C_A	SNP	12755.63	23	8	12	12	6	14	18	16	20	11	6	10	4	7	[illegible]
Chr22	2817684	C	A	Chr22_28176 C_A	SNP	12755.63	23	8	12	12	6	14	18	16	20	11	6	10	4	7	[illegible]
Chr22	2817684	C	A	Chr22_28176 C_A	SNP	12755.63	23	8	12	12	6	14	18	16	20	11	6	10	4	7	[illegible]
Chr22	2817684	C	A	Chr22_28176 C_A	SNP	12755.63	23	8	12	12	6	14	18	16	20	11	6	10	4	7	[illegible]
Chr22	2817684	C	A	Chr22_28176 C_A	SNP	12755.63	23	8	12	12	6	14	18	16	20	11	6	10	4	7	[illegible]
Chr22	2817684	C	A	Chr22_28176 C_A	SNP	12755.63	23	8	12	12	6	14	18	16	20	11	6	10	4	7	[illegible]
Chr22	2817684	C	A	Chr22_28176 C_A	SNP	12755.63	23	8	12	12	6	14	18	16	20	11	6	10	4	7	[illegible]
Chr22	2817684	C	A	Chr22_28176 C_A	SNP	12755.63	23	8	12	12	6	14	18	16	20	11	6	10	4	7	[illegible]
Chr22	2817684	C	A	Chr22_28176 C_A	SNP	12755.63	23	8	12	12	6	14	18	16	20	11	6	10	4	7	[illegible]
Chr22	2817684	C	A	Chr22_28176 C_A	SNP	12755.63	23	8	12	12	6	14	18	16	20	11	6	10	4	7	[illegible]
Chr22	2817686	TTGG	T	Chr22_28176 TTGG_T	INDEL	12701.59	24	8	12	12	7	14	19	15	20	11	6	10	4	7	[illegible]
Chr22	2817686	TTGG	T	Chr22_28176 TTGG_T	INDEL	12701.59	24	8	12	12	7	14	19	15	20	11	6	10	4	7	[illegible]
Chr22	2817686	TTGG	T	Chr22_28176 TTGG_T	INDEL	12701.59	24	8	12	12	7	14	19	15	20	11	6	10	4	7	[illegible]
Chr22	2817686	TTGG	T	Chr22_28176 TTGG_T	INDEL	12701.59	24	8	12	12	7	14	19	15	20	11	6	10	4	7	[illegible]
Chr22	2817686	TTGG	T	Chr22_28176 TTGG_T	INDEL	12701.59	24	8	12	12	7	14	19	15	20	11	6	10	4	7	[illegible]
Chr22	2817686	TTGG	T	Chr22_28176 TTGG_T	INDEL	12701.59	24	8	12	12	7	14	19	15	20	11	6	10	4	7	[illegible]
[illegible]	[illegible]	[illegible]	[illegible]	[illegible]	[illegible]	[illegible]	[illegible]	[illegible]	[illegible]	[illegible]	[illegible]	[illegible]	[illegible]	[illegible]	[illegible]	[illegible]	[illegible]	[illegible]	[illegible]	[illegible]	[illegible]

Chr22	8370451	T	TTA	Chr22_83704	T_TTA	INDEL	5834.59	17	7	9	7	9	10
Chr22	8482724	C	A	Chr22_84827	C_A	SNP	10590.63	16	14	8	10	14	26
Chr22	8482954	TC	T	Chr22_84829	TC_T	INDEL	11523.59	24	9	10	13	16	16
Chr22	8488537	C	A	Chr22_84885	C_A	SNP	15201.63	21	7	13	21	13	19
Chr22	8488597	A	AT	Chr22_84885	A_AT	INDEL	16802.59	22	9	11	26	15	24
Chr22	8488622	G	T	Chr22_84886	G_T	SNP	18954.63	27	9	11	27	17	27
Chr22	8488634	G	A	Chr22_84886	G_A	SNP	18783.63	24	8	13	26	20	29
Chr22	8488636	G	T	Chr22_84886	G_T	SNP	18378.63	23	8	13	25	20	31
Chr22	8488663	G	A	Chr22_84886	G_A	SNP	17405.63	23	10	18	21	20	22
Chr22	8780655	T	A	Chr22_87806	T_A	SNP	1073.39	13	7	7	0	0	0
Chr22	8877358	A	AT	Chr22_88773	A_AT	INDEL	16370.59	24	15	15	25	23	24
Chr22	8904550	G	C	Chr22_89045	G_C	SNP	21569.63	37	21	14	24	24	31
Chr22	8920519	G	A	Chr22_89205	G_A	SNP	5171.29	0	0	0	21	19	13
Chr22	8972312	G	T	Chr22_89723	G_T	SNP	17504.63	18	4	17	17	14	23
Chr22	9146235	G	GA	Chr22_91462	G_GA	INDEL	15645.59	23	7	21	30	20	30
Chr22	9146235	G	GA	Chr22_91462	G_GA	INDEL	15645.59	23	7	21	30	20	30
Chr22	9146235	G	GA	Chr22_91462	G_GA	INDEL	15645.59	23	7	21	30	20	30
Chr22	9546692	AATAT	A	Chr22_95466	AATAT_A	INDEL	15494.59	16	6	16	18	16	22
Chr22	9546692	AATAT	A	Chr22_95466	AATAT_A	INDEL	15494.59	16	6	16	18	16	22
Chr22	9546692	AATAT	A	Chr22_95466	AATAT_A	INDEL	15494.59	16	6	16	18	16	22
Chr22	9546692	AATAT	A	Chr22_95466	AATAT_A	INDEL	15494.59	16	6	16	18	16	22
Chr22	9546692	AATAT	A	Chr22_95466	AATAT_A	INDEL	15494.59	16	6	16	18	16	22
Chr22	9890315	T	C	Chr22_98903	T_C	SNP	17123.63	31	13	18	30	21	27
Chr22	10045436	T	TC	Chr22_10045	T_TC	INDEL	16315.59	23	16	17	26	15	24

[illegible]

Chr23	4831049	A	AAT	Chr23_48310	A_AAT	INDEL	8732.59	21	9	12	18	7	18	[illegible]
Chr23	4831049	A	AAT	Chr23_48310	A_AAT	INDEL	8732.59	21	9	12	18	7	18	[illegible]
Chr23	4831049	A	AAT	Chr23_48310	A_AAT	INDEL	8732.59	21	9	12	18	7	18	[illegible]
Chr23	4831049	A	AAT	Chr23_48310	A_AAT	INDEL	8732.59	21	9	12	18	7	18	[illegible]
Chr23	4831049	A	AAT	Chr23_48310	A_AAT	INDEL	8732.59	21	9	12	18	7	18	[illegible]
Chr23	4831049	A	AAT	Chr23_48310	A_AAT	INDEL	8732.59	21	9	12	18	7	18	[illegible]
Chr23	4951364	C	A	Chr23_49513	C_A	SNP	14878.63	26	9	13	21	11	31	[illegible]
Chr23	4951366	T	A	Chr23_49513	T_A	SNP	15103.63	27	10	13	22	11	30	[illegible]
Chr23	4951368	C	CG	Chr23_49513	C_CG	INDEL	15276.59	28	10	13	21	11	31	[illegible]
Chr23	4951369	C	T	Chr23_49513	C_T	SNP	15240.63	28	9	13	21	12	30	[illegible]
Chr23	5025297	G	T	Chr23_50252	G_T	SNP	15964.63	14	10	14	15	29	26	[illegible]
Chr23	5025297	G	T	Chr23_50252	G_T	SNP	15964.63	14	10	14	15	29	26	[illegible]
Chr23	5025297	G	T	Chr23_50252	G_T	SNP	15964.63	14	10	14	15	29	26	[illegible]
Chr23	5025298	CCTTTTGA	C	Chr23_50252	CCTTTTGA_C	INDEL	16000.59	14	10	14	15	29	26	[illegible]
Chr23	5025298	CCTTTTGA	C	Chr23_50252	CCTTTTGA_C	INDEL	16000.59	14	10	14	15	29	26	[illegible]
Chr23	5025298	CCTTTTGA	C	Chr23_50252	CCTTTTGA_C	INDEL	16000.59	14	10	14	15	29	26	[illegible]
Chr23	5025307	C	CAAATG	Chr23_50253	C_CAAATG	INDEL	16256.59	14	11	12	14	29	26	[illegible]
Chr23	5025307	C	CAAATG	Chr23_50253	C_CAAATG	INDEL	16256.59	14	11	12	14	29	26	[illegible]
Chr23	5025307	C	CAAATG	Chr23_50253	C_CAAATG	INDEL	16256.59	14	11	12	14	29	26	[illegible]
Chr23	5025311	C	A	Chr23_50253	C_A	SNP	15500.63	14	10	11	15	28	25	[illegible]
Chr23	5025311	C	A	Chr23_50253	C_A	SNP	15500.63	14	10	11	15	28	25	[illegible]
Chr23	5025311	C	A	Chr23_50253	C_A	SNP	15500.63	14	10	11	15	28	25	[illegible]
Chr23	5025315	T	TG	Chr23_50253	T_TG	INDEL	15716.59	13	11	11	13	28	25	[illegible]
Chr23	5025315	T	TG	Chr23_50253	T_TG	INDEL	15716.59	13	11	11	13	28	25	[illegible]
Chr23	5025315	T	TG	Chr23_50253	T_TG	INDEL	15716.59	13	11	11	13	28	25	[illegible]
Chr23	5025316	A	C	Chr23_50253	A_C	SNP	15545.63	13	11	11	15	28	26	[illegible]
Chr23	5025316	A	C	Chr23_50253	A_C	SNP	15545.63	13	11	11	15	28	26	[illegible]
Chr23	5025316	A	C	Chr23_50253	A_C	SNP	15545.63	13	11	11	15	28	26	[illegible]
Chr23	5025319	CCA	C	Chr23_50253	CCA_C	INDEL	15266.59	13	11	11	15	27	24	[illegible]
Chr23	5025319	CCA	C	Chr23_50253	CCA_C	INDEL	15266.59	13	11	11	15	27	24	[illegible]
Chr23	5025319	CCA	C	Chr23_50253	CCA_C	INDEL	15266.59	13	11	11	15	27	24	[illegible]
Chr23	5025323	T	TGTGTC	Chr23_50253	T_TGTGTC	INDEL	15671.59	13	11	11	15	27	23	[illegible]
Chr23	5025323	T	TGTGTC	Chr23_50253	T_TGTGTC	INDEL	15671.59	13	11	11	15	27	23	[illegible]
Chr23	5025323	T	TGTGTC	Chr23_50253	T_TGTGTC	INDEL	15671.59	13	11	11	15	27	23	[illegible]
Chr23	5025324	A	T	Chr23_50253	A_T	SNP	15815.63	15	10	10	14	28	22	[illegible]
Chr23	5025324	A	T	Chr23_50253	A_T	SNP	15815.63	15	10	10	14	28	22	[illegible]
Chr23	5025324	A	T	Chr23_50253	A_T	SNP	15815.63	15	10	10	14	28	22	[illegible]
Chr23	5025328	T	G	Chr23_50253	T_G	SNP	15230.63	14	10	10	15	28	23	[illegible]
Chr23	5025328	T	G	Chr23_50253	T_G	SNP	15230.63	14	10	10	15	28	23	[illegible]
Chr23	5025328	T	G	Chr23_50253	T_G	SNP	15230.63	14	10	10	15	28	23	[illegible]
Chr23	5025329	T	A	Chr23_50253	T_A	SNP	15230.63	14	10	10	15	28	23	[illegible]
Chr23	5025329	T	A	Chr23_50253	T_A	SNP	15230.63	14	10	10	15	28	23	[illegible]
Chr23	5025329	T	A	Chr23_50253	T_A	SNP	15230.63	14	10	10	15	28	23	[illegible]
Chr23	5025331	T	C	Chr23_50253	T_C	SNP	15635.63	14	10	9	16	28	23	[illegible]
Chr23	5025331	T	C	Chr23_50253	T_C	SNP	15635.63	14	10	9	16	28	23	[illegible]
Chr23	5025331	T	C	Chr23_50253	T_C	SNP	15635.63	14	10	9	16	28	23	[illegible]
Chr23	5025333	A	ATTC	Chr23_50253	A_ATTC	INDEL	15626.59	15	10	9	17	27	22	[illegible]
Chr23	5025333	A	ATTC	Chr23_50253	A_ATTC	INDEL	15626.59	15	10	9	17	27	22	[illegible]
Chr23	5025333	A	ATTC	Chr23_50253	A_ATTC	INDEL	15626.59	15	10	9	17	27	22	[illegible]
Chr23	5025337	C	A	Chr23_50253	C_A	SNP	15545.63	15	10	8	18	26	21	[illegible]
Chr23	5025337	C	A	Chr23_50253	C_A	SNP	15545.63	15	10	8	18	26	21	[illegible]
Chr23	5025337	C	A	Chr23_50253	C_A	SNP	15545.63	15	10	8	18	26	21	[illegible]
Chr23	5025338	T	C	Chr23_50253	T_C	SNP	15500.63	16	11	8	18	25	20	[illegible]
Chr23	5025338	T	C	Chr23_50253	T_C	SNP	15500.63	16	11	8	18	25	20	[illegible]
Chr23	5025338	T	C	Chr23_50253	T_C	SNP	15500.63	16	11	8	18	25	20	[illegible]
Chr23	5025341	T	TATGCA	Chr23_50253	T_TATGCA	INDEL	15986.59	15	10	8	18	23	22	[illegible]
Chr23	5025341	T	TATGCA	Chr23_50253	T_TATGCA	INDEL	15986.59	15	10	8	18	23	22	[illegible]
Chr23	5025341	T	TATGCA	Chr23_50253	T_TATGCA	INDEL	15986.59	15	10	8	18	23	22	[illegible]
Chr23	5025344	A	AG	Chr23_50253	A_AG	INDEL	14951.59	15	10	8	19	24	20	[illegible]
Chr23	5025344	A	AG	Chr23_50253	A_AG	INDEL	14951.59	15	10	8	19	24	20	[illegible]
Chr23	5025344	A	AG	Chr23_50253	A_AG	INDEL	14951.59	15	10	8	19	24	20	[illegible]
Chr23	5025347	A	C	Chr23_50253	A_C	SNP	14465.63	14	11	7	19	19	18	[illegible]
Chr23	5025347	A	C	Chr23_50253	A_C	SNP	14465.63	14	11	7	19	19	18	[illegible]
Chr23	5025347	A	C	Chr23_50253	A_C	SNP	14465.63	14	11	7	19	19	18	[illegible]
Chr23	5025355	T	C	Chr23_50253	T_C	SNP	14240.63	16	12	5	17	19	17	[illegible]
Chr23	5025355	T	C	Chr23_50253	T_C	SNP	14240.63	16	12	5	17	19	17	[illegible]
Chr23	5025355	T	C	Chr23_50253	T_C	SNP	14240.63	16	12	5	17	19	17	[illegible]
Chr23	5025356	AAGAGTT	A	Chr23_50253	AAGAGTT_A	INDEL	14456.59	16	12	5	17	20	17	[illegible]
Chr23	5025356	AAGAGTT	A	Chr23_50253	AAGAGTT_A	INDEL	14456.59	16	12	5	17	20	17	[illegible]
Chr23	5025356	AAGAGTT	A	Chr23_50253	AAGAGTT_A	INDEL	14456.59	16	12	5	17	20	17	[illegible]
Chr23	5025366	GGATAA	G	Chr23_50253	GGATAA_G	INDEL	14321.59	15	11	6	17	21	20	[illegible]
Chr23	5025366	GGATAA	G	Chr23_50253	GGATAA_G	INDEL	14321.59	15	11	6	17	21	20	[illegible]
Chr23	5025366	GGATAA	G	Chr23_50253	GGATAA_G	INDEL	14321.59	15	11	6	17	21	20	[illegible]
Chr23	5025373	T	TTCA	Chr23_50253	T_TTCA	INDEL	14446.59	15	11	6	17	21	20	[illegible]
Chr23	5025373	T	TTCA	Chr23_50253	T_TTCA	INDEL	14446.59	15	11	6	17	21	20	[illegible]
Chr23	5025373	T	TTCA	Chr23_50253	T_TTCA	INDEL	14446.59	15	11	6	17	21	20	[illegible]
Chr23	5068772	C	CA	Chr23_50687	C_CA	INDEL	16321.59	31	16	15	26	20	28	[illegible]
Chr23	5068772	C	CA	Chr23_50687	C_CA	INDEL	16321.59	31	16	15	26	20	28	[illegible]
Chr23	5068772	C	CA	Chr23_50687	C_CA	INDEL	16321.59	31	16	15	26	20	28	[illegible]
Chr23	5107784	C	CG	Chr23_51077	C_CG	INDEL	20128.59	31	11	7	21	27	34	[illegible]
Chr23	5107784	C	CG	Chr23_51077	C_CG	INDEL	20128.59	31	11	7	21	27	34	[illegible]
Chr23	5107784	C	CG	Chr23_51077	C_CG	INDEL	20128.59	31	11	7	21	27	34	[illegible]
Chr23	5107784	C	CG	Chr23_51077	C_CG	INDEL	20128.59	31	11	7	21	27	34	[illegible]
Chr23	5107784	C	CG	Chr23_51077	C_CG	INDEL	20128.59	31	11	7	21	27	34	[illegible]
Chr23	5107784	C	CG	Chr23_51077	C_CG	INDEL	20128.59	31	11	7	21	27	34	[illegible]
Chr23	5107784	C	CG	Chr23_51077	C_CG	INDEL	20128.59	31	11	7	21	27	34	[illegible]
Chr23	5107788	C	CA	Chr23_51077	C_CA	INDEL	20337.59	33	11	7	22	28	36	[illegible]
Chr23	5107788	C	CA	Chr23_51077	C_CA	INDEL	20337.59	33	11	7	22	28	36	[illegible]
Chr23	5107788	C	CA	Chr23_51077	C_CA	INDEL	20337.59	33	11	7	22	28	36	[illegible]
Chr23	5107788	C	CA	Chr23_51077	C_CA	INDEL	20337.59	33	11	7	22	28	36	[illegible]
Chr23	5107788	C	CA	Chr23_51077	C_CA	INDEL	20337.59	33	11	7	22	28	36	[illegible]
Chr23	5107788	C	CA	Chr23_51077	C_CA	INDEL	20337.59	33	11	7	22	28	36	[illegible]
Chr23	5107788	C	CA	Chr23_51077	C_CA	INDEL	20337.59	33	11	7	22	28	36	[illegible]
Chr23	5153219	G	T	Chr23_51532	G_T	SNP	5932.29	0	0	0	30	15	17	[illegible]
Chr23	5215758	T	TA	Chr23_52157	T_TA	INDEL	10693.59	24	9	12	15	13	17	[illegible]
Chr23	5215758	T	TA	Chr23_52157	T_TA	INDEL	10693.59	24	9	12	15	13	17	[illegible]
Chr23	5215758	T	TA	Chr23_52157	T_TA	INDEL	10693.59	24	9	12	15	13	17	[illegible]
Chr23	5311469	G	GTA	Chr23_53114	G_GTA	INDEL	9050.59	20	17	10	17	13	19	[illegible]
Chr23	5311469	G	GTA	Chr23_53114	G_GTA	INDEL	9050.59	20	17	10	17	13	19	[illegible]
Chr23	5311469	G	GTA	Chr23_53114	G_GTA	INDEL	9050.59	20	17	10	17	13	19	[illegible]
Chr23	5311469	G	GTA	Chr23_53114	G_GTA	INDEL	9050.59	20	17	10	17	13	19	[illegible]
Chr23	5311469	G	GTA	Chr23_53114	G_GTA	INDEL	9050.59	20	17	10	17	13	19	[illegible]
Chr23	5311469	G	GTA	Chr23_53114	G_GTA	INDEL	9050.59	20	17	10	17	13	19	[illegible]
Chr23	5311469	G	GTA	Chr23_53114	G_GTA	INDEL	9050.59	20	17	10	17	13	19	[illegible]
Chr23	5311469	G	GTA	Chr23_53114	G_GTA	INDEL	9050.59	20	17	10	17	13	19	[illegible]
Chr23	5414449	A	ACC	Chr23_54144	A_ACC	INDEL	19626.59	31	14	16	27	14	23	[illegible]
Chr23	5414449	A	ACC	Chr23_54144	A_ACC	INDEL	19626.59	31	14	16	27	14	23	[illegible]
Chr23	5414449	A	ACC	Chr23_54144	A_ACC	INDEL	19626.59	31	14	16	27	14	23	[illegible]
Chr23	5414450	GAA	G	Chr23_54144	GAA_G	INDEL	19626.59	31	13	16	26	14	23	[illegible]
Chr23	5414450	GAA	G	Chr23_54144	GAA_G	INDEL	19626.59	31	13	16	26	14	23	[illegible]
Chr23	5414450	GAA	G	Chr23_54144	GAA_G	INDEL	19626.59	31	13	16	26	14	23	[illegible]
Chr23	5551104	AAT	A	Chr23_55511	AAT_A	INDEL	1740.5	1	0	0	1	0	1	[illegible]
Chr23	5555172	TG	T	Chr23_55551	TG_T	INDEL	15211.59	28	12	11	23	20	25	[illegible]
Chr23	5555196	CA	C	Chr23_55551	CA_C	INDEL	14931.59	27	14	10	23	18	26	[illegible]
Chr23	5555267	C	A	Chr23_55552	C_A	SNP	17362.63	25	13	11	17	16	28	[illegible]
Chr23	5555352	G	A	Chr23_55553	G_A	SNP	12650.63	26	6	13	11	13	16	[illegible]
Chr23	5555533	T	A	Chr23_55555	T_A	SNP	9096.63	15	8	7	7	6	11	[illegible]
Chr23	5580103	G	A	Chr23_55801	G_A	SNP	2495.39	32	10	18	0	0	0	[illegible]
Chr23	5580132	G	T	Chr23_55801	G_T	SNP	4357.98	0	0	0	0	0	0	[illegible]
Chr23	5588631	C	T	Chr23_55886	C_T	SNP	11695.29	38	13	21	0	0	0	[illegible]
Chr23	5676049	A	AAT	Chr23_56760	A_AAT	INDEL	2203.24	1	1	1	15	15	19	[illegible]
Chr23	5676049	A	AAT	Chr23_56760	A_AAT	INDEL	2203.24	1	1	1	15	15	19	[illegible]
Chr23	5676049	A	AAT	Chr23_56760	A_AAT	INDEL	2203.24	1	1	1	15	15	19	[illegible]
Chr23	5676049	A	AAT	Chr23_56760	A_AAT	INDEL	2203.24	1	1	1	15	15	19	[illegible]
Chr23	5676049	A	AAT	Chr23_56760	A_AAT	INDEL	2203.24	1	1	1	15	15	19	[illegible]
Chr23	5676049	A	AAT	Chr23_56760	A_AAT	INDEL	2203.24	1	1	1	15	15	19	[illegible]
Chr23	5676049	A	AAT	Chr23_56760	A_AAT	INDEL	2203.24	1	1	1	15	15	19	[illegible]
Chr23	5676049	A	AAT	Chr23_56760	A_AAT	INDEL	2203.24	1	1	1	15	15	19	[illegible]
Chr23	5676049	A	AAT	Chr23_56760	A_AAT	INDEL	2203.24	1	1	1	15	15	19	[illegible]
Chr23	5676049	A	AAT	Chr23_56760	A_AAT	INDEL	2203.24	1	1	1	15	15	19	[illegible]
Chr23	5676049	A	AAT	Chr23_56760	A_AAT	INDEL	2203.24	1	1	1	15	15	19	[illegible]
Chr23	5799779	T	G	Chr23_57997	T_G	SNP	21563.63	38	10	15	39	18	15	[illegible]
Chr23	5799780	G	T	Chr23_57997	G_T	SNP	21563.63	38	12	15	40	19	15	[illegible]
Chr23	5889543	A	AAT	Chr23_58895	A_AAT	INDEL	7976.59	21	7	13	13	10	21	[illegible]
Chr23	5889543	A	AAT	Chr23_58895	A_AAT	INDEL	7976.59	21	7	13	13	10	21	[illegible]
Chr23	5889543	A	AAT	Chr23_58895	A_AAT	INDEL	7976.59	21	7	13	13	10	21	[illegible]
Chr23	6137717	T	TA	Chr23_61377	T_TA	INDEL	19851.59	37	12	7	21	27	22	[illegible]
Chr23	6137718	C	A	Chr23_61377	C_A	SNP	19995.63	38	12	7	21	27	22	[illegible]
Chr23	6137721	T	A	Chr23_61377	T_A	SNP	20257.63	37	13	8	21	26	22	[illegible]
Chr23	6137722	C	T	Chr23_61377	C_T	SNP	20032.63	37	13	8	21	26	22	[illegible]
Chr23	6138003	T	TA	Chr23_61380	T_TA	INDEL	19053.59	33	14	15	39	27	38	[illegible]
Chr23	6493032	G	T	Chr23_64930	G_T	SNP	10755.29	31	14	14	0	0	0	[illegible]
Chr23	6493032	G	T	Chr23_64930	G_T	SNP	10755.29	31	14	14	0	0	0	[illegible]
Chr23	6493032	G	T	Chr23_64930	G_T	SNP	10755.29	31	14	14	0	0	0	[illegible]
Chr23	6493032	G	T	Chr23_64930	G_T	SNP	10755.29	31	14	14	0	0	0	[illegible]
Chr23	6555488	G	A	Chr23_65554	G_A	SNP	16757.63	20	4	17	15	25	19	[illegible]
Chr23	6557381	T	C	Chr23_65573	T_C	SNP	14343.63	27	11	20	17	11	19	[illegible]
Chr23	6559258	G	T	Chr23_65592	G_T	SNP	15203.63	24	7	11	19	17	15	[illegible]
Chr23	6559286	G	T	Chr23_65592	G_T	SNP	16322.63	24	8	14	18	20	15	[illegible]
Chr23	6559450	G	T	Chr23_65594	G_T	SNP	14052.63	25	20	14	8	20	10	[illegible]
Chr23	6559460	A	T	Chr23_65594	A_T	SNP	15114.63	23	22	14	12	21	12	[illegible]
Chr23	6559462	A	T	Chr23_65594	A_T	SNP	14989.63	23	22	14	14	21	13	[illegible]
Chr23	6559463	A	T	Chr23_65594	A_T	SNP	14944.63	24	21	14	14	21	13	[illegible]
Chr23	6561999	AG	A	Chr23_65619	AG_A	INDEL	15147.59	25	10	18	19	20	19	[illegible]
Chr23	6562052	A	T	Chr23_65620	A_T	SNP	13056.63	20	11	14	16	9	15	[illegible]
Chr23	6563623	A	T	Chr23_65636	A_T	SNP	13798.63	17	12	8	16	10	29	[illegible]
Chr23	6563813	A	G	Chr23_65638	A_G	SNP	16568.63	22	14	15	13	15	25	[illegible]
Chr23	6563814	A	C	Chr23_65638	A_C	SNP	16687.63	22	13	15	13	15	25	[illegible]
Chr23	6563834	G	C	Chr23_65638	G_C	SNP	16225.63	21	14	17	11	16	25	[illegible]
Chr23	6572504	T	C	Chr23_65725	T_C	SNP	18531.63	26	15	14	23	9	27	[illegible]
Chr23	6572507	T	A	Chr23_65725	T_A	SNP	18423.63	24	15	13	22	9	27	[illegible]
Chr23	6981177	GC	G	Chr23_69811	GC_G	INDEL	19150.59	33	6	13	27	18	27	[illegible]
Chr23	6981344	C	A	Chr23_69813	C_A	SNP	20251.63	25	10	16	26	15	32	[illegible]
Chr23	6981347	C	A	Chr23_69813	C_A	SNP	20468.63	26	9	15	26	15	33	[illegible]
Chr23	6981355	C	A	Chr23_69813	C_A	SNP	20556.63	26	9	16	28	16	36	[illegible]
Chr23	7146773	G	GTA	Chr23_71467	G_GTA	INDEL	4056.24	2	0	0	21	20	25	[illegible]
Chr23	7146773	G	GTA	Chr23_71467	G_GTA	INDEL	4056.24	2	0	0	21	20	25	[illegible]
Chr23	7146773	G	GTA	Chr23_71467	G_GTA	INDEL	4056.24	2	0	0	21	20	25	[illegible]
Chr23	7146773	G	GTA	Chr23_71467	G_GTA	INDEL	4056.24	2	0	0	21	20	25	[illegible]
Chr23	7180173	CATATAT	C	Chr23_71801	CATATAT_C	INDEL	494.37	0	0	0	0	0	0	[illegible]
Chr23	7180173	CATATAT	C	Chr23_71801	CATATAT_C	INDEL	494.37	0	0	0	0	0	0	[illegible]
Chr23	7180173	CATATAT	C	Chr23_71801	CATATAT_C	INDEL	494.37	0	0	0	0	0	0	[illegible]
Chr23	7180173	CATATAT	C	Chr23_71801	CATATAT_C	INDEL	494.37	0	0	0	0	0	0	[illegible]
Chr23	7180173	CATATAT	C	Chr23_71801	CATATAT_C	INDEL	494.37	0	0	0	0	0	0	[illegible]
Chr23	7180173	CATATAT	C	Chr23_71801	CATATAT_C	INDEL	494.37	0	0	0	0	0	0	[illegible]
Chr23	7180173	CATATAT	C	Chr23_71801	CATATAT_C	INDEL	494.37	0	0	0	0	0	0	[illegible]
Chr23	7180173	CATATAT	C	Chr23_71801	CATATAT_C	INDEL	494.37	0	0	0	0	0	0	[illegible]
Chr23	7180173	CATATAT	C	Chr23_71801	CATATAT_C	INDEL	494.37	0	0	0	0	0	0	[illegible]
Chr23	7180173	CATATAT	C	Chr23_71801	CATATAT_C	INDEL	494.37	0	0	0	0	0	0	[illegible]
Chr23	7248545	T	C	Chr23_72485	T_C	SNP	21407.63	33	15	17	25	23	32	[illegible]
Chr23	7459075	T	TTA	Chr23_74590	T_TTA	INDEL	4314.24	14	11	12	0	0	1	[illegible]
Chr23	7860452	C	CAT	Chr23_78604	C_CAT	INDEL	2498.24	0	0	0	13	20	19	[illegible]
Chr23	7860452	C	CAT	Chr23_78604	C_CAT	INDEL	2498.24	0	0	0	13	20	19	[illegible]
Chr23	7860452	C	CAT	Chr23_78604	C_CAT	INDEL	2498.24	0	0	0	13	20	19	[illegible]
Chr23	7860452	C	CAT	Chr23_78604	C_CAT	INDEL	2498.24	0	0	0	13	20	19	[illegible]
Chr23	7860452	C	CAT	Chr23_78604	C_CAT	INDEL	2498.24	0	0	0	13	20	19	[illegible]
Chr23	7860452	C	CAT	Chr23_78604	C_CAT	INDEL	2498.24	0	0	0	13	20	19	[illegible]
Chr23	7860452	C	CAT	Chr23_78604	C_CAT	INDEL	2498.24	0	0	0	13	20	19	[illegible]
Chr23	7860452	C	CAT	Chr23_78604	C_CAT	INDEL	2498.24	0	0	0	13	20	19	[illegible]
Chr23	7860452	C	CAT	Chr23_78604	C_CAT	INDEL	2498.24	0	0	0	13	20	19	[illegible]
Chr23	7860452	C	CAT	Chr23_78604	C_CAT	INDEL	2498.24	0	0	0	13	20	19	[illegible]
Chr23	7860452	C	CAT	Chr23_78604	C_CAT	INDEL	2498.24	0	0	0	13	20	19	[illegible]
Chr23	7860452	C	CAT	Chr23_78604	C_CAT	INDEL	2498.24	0	0	0	13	20	19	[illegible]
Chr23	7860452	C	CAT	Chr23_78604	C_CAT	INDEL	2498.24	0	0	0	13	20	19	[illegible]
Chr23	7860452	C	CAT	Chr23_78604	C_CAT	INDEL	2498.24	0	0	0	13	20	19	[illegible]
Chr23	7951360	G	GTA	Chr23_79513	G_GTA	INDEL	9529.59	20	12	7	24	18	18	[illegible]
Chr23	7951360	G	GTA	Chr23_79513	G_GTA	INDEL	9529.59	20	12	7	24	18	18	[illegible]
Chr23	7951360	G	GTA	Chr23_79513	G_GTA	INDEL	9529.59	20	12	7	24	18	18	[illegible]
Chr23	7951360	G	GTA	Chr23_79513	G_GTA	INDEL	9529.59	20	12	7	24	18	18	[illegible]
Chr23	7951360	G	GTA	Chr23_79513	G_GTA	INDEL	9529.59	20	12	7	24	18	18	[illegible]
Chr23	7951360	G	GTA	Chr23_79513	G_GTA	INDEL	9529.59	20	12	7	24	18	18	[illegible]
Chr23	8109011	G	A	Chr23_81090	G_A	SNP	7608.29	17	13	11	0	0	0	[illegible]

Chr23	8190077	AT	A	Chr23_81900	AT_A	INDEL	14737.59	44	20	23
Chr23	8333117	C	T	Chr23_83331	C_T	SNP	3535.98	0	0	0
Chr23	8392019	A	T	Chr23_83920	A_T	SNP	15767.63	24	17	18
Chr23	8392027	C	T	Chr23_83920	C_T	SNP	15706.63	22	16	18
Chr23	8392093	G	C	Chr23_83920	G_C	SNP	14929.63	19	13	14
Chr23	8461436	G	GTA	Chr23_84614	G_GTA	INDEL	174.37	1	1	9
Chr23	8461436	G	GTA	Chr23_84614	G_GTA	INDEL	174.37	1	1	9
Chr23	8461436	G	GTA	Chr23_84614	G_GTA	INDEL	174.37	1	1	9
Chr23	8461436	G	GTA	Chr23_84614	G_GTA	INDEL	174.37	1	1	9
Chr23	8461436	G	GTA	Chr23_84614	G_GTA	INDEL	174.37	1	1	9
Chr23	8461436	G	GTA	Chr23_84614	G_GTA	INDEL	174.37	1	1	9
Chr23	8461436	G	GTA	Chr23_84614	G_GTA	INDEL	174.37	1	1	9
Chr23	8461436	G	GTA	Chr23_84614	G_GTA	INDEL	174.37	1	1	9
Chr23	8461436	G	GTA	Chr23_84614	G_GTA	INDEL	174.37	1	1	9
Chr23	8461436	G	GTA	Chr23_84614	G_GTA	INDEL	174.37	1	1	9
Chr23	8461436	G	GTA	Chr23_84614	G_GTA	INDEL	174.37	1	1	9
Chr23	8461436	G	GTA	Chr23_84614	G_GTA	INDEL	174.37	1	1	9
Chr23	8461436	G	GTA	Chr23_84614	G_GTA	INDEL	174.37	1	1	9
Chr23	8461436	G	GTA	Chr23_84614	G_GTA	INDEL	174.37	1	1	9
Chr23	8461436	G	GTA	Chr23_84614	G_GTA	INDEL	174.37	1	1	9
Chr23	8461436	G	GTA	Chr23_84614	G_GTA	INDEL	174.37	1	1	9
Chr23	8461436	G	GTA	Chr23_84614	G_GTA	INDEL	174.37	1	1	9
Chr23	8461436	G	GTA	Chr23_84614	G_GTA	INDEL	174.37	1	1	9
Chr23	8568639	G	A	Chr23_85686	G_A	SNP	20150.63	31	14	14
Chr23	8568640	T	A	Chr23_85686	T_A	SNP	20242.63	31	13	14
Chr23	8568642	G	A	Chr23_85686	G_A	SNP	20162.63	31	13	14
Chr23	8571627	C	T	Chr23_85716	C_T	SNP	19322.63	18	10	12
Chr23	8571634	T	G	Chr23_85716	T_G	SNP	20004.63	16	10	12
Chr23	8571636	T	C	Chr23_85716	T_C	SNP	19746.63	17	11	11
Chr23	8571637	A	T	Chr23_85716	A_T	SNP	19566.63	17	11	11
Chr23	8571771	C	T	Chr23_85717	C_T	SNP	20755.63	22	17	16
Chr23	8572061	G	T	Chr23_85720	G_T	SNP	19793.63	31	15	28
Chr23	8572181	C	T	Chr23_85721	C_T	SNP	19181.63	32	11	20
Chr23	8718864	T	A	Chr23_87188	T_A	SNP	9105.63	13	6	4
Chr23	8718864	T	A	Chr23_87188	T_A	SNP	9105.63	13	6	4
Chr23	8718864	T	A	Chr23_87188	T_A	SNP	9105.63	13	6	4
Chr23	8734532	G	GA	Chr23_87345	G_GA	INDEL	16073.59	28	12	11
Chr23	8744036	C	T	Chr23_87440	C_T	SNP	2837.39	36	14	16
Chr23	8817737	T	TA	Chr23_88177	T_TA	INDEL	10974.59	17	11	11
Chr23	9236594	AAT	A	Chr23_92365	AAT_A	INDEL	5327.93	1	1	1
Chr23	9236594	AAT	A	Chr23_92365	AAT_A	INDEL	5327.93	1	1	1
Chr23	9236594	AAT	A	Chr23_92365	AAT_A	INDEL	5327.93	1	1	1
Chr23	9236594	AAT	A	Chr23_92365	AAT_A	INDEL	5327.93	1	1	1
Chr23	9236594	AAT	A	Chr23_92365	AAT_A	INDEL	5327.93	1	1	1
Chr23	9236594	AAT	A	Chr23_92365	AAT_A	INDEL	5327.93	1	1	1
Chr23	9236594	AAT	AATAT	Chr23_92365	AAT_AATAT	INDEL	5327.93	1	0	0
Chr23	9236594	AAT	AATAT	Chr23_92365	AAT_AATAT	INDEL	5327.93	1	0	0
Chr23	9236594	AAT	AATAT	Chr23_92365	AAT_AATAT	INDEL	5327.93	1	0	0
Chr23	9236594	AAT	AATAT	Chr23_92365	AAT_AATAT	INDEL	5327.93	1	0	0
Chr23	9236594	AAT	AATAT	Chr23_92365	AAT_AATAT	INDEL	5327.93	1	0	0
Chr23	9236594	AAT	AATAT	Chr23_92365	AAT_AATAT	INDEL	5327.93	1	0	0
Chr23	9277732	T	TA	Chr23_92777	T_TA	INDEL	12952.59	20	19	9
Chr23	9280472	G	T	Chr23_92804	G_T	SNP	12546.63	24	8	8
Chr23	9280481	G	A	Chr23_92804	G_A	SNP	13043.63	25	9	8
Chr23	9280515	C	A	Chr23_92805	C_A	SNP	13904.63	28	8	9
Chr23	9285303	A	G	Chr23_92853	A_G	SNP	20574.63	32	18	16
Chr23	9285372	T	A	Chr23_92853	T_A	SNP	20181.63	30	24	14
Chr23	9298935	AT	A	Chr23_92989	AT_A	INDEL	9770.59	9	8	10
Chr23	9298938	T	TA	Chr23_92989	T_TA	INDEL	9829.59	9	8	10
Chr23	9474537	AT	A	Chr23_94745	AT_A	INDEL	2912.24	0	0	0
Chr23	9474537	AT	A	Chr23_94745	AT_A	INDEL	2912.24	0	0	0
Chr23	9474537	AT	A	Chr23_94745	AT_A	INDEL	2912.24	0	0	0
Chr23	9475750	G	GAT	Chr23_94757	G_GAT	INDEL	2114.93	0	0	0
Chr23	9475750	G	GAT	Chr23_94757	G_GAT	INDEL	2114.93	0	0	0
Chr23	9475750	G	GAT	Chr23_94757	G_GAT	INDEL	2114.93	0	0	0
Chr23	9499597	GAT	G	Chr23_94995	GAT_G	INDEL	11564.59	21	12	9
Chr23	9499597	GAT	G	Chr23_94995	GAT_G	INDEL	11564.59	21	12	9
Chr23	9499597	GAT	G	Chr23_94995	GAT_G	INDEL	11564.59	21	12	9
Chr23	9499597	GAT	G	Chr23_94995	GAT_G	INDEL	11564.59	21	12	9
Chr23	9499597	GAT	G	Chr23_94995	GAT_G	INDEL	11564.59	21	12	9
Chr23	9499597	GAT	G	Chr23_94995	GAT_G	INDEL	11564.59	21	12	9
Chr23	9499597	GAT	G	Chr23_94995	GAT_G	INDEL	11564.59	21	12	9
Chr23	9499597	GAT	G	Chr23_94995	GAT_G	INDEL	11564.59	21	12	9
Chr23	9499597	GAT	G	Chr23_94995	GAT_G	INDEL	11564.59	21	12	9
Chr23	9499597	GAT	G	Chr23_94995	GAT_G	INDEL	11564.59	21	12	9
Chr23	9499597	GAT	G	Chr23_94995	GAT_G	INDEL	11564.59	21	12	9
Chr23	9658999	G	GAT	Chr23_96589	G_GAT	INDEL	958.37	0	1	0
Chr23	9658999	G	GAT	Chr23_96589	G_GAT	INDEL	958.37	0	1	0
Chr23	9683085	CG	C	Chr23_96830	CG_C	INDEL	11429.59	29	11	12
Chr23	9683085	CG	C	Chr23_96830	CG_C	INDEL	11429.59	29	11	12
Chr23	9683085	CG	C	Chr23_96830	CG_C	INDEL	11429.59	29	11	12
Chr23	9761674	G	T	Chr23_97616	G_T	SNP	12806.29	27	18	18
Chr23	9942713	A	C	Chr23_99427	A_C	SNP	1350.29	0	1	0
Chr23	9955022	A	AT	Chr23_99550	A_AT	INDEL	16560.59	31	21	13
Chr23	9955022	A	AT	Chr23_99550	A_AT	INDEL	16560.59	31	21	13
Chr23	9955022	A	AT	Chr23_99550	A_AT	INDEL	16560.59	31	21	13
Chr23	10333775	G	GTA	Chr23_10333	G_GTA	INDEL	1218.35	21	13	7
Chr23	10333775	G	GTA	Chr23_10333	G_GTA	INDEL	1218.35	21	13	7
Chr23	10333775	G	GTA	Chr23_10333	G_GTA	INDEL	1218.35	21	13	7
Chr23	10333775	G	GTA	Chr23_10333	G_GTA	INDEL	1218.35	21	13	7
Chr23	10333775	G	GTA	Chr23_10333	G_GTA	INDEL	1218.35	21	13	7
Chr23	10333775	G	GTA	Chr23_10333	G_GTA	INDEL	1218.35	21	13	7
Chr23	10333775	G	GTA	Chr23_10333	G_GTA	INDEL	1218.35	21	13	7
Chr23	10409804	C	A	Chr23_10409	C_A	SNP	19592.63	24	16	19
Chr23	10409886	GA	G	Chr23_10409	GA_G	INDEL	17353.59	29	18	20
Chr23	10412353	C	T	Chr23_10412	C_T	SNP	20314.63	25	15	9
Chr23	10412354	T	C	Chr23_10412	T_C	SNP	20529.63	25	15	9
Chr23	10412356	T	A	Chr23_10412	T_A	SNP	20417.63	26	15	10
Chr23	10486788	C	CTA	Chr23_10486	C_CTA	INDEL	719.35	11	6	12
Chr23	10486788	C	CTA	Chr23_10486	C_CTA	INDEL	719.35	11	6	12
Chr23	10486788	C	CTA	Chr23_10486	C_CTA	INDEL	719.35	11	6	12
Chr23	10486788	C	CTA	Chr23_10486	C_CTA	INDEL	719.35	11	6	12
Chr23	10486788	C	CTA	Chr23_10486	C_CTA	INDEL	719.35	11	6	12
Chr23	10486788	C	CTA	Chr23_10486	C_CTA	INDEL	719.35	11	6	12
Chr23	10486788	C	CTA	Chr23_10486	C_CTA	INDEL	719.35	11	6	12
Chr23	10486788	C	CTA	Chr23_10486	C_CTA	INDEL	719.35	11	6	12
Chr23	10486788	C	CTA	Chr23_10486	C_CTA	INDEL	719.35	11	6	12
Chr23	10486788	C	CTA	Chr23_10486	C_CTA	INDEL	719.35	11	6	12
Chr23	10486788	C	CTA	Chr23_10486	C_CTA	INDEL	719.35	11	6	12
Chr23	10486788	C	CTA	Chr23_10486	C_CTA	INDEL	719.35	11	6	12
Chr23	10486788	C	CTA	Chr23_10486	C_CTA	INDEL	719.35	11	6	12
Chr23	10486788	C	CTA	Chr23_10486	C_CTA	INDEL	719.35	11	6	12
Chr23	10486788	C	CTA	Chr23_10486	C_CTA	INDEL	719.35	11	6	12
Chr23	10486788	C	CTA	Chr23_10486	C_CTA	INDEL	719.35	11	6	12
Chr23	10527327	A	AAT	Chr23_10527	A_AAT	INDEL	1510.93	0	1	2
Chr23	10527327	A	AAT	Chr23_10527	A_AAT	INDEL	1510.93	0	1	2
Chr23	10574051	T	A	Chr23_10574	T_A	SNP	5664.29	0	0	0
Chr23	10574051	T	A	Chr23_10574	T_A	SNP	5664.29	0	0	0
Chr23	10574051	T	A	Chr23_10574	T_A	SNP	5664.29	0	0	0
Chr23	10574051	T	A	Chr23_10574	T_A	SNP	5664.29	0	0	0
Chr23	10574051	T	A	Chr23_10574	T_A	SNP	5664.29	0	0	0
Chr23	10630205	C	G	Chr23_10630	C_G	SNP	12945.63	26	8	8
Chr23	10654664	C	CT	Chr23_10654	C_CT	INDEL	16211.59	18	16	30
Chr23	10758209	C	A	Chr23_10758	C_A	SNP	2222.39	23	14	15
Chr23	10758209	C	A	Chr23_10758	C_A	SNP	2222.39	23	14	15
Chr23	10758209	C	A	Chr23_10758	C_A	SNP	2222.39	23	14	15
Chr23	10977414	G	A	Chr23_10977	G_A	SNP	11536.63	18	6	14
Chr23	11053473	T	TG	Chr23_11053	T_TG	INDEL	16500.59	24	16	11
Chr23	11053473	T	TG	Chr23_11053	T_TG	INDEL	16500.59	24	16	11
Chr23	11053511	G	T	Chr23_11053	G_T	SNP	18945.63	21	11	12
Chr23	11053511	G	T	Chr23_11053	G_T	SNP	18945.63	21	11	12
Chr23	11053545	G	T	Chr23_11053	G_T	SNP	18758.63	23	12	12
Chr23	11053545	G	T	Chr23_11053	G_T	SNP	18758.63	23	12	12
Chr23	11125929	TTA	T	Chr23_11125	TTA_T	INDEL	10752.59	22	9	13
Chr23	11125929	TTA	T	Chr23_11125	TTA_T	INDEL	10752.59	22	9	13
Chr23	11125929	TTA	T	Chr23_11125	TTA_T	INDEL	10752.59	22	9	13
Chr23	11125929	TTA	T	Chr23_11125	TTA_T	INDEL	10752.59	22	9	13
Chr23	11125929	TTA	T	Chr23_11125	TTA_T	INDEL	10752.59	22	9	13
Chr23	11125929	TTA	T	Chr23_11125	TTA_T	INDEL	10752.59	22	9	13
Chr23	11125929	TTA	T	Chr23_11125	TTA_T	INDEL	10752.59	22	9	13
Chr23	11151915	GCT	G	Chr23_11151	GCT_G	INDEL	19671.59	19	13	9
Chr23	11151915	GCT	G	Chr23_11151	GCT_G	INDEL	19671.59	19	13	9
Chr23	11151915	GCT	G	Chr23_11151	GCT_G	INDEL	19671.59	19	13	9
Chr23	11151915	GCT	G	Chr23_11151	GCT_G	INDEL	19671.59	19	13	9
Chr23	11151915	GCT	G	Chr23_11151	GCT_G	INDEL	19671.59	19	13	9
Chr23	11151915	GCT	G	Chr23_11151	GCT_G	INDEL	19671.59	19	13	9
Chr23	11151915	GCT	G	Chr23_11151	GCT_G	INDEL	19671.59	19	13	9
Chr23	11151915	GCT	G	Chr23_11151	GCT_G	INDEL	19671.59	19	13	9
Chr23	11151918	T	TGG	Chr23_11151	T_TGG	INDEL	19671.59	19	13	9
Chr23	11151918	T	TGG	Chr23_11151	T_TGG	INDEL	19671.59	19	13	9
Chr23	11151918	T	TGG	Chr23_11151	T_TGG	INDEL	19671.59	19	13	9
Chr23	11151918	T	TGG	Chr23_11151	T_TGG	INDEL	19671.59	19	13	9
Chr23	11151918	T	TGG	Chr23_11151	T_TGG	INDEL	19671.59	19	13	9
Chr23	11151918	T	TGG	Chr23_11151	T_TGG	INDEL	19671.59	19	13	9
Chr23	11151918	T	TGG	Chr23_11151	T_TGG	INDEL	19671.59	19	13	9
Chr23	11151918	T	TGG	Chr23_11151	T_TGG	INDEL	19671.59	19	13	9
Chr23	11248087	T	C	Chr23_11248	T_C	SNP	15342.63	24	10	7
Chr23	11248087	T	C	Chr23_11248	T_C	SNP	15342.63	24	10	7
Chr23	11248087	T	C	Chr23_11248	T_C	SNP	15342.63	24	10	7
Chr23	11248087	T	C	Chr23_11248	T_C	SNP	15342.63	24	10	7
Chr23	11248087	T	C	Chr23_11248	T_C	SNP	15342.63	24	10	7
Chr23	11319247	AAT	A	Chr23_11319	AAT_A	INDEL	3746.24	0	0	0
Chr23	11515367	AG	A	Chr23_11515	AG_A	INDEL	7898.59	14	10	16
Chr23	11667905	C	CA	Chr23_11667	C_CA	INDEL	15317.59	29	10	23
Chr23	11691297	G	A	Chr23_11691	G_A	SNP	6411.29	0	0	0
Chr23	11704851	TTC	T	Chr23_11704	TTC_T	INDEL	11408.59	18	7	7
Chr23	11704854	C	CTT	Chr23_11704	C_CTT	INDEL	11408.59	18	7	7
Chr23	11705140	G	T	Chr23_11705	G_T	SNP	13666.63	24	12	11
Chr23	11705155	G	T	Chr23_11705	G_T	SNP	14805.63	25	13	12
Chr23	11705255	C	CT	Chr23_11705	C_CT	INDEL	16586.59	27	24	16
Chr23	11707734	TA	T	Chr23_11707	TA_T	INDEL	15134.59	29	12	20
Chr23	11709925	A	T	Chr23_11709	A_T	SNP	16719.63	20	30	22
Chr23	11724368	C	T	Chr23_11724	C_T	SNP	2710.39	36	11	17
Chr23	11728895	A	AAT	Chr23_11728	A_AAT	INDEL	2182.93	0	0	0
Chr23	11834375	G	GT	Chr23_11834	G_GT	INDEL	18782.59	20	15	19
Chr23	11834375	G	GT	Chr23_11834	G_GT	INDEL	18782.59	20	15	19
Chr23	11891242	G	A	Chr23_11891	G_A	SNP	15396.63	18	17	9
Chr23	11891287	G	A	Chr23_11891	G_A	SNP	17659.63	17	19	12
Chr23	11891297	A	G	Chr23_11891	A_G	SNP	18037.63	18	20	12
Chr23	11893732	A	G	Chr23_11893	A_G	SNP	17459.63	28	7	15
Chr23	12129077	G	T	Chr23_12129	G_T	SNP	16794.63	31	17	8
Chr23	12129077	G	T	Chr23_12129	G_T	SNP	16794.63	31	17	8
Chr23	12129077	G	T	Chr23_12129	G_T	SNP	16794.63	31	17	8
Chr23	12129077	G	T	Chr23_12129	G_T	SNP	16794.63	31	17	8
Chr23	12129077	G	T	Chr23_12129	G_T	SNP	16794.63	31	17	8
Chr23	12129077	G	T	Chr23_12129	G_T	SNP	16794.63	31	17	8
Chr23	12129077	G	T	Chr23_12129	G_T	SNP	16794.63	31	17	8
Chr23	12129080	G	T	Chr23_12129	G_T	SNP	16819.63	31	16	9
Chr23	12129080	G	T	Chr23_12129	G_T	SNP	16819.63	31	16	9
Chr23	12129080	G	T	Chr23_12129	G_T	SNP	16819.63	31	16	9
Chr23	12129080	G	T	Chr23_12129	G_T	SNP	16819.63	31	16	9
Chr23	12129080	G	T	Chr23_12129	G_T	SNP	16819.63	31	16	9
Chr23	12129080	G	T	Chr23_12129	G_T	SNP	16819.63	31	16	9
Chr23	12129080	G	T	Chr23_12129	G_T	SNP	16819.63	31	16	9
Chr23	12129150	C	A	Chr23_12129	C_A	SNP	12561.63	19	11	7
Chr23	12129150	C	A	Chr23_12129	C_A	SNP	12561.63	19	11	7
Chr23	12129150	C	A	Chr23_12129	C_A	SNP	12561.63	19	11	7
Chr23	12129150	C	A	Chr23_12129	C_A	SNP	12561.63	19	11	7
Chr23	12129150	C	A	Chr23_12129	C_A	SNP	12561.63	19	11	7
Chr23	12129150	C	A	Chr23_12129	C_A	SNP	12561.63	19	11	7
Chr23	12129150	C	A	Chr23_12129	C_A	SNP	12561.63	19	11	7
Chr23	12190734	C	A	Chr23_12190	C_A	SNP	19984.63	29	10	11

[illegible]

Chr23	12244566	T	G	Chr23_12244T_G	SNP	15904.63	15	14	15	23	18	19	13	26	16	23	15	15	11	9	15	14	15	23	18	19	13	26	16	23	15	15	11	9	1	1	1	1	1	1	1
Chr23	12250152	A	AAAT	Chr23_12250A_AAAT	INDEL	3065.93	0	0	0	0	0	0	0	0	11	14	11	6	9	6	20	13	9	19	19	17	25	14	11	14	11	6	9	6	0	0	0	0	0	0	0
Chr23	12366739	G	GAT	Chr23_12366G_GAT	INDEL	3161.24	0	0	0	15	18	11	31	19	1	2	0	1	2	0	16	8	19	15	19	12	32	19	16	15	10	11	9	5	0	0	0	1	0.947	0.917	0.969
Chr23	12366739	G	GAT	Chr23_12366G_GAT	INDEL	3161.24	0	0	0	15	18	11	31	19	1	2	0	1	2	0	16	8	19	15	19	12	32	19	16	15	10	11	9	5	0	0	0	1	0.947	0.917	0.969
Chr23	12366739	G	GAT	Chr23_12366G_GAT	INDEL	3161.24	0	0	0	15	18	11	31	19	1	2	0	1	2	0	16	8	19	15	19	12	32	19	16	15	10	11	9	5	0	0	0	1	0.947	0.917	0.969
Chr23	12366739	G	GAT	Chr23_12366G_GAT	INDEL	3161.24	0	0	0	15	18	11	31	19	1	2	0	1	2	0	16	8	19	15	19	12	32	19	16	15	10	11	9	5	0	0	0	1	0.947	0.917	0.969
Chr23	12366739	G	GAT	Chr23_12366G_GAT	INDEL	3161.24	0	0	0	15	18	11	31	19	1	2	0	1	2	0	16	8	19	15	19	12	32	19	16	15	10	11	9	5	0	0	0	1	0.947	0.917	0.969
Chr23	12452127	T	TTA	Chr23_12452T_TTA	INDEL	2314.24	0	0	0	13	10	12	21	17	0	1	1	1	1	0	15	6	18	13	10	12	21	18	13	15	5	10	4	8	0	0	0	1	1	1	1
Chr23	12452127	T	TTA	Chr23_12452T_TTA	INDEL	2314.24	0	0	0	13	10	12	21	17	0	1	1	1	1	0	15	6	18	13	10	12	21	18	13	15	5	10	4	8	0	0	0	1	1	1	1
Chr23	12452127	T	TTA	Chr23_12452T_TTA	INDEL	2314.24	0	0	0	13	10	12	21	17	0	1	1	1	1	0	15	6	18	13	10	12	21	18	13	15	5	10	4	8	0	0	0	1	1	1	1
Chr23	12452127	T	TTA	Chr23_12452T_TTA	INDEL	2314.24	0	0	0	13	10	12	21	17	0	1	1	1	1	0	15	6	18	13	10	12	21	18	13	15	5	10	4	8	0	0	0	1	1	1	1
Chr23	12452127	T	TTA	Chr23_12452T_TTA	INDEL	2314.24	0	0	0	13	10	12	21	17	0	1	1	1	1	0	15	6	18	13	10	12	21	18	13	15	5	10	4	8	0	0	0	1	1	1	1
Chr23	12452127	T	TTA	Chr23_12452T_TTA	INDEL	2314.24	0	0	0	13	10	12	21	17	0	1	1	1	1	0	15	6	18	13	10	12	21	18	13	15	5	10	4	8	0	0	0	1	1	1	1
Chr23	12452127	T	TTA	Chr23_12452T_TTA	INDEL	2314.24	0	0	0	13	10	12	21	17	0	1	1	1	1	0	15	6	18	13	10	12	21	18	13	15	5	10	4	8	0	0	0	1	1	1	1
Chr23	12452127	T	TTA	Chr23_12452T_TTA	INDEL	2314.24	0	0	0	13	10	12	21	17	0	1	1	1	1	0	15	6	18	13	10	12	21	18	13	15	5	10	4	8	0	0	0	1	1	1	1
Chr23	12452127	T	TTA	Chr23_12452T_TTA	INDEL	2314.24	0	0	0	13	10	12	21	17	0	1	1	1	1	0	15	6	18	13	10	12	21	18	13	15	5	10	4	8	0	0	0	1	1	1	1
Chr23	12452127	T	TTA	Chr23_12452T_TTA	INDEL	2314.24	0	0	0	13	10	12	21	17	0	1	1	1	1	0	15	6	18	13	10	12	21	18	13	15	5	10	4	8	0	0	0	1	1	1	1
Chr23	12480321	C	CAT	Chr23_12480C_CAT	INDEL	3761.24	11	4	7	0	0	1	0	0	10	8	4	9	6	4	11	4	8	16	15	20	25	19	11	8	4	9	6	4	1	1	0.875	0	0	0	0
Chr23	12480321	C	CAT	Chr23_12480C_CAT	INDEL	3761.24	11	4	7	0	0	1	0	0	10	8	4	9	6	4	11	4	8	16	15	20	25	19	11	8	4	9	6	4	1	1	0.875	0	0	0	0
Chr23	12480321	C	CAT	Chr23_12480C_CAT	INDEL	3761.24	11	4	7	0	0	1	0	0	10	8	4	9	6	4	11	4	8	16	15	20	25	19	11	8	4	9	6	4	1	1	0.875	0	0	0	0
Chr23	12480321	C	CAT	Chr23_12480C_CAT	INDEL	3761.24	11	4	7	0	0	1	0	0	10	8	4	9	6	4	11	4	8	16	15	20	25	19	11	8	4	9	6	4	1	1	0.875	0	0	0	0
Chr23	12973270	T	TA	Chr23_12973T_TA	INDEL	6924.59	14	7	7	7	9	15	6	13	11	15	5	5	11	7	14	7	7	7	9	15	6	13	11	15	5	5	11	7	1	1	1	1	1	1	1
Chr23	13262093	A	AT	Chr23_13262A_AT	INDEL	16927.59	32	15	17	18	30	28	40	22	34	23	20	18	6	15	32	15	17	18	30	28	40	22	34	23	20	18	6	15	1	1	1	1	1	1	1
Chr23	13262093	A	AT	Chr23_13262A_AT	INDEL	16927.59	32	15	17	18	30	28	40	22	34	23	20	18	6	15	32	15	17	18	30	28	40	22	34	23	20	18	6	15	1	1	1	1	1	1	1
Chr23	13262093	A	AT	Chr23_13262A_AT	INDEL	16927.59	32	15	17	18	30	28	40	22	34	23	20	18	6	15	32	15	17	18	30	28	40	22	34	23	20	18	6	15	1	1	1	1	1	1	1
Chr23	13262093	A	AT	Chr23_13262A_AT	INDEL	16927.59	32	15	17	18	30	28	40	22	34	23	20	18	6	15	32	15	17	18	30	28	40	22	34	23	20	18	6	15	1	1	1	1	1	1	1
Chr23	13274532	G	A	Chr23_13274G_A	SNP	16229.63	31	10	16	31	30	19	29	17	15	17	5	8	6	10	31	10	16	31	31	19	29	17	15	17	5	8	6	10	1	1	1	1	0.968	1	1
Chr23	13274532	G	A	Chr23_13274G_A	SNP	16229.63	31	10	16	31	30	19	29	17	15	17	5	8	6	10	31	10	16	31	31	19	29	17	15	17	5	8	6	10	1	1	1	1	0.968	1	1
Chr23	13274532	G	A	Chr23_13274G_A	SNP	16229.63	31	10	16	31	30	19	29	17	15	17	5	8	6	10	31	10	16	31	31	19	29	17	15	17	5	8	6	10	1	1	1	1	0.968	1	1
Chr23	13274532	G	A	Chr23_13274G_A	SNP	16229.63	31	10	16	31	30	19	29	17	15	17	5	8	6	10	31	10	16	31	31	19	29	17	15	17	5	8	6	10	1	1	1	1	0.968	1	1
Chr23	13274532	G	A	Chr23_13274G_A	SNP	16229.63	31	10	16	31	30	19	29	17	15	17	5	8	6	10	31	10	16	31	31	19	29	17	15	17	5	8	6	10	1	1	1	1	0.968	1	1
Chr23	13274532	G	A	Chr23_13274G_A	SNP	16229.63	31	10	16	31	30	19	29	17	15	17	5	8	6	10	31	10	16	31	31	19	29	17	15	17	5	8	6	10	1	1	1	1	0.968	1	1
Chr23	13274532	G	A	Chr23_13274G_A	SNP	16229.63	31	10	16	31	30	19	29	17	15	17	5	8	6	10	31	10	16	31	31	19	29	17	15	17	5	8	6	10	1	1	1	1	0.968	1	1
Chr23	13431661	G	T	Chr23_13431G_T	SNP	13240.63	15	19	14	8	17	23	25	15	16	29	14	13	11	13	16	19	14	8	17	23	25	15	16	29	14	13	11	13	0.938	1	1	1	1	1	1
Chr23	13441809	T	TA	Chr23_13441T_TA	INDEL	14957.59	29	9	16	22	22	19	17	14	25	22	20	22	11	10	29	9	16	22	22	19	17	14	25	22	20	22	11	10	1	1	1	1	1	1	1
Chr23	13476168	A	AAT	Chr23_13476A_AAT	INDEL	2494.24	2	0	0	10	7	22	18	13	1	0	0	0	0	0	22	11	9	10	7	22	18	13	11	4	11	12	5	7	0.091	0	0	1	1	1	1
Chr23	13476168	A	AAT	Chr23_13476A_AAT	INDEL	2494.24	2	0	0	10	7	22	18	13	1	0	0	0	0	0	22	11	9	10	7	22	18	13	11	4	11	12	5	7	0.091	0	0	1	1	1	1
Chr23	13476168	A	AAT	Chr23_13476A_AAT	INDEL	2494.24	2	0	0	10	7	22	18	13	1	0	0	0	0	0	22	11	9	10	7	22	18	13	11	4	11	12	5	7	0.091	0	0	1	1	1	1
Chr23	13476168	A	AAT	Chr23_13476A_AAT	INDEL	2494.24	2	0	0	10	7	22	18	13	1	0	0	0	0	0	22	11	9	10	7	22	18	13	11	4	11	12	5	7	0.091	0	0	1	1	1	1
Chr23	13534559	G	C	Chr23_13534G_C	SNP	13266.63	22	9	16	16	19	18	32	8	11	15	9	11	6	7	22	9	16	16	19	18	32	8	11	15	9	11	6	7	1	1	1	1	1	1	1
Chr23	13535135	A	T	Chr23_13535A_T	SNP	13978.63	22	8	15	15	15	23	26	12	14	20	11	11	12	7	22	8	15	15	15	23	26	12	14	20	11	11	12	7	1	1	1	1	1	1	1
Chr23	13535163	G	C	Chr23_13535G_C	SNP	14228.63	23	9	15	10	16	26	27	16	10	18	13	14	12	9	23	9	15	10	16	26	27	16	10	18	13	14	12	9	1	1	1	1	1	1	1
Chr23	13535189	G	C	Chr23_13535G_C	SNP	14806.63	20	8	13	9	19	28	24	15	11	28	13	17	13	6	20	8	13	9	19	28	24	15	11	28	13	17	13	6	1	1	1	1	1	1	1
Chr23	13535205	T	A	Chr23_13535T_A	SNP	14071.63	19	5	10	7	18	28	21	15	12	29	12	17	13	6	19	5	10	7	18	28	21	15	13	29	12	17	13	6	1	1	1	1	1	1	1
Chr23	13566832	G	T	Chr23_13566G_T	SNP	6306.29	0	0	0	31	22	23	22	30	0	0	0	0	0	0	19	15	17	31	22	23	22	30	40	30	23	26	12	6	0	0	0	1	1	1	1
Chr23	13598266	C	A	Chr23_13598C_A	SNP	3462.98	0	0	0	0	0	0	0	0	13	21	14	14	13	7	24	16	16	21	16	20	33	19	13	22	14	14	13	7	0	0	0	0	0	0	0
Chr23	13598266	C	A	Chr23_13598C_A	SNP	3462.98	0	0	0	0	0	0	0	0	13	21	14	14	13	7	24	16	16	21	16	20	33	19	13	22	14	14	13	7	0	0	0	0	0	0	0

Chr24	1475132	C	G	Chr24_14751 C_G	SNP	18820.63	21	15	13	19	15	25
Chr24	1475132	C	G	Chr24_14751 C_G	SNP	18820.63	21	15	13	19	15	25
Chr24	1475132	C	G	Chr24_14751 C_G	SNP	18820.63	21	15	13	19	15	25
Chr24	1475132	C	G	Chr24_14751 C_G	SNP	18820.63	21	15	13	19	15	25
Chr24	1475132	C	G	Chr24_14751 C_G	SNP	18820.63	21	15	13	19	15	25
Chr24	1475132	C	G	Chr24_14751 C_G	SNP	18820.63	21	15	13	19	15	25
Chr24	1475132	C	G	Chr24_14751 C_G	SNP	18820.63	21	15	13	19	15	25
Chr24	1475132	C	G	Chr24_14751 C_G	SNP	18820.63	21	15	13	19	15	25
Chr24	1475132	C	G	Chr24_14751 C_G	SNP	18820.63	21	15	13	19	15	25
Chr24	1481079	A	AAT	Chr24_14810 A_AAT	INDEL	2790.93	1	0	0	0	0	0
Chr24	1481079	A	AAT	Chr24_14810 A_AAT	INDEL	2790.93	1	0	0	0	0	0
Chr24	1481079	A	AAT	Chr24_14810 A_AAT	INDEL	2790.93	1	0	0	0	0	0
Chr24	1481079	A	AAT	Chr24_14810 A_AAT	INDEL	2790.93	1	0	0	0	0	0
Chr24	1581479	A	AT	Chr24_15814 A_AT	INDEL	11459.59	19	7	14	12	15	31
Chr24	1782511	A	AT	Chr24_17825 A_AT	INDEL	14543.59	28	14	17	35	21	28
Chr24	1969145	C	A	Chr24_19691 C_A	SNP	16502.63	27	9	13	27	18	26
Chr24	2055666	AC	A	Chr24_20556 AC_A	INDEL	6221.59	12	5	6	7	4	6
Chr24	2164754	G	GAT	Chr24_21647 G_GAT	INDEL	9559.59	23	14	14	13	21	9
Chr24	2164754	G	GAT	Chr24_21647 G_GAT	INDEL	9559.59	23	14	14	13	21	9
Chr24	2164754	G	GAT	Chr24_21647 G_GAT	INDEL	9559.59	23	14	14	13	21	9
Chr24	2164754	G	GAT	Chr24_21647 G_GAT	INDEL	9559.59	23	14	14	13	21	9
Chr24	2275194	C	A	Chr24_22751 C_A	SNP	17767.63	23	15	10	22	22	29
Chr24	2275253	AT	A	Chr24_22752 AT_A	INDEL	14978.59	18	13	18	19	21	24
Chr24	2289439	G	T	Chr24_22894 G_T	SNP	11807.63	24	10	12	26	22	13

Chr24	6410064	C	G	Chr24_64100C_G	SNP	21487.63	[illegible]
Chr24	6427642	A	AAT	Chr24_64276A_AAT	INDEL	6718.59	[illegible]
Chr24	6427642	A	AAT	Chr24_64276A_AAT	INDEL	6718.59	[illegible]
Chr24	6427642	A	AAT	Chr24_64276A_AAT	INDEL	6718.59	[illegible]
Chr24	6427642	A	AAT	Chr24_64276A_AAT	INDEL	6718.59	[illegible]
Chr24	6427642	A	AAT	Chr24_64276A_AAT	INDEL	6718.59	[illegible]
Chr24	6427642	A	AAT	Chr24_64276A_AAT	INDEL	6718.59	[illegible]
Chr24	6427642	A	AAT	Chr24_64276A_AAT	INDEL	6718.59	[illegible]
Chr24	6461296	C	A	Chr24_64612C_A	SNP	22451.63	[illegible]
Chr24	6461473	G	T	Chr24_64614G_T	SNP	19808.63	[illegible]
Chr24	6479997	TA	T	Chr24_64799TA_T	INDEL	22652.59	[illegible]
Chr24	6479997	TA	T	Chr24_64799TA_T	INDEL	22652.59	[illegible]
Chr24	6479997	TA	T	Chr24_64799TA_T	INDEL	22652.59	[illegible]
Chr24	6479997	TA	T	Chr24_64799TA_T	INDEL	22652.59	[illegible]
Chr24	6480004	A	T	Chr24_64800A_T	SNP	23007.63	[illegible]
Chr24	6480004	A	T	Chr24_64800A_T	SNP	23007.63	[illegible]
Chr24	6480004	A	T	Chr24_64800A_T	SNP	23007.63	[illegible]
Chr24	6480004	A	T	Chr24_64800A_T	SNP	23007.63	[illegible]
Chr24	6480007	T	A	Chr24_64800T_A	SNP	22790.63	[illegible]
Chr24	6480007	T	A	Chr24_64800T_A	SNP	22790.63	[illegible]
Chr24	6480007	T	A	Chr24_64800T_A	SNP	22790.63	[illegible]
Chr24	6480007	T	A	Chr24_64800T_A	SNP	22790.63	[illegible]
Chr24	6568707	A	G	Chr24_65687A_G	SNP	14742.63	[illegible]
Chr24	6568707	A	G	Chr24_65687A_G	SNP	14742.63	[illegible]
Chr24	6568707	A	G	Chr24_65687A_G	SNP	14742.63	[illegible]
Chr24	6914414	C	A	Chr24_69144C_A	SNP	2147.39	[illegible]
Chr24	6937804	T	TTA	Chr24_69378T_TTA	INDEL	719.37	[illegible]
Chr24	6937804	T	TTA	Chr24_69378T_TTA	INDEL	719.37	[illegible]
Chr24	6937804	T	TTA	Chr24_69378T_TTA	INDEL	719.37	[illegible]
Chr24	6937804	T	TTA	Chr24_69378T_TTA	INDEL	719.37	[illegible]
Chr24	6998169	G	T	Chr24_69981G_T	SNP	17702.63	[illegible]
Chr24	7034794	T	A	Chr24_70347T_A	SNP	16928.63	[illegible]
Chr24	7036331	GCT	G	Chr24_70363GCT_G	INDEL	17858.59	[illegible]
Chr24	7036333	T	TAAGA	Chr24_70363T_TAAGA	INDEL	17858.59	[illegible]
Chr24	7041316	G	C	Chr24_70413G_C	SNP	12714.63	[illegible]
Chr24	7043130	AC	A	Chr24_70431AC_A	INDEL	7479.59	[illegible]
Chr24	7043133	C	G	Chr24_70431C_G	SNP	7488.63	[illegible]
Chr24	7043135	CTTTTTATTG	C	Chr24_70431CTTTTTATTG	INDEL	6738.37	[illegible]
Chr24	7051324	G	T	Chr24_70513G_T	SNP	2207.39	[illegible]
Chr24	7051324	G	T	Chr24_70513G_T	SNP	2207.39	[illegible]
Chr24	7055372	C	T	Chr24_70553C_T	SNP	17923.63	[illegible]
Chr24	7055387	G	T	Chr24_70553G_T	SNP	18398.63	[illegible]
Chr24	7113528	T	C	Chr24_71135T_C	SNP	8633.63	[illegible]
Chr24	7114318	A	G	Chr24_71143A_G	SNP	14615.63	[illegible]
Chr24	7114345	GA	G	Chr24_71143GA_G	INDEL	12180.59	[illegible]
Chr24	7114351	C	CA	Chr24_71143C_CA	INDEL	12672.59	[illegible]
Chr24	7122805	T	TA	Chr24_71228T_TA	INDEL	18647.59	[illegible]
Chr24	7122833	C	A	Chr24_71228C_A	SNP	19708.63	[illegible]
Chr24	7234960	C	A	Chr24_72349C_A	SNP	17256.63	[illegible]
Chr24	7317730	C	T	Chr24_73177C_T	SNP	20687.63	[illegible]
Chr24	7698217	C	G	Chr24_76982C_G	SNP	13509.63	[illegible]
Chr24	7698217	C	G	Chr24_76982C_G	SNP	13509.63	[illegible]
Chr24	7698217	C	G	Chr24_76982C_G	SNP	13509.63	[illegible]
Chr24	7698217	C	G	Chr24_76982C_G	SNP	13509.63	[illegible]
Chr24	7698217	C	G	Chr24_76982C_G	SNP	13509.63	[illegible]
Chr24	7772813	C	CCAAATCTTA	Chr24_77728C_CCAAATCT	INDEL	19059.59	[illegible]
Chr24	7862390	AC	A	Chr24_78623AC_A	INDEL	20094.59	[illegible]
Chr24	7862390	AC	A	Chr24_78623AC_A	INDEL	20094.59	[illegible]
Chr24	7862390	AC	A	Chr24_78623AC_A	INDEL	20094.59	[illegible]
Chr24	7862390	AC	A	Chr24_78623AC_A	INDEL	20094.59	[illegible]
Chr24	8013001	TC	T	Chr24_80130TC_T	INDEL	5933.59	[illegible]
Chr24	8304824	AAT	A	Chr24_83048AAT_A	INDEL	2475.93	[illegible]
Chr24	8324441	G	C	Chr24_83244G_C	SNP	17554.63	[illegible]
Chr24	8343483	G	GT	Chr24_83434G_GT	INDEL	16384.59	[illegible]
Chr24	8343483	G	GT	Chr24_83434G_GT	INDEL	16384.59	[illegible]
Chr24	8570408	ACTGG	A	Chr24_85704ACTGG_A	INDEL	20270.59	[illegible]
Chr24	8570408	ACTGG	A	Chr24_85704ACTGG_A	INDEL	20270.59	[illegible]
Chr24	8570408	ACTGG	A	Chr24_85704ACTGG_A	INDEL	20270.59	[illegible]
Chr24	8570408	ACTGG	A	Chr24_85704ACTGG_A	INDEL	20270.59	[illegible]
Chr24	8570408	ACTGG	A	Chr24_85704ACTGG_A	INDEL	20270.59	[illegible]
Chr24	8570408	ACTGG	A	Chr24_85704ACTGG_A	INDEL	20270.59	[illegible]
Chr24	8570413	A	AGCAC	Chr24_85704A_AGCAC	INDEL	20307.59	[illegible]
Chr24	8570413	A	AGCAC	Chr24_85704A_AGCAC	INDEL	20307.59	[illegible]
Chr24	8570413	A	AGCAC	Chr24_85704A_AGCAC	INDEL	20307.59	[illegible]
Chr24	8570413	A	AGCAC	Chr24_85704A_AGCAC	INDEL	20307.59	[illegible]
Chr24	8570413	A	AGCAC	Chr24_85704A_AGCAC	INDEL	20307.59	[illegible]
Chr24	8570413	A	AGCAC	Chr24_85704A_AGCAC	INDEL	20307.59	[illegible]
Chr24	8570415	A	T	Chr24_85704A_T	SNP	20316.63	[illegible]
Chr24	8570415	A	T	Chr24_85704A_T	SNP	20316.63	[illegible]
Chr24	8570415	A	T	Chr24_85704A_T	SNP	20316.63	[illegible]
Chr24	8570415	A	T	Chr24_85704A_T	SNP	20316.63	[illegible]
Chr24	8570415	A	T	Chr24_85704A_T	SNP	20316.63	[illegible]
Chr24	8570415	A	T	Chr24_85704A_T	SNP	20316.63	[illegible]
Chr24	8857549	T	TGAGCCTCCT	Chr24_88575T_TGAGCCT	INDEL	19941.59	[illegible]
Chr24	8857549	T	TGAGCCTCCT	Chr24_88575T_TGAGCCT	INDEL	19941.59	[illegible]
Chr24	8857549	T	TGAGCCTCCT	Chr24_88575T_TGAGCCT	INDEL	19941.59	[illegible]
Chr24	8857549	T	TGAGCCTCCT	Chr24_88575T_TGAGCCT	INDEL	19941.59	[illegible]
Chr24	8857549	T	TGAGCCTCCT	Chr24_88575T_TGAGCCT	INDEL	19941.59	[illegible]
Chr24	8857549	T	TGAGCCTCCT	Chr24_88575T_TGAGCCT	INDEL	19941.59	[illegible]
Chr24	8857549	T	TGAGCCTCCT	Chr24_88575T_TGAGCCT	INDEL	19941.59	[illegible]
Chr24	8857549	T	TGAGCCTCCT	Chr24_88575T_TGAGCCT	INDEL	19941.59	[illegible]
Chr24	8857553	C	CCAAAT	Chr24_88575C_CCAAAT	INDEL	20216.59	[illegible]
Chr24	8857553	C	CCAAAT	Chr24_88575C_CCAAAT	INDEL	20216.59	[illegible]
Chr24	8857553	C	CCAAAT	Chr24_88575C_CCAAAT	INDEL	20216.59	[illegible]
Chr24	8857553	C	CCAAAT	Chr24_88575C_CCAAAT	INDEL	20216.59	[illegible]
Chr24	8857553	C	CCAAAT	Chr24_88575C_CCAAAT	INDEL	20216.59	[illegible]
Chr24	8857553	C	CCAAAT	Chr24_88575C_CCAAAT	INDEL	20216.59	[illegible]
Chr24	8857553	C	CCAAAT	Chr24_88575C_CCAAAT	INDEL	20216.59	[illegible]
Chr24	8857553	C	CCAAAT	Chr24_88575C_CCAAAT	INDEL	20216.59	[illegible]
Chr24	8857555	G	A	Chr24_88575G_A	SNP	20225.63	[illegible]
Chr24	8857555	G	A	Chr24_88575G_A	SNP	20225.63	[illegible]
Chr24	8857555	G	A	Chr24_88575G_A	SNP	20225.63	[illegible]
Chr24	8857555	G	A	Chr24_88575G_A	SNP	20225.63	[illegible]
Chr24	8857555	G	A	Chr24_88575G_A	SNP	20225.63	[illegible]
Chr24	8857555	G	A	Chr24_88575G_A	SNP	20225.63	[illegible]
Chr24	8857555	G	A	Chr24_88575G_A	SNP	20225.63	[illegible]
Chr24	8857555	G	A	Chr24_88575G_A	SNP	20225.63	[illegible]
Chr24	8857557	GT	G	Chr24_88575GT_G	INDEL	19406.59	[illegible]
Chr24	8857557	GT	G	Chr24_88575GT_G	INDEL	19406.59	[illegible]
Chr24	8857557	GT	G	Chr24_88575GT_G	INDEL	19406.59	[illegible]
Chr24	8857557	GT	G	Chr24_88575GT_G	INDEL	19406.59	[illegible]
Chr24	8857557	GT	G	Chr24_88575GT_G	INDEL	19406.59	[illegible]
Chr24	8857557	GT	G	Chr24_88575GT_G	INDEL	19406.59	[illegible]
Chr24	8857557	GT	G	Chr24_88575GT_G	INDEL	19406.59	[illegible]
Chr24	8857557	GT	G	Chr24_88575GT_G	INDEL	19406.59	[illegible]
Chr24	8857561	G	GAAGA	Chr24_88575G_GAAGA	INDEL	19946.59	[illegible]
Chr24	8857561	G	GAAGA	Chr24_88575G_GAAGA	INDEL	19946.59	[illegible]
Chr24	8857561	G	GAAGA	Chr24_88575G_GAAGA	INDEL	19946.59	[illegible]
Chr24	8857561	G	GAAGA	Chr24_88575G_GAAGA	INDEL	19946.59	[illegible]
Chr24	8857561	G	GAAGA	Chr24_88575G_GAAGA	INDEL	19946.59	[illegible]
Chr24	8857561	G	GAAGA	Chr24_88575G_GAAGA	INDEL	19946.59	[illegible]
Chr24	8857561	G	GAAGA	Chr24_88575G_GAAGA	INDEL	19946.59	[illegible]
Chr24	8857561	G	GAAGA	Chr24_88575G_GAAGA	INDEL	19946.59	[illegible]
Chr24	8857565	CCCGACTTCT	C	Chr24_88575CCCGACTTCT	INDEL	18821.59	[illegible]
Chr24	8857565	CCCGACTTCT	C	Chr24_88575CCCGACTTCT	INDEL	18821.59	[illegible]
Chr24	8857565	CCCGACTTCT	C	Chr24_88575CCCGACTTCT	INDEL	18821.59	[illegible]
Chr24	8857565	CCCGACTTCT	C	Chr24_88575CCCGACTTCT	INDEL	18821.59	[illegible]
Chr24	8857565	CCCGACTTCT	C	Chr24_88575CCCGACTTCT	INDEL	18821.59	[illegible]
Chr24	8857565	CCCGACTTCT	C	Chr24_88575CCCGACTTCT	INDEL	18821.59	[illegible]
Chr24	8857565	CCCGACTTCT	C	Chr24_88575CCCGACTTCT	INDEL	18821.59	[illegible]
Chr24	8857565	CCCGACTTCT	C	Chr24_88575CCCGACTTCT	INDEL	18821.59	[illegible]
Chr24	8857580	A	C	Chr24_88575A_C	SNP	18785.63	[illegible]
Chr24	8857580	A	C	Chr24_88575A_C	SNP	18785.63	[illegible]
Chr24	8857580	A	C	Chr24_88575A_C	SNP	18785.63	[illegible]
Chr24	8857580	A	C	Chr24_88575A_C	SNP	18785.63	[illegible]
Chr24	8857580	A	C	Chr24_88575A_C	SNP	18785.63	[illegible]
Chr24	8857580	A	C	Chr24_88575A_C	SNP	18785.63	[illegible]
Chr24	8857580	A	C	Chr24_88575A_C	SNP	18785.63	[illegible]
Chr24	8857580	A	C	Chr24_88575A_C	SNP	18785.63	[illegible]
Chr24	8857584	TCACC	T	Chr24_88575TCACC_T	INDEL	18821.59	[illegible]
Chr24	8857584	TCACC	T	Chr24_88575TCACC_T	INDEL	18821.59	[illegible]
Chr24	8857584	TCACC	T	Chr24_88575TCACC_T	INDEL	18821.59	[illegible]
Chr24	8857584	TCACC	T	Chr24_88575TCACC_T	INDEL	18821.59	[illegible]
Chr24	8857584	TCACC	T	Chr24_88575TCACC_T	INDEL	18821.59	[illegible]
Chr24	8857584	TCACC	T	Chr24_88575TCACC_T	INDEL	18821.59	[illegible]
Chr24	8857584	TCACC	T	Chr24_88575TCACC_T	INDEL	18821.59	[illegible]
Chr24	8857584	TCACC	T	Chr24_88575TCACC_T	INDEL	18821.59	[illegible]
Chr24	8857589	A	AT	Chr24_88575A_AT	INDEL	18641.59	[illegible]
Chr24	8857589	A	AT	Chr24_88575A_AT	INDEL	18641.59	[illegible]
Chr24	8857589	A	AT	Chr24_88575A_AT	INDEL	18641.59	[illegible]
Chr24	8857589	A	AT	Chr24_88575A_AT	INDEL	18641.59	[illegible]
Chr24	8857589	A	AT	Chr24_88575A_AT	INDEL	18641.59	[illegible]
Chr24	8857589	A	AT	Chr24_88575A_AT	INDEL	18641.59	[illegible]
Chr24	8857589	A	AT	Chr24_88575A_AT	INDEL	18641.59	[illegible]
Chr24	8857589	A	AT	Chr24_88575A_AT	INDEL	18641.59	[illegible]
Chr24	8857592	TGCGGCGAG	T	Chr24_88575TGCGGCGAG	INDEL	18506.59	[illegible]
Chr24	8857592	TGCGGCGAG	T	Chr24_88575TGCGGCGAG	INDEL	18506.59	[illegible]
Chr24	8857592	TGCGGCGAG	T	Chr24_88575TGCGGCGAG	INDEL	18506.59	[illegible]
Chr24	8857592	TGCGGCGAG	T	Chr24_88575TGCGGCGAG	INDEL	18506.59	[illegible]
Chr24	8857592	TGCGGCGAG	T	Chr24_88575TGCGGCGAG	INDEL	18506.59	[illegible]
Chr24	8857592	TGCGGCGAG	T	Chr24_88575TGCGGCGAG	INDEL	18506.59	[illegible]
Chr24	8857592	TGCGGCGAG	T	Chr24_88575TGCGGCGAG	INDEL	18506.59	[illegible]
Chr24	8857592	TGCGGCGAG	T	Chr24_88575TGCGGCGAG	INDEL	18506.59	[illegible]
Chr24	8857603	G	T	Chr24_88576G_T	SNP	18380.63	[illegible]
Chr24	8857603	G	T	Chr24_88576G_T	SNP	18380.63	[illegible]
Chr24	8857603	G	T	Chr24_88576G_T	SNP	18380.63	[illegible]
Chr24	8857603	G	T	Chr24_88576G_T	SNP	18380.63	[illegible]
Chr24	8857603	G	T	Chr24_88576G_T	SNP	18380.63	[illegible]
Chr24	8857603	G	T	Chr24_88576G_T	SNP	18380.63	[illegible]
Chr24	8857603	G	T	Chr24_88576G_T	SNP	18380.63	[illegible]
Chr24	8857603	G	T	Chr24_88576G_T	SNP	18380.63	[illegible]
Chr24	8857605	A	AT	Chr24_88576A_AT	INDEL	18281.59	[illegible]
Chr24	8857605	A	AT	Chr24_88576A_AT	INDEL	18281.59	[illegible]
Chr24	8857605	A	AT	Chr24_88576A_AT	INDEL	18281.59	[illegible]
Chr24	8857605	A	AT	Chr24_88576A_AT	INDEL	18281.59	[illegible]
Chr24	8857605	A	AT	Chr24_88576A_AT	INDEL	18281.59	[illegible]
Chr24	8857605	A	AT	Chr24_88576A_AT	INDEL	18281.59	[illegible]
Chr24	8857605	A	AT	Chr24_88576A_AT	INDEL	18281.59	[illegible]
Chr24	8857605	A	AT	Chr24_88576A_AT	INDEL	18281.59	[illegible]
Chr24	8857606	AGC	A	Chr24_88576AGC_A	INDEL	19258.59	[illegible]
Chr24	8857606	AGC	A	Chr24_88576AGC_A	INDEL	19258.59	[illegible]
Chr24	8857606	AGC	A	Chr24_88576AGC_A	INDEL	19258.59	[illegible]
Chr24	8857606	AGC	A	Chr24_88576AGC_A	INDEL	19258.59	[illegible]
Chr24	8857606	AGC	A	Chr24_88576AGC_A	INDEL	19258.59	[illegible]
Chr24	8857606	AGC	A	Chr24_88576AGC_A	INDEL	19258.59	[illegible]
Chr24	8857606	AGC	A	Chr24_88576AGC_A	INDEL	19258.59	[illegible]
Chr24	8857606	AGC	A	Chr24_88576AGC_A	INDEL	19258.59	[illegible]
Chr24	8857609	C	CAGGGTTG	Chr24_88576C_CAGGGTT	INDEL	18718.59	[illegible]
Chr24	8857609	C	CAGGGTTG	Chr24_88576C_CAGGGTT	INDEL	18718.59	[illegible]
Chr24	8857609	C	CAGGGTTG	Chr24_88576C_CAGGGTT	INDEL	18718.59	[illegible]
Chr24	8857609	C	CAGGGTTG	Chr24_88576C_CAGGGTT	INDEL	18718.59	[illegible]
Chr24	8857609	C	CAGGGTTG	Chr24_88576C_CAGGGTT	INDEL	18718.59	[illegible]
Chr24	8857609	C	CAGGGTTG	Chr24_88576C_CAGGGTT	INDEL	18718.59	[illegible]
Chr24	8857609	C	CAGGGTTG	Chr24_88576C_CAGGGTT	INDEL	18718.59	[illegible]
Chr24	8857609	C	CAGGGTTG	Chr24_88576C_CAGGGTT	INDEL	18718.59	[illegible]
Chr24	8858701	T	TGCATA	Chr24_88587T_TGCATA	INDEL	193873.59	[illegible]
Chr24	8858701	T	TGCATA	Chr24_88587T_TGCATA	INDEL	193873.59	[illegible]
Chr24	8858701	T	TGCATA	Chr24_88587T_TGCATA	INDEL	193873.59	[illegible]
Chr24	8858701	T	TGCATA	Chr24_88587T_TGCATA	INDEL	193873.59	[illegible]
Chr24	8858701	T	TGCATA	Chr24_88587T_TGCATA	INDEL	193873.59	[illegible]
Chr24	8858701	T	TGCATA	Chr24_88587T_TGCATA	INDEL	193873.59	[illegible]
Chr24	8858701	T	TGCATA	Chr24_88587T_TGCATA	INDEL	193873.59	[illegible]

Chr24	8858701	T	TGCATA	Chr24_88587	T_TGCATA	INDEL	193873.59	316	178	161	272	226	316	373	249	256	260	150	163	119	120
Chr24	8858701	T	TGCATA	Chr24_88587	T_TGCATA	INDEL	193873.59	316	178	161	272	226	316	373	249	256	260	150	163	119	120
Chr24	8862678	G	GCCTGAACG	Chr24_88626	G_GCCTGAA	INDEL	81003.59	42	25	25	43	36	27	46	40	37	32	21	23	17	24
Chr24	8862678	G	GCCTGAACG	Chr24_88626	G_GCCTGAA	INDEL	81003.59	42	25	25	43	36	27	46	40	37	32	21	23	17	24
Chr24	8862678	G	GCCTGAACG	Chr24_88626	G_GCCTGAA	INDEL	81003.59	42	25	25	43	36	27	46	40	37	32	21	23	17	24
Chr24	8862678	G	GCCTGAACG	Chr24_88626	G_GCCTGAA	INDEL	81003.59	42	25	25	43	36	27	46	40	37	32	21	23	17	24
Chr24	8862678	G	GCCTGAACG	Chr24_88626	G_GCCTGAA	INDEL	81003.59	42	25	25	43	36	27	46	40	37	32	21	23	17	24
Chr24	8862678	G	GCCTGAACG	Chr24_88626	G_GCCTGAA	INDEL	81003.59	42	25	25	43	36	27	46	40	37	32	21	23	17	24
Chr24	8862678	G	GCCTGAACG	Chr24_88626	G_GCCTGAA	INDEL	81003.59	42	25	25	43	36	27	46	40	37	32	21	23	17	24
Chr24	8862778	CGGCATTCG	C	Chr24_88627	CGGCATTCG	INDEL	239164.59	373	188	215	372	273	330	434	326	301	301	154	174	119	130
Chr24	8862778	CGGCATTCG	C	Chr24_88627	CGGCATTCG	INDEL	239164.59	373	188	215	372	273	330	434	326	301	301	154	174	119	130
Chr24	8862778	CGGCATTCG	C	Chr24_88627	CGGCATTCG	INDEL	239164.59	373	188	215	372	273	330	434	326	301	301	154	174	119	130
Chr24	8862778	CGGCATTCG	C	Chr24_88627	CGGCATTCG	INDEL	239164.59	373	188	215	372	273	330	434	326	301	301	154	174	119	130
Chr24	8862778	CGGCATTCG	C	Chr24_88627	CGGCATTCG	INDEL	239164.59	373	188	215	372	273	330	434	326	301	301	154	174	119	130
Chr24	8862778	CGGCATTCG	C	Chr24_88627	CGGCATTCG	INDEL	239164.59	373	188	215	372	273	330	434	326	301	301	154	174	119	130
Chr24	8862778	CGGCATTCG	C	Chr24_88627	CGGCATTCG	INDEL	239164.59	373	188	215	372	273	330	434	326	301	301	154	174	119	130
Chr24	8992599	C	CT	Chr24_89925	C_CT	INDEL	20566.59	24	15	8	21	23	27	35	26	22	27	13	16	19	7
Chr24	8992599	C	CT	Chr24_89925	C_CT	INDEL	20566.59	24	15	8	21	23	27	35	26	22	27	13	16	19	7
Chr24	8992599	C	CT	Chr24_89925	C_CT	INDEL	20566.59	24	15	8	21	23	27	35	26	22	27	13	16	19	7
Chr24	8992606	G	A	Chr24_89926	G_A	SNP	21132.63	27	17	8	25	23	29	35	27	23	29	12	16	19	7
Chr24	8992606	G	A	Chr24_89926	G_A	SNP	21132.63	27	17	8	25	23	29	35	27	23	29	12	16	19	7
Chr24	8992606	G	A	Chr24_89926	G_A	SNP	21132.63	27	17	8	25	23	29	35	27	23	29	12	16	19	7
Chr24	8992620	G	T	Chr24_89926	G_T	SNP	20855.63	27	21	7	25	21	26	35	27	25	32	12	14	19	7
Chr24	8992620	G	T	Chr24_89926	G_T	SNP	20855.63	27	21	7	25	21	26	35	27	25	32	12	14	19	7
[illegible]																					

Chr24	10403555	G	T	Chr24_10403G_T	SNP	18294.63	18	16	18	21	23	23	23	21	21	26	14	19	5	11	18	16
Chr24	10403555	G	T	Chr24_10403G_T	SNP	18294.63	18	16	18	21	23	23	23	21	21	26	14	19	5	11	18	16
Chr24	10403555	G	T	Chr24_10403G_T	SNP	18294.63	18	16	18	21	23	23	23	21	21	26	14	19	5	11	18	16
Chr24	10403555	G	T	Chr24_10403G_T	SNP	18294.63	18	16	18	21	23	23	23	21	21	26	14	19	5	11	18	16
Chr24	10403555	G	T	Chr24_10403G_T	SNP	18294.63	18	16	18	21	23	23	23	21	21	26	14	19	5	11	18	16
Chr24	10403555	G	T	Chr24_10403G_T	SNP	18294.63	18	16	18	21	23	23	23	21	21	26	14	19	5	11	18	16
Chr24	10403555	G	T	Chr24_10403G_T	SNP	18294.63	18	16	18	21	23	23	23	21	21	26	14	19	5	11	18	16
Chr24	10403555	G	T	Chr24_10403G_T	SNP	18294.63	18	16	18	21	23	23	23	21	21	26	14	19	5	11	18	16
Chr24	10403555	G	T	Chr24_10403G_T	SNP	18294.63	18	16	18	21	23	23	23	21	21	26	14	19	5	11	18	16
Chr24	10403555	G	T	Chr24_10403G_T	SNP	18294.63	18	16	18	21	23	23	23	21	21	26	14	19	5	11	18	16
Chr24	10403555	G	T	Chr24_10403G_T	SNP	18294.63	18	16	18	21	23	23	23	21	21	26	14	19	5	11	18	16
Chr24	10403555	G	T	Chr24_10403G_T	SNP	18294.63	18	16	18	21	23	23	23	21	21	26	14	19	5	11	18	16
Chr24	10403555	G	T	Chr24_10403G_T	SNP	18294.63	18	16	18	21	23	23	23	21	21	26	14	19	5	11	18	16
Chr24	10403555	G	T	Chr24_10403G_T	SNP	18294.63	18	16	18	21	23	23	23	21	21	26	14	19	5	11	18	16
Chr24	10403555	G	T	Chr24_10403G_T	SNP	18294.63	18	16	18	21	23	23	23	21	21	26	14	19	5	11	18	16
Chr24	10403555	G	T	Chr24_10403G_T	SNP	18294.63	18	16	18	21	23	23	23	21	21	26	14	19	5	11	18	16
Chr24	10403555	G	T	Chr24_10403G_T	SNP	18294.63	18	16	18	21	23	23	23	21	21	26	14	19	5	11	18	16
Chr24	10403555	G	T	Chr24_10403G_T	SNP	18294.63	18	16	18	21	23	23	23	21	21	26	14	19	5	11	18	16
Chr24	10403555	G	T	Chr24_10403G_T	SNP	18294.63	18	16	18	21	23	23	23	21	21	26	14	19	5	11	18	16
Chr24	10403555	G	T	Chr24_10403G_T	SNP	18294.63	18	16	18	21	23	23	23	21	21	26	14	19	5	11	18	16
Chr24	10535500	A	AAT	Chr24_10535A_AAT	INDEL	3186.24	0	3	2	16	15	20	31	20	1	1	0	0	1	0	25	16
Chr24	10539757	GGGGAT	G	Chr24_10539GGGGAT_G	INDEL	12650.59	19	11	13	10	15	18	17	15	8	14	9	9	4	8	19	11
Chr24	10539765	A	G	Chr24_10539A_G	SNP	12845.63	20	12	12	10	15	20	17	14	8	14	10	9	4	8	20	12
Chr24	10539766	C	CT	Chr24_10539C_CT	INDEL	12971.59	20	12	12	10	16	21	17	14	8	15	10	9	4	8	20	12
Chr24	10542755	G	GAT	Chr24_10542G_GAT	INDEL	1068.35	22	9	8	0	0	0	2	0	0	0	0	0	1	0	22	10
Chr24	10596515	A	AAT	Chr24_10596A_AAT	INDEL	1315.35	20	15	12	0	1	0	0	0	0	0	0	1	0	0	20	15
Chr24	10596515	A	AAT	Chr24_10596A_AAT	INDEL	1315.35	20	15	12	0	1	0	0	0	0	0	0	1	0	0	20	15
Chr24	10596515	A	AAT	Chr24_10596A_AAT	INDEL	1315.35	20	15	12	0	1	0	0	0	0	0	0	1	0	0	20	15
Chr24	10596515	A	AAT	Chr24_10596A_AAT	INDEL	1315.35	20	15	12	0	1	0	0	0	0	0	0	1	0	0	20	15
Chr24	10596515	A	AAT	Chr24_10596A_AAT	INDEL	1315.35	20	15	12	0	1	0	0	0	0	0	0	1	0	0	20	15
Chr24	10596515	A	AAT	Chr24_10596A_AAT	INDEL	1315.35	20	15	12	0	1	0	0	0	0	0	0	1	0	0	20	15
Chr24	10596515	A	AAT	Chr24_10596A_AAT	INDEL	1315.35	20	15	12	0	1	0	0	0	0	0	0	1	0	0	20	15
Chr24	10653666	G	GA	Chr24_10653G_GA	INDEL	14262.59	29	9	17	22	17	27	26	23	21	15	9	19	7	13	29	9
Chr24	10653666	G	GA	Chr24_10653G_GA	INDEL	14262.59	29	9	17	22	17	27	26	23	21	15	9	19	7	13	29	9
Chr24	10653666	G	GA	Chr24_10653G_GA	INDEL	14262.59	29	9	17	22	17	27	26	23	21	15	9	19	7	13	29	9
Chr24	10653666	G	GA	Chr24_10653G_GA	INDEL	14262.59	29	9	17	22	17	27	26	23	21	15	9	19	7	13	29	9
Chr24	11042072	G	C	Chr24_11042G_C	SNP	16627.63	23	8	15	27	20	25	30	15	24	23	16	11	8	8	23	8
Chr24	11265847	C	G	Chr24_11265C_G	SNP	10639.29	27	12	11	0	0	0	0	0	13	25	23	15	6	11	27	12
Chr24	11643283	C	T	Chr24_11643C_T	SNP	7673.63	10	8	5	7	8	5	15	8	8	5	6	7	5	4	10	8
Chr24	11643285	A	G	Chr24_11643A_G	SNP	7673.63	10	8	5	7	8	5	15	7	8	5	5	5	5	4	10	8
Chr24	11684550	T	C	Chr24_11684T_C	SNP	19827.63	19	28	16	24	17	16	17	14	22	31	24	14	17	9	19	28
Chr24	11684551	C	A	Chr24_11684C_A	SNP	19827.63	19	28	16	24	17	17	17	14	22	31	25	15	17	9	19	28
Chr24	11684633	AT	A	Chr24_11684AT_A	INDEL	14378.59	23	24	13	23	20	20	17	8	13	29	19	24	20	9	23	24
Chr24	11701557	C	T	Chr24_11701C_T	SNP	4204.98	0	0	0	0	0	0	0	0	20	27	10	14	14	11	36	13
Chr24	11763611	A	G	Chr24_11763A_G	SNP	13819.63	13	10	15	19	25	27	32	16	18	12	9	8	6	11	13	10
Chr24	11780321	TA	T	Chr24_11780TA_T	INDEL	15651.59	25	11	15	22	24	28	22	26	29	29	14	16	7	8	25	11
Chr24	11780357	A	T	Chr24_11780A_T	SNP	18786.63	26	13	14	22	18	26	25	26	24	33	11	20	10	9	26	13
Chr24	11807247	AT	A	Chr24_11807AT_A	INDEL	9285.59	14	8	6	15	9	15	9	17	11	14	10	16	5	5	14	8
Chr24	11813404	T	TA	Chr24_11813T_TA	INDEL	13888.59	28	7	16	32	25	24	20	28	16	17	11	13	11	12	28	7
Chr24	11960924	T	TTA	Chr24_11960T_TTA	INDEL	1259.35	18	10	13	1	0	0	0	0	1	1	0	0	0	1	18	10
Chr24	11966502	A	T	Chr24_11966A_T	SNP	3995.98	0	0	0	0	0	0	0	0	23	17	12	20	9	11	18	14
Chr24	12022442	C	A	Chr24_12022C_A	SNP	18434.63	18	23	12	26	24	29	23	24	18	20	14	23	11	6	18	23
Chr24	12127865	A	G	Chr24_12127A_G	SNP	9582.63	12	5	6	11	10	11	20	9	9	7	6	13	4	4	12	5
Chr24	12153673	A	G	Chr24_12153A_G	SNP	1667.39	15	18	10	0	0	0	0	0	0	0	0	0	0	0	15	18
Chr24	12211680	G	T	Chr24_12211G_T	SNP	2833.39	26	19	23	0	0	0	0	0	0	0	0	0	0	0	26	19
Chr24	12218287	C	A	Chr24_12218C_A	SNP	16155.63	19	17	25	17	14	15	24	30	18	15	13	18	5	9	19	17
Chr24	12218287	C	A	Chr24_12218C_A	SNP	16155.63	19	17	25	17	14	15	24	30	18	15	13	18	5	9	19	17
Chr24	12218287	C	A	Chr24_12218C_A	SNP	16155.63	19	17	25	17	14	15	24	30	18	15	13	18	5	9	19	17
Chr24	12218287	C	A	Chr24_12218C_A	SNP	16155.63	19	17	25	17	14	15	24	30	18	15	13	18	5	9	19	17
Chr24	12222235	C	A	Chr24_12222C_A	SNP	11386.63	21	5	10	17	8	22	18	16	8	14	7	7	4	7	21	5
Chr24	12222235	C	A	Chr24_12222C_A	SNP	11386.63	21	5	10	17	8	22	18	16	8	14	7	7	4	7	21	5
Chr24	12222235	C	A	Chr24_12222C_A	SNP	11386.63	21	5	10	17	8	22	18	16	8	14	7	7	4	7	21	5
Chr24	12222235	C	A	Chr24_12222C_A	SNP	11386.63	21	5	10	17	8	22	18	16	8	14	7	7	4	7	21	5
Chr24	12234361	G	T	Chr24_12234G_T	SNP	18999.63	31	18	16	23	20	26	36	24	25	22	15	25	11	9	31	18
Chr24	12234361	G	T	Chr24_12234G_T	SNP	18999.63	31	18	16	23	20	26	36	24	25	22	15	25	11	9	31	18
Chr24	12234361	G	T	Chr24_12234G_T	SNP	18999.63	31	18	16	23	20	26	36	24	25	22	15	25	11	9	31	18
Chr24	12234361	G	T	Chr24_12234G_T	SNP	18999.63	31	18	16	23	20	26	36	24	25	22	15	25	11	9	31	18
Chr24	12234361	G	T	Chr24_12234G_T	SNP	18999.63	31	18	16	23	20	26	36	24	25	22	15	25	11	9	31	18
Chr24	12234361	G	T	Chr24_12234G_T	SNP	18999.63	31	18	16	23	20	26	36	24	25	22	15	25	11	9	31	18
Chr24	12234399	G	A	Chr24_12234G_A	SNP	19973.63	28	21	15	26	19	22	39	24	19	26	23	27	12	11	28	21
Chr24	12234399	G	A	Chr24_12234G_A	SNP	19973.63	28	21	15	26	19	22	39	24	19	26	23	27	12	11	28	21
Chr24	12234399	G	A	Chr24_12234G_A	SNP	19973.63	28	21	15	26	19	22	39	24	19	26	23	27	12	11	28	21
Chr24	12234399	G	A	Chr24_12234G_A	SNP	19973.63	28	21	15	26	19	22	39	24	19	26	23	27	12	11	28	21
Chr24	12234399	G	A	Chr24_12234G_A	SNP	19973.63	28	21	15	26	19	22	39	24	19	26	23	27	12	11	28	21
Chr24	12234399	G	A	Chr24_12234G_A	SNP	19973.63	28	21	15	26	19	22	39	24	19	26	23	27	12	11	28	21
Chr24	12278633	G	T	Chr24_12278G_T	SNP	20169.63	30	12	11	32	21	27	34	11	25	28	17	16	16	26	30	12
Chr24	12278676	G	T	Chr24_12278G_T	SNP	21126.63	30	18	14	35	19	26	31	16	21	30	20	24	19	24	30	18
Chr24	12285687	AAT	A	Chr24_12285AAT_A	INDEL	10731.59	20	10	6	21	15	25	27	20	14	18	5	10	8	7	20	10
Chr24	12285687	AAT	A	Chr24_12285AAT_A	INDEL	10731.59	20	10	6	21	15	25	27	20	14	18	5	10	8	7	20	10
Chr24	12285687	AAT	A	Chr24_12285AAT_A	INDEL	10731.59	20	10	6	21	15	25	27	20	14	18	5	10	8	7	20	10
Chr24	12285687	AAT	A	Chr24_12285AAT_A	INDEL	10731.59	20	10	6	21	15	25	27	20	14	18	5	10	8	7	20	10
Chr24	12285687	AAT	A	Chr24_12285AAT_A	INDEL	10731.59	20	10	6	21	15	25	27	20	14	18	5	10	8	7	20	10
Chr24	12285687	AAT	A	Chr24_12285AAT_A	INDEL	10731.59	20	10	6	21	15	25	27	20	14	18	5	10	8	7	20	10
Chr24	12295369	T	TTA	Chr24_12295T_TTA	INDEL	960.35	23	10	5	0	0	0	2	0	0	1	2	1	0	1	24	11
Chr24	12295369	T	TTA	Chr24_12295T_TTA	INDEL	960.35	23	10	5	0	0	0	2	0	0	1	2	1	0	1	24	11
Chr24	12295369	T	TTA	Chr24_12295T_TTA	INDEL	960.35	23	10	5	0	0	0	2	0	0	1	2	1	0	1	24	11
Chr24	12295369	T	TTA	Chr24_12295T_TTA	INDEL	960.35	23	10	5	0	0	0	2	0	0	1	2	1	0	1	24	11
Chr24	12295369	T	TTA	Chr24_12295T_TTA	INDEL	960.35	23	10	5	0	0	0	2	0	0	1	2	1	0	1	24	11
Chr24	12295369	T	TTA	Chr24_12295T_TTA	INDEL	960.35	23	10	5	0	0	0	2	0	0	1	2	1	0	1	24	11
Chr24	12295369	T	TTA	Chr24_12295T_TTA	INDEL	960.35	23	10	5	0	0	0	2	0	0	1	2	1	0	1	24	11
Chr24	12295369	T	TTA	Chr24_12295T_TTA	INDEL	960.35	23	10	5	0	0	0	2	0	0	1	2	1	0	1	24	11
Chr24	12433251	G	GA	Chr24_12433G_GA	INDEL	14549.59	25	13	16	24	18	31	26	16	25	30	13	16	12	7	25	13
Chr24	12433251	G	GA	Chr24_12433G_GA	INDEL	14549.59	25	13	16	24	18	31	26	16	25	30	13	16	12	7	25	13
Chr24	12433251	G	GA	Chr24_12433G_GA	INDEL	14549.59	25	13	16	24	18	31	26	16	25	30	13	16	12	7	25	13
Chr24	12433251	G	GA	Chr24_12433G_GA	INDEL	14549.59	25	13	16	24	18	31	26	16	25	30	13	16	12	7	25	13
Chr24	12433251	G	GA	Chr24_12433G_GA	INDEL	14549.59	25	13	16	24	18	31	26	16	25	30	13	16	12	7	25	13
Chr24	12433251	G	GA	Chr24_12433G_GA	INDEL	14549.59	25	13	16	24	18	31	26	16	25	30	13	16	12	7	25	13
Chr24	12433251	G	GA	Chr24_12433G_GA	INDEL	14549.59	25	13	16	24	18	31	26	16	25	30	13	16	12	7	25	13
Chr24	12473698	T	TTA	Chr24_12473T_TTA	INDEL	2669.24	0	0	1	10	10	15	22	14	1	0	0	1	0	1	21	6
Chr24	12581254	A	AAT	Chr24_12581A_AAT	INDEL	8062.59	12	7	8	15	13	12	20	9	12	14	9	8	10	7	12	7
Chr24	12581254	A	AAT	Chr24_12581A_AAT	INDEL	8062.59	12	7	8	15	13	12	20	9	12	14	9	8	10	7	12	7
Chr24	12581254	A	AAT	Chr24_12581A_AAT	INDEL	8062.59	12	7	8	15	13	12	20	9	12	14	9	8	10	7	12	7
Chr24	12581254	A	AAT	Chr24_12581A_AAT	INDEL	8062.59	12	7	8	15	13	12	20	9	12	14	9	8	10	7	12	7
Chr24	12581254	A	AAT	Chr24_12581A_AAT	INDEL	8062.59	12	7	8	15	13	12	20	9	12	14	9	8	10	7	12	7
Chr24	12581254	A	AAT	Chr24_12581A_AAT	INDEL	8062.59	12	7	8	15	13	12	20	9	12	14	9	8	10	7	12	7
Chr24	12692498	T	TTA	Chr24_12692T_TTA	INDEL	1702.93	0	1	0	0	1	1	1	0	8	14	8	6	7	6	23	9
Chr24	12692498	T	TTA	Chr24_12692T_TTA	INDEL	1702.93	0	1	0	0	1	1	1	0	8	14	8	6	7	6	23	9
Chr24	12692498	T	TTA	Chr24_12692T_TTA	INDEL	1702.93	0	1	0	0	1	1	1	0	8	14	8	6	7	6	23	9
Chr24	12834742	C	A	Chr24_12834C_A	SNP	12226.29	29	11	22	0	0	0	1	0	24	21	13	15	13	9	29	11
Chr24	12903062	A	AAT	Chr24_12903A_AAT	INDEL	7152.24	28	9	11	0	0	0	0	0	10	13	15	8	13	10	28	9
Chr24	12903062	A	AAT	Chr24_12903A_AAT	INDEL	7152.24	28	9	11	0	0	0	0	0	10	13	15	8	13	10	28	9
Chr24	12903062	A	AAT	Chr24_12903A_AAT	INDEL	7152.24	28	9	11	0	0	0	0	0	10	13	15	8	13	10	28	9
Chr24	12910443	C	T	Chr24_12910C_T	SNP	11735.63	16	6	7	10	9	13	13	13	14	15	12	11	4	8	16	6
Chr24	12910443	C	T	Chr24_12910C_T	SNP	11735.63	16	6	7	10	9	13	13	13	14	15	12	11	4	8	16	6
Chr24	12910443	C	T	Chr24_12910C_T	SNP	11735.63	16	6	7	10	9	13	13	13	14	15	12	11	4	8	16	6
Chr24	12910443	C	T	Chr24_12910C_T	SNP	11735.63	16	6	7	10	9	13	13	13	14	15	12	11	4	8	16	6
Chr24	12910450	A	G	Chr24_12910A_G	SNP	12147.63	18	6	7	11	12	12	15	16	14	15	13	11	4	8	18	6
Chr24	12910450	A	G	Chr24_12910A_G	SNP	12147.63	18	6	7	11	12	12	15	16	14	15	13	11	4	8	18	6
Chr24	12910450	A	G	Chr24_12910A_G	SNP	12147.63	18	6	7	11	12	12	15	16	14	15	13	11	4	8	18	6
Chr24	12910450	A	G	Chr24_12910A_G	SNP	12147.63	18	6	7	11	12	12	15	16	14	15	13	11	4	8	18	6
Chr24	12910608	T	C	Chr24_12910T_C	SNP	11271.63	18	4	7	14	8	25	23	9	15	16	15	15	7	7	18	4
Chr24	12910608	T	C	Chr24_12910T_C	SNP	11271.63	18	4	7	14	8	25	23	9	15	16	15	15	7	7	18	4
Chr24	12910608	T	C	Chr24_12910T_C	SNP	11271.63	18	4	7	14	8	25	23	9	15	16	15	15	7	7	18	4
Chr24	12910608	T	C	Chr24_12910T_C	SNP	11271.63	18	4	7	14	8	25	23	9	15	16	15	15	7	7	18	4
Chr24	12998431	C	CTA	Chr24_12998C_CTA	INDEL	3668.24	0	0	0	10	15	23	25	18	0	0	0	0	0	0	22	12
Chr24	12998431	C	CTA	Chr24_12998C_CTA	INDEL	3668.24	0	0	0	10	15	23	25	18	0	0	0	0	0	0	22	12
Chr24	12998431	C	CTA	Chr24_12998C_CTA	INDEL	3668.24	0	0	0	10	15	23	25	18	0	0	0	0	0	0	22	12
Chr24	12998431	C	CTA	Chr24_12998C_CTA	INDEL	3668.24	0	0	0	10	15	23	25	18	0	0	0	0	0	0	22	12
Chr24	12998431	C	CTA	Chr24_12998C_CTA	INDEL	3668.24	0	0	0	10	15	23	25	18	0	0	0	0	0	0	22	12
Chr24	12998431	C	CTA	Chr24_12998C_CTA	INDEL	3668.24	0	0	0	10	15	23	25	18	0	0	0	0	0	0	22	12
Chr24	12998431	C	CTA	Chr24_12998C_CTA	INDEL	3668.24	0	0	0	10	15	23	25	18	0	0	0	0	0	0	22	12
Chr24	12998431	C	CTA	Chr24_12998C_CTA	INDEL	3668.24	0	0	0	10	15	23	25	18	0	0	0	0	0	0	22	12
Chr24	12998431	C	CTA	Chr24_12998C_CTA	INDEL	3668.24	0	0	0	10	15	23	25	18	0	0	0	0	0	0	22	12
Chr24	12998431	C	CTA	Chr24_12998C_CTA	INDEL	3668.24	0	0	0	10	15	23	25	18	0	0	0	0	0	0	22	12
Chr24	13016959	A	AAT	Chr24_13016A_AAT	INDEL	3318.24	0	0	0	21	15	10	29	15	3	0	0	0	0	0	28	9
Chr24	13016959	A	AAT	Chr24_13016A_AAT	INDEL	3318.24	0	0	0	21	15	10	29	15	3	0	0	0	0	0	28	9
Chr24	13016959	A	AAT	Chr24_13016A_AAT	INDEL	3318.24	0	0	0	21	15	10	29	15	3	0	0	0	0	0	28	9
Chr24	13016959	A	AAT	Chr24_13016A_AAT	INDEL	3318.24	0	0	0	21	15	10	29	15	3	0	0	0	0	0	28	9
Chr24	13016959	A	AAT	Chr24_13016A_AAT	INDEL	3318.24	0	0	0	21	15	10	29	15	3	0	0	0	0	0	28	9
Chr24	13016959	A	AAT	Chr24_13016A_AAT	INDEL	3318.24	0	0	0	21	15	10	29	15	3	0	0	0	0	0	28	9
Chr24	13171123	G	GTA	Chr24_13171G_GTA	INDEL	3075.24	0	0	1	21	16	15	18	18	0	1	3	1	0	0	16	12
Chr24	13171123	G	GTA	Chr24_13171G_GTA	INDEL	3075.24	0	0	1	21	16	15	18	18	0	1	3	1	0	0	16	12
Chr24	13171123	G	GTA	Chr24_13171G_GTA	INDEL	3075.24	0	0	1	21	16	15	18	18	0	1	3	1	0	0	16	12
Chr24	13171123	G	GTA	Chr24_13171G_GTA	INDEL	3075.24	0	0	1	21	16	15	18	18	0	1	3	1	0	0	16	12
Chr24	13212843	A	AC	Chr24_13212A_AC	INDEL	17611.59	37	18	9	16	27	36	28	17	31	27	17	12	5	15	37	18
Chr24	13212843	A	AC	Chr24_13212A_AC	INDEL	17611.59	37	18	9	16	27	36	28	17	31	27	17	12	5	15	37	18
Chr24	13212843	A	AC	Chr24_13212A_AC	INDEL	17611.59	37	18	9	16	27	36	28	17	31	27	17	12	5	15	37	18
Chr24	13306133	AG	A	Chr24_13306AG_A	INDEL	15646.59	24	12	12	22	24	25	28	24	22	25	13	15	8	17	24	12
Chr24	13306133	AG	A	Chr24_13306AG_A	INDEL	15646.59	24	12	12	22	24	25	28	24	22	25	13	15	8	17	24	12
Chr24	13306133	AG	A	Chr24_13306AG_A	INDEL	15646.59	24	12	12	22	24	25	28	24	22	25	13	15	8	17	24	12
Chr24	13306133	AG	A	Chr24_13306AG_A	INDEL	15646.59	24	12	12	22	24	25	28	24	22	25	13	15	8	17	24	12
Chr24	13308785	TG	T	Chr24_13308TG_T	INDEL	14021.59	14	9	10	15	23	21	28	20	11	6	23	12	8	8	14	9
Chr24	13308785	TG	T	Chr24_13308TG_T	INDEL	14021.59	14	9	10	15	23	21	28	20	11	6	23	12	8	8	14	9
Chr24	13308785	TG	T	Chr24_13308TG_T	INDEL	14021.59	14	9	10	15	23	21	28	20	11	6	23	12	8	8	14	9
Chr24	13308807	T	G	Chr24_13308T_G	SNP	13896.63	13	10	10	16	20	18	24	22	12	5	19	14	8	9	13	10
Chr24	13308807	T	G	Chr24_13308T_G	SNP	13896.63	13	10	10	16	20	18	24	22	12	5	19	14	8	9	13	10
Chr24	13308807	T	G	Chr24_13308T_G	SNP	13896.63	13	10	10	16	20	18	24	22	12	5	19	14	8	9	13	10
Chr24	13308920	G	T	Chr24_13308G_T	SNP	13054.63	15	14	10	10	22	22	24	19	11	8	18	22	13	9	15	14
Chr24	13308920	G	T	Chr24_13308G_T	SNP	13054.63	15	14	10	10	22	22	24	19	11	8	18	22	13	9	15	14
Chr24	13308920	G	T	Chr24_13308G_T	SNP	13054.63	15	14	10	10	22	22	24	19	11	8	18	22	13	9	15	14
Chr24	13315883	T	C	Chr24_13315T_C	SNP	16368.63	18	8	14	18	18	24	31	28	13	18	12	23	7	7	18	8
Chr24	13315883	T	C	Chr24_13315T_C	SNP	16368.63	18	8	14	18	18	24	31	28	13	18	12	23	7	7	18	8
Chr24	13315883	T	C	Chr24_13315T_C	SNP	16368.63	18	8	14	18	18	24	31	28	13	18	12	23	7	7	18	8
Chr24	13315883	T	C	Chr24_13315T_C	SNP	16368.63	18	8	14	18	18	24	31	28	13	18	12	23	7	7	18	8
Chr24	13402689	C	T	Chr24_13402C_T	SNP	5204.29	0	0	0	14	23	25	25	21	0	0	0	0	0	0	24	9
Chr24	13490675	C	CT	Chr24_13490C_CT	INDEL	8613.59	11	7	6	14	7	13	12	12	14	14	6	6	9	6	11	7
Chr24	13490736	A	AT	Chr24_13490A_AT	INDEL	10730.59	11	8	11	18	10	6	4	12	8	13	9	5	8	10	11	8
Chr24	13490741	CT	C	Chr24_13490CT_C	INDEL	10892.59	11	8	11	19	9	6	4	12	12	9	10	7	7	10	11	8
Chr24	13506588	C	A	Chr24_13506C_A	SNP	19075.63	28	11	18	23	21	21	21	18	19	35	13	21	12	9	28	11
Chr24	13506814	A	T	Chr24_13506A_T	SNP	12877.63	14	6	10	15	7	14	18	15	17	20	6	11	11	10	14	6
Chr24	13506815	G	A	Chr24_13506G_A	SNP	12877.63	14	6	10	15	7	14	18	15	17	20	6	11	11	9	14	6
Chr24	13506932	G	T	Chr24_13506G_T	SNP	21668.63	20	17	12	30	18	23	25	30	29	28	15	22	13	14	20	17
Chr24	13506953	C	CA	Chr24_13506C_CA	INDEL	20770.59	20	15	12	28	18	25	23	30	32	24	15	19	12	13	20	15
Chr24	13738412	A	T	Chr24_13738A_T	SNP	2424.39	27	18	13	0	0	0	0	0	0	0	0	0	0	0	27	18
Chr24	13738412	A	T	Chr24_13738A_T	SNP	2424.39	27	18	13	0	0	0	0	0	0	0	0	0	0	0	27	18
Chr25	248562	GTATATATA	G	Chr25_24856GTATATATA_	INDEL	12889.59	28	10	13	11	16	22	22	14	26	20	9	8	6	4	28	10
Chr25	248562	GTATATATA	G	Chr25_24856GTATATATA_	INDEL	12889.59	28	10	13	11	16	22	22	14	26	20	9	8	6	4	28	10
Chr25	248562	GTATATATA	G	Chr25_24856GTATATATA_	INDEL	12889.59	28	10	13	11	16	22	22	14	26	20	9	8	6	4	28	10
Chr25	338617	C	A	Chr25_33861C_A	SNP	23045.63	26	8	16	23	20	42	27	33	31	32	9	27	19	9	26	8
Chr25	338623	C	G	Chr25_33862C_G	SNP	22911.63	26	8	15	22	20	39	28	35	33	32	11	27	20	9	26	8
Chr25	338634	C	T	Chr25_33863C_T	SNP	21401.63	32	7	13	21	18	43	26	33	35	35	11	25	20	8	32	7
Chr25	341733	T	TGCATCAAA	Chr25_34173T_TGCATCA	INDEL	9445.59	10	6	9	20	6	10	12	9	12	7	9	8	7	4	11	7
Chr25	341733	T	TGCATCAAA	Chr25_34173T_TGCATCA	INDEL	9445.59	10	6	9	20	6	10	12	9	12	7	9	8	7	4	11	7
Chr25	341733	T	TGCATCAAA	Chr25_34173T_TGCATCA	INDEL	9445.59	10	6	9	20	6	10	12	9	12	7	9	8	7	4	11	7
Chr25	452268	A	AT	Chr25_45226A_AT	INDEL	13162.59	15	11	14	14	14	24	19	17	13	20	17	16	9	7	15	11
Chr25	452268	A	AT	Chr25_45226A_AT	INDEL	13162.59	15	11	14	14	14	24	19	17	13	20	17	16	9	7	15	11
Chr25	452268	A	AT	Chr25_45226A_AT	INDEL	13162.59	15	11	14	14	14	24	19	17	13	20	17	16	9	7	15	11
Chr25	452268	A	AT	Chr25_45226A_AT	INDEL	13162.59	15	11	14	14	14	24	19	17	13	20	17	16	9	7	15	11
Chr25	456649	G	GTA	Chr25_45664G_GTA	INDEL	1131.35	17	8	9	1	0	0	0	0	0	0	0	0	0	0	17	8
Chr25	620582	A	AG	Chr25_62058A_AG	INDEL	18819.59	32	14	13	26	32	30	27	25	21	20	20	18	11	7	32	14
Chr25	934993	C	A	Chr25_93499C_A	SNP	9065.63	6	4	7	10	9	15	10	15	10	7	4	6	6	10	6	4
Chr25	934993	C	A	Chr25_93499C_A	SNP	9065.63	6	4	7	10	9	15	10	15	10	7	4	6	6	10	6	4
Chr25	934993	C	A	Chr25_93499C_A	SNP	9065.63	6	4	7	10	9	15	10	15	10	7	4	6	6	10	6	4
Chr25	934993	C	A	Chr25_93499C_A	SNP	9065.63	6	4	7	10	9	15	10	15	10	7	4	6	6	10	6	4
Chr25	934993	C	A	Chr25_93499C_A	SNP	9065.63	6	4	7	10	9	15	10	15	10	7	4	6	6	10	6	4
Chr25	934993	C	A	Chr25_93499C_A	SNP	9065.63	6	4	7	10	9	15	10	15	10	7	4	6	6	10	6	4
Chr25	981494	G	T	Chr25_98149G_T	SNP	13948.63	14	15	13	18	15	23	31	27	14	18	9	9	7	8	14	15
Chr25	981494	G	T	Chr25_98149G_T	SNP	13948.63	14	15	13	18	15	23	31	27	14	18	9	9	7	8	14	15
Chr25	981494	G	T	Chr25_98149G_T	SNP	13948.63	14	15	13	18	15	23	31	27	14	18	9	9	7	8	14	15
Chr25	981494	G	T	Chr25_98149G_T	SNP	13948.63	14	15	13	18	15	23	31	27	14	18	9	9	7	8	14	15
Chr25	981494	G	T	Chr25_98149G_T	SNP	13948.63	14	15	13	18	15	23	31	27	14	18	9	9	7	8	14	15
Chr25	981494	G	T	Chr25_98149G_T	SNP	13948.63	14	15	13	18	15	23	31	27	14	18	9	9	7	8	14	15

Chr25	981542	G	T	Chr25_98154G_T	SNP	15266.63	13	13	11	21	14	21	31	20	14	16	9	12	7	9
Chr25	981542	G	T	Chr25_98154G_T	SNP	15266.63	13	13	11	21	14	21	31	20	14	16	9	12	7	9
Chr25	981542	G	T	Chr25_98154G_T	SNP	15266.63	13	13	11	21	14	21	31	20	14	16	9	12	7	9
Chr25	981542	G	T	Chr25_98154G_T	SNP	15266.63	13	13	11	21	14	21	31	20	14	16	9	12	7	9
Chr25	981542	G	T	Chr25_98154G_T	SNP	15266.63	13	13	11	21	14	21	31	20	14	16	9	12	7	9
Chr25	981542	G	T	Chr25_98154G_T	SNP	15266.63	13	13	11	21	14	21	31	20	14	16	9	12	7	9
Chr25	981545	G	T	Chr25_98154G_T	SNP	15258.63	13	13	11	23	13	21	32	19	14	16	10	13	7	9
Chr25	981545	G	T	Chr25_98154G_T	SNP	15258.63	13	13	11	23	13	21	32	19	14	16	10	13	7	9
Chr25	981545	G	T	Chr25_98154G_T	SNP	15258.63	13	13	11	23	13	21	32	19	14	16	10	13	7	9
Chr25	981545	G	T	Chr25_98154G_T	SNP	15258.63	13	13	11	23	13	21	32	19	14	16	10	13	7	9
Chr25	981545	G	T	Chr25_98154G_T	SNP	15258.63	13	13	11	23	13	21	32	19	14	16	10	13	7	9
Chr25	981545	G	T	Chr25_98154G_T	SNP	15258.63	13	13	11	23	13	21	32	19	14	16	10	13	7	9
Chr25	981639	AC	A	Chr25_98163AC_A	INDEL	12740.59	18	9	10	24	8	18	22	17	19	13	12	10	8	10
Chr25	981639	AC	A	Chr25_98163AC_A	INDEL	12740.59	18	9	10	24	8	18	22	17	19	13	12	10	8	10
Chr25	981639	AC	A	Chr25_98163AC_A	INDEL	12740.59	18	9	10	24	8	18	22	17	19	13	12	10	8	10
Chr25	981639	AC	A	Chr25_98163AC_A	INDEL	12740.59	18	9	10	24	8	18	22	17	19	13	12	10	8	10
Chr25	981639	AC	A	Chr25_98163AC_A	INDEL	12740.59	18	9	10	24	8	18	22	17	19	13	12	10	8	10
Chr25	981639	AC	A	Chr25_98163AC_A	INDEL	12740.59	18	9	10	24	8	18	22	17	19	13	12	10	8	10
Chr25	987130	TTA	T	Chr25_98713TTA_T	INDEL	777.35	14	11	5	0	0	0	0	0	1	0	1	0	0	0
Chr25	987130	TTA	T	Chr25_98713TTA_T	INDEL	777.35	14	11	5	0	0	0	0	0	1	0	1	0	0	0
Chr25	987130	TTA	T	Chr25_98713TTA_T	INDEL	777.35	14	11	5	0	0	0	0	0	1	0	1	0	0	0
Chr25	1054939	C	T	Chr25_10549C_T	SNP	5833.29	0	0	0	19	12	19	30	28	0	0	0	0	0	0
Chr25	1060836	C	T	Chr25_10608C_T	SNP	16138.63	24	12	13	27	16	37	31	14	18	18	16	22	16	9
Chr25	1150396	T	TTA	Chr25_11503T_TTA	INDEL	2731.24	0	0	1	17	13	15	25	16	1	0	0	0	0	0

Chr25	3338260	G	GCCTAAC	Chr25_33382	G_GCCTAAC	INDEL	18461.59	23	9	7	[illegible]
Chr25	3338260	G	GCCTAAC	Chr25_33382	G_GCCTAAC	INDEL	18461.59	23	9	7	[illegible]
Chr25	3338264	C	A	Chr25_33382	C_A	SNP	18650.63	27	8	7	[illegible]
Chr25	3338264	C	A	Chr25_33382	C_A	SNP	18650.63	27	8	7	[illegible]
Chr25	3338264	C	A	Chr25_33382	C_A	SNP	18650.63	27	8	7	[illegible]
Chr25	3338266	C	CACATTAAA	Chr25_33382	C_CACATTAA	INDEL	19541.59	27	9	7	[illegible]
Chr25	3338266	C	CACATTAAA	Chr25_33382	C_CACATTAA	INDEL	19541.59	27	9	7	[illegible]
Chr25	3338266	C	CACATTAAA	Chr25_33382	C_CACATTAA	INDEL	19541.59	27	9	7	[illegible]
Chr25	3338269	C	A	Chr25_33382	C_A	SNP	19204.63	30	10	8	[illegible]
Chr25	3338269	C	A	Chr25_33382	C_A	SNP	19204.63	30	10	8	[illegible]
Chr25	3338269	C	A	Chr25_33382	C_A	SNP	19204.63	30	10	8	[illegible]
Chr25	3338270	G	GGAAA	Chr25_33382	G_GGAAA	INDEL	19060.59	30	10	8	[illegible]
Chr25	3338270	G	GGAAA	Chr25_33382	G_GGAAA	INDEL	19060.59	30	10	8	[illegible]
Chr25	3338270	G	GGAAA	Chr25_33382	G_GGAAA	INDEL	19060.59	30	10	8	[illegible]
Chr25	3338576	T	C	Chr25_33385	T_C	SNP	17226.63	28	18	23	[illegible]
Chr25	3338576	T	C	Chr25_33385	T_C	SNP	17226.63	28	18	23	[illegible]
Chr25	3338576	T	C	Chr25_33385	T_C	SNP	17226.63	28	18	23	[illegible]
Chr25	3340604	A	G	Chr25_33406	A_G	SNP	18645.63	20	17	17	[illegible]
Chr25	3340604	A	G	Chr25_33406	A_G	SNP	18645.63	20	17	17	[illegible]
Chr25	3340604	A	G	Chr25_33406	A_G	SNP	18645.63	20	17	17	[illegible]
Chr25	3340611	A	G	Chr25_33406	A_G	SNP	18873.63	22	18	18	[illegible]
Chr25	3340611	A	G	Chr25_33406	A_G	SNP	18873.63	22	18	18	[illegible]
Chr25	3340611	A	G	Chr25_33406	A_G	SNP	18873.63	22	18	18	[illegible]
Chr25	3368783	C	A	Chr25_33687	C_A	SNP	42201.63	56	36	29	[illegible]
Chr25	3368783	C	A	Chr25_33687	C_A	SNP	42201.63	56	36	29	[illegible]
Chr25	3368783	C	A	Chr25_33687	C_A	SNP	42201.63	56	36	29	[illegible]
Chr25	3368784	T	TG	Chr25_33687	T_TG	INDEL	42193.59	55	37	29	[illegible]
Chr25	3368784	T	TG	Chr25_33687	T_TG	INDEL	42193.59	55	37	29	[illegible]
Chr25	3368784	T	TG	Chr25_33687	T_TG	INDEL	42193.59	55	37	29	[illegible]
Chr25	3553939	TTA	T	Chr25_35539	TTA_T	INDEL	5781.59	12	13	8	[illegible]
Chr25	3631385	A	AT	Chr25_36313	A_AT	INDEL	17559.59	27	9	15	[illegible]
Chr25	3631385	A	AT	Chr25_36313	A_AT	INDEL	17559.59	27	9	15	[illegible]
Chr25	3631385	A	AT	Chr25_36313	A_AT	INDEL	17559.59	27	9	15	[illegible]
Chr25	3631385	A	AT	Chr25_36313	A_AT	INDEL	17559.59	27	9	15	[illegible]
Chr25	3631385	A	AT	Chr25_36313	A_AT	INDEL	17559.59	27	9	15	[illegible]
Chr25	3632730	T	TA	Chr25_36327	T_TA	INDEL	20480.59	36	14	12	[illegible]
Chr25	3632730	T	TA	Chr25_36327	T_TA	INDEL	20480.59	36	14	12	[illegible]
Chr25	3632730	T	TA	Chr25_36327	T_TA	INDEL	20480.59	36	14	12	[illegible]
Chr25	3632730	T	TA	Chr25_36327	T_TA	INDEL	20480.59	36	14	12	[illegible]
Chr25	3632730	T	TA	Chr25_36327	T_TA	INDEL	20480.59	36	14	12	[illegible]
Chr25	3632744	C	CA	Chr25_36327	C_CA	INDEL	20735.59	30	13	12	[illegible]
Chr25	3632744	C	CA	Chr25_36327	C_CA	INDEL	20735.59	30	13	12	[illegible]
Chr25	3632744	C	CA	Chr25_36327	C_CA	INDEL	20735.59	30	13	12	[illegible]
Chr25	3632744	C	CA	Chr25_36327	C_CA	INDEL	20735.59	30	13	12	[illegible]
Chr25	3632744	C	CA	Chr25_36327	C_CA	INDEL	20735.59	30	13	12	[illegible]
Chr25	4150647	CT	C	Chr25_41506	CT_C	INDEL	18045.59	36	21	20	[illegible]
Chr25	4150647	CT	C	Chr25_41506	CT_C	INDEL	18045.59	36	21	20	[illegible]
Chr25	4150647	CT	C	Chr25_41506	CT_C	INDEL	18045.59	36	21	20	[illegible]
Chr25	4150647	CT	C	Chr25_41506	CT_C	INDEL	18045.59	36	21	20	[illegible]
Chr25	4160328	A	AT	Chr25_41603	A_AT	INDEL	13411.59	17	15	9	[illegible]
Chr25	4160328	A	AT	Chr25_41603	A_AT	INDEL	13411.59	17	15	9	[illegible]
Chr25	4160328	A	AT	Chr25_41603	A_AT	INDEL	13411.59	17	15	9	[illegible]
Chr25	4160328	A	AT	Chr25_41603	A_AT	INDEL	13411.59	17	15	9	[illegible]
Chr25	4232011	T	TACC	Chr25_42320	T_TACC	INDEL	18164.59	33	14	20	[illegible]
Chr25	4232011	T	TACC	Chr25_42320	T_TACC	INDEL	18164.59	33	14	20	[illegible]
Chr25	4232016	AG	A	Chr25_42320	AG_A	INDEL	17911.59	33	13	20	[illegible]
Chr25	4232016	AG	A	Chr25_42320	AG_A	INDEL	17911.59	33	13	20	[illegible]
Chr25	4407894	A	AAT	Chr25_44078	A_AAT	INDEL	2043.93	0	0	0	[illegible]
Chr25	4452073	A	T	Chr25_44520	A_T	SNP	3744.98	0	0	0	[illegible]
Chr25	4452073	A	T	Chr25_44520	A_T	SNP	3744.98	0	0	0	[illegible]
Chr25	4452073	A	T	Chr25_44520	A_T	SNP	3744.98	0	0	0	[illegible]
Chr25	4452073	A	T	Chr25_44520	A_T	SNP	3744.98	0	0	0	[illegible]
Chr25	4452073	A	T	Chr25_44520	A_T	SNP	3744.98	0	0	0	[illegible]
Chr25	4452073	A	T	Chr25_44520	A_T	SNP	3744.98	0	0	0	[illegible]
Chr25	4452073	A	T	Chr25_44520	A_T	SNP	3744.98	0	0	0	[illegible]
Chr25	4452073	A	T	Chr25_44520	A_T	SNP	3744.98	0	0	0	[illegible]
Chr25	4508066	G	GT	Chr25_45080	G_GT	INDEL	15990.59	29	16	13	[illegible]
Chr25	4617394	G	T	Chr25_46173	G_T	SNP	12842.63	20	13	8	[illegible]
Chr25	4617394	G	T	Chr25_46173	G_T	SNP	12842.63	20	13	8	[illegible]
Chr25	4617394	G	T	Chr25_46173	G_T	SNP	12842.63	20	13	8	[illegible]
Chr25	4617394	G	T	Chr25_46173	G_T	SNP	12842.63	20	13	8	[illegible]
Chr25	4617394	G	T	Chr25_46173	G_T	SNP	12842.63	20	13	8	[illegible]
Chr25	4617516	C	A	Chr25_46175	C_A	SNP	14168.63	21	10	12	[illegible]
Chr25	4617516	C	A	Chr25_46175	C_A	SNP	14168.63	21	10	12	[illegible]
Chr25	4617516	C	A	Chr25_46175	C_A	SNP	14168.63	21	10	12	[illegible]
Chr25	4617516	C	A	Chr25_46175	C_A	SNP	14168.63	21	10	12	[illegible]
Chr25	4617516	C	A	Chr25_46175	C_A	SNP	14168.63	21	10	12	[illegible]
Chr25	4617523	C	A	Chr25_46175	C_A	SNP	14098.63	21	9	12	[illegible]
Chr25	4617523	C	A	Chr25_46175	C_A	SNP	14098.63	21	9	12	[illegible]
Chr25	4617523	C	A	Chr25_46175	C_A	SNP	14098.63	21	9	12	[illegible]
Chr25	4617523	C	A	Chr25_46175	C_A	SNP	14098.63	21	9	12	[illegible]
Chr25	4617523	C	A	Chr25_46175	C_A	SNP	14098.63	21	9	12	[illegible]
Chr25	4708627	G	T	Chr25_47086	G_T	SNP	19262.63	23	10	13	[illegible]
Chr25	4708627	G	T	Chr25_47086	G_T	SNP	19262.63	23	10	13	[illegible]
Chr25	4708627	G	T	Chr25_47086	G_T	SNP	19262.63	23	10	13	[illegible]
Chr25	4708651	G	A	Chr25_47086	G_A	SNP	19927.63	27	15	11	[illegible]
Chr25	4708651	G	A	Chr25_47086	G_A	SNP	19927.63	27	15	11	[illegible]
Chr25	4708651	G	A	Chr25_47086	G_A	SNP	19927.63	27	15	11	[illegible]
Chr25	4815466	A	AT	Chr25_48154	A_AT	INDEL	7446.59	14	8	6	[illegible]
Chr25	4864349	A	AATAT	Chr25_48643	A_AATAT	INDEL	8522.59	22	11	7	[illegible]
Chr25	4864349	A	AATAT	Chr25_48643	A_AATAT	INDEL	8522.59	22	11	7	[illegible]
Chr25	4864349	A	AATAT	Chr25_48643	A_AATAT	INDEL	8522.59	22	11	7	[illegible]
Chr25	4864349	A	AATAT	Chr25_48643	A_AATAT	INDEL	8522.59	22	11	7	[illegible]
Chr25	4864349	A	AATAT	Chr25_48643	A_AATAT	INDEL	8522.59	22	11	7	[illegible]
Chr25	4864349	A	AATAT	Chr25_48643	A_AATAT	INDEL	8522.59	22	11	7	[illegible]
Chr25	4864349	A	AATAT	Chr25_48643	A_AATAT	INDEL	8522.59	22	11	7	[illegible]
Chr25	4864349	A	AATAT	Chr25_48643	A_AATAT	INDEL	8522.59	22	11	7	[illegible]
Chr25	5092882	C	T	Chr25_50928	C_T	SNP	19743.63	27	22	20	[illegible]
Chr25	5092882	C	T	Chr25_50928	C_T	SNP	19743.63	27	22	20	[illegible]
Chr25	5092882	C	T	Chr25_50928	C_T	SNP	19743.63	27	22	20	[illegible]
Chr25	5092882	C	T	Chr25_50928	C_T	SNP	19743.63	27	22	20	[illegible]
Chr25	5092909	C	T	Chr25_50929	C_T	SNP	22991.63	27	23	17	[illegible]
Chr25	5092909	C	T	Chr25_50929	C_T	SNP	22991.63	27	23	17	[illegible]
Chr25	5092909	C	T	Chr25_50929	C_T	SNP	22991.63	27	23	17	[illegible]
Chr25	5092909	C	T	Chr25_50929	C_T	SNP	22991.63	27	23	17	[illegible]
Chr25	5288880	A	AT	Chr25_52888	A_AT	INDEL	17169.59	21	16	17	[illegible]
Chr25	5288880	A	AT	Chr25_52888	A_AT	INDEL	17169.59	21	16	17	[illegible]
Chr25	5288880	A	AT	Chr25_52888	A_AT	INDEL	17169.59	21	16	17	[illegible]
Chr25	5288880	A	AT	Chr25_52888	A_AT	INDEL	17169.59	21	16	17	[illegible]
Chr25	5288907	C	CT	Chr25_52889	C_CT	INDEL	21109.59	29	18	16	[illegible]
Chr25	5288907	C	CT	Chr25_52889	C_CT	INDEL	21109.59	29	18	16	[illegible]
Chr25	5288907	C	CT	Chr25_52889	C_CT	INDEL	21109.59	29	18	16	[illegible]
Chr25	5288907	C	CT	Chr25_52889	C_CT	INDEL	21109.59	29	18	16	[illegible]
Chr25	5288929	C	G	Chr25_52889	C_G	SNP	21732.63	30	16	16	[illegible]
Chr25	5288929	C	G	Chr25_52889	C_G	SNP	21732.63	30	16	16	[illegible]
Chr25	5288929	C	G	Chr25_52889	C_G	SNP	21732.63	30	16	16	[illegible]
Chr25	5288929	C	G	Chr25_52889	C_G	SNP	21732.63	30	16	16	[illegible]
Chr25	5642164	A	AT	Chr25_56421	A_AT	INDEL	15232.59	24	14	22	[illegible]
Chr25	5676050	T	TCAAGAAAG	Chr25_56760	T_TCAAGAA	INDEL	25011.59	31	23	12	[illegible]
Chr25	5714729	C	G	Chr25_57147	C_G	SNP	26649.63	33	23	19	[illegible]
Chr25	5714729	C	G	Chr25_57147	C_G	SNP	26649.63	33	23	19	[illegible]
Chr25	5714729	C	G	Chr25_57147	C_G	SNP	26649.63	33	23	19	[illegible]
Chr25	5714729	C	G	Chr25_57147	C_G	SNP	26649.63	33	23	19	[illegible]
Chr25	5714729	C	G	Chr25_57147	C_G	SNP	26649.63	33	23	19	[illegible]
Chr25	5714729	C	G	Chr25_57147	C_G	SNP	26649.63	33	23	19	[illegible]
Chr25	5714729	C	G	Chr25_57147	C_G	SNP	26649.63	33	23	19	[illegible]
Chr25	5714729	C	G	Chr25_57147	C_G	SNP	26649.63	33	23	19	[illegible]
Chr25	5714729	C	G	Chr25_57147	C_G	SNP	26649.63	33	23	19	[illegible]
Chr25	5714733	G	GC	Chr25_57147	G_GC	INDEL	27263.59	33	23	19	[illegible]
Chr25	5714733	G	GC	Chr25_57147	G_GC	INDEL	27263.59	33	23	19	[illegible]
Chr25	5714733	G	GC	Chr25_57147	G_GC	INDEL	27263.59	33	23	19	[illegible]
Chr25	5714733	G	GC	Chr25_57147	G_GC	INDEL	27263.59	33	23	19	[illegible]
Chr25	5714733	G	GC	Chr25_57147	G_GC	INDEL	27263.59	33	23	19	[illegible]
Chr25	5714733	G	GC	Chr25_57147	G_GC	INDEL	27263.59	33	23	19	[illegible]
Chr25	5714733	G	GC	Chr25_57147	G_GC	INDEL	27263.59	33	23	19	[illegible]
Chr25	5714738	T	A	Chr25_57147	T_A	SNP	27477.63	33	22	19	[illegible]
Chr25	5714738	T	A	Chr25_57147	T_A	SNP	27477.63	33	22	19	[illegible]
Chr25	5714738	T	A	Chr25_57147	T_A	SNP	27477.63	33	22	19	[illegible]
Chr25	5714738	T	A	Chr25_57147	T_A	SNP	27477.63	33	22	19	[illegible]
Chr25	5714738	T	A	Chr25_57147	T_A	SNP	27477.63	33	22	19	[illegible]
Chr25	5714738	T	A	Chr25_57147	T_A	SNP	27477.63	33	22	19	[illegible]
Chr25	5714738	T	A	Chr25_57147	T_A	SNP	27477.63	33	22	19	[illegible]
Chr25	5714739	A	C	Chr25_57147	A_C	SNP	27207.63	33	23	20	[illegible]
Chr25	5714739	A	C	Chr25_57147	A_C	SNP	27207.63	33	23	20	[illegible]
Chr25	5714739	A	C	Chr25_57147	A_C	SNP	27207.63	33	23	20	[illegible]
Chr25	5714739	A	C	Chr25_57147	A_C	SNP	27207.63	33	23	20	[illegible]
Chr25	5714739	A	C	Chr25_57147	A_C	SNP	27207.63	33	23	20	[illegible]
Chr25	5714739	A	C	Chr25_57147	A_C	SNP	27207.63	33	23	20	[illegible]
Chr25	5714739	A	C	Chr25_57147	A_C	SNP	27207.63	33	23	20	[illegible]
Chr25	5799556	G	T	Chr25_57995	G_T	SNP	17100.63	29	16	13	[illegible]
Chr25	5800595	C	CA	Chr25_58005	C_CA	INDEL	19746.59	33	20	14	[illegible]
Chr25	5828907	G	T	Chr25_58289	G_T	SNP	20837.63	28	22	19	[illegible]
Chr25	6081817	GA	G	Chr25_60818	GA_G	INDEL	13960.59	20	17	14	[illegible]
Chr25	6160683	G	C	Chr25_61606	G_C	SNP	15322.63	19	7	17	[illegible]
Chr25	6160684	C	A	Chr25_61606	C_A	SNP	15322.63	19	7	17	[illegible]
Chr25	6323925	G	T	Chr25_63239	G_T	SNP	21139.63	33	11	18	[illegible]
Chr25	6324145	A	G	Chr25_63241	A_G	SNP	6702.29	0	0	0	[illegible]
Chr25	6662988	C	CA	Chr25_66629	C_CA	INDEL	17145.59	27	16	19	[illegible]
Chr25	6735972	A	AAT	Chr25_67359	A_AAT	INDEL	1165.35	21	6	10	[illegible]
Chr25	6735972	A	AAT	Chr25_67359	A_AAT	INDEL	1165.35	21	6	10	[illegible]
Chr25	6735972	A	AAT	Chr25_67359	A_AAT	INDEL	1165.35	21	6	10	[illegible]
Chr25	6735972	A	AAT	Chr25_67359	A_AAT	INDEL	1165.35	21	6	10	[illegible]
Chr25	6735972	A	AAT	Chr25_67359	A_AAT	INDEL	1165.35	21	6	10	[illegible]
Chr25	6808547	G	C	Chr25_68085	G_C	SNP	12546.29	27	10	24	[illegible]
Chr25	6814402	A	T	Chr25_68144	A_T	SNP	16822.63	19	8	19	[illegible]
Chr25	6814513	G	A	Chr25_68145	G_A	SNP	17258.63	23	13	18	[illegible]
Chr25	6814601	C	A	Chr25_68146	C_A	SNP	11187.63	11	5	11	[illegible]
Chr25	6814602	C	A	Chr25_68146	C_A	SNP	11187.63	11	5	11	[illegible]
Chr25	6814658	A	T	Chr25_68146	A_T	SNP	7425.63	5	7	7	[illegible]
Chr25	6839070	C	T	Chr25_68390	C_T	SNP	35988.63	38	30	34	[illegible]
Chr25	6839206	C	T	Chr25_68392	C_T	SNP	21220.63	29	11	22	[illegible]
Chr25	6839207	C	T	Chr25_68392	C_T	SNP	20040.63	29	11	20	[illegible]
Chr25	6839235	G	A	Chr25_68392	G_A	SNP	18541.63	28	14	14	[illegible]
Chr25	6842284	C	T	Chr25_68422	C_T	SNP	33263.63	40	22	20	[illegible]
Chr25	6842294	G	A	Chr25_68422	G_A	SNP	33739.63	42	24	21	[illegible]
Chr25	6842535	C	T	Chr25_68425	C_T	SNP	10934.63	9	9	8	[illegible]
Chr25	6842550	G	A	Chr25_68425	G_A	SNP	10788.63	16	7	7	[illegible]
Chr25	6842561	G	T	Chr25_68425	G_T	SNP	10483.63	21	6	5	[illegible]
Chr25	6846217	G	T	Chr25_68462	G_T	SNP	15198.63	35	17	9	[illegible]
Chr25	6892143	C	CT	Chr25_68921	C_CT	INDEL	16356.59	27	19	17	[illegible]
Chr25	6892143	C	CT	Chr25_68921	C_CT	INDEL	16356.59	27	19	17	[illegible]
Chr25	6892143	C	CT	Chr25_68921	C_CT	INDEL	16356.59	27	19	17	[illegible]
Chr25	6892230	G	C	Chr25_68922	G_C	SNP	18566.63	33	19	17	[illegible]
Chr25	6892230	G	C	Chr25_68922	G_C	SNP	18566.63	33	19	17	[illegible]
Chr25	6892230	G	C	Chr25_68922	G_C	SNP	18566.63	33	19	17	[illegible]
Chr25	6994605	G	A	Chr25_69946	G_A	SNP	6224.29	0	0	0	[illegible]
Chr25	7038284	T	TC	Chr25_70382	T_TC	INDEL	15119.59	31	12	16	[illegible]
Chr25	7040583	A	T	Chr25_70405	A_T	SNP	12586.63	23	11	10	[illegible]
Chr25	7040603	G	A	Chr25_70406	G_A	SNP	12729.63	19	8	12	[illegible]
Chr25	7040610	G	A	Chr25_70406	G_A	SNP	12832.63	19	8	12	[illegible]
Chr25	7040611	G	A	Chr25_70406	G_A	SNP	12723.63	19	8	12	[illegible]
Chr25	7051092	C	T	Chr25_70510	C_T	SNP	12611.63	30	19	14	[illegible]
Chr25	7174345	A	T	Chr25_71743	A_T	SNP	15144.63	32	22	8	[illegible]
Chr25	7174432	T	G	Chr25_71744	T_G	SNP	15497.63	32	14	9	[illegible]
Chr25	7315874	G	GA	Chr25_73158	G_GA	INDEL	17201.59	27	17	15	[illegible]
Chr25	7407238	G	A	Chr25_74072	G_A	SNP	4352.29	0	0	0	[illegible]
Chr25	7766337	A	AT	Chr25_77663	A_AT	INDEL	13422.59	20	15	16	[illegible]
Chr25	7768373	G	GA	Chr25_77683	G_GA	INDEL	13472.59	13	13	13	[illegible]
Chr25	7768393	G	GA	Chr25_77683	G_GA	INDEL	11554.59	12	11	6	[illegible]
Chr25	7768564	A	T	Chr25_77685	A_T	SNP	12424.63	22	10	9	[illegible]
Chr25	7772216	G	T	Chr25_77722	G_T	SNP	18311.63	23	16	20	[illegible]
Chr25	7772422	C	CA	Chr25_77724	C_CA	INDEL	15049.59	24	17	13	[illegible]

Chr25	7977681	G	A	Chr25_79776G_A	SNP	16371.63	26	20	[illegible]
Chr25	7996235	A	T	Chr25_79962A_T	SNP	10206.29	39	8	[illegible]
Chr25	8037331	G	A	Chr25_80373G_A	SNP	11024.63	14	12	[illegible]
Chr25	8037331	G	A	Chr25_80373G_A	SNP	11024.63	14	12	[illegible]
Chr25	8037331	G	A	Chr25_80373G_A	SNP	11024.63	14	12	[illegible]
Chr25	8037331	G	A	Chr25_80373G_A	SNP	11024.63	14	12	[illegible]
Chr25	8037331	G	A	Chr25_80373G_A	SNP	11024.63	14	12	[illegible]
Chr25	8037331	G	A	Chr25_80373G_A	SNP	11024.63	14	12	[illegible]
Chr25	8037374	A	G	Chr25_80373A_G	SNP	14785.63	20	14	[illegible]
Chr25	8037374	A	G	Chr25_80373A_G	SNP	14785.63	20	14	[illegible]
Chr25	8037374	A	G	Chr25_80373A_G	SNP	14785.63	20	14	[illegible]
Chr25	8037374	A	G	Chr25_80373A_G	SNP	14785.63	20	14	[illegible]
Chr25	8037374	A	G	Chr25_80373A_G	SNP	14785.63	20	14	[illegible]
Chr25	8037374	A	G	Chr25_80373A_G	SNP	14785.63	20	14	[illegible]
Chr25	8038962	TCTA	T	Chr25_80389TCTA_T	INDEL	55020.59	63	56	[illegible]
Chr25	8038962	TCTA	T	Chr25_80389TCTA_T	INDEL	55020.59	63	56	[illegible]
Chr25	8038962	TCTA	T	Chr25_80389TCTA_T	INDEL	55020.59	63	56	[illegible]
Chr25	8038962	TCTA	T	Chr25_80389TCTA_T	INDEL	55020.59	63	56	[illegible]
Chr25	8076242	G	T	Chr25_80762G_T	SNP	5614.29	0	0	[illegible]
Chr25	8170536	C	CTA	Chr25_81705C_CTA	INDEL	10822.59	24	12	[illegible]
Chr25	8170536	C	CTA	Chr25_81705C_CTA	INDEL	10822.59	24	12	[illegible]
Chr25	8170536	C	CTA	Chr25_81705C_CTA	INDEL	10822.59	24	12	[illegible]
Chr25	8170536	C	CTA	Chr25_81705C_CTA	INDEL	10822.59	24	12	[illegible]
Chr25	8170536	C	CTA	Chr25_81705C_CTA	INDEL	10822.59	24	12	[illegible]
Chr25	8170536	C	CTA	Chr25_81705C_CTA	INDEL	10822.59	24	12	[illegible]
Chr25	8171765	AAT	A	Chr25_81717AAT_A	INDEL	8051.5	29	17	[illegible]
Chr25	8171765	AAT	A	Chr25_81717AAT_A	INDEL	8051.5	29	17	[illegible]
Chr25	8171765	AAT	A	Chr25_81717AAT_A	INDEL	8051.5	29	17	[illegible]
Chr25	8171765	AAT	A	Chr25_81717AAT_A	INDEL	8051.5	29	17	[illegible]
Chr25	8171765	AAT	A	Chr25_81717AAT_A	INDEL	8051.5	29	17	[illegible]
Chr25	8171765	AAT	A	Chr25_81717AAT_A	INDEL	8051.5	29	17	[illegible]
Chr25	8337442	C	G	Chr25_83374C_G	SNP	10969.63	15	4	[illegible]
Chr25	8337445	C	T	Chr25_83374C_T	SNP	11031.63	15	4	[illegible]
Chr25	8375579	A	AT	Chr25_83755A_AT	INDEL	11784.59	15	7	[illegible]
Chr25	8375579	A	AT	Chr25_83755A_AT	INDEL	11784.59	15	7	[illegible]
Chr25	8375579	A	AT	Chr25_83755A_AT	INDEL	11784.59	15	7	[illegible]
Chr25	8480215	G	A	Chr25_84802G_A	SNP	21054.63	29	18	[illegible]
Chr25	8480215	G	A	Chr25_84802G_A	SNP	21054.63	29	18	[illegible]
Chr25	8480215	G	A	Chr25_84802G_A	SNP	21054.63	29	18	[illegible]
Chr25	8480224	G	A	Chr25_84802G_A	SNP	21429.63	32	16	[illegible]
Chr25	8480224	G	A	Chr25_84802G_A	SNP	21429.63	32	16	[illegible]
Chr25	8480224	G	A	Chr25_84802G_A	SNP	21429.63	32	16	[illegible]
Chr25	8537553	T	TA	Chr25_85375T_TA	INDEL	16042.59	28	17	[illegible]
Chr25	8635523	T	TTGAGAGGA	Chr25_86355T_TTGAGAG	INDEL	24863.59	30	12	[illegible]
Chr25	8640366	A	T	Chr25_86403A_T	SNP	19100.63	31	12	[illegible]
Chr25	8640366	A	T	Chr25_86403A_T	SNP	19100.63	31	12	[illegible]
Chr25	8643970	AAT	A	Chr25_86439AAT_A	INDEL	13536.59	34	7	[illegible]
Chr25	8643970	AAT	A	Chr25_86439AAT_A	INDEL	13536.59	34	7	[illegible]
Chr25	8718303	C	CCTTAACCAA	Chr25_87183C_CCTTAACC	INDEL	23303.59	23	18	[illegible]
Chr25	8785469	CATATAT	C	Chr25_87854CATATAT_C	INDEL	63.37	0	0	[illegible]
Chr25	8785469	CATATAT	C	Chr25_87854CATATAT_C	INDEL	63.37	0	0	[illegible]
Chr25	8785469	CATATAT	C	Chr25_87854CATATAT_C	INDEL	63.37	0	0	[illegible]
Chr25	8785469	CATATAT	C	Chr25_87854CATATAT_C	INDEL	63.37	0	0	[illegible]
Chr25	8923068	G	GA	Chr25_89230G_GA	INDEL	16433.59	37	10	[illegible]
Chr25	8995004	C	CA	Chr25_89950C_CA	INDEL	17554.59	22	25	[illegible]
Chr25	9164472	T	TTA	Chr25_91644T_TTA	INDEL	1445.35	27	11	[illegible]
Chr25	9164472	T	TTA	Chr25_91644T_TTA	INDEL	1445.35	27	11	[illegible]
Chr25	9164472	T	TTA	Chr25_91644T_TTA	INDEL	1445.35	27	11	[illegible]
Chr25	9164472	T	TTA	Chr25_91644T_TTA	INDEL	1445.35	27	11	[illegible]
Chr25	9164472	T	TTA	Chr25_91644T_TTA	INDEL	1445.35	27	11	[illegible]
Chr25	9164472	T	TTA	Chr25_91644T_TTA	INDEL	1445.35	27	11	[illegible]
Chr25	9164472	T	TTA	Chr25_91644T_TTA	INDEL	1445.35	27	11	[illegible]
Chr25	9164472	T	TTA	Chr25_91644T_TTA	INDEL	1445.35	27	11	[illegible]
Chr25	9177796	T	TTA	Chr25_91777T_TTA	INDEL	1952.93	0	1	[illegible]
Chr25	9220415	C	CAT	Chr25_92204C_CAT	INDEL	2877.24	0	0	[illegible]
Chr25	9231062	A	AAT	Chr25_92310A_AAT	INDEL	1691.93	0	0	[illegible]
Chr25	9231062	A	AAT	Chr25_92310A_AAT	INDEL	1691.93	0	0	[illegible]
Chr25	9231062	A	AAT	Chr25_92310A_AAT	INDEL	1691.93	0	0	[illegible]
Chr25	9231062	A	AAT	Chr25_92310A_AAT	INDEL	1691.93	0	0	[illegible]
Chr25	9412827	CAT	C	Chr25_94128CAT_C	INDEL	411.37	0	0	[illegible]
Chr25	9412827	CAT	C	Chr25_94128CAT_C	INDEL	411.37	0	0	[illegible]
Chr25	9412827	CAT	C	Chr25_94128CAT_C	INDEL	411.37	0	0	[illegible]
Chr25	9412827	CAT	C	Chr25_94128CAT_C	INDEL	411.37	0	0	[illegible]
Chr25	9412827	CAT	C	Chr25_94128CAT_C	INDEL	411.37	0	0	[illegible]
Chr25	9412827	CAT	C	Chr25_94128CAT_C	INDEL	411.37	0	0	[illegible]
Chr25	9412827	CAT	C	Chr25_94128CAT_C	INDEL	411.37	0	0	[illegible]
Chr25	9412827	CAT	C	Chr25_94128CAT_C	INDEL	411.37	0	0	[illegible]
Chr25	9412827	CAT	C	Chr25_94128CAT_C	INDEL	411.37	0	0	[illegible]
Chr25	9412827	CAT	C	Chr25_94128CAT_C	INDEL	411.37	0	0	[illegible]
Chr25	9474197	A	AT	Chr25_94741A_AT	INDEL	18631.59	16	15	[illegible]
Chr25	9474238	T	A	Chr25_94742T_A	SNP	20885.63	22	17	[illegible]
Chr25	9474260	C	G	Chr25_94742C_G	SNP	20717.63	24	15	[illegible]
Chr25	9474261	A	C	Chr25_94742A_C	SNP	20489.63	24	15	[illegible]
Chr25	9474269	T	A	Chr25_94742T_A	SNP	19946.63	24	14	[illegible]
Chr25	9500301	A	T	Chr25_95003A_T	SNP	12117.63	23	7	[illegible]
Chr25	9500301	A	T	Chr25_95003A_T	SNP	12117.63	23	7	[illegible]
Chr25	9500320	C	CTTATTTTTG	Chr25_95003C_CTTATTTT	INDEL	11067.59	18	7	[illegible]
Chr25	9500320	C	CTTATTTTTG	Chr25_95003C_CTTATTTT	INDEL	11067.59	18	7	[illegible]
Chr25	9684222	A	AATTTCTTCA	Chr25_96842A_AATTTCTT	INDEL	10516.59	15	7	[illegible]
Chr25	9797236	G	GA	Chr25_97972G_GA	INDEL	15480.59	42	16	[illegible]
Chr25	9800260	G	GTA	Chr25_98002G_GTA	INDEL	809.35	18	6	[illegible]
Chr25	9800260	G	GTA	Chr25_98002G_GTA	INDEL	809.35	18	6	[illegible]
Chr25	9800260	G	GTA	Chr25_98002G_GTA	INDEL	809.35	18	6	[illegible]
Chr25	9988345	T	A	Chr25_99883T_A	SNP	16741.63	23	16	[illegible]
Chr25	10103831	AC	A	Chr25_10103AC_A	INDEL	17464.59	32	17	[illegible]
Chr25	10158420	CG	C	Chr25_10158CG_C	INDEL	18352.59	20	10	[illegible]
Chr25	10158424	A	ACCC	Chr25_10158A_ACCC	INDEL	18777.59	21	12	[illegible]
Chr25	10158425	G	T	Chr25_10158G_T	SNP	18653.63	21	12	[illegible]
Chr25	10195832	G	A	Chr25_10195G_A	SNP	5347.29	0	0	[illegible]
Chr25	10255252	G	T	Chr25_10255G_T	SNP	19687.63	37	23	[illegible]
Chr25	10255252	G	T	Chr25_10255G_T	SNP	19687.63	37	23	[illegible]
Chr25	10255252	G	T	Chr25_10255G_T	SNP	19687.63	37	23	[illegible]
Chr25	10255252	G	T	Chr25_10255G_T	SNP	19687.63	37	23	[illegible]
Chr25	10255252	G	T	Chr25_10255G_T	SNP	19687.63	37	23	[illegible]
Chr25	10255252	G	T	Chr25_10255G_T	SNP	19687.63	37	23	[illegible]
Chr25	10255252	G	T	Chr25_10255G_T	SNP	19687.63	37	23	[illegible]
Chr25	10255277	C	T	Chr25_10255C_T	SNP	19233.63	35	21	[illegible]
Chr25	10255277	C	T	Chr25_10255C_T	SNP	19233.63	35	21	[illegible]
Chr25	10255277	C	T	Chr25_10255C_T	SNP	19233.63	35	21	[illegible]
Chr25	10255277	C	T	Chr25_10255C_T	SNP	19233.63	35	21	[illegible]
Chr25	10255277	C	T	Chr25_10255C_T	SNP	19233.63	35	21	[illegible]
Chr25	10255277	C	T	Chr25_10255C_T	SNP	19233.63	35	21	[illegible]
Chr25	10255277	C	T	Chr25_10255C_T	SNP	19233.63	35	21	[illegible]
Chr25	10411613	A	AAT	Chr25_10411A_AAT	INDEL	7930.59	18	7	[illegible]
Chr25	10411613	A	AAT	Chr25_10411A_AAT	INDEL	7930.59	18	7	[illegible]
Chr25	10411613	A	AAT	Chr25_10411A_AAT	INDEL	7930.59	18	7	[illegible]
Chr25	10411613	A	AAT	Chr25_10411A_AAT	INDEL	7930.59	18	7	[illegible]
Chr25	10411613	A	AAT	Chr25_10411A_AAT	INDEL	7930.59	18	7	[illegible]
Chr25	10411613	A	AAT	Chr25_10411A_AAT	INDEL	7930.59	18	7	[illegible]
Chr25	10472513	TTA	T	Chr25_10472TTA_T	INDEL	9236.59	27	10	[illegible]
Chr25	10472513	TTA	T	Chr25_10472TTA_T	INDEL	9236.59	27	10	[illegible]
Chr25	10472513	TTA	T	Chr25_10472TTA_T	INDEL	9236.59	27	10	[illegible]
Chr25	10479627	TTA	T	Chr25_10479TTA_T	INDEL	5536.49	12	11	[illegible]
Chr25	10479627	TTA	T	Chr25_10479TTA_T	INDEL	5536.49	12	11	[illegible]
Chr25	10479627	TTA	T	Chr25_10479TTA_T	INDEL	5536.49	12	11	[illegible]
Chr25	10479627	TTA	T	Chr25_10479TTA_T	INDEL	5536.49	12	11	[illegible]
Chr25	10479627	TTA	T	Chr25_10479TTA_T	INDEL	5536.49	12	11	[illegible]
Chr25	10479627	TTA	T	Chr25_10479TTA_T	INDEL	5536.49	12	11	[illegible]
Chr25	10479627	TTA	T	Chr25_10479TTA_T	INDEL	5536.49	12	11	[illegible]
Chr25	10509743	G	GA	Chr25_10509G_GA	INDEL	14350.59	20	18	[illegible]
Chr25	10573225	A	AT	Chr25_10573A_AT	INDEL	24131.59	35	17	[illegible]
Chr25	10573225	A	AT	Chr25_10573A_AT	INDEL	24131.59	35	17	[illegible]
Chr25	10573225	A	AT	Chr25_10573A_AT	INDEL	24131.59	35	17	[illegible]
Chr25	10573225	A	AT	Chr25_10573A_AT	INDEL	24131.59	35	17	[illegible]
Chr25	10573225	A	AT	Chr25_10573A_AT	INDEL	24131.59	35	17	[illegible]
Chr25	10573225	A	AT	Chr25_10573A_AT	INDEL	24131.59	35	17	[illegible]
Chr25	10573225	A	AT	Chr25_10573A_AT	INDEL	24131.59	35	17	[illegible]
Chr25	10573225	A	AT	Chr25_10573A_AT	INDEL	24131.59	35	17	[illegible]
Chr25	10573231	TA	T	Chr25_10573TA_T	INDEL	24106.59	36	18	[illegible]
Chr25	10573231	TA	T	Chr25_10573TA_T	INDEL	24106.59	36	18	[illegible]
Chr25	10573231	TA	T	Chr25_10573TA_T	INDEL	24106.59	36	18	[illegible]
Chr25	10573231	TA	T	Chr25_10573TA_T	INDEL	24106.59	36	18	[illegible]
Chr25	10573231	TA	T	Chr25_10573TA_T	INDEL	24106.59	36	18	[illegible]
Chr25	10573231	TA	T	Chr25_10573TA_T	INDEL	24106.59	36	18	[illegible]
Chr25	10573231	TA	T	Chr25_10573TA_T	INDEL	24106.59	36	18	[illegible]
Chr25	10573231	TA	T	Chr25_10573TA_T	INDEL	24106.59	36	18	[illegible]
Chr25	10798129	A	C	Chr25_10798A_C	SNP	13013.29	39	12	[illegible]
Chr25	10893600	G	A	Chr25_10893G_A	SNP	1878.39	28	11	[illegible]
Chr25	10893600	G	A	Chr25_10893G_A	SNP	1878.39	28	11	[illegible]
Chr25	10893600	G	A	Chr25_10893G_A	SNP	1878.39	28	11	[illegible]
Chr25	10893600	G	A	Chr25_10893G_A	SNP	1878.39	28	11	[illegible]
Chr25	10955485	G	A	Chr25_10955G_A	SNP	14936.63	21	16	[illegible]
Chr25	10955487	C	T	Chr25_10955C_T	SNP	15026.63	20	16	[illegible]
Chr25	10955489	C	T	Chr25_10955C_T	SNP	14884.63	20	15	[illegible]
Chr25	10955498	C	T	Chr25_10955C_T	SNP	14243.63	19	15	[illegible]
Chr25	11025256	T	TTA	Chr25_11025T_TTA	INDEL	395.37	2	0	[illegible]
Chr25	11025256	T	TTA	Chr25_11025T_TTA	INDEL	395.37	2	0	[illegible]
Chr25	11025256	T	TTA	Chr25_11025T_TTA	INDEL	395.37	2	0	[illegible]
Chr25	11025256	T	TTA	Chr25_11025T_TTA	INDEL	395.37	2	0	[illegible]
Chr25	11025256	T	TTA	Chr25_11025T_TTA	INDEL	395.37	2	0	[illegible]
Chr25	11025636	C	G	Chr25_11025C_G	SNP	10916.29	29	12	[illegible]
Chr25	11025636	C	G	Chr25_11025C_G	SNP	10916.29	29	12	[illegible]
Chr25	11025636	C	G	Chr25_11025C_G	SNP	10916.29	29	12	[illegible]
Chr25	11025636	C	G	Chr25_11025C_G	SNP	10916.29	29	12	[illegible]
Chr25	11025636	C	G	Chr25_11025C_G	SNP	10916.29	29	12	[illegible]
Chr25	11029535	G	C	Chr25_11029G_C	SNP	13066.63	21	8	[illegible]
Chr25	11029535	G	C	Chr25_11029G_C	SNP	13066.63	21	8	[illegible]
Chr25	11060666	TTA	T	Chr25_11060TTA_T	INDEL	7214.59	12	9	[illegible]
Chr25	11060666	TTA	T	Chr25_11060TTA_T	INDEL	7214.59	12	9	[illegible]
Chr25	11060666	TTA	T	Chr25_11060TTA_T	INDEL	7214.59	12	9	[illegible]
Chr25	11060666	TTA	T	Chr25_11060TTA_T	INDEL	7214.59	12	9	[illegible]
Chr25	11060666	TTA	T	Chr25_11060TTA_T	INDEL	7214.59	12	9	[illegible]
Chr25	11060666	TTA	T	Chr25_11060TTA_T	INDEL	7214.59	12	9	[illegible]
Chr25	11060666	TTA	T	Chr25_11060TTA_T	INDEL	7214.59	12	9	[illegible]
Chr25	11108425	TA	T	Chr25_11108TA_T	INDEL	12319.59	27	8	[illegible]
Chr25	11137229	A	G	Chr25_11137A_G	SNP	16400.63	34	11	[illegible]
Chr25	11137229	A	G	Chr25_11137A_G	SNP	16400.63	34	11	[illegible]
Chr25	11137229	A	G	Chr25_11137A_G	SNP	16400.63	34	11	[illegible]
Chr25	11137229	A	G	Chr25_11137A_G	SNP	16400.63	34	11	[illegible]
Chr25	11137229	A	G	Chr25_11137A_G	SNP	16400.63	34	11	[illegible]
Chr25	11137229	A	G	Chr25_11137A_G	SNP	16400.63	34	11	[illegible]
Chr25	11137229	A	G	Chr25_11137A_G	SNP	16400.63	34	11	[illegible]
Chr25	11137229	A	G	Chr25_11137A_G	SNP	16400.63	34	11	[illegible]
Chr25	11137229	A	G	Chr25_11137A_G	SNP	16400.63	34	11	[illegible]
Chr25	11138590	A	C	Chr25_11138A_C	SNP	13635.63	20	10	[illegible]
Chr25	11138590	A	C	Chr25_11138A_C	SNP	13635.63	20	10	[illegible]
Chr25	11138590	A	C	Chr25_11138A_C	SNP	13635.63	20	10	[illegible]
Chr25	11138590	A	C	Chr25_11138A_C	SNP	13635.63	20	10	[illegible]
Chr25	11138590	A	C	Chr25_11138A_C	SNP	13635.63	20	10	[illegible]
Chr25	11138590	A	C	Chr25_11138A_C	SNP	13635.63	20	10	[illegible]
Chr25	11138590	A	C	Chr25_11138A_C	SNP	13635.63	20	10	[illegible]
Chr25	11138590	A	C	Chr25_11138A_C	SNP	13635.63	20	10	[illegible]
Chr25	11138590	A	C	Chr25_11138A_C	SNP	13635.63	20	10	[illegible]
Chr25	11138648	G	A	Chr25_11138G_A	SNP	10411.63	21	8	[illegible]
Chr25	11138648	G	A	Chr25_11138G_A	SNP	10411.63	21	8	[illegible]
Chr25	11138648	G	A	Chr25_11138G_A	SNP	10411.63	21	8	[illegible]
Chr25	11138648	G	A	Chr25_11138G_A	SNP	10411.63	21	8	[illegible]
Chr25	11138648	G	A	Chr25_11138G_A	SNP	10411.63	21	8	[illegible]
Chr25	11138648	G	A	Chr25_11138G_A	SNP	10411.63	21	8	[illegible]
Chr25	11138648	G	A	Chr25_11138G_A	SNP	10411.63	21	8	[illegible]
Chr25	11138648	G	A	Chr25_11138G_A	SNP	10411.63	21	8	[illegible]
Chr25	11138648	G	A	Chr25_11138G_A	SNP	10411.63	21	8	[illegible]

Chr26	178158	G	GTA	Chr26_17815G_GTA	INDEL	1362.35	24	10	9	0	0	0	0	0	0	0	0	0	0	0	24	10	9	18	21	17	22
Chr26	178158	G	GTA	Chr26_17815G_GTA	INDEL	1362.35	24	10	9	0	0	0	0	0	0	0	0	0	0	0	24	10	9	18	21	17	22
Chr26	178158	G	GTA	Chr26_17815G_GTA	INDEL	1362.35	24	10	9	0	0	0	0	0	0	0	0	0	0	0	24	10	9	18	21	17	22
Chr26	178158	G	GTA	Chr26_17815G_GTA	INDEL	1362.35	24	10	9	0	0	0	0	0	0	0	0	0	0	0	24	10	9	18	21	17	22
Chr26	178158	G	GTA	Chr26_17815G_GTA	INDEL	1362.35	24	10	9	0	0	0	0	0	0	0	0	0	0	0	24	10	9	18	21	17	22
Chr26	178158	G	GTA	Chr26_17815G_GTA	INDEL	1362.35	24	10	9	0	0	0	0	0	0	0	0	0	0	0	24	10	9	18	21	17	22
Chr26	178158	G	GTA	Chr26_17815G_GTA	INDEL	1362.35	24	10	9	0	0	0	0	0	0	0	0	0	0	0	24	10	9	18	21	17	22
Chr26	178158	G	GTA	Chr26_17815G_GTA	INDEL	1362.35	24	10	9	0	0	0	0	0	0	0	0	0	0	0	24	10	9	18	21	17	22
Chr26	178158	G	GTA	Chr26_17815G_GTA	INDEL	1362.35	24	10	9	0	0	0	0	0	0	0	0	0	0	0	24	10	9	18	21	17	22
Chr26	221114	CGAAG	C	Chr26_22111CGAAG_C	INDEL	12971.59	14	12	13	15	11	14	16	13	16	18	6	8	4	6	14	12	13	15	11	14	16
Chr26	221114	CGAAG	C	Chr26_22111CGAAG_C	INDEL	12971.59	14	12	13	15	11	14	16	13	16	18	6	8	4	6	14	12	13	15	11	14	16
Chr26	221114	CGAAG	C	Chr26_22111CGAAG_C	INDEL	12971.59	14	12	13	15	11	14	16	13	16	18	6	8	4	6	14	12	13	15	11	14	16
Chr26	221114	CGAAG	C	Chr26_22111CGAAG_C	INDEL	12971.59	14	12	13	15	11	14	16	13	16	18	6	8	4	6	14	12	13	15	11	14	16
Chr26	221114	CGAAG	C	Chr26_22111CGAAG_C	INDEL	12971.59	14	12	13	15	11	14	16	13	16	18	6	8	4	6	14	12	13	15	11	14	16
Chr26	221114	CGAAG	C	Chr26_22111CGAAG_C	INDEL	12971.59	14	12	13	15	11	14	16	13	16	18	6	8	4	6	14	12	13	15	11	14	16
Chr26	221120	TTGGA	T	Chr26_22112TTGGA_T	INDEL	13466.59	15	12	13	15	11	15	17	13	16	18	6	9	4	5	15	12	13	15	11	15	17
Chr26	221120	TTGGA	T	Chr26_22112TTGGA_T	INDEL	13466.59	15	12	13	15	11	15	17	13	16	18	6	9	4	5	15	12	13	15	11	15	17
Chr26	221120	TTGGA	T	Chr26_22112TTGGA_T	INDEL	13466.59	15	12	13	15	11	15	17	13	16	18	6	9	4	5	15	12	13	15	11	15	17
Chr26	221120	TTGGA	T	Chr26_22112TTGGA_T	INDEL	13466.59	15	12	13	15	11	15	17	13	16	18	6	9	4	5	15	12	13	15	11	15	17
Chr26	221120	TTGGA	T	Chr26_22112TTGGA_T	INDEL	13466.59	15	12	13	15	11	15	17	13	16	18	6	9	4	5	15	12	13	15	11	15	17
Chr26	221120	TTGGA	T	Chr26_22112TTGGA_T	INDEL	13466.59	15	12	13	15	11	15	17	13	16	18	6	9	4	5	15	12	13	15	11	15	17
Chr26	221124	A	ACCCC	Chr26_22112A_ACCCC	INDEL	13376.59	15	12	13	15	11	15	17	13	16	18	6	9	4	5	15	12	13	15	11	15	17
Chr26	221124	A	ACCCC	Chr26_22112A_ACCCC	INDEL	13376.59	15	12	13	15	11	15	17	13	16	18	6	9	4	5	15	12	13	15	11	15	17
Chr26	221124	A	ACCCC	Chr26_22112A_ACCCC	INDEL	13376.59	15	12	13	15	11	15	17	13	16	18	6	9	4	5	15	12	13	15	11	15	17
Chr26	221124	A	ACCCC	Chr26_22112A_ACCCC	INDEL	13376.59	15	12	13	15	11	15	17	13	16	18	6	9	4	5	15	12	13	15	11	15	17
Chr26	221124	A	ACCCC	Chr26_22112A_ACCCC	INDEL	13376.59	15	12	13	15	11	15	17	13	16	18	6	9	4	5	15	12	13	15	11	15	17
Chr26	221124	A	ACCCC	Chr26_22112A_ACCCC	INDEL	13376.59	15	12	13	15	11	15	17	13	16	18	6	9	4	5	15	12	13	15	11	15	17
Chr26	221128	C	CAAA	Chr26_22112C_CAAA	INDEL	13826.59	15	12	13	16	8	16	17	12	16	16	4	8	4	5	15	12	13	16	8	16	17
Chr26	221128	C	CAAA	Chr26_22112C_CAAA	INDEL	13826.59	15	12	13	16	8	16	17	12	16	16	4	8	4	5	15	12	13	16	8	16	17
Chr26	221128	C	CAAA	Chr26_22112C_CAAA	INDEL	13826.59	15	12	13	16	8	16	17	12	16	16	4	8	4	5	15	12	13	16	8	16	17
Chr26	221128	C	CAAA	Chr26_22112C_CAAA	INDEL	13826.59	15	12	13	16	8	16	17	12	16	16	4	8	4	5	15	12	13	16	8	16	17
Chr26	221128	C	CAAA	Chr26_22112C_CAAA	INDEL	13826.59	15	12	13	16	8	16	17	12	16	16	4	8	4	5	15	12	13	16	8	16	17
Chr26	221128	C	CAAA	Chr26_22112C_CAAA	INDEL	13826.59	15	12	13	16	8	16	17	12	16	16	4	8	4	5	15	12	13	16	8	16	17
Chr26	221135	C	CA	Chr26_22113C_CA	INDEL	13430.59	15	10	13	17	11	17	20	11	16	17	4	7	4	4	15	10	13	17	11	17	20
Chr26	221135	C	CA	Chr26_22113C_CA	INDEL	13430.59	15	10	13	17	11	17	20	11	16	17	4	7	4	4	15	10	13	17	11	17	20
Chr26	221135	C	CA	Chr26_22113C_CA	INDEL	13430.59	15	10	13	17	11	17	20	11	16	17	4	7	4	4	15	10	13	17	11	17	20
Chr26	221135	C	CA	Chr26_22113C_CA	INDEL	13430.59	15	10	13	17	11	17	20	11	16	17	4	7	4	4	15	10	13	17	11	17	20
Chr26	221135	C	CA	Chr26_22113C_CA	INDEL	13430.59	15	10	13	17	11	17	20	11	16	17	4	7	4	4	15	10	13	17	11	17	20
Chr26	221135	C	CA	Chr26_22113C_CA	INDEL	13430.59	15	10	13	17	11	17	20	11	16	17	4	7	4	4	15	10	13	17	11	17	20
Chr26	543941	G	GTA	Chr26_54394G_GTA	INDEL	1084.35	16	10	7	1	0	0	0	0	0	0	0	0	0	0	16	10	7	17	19	20	13
Chr26	543941	G	GTA	Chr26_54394G_GTA	INDEL	1084.35	16	10	7	1	0	0	0	0	0	0	0	0	0	0	16	10	7	17	19	20	13
Chr26	543941	G	GTA	Chr26_54394G_GTA	INDEL	1084.35	16	10	7	1	0	0	0	0	0	0	0	0	0	0	16	10	7	17	19	20	13
Chr26	543941	G	GTA	Chr26_54394G_GTA	INDEL	1084.35	16	10	7	1	0	0	0	0	0	0	0	0	0	0	16	10	7	17	19	20	13
Chr26	543941	G	GTA	Chr26_54394G_GTA	INDEL	1084.35	16	10	7	1	0	0	0	0	0	0	0	0	0	0	16	10	7	17	19	20	13
Chr26	576643	T	TTA	Chr26_57664T_TTA	INDEL	993.35	26	4	9	0	1	1	0	0	0	1	2	0	1	0	26	4	9	13	10	13	23
Chr26	576643	T	TTA	Chr26_57664T_TTA	INDEL	993.35	26	4	9	0	1	1	0	0	0	1	2	0	1	0	26	4	9	13	10	13	23
Chr26	576643	T	TTA	Chr26_57664T_TTA	INDEL	993.35	26	4	9	0	1	1	0	0	0	1	2	0	1	0	26	4	9	13	10	13	23
Chr26	576643	T	TTA	Chr26_57664T_TTA	INDEL	993.35	26	4	9	0	1	1	0	0	0	1	2	0	1	0	26	4	9	13	10	13	23
Chr26	576643	T	TTA	Chr26_57664T_TTA	INDEL	993.35	26	4	9	0	1	1	0	0	0	1	2	0	1	0	26	4	9	13	10	13	23
Chr26	576643	T	TTA	Chr26_57664T_TTA	INDEL	993.35	26	4	9	0	1	1	0	0	0	1	2	0	1	0	26	4	9	13	10	13	23
[illegible]																											

Chr26	3474180	T	TTA	Chr26_34741	T_TTA	INDEL	14024.59	15	12	14	[illegible]
Chr26	3474180	T	TTA	Chr26_34741	T_TTA	INDEL	14024.59	15	12	14	[illegible]
Chr26	3474180	T	TTA	Chr26_34741	T_TTA	INDEL	14024.59	15	12	14	[illegible]
Chr26	3474180	T	TTA	Chr26_34741	T_TTA	INDEL	14024.59	15	12	14	[illegible]
Chr26	3474180	T	TTA	Chr26_34741	T_TTA	INDEL	14024.59	15	12	14	[illegible]
Chr26	3563595	G	A	Chr26_35635	G_A	SNP	6176.29	0	0	0	[illegible]
Chr26	3571654	A	AAT	Chr26_35716	A_AAT	INDEL	195.37	0	0	0	[illegible]
Chr26	3571654	A	AAT	Chr26_35716	A_AAT	INDEL	195.37	0	0	0	[illegible]
Chr26	3571654	A	AAT	Chr26_35716	A_AAT	INDEL	195.37	0	0	0	[illegible]
Chr26	3654039	A	G	Chr26_36540	A_G	SNP	16345.63	23	14	18	[illegible]
Chr26	3741818	G	A	Chr26_37418	G_A	SNP	17962.63	29	15	15	[illegible]
Chr26	3741837	C	T	Chr26_37418	C_T	SNP	20077.63	38	16	18	[illegible]
Chr26	3758736	A	G	Chr26_37587	A_G	SNP	20973.63	50	24	22	[illegible]
Chr26	3758736	A	G	Chr26_37587	A_G	SNP	20973.63	50	24	22	[illegible]
Chr26	3758736	A	G	Chr26_37587	A_G	SNP	20973.63	50	24	22	[illegible]
Chr26	3759914	T	A	Chr26_37599	T_A	SNP	13650.63	26	11	9	[illegible]
Chr26	3759914	T	A	Chr26_37599	T_A	SNP	13650.63	26	11	9	[illegible]
Chr26	3759914	T	A	Chr26_37599	T_A	SNP	13650.63	26	11	9	[illegible]
Chr26	3762108	A	T	Chr26_37621	A_T	SNP	39641.63	72	38	33	[illegible]
Chr26	3762108	A	T	Chr26_37621	A_T	SNP	39641.63	72	38	33	[illegible]
Chr26	3762180	A	T	Chr26_37621	A_T	SNP	37750.63	69	33	29	[illegible]
Chr26	3762180	A	T	Chr26_37621	A_T	SNP	37750.63	69	33	29	[illegible]
Chr26	3762264	T	C	Chr26_37622	T_C	SNP	37673.63	69	30	29	[illegible]
Chr26	3762264	T	C	Chr26_37622	T_C	SNP	37673.63	69	30	29	[illegible]
Chr26	3828007	C	CTA	Chr26_38280	C_CTA	INDEL	1751.25	2	1	2	[illegible]
Chr26	3841147	C	A	Chr26_38411	C_A	SNP	16664.63	27	16	10	[illegible]
Chr26	3841147	C	A	Chr26_38411	C_A	SNP	16664.63	27	16	10	[illegible]
Chr26	3841147	C	A	Chr26_38411	C_A	SNP	16664.63	27	16	10	[illegible]
Chr26	3841147	C	A	Chr26_38411	C_A	SNP	16664.63	27	16	10	[illegible]
Chr26	3858539	A	AG	Chr26_38585	A_AG	INDEL	25157.59	34	19	20	[illegible]
Chr26	3859354	C	T	Chr26_38593	C_T	SNP	27353.63	40	23	21	[illegible]
Chr26	3866284	A	T	Chr26_38662	A_T	SNP	7973.63	13	5	8	[illegible]
Chr26	3916438	G	A	Chr26_39164	G_A	SNP	28903.63	45	26	34	[illegible]
Chr26	3939569	C	T	Chr26_39395	C_T	SNP	12197.63	31	12	8	[illegible]
Chr26	3939569	C	T	Chr26_39395	C_T	SNP	12197.63	31	12	8	[illegible]
Chr26	3982383	G	A	Chr26_39823	G_A	SNP	13687.63	20	14	15	[illegible]
Chr26	3986141	G	A	Chr26_39861	G_A	SNP	25711.63	41	20	26	[illegible]
Chr26	3986151	C	T	Chr26_39861	C_T	SNP	26475.63	42	20	24	[illegible]
Chr26	3990453	G	C	Chr26_39904	G_C	SNP	31800.63	44	23	28	[illegible]
Chr26	3993112	A	T	Chr26_39931	A_T	SNP	26662.63	37	18	15	[illegible]
Chr26	3993336	A	G	Chr26_39933	A_G	SNP	31009.63	35	22	20	[illegible]
Chr26	3993488	G	T	Chr26_39934	G_T	SNP	41560.63	58	24	34	[illegible]
Chr26	3993512	G	GA	Chr26_39935	G_GA	INDEL	43430.59	60	28	33	[illegible]
Chr26	3993519	A	G	Chr26_39935	A_G	SNP	44398.63	61	29	34	[illegible]
Chr26	3997621	A	G	Chr26_39976	A_G	SNP	45726.63	60	41	37	[illegible]
Chr26	3997996	T	TCTAATAA	Chr26_39979	T_TCTAATAA	INDEL	21752.59	24	12	14	[illegible]
Chr26	4022097	A	C	Chr26_40220	A_C	SNP	8836.63	15	7	12	[illegible]
Chr26	4022097	A	C	Chr26_40220	A_C	SNP	8836.63	15	7	12	[illegible]
Chr26	4022097	A	C	Chr26_40220	A_C	SNP	8836.63	15	7	12	[illegible]
Chr26	4066161	C	A	Chr26_40661	C_A	SNP	21298.63	24	13	11	[illegible]
Chr26	4180552	CT	C	Chr26_41805	CT_C	INDEL	8206.59	8	13	9	[illegible]
Chr26	4243738	A	C	Chr26_42437	A_C	SNP	18193.63	34	18	11	[illegible]
Chr26	4243738	A	C	Chr26_42437	A_C	SNP	18193.63	34	18	11	[illegible]
Chr26	4243738	A	C	Chr26_42437	A_C	SNP	18193.63	34	18	11	[illegible]
Chr26	4243738	A	C	Chr26_42437	A_C	SNP	18193.63	34	18	11	[illegible]
Chr26	4255125	T	C	Chr26_42551	T_C	SNP	10532.63	17	10	9	[illegible]
Chr26	4420325	C	A	Chr26_44203	C_A	SNP	18298.63	27	19	21	[illegible]
Chr26	4420382	C	G	Chr26_44203	C_G	SNP	19220.63	37	14	27	[illegible]
Chr26	4597378	AG	A	Chr26_45973	AG_A	INDEL	17733.59	27	15	17	[illegible]
Chr26	4597386	G	C	Chr26_45973	G_C	SNP	17785.63	26	14	15	[illegible]
Chr26	4597387	C	T	Chr26_45973	C_T	SNP	17630.63	27	14	15	[illegible]
Chr26	4597389	G	T	Chr26_45973	G_T	SNP	17660.63	27	14	15	[illegible]
Chr26	4597394	G	A	Chr26_45973	G_A	SNP	17472.63	28	13	15	[illegible]
Chr26	4597398	C	CT	Chr26_45973	C_CT	INDEL	17224.59	27	13	14	[illegible]
Chr26	4874988	TG	T	Chr26_48749	TG_T	INDEL	19397.59	20	14	14	[illegible]
Chr26	4874988	TG	T	Chr26_48749	TG_T	INDEL	19397.59	20	14	14	[illegible]
Chr26	4874988	TG	T	Chr26_48749	TG_T	INDEL	19397.59	20	14	14	[illegible]
Chr26	5029456	G	GA	Chr26_50294	G_GA	INDEL	11129.59	17	4	7	[illegible]
Chr26	5029458	C	A	Chr26_50294	C_A	SNP	11270.63	16	5	6	[illegible]
Chr26	5029465	A	AT	Chr26_50294	A_AT	INDEL	10987.59	16	5	6	[illegible]
Chr26	5029513	C	T	Chr26_50295	C_T	SNP	10388.63	20	6	9	[illegible]
Chr26	5029520	C	A	Chr26_50295	C_A	SNP	11205.63	17	6	11	[illegible]
Chr26	5029531	G	A	Chr26_50295	G_A	SNP	11135.63	15	10	9	[illegible]
Chr26	5029535	C	A	Chr26_50295	C_A	SNP	11417.63	15	10	11	[illegible]
Chr26	5029547	T	TA	Chr26_50295	T_TA	INDEL	12301.59	16	11	11	[illegible]
Chr26	5314135	T	C	Chr26_53141	T_C	SNP	5428.29	0	0	0	[illegible]
Chr26	5314135	T	C	Chr26_53141	T_C	SNP	5428.29	0	0	0	[illegible]
Chr26	5314135	T	C	Chr26_53141	T_C	SNP	5428.29	0	0	0	[illegible]
Chr26	5314135	T	C	Chr26_53141	T_C	SNP	5428.29	0	0	0	[illegible]
Chr26	5314135	T	C	Chr26_53141	T_C	SNP	5428.29	0	0	0	[illegible]
Chr26	5360864	C	CA	Chr26_53608	C_CA	INDEL	15342.59	31	10	11	[illegible]
Chr26	5361011	T	C	Chr26_53610	T_C	SNP	17708.63	29	12	15	[illegible]
Chr26	5375974	A	AATAT	Chr26_53759	A_AATAT	INDEL	2995.93	0	1	0	[illegible]
Chr26	5375974	A	AATAT	Chr26_53759	A_AATAT	INDEL	2995.93	0	1	0	[illegible]
Chr26	5375974	A	AATAT	Chr26_53759	A_AATAT	INDEL	2995.93	0	1	0	[illegible]
Chr26	5375974	A	AATAT	Chr26_53759	A_AATAT	INDEL	2995.93	0	1	0	[illegible]
Chr26	5509465	A	G	Chr26_55094	A_G	SNP	17942.63	31	13	18	[illegible]
Chr26	5537905	TA	T	Chr26_55379	TA_T	INDEL	15399.59	36	8	11	[illegible]
Chr26	5548425	C	T	Chr26_55484	C_T	SNP	17598.63	26	16	15	[illegible]
Chr26	5548532	C	G	Chr26_55485	C_G	SNP	20549.63	38	20	13	[illegible]
Chr26	5550091	C	G	Chr26_55500	C_G	SNP	6613.63	9	6	11	[illegible]
Chr26	5550124	C	T	Chr26_55501	C_T	SNP	8734.63	12	10	16	[illegible]
Chr26	5555092	G	T	Chr26_55550	G_T	SNP	9980.63	13	9	11	[illegible]
Chr26	5582657	AT	A	Chr26_55826	AT_A	INDEL	9073.59	16	12	7	[illegible]
Chr26	5582709	G	T	Chr26_55827	G_T	SNP	13081.63	16	14	11	[illegible]
Chr26	5582710	A	T	Chr26_55827	A_T	SNP	13081.63	16	14	11	[illegible]
Chr26	5584000	C	A	Chr26_55840	C_A	SNP	17066.63	32	12	18	[illegible]
Chr26	5584309	TC	T	Chr26_55843	TC_T	INDEL	16696.59	32	10	16	[illegible]
Chr26	5584856	C	A	Chr26_55848	C_A	SNP	14867.63	27	16	12	[illegible]
Chr26	5587858	A	T	Chr26_55878	A_T	SNP	10561.63	17	6	9	[illegible]
Chr26	5591821	G	GT	Chr26_55918	G_GT	INDEL	16968.59	26	17	17	[illegible]
Chr26	5591918	T	G	Chr26_55919	T_G	SNP	16978.63	19	16	13	[illegible]
Chr26	5591920	A	T	Chr26_55919	A_T	SNP	17203.63	20	16	13	[illegible]
Chr26	5591922	A	G	Chr26_55919	A_G	SNP	17153.63	19	17	13	[illegible]
Chr26	5591933	T	G	Chr26_55919	T_G	SNP	17483.63	18	19	13	[illegible]
Chr26	5592042	A	G	Chr26_55920	A_G	SNP	15837.63	28	18	21	[illegible]
Chr26	5593721	A	C	Chr26_55937	A_C	SNP	9292.63	11	4	7	[illegible]
Chr26	5624058	G	GT	Chr26_56240	G_GT	INDEL	18711.59	30	16	10	[illegible]
Chr26	5624077	C	CT	Chr26_56240	C_CT	INDEL	19329.59	28	16	14	[illegible]
Chr26	5624095	C	A	Chr26_56240	C_A	SNP	17840.63	28	15	12	[illegible]
Chr26	5624132	C	G	Chr26_56241	C_G	SNP	16287.63	31	14	11	[illegible]
Chr26	5630187	GA	G	Chr26_56301	GA_G	INDEL	15874.59	36	19	17	[illegible]
Chr26	5630235	T	C	Chr26_56302	T_C	SNP	17902.63	36	17	20	[illegible]
Chr26	5658302	A	T	Chr26_56583	A_T	SNP	17805.63	26	14	14	[illegible]
Chr26	5961113	G	A	Chr26_59611	G_A	SNP	3630.98	0	0	0	[illegible]
Chr26	5961113	G	A	Chr26_59611	G_A	SNP	3630.98	0	0	0	[illegible]
Chr26	5961113	G	A	Chr26_59611	G_A	SNP	3630.98	0	0	0	[illegible]
Chr26	6308377	G	T	Chr26_63083	G_T	SNP	17263.63	28	15	10	[illegible]
Chr26	6424200	G	GA	Chr26_64242	G_GA	INDEL	13561.59	35	12	9	[illegible]
Chr26	6424200	G	GA	Chr26_64242	G_GA	INDEL	13561.59	35	12	9	[illegible]
Chr26	6424200	G	GA	Chr26_64242	G_GA	INDEL	13561.59	35	12	9	[illegible]
Chr26	6514012	AC	A	Chr26_65140	AC_A	INDEL	16178.59	28	12	20	[illegible]
Chr26	6514012	AC	A	Chr26_65140	AC_A	INDEL	16178.59	28	12	20	[illegible]
Chr26	6514012	AC	A	Chr26_65140	AC_A	INDEL	16178.59	28	12	20	[illegible]
Chr26	6630678	G	GT	Chr26_66306	G_GT	INDEL	14484.59	23	9	11	[illegible]
Chr26	6630678	G	GT	Chr26_66306	G_GT	INDEL	14484.59	23	9	11	[illegible]
Chr26	6630678	G	GT	Chr26_66306	G_GT	INDEL	14484.59	23	9	11	[illegible]
Chr26	6642282	C	A	Chr26_66422	C_A	SNP	20472.63	28	17	19	[illegible]
Chr26	6746525	C	T	Chr26_67465	C_T	SNP	15718.63	29	15	14	[illegible]
Chr26	6746525	C	T	Chr26_67465	C_T	SNP	15718.63	29	15	14	[illegible]
Chr26	6746525	C	T	Chr26_67465	C_T	SNP	15718.63	29	15	14	[illegible]
Chr26	6746525	C	T	Chr26_67465	C_T	SNP	15718.63	29	15	14	[illegible]
Chr26	6746525	C	T	Chr26_67465	C_T	SNP	15718.63	29	15	14	[illegible]
Chr26	6746525	C	T	Chr26_67465	C_T	SNP	15718.63	29	15	14	[illegible]
Chr26	6746525	C	T	Chr26_67465	C_T	SNP	15718.63	29	15	14	[illegible]
Chr26	6746525	C	T	Chr26_67465	C_T	SNP	15718.63	29	15	14	[illegible]
Chr26	6746525	C	T	Chr26_67465	C_T	SNP	15718.63	29	15	14	[illegible]
Chr26	6746525	C	T	Chr26_67465	C_T	SNP	15718.63	29	15	14	[illegible]
Chr26	6814291	A	AT	Chr26_68142	A_AT	INDEL	16280.59	31	15	12	[illegible]
Chr26	6814291	A	AT	Chr26_68142	A_AT	INDEL	16280.59	31	15	12	[illegible]
Chr26	6943752	T	TG	Chr26_69437	T_TG	INDEL	18266.59	35	12	22	[illegible]
Chr26	6946608	A	T	Chr26_69466	A_T	SNP	13836.63	35	8	12	[illegible]
Chr26	6946615	T	A	Chr26_69466	T_A	SNP	13922.63	35	7	13	[illegible]
Chr26	7244829	C	CAT	Chr26_72448	C_CAT	INDEL	1365.35	26	12	10	[illegible]
Chr26	7244829	C	CAT	Chr26_72448	C_CAT	INDEL	1365.35	26	12	10	[illegible]
Chr26	7244829	C	CAT	Chr26_72448	C_CAT	INDEL	1365.35	26	12	10	[illegible]
Chr26	7244829	C	CAT	Chr26_72448	C_CAT	INDEL	1365.35	26	12	10	[illegible]
Chr26	7244829	C	CAT	Chr26_72448	C_CAT	INDEL	1365.35	26	12	10	[illegible]
Chr26	7244829	C	CAT	Chr26_72448	C_CAT	INDEL	1365.35	26	12	10	[illegible]
Chr26	7323313	A	T	Chr26_73233	A_T	SNP	15989.63	17	16	17	[illegible]
Chr26	7323313	A	T	Chr26_73233	A_T	SNP	15989.63	17	16	17	[illegible]
Chr26	7323313	A	T	Chr26_73233	A_T	SNP	15989.63	17	16	17	[illegible]
Chr26	7323313	A	T	Chr26_73233	A_T	SNP	15989.63	17	16	17	[illegible]
Chr26	7323313	A	T	Chr26_73233	A_T	SNP	15989.63	17	16	17	[illegible]
Chr26	7323313	A	T	Chr26_73233	A_T	SNP	15989.63	17	16	17	[illegible]
Chr26	7446826	TA	T	Chr26_74468	TA_T	INDEL	16124.59	26	18	22	[illegible]
Chr26	7674519	C	CGTCCTTGT	Chr26_76745	C_CGTCCTTG	INDEL	18446.59	18	9	16	[illegible]
Chr26	7674519	C	CGTCCTTGT	Chr26_76745	C_CGTCCTTG	INDEL	18446.59	18	9	16	[illegible]
Chr26	7674519	C	CGTCCTTGT	Chr26_76745	C_CGTCCTTG	INDEL	18446.59	18	9	16	[illegible]
Chr26	7674519	C	CGTCCTTGT	Chr26_76745	C_CGTCCTTG	INDEL	18446.59	18	9	16	[illegible]
Chr26	7674519	C	CGTCCTTGT	Chr26_76745	C_CGTCCTTG	INDEL	18446.59	18	9	16	[illegible]
Chr26	7674519	C	CGTCCTTGT	Chr26_76745	C_CGTCCTTG	INDEL	18446.59	18	9	16	[illegible]
Chr26	7674519	C	CGTCCTTGT	Chr26_76745	C_CGTCCTTG	INDEL	18446.59	18	9	16	[illegible]
Chr26	7674521	C	CG	Chr26_76745	C_CG	INDEL	18806.59	19	7	14	[illegible]
Chr26	7674521	C	CG	Chr26_76745	C_CG	INDEL	18806.59	19	7	14	[illegible]
Chr26	7674521	C	CG	Chr26_76745	C_CG	INDEL	18806.59	19	7	14	[illegible]
Chr26	7674521	C	CG	Chr26_76745	C_CG	INDEL	18806.59	19	7	14	[illegible]
Chr26	7674521	C	CG	Chr26_76745	C_CG	INDEL	18806.59	19	7	14	[illegible]
Chr26	7674521	C	CG	Chr26_76745	C_CG	INDEL	18806.59	19	7	14	[illegible]
Chr26	7674521	C	CG	Chr26_76745	C_CG	INDEL	18806.59	19	7	14	[illegible]
Chr26	7674524	T	A	Chr26_76745	T_A	SNP	17795.63	19	7	13	[illegible]
Chr26	7674524	T	A	Chr26_76745	T_A	SNP	17795.63	19	7	13	[illegible]
Chr26	7674524	T	A	Chr26_76745	T_A	SNP	17795.63	19	7	13	[illegible]
Chr26	7674524	T	A	Chr26_76745	T_A	SNP	17795.63	19	7	13	[illegible]
Chr26	7674524	T	A	Chr26_76745	T_A	SNP	17795.63	19	7	13	[illegible]
Chr26	7674524	T	A	Chr26_76745	T_A	SNP	17795.63	19	7	13	[illegible]
Chr26	7674524	T	A	Chr26_76745	T_A	SNP	17795.63	19	7	13	[illegible]
Chr26	7674527	G	GGCTT	Chr26_76745	G_GGCTT	INDEL	18281.59	19	8	13	[illegible]
Chr26	7674527	G	GGCTT	Chr26_76745	G_GGCTT	INDEL	18281.59	19	8	13	[illegible]
Chr26	7674527	G	GGCTT	Chr26_76745	G_GGCTT	INDEL	18281.59	19	8	13	[illegible]
Chr26	7674527	G	GGCTT	Chr26_76745	G_GGCTT	INDEL	18281.59	19	8	13	[illegible]
Chr26	7674527	G	GGCTT	Chr26_76745	G_GGCTT	INDEL	18281.59	19	8	13	[illegible]
Chr26	7674527	G	GGCTT	Chr26_76745	G_GGCTT	INDEL	18281.59	19	8	13	[illegible]
Chr26	7674527	G	GGCTT	Chr26_76745	G_GGCTT	INDEL	18281.59	19	8	13	[illegible]
Chr26	7674546	CTTTTG	C	Chr26_76745	CTTTTG_C	INDEL	17426.59	22	8	10	[illegible]
Chr26	7674546	CTTTTG	C	Chr26_76745	CTTTTG_C	INDEL	17426.59	22	8	10	[illegible]
Chr26	7674546	CTTTTG	C	Chr26_76745	CTTTTG_C	INDEL	17426.59	22	8	10	[illegible]
Chr26	7674546	CTTTTG	C	Chr26_76745	CTTTTG_C	INDEL	17426.59	22	8	10	[illegible]
Chr26	7674546	CTTTTG	C	Chr26_76745	CTTTTG_C	INDEL	17426.59	22	8	10	[illegible]
Chr26	7674546	CTTTTG	C	Chr26_76745	CTTTTG_C	INDEL	17426.59	22	8	10	[illegible]
Chr26	7674546	CTTTTG	C	Chr26_76745	CTTTTG_C	INDEL	17426.59	22	8	10	[illegible]
Chr26	7766959	AAT	A	Chr26_77669	AAT_A	INDEL	8260.59	20	7	9	[illegible]
Chr26	7766959	AAT	A	Chr26_77669	AAT_A	INDEL	8260.59	20	7	9	[illegible]
Chr26	7766959	AAT	A	Chr26_77669	AAT_A	INDEL	8260.59	20	7	9	[illegible]
Chr26	7766959	AAT	A	Chr26_77669	AAT_A	INDEL	8260.59	20	7	9	[illegible]
Chr26	7766959	AAT	A	Chr26_77669	AAT_A	INDEL	8260.59	20	7	9	[illegible]
Chr26	7766959	AAT	A	Chr26_77669	AAT_A	INDEL	8260.59	20	7	9	[illegible]
Chr26	7803532	A	AAT	Chr26_78035	A_AAT	INDEL	3789.24	19	8	10	[illegible]
Chr26	7803532	A	AAT	Chr26_78035	A_AAT	INDEL	3789.24	19	8	10	[illegible]
Chr26	7803532	A	AAT	Chr26_78035	A_AAT	INDEL	3789.24	19	8	10	[illegible]
Chr26	7827420	AAAGATAGT	A	Chr26_78274	AAAGATAGT	INDEL	17422.59	23	7	11	[illegible]
Chr26	7919246	T	A	Chr26_79192	T_A	SNP	19229.63	28	7	22	[illegible]

Chr26	7919257	A	T	Chr26_79192	A_T	SNP	19524.63
Chr26	7919261	A	T	Chr26_79192	A_T	SNP	18968.63
Chr26	8021982	C	T	Chr26_80219	C_T	SNP	18604.63
Chr26	8022167	G	T	Chr26_80221	G_T	SNP	11534.63
Chr26	8022222	G	T	Chr26_80222	G_T	SNP	20016.63
Chr26	8022234	TG	T	Chr26_80222	TG_T	INDEL	19784.59
Chr26	8037579	T	TTA	Chr26_80375	T_TTA	INDEL	4993.59
Chr26	8245924	C	CT	Chr26_82459	C_CT	INDEL	16082.59
Chr26	8245924	C	CT	Chr26_82459	C_CT	INDEL	16082.59
Chr26	8245924	C	CT	Chr26_82459	C_CT	INDEL	16082.59
Chr26	8245924	C	CT	Chr26_82459	C_CT	INDEL	16082.59
Chr26	8245924	C	CT	Chr26_82459	C_CT	INDEL	16082.59
Chr26	8245924	C	CT	Chr26_82459	C_CT	INDEL	16082.59
Chr26	8245924	C	CT	Chr26_82459	C_CT	INDEL	16082.59
Chr26	8339280	G	T	Chr26_83392	G_T	SNP	17102.63
Chr26	8339280	G	T	Chr26_83392	G_T	SNP	17102.63
Chr26	8339414	G	C	Chr26_83394	G_C	SNP	19512.63
Chr26	8339414	G	C	Chr26_83394	G_C	SNP	19512.63
Chr26	8340746	A	G	Chr26_83407	A_G	SNP	17936.63
Chr26	8340746	A	G	Chr26_83407	A_G	SNP	17936.63
Chr26	8353737	AAT	A	Chr26_83537	AAT_A	INDEL	1144.35
Chr26	8353737	AAT	A	Chr26_83537	AAT_A	INDEL	1144.35
Chr26	8353737	AAT	A	Chr26_83537	AAT_A	INDEL	1144.35
Chr26	8353737	AAT	A	Chr26_83537	AAT_A	INDEL	1144.35
Chr26	8353737	AAT	A	Chr26_83537	AAT_A	INDEL	1144.35
Chr26	8353737	AAT	A	Chr26_83537	AAT_A	INDEL	1144.35
Chr26	8353737	AAT	A	Chr26_83537	AAT_A	INDEL	1144.35
Chr26	8353737	AAT	A	Chr26_83537	AAT_A	INDEL	1144.35
Chr26	8521070	G	A	Chr26_85210	G_A	SNP	15994.63
Chr26	8521070	G	A	Chr26_85210	G_A	SNP	15994.63
Chr26	8521070	G	A	Chr26_85210	G_A	SNP	15994.63
Chr26	8521070	G	A	Chr26_85210	G_A	SNP	15994.63
Chr26	8521151	G	T	Chr26_85211	G_T	SNP	19729.63
Chr26	8521151	G	T	Chr26_85211	G_T	SNP	19729.63
Chr26	8521151	G	T	Chr26_85211	G_T	SNP	19729.63
Chr26	8521151	G	T	Chr26_85211	G_T	SNP	19729.63
Chr26	8521172	C	G	Chr26_85211	C_G	SNP	20586.63
Chr26	8521172	C	G	Chr26_85211	C_G	SNP	20586.63
Chr26	8521172	C	G	Chr26_85211	C_G	SNP	20586.63
Chr26	8521172	C	G	Chr26_85211	C_G	SNP	20586.63
Chr26	8521173	A	C	Chr26_85211	A_C	SNP	20648.63
Chr26	8521173	A	C	Chr26_85211	A_C	SNP	20648.63
Chr26	8521173	A	C	Chr26_85211	A_C	SNP	20648.63
Chr26	8521173	A	C	Chr26_85211	A_C	SNP	20648.63
Chr26	8521178	CT	C	Chr26_85211	CT_C	INDEL	20210.59
Chr26	8521178	CT	C	Chr26_85211	CT_C	INDEL	20210.59
Chr26	8521178	CT	C	Chr26_85211	CT_C	INDEL	20210.59
Chr26	8521178	CT	C	Chr26_85211	CT_C	INDEL	20210.59
Chr26	8581598	T	G	Chr26_85815	T_G	SNP	19256.63
Chr26	8758252	A	G	Chr26_87582	A_G	SNP	20127.63
Chr26	8758252	A	G	Chr26_87582	A_G	SNP	20127.63
Chr26	8758252	A	G	Chr26_87582	A_G	SNP	20127.63
Chr26	8758252	A	G	Chr26_87582	A_G	SNP	20127.63
Chr26	8758252	A	G	Chr26_87582	A_G	SNP	20127.63
Chr26	8758252	A	G	Chr26_87582	A_G	SNP	20127.63
Chr26	8778944	C	A	Chr26_87789	C_A	SNP	18766.63
Chr26	8800628	G	GAT	Chr26_88006	G_GAT	INDEL	1933.24
Chr26	8800628	G	GAT	Chr26_88006	G_GAT	INDEL	1933.24
Chr26	8800628	G	GAT	Chr26_88006	G_GAT	INDEL	1933.24
Chr26	8800628	G	GAT	Chr26_88006	G_GAT	INDEL	1933.24
Chr26	8800628	G	GAT	Chr26_88006	G_GAT	INDEL	1933.24
Chr26	8820794	C	CAT	Chr26_88207	C_CAT	INDEL	6678.24
Chr26	8901656	A	G	Chr26_89016	A_G	SNP	16407.63
Chr26	8929083	A	AT	Chr26_89290	A_AT	INDEL	21774.59
Chr26	8929083	A	AT	Chr26_89290	A_AT	INDEL	21774.59
Chr26	8929102	A	T	Chr26_89291	A_T	SNP	22425.63
Chr26	8929102	A	T	Chr26_89291	A_T	SNP	22425.63
Chr26	8929153	G	T	Chr26_89291	G_T	SNP	20693.63
Chr26	8929153	G	T	Chr26_89291	G_T	SNP	20693.63
Chr26	9105021	C	CAAAGCT	Chr26_91050	C_CAAAGCT	INDEL	20498.59
Chr26	9105021	C	CAAAGCT	Chr26_91050	C_CAAAGCT	INDEL	20498.59
Chr26	9105021	C	CAAAGCT	Chr26_91050	C_CAAAGCT	INDEL	20498.59
Chr26	9105021	C	CAAAGCT	Chr26_91050	C_CAAAGCT	INDEL	20498.59
Chr26	9105021	C	CAAAGCT	Chr26_91050	C_CAAAGCT	INDEL	20498.59
Chr26	9105024	T	TGG	Chr26_91050	T_TGG	INDEL	20519.59
Chr26	9105024	T	TGG	Chr26_91050	T_TGG	INDEL	20519.59
Chr26	9105024	T	TGG	Chr26_91050	T_TGG	INDEL	20519.59
Chr26	9105024	T	TGG	Chr26_91050	T_TGG	INDEL	20519.59
Chr26	9105024	T	TGG	Chr26_91050	T_TGG	INDEL	20519.59
Chr26	9105025	TCCAGC	T	Chr26_91050	TCCAGC_T	INDEL	20429.59
Chr26	9105025	TCCAGC	T	Chr26_91050	TCCAGC_T	INDEL	20429.59
Chr26	9105025	TCCAGC	T	Chr26_91050	TCCAGC_T	INDEL	20429.59
Chr26	9105025	TCCAGC	T	Chr26_91050	TCCAGC_T	INDEL	20429.59
Chr26	9105025	TCCAGC	T	Chr26_91050	TCCAGC_T	INDEL	20429.59
Chr26	9312394	G	T	Chr26_93123	G_T	SNP	17450.63
Chr26	9312409	A	T	Chr26_93124	A_T	SNP	18819.63
Chr26	9467165	G	T	Chr26_94671	G_T	SNP	5287.29
Chr26	9493468	C	A	Chr26_94934	C_A	SNP	7389.63
Chr26	9574811	A	G	Chr26_95748	A_G	SNP	19902.63
Chr26	9574812	G	T	Chr26_95748	G_T	SNP	19902.63
Chr26	9574933	TG	T	Chr26_95749	TG_T	INDEL	15369.59
Chr26	9590319	T	TTA	Chr26_95903	T_TTA	INDEL	5499.24
Chr26	9590319	T	TTA	Chr26_95903	T_TTA	INDEL	5499.24
Chr26	9590319	T	TTA	Chr26_95903	T_TTA	INDEL	5499.24
Chr26	9590319	T	TTA	Chr26_95903	T_TTA	INDEL	5499.24
Chr26	9590319	T	TTA	Chr26_95903	T_TTA	INDEL	5499.24
Chr26	9590319	T	TTA	Chr26_95903	T_TTA	INDEL	5499.24
Chr26	9590319	T	TTA	Chr26_95903	T_TTA	INDEL	5499.24
Chr26	9590319	T	TTA	Chr26_95903	T_TTA	INDEL	5499.24
Chr26	9590319	T	TTA	Chr26_95903	T_TTA	INDEL	5499.24
Chr26	9965620	G	C	Chr26_99656	G_C	SNP	18168.63
Chr26	9965620	G	C	Chr26_99656	G_C	SNP	18168.63
Chr26	10013915	A	T	Chr26_10013	A_T	SNP	26337.63
Chr26	10013915	A	T	Chr26_10013	A_T	SNP	26337.63
Chr26	10013915	A	T	Chr26_10013	A_T	SNP	26337.63
Chr26	10013915	A	T	Chr26_10013	A_T	SNP	26337.63
Chr26	10013915	A	T	Chr26_10013	A_T	SNP	26337.63
Chr26	10013916	C	G	Chr26_10013	C_G	SNP	26337.63
Chr26	10013916	C	G	Chr26_10013	C_G	SNP	26337.63
Chr26	10013916	C	G	Chr26_10013	C_G	SNP	26337.63
Chr26	10013916	C	G	Chr26_10013	C_G	SNP	26337.63
Chr26	10013916	C	G	Chr26_10013	C_G	SNP	26337.63
Chr26	10016094	C	T	Chr26_10016	C_T	SNP	17778.63
Chr26	10016094	C	T	Chr26_10016	C_T	SNP	17778.63
Chr26	10016094	C	T	Chr26_10016	C_T	SNP	17778.63
Chr26	10016094	C	T	Chr26_10016	C_T	SNP	17778.63
Chr26	10016094	C	T	Chr26_10016	C_T	SNP	17778.63
Chr26	10016094	C	T	Chr26_10016	C_T	SNP	17778.63
Chr26	10103989	C	T	Chr26_10103	C_T	SNP	15525.63
Chr26	10103989	C	T	Chr26_10103	C_T	SNP	15525.63
Chr26	10103989	C	T	Chr26_10103	C_T	SNP	15525.63
Chr26	10103989	C	T	Chr26_10103	C_T	SNP	15525.63
Chr26	10103989	C	T	Chr26_10103	C_T	SNP	15525.63
Chr26	10110162	C	A	Chr26_10110	C_A	SNP	7601.63
Chr26	10110162	C	A	Chr26_10110	C_A	SNP	7601.63
Chr26	10110162	C	A	Chr26_10110	C_A	SNP	7601.63
Chr26	10110162	C	A	Chr26_10110	C_A	SNP	7601.63
Chr26	10110162	C	A	Chr26_10110	C_A	SNP	7601.63
Chr26	10110162	C	A	Chr26_10110	C_A	SNP	7601.63
Chr27	11273	C	A	Chr27_11273	C_A	SNP	16299.63
Chr27	11275	C	T	Chr27_11275	C_T	SNP	16947.63
Chr27	11277	C	A	Chr27_11277	C_A	SNP	16671.63
Chr27	12683	TTATA	TTA	Chr27_12683	TTATA_TTA	INDEL	8029.59
Chr27	12683	TTATA	TTA	Chr27_12683	TTATA_TTA	INDEL	8029.59
Chr27	12683	TTATA	TTA	Chr27_12683	TTATA_TTA	INDEL	8029.59
Chr27	74870	T	TTA	Chr27_74870	T_TTA	INDEL	2506.24
Chr27	355394	T	A	Chr27_35539	T_A	SNP	9663.63
Chr27	355398	T	TA	Chr27_35539	T_TA	INDEL	9655.59
Chr27	355400	T	A	Chr27_35540	T_A	SNP	9663.63
Chr27	355402	T	A	Chr27_35540	T_A	SNP	16932.63
Chr27	648795	A	AT	Chr27_64879	A_AT	INDEL	14043.59
Chr27	660912	AT	A	Chr27_66091	AT_A	INDEL	18474.59
Chr27	660912	AT	A	Chr27_66091	AT_A	INDEL	18474.59
Chr27	660912	AT	A	Chr27_66091	AT_A	INDEL	18474.59
Chr27	660912	AT	A	Chr27_66091	AT_A	INDEL	18474.59
Chr27	660912	AT	A	Chr27_66091	AT_A	INDEL	18474.59
Chr27	660912	AT	A	Chr27_66091	AT_A	INDEL	18474.59
Chr27	660912	AT	A	Chr27_66091	AT_A	INDEL	18474.59
Chr27	660919	G	GA	Chr27_66091	G_GA	INDEL	18956.59
Chr27	660919	G	GA	Chr27_66091	G_GA	INDEL	18956.59
Chr27	660919	G	GA	Chr27_66091	G_GA	INDEL	18956.59
Chr27	660919	G	GA	Chr27_66091	G_GA	INDEL	18956.59
Chr27	660919	G	GA	Chr27_66091	G_GA	INDEL	18956.59
Chr27	660919	G	GA	Chr27_66091	G_GA	INDEL	18956.59
Chr27	660919	G	GA	Chr27_66091	G_GA	INDEL	18956.59
Chr27	706030	G	GAT	Chr27_70603	G_GAT	INDEL	1665.35
Chr27	723426	T	A	Chr27_72342	T_A	SNP	16508.63
Chr27	723426	T	A	Chr27_72342	T_A	SNP	16508.63
Chr27	723426	T	A	Chr27_72342	T_A	SNP	16508.63
Chr27	743755	T	TTA	Chr27_74375	T_TTA	INDEL	1992.93
Chr27	743755	T	TTA	Chr27_74375	T_TTA	INDEL	1992.93
Chr27	743755	T	TTA	Chr27_74375	T_TTA	INDEL	1992.93
Chr27	770951	TTA	TTATA	Chr27_77095	TTA_TTATA	INDEL	6699.11
Chr27	773039	C	T	Chr27_77303	C_T	SNP	21841.63
Chr27	773039	C	T	Chr27_77303	C_T	SNP	21841.63
Chr27	869515	C	CTA	Chr27_86951	C_CTA	INDEL	7317.59
Chr27	869515	C	CTA	Chr27_86951	C_CTA	INDEL	7317.59
Chr27	1000500	TGG	T	Chr27_10005	TGG_T	INDEL	19353.59
Chr27	1000502	G	GCAA	Chr27_10005	G_GCAA	INDEL	19353.59
Chr27	1363479	A	AT	Chr27_13634	A_AT	INDEL	14244.59
Chr27	1377587	T	C	Chr27_13775	T_C	SNP	11814.63
Chr27	1390448	T	TA	Chr27_13904	T_TA	INDEL	8479.59
Chr27	1390448	T	TA	Chr27_13904	T_TA	INDEL	8479.59
Chr27	1390448	T	TA	Chr27_13904	T_TA	INDEL	8479.59
Chr27	1390449	T	TA	Chr27_13904	T_TA	INDEL	15226.59
Chr27	1390449	T	TA	Chr27_13904	T_TA	INDEL	15226.59
Chr27	1390449	T	TA	Chr27_13904	T_TA	INDEL	15226.59
Chr27	1501615	A	C	Chr27_15016	A_C	SNP	6566.29
Chr27	1501615	A	C	Chr27_15016	A_C	SNP	6566.29
Chr27	1501615	A	C	Chr27_15016	A_C	SNP	6566.29
Chr27	1543577	CTA	C	Chr27_15435	CTA_C	INDEL	5229.24
Chr27	1702056	C	A	Chr27_17020	C_A	SNP	8969.63
Chr27	1702056	C	A	Chr27_17020	C_A	SNP	8969.63
Chr27	1702056	C	A	Chr27_17020	C_A	SNP	8969.63
Chr27	1702056	C	A	Chr27_17020	C_A	SNP	8969.63
Chr27	1730052	C	CAT	Chr27_17300	C_CAT	INDEL	1308.35
Chr27	1730052	C	CAT	Chr27_17300	C_CAT	INDEL	1308.35
Chr27	1730052	C	CAT	Chr27_17300	C_CAT	INDEL	1308.35
Chr27	1730052	C	CAT	Chr27_17300	C_CAT	INDEL	1308.35
Chr27	1730052	C	CAT	Chr27_17300	C_CAT	INDEL	1308.35
Chr27	1771990	A	AAT	Chr27_17719	A_AAT	INDEL	7835.59
Chr27	1771990	A	AAT	Chr27_17719	A_AAT	INDEL	7835.59
Chr27	1771990	A	AAT	Chr27_17719	A_AAT	INDEL	7835.59
Chr27	1771990	A	AAT	Chr27_17719	A_AAT	INDEL	7835.59
Chr27	1771990	A	AAT	Chr27_17719	A_AAT	INDEL	7835.59
Chr27	1774642	C	T	Chr27_17746	C_T	SNP	14668.63
Chr27	1774642	C	T	Chr27_17746	C_T	SNP	14668.63
Chr27	1774642	C	T	Chr27_17746	C_T	SNP	14668.63
Chr27	1774642	C	T	Chr27_17746	C_T	SNP	14668.63
Chr27	1877399	A	AT	Chr27_18773	A_AT	INDEL	13427.59
Chr27	1877399	A	AT	Chr27_18773	A_AT	INDEL	13427.59
Chr27	1877399	A	AT	Chr27_18773	A_AT	INDEL	13427.59

Chr27	1877399	A	AT	Chr27_18773	A_AT	INDEL	13427.59	[illegible]	7	5	3	downstream	MODIFIER	Pp3c27_329(	Pp3c27_329(	transcript	PAC:3295240	protein_codi	NA	c.*679_*680	NA	NA	NA	NA	662
Chr27	1877399	A	AT	Chr27_18773	A_AT	INDEL	13427.59	[illegible]	7	5	3	downstream	MODIFIER	Pp3c27_329(	Pp3c27_329(	transcript	PAC:3295241	protein_codi	NA	c.*1521_*15	NA	NA	NA	NA	1497
Chr27	1877399	A	AT	Chr27_18773	A_AT	INDEL	13427.59	[illegible]	7	5	3	downstream	MODIFIER	Pp3c27_329(	Pp3c27_329(	transcript	PAC:3295240	protein_codi	NA	c.*1538_*15	NA	NA	NA	NA	1497
Chr27	1877399	A	AT	Chr27_18773	A_AT	INDEL	13427.59	[illegible]	7	5	3	downstream	MODIFIER	Pp3c27_329(	Pp3c27_329(	transcript	PAC:3295241	protein_codi	NA	c.*1521_*15	NA	NA	NA	NA	1497
Chr27	1877399	A	AT	Chr27_18773	A_AT	INDEL	13427.59	[illegible]	7	5	3	intergenic_re	MODIFIER	Pp3c27_328(	Pp3c27_328(	intergenic_re	Pp3c27_328(	NA	NA	n.1877399_1	NA	NA	NA	NA	NA
Chr27	1877408	AACTCGCCCC	A	Chr27_18774	AACTCGCCCC	INDEL	14428.59	[illegible]	7	5	3	downstream	MODIFIER	Pp3c27_328(	Pp3c27_328(	transcript	PAC:3295166	protein_codi	NA	c.*1966_*19	NA	NA	NA	NA	1966
Chr27	1877408	AACTCGCCCC	A	Chr27_18774	AACTCGCCCC	INDEL	14428.59	[illegible]	7	5	3	downstream	MODIFIER	Pp3c27_329(	Pp3c27_329(	transcript	PAC:3295240	protein_codi	NA	c.*658_*670	NA	NA	NA	NA	670
Chr27	1877408	AACTCGCCCC	A	Chr27_18774	AACTCGCCCC	INDEL	14428.59	[illegible]	7	5	3	downstream	MODIFIER	Pp3c27_328(	Pp3c27_328(	transcript	PAC:3295166	protein_codi	NA	c.*2176_*21	NA	NA	NA	NA	2176
Chr27	1877408	AACTCGCCCC	A	Chr27_18774	AACTCGCCCC	INDEL	14428.59	[illegible]	7	5	3	downstream	MODIFIER	Pp3c27_329(	Pp3c27_329(	transcript	PAC:3295240	protein_codi	NA	c.*658_*670	NA	NA	NA	NA	653
Chr27	1877408	AACTCGCCCC	A	Chr27_18774	AACTCGCCCC	INDEL	14428.59	[illegible]	7	5	3	downstream	MODIFIER	Pp3c27_329(	Pp3c27_329(	transcript	PAC:3295241	protein_codi	NA	c.*1500_*15	NA	NA	NA	NA	1488
Chr27	1877408	AACTCGCCCC	A	Chr27_18774	AACTCGCCCC	INDEL	14428.59	[illegible]	7	5	3	downstream	MODIFIER	Pp3c27_329(	Pp3c27_329(	transcript	PAC:3295240	protein_codi	NA	c.*1517_*15	NA	NA	NA	NA	1488
Chr27	1877408	AACTCGCCCC	A	Chr27_18774	AACTCGCCCC	INDEL	14428.59	[illegible]	7	5	3	downstream	MODIFIER	Pp3c27_329(	Pp3c27_329(	transcript	PAC:3295241	protein_codi	NA	c.*1500_*15	NA	NA	NA	NA	1488
Chr27	1877408	AACTCGCCCC	A	Chr27_18774	AACTCGCCCC	INDEL	14428.59	[illegible]	7	5	3	intergenic_re	MODIFIER	Pp3c27_328(	Pp3c27_328(	intergenic_re	Pp3c27_328(	NA	NA	n.1877409_1	NA	NA	NA	NA	NA
Chr27	1877423	T	A	Chr27_18774	T_A	SNP	14915.63	[illegible]	7	5	3	downstream	MODIFIER	Pp3c27_328(	Pp3c27_328(	transcript	PAC:3295166	protein_codi	NA	c.*1980T>A	NA	NA	NA	NA	1980
Chr27	1877423	T	A	Chr27_18774	T_A	SNP	14915.63	[illegible]	7	5	3	downstream	MODIFIER	Pp3c27_329(	Pp3c27_329(	transcript	PAC:3295240	protein_codi	NA	c.*656A>T	NA	NA	NA	NA	656
Chr27	1877423	T	A	Chr27_18774	T_A	SNP	14915.63	[illegible]	7	5	3	downstream	MODIFIER	Pp3c27_328(	Pp3c27_328(	transcript	PAC:3295166	protein_codi	NA	c.*2190T>A	NA	NA	NA	NA	2190
Chr27	1877423	T	A	Chr27_18774	T_A	SNP	14915.63	[illegible]	7	5	3	downstream	MODIFIER	Pp3c27_329(	Pp3c27_329(	transcript	PAC:3295240	protein_codi	NA	c.*656A>T	NA	NA	NA	NA	639
Chr27	1877423	T	A	Chr27_18774	T_A	SNP	14915.63	[illegible]	7	5	3	downstream	MODIFIER	Pp3c27_329(	Pp3c27_329(	transcript	PAC:3295241	protein_codi	NA	c.*1498A>T	NA	NA	NA	NA	1474
Chr27	1877423	T	A	Chr27_18774	T_A	SNP	14915.63	[illegible]	7	5	3	downstream	MODIFIER	Pp3c27_329(	Pp3c27_329(	transcript	PAC:3295240	protein_codi	NA	c.*1515A>T	NA	NA	NA	NA	1474
Chr27	1877423	T	A	Chr27_18774	T_A	SNP	14915.63	[illegible]	7	5	3	downstream	MODIFIER	Pp3c27_329(	Pp3c27_329(	transcript	PAC:3295241	protein_codi	NA	c.*1498A>T	NA	NA	NA	NA	1474
Chr27	1877423	T	A	Chr27_18774	T_A	SNP	14915.63	[illegible]	7	5	3	intergenic_re	MODIFIER	Pp3c27_328(	Pp3c27_328(	intergenic_re	Pp3c27_328(	NA	NA	n.1877423T>	NA	NA	NA	NA	NA
Chr27	1877424	T	TGA	Chr27_18774	T_TGA	INDEL	14996.59	[illegible]	7	5	3	downstream	MODIFIER	Pp3c27_328(	Pp3c27_328(	transcript	PAC:3295166	protein_codi	NA	c.*1981_*19	NA	NA	NA	NA	1982
Chr27	1877424	T	TGA	Chr27_18774	T_TGA	INDEL	14996.59	[illegible]	7	5	3	downstream	MODIFIER	Pp3c27_329(	Pp3c27_329(	transcript	PAC:3295240	protein_codi	NA	c.*654_*655	NA	NA	NA	NA	654
Chr27	1877424	T	TGA	Chr27_18774	T_TGA	INDEL	14996.59	[illegible]	7	5	3	downstream	MODIFIER	Pp3c27_328(	Pp3c27_328(	transcript	PAC:3295166	protein_codi	NA	c.*2191_*21	NA	NA	NA	NA	2192
Chr27	1877424	T	TGA	Chr27_18774	T_TGA	INDEL	14996.59	[illegible]	7	5	3	downstream	MODIFIER	Pp3c27_329(	Pp3c27_329(	transcript	PAC:3295240	protein_codi	NA	c.*654_*655	NA	NA	NA	NA	637
Chr27	1877424	T	TGA	Chr27_18774	T_TGA	INDEL	14996.59	[illegible]	7	5	3	downstream	MODIFIER	Pp3c27_329(	Pp3c27_329(	transcript	PAC:3295241	protein_codi	NA	c.*1496_*14	NA	NA	NA	NA	1472
Chr27	1877424	T	TGA	Chr27_18774	T_TGA	INDEL	14996.59	[illegible]	7	5	3	downstream	MODIFIER	Pp3c27_329(	Pp3c27_329(	transcript	PAC:3295240	protein_codi	NA	c.*1513_*15	NA	NA	NA	NA	1472
Chr27	1877424	T	TGA	Chr27_18774	T_TGA	INDEL	14996.59	[illegible]	7	5	3	downstream	MODIFIER	Pp3c27_329(	Pp3c27_329(	transcript	PAC:3295241	protein_codi	NA	c.*1496_*14	NA	NA	NA	NA	1472
Chr27	1877424	T	TGA	Chr27_18774	T_TGA	INDEL	14996.59	[illegible]	7	5	3	intergenic_re	MODIFIER	Pp3c27_328(	Pp3c27_328(	intergenic_re	Pp3c27_328(	NA	NA	n.1877424_1	NA	NA	NA	NA	NA
Chr27	1877426	T	A	Chr27_18774	T_A	SNP	15185.63	[illegible]	7	5	3	downstream	MODIFIER	Pp3c27_328(	Pp3c27_328(	transcript	PAC:3295166	protein_codi	NA	c.*1983T>A	NA	NA	NA	NA	1983
Chr27	1877426	T	A	Chr27_18774	T_A	SNP	15185.63	[illegible]	7	5	3	downstream	MODIFIER	Pp3c27_329(	Pp3c27_329(	transcript	PAC:3295240	protein_codi	NA	c.*653A>T	NA	NA	NA	NA	653
Chr27	1877426	T	A	Chr27_18774	T_A	SNP	15185.63	[illegible]	7	5	3	downstream	MODIFIER	Pp3c27_328(	Pp3c27_328(	transcript	PAC:3295166	protein_codi	NA	c.*2193T>A	NA	NA	NA	NA	2193
Chr27	1877426	T	A	Chr27_18774	T_A	SNP	15185.63	[illegible]	7	5	3	downstream	MODIFIER	Pp3c27_329(	Pp3c27_329(	transcript	PAC:3295240	protein_codi	NA	c.*653A>T	NA	NA	NA	NA	636
Chr27	1877426	T	A	Chr27_18774	T_A	SNP	15185.63	[illegible]	7	5	3	downstream	MODIFIER	Pp3c27_329(	Pp3c27_329(	transcript	PAC:3295241	protein_codi	NA	c.*1495A>T	NA	NA	NA	NA	1471
Chr27	1877426	T	A	Chr27_18774	T_A	SNP	15185.63	[illegible]	7	5	3	downstream	MODIFIER	Pp3c27_329(	Pp3c27_329(	transcript	PAC:3295240	protein_codi	NA	c.*1512A>T	NA	NA	NA	NA	1471
Chr27	1877426	T	A	Chr27_18774	T_A	SNP	15185.63	[illegible]	7	5	3	downstream	MODIFIER	Pp3c27_329(	Pp3c27_329(	transcript	PAC:3295241	protein_codi	NA	c.*1495A>T	NA	NA	NA	NA	1471
Chr27	1877426	T	A	Chr27_18774	T_A	SNP	15185.63	[illegible]	7	5	3	intergenic_re	MODIFIER	Pp3c27_328(	Pp3c27_328(	intergenic_re	Pp3c27_328(	NA	NA	n.1877426T>	NA	NA	NA	NA	NA
Chr27	1877429	T	TGACACATC	Chr27_18774	T_TGACACAT	INDEL	16777.59	[illegible]	7	5	3	downstream	MODIFIER	Pp3c27_328(	Pp3c27_328(	transcript	PAC:3295166	protein_codi	NA	c.*1986_*19	NA	NA	NA	NA	1987
Chr27	1877429	T	TGACACATC	Chr27_18774	T_TGACACAT	INDEL	16777.59	[illegible]	7	5	3	downstream	MODIFIER	Pp3c27_329(	Pp3c27_329(	transcript	PAC:3295240	protein_codi	NA	c.*649_*650	NA	NA	NA	NA	649
Chr27	1877429	T	TGACACATC	Chr27_18774	T_TGACACAT	INDEL	16777.59	[illegible]	7	5	3	downstream	MODIFIER	Pp3c27_328(	Pp3c27_328(	transcript	PAC:3295166	protein_codi	NA	c.*2196_*21	NA	NA	NA	NA	2197
Chr27	1877429	T	TGACACATC	Chr27_18774	T_TGACACAT	INDEL	16777.59	[illegible]	7	5	3	downstream	MODIFIER	Pp3c27_329(	Pp3c27_329(	transcript	PAC:3295240	protein_codi	NA	c.*649_*650	NA	NA	NA	NA	632
Chr27	1877429	T	TGACACATC	Chr27_18774	T_TGACACAT	INDEL	16777.59	[illegible]	7	5	3	downstream	MODIFIER	Pp3c27_329(	Pp3c27_329(	transcript	PAC:3295241	protein_codi	NA	c.*1491_*14	NA	NA	NA	NA	1467
Chr27	1877429	T	TGACACATC	Chr27_18774	T_TGACACAT	INDEL	16777.59	[illegible]	7	5	3	downstream	MODIFIER	Pp3c27_329(	Pp3c27_329(	transcript	PAC:3295240	protein_codi	NA	c.*1508_*15	NA	NA	NA	NA	1467
Chr27	1877429	T	TGACACATC	Chr27_18774	T_TGACACAT	INDEL	16777.59	[illegible]	7	5	3	downstream	MODIFIER	Pp3c27_329(	Pp3c27_329(	transcript	PAC:3295241	protein_codi	NA	c.*1491_*14	NA	NA	NA	NA	1467
Chr27	1877429	T	TGACACATC	Chr27_18774	T_TGACACAT	INDEL	16777.59	[illegible]	7	5	3	intergenic_re	MODIFIER	Pp3c27_328(	Pp3c27_328(	intergenic_re	Pp3c27_328(	NA	NA	n.1877429_1	NA	NA	NA	NA	NA
Chr27	1877444	G	GT	Chr27_18774	G_GT	INDEL	15215.59	[illegible]	7	5	3	downstream	MODIFIER	Pp3c27_328(	Pp3c27_328(	transcript	PAC:3295166	protein_codi	NA	c.*2001_*20	NA	NA	NA	NA	2002
Chr27	1877444	G	GT	Chr27_18774	G_GT	INDEL	15215.59	[illegible]	7	5	3	downstream	MODIFIER	Pp3c27_329(	Pp3c27_329(	transcript	PAC:3295240	protein_codi	NA	c.*634_*635	NA	NA	NA	NA	634
Chr27	1877444	G	GT	Chr27_18774	G_GT	INDEL	15215.59	[illegible]	7	5	3	downstream	MODIFIER	Pp3c27_328(	Pp3c27_328(	transcript	PAC:3295166	protein_codi	NA	c.*2211_*22	NA	NA	NA	NA	2212
Chr27	1877444	G	GT	Chr27_18774	G_GT	INDEL	15215.59	[illegible]	7	5	3	downstream	MODIFIER	Pp3c27_329(	Pp3c27_329(	transcript	PAC:3295240	protein_codi	NA	c.*634_*635	NA	NA	NA	NA	617
Chr27	1877444	G	GT	Chr27_18774	G_GT	INDEL	15215.59	[illegible]	7	5	3	downstream	MODIFIER	Pp3c27_329(	Pp3c27_329(	transcript	PAC:3295241	protein_codi	NA	c.*1476_*14	NA	NA	NA	NA	1452
Chr27	1877444	G	GT	Chr27_18774	G_GT	INDEL	15215.59	[illegible]	7	5	3	downstream	MODIFIER	Pp3c27_329(	Pp3c27_329(	transcript	PAC:3295240	protein_codi	NA	c.*1493_*14	NA	NA	NA	NA	1452
Chr27	1877444	G	GT	Chr27_18774	G_GT	INDEL	15215.59	[illegible]	7	5	3	downstream	MODIFIER	Pp3c27_329(	Pp3c27_329(	transcript	PAC:3295241	protein_codi	NA	c.*1476_*14	NA	NA	NA	NA	1452
Chr27	1877444	G	GT	Chr27_18774	G_GT	INDEL	15215.59	[illegible]	7	5	3	intergenic_re	MODIFIER	Pp3c27_328(	Pp3c27_328(	intergenic_re	Pp3c27_328(	NA	NA	n.1877444_1	NA	NA	NA	NA	NA
Chr27	1933576	T	TTTATTTATT	Chr27_19335	T_TTTATTTA	INDEL	6138.24	[illegible]	0	5	0	intergenic_re	MODIFIER	Pp3c27_330(	Pp3c27_330(	intergenic_re	Pp3c27_330(	NA	NA	n.1933576_1	NA	NA	NA	NA	NA
Chr27	2013203	T	TTA	Chr27_20132	T_TTA	INDEL	401.37	[illegible]	0	1	0	upstream_ge	MODIFIER	Pp3c27_344(	Pp3c27_344(	transcript	PAC:3295194	protein_codi	NA	c.-4365_-436	NA	NA	NA	NA	3805
Chr27	2013203	T	TTA	Chr27_20132	T_TTA	INDEL	401.37	[illegible]	0	1	0	upstream_ge	MODIFIER	Pp3c27_344(	Pp3c27_344(	transcript	PAC:3295194	protein_codi	NA	c.-4365_-436	NA	NA	NA	NA	3800
Chr27	2013203	T	TTA	Chr27_20132	T_TTA	INDEL	401.37	[illegible]	0	1	0	upstream_ge	MODIFIER	Pp3c27_344(	Pp3c27_344(	transcript	PAC:3295194	protein_codi	NA	c.-4365_-436	NA	NA	NA	NA	3800
Chr27	2013203	T	TTA	Chr27_20132	T_TTA	INDEL	401.37	[illegible]	0	1	0	downstream	MODIFIER	Pp3c27_346(	Pp3c27_346(	transcript	PAC:3295190	protein_codi	NA	c.*1452_*14	NA	NA	NA	NA	920
Chr27	2013203	T	TTA	Chr27_20132	T_TTA	INDEL	401.37	[illegible]	0	1	0	downstream	MODIFIER	Pp3c27_346(	Pp3c27_346(	transcript	PAC:3295191	protein_codi	NA	c.*1452_*14	NA	NA	NA	NA	843
Chr27	2013203	T	TTA	Chr27_20132	T_TTA	INDEL	401.37	[illegible]	0	1	0	downstream	MODIFIER	Pp3c27_346(	Pp3c27_346(	transcript	PAC:3295191	protein_codi	NA	c.*1761_*17	NA	NA	NA	NA	843
Chr27	2013203	T	TTA	Chr27_20132	T_TTA	INDEL	401.37	[illegible]	0	1	0	downstream	MODIFIER	Pp3c27_346(	Pp3c27_346(	transcript	PAC:3295191	protein_codi	NA	c.*1452_*14	NA	NA	NA	NA	920
Chr27	2013203	T	TTA	Chr27_20132	T_TTA	INDEL	401.37	[illegible]	0	1	0	downstream	MODIFIER	Pp3c27_346(	Pp3c27_346(	transcript	PAC:3295191	protein_codi	NA	c.*2365_*23	NA	NA	NA	NA	920
Chr27	2013203	T	TTA	Chr27_20132	T_TTA	INDEL	401.37	[illegible]	0	1	0	downstream	MODIFIER	Pp3c27_346(	Pp3c27_346(	transcript	PAC:3295191	protein_codi	NA	c.*1761_*17	NA	NA	NA	NA	1666
Chr27	2013203	T	TTA	Chr27_20132	T_TTA	INDEL	401.37	[illegible]	0	1	0	intergenic_re	MODIFIER	Pp3c27_344(	Pp3c27_344(	intergenic_re	Pp3c27_344(	NA	NA	n.2013203_2	NA	NA	NA	NA	NA
Chr27	2330313	C	A	Chr27_23303	C_A	SNP	14967.63	[illegible]	7	5	3	missense_va	MODERATE	Pp3c27_416(	Pp3c27_416(	transcript	PAC:3295241	protein_codi	01-janv	c.208C>A	p.Leu70Met	326/1478	208/951	70/316	NA
Chr27	2330313	C	A	Chr27_23303	C_A	SNP	14967.63	[illegible]	7	5	3	missense_va	MODERATE	Pp3c27_416(	Pp3c27_416(	transcript	PAC:3295241	protein_codi	01-janv	c.58C>A	p.Leu20Met	227/1328	58/801	20/266	NA
Chr27	2330313	C	A	Chr27_23303	C_A	SNP	14967.63	[illegible]	7	5	3	3_prime_UTI	MODIFIER	Pp3c27_415(	Pp3c27_415(	transcript	PAC:3295172	protein_codi	05-mai	c.*1864G>T	NA	NA	NA	NA	1864
Chr27	2330313	C	A	Chr27_23303	C_A	SNP	14967.63	[illegible]	7	5	3	upstream_ge	MODIFIER	Pp3c27_414(	Pp3c27_414(	transcript	PAC:3295157	protein_codi	NA	c.-2079G>T	NA	NA	NA	NA	1982
Chr27	2330313	C	A	Chr27_23303	C_A	SNP	14967.63	[illegible]	7	5	3	upstream_ge	MODIFIER	Pp3c27_414(	Pp3c27_414(	transcript	PAC:3295157	protein_codi	NA	c.-2079G>T	NA	NA	NA	NA	1980
Chr27	2330313	C	A	Chr27_23303	C_A	SNP	14967.63	[illegible]	7	5	3	upstream_ge	MODIFIER	Pp3c27_414(	Pp3c27_414(	transcript	PAC:3295157	protein_codi	NA	c.-2079G>T	NA	NA	NA	NA	1980
Chr27	2330313	C	A	Chr27_23303	C_A	SNP	14967.63	[illegible]	7	5	3	downstream	MODIFIER	Pp3c27_415(	Pp3c27_415(	transcript	PAC:3295172	protein_codi	NA	c.*1864G>T	NA	NA	NA	NA	1440
Chr27	2330313	C	A	Chr27_23303	C_A	SNP	14967.63	[illegible]	7	5	3	downstream	MODIFIER	Pp3c27_415(	Pp3c27_415(	transcript	PAC:3295172	protein_codi	NA	c.*1864G>T	NA	NA	NA	NA	1440
Chr27	2330313	C	A	Chr27_23303	C_A	SNP	14967.63	[illegible]	7	5	3	downstream	MODIFIER	Pp3c27_415(	Pp3c27_415(	transcript	PAC:3295173	protein_codi	NA	c.*1864G>T	NA	NA	NA	NA	1473
Chr27	2330313	C	A	Chr27_23303	C_A	SNP	14967.63	[illegible]	7	5	3	downstream	MODIFIER	Pp3c27_415(	Pp3c27_415(	transcript	PAC:3295172	protein_codi	NA	c.*1864G>T	NA	NA	NA	NA	1473
Chr27	2330318	C	G	Chr27_23303	C_G	SNP	14942.63	[illegible]	7	5	3	missense_va	MODERATE	Pp3c27_416(	Pp3c27_416(	transcript	PAC:3295241	protein_codi	01-janv	c.213C>G	p.Asn71Lys	331/1478	213/951	71/316	NA
Chr27	2330318	C	G	Chr27_23303	C_G	SNP	14942.63	[illegible]	7	5	3	missense_va	MODERATE	Pp3c27_416(	Pp3c27_416(	transcript	PAC:3295241	protein_codi	01-janv	c.63C>G	p.Asn21Lys	232/1328	63/801	21/266	NA
Chr27	2330318	C	G	Chr27_23303	C_G	SNP	14942.63	[illegible]	7	5	3	3_prime_UTI	MODIFIER	Pp3c27_415(	Pp3c27_415(	transcript	PAC:3295172	protein_codi	05-mai	c.*1859G>C	NA	NA	NA	NA	1859
Chr27	2330318	C	G	Chr27_23303	C_G	SNP	14942.63	[illegible]	7	5	3	upstream_ge	MODIFIER	Pp3c27_414(	Pp3c27_414(	transcript	PAC:3295157	protein_codi	NA	c.-2084G>C	NA	NA	NA	NA	1987
Chr27	2330318	C	G	Chr27_23303	C_G	SNP	14942.63	[illegible]	7	5	3	upstream_ge	MODIFIER	Pp3c27_414(	Pp3c27_414(	transcript	PAC:3295157	protein_codi	NA	c.-2084G>C	NA	NA	NA	NA	1985
Chr27	2330318	C	G	Chr27_23303	C_G	SNP	14942.63	[illegible]	7	5	3	upstream_ge	MODIFIER	Pp3c27_414(	Pp3c27_414(	transcript	PAC:3295157	protein_codi	NA	c.-2084G>C	NA	NA	NA	NA	1985
Chr27	2330318	C	G	Chr27_23303	C_G	SNP	14942.63	[illegible]	7	5	3	downstream	MODIFIER	Pp3c27_415(	Pp3c27_415(	transcript	PAC:3295172	protein_codi	NA	c.*1859G>C	NA	NA	NA	NA	1435
Chr27	2330318	C	G	Chr27_23303	C_G	SNP	14942.63	[illegible]	7	5	3	downstream	MODIFIER	Pp3c27_415(	Pp3c27_415(	transcript	PAC:3295172	protein_codi	NA	c.*1859G>C	NA	NA	NA	NA	1435
Chr27	2330318	C	G	Chr27_23303	C_G	SNP	14942.63	[illegible]	7	5	3	downstream	MODIFIER	Pp3c27_415(	Pp3c27_415(	transcript	PAC:3295173	protein_codi	NA	c.*1859G>C	NA	NA	NA	NA	1468
Chr27	2330318	C	G	Chr27_23303	C_G	SNP	14942.63	[illegible]	7	5	3	downstream	MODIFIER	Pp3c27_415(	Pp3c27_415(	transcript	PAC:3295172	protein_codi	NA	c.*1859G>C	NA	NA	NA	NA	1468
Chr27	2372340	C	CA	Chr27_23723	C_CA	INDEL	17407.59	[illegible]	7	5	3	intergenic_re	MODIFIER	Pp3c27_425(	Pp3c27_425(	intergenic_re	Pp3c27_425(	NA	NA	n.2372340_2	NA	NA	NA	NA	NA
Chr27	2379191	C	A	Chr27_23791	C_A	SNP	12731.63	[illegible]	7	5	3	missense_va	MODERATE	Pp3c27_426(	Pp3c27_426(	transcript	PAC:3295197	protein_codi	01-avr	c.5C>A	p.Pro2Gln	186/1374	5/918	2/305	NA
Chr27	2379191	C	A	Chr27_23791	C_A	SNP	12731.63	[illegible]	7	5	3	missense_va	MODERATE	Pp3c27_426(	Pp3c27_426(	transcript	PAC:3295197	protein_codi	01-avr	c.5C>A	p.Pro2Gln	168/1284	5/918	2/305	NA
Chr27	2379472	C	G	Chr27_23794	C_G	SNP	9978.63	[illegible]	7	5	3	missense_va	MODERATE	Pp3c27_426(	Pp3c27_426(	transcript	PAC:3295197	protein_codi	01-avr	c.286C>G	p.Pro96Ala	467/1374	286/918	96/305	NA
Chr27	2379472	C	G	Chr27_23794	C_G	SNP	9978.63	[illegible]	7	5	3	missense_va	MODERATE	Pp3c27_426(	Pp3c27_426(	transcript	PAC:3295197	protein_codi	01-avr	c.286C>G	p.Pro96Ala	449/1284	286/918	96/305	NA
Chr27	2388132	C	CAT	Chr27_23881	C_CAT	INDEL	9223.59	[illegible]	7	5	3	intergenic_re	MODIFIER	Pp3c27_426(	Pp3c27_426(	intergenic_re	Pp3c27_426(	NA	NA	n.2388132_2	NA	NA	NA	NA	NA
Chr27	2438202	T	TTA	Chr27_24382	T_TTA	INDEL	62.37	[illegible]	0	0	1	intergenic_re	MODIFIER	Pp3c27_435(	Pp3c27_435(	intergenic_re	Pp3c27_435(	NA	NA	n.2438202_2	NA	NA	NA	NA	NA
Chr27	2486761	A	AAT	Chr27_24867	A_AAT	INDEL	6077.59	[illegible]	7	5	3	downstream	MODIFIER	Pp3c27_440(	Pp3c27_440(	transcript	PAC:3295219	protein_codi	NA	c.*3067_*30	NA	NA	NA	NA	2694
Chr27	2486761	A	AAT	Chr27_24867	A_AAT	INDEL	6077.59	[illegible]	7	5	3	downstream	MODIFIER	Pp3c27_440(	Pp3c27_440(	transcript	PAC:3295219	protein_codi	NA	c.*3067_*30	NA	NA	NA	NA	3068
Chr27	2486761	A	AAT	Chr27_24867	A_AAT	INDEL	6077.59	[illegible]	7	5	3	intergenic_re	MODIFIER	Pp3c27_440(	Pp3c27_440(	intergenic_re	Pp3c27_440(	NA	NA	n.2486761_2	NA	NA	NA	NA	NA
Chr27	2534049	C	CAT	Chr27_25340	C_CAT	INDEL	45.37	[illegible]	1	0	0	intergenic_re	MODIFIER	Pp3c27_444(	Pp3c27_444(	intergenic_re	Pp3c27_444(	NA	NA	n.2534049_2	NA	NA	NA	NA	NA
Chr27	2627806	C	T	Chr27_26278	C_T	SNP	19921.63	[illegible]	7	5	3	downstream	MODIFIER	Pp3c27_457(	Pp3c27_457(	transcript	PAC:3295154	protein_codi	NA	c.*1483G>A	NA	NA	NA	NA	1483
Chr27	2627806	C	T	Chr27_26278	C_T	SNP	19921.63	[illegible]	7	5	3	intergenic_re	MODIFIER	Pp3c27_453(	Pp3c27_453(	intergenic_re	Pp3c27_453(	NA	NA	n.2627806C>	NA	NA	NA	NA	NA
Chr27	2627943	G	T	Chr27_26279	G_T	SNP	16826.63	[illegible]	7	5	3	downstream	MODIFIER	Pp3c27_457(	Pp3c27_457(	transcript	PAC:3295154	protein_codi	NA	c.*1346C>A	NA	NA	NA	NA	1346
Chr27	2627943	G	T	Chr27_26279	G_T	SNP	16826.63	[illegible]	7	5	3	intergenic_re	MODIFIER	Pp3c27_453(	Pp3c27_453(	intergenic_re	Pp3c27_453(	NA	NA	n.2627943G>	NA	NA	NA	NA	NA
Chr27	2628049	A	T	Chr27_26280	A_T	SNP	18512.63	[illegible]	7	5	3	downstream	MODIFIER	Pp3c27_457(	Pp3c27_457(	transcript	PAC:3295154	protein_codi	NA	c.*1240T>A	NA	NA	NA	NA	1240
Chr27	2628049	A	T	Chr27_26280	A_T	SNP	18512.63	[illegible]	7	5	3	intergenic_re	MODIFIER	Pp3c27_453(	Pp3c27_453(	intergenic_re	Pp3c27_453(	NA	NA	n.2628049A>	NA	NA	NA	NA	NA
Chr27	2628070	C	T	Chr27_26280	C_T	SNP	18825.63	[illegible]	7	5	3	downstream	MODIFIER	Pp3c27_457(	Pp3c27_457(	transcript	PAC:3295154	protein_codi	NA	c.*1219G>A	NA	NA	NA	NA	1219
Chr27	2628070	C	T	Chr27_26280	C_T	SNP	18825.63	[illegible]	7	5	3	intergenic_re	MODIFIER	Pp3c27_453(	Pp3c27_453(	intergenic_re	Pp3c27_453(	NA	NA	n.2628070C>	NA	NA	NA	NA	NA
Chr27	2659499	CAT	C	Chr27_26594	CAT_C	INDEL	10754.59	[illegible]	7	5	3	upstream_ge	MODIFIER	Pp3c27_461(	Pp3c27_461(	transcript	PAC:3295199	protein_codi	NA	c.-1815_-181	NA	NA	NA	NA	1370
Chr27	2659499	CAT	C	Chr27_26594	CAT_C	INDEL	10754.59	[illegible]	7	5	3	upstream_ge	MODIFIER	Pp3c27_461(	Pp3c27_461(	transcript	PAC:3295199	protein_codi	NA	c.-1815_-181	NA	NA	NA	NA	1473
Chr27	2659499	CAT	C	Chr27_26594	CAT_C	INDEL	10754.59	[illegible]	7	5	3	upstream_ge	MODIFIER	Pp3c27_461(	Pp3c27_461(	transcript	PAC:3295199	protein_codi	NA	c.-1815_-181	NA	NA	NA	NA	1368
Chr27	2659499	CAT	C	Chr27_26594	CAT_C	INDEL	10754.59	[illegible]	7	5	3	downstream	MODIFIER	Pp3c27_459(	Pp3c27_459(	transcript	PAC:3295189	protein_codi	NA	c.*3619_*36	NA	NA	NA	NA	3297
Chr27	2659499	CAT	C	Chr27_26594	CAT_C	INDEL	10754.59	[illegible]	7	5	3	downstream	MODIFIER	Pp3c27_459(	Pp3c27_459(	transcript	PAC:3295189	protein_codi	NA	c.*3619_*36	NA	NA	NA	NA	3334
Chr27	2659499	CAT	C	Chr27_26594	CAT_C	INDEL	10754.59	[illegible]	7	5	3	downstream	MODIFIER	Pp3c27_459(	Pp3c27_459(	transcript	PAC:3295190	protein_codi	NA	c.*3619_*36	NA	NA	NA	NA	3334
Chr27	2659499	CAT	C	Chr27_26594	CAT_C	INDEL	10754.59	[illegible]	7	5	3	downstream	MODIFIER	Pp3c27_459(	Pp3c27_459(	transcript	PAC:3295190	protein_codi	NA	c.*3619_*36	NA	NA	NA	NA	3334
Chr27	2659499	CAT	C	Chr27_26594	CAT_C	INDEL	10754.59	[illegible]	7	5	3	downstream	MODIFIER	Pp3c27_459(	Pp3c27_459(	transcript	PAC:3295190	protein_codi	NA	c.*3619_*36	NA	NA	NA	NA	3334
Chr27	2659499	CAT	C	Chr27_26594	CAT_C	INDEL	10754.59	[illegible]	7	5	3	downstream	MODIFIER	Pp3c27_459(	Pp3c27_459(	transcript	PAC:3295190	protein_codi	NA	c.*3619_*36	NA	NA	NA	NA	3334
Chr27	2659499	CAT	C	Chr27_26594	CAT_C	INDEL	10754.59	[illegible]	7	5	3	intergenic_re	MODIFIER	Pp3c27_461(	Pp3c27_461(	intergenic_re	Pp3c27_461(	NA	NA	n.2659500_2	NA	NA	NA	NA	NA
Chr27	2698988	G	GAT	Chr27_26989	G_GAT	INDEL	94.37	[illegible]	1	0	0	downstream	MODIFIER	Pp3c27_466(	Pp3c27_466(	transcript	PAC:3295171	protein_codi	NA	c.*2531_*25	NA	NA	NA	NA	2532
Chr27	2698988	G	GAT	Chr27_26989	G_GAT	INDEL	94.37	[illegible]	1	0	0	downstream	MODIFIER	Pp3c27_466(	Pp3c27_466(	transcript	PAC:3295171	protein_codi	NA	c.*2531_*25	NA	NA	NA	NA	2445
Chr27	2698988	G	GAT	Chr27_26989	G_GAT	INDEL	94.37	[illegible]	1	0	0	intergenic_re	MODIFIER	Pp3c27_466(	Pp3c27_466(	intergenic_re	Pp3c27_466(	NA	NA	n.2698988_2	NA	NA	NA	NA	NA
Chr27	2712909	A	AAT	Chr27_27129	A_AAT	INDEL	8945.59	[illegible]	7	5	3	upstream_ge	MODIFIER	Pp3c27_470(	Pp3c27_470(	transcript	PAC:3295161	protein_codi	NA	c.-3724_-372	NA	NA	NA	NA	2645
Chr27	2712909	A	AAT	Chr27_27129	A_AAT	INDEL	8945.59	[illegible]	7	5	3	upstream_ge	MODIFIER	Pp3c27_470(	Pp3c27_470(	transcript	PAC:3295162	protein_codi	NA	c.-3724_-372	NA	NA	NA	NA	2645
Chr27	2712909	A	AAT	Chr27_27129	A_AAT	INDEL	8945.59	[illegible]	7	5	3	upstream_ge	MODIFIER	Pp3c27_470(	Pp3c27_470(	transcript	PAC:3295162	protein_codi	NA	c.-3724_-372	NA	NA	NA	NA	2653
Chr27	2712909	A	AAT	Chr27_27129	A_AAT	INDEL	8945.59	[illegible]	7	5	3	downstream	MODIFIER	Pp3c27_468(	Pp3c27_468(	transcript	PAC:3295198	protein_codi	NA	c.*2625_*26	NA	NA	NA	NA	1407
Chr27	2712909	A	AAT	Chr27_27129	A_AAT	INDEL	8945.59	[illegible]	7	5	3	downstream	MODIFIER	Pp3c27_468(	Pp3c27_468(	transcript	PAC:3295198	protein_codi	NA	c.*2675_*26	NA	NA	NA	NA	1407
Chr27	2712909	A	AAT	Chr27_27129	A_AAT	INDEL	8945.59	[illegible]	7	5	3	downstream	MODIFIER	Pp3c27_468(	Pp3c27_468(	transcript	PAC:3295198	protein_codi	NA	c.*2470_*24	NA	NA	NA	NA	2357
Chr27	2712909	A	AAT	Chr27_27129	A_AAT	INDEL	8945.59	[illegible]	7	5	3	intergenic_re	MODIFIER	Pp3c27_468(	Pp3c27_468(	intergenic_re	Pp3c27_468(	NA	NA	n.2712909_2	NA	NA	NA	NA	NA
Chr27	2729390	A	AT	Chr27_27293	A_AT	INDEL	13340.59	[illegible]	7	5	3	downstream	MODIFIER	Pp3c27_473(	Pp3c27_473(	transcript	PAC:3295199	protein_codi	NA	c.*526_*527	NA	NA	NA	NA	526
Chr27	2729390	A	AT	Chr27_27293	A_AT	INDEL	13340.59	[illegible]	7	5	3	downstream	MODIFIER	Pp3c27_474(	Pp3c27_474(	transcript	PAC:3295141	protein_codi	NA	c.*4125_*41	NA	NA	NA	NA	3787
Chr27	2729390	A	AT	Chr27_27293	A_AT	INDEL	13340.59	[illegible]	7	5	3	downstream	MODIFIER	Pp3c27_473(	Pp3c27_473(	transcript	PAC:3295199	protein_codi	NA	c.*526_*527	NA	NA	NA	NA	174
Chr27	2729390	A	AT	Chr27_27293	A_AT	INDEL	13340.59	[illegible]	7	5	3	downstream	MODIFIER	Pp3c27_474(	Pp3c27_474(	transcript	PAC:3295142	protein_codi	NA	c.*4125_*41	NA	NA	NA	NA	3547
Chr27	2729390	A	AT	Chr27_27293	A_AT	INDEL	13340.59	[illegible]	7	5	3	intron_variar	MODIFIER	Pp3c27_471(	Pp3c27_471(	transcript	PAC:3295222	protein_codi	12-déc	c.1067-79_1(	NA	NA	NA	NA	NA
Chr27	2729390	A	AT	Chr27_27293	A_AT	INDEL	13340.59	[illegible]	7	5	3	intron_variar	MODIFIER	Pp3c27_471(	Pp3c27_471(	transcript	PAC:3295222	protein_codi	12-déc	c.1067-79_1(	NA	NA	NA	NA	NA
Chr27	2729390	A	AT	Chr27_27293	A_AT	INDEL	13340.59	[illegible]	7	5	3	intron_variar	MODIFIER	Pp3c27_471(	Pp3c27_471(	transcript	PAC:3295222	protein_codi	13/13	c.932-79_93.	NA	NA	NA	NA	NA
Chr27	2729390	A	AT	Chr27_27293	A_AT	INDEL	13340.59	[illegible]	7	5	3	intron_variar	MODIFIER	Pp3c27_471(	Pp3c27_471(	transcript	PAC:3295222	protein_codi	12-déc	c.932-79_93.	NA	NA	NA	NA	NA
Chr27	2742603	A	AAT	Chr27_27426	A_AAT	INDEL	99.37	[illegible]	1	0	0	upstream_ge	MODIFIER	Pp3c27_475(	Pp3c27_475(	transcript	PAC:3295147	protein_codi	NA	c.-939_-938i	NA	NA	NA	NA	938
Chr27	2742603	A	AAT	Chr27_27426	A_AAT	INDEL	99.37	[illegible]	1	0	0	upstream_ge	MODIFIER	Pp3c27_476(	Pp3c27_476(	transcript	PAC:3295191	protein_codi	NA	c.-1582_-158	NA	NA	NA	NA	1505
Chr27	2742603	A	AAT	Chr27_27426	A_AAT	INDEL	99.37	[illegible]	1	0	0	upstream_ge	MODIFIER	Pp3c27_476(	Pp3c27_476(	transcript	PAC:3295191	protein_codi	NA	c.-1582_-158	NA	NA	NA	NA	1581
Chr27	2742603	A	AAT	Chr27_27426	A_AAT	INDEL	99.37	[illegible]	1	0	0	intergenic_re	MODIFIER	Pp3c27_474(	Pp3c27_474(	intergenic_re	Pp3c27_474(	NA	NA	n.2742603_2	NA	NA	NA	NA	NA
Chr27	2767046	C	CAT	Chr27_27670	C_CAT	INDEL	3824.24	[illegible]	0	5	0	intergenic_re	MODIFIER	Pp3c27_478(	Pp3c27_478(	intergenic_re	Pp3c27_478(	NA	NA	n.2767046_2	NA	NA	NA	NA	NA
Chr27	2902928	C	A	Chr27_29029	C_A	SNP	15398.63	[illegible]	7	5	3	intergenic_re	MODIFIER	Pp3c27_497(	Pp3c27_497(	intergenic_re	Pp3c27_497(	NA	NA	n.2902928C>	NA	NA	NA	NA	NA
Chr27	2902936	C	CA	Chr27_29029	C_CA	INDEL	15388.59	[illegible]	7	5	3	intergenic_re	MODIFIER	Pp3c27_497(	Pp3c27_497(	intergenic_re	Pp3c27_497(	NA	NA	n.2902936_2	NA	NA	NA	NA	NA
Chr27	2920707	G	GA	Chr27_29207	G_GA	INDEL	15996.59	[illegible]	7	5	3	downstream	MODIFIER	Pp3c27_503(	Pp3c27_503(	transcript	PAC:3295196	protein_codi	NA	c.*1632_*16	NA	NA	NA	NA	1165
Chr27	2920707	G	GA	Chr27_29207	G_GA	INDEL	15996.59	[illegible]	7	5	3	downstream	MODIFIER	Pp3c27_503(	Pp3c27_503(	transcript	PAC:3295196	protein_codi	NA	c.*1632_*16	NA	NA	NA	NA	1165
Chr27	2920707	G	GA	Chr27_29207	G_GA	INDEL	15996.59	[illegible]	7	5	3	intergenic_re	MODIFIER	Pp3c27_497(	Pp3c27_497(	intergenic_re	Pp3c27_497(	NA	NA	n.2920707_2	NA	NA	NA	NA	NA
Chr27	3189033	G	C	Chr27_31890	G_C	SNP	3332.29	[illegible]	0	5	0	downstream	MODIFIER	Pp3c27_559(	Pp3c27_559(	transcript	PAC:3295211	protein_codi	NA	c.*4468G>C	NA	NA	NA	NA	4468
Chr27	3189033	G	C	Chr27_31890	G_C	SNP	3332.29	[illegible]	0	5	0	intergenic_re	MODIFIER	Pp3c27_559(	Pp3c27_559(	intergenic_re	Pp3c27_559(	NA	NA	n.3189033G>	NA	NA	NA	NA	NA
Chr27	3197121	A	G	Chr27_31971	A_G	SNP	18497.63	[illegible]	7	5	3	intergenic_re	MODIFIER	Pp3c27_559(	Pp3c27_559(	intergenic_re	Pp3c27_559(	NA	NA	n.3197121A>	NA	NA	NA	NA	NA
Chr27	3197167	T	TA	Chr27_31971	T_TA	INDEL	12452.59	[illegible]	7	5	3	intergenic_re	MODIFIER	Pp3c27_559(	Pp3c27_559(	intergenic_re	Pp3c27_559(	NA	NA	n.3197167_3	NA	NA	NA	NA	NA
Chr27	3347463	G	A	Chr27_33474	G_A	SNP	15737.63	[illegible]	7	5	3	intergenic_re	MODIFIER	Pp3c27_598(	Pp3c27_598(	intergenic_re	Pp3c27_598(	NA	NA	n.3347463G>	NA	NA	NA	NA	NA
Chr27	3352630	T	C	Chr27_33526	T_C	SNP	6039.29	[illegible]	7	0	3	intergenic_re	MODIFIER	Pp3c27_598(	Pp3c27_598(	intergenic_re	Pp3c27_598(	NA	NA	n.3352630T>	NA	NA	NA	NA	NA
Chr27	3434305	G	A	Chr27_34343	G_A	SNP	17974.63	[illegible]	7	5	3	intergenic_re	MODIFIER	Pp3c27_610(	Pp3c27_610(	intergenic_re	Pp3c27_610(	NA	NA	n.3434305G>	NA	NA	NA	NA	NA
Chr27	3434419	A	T	Chr27_34344	A_T	SNP	22186.63	[illegible]	7	5	3	intergenic_re	MODIFIER	Pp3c27_610(	Pp3c27_610(	intergenic_re	Pp3c27_610(	NA	NA	n.3434419A>	NA	NA	NA	NA	NA
Chr27	3434423	A	T	Chr27_34344	A_T	SNP	22413.63	[illegible]	7	5	3	intergenic_re	MODIFIER	Pp3c27_610(	Pp3c27_610(	intergenic_re	Pp3c27_610(	NA	NA	n.3434423A>	NA	NA	NA	NA	NA
Chr27	3445442	C	T	Chr27_34454	C_T	SNP	16904.63	[illegible]	7	5	3	intergenic_re	MODIFIER	Pp3c27_610(	Pp3c27_610(	intergenic_re	Pp3c27_610(	NA	NA	n.3445442C>	NA	NA	NA	NA	NA
Chr27	3651276	A	AT	Chr27_36512	A_AT	INDEL	9890.59	[illegible]	7	5	3	downstream	MODIFIER	Pp3c27_638(	Pp3c27_638(	transcript	PAC:3295233	protein_codi	NA	c.*3506_*35	NA	NA	NA	NA	3122
Chr27	3651276	A	AT	Chr27_36512	A_AT	INDEL	9890.59	[illegible]	7	5	3	intergenic_re	MODIFIER	Pp3c27_635(	Pp3c27_635(	intergenic_re	Pp3c27_635(	NA	NA	n.3651276_3	NA	NA	NA	NA	NA
Chr27	3651351	G	A	Chr27_36513	G_A	SNP	14939.63	[illegible]	7	5	3	downstream	MODIFIER	Pp3c27_638(	Pp3c27_638(	transcript	PAC:3295233	protein_codi	NA	c.*3432C>T	NA	NA	NA	NA	3048
Chr27	3651351	G	A	Chr27_36513	G_A	SNP	14939.63	[illegible]	7	5	3	intergenic_re	MODIFIER	Pp3c27_635(	Pp3c27_635(	intergenic_re	Pp3c27_635(	NA	NA	n.3651351G>	NA	NA	NA	NA	NA
Chr27	3651352	G	A	Chr27_36513	G_A	SNP	14939.63	[illegible]	7	5	3	downstream	MODIFIER	Pp3c27_638(	Pp3c27_638(	transcript	PAC:3295233	protein_codi	NA	c.*3431C>T	NA	NA	NA	NA	3047
Chr27	3651352	G	A	Chr27_36513	G_A	SNP	14939.63	[illegible]	7	5	3	intergenic_re	MODIFIER	Pp3c27_635(	Pp3c27_635(	intergenic_re	Pp3c27_635(	NA	NA	n.3651352G>	NA	NA	NA	NA	NA
Chr27	3651404	G	T	Chr27_36514	G_T	SNP	15198.63	[illegible]	7	5	3	downstream	MODIFIER	Pp3c27_638(	Pp3c27_638(	transcript	PAC:3295233	protein_codi	NA	c.*3379C>A	NA	NA	NA	NA	2995
Chr27	3651404	G	T	Chr27_36514	G_T	SNP	15198.63	[illegible]	7	5	3	intergenic_re	MODIFIER	Pp3c27_635(	Pp3c27_635(	intergenic_re	Pp3c27_635(	NA	NA	n.3651404G>	NA	NA	NA	NA	NA
Chr27	3805399	G	GTA	Chr27_38053	G_GTA	INDEL	2795.93	[illegible]	7	0	0	upstream_ge	MODIFIER	Pp3c27_660(	Pp3c27_660(	transcript	PAC:3295168	protein_codi	NA	c.-4768_-476	NA	NA	NA	NA	4768
Chr27	3805399	G	GTA	Chr27_38053	G_GTA	INDEL	2795.93	[illegible]	7	0	0	upstream_ge	MODIFIER	Pp3c27_660(	Pp3c27_660(	transcript	PAC:3295168	protein_codi	NA	c.-4768_-476	NA	NA	NA	NA	4601
Chr27	3805399	G	GTA	Chr27_38053	G_GTA	INDEL	2795.93	[illegible]	7	0	0	downstream	MODIFIER	Pp3c27_661(	Pp3c27_661(	transcript	PAC:3295227	protein_codi	NA	c.*502_*503	NA	NA	NA	NA	23
Chr27	3805399	G	GTA	Chr27_38053	G_GTA	INDEL	2795.93	[illegible]	7	0	0	downstream	MODIFIER	Pp3c27_661(	Pp3c27_661(	transcript	PAC:3295228	protein_codi	NA	c.*502_*503	NA	NA	NA	NA	42
Chr27	3805399	G	GTA	Chr27_38053	G_GTA	INDEL	2795.93	[illegible]	7	0	0	downstream	MODIFIER	Pp3c27_661(	Pp3c27_661(	transcript	PAC:3295228	protein_codi	NA	c.*502_*503	NA	NA	NA	NA	46
Chr27	3805399	G	GTA	Chr27_38053	G_GTA	INDEL	2795.93	[illegible]	7	0	0	intergenic_re	MODIFIER	Pp3c27_661(	Pp3c27_661(	intergenic_re	Pp3c27_661(	NA	NA	n.3805399_3	NA	NA	NA	NA	NA
Chr27	3822779	CT	C	Chr27_38227	CT_C	INDEL	13645.59	[illegible]	7	5	3	intergenic_re	MODIFIER	Pp3c27_661(	Pp3c27_661(	intergenic_re	Pp3c27_661(	NA	NA	n.3822780de	NA	NA	NA	NA	NA
Chr27	3822797	TC	T	Chr27_38227	TC_T	INDEL	14974.59	[illegible]	7	5	3	intergenic_re	MODIFIER	Pp3c27_661(	Pp3c27_661(	intergenic_re	Pp3c27_661(	NA	NA	n.3822798de	NA	NA	NA	NA	NA
Chr27	3822801	A	T	Chr27_38228	A_T	SNP	14770.63	[illegible]	7	5	3	intergenic_re	MODIFIER	Pp3c27_661(	Pp3c27_661(	intergenic_re	Pp3c27_661(	NA	NA	n.3822801A>	NA	NA	NA	NA	NA
Chr27	3822824	A	T	Chr27_38228	A_T	SNP	13748.63	[illegible]	7	5	3	intergenic_re	MODIFIER	Pp3c27_661(	Pp3c27_661(	intergenic_re	Pp3c27_661(	NA	NA	n.3822824A>	NA	NA	NA	NA	NA
Chr27	3822849	TA	T	Chr27_38228	TA_T	INDEL	14313.59	[illegible]	7	5	3	intergenic_re	MODIFIER	Pp3c27_661(	Pp3c27_661(	intergenic_re	Pp3c27_661(	NA	NA	n.3822850de	NA	NA	NA	NA	NA
Chr27	3822859	TC	T	Chr27_38228	TC_T	INDEL	15160.59	[illegible]	7	5	3	intergenic_re	MODIFIER	Pp3c27_661(	Pp3c27_661(	intergenic_re	Pp3c27_661(	NA	NA	n.3822860de	NA	NA	NA	NA	NA
Chr27	3825673	G	GT	Chr27_38256	G_GT	INDEL	20329.59	[illegible]	7	5	3	intergenic_re	MODIFIER	Pp3c27_661(	Pp3c27_661(	intergenic_re	Pp3c27_661(	NA	NA	n.3825673_3	NA	NA	NA	NA	NA
Chr27	3825681	G	GA	Chr27_38256	G_GA	INDEL	20780.59	[illegible]	7	5	3	intergenic_re	MODIFIER	Pp3c27_661(	Pp3c27_661(	intergenic_re	Pp3c27_661(	NA	NA	n.3825681_3	NA	NA	NA	NA	NA
Chr27	3825685	G	GA	Chr27_38256	G_GA	INDEL	20196.59	[illegible]	7	5	3	intergenic_re	MODIFIER	Pp3c27_661(	Pp3c27_661(	intergenic_re	Pp3c27_661(	NA	NA	n.3825685_3	NA	NA	NA	NA	NA
Chr27	3922525	A	AAT	Chr27_39225	A_AAT	INDEL	2618.24	[illegible]	0	5	0	intergenic_re	MODIFIER	Pp3c27_671(	Pp3c27_671(	intergenic_re	Pp3c27_671(	NA	NA	n.3922525_3	NA	NA	NA	NA	NA
Chr27	4184756	C	T	Chr27_41847	C_T	SNP	5820.29	[illegible]	0	5	0	intergenic_re	MODIFIER	Pp3c27_702(	Pp3c27_702(	intergenic_re	Pp3c27_702(	NA	NA	n.4184756C>	NA	NA	NA	NA	NA
Chr27	4197375	G	GA	Chr27_41973	G_GA	INDEL	14811.59	[illegible]	7	5	3	intergenic_re	MODIFIER	Pp3c27_702(	Pp3c27_702(	intergenic_re	Pp3c27_702(	NA	NA	n.4197375_4	NA	NA	NA	NA	NA
Chr27	4303749	C	A	Chr27_43037	C_A	SNP	22782.63	[illegible]	7	5	3	intergenic_re	MODIFIER	Pp3c27_717(	Pp3c27_717(	intergenic_re	Pp3c27_717(	NA	NA	n.4303749C>	NA	NA	NA	NA	NA
Chr27	4303757	T	TA	Chr27_43037	T_TA	INDEL	22756.59	[illegible]	7	5	3	intergenic_re	MODIFIER	Pp3c27_717(	Pp3c27_717(	intergenic_re	Pp3c27_717(	NA	NA	n.4303757_4	NA	NA	NA	NA	NA
Chr27	4442090	G	T	Chr27_44420	G_T	SNP	15816.63	[illegible]	7	5	3	intergenic_re	MODIFIER	Pp3c27_738(	Pp3c27_738(	intergenic_re	Pp3c27_738(	NA	NA	n.4442090G>	NA	NA	NA	NA	NA
Chr27	4608859	A	AAT	Chr27_46088	A_AAT	INDEL	2874.93	[illegible]	7	0	0	upstream_ge	MODIFIER	Pp3c27_771(	Pp3c27_771(	transcript	PAC:3295183	protein_codi	NA	c.-3735_-373	NA	NA	NA	NA	3517
Chr27	4608859	A	AAT	Chr27_46088	A_AAT	INDEL	2874.93	[illegible]	7	0	0	upstream_ge	MODIFIER	Pp3c27_771(	Pp3c27_771(	transcript	PAC:3295183	protein_codi	NA	c.-3735_-373	NA	NA	NA	NA	3553
Chr27	4608859	A	AAT	Chr27_46088	A_AAT	INDEL	2874.93	[illegible]	7	0	0	downstream	MODIFIER	Pp3c27_770(	Pp3c27_770(	transcript	PAC:3295213	protein_codi	NA	c.*2417_*24	NA	NA	NA	NA	1907
Chr27	4608859	A	AAT	Chr27_46088	A_AAT	INDEL	2874.93	[illegible]	7	0	0	downstream	MODIFIER	Pp3c27_770(	Pp3c27_770(	transcript	PAC:3295213	protein_codi	NA	c.*2417_*24	NA	NA	NA	NA	1899
Chr27	4608859	A	AAT	Chr27_46088	A_AAT	INDEL	2874.93	[illegible]	7	0	0	downstream	MODIFIER	Pp3c27_770(	Pp3c27_770(	transcript	PAC:3295213	protein_codi	NA	c.*2417_*24	NA	NA	NA	NA	1907
Chr27	4608859	A	AAT	Chr27_46088	A_AAT	INDEL	2874.93	[illegible]	7	0	0	downstream	MODIFIER	Pp3c27_770(	Pp3c27_770(	transcript	PAC:3295213	protein_codi	NA	c.*2417_*24	NA	NA	NA	NA	1897
Chr27	4608859	A	AAT	Chr27_46088	A_AAT	INDEL	2874.93	[illegible]	7	0	0	downstream	MODIFIER	Pp3c27_770(	Pp3c27_770(	transcript	PAC:3295213	protein_codi	NA	c.*2417_*24	NA	NA	NA	NA	1602
Chr27	4608859	A	AAT	Chr27_46088	A_AAT	INDEL	2874.93	[illegible]	7	0	0	downstream	MODIFIER	Pp3c27_770(	Pp3c27_770(	transcript	PAC:3295213	protein_codi	NA	c.*2417_*24	NA	NA	NA	NA	1898
Chr27	4608859	A	AAT	Chr27_46088	A_AAT	INDEL	2874.93	[illegible]	7	0	0	intergenic_re	MODIFIER	Pp3c27_770(	Pp3c27_770(	intergenic_re	Pp3c27_770(	NA	NA	n.4608859_4	NA	NA	NA	NA	NA
Chr27	4647279	A	AAT	Chr27_46472	A_AAT	INDEL	3691.24	[illegible]	0	5	0	upstream_ge	MODIFIER	Pp3c27_782(	Pp3c27_782(	transcript	PAC:3295209	protein_codi	NA	c.-750_-749i	NA	NA	NA	NA	580
Chr27	4647279	A	AAT	Chr27_46472	A_AAT	INDEL	3691.24	[illegible]	0	5	0	upstream_ge	MODIFIER	Pp3c27_783(	Pp3c27_783(	transcript	PAC:3295228	protein_codi	NA	c.-3094_-309	NA	NA	NA	NA	1660
Chr27	4647279	A	AAT	Chr27_46472	A_AAT	INDEL	3691.24	[illegible]	0	5	0	upstream_ge	MODIFIER	Pp3c27_782(	Pp3c27_782(	transcript	PAC:3295209	protein_codi	NA	c.-750_-749i	NA	NA	NA	NA	559
Chr27	4647279	A	AAT	Chr27_46472	A_AAT	INDEL	3691.24	[illegible]	0	5	0	upstream_ge	MODIFIER	Pp3c27_783(	Pp3c27_783(	transcript	PAC:3295228	protein_codi	NA	c.-3094_-309	NA	NA	NA	NA	1660
Chr27	4647279	A	AAT	Chr27_46472	A_AAT	INDEL	3691.24	[illegible]	0	5	0	upstream_ge	MODIFIER	Pp3c27_783(	Pp3c27_783(	transcript	PAC:3295228	protein_codi	NA	c.-3094_-309	NA	NA	NA	NA	1744
Chr27	4647279	A	AAT	Chr27_46472	A_AAT	INDEL	3691.24	[illegible]	0	5	0	downstream	MODIFIER	Pp3c27_781(	Pp3c27_781(	transcript	PAC:3295165	protein_codi	NA	c.*4013_*40	NA	NA	NA	NA	3239
Chr27	4647279	A	AAT	Chr27_46472	A_AAT	INDEL	3691.24	[illegible]	0	5	0	downstream	MODIFIER	Pp3c27_781(	Pp3c27_781(	transcript	PAC:3295165	protein_codi	NA	c.*4013_*40	NA	NA	NA	NA	3951
Chr27	4647279	A	AAT	Chr27_46472	A_AAT	INDEL	3691.24	[illegible]	0	5	0	intergenic_re	MODIFIER	Pp3c27_782(	Pp3c27_782(	intergenic_re	Pp3c27_782(	NA	NA	n.4647279_4	NA	NA	NA	NA	NA
Chr27	4836074	G	T	Chr27_48360	G_T	SNP	10380.29	[illegible]	7	0	3	intergenic_re	MODIFIER	Pp3c27_791(	Pp3c27_791(	intergenic_re	Pp3c27_791(	NA	NA	n.4836074G>	NA	NA	NA	NA	NA
Chr27	4899384	T	TTA	Chr27_48993	T_TTA	INDEL	3358.24	[illegible]	0	5	0	downstream	MODIFIER	Pp3c27_805(	Pp3c27_805(	transcript	PAC:3295233	protein_codi	NA	c.*2086_*20	NA	NA	NA	NA	1551
Chr27	4899384	T	TTA	Chr27_48993	T_TTA	INDEL	3358.24	[illegible]	0	5	0	downstream	MODIFIER	Pp3c27_805(	Pp3c27_805(	transcript	PAC:3295233	protein_codi	NA	c.*2086_*20	NA	NA	NA	NA	1545
Chr27	4899384	T	TTA	Chr27_48993	T_TTA	INDEL	3358.24	[illegible]	0	5	0	downstream	MODIFIER	Pp3c27_805(	Pp3c27_805(	transcript	PAC:3295233	protein_codi	NA	c.*2086_*20	NA	NA	NA	NA	1551
Chr27	4899384	T	TTA	Chr27_48993	T_TTA	INDEL	3358.24	[illegible]	0	5	0	intergenic_re	MODIFIER	Pp3c27_791(	Pp3c27_791(	intergenic_re	Pp3c27_791(	NA	NA	n.4899384_4	NA	NA	NA	NA	NA

Chr27	4975439	T	C	Chr27_49754T_C	SNP	14951.63	26	22	4	16	8	19	32	15	19	20	11	17	9	15	26	22	4	16	8	19	32	15	19	20	11	17	9	15	1	1	1	1	1	1	1	1	1	1	1	1	1	1	0	15	15	0	7	5	3	5_prime_UTI	MODIFIER	Pp3c27_824	Pp3c27_824	transcript	PAC:3295142	protein_codi		01-janv	c.-496T>C	NA	NA	NA	NA	496
Chr27	5115871	G	A	Chr27_51158G_A	SNP	5299.98	0	0	0	0	0	0	0	0	23	27	6	23	14	19	30	12	14	21	15	21	28	24	23	27	6	23	14	19	0	0	0	0	0	0	0	0	1	1	1	1	1	1	0	7	7	0	7	0	0	intergenic_re	MODIFIER	Pp3c27_841	Pp3c27_841	intergenic_re	Pp3c27_841	NA	NA		n.5115871G>	NA	NA	NA	NA	NA
Chr27	5293799	G	C	Chr27_52937G_C	SNP	19404.63	23	19	12	27	22	26	26	16	28	26	17	15	10	10	23	19	12	27	22	26	26	16	28	26	17	15	10	10	1	1	1	1	1	1	1	1	1	1	1	1	1	1	0	15	15	0	7	5	3	5_prime_UTI	MODIFIER	Pp3c27_883	Pp3c27_883	transcript	PAC:3295193	protein_codi		01-janv	c.-44G>C	NA	NA	NA	NA	44
Chr27	5293799	G	C	Chr27_52937G_C	SNP	19404.63	23	19	12	27	22	26	26	16	28	26	17	15	10	10	23	19	12	27	22	26	26	16	28	26	17	15	10	10	1	1	1	1	1	1	1	1	1	1	1	1	1	1	0	15	15	0	7	5	3	downstream	MODIFIER	Pp3c27_882	Pp3c27_882	transcript	PAC:3295144	protein_codi	NA		c.*2443G>C	NA	NA	NA	NA	2269
Chr27	5293799	G	C	Chr27_52937G_C	SNP	19404.63	23	19	12	27	22	26	26	16	28	26	17	15	10	10	23	19	12	27	22	26	26	16	28	26	17	15	10	10	1	1	1	1	1	1	1	1	1	1	1	1	1	1	0	15	15	0	7	5	3	downstream	MODIFIER	Pp3c27_881	Pp3c27_881	transcript	PAC:3295225	protein_codi	NA		c.*2443G>C	NA	NA	NA	NA	2443
Chr27	5293799	G	C	Chr27_52937G_C	SNP	19404.63	23	19	12	27	22	26	26	16	28	26	17	15	10	10	23	19	12	27	22	26	26	16	28	26	17	15	10	10	1	1	1	1	1	1	1	1	1	1	1	1	1	1	0	15	15	0	7	5	3	downstream	MODIFIER	Pp3c27_884	Pp3c27_884	transcript	PAC:3295232	protein_codi	NA		c.*721C>G	NA	NA	NA	NA	721
Chr27	5293799	G	C	Chr27_52937G_C	SNP	19404.63	23	19	12	27	22	26	26	16	28	26	17	15	10	10	23	19	12	27	22	26	26	16	28	26	17	15	10	10	1	1	1	1	1	1	1	1	1	1	1	1	1	1	0	15	15	0	7	5	3	downstream	MODIFIER	Pp3c27_881	Pp3c27_881	transcript	PAC:3295225	protein_codi	NA		c.*4609G>C	NA	NA	NA	NA	4609
