# Supplementary material for: Genome-wide specificity of plant genome editing by both CRISPR–Cas9 and TALEN
Source: Sci Rep. 2022 Jun 4;12:9330. doi: 10.1038/s41598-022-13034-2 (PMC9167288; doi:10.1038/s41598-022-13034-2)
Supplement: Supplementary file 2 — Supplementary Information 2. [file 41598_2022_13034_MOESM2_ESM.pdf]

CHROM	POS	REF	ALT	ID	CHANGE	CHANGE_TY	QUAL	WTB_AD
Chr01	6348013	G	A	Chr01_63480	G_A	SNP	724.41	0
Chr01	9544453	C	T	Chr01_95444	C_T	SNP	287.41	0
Chr01	13711188	T	TTA	Chr01_13711	T_TTA	INDEL	324.37	0
Chr01	13711188	T	TTA	Chr01_13711	T_TTA	INDEL	324.37	0
Chr01	13711188	T	TTA	Chr01_13711	T_TTA	INDEL	324.37	0
Chr01	16271546	AATAT	A	Chr01_16271	AATAT_A	INDEL	6054.37	0
Chr01	16331294	CATATAT	C	Chr01_16331	CATATAT_C	INDEL	168.37	0
Chr01	16331294	CATATAT	C	Chr01_16331	CATATAT_C	INDEL	168.37	0
Chr01	16331294	CATATAT	C	Chr01_16331	CATATAT_C	INDEL	168.37	0
Chr01	16331294	CATATAT	C	Chr01_16331	CATATAT_C	INDEL	168.37	0
Chr01	16331294	CATATAT	C	Chr01_16331	CATATAT_C	INDEL	168.37	0
Chr01	16331294	CATATAT	C	Chr01_16331	CATATAT_C	INDEL	168.37	0
Chr01	16331294	CATATAT	C	Chr01_16331	CATATAT_C	INDEL	168.37	0
Chr01	17645428	C	CTA	Chr01_17645	C_CTA	INDEL	156.37	0
Chr01	17645428	C	CTA	Chr01_17645	C_CTA	INDEL	156.37	0
Chr01	17645428	C	CTA	Chr01_17645	C_CTA	INDEL	156.37	0
Chr01	17645428	C	CTA	Chr01_17645	C_CTA	INDEL	156.37	0
Chr01	17645428	C	CTA	Chr01_17645	C_CTA	INDEL	156.37	0
Chr01	17645428	C	CTA	Chr01_17645	C_CTA	INDEL	156.37	0
Chr01	19176948	G	A	Chr01_19176	G_A	SNP	700.41	0
Chr01	20185266	C	CAT	Chr01_20185	C_CAT	INDEL	115.37	0
Chr01	20185266	C	CAT	Chr01_20185	C_CAT	INDEL	115.37	0
Chr01	20185266	C	CAT	Chr01_20185	C_CAT	INDEL	115.37	0
Chr01	22972213	C	CTA	Chr01_22972	C_CTA	INDEL	374.37	1
Chr01	22972213	C	CTA	Chr01_22972	C_CTA	INDEL	374.37	1
Chr01	23859125	T	TTA	Chr01_23859	T_TTA	INDEL	48.37	1
Chr01	23859125	T	TTA	Chr01_23859	T_TTA	INDEL	48.37	1
Chr01	23859125	T	TTA	Chr01_23859	T_TTA	INDEL	48.37	1
Chr01	23859125	T	TTA	Chr01_23859	T_TTA	INDEL	48.37	1
Chr01	23859125	T	TTA	Chr01_23859	T_TTA	INDEL	48.37	1
Chr01	23859125	T	TTA	Chr01_23859	T_TTA	INDEL	48.37	1
Chr01	23859125	T	TTA	Chr01_23859	T_TTA	INDEL	48.37	1
Chr01	23859125	T	TTA	Chr01_23859	T_TTA	INDEL	48.37	1
Chr01	23859125	T	TTA	Chr01_23859	T_TTA	INDEL	48.37	1
Chr01	23859125	T	TTA	Chr01_23859	T_TTA	INDEL	48.37	1
Chr01	23859125	T	TTA	Chr01_23859	T_TTA	INDEL	48.37	1
Chr01	23859125	T	TTA	Chr01_23859	T_TTA	INDEL	48.37	1
Chr01	23859125	T	TTA	Chr01_23859	T_TTA	INDEL	48.37	1
Chr01	23859125	T	TTA	Chr01_23859	T_TTA	INDEL	48.37	1
Chr01	23958265	C	T	Chr01_23958	C_T	SNP	678.41	0
Chr01	24253532	AAT	A	Chr01_24253	AAT_A	INDEL	385.37	0
Chr01	25467269	G	A	Chr01_25467	G_A	SNP	271.41	0
Chr01	26753519	C	A	Chr01_26753	C_A	SNP	211.89	0
Chr01	26753519	C	A	Chr01_26753	C_A	SNP	211.89	0
Chr01	26753519	C	A	Chr01_26753	C_A	SNP	211.89	0
Chr01	26753519	C	A	Chr01_26753	C_A	SNP	211.89	0
Chr01	29237036	G	A	Chr01_29237	G_A	SNP	546.41	0
Chr02	121084	A	AAT	Chr02_12108	A_AAT	INDEL	118.37	0
Chr02	121084	A	AAT	Chr02_12108	A_AAT	INDEL	118.37	0
Chr02	121084	A	AAT	Chr02_12108	A_AAT	INDEL	118.37	0
Chr02	121084	A	AAT	Chr02_12108	A_AAT	INDEL	118.37	0
Chr02	121084	A	AAT	Chr02_12108	A_AAT	INDEL	118.37	0
Chr02	121084	A	AAT	Chr02_12108	A_AAT	INDEL	118.37	0
Chr02	121084	A	AAT	Chr02_12108	A_AAT	INDEL	118.37	0
Chr02	121084	A	AAT	Chr02_12108	A_AAT	INDEL	118.37	0
Chr02	6914691	G	T	Chr02_69146	G_T	SNP	364.41	0
Chr02	6914691	G	T	Chr02_69146	G_T	SNP	364.41	0
Chr02	7270496	A	AAT	Chr02_72704	A_AAT	INDEL	446.37	0
Chr02	7270496	A	AAT	Chr02_72704	A_AAT	INDEL	446.37	0
Chr02	7270496	A	AAT	Chr02_72704	A_AAT	INDEL	446.37	0
Chr02	7270496	A	AAT	Chr02_72704	A_AAT	INDEL	446.37	0
Chr02	7270496	A	AAT	Chr02_72704	A_AAT	INDEL	446.37	0
Chr02	7270496	A	AAT	Chr02_72704	A_AAT	INDEL	446.37	0
Chr02	7270496	A	AAT	Chr02_72704	A_AAT	INDEL	446.37	0
Chr02	7270496	A	AAT	Chr02_72704	A_AAT	INDEL	446.37	0
Chr02	7345294	AAT	AATATATAT	Chr02_73452	AAT_AATAT	INDEL	4570.92	0
Chr02	8215396	T	TTA	Chr02_82153	T_TTA	INDEL	238.37	1
Chr02	8215396	T	TTA	Chr02_82153	T_TTA	INDEL	238.37	1
Chr02	8215396	T	TTA	Chr02_82153	T_TTA	INDEL	238.37	1
Chr02	8215396	T	TTA	Chr02_82153	T_TTA	INDEL	238.37	1
Chr02	8215396	T	TTA	Chr02_82153	T_TTA	INDEL	238.37	1
Chr02	8215396	T	TTA	Chr02_82153	T_TTA	INDEL	238.37	1
Chr02	8215396	T	TTA	Chr02_82153	T_TTA	INDEL	238.37	1
Chr02	8215396	T	TTA	Chr02_82153	T_TTA	INDEL	238.37	1
Chr02	8215396	T	TTA	Chr02_82153	T_TTA	INDEL	238.37	1
Chr02	8215396	T	TTA	Chr02_82153	T_TTA	INDEL	238.37	1
Chr02	8877478	A	G	Chr02_88774	A_G	SNP	271.41	0
Chr02	14820415	T	TTC	Chr02_14820	T_TTC	INDEL	383.37	0
Chr02	17348526	C	G	Chr02_17348	C_G	SNP	673.41	0
Chr02	18321014	A	G	Chr02_18321	A_G	SNP	636.41	0
Chr02	18321014	A	G	Chr02_18321	A_G	SNP	636.41	0
Chr02	18545650	C	T	Chr02_18545	C_T	SNP	356.41	0
Chr02	19513452	A	AAT	Chr02_19513	A_AAT	INDEL	332.37	0
Chr02	19513452	A	AAT	Chr02_19513	A_AAT	INDEL	332.37	0
Chr02	19513452	A	AAT	Chr02_19513	A_AAT	INDEL	332.37	0
Chr02	19513452	A	AAT	Chr02_19513	A_AAT	INDEL	332.37	0
Chr02	19513452	A	AAT	Chr02_19513	A_AAT	INDEL	332.37	0
Chr02	19513452	A	AAT	Chr02_19513	A_AAT	INDEL	332.37	0
Chr02	19513452	A	AAT	Chr02_19513	A_AAT	INDEL	332.37	0
Chr02	19513452	A	AAT	Chr02_19513	A_AAT	INDEL	332.37	0
Chr02	19513452	A	AAT	Chr02_19513	A_AAT	INDEL	332.37	0
Chr02	19769683	A	AAT	Chr02_19769	A_AAT	INDEL	259.37	1
Chr02	19769683	A	AAT	Chr02_19769	A_AAT	INDEL	259.37	1
Chr02	19769683	A	AAT	Chr02_19769	A_AAT	INDEL	259.37	1
Chr02	19769683	A	AAT	Chr02_19769	A_AAT	INDEL	259.37	1
Chr02	19769683	A	AAT	Chr02_19769	A_AAT	INDEL	259.37	1
Chr02	20244584	G	GTA	Chr02_20244	G_GTA	INDEL	253.37	1
Chr02	20244584	G	GTA	Chr02_20244	G_GTA	INDEL	253.37	1
Chr02	20244584	G	GTA	Chr02_20244	G_GTA	INDEL	253.37	1
Chr02	23820260	T	TTA	Chr02_23820	T_TTA	INDEL	312.37	0
Chr03	600754	T	TTA	Chr03_60075	T_TTA	INDEL	277.37	1
Chr03	600754	T	TTA	Chr03_60075	T_TTA	INDEL	277.37	1
Chr03	3170711	G	A	Chr03_31707	G_A	SNP	396.41	0
Chr03	3376107	A	G	Chr03_33761	A_G	SNP	393.41	0
Chr03	4414859	C	A	Chr03_44148	C_A	SNP	224.41	0
Chr03	4719499	T	TTA	Chr03_47194	T_TTA	INDEL	654.37	0
Chr03	4719499	T	TTA	Chr03_47194	T_TTA	INDEL	654.37	0
Chr03	4719499	T	TTA	Chr03_47194	T_TTA	INDEL	654.37	0
Chr03	4719499	T	TTA	Chr03_47194	T_TTA	INDEL	654.37	0
Chr03	4750087	CATATAT	C	Chr03_47500	CATATAT_C	INDEL	3434.93	0
Chr03	4750087	CATATAT	C	Chr03_47500	CATATAT_C	INDEL	3434.93	0
Chr03	4750087	CATATAT	C	Chr03_47500	CATATAT_C	INDEL	3434.93	0
Chr03	4750087	CATATAT	C	Chr03_47500	CATATAT_C	INDEL	3434.93	0
Chr03	4750087	CATATAT	C	Chr03_47500	CATATAT_C	INDEL	3434.93	0
Chr03	4750087	CATATAT	C	Chr03_47500	CATATAT_C	INDEL	3434.93	0
Chr03	4750087	CATATAT	C	Chr03_47500	CATATAT_C	INDEL	3434.93	0
Chr03	4750087	CATATAT	C	Chr03_47500	CATATAT_C	INDEL	3434.93	0
Chr03	4750087	CATATAT	C	Chr03_47500	CATATAT_C	INDEL	3434.93	0
Chr03	6000925	C	CAT	Chr03_60009	C_CAT	INDEL	817.37	0
Chr03	6916014	C	T	Chr03_69160	C_T	SNP	157.41	0
Chr03	6916014	C	T	Chr03_69160	C_T	SNP	157.41	0
Chr03	6916014	C	T	Chr03_69160	C_T	SNP	157.41	0
Chr03	6916014	C	T	Chr03_69160	C_T	SNP	157.41	0
Chr03	10871116	T	TTA	Chr03_10871	T_TTA	INDEL	97.37	0
Chr03	10871116	T	TTA	Chr03_10871	T_TTA	INDEL	97.37	0
Chr03	10871116	T	TTA	Chr03_10871	T_TTA	INDEL	97.37	0
Chr03	11711783	TTA	T	Chr03_11711	TTA_T	INDEL	449.37	0
Chr03	11711783	TTA	T	Chr03_11711	TTA_T	INDEL	449.37	0
Chr03	12394332	C	CAT	Chr03_12394	C_CAT	INDEL	304.37	0
Chr03	12394332	C	CAT	Chr03_12394	C_CAT	INDEL	304.37	0
Chr03	12394332	C	CAT	Chr03_12394	C_CAT	INDEL	304.37	0
Chr03	13918915	C	CTA	Chr03_13918	C_CTA	INDEL	217.37	0
Chr03	13918915	C	CTA	Chr03_13918	C_CTA	INDEL	217.37	0
Chr03	13918915	C	CTA	Chr03_13918	C_CTA	INDEL	217.37	0
Chr03	13918915	C	CTA	Chr03_13918	C_CTA	INDEL	217.37	0
Chr03	13918915	C	CTA	Chr03_13918	C_CTA	INDEL	217.37	0
Chr03	13918915	C	CTA	Chr03_13918	C_CTA	INDEL	217.37	0
Chr03	13918915	C	CTA	Chr03_13918	C_CTA	INDEL	217.37	0
Chr03	15558655	C	A	Chr03_15558	C_A	SNP	790.41	8
Chr03	16923154	GTA	G	Chr03_16923	GTA_G	INDEL	1215.7	0
Chr03	16923154	GTA	G	Chr03_16923	GTA_G	INDEL	1215.7	0
Chr03	16923154	GTA	G	Chr03_16923	GTA_G	INDEL	1215.7	0
Chr03	18329573	A	G	Chr03_18329	A_G	SNP	322.41	0
Chr03	18329573	A	G	Chr03_18329	A_G	SNP	322.41	0
Chr03	19118263	C	CTA	Chr03_19118	C_CTA	INDEL	146.37	1
Chr03	19609813	C	T	Chr03_19609	C_T	SNP	741.41	0
Chr03	19660713	T	TTA	Chr03_19660	T_TTA	INDEL	230.02	0
Chr03	20748752	G	GTA	Chr03_20748	G_GTA	INDEL	276.37	0
Chr03	23982485	T	TTA	Chr03_23982	T_TTA	INDEL	482.37	0
Chr04	389031	G	A	Chr04_38903	G_A	SNP	277.41	0
Chr04	897810	A	AAT	Chr04_89781	A_AAT	INDEL	130.37	2
Chr04	897810	A	AAT	Chr04_89781	A_AAT	INDEL	130.37	2
Chr04	897810	A	AAT	Chr04_89781	A_AAT	INDEL	130.37	2
Chr04	897810	A	AAT	Chr04_89781	A_AAT	INDEL	130.37	2
Chr04	897810	A	AAT	Chr04_89781	A_AAT	INDEL	130.37	2
Chr04	897810	A	AAT	Chr04_89781	A_AAT	INDEL	130.37	2
Chr04	897810	A	AAT	Chr04_89781	A_AAT	INDEL	130.37	2
Chr04	897810	A	AAT	Chr04_89781	A_AAT	INDEL	130.37	2
Chr04	897810	A	AAT	Chr04_89781	A_AAT	INDEL	130.37	2
Chr04	897810	A	AAT	Chr04_89781	A_AAT	INDEL	130.37	2
Chr04	897810	A	AAT	Chr04_89781	A_AAT	INDEL	130.37	2
Chr04	897810	A	AAT	Chr04_89781	A_AAT	INDEL	130.37	2
Chr04	897810	A	AAT	Chr04_89781	A_AAT	INDEL	130.37	2
Chr04	897810	A	AAT	Chr04_89781	A_AAT	INDEL	130.37	2
Chr04	897810	A	AAT	Chr04_89781	A_AAT	INDEL	130.37	2
Chr04	1137679	A	AAT	Chr04_11376	A_AAT	INDEL	297.37	0
Chr04	3353766	TTA	T	Chr04_33537	TTA_T	INDEL	611.37	1
Chr04	3353766	TTA	T	Chr04_33537	TTA_T	INDEL	611.37	1
Chr04	3353766	TTA	T	Chr04_33537	TTA_T	INDEL	611.37	1
Chr04	3439043	A	AAT	Chr04_34390	A_AAT	INDEL	352.37	0
Chr04	3439043	A	AAT	Chr04_34390	A_AAT	INDEL	352.37	0
Chr04	3439043	A	AAT	Chr04_34390	A_AAT	INDEL	352.37	0
Chr04	3439043	A	AAT	Chr04_34390	A_AAT	INDEL	352.37	0
Chr04	3439043	A	AAT	Chr04_34390	A_AAT	INDEL	352.37	0
Chr04	3439043	A	AAT	Chr04_34390	A_AAT	INDEL	352.37	0
Chr04	3439043	A	AAT	Chr04_34390	A_AAT	INDEL	352.37	0
Chr04	3439043	A	AAT	Chr04_34390	A_AAT	INDEL	352.37	0
Chr04	3439043	A	AAT	Chr04_34390	A_AAT	INDEL	352.37	0
Chr04	3439043	A	AAT	Chr04_34390	A_AAT	INDEL	352.37	0
Chr04	3439043	A	AAT	Chr04_34390	A_AAT	INDEL	352.37	0
Chr04	3439043	A	AAT	Chr04_34390	A_AAT	INDEL	352.37	0
Chr04	3439043	A	AAT	Chr04_34390	A_AAT	INDEL	352.37	0
Chr04	3439043	A	AAT	Chr04_34390	A_AAT	INDEL	352.37	0
Chr04	5609076	A	AATAT	Chr04_56090	A_AATAT	INDEL	538.37	0
Chr04	5609076	A	AATAT	Chr04_56090	A_AATAT	INDEL	538.37	0
Chr04	5609076	A	AATAT	Chr04_56090	A_AATAT	INDEL	538.37	0
Chr04	5609076	A	AATAT	Chr04_56090	A_AATAT	INDEL	538.37	0
Chr04	5609076	A	AATAT	Chr04_56090	A_AATAT	INDEL	538.37	0
Chr04	5609076	A	AATAT	Chr04_56090	A_AATAT	INDEL	538.37	0
Chr04	5724421	G	T	Chr04_57244	G_T	SNP	802.41	0
Chr04	5878614	G	T	Chr04_58786	G_T	SNP	539.41	0
Chr04	5878614	G	T	Chr04_58786	G_T	SNP	539.41	0
Chr04	5878614	G	T	Chr04_58786	G_T	SNP	539.41	0
Chr04	5878614	G	T	Chr04_58786	G_T	SNP	539.41	0
Chr04	5878614	G	T	Chr04_58786	G_T	SNP	539.41	0
Chr04	5878614	G	T	Chr04_58786	G_T	SNP	539.41	0
Chr04	5878614	G	T	Chr04_58786	G_T	SNP	539.41	0
Chr04	5878614	G	T	Chr04_58786	G_T	SNP	539.41	0
Chr04	5878614	G	T	Chr04_58786	G_T	SNP	539.41	0
Chr04	5878614	G	T	Chr04_58786	G_T	SNP	539.41	0
Chr04	5878614	G	T	Chr04_58786	G_T	SNP	539.41	0

CHROM	POS	TAL#1_AD	TAL#2_AD	WTA_AD	CRIS1#1_AD	CRIS1#2_AD	CRIS2#1_AD	CRIS2#2_AD	WTC_AD	Control#1_AD	Control#2_AD	PEG#1_AD	PEG#2_AD	PEG-DNA#1_AD
Chr01	6348013	0	0	0	2	0	0	17	0	0	0	0	0	0
Chr01	9544453	0	0	0	0	0	0	0	0	0	0	0	8	0
Chr01	13711188	1	1	0	2	0	0	15	0	0	NA	2	0	0
Chr01	13711188	1	1	0	2	0	0	15	0	0	NA	2	0	0
Chr01	13711188	1	1	0	2	0	0	15	0	0	NA	2	0	0
Chr01	16271546	0	0	0	10	0	0	2	0	0	0	2	1	NA
Chr01	16331294	0	0	0	0	0	0	0	0	0	6	0	0	0
Chr01	16331294	0	0	0	0	0	0	0	0	0	6	0	0	0
Chr01	16331294	0	0	0	0	0	0	0	0	0	6	0	0	0
Chr01	16331294	0	0	0	0	0	0	0	0	0	6	0	0	0
Chr01	16331294	0	0	0	0	0	0	0	0	0	6	0	0	0
Chr01	16331294	0	0	0	0	0	0	0	0	0	6	0	0	0
Chr01	16331294	0	0	0	0	0	0	0	0	0	6	0	0	0

CHROM	POS	WTB_DP	TAL#1_DP	TAL#2_DP	WTA_DP	CRIS1#1_DP	CRIS1#2_DP	CRIS2#1_DP	CRIS2#2_DP	WTC_DP	Control#1_DP	Control#2_DP	PEG#1_DP	PEG#2_DP	PEG-DNA#1_DP
Chr01	6348013	28	14	13	27	22	25	39	17	15	11	13	12	12	8
Chr01	9544453	20	16	14	23	16	25	33	22	16	24	13	15	8	6
Chr01	13711188	29	10	14	18	10	21	19	18	13	19	NA	12	6	6
Chr01	13711188	29	10	14	18	10	21	19	18	13	19	NA	12	6	6
Chr01	13711188	29	10	14	18	10	21	19	18	13	19	NA	12	6	6
Chr01	16271546	21	8	8	13	12	15	18	17	6	11	10	13	8	NA
Chr01	16331294	19	14	12	12	18	22	15	9	18	10	7	10	8	7
Chr01	16331294	19	14	12	12	18	22	15	9	18	10	7	10	8	7
Chr01	16331294	19	14	12	12	18	22	15	9	18	10	7	10	8	7
Chr01	16331294	19	14	12	12	18	22	15	9	18	10	7	10	8	7
Chr01	16331294	19	14	12	12	18	22	15	9	18	10	7	10	8	7
Chr01	16331294	19	14	12	12	18	22	15	9	18	10	7	10	8	7
Chr01	16331294	19	14	12	12	18	22	15	9	18	10	7	10	8	7

CHROM	POS	WTB_AR	TAL#1_AR	TAL#2_AR	WTA_AR	CRIS1#1_AR	CRIS1#2_AR	CRIS2#1_AR	CRIS2#2_AR	WTC_AR	Control#1_AR	Control#2_AR	PEG#1_AR	PEG#2_AR	PEG-DNA#1_AR	NCALLED
Chr01	6348013	0	0	0	0	0.091	0	0	1	0	0	0	0	0	0	0
Chr01	9544453	0	0	0	0	0	0	0	0	0	0	0	0	1	0	0
Chr01	13711188	0	0	0	0	0.2	0	0	0.833	0	0	NA	0.167	0	0	1
Chr01	13711188	0	0	0	0	0.2	0	0	0.833	0	0	NA	0.167	0	0	1
Chr01	13711188	0	0	0	0	0.2	0	0	0.833	0	0	NA	0.167	0	0	1
Chr01	16271546	0	0	0	0	0.833	0	0	0.118	0	0	0	0.154	0	NA	1
Chr01	16331294	0	0	0	0	0	0	0	0	0	0	0.857	0	0	0	0
Chr01	16331294	0	0	0	0	0	0	0	0	0	0	0.857	0	0	0	0
Chr01	16331294	0	0	0	0	0	0	0	0	0	0	0.857	0	0	0	0
Chr01	16331294	0	0	0	0	0	0	0	0	0	0	0.857	0	0	0	0
Chr01	16331294	0	0	0	0	0	0	0	0	0	0	0.857	0	0	0	0
Chr01	16331294	0	0	0	0	0	0	0	0	0	0	0.857	0	0	0	0
Chr01	16331294	0	0	0	0	0	0	0	0	0	0	0.857	0	0	0	0

CHROM	POS	VAR_OCC	VAR_OCC_H	VAR_OCC_F	VAR_OCC_cc	VAR_OCC_cr	VAR_OCC_ta	ANNOTATION	IMPACT	GENE_NAME	GENE_ID	FEATURE_TY	FEATURE_ID	CODING	EXON_RANK	HGVS.c	HGVS.p	cDNA_POSIT	CDS_POSITIC	PROT_POSIT	DISTANCEtoFEATURE
Chr01	6348013	1	1	1	0	1	0	intergenic_re	MODIFIER	Pp3c1_8710-	Pp3c1_8710-	intergenic_re	Pp3c1_8710-	NA	NA	n.6348013G>	NA	NA	NA	NA	NA
Chr01	9544453	1	1	0	1	0	0	intergenic_re	MODIFIER	Pp3c1_1308(	Pp3c1_1308(	intergenic_re	Pp3c1_1308(	NA	NA	n.9544453C>	NA	NA	NA	NA	NA
Chr01	13711188	1	1	2	0	1	0	downstream_	MODIFIER	Pp3c1_1821(	Pp3c1_1821(	transcript	PAC:3296862	protein_codi	NA	c.*1049_*10	NA	NA	NA	NA	667
Chr01	13711188	1	1	2	0	1	0	downstream_	MODIFIER	Pp3c1_1821(	Pp3c1_1821(	transcript	PAC:3296862	protein_codi	NA	c.*1049_*10	NA	NA	NA	NA	569
Chr01	13711188	1	1	2	0	1	0	intergenic_re	MODIFIER	Pp3c1_1821(	Pp3c1_1821(	intergenic_re	Pp3c1_1821(	NA	NA	n.13711188_	NA	NA	NA	NA	NA
Chr01	16271546	1	1	2	0	1	0	intergenic_re	MODIFIER	Pp3c1_2165(	Pp3c1_2165(	intergenic_re	Pp3c1_2165(	NA	NA	n.16271547_	NA	NA	NA	NA	NA
Chr01	16331294	1	1	0	1	0	0	upstream_ge	MODIFIER	Pp3c1_2178(	Pp3c1_2178(	transcript	PAC:3296990	protein_codi	NA	c.-4762_-475	NA	NA	NA	NA	4757
Chr01	16331294	1	1	0	1	0	0	upstream_ge	MODIFIER	Pp3c1_2179(	Pp3c1_2179(	transcript	PAC:3296674	protein_codi	NA	c.-864_-859(	NA	NA	NA	NA	731
Chr01	16331294	1	1	0	1	0	0	upstream_ge	MODIFIER	Pp3c1_2180(	Pp3c1_2180(	transcript	PAC:3297122	protein_codi	NA	c.-4246_-424	NA	NA	NA	NA	4072
Chr01	16331294	1	1	0	1	0	0	upstream_ge	MODIFIER	Pp3c1_2179(	Pp3c1_2179(	transcript	PAC:3296674	protein_codi	NA	c.-864_-859(	NA	NA	NA	NA	701
Chr01	16331294	1	1	0	1	0	0	upstream_ge	MODIFIER	Pp3c1_2180(	Pp3c1_2180(	transcript	PAC:3297122	protein_codi	NA	c.-4246_-424	NA	NA	NA	NA	3986
Chr01	16331294	1	1	0	1	0	0	downstream_	MODIFIER	Pp3c1_2178:	Pp3c1_2178:	transcript	PAC:3297036	protein_codi	NA	c.*2829_*28	NA	NA	NA	NA	2517
Chr01	16331294	1	1	0	1	0	0	intergenic_re	MODIFIER	Pp3c1_2178:	Pp3c1_2178:	intergenic_re	Pp3c1_2178:	NA	NA	n.16331295_	NA	NA	NA	NA	NA

Chr04	5878614	G	T	Chr04_58786	G_T	SNP	539.41	0	0	0	[illegible]
Chr04	5878614	G	T	Chr04_58786	G_T	SNP	539.41	0	0	0	[illegible]
Chr04	5878614	G	T	Chr04_58786	G_T	SNP	539.41	0	0	0	[illegible]
Chr04	5878614	G	T	Chr04_58786	G_T	SNP	539.41	0	0	0	[illegible]
Chr04	5878614	G	T	Chr04_58786	G_T	SNP	539.41	0	0	0	[illegible]
Chr04	5878614	G	T	Chr04_58786	G_T	SNP	539.41	0	0	0	[illegible]
Chr04	9851120	A	T	Chr04_98511	A_T	SNP	385.37	0	0	0	[illegible]
Chr04	9851120	A	T	Chr04_98511	A_T	SNP	385.37	0	0	0	[illegible]
Chr04	9851120	A	T	Chr04_98511	A_T	SNP	385.37	0	0	0	[illegible]
Chr04	9851120	A	T	Chr04_98511	A_T	SNP	385.37	0	0	0	[illegible]
Chr04	10691875	A	AAT	Chr04_10691	A_AAT	INDEL	271.37	1	10	0	[illegible]
Chr04	10691875	A	AAT	Chr04_10691	A_AAT	INDEL	271.37	1	10	0	[illegible]
Chr04	10691875	A	AAT	Chr04_10691	A_AAT	INDEL	271.37	1	10	0	[illegible]
Chr04	10691875	A	AAT	Chr04_10691	A_AAT	INDEL	271.37	1	10	0	[illegible]
Chr04	10691875	A	AAT	Chr04_10691	A_AAT	INDEL	271.37	1	10	0	[illegible]
Chr04	10691875	A	AAT	Chr04_10691	A_AAT	INDEL	271.37	1	10	0	[illegible]
Chr04	10691875	A	AAT	Chr04_10691	A_AAT	INDEL	271.37	1	10	0	[illegible]
Chr04	10691875	A	AAT	Chr04_10691	A_AAT	INDEL	271.37	1	10	0	[illegible]
Chr04	11455249	T	TTA	Chr04_11455	T_TTA	INDEL	284.37	0	2	0	[illegible]
Chr04	11455249	T	TTA	Chr04_11455	T_TTA	INDEL	284.37	0	2	0	[illegible]
Chr04	11455249	T	TTA	Chr04_11455	T_TTA	INDEL	284.37	0	2	0	[illegible]
Chr04	11455249	T	TTA	Chr04_11455	T_TTA	INDEL	284.37	0	2	0	[illegible]
Chr04	11673629	CAT	C	Chr04_11673	CAT_C	INDEL	190.37	1	NA	0	[illegible]
Chr04	11673629	CAT	C	Chr04_11673	CAT_C	INDEL	190.37	1	NA	0	[illegible]
Chr04	11673629	CAT	C	Chr04_11673	CAT_C	INDEL	190.37	1	NA	0	[illegible]
Chr04	11673629	CAT	C	Chr04_11673	CAT_C	INDEL	190.37	1	NA	0	[illegible]
Chr04	11673629	CAT	C	Chr04_11673	CAT_C	INDEL	190.37	1	NA	0	[illegible]
Chr04	11673629	CAT	C	Chr04_11673	CAT_C	INDEL	190.37	1	NA	0	[illegible]
Chr04	11673629	CAT	C	Chr04_11673	CAT_C	INDEL	190.37	1	NA	0	[illegible]
Chr04	11673629	CAT	C	Chr04_11673	CAT_C	INDEL	190.37	1	NA	0	[illegible]
Chr04	11673629	CAT	C	Chr04_11673	CAT_C	INDEL	190.37	1	NA	0	[illegible]
Chr04	11673629	CAT	C	Chr04_11673	CAT_C	INDEL	190.37	1	NA	0	[illegible]
Chr04	11673629	CAT	C	Chr04_11673	CAT_C	INDEL	190.37	1	NA	0	[illegible]
Chr04	11673629	CAT	C	Chr04_11673	CAT_C	INDEL	190.37	1	NA	0	[illegible]
Chr04	12251280	A	AAT	Chr04_12251	A_AAT	INDEL	376.37	0	0	0	[illegible]
Chr04	12251280	A	AAT	Chr04_12251	A_AAT	INDEL	376.37	0	0	0	[illegible]
Chr04	18842965	C	A	Chr04_18842	C_A	SNP	1071.41	0	0	0	[illegible]
Chr05	1696894	T	A	Chr05_16968	T_A	SNP	80.41	0	0	4	[illegible]
Chr05	2042829	G	GTA	Chr05_20428	G_GTA	INDEL	386.37	0	1	0	[illegible]
Chr05	2042829	G	GTA	Chr05_20428	G_GTA	INDEL	386.37	0	1	0	[illegible]
Chr05	2042829	G	GTA	Chr05_20428	G_GTA	INDEL	386.37	0	1	0	[illegible]
Chr05	2042829	G	GTA	Chr05_20428	G_GTA	INDEL	386.37	0	1	0	[illegible]
Chr05	3284447	A	AAT	Chr05_32844	A_AAT	INDEL	424.37	0	0	0	[illegible]
Chr05	3370462	CAT	C	Chr05_33704	CAT_C	INDEL	243.37	0	10	2	[illegible]
Chr05	6410263	C	A	Chr05_64102	C_A	SNP	804.41	0	0	0	[illegible]
Chr05	6961151	G	A	Chr05_69611	G_A	SNP	936.41	0	0	22	[illegible]
Chr05	7115825	T	TTATA	Chr05_71158	T_TTATA	INDEL	392.37	0	0	9	[illegible]
Chr05	8798241	T	TTA	Chr05_87982	T_TTA	INDEL	88.37	0	0	0	[illegible]
Chr05	8798241	T	TTA	Chr05_87982	T_TTA	INDEL	88.37	0	0	0	[illegible]
Chr05	8798241	T	TTA	Chr05_87982	T_TTA	INDEL	88.37	0	0	0	[illegible]
Chr05	8798241	T	TTA	Chr05_87982	T_TTA	INDEL	88.37	0	0	0	[illegible]
Chr05	8798241	T	TTA	Chr05_87982	T_TTA	INDEL	88.37	0	0	0	[illegible]
Chr05	8798241	T	TTA	Chr05_87982	T_TTA	INDEL	88.37	0	0	0	[illegible]
Chr05	8798241	T	TTA	Chr05_87982	T_TTA	INDEL	88.37	0	0	0	[illegible]
Chr05	8798241	T	TTA	Chr05_87982	T_TTA	INDEL	88.37	0	0	0	[illegible]
Chr05	8798241	T	TTA	Chr05_87982	T_TTA	INDEL	88.37	0	0	0	[illegible]
Chr05	9350354	C	A	Chr05_93503	C_A	SNP	1022.41	0	0	0	[illegible]
Chr05	10460070	C	CTA	Chr05_10460	C_CTA	INDEL	340.49	3	1	0	[illegible]
Chr05	10460070	C	CTA	Chr05_10460	C_CTA	INDEL	340.49	3	1	0	[illegible]
Chr05	10460070	C	CTA	Chr05_10460	C_CTA	INDEL	340.49	3	1	0	[illegible]
Chr05	10695758	A	AAT	Chr05_10695	A_AAT	INDEL	546.37	0	0	0	[illegible]
Chr05	15048587	A	AAT	Chr05_15048	A_AAT	INDEL	280.37	2	0	0	[illegible]
Chr05	15048587	A	AAT	Chr05_15048	A_AAT	INDEL	280.37	2	0	0	[illegible]
Chr05	15048587	A	AAT	Chr05_15048	A_AAT	INDEL	280.37	2	0	0	[illegible]
Chr05	15048587	A	AAT	Chr05_15048	A_AAT	INDEL	280.37	2	0	0	[illegible]
Chr05	15048587	A	AAT	Chr05_15048	A_AAT	INDEL	280.37	2	0	0	[illegible]
Chr05	15048587	A	AAT	Chr05_15048	A_AAT	INDEL	280.37	2	0	0	[illegible]
Chr05	15049377	G	GTA	Chr05_15049	G_GTA	INDEL	190.37	2	9	0	[illegible]
Chr05	15049377	G	GTA	Chr05_15049	G_GTA	INDEL	190.37	2	9	0	[illegible]
Chr05	15049377	G	GTA	Chr05_15049	G_GTA	INDEL	190.37	2	9	0	[illegible]
Chr05	15049377	G	GTA	Chr05_15049	G_GTA	INDEL	190.37	2	9	0	[illegible]
Chr05	15049377	G	GTA	Chr05_15049	G_GTA	INDEL	190.37	2	9	0	[illegible]
Chr05	15049377	G	GTA	Chr05_15049	G_GTA	INDEL	190.37	2	9	0	[illegible]
Chr05	15241968	A	AAT	Chr05_15241	A_AAT	INDEL	175.37	0	0	0	[illegible]
Chr05	15241968	A	AAT	Chr05_15241	A_AAT	INDEL	175.37	0	0	0	[illegible]
Chr05	15241968	A	AAT	Chr05_15241	A_AAT	INDEL	175.37	0	0	0	[illegible]
Chr05	15241968	A	AAT	Chr05_15241	A_AAT	INDEL	175.37	0	0	0	[illegible]
Chr05	15241968	A	AAT	Chr05_15241	A_AAT	INDEL	175.37	0	0	0	[illegible]
Chr05	15241968	A	AAT	Chr05_15241	A_AAT	INDEL	175.37	0	0	0	[illegible]
Chr05	15241968	A	AAT	Chr05_15241	A_AAT	INDEL	175.37	0	0	0	[illegible]
Chr05	15241968	A	AAT	Chr05_15241	A_AAT	INDEL	175.37	0	0	0	[illegible]
Chr05	15241968	A	AAT	Chr05_15241	A_AAT	INDEL	175.37	0	0	0	[illegible]
Chr05	15241968	A	AAT	Chr05_15241	A_AAT	INDEL	175.37	0	0	0	[illegible]
Chr05	15241968	A	AAT	Chr05_15241	A_AAT	INDEL	175.37	0	0	0	[illegible]
Chr05	15241968	A	AAT	Chr05_15241	A_AAT	INDEL	175.37	0	0	0	[illegible]
Chr05	15241968	A	AAT	Chr05_15241	A_AAT	INDEL	175.37	0	0	0	[illegible]
Chr05	15588541	G	A	Chr05_15588	G_A	SNP	463.41	0	0	0	[illegible]
Chr05	15598999	C	CTA	Chr05_15598	C_CTA	INDEL	222.37	1	1	0	[illegible]
Chr05	15598999	C	CTA	Chr05_15598	C_CTA	INDEL	222.37	1	1	0	[illegible]
Chr05	15598999	C	CTA	Chr05_15598	C_CTA	INDEL	222.37	1	1	0	[illegible]
Chr05	16414462	G	T	Chr05_16414	G_T	SNP	486.41	0	0	13	[illegible]
Chr05	16551910	G	GTA	Chr05_16551	G_GTA	INDEL	142.37	0	6	1	[illegible]
Chr05	16551910	G	GTA	Chr05_16551	G_GTA	INDEL	142.37	0	6	1	[illegible]
Chr05	16551910	G	GTA	Chr05_16551	G_GTA	INDEL	142.37	0	6	1	[illegible]
Chr05	17101862	CAT	C	Chr05_17101	CAT_C	INDEL	1180.35	0	10	0	[illegible]
Chr05	17101862	CAT	C	Chr05_17101	CAT_C	INDEL	1180.35	0	10	0	[illegible]
Chr05	17101862	CAT	C	Chr05_17101	CAT_C	INDEL	1180.35	0	10	0	[illegible]
Chr05	17101862	CAT	C	Chr05_17101	CAT_C	INDEL	1180.35	0	10	0	[illegible]
Chr05	17101862	CAT	C	Chr05_17101	CAT_C	INDEL	1180.35	0	10	0	[illegible]
Chr05	17101862	CAT	C	Chr05_17101	CAT_C	INDEL	1180.35	0	10	0	[illegible]
Chr05	17101862	CAT	C	Chr05_17101	CAT_C	INDEL	1180.35	0	10	0	[illegible]
Chr05	17101862	CAT	C	Chr05_17101	CAT_C	INDEL	1180.35	0	10	0	[illegible]
Chr05	17101862	CAT	C	Chr05_17101	CAT_C	INDEL	1180.35	0	10	0	[illegible]
Chr05	17101862	CAT	C	Chr05_17101	CAT_C	INDEL	1180.35	0	10	0	[illegible]
Chr05	17101862	CAT	C	Chr05_17101	CAT_C	INDEL	1180.35	0	10	0	[illegible]
Chr05	17101862	CAT	C	Chr05_17101	CAT_C	INDEL	1180.35	0	10	0	[illegible]
Chr05	17101862	CAT	C	Chr05_17101	CAT_C	INDEL	1180.35	0	10	0	[illegible]
Chr05	17101862	CAT	C	Chr05_17101	CAT_C	INDEL	1180.35	0	10	0	[illegible]
Chr05	17101862	CAT	C	Chr05_17101	CAT_C	INDEL	1180.35	0	10	0	[illegible]
Chr05	17101862	CAT	C	Chr05_17101	CAT_C	INDEL	1180.35	0	10	0	[illegible]
Chr05	17101862	CAT	C	Chr05_17101	CAT_C	INDEL	1180.35	0	10	0	[illegible]
Chr05	17101862	CAT	C	Chr05_17101	CAT_C	INDEL	1180.35	0	10	0	[illegible]
Chr05	17101862	CAT	C	Chr05_17101	CAT_C	INDEL	1180.35	0	10	0	[illegible]
Chr05	20222233	A	AT	Chr05_20222	A_AT	INDEL	440.37	0	16	0	[illegible]
Chr06	2654981	A	AAT	Chr06_26549	A_AAT	INDEL	355.63	0	NA	1	[illegible]
Chr06	3570175	AAT	AATAT	Chr06_35701	AAT_AATAT	INDEL	1627.93	2	0	0	[illegible]
Chr06	3570175	AAT	AATAT	Chr06_35701	AAT_AATAT	INDEL	1627.93	2	0	0	[illegible]
Chr06	3570175	AAT	AATAT	Chr06_35701	AAT_AATAT	INDEL	1627.93	2	0	0	[illegible]
Chr06	3570175	AAT	AATAT	Chr06_35701	AAT_AATAT	INDEL	1627.93	2	0	0	[illegible]
Chr06	4665245	A	AAT	Chr06_46652	A_AAT	INDEL	482.37	0	1	0	[illegible]
Chr06	4665245	A	AAT	Chr06_46652	A_AAT	INDEL	482.37	0	1	0	[illegible]
Chr06	4665245	A	AAT	Chr06_46652	A_AAT	INDEL	482.37	0	1	0	[illegible]
Chr06	4665245	A	AAT	Chr06_46652	A_AAT	INDEL	482.37	0	1	0	[illegible]
Chr06	4665245	A	AAT	Chr06_46652	A_AAT	INDEL	482.37	0	1	0	[illegible]
Chr06	4665245	A	AAT	Chr06_46652	A_AAT	INDEL	482.37	0	1	0	[illegible]
Chr06	4665245	A	AAT	Chr06_46652	A_AAT	INDEL	482.37	0	1	0	[illegible]
Chr06	4665245	A	AAT	Chr06_46652	A_AAT	INDEL	482.37	0	1	0	[illegible]
Chr06	4665245	A	AAT	Chr06_46652	A_AAT	INDEL	482.37	0	1	0	[illegible]
Chr06	4665245	A	AAT	Chr06_46652	A_AAT	INDEL	482.37	0	1	0	[illegible]
Chr06	4665245	A	AAT	Chr06_46652	A_AAT	INDEL	482.37	0	1	0	[illegible]
Chr06	4665245	A	AAT	Chr06_46652	A_AAT	INDEL	482.37	0	1	0	[illegible]
Chr06	5739121	T	TTA	Chr06_57391	T_TTA	INDEL	267.37	0	0	1	[illegible]
Chr06	5739121	T	TTA	Chr06_57391	T_TTA	INDEL	267.37	0	0	1	[illegible]
Chr06	5739121	T	TTA	Chr06_57391	T_TTA	INDEL	267.37	0	0	1	[illegible]
Chr06	5739121	T	TTA	Chr06_57391	T_TTA	INDEL	267.37	0	0	1	[illegible]
Chr06	5739121	T	TTA	Chr06_57391	T_TTA	INDEL	267.37	0	0	1	[illegible]
Chr06	5739121	T	TTA	Chr06_57391	T_TTA	INDEL	267.37	0	0	1	[illegible]
Chr06	6220112	C	CAT	Chr06_62201	C_CAT	INDEL	290.37	0	1	10	[illegible]
Chr06	6220112	C	CAT	Chr06_62201	C_CAT	INDEL	290.37	0	1	10	[illegible]
Chr06	6220112	C	CAT	Chr06_62201	C_CAT	INDEL	290.37	0	1	10	[illegible]
Chr06	6220112	C	CAT	Chr06_62201	C_CAT	INDEL	290.37	0	1	10	[illegible]
Chr06	6220112	C	CAT	Chr06_62201	C_CAT	INDEL	290.37	0	1	10	[illegible]
Chr06	6220112	C	CAT	Chr06_62201	C_CAT	INDEL	290.37	0	1	10	[illegible]
Chr06	6220112	C	CAT	Chr06_62201	C_CAT	INDEL	290.37	0	1	10	[illegible]
Chr06	6220112	C	CAT	Chr06_62201	C_CAT	INDEL	290.37	0	1	10	[illegible]
Chr06	6220112	C	CAT	Chr06_62201	C_CAT	INDEL	290.37	0	1	10	[illegible]
Chr06	7346938	G	T	Chr06_73469	G_T	SNP	537.41	0	0	13	[illegible]
Chr06	7346938	G	T	Chr06_73469	G_T	SNP	537.41	0	0	13	[illegible]
Chr06	7346938	G	T	Chr06_73469	G_T	SNP	537.41	0	0	13	[illegible]
Chr06	7346938	G	T	Chr06_73469	G_T	SNP	537.41	0	0	13	[illegible]
Chr06	7371055	C	T	Chr06_73710	C_T	SNP	736.41	0	0	0	[illegible]
Chr06	8865209	C	CAT	Chr06_88652	C_CAT	INDEL	319.37	0	0	11	[illegible]
Chr06	8865209	C	CAT	Chr06_88652	C_CAT	INDEL	319.37	0	0	11	[illegible]
Chr06	8865209	C	CAT	Chr06_88652	C_CAT	INDEL	319.37	0	0	11	[illegible]
Chr06	8865209	C	CAT	Chr06_88652	C_CAT	INDEL	319.37	0	0	11	[illegible]
Chr06	8865209	C	CAT	Chr06_88652	C_CAT	INDEL	319.37	0	0	11	[illegible]
Chr06	8865209	C	CAT	Chr06_88652	C_CAT	INDEL	319.37	0	0	11	[illegible]
Chr06	8865209	C	CAT	Chr06_88652	C_CAT	INDEL	319.37	0	0	11	[illegible]
Chr06	8865209	C	CAT	Chr06_88652	C_CAT	INDEL	319.37	0	0	11	[illegible]
Chr06	8865209	C	CAT	Chr06_88652	C_CAT	INDEL	319.37	0	0	11	[illegible]
Chr06	8865209	C	CAT	Chr06_88652	C_CAT	INDEL	319.37	0	0	11	[illegible]
Chr06	8865209	C	CAT	Chr06_88652	C_CAT	INDEL	319.37	0	0	11	[illegible]
Chr06	8865209	C	CAT	Chr06_88652	C_CAT	INDEL	319.37	0	0	11	[illegible]
Chr06	9320007	G	GAT	Chr06_93200	G_GAT	INDEL	552.37	0	0	0	[illegible]
Chr06	9320007	G	GAT	Chr06_93200	G_GAT	INDEL	552.37	0	0	0	[illegible]
Chr06	9320007	G	GAT	Chr06_93200	G_GAT	INDEL	552.37	0	0	0	[illegible]
Chr06	9320007	G	GAT	Chr06_93200	G_GAT	INDEL	552.37	0	0	0	[illegible]
Chr06	9320007	G	GAT	Chr06_93200	G_GAT	INDEL	552.37	0	0	0	[illegible]
Chr06	9320007	G	GAT	Chr06_93200	G_GAT	INDEL	552.37	0	0	0	[illegible]
Chr06	11512859	C	CAT	Chr06_11512	C_CAT	INDEL	347.37	1	0	0	[illegible]
Chr06	11512859	C	CAT	Chr06_11512	C_CAT	INDEL	347.37	1	0	0	[illegible]
Chr06	11512859	C	CAT	Chr06_11512	C_CAT	INDEL	347.37	1	0	0	[illegible]
Chr06	11512859	C	CAT	Chr06_11512	C_CAT	INDEL	347.37	1	0	0	[illegible]
Chr06	13309828	AAT	A	Chr06_13309	AAT_A	INDEL	1008.37	0	0	0	[illegible]
Chr06	13309828	AAT	A	Chr06_13309	AAT_A	INDEL	1008.37	0	0	0	[illegible]
Chr06	13309828	AAT	A	Chr06_13309	AAT_A	INDEL	1008.37	0	0	0	[illegible]
Chr06	13309828	AAT	A	Chr06_13309	AAT_A	INDEL	1008.37	0	0	0	[illegible]
Chr06	13309828	AAT	A	Chr06_13309	AAT_A	INDEL	1008.37	0	0	0	[illegible]
Chr06	13309828	AAT	A	Chr06_13309	AAT_A	INDEL	1008.37	0	0	0	[illegible]
Chr06	13309828	AAT	A	Chr06_13309	AAT_A	INDEL	1008.37	0	0	0	[illegible]
Chr06	14762240	G	GTA	Chr06_14762	G_GTA	INDEL	268.37	0	0	11	[illegible]
Chr06	14762240	G	GTA	Chr06_14762	G_GTA	INDEL	268.37	0	0	11	[illegible]
Chr06	14762240	G	GTA	Chr06_14762	G_GTA	INDEL	268.37	0	0	11	[illegible]
Chr06	14762240	G	GTA	Chr06_14762	G_GTA	INDEL	268.37	0	0	11	[illegible]
Chr06	14762240	G	GTA	Chr06_14762	G_GTA	INDEL	268.37	0	0	11	[illegible]
Chr06	14771989	C	T	Chr06_14771	C_T	SNP	900.41	0	0	0	[illegible]
Chr06	14871263	T	TTA	Chr06_14871	T_TTA	INDEL	1128.37	0	0	0	[illegible]
Chr06	14871263	T	TTA	Chr06_14871	T_TTA	INDEL	1128.37	0	0	0	[illegible]
Chr06	14871263	T	TTA	Chr06_14871	T_TTA	INDEL	1128.37	0	0	0	[illegible]
Chr06	14871263	T	TTA	Chr06_14871	T_TTA	INDEL	1128.37	0	0	0	[illegible]
Chr06	14871263	T	TTA	Chr06_14871	T_TTA	INDEL	1128.37	0	0	0	[illegible]
Chr06	14871263	T	TTA	Chr06_14871	T_TTA	INDEL	1128.37	0	0	0	[illegible]
Chr06	14871263	T	TTA	Chr06_14871	T_TTA	INDEL	1128.37	0	0	0	[illegible]
Chr06	14871263	T	TTA	Chr06_14871	T_TTA	INDEL	1128.37	0	0	0	[illegible]
Chr06	14871263	T	TTA	Chr06_14871	T_TTA	INDEL	1128.37	0	0	0	[illegible]
Chr06	14871263	T	TTA	Chr06_14871	T_TTA	INDEL	1128.37	0	0	0	[illegible]
Chr06	14871263	T	TTA	Chr06_14871	T_TTA	INDEL	1128.37	0	0	0	[illegible]
Chr06	14871263	T	TTA	Chr06_14871	T_TTA	INDEL	1128.37	0	0	0	[illegible]
Chr06	15196371	A	AAT	Chr06_15196	A_AAT	INDEL	188.37	1	0	0	[illegible]
Chr06	15196371	A	AAT	Chr06_15196	A_AAT	INDEL	188.37	1	0	0	[illegible]
Chr06	15196371	A	AAT	Chr06_15196	A_AAT	INDEL	188.37	1	0	0	[illegible]
Chr06	15196371	A	AAT	Chr06_15196	A_AAT	INDEL	188.37	1	0	0	[illegible]

Chr06	15196371	A	AAT	Chr06_15196	A_AAT	INDEL	188.37	1	0	0	0	0	0	1	0	1	0	0	0	7
Chr06	15196371	A	AAT	Chr06_15196	A_AAT	INDEL	188.37	1	0	0	0	0	0	1	0	1	0	0	0	7
Chr06	15196371	A	AAT	Chr06_15196	A_AAT	INDEL	188.37	1	0	0	0	0	0	1	0	1	0	0	0	7
Chr06	15196371	A	AAT	Chr06_15196	A_AAT	INDEL	188.37	1	0	0	0	0	0	1	0	1	0	0	0	7
Chr06	15205009	C	CAT	Chr06_15205	C_CAT	INDEL	310.37	0	1	12	0	0	0	0	0	1	0	0	1	0 NA
Chr06	15205009	C	CAT	Chr06_15205	C_CAT	INDEL	310.37	0	1	12	0	0	0	0	0	1	0	0	1	0 NA
Chr06	15205009	C	CAT	Chr06_15205	C_CAT	INDEL	310.37	0	1	12	0	0	0	0	0	1	0	0	1	0 NA
Chr06	15205009	C	CAT	Chr06_15205	C_CAT	INDEL	310.37	0	1	12	0	0	0	0	0	1	0	0	1	0 NA
Chr06	15205009	C	CAT	Chr06_15205	C_CAT	INDEL	310.37	0	1	12	0	0	0	0	0	1	0	0	1	0 NA
Chr06	15205009	C	CAT	Chr06_15205	C_CAT	INDEL	310.37	0	1	12	0	0	0	0	0	1	0	0	1	0 NA
Chr06	15205009	C	CAT	Chr06_15205	C_CAT	INDEL	310.37	0	1	12	0	0	0	0	0	1	0	0	1	0 NA
Chr06	15205009	C	CAT	Chr06_15205	C_CAT	INDEL	310.37	0	1	12	0	0	0	0	0	1	0	0	1	0 NA
Chr06	15205009	C	CAT	Chr06_15205	C_CAT	INDEL	310.37	0	1	12	0	0	0	0	0	1	0	0	1	0 NA
Chr06	15205009	C	CAT	Chr06_15205	C_CAT	INDEL	310.37	0	1	12	0	0	0	0	0	1	0	0	1	0 NA
Chr06	16906943	C	T	Chr06_16906	C_T	SNP	617.41	0	0	0	0	20	0	0	1	0	0	0	0	0
Chr06	17502580	A	AAT	Chr06_17502	A_AAT	INDEL	200.37	0	0	8	0	0	1	1	0	0	0	0	0	0
Chr06	17502580	A	AAT	Chr06_17502	A_AAT	INDEL	200.37	0	0	8	0	0	1	1	0	0	0	0	0	0
Chr06	17502580	A	AAT	Chr06_17502	A_AAT	INDEL	200.37	0	0	8	0	0	1	1	0	0	0	0	0	0
Chr06	17502580	A	AAT	Chr06_17502	A_AAT	INDEL	200.37	0	0	8	0	0	1	1	0	0	0	0	0	0
Chr06	17502580	A	AAT	Chr06_17502	A_AAT	INDEL	200.37	0	0	8	0	0	1	1	0	0	0	0	0	0
Chr06	17502580	A	AAT	Chr06_17502	A_AAT	INDEL	200.37	0	0	8	0	0	1	1	0	0	0	0	0	0
Chr06	17502580	A	AAT	Chr06_17502	A_AAT	INDEL	200.37	0	0	8	0	0	1	1	0	0	0	0	0	0
Chr06	18710749	C	CAT	Chr06_18710	C_CAT	INDEL	295.37	0	0	0	0	2	0	0	13	0	0	0	0	0
Chr06	18710749	C	CAT	Chr06_18710	C_CAT	INDEL	295.37	0	0	0	0	2	0	0	13	0	0	0	0	0
[illegible]																				

Chr10	14320441	C	G	Chr10_14320C_G	SNP	332.41	0	[illegible]
Chr10	14320441	C	G	Chr10_14320C_G	SNP	332.41	0	[illegible]
Chr10	14329574	G	A	Chr10_14329G_A	SNP	682.41	0	[illegible]
Chr10	14329574	G	A	Chr10_14329G_A	SNP	682.41	0	[illegible]
Chr10	14329574	G	A	Chr10_14329G_A	SNP	682.41	0	[illegible]
Chr10	14329574	G	A	Chr10_14329G_A	SNP	682.41	0	[illegible]
Chr10	14329574	G	A	Chr10_14329G_A	SNP	682.41	0	[illegible]
Chr10	14329575	A	T	Chr10_14329A_T	SNP	682.41	0	[illegible]
Chr10	14329575	A	T	Chr10_14329A_T	SNP	682.41	0	[illegible]
Chr10	14329575	A	T	Chr10_14329A_T	SNP	682.41	0	[illegible]
Chr10	14329575	A	T	Chr10_14329A_T	SNP	682.41	0	[illegible]
Chr10	14329575	A	T	Chr10_14329A_T	SNP	682.41	0	[illegible]
Chr10	14329577	A	T	Chr10_14329A_T	SNP	682.41	0	[illegible]
Chr10	14329577	A	T	Chr10_14329A_T	SNP	682.41	0	[illegible]
Chr10	14329577	A	T	Chr10_14329A_T	SNP	682.41	0	[illegible]
Chr10	14329577	A	T	Chr10_14329A_T	SNP	682.41	0	[illegible]
Chr10	14329577	A	T	Chr10_14329A_T	SNP	682.41	0	[illegible]
Chr11	1541587	T	TTA	Chr11_15415T_TTA	INDEL	66.37	1	[illegible]
Chr11	1541587	T	TTA	Chr11_15415T_TTA	INDEL	66.37	1	[illegible]
Chr11	1541587	T	TTA	Chr11_15415T_TTA	INDEL	66.37	1	[illegible]
Chr11	1541587	T	TTA	Chr11_15415T_TTA	INDEL	66.37	1	[illegible]
Chr11	1541587	T	TTA	Chr11_15415T_TTA	INDEL	66.37	1	[illegible]
Chr11	1541587	T	TTA	Chr11_15415T_TTA	INDEL	66.37	1	[illegible]
Chr11	1627481	TCGC	T	Chr11_16274TCGC_T	INDEL	673.37	0	[illegible]
Chr11	1627481	TCGC	T	Chr11_16274TCGC_T	INDEL	673.37	0	[illegible]
Chr11	1627481	TCGC	T	Chr11_16274TCGC_T	INDEL	673.37	0	[illegible]
Chr11	1627481	TCGC	T	Chr11_16274TCGC_T	INDEL	673.37	0	[illegible]
Chr11	1627481	TCGC	T	Chr11_16274TCGC_T	INDEL	673.37	0	[illegible]
Chr11	1627481	TCGC	T	Chr11_16274TCGC_T	INDEL	673.37	0	[illegible]
Chr11	1627481	TCGC	T	Chr11_16274TCGC_T	INDEL	673.37	0	[illegible]
Chr11	1627481	TCGC	T	Chr11_16274TCGC_T	INDEL	673.37	0	[illegible]
Chr11	1627484	C	CAT	Chr11_16274C_CAT	INDEL	673.37	0	[illegible]
Chr11	1627484	C	CAT	Chr11_16274C_CAT	INDEL	673.37	0	[illegible]
Chr11	1627484	C	CAT	Chr11_16274C_CAT	INDEL	673.37	0	[illegible]
Chr11	1627484	C	CAT	Chr11_16274C_CAT	INDEL	673.37	0	[illegible]
Chr11	1627484	C	CAT	Chr11_16274C_CAT	INDEL	673.37	0	[illegible]
Chr11	1627484	C	CAT	Chr11_16274C_CAT	INDEL	673.37	0	[illegible]
Chr11	1627484	C	CAT	Chr11_16274C_CAT	INDEL	673.37	0	[illegible]
Chr11	1627484	C	CAT	Chr11_16274C_CAT	INDEL	673.37	0	[illegible]
Chr11	1688923	T	TTA	Chr11_16889T_TTA	INDEL	357.37	0	[illegible]
Chr11	1688923	T	TTA	Chr11_16889T_TTA	INDEL	357.37	0	[illegible]
Chr11	1688923	T	TTA	Chr11_16889T_TTA	INDEL	357.37	0	[illegible]
Chr11	2912741	C	CTA	Chr11_29127C_CTA	INDEL	340.37	0	[illegible]
Chr11	2912741	C	CTA	Chr11_29127C_CTA	INDEL	340.37	0	[illegible]
Chr11	2912741	C	CTA	Chr11_29127C_CTA	INDEL	340.37	0	[illegible]
Chr11	2912741	C	CTA	Chr11_29127C_CTA	INDEL	340.37	0	[illegible]
Chr11	2912741	C	CTA	Chr11_29127C_CTA	INDEL	340.37	0	[illegible]
Chr11	2912741	C	CTA	Chr11_29127C_CTA	INDEL	340.37	0	[illegible]
Chr11	2912741	C	CTA	Chr11_29127C_CTA	INDEL	340.37	0	[illegible]
Chr11	2912741	C	CTA	Chr11_29127C_CTA	INDEL	340.37	0	[illegible]
Chr11	2912741	C	CTA	Chr11_29127C_CTA	INDEL	340.37	0	[illegible]
Chr11	2912741	C	CTA	Chr11_29127C_CTA	INDEL	340.37	0	[illegible]
Chr11	2912741	C	CTA	Chr11_29127C_CTA	INDEL	340.37	0	[illegible]
Chr11	2912741	C	CTA	Chr11_29127C_CTA	INDEL	340.37	0	[illegible]
Chr11	2912741	C	CTA	Chr11_29127C_CTA	INDEL	340.37	0	[illegible]
Chr11	2912741	C	CTA	Chr11_29127C_CTA	INDEL	340.37	0	[illegible]
Chr11	2912741	C	CTA	Chr11_29127C_CTA	INDEL	340.37	0	[illegible]
Chr11	2912741	C	CTA	Chr11_29127C_CTA	INDEL	340.37	0	[illegible]
Chr11	2912741	C	CTA	Chr11_29127C_CTA	INDEL	340.37	0	[illegible]
Chr11	3682951	G	GTA	Chr11_36829G_GTA	INDEL	268.37	1	[illegible]
Chr11	3682951	G	GTA	Chr11_36829G_GTA	INDEL	268.37	1	[illegible]
Chr11	3682951	G	GTA	Chr11_36829G_GTA	INDEL	268.37	1	[illegible]
Chr11	3682951	G	GTA	Chr11_36829G_GTA	INDEL	268.37	1	[illegible]
Chr11	3682951	G	GTA	Chr11_36829G_GTA	INDEL	268.37	1	[illegible]
Chr11	3682951	G	GTA	Chr11_36829G_GTA	INDEL	268.37	1	[illegible]
Chr11	6047525	C	CAT	Chr11_60475C_CAT	INDEL	681.37	0	[illegible]
Chr11	6047525	C	CAT	Chr11_60475C_CAT	INDEL	681.37	0	[illegible]
Chr11	6047525	C	CAT	Chr11_60475C_CAT	INDEL	681.37	0	[illegible]
Chr11	6047525	C	CAT	Chr11_60475C_CAT	INDEL	681.37	0	[illegible]
Chr11	6608033	G	T	Chr11_66080G_T	SNP	316.41	0	[illegible]
Chr11	7699126	G	A	Chr11_76991G_A	SNP	547.41	0	[illegible]
Chr11	7699131	A	G	Chr11_76991A_G	SNP	547.41	0	[illegible]
Chr11	8646691	T	A	Chr11_86466T_A	SNP	463.41	0	[illegible]
Chr11	10649828	A	AAT	Chr11_10649A_AAT	INDEL	495.37	2	[illegible]
Chr11	10649828	A	AAT	Chr11_10649A_AAT	INDEL	495.37	2	[illegible]
Chr11	15145460	G	A	Chr11_15145G_A	SNP	683.41	0	[illegible]
Chr11	16493050	G	A	Chr11_16493G_A	SNP	463.41	0	[illegible]
Chr11	16727439	A	T	Chr11_16727A_T	SNP	883.37	0	[illegible]
Chr11	16727439	A	T	Chr11_16727A_T	SNP	883.37	0	[illegible]
Chr11	16727439	A	T	Chr11_16727A_T	SNP	883.37	0	[illegible]
Chr11	16727439	A	T	Chr11_16727A_T	SNP	883.37	0	[illegible]
Chr11	16727439	A	T	Chr11_16727A_T	SNP	883.37	0	[illegible]
Chr11	16727439	A	T	Chr11_16727A_T	SNP	883.37	0	[illegible]
Chr11	16727439	A	T	Chr11_16727A_T	SNP	883.37	0	[illegible]
Chr12	1604497	T	TTA	Chr12_16044T_TTA	INDEL	431.37	3	[illegible]
Chr12	1604497	T	TTA	Chr12_16044T_TTA	INDEL	431.37	3	[illegible]
Chr12	1604497	T	TTA	Chr12_16044T_TTA	INDEL	431.37	3	[illegible]
Chr12	2459708	G	T	Chr12_24597G_T	SNP	566.41	0	[illegible]
Chr12	2459708	G	T	Chr12_24597G_T	SNP	566.41	0	[illegible]
Chr12	2846886	G	GTA	Chr12_28468G_GTA	INDEL	293.37	1	[illegible]
Chr12	2846886	G	GTA	Chr12_28468G_GTA	INDEL	293.37	1	[illegible]
Chr12	2846886	G	GTA	Chr12_28468G_GTA	INDEL	293.37	1	[illegible]
Chr12	2846886	G	GTA	Chr12_28468G_GTA	INDEL	293.37	1	[illegible]
Chr12	2846886	G	GTA	Chr12_28468G_GTA	INDEL	293.37	1	[illegible]
Chr12	2846886	G	GTA	Chr12_28468G_GTA	INDEL	293.37	1	[illegible]
Chr12	2846886	G	GTA	Chr12_28468G_GTA	INDEL	293.37	1	[illegible]
Chr12	2846886	G	GTA	Chr12_28468G_GTA	INDEL	293.37	1	[illegible]
Chr12	2846886	G	GTA	Chr12_28468G_GTA	INDEL	293.37	1	[illegible]
Chr12	2846886	G	GTA	Chr12_28468G_GTA	INDEL	293.37	1	[illegible]
Chr12	2846886	G	GTA	Chr12_28468G_GTA	INDEL	293.37	1	[illegible]
Chr12	2846886	G	GTA	Chr12_28468G_GTA	INDEL	293.37	1	[illegible]
Chr12	2846886	G	GTA	Chr12_28468G_GTA	INDEL	293.37	1	[illegible]
Chr12	3079542	A	AG	Chr12_30795A_AG	INDEL	142.37	0	[illegible]
Chr12	3079542	A	AG	Chr12_30795A_AG	INDEL	142.37	0	[illegible]
Chr12	3079542	A	AG	Chr12_30795A_AG	INDEL	142.37	0	[illegible]
Chr12	3079542	A	AG	Chr12_30795A_AG	INDEL	142.37	0	[illegible]
Chr12	3079542	A	AG	Chr12_30795A_AG	INDEL	142.37	0	[illegible]
Chr12	3079542	A	AG	Chr12_30795A_AG	INDEL	142.37	0	[illegible]
Chr12	3079542	A	AG	Chr12_30795A_AG	INDEL	142.37	0	[illegible]
Chr12	4352393	A	AAT	Chr12_43523A_AAT	INDEL	357.37	0	[illegible]
Chr12	4352393	A	AAT	Chr12_43523A_AAT	INDEL	357.37	0	[illegible]
Chr12	4352393	A	AAT	Chr12_43523A_AAT	INDEL	357.37	0	[illegible]
Chr12	4352393	A	AAT	Chr12_43523A_AAT	INDEL	357.37	0	[illegible]
Chr12	8078381	A	AAT	Chr12_80783A_AAT	INDEL	237.37	1	[illegible]
Chr12	8078381	A	AAT	Chr12_80783A_AAT	INDEL	237.37	1	[illegible]
Chr12	8078381	A	AAT	Chr12_80783A_AAT	INDEL	237.37	1	[illegible]
Chr12	8078381	A	AAT	Chr12_80783A_AAT	INDEL	237.37	1	[illegible]
Chr12	8078381	A	AAT	Chr12_80783A_AAT	INDEL	237.37	1	[illegible]
Chr12	8195075	C	CAT	Chr12_81950C_CAT	INDEL	2680.82	0	[illegible]
Chr12	8195075	C	CAT	Chr12_81950C_CAT	INDEL	2680.82	0	[illegible]
Chr12	8195075	C	CAT	Chr12_81950C_CAT	INDEL	2680.82	0	[illegible]
Chr12	8195075	C	CAT	Chr12_81950C_CAT	INDEL	2680.82	0	[illegible]
Chr12	8195075	C	CAT	Chr12_81950C_CAT	INDEL	2680.82	0	[illegible]
Chr12	8195075	C	CAT	Chr12_81950C_CAT	INDEL	2680.82	0	[illegible]
Chr12	8512723	G	A	Chr12_85127G_A	SNP	511.41	0	[illegible]
Chr12	9904689	A	AAT	Chr12_99046A_AAT	INDEL	180.37	0	[illegible]
Chr12	9904689	A	AAT	Chr12_99046A_AAT	INDEL	180.37	0	[illegible]
Chr12	9904689	A	AAT	Chr12_99046A_AAT	INDEL	180.37	0	[illegible]
Chr12	9904689	A	AAT	Chr12_99046A_AAT	INDEL	180.37	0	[illegible]
Chr12	9904689	A	AAT	Chr12_99046A_AAT	INDEL	180.37	0	[illegible]
Chr12	11925737	C	T	Chr12_11925C_T	SNP	853.41	0	[illegible]
Chr12	11925737	C	T	Chr12_11925C_T	SNP	853.41	0	[illegible]
Chr12	11925737	C	T	Chr12_11925C_T	SNP	853.41	0	[illegible]
Chr12	11925737	C	T	Chr12_11925C_T	SNP	853.41	0	[illegible]
Chr12	11925737	C	T	Chr12_11925C_T	SNP	853.41	0	[illegible]
Chr12	11925737	C	T	Chr12_11925C_T	SNP	853.41	0	[illegible]
Chr12	11925737	C	T	Chr12_11925C_T	SNP	853.41	0	[illegible]
Chr12	12462655	C	CAT	Chr12_12462C_CAT	INDEL	422.37	0	[illegible]
Chr12	12462655	C	CAT	Chr12_12462C_CAT	INDEL	422.37	0	[illegible]
Chr12	12462655	C	CAT	Chr12_12462C_CAT	INDEL	422.37	0	[illegible]
Chr12	12462655	C	CAT	Chr12_12462C_CAT	INDEL	422.37	0	[illegible]
Chr12	12504793	CTA	C	Chr12_12504CTA_C	INDEL	133.37	1	[illegible]
Chr12	13562992	G	GAT	Chr12_13562G_GAT	INDEL	366.37	0	[illegible]
Chr12	13562992	G	GAT	Chr12_13562G_GAT	INDEL	366.37	0	[illegible]
Chr12	13562992	G	GAT	Chr12_13562G_GAT	INDEL	366.37	0	[illegible]
Chr12	13847800	G	A	Chr12_13847G_A	SNP	338.41	0	[illegible]
Chr12	13847800	G	A	Chr12_13847G_A	SNP	338.41	0	[illegible]
Chr12	13925642	A	G	Chr12_13925A_G	SNP	251.41	0	[illegible]
Chr12	16266255	CAT	C	Chr12_16266CAT_C	INDEL	1338.5	1	[illegible]
Chr12	16684987	G	C	Chr12_16684G_C	SNP	448.41	0	[illegible]
Chr13	356133	A	G	Chr13_35613A_G	SNP	200.41	0	[illegible]
Chr13	356175	A	G	Chr13_35617A_G	SNP	292.41	0	[illegible]
Chr13	744815	G	GAT	Chr13_74481G_GAT	INDEL	180.37	1	[illegible]
Chr13	744815	G	GAT	Chr13_74481G_GAT	INDEL	180.37	1	[illegible]
Chr13	744815	G	GAT	Chr13_74481G_GAT	INDEL	180.37	1	[illegible]
Chr13	744815	G	GAT	Chr13_74481G_GAT	INDEL	180.37	1	[illegible]
Chr13	744815	G	GAT	Chr13_74481G_GAT	INDEL	180.37	1	[illegible]
Chr13	2227619	C	A	Chr13_22276C_A	SNP	454.41	0	[illegible]
Chr13	2973583	C	A	Chr13_29735C_A	SNP	736.41	0	[illegible]
Chr13	5543281	C	CTA	Chr13_55432C_CTA	INDEL	167.37	0	[illegible]
Chr13	5543281	C	CTA	Chr13_55432C_CTA	INDEL	167.37	0	[illegible]
Chr13	5543281	C	CTA	Chr13_55432C_CTA	INDEL	167.37	0	[illegible]
Chr13	5543281	C	CTA	Chr13_55432C_CTA	INDEL	167.37	0	[illegible]
Chr13	8683448	G	A	Chr13_86834G_A	SNP	639.62	0	[illegible]
Chr13	8750287	A	G	Chr13_87502A_G	SNP	1136.41	0	[illegible]
Chr13	11046810	C	T	Chr13_11046C_T	SNP	142.41	0	[illegible]
Chr13	11987612	C	T	Chr13_11987C_T	SNP	506.41	0	[illegible]
Chr13	12620517	A	T	Chr13_12620A_T	SNP	1866.41	0	[illegible]
Chr13	12620517	A	T	Chr13_12620A_T	SNP	1866.41	0	[illegible]
Chr13	12620517	A	T	Chr13_12620A_T	SNP	1866.41	0	[illegible]
Chr13	12620517	A	T	Chr13_12620A_T	SNP	1866.41	0	[illegible]
Chr13	12620517	A	T	Chr13_12620A_T	SNP	1866.41	0	[illegible]
Chr13	12620517	A	T	Chr13_12620A_T	SNP	1866.41	0	[illegible]
Chr13	12620517	A	T	Chr13_12620A_T	SNP	1866.41	0	[illegible]
Chr13	13765314	C	A	Chr13_13765C_A	SNP	413.41	0	[illegible]
Chr13	13973272	C	CAT	Chr13_13973C_CAT	INDEL	678.37	0	[illegible]
Chr13	13973272	C	CAT	Chr13_13973C_CAT	INDEL	678.37	0	[illegible]
Chr13	13973272	C	CAT	Chr13_13973C_CAT	INDEL	678.37	0	[illegible]
Chr13	13973272	C	CAT	Chr13_13973C_CAT	INDEL	678.37	0	[illegible]
Chr13	13973272	C	CAT	Chr13_13973C_CAT	INDEL	678.37	0	[illegible]
Chr13	13973272	C	CAT	Chr13_13973C_CAT	INDEL	678.37	0	[illegible]
Chr13	13973272	C	CAT	Chr13_13973C_CAT	INDEL	678.37	0	[illegible]
Chr13	15378010	G	GTA	Chr13_15378G_GTA	INDEL	112.37	0	[illegible]
Chr13	15378010	G	GTA	Chr13_15378G_GTA	INDEL	112.37	0	[illegible]
Chr13	15378010	G	GTA	Chr13_15378G_GTA	INDEL	112.37	0	[illegible]
Chr13	16921465	AAT	A	Chr13_16921AAT_A	INDEL	346.37	0	[illegible]
Chr13	16921465	AAT	A	Chr13_16921AAT_A	INDEL	346.37	0	[illegible]
Chr13	16921465	AAT	A	Chr13_16921AAT_A	INDEL	346.37	0	[illegible]
Chr13	16921465	AAT	A	Chr13_16921AAT_A	INDEL	346.37	0	[illegible]
Chr13	16921465	AAT	A	Chr13_16921AAT_A	INDEL	346.37	0	[illegible]
Chr14	623158	G	A	Chr14_62315G_A	SNP	489.41	0	[illegible]
Chr14	824852	A	AAT	Chr14_82485A_AAT	INDEL	99.37	0	[illegible]
Chr14	3958333	G	C	Chr14_39583G_C	SNP	601.41	0	[illegible]
Chr14	5108834	G	GTA	Chr14_51088G_GTA	INDEL	390.37	0	[illegible]
Chr14	5108834	G	GTA	Chr14_51088G_GTA	INDEL	390.37	0	[illegible]
Chr14	5108834	G	GTA	Chr14_51088G_GTA	INDEL	390.37	0	[illegible]
Chr14	5108834	G	GTA	Chr14_51088G_GTA	INDEL	390.37	0	[illegible]
Chr14	5108834	G	GTA	Chr14_51088G_GTA	INDEL	390.37	0	[illegible]
Chr14	5108834	G	GTA	Chr14_51088G_GTA	INDEL	390.37	0	[illegible]
Chr14	7323520	AAT	A	Chr14_73235AAT_A	INDEL	331.37	1	[illegible]
Chr14	7323520	AAT	A	Chr14_73235AAT_A	INDEL	331.37	1	[illegible]
Chr14	7323520	AAT	A	Chr14_73235AAT_A	INDEL	331.37	1	[illegible]

Chr14	7323520	AAT	A	Chr14_73235	AAT_A	INDEL	331.37	1	12	0	0	0	1	1	2	0	0	1	0	0	0	23	12	15	18	13	21	28	23	22	22	12	[illegible]	[illegible]	[illegible]	[illegible]	[illegible]	[illegible]	[illegible]
Chr14	7323520	AAT	A	Chr14_73235	AAT_A	INDEL	331.37	1	12	0	0	0	1	1	2	0	0	1	0	0	0	23	12	15	18	13	21	28	23	22	22	12	[illegible]	[illegible]	[illegible]	[illegible]	[illegible]	[illegible]	[illegible]
Chr14	7323520	AAT	A	Chr14_73235	AAT_A	INDEL	331.37	1	12	0	0	0	1	1	2	0	0	1	0	0	0	23	12	15	18	13	21	28	23	22	22	12	[illegible]	[illegible]	[illegible]	[illegible]	[illegible]	[illegible]	[illegible]
Chr14	7323520	AAT	A	Chr14_73235	AAT_A	INDEL	331.37	1	12	0	0	0	1	1	2	0	0	1	0	0	0	23	12	15	18	13	21	28	23	22	22	12	[illegible]	[illegible]	[illegible]	[illegible]	[illegible]	[illegible]	[illegible]
Chr14	7323520	AAT	A	Chr14_73235	AAT_A	INDEL	331.37	1	12	0	0	0	1	1	2	0	0	1	0	0	0	23	12	15	18	13	21	28	23	22	22	12	[illegible]	[illegible]	[illegible]	[illegible]	[illegible]	[illegible]	[illegible]
Chr14	9749239	A	AAT	Chr14_97492	A_AAT	INDEL	447.37	0	0	0	0	0	0	16	1	0	0	0	0	0	0	19	14	7	16	16	18	16	18	10	10	7	[illegible]	[illegible]	[illegible]	[illegible]	[illegible]	[illegible]	[illegible]
Chr14	9749239	A	AAT	Chr14_97492	A_AAT	INDEL	447.37	0	0	0	0	0	0	16	1	0	0	0	0	0	0	19	14	7	16	16	18	16	18	10	10	7	[illegible]	[illegible]	[illegible]	[illegible]	[illegible]	[illegible]	[illegible]
Chr14	9749239	A	AAT	Chr14_97492	A_AAT	INDEL	447.37	0	0	0	0	0	0	16	1	0	0	0	0	0	0	19	14	7	16	16	18	16	18	10	10	7	[illegible]	[illegible]	[illegible]	[illegible]	[illegible]	[illegible]	[illegible]
Chr14	9999062	G	T	Chr14_99990	G_T	SNP	422.41	0	0	0	0	0	0	0	0	0	0	0	0	13	0	18	14	20	27	23	26	34	21	23	22	16	[illegible]	[illegible]	[illegible]	[illegible]	[illegible]	[illegible]	[illegible]
Chr14	9999062	G	T	Chr14_99990	G_T	SNP	422.41	0	0	0	0	0	0	0	0	0	0	0	0	13	0	18	14	20	27	23	26	34	21	23	22	16	[illegible]	[illegible]	[illegible]	[illegible]	[illegible]	[illegible]	[illegible]
Chr14	9999062	G	T	Chr14_99990	G_T	SNP	422.41	0	0	0	0	0	0	0	0	0	0	0	0	13	0	18	14	20	27	23	26	34	21	23	22	16	[illegible]	[illegible]	[illegible]	[illegible]	[illegible]	[illegible]	[illegible]
Chr14	9999062	G	T	Chr14_99990	G_T	SNP	422.41	0	0	0	0	0	0	0	0	0	0	0	0	13	0	18	14	20	27	23	26	34	21	23	22	16	[illegible]	[illegible]	[illegible]	[illegible]	[illegible]	[illegible]	[illegible]
Chr14	9999062	G	T	Chr14_99990	G_T	SNP	422.41	0	0	0	0	0	0	0	0	0	0	0	0	13	0	18	14	20	27	23	26	34	21	23	22	16	[illegible]	[illegible]	[illegible]	[illegible]	[illegible]	[illegible]	[illegible]
Chr14	9999062	G	T	Chr14_99990	G_T	SNP	422.41	0	0	0	0	0	0	0	0	0	0	0	0	13	0	18	14	20	27	23	26	34	21	23	22	16	[illegible]	[illegible]	[illegible]	[illegible]	[illegible]	[illegible]	[illegible]
Chr14	9999062	G	T	Chr14_99990	G_T	SNP	422.41	0	0	0	0	0	0	0	0	0	0	0	0	13	0	18	14	20	27	23	26	34	21	23	22	16	[illegible]	[illegible]	[illegible]	[illegible]	[illegible]	[illegible]	[illegible]
Chr14	9999062	G	T	Chr14_99990	G_T	SNP	422.41	0	0	0	0	0	0	0	0	0	0	0	0	13	0	18	14	20	27	23	26	34	21	23	22	16	[illegible]	[illegible]	[illegible]	[illegible]	[illegible]	[illegible]	[illegible]
Chr14	9999062	G	T	Chr14_99990	G_T	SNP	422.41	0	0	0	0	0	0	0	0	0	0	0	0	13	0	18	14	20	27	23	26	34	21	23	22	16	[illegible]	[illegible]	[illegible]	[illegible]	[illegible]	[illegible]	[illegible]
Chr14	9999062	G	T	Chr14_99990	G_T	SNP	422.41	0	0	0	0	0	0	0	0	0	0	0	0	13	0	18	14	20	27	23	26	34	21	23	22	16	[illegible]	[illegible]	[illegible]	[illegible]	[illegible]	[illegible]	[illegible]
Chr14	9999062	G	T	Chr14_99990	G_T	SNP	422.41	0	0	0	0	0	0	0	0	0	0	0	0	13	0	18	14	20	27	23	26	34	21	23	22	16	[illegible]	[illegible]	[illegible]	[illegible]	[illegible]	[illegible]	[illegible]
Chr14	10451855	T	TTA	Chr14_10451	T_TTA	INDEL	117.58	0	0	6	0	0	0	0	0	1	0	1	1	NA	NA	13	6	6	10	10	10	11	9	4	6	9	[illegible]	[illegible]	[illegible]	[illegible]	[illegible]	[illegible]	[illegible]
Chr14	11360033	C	CAT	Chr14_11360	C_CAT	INDEL	151.37	0	0	0	1	0	0	0	0	0	2	1	0	1	6	27	12	13	22	16	31	27	21	16	15	10	20	10	6	0	0	0	[illegible]
Chr14	11360033	C	CAT	Chr14_11360	C_CAT	INDEL	151.37	0	0	0	1	0	0	0	0	0	2	1	0	1	6	27	12	13	22	16	31	27	21	16	15	10	20	10	6	0	0	0	[illegible]
Chr14	11360033	C	CAT	Chr14_11360	C_CAT	INDEL	151.37	0	0	0	1	0	0	0	0	0	2	1	0	1	6	27	12	13	22	16	31	27	21	16	15	10	20	10	6	0	0	0	[illegible]
Chr14	12797646	TGTTCACGA	T	Chr14_12797	TGTTCACGA_	INDEL	448.37	0	11	0	0	0	0	0	0	0	0	0	0	0	0	25	11	11	24	17	18	33	16	17	31	21	12	15	10	0	1	0	[illegible]
Chr14	12797646	TGTTCACGA	T	Chr14_12797	TGTTCACGA_	INDEL	448.37	0	11	0	0	0	0	0	0	0	0	0	0	0	0	25	11	11	24	17	18	33	16	17	31	21	12	15	10	0	1	0	[illegible]
Chr14	12797646	TGTTCACGA	T	Chr14_12797	TGTTCACGA_	INDEL	448.37	0	11	0	0	0	0	0	0	0	0	0	0	0	0	25	11	11	24	17	18	33	16	17	31	21	12	15	10	0	1	0	[illegible]
Chr14	12797646	TGTTCACGA	T	Chr14_12797	TGTTCACGA_	INDEL	448.37	0	11	0	0	0	0	0	0	0	0	0	0	0	0	25	11	11	24	17	18	33	16	17	31	21	12	15	10	0	1	0	[illegible]
Chr14	12797646	TGTTCACGA	T	Chr14_12797	TGTTCACGA_	INDEL	448.37	0	11	0	0	0	0	0	0	0	0	0	0	0	0	25	11	11	24	17	18	33	16	17	31	21	12	15	10	0	1	0	[illegible]
Chr14	12797646	TGTTCACGA	T	Chr14_12797	TGTTCACGA_	INDEL	448.37	0	11	0	0	0	0	0	0	0	0	0	0	0	0	25	11	11	24	17	18	33	16	17	31	21	12	15	10	0	1	0	[illegible]
Chr14	12797646	TGTTCACGA	T	Chr14_12797	TGTTCACGA_	INDEL	448.37	0	11	0	0	0	0	0	0	0	0	0	0	0	0	25	11	11	24	17	18	33	16	17	31	21	12	15	10	0	1	0	[illegible]
Chr14	12797646	TGTTCACGA	T	Chr14_12797	TGTTCACGA_	INDEL	448.37	0	11	0	0	0	0	0	0	0	0	0	0	0	0	25	11	11	24	17	18	33	16	17	31	21	12	15	10	0	1	0	[illegible]
Chr14	13261026	T	TTA	Chr14_13261	T_TTA	INDEL	200.37	1	1	0	0	0	1	1	1	0	2	0	0	0	9	13	10	8	12	11	11	14	11	7	10	4	6	9	9	0	0	0	[illegible]
Chr14	13513858	GTATATA	G	Chr14_13513	GTATATA_G	INDEL	313.37	0	0	0	0	0	0	0	0	0	0	0	0	0	8	29	18	23	23	20	33	30	19	13	14	9	9	11	8	0	0	0	[illegible]
Chr14	13513858	GTATATA	G	Chr14_13513	GTATATA_G	INDEL	313.37	0	0	0	0	0	0	0	0	0	0	0	0	0	8	29	18	23	23	20	33	30	19	13	14	9	9	11	8	0	0	0	[illegible]
Chr14	13513858	GTATATA	G	Chr14_13513	GTATATA_G	INDEL	313.37	0	0	0	0	0	0	0	0	0	0	0	0	0	8	29	18	23	23	20	33	30	19	13	14	9	9	11	8	0	0	0	[illegible]
Chr14	13513858	GTATATA	G	Chr14_13513	GTATATA_G	INDEL	313.37	0	0	0	0	0	0	0	0	0	0	0	0	0	8	29	18	23	23	20	33	30	19	13	14	9	9	11	8	0	0	0	[illegible]
Chr15	2685936	A	AAT	Chr15_26859	A_AAT	INDEL	105.37	0	0	0	0	1	0	0	0	0	0	0	1	5	0	17	9	9	19	18	24	22	17	23	15	8	13	5	9	0	0	0	[illegible]
Chr15	2685936	A	AAT	Chr15_26859	A_AAT	INDEL	105.37	0	0	0	0	1	0	0	0	0	0	0	1	5	0	17	9	9	19	18	24	22	17	23	15	8	13	5	9	0	0	0	[illegible]
Chr15	2685936	A	AAT	Chr15_26859	A_AAT	INDEL	105.37	0	0	0	0	1	0	0	0	0	0	0	1	5	0	17	9	9	19	18	24	22	17	23	15	8	13	5	9	0	0	0	[illegible]
Chr15	2685936	A	AAT	Chr15_26859	A_AAT	INDEL	105.37	0	0	0	0	1	0	0	0	0	0	0	1	5	0	17	9	9	19	18	24	22	17	23	15	8	13	5	9	0	0	0	[illegible]
Chr15	2685936	A	AAT	Chr15_26859	A_AAT	INDEL	105.37	0	0	0	0	1	0	0	0	0	0	0	1	5	0	17	9	9	19	18	24	22	17	23	15	8	13	5	9	0	0	0	[illegible]
Chr15	2685936	A	AAT	Chr15_26859	A_AAT	INDEL	105.37	0	0	0	0	1	0	0	0	0	0	0	1	5	0	17	9	9	19	18	24	22	17	23	15	8	13	5	9	0	0	0	[illegible]
Chr15	2685936	A	AAT	Chr15_26859	A_AAT	INDEL	105.37	0	0	0	0	1	0	0	0	0	0	0	1	5	0	17	9	9	19	18	24	22	17	23	15	8	13	5	9	0	0	0	[illegible]
Chr15	2685936	A	AAT	Chr15_26859	A_AAT	INDEL	105.37	0	0	0	0	1	0	0	0	0	0	0	1	5	0	17	9	9	19	18	24	22	17	23	15	8	13	5	9	0	0	0	[illegible]
Chr15	2685936	A	AAT	Chr15_26859	A_AAT	INDEL	105.37	0	0	0	0	1	0	0	0	0	0	0	1	5	0	17	9	9	19	18	24	22	17	23	15	8	13	5	9	0	0	0	[illegible]
Chr15	3343023	A	AAT	Chr15_33430	A_AAT	INDEL	111.37	0	0	0	0	0	0	0	0	1	0	1	0	0	5	19	11	13	16	23	16	25	19	21	11	8	20	8	5	0	0	0	[illegible]
Chr15	3343023	A	AAT	Chr15_33430	A_AAT	INDEL	111.37	0	0	0	0	0	0	0	0	1	0	1	0	0	5	19	11	13	16	23	16	25	19	21	11	8	20	8	5	0	0	0	[illegible]
Chr15	3343023	A	AAT	Chr15_33430	A_AAT	INDEL	111.37	0	0	0	0	0	0	0	0	1	0	1	0	0	5	19	11	13	16	23	16	25	19	21	11	8	20	8	5	0	0	0	[illegible]
Chr15	3343023	A	AAT	Chr15_33430	A_AAT	INDEL	111.37	0	0	0	0	0	0	0	0	1	0	1	0	0	5	19	11	13	16	23	16	25	19	21	11	8	20	8	5	0	0	0	[illegible]
Chr15	3343023	A	AAT	Chr15_33430	A_AAT	INDEL	111.37	0	0	0	0	0	0	0	0	1	0	1	0	0	5	19	11	13	16	23	16	25	19	21	11	8	20	8	5	0	0	0	[illegible]
Chr15	3343023	A	AAT	Chr15_33430	A_AAT	INDEL	111.37	0	0	0	0	0	0	0	0	1	0	1	0	0	5	19	11	13	16	23	16	25	19	21	11	8	20	8	5	0	0	0	[illegible]
Chr15	3343023	A	AAT	Chr15_33430	A_AAT	INDEL	111.37	0	0	0	0	0	0	0	0	1	0	1	0	0	5	19	11	13	16	23	16	25	19	21	11	8	20	8	5	0	0	0	[illegible]
Chr15	3343023	A	AAT	Chr15_33430	A_AAT	INDEL	111.37	0	0	0	0	0	0	0	0	1	0	1	0	0	5	19	11	13	16	23	16	25	19	21	11	8	20	8	5	0	0	0	[illegible]
Chr15	3343023	A	AAT	Chr15_33430	A_AAT	INDEL	111.37	0	0	0	0	0	0	0	0	1	0	1	0	0	5	19	11	13	16	23	16	25	19	21	11	8	20	8	5	0	0	0	[illegible]
Chr15	3343023	A	AAT	Chr15_33430	A_AAT	INDEL	111.37	0	0	0	0	0	0	0	0	1	0	1	0	0	5	19	11	13	16	23	16	25	19	21	11	8	20	8	5	0	0	0	[illegible]
Chr15	3343023	A	AAT	Chr15_33430	A_AAT	INDEL	111.37	0	0	0	0	0	0	0	0	1	0	1	0	0	5	19	11	13	16	23	16	25	19	21	11	8	20	8	5	0	0	0	[illegible]
Chr15	3473985	A	AAT	Chr15_34739	A_AAT	INDEL	367.37	6	13	0	0	0	0	0	0	0	1	0	2	0	0	22	13	14	14	11	19	29	19	20	11	10	16	12	9	0.273	1	0	[illegible]
Chr15	3473985	A	AAT	Chr15_34739	A_AAT	INDEL	367.37	6	13	0	0	0	0	0	0	0	1	0	2	0	0	22	13	14	14	11	19	29	19	20	11	10	16	12	9	0.273	1	0	[illegible]
Chr15	3473985	A	AAT	Chr15_34739	A_AAT	INDEL	367.37	6	13	0	0	0	0	0	0	0	1	0	2	0	0	22	13	14	14	11	19	29	19	20	11	10	16	12	9	0.273	1	0	[illegible]
Chr15	3473985	A	AAT	Chr15_34739	A_AAT	INDEL	367.37	6	13	0	0	0	0	0	0	0	1	0	2	0	0	22	13	14	14	11	19	29	19	20	11	10	16	12	9	0.273	1	0	[illegible]
Chr15	3473985	A	AAT	Chr15_34739	A_AAT	INDEL	367.37	6	13	0	0	0	0	0	0	0	1	0	2	0	0	22	13	14	14	11	19	29	19	20	11	10	16	12	9	0.273	1	0	[illegible]
Chr15	3473985	A	AAT	Chr15_34739	A_AAT	INDEL	367.37	6	13	0	0	0	0	0	0	0	1	0	2	0	0	22	13	14	14	11	19	29	19	20	11	10	16	12	9	0.273	1	0	[illegible]
Chr15	3473985	A	AAT	Chr15_34739	A_AAT	INDEL	367.37	6	13	0	0	0	0	0	0	0	1	0	2	0	0	22	13	14	14	11	19	29	19	20	11	10	16	12	9	0.273	1	0	[illegible]
Chr15	3473985	A	AAT	Chr15_34739	A_AAT	INDEL	367.37	6	13	0	0	0	0	0	0	0	1	0	2	0	0	22	13	14	14	11	19	29	19	20	11	10	16	12	9	0.273	1	0	[illegible]
Chr15	7085178	C	T	Chr15_70851	C_T	SNP	851.41	0	0	0	0	1	20	0	0	0	0	0	0	0	0	36	14	20	27	20	20	30	26	22	28	10	16	10	8	0	0	0	[illegible]
Chr15	8082036	AAT	A	Chr15_80820	AAT_A	INDEL	5538.5	0	0	0	0	0	24	0	0	0	0	0	0	0	NA	23	7	10	18	9	24	16	10	12	13	9	5	7	NA		0	0	[illegible]
Chr15	8082036	AAT	A	Chr15_80820	AAT_A	INDEL	5538.5	0	0	0	0	0	24	0	0	0	0	0	0	0	NA	23	7	10	18	9	24	16	10	12	13	9	5	7	NA		0	0	[illegible]
Chr15	8082036	AAT	A	Chr15_80820	AAT_A	INDEL	5538.5	0	0	0	0	0	24	0	0	0	0	0	0	0	NA	23	7	10	18	9	24	16	10	12	13	9	5	7	NA		0	0	[illegible]
Chr15	8082036	AAT	A	Chr15_80820	AAT_A	INDEL	5538.5	0	0	0	0	0	24	0	0	0	0	0	0	0	NA	23	7	10	18	9	24	16	10	12	13	9	5	7	NA		0	0	[illegible]
Chr15	8082036	AAT	A	Chr15_80820	AAT_A	INDEL	5538.5	0	0	0	0	0	24	0	0	0	0	0	0	0	NA	23	7	10	18	9	24	16	10	12	13	9	5	7	NA		0	0	[illegible]
Chr15	10315454	C	T	Chr15_10315	C_T	SNP	1000.41	0	0	0	0	2	0	0	25	0	0	0	0	0	2	31	20	16	18	18	26	23	26	18	21	13	30	13	12		0	0	[illegible]
Chr15	10501016	G	GAT	Chr15_10501	G_GAT	INDEL	222.37	0	0	0	1	0	0	0	0	1	0	0	0	8		27	18	11	21	22	16	23	18	14	21	14	20	8	4	0	0	0	[illegible]
Chr15	11425998	T	TTA	Chr15_11425	T_TTA	INDEL	512.37	2	1	0	0	0	18	0	0	0	0	0	0	0	0	13	14	7	16	12	18	20	9	12	15	4	10	6	6	0.154	0	0	[illegible]
Chr15	13362617	A	AAT	Chr15_13362	A_AAT	INDEL	466.37	2	0	0	0	2	1	17	1	1	0	0	0	0	0	17	11	10	18	16	20	17	16	17	16	11	10	4	11	0.118	0	0	[illegible]
Chr15	13362617	A	AAT	Chr15_13362	A_AAT	INDEL	466.37	2	0	0	0	2	1	17	1	1	0	0	0	0	0	17	11	10	18	16	20	17	16	17	16	11	10	4	11	0.118	0	0	[illegible]
Chr15	13362617	A	AAT	Chr15_13362	A_AAT	INDEL	466.37	2	0	0	0	2	1	17	1	1	0	0	0	0	0	17	11	10	18	16	20	17	16	17	16	11	10	4	11	0.118	0	0	[illegible]
Chr15	13362617	A	AAT	Chr15_13362	A_AAT	INDEL	466.37	2	0	0	0	2	1	17	1	1	0	0	0	0	0	17	11	10	18	16	20	17	16	17	16	11	10	4	11	0.118	0	0	[illegible]
Chr15	13362617	A	AAT	Chr15_13362	A_AAT	INDEL	466.37	2	0	0	0	2	1	17	1	1	0	0	0	0	0	17	11	10	18	16	20	17	16	17	16	11	10	4	11	0.118	0	0	[illegible]
Chr16	181719	G	GTA	Chr16_18171	G_GTA	INDEL	86.37	0	0	0	0	0	0	0	0	0	1	0	4	0	0	22	11	13	18	14	18	14	18	14	9	7	4	5	4	0	0	0	[illegible]
Chr16	181719	G	GTA	Chr16_18171	G_GTA	INDEL	86.37	0	0	0	0	0	0	0	0	0	1	0	4	0	0	22	11	13	18	14	18	14	18	14	9	7	4	5	4	0	0	0	[illegible]
Chr16	181719	G	GTA	Chr16_18171	G_GTA	INDEL	86.37	0	0	0	0	0	0	0	0	0	1	0	4	0	0	22	11	13	18	14	18	14	18	14	9	7	4	5	4	0	0	0	[illegible]
Chr16	181719	G	GTA	Chr16_18171	G_GTA	INDEL	86.37	0	0	0	0	0	0	0	0	0	1	0	4	0	0	22	11	13	18	14	18	14	18	14	9	7	4	5	4	0	0	0	[illegible]
Chr16	181719	G	GTA	Chr16_18171	G_GTA	INDEL	86.37	0	0	0	0	0	0	0	0	0	1	0	4	0	0	22	11	13	18	14	18	14	18	14	9	7	4	5	4	0	0	0	[illegible]
Chr16	181719	G	GTA	Chr16_18171	G_GTA	INDEL	86.37	0	0	0	0	0	0	0	0	0	1	0	4	0	0	22	11	13	18	14	18	14	18	14	9	7	4	5	4	0	0	0	[illegible]
Chr16	677406	TAAAAAAAT	T	Chr16_67740	TAAAAAAAT	INDEL	70.37	0	0	1	0	5	1	0	2	0	2	0	0	NA	1	8	7	4	10	6	5	9	10	8	9	5	7	NA	4	0	0	0	[illegible]
Chr16	677406	TAAAAAAAT	T	Chr16_67740	TAAAAAAAT	INDEL	70.37	0	0	1	0	5	1	0	2	0	2	0	0	NA	1	8	7	4	10	6	5	9	10	8	9	5	7	NA	4	0	0	0	[illegible]
[illegible]	[illegible]	[illegible]	[illegible]	[illegible]	[illegible]	[illegible]	[illegible]	[illegible]	[illegible]	[illegible]	[illegible]	[illegible]	[illegible]	[illegible]	[illegible]	[illegible]	[illegible]	[illegible]	[illegible]	[illegible]	[illegible]	[illegible]	[illegible]	[illegible]	[illegible]	[illegible]	[illegible]	[illegible]	[illegible]	[illegible]	[illegible]	[illegible]	[illegible]	[illegible]	[illegible]	[illegible]	[illegible]	[illegible]	[illegible]

Chr18	15084058	C	CTA	Chr18_15084C_CTA	INDEL	754.37	1	0	2	0	0	1	25	0	2	0	0	0	0	1
Chr18	15084058	C	CTA	Chr18_15084C_CTA	INDEL	754.37	1	0	2	0	0	1	25	0	2	0	0	0	0	1
Chr18	15084058	C	CTA	Chr18_15084C_CTA	INDEL	754.37	1	0	2	0	0	1	25	0	2	0	0	0	0	1
Chr18	15084058	C	CTA	Chr18_15084C_CTA	INDEL	754.37	1	0	2	0	0	1	25	0	2	0	0	0	0	1
Chr18	15084058	C	CTA	Chr18_15084C_CTA	INDEL	754.37	1	0	2	0	0	1	25	0	2	0	0	0	0	1
Chr19	3245332	T	TTA	Chr19_32453T_TTA	INDEL	461.37	0	1	1	1	1	2	19	1	0	0	NA	0	0	0
Chr19	3245332	T	TTA	Chr19_32453T_TTA	INDEL	461.37	0	1	1	1	1	2	19	1	0	0	NA	0	0	0
Chr19	3245332	T	TTA	Chr19_32453T_TTA	INDEL	461.37	0	1	1	1	1	2	19	1	0	0	NA	0	0	0
Chr19	3245332	T	TTA	Chr19_32453T_TTA	INDEL	461.37	0	1	1	1	1	2	19	1	0	0	NA	0	0	0
Chr19	3245332	T	TTA	Chr19_32453T_TTA	INDEL	461.37	0	1	1	1	1	2	19	1	0	0	NA	0	0	0
Chr19	3245332	T	TTA	Chr19_32453T_TTA	INDEL	461.37	0	1	1	1	1	2	19	1	0	0	NA	0	0	0
Chr19	4236598	T	TTA	Chr19_42365T_TTA	INDEL	316.37	1	0	0	1	0	0	14	1	1	0	1	0	1	NA
Chr19	4236598	T	TTA	Chr19_42365T_TTA	INDEL	316.37	1	0	0	1	0	0	14	1	1	0	1	0	1	NA
Chr19	4236598	T	TTA	Chr19_42365T_TTA	INDEL	316.37	1	0	0	1	0	0	14	1	1	0	1	0	1	NA
Chr19	4236598	T	TTA	Chr19_42365T_TTA	INDEL	316.37	1	0	0	1	0	0	14	1	1	0	1	0	1	NA
Chr19	4236598	T	TTA	Chr19_42365T_TTA	INDEL	316.37	1	0	0	1	0	0	14	1	1	0	1	0	1	NA
Chr19	4639546	G	GAT	Chr19_46395G_GAT	INDEL	370.37	0	0	0	0	14	1	0	2	0	0	0	0	0	0
Chr19	5097814	G	A	Chr19_50978G_A	SNP	933.41	0	0	0	0	0	0	34	0	0	0	0	0	0	0
Chr19	5097814	G	A	Chr19_50978G_A	SNP	933.41	0	0	0	0	0	0	34	0	0	0	0	0	0	0
Chr19	8165516	G	A	Chr19_81655G_A	SNP	623.41	0	16	0	0	0	0	0	0	0	0	0	0	0	0
Chr19	8165516	G	A	Chr19_81655G_A	SNP	623.41	0	16	0	0	0	0	0	0	0	0	0	0	0	0
Chr19	8165516	G	A	Chr19_81655G_A	SNP	623.41	0	16	0	0	0	0	0	0	0	0	0	0	0	0
Chr19	8611737	A	AAT	Chr19_86117A_AAT	INDEL	345.37	1	0	0	0	14	0	0	2	0	0	0	0	0	NA
Chr19	8611737	A	AAT	Chr19_86117A_AAT	INDEL	345.37	1	0	0	0	14	0	0	2	0	0	0	0	0	NA
[illegible]																				

Chr24	7245586	G	A	Chr24_72455	G_A	SNP	361.41	0	0	9	0	0	0	0	0	0	0	0	0	NA	0	24	16	9	20	16	18	28	11	18	14	18	17	NA	10	0	0	1	0	0	0	0	0	0	0	0	0	NA	0	1	1	1	0	0	0	1	intergenic_re	MODIFIER	Pp3c24_1038	Pp3c24_1038	intergenic_re	Pp3c24_1038	NA	NA	n.7245586G	NA	NA	NA	NA	NA
Chr24	7884750	G	A	Chr24_78847	G_A	SNP	815.41	0	0	0	0	0	0	0	0	0	0	0	23	0	0	27	15	12	21	21	25	31	22	21	23	14	23	13	11	0	0	0	0	0	0	0	0	0	0	0	1	0	0	0	1	1	0	1	0	0	intergenic_re	MODIFIER	Pp3c24_1130	Pp3c24_1130	intergenic_re	Pp3c24_1130	NA	NA	n.7884750G	NA	NA	NA	NA	NA
Chr24	8741521	A	AAT	Chr24_87415	A_AAT	INDEL	499.37	1	0	0	0	0	0	18	0	1	0	0	1	NA	0	13	9	4	10	18	11	18	8	10	15	7	13	NA	7	0	0	0	0	0	0	1	0	0	0	0	0	NA	0	1	1	1	0	0	1	0	downstream	MODIFIER	Pp3c24_1286	Pp3c24_1286	transcript	PAC:3290916	protein_codi	NA	c.*954_*955	NA	NA	NA	NA	955
Chr24	8741521	A	AAT	Chr24_87415	A_AAT	INDEL	499.37	1	0	0	0	0	0	18	0	1	0	0	1	NA	0	13	9	4	10	18	11	18	8	10	15	7	13	NA	7	0	0	0	0	0	0	1	0	0	0	0	0	NA	0	1	1	1	0	0	1	0	intergenic_re	MODIFIER	Pp3c24_1286	Pp3c24_1286	intergenic_re	Pp3c24_1286	NA	NA	n.8741521_8	NA	NA	NA	NA	NA
Chr24	10845297	T	C	Chr24_10845	T_C	SNP	322.41	0	0	0	0	0	0	0	0	0	0	0	0	0	8	14	12	15	18	15	23	28	18	8	7	13	13	5	8	0	0	0	0	0	0	0	0	0	0	0	0	0	1	0	1	1	0	1	0	0	5_prime_UT	MODIFIER	Pp3c24_1655	Pp3c24_1655	transcript	PAC:3290860	protein_codi	01-avr	c.-1066A>G	NA	NA	NA	NA	1346
Chr24	10845297	T	C	Chr24_10845	T_C	SNP	322.41	0	0	0	0	0	0	0	0	0	0	0	0	0	8	14	12	15	18	15	23	28	18	8	7	13	13	5	8	0	0	0	0	0	0	0	0	0	0	0	0	0	1	0	1	1	0	1	0	0	downstream	MODIFIER	Pp3c24_1654	Pp3c24_1654	transcript	PAC:3290871	protein_codi	NA	c.*3259T>C	NA	NA	NA	NA	3259
Chr24	10845297	T	C	Chr24_10845	T_C	SNP	322.41	0	0	0	0	0	0	0	0	0	0	0	0	0	8	14	12	15	18	15	23	28	18	8	7	13	13	5	8	0	0	0	0	0	0	0	0	0	0	0	0	0	1	0	1	1	0	1	0	0	intron_variar	MODIFIER	Pp3c24_1655	Pp3c24_1655	transcript	PAC:3290860	protein_codi	01-mars	c.-323+134A	NA	NA	NA	NA	NA
Chr24	11424159	A	G	Chr24_11424	A_G	SNP	368.41	0	0	0	0	0	0	0	0	0	0	0	0	0	10	22	5	10	26	17	29	34	22	22	23	18	17	5	10	0	0	0	0	0	0	0	0	0	0	0	0	0	1	0	1	1	0	1	0	0	intergenic_re	MODIFIER	Pp3c24_1740	Pp3c24_1740	intergenic_re	Pp3c24_1740	NA	NA	n.11424159A	NA	NA	NA	NA	NA
Chr24	12816696	G	GAT	Chr24_12816	G_GAT	INDEL	345.37	0	0	12	1	0	1	0	1	1	1	2	0	0	NA	34	11	12	12	10	23	35	18	14	16	13	8	8	NA	0	0	1	0	0	0	0	0	0	0	0.154	0	0	NA	1	1	1	1	0	0	1	downstream	MODIFIER	Pp3c24_1914	Pp3c24_1914	transcript	PAC:3290911	protein_codi	NA	c.*1342_*13	NA	NA	NA	NA	827
Chr24	12816696	G	GAT	Chr24_12816	G_GAT	INDEL	345.37	0	0	12	1	0	1	0	1	1	1	2	0	0	NA	34	11	12	12	10	23	35	18	14	16	13	8	8	NA	0	0	1	0	0	0	0	0	0	0	0.154	0	0	NA	1	1	1	1	0	0	1	downstream	MODIFIER	Pp3c24_1915	Pp3c24_1915	transcript	PAC:3290886	protein_codi	NA	c.*2034_*20	NA	NA	NA	NA	1578
Chr24	12816696	G	GAT	Chr24_12816	G_GAT	INDEL	345.37	0	0	12	1	0	1	0	1	1	1	2	0	0	NA	34	11	12	12	10	23	35	18	14	16	13	8	8	NA	0	0	1	0	0	0	0	0	0	0	0.154	0	0	NA	1	1	1	1	0	0	1	downstream	MODIFIER	Pp3c24_1914	Pp3c24_1914	transcript	PAC:3290911	protein_codi	NA	c.*1342_*13	NA	NA	NA	NA	895
Chr24	12816696	G	GAT	Chr24_12816	G_GAT	INDEL	345.37	0	0	12	1	0	1	0	1	1	1	2	0	0	NA	34	11	12	12	10	23	35	18	14	16	13	8	8	NA	0	0	1	0	0	0	0	0	0	0	0.154	0	0	NA	1	1	1	1	0	0	1	downstream	MODIFIER	Pp3c24_1915	Pp3c24_1915	transcript	PAC:3290886	protein_codi	NA	c.*2034_*20	NA	NA	NA	NA	1513
Chr24	12816696	G	GAT	Chr24_12816	G_GAT	INDEL	345.37	0	0	12	1	0	1	0	1	1	1	2	0	0	NA	34	11	12	12	10	23	35	18	14	16	13	8	8	NA	0	0	1	0	0	0	0	0	0	0	0.154	0	0	NA	1	1	1	1	0	0	1	downstream	MODIFIER	Pp3c24_1915	Pp3c24_1915	transcript	PAC:3290886	protein_codi	NA	c.*2034_*20	NA	NA	NA	NA	1528
Chr24	12816696	G	GAT	Chr24_12816	G_GAT	INDEL	345.37	0	0	12	1	0	1	0	1	1	1	2	0	0	NA	34	11	12	12	10	23	35	18	14	16	13	8	8	NA	0	0	1	0	0	0	0	0	0	0	0.154	0	0	NA	1	1	1	1	0	0	1	downstream	MODIFIER	Pp3c24_1915	Pp3c24_1915	transcript	PAC:3290886	protein_codi	NA	c.*2952_*29	NA	NA	NA	NA	1536
Chr24	12816696	G	GAT	Chr24_12816	G_GAT	INDEL	345.37	0	0	12	1	0	1	0	1	1	1	2	0	0	NA	34	11	12	12	10	23	35	18	14	16	13	8	8	NA	0	0	1	0	0	0	0	0	0	0	0.154	0	0	NA	1	1	1	1	0	0	1	downstream	MODIFIER	Pp3c24_1915	Pp3c24_1915	transcript	PAC:3290886	protein_codi	NA	c.*2952_*29	NA	NA	NA	NA	1578
Chr24	12816696	G	GAT	Chr24_12816	G_GAT	INDEL	345.37	0	0	12	1	0	1	0	1	1	1	2	0	0	NA	34	11	12	12	10	23	35	18	14	16	13	8	8	NA	0	0	1	0	0	0	0	0	0	0	0.154	0	0	NA	1	1	1	1	0	0	1	downstream	MODIFIER	Pp3c24_1915	Pp3c24_1915	transcript	PAC:3290886	protein_codi	NA	c.*2952_*29	NA	NA	NA	NA	1578
Chr24	12816696	G	GAT	Chr24_12816	G_GAT	INDEL	345.37	0	0	12	1	0	1	0	1	1	1	2	0	0	NA	34	11	12	12	10	23	35	18	14	16	13	8	8	NA	0	0	1	0	0	0	0	0	0	0	0.154	0	0	NA	1	1	1	1	0	0	1	downstream	MODIFIER	Pp3c24_1915	Pp3c24_1915	transcript	PAC:3290886	protein_codi	NA	c.*2034_*20	NA	NA	NA	NA	1578
Chr24	12816696	G	GAT	Chr24_12816	G_GAT	INDEL	345.37	0	0	12	1	0	1	0	1	1	1	2	0	0	NA	34	11	12	12	10	23	35	18	14	16	13	8	8	NA	0	0	1	0	0	0	0	0	0	0	0.154	0	0	NA	1	1	1	1	0	0	1	downstream	MODIFIER	Pp3c24_1915	Pp3c24_1915	transcript	PAC:3290887	protein_codi	NA	c.*2034_*20	NA	NA	NA	NA	1578
Chr24	12816696	G	GAT	Chr24_12816	G_GAT	INDEL	345.37	0	0	12	1	0	1	0	1	1	1	2	0	0	NA	34	11	12	12	10	23	35	18	14	16	13	8	8	NA	0	0	1	0	0	0	0	0	0	0	0.154	0	0	NA	1	1	1	1	0	0	1	intergenic_re	MODIFIER	Pp3c24_1914	Pp3c24_1914	intergenic_re	Pp3c24_1914	NA	NA	n.12816696_	NA	NA	NA	NA	NA
Chr24	13325300	T	TTA	Chr24_13325	T_TTA	INDEL	416.37	0	0	14	3	1	0	0	0	0	0	0	1	0	NA	11	14	14	23	20	15	22	9	14	20	8	7	10	NA	0	0	1	0.13	0	0	0	0	0	0	0	0	0	NA	1	1	1	1	0	0	1	upstream_ge	MODIFIER	Pp3c24_2015	Pp3c24_2015	transcript	PAC:3290978	protein_codi	NA	c.-2253_-225	NA	NA	NA	NA	1909
Chr24	13325300	T	TTA	Chr24_13325	T_TTA	INDEL	416.37	0	0	14	3	1	0	0	0	0	0	0	1	0	NA	11	14	14	23	20	15	22	9	14	20	8	7	10	NA	0	0	1	0.13	0	0	0	0	0	0	0	0	0	NA	1	1	1	1	0	0	1	upstream_ge	MODIFIER	Pp3c24_2015	Pp3c24_2015	transcript	PAC:3290978	protein_codi	NA	c.-2253_-225	NA	NA	NA	NA	2252
Chr24	13325300	T	TTA	Chr24_13325	T_TTA	INDEL	416.37	0	0	14	3	1	0	0	0	0	0	0	1	0	NA	11	14	14	23	20	15	22	9	14	20	8	7	10	NA	0	0	1	0.13	0	0	0	0	0	0	0	0	0	NA	1	1	1	1	0	0	1	downstream	MODIFIER	Pp3c24_2016	Pp3c24_2016	transcript	PAC:3290903	protein_codi	NA	c.*6322_*63	NA	NA	NA	NA	4051
Chr24	13325300	T	TTA	Chr24_13325	T_TTA	INDEL	416.37	0	0	14	3	1	0	0	0	0	0	0	1	0	NA	11	14	14	23	20	15	22	9	14	20	8	7	10	NA	0	0	1	0.13	0	0	0	0	0	0	0	0	0	NA	1	1	1	1	0	0	1	intergenic_re	MODIFIER	Pp3c24_2012	Pp3c24_2012	intergenic_re	Pp3c24_2012	NA	NA	n.13325300_	NA	NA	NA	NA	NA
Chr25	2444697	A	AAT	Chr25_24446	A_AAT	INDEL	450.37	0	0	0	0	1	0	3	17	1	0	1	0	0	NA	21	13	5	19	15	18	25	18	12	12	9	10	12	NA	0	0	0	0	0	0	0.12	0.944	0	0	0	0	0	NA	1	1	1	1	0	1	0	downstream	MODIFIER	Pp3c25_3840	Pp3c25_3840	transcript	PAC:3297954	protein_codi	NA	c.*2703_*27	NA	NA	NA	NA	1871
Chr25	2444697	A	AAT	Chr25_24446	A_AAT	INDEL	450.37	0	0	0	0	1	0	3	17	1	0	1	0	0	NA	21	13	5	19	15	18	25	18	12	12	9	10	12	NA	0	0	0	0	0	0	0.12	0.944	0	0	0	0	0	NA	1	1	1	1	0	1	0	downstream	MODIFIER	Pp3c25_3840	Pp3c25_3840	transcript	PAC:3297954	protein_codi	NA	c.*2703_*27	NA	NA	NA	NA	1865
Chr25	2444697	A	AAT	Chr25_24446	A_AAT	INDEL	450.37	0	0	0	0	1	0	3	17	1	0	1	0	0	NA	21	13	5	19	15	18	25	18	12	12	9	10	12	NA	0	0	0	0	0	0	0.12	0.944	0	0	0	0	0	NA	1	1	1	1	0	1	0	downstream	MODIFIER	Pp3c25_3840	Pp3c25_3840	transcript	PAC:3297955	protein_codi	NA	c.*2703_*27	NA	NA	NA	NA	1865
Chr25	2444697	A	AAT	Chr25_24446	A_AAT	INDEL	450.37	0	0	0	0	1	0	3	17	1	0	1	0	0	NA	21	13	5	19	15	18	25	18	12	12	9	10	12	NA	0	0	0	0	0	0	0.12	0.944	0	0	0	0	0	NA	1	1	1	1	0	1	0	intergenic_re	MODIFIER	Pp3c25_3840	Pp3c25_3840	intergenic_re	Pp3c25_3840	NA	NA	n.2444697_2	NA	NA	NA	NA	NA
Chr25	4852585	A	C	Chr25_48525	A_C	SNP	692.41	0	0	0	0	0	0	0	0	0	0	0	19	0	0	26	16	12	27	9	40	31	29	27	37	15	19	16	7	0	0	0	0	0	0	0	0	0	0	0	1	0	0	0	1	1	0	1	0	0	downstream	MODIFIER	Pp3c25_7310	Pp3c25_7310	transcript	PAC:3297914	protein_codi	NA	c.*3297T>G	NA	NA	NA	NA	2877
Chr25	4852585	A	C	Chr25_48525	A_C	SNP	692.41	0	0	0	0	0	0	0	0	0	0	0	19	0	0	26	16	12	27	9	40	31	29	27	37	15	19	16	7	0	0	0	0	0	0	0	0	0	0	0	1	0	0	0	1	1	0	1	0	0	downstream	MODIFIER	Pp3c25_7310	Pp3c25_7310	transcript	PAC:3297914	protein_codi	NA	c.*3297T>G	NA	NA	NA	NA	2838
Chr25	4852585	A	C	Chr25_48525	A_C	SNP	692.41	0	0	0	0	0	0	0	0	0	0	0	19	0	0	26	16	12	27	9	40	31	29	27	37	15	19	16	7	0	0	0	0	0	0	0	0	0	0	0	1	0	0	0	1	1	0	1	0	0	downstream	MODIFIER	Pp3c25_7310	Pp3c25_7310	transcript	PAC:3297914	protein_codi	NA	c.*3297T>G	NA	NA	NA	NA	2846
Chr25	4852585	A	C	Chr25_48525	A_C	SNP	692.41	0	0	0	0	0	0	0	0	0	0	0	19	0	0	26	16	12	27	9	40	31	29	27	37	15	19	16	7	0	0	0	0	0	0	0	0	0	0	0	1	0	0	0	1	1	0	1	0	0	downstream	MODIFIER	Pp3c25_7310	Pp3c25_7310	transcript	PAC:3297915	protein_codi	NA	c.*4274T>G	NA	NA	NA	NA	2872
Chr25	4852585	A	C	Chr25_48525	A_C	SNP	692.41	0	0	0	0	0	0	0	0	0	0	0	19	0	0	26	16	12	27	9	40	31	29	27	37	15	19	16	7	0	0	0	0	0	0	0	0	0	0	0	1	0	0	0	1	1	0	1	0	0	downstream	MODIFIER	Pp3c25_7310	Pp3c25_7310	transcript	PAC:3297915	protein_codi	NA	c.*4274T>G	NA	NA	NA	NA	2872
Chr25	4852585	A	C	Chr25_48525	A_C	SNP	692.41	0	0	0	0	0	0	0	0	0	0	0	19	0	0	26	16	12	27	9	40	31	29	27	37	15	19	16	7	0	0	0	0	0	0	0	0	0	0	0	1	0	0	0	1	1	0	1	0	0	intergenic_re	MODIFIER	Pp3c25_7300	Pp3c25_7300	intergenic_re	Pp3c25_7300	NA	NA	n.4852585A>	NA	NA	NA	NA	NA
Chr25	8785469	CATATAT	C	Chr25_87854	CATATAT_C	INDEL	63.37	0	0	0	0	0	0	0	0	0	0	0	0	0	4	12	11	14	26	18	15	31	23	18	20	16	8	4	5	0	0	0	0	0	0	0	0	0	0	0	0	0	0.8	0	1	1	0	1	0	0	upstream_ge	MODIFIER	Pp3c25_1240	Pp3c25_1240	transcript	PAC:3297992	protein_codi	NA	c.-990_-985d	NA	NA	NA	NA	990
Chr25	8785469	CATATAT	C	Chr25_87854	CATATAT_C	INDEL	63.37	0	0	0	0	0	0	0	0	0	0	0	0	0	4	12	11	14	26	18	15	31	23	18	20	16	8	4	5	0	0	0	0	0	0	0	0	0	0	0	0	0	0.8	0	1	1	0	1	0	0	upstream_ge	MODIFIER	Pp3c25_1240	Pp3c25_1240	transcript	PAC:3297992	protein_codi	NA	c.-990_-985d	NA	NA	NA	NA	990
Chr25	8785469	CATATAT	C	Chr25_87854	CATATAT_C	INDEL	63.37	0	0	0	0	0	0	0	0	0	0	0	0	0	4	12	11	14	26	18	15	31	23	18	20	16	8	4	5	0	0	0	0	0	0	0	0	0	0	0	0	0	0.8	0	1	1	0	1	0	0	downstream	MODIFIER	Pp3c25_1241	Pp3c25_1241	transcript	PAC:3297932	protein_codi	NA	c.*1736_*17	NA	NA	NA	NA	1741
Chr25	8785469	CATATAT	C	Chr25_87854	CATATAT_C	INDEL	63.37	0	0	0	0	0	0	0	0	0	0	0	0	0	4	12	11	14	26	18	15	31	23	18	20	16	8	4	5	0	0	0	0	0	0	0	0	0	0	0	0	0	0.8	0	1	1	0	1	0	0	intergenic_re	MODIFIER	Pp3c25_1237	Pp3c25_1237	intergenic_re	Pp3c25_1237	NA	NA	n.8785470_8	NA	NA	NA	NA	NA
Chr25	9412827	CAT	C	Chr25_94128	CAT_C	INDEL	411.37	0	0	0	0	0	0	0	1	0	0	1	14	1	0	20	10	13	16	23	8	17	17	23	20	6	14	7	10	0	0	0	0	0	0	0	0	0	0	0	1	0	0	0	1	1	0	1	0	0	downstream	MODIFIER	Pp3c25_1325	Pp3c25_1325	transcript	PAC:3297989	protein_codi	NA	c.*2913_*29	NA	NA	NA	NA	1918
Chr25	9412827	CAT	C	Chr25_94128	CAT_C	INDEL	411.37	0	0	0	0	0	0	0	1	0	0	1	14	1	0	20	10	13	16	23	8	17	17	23	20	6	14	7	10	0	0	0	0	0	0	0	0	0	0	0	1	0	0	0	1	1	0	1	0	0	downstream	MODIFIER	Pp3c25_1325	Pp3c25_1325	transcript	PAC:3297989	protein_codi	NA	c.*2913_*29	NA	NA	NA	NA	2006
Chr25	9412827	CAT	C	Chr25_94128	CAT_C	INDEL	411.37	0	0	0	0	0	0	0	1	0	0	1	14	1	0	20	10	13	16	23	8	17	17	23	20	6	14	7	10	0	0	0	0	0	0	0	0	0	0	0	1	0	0	0	1	1	0	1	0	0	downstream	MODIFIER	Pp3c25_1325	Pp3c25_1325	transcript	PAC:3297989	protein_codi	NA	c.*2913_*29	NA	NA	NA	NA	2006
Chr25	9412827	CAT	C	Chr25_94128	CAT_C	INDEL	411.37	0	0	0	0	0	0	0	1	0	0	1	14	1	0	20	10	13	16	23	8	17	17	23	20	6	14	7	10	0	0	0	0	0	0	0	0	0	0	0	1	0	0	0	1	1	0	1	0	0	downstream	MODIFIER	Pp3c25_1325	Pp3c25_1325	transcript	PAC:3297989	protein_codi	NA	c.*2913_*29	NA	NA	NA	NA	2006
Chr25	9412827	CAT	C	Chr25_94128	CAT_C	INDEL	411.37	0	0	0	0	0	0	0	1	0	0	1	14	1	0	20	10	13	16	23	8	17	17	23	20	6	14	7	10	0	0	0	0	0	0	0	0	0	0	0	1	0	0	0	1	1	0	1	0	0	downstream	MODIFIER	Pp3c25_1325	Pp3c25_1325	transcript	PAC:3297989	protein_codi	NA	c.*2913_*29	NA	NA	NA	NA	2006
Chr25	9412827	CAT	C	Chr25_94128	CAT_C	INDEL	411.37	0	0	0	0	0	0	0	1	0	0	1	14	1	0	20	10	13	16	23	8	17	17	23	20	6	14	7	10	0	0	0	0	0	0	0	0	0	0	0	1	0	0	0	1	1	0	1	0	0	downstream	MODIFIER	Pp3c25_1325	Pp3c25_1325	transcript	PAC:3297990	protein_codi	NA	c.*2913_*29	NA	NA	NA	NA	2006
Chr25	9412827	CAT	C	Chr25_94128	CAT_C	INDEL	411.37	0	0	0	0	0	0	0	1	0	0	1	14	1	0	20	10	13	16	23	8	17	17	23	20	6	14	7	10	0	0	0	0	0	0	0	0	0	0	0	1	0	0	0	1	1	0	1	0	0	downstream	MODIFIER	Pp3c25_1325	Pp3c25_1325	transcript	PAC:3297990	protein_codi	NA	c.*2913_*29	NA	NA	NA	NA	2006
Chr25	9412827	CAT	C	Chr25_94128	CAT_C	INDEL	411.37	0	0	0	0	0	0	0	1	0	0	1	14	1	0	20	10	13	16	23	8	17	17	23	20	6	14	7	10	0	0	0	0	0	0	0	0	0	0	0	1	0	0	0	1	1	0	1	0	0	downstream	MODIFIER	Pp3c25_1325	Pp3c25_1325	transcript	PAC:3297990	protein_codi	NA	c.*2913_*29	NA	NA	NA	NA	2006
Chr25	9412827	CAT	C	Chr25_94128	CAT_C	INDEL	411.37	0	0	0	0	0	0	0	1	0	0	1	14	1	0	20	10	13	16	23	8	17	17	23	20	6	14	7	10	0	0	0	0	0	0	0	0	0	0	0	1	0	0	0	1	1	0	1	0	0	downstream	MODIFIER	Pp3c25_1325	Pp3c25_1325	transcript	PAC:3297990	protein_codi	NA	c.*2913_*29	NA	NA	NA	NA	2006
Chr25	9412827	CAT	C	Chr25_94128	CAT_C	INDEL	411.37	0	0	0	0	0	0	0	1	0	0	1	14	1	0	20	10	13	16	23	8	17	17	23	20	6	14	7	10	0	0	0	0	0	0	0	0	0	0	0	1	0	0	0	1	1	0	1	0	0	intergenic_re	MODIFIER	Pp3c25_1323	Pp3c25_1323	intergenic_re	Pp3c25_1323	NA	NA	n.9412828_9	NA	NA	NA	NA	NA
Chr25	11025256	T	TTA	Chr25_11025	T_TTA	INDEL	395.37	2	0	14	0	0	0	0	0	1	0	0	0	0	0	25	6	14	19	14	16	24	25	17	23	7	11	12	7	0.08	0	1	0	0	0	0	0	0	0	0	0	0	0	0	1	1	1	0	0	1	upstream_ge	MODIFIER	Pp3c25_1524	Pp3c25_1524	transcript	PAC:3297945	protein_codi	NA	c.-3962_-396	NA	NA	NA	NA	3962
Chr25	11025256	T	TTA	Chr25_11025	T_TTA	INDEL	395.37	2	0	14	0	0	0	0	0	1	0	0	0	0	0	25	6	14	19	14	16	24	25	17	23	7	11	12	7	0.08	0	1	0	0	0	0	0	0	0	0	0	0	0	0	1	1	1	0	0	1	upstream_ge	MODIFIER	Pp3c25_1526	Pp3c25_1526	transcript	PAC:3297950	protein_codi	NA	c.-4471_-447	NA	NA	NA	NA	4075
Chr25	11025256	T	TTA	Chr25_11025	T_TTA	INDEL	395.37	2	0	14	0	0	0	0	0	1	0	0	0	0	0	25	6	14	19	14	16	24	25	17	23	7	11	12	7	0.08	0	1	0	0	0	0	0	0	0	0	0	0	0	0	1	1	1	0	0	1	upstream_ge	MODIFIER	Pp3c25_1524	Pp3c25_1524	transcript	PAC:3297945	protein_codi	NA	c.-3962_-396	NA	NA	NA	NA	3863
Chr25	11025256	T	TTA	Chr25_11025	T_TTA	INDEL	395.37	2	0	14	0	0	0	0	0	1	0	0	0	0	0	25	6	14	19	14	16	24	25	17	23	7	11	12	7	0.08	0	1	0	0	0	0	0	0	0	0	0	0	0	0	1	1	1	0	0	1	upstream_ge	MODIFIER	Pp3c25_1526	Pp3c25_1526	transcript	PAC:3297950	protein_codi	NA	c.-4471_-447	NA	NA	NA	NA	4201
Chr25	11025256	T	TTA	Chr25_11025	T_TTA	INDEL	395.37	2	0	14	0	0	0	0	0	1	0	0	0	0	0	25	6	14	19	14	16	24	25	17	23	7	11	12	7	0.08	0	1	0	0	0	0	0	0	0	0	0	0	0	0	1	1	1	0	0	1	intergenic_re	MODIFIER	Pp3c25_1524	Pp3c25_1524	intergenic_re	Pp3c25_1524	NA	NA	n.11025256_	NA	NA	NA	NA	NA
Chr26	2327832	C	CTA	Chr26_23278	C_CTA	INDEL	520.37	0	0	0	0	12	0	0	3	1	2	0	2	0	1	19	11	15	16	13	14	42	18	19	18	10	12	8	5	0	0	0	0	0.923	0	0	0.167	0	0.111	0	0.167	0	0	1	1	1	3	0	1	0	intergenic_re	MODIFIER	Pp3c26_4630	Pp3c26_4630	intergenic_re	Pp3c26_4630	NA	NA	n.2327832_2	NA	NA	NA	NA	NA
Chr26	3499854	A	AAT	Chr26_34998	A_AAT	INDEL	227.37	3	10	0	0	0	1	0	0	0	0	1	1	1	NA	15	10	7	8	12	18	20	16	13	9	14	6	5	NA	0.2	1	0	0	0	0	0	0	0	0	0	0	0	NA	1	1	1	1	0	0	1	upstream_ge	MODIFIER	Pp3c26_6470	Pp3c26_6470	transcript	PAC:3291849	protein_codi	NA	c.-2222_-222	NA	NA	NA	NA	1903
Chr26	3499854	A	AAT	Chr26_34998	A_AAT	INDEL	227.37	3	10	0	0	0	1	0	0	0	0	1	1	1	NA	15	10	7	8	12	18	20	16	13	9	14	6	5	NA	0.2	1	0	0	0	0	0	0	0	0	0	0	0	NA	1	1	1	1	0	0	1	upstream_ge	MODIFIER	Pp3c26_6470	Pp3c26_6470	transcript	PAC:3291849	protein_codi	NA	c.-2222_-222	NA	NA	NA	NA	1861
Chr26	3499854	A	AAT	Chr26_34998	A_AAT	INDEL	227.37	3	10	0	0	0	1	0	0	0	0	1	1	1	NA	15	10	7	8	12	18	20	16	13	9	14	6	5	NA	0.2	1	0	0	0	0	0	0	0	0	0	0	0	NA	1	1	1	1	0	0	1	downstream	MODIFIER	Pp3c26_6460	Pp3c26_6460	transcript	PAC:3291710	protein_codi	NA	c.*2910_*29	NA	NA	NA	NA	2911
Chr26	3499854	A	AAT	Chr26_34998	A_AAT	INDEL	227.37	3	10	0	0	0	1	0	0	0	0	1	1	1	NA	15	10	7	8	12	18	20	16	13	9	14	6	5	NA	0.2	1	0	0	0	0	0	0	0	0	0	0	0	NA	1	1	1	1	0	0	1	downstream	MODIFIER	Pp3c26_6460	Pp3c26_6460	transcript	PAC:3291710	protein_codi	NA	c.*2120_*21	NA	NA	NA	NA	2121
Chr26	3499854	A	AAT	Chr26_34998	A_AAT	INDEL	227.37	3	10	0	0	0	1	0	0	0	0	1	1	1	NA	15	10	7	8	12	18	20	16	13	9	14	6	5	NA	0.2	1	0	0	0	0	0	0	0	0	0	0	0	NA	1	1	1	1	0	0	1	intergenic_re	MODIFIER	Pp3c26_6460	Pp3c26_6460	intergenic_re	Pp3c26_6460	NA	NA	n.3499854_3	NA	NA	NA	NA	NA
Chr26	3571654	A	AAT	Chr26_35716	A_AAT	INDEL	195.37	0	0	0	0	0	1	0	1	1	0	0	0	8	1	15	9	10	15	12	13	16	12	14	13	9	7	8	8	0	0	0	0	0	0	0	0	0	0	0	0	1	0	0	1	1	0	1	0	0	downstream	MODIFIER	Pp3c26_6630	Pp3c26_6630	transcript	PAC:3291771	protein_codi	NA	c.*1176_*11	NA	NA	NA	NA	1177
Chr26	3571654	A	AAT	Chr26_35716	A_AAT	INDEL	195.37	0	0	0	0	0	1	0	1	1	0	0	0	8	1	15	9	10	15	12	13	16	12	14	13	9	7	8	8	0	0	0	0	0	0	0	0	0	0	0	0	1	0	0	1	1	0	1	0	0	downstream	MODIFIER	Pp3c26_6631	Pp3c26_6631	transcript	PAC:3291727	protein_codi	NA	c.*1176_*11	NA	NA	NA	NA	1177
Chr26	3571654	A	AAT	Chr26_35716	A_AAT	INDEL	195.37	0	0	0	0	0	1	0	1	1	0	0	0	8	1	15	9	10	15	12	13	16	12	14	13	9	7	8	8	0	0	0	0	0	0	0	0	0	0	0	0	1	0	0	1	1	0	1	0	0	intergenic_re	MODIFIER	Pp3c26_6630	Pp3c26_6630	intergenic_re	Pp3c26_6630	NA	NA	n.3571654_3	NA	NA	NA	NA	NA
Chr26	5230207	G	T	Chr26_52302	G_T	SNP	676.41	0	0	0	0	21	0	0	2	0	0	0	0	0	0	28	12	12	25	25	19	25	21	12	20	19	12	7	6	0	0	0	0	0.84	0	0	0.095	0	0	0	0	0	0	0	1	1	1	0	1	0	intergenic_re	MODIFIER	Pp3c26_8890	Pp3c26_8890	intergenic_re	Pp3c26_8890	NA	NA	n.5230207G	NA	NA	NA	NA	NA
Chr26	7256703	T	TTA	Chr26_72567	T_TTA	INDEL	161.37	0	7	1	1	0	1	0	1	0	2	0	0	0	NA	21	7	12	14	6	22	14	7	9	9	6	7	6	NA	0	1	0	0	0	0	0	0	0	0.222	0	0	0	NA	1	1	1	1	0	0	1	upstream_ge	MODIFIER	Pp3c26_1155	Pp3c26_1155	transcript	PAC:3291842	protein_codi	NA	c.-1681_-168	NA	NA	NA	NA	1681
Chr26	7256703	T	TTA	Chr26_72567	T_TTA	INDEL	161.37	0	7	1	1	0	1	0	1	0	2	0	0	0	NA	21	7	12	14	6	22	14	7	9	9	6	7	6	NA	0	1	0	0	0	0	0	0	0	0.222	0	0	0	NA	1	1	1	1	0	0	1	upstream_ge	MODIFIER	Pp3c26_1155	Pp3c26_1155	transcript	PAC:3291842	protein_codi	NA	c.-1681_-168	NA	NA	NA	NA	569
Chr26	7256703	T	TTA	Chr26_72567	T_TTA	INDEL	161.37	0	7	1	1	0	1	0	1	0	2	0	0	0	NA	21	7	12	14	6	22	14	7	9	9	6	7	6	NA	0	1	0	0	0	0	0	0	0	0.222	0	0	0	NA	1	1	1	1	0	0	1	downstream	MODIFIER	Pp3c26_1157	Pp3c26_1157	transcript	PAC:3291867	protein_codi	NA	c.*1995_*19	NA	NA	NA	NA	1995
Chr26	7256703	T	TTA	Chr26_72567	T_TTA	INDEL	161.37	0	7	1	1	0	1	0	1	0	2	0	0	0	NA	21	7	12	14	6	22	14	7	9	9	6	7	6	NA	0	1	0	0	0	0	0	0	0	0.222	0	0	0	NA	1	1	1	1	0	0	1	intergenic_re	MODIFIER	Pp3c26_1155	Pp3c26_1155	intergenic_re	Pp3c26_1155	NA	NA	n.7256703_7	NA	NA	NA	NA	NA
Chr26	8082291	C	T	Chr26_80822	C_T	SNP	750.41	0	0	0	0	0	19	0	0	0	0	0	0	0	0	35	23	18	24	21	19	28	33	10	24	9	12	14	10	0	0	0	0	0	1	0	0	0	0	0	0	0	0	0	1	1	0	0	1	0	intergenic_re	MODIFIER	Pp3c26_1300	Pp3c26_1300	intergenic_re	Pp3c26_1300	NA	NA	n.8082291C>	NA	NA	NA	NA	NA
Chr26	9532829	A	C	Chr26_95328	A_C	SNP	843.41	0	0	0	4	0	22	0	0	0	0	1	0	0	0	29	16	19	17	18	22	25	17	20	19	7	20	12	7	0	0	0	0.235	0	1	0	0	0	0	0	0	0	0	0	1	1	1	0	1	0	intergenic_re	MODIFIER	Pp3c26_1503	Pp3c26_1503	intergenic_re	Pp3c26_1503	NA	NA	n.9532829A>	NA	NA	NA	NA	NA
Chr27	77812	T	TTA	Chr27_77812	T_TTA	INDEL	435.37	1	17	1	0	1	2	1	2	0	0	0	1	0	NA	16	17	6	16	8	17	21	11	4	10	7	16	4	NA	0	1	0	0	0	0.118	0	0.182	0	0	0	0	0	NA	1	1	1	2	0	0	1	upstream_ge	MODIFIER	Pp3c27_90	Pp3c27_90	transcript	PAC:3295143	protein_codi	NA	c.-3344_-334	NA	NA	NA	NA	3343
Chr27	77812	T	TTA	Chr27_77812	T_TTA	INDEL	435.37	1	17	1	0	1	2	1	2	0	0	0	1	0	NA	16	17	6	16	8	17	21	11	4	10	7	16	4	NA	0	1	0	0	0	0.118	0	0.182	0	0	0	0	0	NA	1	1	1	2	0	0	1	upstream_ge	MODIFIER	Pp3c27_90	Pp3c27_90	transcript	PAC:3295143	protein_codi	NA	c.-3344_-334	NA	NA	NA	NA	2787
Chr27	77812	T	TTA	Chr27_77812	T_TTA	INDEL	435.37	1	17	1	0	1	2	1	2	0	0	0	1	0	NA	16	17	6	16	8	17	21	11	4	10	7	16	4	NA	0	1	0	0	0	0.118	0	0.182	0	0	0	0	0	NA	1	1	1	2	0	0	1	intergenic_re	MODIFIER	Pp3c27_70-P	Pp3c27_70-P	intergenic_re	Pp3c27_70-P	NA	NA	n.77812_778	NA	NA	NA	NA	NA
Chr27	2013203	T	TTA	Chr27_20132	T_TTA	INDEL	401.37	0	0	1	0	0	0	16	0	0	0	0	0	0	0	24	8	13	12	17	11	16	18	10	19	4	11	6	11	0	0	0	0	0	0	1	0	0	0	0	0	0	0	0	1	1	0	0	1	0	upstream_ge	MODIFIER	Pp3c27_3440	Pp3c27_3440	transcript	PAC:3295194	protein_codi	NA	c.-4365_-436	NA	NA	NA	NA	3805
Chr27	2013203	T	TTA	Chr27_20132	T_TTA	INDEL	401.37	0	0	1	0	0	0	16	0	0	0	0	0	0	0	24	8	13	12	17	11	16	18	10	19	4	11	6	11	0	0	0	0	0	0	1	0	0	0	0	0	0	0	0	1	1	0	0	1	0	upstream_ge	MODIFIER	Pp3c27_3440	Pp3c27_3440	transcript	PAC:3295194	protein_codi	NA	c.-4365_-436	NA	NA	NA	NA	3800
Chr27	2013203	T	TTA	Chr27_20132	T_TTA	INDEL	401.37	0	0	1	0	0	0	16	0	0	0	0	0	0	0	24	8	13	12	17	11	16	18	10	19	4	11	6	11	0	0	0	0	0	0	1	0	0	0	0	0	0	0	0	1	1	0	0	1	0	upstream_ge	MODIFIER	Pp3c27_3440	Pp3c27_3440	transcript	PAC:3295194	protein_codi	NA	c.-4365_-436	NA	NA	NA	NA	3800
Chr27	2013203	T	TTA	Chr27_20132	T_TTA	INDEL	401.37	0	0	1	0	0	0	16	0	0	0	0	0	0	0	24	8	13	12	17	11	16	18	10	19	4	11	6	11	0	0	0	0	0	0	1	0	0	0	0	0	0	0	0	1	1	0	0	1	0	downstream	MODIFIER	Pp3c27_3460	Pp3c27_3460	transcript	PAC:3295190	protein_codi	NA	c.*1452_*14	NA	NA	NA	NA	920
Chr27	2013203	T	TTA	Chr27_20132	T_TTA	INDEL	401.37	0	0	1	0	0	0	16	0	0	0	0	0	0	0	24	8	13	12	17	11	16	18	10	19	4	11	6	11	0	0	0	0	0	0	1	0	0	0	0	0	0	0	0	1	1	0	0	1	0	downstream	MODIFIER	Pp3c27_3460	Pp3c27_3460	transcript	PAC:3295191	protein_codi	NA	c.*1452_*14	NA	NA	NA	NA	843
Chr27	2013203	T	TTA	Chr27_20132	T_TTA	INDEL	401.37	0	0	1	0	0	0	16	0	0	0	0	0	0	0	24	8	13	12	17	11	16	18	10	19	4	11	6	11	0	0	0	0	0	0	1	0	0	0	0	0	0	0	0	1	1	0	0	1	0	downstream	MODIFIER	Pp3c27_3460	Pp3c27_3460	transcript	PAC:3295191	protein_codi	NA	c.*1761_*17	NA	NA	NA	NA	843
Chr27	2013203	T	TTA	Chr27_20132	T_TTA	INDEL	401.37	0	0	1	0	0	0	16	0	0	0	0	0	0	0	24	8	13	12	17	11	16	18	10	19	4	11	6	11	0	0	0	0	0	0	1	0	0	0	0	0	0	0	0	1	1	0	0	1	0	downstream	MODIFIER	Pp3c27_3460	Pp3c27_3460	transcript	PAC:3295191	protein_codi	NA	c.*1452_*14	NA	NA	NA	NA	920
Chr27	2013203	T	TTA	Chr27_20132	T_TTA	INDEL	401.37	0	0	1	0	0	0	16	0	0	0	0	0	0	0	24	8	13	12	17	11	16	18	10	19	4	11	6	11	0	0	0	0	0	0	1	0	0	0	0	0	0	0	0	1	1	0	0	1	0	downstream	MODIFIER	Pp3c27_3460	Pp3c27_3460	transcript	PAC:3295191	protein_codi	NA	c.*2365_*23	NA	NA	NA	NA	920
Chr27	2013203	T	TTA	Chr27_20132	T_TTA	INDEL	401.37	0	0	1	0	0	0	16	0	0	0	0	0	0	0	24	8	13	12	17	11	16	18	10	19	4	11	6	11	0	0	0	0	0	0	1	0	0	0	0	0	0	0	0	1	1	0	0	1	0	downstream	MODIFIER	Pp3c27_3460	Pp3c27_3460	transcript	PAC:3295191	protein_codi	NA	c.*1761_*17	NA	NA	NA	NA	1666
Chr27	2013203	T	TTA	Chr27_20132	T_TTA	INDEL	401.37	0	0	1	0	0	0	16	0	0	0	0	0	0	0	24	8	13	12	17	11	16	18	10	19	4	11	6	11	0	0	0	0	0	0	1	0	0	0	0	0	0	0	0	1	1	0	0	1	0	intergenic_re	MODIFIER	Pp3c27_3440	Pp3c27_3440	intergenic_re	Pp3c27_3440	NA	NA	n.2013203_2	NA	NA	NA	NA	NA
Chr27	2438202	T	TTA	Chr27_24382	T_TTA	INDEL	62.37	0	0	5	0	1	0	0	0	0	0	0	2	0	0	20	8	6	16	9	12	19	10	10	10	5	14	7	5	0	0	0.833	0	0	0	0	0	0	0	0	0.143	0	0	0	1	1	1	0	0	1	intergenic_re	MODIFIER	Pp3c27_4350	Pp3c27_4350	intergenic_re	Pp3c27_4350	NA	NA	n.2438202_2	NA	NA	NA	NA	NA
