# Supplementary material for: Genome-wide specificity of plant genome editing by both CRISPR–Cas9 and TALEN
Source: Sci Rep. 2022 Jun 4;12:9330. doi: 10.1038/s41598-022-13034-2 (PMC9167288; doi:10.1038/s41598-022-13034-2)
Supplement: Supplementary file 4 — Supplementary Information 4. [file 41598_2022_13034_MOESM4_ESM.pdf]

Table S1. Sample description and depth coverage.

Plant	Sequencing technology	Experiment	Mean	Granular median	% bases above 15X	% mapped reads
Control#1	NovaSeq	control	30.16	23	88.9	99.97
Control#2	HiSeq4000	control	21.11	16	53.5	99.64
PEG#1	HiSeq4000	control	24.26	18	66.0	99.65
PEG#2	NovaSeq	control	14.84	11	16.7	99.96
PEG-DNA#1	NovaSeq	control	13.87	11	15.4	99.97
WTC	NovaSeq	control	28.29	23	88.0	99.95
CRIS1#1	HiSeq4000	Crispr	26.93	20	80.4	99.91
CRIS1#2	HiSeq4000	Crispr	34.20	26	95.1	99.89
CRIS2#1	HiSeq4000	Crispr	39.05	30	97.4	99.91
CRIS2#2	HiSeq4000	Crispr	32.91	23	91.8	95.15
WTA	HiSeq4000	Crispr	34.08	25	94.6	99.92
TAL#1	HiSeq4000	Talen	21.06	16	51.6	99.96
TAL#2	HiSeq4000	Talen	19.76	15	48.4	99.71
WTB	HiSeq4000	Talen	36.70	28	96.6	99.97

Table S2. Repartition of variants (SNVs+InDel) on the different chromosomes.

Chromosome number	Chromosome length	Observed SNVs + InDels	Number of genes
Chr01	30242098	561	2047
Chr02	25998084	516	1872
Chr03	25551832	497	1815
Chr04	22344759	416	1513
Chr05	20681290	458	1310
Chr06	19533230	379	1414
Chr07	18116774	377	1333
Chr08	17934281	323	1158
Chr09	17794193	293	1197
Chr10	17530623	294	1224
Chr11	17494229	251	1319
Chr12	17435539	332	1172
Chr13	17250819	398	1070
Chr14	17248380	332	1340
Chr15	17009704	303	1184
Chr16	16572167	296	1265
Chr17	15984037	343	1158
Chr18	15735161	334	937
Chr19	15673097	310	1025
Chr20	15486712	328	1116
Chr21	15510460	361	1016
Chr22	15354560	235	989
Chr23	15138416	310	1007
Chr24	13755829	268	959
Chr25	11183739	217	658
Chr26	10125990	219	716
Chr27	5299892	97	420

Table S3. SNPs present in the Switzerland (CH) Gransden pedigree (Haas et al. 2020) are detected in the three different WT lines.

Position on Chromosomes*	Variant* type	WT_A ratio	WT_B ratio	WT_C ratio
Chr04:9964399	A>T	0	1	1
Chr07:9516941	C>T	0	1	1
Chr13:4445259	T>C	1	1	1
Chr20:832125	A>T	0	1	1
Chr20:14648709	C>T	0	1	1
Chr23:11248087	T>C	1	1	1
Chr23:13598266	C>A	0	0	1
Chr24:13315883	T>C	1	1	1

* Data collected from Haas et al., 2020 (Supplementary file 3).

Table S4. Sanger analysis of variants found by whole genome sequence analysis in the different control plants.

Position on chromosomes	Variant type	WT_C		Control#1		Control#2		PEG#1		PEG#2		PEG-DNA#1		PEG-DNA#2	
		Ratio[a]	Sanger[b]	Ratio	Sanger	Ratio	Sanger	Ratio	Sanger	Ratio	Sanger	Ratio	Sanger	Ratio	Sanger
Chr16:14154133	T>G	0	(-)	0	(-)	0	(-)	0	(-)	1	(+)	0	(-)	nd[c]	nd
Chr22:5281637	G>C	0	(-)	0	(-)	0	(-)	1	(+)	0	(-)	0	(-)	0	(-)
Chr24:10845297	T>C	0	(-)	0	(-)	0	(-)	0	(-)	0	(-)	1	(+)	0	(-)

[a] correspond to the variant ratio (number of reads supporting called variant/number of reads covering the variant position).
[b] presence (+) or absence (-) of the variant in the different plants was analyzed by Sanger analysis.
[c] Not determined (nd).

Table S5. Sanger analysis of variants found by whole genome sequence analysis in the different CRISPR-Cas edited plants.

Position on chromosomes	Variant type	WT_A		CRIS1#1		CRIS1#2		CRIS2#1		CRIS2#2		Primer name
		Ratio[a]	Sanger[b]	Ratio	Sanger	Ratio	Sanger	Ratio	Sanger	Ratio[a]	Sanger	
Chr11:6608033	G>T	0	(-)	1	(+)	0	nd	0	(-)	0	(-)	chr11_660
Chr03:4719499	T>TTA	0	(-)	1	(+)	0	(-)	0	(-)	0	(-)	chr03_471
Chr22:874414	C>A	0	(-)	0	(-)	1	(+)	0	(-)	0	(-)	chr22_87
Chr06:14871263	T>TTA	0	(-)	0	nd[c]	0	(-)	1	(+)	0	(-)	chr06_148
Chr19:5097814	G>A	0	(-)	0	nd	0	(-)	1	(+)	0	(-)	chr19_509
Chr06:18710749	C>CAT	0	(-)	0	(-)	0	(-)	0	(-)	1	(+)	chr06_187
Chr01:21565844[d]	A>G	1	(+)	1	(+)	1	(+)	1	(+)	1	(+)	chr01_215

[a] correspond to the variant ratio.
[b] presence (+) or absence (-) of the variant in the different plants was analyzed by Sanger analysis.
[c] Not determined (nd).
[d] This marker was used as a control.

Table S6. Sanger analysis of variants found by whole genome sequence analysis in the different TALEN edited plants.

Position on chromosomes	Variant type	WT_B		TAL#1		TAL#2		Primer name
		Ratio[a]	Sanger[b]	Ratio	Sanger	Ratio	Sanger	
Chr14:12797646	TGTTCACGA>T	0	(-)	0	(-)	1	(+)	chr14_127
Chr10:14315450	A17del>A	0	(-)	1	(+)	0	(-)	chr10_143
Chr10:14329574	GAAA>ATAT	0	(-)	1	(+)	0	(-)	chr10_143_2
Chr07:17384449c	G56del>G	1	(+)	1	(+)	1	(+)	chr07_173

[a] correspond to the variant ratio.

[b] presence (+) or absence (-) of the variant in the different plants was analyzed by Sanger analysis.

[c] This marker was used as a control.

Table S7. Spontaneous mutations observed in treated plants compared to WTs.

Position on chromosomes	Variant type	Treated plants (ratio= 1)	Corresponding WT ratio
Chr07:5186318	C>CTA	PEG#2	0.529
Chr03:15558655	C>A	TAL#1	0.348
Chr16:11843701	C>T	TAL#1	0.333
Chr22:3967666	C>T	TAL#1	0.421
Chr17:11774744	A>AAT	TAL#1	0.35
Chr07:7684665	G>A	CRIS1#2	0.3
Chr13:12620517	A>T	CRIS1#2; CRIS2#2	0.148
Chr20:7057654	C>T	CRIS1#1; CRIS1#2; CRIS2#1; CRIS2#2	0.176
Chr08:3426363	GAT>G	CRIS1#2	0.316
Chr08:4839068	G>GAT	CRIS1#1; CRIS1#2; CRIS2#1; CRIS2#2	0.278
Chr09:12282642	G>GTA	CRIS1#2	0.357
Chr12:8195075	C>CAT	CRIS1#2; CRIS2#2	0.2
Chr12:16266255	C>CAT	CRIS1#1; CRIS1#2; CRIS2#1;CRIS2#2	0.222
Chr18:5002913	CAT>C	CRIS1#1; CRIS1#2; CRIS2#1; CRIS2#2	0.095

Table S8. Variations detected in genes and possible consequences on corresponding proteins.

Position on chromosomes	Variant change	Gene Name	Variant location	conditions	Protein change
Chr24:10845297	T>C	Pp3c24_16550	Intron	PEG-DNA#1	np
Chr22:14586029	A>AAT	Pp3c22_21780	Intron	CRIS2#2	np
Chr22:5281637	G>C	Pp3c22_8540	5_prime_UTR	PEG#1	np
Chr20:5018396	G>A	Pp3c20_8180	Intron	TAL#1	np
Chr19:5097814	G>A	Pp3c19_8330	3_prime_UTR	CRIS2#1	np
Chr17:1436880	C>CAT	Pp3c17_1970	Intron	PEG-DNA#1	np
Chr17:793466	G>A	Pp3c17_930	Exon1	PEG#1	Leu260Leu
Chr16:14154133	T>G	Pp3c16_22830	Exon2	PEG#2	Leu260Leu
Chr16:677406	T14del>T	Pp3c16_1070	Intron	CRIS1#1	np
Chr14:12797646	TGTTCACGA>T	Pp3c14_19390	Intron	TAL#2	np
Chr13_12620517	A>T	Pp3c13_17680	3' UTR	CRIS2#2	np
Chr11:1627481	CGC>AT	Pp3c11_2830	Intron	TAL#2	np
Chr10:14315450	A17del>A	Pp3c10_20860	5_prime_UTR	TAL#1	np
Chr10:14316818	C>A	Pp3c10_20860	Intron	TAL#1	np
Chr10:14320441	C>G	Pp3c10_20860	Intron	TAL#1	np
Chr10:14329574	GAAA>ATAT	Pp3c10_20860	Intron	TAL#1	np
Chr09:16179557	G>GTA	Pp3c9_23640	Intron	PEG-DNA#1	np
Chr09:11300798	GTATA>G	Pp3c9_16610	3_prime_UTR	PEG#1	np
Chr09:9844481	T>TTA	Pp3c9_14700	Intron	CRIS1#2	np
Chr06:14871263	T>TTA	Pp3c6_23030	Intron	CRIS2#1	np
Chr06:7346938	G>T	Pp3c6_11420	Intron	TAL#1	np

[a] Not predictable (np).

Table S9. Sequences and positions of possible off target sites for sgRNA1 and sgRNA2 identified using the CRISPOR v2.0 tool (http://crispor.tefor.net).

Locus name	Target sequence [a] + PAM	Position on *P. patens* reference genome from Phytozome [b]
**sgRNA1 Target**	**gaagagtatagtctagagtaTGG**	Chr08:10812103 to 10812081
sgRNA1 Off-Target#1	gaagagtgtagagtagagtaGGG	Chr07:4705222 to 4705244
sgRNA1 Off-Target#2	gaagcttattgtctagagttAGG	Chr05:17438841 to 17438819
sgRNA1 Off-Target#3	gtaaagtatagtctagggttGGG	Chr24:6867383 to 6867405
sgRNA1 Off-Target#4	caagcgtatactcttgagtaTGG	Chr14:2675353 to 2675375
sgRNA1 Off-Target#5	gaatattataatctagagttGGG	Chr22:3453377 to 3453399
sgRNA1 Off-Target#6	gcaaagtatagtataaagtaGGG	Chr06:582960 to 582982
sgRNA1 Off-Target#7	gaagactatagtttagagggCGG	Chr02:13705150 to 13705128
sgRNA1 Off-Target#8	gaaaagtattgtcaagactaAGG	Chr05:6043528 to 6043550
sgRNA1 Off-Target#9	gaagactatattctatactaCGG	Chr17:5887705 to 5887683
**sgRNA2 Target**	**tgagcgttaccgggaccagaAGG**	Chr08:10812681 to 10812659
sgRNA2 Off-Target#1	tgaĝgagtccaagggaccagaAGG	Chr16:7631517 to 7631494
sgRNA2 Off-Target#2	tgaaggttacctggaccag=AGG	Chr4:12028646 to 12028667
sgRNA2 Off-Target#3	tgggcgtgacggggagcagaAGG	Chr17:4446370 to 4446348
sgRNA2 Off-Target#4	tgagcgtttcaggtacctgaAGG	Chr09:9115552 to 9115574

[a] Mismatches with the target sites are indicated in red).

[b] Coordinates of the off-target sequences were identified using Basic Local Alignment Search Tool (http://phytozome.jgi.doe.gov/pz/portal.html#!search).

Table S10. List of PCR primers used for validation of the variants.

Position of variants	Forward sequence (5'-3')	Reverse sequence (5'-3')
Chr16:14154133	chr16_141f: ATCCTCAAGGAAAAGGGCCG	chr16_141r: TAGGGCACATAGCGTGGTTG
Chr22:5281637	chr22_528f: AGTCTTCCAGGCCGCTAAAC	chr22_528r: TTCGTTGGCTGGAACGATGA
Chr24:10845297	chr24_108f: AGTCAAGCTCCTGTGCAGTG	chr24_108r: CCTCCTTCCTCCCTCCTACC
Chr11:6608033	chr11_660f: TGCTTTGGCTCATGTTTGTACT	chr11_660: TGAAGCATGGCAAGATCTACA
Chr03:4719499	chr03_471f: GTTTGCCTAAAGGCTCCTCCT	chr03_471r: AAAGGCTGGCGCATCACTAT
Chr22:874414	chr22_87f: TGCACTTCTTCAGCCATTAGC	chr22_87r: TCCATTCCGCTTGCCCAATA
Chr06:14871263	chr06_148f: TCCTGCATGACAGTGAGCAA	chr06_148r: TACGTGTCGCGATTGTGGAA
Chr19:5097814	chr19_509f: ATACTCCGTCCTGTCGTCCA	chr19_509r: CACACGAATGGTTGGCAGTG
Chr06:18710749	chr06_187f: CCGCTCAAAGCTAACTGCTC	chr06_187r: TTAATGCATTTAAGTTCTGACCACA
Chr01:21565844	chr01_215f: CAGGGTTTCTCACGCCAGAT	chr01_215r: TCCTTCGACCTCCCAGATGT
Chr14:12797646	chr14_127f: CTCAAGTTTGTTCAGAGAGGAGA	chr14_127r: TGGTTAGCGGATGGGGTCTA
Chr10:14315450	chr10_143f: GCTGACACAGACCCTTCTCC	chr10_143r: AGGCAAAATGAGACGAGCGA
Chr10:14329574	chr10_143_2f: AACACCTCGTACGCCAAGAG	chr10_143_2r: GACCAGGTGTCCGTCAACAA
Chr07:17384449	chr07_173f: CATTCAGTCCGTGTCACCCA	chr07_173r: AGATGCCCCATTTCCACCTG

Table S11. List of PCR primers used for the *APT* gene sequence analysis.

Primer name	Sequence (5'-3')
Rad51.1#6	TGAGGAGGAAGTTCATCATGG
Rad51.1#7	ACCGCCAATGGGTTTATGC
PpAPT#25	GTCGTCACTCTCGGTTTTG
PpAPT#26	CATGCTTCACAGGTTGTTG
