# Supplementary material for: Genome-wide specificity of plant genome editing by both CRISPR–Cas9 and TALEN
Source: Sci Rep. 2022 Jun 4;12:9330. doi: 10.1038/s41598-022-13034-2 (PMC9167288; doi:10.1038/s41598-022-13034-2)
Supplement: Supplementary file 3 — Supplementary Information 3. [file 41598_2022_13034_MOESM3_ESM.pdf]

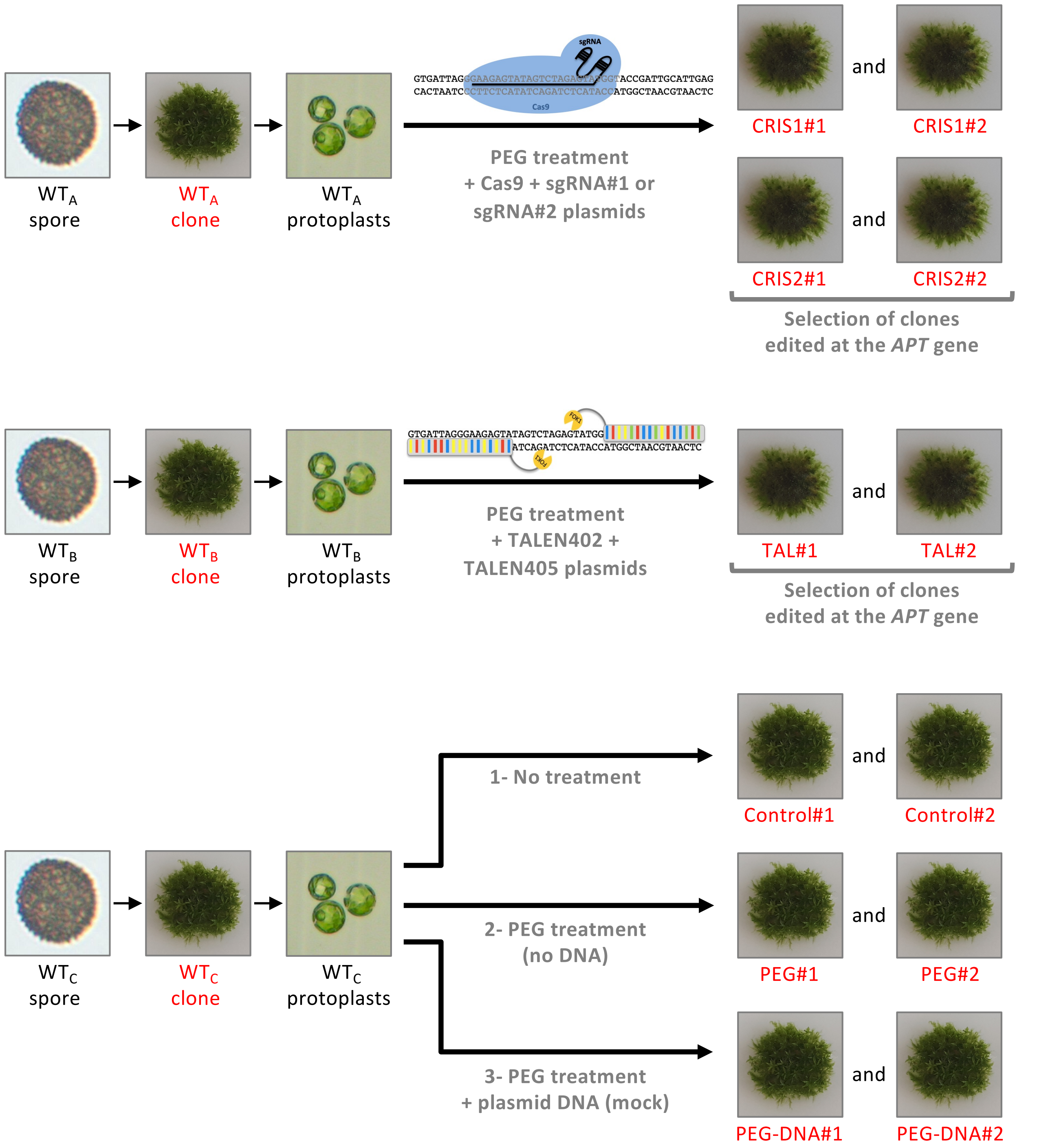

Figure S1. Schematic diagram of the whole procedure of the CRISPR-Cas and TALEN systems for gene editing in *P. patens* plants.

Wild-type plants issued from WT_A, WT_B and WT_C single spores were vegetatively propagated with regular grinding of protonemal tissue for at least 8 weeks to give respectively the WT_A, WT_B and WT_C clones. Wild type protoplasts are issued from 6-day-old protonemal tissue from the WT_A, WT_B or WT_C clones. Non treated or treated (PEG, PEG + mock plasmid, PEG + SDNs plasmids) regenerating plants where vegetatively propagated with regular grinding of protonemal tissue for 8 weeks before DNA extraction. Wild-type, controls and edited plants in red are the ones that have been sequenced. Pictures of this figure are just symbolic and do not represent the actual samples/plants that have been used in the study.

TALEn$_{402}$-TALEn$_{405}$

```
                    TALEn402                       TALEn405
WT        AGGCCCTGTGATTAGGGAAGAGTATAGTCTAGAGTATGGTACCGATTGCATTGAGATGCACGTTGG
TAL#1     AGGCCCTGTGATTAGGGAAGAGTAT------------GGTACCGATTGCATTGAGATGCACGTTGG
TAL#2     AGGCCCTGTGATTAGGGAAGAGTATAGT-------ATGGTACCGATTGCATTGAGATGCACGTTGG
TAL#3     AGGCCCTGTGATTAGGGAAGAGTAT------------GGTACCGATTGCATTGAGATGCACGTTGG
TAL#4     AGGCCCTGTGATTAGGGAAGAGTAT------------GGTACCGATTGCATTGAGATGCACGTTGG
TAL#5     AGGCCCTGTGATTAGGGAAGAGTAT------------AGTACCGATTGCATTGAGATGCACGTTGG
TAL#6     AGGCCCTGTGATTAGGGAAGAGTAT------------GGTACCGATTGCATTGAGATGCACGTTGG
TAL#7     AGGCCCTGTGATTAGGGAAGAGTAT------------AGTACCGATTGCATTGAGATGCACGTTGG
TAL#8     AGGCCCTGTGATTAGGGAAGAGTAT------------GGTACCGATTGCATTGAGATGCACGTTGG
TAL#9     AGGCCCTGTGATTAGGGAAGAGTAT------------GGTACCGATTGCATTGAGATGCACGTTGG
TAL#10    AGGCCCTGTGATTAGGGAAGAGTATAGT-------ATGGTACCGATTGCATTGAGATGCACGTTGG
TAL#11    AGGCCCTGTGATTAGGGAAGAGTAT------------GGTACCGATTGCATTGAGATGCACGTTGG
TAL#12    AGGCCCTGTGATTAGGGAAGAGTAT------------GGTACCGATTGCATTGAGATGCACGTTGG
TAL#13    AGGCCCTGTGATTAGGGAAGAGTAT------------GGTACCGATTGCATTGAGATGCACGTTGG
TAL#14    AGGCCCTGTGATTAGGGAAGAGTAT------------GGTACCGATTGCATTGAGATGCACGTTGG
```

Figure S2. Mutations induced by the TALEN system on the *APT* gene.
Blue and purple letters plus arrows indicate the sequences targeted by TALEN$_{402}$ and TALEN$_{405}$ respectively, in red target sequence for the FOK1 nuclease dimer. Point mutations are in bold capital letters on a yellow background and deletions with dashes. In the frames, the microhomologies potentially involved in Alt-EJ-mediated repair of the TALEn-induced DSBs.

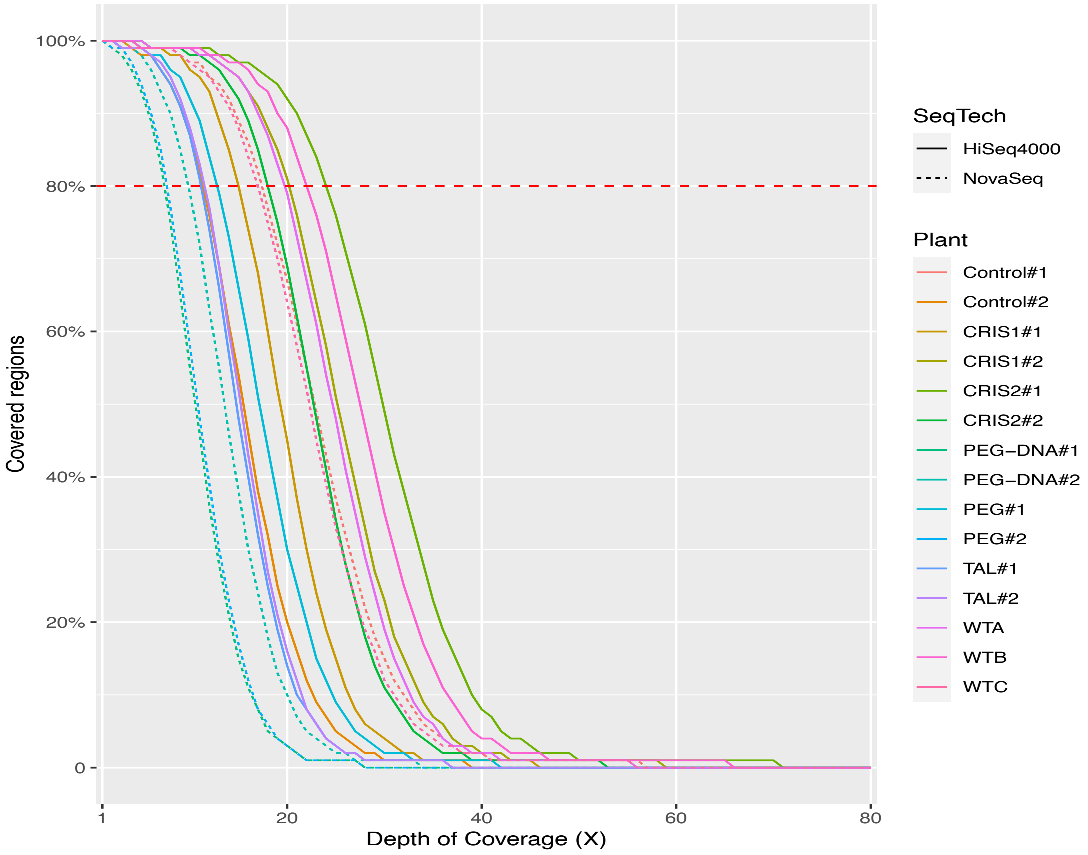

Figure S3. Depth of coverage.
Mean sequencing read depth for each conditions.

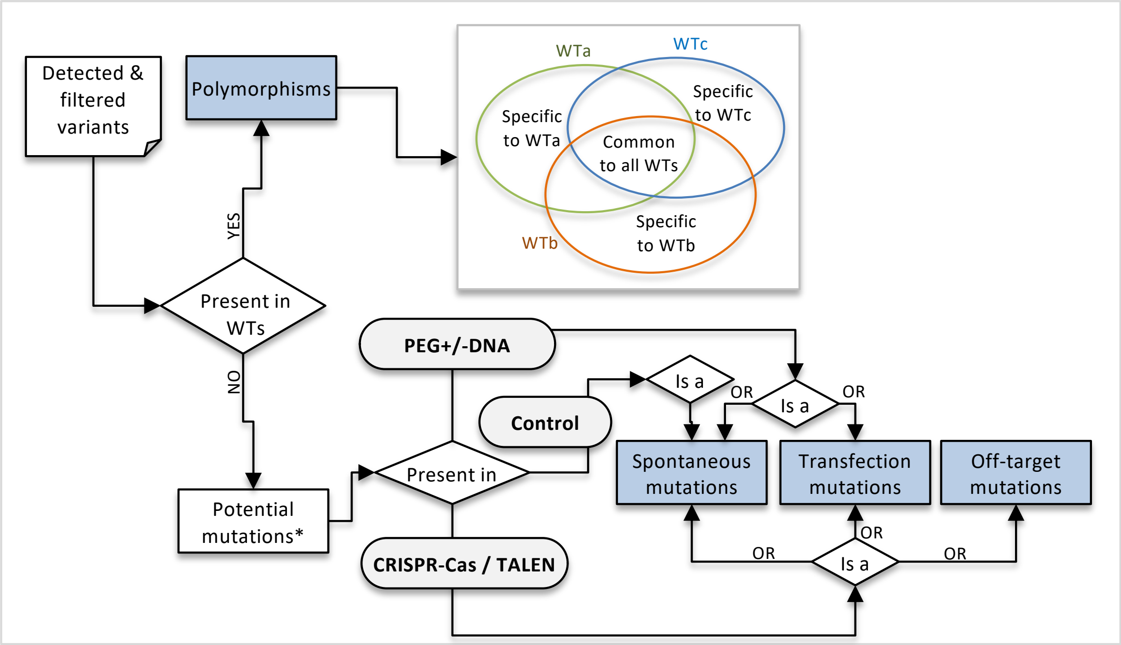

Figure S4. Classification of variants.
The variants detected in WTs are classified as polymorphisms. We distinguish the polymorphisms common to the three WTs and polymorphisms specific to each WT line. Variants considered as potential mutations are those detected in treated plants but not in corresponding parents (WT). The spontaneous mutations can occur in control or treated plants, thus present in all treated plants. The mutations due to transfection car occur in plants that have undergone treatment with PEG, thus present in treated plants. The SDN induced mutations (target mutations and off-target mutations) can occur only in plant that have undergone treatment with SDN (CRISPR-Cas or TALEN in our case).

* The variants present in treated plants and also in parents but at sub-clonal level are considered as chimeric variants and classified as spontaneous mutations

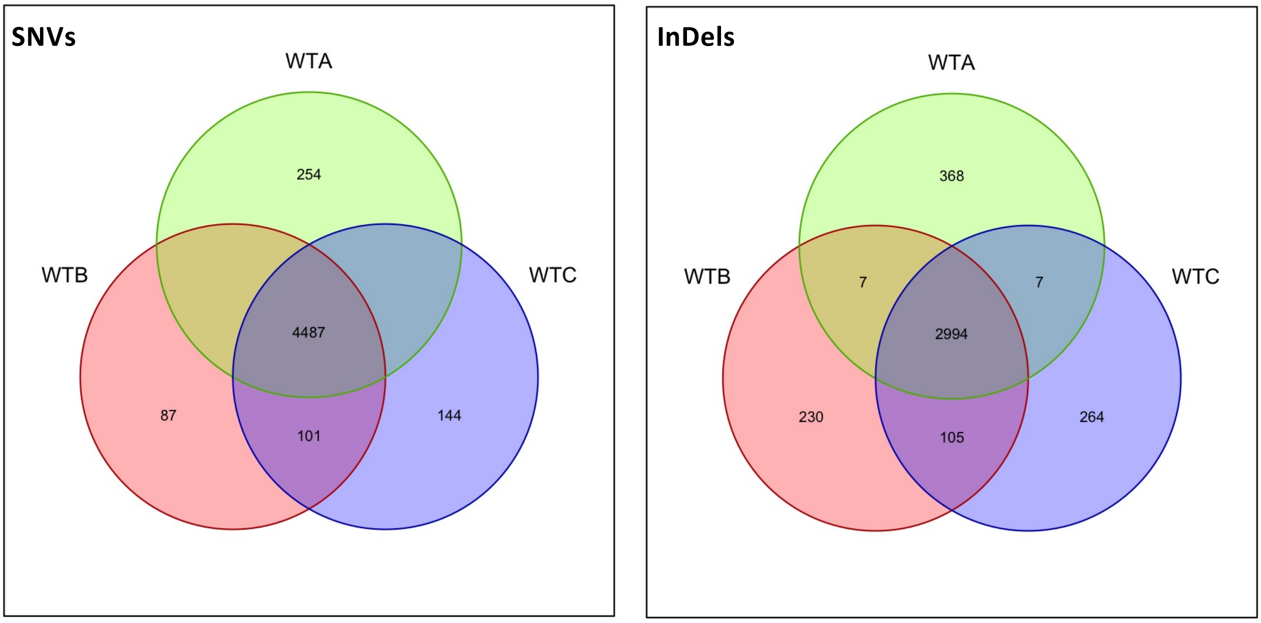

Figure S5. Polymorphism between the wild types used in this study and the reference genome.
Venn diagram of (a) SNVs and (b) InDels diversity of the wild types sequenced in this study with comparison to the reference genome sequences previously released.

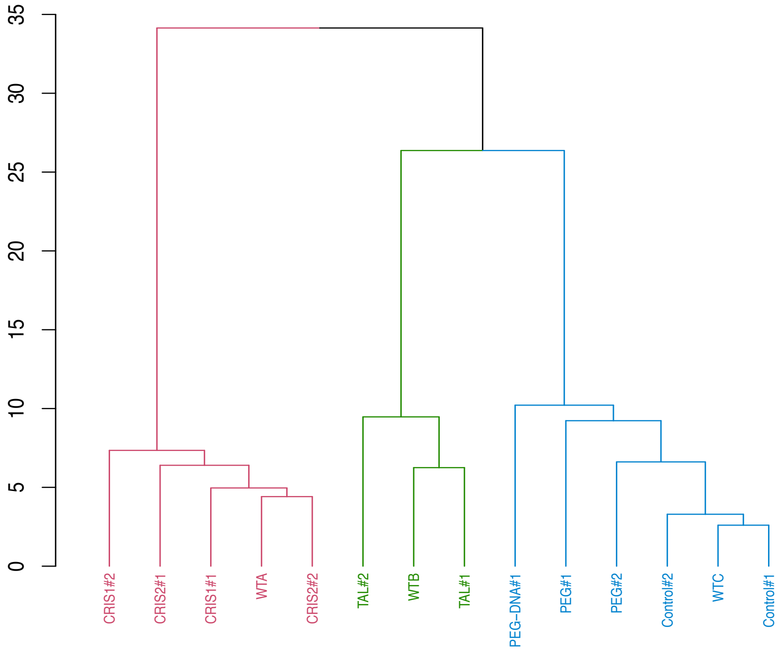

Figure S6. Sample clustering based on all detected variants.
Hierarchical clustering of the different samples, based on Euclidian distances between detected variants per sample.
Used functions :

```
dist.mat <- dist(t(var.mat), method = "euclidean")
hclust(dist.mat, method = "complete")
```

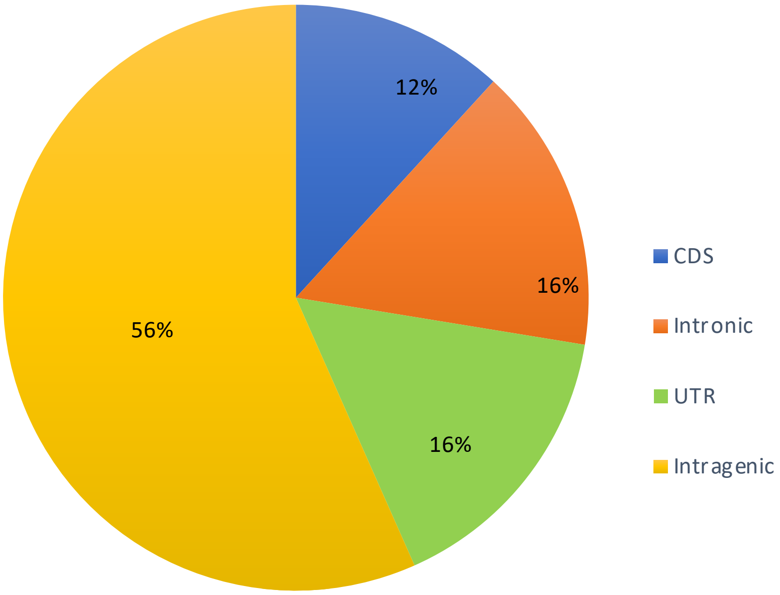

Figure S7. Distribution of variants normalised by genomic regions sizes.
The number of variants for each type of genomic regions has been normalised by the proportion of each of these regions in the genome.

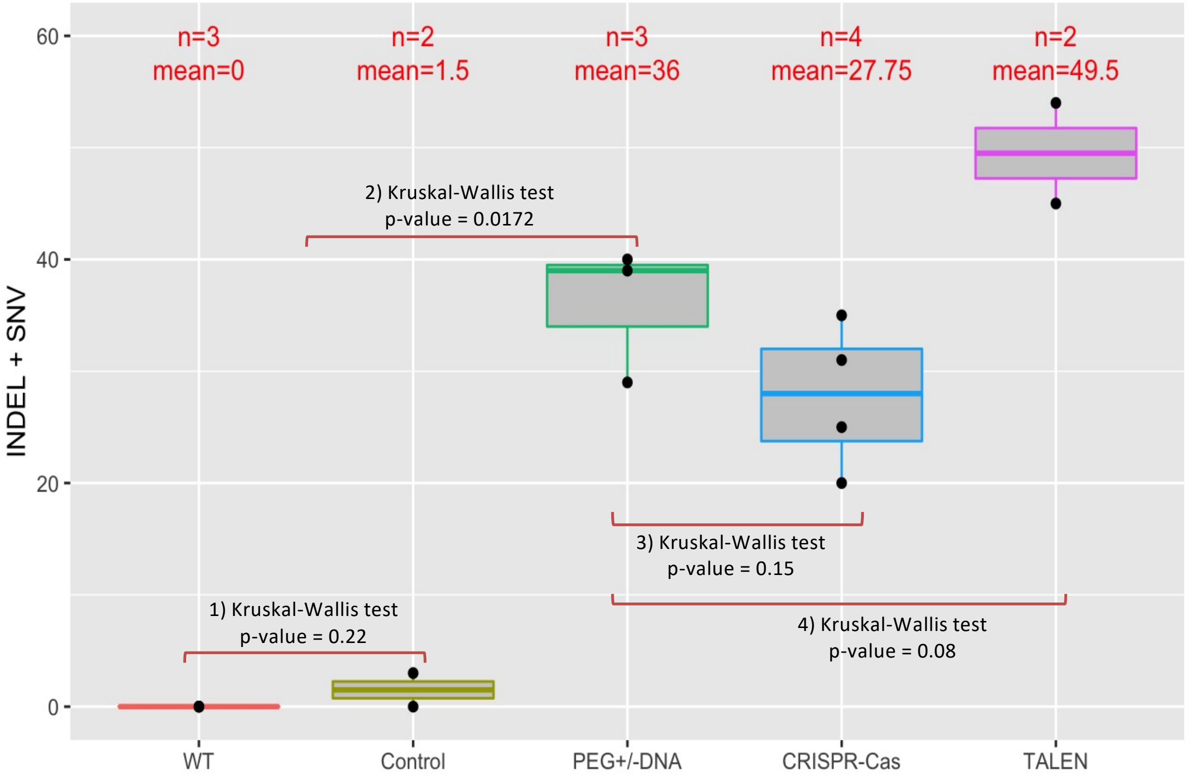

Figure S8. Boxplot for Kruskal-Wallis test of number of variants for the different treatments.
1) test between WT plants and Control plants.
2) test between WT + control plants and PEG+/-DNA treated plants.
3) test between PEG+/-DNA treated plants and CRISPR-Cas treated plants.
4) test between PEG+/-DNA treated plants and TALEN treated plants.

TCGAGGTCATTCATATGCTTGAGAAGAGAGTCGGGATAGTCCAAAATAAAACAAAGGTAAGATTACCTGGTCAAAAGTGAAAACATCAGTTAAA
AGGTGGTATAAGTAAAATATCGGTAATAAAAGGTGGCCCAAAGTGAAATTTACTCTTTTCTACTATTATAAAAATTGAGGATGTTTTGTCGGTA
CTTTGATACGTCATTTTTGTATGAATTGGTTTTTAAGTTTATTCGCGATTTGGAAATGCATATCTGTATTTGAGTCGGTTTTTAAGTTCGTTGC
TTTTGTAAATACAGAGGGATTTGTATAAGAAATATCTTTAAAAAACCCATATGCTAATTTGACATAATTTTTGAGAAAAATATATATTCAGGCG
AATTCCACAATGAACAATAATAAGATTAAAATAGCTTGCCCCCGTTGCAGCGATGGGTATTTTTTCTAGTAAAATAAAAGATAAACTTAGACTC
AAAACATTTACAAAAACAACCCCTAAAGTCCTAAAGCCCAAAGTGCTATGCACGATCCATAGCAAGCCCAGCCCAACCCAACCCAACCCAACCC
ACCCCAGTGCAGCCAACTGGCAAATAGTCTCCACCCCCGGCACTATCACCGTGAGTTGTCCGCACCACCGCACGTCTCGCAGCCAAAAAAAAAA
AAAGAAAGAAAAAAAAAGAAAAAGAAAAACAGCAGGTGGGTCCGGGTCGTGGGGGCCGGAAAAGCGAGGAGGATCGCGAGCAGCGACGAGGCCCG
GCCCTCCCTCCGCTTCCAAAGAAACGCCCCCCATCGCCACTATATACATACCCCCCCCTCTCCTCCCATCCCCCCAACCCTACCACCACCACCA
CCACCACCTCCTCCCCCCTCGCTGCCGGACGACGAGCTCCTCCCCCCTCCCCCTCCGCCGCCGCCGGTAACCACCCCGCCCCTCTCCTCTTTCT
TTCTCCGTTTTTTTTTTCGTCTCGGTCTCGATCTTTGGCCTTGGTAGTTTGGGTGGGCGAGAGCGGCTTCGTCGCCCAGATCGGTGCGCGGGAG
GGGCGGGATCTCGCGGCTGGCGTCTCCGGGCGTGAGTCGGCCCGGATCCTCGCGGGGAATGGGGCTCTCGGATGTAGATCTTCTTTCTTTCTTC
TTTTTGTGGTAGAATTTGAATCCCTCAGCATTGTTCATCGGTAGTTTTTCTTTTCATGATTTGTGACAAATGCAGCCTCGTGCGGAGCTTTTTT
GTAGGTAGACCATGGCTGACGCCGAGCTTAAGCTGATCCACTAGTCCAGTGTGGTGGAATTCGCCATGGACTACAAAGACCATGACGGTGATTA
TAAAGATCATGACATCGATTACAAGGATGACGATGACAAGATGGCCCCCAAGAAGAAGAGGAAGGTGGGCATCCACGGGGTACCCATGGTGGAC
CTGAGAACCCTGGGCTACAGCCAGCAGCAGCAGGAGAAGATCAAGCCCAAGGTGAGAAGCACCGTGGCCCAGCACCACGAGGCCCTGGTGGGCC
ACGGCTTCACCCACGCCCACATCGTGGCCCTGAGCCAGCACCCCGCCGCCCTGGGCACCGTGGCCGTGAAGTACCAGGACATGATCGCCGCCCT
GCCCGAGGCCACCCACGAGGCCATCGTGGGCGTGGGCAAGCAGTGGAGCGGCGCCAGAGCCCTGGAGGCCCTGCTGACCGTGGCCGGCGAGCTG
AGAGGCCCCCCCGGGACCCTGGACACCGGCCAGCTGCTGAAGATCGCCAAGAGAGGCGGCGTGACTGCAGTGGAGGCAGTGCATGCATGGCGCA
ATGCACTGACGGGTGCCCCCCTGAACCTGACCCCGGAGCAGGTGGTGGCCATCGCTAGTAACAATGGTGGCAAACAGGCTCTTGAAACCGTACA
GCGCCTACTGCCAGTTCTCTGTCAAGCCCACGGTCTGACTCCGGAGCAAGTTGTAGCGATTGCTAGTAATGGGGGTGGCAAACAGGCTCTTGAG
ACTGTTCAGCGCCTTCTACCAGTTCTCTGTCAAGCCCACGGCCTGACGCCCGAGCAAGTTGTAGCGATTGCTAGTAACAATGGTGGCAAACAGG
CTCTTGAAACCGTACAGCGCCTACTGCCAGTTCTCTGTCAAGCCCACGGTCTGACTCCGGAGCAAGTTGTAGCGATTGCTAGTAATATTGGTGG
CAAACAGGCTCTTGAGACGGTTCAGCGCCTCCTTCCAGTTCTCTGTCAAGCCCACGGACTCACCCCAGATCAAGTTGTAGCGATTGCTAGTAAT
GGGGGTGGCAAACAGGCTCTTGAGACTGTTCAGCGCCTTCTACCAGTTCTCTGTCAAGCCCACGGCCTGACGCCCGAGCAAGTTGTAGCGATTG
CTAGTAATGGGGGTGGCAAACAGGCTCTTGAGACTGTTCAGCGCCTTCTACCAGTTCTCTGTCAAGCCCACGGCCTGACGCCCGAGCAAGTTGT
AGCGATTGCTAGTAATATTGGTGGCAAACAGGCTCTTGAGACGGTTCAGCGCCTCCTTCCAGTTCTCTGTCAAGCCCACGGACTCACCCCAGAT
CAAGTTGTAGCGATTGCTAGTAACAATGGTGGCAAACAGGCTCTTGAAACCGTACAGCGCCTACTGCCAGTTCTCTGTCAAGCCCACGGTCTGA
CTCCGGAGCAAGTTGTAGCGATTGCTAGTAACAATGGTGGCAAACAGGCTCTTGAAACCGTACAGCGCCTACTGCCAGTTCTCTGTCAAGCCCA
CGGTCTGACTCCGGAGCAAGTTGTAGCGATTGCTAGTAACAATGGTGGCAAACAGGCTCTTGAAACCGTACAGCGCCTACTGCCAGTTCTCTGT
CAAGCCCACGGTCTGACTCCGGAGCAAGTTGTAGCGATTGCTAGTAATATTGGTGGCAAACAGGCTCTTGAGACGGTCAGCGCCTCCTTCCAGT
TCTCTGTCAAGCCCACGGACTCACCCCAGATCAAGTTGTAGCGATTGCTAGTAATATTGGTGGCAAACAGGCTCTTGAGACGGTTCAGCGCCTC
CTTCCAGTTCTCTGTCAAGCCCACGGACTCACCCCAGATCAAGTTGTAGCGATTGCTAGTAACAATGGTGGCAAACAGGCTCTTGAAACCGTAC
AGCGCCTACTGCCAGTTCTCTGTCAAGCCCACGGTCTGACTCCGGAGCAAGTTGTAGCGATTGCTAGTAATATTGGTGGCAAACAGGCTCTTGA
GACGGTTCAGCGCCTCCTTCCAGTTCTCTGTCAAGCCCACGGACTCACCCCAGATCAAGTTGTAGCGATTGCTAGTAACAATGGTGGCAAACAG
GCTCTTGAAACCGTACAGCGCCTACTGCCAGTTCTCTGTCAAGCCCACGGTCTGACTCCGGAGCAAGTTGTAGCGATTGCTAGTAATGGGGGTG
GCAAACAGGCTCTTGAGACTGTTCAGCGCCTTCTACCAGTTCTCTGTCAAGCCCACGGCCTGACGCCCGAGCAAGTTGTAGCGATTGCTAGCAA
TATTGGCGGCAGGCCGGCGCTCGAGAGCATCGTGGCCCAGCTGAGCAGACCCGACCCCGCCCTGGCCGCCCTGACCAACGACCACCTGGTGGCC
CTGGCCTGCCTGGGCGGCAGACCCGCCCTGGACGCCGTGAAGAAGGGCCTGCCCCACGCCCCCGCCCTGATCAAGAGAACCAACAGAAGAATCC
CCGAGAGAACCAGCCACAGAGTGGCCGGATCCCAGCTGGTGAAGAGCGAGCTGGAGGAGAAGAAGAGCGAGCTGAGACACAAGCTGAAGTACGT
GCCCCACGAGTACATCGAGCTGATCGAGATCGCCAGAAACAGCACCCAGGACAGAATCCTGGAGATGAAGGTGATGGAGTTCTTCATGAAGGTG
TACGGCTACAGAGGCAAGCACCTGGGCGGCAGCAGAAAGCCCGACGGCGCCATCTACACCGTGGGCAGCCCCATCGACTACGGCGTGATCGTGG
ACACCAAGGCCTACAGCGGCGGCTACAACCTGCCCATCGGCCAGGCCGACGAGATGCAGAGATACGTGGAGGAGAACCAGACCAGAAACAAGCA
CATCAACCCCAACGAGTGGTGGAAGGTGTACCCCAGCAGCGTGACCGAGTTCAAGTTCCTGTTCGTGAGCGGCCACTTCAAGGGCAACTACAAG
GCCCAGCTGACCAGACTGAACCACATCACCAACTGCAACGGCGCCGTGCTGAGCGTGGAGGAGCTGCTGATCGGCGGCGAGATGATCAAGGCCG
GCACCCTGACCCTGGAGGAGGTGAGAAGAAAGTTCAACAACGGCGAGATCAACTTCAGAAGCTGATAACTGGAGTCTAGAGTCGACCTGCAGGC
ATGCCCGCTGAAATCACCAGTCTCTCTCTACAAATCTATCTCTCTCTATAATAATGTGTGAGTAGTTCCCAGATAAGGGAATTAGGGTTCTTAT
AGGGTTTCGCTCATGTGTTGAGCATATAAGAAACCCTTAGTATGTATTTGTATTTGTAAAATACTTCTATCAATAAAATTTCTAATTCCTAAAA
CCAAAATCCAGGGGTACCGAGCTCGAATTCAAGCT

Figure S9. Sequence of the TALEN$_{402}$ expression cassette.

The rice Actin 1 promoter is in blue, the three tandem FLAG epitope tags is in red, the SV40 NLS is in green, in pink the TAL effector repeat array is in pink, the nonspecific DNA cleavage domain of the FokI endonuclease is in orange, the CaMV35S terminator is in black. Codons corresponding to the repeat Variable Diresidue (RVD), specific to A (NI) are highlighted in blue, specific to T (NG) are highlighted in red, specific to G (NS) are highlighted in yellow. Nucleotide sequence recognized is GTGATTAGGGAAGAGTA.

TCGAGGTCATTCATATGCTTGAGAAGAGAGTCGGGATAGTCCAAAATAAAACAAAGGTAAGATTACCTGGTCAAAAGTGAAAACATCAGTTAAA
AGGTGGTATAAGTAAAATATCGGTAATAAAAGGTGGCCCAAAGTGAAATTTACTCTTTTCTACTATTATAAAAATTGAGGATGTTTTGTCGGTA
CTTTGATACGTCATTTTTGTATGAATTGGTTTTTAAGTTTATTCGCGATTTGGAAATGCATATCTGTATTTGAGTCGGTTTTTAAGTTCGTTGC
TTTTGTAAATACAGAGGGATTTGTATAAGAAATATCTTTAAAAAACCCATATGCTAATTTGACATAATTTTTGAGAAAAATATATATTCAGGCG
AATTCCACAATGAACAATAATAAGATTAAAATAGCTTGCCCCCGTTGCAGCGATGGGTATTTTTTCTAGTAAAATAAAAGATAAACTTAGACTC
AAAACATTTACAAAAACAACCCCTAAAGTCCTAAAGCCCAAAGTGCTATGCACGATCCATAGCAAGCCCAGCCCAACCCAACCCAACCCAACCC
ACCCCAGTGCAGCCAACTGGCAAATAGTCTCCACCCCCGGCACTATCACCGTGAGTTGTCCGCACCACCGCACGTCTCGCAGCCAAAAAAAAAA
AAAGAAAGAAAAAAAAAGAAAAAGAAAAACAGCAGGTGGGTCCGGGTCGTGGGGGCCGGAAAAGCGAGGAGGATCGCGAGCAGCGACGAGGCCCG
GCCCTCCCTCCGCTTCCAAAGAAACGCCCCCCATCGCCACTATATACATACCCCCCCCTCTCCTCCCATCCCCCCAACCCTACCACCACCACCA
CCACCACCTCCTCCCCCCTCGCTGCCGGACGACGAGCTCCTCCCCCCTCCCCCTCCGCCGCCGCCGGTAACCACCCCGCCCCTCTCCTCTTTCT
TTCTCCGTTTTTTTTTTCGTCTCGGTCTCGATCTTTGGCCTTGGTAGTTTGGGTGGGCGAGAGCGGCTTCGTCGCCCAGATCGGTGCGCGGGAG
GGGCGGGATCTCGCGGCTGGCGTCTCCGGGCGTGAGTCGGCCCGGATCCTCGCGGGGAATGGGGCTCTCGGATGTAGATCTTCTTTCTTTCTTC
TTTTTGTGGTAGAATTTGAATCCCTCAGCATTGTTCATCGGTAGTTTTTCTTTTCATGATTTGTGACAAATGCAGCCTCGTGCGGAGCTTTTTT
GTAGGTAGACCATGGCTGACGCCGAGCTTAAGCTGATCCACTAGTCCAGTGTGGTGGAATTCGCCATGGACTACAAAGACCATGACGGTGATTA
TAAAGATCATGACATCGATTACAAGGATGACGATGACAAGATGGCCCCCAAGAAGAAGAGGAAGGTGGGCATCCACGGGGTACCCATGGTGGAC
CTGAGAACCCTGGGCTACAGCCAGCAGCAGCAGGAGAAGATCAAGCCCAAGGTGAGAAGCACCGTGGCCCAGCACCACGAGGCCCTGGTGGGCC
ACGGCTTCACCCACGCCCACATCGTGGCCCTGAGCCAGCACCCCGCCGCCCTGGGCACCGTGGCCGTGAAGTACCAGGACATGATCGCCGCCCT
GCCCGAGGCCACCCACGAGGCCATCGTGGGCGTGGGCAAGCAGTGGAGCGGCGCCAGAGCCCTGGAGGCCCTGCTGACCGTGGCCGGCGAGCTG
AGAGGCCCCCCCGGGACCCTGGACACCGGCCAGCTGCTGAAGATCGCCAAGAGAGGCGGCGTGACTGCAGTGGAGGCAGTGCATGCATGGCGCA
ATGCACTGACGGGTGCCCCCCTGAACCTGACCCCGGAGCAGGTGGTGGCCATCGCTAGTCATGACGGTGGCAAACAGGCTCTTGAGACCGTCCA
ACGCCTTCTACCAGTTCTCTGTCAAGCCCACGGACTAACCCCAGCGCAAGTTGTAGCGATTGCTAGTAATGGGGGTGGCAAACAGGCTCTTGAG
ACTGTTCAGCGCCTTCTACCAGTTCTCTGTCAAGCCCACGGCCTGACGCCCGAGCAAGTTGTAGCGATTGCTAGTCATGACGGTGGCAAACAGG
CTCTTGAGACCGTCCAACGCCTTCTACCAGTTCTCTGTCAAGCCCACGGACTAACCCCAGCGCAAGTTGTAGCGATTGCTAGTAATATTGGTGG
CAAACAGGCTCTTGAGACGGTTCAGCGCCTCCTTCCAGTTCTCTGTCAAGCCCACGGACTCACCCCAGATCAAGTTGTAGCGATTGCTAGTAAT
ATTGGTGGCAAACAGGCTCTTGAGACGGTTCAGCGCCTCCTTCCAGTTCTCTGTCAAGCCCACGGACTCACCCCAGATCAAGTTGTAGCGATTG
CTAGTAATGGGGGTGGCAAACAGGCTCTTGAGACTGTTCAGCGCCTTCTACCAGTTCTCTGTCAAGCCCACGGCCTGACGCCCGAGCAAGTTGT
AGCGATTGCTAGTAACAATGGTGGCAAACAGGCTCTTGAAACCGTACAGCGCCTACTGCCAGTTCTCTGTCAAGCCCACGGTCTGACTCCGGAG
CAAGTTGTAGCGATTGCTAGTCATGACGGTGGCAAACAGGCTCTTGAGACCGTCCAACGCCTTCTACCAGTTCTCTGTCAAGCCCACGGACTAA
CCCCAGCGCAAGTTGTAGCGATTGCTAGTAATATTGGTGGCAAACAGGCTCTTGAGACGGTTCAGCGCCTCCTTCCAGTTCTCTGTCAAGCCCA
CGGACTCACCCCAGATCAAGTTGTAGCGATTGCTAGTAATATTGGTGGCAAACAGGCTCTTGAGACGGTTCAGCGCCTCCTTCCAGTTCTCTGT
CAAGCCCACGGACTCACCCCAGATCAAGTTGTAGCGATTGCTAGTAATGGGGGTGGCAAACAGGCTCTTGAGACTGTTCAGCGCCTTCTACCAG
TTCTCTGTCAAGCCCACGGCCTGACGCCCGAGCAAGTTGTAGCGATTGCTAGTCATGACGGTGGCAAACAGGCTCTTGAGACCGTCCAACGCCT
TCTACCAGTTCTCTGTCAAGCCCACGGACTAACCCCAGCGCAAGTTGTAGCGATTGCTAGTAACAATGGTGGCAAACAGGCTCTTGAAACCGTA
CAGCGCCTACTGCCAGTTCTCTGTCAAGCCCACGGTCTGACTCCGGAGCAAGTTGTAGCGATTGCTAGTAACAATGGTGGCAAACAGGCTCTTG
AAACCGTACAGCGCCTACTGCCAGTTCTCTGTCAAGCCCACGGTCTGACTCCGGAGCAAGTTGTAGCGATTGCTAGTAATGGGGGTGGCAAACA
GGCTCTTGAGACTGTTCAGCGCCTTCTACCAGTTCTCTGTCAAGCCCACGGCCTGACGCCCGAGCAAGTTGTAGCGATTGCTAGTAATATTGGT
GGCAAACAGGCTCTTGAGACGGTTCAGCGCCTCCTTCCAGTTCTCTGTCAAGCCCACGGACTCACCCCAGATCAAGTTGTAGCGATTGCTAGCC
ATGACGGCGGCAGGCCGGCGCTCGAGAGCATCGTGGCCCAGCTGAGCAGACCCGACCCCGCCCTGGCCGCCCTGACCAACGACCACCTGGTGGC
CCTGGCCTGCCTGGGCGGCAGACCCGCCCTGGACGCCGTGAAGAAGGGCCTGCCCCACGCCCCCGCCCTGATCAAGAGAACCAACAGAAGAATC
CCCGAGAGAACCAGCCACAGAGTGGCCGGATCCCAGCTGGTGAAGAGCGAGCTGGAGGAGAAGAAGAGCGAGCTGAGACACAAGCTGAAGTACG
TGCCCCACGAGTACATCGAGCTGATCGAGATCGCCAGAAACAGCACCCAGGACAGAATCCTGGAGATGAAGGTGATGGAGTTCTTCATGAAGGT
GTACGGCTACAGAGGCAAGCACCTGGGCGGCAGCAGAAAGCCCGACGGCGCCATCTACACCGTGGGCAGCCCCATCGACTACGGCGTGATCGTG
GACACCAAGGCCTACAGCGGCGGCTACAACCTGCCCATCGGCCAGGCCGACGAGATGCAGAGATACGTGGAGGAGAACCAGACCAGAAACAAGC
ACATCAACCCCAACGAGTGGTGGAAGGTGTACCCCAGCAGCGTGACCGAGTTCAAGTTCCTGTTCGTGAGCGGCCACTTCAAGGGCAACTACAA
GGCCCAGCTGACCAGACTGAACCACATCACCAACTGCAACGGCGCCGTGCTGAGCGTGGAGGAGCTGCTGATCGGCGGCGAGATGATCAAGGCC
GGCACCCTGACCCTGGAGGAGGTGAGAAGAAAGTTCAACAACGGCGAGATCAACTTCAGAAGCTGATAACTGGAGTCTAGAGTCGACCTGCAGG
CATGCCCGCTGAAATCACCAGTCTCTCTCTACAAATCTATCTCTCTCTATAATAATGTGTGAGTAGTTCCCAGATAAGGGAATTAGGGTTCTTA
TAGGGTTTCGCTCATGTGTTGAGCATATAAGAAACCCTTAGTATGTATTTGTATTTGTAAAATACTTCTATCAATAAAATTTCTAATTCCTAAA
ACCAAAATCCAGGGGTACCGAGCTCGAATTCAAGCT

Figure S10. Sequence of the $TALEN_{405}$ expression cassette.
The rice Actin 1 promoter is in blue, the three tandem FLAG epitope tags is in red, the SV40 NLS is in green, in pink the TAL effector repeat array is in pink, the nonspecific DNA cleavage domain of the FokI endonuclease is in orange, the CaMV35S terminator is in black. Codons corresponding to the repeat Variable Diresidue (RVD), specific to A (NI) are highlighted in blue, specific to T (NG) are highlighted in red, specific to G (NS) are highlighted in yellow, specific to C (HD) are highlighted in green. Nucleotide sequence recognized is ATGGCTAACGTAACTC.
